# Optimized Red-Absorbing Dyes for Imaging and Sensing

**DOI:** 10.1021/jacs.3c05273

**Published:** 2023-10-16

**Authors:** Jonathan B. Grimm, Ariana N. Tkachuk, Ronak Patel, S. Thomas Hennigan, Alina Gutu, Peng Dong, Valentina Gandin, Anastasia M. Osowski, Katie L. Holland, Zhe J. Liu, Timothy A. Brown, Luke D. Lavis

**Affiliations:** Janelia Research Campus, Howard Hughes Medical Institute, 19700 Helix Drive, Ashburn, Virginia 20147, United States

## Abstract

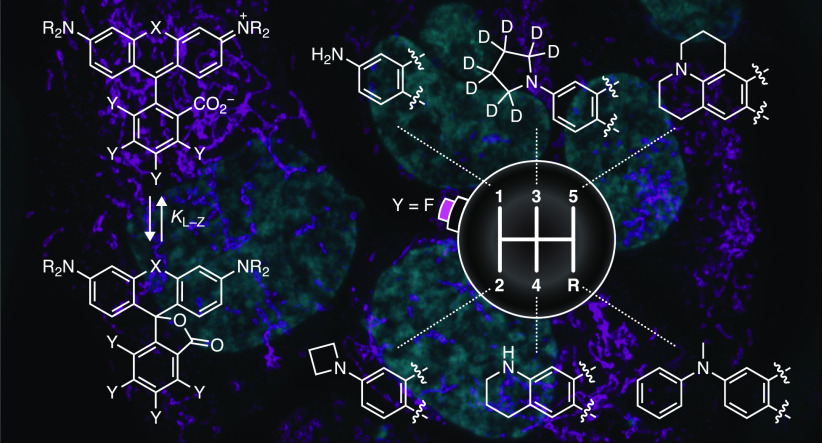

Rhodamine dyes are excellent scaffolds for developing a broad range of fluorescent probes. A key property of rhodamines is their equilibrium between a colorless lactone and fluorescent zwitterion. Tuning the lactone–zwitterion equilibrium constant (*K*_L–Z_) can optimize dye properties for specific biological applications. Here, we use known and novel organic chemistry to prepare a comprehensive collection of rhodamine dyes to elucidate the structure–activity relationships that govern *K*_L–Z_. We discovered that the auxochrome substituent strongly affects the lactone–zwitterion equilibrium, providing a roadmap for the rational design of improved rhodamine dyes. Electron-donating auxochromes, such as julolidine, work in tandem with fluorinated pendant phenyl rings to yield bright, red-shifted fluorophores for live-cell single-particle tracking (SPT) and multicolor imaging. The *N*-aryl auxochrome combined with fluorination yields red-shifted Förster resonance energy transfer (FRET) quencher dyes useful for creating a new semisynthetic indicator to sense cAMP using fluorescence lifetime imaging microscopy (FLIM). Together, this work expands the synthetic methods available for rhodamine synthesis, generates new reagents for advanced fluorescence imaging experiments, and describes structure–activity relationships that will guide the design of future probes.

## Introduction

Fluorescence microscopy depends on the labeling of cellular components with fluorophores. Beginning with the development of fluorescein isocyanate (FIC) by Coons in the 1940s,^1^ followed by the shelf-stable analog fluorescein isothiocyanate (FITC) by Metcalf in the 1950s,^2^ small-molecule fluorophores have become ubiquitous labels for antibodies and other affinity reagents used in cellular imaging.^3^ Chemical dyes are also employed in self-labeling tag systems, such as the SNAP-tag^4,5^ and HaloTag,^6,7^ and in fluorogenic ligands that bind endogenous biomolecular targets.^8,9^ These strategies have expanded the utility of small-molecule fluorophores to label proteins inside living cells and animals, complementing genetically encoded labels such as green fluorescent protein (GFP).^10^ Of the extant classes of fluorophores, the rhodamine dyes remain in wide use as biomolecule labels, self-labeling tag ligands, and fluorogenic stains due to their excellent photophysical properties, tunable structures, and bioavailability.^11−13^ Elucidation of structure–activity relationships in rhodamines combined with new methods to synthesize functional dye derivatives leads to the creation of optimized reagents.

**Figure 1:**
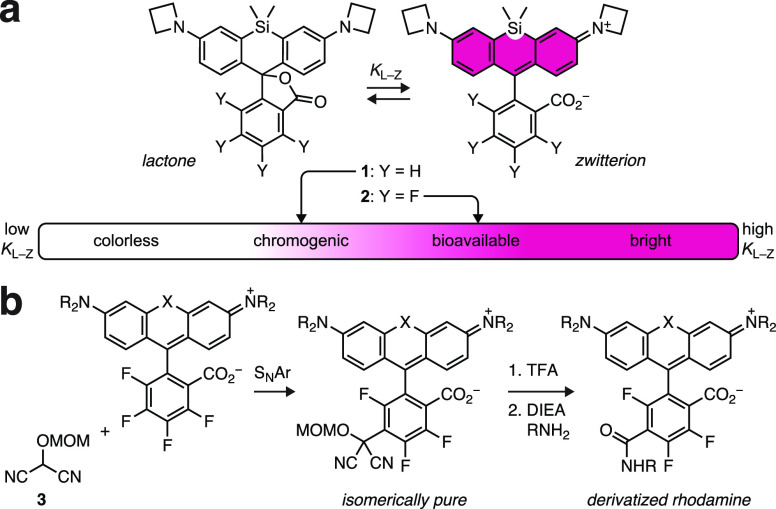
Fluorination adjusts *K*_L–Z_ and allows facile derivatization of rhodamine dyes. (a) The lactone–zwitterion equilibrium of JF_646_ (**1**) and JF_669_ (**2**) with scale showing the different categories of dyes based on *K*_L–Z_. (b) Synthesis of functional fluorinated rhodamines using the masked acyl cyanide (MAC) reagent **3**.

Rhodamine dyes bearing an *ortho*-carboxyl substituent on the pendant phenyl ring exist in equilibrium between a lipophilic lactone (L) and fluorescent zwitterion (Z). We previously established that the lactone–zwitterion equilibrium constant (*K*_L–Z_) could be used to rationalize the performance of rhodamine dyes in biological systems (Figure 1a).^14−16^ We categorize four different classes of dyes based on *K*_L–Z_. First, rhodamines with very low *K*_L–Z_ values are colorless and not useful for biological imaging. Second, rhodamines with slightly higher *K*_L–Z_ values also preferentially lactonize in aqueous solution but have some propensity to adopt the zwitterionic form. Such dyes can be used to fashion chromogenic compounds; the change in environment that accompanies dye ligand binding shifts the equilibrium to the zwitterionic form.^15,16^ Third, dyes with modestly higher *K*_L–Z_ values mostly adopt the zwitterionic form in aqueous solution but can lactonize in hydrophobic environments; ligands based on these fluorophores often show improved membrane permeability making them more bioavailable.^16^ Finally, fluorophores with large *K*_L–Z_ values exhibit high absorptivity, making them bright, environmentally insensitive labels.^14^ Understanding the *K*_L–Z_ trends of a particular dye scaffold and tuning this property through structural modifications can allow the optimization of fluorescent labels for biological imaging experiments.

We recently demonstrated that fluorination of the pendant phenyl ring increases the *K*_L–Z_ of azetidine-containing rhodamine dyes. For example, Janelia Fluor 646 (JF_646_, **1**; Figure 1a) and similar Si-bridged rhodamine dyes preferentially adopt the nonfluorescent lactone form in aqueous solution, allowing the creation of chromogenic (and therefore fluorogenic) molecules.^15−18^ Incorporation of fluorine atoms on the pendant phenyl ring results in JF_669_ (**2**; Figure 1a), which shows a higher *K*_L–Z_ and can be used to make bioavailable fluorescent labels.^14,16^ In addition to modulating *K*_L–Z_, fluorination of the pendant phenyl ring increases the absorption maximum (λ_abs_) and the emission maximum (λ_em_) of xanthene dyes and their analogs.^14,19−21^ We also discovered that fluorinated phenyl rings enable facile derivatization of rhodamine dyes using Yamamoto’s masked acyl cyanide (MAC) reagent concept.^22^ In this *umpolung* approach, we employ a 2-hydroxymalononitrile MAC reagent with a methoxymethyl ether (MOM) protecting group (**3**; Figure 1b). This reacts with a rhodamine dye bearing a perfluorinated pendant phenyl ring in a regioselective nucleophilic aromatic substitution (S_N_Ar) reaction.^16^ Removal of the MOM group using acid, followed by neutralization with a tertiary amine base, generates a putative acyl cyanide that is primed for subsequent chemistry to generate an acid, ester, or amide at the 6-position of the rhodamine; this regiochemistry is optimal for self-labeling tag ligands.^23,24^ The late-stage derivatization chemistry using an acyl anion equivalent circumvents the isomeric mixtures that result from classic synthetic strategies^12,13,25−28^ and the protecting group challenges that come with modern methods,^29−34^ allowing the transformation of an unsubstituted dye to a functional derivative in just two synthetic steps.

In previous work, we investigated the structure–activity relationships governing *K*_L–Z_, the effect of fluorination, and the generality of the MAC chemistry on a limited set of rhodamine dyes containing four-membered azetidine substituents.^16^ Here, we think outside the azetidine “box” and explore dyes containing different auxochromes. We first investigate different synthetic methods to prepare rhodamine and related dyes, expanding several known routes and developing a new succinct strategy for preparing fluorinated dyes using Li/H exchange. We used this suite of methods to synthesize a comprehensive collection of fluorinated and nonfluorinated rhodamine dyes with different nitrogen substituents. Evaluation of the properties of these known and novel molecules reveals that the range of accessible *K*_L–Z_ values within a given rhodamine scaffold is strongly dependent on the identity of the auxochrome group. Despite these large differences in lactone–zwitterion equilibrium, fluorination universally increases *K*_L–Z_ regardless of the dye structure. For rhodamines bearing electron-donating julolidine substituents, which exhibit high *K*_L–Z_ values, incorporation of fluorine substituents results in a bathochromic shift and increased absorptivity, yielding bright, red-shifted fluorophores useful for multicolor imaging and live-cell single-particle tracking (SPT). For dyes containing electron-withdrawing aniline substituents, fluorination improves chromogenicity, giving a live-cell-compatible Förster resonance energy transfer (FRET) quencher dye that is useful for fluorescence lifetime imaging microscopy (FLIM). Together, this work expands the synthetic methods available for rhodamine synthesis, demonstrates the practicability of *K*_L–Z_ tuning beyond azetidine-containing dyes, and yields new probes with improved properties for advanced fluorescence imaging experiments such as SPT and FLIM.

## Results and Discussion

### Synthesis of Fluorinated Fluorophores

**Scheme 1:**
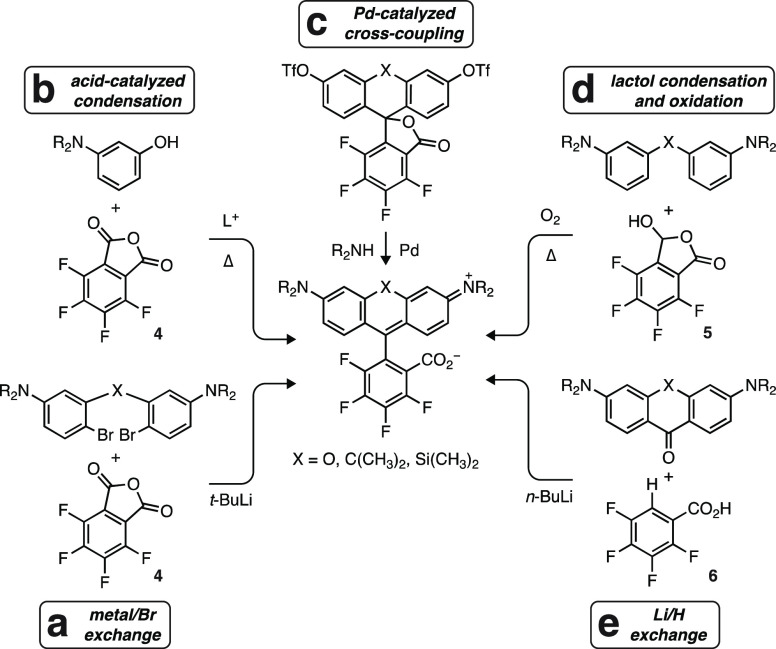
Synthetic Approaches for Fluorinated Rhodamines

We considered different methods to construct rhodamine dyes, focusing on the synthesis of the less-explored fluorinated derivatives. In prior work,^16^ we made fluorinated rhodamines using a synthetic approach first developed in our laboratory,^14,35−37^ where a di(bromoarene) undergoes metal/Br exchange and addition to tetrafluorophthalic anhydride (**4**) to yield fluorinated rhodamine dyes (Scheme 1a). This complements the standard method of fluorinated rhodamine synthesis involving protic or Lewis acid-catalyzed condensation of 3-aminophenols and **4** (Scheme 1b).^38,39^ In addition to these established methods to construct dyes with fluorinated phenyl systems, we considered repurposing other strategies for fluorinated rhodamine synthesis. These include the Pd-catalyzed cross-coupling using fluorescein ditriflates^40,41^ (Scheme 1c) and the oxidative condensation of phthalaldehydic acids with 3-aminophenols or diphenyl ethers^42^ (Scheme 1d). Neither method has been used to make fluorinated rhodamines, but we surmised that use of fluorinated fluorescein ditriflates or tetrafluorophthalaldehydic acid (**5**), respectively, could allow synthesis of the desired compounds. We also considered a novel strategy to prepare fluorinated dyes by lithiation of 2,3,4,5-tetrafluorobenzoic acid^43^ (**6**) and addition to substituted xanthone derivatives and their congeners (Scheme 1e). We set out to explore the scope of each synthetic method, evaluating the utility of these strategies to prepare standard oxygen-containing rhodamines, Si-rhodamines, and carborhodamines along with related dyes based on fluoresceins and Malachite Green.

### Lactol Condensation and Oxidation for the Synthesis of Fluorinated Rhodamines

**Scheme 2:**
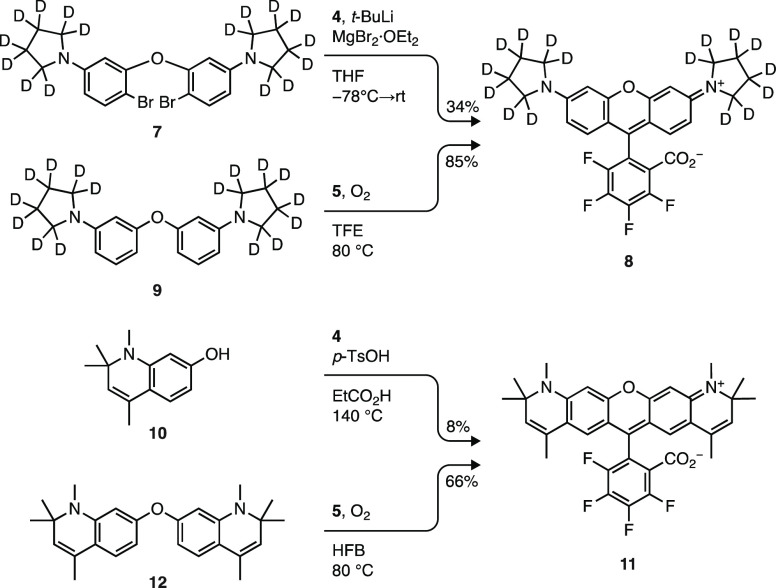
Synthesis of Fluorinated Rhodamines

We first considered the reaction of aminophenols with partially reduced phthalic anhydrides (phthalaldehydic acids) in the presence of O_2_ (Scheme 1d). This recently described strategy^42^ is an efficient and versatile method for the regioselective synthesis of rhodamine dyes including carboxy derivatives but has not been applied to fluorinated dyes. We use the shorthand term “lactol condensation” to refer to this approach, as phthalaldehydic acids mostly adopt the closed, 3-hydroxyphthalide (lactol) form in solution.^44^ We compared this strategy to previously described methods of rhodamine synthesis. Metal/bromide exchange of the dibromide **7** (Scheme S1a) and addition to tetrafluorophthalic anhydride (**4**) afforded the novel deuterated dye **8** in 34% yield (Scheme 2); we named this fluorophore “JFX_576_” due to the deuterium substitution^45^ and its absorption maximum in aqueous solution. Reaction of diphenyl ether **9** (Scheme S1a) with tetrafluorolactol **5** in 2,2,2-trifluoroethanol (TFE) allowed convenient access to **8** in a higher yield (85%), demonstrating the utility of this lactol chemistry in making fluorinated rhodamines.

We then compared this lactol condensation strategy with the classic acid-catalyzed reaction method, which remains the most common protocol for rhodamine synthesis but shows variable results when preparing fluorinated rhodamines.^38,39^ Reaction of dihydroquinoline **10** with **4** in refluxing propionic acid gave rhodamine **11** in a low yield (8%; Scheme 2); other condensation reaction conditions were unsuccessful in our hands (Table S1). This is consistent with previous reports of the synthesis of **11**,^38,39^ where the dye was prepared in low yield using standard acid-mediated chemistry. The lactol strategy proved superior with diphenyl ether **12** (Scheme S1b) reacting with **5** to give **11** in 66% yield (Scheme 2).

**Scheme 3:**
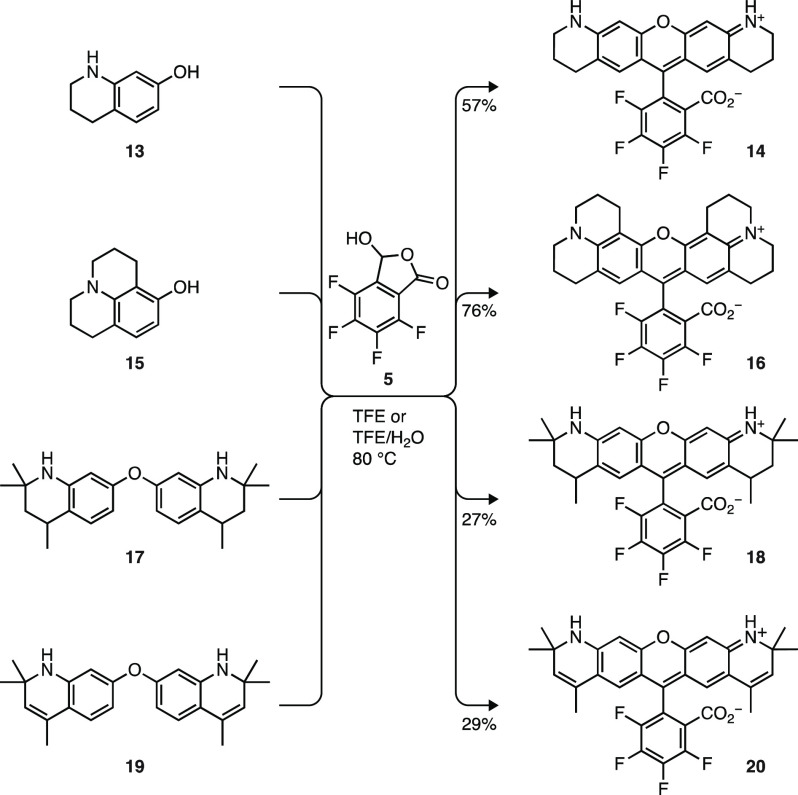
Synthesis of Rhodamines via Lactol Condensation

We then explored the generality of the lactol synthesis for other fluorinated rhodamine dyes (Scheme 3). In addition to diphenyl ethers such as **12**, 3-aminophenol derivatives were also accommodated by this chemistry. Reaction of 7-hydroxytetrahydroquinoline (**13**) with **5** afforded 4,5,6,7-tetrafluoro-Q-rhodamine (FRh_Q_, **14**). 8-Hydroxyjulolidine (**15**) reacted with **5** to give the fluorinated derivative of rhodamine 101 (FRh_101_, **16**).^46,47^ Diphenyl ether **17** (Scheme S1b) produced **18**, a fluorinated analog of ATTO 550 that we called “Janelia Fluor 563” (JF_563_). Finally, reaction of **5** and ether **19** (Scheme S1b) gave **20**, the *des*-methyl analog of compound **11**.^38^ We note that reactions using secondary amine reactants **13**, **17**, and **19** afforded lower yields (27–57%) than those using fully substituted anilines **9**, **12**, and **15** (66–85%; Schemes 2 and 3), likely due to side reactions involving the secondary amine and aldehyde tautomer of **5**. We also prepared several nonfluorinated analogs of these dyes using the lactol condensation (Scheme S2).

### Lactol Condensation for the Synthesis of Fluorinated Si-Rhodamines

Substitution of the xanthene oxygen in rhodamines for a silicon moiety causes a substantial bathchromic shift of ∼100 nm, making Si-rhodamines useful fluorophores that are excited by long-wavelength light.^21,29,31−34,48−50^ This substitution also decreases the *K*_L–Z_ compared to standard oxygen-containing rhodamine dyes. As mentioned above, some Si-rhodamines such as JF_646_ (**1**; Figure 1a) preferentially adopt the nonfluorescent lactone form in aqueous solution and can be used to create chromogenic/fluorogenic labels.^15,17,18^ Installation of fluorine atoms on the pendant phenyl ring shifts the lactone–zwitterion equilibrium toward the zwitterionic form, yielding JF_669_ (**2**; Figure 1a), a dye that shows higher ε values and improved bioavailability compared to the nonfluorinated congener **1**.^14,16^

**Scheme 4:**
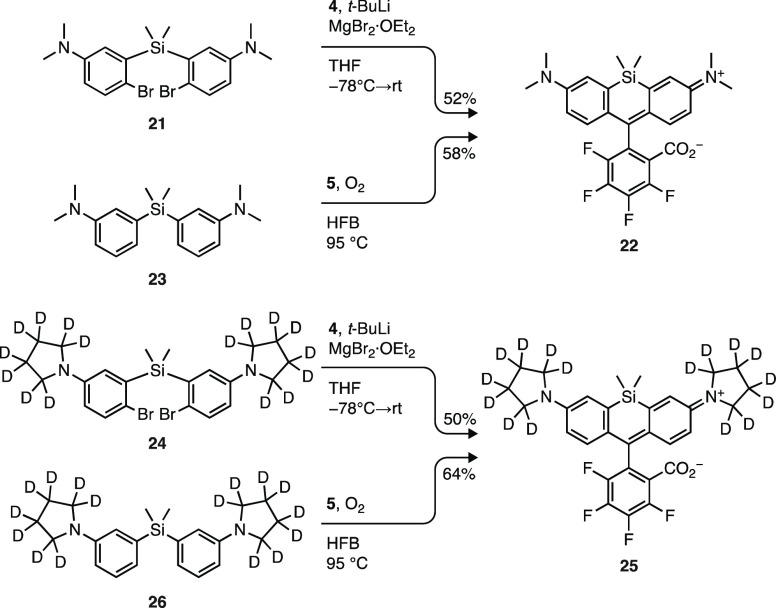
Synthesis of Fluorinated Si-Rhodamines

Given the utility of JF_669_, we investigated the condensation of fluorinated lactol **5** to synthesize fluorinated Si-rhodamine dyes, again comparing this chemistry to the bis(arylmetal) addition strategy (Scheme 4). We note that this lactol condensation approach has not been used to make any Si-rhodamines, fluorinated or otherwise. As described previously, metal/bromide exchange of dibromide **21** and addition to anhydride **4** gives the fluorinated derivative of Si-tetramethylrhodamine^29^ (SiRF_667_; **22**) in moderate yield (52%). Application of the published protocol^42^ for the lactol condensation using 2,2,2-trifluoroethanol (TFE) at 80 °C and a concentration of 0.05 M (Schemes 2 and 3) gave poor yields of the silicon-containing variants. To remedy this problem, we explored different reaction conditions using diarylsilane **23** as a model reactant (Table S2). Replacing TFE with the higher-boiling solvent 2,2,3,3,4,4,4-heptafluoro-1-butanol (HFB) allowed reaction at elevated temperature (95 °C). This solvent change, combined with performing the condensation at higher concentration (0.2–0.3 M), afforded Si-rhodamine **22** in a similar yield (58%) to the more operationally complex reaction involving the bis(arylmetal) species (Scheme 4). Reaction of pyrrolidine-*d*_8_-containing dibromide **24** (Scheme S1a) with **4** produced fluorophore **25** (JFX_673_) in 50% yield; synthesis of the same dye via the straightforward condensation of silane **26** (Scheme S1a) and lactol **5** gave a higher yield (64%).

**Scheme 5:**
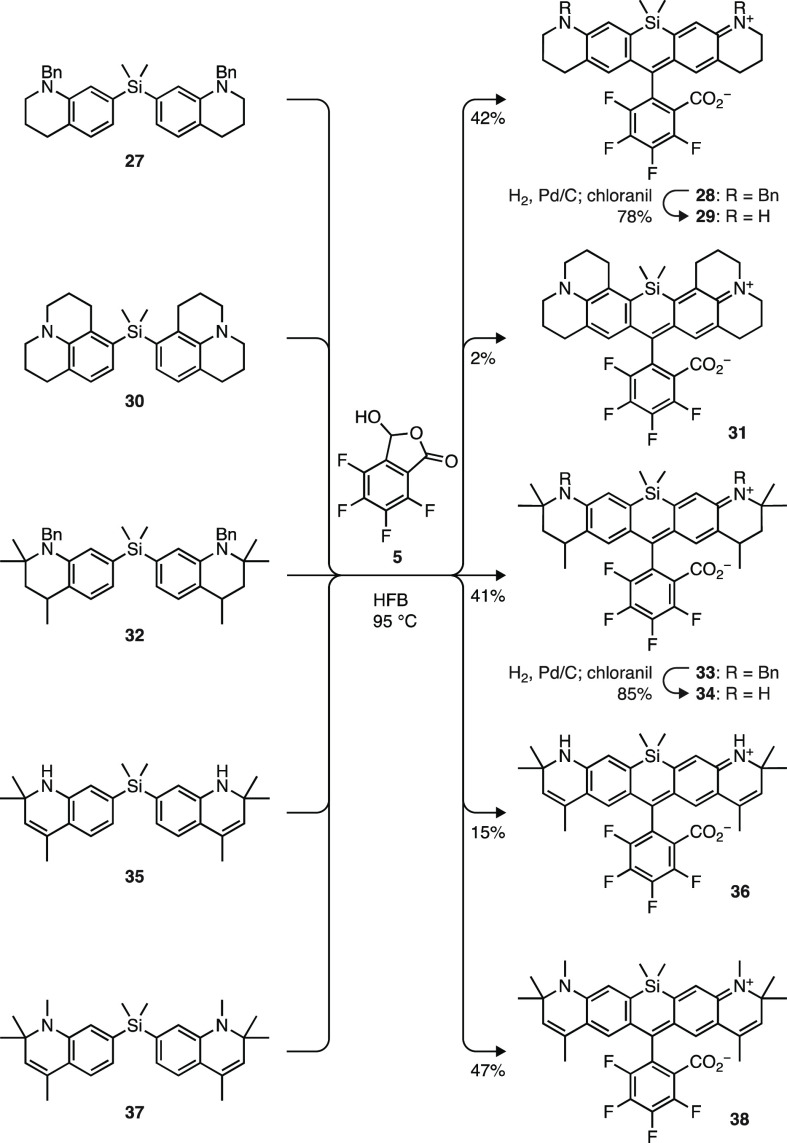
Synthesis of Si-Rhodamines via Lactol Condensation

We expanded our investigation of the lactol condensation chemistry to synthesize other fluorinated Si-rhodamines (Scheme 5). As with oxygen-containing rhodamines (Scheme 3), the use of secondary amine reactants gave lower yields of the fluorophore products. This problem prompted protection of the aniline nitrogens in some cases to mitigate side reactions driven by the higher reaction temperature. *N*-Benzyl-protected silane **27** (Scheme S1c) gave Si-rhodamine **28** in 42% yield; hydrogenation of **28** yielded fluorinated Si-Q-rhodamine (FSiRh_Q_, **29**). Compound **30** (Scheme S1c) combined with **5** to furnish dye **31**, which we called “JF_698_”, albeit in a very low yield (2%) due to competing protodesilylation of **30**. We also used these conditions to prepare the silicon analogs of rhodamines **11**, **18**, and **20** (Schemes 2 and 3). Condensation of **32** (Scheme S1c) with **5** afforded dibenzyl dye **33** in 41% yield, which was deprotected to give Si-rhodamine **34**. Direct reaction of **35** (Scheme S1c) provided a 15% yield of dye **36**; here, the dihydroquinoline moieties precluded the use of *N*-benzyl protecting groups, which resulted in the relatively low yield. Reaction of silane **37** (Scheme S1c) and phthalaldehydic acid **5** formed Si-rhodamine **38** (47% yield). Attempts to synthesize dihydroquinoline dyes such as **38** via the dibromide/bis(arylmetal) route failed. We note that the yields for some of these reactions were modest, and in a few cases—most notably JF_698_ (**31**)—the dibromide strategy gave better results (Scheme S3). Nonetheless, these optimized, simple conditions using HFB and elevated temperature were general for the synthesis of fluorinated dyes and also useful for expediently accessing various nonfluorinated Si-rhodamines (Scheme S2).

### Fluorinated Carborhodamines and Other Dyes using Li/H Exchange

**Scheme 6:**
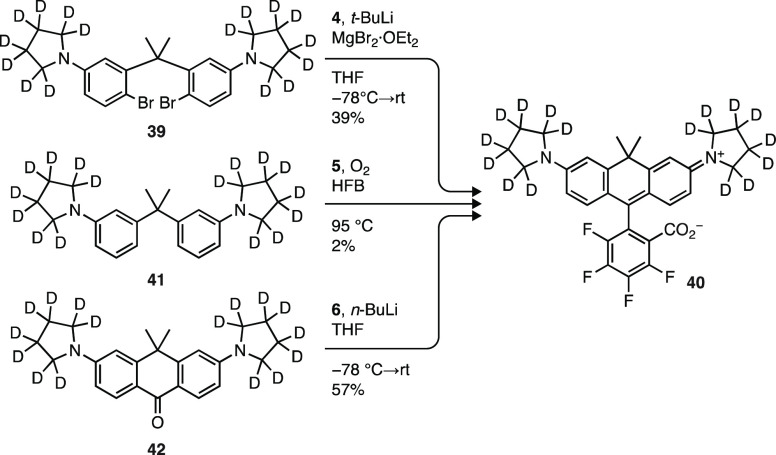
Synthesis of Fluorinated Carborhodamines

We then explored the synthesis of a novel class of fluorophore: carbon-containing analogs of rhodamines and fluoresceins bearing a fluorinated pendant phenyl ring. Relative to the standard oxygen-bridged rhodamine dyes, the *gem*-dimethyl carbon substituent elicits a ∼60 nm bathochromic shift in absorption and emission wavelengths and a shift toward lower *K*_L–Z_ values.^41,51,52^ As with other rhodamines, fluorination of carborhodamines should elicit a further bathochromic shift in λ_abs_ and λ_em_ and increase *K*_L–Z_, leading to higher absorptivity. We initially attempted to access fluorinated carborhodamines using dibromide starting materials (Scheme 1a) or lactol condensation chemistry (Schemes 1d and 6). Li/Br exchange of deuterated dibromide **39** (Scheme S4a) and addition to tetrafluorophthalic anhydride **4** gave carborhodamine **40** (JFX_637_) in a modest yield (39%). This route was complicated by the longer, lower-yielding, and relatively complex synthesis of **39** compared to those of the oxygen and silicon dibromides (e.g., **7** and **24**; Schemes 2 and 4). Use of the lactol condensation conditions to react **41** (Scheme S4a) with **5** was unsuccessful, yielding only a small amount (<2%) of dye **40** even after extended reaction time (>72 h).

These issues with existing synthetic methods prompted the consideration of a novel method to prepare carborhodamine dyes. In previous work, we synthesized carbofluoresceins by reacting aryl Grignards with dihydroxyanthrones; these could be converted to carborhodamines using Pd-catalyzed cross-coupling.^41^ The use of a Grignard was necessary to accommodate *t*-butyl-protected carboxyl groups, but this arylmagnesium species was not reactive enough to add to more electron-rich diaminoanthrones and directly generate rhodamine dyes.^32^ Aryllithium reagents are more commonly used for addition to diaminoanthrones due to their increased nucleophilicity, which limits functional group compatibility. Aside from one recent example,^53^ all existing syntheses of carboxy-substituted carbo- and Si-rhodamines involve lithium/bromide exchange on a rigorously protected phthalic acid equivalent and addition of the resulting aryllithium species to a diaminoanthrone.^29−34^ We surmised that later-stage incorporation of the 6-carboxyl group using the MAC chemistry approach would minimize protecting group issues and simplify the use of the more reactive aryllithium reagents; the bespoke fluorinated phenyl ring could also facilitate the exchange, transmetalation, or deprotonation needed to form the desired organometallic species. Nonetheless, previous attempts to generate tetrafluoroaryllithium reagents containing protected 2-carboxyl groups via metal/halide exchange have proven unsuccessful.^21^ We found that direct metalation (i.e., deprotonation or Li/H exchange) of unprotected 2,3,4,5-tetrafluorobenzoic acid (**6**) with 2 equiv of *n*-butyllithium^43^ (Scheme 1e) generated a dianionic aryllithium species that underwent facile addition to anthrone **42** (Scheme S5a), affording deuterated rhodamine JFX_637_ (**40**) in a higher yield (57%) compared to the other two synthetic methods (Scheme 6).

**Scheme 7:**
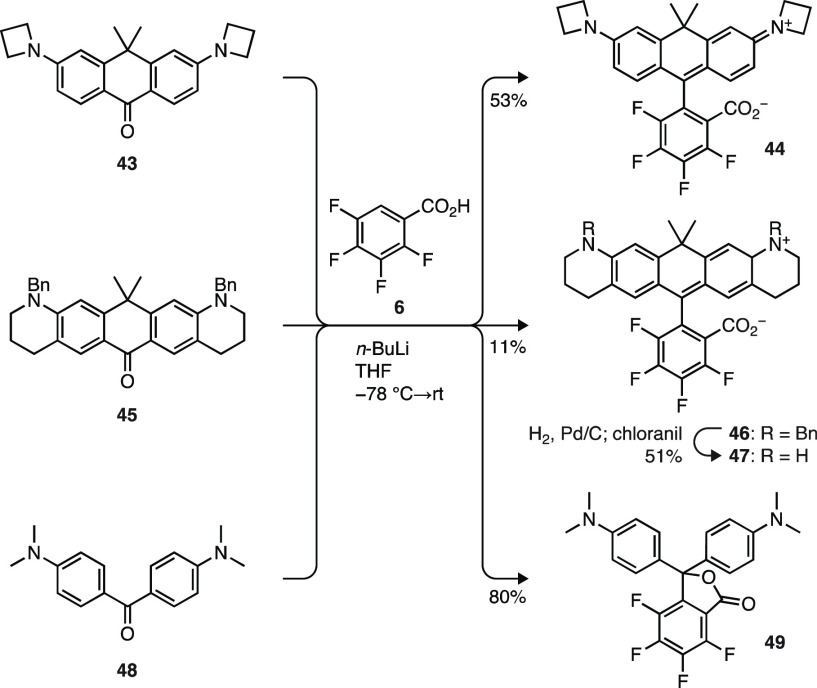
Synthesis of Carborhodamines and Fluorinated Malachite Green Lactone using Li/H Exchange

**Scheme 8:**
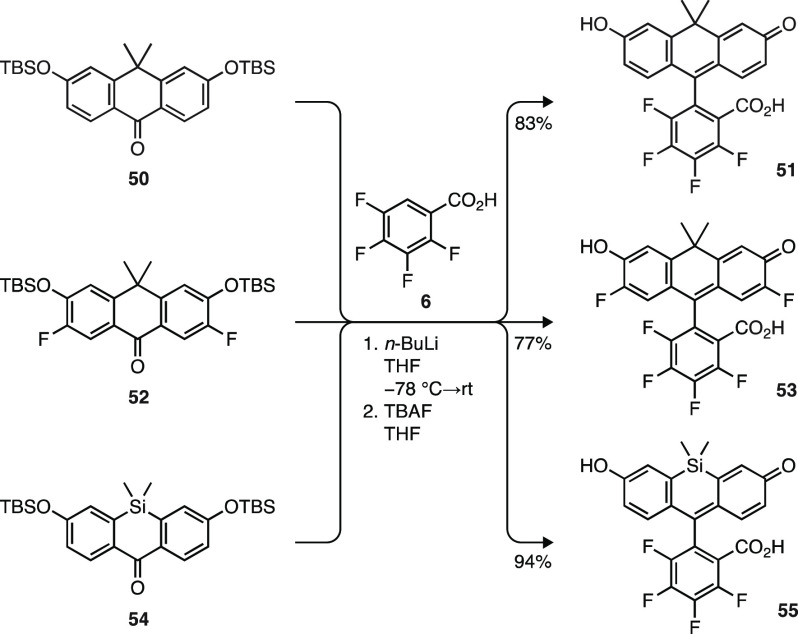
Synthesis of Carbofluoresceins and Si-Fluoresceins using Li/H Exchange

We then applied this new Li/H exchange approach to prepare other fluorinated dyes. Use of anthrone **43** (Scheme S5a) gave the novel azetidinyl dye **44** (JF_632_; Scheme 7) in reasonable yield (53%). The dibenzyl anthrone **45** (Scheme S5b) afforded compound **46**, which could be deprotected to yield 4,5,6,7-tetrafluoro-carbo-Q-rhodamine (FCRh_Q_, **47**). Similarly, reaction of Michler’s ketone (**48**) with the dianion of **6** furnished the fluorinated analog of Malachite Green lactone (FMGL; **49**) in excellent yield (80%). This chemistry could also be used to synthesize fluorescein analogs though a two-step, high-yielding protocol (77–94%; Scheme 8). Addition of lithiated **6** to di(silyloxy)anthrone **50** yielded the fluorinated carbofluorescein **51** after TBS deprotection with TBAF. Use of anthrone **52** in this sequence gave highly fluorinated **53**, an analog of Virginia Orange.^54^ This method could be extended to Si-anthrone **54** to provide the known,^14^ pH-sensitive, fluorinated Si-fluorescein **55** in high yield. These examples demonstrate the broad scope of 2,3,4,5-tetrafluorobenzoic acid (**6**) lithiation/addition in the synthesis of both rhodamine and fluorescein dyes (Scheme S6).

### Lewis Acid-Catalyzed Condensation for Carborhodamine Synthesis

**Scheme 9:**
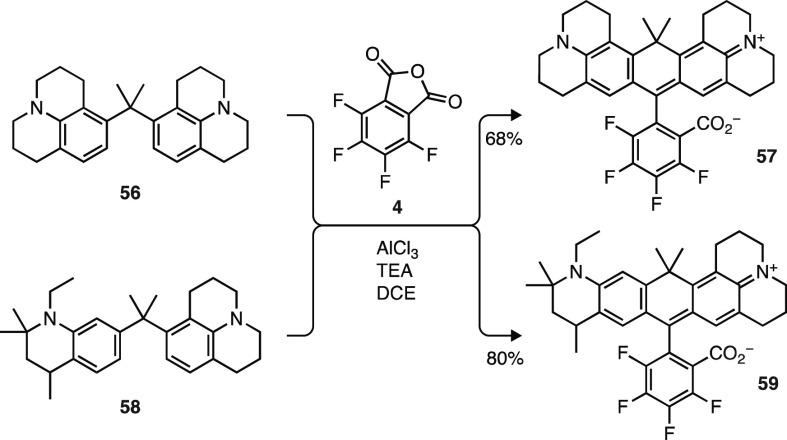
Synthesis of Carborhodamines by Lewis Acid-Catalyzed Condensation

We then further expanded the collection of fluorinated carborhodamines. Although the dibromide and site-selective lithiation routes (Scheme 1a,e) give good yields of carborhodamine dyes with compact substituents such as pyrrolidine and azetidine (Schemes 6 and 7), this chemistry proved ineffective for dyes with highly substituted, fused-ring auxochromes such as those derived from reduced quinolines or julolidine.^34,55^ Inspired by a brief report in the patent literature,^56^ we explored a different approach, returning to classic acid-catalyzed chemistry but using the Lewis acid AlCl_3_ (Scheme 9). Reaction of *bis*-julolidine **56** (Scheme S4a) with tetrafluorophthalic anhydride (**4**) in the presence of AlCl_3_ afforded a 68% yield of the fluorinated carbon-containing analog of rhodamine 101 (JF_660_, **57**). Likewise, AlCl_3_-mediated condensation of **58** (Scheme S4b) and **4** gave **59**, a fluorinated analogue of ATTO 647N, in excellent yield (80%). We also achieved high-yielding syntheses of the *des*-fluoro analogs of rhodamines **57** and **59** using this chemistry (Scheme S7). In contrast, all attempts to synthesize **57** or **59** through addition of lithiated **6** to anthrones containing a julolidine motif^57^ or via dibromination of **56** or **58** failed to provide appreciable amounts of desired carborhodamine products.

### Pd-Catalyzed Cross-Coupling to Prepare Unsubstituted and *N*-Aryl Rhodamines

**Scheme 10:**
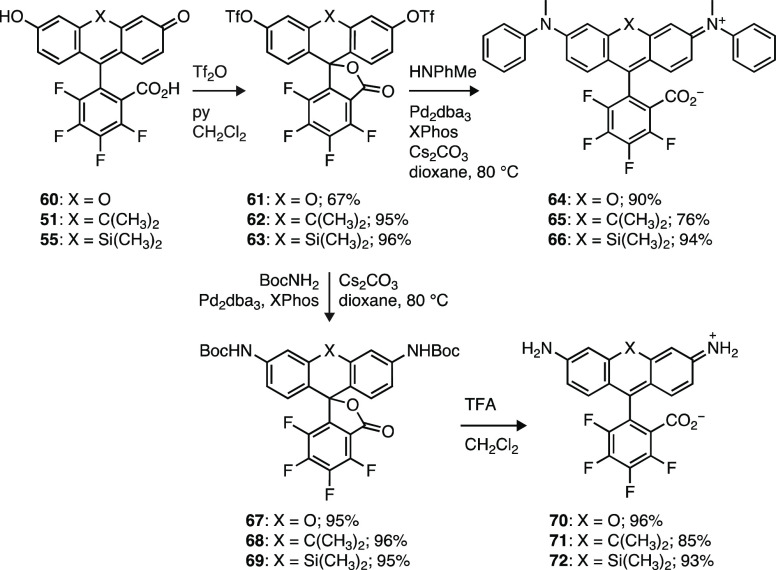
Synthesis of Rhodamines from Fluoresceins using Pd-Catalyzed Cross-Coupling

In a prior report, we demonstrated that cross-coupling of fluorescein ditriflates with amines is an efficient method to synthesize rhodamine dyes.^40^ Nevertheless, use of the known^19^ 4,5,6,7-tetrafluorofluorescein (**60**) or analogs such as **51**, **53**, and **55** (Scheme 8) requires special considerations due to the electrophilicity of the perfluorinated arene. In our hands, cross-coupling reactions using primary or secondary aliphatic amine partners were unsuccessful due to facile substitution reactions by these nucleophilic species on the tetrafluoroaryl moiety^16,58,59^ under the requisite reaction conditions (Cs_2_CO_3_, 80–100 °C). Less nucleophilic aromatic amines or *N*-acyl derivatives are compatible with these conditions, allowing for the synthesis of novel dyes (Scheme 10). Reaction of **60**, the carbon-containing analog **51**, and Si-fluorescein **55** with triflic anhydride yielded ditriflates **61**–**63**. Cross-coupling with *N*-methylaniline provided *N*-arylrhodamines **64**–**66**. Likewise, diacylrhodamines **67**–**69** were accessed in nearly quantitative yield through cross-coupling of the fluorescein ditriflates with *t*-butyl carbamate. Deprotection of the *t*-Boc groups with TFA gave rhodamine 110 derivatives **70** (FRh_110_), **71** (FCRh_110_), and **72** (FSiRh_110_). We note that fluorinated rhodamine 110 variants are largely unknown, with only a brief report in the patent literature^38^ detailing the synthesis of 4,5,6,7-tetrafluoro-Rh_110_ (**70**) in low yield (∼5%) through an acid-catalyzed synthesis. These results demonstrate that fluorinated fluorescein ditriflates can be used in cross-coupling reactions, albeit with a reduced scope limited to less nucleophilic nitrogen reactants.

### Spectral Properties of Fluorophores

Combined with previous investigations,^14,16−18,32,40,41,45^ the work described above (Schemes 1–10 and Schemes S2, S6, and S7) represents a comprehensive collection of fluoresceins and rhodamines containing O, C, or Si bridging substituents, fluorinated or nonfluorinated pendant phenyl rings, and different auxochrome groups. We began our investigation of their spectral properties by examining the fluorescein variants, measuring the λ_abs_, λ_em_, extinction coefficient (ε), and fluorescence quantum yield (Φ_f_) of each dye (Table 1, Figure S1). Fluorescein and many of its derivatives exhibit pH-sensitive spectral properties, so we also determined the p*K*_a_ values and investigated the cooperativity of the pH-mediated spectral changes by determining the Hill coefficients (η_H_) calculated from the pH titration data. Consistent with previous work describing 4,5,6,7-tetrafluorofluorescein (**60**)^19^ and 4,5,6,7-tetrafluoro-Si-fluorescein (**55**),^14^ the tetrafluorophenyl ring system in dyes **51** and **53** elicited a >15 nm bathochromic shift in λ_abs_ and λ_em_ and a lower p*K*_a_ relative to the parent carbofluorescein (**74**) and Virginia Orange (**78**) dyes. Fluorophores **51** and **53** showed a noncooperative pH-driven transition with η_H_ ≈ 1. This is in contrast to the behavior of carbofluoresceins **74** and **78**, which do not contain the perfluorinated ring system and show cooperative transitions (η_H_ > 1) from a colored to colorless species as pH decreases.^41,54^ The Si-fluoresceins **55** and **80** maintain a cooperative pH titration even with the fluorinated ring system installed. These data reveal a subtle interplay between the nucleophilicity of the *ortho*-carboxyl group and the propensity of the dye to lactonize that controls the cooperative quinoid → lactone transition upon protonation. This understanding will help guide the future development of pH sensors based on red-shifted fluorescein analogs.^41,54,60^

**Table 1:**
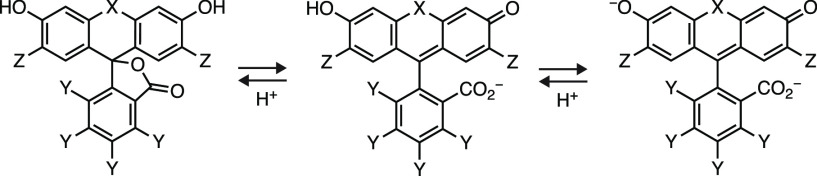

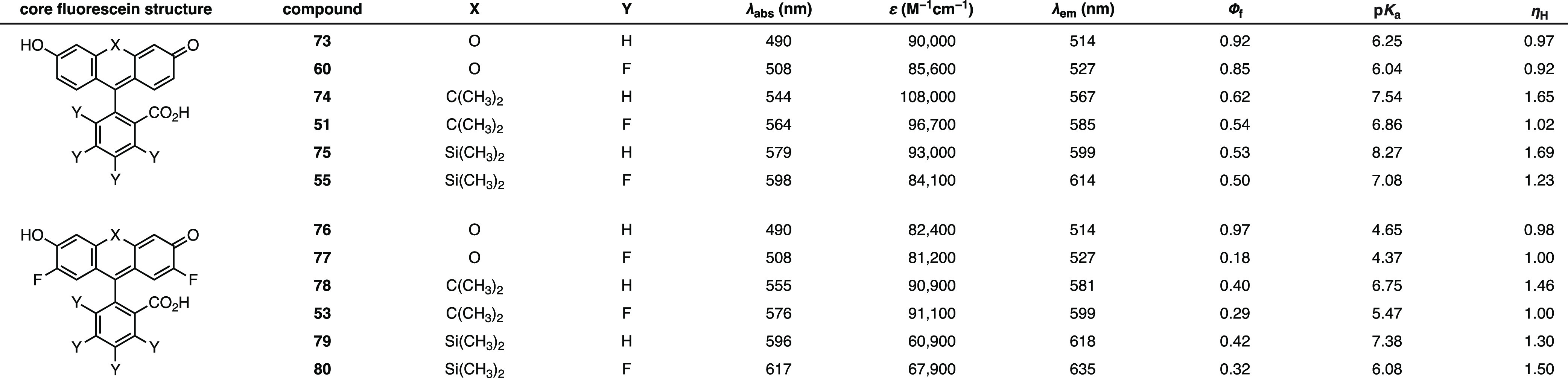
Spectral Properties of Fluorescein Derivatives^a^

a All properties were measured in 0.1 N NaOH.

We then investigated the rhodamines (Table 2, Figures S2 and S3) measuring the standard spectral properties (λ_abs_, ε, λ_em_, and Φ_f_) along with *K*_L–Z_.^16^ Consistent with previous data, we observed that fluorination of the pendant phenyl ring increases λ_abs_ and λ_em_ by 15–25 nm; the fluorinated dyes also exhibit a ∼5 nm smaller Stokes shift compared to their *des*-fluoro analogs. This modification also modestly increases ε and decreases Φ_f_. The lower quantum yields could be caused by the energy gap law^61^ or photoinduced electron transfer (PeT) from the xanthene system to the electron-deficient pendant ring;^62^ plotting Φ_f_ vs λ_abs_ showed no substantial difference in trends between fluorinated and nonfluorinated dyes (Figure S4a) thereby supporting the energy gap law hypothesis. Carborhodamine **86** (JF_608_) shows λ_abs_/λ_em_ = 608 nm/631 nm with ε = 99,000 M^–1^cm^–1^ and Φ_f_ = 0.67. The fluorinated analog JF_632_ (**44**) exhibits red-shifted spectra (λ_abs_/λ_em_ = 632 nm/649 nm), a higher absorptivity (ε = 139,000 M^–1^cm^–1^), and a lower Φ_f_ = 0.54. These trends are mirrored in the analogous deuterated JFX dyes **88** and **40**. Likewise, the previously reported Si-Q-rhodamine^32^ (**95**) exhibits λ_abs_/λ_em_ = 637 nm/654 nm with ε = 77,000 M^–1^cm^–1^ and Φ_f_ = 0.38. The fluorinated analogue **29** shows a bathochromic shift with λ_abs_/λ_em_ = 659 nm/675 nm, higher ε = 124,000 M^–1^cm^–1^, and lower Φ_f_ = 0.25. These trends in λ_abs_ and ε are also present in nonfluorescent *N*-arylrhodamines. Rhodamine **90** displays λ_abs_ = 549 nm with modest absorptivity (ε = 16,000 M^–1^cm^–1^); incorporation of fluorine atoms to give **64** increases the absorption maximum and extinction coefficient (λ_abs_ = 570 nm; ε = 81,700 M^–1^cm^–1^).

**Table 2:**
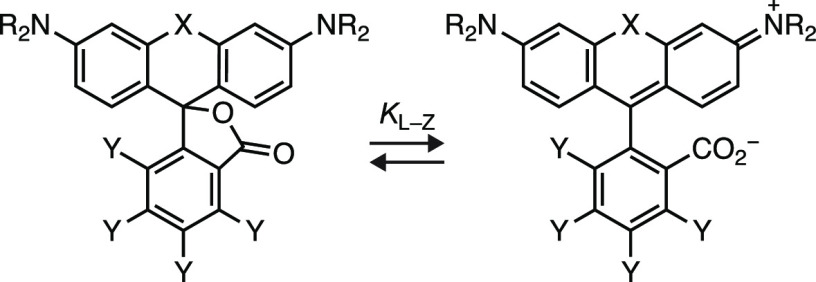

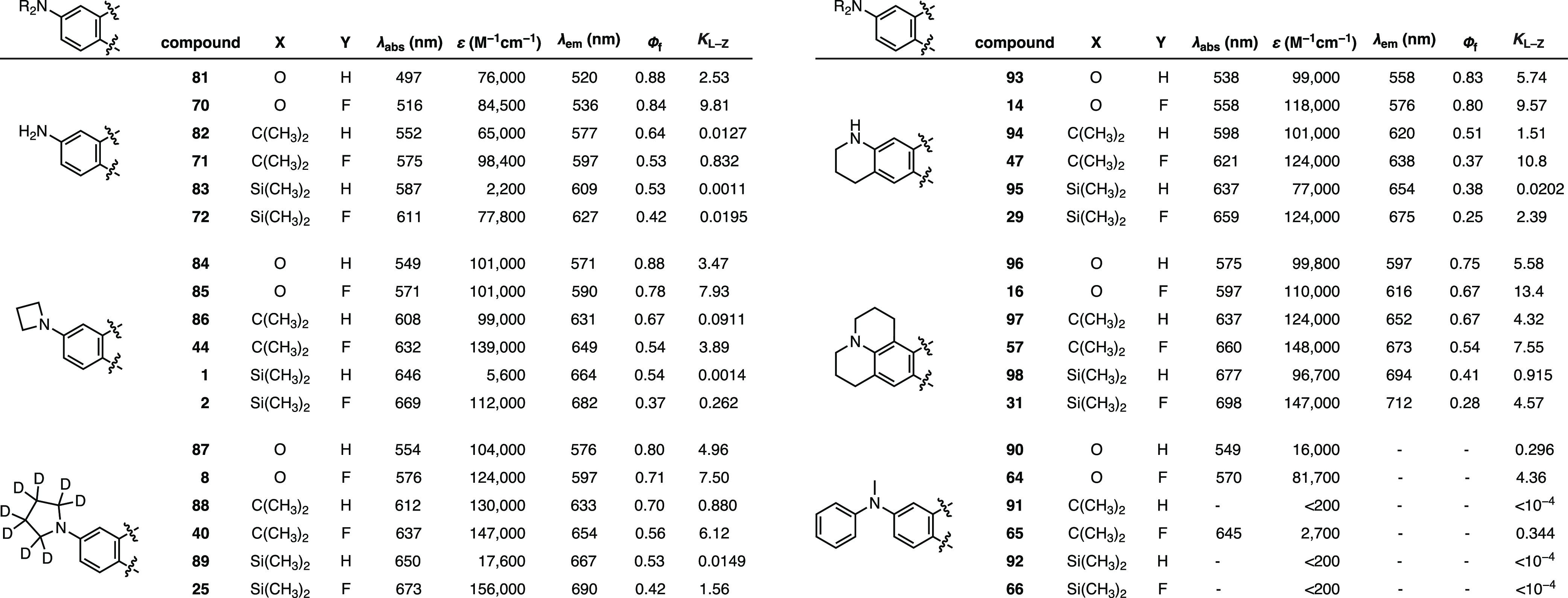
Spectral Properties of Rhodamine Derivatives^a^

a All properties were measured in 10 mM HEPES, pH 7.3 except for *K*_L-Z_, which was determined in 1:1 dioxane:H_2_O.

**Figure 2:**
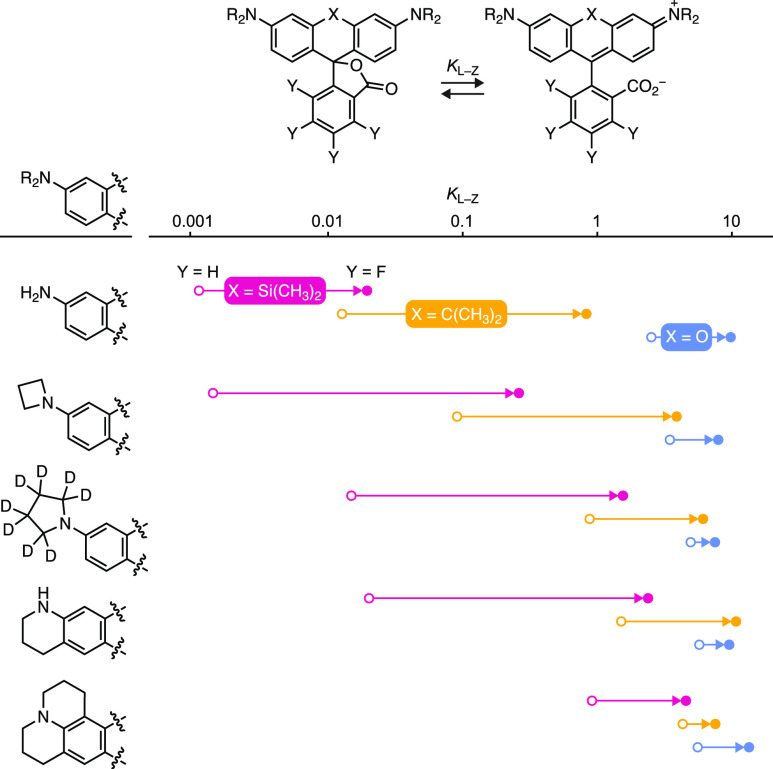
Effect of the auxochrome type and fluorination on *K*_L–Z_. Plot of *K*_L–Z_ vs rhodamine auxochrome structure; arrows show a shift in *K*_L–Z_ upon fluorination (open circles: Y = H; closed circles: Y = F); colors indicate Si-rhodamines (X = Si(CH_3_)_2_; magenta), carborhodamines (X = C(CH_3_)_2_; yellow), or rhodamines (X = O; blue).

We then examined the lactone–zwitterion equilibrium across the different dye series (Table 2); *K*_L–Z_ was measured in 1:1 (v/v) dioxane:water mixtures. These conditions provide the broad range of values necessary for identifying structure–activity relationships.^18^ Plotting the auxochrome structure vs *K*_L–Z_ (Figure 2) showed two general trends. First, fluorination universally increases *K*_L–Z_; this shift ranged from 2 orders of magnitude (e.g., **1** → **2**) to 2-fold (e.g., **97** → **57**) depending on dye structure. Second, *K*_L–Z_ is strongly dependent on both the bridging substituent (Si(CH_3_)_2_; C(CH_3_)_2_; and O) and the auxochrome moiety. The effect of the bridging substituent on *K*_L–Z_ is well-established but the dependence on the auxochrome groups—especially highly electron-donating moieties—has not been rigorously explored.^15,16,63^ Although the Hammett constants^64^ for most of these functionalities are unknown, the electron-donating capability of the auxochrome groups can be ranked as NH_2_ < azetidine < pyrrolidine < tetrahydroquinoline < julolidine based on data from substituted azobenzene dyes.^65,66^ The *K*_L–Z_ values follow this trend with Si-rhodamines SiRh_110_ (**83**) and JF_646_ (**1**) showing *K*_L–Z_ ≈ 10^–3^, JFX_650_ (**89**) and SiRh_Q_ (**95**) exhibiting *K*_L–Z_ ≈ 10^–2^, and the julolidine-containing SiRh_101_ (**98**) giving *K*_L–Z_ ≈ 1. The correlation between the electron-donating character of the auxochrome and *K*_L–Z_ is also observed for the carborhodamines and rhodamine dyes at commensurately higher *K*_L–Z_ values (Figure 2). *N*-Aryl auxochromes substantially shift the lactone–zwitterion equilibrium lower. Rhodamine **90** had a relatively small *K*_L–Z_ = 0.296, but fluorination shifted the equilibrium constant higher; **64** showed *K*_L–Z_ = 4.36 (Table 2). Overall, these data show that rhodamines that typically favor the lactone form, such as SiRh_110_ (**83**; ε = 2,200 M^–1^cm^–1^, *K*_L–Z_ = 0.0011), can be tuned through cooperative structural modifications—introduction of electron-donating auxochromes and fluorine substituents—to yield JF_698_ (**31**), a dye that exhibits high absorptivity in aqueous solution (ε = 147,000 M^–1^cm^–1^, *K*_L–Z_ = 4.57). We also note that dyes containing electron-donating substituents, such as julolidine, exhibit higher and more compressed *K*_L–Z_ ranges. This makes such scaffolds attractive for constructing bright, highly absorbing labels—ε is correlated with *K*_L–Z_ (Figure S4b)—but could limit efforts to fine-tune such fluorophores to access properties such as improved bioavailability or chromogenicity (Figure 1a).

Finally, we compared the oxygen- and silicon-containing rhodamines with more complicated auxochrome substituents (Table 3, Figure S5). Again, the fluorine modification universally elicited longer λ_abs_ and λ_em_, a smaller Stokes shift, larger ε and *K*_L–Z_, and lower Φ_f_. For example, tetrahydroquinoline rhodamine **99**—the free dye form of ATTO 550—shows λ_abs_/λ_em_ = 542 nm/564 nm with ε = 112,000 M^–1^cm^–1^, Φ_f_ = 0.83, and *K*_L–Z_ = 6.30. The fluorinated analogue **18** (JF_563_) exhibits red-shifted spectra (λ_abs_/λ_em_ = 563 nm/581 nm), higher *K*_L–Z_ = 15.3 and absorptivity (ε = 125,000 M^–1^cm^–1^), and slightly lower Φ_f_ = 0.73. The tetrahydroquinoline-containing Si-rhodamines **100** and **34** followed this trend. The dihydroquinoline dyes (**11**, **20**, **36**, **38**, and **101**–**104**) exhibited lower *K*_L–Z_ values than the tetrahydroquinoline dyes (**18**, **34**, **99**, and **100**) and the structurally simpler Q-rhodamines (**14**, **29**, **93**, and **95**), illustrating that this extended conjugated system shifts *K*_L–Z_ toward the lactone form (Figure S6). These data also revealed that Si-rhodamines containing dihydroquinoline systems (**102**, **36**, **104**, and **38**) possess substantially lower Φ_f_ ≤ 0.03 compared to tetrahydroquinoline Si-dyes **100** and **34** (Φ_f_ ≈ 0.3), and the directly analogous oxygen-containing rhodamines **101**, **20**, **103**, and **11 (**Φ_f_ = 0.64–0.83). This suggests that the extended conjugation of the dihydroquinoline-containing dye scaffold promotes a nonradiative decay pathway in the Si-rhodamine system, making such dyes less attractive as fluorescent labels.

**Table 3:**
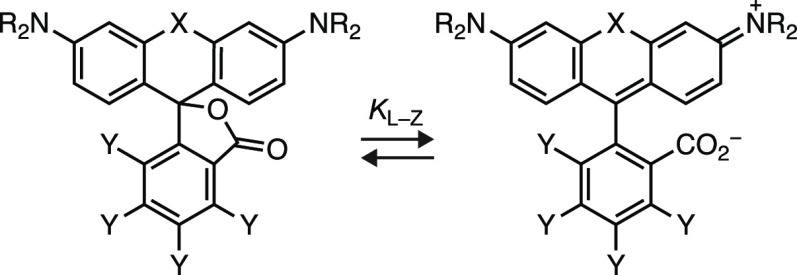

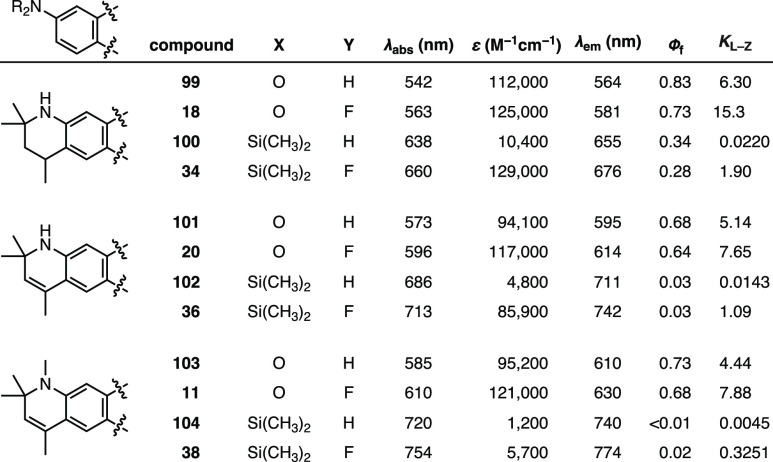
Spectral Properties of Quinolinyl Rhodamines^a^

a All properties were measured in 10 mM HEPES, pH 7.3 except for *K*_L-Z_, which was determined in 1:1 dioxane:H_2_O.

### Synthesis of HaloTag Ligands using MAC Chemistry

With this expanded collection of fluorinated dyes in hand, we tested the MAC chemistry derivatization strategy, preparing the HaloTag ligands of **8** (JFX_576_), **11**, **18** (JF_563_), **25** (JFX_673_), **29** (FSiRh_Q_), **31** (JF_698_), **36**, **38**, **40** (JFX_637_), **44** (JF_632_), **49** (FMGL), **51**, **55**, **57** (JF_660_), **59** (JF_657_), **60**, **64**–**66**, and **70**–**72**. The MAC chemistry proceeded in good-to-excellent yield regardless of the dye reactant structure (Scheme S8). We note that this approach was successful even with fluoresceins **51** and **55**, which bear nucleophilic phenolic substituents that could react with the electrophilic ring system or acyl cyanide intermediate. We evaluated the HaloTag labeling rates of the matched pairs of nonfluorinated/fluorinated dye ligands: **84**_**HTL**_/**85**_**HTL**_, **81**_**HTL**_/**70**_**HTL**_, **86**_**HTL**_/**44**_**HTL**_, **87**_**HTL**_/**8**_**HTL**_, and **88**_**HTL**_/**40**_**HTL**_ where a modest decrease of 4–14-fold in rate was observed for the fluorinated compounds (Figure S7, Table S3). The dye ligand–HaloTag conjugates proved stable regardless of fluorination (Figure S8), the rhodamine 110 analogs efficiently labeled HaloTag fusion proteins in cells (Figure S9), and the novel fluorinated carborhodamines **44** (JF_632_) and **40** (JFX_637_; Table 2) showed comparable performance to previously reported Si-rhodamine dyes JF_646_ (**1**_**HTL**_) and JFX_650_ (**89**_**HTL**_) in single-particle tracking (SPT) experiments (Figure S10). Together, these data show that fluorination does not adversely affect the performance of dyes in live-cell experiments.

### Live-Cell-Compatible Analog of ATTO 647N

**Figure 3:**
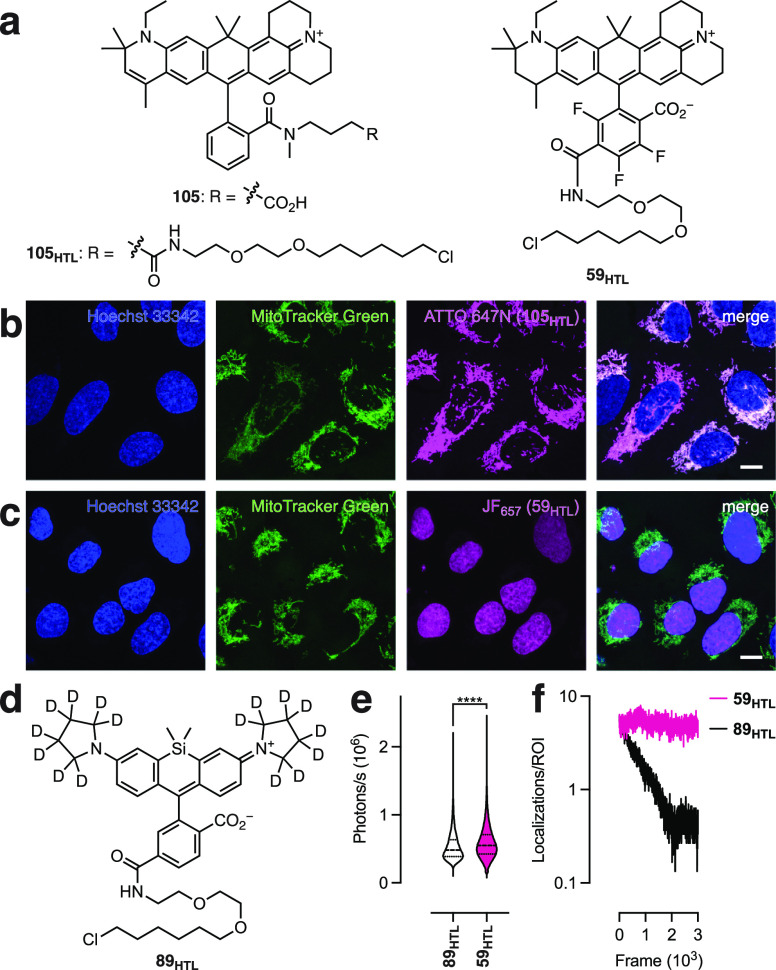
Performance of JF_657_. (a) Chemical structures of ATTO 647N (**105**), the ATTO 647N-HaloTag ligand (**105**_**HTL**_), and the JF_657_–HaloTag ligand (**59**_**HTL**_). (b, c) Fluorescence images of live U2OS cells expressing histone H2B–HaloTag fusion proteins and labeled with Hoechst 33342 to stain the nucleus, MitoTracker Green to stain mitochondria, and either **105**_**HTL**_ (b) or **59**_**HTL**_ (c); scale bars: 10 μm. (d) Chemical structure of JFX_650_-HaloTag ligand (**89**_**HTL**_). (e) Violin plot of intensity (photons/s) of ligands **89**_**HTL**_ and **59**_**HTL**_ in SPT experiments; **** indicates *P* < 0.0001 by the unpaired *t* test. (f) Plot of localizations per region of interest (ROI) vs frame for ligands **89**_**HTL**_ and **59**_**HTL**_ in SPT experiments. SPT imaging was performed at 100 Hz (10 ms/frame).

We chose three new fluorinated dye compounds to examine more closely for biological imaging applications. We first focused on the novel carborhodamine **59** (Scheme 9), which is a fluorine-containing analog of ATTO 647N (**105**; Figure 3a). The “famous”^67^ ATTO 647N is perhaps the best dye for *in vitro* single-molecule imaging and has found widespread use as a label for advanced imaging experiments.^52,57,68^ ATTO 647N conjugates have not been used in live-cell imaging experiments, however, due to structural constraints. A key design principle used in many ATTO dyes involves amidation of the *ortho*-carboxyl group on the pendant ring with 4-(methylamino)butanoic acid. This conjugation strategy does three things: (i) elicits a bathochromic shift in spectral properties to give λ_abs_ > 640 nm, longer than the parent, unmodified dye (λ_abs_ = 632 nm; Figure S11); (ii) enforces the visible-absorbing form by blocking lactonization; and (iii) provides a carboxylic acid moiety for bioconjugation thus circumventing issues with isomeric mixtures. This strategy has an unfortunate side effect, however, as it removes the *ortho*-carboxylate negative charge, resulting in cationic species after derivatization. The ATTO 647N-HaloTag ligand^69^ (**105**_**HTL**_) does not show appreciable labeling in the nucleus when applied to live cells expressing histone H2B–HaloTag fusion proteins and instead stains mitochondria (Figure 3b). This is consistent with the propensity of cationic rhodamine derivatives to accumulate in the mitochondria of living cells.^26^

Our approach of fluorinating fluorophores mirrors the spectral and structural advantages of using the *ortho*-carboxyl group for bioconjugation but does not produce a cationic species. The fluorine substitution confers a bathochromic shift in λ_abs_/λ_em_ and works in concert with the julolidine and tetrahydroquinoline auxochromes to increase absorptivity and brightness by shifting the lactone–zwitterion equilibrium toward the zwitterionic form. Introduction of a reactive handle via the straightforward MAC chemistry approach preserves the zwitterionic character of the dye, which should minimize unwanted mitochondrial accumulation. Both diastereomers of **59** displayed identical spectral properties: λ_abs_ = 657 nm, ε = 137,000 M^–1^cm^–1^, λ_em_ = 672 nm, Φ_f_ = 0.50, and *K*_L–Z_ = 4.97 (Figure S11). Based on these data, we gave it the moniker “JF_657_” and the JF_657_–HaloTag ligand (**59**_**HTL**_) was prepared without separating the diastereomers (Scheme S8). Incubation of cells expressing histone H2B–HaloTag fusion proteins with **59**_**HTL**_ showed excellent nuclear staining with little cellular background after washing (Figure 3c). We then compared the performance of **59**_**HTL**_ in SPT experiments to the current best-in-class far-red dye ligand JFX_650_-HaloTag ligand^45^ (**89**_**HTL**_; Figure 3d). We observed a significant improvement in brightness as measured by photons/s (Figure 3e). The novel JF_657_ also showed consistent localizations over time, whereas JFX_650_ exhibited relatively rapid bleaching (Figure 3f). Thus, fluorination and MAC chemistry allow the venerable ATTO 647N scaffold to be used in living cells, and the resulting dye—JF_657_—represents a new standard for SPT labels.

### Bright, Cell-Compatible Label Excited by Near-Infrared (NIR) Light

**Figure 4:**
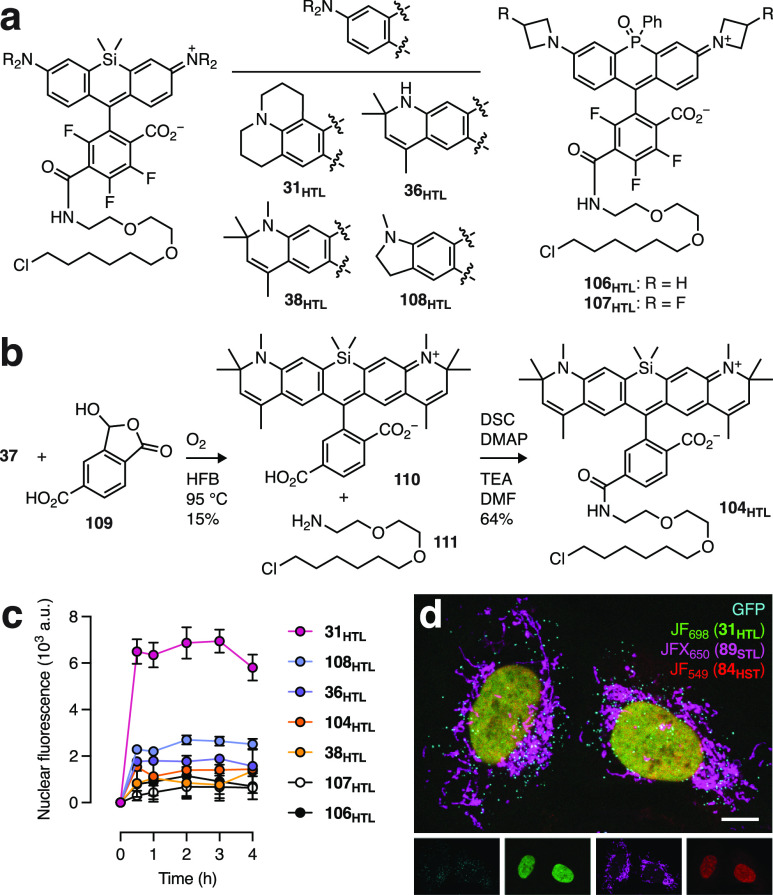
Evaluation of NIR-excited fluorophores. (a) Chemical structures of Si-rhodamine-based HaloTag ligands **31**_**HTL**_, **36**_**HTL**_, **38**_**HTL**_, and **108**_**HTL**_ and phosphine oxide-rhodamine-based **106**_**HTL**_ and **107**_**HTL**_. (b) Synthesis of **104**_**HTL**_. (c) Nuclear fluorescence vs time upon addition of ligands **31**_**HTL**_, **36**_**HTL**_, **38**_**HTL**_, **104**_**HTL**_, **106**_**HTL**_, **107**_**HTL**_, and **108**_**HTL**_ (200 nM) to live cells expressing HaloTag–histone H2B; error bars indicate SEM; *n* = 100 nuclei from three fields of view. (d) Four-color image of U2OS cells expressing the following fusion proteins: histone H2B–HaloTag; TOMM20–SNAP-tag; GFP–SKL (cyan, peroxisomes) and labeled with **31**_**HTL**_ (green, nucleus), JFX_650_-SNAP-tag ligand (**89**_**STL**_; magenta, mitochondria), and JF_549_–Hoechst (**84**_**HST**_; red, nucleus); panel shows individual channels; scale bar: 10 μm.

The NIR region is an attractive window for biological imaging with low autofluorescence, less scattering, and relatively deep tissue penetration. We considered dyes that exhibit λ_abs_ ≈ 700–750 nm and were particularly interested in the novel fluorinated analog of Si-rhodamine 101 (JF_698_; **31**); its ε = 147,000 M^–1^cm^–1^ and Φ_f_ = 0.28 (Table 2) make it similar in brightness to Alexa Fluor 700 and Cy7^70^ but with the potential for live-cell labeling applications. We compared the JF_698_–HaloTag ligand (**31**_**HTL**_) to other NIR-excited labels (Figure 4a), including Si-rhodamines **36**_**HTL**_ and **38**_**HTL**_ along with our previously reported phosphine oxide-rhodamine HaloTag ligands based on JF_722_ (**106**_**HTL**_) and JF_711_ (**107**_**HTL**_; Figure 4a).^16^ We also prepared two other NIR-excited fluorophores. Our Li/H exchange approach yielded the fluorinated derivative of SiR_700_^31,71^ (**108**); the MAC chemistry afforded its HaloTag derivative **108**_**HTL**_ (Schemes S3 and S8). The spectral properties of **108** were found to be λ_abs_ = 712 nm, ε = 98,800 M^–1^cm^–1^, λ_em_ = 735 nm, Φ_f_ = 0.09, and *K*_L–Z_ = 1.97 (Figure S11); we gave it the name SiRF_712_. Our modified lactol condensation conditions (Schemes 4 and 5) were employed to react **37** and lactol **109** yielding **110** (Figure 4b), which is the 6-carboxy derivative of **104**. Amidation with the HaloTag ligand amine **111** gave **104**_**HTL**_.

We then tested the utility of these NIR-excited HaloTag ligands in living cells. All of the compounds labeled live cells expressing HaloTag fused to histone H2B with similar kinetics but varying intensity. Consistent with the superior brightness of **31***in vitro*, the HaloTag ligand derivative **31**_**HTL**_ gave >3-fold higher nuclear intensity compared to the other ligands when excited at λ_abs_ (Figure 4c, Figure S12a–g). The HaloTag ligand with the second highest cellular intensity was the SiRF_712_ compound (**108**_**HTL**_). The dihydroquinoline Si-rhodamines (**36**_**HTL**_, **38**_**HTL**_, and **104**_**HTL**_) showed relatively low intensity in cells; this is expected based on their extremely small Φ_f_ values (Table 3). The JF_698_–HaloTag ligand (**31**_**HTL**_) was also brighter and more photostable that the phosphine oxide-containing JF_711_ (**107**_**HTL**_) and JF_722_ (**106**_**HTL**_; Figure S12h) and facilitated straightforward multicolor imaging experiments with the 650 nm-excited JFX_650_-SNAP-tag ligand (**89**_**STL**_) and other visible-absorbing fluorescent labels (Figure 4d).

### Far-Red FRET Quencher for Intracellular Labeling

**Figure 5:**
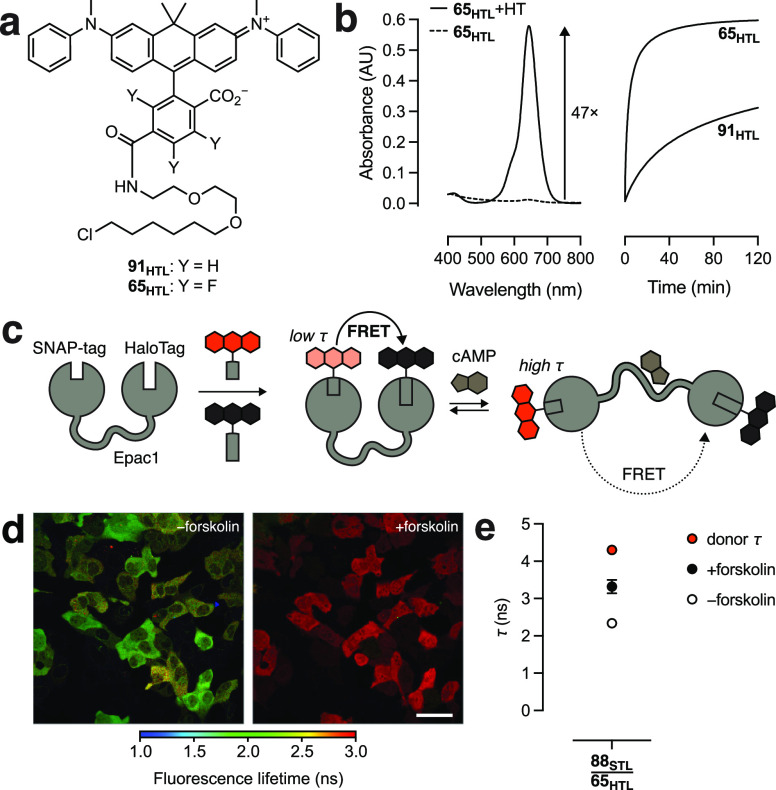
Semisynthetic sensor for cAMP. (a) Structures of *N*,*N*′-diaryl-carborhodamine ligand **91**_**HTL**_ and the fluorinated analog **65**_**HTL**_. (b) Absorbance spectra of **65**_**HTL**_ (5 μM) in the presence (solid line) or absence (dotted line) of the HaloTag protein (10 μM) and absorbance vs time of **91**_**HTL**_ and **65**_**HTL**_ (5 μM) upon addition to the HaloTag protein (10 μM). (c) Schematic of the semisynthetic cAMP indicator (ScAMPI). (d) Fluorescence lifetime image of live U2OS cells expressing ScAMPI and labeled with **88**_**STL**_/**65**_**HTL**_ ± forskolin; scale bar: 50 μm. (e) Quantification of the performance of ScAMPI in cells incorporating the **88**_**STL**_/**65**_**HTL**_ pair ± forskolin; error bars indicate SD; *n* = 20 cells.

Finally, we evaluated the nonfluorescent *N*,*N*′-diaryl-carborhodamine **91** and its fluorinated analog (**65**; Scheme 10). Introduction of *N*-aryl groups into rhodamine dyes severely decreases fluorescence quantum yield due to increased twisted internal charge transfer (TICT),^72,73^ making *N*-arylrhodamines and *N*-aryl-Si-rhodamines useful nonfluorescent acceptor dyes for Förster resonance energy transfer (FRET).^26,74,75^ These nonfluorescent rhodamine systems have not been used for intracellular labeling, however, and *N*-aryl-carborhodamines remain untested as FRET quenchers. Introduction of an *N*-aryl group into rhodamine dyes also shifts the lactone–zwitterion equilibrium toward the colorless lactone form (Table 2); commercial *N*-arylrhodamine quenchers typically have the *ortho*-carboxyl group blocked or removed, presumably to prevent unwanted lactonization.^26,74^ The *N*-aryl-carborhodamine compound **91** shows extremely low visible absorption in aqueous solution (ε < 200 M^–1^cm^–1^) and a vanishingly small *K*_L–Z_.^41^ Incorporation of fluorine atoms into the pendant phenyl ring system, as in compound **65**, shifts the lactone–zwitterion equilibrium toward the zwitterionic form, giving a dye with a measurable absorptivity (ε = 2,700 M^–1^cm^–1^), *K*_L–Z_ = 0.344, and far-red absorption (λ_abs_ = 645 nm; Table 2). We surmised that the properties of **65** could translate to a highly chromogenic HaloTag ligand. Incubation of **65**_**HTL**_ (Figure 5a) with the HaloTag protein gave an increase in absorptivity of 47-fold (Figure 5b) yielding a HaloTag conjugate with ε = 110,000 M^–1^cm^–1^. Unlike the fluorescent *N*-alkyl rhodamine ligands (Figure S7), HaloTag labeling of the fluorinated **65**_**HTL**_ was substantially faster than the nonfluorinated HaloTag ligand **91**_**HTL**_ (Scheme S9) *in vitro* (Figure 5b) and in cells (Figure S13). Based on these properties, we gave **65** the name Janelia Quencher 645 (JQ_645_).

We employed this novel, chromogenic, far-red quencher dye in a semisynthetic sensor for cyclic adenosine monophosphate (cAMP). We linked the genes encoding the SNAP-tag and HaloTag proteins via the sequence for the exchange protein directly activated by cAMP (Epac1),^76^ basing this design on an established cAMP sensor construct that incorporates fluorescent proteins.^77^ Binding of cAMP causes a conformational change that decreases FRET and increases the fluorescence lifetime (τ) of the donor (Figure 5d); this semisynthetic cAMPindicator (“ScAMPI”) construct was expressed in cells and labeled with the JFX_612_-SNAP-tag ligand (**88**_**STL**_) as the donor and the novel JQ_645_-HaloTag ligand (**65**_**HTL**_) as the acceptor. At rest, cells showed a lifetime of 2.34 ± 0.10 ns (mean ± SD) as measured by FLIM (Figure 5d,e). This lifetime is substantially lower than cells labeled with the JFX_612_ donor fluorophore alone (4.30 ± 0.01 ns) showing that the JQ_645_-HaloTag ligand is an effective FRET quencher. Increasing intracellular cAMP concentration by application of forskolin elicited a substantial increase in fluorescence lifetime to 3.32 ± 0.18, giving an overall Δτ = 0.98 ns. This result demonstrates that FRET quenchers can be employed in conjunction with self-labeling tags to create useful, red-shifted FLIM indicators for intracellular analytes.

## Conclusions

Rhodamines remain the most important fluorophores for live-cell imaging and are increasingly used in more complex systems, such as intact animals. Optimizing these fluorophores for specific applications requires the synthetic chemistry methods needed to make new rhodamines and an understanding of the structure–activity relationships that govern the spectral and chemical properties of dyes. Here, we thoroughly surveyed the synthesis of rhodamine dyes, investigating five different methods (Scheme 1). We developed new and improved chemistry, expanding the scope of the oxidative phthalaldehydic acid (“lactol”) condensation^42^ to synthesize fluorinated and nonfluorinated Si-rhodamines (Schemes 4 and 5) and establishing a novel route to fluorinated dyes using the straightforward Li/H exchange of 2,3,4,5-tetrafluorobenzoic acid (**6**; Schemes 6–8). This vetted portfolio of organic chemistry strategies will enable the synthesis of a wide range of rhodamines, particularly fluorinated derivatives.

We used these methods to assemble a systematic set of rhodamine dyes and measured their properties (Tables 1–3). These data revealed structure–activity relationships governing the lactone–zwitterion equilibrium (*K*_L–Z_; Figure 2), which is a key determinant of the performance of rhodamines in biological systems (Figure 1a).^16^ We found julolidine-containing dyes are strongly shifted toward the zwitterionic form, whereas *N*-aryl dyes prefer the lactone form. We also discovered that fluorination of the pendant phenyl ring could increase *K*_L–Z_ regardless of the chemical structure and we exploited this effect to prepare new reagents for cellular imaging, aided by our previously described MAC reagent derivatization strategy.^16^ We made the zwitterionic JF_657_ (**59**), a live-cell-compatible analog of the cationic ATTO 647N with performance in SPT experiments that supersedes the current best-in-class dye JFX_650_ (Figure 3). We leveraged the fluorine-induced bathochromic and *K*_L–Z_ shifts to make JF_698_ (**31**), a bright, NIR-excited dye that enables multicolor imaging (Figure 4). Finally, we developed JQ_645_ (**65**), a chromogenic far-red FRET quencher compatible with self-labeling tag systems. We used this quencher dye to create ScAMPI, a semisynthetic indicator of cAMP useful for FLIM (Figure 5). These novel molecules will immediately enable new biological imaging experiments in living cells.

This work also guides the development of new fluorescent probes. Prior to the synthesis of this comprehensive collection of dyes and exhaustive measurement of their chemical properties, the structure–activity relationships governing absorptivity and *K*_L–Z_ in different classes of rhodamines appeared idiosyncratic. For example, the high Φ_f_ of julolidine-containing dyes has been appreciated for decades^78^ but, to our knowledge, julolidine’s effect on ε was largely unrecognized. We showed that trends in absorptivity and *K*_L–Z_ can be rationalized by the electron-donating capability of the auxochrome group (Figure 2). Dye designers should pick a rhodamine scaffold capable of accessing the desired spectral and chemical properties for their application. Rhodamines containing NH_2_ or azetidine auxochromes can access a broad range of *K*_L–Z_ values and are useful for making a variety of probes including chromogenic or bioavailable reagents. Julolidine-containing rhodamines have a narrower *K*_L–Z_ range and are perhaps better suited for creating bright labels with enhanced absorptivity. Finally, we note that this work introduced novel fluorinated carborhodamines such as JF_657_ and JQ_645_, which represent a new scaffold for development of far-red-absorbing dyes.

Advances in the synthetic chemistry of small-molecule fluorescent probes combined with new labeling strategies are driving a renaissance in the field of reagents for fluorescence microscopy. Many small-molecule fluorophores—including rhodamines—were discovered in the 19th century; the associated archaic chemistry severely limited the synthesis of new derivatives. Investment in new chemistry yields new dyes, which enable new experiments and ultimately new biological discoveries. We no longer have to ask “What fluorophores *can* we make?” but rather “What fluorophores *should* we make?". It is our hope that this comprehensive organic chemistry study and new insight into structure–activity relationships, combined with a better understanding of dye photophysics, optimized computational chemistry approaches, and machine learning, will lead to new tools for studying complex biological systems.

## Supporting Information Available

Supplementary schemes, figures, and tables, experimentals and characterization data for all novel compounds, optical spectroscopy and microscopy methods, and NMR and HPLC/MS data (PDF)

## Abbreviations

MAC: masked acyl cyanide
FIC: fluorescein isocyanate
FITC: fluorescein isothiocyanate
GFP: green fluorescent protein
MOM: methoxymethyl ether
S_N_Ar: nucleophilic aromatic substitution
JF: Janelia Fluor
TFE: 2,2,2-trifluoroethanol
HFB: 2,2,3,3,4,4,4-heptafluoro-1-butanol
TFA: trifluoroacetic acid
SPT: single-particle tracking
FRET: Förster resonance energy transfer
FLIM: fluorescence lifetime imaging microscopy
