# Supplementary material for: Optimized Red-Absorbing Dyes for Imaging and Sensing
Source: J Am Chem Soc. 2023 Oct 16;145(42):23000–13. doi: 10.1021/jacs.3c05273 (PMC10603817; doi:10.1021/jacs.3c05273)

SUPPORTING INFORMATION

Optimized red-absorbing dyes for imaging and sensing

Jonathan B. Grimm, Ariana N. Tkachuk, Ronak Patel, S. Thomas Hennigan, Alina Gutu, Peng Dong, Valentina Gandin, Anastasia M. Osowski, Katie L. Holland, Zhe J. Liu, Timothy A. Brown, and Luke D. Lavis*

Janelia Research Campus, Howard Hughes Medical Institute,
Ashburn, VA 20147, USA

Email: lavisl@janelia.hhmi.org

EXPERIMENTAL INFORMATION

Page	Contents
S2	Schemes S1–S9 and Tables S1–S2
S10	General Experimental Information for Synthesis
S11–S95	Experimentals and Characterization Data for All Compounds
S11	Preparation of Diarylether and Diarylsilane Intermediates
S19	Rhodamine Synthesis via Lactol (Phthalaldehydic Acid) Condensation
S35	Preparation of 2,2-Diarylpropane Intermediates
S44	Preparation of Anthrone Intermediates
S49	Xanthene Synthesis via Lithiation of Tetrafluorobenzoic Acid
S55	Synthesis of Carborhodamines via $AlCl_3$ Condensation
S60	Rhodamine 110 and *N*-Aryl Rhodamine Synthesis via Cross-Coupling
S67	MAC Substitution of 4,5,6,7-Tetrafluoroxanthenes
S78	Conversion of MAC Xanthenes to HaloTag Ligands
S92	Synthesis of Other HaloTag and SNAP-tag Ligands
S96	Figures S1–S13 and Table S3
S105	Optical Spectroscopy and Microscopy Methods
S113	References
S115	NMR and HPLC/MS

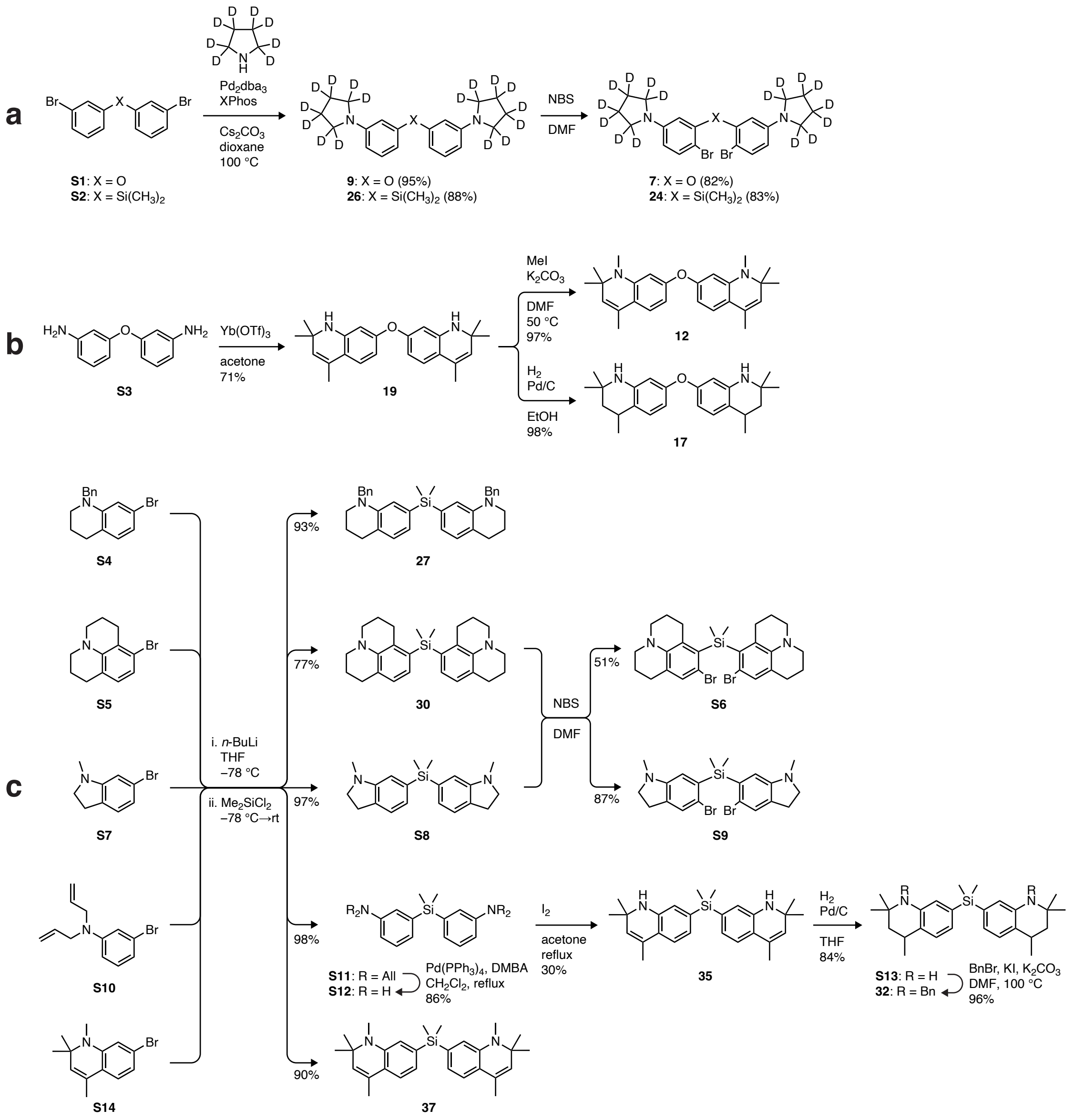

Scheme S1. Bis(3-aminophenyl)ether and bis(3-aminophenyl)dimethylsilane intermediates. (**a**) Synthesis of pyrrolidine-d_8-substituted ethers (**7**, **9**) and silanes (**24**, **26**). (**b**) Synthesis of dihydroquinoline ether **12** and tetrahydroquinoline ether **17**. (**c**) Synthesis of various diaryldimethylsilanes via Li-Br exchange and reaction with dichlorodimethylsilane.

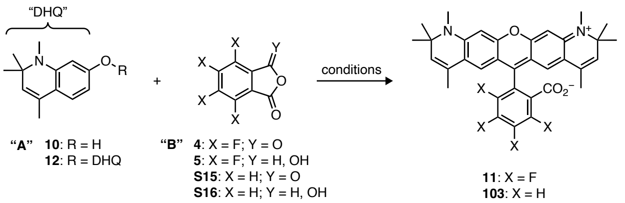

R	X	Y	solvent/reagents	temperature	time	equiv **A**:**B**	yield
H	F	O	$EtCO_2H$, *p*-TsOH	reflux	24 h	2.1:1	8–10%
H	F	O	$EtCO_2H$, *p*-TsOH	reflux	72 h	2.1:1	0%[a]
H	F	O	ODCB	reflux	18 h	2.1:1	0%
H	F	O	TFE	80 °C	24 h	2.1:1	0%
H	F	H, OH	TFE, O_2	80 °C	24 h	2.1:1	24%
DHQ	F	H, OH	TFE, O_2	80 °C	24 h	1:1	66%
DHQ	F	H, OH	TFE, O_2	80 °C	24 h	1:1.5	59%
DHQ	F	O	TFE	80 °C	24 h	1:1	9%
H	H	O	$EtCO_2H$, *p*-TsOH	reflux	24 h	2.1:1	22%
H	H	O	$EtCO_2H$, *p*-TsOH	reflux	72 h	2.1:1	58%
H	H	O	ODCB	reflux	18 h	2.1:1	37%
H	H	O	ODCB	reflux	72 h	2.1:1	84%
DHQ	H	H, OH	TFE, O_2	80 °C	24 h	1:1	69%

[a] **11** slowly decomposes with extended reaction time under these conditions to afford a mixture of several other products, most notably the analog resulting from protodecarboxylation of the *ortho*-carboxyl group.

Table S1. Comparison and optimization of reaction conditions—acid-mediated condensation of phthalic anhydrides versus oxidative condensation of phthalaldehydic acids ("lactols")—for the synthesis of dihydroquinoline rhodamines **11** and **103**.

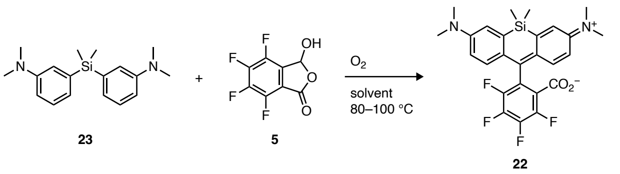

equiv **23**:**5**	solvent[a]	temperature	concentration	time	yield
1:1	TFE	80 °C	0.03 M	72 h	16%
1:1	TFE	80 °C	0.03 M	168 h	18%
1:1	TFP	100 °C	0.03 M	72 h	11%
1:1	HFB	95 °C	0.03 M	72 h	18%
1:1	TFE	80 °C	0.3 M	72 h	25%
1:1	HFB	95 °C	0.3 M	72 h	36%
1:1.5	HFB	95 °C	0.3 M	48 h	44%
1:2	HFB	95 °C	0.3 M	48 h	47%
1:2	HFB	95 °C	0.3 M	72 h	52%
1:3	HFB	95 °C	0.3 M	72 h	58%

[a] TFE = 2,2,2-trifluoroethanol; TFP = 2,2,3,3-tetrafluoro-1-propanol; HFB = 2,2,3,3,4,4,4-heptafluoro-1-butanol.

Table S2. Optimization of oxidative phthalaldehydic acid ("lactol") condensation of **5** with diaryldimethylsilane **23** to access fluorinated SiR dye **22** ($SiRF_{667}$).

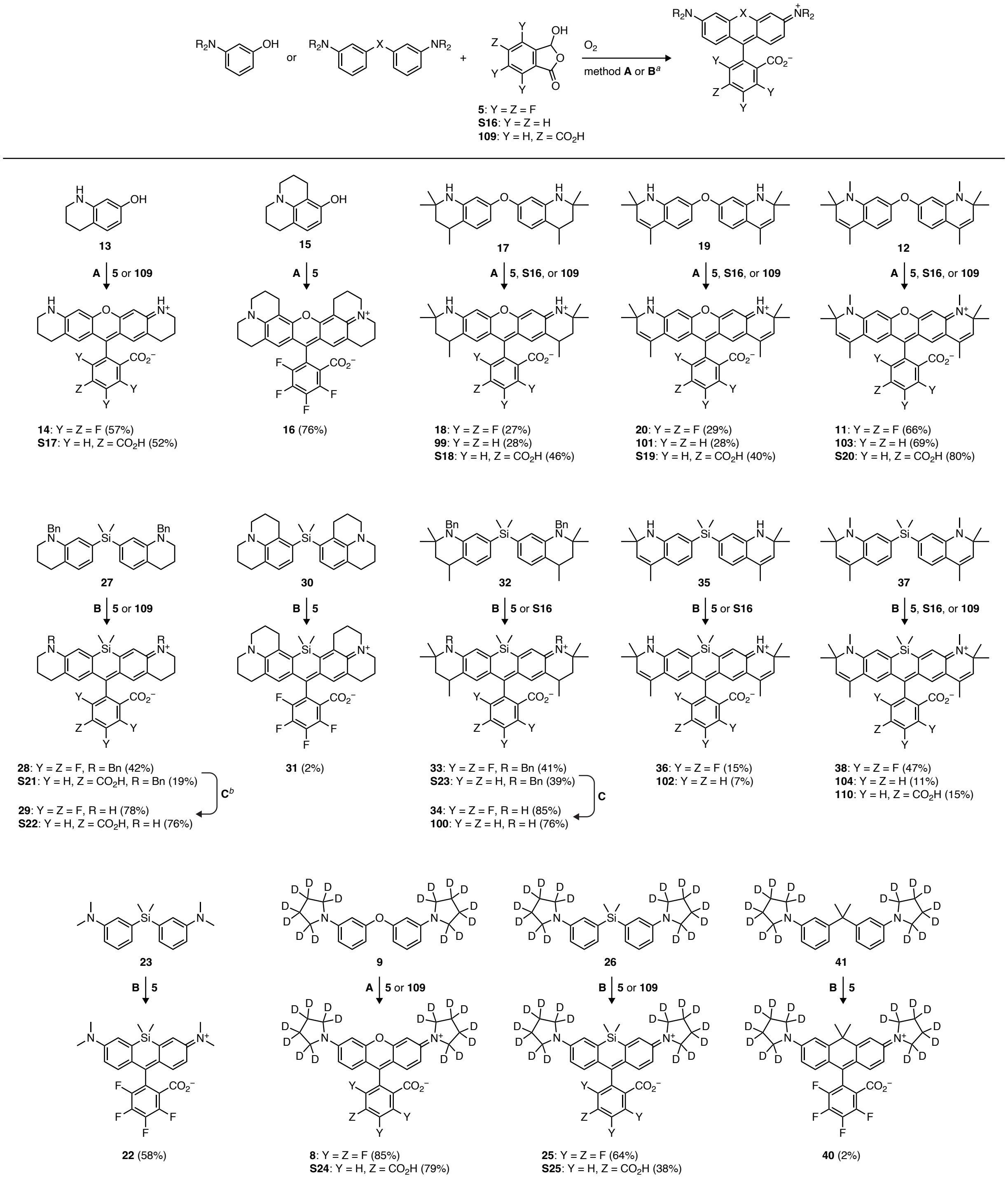

[a] Method A: 2,2,2-trifluoroethanol (0.05 M), 80 °C, 18–72 h; Method B: 2,2,3,3,4,4,4-heptafluoro-1-butanol (0.2–0.3 M), 95 °C, 18–72 h. [b] Method C: (1) H_2, Pd/C, THF; (2) chloranil, CH_2Cl_2/MeOH.

Scheme S2. Exploration of the scope of the phthalaldehydic acid ("lactol") condensation for the synthesis of rhodamines and Si-rhodamines via reaction of various diarylethers and diarylsilanes with lactols **5**, **S16**, and **109**.

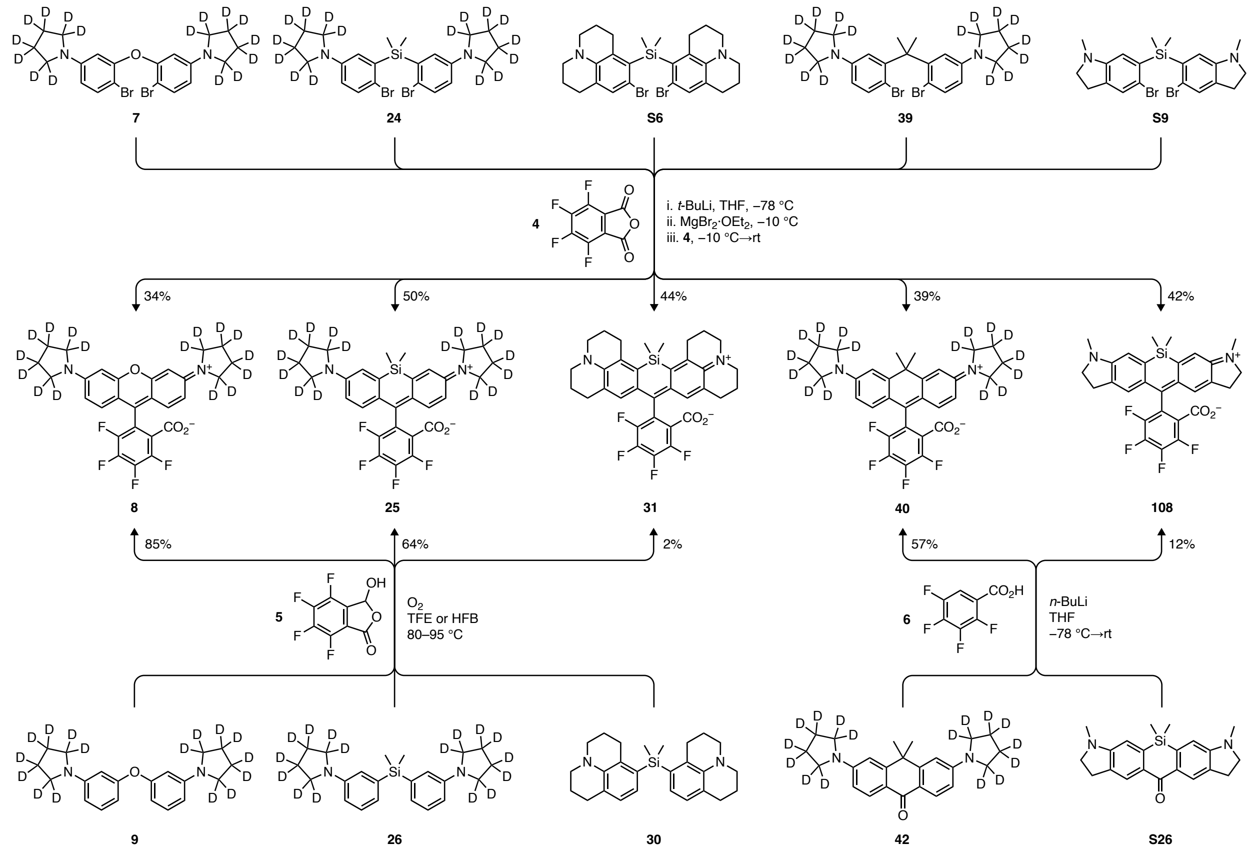

Scheme S3. Alternative syntheses of representative fluorinated rhodamines via (i) Li-Br exchange of bis(5-amino-2-bromophenyl)ethers, -silanes, and -propanes and (ii-iii) addition to tetrafluorophthalic anhydride (**4**), with comparison to the phthalaldehydic acid (**8**, **25**, **31**) and tetrafluorobenzoic acid (**40**, **108**) routes. Only **31** (JF_{698}) and **108** ($SiRF_{712}$) are more efficiently synthesized through the dibromide route.

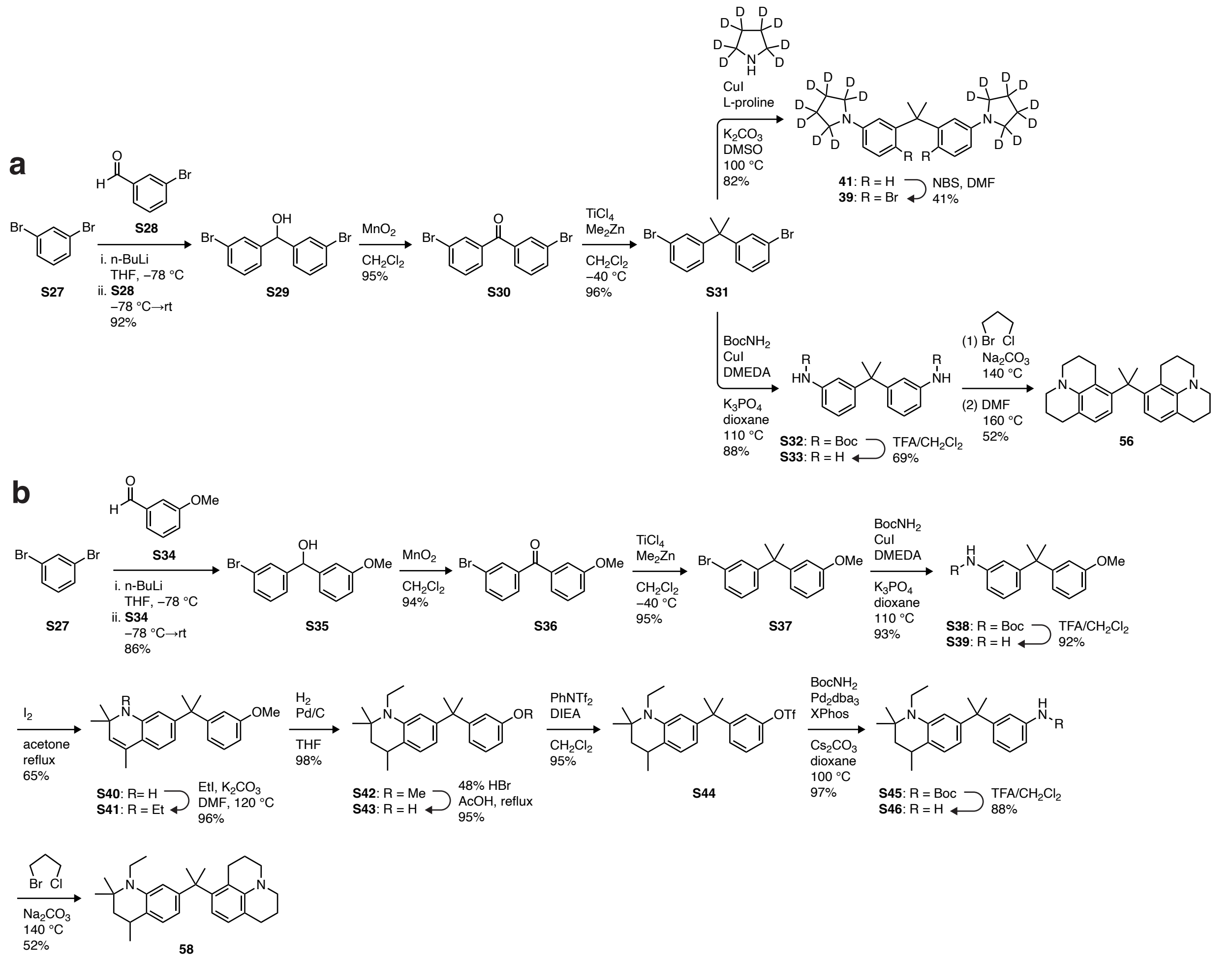

Scheme S4. (**a**) Synthesis of 2,2-bis(3-aminophenyl)propane intermediates **39**, **41**, and **56**. (**b**) Synthesis of JF_{657} precursor **58**.

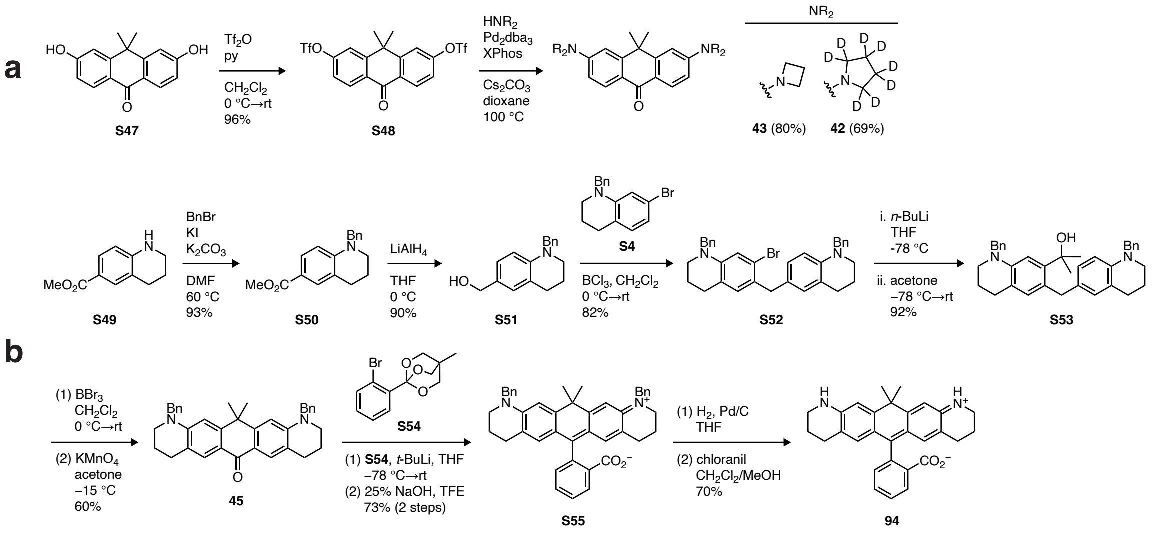

Scheme S5. Synthesis of anthrone intermediates (**42, 43**, and **45**) and carbo-Q-rhodamine (**94**).

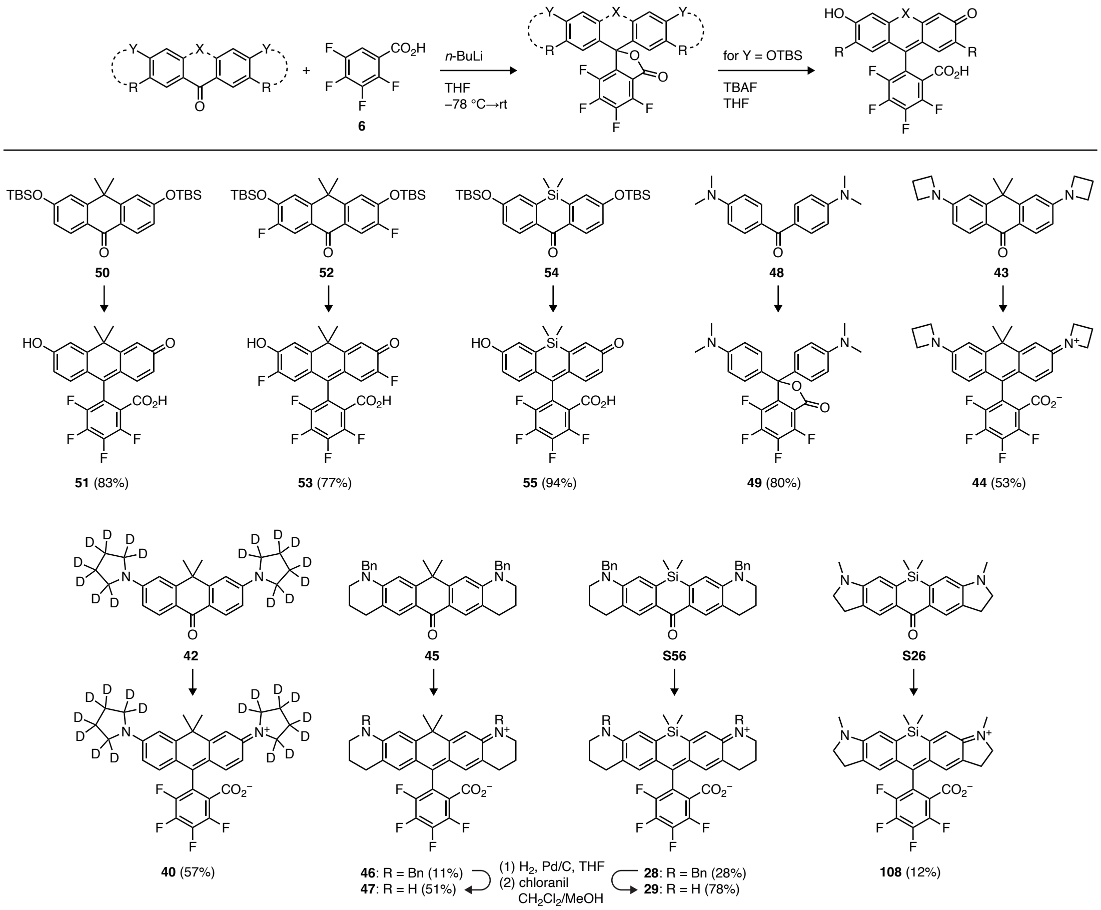

Scheme S6. Scope of tetrafluorobenzoic acid lithiation and ketone addition for the synthesis of fluorinated fluoresceins (**51**, **53**, **55**), Malachite Green lactone (**49**), and rhodamines (**29**, **40**, **44**, **47**, **108**).

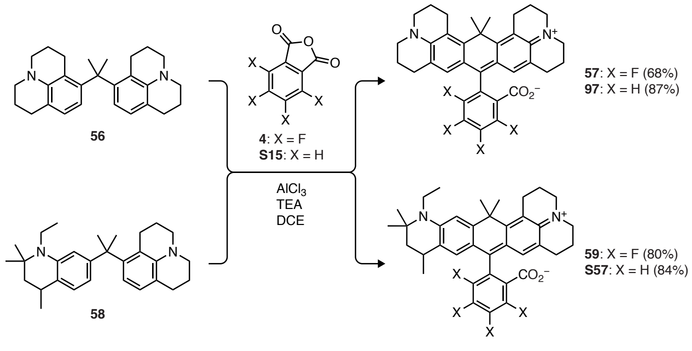

Scheme S7. Synthesis of JF_{660} (**57**), JF_{657} (**59**), carborhodamine 101 (**97**), and free ATTO 647N (**S57**) via $AlCl_3$-mediated condensation of 2,2-bis(3-aminophenyl)propanes with phthalic anhydrides.

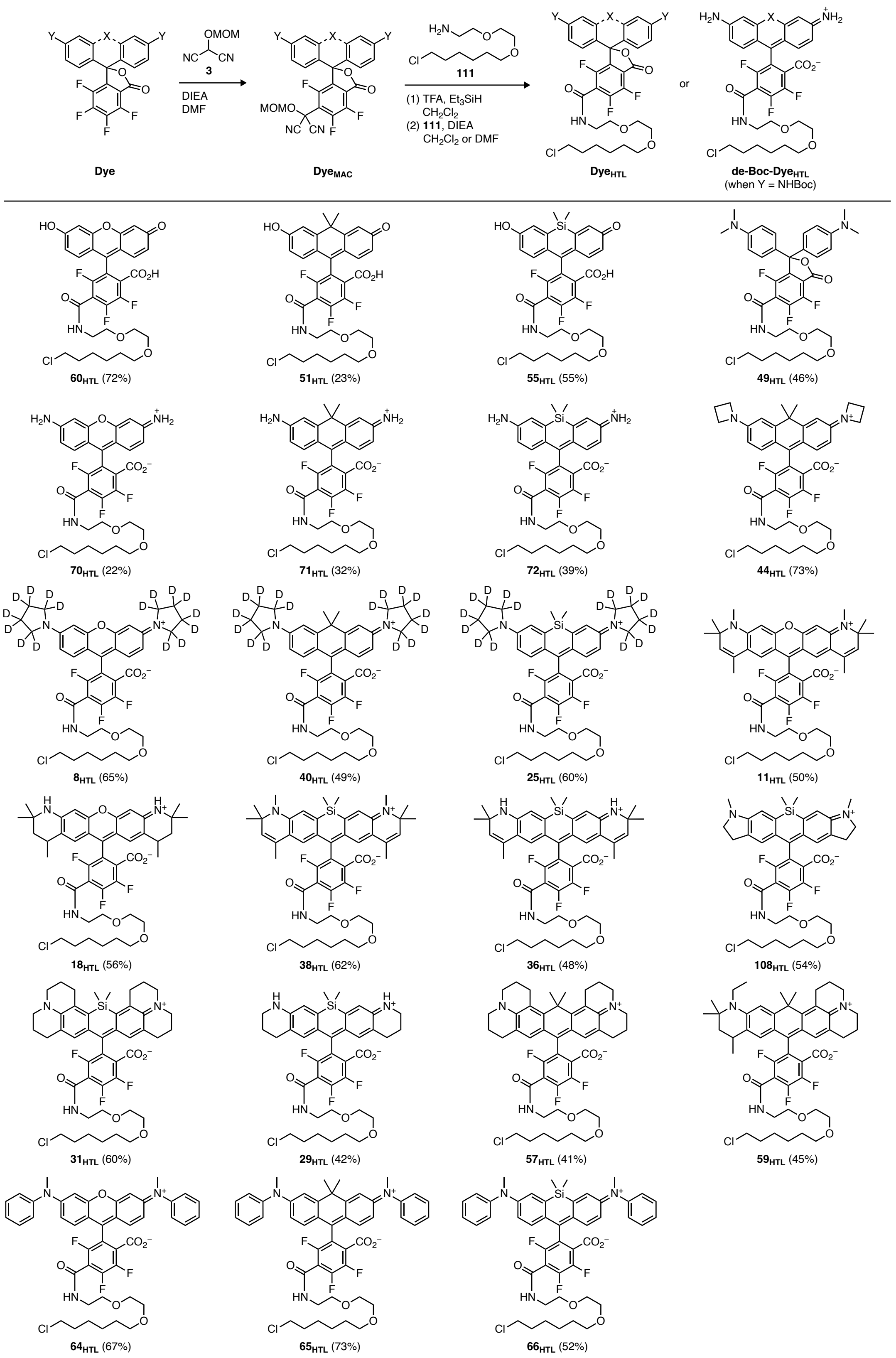

Scheme S8. Substitution of 4,5,6,7-tetrafluoro-fluoresceins and -rhodamines with masked acyl cyanide (MAC) reagent **3** and subsequent derivatization to HaloTag ligand labels.

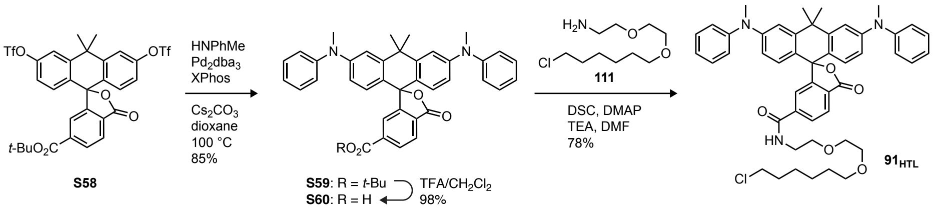

Scheme S9. Synthesis of **91_{HTL}** via C–N cross-coupling of carbofluorescein ditriflate **S58**.

GENERAL EXPERIMENTAL INFORMATION FOR SYNTHESIS

Commercial reagents were obtained from reputable suppliers and used as received. All solvents were purchased in septum-sealed bottles stored under an inert atmosphere. All reactions were sealed with septa through which a nitrogen atmosphere was introduced unless otherwise noted. Reactions were conducted in round-bottomed flasks or septum-capped crimp-top vials containing Teflon-coated magnetic stir bars. Heating of reactions was accomplished with a silicon oil bath or an aluminum reaction block on top of a stirring hotplate equipped with an electronic contact thermometer to maintain the indicated temperatures.

Reactions were monitored by thin layer chromatography (TLC) on precoated TLC glass plates (silica gel 60 F_{254}, 250 µm thickness) or by LC/MS (Phenomenex Kinetex 2.1 mm × 30 mm 2.6 µm C18 column; 5 µL injection; 5–98% $MeCN/H_2O$, linear gradient, with constant 0.1% v/v HCO_2H additive; 6 min run; 0.5 mL/min flow; ESI; positive ion mode). TLC chromatograms were visualized by UV illumination or developed with *p*-anisaldehyde, ceric ammonium molybdate, or $KMnO_4$ stain. Reaction products were purified by flash chromatography on an automated purification system using pre-packed silica gel columns or by preparative HPLC (Phenomenex Gemini–NX 30 × 150 mm 5 µm C18 column). Analytical HPLC analysis was performed with an Agilent Eclipse XDB 4.6 × 150 mm 5 µm C18 column under the indicated conditions. High-resolution mass spectrometry was performed by the High Resolution Mass Spectrometry Facility at the University of Iowa.

NMR spectra were recorded on a 400 MHz spectrometer. ^{1}H and ^{13}C chemical shifts were referenced to TMS or residual solvent peaks, and ^{19}F chemical shifts were referenced to $CFCl_3$. Data for ^{1}H NMR spectra are reported as follows: chemical shift (δ ppm), multiplicity (s = singlet, d = doublet, t = triplet, q = quartet, dd = doublet of doublets, m = multiplet), coupling constant (Hz), integration. Data for ^{13}C NMR spectra are reported by chemical shift (δ ppm) with hydrogen multiplicity (C, CH, CH_2, CH_3) information obtained from DEPT spectra. *Note: The ^{13}C NMR spectra are not reported for compounds containing trifluoro- or tetrafluoro-substituted aryl rings, as the 3–4 distinct fluorine couplings to* ***each*** *of the six different carbons of the bottom ring confounded any useful interpretation of the spectra.*

PREPARATION OF DIARYLETHER AND DIARYLSILANE INTERMEDIATES (SCHEME S1)

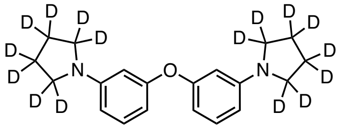

1,1'-(Oxybis(3,1-phenylene))bis(pyrrolidine-2,2,3,3,4,4,5,5-*d*$_8$) (9): A vial was charged with 3,3'-oxybis(bromobenzene)[1] (**S1**; 1.00 g, 3.05 mmol), Pd_2dba_3 (279 mg, 0.305 mmol, 0.1 eq), XPhos (436 mg, 0.915 mmol, 0.3 eq), and Cs_2CO_3 (2.78 g, 8.54 mmol, 2.8 eq). The vial was sealed and evacuated/backfilled with nitrogen (3×). Dioxane (12 mL) was added, and the reaction was flushed again with nitrogen (3×). Following the addition of pyrrolidine-2,2,3,3,4,4,5,5-*d*$_8$ (612 µL, 7.32 mmol, 2.4 eq), the reaction was stirred at 100 °C for 18 h. It was then cooled to room temperature, filtered through Celite with CH_2Cl_2, and concentrated to dryness. The crude product was purified by flash chromatography (0–20% Et_2O/hexanes, linear gradient) to yield 936 mg (95%) of **9** as an off-white solid. ^{1}H NMR ($CDCl_3$, 400 MHz) δ 7.16 – 7.10 (m, 2H), 6.35 – 6.24 (m, 6H); ^{13}C NMR ($CDCl_3$, 101 MHz) δ 158.6 (C), 149.6 (C), 129.9 (CH), 106.7 (CH), 105.9 (CH), 102.5 (CH); HRMS (ESI) calcd for $C_{20}H_9D_{16}N_2O$ $[M+H]^+$ 325.2966, found 325.2957.

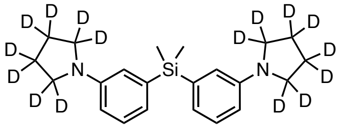

Dimethylbis(3-(pyrrolidin-1-yl-*d*$_8$)phenyl)silane (26): A vial was charged with bis(3-bromophenyl)dimethylsilane[2] (**S2**; 300 mg, 0.810 mmol), Pd_2dba_3 (74.2 mg, 81.0 µmol, 0.1 eq), XPhos (116 mg, 0.243 mmol, 0.3 eq), and Cs_2CO_3 (739 mg, 2.27 mmol, 2.8 eq). The vial was sealed and evacuated/backfilled with nitrogen (3×). Dioxane (3.5 mL) was added, and the reaction was flushed again with nitrogen (3×). Following the addition of pyrrolidine-2,2,3,3,4,4,5,5-*d*$_8$ (163 µL, 1.95 mmol, 2.4 eq), the reaction was stirred at 100 °C for 18 h. It was then cooled to room temperature, filtered through Celite with CH_2Cl_2, and concentrated to dryness. The resulting residue was purified by flash chromatography (0–10% Et_2O/hexanes, linear gradient, with constant 0.1% v/v Et_3N additive) to provide 261 mg (88%) of **26** as an off-white solid. ^{1}H NMR ($CDCl_3$, 400 MHz) δ 7.21 (dd, J = 8.2, 7.1 Hz, 2H), 6.84 (dt, J = 7.1, 1.1 Hz, 2H), 6.75 (dd, J = 2.6, 1.0 Hz, 2H), 6.57 (ddd, J = 8.2, 2.7, 1.1 Hz, 2H), 0.52 (s, 6H); ^{13}C NMR ($CDCl_3$, 101 MHz) δ 147.5 (C), 139.2 (C), 128.6 (CH), 121.5 (CH), 117.3 (CH), 112.5 (CH), -2.0 (CH_3); HRMS (ESI) calcd for $C_{22}H_{15}D_{16}N_2Si$ $[M+H]^+$ 367.3255, found 367.3259.

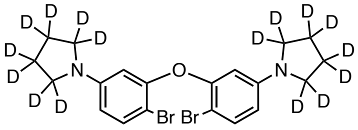

1,1'-(Oxybis(4-bromo-3,1-phenylene))bis(pyrrolidine-2,2,3,3,4,4,5,5-*d*$_8$) (7): 1,1'-(Oxybis(3,1-phenylene))bis-(pyrrolidine-2,2,3,3,4,4,5,5-*d*$_8$) (**9**; 850 mg, 2.62 mmol) was taken up in DMF (20 mL). *N*-Bromosuccinimide (932 mg, 5.24 mmol, 2 eq) was added portion-wise over 5 min, and the reaction was then stirred at room temperature for 4

h. The reaction mixture was concentrated *in vacuo*; the resulting residue was diluted with water and extracted with CH_2Cl_2 (2×). The combined organic extracts were washed with water and brine, dried over anhydrous $MgSO_4$, filtered, and concentrated *in vacuo*. Silica gel chromatography (10–75% CH_2Cl_2/hexanes, linear gradient) afforded 1.04 g (82%) of **7** as a white solid. ^{1}H NMR ($CDCl_3$, 400 MHz) δ 7.36 (d, J = 8.7 Hz, 2H), 6.21 (dd, J = 8.8, 2.8 Hz, 2H), 6.07 (d, J = 2.7 Hz, 2H); ^{13}C NMR ($CDCl_3$, 101 MHz) δ 153.9 (C), 148.6 (C), 133.5 (CH), 108.6 (CH), 103.0 (CH), 98.7 (C); HRMS (ESI) calcd for $C_{20}H_7D_{16}Br_2N_2O$ $[M+H]^+$ 481.1176, found 483.1149.

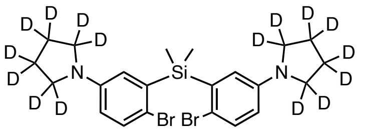

Bis(2-bromo-5-(pyrrolidin-1-yl-*d*$_8$)phenyl)dimethylsilane (24): Silane **26** (1.95 g, 5.32 mmol) was taken up in DMF (60 mL). *N*-Bromosuccinimide (1.89 g, 10.64 mmol, 2 eq) was added portion-wise over 2–3 min, and the reaction was then stirred at room temperature for 1 h. The reaction mixture was concentrated to remove DMF, diluted with water, and extracted with CH_2Cl_2 (2×). The combined organic extracts were washed with brine, dried over anhydrous $MgSO_4$, filtered, and concentrated *in vacuo*. The crude was dissolved in a minimum amount of CH_2Cl_2, diluted with an equivalent volume of hexanes, and gently concentrated until a white solid precipitated. The resulting suspension was filtered; the filter cake was washed with Et_2O and dried to yield 2.32 g (83%) of dibromide **24** as a white solid. ^{1}H NMR ($CDCl_3$, 400 MHz) δ 7.32 (d, J = 8.7 Hz, 2H), 6.66 (d, J = 3.1 Hz, 2H), 6.42 (dd, J = 8.7, 3.1 Hz, 2H), 0.74 (s, 6H); ^{13}C NMR ($CDCl_3$, 101 MHz) δ 146.5 (C), 139.0 (C), 133.1 (CH), 120.7 (CH), 115.4 (C), 114.3 (CH), -0.8 (CH_3); HRMS (ESI) calcd for $C_{22}H_{13}D_{16}Br_2N_2Si$ $[M+H]^+$ 523.1466, found 523.1466.

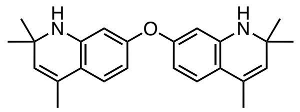

7,7'-Oxybis(2,2,4-trimethyl-1,2-dihydroquinoline) (19): To a solution of 3,3'-oxydianiline (**S3**; 1.00 g, 4.99 mmol) in acetone (30 mL) was added Yb(OTf)$_3$ (465 mg, 0.749 mmol, 0.15 eq). After stirring the reaction at room temperature for 72 h, it was concentrated *in vacuo*, diluted with saturated $NaHCO_3$, and extracted with EtOAc (2×). The combined organic extracts were washed with brine, dried over anhydrous $MgSO_4$, filtered, and concentrated to dryness. Flash chromatography on silica gel (0–25% EtOAc/hexanes, linear gradient) yielded 1.27 g (71%) of **19** as a white solid. ^{1}H NMR ($CDCl_3$, 400 MHz) δ 6.98 (d, J = 8.3 Hz, 2H), 6.29 (dd, J = 8.3, 2.4 Hz, 2H), 6.09 (d, J = 2.4 Hz, 2H), 5.23 (q, J = 1.3 Hz, 2H), 3.66 (s, 2H), 1.97 (d, J = 1.4 Hz, 6H), 1.26 (s, 12H); ^{13}C NMR ($CDCl_3$, 101 MHz) δ 157.8 (C), 144.7 (C), 128.2 (C), 127.0 (CH), 124.8 (CH), 117.2 (C), 107.7 (CH), 103.3 (CH), 52.1 (C), 31.3 (CH_3), 18.8 (CH_3); HRMS (ESI) calcd for $C_{24}H_{29}N_2O$ $[M+H]^+$ 361.2274, found 361.2267.

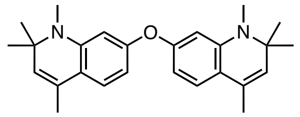

7,7'-Oxybis(1,2,2,4-tetramethyl-1,2-dihydroquinoline) (12): 7,7'-Oxybis(2,2,4-trimethyl-1,2-dihydroquinoline) (**19**; 2.00 g, 5.55 mmol) was taken up in DMF (16 mL); K_2CO_3 (2.30 g, 16.64 mmol, 3 eq) and iodomethane (829 µL, 13.31 mmol, 2.4 eq) were added, and the reaction was stirred at 50 °C for 18 h. It was subsequently cooled to room temperature, diluted with water, and extracted with EtOAc (2×). The combined organic extracts were washed with brine, dried over anhydrous $MgSO_4$, filtered, and concentrated to dryness. Silica gel chromatography (0–20% EtOAc/hexanes, linear gradient) afforded **12** as a white solid (2.10 g, 97%). ^{1}H NMR ($CDCl_3$, 400 MHz) δ 7.00 – 6.93 (m, 2H), 6.31 – 6.23 (m, 4H), 5.22 (q, J = 1.4 Hz, 2H), 2.74 (s, 6H), 1.97 (d, J = 1.4 Hz, 6H), 1.30 (s, 12H); ^{13}C NMR ($CDCl_3$, 101 MHz) δ 158.2 (C), 146.9 (C), 128.5 (CH), 128.0 (C), 124.2 (CH), 118.9 (C), 105.9 (CH), 102.1 (CH), 56.5 (C), 30.9 (CH_3), 27.4 (CH_3), 18.8 (CH_3); HRMS (ESI) calcd for $C_{26}H_{33}N_2O$ $[M+H]^+$ 389.2587, found 389.2579.

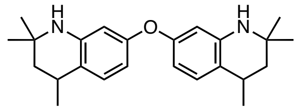

7,7'-Oxybis(2,2,4-trimethyl-1,2,3,4-tetrahydroquinoline) (17): 7,7'-Oxybis(2,2,4-trimethyl-1,2-dihydroquinoline) (**19**; 4.50 g, 12.48 mmol) was dissolved in EtOH (300 mL) in a round-bottom flask under nitrogen, and Pd/C (10%, 2.66 g, 2.50 mmol, 0.2 eq) was added. The sealed flask was evacuated/backfilled with H_2 from a balloon (4×) and then stirred under the H_2 balloon at room temperature for 18 h. The reaction mixture was filtered through Celite with EtOH and concentrated *in vacuo*. Silica gel chromatography (0–15% EtOAc/hexanes, linear gradient) yielded 4.47 g (98%) of **17** as a white solid (mixture of diastereomers). ^{1}H NMR ($CDCl_3$, 400 MHz) δ 7.09 – 7.02 (m, 2H), 6.36 – 6.28 (m, 2H), 6.10 – 6.05 (m, 2H), 3.54 (s, 2H), 2.94 – 2.80 (m, 2H), 1.72 (dd, J = 12.9, 5.5 Hz, 2H), 1.42 (t, J = 12.5 Hz, 2H), 1.31 (d, J = 6.7 Hz, 6H), 1.21 (s, 6H), 1.17 (s, 6H); ^{13}C NMR ($CDCl_3$, 101 MHz) δ 156.6 (C), 144.9 (C), 127.91/127.86 (CH), 120.17/120.15 (C), 107.76/107.67 (CH), 104.13/104.10 (CH), 49.5 (C), 44.7 (CH_2), 31.6 (CH), 28.04/27.96 (CH_3), 27.4 (CH_3), 20.57/20.54 (CH_3); HRMS (ESI) calcd for $C_{24}H_{33}N_2O$ $[M+H]^+$ 365.2587, found 365.2580.

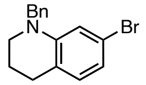

1-Benzyl-7-bromo-1,2,3,4-tetrahydroquinoline (S4): 7-Bromo-1,2,3,4-tetrahydroquinoline (1.00 g, 4.71 mmol), benzyl bromide (1.61 g, 9.43 mmol, 2 eq), K_2CO_3 (1.56 g, 11.3 mmol, 2.4 eq), and KI (157 mg, 0.943 mmol, 0.2 eq) were combined in DMF (15 mL) and stirred at 60 °C for 1 h. The reaction was subsequently diluted with water and extracted with EtOAc (2×). The combined organic extracts were washed with water and brine, dried ($MgSO_4$), filtered, and concentrated *in vacuo*. Silica gel chromatography (0–15% Et_2O/hexanes, linear gradient) yielded **S4** (1.35 g, 95%) as a gum that crystallized into a colorless, low-melting solid upon standing. ^{1}H NMR ($CDCl_3$, 400 MHz) δ 7.36 – 7.29 (m, 2H), 7.28 – 7.20 (m, 3H), 6.80 (d, J = 7.9 Hz, 1H), 6.66 (dd, J = 7.9, 1.9 Hz, 1H), 6.62 (d, J = 1.8 Hz, 1H), 4.45 (s, 2H), 3.36 – 3.28 (m, 2H), 2.73 (t, J = 6.3 Hz, 2H), 2.02 – 1.91 (m, 2H); ^{13}C NMR ($CDCl_3$, 101 MHz) δ 146.9 (C),

138.1 (C), 130.2 (CH), 128.8 (CH), 127.1 (CH), 126.7 (CH), 121.2 (C), 120.9 (C), 118.5 (CH), 113.4 (CH), 55.0 (CH_2), 49.6 (CH_2), 27.9 (CH_2), 22.1 (CH_2); HRMS (ESI) calcd for $C_{16}H_{16}BrNNa$ [M+Na]$^+$ 324.0358, found 324.0360.

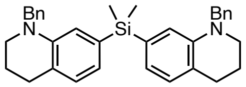

Bis(1-benzyl-1,2,3,4-tetrahydroquinolin-7-yl)dimethylsilane (27): A solution of 1-benzyl-7-bromo-1,2,3,4-tetrahydroquinoline (**S4**; 4.00 g, 13.24 mmol, 2.4 eq) in THF (50 mL) was cooled to -78 °C under nitrogen. *n*-Butyllithium (2.5 M in hexanes, 5.29 mL, 13.24 mmol, 2.4 eq) was added, and the reaction was stirred at -78 °C for 30 min. Dichlorodimethylsilane (665 µL, 5.51 mmol) was then added. The dry ice bath was removed, and the reaction was stirred at room temperature for 2 h. It was subsequently quenched with saturated NH_4Cl, diluted with water, and extracted with EtOAc (2×). The combined organic extracts were washed with brine, dried over anhydrous $MgSO_4$, filtered, and concentrated *in vacuo*. Purification by flash chromatography on silica gel (0–40% CH_2Cl_2/hexanes, linear gradient, with constant 1% v/v Et_3N additive) afforded 2.58 g (93%) of **27** as a white solid. ^{1}H NMR ($CDCl_3$, 400 MHz) δ 7.30 – 7.24 (m, 4H), 7.23 – 7.18 (m, 6H), 6.90 (dt, J = 7.4, 1.0 Hz, 2H), 6.67 – 6.62 (m, 4H), 4.38 (s, 4H), 3.37 – 3.31 (m, 4H), 2.78 (t, J = 6.4 Hz, 4H), 2.02 – 1.94 (m, 4H), 0.24 (s, 6H); ^{13}C NMR ($CDCl_3$, 101 MHz) δ 145.0 (C), 139.3 (C), 136.8 (C), 128.66 (CH), 128.60 (CH), 127.0 (CH), 126.8 (CH), 123.4 (C), 122.0 (CH), 116.9 (CH), 55.6 (CH_2), 50.3 (CH_2), 28.3 (CH_2), 22.5 (CH_2), -2.3 (CH_3); HRMS (ESI) calcd for $C_{34}H_{39}N_2Si$ [M+H]$^+$ 503.2877, found 503.2873.

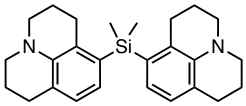

Dimethylbis(2,3,6,7-tetrahydro-1*H*,5*H*-pyrido[3,2,1-*ij*]quinolin-8-yl)silane (30): A solution of 8-bromojulolidine (**S5**; 3.00 g, 11.90 mmol, 2.4 eq) in THF (50 mL) was cooled to -78 °C under nitrogen. *n*-Butyllithium (2.5 M in hexanes, 4.76 mL, 11.90 mmol, 2.4 eq) was added, and the reaction was stirred at -78 °C for 30 min. Dichlorodimethylsilane (598 µL, 4.96 mmol) was then added. The dry ice bath was removed, and the reaction was stirred at room temperature for 2 h. It was subsequently quenched with saturated NH_4Cl, diluted with water, and extracted with EtOAc (2×). The combined organic extracts were washed with brine, dried over anhydrous $MgSO_4$, filtered, and concentrated *in vacuo*. Purification by flash chromatography on silica gel (0–20% Et_2O/hexanes, linear gradient) afforded 1.54 g (77%) of **30** as a white foam. ^{1}H NMR ($CDCl_3$, 400 MHz) δ 6.77 (d, J = 7.4 Hz, 2H), 6.69 (d, J = 7.4 Hz, 2H), 3.16 – 3.10 (m, 4H), 3.10 – 3.04 (m, 4H), 2.76 (t, J = 6.5 Hz, 4H), 2.67 (t, J = 6.5 Hz, 4H), 1.96 (p, J = 6.3 Hz, 4H), 1.85 (p, J = 6.3 Hz, 4H), 0.51 (s, 6H); ^{13}C NMR ($CDCl_3$, 101 MHz) δ 142.8 (C), 135.3 (C), 127.7 (C), 126.7 (CH), 123.3 (C), 122.9 (CH), 50.7 (CH_2), 50.2 (CH_2), 28.7 (CH_2), 28.2 (CH_2), 22.5 (CH_2), 22.2 (CH_2), 0.2 (CH_3); HRMS (ESI) calcd for $C_{26}H_{35}N_2Si$ [M+H]$^+$ 403.2564, found 403.2563.

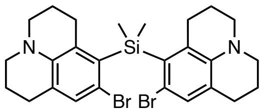

Bis(9-bromo-2,3,6,7-tetrahydro-1*H*,5*H*-pyrido[3,2,1-*ij*]quinolin-8-yl)dimethylsilane (S6): Dimethylbis(2,3,6,7-tetrahydro-1*H*,5*H*-pyrido[3,2,1-*ij*]quinolin-8-yl)silane (**30**; 980 mg, 2.43 mmol) was taken up in DMF (12 mL), and *N*-bromosuccinimide (866 mg, 4.87 mmol, 2 eq) was added portion-wise over 5 min. The reaction was stirred at room temperature for 2 h. It was subsequently diluted with water and extracted with EtOAc (2×). The combined organic extracts were washed with water and brine, dried over anhydrous $MgSO_4$, filtered, and concentrated *in vacuo*. Purification by silica gel chromatography (0–20% Et_2O/hexanes, linear gradient) afforded 690 mg (51%) of dibromide **S6** as a white solid. ^{1}H NMR (DMSO-d_6, 400 MHz, 350 K) δ 6.87 (s, 2H), 3.10 (t, *J* = 6.1 Hz, 4H), 3.03 (t, *J* = 6.0 Hz, 4H), 2.66 (t, *J* = 6.4 Hz, 4H), 2.63 (t, *J* = 6.4 Hz, 4H), 1.83 (p, *J* = 6.3 Hz, 4H), 1.72 (p, *J* = 6.2 Hz, 4H), 0.71 (s, 6H); ^{13}C NMR (DMSO-d_6, 101 MHz, 350 K) δ 141.4 (C), 136.4 (C), 130.8 (CH), 128.6 (C), 124.1 (C), 113.9 (C), 49.4 (CH_2), 48.6 (CH_2), 28.2 (CH_2), 26.7 (CH_2), 21.4 (CH_2), 20.8 (CH_2), 7.1 (CH_3); HRMS (ESI) calcd for $C_{26}H_{33}Br_2N_2Si$ $[M+H]^+$ 561.0754, found 561.0751.

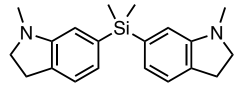

Dimethylbis(1-methylindolin-6-yl)silane (S8): A solution of 6-bromo-1-methylindoline (**S7**; 3.30 g, 15.56 mmol, 2.4 eq) in THF (40 mL) was cooled to -78 °C under nitrogen. *n*-Butyllithium (2.5 M in hexanes, 6.22 mL, 15.56 mmol, 2.4 eq) was added, and the reaction was stirred at -78 °C for 30 min. Dichlorodimethylsilane (782 µL, 6.48 mmol) was then added. The dry ice bath was removed, and the reaction was stirred at room temperature for 2 h. It was subsequently quenched with saturated NH_4Cl, diluted with water, and extracted with EtOAc (2×). The combined organic extracts were washed with brine, dried over anhydrous $MgSO_4$, filtered, and concentrated *in vacuo*. Purification by flash chromatography on silica gel (0–25% EtOAc/hexanes, linear gradient) afforded 2.03 g (97%) of **S8** as a colorless oil. ^{1}H NMR ($CDCl_3$, 400 MHz) δ 7.08 (dq, *J* = 7.2, 1.0 Hz, 2H), 6.86 (dd, *J* = 7.1, 0.9 Hz, 2H), 6.65 (bs, 2H), 3.27 (t, *J* = 8.1 Hz, 4H), 2.93 (td, *J* = 8.1, 1.1 Hz, 4H), 2.74 (s, 6H), 0.50 (s, 6H); ^{13}C NMR ($CDCl_3$, 101 MHz) δ 152.9 (C), 137.4 (C), 131.8 (C), 124.4 (CH), 124.0 (CH), 112.4 (CH), 56.1 (CH_2), 36.4 (CH_3), 28.9 (CH_2), -1.7 (CH_3); HRMS (ESI) calcd for $C_{20}H_{27}N_2Si$ $[M+H]^+$ 323.1938, found 323.1946.

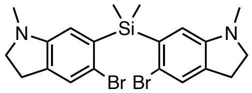

Bis(5-bromo-1-methylindolin-6-yl)dimethylsilane (S9): A solution of dimethylbis(1-methylindolin-6-yl)silane (**S8**; 1.90 g, 5.89 mmol) in DMF (30 mL) was cooled to 0 °C, and *N*-bromosuccinimide (2.10 g, 11.78 mmol, 2 eq) was added portion-wise over 15 min. The reaction was warmed to room temperature and stirred 2 h. It was subsequently diluted with water and extracted with EtOAc (2×). The combined organic extracts were washed with water and brine, dried over anhydrous $MgSO_4$, filtered, and concentrated *in vacuo*. Purification by silica gel chromatography (0–25% EtOAc/hexanes, linear gradient) afforded 2.46 g (87%) of dibromide **S9** as a white solid. ^{1}H NMR ($CDCl_3$, 400 MHz)

δ 7.20 (t, *J* = 1.0 Hz, 2H), 6.56 (s, 2H), 3.29 (t, *J* = 8.2 Hz, 4H), 2.92 (td, *J* = 8.1, 1.2 Hz, 4H), 2.70 (s, 6H), 0.71 (s, 6H); ^{13}C NMR ($CDCl_3$, 101 MHz) δ 152.1 (C), 137.0 (C), 134.2 (C), 128.8 (CH), 117.9 (C), 115.2 (CH), 56.2 (CH_2), 36.3 (CH_3), 28.5 (CH_2), -0.6 (CH_3); HRMS (ESI) calcd for $C_{20}H_{25}Br_2N_2Si$ $[M+H]^+$ 481.0128, found 481.0120.

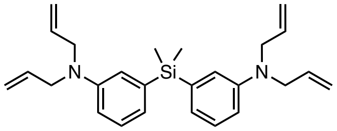

3,3'-(Dimethylsilanediyl)bis(*N*,*N*-diallylaniline) (S11): A solution of *N*,*N*-diallyl-3-bromoaniline (**S10**; 35.00 g, 138.8 mmol, 2.4 eq) in THF (300 mL) was cooled to -78 °C under nitrogen. *n*-Butyllithium (2.5 M in hexanes, 55.52 mL, 138.8 mmol, 2.4 eq) was added, and the reaction was stirred at -78 °C for 30 min. Dichlorodimethylsilane (6.98 mL, 57.83 mmol) was then added. The dry ice bath was removed, and the reaction was stirred at room temperature for 2 h. It was subsequently quenched with saturated NH_4Cl, diluted with water, and extracted with EtOAc (2×). The combined organic extracts were washed with brine, dried over anhydrous $MgSO_4$, filtered, and concentrated *in vacuo*. Purification by flash chromatography on silica gel (0–5% Et_2O/hexanes, linear gradient) afforded 22.92 g (98%) of **S11** as a colorless oil. ^{1}H NMR ($CDCl_3$, 400 MHz) δ 7.18 (dd, *J* = 8.3, 7.1 Hz, 2H), 6.89 – 6.87 (m, 2H), 6.85 (dt, *J* = 7.1, 1.0 Hz, 2H), 6.70 (ddd, *J* = 8.3, 2.8, 1.0 Hz, 2H), 5.83 (ddt, *J* = 17.1, 10.1, 5.0 Hz, 4H), 5.14 (dq, *J* = 17.4, 1.8 Hz, 4H), 5.12 (dq, *J* = 10.4, 1.7 Hz, 4H), 3.89 (dt, *J* = 4.9, 1.7 Hz, 8H), 0.48 (s, 6H); ^{13}C NMR ($CDCl_3$, 101 MHz) δ 148.1 (C), 139.0 (C), 134.3 (CH), 128.6 (CH), 122.4 (CH), 118.3 (CH), 116.2 (CH_2), 113.3 (CH), 53.0 (CH_2), -2.2 (CH_3); HRMS (ESI) calcd for $C_{26}H_{35}N_2Si$ $[M+H]^+$ 403.2564, found 403.2565.

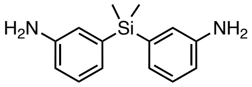

3,3'-(Dimethylsilanediyl)dianiline (S12): A round-bottom flask equipped with a reflux condenser was charged with $Pd(PPh_3)_4$ (5.74 g, 4.97 mmol, 0.1 eq) and 1,3-dimethylbarbituric acid (62.04 g, 397.4 mmol, 8 eq). The flask was sealed and evacuated/backfilled with nitrogen (3×). A solution of 3,3'-(dimethylsilanediyl)bis(*N*,*N*-diallylaniline) (**S11**; 20.00 g, 49.67 mmol) in CH_2Cl_2 (500 mL) was added via cannula, and the resulting mixture was stirred at reflux for 18 h. The reaction was cooled to room temperature and slowly diluted with saturated $NaHCO_3$ (400 mL) while stirring vigorously. The layers were separated, and the aqueous layer was extracted again with CH_2Cl_2. The combined organic extracts were dried over anhydrous $MgSO_4$, filtered, and concentrated *in vacuo*. The residue was purified twice by silica gel chromatography (10–100% EtOAc/hexanes, linear gradient) to afford 10.34 g (86%) of **S12** as an off-white solid. ^{1}H NMR ($CDCl_3$, 400 MHz) δ 7.15 (ddd, *J* = 7.8, 7.1, 0.5 Hz, 2H), 6.92 (dt, *J* = 7.2, 1.1 Hz, 2H), 6.83 – 6.79 (m, 2H), 6.68 (ddd, *J* = 7.9, 2.5, 1.1 Hz, 2H), 3.58 (s, 4H), 0.48 (s, 6H); ^{13}C NMR ($CDCl_3$, 101 MHz) δ 145.8 (C), 139.5 (C), 128.9 (CH), 124.6 (CH), 120.9 (CH), 116.1 (CH), -2.3 (CH_3); HRMS (ESI) calcd for $C_{14}H_{19}N_2Si$ $[M+H]^+$ 243.1312, found 243.1315.

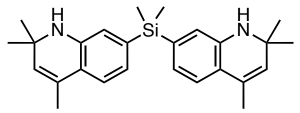

Dimethylbis(2,2,4-trimethyl-1,2-dihydroquinolin-7-yl)silane (35): 3,3'-(Dimethylsilanediyl)dianiline (**S12**; 10.00 g, 41.25 mmol) and iodine (4.19 g, 16.50 mmol, 0.4 eq) were combined in acetone (250 mL) and stirred at reflux for 48 h. The reaction was then cooled to room temperature, concentrated to dryness, and purified by silica gel chromatography (0–30% Et_2O/hexanes, linear gradient) to afford 5.05 g (30%) of **35** as an off-white solid. ^1H NMR ($CDCl_3$, 400 MHz) δ 7.03 (d, *J* = 7.5 Hz, 2H), 6.80 (dd, *J* = 7.5, 1.1 Hz, 2H), 6.54 (d, *J* = 1.1 Hz, 2H), 5.30 (q, *J* = 1.5 Hz, 2H), 3.64 (s, 2H), 1.97 (d, *J* = 1.4 Hz, 6H), 1.26 (s, 12H), 0.44 (s, 6H); ^{13}C NMR ($CDCl_3$, 101 MHz) δ 142.5 (C), 138.6 (C), 128.9 (CH), 128.6 (C), 123.2 (CH), 123.0 (CH), 122.2 (C), 118.7 (CH), 52.0 (C), 31.4 (CH_3), 18.7 (CH_3), -2.2 (CH_3); HRMS (ESI) calcd for $C_{26}H_{35}N_2Si$ $[M+H]^+$ 403.2564, found 403.2562.

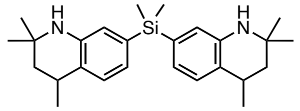

Dimethylbis(2,2,4-trimethyl-1,2,3,4-tetrahydroquinolin-7-yl)silane (S13): Dimethylbis(2,2,4-trimethyl-1,2-dihydroquinolin-7-yl)silane (**35**; 1.48 g, 3.68 mmol) was dissolved in THF (35 mL) in a round-bottom flask under nitrogen, and Pd/C (10%, 782 mg, 0.735 mmol, 0.2 eq) was added. The sealed flask was evacuated/backfilled with H_2 from a balloon (4×) and then stirred under the H_2 balloon at room temperature for 18 h. The reaction mixture was filtered through Celite with EtOAc and concentrated *in vacuo*. Silica gel chromatography (0–25% Et_2O/hexanes, linear gradient) yielded 1.25 g (84%) of **S13** as a white foam (mixture of diastereomers). ^1H NMR ($CDCl_3$, 400 MHz) δ 7.14 (d, *J* = 7.6 Hz, 2H), 6.83 (d, *J* = 7.5 Hz, 2H), 6.61 – 6.57 (m, 2H), 3.57 (s, 2H), 2.96 – 2.84 (m, 2H), 1.72 (dd, *J* = 12.9, 5.5 Hz, 2H), 1.43 (t, *J* = 12.6 Hz, 2H), 1.32 (d, *J* = 6.7 Hz, 6H), 1.22 (s, 6H), 1.17 (s, 6H), 0.44 (s, 6H); Analytical HPLC: t_R = 10.0 min, >99% purity (10–95% $MeCN/H_2O$, linear gradient, with constant 0.1% v/v TFA additive; 20 min run; 1 mL/min flow; ESI; positive ion mode; detection at 230 nm); HRMS (ESI) calcd for $C_{26}H_{39}N_2Si$ $[M+H]^+$ 407.2877, found 407.2873.

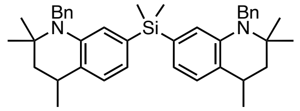

Bis(1-benzyl-2,2,4-trimethyl-1,2,3,4-tetrahydroquinolin-7-yl)dimethylsilane (32): Dimethylbis(2,2,4-trimethyl-1,2,3,4-tetrahydroquinolin-7-yl)silane (**S13**; 1.15 g, 2.83 mmol), K_2CO_3 (2.34 g, 16.97 mmol, 6 eq), and KI (188 mg, 1.13 mmol, 0.4 eq) were combined in DMF (30 mL) in a round-bottom flask. Benzyl bromide (2.90 g, 16.97 mmol, 6 eq) was added, the flask was sealed, and the contents were stirred at 100 °C for 18 h. The reaction was cooled to room temperature, diluted with water, and extracted with EtOAc (2×). The combined organic extracts were washed with water and brine, dried over anhydrous $MgSO_4$, filtered, and concentrated *in vacuo*. Silica gel chromatography (0–10% Et_2O/hexanes, linear gradient) yielded 1.59 g (96%) of **32** as a white foam (mixture of diastereomers). ^1H NMR ($CDCl_3$, 400 MHz) δ 7.28 – 7.15 (m, 10H), 7.03 (d, *J* = 7.4 Hz, 2H), 6.57 (d, *J* = 7.4 Hz, 2H), 6.42 – 6.37 (m, 2H), 4.62 (d, *J* = 17.5 Hz, 2H), 4.13 (d, *J* = 17.6 Hz, 2H), 3.04 – 2.92 (m, 2H), 1.80 (dd, *J* = 13.0, 5.0 Hz, 2H), 1.72 (td, *J*

= 12.7, 2.0 Hz, 2H), 1.34 (d, *J* = 6.6 Hz, 6H), 1.27 (s, 6H), 1.24 (s, 6H), 0.04 (s, 1.5H), 0.00 (s, 3H), -0.04 (s, 1.5H); Analytical HPLC: t_R = 13.9 min, >99% purity (65–95% MeCN/H_2O, linear gradient, with constant 0.1% v/v TFA additive; 20 min run; 1 mL/min flow; ESI; positive ion mode; detection at 254 nm); HRMS (ESI) calcd for $C_{40}H_{51}N_2Si$ $[M+H]^+$ 587.3816, found 587.3808.

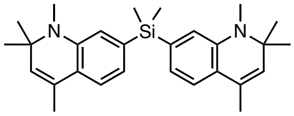

Dimethylbis(1,2,2,4-tetramethyl-1,2-dihydroquinolin-7-yl)silane (37): A solution of 7-bromo-1,2,2,4-tetramethyl-1,2-dihydroquinoline (**S14**; 10.26 g, 38.55 mmol, 2.5 eq) in THF (75 mL) was cooled to -78 °C under nitrogen. *n*-Butyllithium (2.5 M in hexanes, 15.42 mL, 38.55 mmol, 2.5 eq) was added, and the reaction was stirred at -78 °C for 30 min. Dichlorodimethylsilane (1.86 mL, 15.42 mmol) was then added. The dry ice bath was removed, and the reaction was stirred at room temperature for 2 h. It was subsequently quenched with saturated NH_4Cl, diluted with water, and extracted with EtOAc (2×). The combined organic extracts were washed with brine, dried over anhydrous $MgSO_4$, filtered, and concentrated *in vacuo*. Purification by flash chromatography on silica gel (0–5% Et_2O/hexanes, linear gradient) afforded 6.00 g (90%) of **37** as a colorless oil. ^1H NMR ($CDCl_3$, 400 MHz) δ 7.03 (d, *J* = 7.4 Hz, 2H), 6.84 (dd, *J* = 7.3, 1.1 Hz, 2H), 6.68 (d, *J* = 1.0 Hz, 2H), 5.28 (q, *J* = 1.5 Hz, 2H), 2.76 (s, 6H), 1.96 (d, *J* = 1.4 Hz, 6H), 1.28 (s, 12H), 0.50 (s, 6H); ^{13}C NMR ($CDCl_3$, 101 MHz) δ 144.4 (C), 138.8 (C), 130.6 (CH), 128.3 (C), 124.0 (C), 122.59 (CH), 122.57 (CH), 116.1 (CH), 56.3 (C), 30.6 (CH_3), 27.4 (CH_3), 18.7 (CH_3), -2.0 (CH_3); HRMS (ESI) calcd for $C_{28}H_{39}N_2Si$ $[M+H]^+$ 431.2877, found 431.2874.

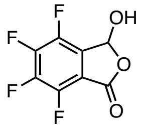

Tetrafluorophthalaldehydic acid (5): A solution of 2,3,4,5-tetrafluorobenzoic acid (2.00 g, 10.31 mmol) in THF (30 mL) was cooled to -78 °C under nitrogen. *N*-Butyllithium (2.5 M in hexanes, 9.89 mL, 24.73 mmol, 2.4 eq) was added, and the reaction was stirred at -78 °C for 3 h. Methyl formate (3.18 mL, 51.53 mmol, 5 eq) was added in one portion; the reaction was then stirred at -78 °C for 30 min, warmed to room temperature, and stirred for 2 h. Following the addition of water (75 mL) and CH_2Cl_2 (75 mL), the pH was adjusted to 10–11 with 2 M NaOH. The aqueous layer was washed again with CH_2Cl_2, acidified to pH ~ 2 with 2 M HCl, and extracted with EtOAc (2×). The combined EtOAc layers were dried over anhydrous $MgSO_4$, filtered, and evaporated. Purification by silica gel chromatography (5–100% EtOAc/hexanes, linear gradient, with constant 1% v/v AcOH additive) afforded **5** as a white solid (1.58 g, 69%). ^1H NMR (DMSO-d_6, 400 MHz) δ 8.77 (s, 1H), 6.88 (s, 1H); ^{19}F NMR (DMSO-d_6, 376 MHz) δ -140.20 (td, *J* = 20.6, 8.4 Hz, 1F), -142.72 (t, *J* = 20.1 Hz, 1F), -143.93 – -144.25 (m, 1F), -150.36 (t, *J* = 20.5 Hz, 1F); Analytical HPLC: t_R = 9.2 min, >99% purity (10–95% MeCN/H_2O, linear gradient, with constant 0.1% v/v TFA additive; 20 min run; 1 mL/min flow; ESI; positive ion mode; detection at 230 nm); HRMS (ESI) calcd for $C_8HF_4O_3$ $[M-H]^-$ 220.9867, found 220.9861.

RHODAMINE SYNTHESIS VIA LACTOL (PHTHALALDEHYDIC ACID) CONDENSATION (SCHEME S2)

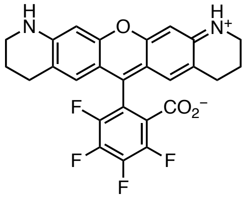

4,5,6,7-Tetrafluoro-Q-rhodamine (FRh_Q, 14): 1,2,3,4-Tetrahydroquinolin-7-ol (**13**; 141 mg, 0.946 mmol, 2.1 eq), tetrafluorophthalaldehydic acid (**5**; 100 mg, 0.450 mmol), 2,2,2-trifluoroethanol (8 mL), and water (2 mL) were combined in a round-bottom flask. The reaction mixture was sparged with O_2 from a balloon for 10 min, then stirred at 80 °C under the O_2 balloon for 24 h. The reaction was then cooled to room temperature, diluted with MeOH, deposited onto Celite, and concentrated to dryness. Purification by silica gel chromatography (0–20% MeOH (2 M NH_3)/CH_2Cl_2, linear gradient; dry load with Celite) followed by reverse phase HPLC (20–70% MeCN/H_2O, linear gradient, with constant 0.1% v/v TFA additive) provided **14** as a dark red solid (152 mg, 57%, TFA salt). ^1H NMR (CD_3OD, 400 MHz) δ 6.98 (s, 2H), 6.64 (s, 2H), 3.51 – 3.44 (m, 4H), 2.78 (t, *J* = 6.2 Hz, 4H), 1.93 (p, *J* = 6.3 Hz, 4H); ^{19}F NMR (CD_3OD, 376 MHz) δ -75.53 (s, 3F), -136.17 – -136.31 (m, 1F), -137.42 – -137.56 (m, 1F), -151.06 – -151.23 (m, 1F), -152.47 – -152.64 (m, 1F); Analytical HPLC: t_R = 11.7 min, >99% purity (10–95% MeCN/H_2O, linear gradient, with constant 0.1% v/v TFA additive; 20 min run; 1 mL/min flow; ESI; positive ion mode; detection at 575 nm); HRMS (ESI) calcd for $C_{26}H_{19}F_4N_2O_3$ $[M+H]^+$ 483.1326, found 483.1321.

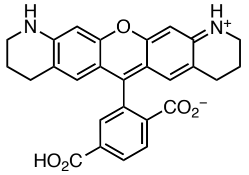

6-Carboxy-Q-rhodamine (S17): 1,2,3,4-Tetrahydroquinolin-7-ol (**13**; 78.3 mg, 0.525 mmol, 2.1 eq), 4-carboxyphthalaldehydic acid[3] (**109**; 48.6 mg, 0.250 mmol), 2,2,2-trifluoroethanol (4 mL), and water (1 mL) were combined in a crimp-top vial. The reaction mixture was sparged with O_2 from a balloon for 10 min, then stirred at 80 °C under the O_2 balloon for 24 h. The reaction was then cooled to room temperature, and concentrated to dryness. Purification by reverse phase HPLC (20–40% MeCN/H_2O, linear gradient, with constant 0.1% v/v TFA additive) provided **S17** as a rust red solid (74.1 mg, 52%, TFA salt). ^1H NMR (DMSO-d_6, 400 MHz) δ 13.61 (s, 1H), 13.35 (s, 1H), 8.80 (s, 2H), 8.31 (d, *J* = 8.1 Hz, 1H), 8.27 (dd, *J* = 8.2, 1.6 Hz, 1H), 7.83 (d, *J* = 1.5 Hz, 1H), 6.69 (s, 2H), 6.68 (s, 2H), 3.42 – 3.34 (m, 4H), 2.64 (t, *J* = 6.4 Hz, 4H), 1.84 – 1.70 (m, 4H); ^{13}C NMR (DMSO-d_6, 101 MHz) δ 166.1 (C), 165.7 (C), 155.7 (C), 155.4 (C), 154.8 (C), 134.5 (C), 134.2 (C), 133.8 (C), 131.4 (CH), 130.7 (CH), 130.7 (CH), 128.3 (CH), 123.4 (C), 112.8 (C), 96.4 (CH), 40.9 (CH_2), 26.1 (CH_2), 19.8 (CH_2); Analytical HPLC: t_R = 9.4 min, >99% purity (10–95% MeCN/H_2O, linear gradient, with constant 0.1% v/v TFA additive; 20 min run; 1 mL/min flow; ESI; positive ion mode; detection at 550 nm); HRMS (ESI) calcd for $C_{27}H_{23}N_2O_5$ $[M+H]^+$ 455.1601, found 455.1598.

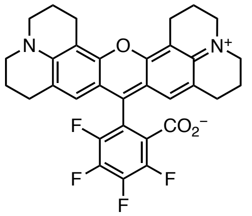

4,5,6,7-Tetrafluoro-rhodamine 101 (FRh_{101}, 16): 8-Hydroxyjulolidine (**15**; 358 mg, 1.89 mmol, 2.1 eq) and tetrafluorophthalaldehydic acid (**5**; 200 mg, 0.901 mmol) were combined in 2,2,2-trifluoroethanol (18 mL) in a round-bottom flask. The reaction mixture was sparged with O_2 from a balloon for 10 min, then stirred at 80 °C under the O_2 balloon for 24 h. The reaction was cooled to room temperature, concentrated *in vacuo*, and purified by silica gel chromatography (0–20% MeOH (2 M NH_3)/CH_2Cl_2, linear gradient) to provide **16** as a dark purple solid (385 mg, 76%). ^{1}H NMR (CD_3OD, 400 MHz) δ 6.87 (s, 2H), 3.59 – 3.48 (m, 8H), 3.05 (t, J = 6.4 Hz, 4H), 2.86 – 2.70 (m, 4H), 2.09 (p, J = 6.3 Hz, 4H), 1.97 (p, J = 6.3 Hz, 4H); ^{19}F NMR (CD_3OD, 376 MHz) δ -139.20 (ddd, J = 21.3, 12.6, 3.4 Hz, 1F), -141.27 (ddd, J = 22.5, 12.6, 3.5 Hz, 1F), -154.15 (ddd, J = 22.7, 18.8, 3.8 Hz, 1F), -158.00 (ddd, J = 22.4, 19.0, 3.5 Hz, 1F); Analytical HPLC: t_R = 13.5 min, >99% purity (10–95% MeCN/H_2O, linear gradient, with constant 0.1% v/v TFA additive; 20 min run; 1 mL/min flow; ESI; positive ion mode; detection at 600 nm); HRMS (ESI) calcd for $C_{32}H_{27}F_4N_2O_3$ $[M+H]^+$ 563.1952, found 563.1943.

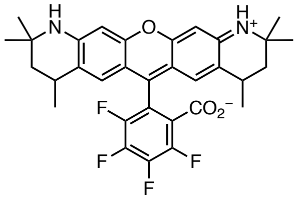

JF_{563} (18): 7,7'-Oxybis(2,2,4-trimethyl-1,2,3,4-tetrahydroquinoline) (**17**; 200 mg, 0.549 mmol) and tetrafluorophthalaldehydic acid (**5**; 183 mg, 0.823 mmol, 1.5 eq) were combined in 2,2,2-trifluoroethanol (11 mL) in a round-bottom flask. The reaction mixture was sparged with O_2 from a balloon for 10 min, then stirred at 80 °C under the O_2 balloon for 24 h. The reaction was cooled to room temperature, concentrated *in vacuo*, and purified by silica gel chromatography (0–20% MeOH (2 M NH_3)/CH_2Cl_2, linear gradient) to provide 84.2 mg (27%) of **18** as a dark red-purple solid (mixture of diastereomers). ^{1}H NMR (CD_3OD, 400 MHz) δ 7.15 – 7.10 (m, 2H), 6.63 – 6.58 (m, 2H), 2.99 – 2.86 (m, 2H), 1.93 – 1.82 (m, 2H), 1.48 – 1.22 (m, 20H); ^{19}F NMR (CD_3OD, 376 MHz) δ -138.47 – -139.23 (m, 1F), -139.52 – -140.42 (m, 1F), -152.78 – -153.26 (m, 1F), -156.57 – -158.07 (m, 1F); Analytical HPLC: t_R (three isomers) = 13.7 min, 14.0 min, 14.2 min; >99% total purity (10–95% MeCN/H_2O, linear gradient, with constant 0.1% v/v TFA additive; 20 min run; 1 mL/min flow; ESI; positive ion mode; detection at 550 nm); HRMS (ESI) calcd for $C_{32}H_{31}F_4N_2O_3$ $[M+H]^+$ 567.2265, found 567.2264.

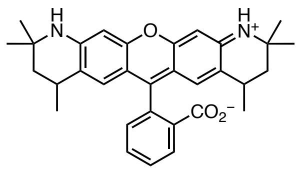

2-(2,2,4,8,10,10-Hexamethyl-1,2,3,4,8,9,10,11-octahydropyrano[3,2-*g*:5,6-*g'*]diquinolin-13-ium-6-yl)benzoate (99): 7,7'-Oxybis(2,2,4-trimethyl-1,2,3,4-tetrahydroquinoline) (**17**; 400 mg, 1.10 mmol) and phthalaldehydic acid (**S16**; 165 mg, 1.10 mmol, 1 eq) were combined in 2,2,2-trifluoroethanol (22 mL) in a round-bottom flask. The reaction mixture was sparged with O_2 from a balloon for 10 min, then stirred at 80 °C under the O_2 balloon for 18 h. The reaction was cooled to room temperature, concentrated *in vacuo*, and purified by silica gel chromatography (0–20% MeOH (2 M NH_3)/CH_2Cl_2, linear gradient) to provide 151 mg (28%) of **99** as a red solid (mixture of diastereomers). An analytically pure sample for spectral characterization was obtained by reverse phase HPLC (10–95% MeCN/H_2O, linear gradient, with constant 0.1% TFA). ^{1}H NMR (CD_3OD, 400 MHz, TFA salt) δ 8.35 – 8.29 (m, 1H), 7.88 – 7.77 (m, 2H), 7.43 – 7.38 (m, 1H), 6.98 – 6.95 (m, 1H), 6.94 – 6.91 (m, 1H), 6.67 – 6.62 (m, 2H), 2.94 – 2.81 (m, 2H), 1.90 – 1.81 (m, 2H), 1.43 – 1.33 (m, 8H), 1.32 – 1.26 (m, 6H), 1.16 – 1.09 (m, 6H); Analytical HPLC: t_R (three isomers) = 13.4 min, 13.6 min, 13.8 min; >99% total purity (10–95% MeCN/H_2O, linear gradient, with constant 0.1% v/v TFA additive; 20 min run; 1 mL/min flow; ESI; positive ion mode; detection at 550 nm); HRMS (ESI) calcd for $C_{32}H_{35}N_2O_3$ $[M+H]^+$ 495.2642, found 495.2634.

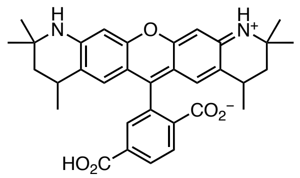

4-Carboxy-2-(2,2,4,8,10,10-hexamethyl-1,2,3,4,8,9,10,11-octahydropyrano[3,2-*g*:5,6-*g*']diquinolin-13-ium-6-yl)benzoate (S18): 7,7'-Oxybis(2,2,4-trimethyl-1,2,3,4-tetrahydroquinoline) (**17**; 300 mg, 0.823 mmol) and 4-carboxyphthalaldehydic acid (**109**; 160 mg, 0.823 mmol, 1 eq) were combined in 2,2,2-trifluoroethanol (15 mL) in a round-bottom flask. The reaction mixture was sparged with O_2 from a balloon for 10 min, then stirred at 80 °C under the O_2 balloon for 18 h. The reaction was cooled to room temperature, diluted with MeOH, deposited onto Celite, and concentrated *in vacuo.* Silica gel chromatography (0–40% MeOH/CH_2Cl_2, linear gradient, with constant 1% v/v AcOH additive; dry load with Celite) produced a broad elution profile for the desired product, so the numerous fractions containing **S18** were concentrated to dryness, suspended in water (50 mL), sonicated (5 min), and filtered. The resulting filter cake was washed (water, MeCN, EtOAc, Et_2O) and dried to afford 229 mg (46%, acetate salt) of **S18** as a dark red-brown solid (mixture of diastereomers). ^{1}H NMR (CD_3OD with 1% TFA, 400 MHz) δ 8.42 – 8.35 (m, 2H), 8.00 – 7.95 (m, 1H), 6.96 – 6.92 (m, 1H), 6.92 – 6.89 (m, 1H), 6.68 – 6.63 (m, 2H), 2.94 – 2.83 (m, 2H), 1.90 – 1.82 (m, 2H), 1.45 – 1.35 (m, 8H), 1.32 – 1.28 (m, 6H), 1.17 – 1.09 (m, 6H); Analytical HPLC: t_R (three isomers) = 12.2 min, 12.5 min, 12.8 min; >99% total purity (30–50% MeCN/H_2O, linear gradient, with constant 0.1% v/v TFA additive; 20 min run; 1 mL/min flow; ESI; positive ion mode; detection at 550 nm); HRMS (ESI) calcd for $C_{33}H_{35}N_2O_5$ $[M+H]^+$ 539.2540, found 539.2543.

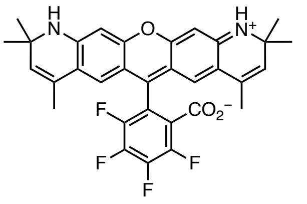

2,3,4,5-Tetrafluoro-6-(2,2,4,8,10,10-hexamethyl-1,2,10,11-tetrahydropyrano[3,2-*g*:5,6-*g′*]diquinolin-13-ium-6-yl)benzoate (20): 7,7'-Oxybis(2,2,4-trimethyl-1,2-dihydroquinoline) (**19**; 180 mg, 0.500 mmol) and tetrafluoro-phthalaldehydic acid (**5**; 111 mg, 0.500 mmol, 1 eq), 2,2,2-trifluoroethanol (8 mL), and water (2 mL) were combined in a round-bottom flask. The reaction mixture was sparged with O_2 from a balloon for 10 min, then stirred at 80 °C under the O_2 balloon for 24 h. The reaction was cooled to room temperature, concentrated *in vacuo*, and purified by silica gel chromatography (0–20% MeOH (2 M NH_3)/CH_2Cl_2, linear gradient) to provide **20** as a dark purple solid (82.6 mg, 29%). ^1H NMR (CD_3OD, 400 MHz) δ 6.90 (s, 2H), 6.54 (s, 2H), 5.65 (q, J = 1.5 Hz, 2H), 1.91 (d, J = 1.4 Hz, 6H), 1.41 (s, 6H), 1.39 (s, 6H); ^{19}F NMR (CD_3OD, 376 MHz) δ -138.77 (ddd, J = 21.2, 12.5, 4.0 Hz, 1F), -139.93 – -140.09 (m, 1F), -152.85 (ddd, J = 22.6, 19.1, 4.0 Hz, 1F), -157.01 – -157.25 (m, 1F); Analytical HPLC: t_R = 13.8 min, >99% purity (10–95% MeCN/H_2O, linear gradient, with constant 0.1% v/v TFA additive; 20 min run; 1 mL/min flow; ESI; positive ion mode; detection at 600 nm); HRMS (ESI) calcd for $C_{32}H_{27}F_4N_2O_3$ $[M+H]^+$ 563.1952, found 563.1942.

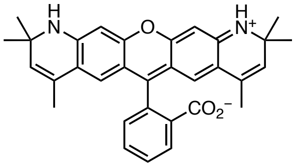

2-(2,2,4,8,10,10-Hexamethyl-1,2,10,11-tetrahydropyrano[3,2-*g*:5,6-*g′*]diquinolin-13-ium-6-yl)benzoate (101): 7,7'-Oxybis(2,2,4-trimethyl-1,2-dihydroquinoline) (**19**; 180 mg, 0.500 mmol), phthalaldehydic acid (**S16**; 75.1 mg, 0.500 mmol, 1 eq), 2,2,2-trifluoroethanol (8 mL), and water (2 mL) were combined in a round-bottom flask. The reaction mixture was sparged with O_2 from a balloon for 10 min, then stirred at 80 °C under the O_2 balloon for 24 h. The reaction was cooled to room temperature, concentrated *in vacuo*, and purified by silica gel chromatography (0–20% MeOH (2 M NH_3)/CH_2Cl_2, linear gradient) to provide **101** as a dark purple solid (68.4 mg, 28%). ^1H NMR (CD_3OD, 400 MHz) δ 8.15 – 8.10 (m, 1H), 7.70 – 7.62 (m, 2H), 7.30 – 7.24 (m, 1H), 6.88 (s, 2H), 6.53 (s, 2H), 5.58 (q, J = 1.4 Hz, 2H), 1.76 (d, J = 1.4 Hz, 6H), 1.38 (s, 6H), 1.37 (s, 6H); ^{13}C NMR (CD_3OD, 101 MHz) δ 171.3 (C), 160.1 (C), 159.0 (C), 154.8 (C), 134.4 (C), 132.5 (CH), 131.97 (CH), 131.94 (C), 131.6 (CH), 131.01 (CH), 130.98 (CH), 127.4 (C), 124.6 (CH), 122.5 (C), 115.4 (C), 97.0 (CH), 55.1 (C), 31.97 (CH_3), 31.94 (CH_3), 18.1 (CH_3); Analytical HPLC: t_R = 13.6 min, >99% purity (10–95% MeCN/H_2O, linear gradient, with constant 0.1% v/v TFA additive; 20 min run; 1 mL/min flow; ESI; positive ion mode; detection at 550 nm); HRMS (ESI) calcd for $C_{32}H_{31}N_2O_3$ $[M+H]^+$ 491.2329, found 491.2325.

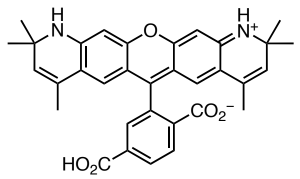

4-Carboxy-2-(2,2,4,8,10,10-hexamethyl-1,2,10,11-tetrahydropyrano[3,2-*g*:5,6-*g*']diquinolin-13-ium-6-yl)-benzoate (S19): 7,7'-Oxybis(2,2,4-trimethyl-1,2-dihydroquinoline) (**19**; 300 mg, 0.832 mmol) and 4-carboxyphthalaldehydic acid (**109**; 162 mg, 0.832 mmol, 1 eq) were combined in 2,2,2-trifluoroethanol (15 mL) in a round-bottom flask. The reaction mixture was sparged with O_2 from a balloon for 10 min, then stirred at 80 °C under the O_2 balloon for 18 h. The reaction was cooled to room temperature, diluted with MeOH, deposited onto Celite, and concentrated *in vacuo.* Purification by silica gel chromatography (0–20% MeOH/CH_2Cl_2, linear gradient, with constant 1% v/v AcOH additive; dry load with Celite) afforded **S19** as a dark red solid (197 mg, 40%, acetate salt). An analytically pure sample for spectral characterization was obtained by reverse phase HPLC (20–50% MeCN/H_2O, linear gradient, with constant 0.1% TFA). ^{1}H NMR (CD_3OD, 400 MHz, TFA salt) δ 8.39 (dd, *J* = 8.2, 1.5 Hz, 1H), 8.36 (dd, *J* = 8.2, 0.7 Hz, 1H), 8.01 (dd, *J* = 1.6, 0.7 Hz, 1H), 6.71 (s, 2H), 6.59 (s, 2H), 5.64 (q, *J* = 1.5 Hz, 2H), 1.74 (d, *J* = 1.3 Hz, 6H), 1.41 (s, 12H); ^{13}C NMR (CD_3OD, 101 MHz, TFA salt) δ 168.0 (C), 167.8 (C), 159.0 (C), 156.7 (C), 155.0 (C), 136.9 (C), 135.9 (C), 135.1 (C), 133.0 (CH), 132.51 (CH), 132.48 (CH), 132.3 (CH), 127.1 (C), 123.6 (CH), 123.0 (C), 115.1 (C), 97.3 (CH), 55.3 (C), 31.91 (CH_3), 31.89 (CH_3), 18.1 (CH_3); Analytical HPLC: t_R = 11.3 min, >99% purity (10–95% MeCN/H_2O, linear gradient, with constant 0.1% v/v TFA additive; 20 min run; 1 mL/min flow; ESI; positive ion mode; detection at 575 nm); HRMS (ESI) calcd for $C_{33}H_{31}N_2O_5$ $[M+H]^+$ 535.2227, found 535.2231.

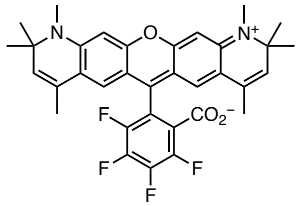

2,3,4,5-Tetrafluoro-6-(1,2,2,4,8,10,10,11-octamethyl-1,2,10,11-tetrahydropyrano[3,2-*g*:5,6-*g*′]diquinolin-13-ium-6-yl)benzoate (11): 7,7'-Oxybis(1,2,2,4-tetramethyl-1,2-dihydroquinoline) (**12**; 1.00 g, 2.57 mmol) and tetrafluorophthalaldehydic acid (**5**; 572 mg, 2.57 mmol, 1 eq) were combined in 2,2,2-trifluoroethanol (50 mL) in a round-bottom flask. The reaction mixture was sparged with O_2 from a balloon for 10 min, then stirred at 80 °C under the O_2 balloon for 24 h. The reaction was cooled to room temperature, concentrated *in vacuo*, and purified by silica gel chromatography (0–20% MeOH (2 M NH_3)/CH_2Cl_2, linear gradient) to provide **11** as a dark blue-purple solid (1.00 g, 66%). ^{1}H NMR (CD_3OD, 400 MHz) δ 6.93 (s, 2H), 6.81 (s, 2H), 5.68 (q, *J* = 1.5 Hz, 2H), 3.19 (s, 6H), 1.93 (d, *J* = 1.4 Hz, 6H), 1.51 (s, 6H), 1.50 (s, 6H); ^{19}F NMR (CD_3OD, 376 MHz) δ -138.84 (ddd, *J* = 21.1, 12.6, 4.0 Hz, 1F), -140.19 (ddd, *J* = 22.4, 12.6, 3.7 Hz, 1F), -152.79 (ddd, *J* = 23.0, 19.2, 4.1 Hz, 1F), -157.56 (ddd, *J* = 21.4, 19.4, 3.8 Hz, 1F); Analytical HPLC: t_R = 14.2 min, >99% purity (10–95% MeCN/H_2O, linear gradient, with constant 0.1%

v/v TFA additive; 20 min run; 1 mL/min flow; ESI; positive ion mode; detection at 600 nm); HRMS (ESI) calcd for $C_{34}H_{31}F_4N_2O_3$ $[M+H]^+$ 591.2265, found 591.2258.

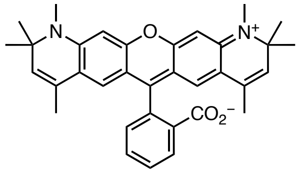

2-(1,2,2,4,8,10,10,11-Octamethyl-1,2,10,11-tetrahydropyrano[3,2-*g*:5,6-*g'*]diquinolin-13-ium-6-yl)benzoate (103): 7,7'-Oxybis(1,2,2,4-tetramethyl-1,2-dihydroquinoline) (**12**; 200 mg, 0.515 mmol) and phthalaldehydic acid (**S16**; 77.3 mg, 0.515 mmol, 1 eq) were combined in 2,2,2-trifluoroethanol (10 mL) in a round-bottom flask. The reaction mixture was sparged with O_2 from a balloon for 10 min, then stirred at 80 °C under the O_2 balloon for 24 h. The reaction was cooled to room temperature, concentrated *in vacuo*, and purified by silica gel chromatography (0–20% MeOH (2 M NH_3)/CH_2Cl_2, linear gradient) to provide **103** as a dark purple solid (185 mg, 69%). ^1H NMR (CD_3OD, 400 MHz) δ 8.13 – 8.08 (m, 1H), 7.65 (td, J = 7.4, 1.6 Hz, 1H), 7.62 (td, J = 7.4, 1.6 Hz, 1H), 7.27 – 7.22 (m, 1H), 6.94 (s, 2H), 6.76 (s, 2H), 5.62 – 5.57 (m, 2H), 3.16 (s, 6H), 1.78 (d, J = 1.4 Hz, 6H), 1.49 (s, 6H), 1.48 (s, 6H); ^{13}C NMR (CD_3OD, 101 MHz) δ 173.3 (C), 161.5 (C), 159.6 (C), 154.5 (C), 141.9 (C), 133.6 (C), 133.4 (CH), 131.1 (CH), 130.8 (CH), 130.5 (CH), 130.4 (CH), 126.9 (C), 124.2 (C), 123.8 (CH), 115.4 (C), 96.0 (CH), 60.9 (C), 33.2 (CH_3), 29.2 (CH_3), 29.1 (CH_3), 18.3 (CH_3); Analytical HPLC: t_R = 13.9 min, 98.5% purity (10–95% MeCN/H_2O, linear gradient, with constant 0.1% v/v TFA additive; 20 min run; 1 mL/min flow; ESI; positive ion mode; detection at 600 nm); HRMS (ESI) calcd for $C_{34}H_{35}N_2O_3$ $[M+H]^+$ 519.2642, found 519.2652.

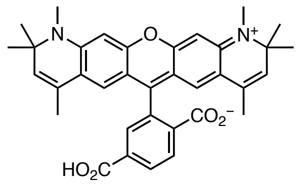

4-Carboxy-2-(1,2,2,4,8,10,10,11-octamethyl-1,2,10,11-tetrahydropyrano[3,2-*g*:5,6-*g*']diquinolin-13-ium-6-yl)-benzoate (S20): 7,7'-Oxybis(1,2,2,4-tetramethyl-1,2-dihydroquinoline) (**12**; 300 mg, 0.772 mmol) and 4-carboxyphthalaldehydic acid (**109**; 150 mg, 0.772 mmol, 1 eq) were combined in 2,2,2-trifluoroethanol (15 mL) in a round-bottom flask. The reaction mixture was sparged with O_2 from a balloon for 10 min, then stirred at 80 °C under the O_2 balloon for 18 h. The reaction was cooled to room temperature, deposited onto Celite, and concentrated *in vacuo*. Purification by silica gel chromatography (0–20% MeOH/CH_2Cl_2, linear gradient, with constant 1% v/v AcOH additive; dry load with Celite) afforded **S20** as a dark red-purple solid (387 mg, 80%, acetate salt). An analytically pure sample for spectral characterization was obtained by reverse phase HPLC (20–60% MeCN/H_2O, linear gradient, with constant 0.1% TFA). ^1H NMR (CD_3OD, 400 MHz, TFA salt) δ 8.40 (dd, J = 8.2, 1.5 Hz, 1H), 8.38 (d, J = 8.0 Hz, 1H), 8.04 – 8.02 (m, 1H), 6.84 (s, 2H), 6.73 (s, 2H), 5.67 (q, J = 1.4 Hz, 2H), 3.20 (s, 6H), 1.75 (d, J = 1.3 Hz, 6H), 1.51 (s, 12H); ^{13}C NMR (CD_3OD, 101 MHz, TFA salt) δ 167.9 (C), 167.7 (C), 159.6 (C), 156.7 (C), 154.8 (C), 136.9 (C), 135.9 (C), 134.9 (C), 134.2 (CH), 132.54 (CH), 132.47 (CH), 132.42 (CH), 126.5 (C), 124.8 (C), 122.2

(CH), 115.1 (C), 96.4 (CH), 61.2 (C), 33.4 (CH_3), 29.14 (CH_3), 29.12 (CH_3), 18.1 (CH_3); Analytical HPLC: t_R = 11.9 min, >99% purity (10–95% MeCN/H_2O, linear gradient, with constant 0.1% v/v TFA additive; 20 min run; 1 mL/min flow; ESI; positive ion mode; detection at 600 nm); HRMS (ESI) calcd for $C_{35}H_{35}N_2O_5$ $[M+H]^+$ 563.2540, found 563.2543.

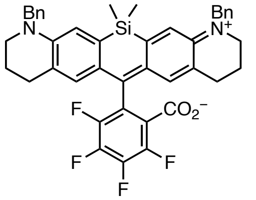

2-(1,11-Dibenzyl-13,13-dimethyl-2,3,4,8,9,10,11,13-octahydrosilino[3,2-*g*:5,6-*g'*]diquinolin-6-ylium-6(1*H*)-yl)-3,4,5,6-tetrafluorobenzoate (28): *Via lactol condensation route:* Bis(1-benzyl-1,2,3,4-tetrahydroquinolin-7-yl)dimethylsilane (**27**; 400 mg, 0.796 mmol) and tetrafluorophthalaldehydic acid (**5**; 265 mg, 1.19 mmol, 1.5 eq) were combined in 2,2,3,3,4,4,4-heptafluoro-1-butanol (4 mL) in a crimp-top vial. The reaction mixture was sparged with O_2 from a balloon for 10 min, then stirred at 95 °C under the O_2 balloon for 24 h. The reaction was cooled to room temperature, concentrated *in vacuo*, and purified by silica gel chromatography (0–50% EtOAc/hexanes, linear gradient) to provide 236 mg (42%) of **28** as a pale yellow-green solid.

Via tetrafluorobenzoic acid route: A solution of 2,3,4,5-tetrafluorobenzoic acid (**6**; 275 mg, 1.42 mmol, 2.5 eq) in THF (5 mL) was cooled to -78 °C under nitrogen. *N*-Butyllithium (2.5 M in hexanes, 1.02 mL, 2.55 mmol, 4.5 eq) was added, and the reaction was stirred at -78 °C for 3 h. A solution of 1,11-dibenzyl-13,13-dimethyl-1,2,3,4,8,9,10,11-octahydrosilino[3,2-*g*:5,6-*g*']diquinolin-6(13*H*)-one[4] (**S56**; 300 mg, 0.567 mmol) in THF (5 mL) was added; the reaction was warmed to room temperature and stirred for 72 h. It was subsequently diluted with saturated NH_4Cl and water and extracted with EtOAc (2×). The combined organic extracts were washed with brine, dried over anhydrous $MgSO_4$, filtered, and concentrated *in vacuo*. The residue was purified twice by silica gel chromatography (10–100% EtOAc/hexanes, linear gradient; then, 0–20% EtOAc/toluene, linear gradient) yielded 110 mg (28%) of **28** as a pale yellow-green solid.

^{1}H NMR ($CDCl_3$, 400 MHz) δ 7.34 – 7.28 (m, 4H), 7.26 – 7.21 (m, 6H), 6.64 (s, 2H), 6.47 (s, 2H), 4.51 (AB quartet, ν_A = 1811.4 Hz, ν_B = 1795.4 Hz, J_{AB} = 16.7 Hz, 4H), 3.40 – 3.32 (m, 4H), 2.73 – 2.58 (m, 4H), 1.94 (p, J = 6.1 Hz, 4H), 0.18 (s, 3H), 0.17 (s, 3H); ^{19}F NMR ($CDCl_3$, 376 MHz) δ -139.43 (td, J = 20.0, 8.2 Hz, 1F), -139.85 (td, J = 20.4, 3.5 Hz, 1F), -143.88 (ddd, J = 21.1, 18.6, 8.1 Hz, 1F), -152.53 (ddd, J = 21.1, 17.9, 3.4 Hz, 1F); Analytical HPLC: t_R = 15.5 min, >99% purity (10–95% MeCN/H_2O, linear gradient, with constant 0.1% v/v TFA additive; 20 min run; 1 mL/min flow; ESI; positive ion mode; detection at 700 nm); HRMS (ESI) calcd for $C_{42}H_{37}F_4N_2O_2Si$ $[M+H]^+$ 705.2555, found 705.2547.

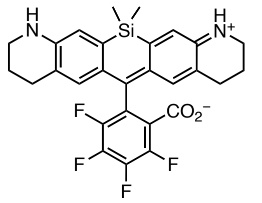

4,5,6,7-Tetrafluoro-Si-Q-rhodamine (FSiRh$_Q$, 29): A round-bottom flask was charged with Pd/C (10%, 69.2 mg, 65.0 µmol, 0.2 eq) under nitrogen, and 2-(1,11-dibenzyl-13,13-dimethyl-2,3,4,8,9,10,11,13-octahydrosilino[3,2-*g*:5,6-*g'*]diquinolin-6-ylium-6(1*H*)-yl)-3,4,5,6-tetrafluorobenzoate (**28**; 229 mg, 0.325 mmol) in THF (10 mL) was added. The sealed flask was evacuated/backfilled with H_2 from a balloon (4×) and then stirred under the H_2 balloon at room temperature for 24 h. The reaction mixture was filtered through Celite with MeOH, and the filtrate was concentrated *in vacuo*. The pale blue residue was resuspended in 3:1 CH_2Cl_2/MeOH (8 mL), and *p*-chloranil (160 mg, 0.650 mmol, 2 eq) was added. After stirring the mixture at room temperature for 2 h, it was deposited onto Celite and concentrated to dryness. Silica gel chromatography (20–100% EtOAc/toluene, linear gradient; dry load with Celite) yielded 133 mg (78%) of **29** as a blue solid. ^{1}H NMR (CD_3OD, 400 MHz) δ 6.93 (s, 2H), 6.80 (s, 2H), 3.50 – 3.42 (m, 4H), 2.67 (dt, J = 16.0, 6.1 Hz, 2H), 2.57 (dt, J = 16.1, 6.2 Hz, 2H), 1.88 (p, J = 6.2 Hz, 4H), 0.45 (s, 3H), 0.44 (s, 3H); ^{19}F NMR (CD_3OD, 376 MHz) δ -140.13 (ddd, J = 21.6, 13.2, 3.2 Hz, 1F), -141.67 (ddd, J = 22.3, 13.1, 3.7 Hz, 1F), -155.20 – -155.37 (m, 1F), -157.47 – -157.65 (m, 1F); Analytical HPLC: t_R = 12.2 min, >99% purity (10–95% MeCN/H_2O, linear gradient, with constant 0.1% v/v TFA additive; 20 min run; 1 mL/min flow; ESI; positive ion mode; detection at 675 nm); HRMS (ESI) calcd for $C_{28}H_{25}F_4N_2O_2Si$ $[M+H]^+$ 525.1616, found 525.1613.

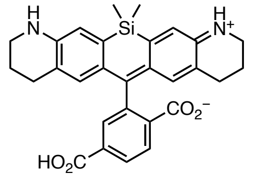

6-Carboxy-Si-Q-rhodamine (S22): *Step 1:* Bis(1-benzyl-1,2,3,4-tetrahydroquinolin-7-yl)dimethylsilane (**27**; 400 mg, 0.796 mmol) and 4-carboxyphthalaldehydic acid (**109**; 232 mg, 1.19 mmol, 1.5 eq) were combined in 2,2,3,3,4,4,4-heptafluoro-1-butanol (4 mL) in a crimp-top vial. The reaction mixture was sparged with O_2 from a balloon for 10 min, then stirred at 95 °C under the O_2 balloon for 24 h. The reaction was cooled to room temperature, concentrated *in vacuo*, and purified by silica gel chromatography (5–100% EtOAc/hexanes, linear gradient, with constant 1% v/v AcOH additive) to provide 102 mg (19%) of 4-carboxy-2-(1,11-dibenzyl-13,13-dimethyl-2,3,4,8,9,10,11,13-octahydrosilino[3,2-*g*:5,6-*g'*]diquinolin-6-ylium-6(1*H*)-yl)benzoate (**S21**) as a green solid.

Step 2: A round-bottom flask was charged with Pd/C (10%, 55.0 mg, 51.7 µmol, 0.2 eq) under nitrogen, and the product from the first step (**S21**; 175 mg, 0.259 mmol) in 1:1 THF/MeOH (20 mL) was added. The sealed flask was evacuated/backfilled with H_2 from a balloon (4×) and then stirred under the H_2 balloon at room temperature for 24 h. The reaction mixture was filtered through Celite with MeOH and EtOAc, and the filtrate was concentrated *in vacuo*. The pale blue residue was resuspended in 3:1 CH_2Cl_2/MeOH (8 mL), and *p*-chloranil (127 mg, 0.517 mmol, 2 eq) was added. After stirring the mixture at room temperature for 1 h, it was deposited onto Celite and concentrated to dryness.

Silica gel chromatography (50–100% EtOAc/hexanes, linear gradient, with constant 1% v/v AcOH additive; dry load with Celite) yielded 98 mg (76%) of **S22** as a blue solid. ^{1}H NMR (CD_3OD with 1% TFA, 400 MHz) δ 8.31 (d, *J* = 8.1 Hz, 1H), 8.28 (dd, *J* = 8.2, 1.6 Hz, 1H), 7.79 (d, *J* = 1.5 Hz, 1H), 6.99 (s, 2H), 6.57 (s, 2H), 3.51 – 3.42 (m, 4H), 2.49 (t, *J* = 6.3 Hz, 4H), 1.86 (p, *J* = 6.2 Hz, 4H), 0.52 (s, 3H), 0.47 (s, 3H); ^{13}C NMR (CD_3OD with 1% TFA, 101 MHz) δ 168.1 (C), 167.7 (C), 154.0 (C), 146.8 (C), 142.7 (C), 140.2 (CH), 136.1 (C), 135.1 (C), 132.5 (CH), 132.3 (CH), 130.9 (CH), 129.1 (C), 124.0 (C), 123.8 (CH), 43.1 (CH_2), 27.6 (CH_2), 21.5 (CH_2), -1.0 (CH_3), -2.1 (CH_3); Analytical HPLC: t_R = 9.9 min, >99% purity (10–95% MeCN/H_2O, linear gradient, with constant 0.1% v/v TFA additive; 20 min run; 1 mL/min flow; ESI; positive ion mode; detection at 650 nm); HRMS (ESI) calcd for $C_{29}H_{29}N_2O_4Si$ $[M+H]^+$ 497.1891, found 497.1887.

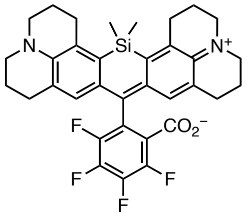

JF$_{698}$ (31): *Via lactol condensation route:* Reaction of **30** and **5** according to the method described for **28** (the typical Si-rhodamine lactol protocol) afforded only a very low yield (2%) of **31** as a dark green solid.

Via dibromide route: A solution of bis(9-bromo-2,3,6,7-tetrahydro-1*H*,5*H*-pyrido[3,2,1-*ij*]quinolin-8-yl)dimethylsilane (**S6**; 400 mg, 0.714 mmol) in THF (15 mL) was cooled to -78 °C under nitrogen. *tert*-Butyllithium (1.7 M in pentane, 1.85 mL, 3.14 mmol, 4.4 eq) was added, and the reaction was stirred at -78 °C for 30 min. It was then warmed to -10 °C before adding a solution of $MgBr_2 \cdot OEt_2$ (405 mg, 1.57 mmol, 2.2 eq) in THF (10 mL). After an additional 30 min at -10 °C, a solution of tetrafluorophthalic anhydride (**4**; 346 mg, 1.57 mmol, 2.2 eq) in THF (10 mL) was added dropwise over 30 min via addition funnel. The reaction was then allowed to warm to room temperature overnight (18 h). It was subsequently diluted with saturated NH_4Cl and water and extracted with EtOAc (2×). The combined organic extracts were washed with saturated $NaHCO_3$ and brine, dried over anhydrous $MgSO_4$, filtered, and concentrated *in vacuo*. Silica gel chromatography (0–15% MeOH (2 M NH_3)/CH_2Cl_2, linear gradient) afforded 192 mg (44%) of **31** as a dark green solid.

^{1}H NMR ($CDCl_3$, 400 MHz) δ 6.42 (s, 2H), 3.22 (t, *J* = 6.0 Hz, 4H), 3.20 – 3.15 (m, 4H), 2.95 – 2.82 (m, 4H), 2.68 – 2.51 (m, 4H), 2.00 (p, *J* = 6.1 Hz, 4H), 1.89 (p, *J* = 6.3 Hz, 4H), 0.67 – 0.63 (m, 3H), 0.59 (s, 3H); ^{19}F NMR ($CDCl_3$, 376 MHz) δ -139.72 (td, *J* = 20.1, 8.2 Hz, 1F), -142.18 (td, *J* = 20.2, 3.3 Hz, 1F), -143.85 (ddd, *J* = 20.4, 18.3, 8.3 Hz, 1F), -153.92 (ddd, *J* = 21.1, 18.3, 3.3 Hz, 1F); Analytical HPLC: t_R = 13.3 min, 98.4% purity (10–95% MeCN/H_2O, linear gradient, with constant 0.1% v/v TFA additive; 20 min run; 1 mL/min flow; ESI; positive ion mode; detection at 700 nm); HRMS (ESI) calcd for $C_{34}H_{33}F_4N_2O_2Si$ $[M+H]^+$ 605.2242, found 605.2249.

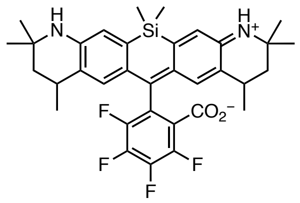

2,3,4,5-Tetrafluoro-6-(2,2,4,8,10,10,13,13-octamethyl-2,3,4,8,9,10,11,13-octahydrosilino[3,2-*g*:5,6-*g'*]diquinolin-6-ylium-6(1*H*)-yl)benzoate (34): *Step 1:* Bis(1-benzyl-2,2,4-trimethyl-1,2,3,4-tetrahydroquinolin-7-yl)dimethylsilane (**32**; 175 mg, 0.298 mmol) and tetrafluorophthalaldehydic acid (**5**; 99.3 mg, 0.447 mmol, 1.5 eq) were combined in 2,2,3,3,4,4,4-heptafluoro-1-butanol (1.5 mL) in a crimp-top vial. The reaction mixture was sparged with O_2 from a balloon for 10 min, then stirred at 95 °C under the O_2 balloon for 24 h. The reaction was cooled to room temperature, concentrated *in vacuo*, and purified by silica gel chromatography (0–30% EtOAc/hexanes, linear gradient) to provide 97.5 mg (41%) of 2-(1,11-dibenzyl-2,2,4,8,10,10,13,13-octamethyl-2,3,4,8,9,10,11,13-octahydrosilino[3,2-*g*:5,6-*g'*]diquinolin-6-ylium-6(1*H*)-yl)-3,4,5,6-tetrafluorobenzoate as an off-white solid (**33**; mixture of diastereomers).

Step 2: A round-bottom flask was charged with Pd/C (10%, 93.6 mg, 88.0 µmol, 0.2 eq) under nitrogen, and a solution of the intermediate from Step 1 (**33**; 347 mg, 0.440 mmol) in THF (10 mL) was added. The sealed flask was evacuated/backfilled with H_2 from a balloon (4×) and then stirred under the H_2 balloon at room temperature for 24 h. A second portion of Pd/C (10%, 93.6 mg, 88.0 µmol, 0.2 eq) was added, and stirring was continued for an additional 24 h (H_2, room temperature). The reaction mixture was filtered through Celite with MeOH and THF, and the filtrate was concentrated *in vacuo*. The pale blue residue was resuspended in 3:1 CH_2Cl_2/MeOH (8 mL), and *p*-chloranil (216 mg, 0.880 mmol, 2 eq) was added. After stirring the mixture at room temperature for 1 h, it was deposited onto Celite and concentrated to dryness. Silica gel chromatography (0–50% EtOAc/toluene, linear gradient; dry load with Celite) yielded 228 mg (85%) of **34** as a blue solid (mixture of diastereomers).

^{1}H NMR (CD_3OD, 400 MHz) δ 7.05 – 7.00 (m, 2H), 6.96 – 6.92 (m, 2H), 2.90 – 2.73 (m, 2H), 1.87 – 1.77 (m, 2H), 1.44 – 1.36 (m, 2H), 1.36 – 1.32 (m, 6H), 1.30 – 1.25 (m, 6H), 1.18 – 1.13 (m, 3H), 1.12 – 1.07 (m, 3H), 0.49 – 0.43 (m, 6H); ^{19}F NMR (CD_3OD, 376 MHz) δ -139.92 – -140.13 (m, 1F), -140.97 – -141.27 (m, 1F), -154.89 – -155.43 (m, 1F), -157.23 – -159.17 (m, 1F); Analytical HPLC: t_R (three isomers) = 14.1 min, 14.4 min, 14.7 min; >99% total purity (10–95% MeCN/H_2O, linear gradient, with constant 0.1% v/v TFA additive; 20 min run; 1 mL/min flow; ESI; positive ion mode; detection at 650 nm); HRMS (ESI) calcd for $C_{34}H_{37}F_4N_2O_2Si$ $[M+H]^+$ 609.2555, found 609.2546.

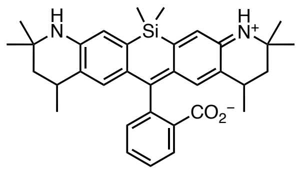

2-(2,2,4,8,10,10,13,13-Octamethyl-2,3,4,8,9,10,11,13-octahydrosilino[3,2-*g*:5,6-*g'*]diquinolin-6-ylium-6(1*H*)-yl)-benzoate (100): *Step 1:* Bis(1-benzyl-2,2,4-trimethyl-1,2,3,4-tetrahydroquinolin-7-yl)dimethylsilane (**32**; 400 mg, 0.682 mmol) and phthalaldehydic acid (**S16**; 128 mg, 0.852 mmol, 1.25 eq) were combined in 2,2,3,3,4,4,4-

heptafluoro-1-butanol (3 mL) in a crimp-top vial. The reaction mixture was sparged with O_2 from a balloon for 10 min, then stirred at 95 °C under the O_2 balloon for 24 h. The reaction was cooled to room temperature, concentrated *in vacuo*, and purified by silica gel chromatography (0–25% EtOAc/hexanes, linear gradient) to provide 190 mg (39%) of 2-(1,11-dibenzyl-2,2,4,8,10,10,13,13-octamethyl-2,3,4,8,9,10,11,13-octahydrosilino[3,2-*g*:5,6-*g′*]diquinolin-6-ylium-6(1*H*)-yl)benzoate as an off-white solid (**S23**; mixture of diastereomers).

Step 2: A round-bottom flask was charged with Pd/C (10%, 56.4 mg, 53.0 μmol, 0.2 eq) under nitrogen, and a solution of the intermediate from Step 1 (**S23**; 190 mg, 0.265 mmol) in THF (10 mL) was added. The sealed flask was evacuated/backfilled with H_2 from a balloon (4×) and then stirred under the H_2 balloon at room temperature for 24 h. A second portion of Pd/C (10%, 56.4 mg, 53.0 μmol, 0.2 eq) was added, and stirring was continued for an additional 24 h (H_2, room temperature). The reaction mixture was filtered through Celite with MeOH, and the filtrate was concentrated *in vacuo*. The residue was resuspended in 3:1 CH_2Cl_2/MeOH (8 mL), and *p*-chloranil (130 mg, 0.530 mmol, 2 eq) was added. After stirring the mixture at room temperature for 2 h, it was deposited onto Celite and concentrated to dryness. Silica gel chromatography (0–40% EtOAc/hexanes, linear gradient; dry load with Celite) yielded 108 mg (76%) of **100** as a blue solid (mixture of diastereomers).

^{1}H NMR (CD_3OD, 400 MHz) δ 8.00 – 7.94 (m, 1H), 7.82 – 7.72 (m, 1H), 7.70 – 7.61 (m, 1H), 7.40 – 7.28 (m, 1H), 6.86 – 6.81 (m, 2H), 6.67 – 6.62 (m, 2H), 2.79 – 2.63 (m, 2H), 1.74 – 1.63 (m, 2H), 1.33 – 1.23 (m, 2H), 1.23 – 1.18 (m, 6H), 1.16 – 1.09 (m, 6H), 1.03 – 0.94 (m, 6H), 0.55 – 0.51 (m, 3H), 0.49 – 0.45 (m, 3H); Analytical HPLC: t_R (three isomers) = 14.2 min, 14.4 min, 14.5 min; >99% total purity (10–95% MeCN/H_2O, linear gradient, with constant 0.1% v/v TFA additive; 20 min run; 1 mL/min flow; ESI; positive ion mode; detection at 650 nm); HRMS (ESI) calcd for $C_{34}H_{41}N_2O_2Si$ $[M+H]^+$ 537.2932, found 537.2927.

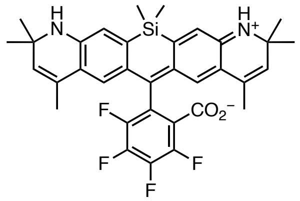

2,3,4,5-Tetrafluoro-6-(2,2,4,8,10,10,13,13-octamethyl-2,10,11,13-tetrahydrosilino[3,2-*g*:5,6-*g′*]diquinolin-6-ylium-6(1*H*)-yl)benzoate (36): Dimethylbis(2,2,4-trimethyl-1,2-dihydroquinolin-7-yl)silane (**35**; 200 mg, 0.497 mmol) and tetrafluorophthalaldehydic acid (**5**; 331 mg, 1.49 mmol, 3 eq) were combined in 2,2,3,3,4,4,4-heptafluoro-1-butanol (2.5 mL) in a crimp-top vial. The reaction mixture was sparged with O_2 from a balloon for 10 min, then stirred at 95 °C under the O_2 balloon for 24 h. The reaction was cooled to room temperature, concentrated *in vacuo*, and purified by silica gel chromatography (0–50% EtOAc/hexanes, linear gradient) to provide **36** as a blue-green solid (44.7 mg, 15%). ^{1}H NMR ($CDCl_3$, 400 MHz) δ 6.64 (s, 2H), 6.53 (s, 2H), 5.29 (q, *J* = 1.5 Hz, 2H), 3.87 (s, 2H), 1.77 (d, *J* = 1.4 Hz, 6H), 1.274 (s, 6H), 1.272 (s, 6H), 0.48 (s, 3H), 0.48 (s, 3H); ^{19}F NMR ($CDCl_3$, 376 MHz) δ -139.01 – -139.26 (m, 2F), -144.21 – -144.35 (m, 1F), -151.82 (t, *J* = 18.0 Hz, 1F); Analytical HPLC: t_R = 11.6 min, >99% purity (30–95% MeCN/H_2O, linear gradient, with constant 0.1% v/v TFA additive; 20 min run; 1 mL/min flow; ESI; positive ion mode; detection at 725 nm); HRMS (ESI) calcd for $C_{34}H_{33}F_4N_2O_2Si$ $[M+H]^+$ 605.2242, found 605.2243.

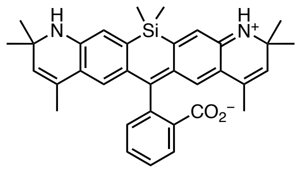

2-(2,2,4,8,10,10,13,13-Octamethyl-2,10,11,13-tetrahydrosilino[3,2-*g*:5,6-*g′*]diquinolin-6-ylium-6(1*H*)-yl)-benzoate (102): Dimethylbis(2,2,4-trimethyl-1,2-dihydroquinolin-7-yl)silane (**35**; 200 mg, 0.497 mmol) and phthalaldehydic acid (**S16**; 224 mg, 1.49 mmol, 3 eq) were combined in 2,2,3,3,4,4,4-heptafluoro-1-butanol (2.5 mL) in a crimp-top vial. The reaction mixture was sparged with O_2 from a balloon for 10 min, then stirred at 95 °C under the O_2 balloon for 24 h. The reaction was cooled to room temperature, concentrated *in vacuo*, and purified by silica gel chromatography (0–50% EtOAc/hexanes, linear gradient) to provide **102** as an off-white solid (18 mg, 7%). ^{1}H NMR ($CDCl_3$, 400 MHz) δ 7.97 (dt, *J* = 7.6, 1.0 Hz, 1H), 7.66 (td, *J* = 7.5, 1.2 Hz, 1H), 7.56 (td, *J* = 7.5, 1.0 Hz, 1H), 7.36 (dt, *J* = 7.7, 1.0 Hz, 1H), 6.66 (s, 2H), 6.53 (s, 2H), 5.23 (q, *J* = 1.1 Hz, 2H), 3.81 (s, 2H), 1.63 (d, *J* = 1.1 Hz, 6H), 1.25 (s, 12H), 0.54 (s, 3H), 0.53 (s, 3H); ^{13}C NMR ($CDCl_3$, 101 MHz) δ 170.8 (C), 154.2 (C), 142.5 (C), 136.8 (C), 133.4 (CH), 133.0 (C), 129.1 (CH), 128.9 (CH), 128.2 (C), 127.4 (C), 125.8 (CH), 124.9 (CH), 122.5 (CH), 121.9 (C), 117.5 (CH), 92.6 (C), 52.1 (C), 31.7 (CH_3), 31.6 (CH_3), 18.1 (CH_3), 0.6 (CH_3), -1.8 (CH_3); Analytical HPLC: t_R = 11.9 min, >99% purity (30–95% MeCN/H_2O, linear gradient, with constant 0.1% v/v TFA additive; 20 min run; 1 mL/min flow; ESI; positive ion mode; detection at 700 nm); HRMS (ESI) calcd for $C_{34}H_{37}N_2O_2Si$ [M+H]$^+$ 533.2619, found 533.2616.

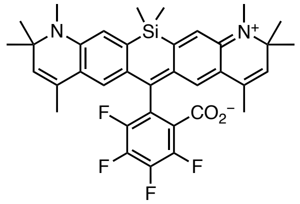

2-(1,2,2,4,8,10,10,11,13,13-Decamethyl-2,10,11,13-tetrahydrosilino[3,2-*g*:5,6-*g′*]diquinolin-6-ylium-6(1*H*)-yl)-3,4,5,6-tetrafluorobenzoate (38): Dimethylbis(1,2,2,4-tetramethyl-1,2-dihydroquinolin-7-yl)silane (**37**; 300 mg, 0.697 mmol) and tetrafluorophthalaldehydic acid (**5**; 309 mg, 1.39 mmol, 2 eq) were combined in 2,2,3,3,4,4,4-heptafluoro-1-butanol (2.3 mL) in a crimp-top vial. The reaction mixture was sparged with O_2 from a balloon for 10 min, then stirred at 95 °C under the O_2 balloon for 24 h. The reaction was cooled to room temperature, diluted with saturated $NaHCO_3$, and extracted with EtOAc (2×). The combined organic extracts were washed with brine, dried over anhydrous $MgSO_4$, filtered, and concentrated *in vacuo*. Silica gel chromatography (0–50% EtOAc/hexanes, linear gradient) provided **38** as a pale yellow-green solid (206 mg, 47%). ^{1}H NMR ($CDCl_3$, 400 MHz) δ 6.66 (s, 2H), 6.53 (s, 2H), 5.28 (q, *J* = 1.4 Hz, 2H), 2.86 (s, 6H), 1.76 (d, *J* = 1.4 Hz, 6H), 1.31 (s, 6H), 1.30 (s, 6H), 0.55 (s, 3H), 0.52 (s, 3H); ^{19}F NMR ($CDCl_3$, 376 MHz) δ -139.29 (td, *J* = 20.0, 8.2 Hz, 1F), -140.03 (td, *J* = 20.0, 3.5 Hz, 1F), -144.18 (ddd, *J* = 20.7, 18.5, 8.3 Hz, 1F), -152.19 (ddd, *J* = 21.1, 18.3, 3.6 Hz, 1F); Analytical HPLC: t_R = 12.6 min, 98.9% purity (30–95% MeCN/H_2O, linear gradient, with constant 0.1% v/v TFA additive; 20 min run; 1 mL/min flow; ESI; positive ion mode; detection at 750 nm); HRMS (ESI) calcd for $C_{36}H_{37}F_4N_2O_2Si$ [M+H]$^+$ 633.2555, found 633.2549.

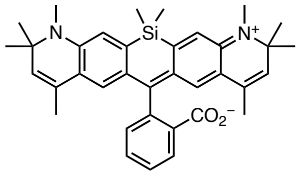

2-(1,2,2,4,8,10,10,11,13,13-Decamethyl-2,10,11,13-tetrahydrosilino[3,2-*g*:5,6-*g*']diquinolin-6-ylium-6(1*H*)-yl)-benzoate (104): Dimethylbis(1,2,2,4-tetramethyl-1,2-dihydroquinolin-7-yl)silane (**37**; 300 mg, 0.697 mmol) and phthalaldehydic acid (**S16**; 157 mg, 1.04 mmol, 1.5 eq) were combined in 2,2,3,3,4,4,4-heptafluoro-1-butanol (2.3 mL) in a crimp-top vial. The reaction mixture was sparged with O_2 from a balloon for 10 min, then stirred at 95 °C under the O_2 balloon for 18 h. The reaction was cooled to room temperature, diluted with saturated $NaHCO_3$, and extracted with EtOAc (2×). The combined organic extracts were washed with brine, dried over anhydrous $MgSO_4$, filtered, and concentrated *in vacuo*. The crude material was purified twice by silica gel chromatography (0–25% EtOAc/toluene, linear gradient; then, 0–50% EtOAc/hexanes, linear gradient) to provide **104** as an off-white solid (41.8 mg, 11%). ^{1}H NMR ($CDCl_3$, 400 MHz) δ 7.96 (dt, *J* = 7.6, 1.0 Hz, 1H), 7.61 (td, *J* = 7.5, 1.2 Hz, 1H), 7.52 (td, *J* = 7.5, 1.0 Hz, 1H), 7.28 (d, *J* = 7.7 Hz, 1H), 6.69 (s, 2H), 6.53 (s, 2H), 5.21 (t, *J* = 1.5 Hz, 2H), 2.85 (s, 6H), 1.63 (d, *J* = 1.4 Hz, 6H), 1.29 (s, 6H), 1.27 (s, 6H), 0.61 (s, 3H), 0.57 (s, 3H); ^{13}C NMR ($CDCl_3$, 101 MHz) δ 171.1 (C), 155.1 (C), 144.2 (C), 136.6 (C), 133.6 (CH), 131.9 (C), 130.8 (CH), 128.7 (CH), 127.8 (C), 127.0 (C), 125.6 (CH), 124.5 (CH), 123.8 (C), 122.2 (CH), 114.4 (CH), 92.3 (C), 56.6 (C), 30.8 (CH_3), 28.2 (CH_3), 27.6 (CH_3), 18.2 (CH_3), 0.5 (CH_3), -1.2 (CH_3); Analytical HPLC: t_R = 12.2 min, >99% purity (30–95% MeCN/H_2O, linear gradient, with constant 0.1% v/v TFA additive; 20 min run; 1 mL/min flow; ESI; positive ion mode; detection at 725 nm); HRMS (ESI) calcd for $C_{36}H_{41}N_2O_2Si$ [M+H]$^+$ 561.2932, found 561.2921.

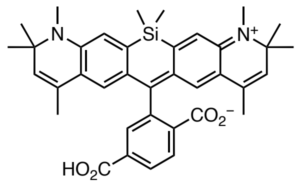

4-Carboxy-2-(1,2,2,4,8,10,10,11,13,13-decamethyl-2,10,11,13-tetrahydrosilino[3,2-*g*:5,6-*g*']diquinolin-6-ylium-6(1*H*)-yl)benzoate (110): Dimethylbis(1,2,2,4-tetramethyl-1,2-dihydroquinolin-7-yl)silane (**37**; 300 mg, 0.697 mmol) and 4-carboxyphthalaldehydic acid (**109**; 203 mg, 1.04 mmol, 1.5 eq) were combined in 2,2,3,3,4,4,4-heptafluoro-1-butanol (3.5 mL) in a crimp-top vial. The reaction mixture was sparged with O_2 from a balloon for 10 min, then stirred at 95 °C under the O_2 balloon for 24 h. The reaction was cooled to room temperature, concentrated *in vacuo*, and purified twice by silica gel chromatography (10–100% EtOAc/hexanes, linear gradient with constant 0.1% v/v AcOH additive) to provide **110** as a green solid (63.4 mg, 15%). An analytically pure sample for spectral characterization was obtained by reverse phase HPLC (30–70% MeCN/H_2O, linear gradient, with constant 0.1% TFA). ^{1}H NMR (CD_3OD, 400 MHz, TFA salt) δ 8.23 (dd, *J* = 8.0, 1.3 Hz, 1H), 8.06 (dd, *J* = 8.0, 0.7 Hz, 1H), 7.87 (dd, *J* = 1.3, 0.7 Hz, 1H), 6.84 (s, 2H), 6.54 (s, 2H), 5.31 (q, *J* = 1.5 Hz, 2H), 2.91 (s, 6H), 1.61 (d, *J* = 1.4 Hz, 6H), 1.29 (s, 6H), 1.27 (s, 6H), 0.65 (s, 3H), 0.56 (s, 3H); ^{13}C NMR (CD_3OD, 101 MHz, TFA salt) δ 171.7 (C), 168.5 (C), 154.9 (C), 146.7 (C), 139.3 (C), 138.0 (C), 132.4 (CH), 131.8 (C), 131.25 (C), 131.21 (CH), 128.2 (C), 127.26 (CH),

127.21 (CH), 125.1 (C), 123.8 (CH), 116.4 (CH), 58.2 (C), 31.4 (CH_3), 27.9 (CH_3), 18.1 (CH_3), 0.0 (CH_3), -1.1 (CH_3); Analytical HPLC: t_R = 12.7 min, >99% purity (10–95% MeCN/H_2O, linear gradient, with constant 0.1% v/v TFA additive; 20 min run; 1 mL/min flow; ESI; positive ion mode; detection at 725 nm); HRMS (ESI) calcd for $C_{37}H_{41}N_2O_4Si$ $[M+H]^+$ 605.2830, found 605.2816.

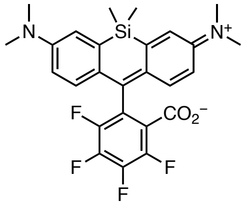

SiRF$_{667}$ (22): 3,3'-(Dimethylsilanediyl)bis(*N*,*N*-dimethylaniline)[1] (**23**; 200 mg, 0.670 mmol) and tetrafluorophthalaldehydic acid (**5**; 446 mg, 2.01 mmol, 3 eq) were combined in 2,2,3,3,4,4,4-heptafluoro-1-butanol (2.2 mL) in a crimp-top vial. The reaction mixture was sparged with O_2 from a balloon for 10 min, then stirred at 95 °C under the O_2 balloon for 72 h. The reaction was cooled to room temperature, diluted with saturated $NaHCO_3$, and extracted with EtOAc (2×). The combined organic extracts were washed with brine, dried over anhydrous $MgSO_4$, filtered, and concentrated *in vacuo*. Silica gel chromatography (0–50% EtOAc/hexanes, linear gradient) provided **22** as a blue-green solid (194 mg, 58%). The characterization data for **22** matched the previously reported spectra.[1]

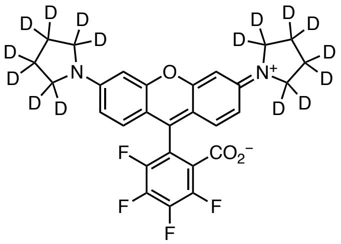

JFX$_{576}$ (8): *Via lactol condensation route:* 1,1'-(Oxybis(3,1-phenylene))bis(pyrrolidine-2,2,3,3,4,4,5,5-*d*$_8$) (**9**; 248 mg, 0.764 mmol) and tetrafluorophthalaldehydic acid (**5**; 170 mg, 0.764 mmol, 1 eq) were combined in 2,2,2-trifluoroethanol (15 mL) in a round-bottom flask. The reaction mixture was sparged with O_2 from a balloon for 10 min, then stirred at 80 °C under the O_2 balloon for 24 h. The reaction was cooled to room temperature, concentrated *in vacuo*, and purified by silica gel chromatography (0–10% MeOH (2 M NH_3)/CH_2Cl_2, linear gradient) to provide **8** as a dark purple solid (343 mg, 85%).

Via dibromide route: A solution of 1,1'-(oxybis(4-bromo-3,1-phenylene))bis(pyrrolidine-2,2,3,3,4,4,5,5-*d*$_8$) (**7**; 300 mg, 0.622 mmol) in THF (15 mL) was cooled to -78 °C under nitrogen. *tert*-Butyllithium (1.7 M in pentane, 1.61 mL, 2.74 mmol, 4.4 eq) was added, and the reaction was stirred at -78 °C for 30 min. It was then warmed to -10 °C before adding a solution of $MgBr_2·OEt_2$ (353 mg, 1.37 mmol, 2.2 eq) in THF (10 mL). After an additional 30 min at -10 °C, a solution of tetrafluorophthalic anhydride (**4**; 301 mg, 1.37 mmol, 2.2 eq) in THF (10 mL) was added dropwise over 30 min via addition funnel. The reaction was then allowed to warm to room temperature overnight (18 h). Following the addition of AcOH (500 μL), the mixture was diluted with MeOH, deposited onto Celite, and

concentrated to dryness. Silica gel chromatography (0–10% MeOH (2 M NH_3)/CH_2Cl_2, linear gradient; dry load with Celite) afforded 113 mg (34%) of **8** as a dark purple solid.

^{1}H NMR (CD_3OD, 400 MHz) δ 7.34 (dd, *J* = 9.3, 0.9 Hz, 1H), 6.93 (dd, *J* = 9.4, 2.3 Hz, 1H), 6.76 (d, *J* = 2.3 Hz, 1H); ^{19}F NMR (CD_3OD, 376 MHz) δ -139.00 (ddd, *J* = 21.1, 12.5, 4.1 Hz, 1F), -140.94 (ddd, *J* = 22.4, 12.8, 3.7 Hz, 1F), -153.55 (ddd, *J* = 22.7, 19.1, 4.1 Hz, 1F), -157.66 – -157.87 (m, 1F); Analytical HPLC: t_R = 12.4 min, >99% purity (10–95% MeCN/H_2O, linear gradient, with constant 0.1% v/v TFA additive; 20 min run; 1 mL/min flow; ESI; positive ion mode; detection at 575 nm); HRMS (ESI) calcd for $C_{28}H_7D_{16}F_4N_2O_3$ $[M+H]^+$ 527.2644, found 527.2640.

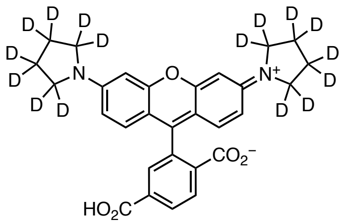

6-Carboxy-JFX$_{554}$ (S24): 1,1'-(Oxybis(3,1-phenylene))bis(pyrrolidine-2,2,3,3,4,4,5,5-*d*$_8$) (**9**; 45 mg, 0.139 mmol) and 4-carboxyphthalaldehydic acid (**109**; 40.4 mg, 0.208 mmol, 1.5 eq) were combined in 2,2,2-trifluoroethanol (3 mL) in a crimp-top vial. The reaction mixture was sparged with O_2 from a balloon for 10 min, then stirred at 80 °C under the O_2 balloon for 24 h. The reaction was cooled to room temperature, concentrated *in vacuo*, and purified by silica gel chromatography (0–20% MeOH/CH_2Cl_2, linear gradient, with constant 1% v/v AcOH additive) to provide **S24** as a dark red solid (61.2 mg, 79%, acetate salt). The characterization data for **S24** matched the previously reported spectra.[5]

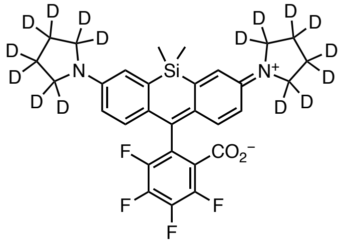

JFX$_{673}$ (25): *Via lactol condensation route:* Dimethylbis(3-(pyrrolidin-1-yl-*d*$_8$)phenyl)silane (**26**; 250 mg, 0.682 mmol) and tetrafluorophthalaldehydic acid (**5**; 151 mg, 0.682 mmol, 1 eq) were combined in 2,2,3,3,4,4,4-heptafluoro-1-butanol (3 mL) in a crimp-top vial. The reaction mixture was sparged with O_2 from a balloon for 10 min, then stirred at 95 °C under the O_2 balloon for 48 h. The reaction was cooled to room temperature, concentrated *in vacuo*, and purified by silica gel chromatography (0–75% EtOAc/hexanes, linear gradient) to provide **25** as a blue solid (247 mg, 64%).

Via dibromide route: A solution of bis(2-bromo-5-(pyrrolidin-1-yl-*d*$_8$)phenyl)dimethylsilane (**24**; 600 mg, 1.14 mmol) in THF (40 mL) was cooled to -78 °C under nitrogen. *tert*-Butyllithium (1.7 M in pentane, 2.96 mL, 5.03 mmol, 4.4 eq) was added, and the reaction was stirred at -78 °C for 30 min. It was then warmed to -10 °C before adding a solution of $MgBr_2 \cdot OEt_2$ (650 mg, 2.52 mmol, 2.2 eq) in THF (10 mL). After an additional 30 min at -10 °C,

a solution of tetrafluorophthalic anhydride (**4**; 554 mg, 2.52 mmol, 2.2 eq) in THF (10 mL) was added dropwise over 30 min via addition funnel. The reaction was then allowed to warm to room temperature overnight (18 h). It was subsequently diluted with saturated NH_4Cl and water and extracted with EtOAc (2×). The combined organic extracts were washed with saturated $NaHCO_3$ and brine, dried over anhydrous $MgSO_4$, filtered, and concentrated *in vacuo*. Silica gel chromatography (0–75% EtOAc/hexanes, linear gradient) afforded 326 mg (50%) of **25** as a blue solid.

^{1}H NMR ($CDCl_3$, 400 MHz) δ 6.78 (d, J = 2.9 Hz, 2H), 6.77 (d, J = 8.6 Hz, 2H), 6.45 (dd, J = 8.8, 2.7 Hz, 2H), 0.58 (s, 3H), 0.55 (s, 3H); ^{19}F NMR ($CDCl_3$, 376 MHz) δ -139.43 – -139.69 (m, 2F), -144.25 – -144.45 (m, 1F), -152.39 – -152.57 (m, 1F); Analytical HPLC: t_R = 13.4 min, 98.8% purity (10–95% $MeCN/H_2O$, linear gradient, with constant 0.1% v/v TFA additive; 20 min run; 1 mL/min flow; ESI; positive ion mode; detection at 675 nm); HRMS (ESI) calcd for $C_{30}H_{13}D_{16}F_4N_2O_2Si$ $[M+H]^+$ 569.2933, found 569.2943.

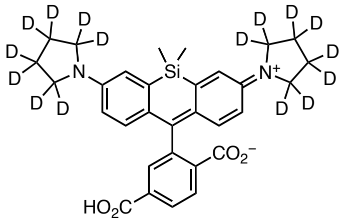

6-Carboxy-JFX$_{650}$ (S25): Dimethylbis(3-(pyrrolidin-1-yl-d_8)phenyl)silane (**26**; 250 mg, 0.682 mmol) and 4-carboxyphthalaldehydic acid (**109**; 132 mg, 0.682 mmol, 1 eq) were combined in 2,2,3,3,4,4,4-heptafluoro-1-butanol (2.5 mL) in a crimp-top vial. The reaction mixture was sparged with O_2 from a balloon for 10 min, then stirred at 95 °C under the O_2 balloon for 72 h. The reaction was cooled to room temperature, concentrated *in vacuo*, and purified by silica gel chromatography (10–100% EtOAc/hexanes, linear gradient, with constant 0.1% v/v AcOH additive) to provide **S25** as a blue solid (139 mg, 38%). The characterization data for **S25** matched the previously reported spectra.[5]

Preparation of 2,2-Diarylpropane Intermediates (Scheme S4)

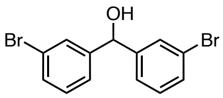

Bis(3-bromophenyl)methanol (S29): A solution of 1,3-dibromobenzene (**S27**; 14.02 g, 59.45 mmol, 1.1 eq) in THF (100 mL) was cooled to -78 °C under nitrogen. *n*-Butyllithium (2.5 M in hexanes, 23.78 mL, 59.45 mmol, 1.1 eq) was added, and the reaction was stirred at -78 °C for 30 min. 3-Bromobenzaldehye (**S28**; 10.00 g, 54.05 mmol) was then added. The dry ice bath was removed, and the reaction was stirred at room temperature for 2 h. It was subsequently quenched with saturated NH_4Cl, diluted with water, and extracted with EtOAc (2×). The combined organic extracts were washed with brine, dried over anhydrous $MgSO_4$, filtered, and concentrated *in vacuo*. Purification by flash chromatography on silica gel (0–50% Et_2O/hexanes, linear gradient) afforded 16.15 g (92%) of **S29** as a colorless oil. ^{1}H NMR ($CDCl_3$, 400 MHz) δ 7.54 (t, J = 1.9 Hz, 2H), 7.42 (ddd, J = 7.8, 2.0, 1.2 Hz, 2H), 7.30 – 7.26 (m, 2H), 7.22 (t, J = 7.7 Hz, 2H), 5.76 (s, 1H), 2.25 (s, 1H); ^{13}C NMR ($CDCl_3$, 101 MHz) δ 145.4 (C), 131.1 (CH), 130.4 (CH), 129.6 (CH), 125.3 (CH), 122.9 (C), 75.1 (CH); HRMS (EI) calcd for $C_{13}H_{10}Br_2O$ $[M]^{+\bullet}$ 339.9098, found 339.3088.

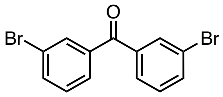

Bis(3-bromophenyl)methanone (S30): To a solution of bis(3-bromophenyl)methanol (**S29**; 11.80 g, 34.50 mmol) in CH_2Cl_2 (100 mL) was added MnO_2 (50.00 g) in two portions. After stirring the reaction at room temperature for 18 h, it was filtered through Celite with CH_2Cl_2 and concentrated *in vacuo*. The resulting white solid was recrystallized from hot EtOAc to yield 11.20 g (95%) of **S30** as white crystals. ^{1}H NMR ($CDCl_3$, 400 MHz) δ 7.93 (t, J = 1.8 Hz, 2H), 7.74 (ddd, J = 8.0, 2.1, 1.1 Hz, 2H), 7.71 – 7.67 (m, 2H), 7.38 (t, J = 7.9 Hz, 2H); ^{13}C NMR ($CDCl_3$, 101 MHz) δ 193.8 (C), 138.9 (C), 135.9 (CH), 132.9 (CH), 130.2 (CH), 128.7 (CH), 123.0 (C); HRMS (EI) calcd for $C_{13}H_8Br_2O$ $[M]^{+\bullet}$ 337.8942, found 337.8947.

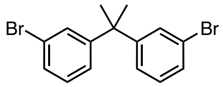

3,3'-(Propane-2,2-diyl)bis(bromobenzene) (S31): An oven-dried 500 mL 3-neck round-bottom flask equipped with two addition funnels was charged with CH_2Cl_2 (100 mL) under nitrogen and cooled to -40 °C. Solutions of $TiCl_4$ (1 M in CH_2Cl_2, 80.00 mL, 80.00 mmol, 4 eq) followed by Me_2Zn (2 M in toluene, 40.00 mL, 80.00 mmol, 4 eq) were successively added dropwise via the addition funnels. The reaction was stirred at -40 °C to -30 °C for 20 min. A solution of bis(3-bromophenyl)methanone (**S30**; 6.80 g, 20.00 mmol) in CH_2Cl_2 (100 mL) was added via cannula; the resulting suspension was then warmed to room temperature and vigorously stirred for 3 h. The mixture was carefully diluted with water (~200 mL) and extracted with CH_2Cl_2 (2×). The combined organic extracts were dried over anhydrous $MgSO_4$, filtered through Celite, and concentrated *in vacuo*. Silica gel chromatography (100% hexanes, isocratic) afforded 6.83 g (96%) of **S31** as a colorless oil. ^{1}H NMR ($CDCl_3$, 400 MHz) δ 7.37 (t, J = 1.8 Hz, 2H), 7.33 (ddd, J = 7.6, 2.0, 1.3 Hz, 2H), 7.14 (t, J = 7.8 Hz, 2H), 7.11 – 7.08 (m, 2H), 1.64 (s, 6H); ^{13}C NMR ($CDCl_3$, 101

MHz) δ 152.4 (C), 129.90 (CH), 129.86 (CH), 129.2 (CH), 125.8 (CH), 122.6 (C), 43.3 (C), 30.6 (CH_3); HRMS (EI) calcd for $C_{15}H_{14}Br_2$ $[M]^{+•}$ 351.9462, found 351.9480.

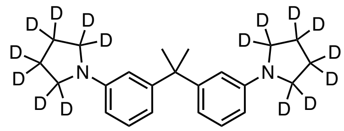

1,1'-(Propane-2,2-diylbis(3,1-phenylene))bis(pyrrolidine-2,2,3,3,4,4,5,5-*d*$_8$) (41): An oven-dried crimp-top vial was charged with CuI (108 mg, 0.565 mmol, 0.2 eq), L-proline (130 mg, 1.13 mmol, 0.4 eq), and K_2CO_3 (1.56 g, 11.30 mmol, 4 eq). The flask was sealed and evacuated/backfilled with nitrogen (3×). A solution of 3,3'-(propane-2,2-diyl)bis(bromobenzene) (**S31**; 1.00 g, 2.82 mmol) in DMSO (11 mL) was added, and the reaction was flushed again with nitrogen (3×). Following the addition of pyrrolidine-2,2,3,3,4,4,5,5-*d*$_8$ (1.42 mL, 16.94 mmol, 6 eq), the reaction was stirred at 100 °C for 18 h. It was then cooled to room temperature, diluted with saturated NH_4Cl, and extracted with EtOAc (2×). The combined organic extracts were washed with water and brine, dried over anhydrous $MgSO_4$, filtered, and concentrated *in vacuo*. Purification by flash chromatography on silica gel (0–20% Et_2O/hexanes, linear gradient) afforded 809 mg (82%) of **41** as a white solid. ^1H NMR ($CDCl_3$, 400 MHz) δ 7.10 (t, *J* = 7.9 Hz, 2H), 6.58 – 6.52 (m, 2H), 6.51 (t, *J* = 2.1 Hz, 2H), 6.42 – 6.35 (m, 2H), 1.67 (s, 6H); ^{13}C NMR ($CDCl_3$, 101 MHz) δ 152.0 (C), 147.9 (C), 128.7 (CH), 114.9 (CH), 110.4 (CH), 109.0 (CH), 43.3 (C), 31.0 (CH_3); HRMS (ESI) calcd for $C_{23}H_{15}D_{16}N_2$ $[M+H]^+$ 351.3486, found 351.3478.

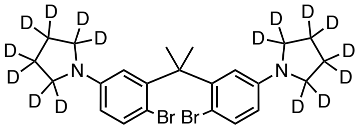

1,1'-(Propane-2,2-diylbis(4-bromo-3,1-phenylene))bis(pyrrolidine-2,2,3,3,4,4,5,5-*d*$_8$) (39): 1,1'-(Propane-2,2-diylbis(3,1-phenylene))bis(pyrrolidine-2,2,3,3,4,4,5,5-*d*$_8$) (**41**; 1.19 g, 3.39 mmol) was taken up in DMF (30 mL) and cooled to 0 °C. *N*-Bromosuccinimide (1.21 g, 6.79 mmol, 2 eq) was added portion-wise over 5 min, and the reaction was subsequently stirred at 0 °C for 2 h. After removing DMF by rotary evaporation, the resulting residue was diluted with water and extracted with CH_2Cl_2 (2×). The combined organic extracts were washed with water and brine, dried over anhydrous $MgSO_4$, filtered, and concentrated *in vacuo*. Purification by silica gel chromatography (0–100% CH_2Cl_2/hexanes, linear gradient) afforded 716 mg (41%) of **39** as a white solid. ^1H NMR ($CDCl_3$, 400 MHz) δ 7.24 (d, *J* = 8.7 Hz, 2H), 6.82 (d, *J* = 2.9 Hz, 2H), 6.29 (dd, *J* = 8.6, 2.9 Hz, 2H), 1.81 (s, 6H); ^{13}C NMR ($CDCl_3$, 101 MHz) δ 147.41 (C), 147.36 (C), 135.1 (CH), 112.5 (CH), 111.3 (CH), 108.1 (C), 46.2 (C), 29.5 (CH_3); HRMS (ESI) calcd for $C_{23}H_{13}D_{16}Br_2N_2$ $[M+H]^+$ 509.1676, found 509.1668.

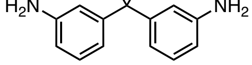

3,3'-(Propane-2,2-diyl)dianiline (S33): *Step 1:* An oven-dried round-bottom flask was charged with *tert*-butyl carbamate (4.76 g, 40.67 mmol, 2.4 eq), CuI (3.23 g, 16.94 mmol, 1 eq), and K_3PO_4 (10.79 g, 50.83 mmol, 3 eq). The

flask was sealed and evacuated/backfilled with nitrogen (3×). A solution of 3,3'-(propane-2,2-diyl)bis(bromobenzene) (**S31**; 6.00 g, 16.94 mmol) in dioxane (60 mL) was added via cannula, and the reaction was flushed again with nitrogen (3×). Following the addition of *N*,*N'*-dimethylethylenediamine (1.82 mL, 16.94 mmol, 1 eq), the reaction was stirred at 110 °C for 18 h. It was then cooled to room temperature, diluted with EtOAc (~250 mL) and saturated NH_4Cl (~250 mL), and vigorously stirred for 15 min while open to air. The resulting suspension was filtered; the filter cake was washed (water, EtOAc) and dried to provide a large crop of white powder that was identified as the desired dicarbamate product by NMR and LC/MS analysis. The filtrate was partitioned, and the aqueous layer was extracted again with EtOAc (3×). The combined organic extracts were washed with brine, dried over anhydrous $MgSO_4$, filtered, and evaporated. The white residue was triturated with Et_2O, filtered, washed with additional Et_2O, and dried to afford a second, smaller crop of the product. The two batches were combined to yield a total of 6.39 g (88%) of di-*tert*-butyl (propane-2,2-diylbis(3,1-phenylene))dicarbamate (**S32**) as a white solid.

Step 2: The dicarbamate (**S32**; 6.39 g, 14.98 mmol) was suspended in CH_2Cl_2 (60 mL), and trifluoroacetic acid (12 mL) was added. The reaction was stirred at room temperature for 4 h. It was then diluted with toluene (60 mL) and concentrated to dryness. The residue was taken up in saturated $NaHCO_3$ and extracted with EtOAc (2×). The combined organic extracts were washed with brine, dried over anhydrous $MgSO_4$, filtered, and evaporated. Flash chromatography (10–100% EtOAc/hexanes, linear gradient) afforded 2.34 g (69%) of **S33** as an off-white solid. ^1H NMR ($CDCl_3$, 400 MHz) δ 7.06 (t, J = 7.8 Hz, 2H), 6.68 (ddd, J = 7.8, 1.8, 1.0 Hz, 2H), 6.54 (t, J = 2.0 Hz, 2H), 6.51 (ddd, J = 7.8, 2.3, 1.0 Hz, 2H), 3.56 (s, 4H), 1.60 (s, 6H); ^{13}C NMR ($CDCl_3$, 101 MHz) δ 152.2 (C), 146.1 (C), 128.9 (CH), 117.4 (CH), 114.3 (CH), 112.7 (CH), 42.9 (C), 30.7 (CH_3); HRMS (ESI) calcd for $C_{15}H_{19}N_2$ $[M+H]^+$ 227.1543, found 227.1544.

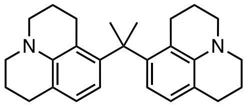

8,8'-(Propane-2,2-diyl)bis(2,3,6,7-tetrahydro-1*H*,5*H*-pyrido[3,2,1-*ij*]quinoline) (56): A mixture of 3,3'-(propane-2,2-diyl)dianiline (**S33**; 2.30 g, 10.16 mmol), Na_2CO_3 (8.62 g, 81.30 mmol, 8 eq), and 1-bromo-3-chloropropane (24.15 mL, 243.9 mmol, 24 eq) was stirred at 140 °C for 48 h. The gummy, heterogeneous mixture that resulted was cooled to room temperature, diluted with water, and extracted with EtOAc (2×). The organic extracts were washed with brine, dried over anhydrous $MgSO_4$, filtered, and concentrated *in vacuo*. The resulting red residue was taken back up in DMF (15 mL) and stirred at 160 °C for 18 h. The reaction was cooled to room temperature, diluted with saturated $NaHCO_3$, and extracted with CH_2Cl_2 (2×). The CH_2Cl_2 extracts were dried over anhydrous $MgSO_4$, filtered, and evaporated. Silica gel chromatography (0–20% Et_2O/hexanes, linear gradient) yielded 2.04 g (52%) of **56** as a white solid. ^1H NMR ($CDCl_3$, 400 MHz) δ 6.76 (AB quartet, ν_A = 2710.7 Hz, ν_B = 2693.5 Hz, J_{AB} = 8.0 Hz, 4H), 3.09 (t, J = 5.7 Hz, 4H), 2.97 (t, J = 6.3 Hz, 4H), 2.73 (t, J = 6.5 Hz, 4H), 2.42 – 1.82 (m, 8H), 1.65 – 1.54 (m, 10H) ^{13}C NMR ($CDCl_3$, 101 MHz) δ 146.0 (C), 143.5 (C), 126.7 (CH), 121.7 (C), 119.7 (C), 113.1 (CH), 51.0 (CH_2), 50.2 (CH_2), 43.6 (C), 31.4 (CH_3), 28.2 (CH_2), 25.3 (CH_2), 22.4 (CH_2); HRMS (ESI) calcd for $C_{27}H_{35}N_2$ $[M+H]^+$ 387.2795, found 387.2796.

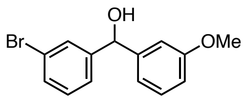

(3-Bromophenyl)(3-methoxyphenyl)methanol (S35): A solution of 1,3-dibromobenzene (**S27**; 25.00 g, 106.0 mmol, 1.2 eq) in THF (200 mL) was cooled to -78 °C under nitrogen. *n*-Butyllithium (2.7 M in hexanes, 39.25 mL, 106.0 mmol, 1.2 eq) was added, and the reaction was stirred at -78 °C for 30 min. *m*-Anisaldehyde (**S34**; 10.76 mL, 88.31 mmol) was then added. The dry ice bath was removed, and the reaction was stirred at room temperature for 2 h. It was subsequently quenched with saturated NH_4Cl, diluted with water, and extracted with EtOAc (2×). The combined organic extracts were washed with brine, dried over anhydrous $MgSO_4$, filtered, and concentrated *in vacuo*. Purification by flash chromatography on silica gel (0–50% Et_2O/hexanes, linear gradient) afforded 22.23 g (86%) of **S35** as a pale yellow oil. ^{1}H NMR ($CDCl_3$, 400 MHz) δ 7.56 (t, *J* = 1.9 Hz, 1H), 7.39 (ddd, *J* = 7.9, 2.1, 1.2 Hz, 1H), 7.31 – 7.27 (m, 1H), 7.26 (t, *J* = 8.2 Hz, 1H), 7.19 (t, *J* = 7.8 Hz, 1H), 6.95 – 6.90 (m, 2H), 6.82 (ddd, *J* = 8.4, 2.5, 1.2 Hz, 1H), 5.77 (d, *J* = 3.4 Hz, 1H), 3.79 (s, 3H), 2.22 (d, *J* = 3.4 Hz, 1H); ^{13}C NMR ($CDCl_3$, 101 MHz) δ 160.0 (C), 146.0 (C), 145.0 (C), 130.8 (CH), 130.2 (CH), 129.9 (CH), 129.6 (CH), 125.2 (CH), 122.8 (C), 119.0 (CH), 113.4 (CH), 112.4 (CH), 75.7 (CH), 55.4 (CH_3); HRMS (EI) calcd for $C_{14}H_{13}BrO_2$ $[M]^{+\bullet}$ 292.0094, found 292.0088.

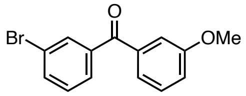

(3-Bromophenyl)(3-methoxyphenyl)methanone (S36): To a solution of (3-bromophenyl)(3-methoxyphenyl)-methanol (**S35**; 21.75 g, 74.19 mmol) in CH_2Cl_2 (400 mL) was added MnO_2 (100 g, portion-wise over 10 min). After stirring the reaction at room temperature for 18 h, it was filtered through Celite with CH_2Cl_2 and concentrated *in vacuo*. Silica gel chromatography (0–40% Et_2O/hexanes, linear gradient) yielded 20.34 g (94%) of **S36** as a viscous, colorless oil that crystallized into a white solid upon standing. ^{1}H NMR ($CDCl_3$, 400 MHz) δ 7.94 (t, *J* = 1.8 Hz, 1H), 7.74 – 7.69 (m, 2H), 7.40 (t, *J* = 7.2 Hz, 1H), 7.38 – 7.33 (m, 2H), 7.31 (dt, *J* = 7.6, 1.3 Hz, 1H), 7.16 (ddd, *J* = 8.2, 2.7, 1.1 Hz, 1H), 3.87 (s, 3H); ^{13}C NMR ($CDCl_3$, 101 MHz) δ 195.1 (C), 159.9 (C), 139.7 (C), 138.4 (C), 135.4 (CH), 132.9 (CH), 130.0 (CH), 129.5 (CH), 128.7 (CH), 123.0 (CH), 122.7 (C), 119.5 (CH), 114.4 (CH), 55.6 (CH_3); HRMS (ESI) calcd for $C_{14}H_{12}BrO_2$ $[M+H]^+$ 291.0015, found 291.0021.

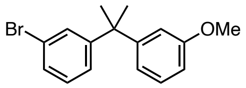

1-Bromo-3-(2-(3-methoxyphenyl)propan-2-yl)benzene (S37): An oven-dried 1 L 3-neck round-bottom flask equipped with two addition funnels was charged with CH_2Cl_2 (150 mL) under nitrogen and cooled to -30 °C. Solutions of $TiCl_4$ (1 M in CH_2Cl_2, 100.0 mL, 100.0 mmol, 4 eq) followed by Me_2Zn (1.2 M in toluene, 83.33 mL, 100.0 mmol, 4 eq) were successively added dropwise via the addition funnels. The reaction was stirred at -30 °C for 20 min. A solution of (3-bromophenyl)(3-methoxyphenyl)methanone (**S36**; 7.28 g, 25.00 mmol) in CH_2Cl_2 (50 mL) was added dropwise via cannula; the resulting brown suspension was then warmed to room temperature and vigorously stirred for 3 h. The mixture was carefully diluted with water (~400 mL) and extracted with CH_2Cl_2 (2×). The combined organic extracts were dried over anhydrous $MgSO_4$, filtered through Celite, and concentrated *in vacuo*. Silica gel

chromatography (0–10% Et_2O/hexanes, linear gradient) afforded 7.23 g (95%) of **S37** as a viscous, colorless oil. ^{1}H NMR ($CDCl_3$, 400 MHz) δ 7.41 – 7.37 (m, 1H), 7.33 – 7.27 (m, 1H), 7.23 – 7.17 (m, 1H), 7.14 – 7.09 (m, 2H), 6.81 – 6.76 (m, 2H), 6.75 – 6.71 (m, 1H), 3.77 (s, 3H), 1.65 (s, 6H); ^{13}C NMR ($CDCl_3$, 101 MHz) δ 159.5 (C), 153.1 (C), 151.7 (C), 129.9 (CH), 129.7 (CH), 129.2 (CH), 129.0 (CH), 125.8 (CH), 122.5 (C), 119.5 (CH), 113.6 (CH), 110.5 (CH), 55.3 (CH_3), 43.2 (C), 30.7 (CH_3); HRMS (EI) calcd for $C_{16}H_{17}BrO$ $[M]^{+\bullet}$ 304.0458, found 304.0469.

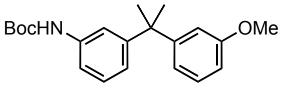

***tert*-Butyl (3-(2-(3-methoxyphenyl)propan-2-yl)phenyl)carbamate (S38):** An oven-dried round-bottom flask was charged with *tert*-butyl carbamate (4.61 g, 39.32 mmol, 1.2 eq), CuI (3.12 g, 16.38 mmol, 0.5 eq), and K_3PO_4 (10.43 g, 49.14 mmol, 1.5 eq). The flask was sealed and evacuated/backfilled with nitrogen (3×). A solution of 1-bromo-3-(2-(3-methoxyphenyl)propan-2-yl)benzene (**S37**; 10.00 g, 32.76 mmol) in dioxane (100 mL) was added via cannula, and the reaction was flushed again with nitrogen (3×). Following the addition of *N*,*N′*-dimethylethylenediamine (1.76 mL, 16.38 mmol, 0.5 eq), the reaction was stirred at 110 °C for 18 h. It was then cooled to room temperature, stirred open to air for 3 h, filtered through Celite with EtOAc, and concentrated *in vacuo*. Purification of the crude residue by flash chromatography (0–25% EtOAc/hexanes, linear gradient) yielded 10.42 g (93%) of **S38** as a viscous, colorless gum that crystallized into a white solid upon standing. ^{1}H NMR ($CDCl_3$, 400 MHz) δ 7.33 (d, J = 8.0 Hz, 1H), 7.18 (td, J = 8.1, 2.0 Hz, 2H), 7.05 (t, J = 2.0 Hz, 1H), 6.90 (ddd, J = 7.8, 1.9, 1.0 Hz, 1H), 6.83 – 6.77 (m, 2H), 6.71 (ddd, J = 8.1, 2.4, 1.1 Hz, 1H), 6.40 (s, 1H), 3.75 (s, 3H), 1.64 (s, 6H), 1.49 (s, 9H); ^{13}C NMR ($CDCl_3$, 101 MHz) δ 159.4 (C), 152.9 (C), 152.4 (C), 151.6 (C), 138.2 (C), 129.0 (CH), 128.8 (CH), 121.8 (CH), 119.6 (CH), 117.3 (CH), 116.2 (CH), 113.5 (CH), 110.4 (CH), 80.5 (C), 55.3 (CH_3), 43.1 (C), 30.8 (CH_3), 28.5 (CH_3); HRMS (ESI) calcd for $C_{21}H_{27}NO_3Na$ $[M+Na]^+$ 364.1883, found 364.1884.

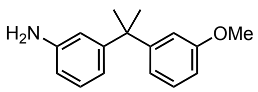

3-(2-(3-Methoxyphenyl)propan-2-yl)aniline (S39): *tert*-Butyl (3-(2-(3-methoxyphenyl)propan-2-yl)phenyl)-carbamate (**S38**; 10.30 g, 30.17 mmol) was taken up in CH_2Cl_2 (200 mL), and trifluoroacetic acid (40 mL) was added. The reaction was stirred at room temperature for 2 h. It was then diluted with toluene (100 mL) and concentrated to dryness. The residue was taken up in saturated $NaHCO_3$ and extracted with EtOAc (2×). The combined organic extracts were washed with brine, dried over anhydrous $MgSO_4$, filtered, and concentrated *in vacuo*. Flash chromatography (0–40% EtOAc/hexanes, linear gradient) afforded 6.72 g (92%) of **S39** as an off-white solid. ^{1}H NMR ($CDCl_3$, 400 MHz) δ 7.18 (t, J = 8.2 Hz, 1H), 7.05 (t, J = 7.8 Hz, 1H), 6.84 – 6.80 (m, 2H), 6.71 (ddd, J = 8.2, 2.5, 1.0 Hz, 1H), 6.66 (ddd, J = 7.8, 1.9, 1.0 Hz, 1H), 6.53 (t, J = 2.1 Hz, 1H), 6.50 (ddd, J = 7.8, 2.4, 1.0 Hz, 1H), 3.76 (s, 3H), 3.45 (s, 2H), 1.63 (s, 6H); ^{13}C NMR ($CDCl_3$, 101 MHz) δ 159.4 (C), 152.7 (C), 151.9 (C), 146.1 (C), 128.97 (CH), 128.96 (CH), 119.7 (CH), 117.4 (CH), 114.2 (CH), 113.5 (CH), 112.8 (CH), 110.3 (CH), 55.3 (CH_3), 43.0 (C), 30.7 (CH_3); HRMS (ESI) calcd for $C_{16}H_{20}NO$ $[M+H]^+$ 242.1539, found 242.1541.

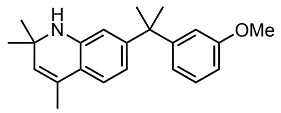

7-(2-(3-Methoxyphenyl)propan-2-yl)-2,2,4-trimethyl-1,2-dihydroquinoline (S40): 3-(2-(3-Methoxyphenyl)-propan-2-yl)aniline (**S39**; 2.20 g, 9.12 mmol) and iodine (463 mg, 1.82 mmol, 0.2 eq) were combined in acetone (50 mL) and stirred at reflux for 18 h. The reaction was then cooled to room temperature, concentrated to dryness, and purified by silica gel chromatography (0–20% Et_2O/hexanes, linear gradient) to afford 1.91 g (65%) of **S40** as a colorless oil that crystallized into an off-white solid upon standing. ^{1}H NMR ($CDCl_3$, 400 MHz) δ 7.17 (t, *J* = 8.2 Hz, 1H), 6.95 (d, *J* = 8.0 Hz, 1H), 6.85 – 6.81 (m, 2H), 6.70 (ddd, *J* = 8.1, 2.5, 1.0 Hz, 1H), 6.52 (dd, *J* = 8.0, 1.9 Hz, 1H), 6.27 (d, *J* = 1.9 Hz, 1H), 5.24 (q, *J* = 1.5 Hz, 1H), 3.76 (s, 3H), 3.60 (s, 1H), 1.95 (d, *J* = 1.4 Hz, 3H), 1.61 (s, 6H), 1.24 (s, 6H); ^{13}C NMR ($CDCl_3$, 101 MHz) δ 159.4 (C), 152.7 (C), 151.1 (C), 142.9 (C), 128.9 (CH), 128.5 (C), 127.8 (CH), 123.3 (CH), 119.7 (CH), 119.3 (C), 115.9 (CH), 113.5 (CH), 111.8 (CH), 110.3 (CH), 55.3 (CH_3), 52.0 (C), 42.9 (C), 31.3 (CH_3), 30.7 (CH_3), 18.6 (CH_3); HRMS (ESI) calcd for $C_{22}H_{28}NO$ $[M+H]^+$ 322.2165, found 322.2168.

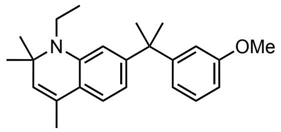

1-Ethyl-7-(2-(3-methoxyphenyl)propan-2-yl)-2,2,4-trimethyl-1,2-dihydroquinoline (S41): 7-(2-(3-Methoxy-phenyl)propan-2-yl)-2,2,4-trimethyl-1,2-dihydroquinoline (**S40**; 1.50 g, 4.67 mmol), K_2CO_3 (3.87 g, 28.00 mmol, 6 eq), and iodoethane (4.50 mL, 55.99 mmol, 12 eq) were combined in DMF (24 mL) in a heavy-wall pressure flask. The vessel was sealed (PTFE bushing, Viton O-ring) and stirred at 120 °C for 4 h. After cooling to room temperature, the mixture was diluted with water and extracted with EtOAc (2×). The organic extracts were washed with water and brine, dried over anhydrous $MgSO_4$, filtered, and concentrated *in vacuo*. Purification by flash chromatography on silica gel (0–10% Et_2O/hexanes, linear gradient) afforded 1.57 g (96%) of **S41** as a colorless gum. ^{1}H NMR ($CDCl_3$, 400 MHz) δ 7.20 – 7.14 (m, 1H), 6.94 (d, *J* = 7.9 Hz, 1H), 6.87 (d, *J* = 1.7 Hz, 1H), 6.87 – 6.84 (m, 1H), 6.74 – 6.66 (m, 1H), 6.48 (dd, *J* = 7.9, 1.8 Hz, 1H), 6.26 (d, *J* = 1.8 Hz, 1H), 5.13 (q, *J* = 1.5 Hz, 1H), 3.76 (s, 3H), 3.19 (q, *J* = 7.0 Hz, 2H), 1.93 (d, *J* = 1.4 Hz, 3H), 1.65 (s, 6H), 1.28 (s, 6H), 1.02 (t, *J* = 7.0 Hz, 3H); ^{13}C NMR ($CDCl_3$, 101 MHz) δ 159.4 (C), 152.9 (C), 151.1 (C), 143.1 (C), 128.9 (CH), 128.7 (CH), 127.6 (C), 123.3 (CH), 120.3 (C), 119.8 (CH), 113.6 (CH), 113.5 (CH), 110.2 (CH), 109.7 (CH), 57.0 (C), 55.3 (CH_3), 43.2 (C), 38.1 (CH_2), 30.7 (CH_3), 28.9 (CH_3), 18.8 (CH_3), 14.2 (CH_3); HRMS (ESI) calcd for $C_{24}H_{32}NO$ $[M+H]^+$ 350.2478, found 350.2481.

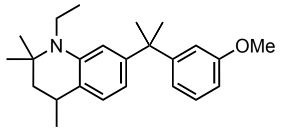

1-Ethyl-7-(2-(3-methoxyphenyl)propan-2-yl)-2,2,4-trimethyl-1,2,3,4-tetrahydroquinoline (S42): A round-bottom flask was charged with Pd/C (10%, 1.52 g, 1.43 mmol, 0.2 eq) under nitrogen, and 1-ethyl-7-(2-(3-methoxyphenyl)propan-2-yl)-2,2,4-trimethyl-1,2-dihydroquinoline (**S41**; 2.50 g, 7.15 mmol) in THF (70 mL) was added. The sealed flask was evacuated/backfilled with H_2 from a balloon (4×) and then stirred under the H_2 balloon at

room temperature for 4 h. The reaction mixture was filtered through Celite with EtOAc and concentrated *in vacuo*. Silica gel chromatography (0–10% Et_2O/hexanes, linear gradient) yielded 2.46 g (98%) of **S42** as a colorless oil. ^1H NMR ($CDCl_3$, 400 MHz) δ 7.18 (t, *J* = 8.2 Hz, 1H), 7.01 (dd, *J* = 7.9, 1.2 Hz, 1H), 6.90 – 6.85 (m, 2H), 6.70 (ddd, *J* = 8.1, 2.4, 1.0 Hz, 1H), 6.49 (dd, *J* = 7.9, 1.8 Hz, 1H), 6.35 (d, *J* = 1.9 Hz, 1H), 3.77 (s, 3H), 3.33 (dq, *J* = 14.2, 7.0 Hz, 1H), 3.04 (dq, *J* = 13.9, 6.9 Hz, 1H), 2.90 – 2.78 (m, 1H), 1.67 (dd, *J* = 12.9, 4.8 Hz, 1H), 1.66 (s, 6H), 1.54 (t, *J* = 12.8 Hz, 1H), 1.29 (d, *J* = 6.5 Hz, 3H), 1.28 (s, 3H), 1.14 (s, 3H), 1.02 (t, *J* = 6.9 Hz, 3H); ^{13}C NMR ($CDCl_3$, 101 MHz) δ 159.4 (C), 153.0 (C), 149.0 (C), 144.2 (C), 128.8 (CH), 125.3 (CH), 125.0 (C), 119.9 (CH), 113.6 (CH), 113.5 (CH), 110.4 (CH), 110.1 (CH), 55.3 (CH_3), 54.4 (C), 47.2 (CH_2), 43.0 (C), 39.1 (CH_2), 30.9 (CH_3), 30.8 (CH_3), 29.9 (CH_3), 27.1 (CH), 25.4 (CH_3), 20.0 (CH_3), 15.0 (CH_3); HRMS (ESI) calcd for $C_{24}H_{34}NO$ $[M+H]^+$ 352.2635, found 352.2636.

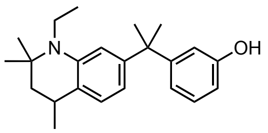

3-(2-(1-Ethyl-2,2,4-trimethyl-1,2,3,4-tetrahydroquinolin-7-yl)propan-2-yl)phenol (S43): 1-Ethyl-7-(2-(3-methoxyphenyl)propan-2-yl)-2,2,4-trimethyl-1,2,3,4-tetrahydroquinoline (**S42**; 2.00 g, 5.69 mmol) was dissolved in glacial AcOH (12 mL); 48% aqueous HBr (12 mL) was added, and the mixture was stirred at reflux for 4 h. After cooling to room temperature, the solution was carefully diluted into water (100 mL), adjusted to pH 5–6 with 25% w/w NaOH, and extracted with EtOAc (2×). The combined organic extracts were washed with saturated $NaHCO_3$ and brine, dried over anhydrous $MgSO_4$, filtered, and concentrated *in vacuo*. Purification by flash chromatography on silica gel (0–40% EtOAc/hexanes, linear gradient) afforded 1.83 g (95%) of **S43** as a colorless gum. ^1H NMR ($CDCl_3$, 400 MHz) δ 7.13 (t, *J* = 7.9 Hz, 1H), 7.02 (dd, *J* = 7.9, 1.2 Hz, 1H), 6.92 – 6.87 (m, 1H), 6.73 (t, *J* = 2.1 Hz, 1H), 6.65 – 6.61 (m, 1H), 6.50 (dd, *J* = 7.9, 1.9 Hz, 1H), 6.34 (d, *J* = 1.9 Hz, 1H), 4.52 (s, 1H), 3.33 (dq, *J* = 14.2, 7.0 Hz, 1H), 3.05 (dq, *J* = 14.1, 6.9 Hz, 1H), 2.90 – 2.79 (m, 1H), 1.67 (dd, *J* = 12.9, 4.8 Hz, 1H), 1.64 (s, 6H), 1.54 (t, *J* = 12.8 Hz, 1H), 1.29 (d, *J* = 7.3 Hz, 3H), 1.28 (s, 3H), 1.15 (s, 3H), 1.02 (t, *J* = 7.0 Hz, 3H); ^{13}C NMR ($CDCl_3$, 101 MHz) δ 155.2 (C), 153.4 (C), 149.0 (C), 144.2 (C), 129.1 (CH), 125.3 (CH), 125.0 (C), 119.5 (CH), 114.3 (CH), 113.4 (CH), 112.4 (CH), 110.5 (CH), 54.4 (C), 47.2 (CH_2), 42.9 (C), 39.1 (CH_2), 30.8 (CH_3), 29.9 (CH_3), 27.0 (CH), 25.4 (CH_3), 20.0 (CH_3), 15.0 (CH_3); HRMS (ESI) calcd for $C_{23}H_{32}NO$ $[M+H]^+$ 338.2478, found 338.2478.

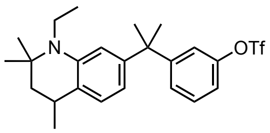

3-(2-(1-Ethyl-2,2,4-trimethyl-1,2,3,4-tetrahydroquinolin-7-yl)propan-2-yl)phenyl trifluoromethanesulfonate (S44): 3-(2-(1-Ethyl-2,2,4-trimethyl-1,2,3,4-tetrahydroquinolin-7-yl)propan-2-yl)phenol (**S43**; 1.70 g, 5.04 mmol) was taken up in CH_2Cl_2 (50 mL). DIEA (2.63 mL, 15.11 mmol, 3 eq) and *N*-phenyl-bis(trifluoromethanesulfonimide) (3.60 g, 10.07 mmol, 2 eq) were added, and the reaction was stirred at room temperature for 18 h. The solvent was removed by rotary evaporation, and the crude residue was purified by silica gel chromatography (0–40%

CH_2Cl_2/hexanes, linear gradient) to afford 2.24 g (95%) of **S44** as a white solid. ^1H NMR ($CDCl_3$, 400 MHz) δ 7.35 – 7.27 (m, 2H), 7.19 (t, *J* = 2.0 Hz, 1H), 7.07 (dt, *J* = 7.4, 2.2 Hz, 1H), 7.04 (d, *J* = 7.9 Hz, 1H), 6.48 (dd, *J* = 7.9, 1.9 Hz, 1H), 6.23 (d, *J* = 1.9 Hz, 1H), 3.31 (dq, *J* = 14.3, 7.1 Hz, 1H), 3.01 (dq, *J* = 14.1, 6.9 Hz, 1H), 2.90 – 2.79 (m, 1H), 1.68 (dd, *J* = 12.9, 4.7 Hz, 1H), 1.67 (s, 6H), 1.53 (t, *J* = 12.8 Hz, 1H), 1.30 (d, *J* = 6.6 Hz, 3H), 1.28 (s, 3H), 1.15 (s, 3H), 0.98 (t, *J* = 7.0 Hz, 3H); ^{19}F NMR ($CDCl_3$, 376 MHz) δ -73.41 (s); ^{13}C NMR ($CDCl_3$, 101 MHz) δ 154.7 (C), 149.7 (C), 147.9 (C), 144.4 (C), 129.6 (CH), 127.4 (CH), 125.6 (CH), 119.9 (CH), 118.9 (q, $^1J_{CF}$ = 320.8 Hz, CF_3), 118.3 (CH), 113.1 (CH), 110.4 (CH), 54.5 (C), 47.1 (CH_2), 43.2 (C), 39.0 (CH_2), 30.64 (CH_3), 30.63 (CH_3), 29.8 (CH_3), 27.1 (CH), 25.3 (CH_3), 20.0 (CH_3), 14.8 (CH_3); HRMS (ESI) calcd for $C_{24}H_{31}F_3NO_3S$ $[M+H]^+$ 470.1971, found 470.1974.

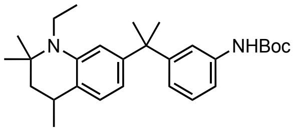

***tert*-Butyl (3-(2-(1-ethyl-2,2,4-trimethyl-1,2,3,4-tetrahydroquinolin-7-yl)propan-2-yl)phenyl)carbamate (S45):** An oven-dried crimp-top vial was charged with *tert*-butyl carbamate (299 mg, 2.56 mmol, 1.2 eq), Pd_2dba_3 (97.5 mg, 0.106 mmol, 0.05 eq), XPhos (152 mg, 0.319 mmol, 0.15 eq), and Cs_2CO_3 (971 mg, 2.98 mmol, 1.4 eq). The vial was sealed and evacuated/backfilled with nitrogen (3×). A solution of 3-(2-(1-ethyl-2,2,4-trimethyl-1,2,3,4-tetrahydroquinolin-7-yl)propan-2-yl)phenyl trifluoromethanesulfonate (**S44**; 1.00 g, 2.13 mmol) in dioxane (10 mL) was added; after flushing the reaction again with nitrogen (3×), it was stirred at 100 °C for 4 h. It was then cooled to room temperature, filtered through Celite with CH_2Cl_2, and evaporated. The residue was purified by silica gel chromatography (0–20% Et_2O/hexanes, linear gradient) to afford **S45** (906 mg, 97%) as pale yellow gum. ^1H NMR ($CDCl_3$, 400 MHz) δ 7.43 – 7.30 (m, 1H), 7.19 (t, *J* = 7.9 Hz, 1H), 7.05 (t, *J* = 2.0 Hz, 1H), 7.01 (dd, *J* = 7.9, 1.2 Hz, 1H), 6.97 (ddd, *J* = 7.8, 1.9, 1.0 Hz, 1H), 6.48 (dd, *J* = 7.9, 1.9 Hz, 1H), 6.38 (s, 1H), 6.34 (d, *J* = 1.9 Hz, 1H), 3.33 (dq, *J* = 14.2, 7.0 Hz, 1H), 3.05 (dq, *J* = 13.9, 6.9 Hz, 1H), 2.90 – 2.78 (m, 1H), 1.67 (dd, *J* = 12.9, 4.7 Hz, 1H), 1.65 (s, 6H), 1.54 (t, *J* = 12.9 Hz, 1H), 1.49 (s, 9H), 1.29 (d, *J* = 7.0 Hz, 3H), 1.28 (s, 3H), 1.15 (s, 3H), 1.02 (t, *J* = 7.0 Hz, 3H); ^{13}C NMR ($CDCl_3$, 101 MHz) δ 152.9 (C), 152.2 (C), 149.0 (C), 144.2 (C), 138.0 (C), 128.6 (CH), 125.3 (CH), 125.0 (C), 121.9 (CH), 117.5 (CH), 116.0 (CH), 113.5 (CH), 110.4 (CH), 80.4 (C), 54.4 (C), 47.2 (CH_2), 43.0 (C), 39.0 (CH_2), 30.9 (CH_3), 29.9 (CH_3), 28.5 (CH_3), 27.1 (CH), 25.4 (CH_3), 20.0 (CH_3), 15.0 (CH_3); HRMS (ESI) calcd for $C_{28}H_{41}N_2O_2$ $[M+H]^+$ 437.3163, found 437.3163.

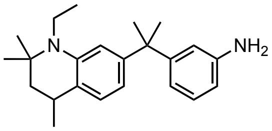

3-(2-(1-Ethyl-2,2,4-trimethyl-1,2,3,4-tetrahydroquinolin-7-yl)propan-2-yl)aniline (S46): *tert*-Butyl (3-(2-(1-ethyl-2,2,4-trimethyl-1,2,3,4-tetrahydroquinolin-7-yl)propan-2-yl)phenyl)carbamate (**S45**; 1.90 g, 4.35 mmol) was taken up in CH_2Cl_2 (35 mL), and trifluoroacetic acid (7 mL) was added. The reaction was stirred at room temperature for 2 h. It was then diluted with toluene (30 mL) and concentrated to dryness. The residue was taken up in saturated

$NaHCO_3$ and extracted with EtOAc (2×). The combined organic extracts were washed with brine, dried over anhydrous $MgSO_4$, filtered, concentrated *in vacuo*. Flash chromatography (0–30% EtOAc/hexanes, linear gradient) afforded 1.29 g (88%) of **S46** as an off-white solid. ^1H NMR ($CDCl_3$, 400 MHz) δ 7.05 (t, J = 7.8 Hz, 1H), 7.01 (dd, J = 7.9, 1.0 Hz, 1H), 6.73 (ddd, J = 7.8, 1.7, 0.9 Hz, 1H), 6.61 (t, J = 2.1 Hz, 1H), 6.53 – 6.47 (m, 2H), 6.38 (d, J = 1.9 Hz, 1H), 3.53 (s, 2H), 3.33 (dq, J = 14.2, 7.0 Hz, 1H), 3.06 (dq, J = 14.2, 6.9 Hz, 1H), 2.90 – 2.78 (m, 1H), 1.67 (dd, J = 12.9, 4.8 Hz, 1H), 1.63 (s, 6H), 1.54 (t, J = 12.8 Hz, 1H), 1.29 (d, J = 6.7 Hz, 3H), 1.28 (s, 3H), 1.15 (s, 3H), 1.04 (t, J = 7.0 Hz, 3H); ^{13}C NMR ($CDCl_3$, 101 MHz) δ 152.5 (C), 149.3 (C), 146.0 (C), 144.1 (C), 128.8 (CH), 125.3 (CH), 124.9 (C), 117.5 (CH), 114.4 (CH), 113.5 (CH), 112.6 (CH), 110.5 (CH), 54.4 (C), 47.2 (CH_2), 42.8 (C), 39.1 (CH_2), 30.86 (CH_3), 30.84 (CH_3), 29.9 (CH_3), 27.0 (CH), 25.4 (CH_3), 20.0 (CH_3), 15.0 (CH_3); HRMS (ESI) calcd for $C_{23}H_{33}N_2$ $[M+H]^+$ 337.2638, found 337.2637.

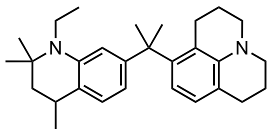

8-(2-(1-Ethyl-2,2,4-trimethyl-1,2,3,4-tetrahydroquinolin-7-yl)propan-2-yl)-2,3,6,7-tetrahydro-1*H*,5*H*-pyrido-[3,2,1-*ij*]quinoline (58): A mixture of 3-(2-(1-ethyl-2,2,4-trimethyl-1,2,3,4-tetrahydroquinolin-7-yl)propan-2-yl)aniline (**S46**; 700 mg, 2.08 mmol), Na_2CO_3 (2.20 g, 20.80 mmol, 10 eq), and 1-bromo-3-chloropropane (15.44 mL, 156.0 mmol, 75 eq) was stirred at 140 °C for 18 h. The reaction was subsequently cooled to room temperature, diluted with water, and extracted with EtOAc (2×). The combined organic extracts were dried over anhydrous $MgSO_4$, filtered, and evaporated. Silica gel chromatography (0–10% Et_2O/hexanes, linear gradient) yielded 453 mg (52%) of **58** as a colorless gum. ^1H NMR ($CDCl_3$, 400 MHz) δ 6.95 (d, J = 7.9 Hz, 1H), 6.79 (s, 2H), 6.40 (dd, J = 7.9, 1.8 Hz, 1H), 6.36 (d, J = 1.8 Hz, 1H), 3.32 (dq, J = 14.2, 7.0 Hz, 1H), 3.13 – 3.08 (m, 2H), 3.04 (dq, J = 15.0, 7.0 Hz, 1H), 2.99 (t, J = 6.3 Hz, 2H), 2.89 – 2.77 (m, 1H), 2.74 (t, J = 6.4 Hz, 2H), 2.25 – 2.12 (m, 2H), 1.97 – 1.88 (m, 2H), 1.67 (dd, J = 12.9, 4.8 Hz, 1H), 1.614 (s, 3H), 1.607 (s, 3H), 1.57 – 1.48 (m, 3H), 1.272 (s, 3H), 1.271 (d, J = 6.7 Hz, 3H), 1.14 (s, 3H), 1.01 (t, J = 6.9 Hz, 3H); ^{13}C NMR ($CDCl_3$, 101 MHz) δ 150.3 (C), 145.3 (C), 144.3 (C), 143.7 (C), 125.9 (CH), 125.3 (CH), 124.6 (C), 122.4 (C), 120.4 (C), 114.3 (CH), 112.9 (CH), 109.3 (CH), 54.3 (C), 50.9 (CH_2), 50.3 (CH_2), 47.5 (CH_2), 43.4 (C), 39.0 (CH_2), 32.0 (CH_3), 31.3 (CH_3), 29.8 (CH_3), 28.2 (CH_2), 27.1 (CH), 26.2 (CH_2), 25.2 (CH_3), 22.4 (CH_2), 22.1 (CH_2), 20.2 (CH_3), 15.0 (CH_3); HRMS (ESI) calcd for $C_{29}H_{41}N_2$ $[M+H]^+$ 417.3264, found 417.3264.

PREPARATION OF ANTHRONE INTERMEDIATES (SCHEME S5)

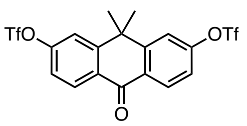

9,9-Dimethyl-10-oxo-9,10-dihydroanthracene-2,7-diyl bis(trifluoromethanesulfonate) (S48): 3,6-Dihydroxy-10,10-dimethylanthracen-9(10*H*)-one[6] (**S47**; 747 mg, 2.94 mmol) was taken up in CH_2Cl_2 (15 mL) and cooled to 0 °C. Pyridine (1.90 mL, 23.50 mmol, 8 eq) and trifluoromethanesulfonic anhydride (1.98 mL, 11.75 mmol, 4 eq) were added, and the ice bath was removed. The reaction was stirred at room temperature for 2 h. It was subsequently diluted with water and extracted with CH_2Cl_2 (2×). The combined organic extracts were washed with saturated $CuSO_4$ and brine, dried over anhydrous $MgSO_4$, filtered, and concentrated *in vacuo*. Flash chromatography on silica gel (0–30% EtOAc/hexanes, linear gradient) afforded 1.46 g (96%) of **S48** as a white solid. ^{1}H NMR ($CDCl_3$, 400 MHz) δ 8.47 (d, J = 8.7 Hz, 2H), 7.58 (d, J = 2.4 Hz, 2H), 7.38 (dd, J = 8.8, 2.4 Hz, 2H), 1.78 (s, 6H); ^{19}F NMR ($CDCl_3$, 376 MHz) δ -73.13 (s); ^{13}C NMR ($CDCl_3$, 101 MHz) δ 180.9 (C), 153.4 (C), 152.7 (C), 130.9 (CH), 129.4 (C), 120.5 (CH), 120.0 (CH), 118.9 (q, $^1J_{CF}$ = 320.8 Hz, CF_3) 38.8 (C), 33.0 (CH_3); HRMS (ESI) calcd for $C_{18}H_{13}F_6O_7S_2$ [M+H]$^+$ 519.0001, found 518.9993.

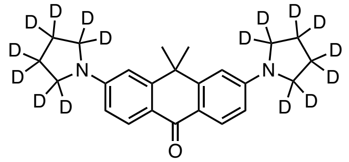

10,10-Dimethyl-3,6-bis(pyrrolidin-1-yl-*d*$_8$)anthracen-9(10*H*)-one (42): A vial was charged with 9,9-dimethyl-10-oxo-9,10-dihydroanthracene-2,7-diyl bis(trifluoromethanesulfonate) (**S48**; 1.00 g, 1.93 mmol), Pd_2dba_3 (177 mg, 0.193 mmol, 0.1 eq), XPhos (276 mg, 0.579 mmol, 0.3 eq), and Cs_2CO_3 (1.76 g, 5.40 mmol, 2.8 eq). The vial was sealed and evacuated/backfilled with nitrogen (3×). Dioxane (10 mL) was added, and the reaction was flushed again with nitrogen (3×). Following the addition of pyrrolidine-2,2,3,3,4,4,5,5-*d*$_8$ (387 µL, 4.63 mmol, 2.4 eq), the reaction was stirred at 100 °C for 3 h. It was then cooled to room temperature, filtered through Celite with CH_2Cl_2, and concentrated to dryness. Purification by silica gel chromatography (0–40% EtOAc/hexanes, linear gradient, with constant 40% v/v CH_2Cl_2 additive) afforded **42** (498 mg, 69%) as a yellow solid. ^{1}H NMR ($CDCl_3$, 400 MHz) δ 8.28 – 8.25 (m, 2H), 6.64 – 6.60 (m, 4H), 1.71 (s, 6H); ^{13}C NMR ($CDCl_3$, 101 MHz) δ 181.3 (C), 152.6 (C), 150.8 (C), 129.5 (CH), 119.6 (C), 111.0 (CH), 107.6 (CH), 38.2 (C), 33.9 (CH_3); HRMS (ESI) calcd for $C_{24}H_{13}D_{16}N_2O$ [M+H]$^+$ 377.3279, found 377.3264.

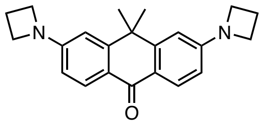

3,6-Di(azetidin-1-yl)-10,10-dimethylanthracen-9(10*H*)-one (43): A vial was charged with 9,9-dimethyl-10-oxo-9,10-dihydroanthracene-2,7-diyl bis(trifluoromethanesulfonate) (**S48**; 1.25 g, 2.41 mmol), Pd_2dba_3 (221 mg, 0.241 mmol, 0.1 eq), XPhos (345 mg, 0.723 mmol, 0.3 eq), and Cs_2CO_3 (2.20 g, 6.75 mmol, 2.8 eq). The vial was sealed and evacuated/backfilled with nitrogen (3×). Dioxane (12 mL) was added, and the reaction was flushed again with nitrogen (3×). Following the addition of azetidine (390 μL, 5.79 mmol, 2.4 eq), the reaction was stirred at 100 °C for 4 h. It was then cooled to room temperature, filtered through Celite with CH_2Cl_2, and concentrated to dryness. Purification by silica gel chromatography (0–40% EtOAc/hexanes, linear gradient, with constant 40% v/v CH_2Cl_2 additive) afforded **43** (645 mg, 80%) as a yellow solid. ^{1}H NMR ($CDCl_3$, 400 MHz) δ 8.26 – 8.21 (m, 2H), 6.46 – 6.40 (m, 4H), 4.03 (t, J = 7.3 Hz, 8H), 2.43 (p, J = 7.2 Hz, 4H), 1.66 (s, 6H); ^{13}C NMR ($CDCl_3$, 101 MHz) δ 181.4 (C), 154.3 (C), 152.4 (C), 129.3 (CH), 120.7 (C), 109.8 (CH), 106.5 (CH), 51.8 (CH_2), 38.1 (C), 33.6 (CH_3), 16.7 (CH_2); HRMS (ESI) calcd for $C_{22}H_{25}N_2O$ $[M+H]^+$ 333.1961, found 333.1959.

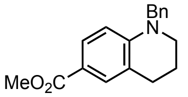

Methyl 1-benzyl-1,2,3,4-tetrahydroquinoline-6-carboxylate (S50): Methyl 1,2,3,4-tetrahydroquinoline-6-carboxylate (**S49**; 1.90 g, 9.94 mmol), benzyl bromide (4.25 g, 24.8 mmol, 2.5 eq), K_2CO_3 (4.12 g, 29.8 mmol, 3 eq), and KI (412 mg, 2.49 mmol, 0.25 eq) were combined in DMF (25 mL) and stirred at 60 °C for 4 h. The reaction was subsequently diluted with water and extracted with EtOAc (2×). The combined organic extracts were washed with water and brine, dried ($MgSO_4$), filtered, and concentrated *in vacuo*. Silica gel chromatography (0–25% EtOAc/hexanes, linear gradient) afforded 2.60 g (93%) of **S50** as a gum that crystallized into a colorless solid upon standing. ^{1}H NMR ($CDCl_3$, 400 MHz) δ 7.69 – 7.62 (m, 2H), 7.36 – 7.29 (m, 2H), 7.28 – 7.24 (m, 1H), 7.24 – 7.19 (m, 2H), 6.49 – 6.43 (m, 1H), 4.55 (s, 2H), 3.82 (s, 3H), 3.47 – 3.40 (m, 2H), 2.84 (t, J = 6.2 Hz, 2H), 2.07 – 1.96 (m, 2H); ^{13}C NMR ($CDCl_3$, 101 MHz) δ 167.6 (C), 149.3 (C), 137.7 (C), 130.7 (CH), 129.8 (CH), 128.9 (CH), 127.2 (CH), 126.5 (CH), 121.3 (C), 116.8 (C), 109.8 (CH), 54.8 (CH_2), 51.5 (CH_3), 50.1 (CH_2), 28.2 (CH_2), 22.0 (CH_2); HRMS (ESI) calcd for $C_{18}H_{20}NO_2$ $[M+H]^+$ 282.1489, found 282.1488.

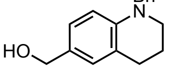

(1-Benzyl-1,2,3,4-tetrahydroquinolin-6-yl)methanol (S51): A solution of methyl 1-benzyl-1,2,3,4-tetrahydroquinoline-6-carboxylate (**S50**; 4.25 g, 15.1 mmol) in THF (50 mL) was cooled to 0 °C under nitrogen, and $LiAlH_4$ (1.0 M in THF, 30.2 mL, 30.2 mmol, 2 eq) was added. The reaction was stirred for 2 h at 0 °C. The Fieser workup was performed by the sequential addition of (1) H_2O (1.15 mL), (2) 15% NaOH (1.15 mL), and (3) H_2O (3 × 1.15 mL). The resulting suspension was vigorously stirred for 10 min. It was then diluted with Et_2O (100 mL), and $MgSO_4$ (~5 g) was added. After stirring for another 5 min, the mixture was filtered through Celite with Et_2O and

concentrated to dryness. Flash chromatography on silica gel (10–50% EtOAc/hexanes, linear gradient) provided **S51** (3.43 g, 90%) as a colorless gum. **Note:** Alcohol **S51** is photosensitive and decomposes rapidly in chlorinated solvents. ^{1}H NMR (acetone-d_6, 400 MHz) δ 7.34 – 7.25 (m, 4H), 7.25 – 7.19 (m, 1H), 6.93 – 6.90 (m, 1H), 6.90 – 6.85 (m, 1H), 6.45 (d, J = 8.3 Hz, 1H), 4.49 (s, 2H), 4.41 (d, J = 5.8 Hz, 2H), 3.71 (t, J = 5.7 Hz, 1H), 3.41 – 3.35 (m, 2H), 2.77 (t, J = 6.3 Hz, 2H), 2.02 – 1.93 (m, 2H); ^{13}C NMR (acetone-d_6, 101 MHz) δ 145.5 (C), 140.2 (C), 130.5 (C), 129.3 (CH), 129.0 (CH), 127.5 (CH), 127.4 (CH), 126.9 (CH), 122.6 (C), 111.6 (CH), 64.9 (CH_2), 55.6 (CH_2), 50.7 (CH_2), 28.9 (CH_2), 23.20 (CH_2); HRMS (ESI) calcd for $C_{17}H_{19}NONa$ [M+Na]$^+$ 276.1359, found 276.1354.

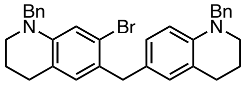

1-Benzyl-6-((1-benzyl-1,2,3,4-tetrahydroquinolin-6-yl)methyl)-7-bromo-1,2,3,4-tetrahydroquinoline (S52): 1-Benzyl-7-bromo-1,2,3,4-tetrahydroquinoline (**S4**; 2.16 g, 7.15 mmol) and (1-benzyl-1,2,3,4-tetrahydroquinolin-6-yl)methanol (**S51**; 1.90 g, 7.50 mmol, 1.05 eq) were combined in CH_2Cl_2 (50 mL) and cooled to 0 °C under nitrogen. BCl_3 (1 M in CH_2Cl_2, 21.4 mL, 21.4 mmol, 3 eq) was added over 5 min; the reaction was then allowed to warm to room temperature overnight while stirring (18 h). The reaction was carefully neutralized by the slow addition of saturated $NaHCO_3$ with vigorous stirring. The mixture was then extracted with CH_2Cl_2 (2×). The combined organics were dried ($MgSO_4$), filtered, and evaporated. Flash chromatography (0–20% Et_2O/hexanes, linear gradient) afforded 3.15 g (82%) of **S52** as a colorless, gummy foam. ^{1}H NMR ($CDCl_3$, 400 MHz) δ 7.35 – 7.19 (m, 10H), 6.83 (d, J = 2.0 Hz, 1H), 6.80 (dd, J = 8.3, 2.2 Hz, 1H), 6.74 (s, 1H), 6.69 (s, 1H), 6.43 (d, J = 8.3 Hz, 1H), 4.43 (s, 2H), 4.42 (s, 2H), 3.79 (s, 2H), 3.35 – 3.30 (m, 2H), 3.30 – 3.24 (m, 2H), 2.78 (t, J = 6.4 Hz, 2H), 2.67 (t, J = 6.3 Hz, 2H), 2.04 – 1.89 (m, 4H); ^{13}C NMR ($CDCl_3$, 101 MHz) δ 145.1 (C), 144.1 (C), 139.4 (C), 138.5 (C), 131.1 (CH), 129.6 (CH), 128.8 (CH), 128.7 (CH), 128.3 (C), 128.1 (C), 127.5 (CH), 127.1 (CH), 126.83 (CH), 126.81 (CH), 122.8 (C), 122.4 (C), 122.0 (C), 114.4 (CH), 111.2 (CH), 55.6 (CH_2), 55.2 (CH_2), 50.1 (CH_2), 49.5 (CH_2), 39.7 (CH_2), 28.4 (CH_2), 27.9 (CH_2), 22.7 (CH_2), 22.3 (CH_2); HRMS (ESI) calcd for $C_{33}H_{34}BrN_2$ [M+H]$^+$ 537.1900, found 537.1914.

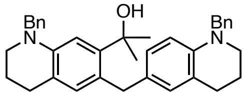

2-(1-Benzyl-6-((1-benzyl-1,2,3,4-tetrahydroquinolin-6-yl)methyl)-1,2,3,4-tetrahydroquinolin-7-yl)propan-2-ol (S53): A solution of 1-benzyl-6-((1-benzyl-1,2,3,4-tetrahydroquinolin-6-yl)methyl)-7-bromo-1,2,3,4-tetrahydro-quinoline (**S52**; 2.35 g, 4.37 mmol) in THF (40 mL) was cooled to −78 °C under nitrogen. *n*-Butyllithium (2.5 M in hexanes, 4.37 mL, 10.9 mmol, 2.5 eq) was added, and the reaction was stirred for 15 min at −78 °C. Acetone (3.21 mL, 43.7 mmol, 10 eq) was then added. The dry ice bath was removed, and the reaction was stirred at room temperature for 4 h. It was subsequently quenched with saturated NH_4Cl and extracted with EtOAc (2×). The combined organic extracts were washed with brine, dried ($MgSO_4$), filtered, and concentrated *in vacuo*. The residue was purified by silica gel chromatography (10–40% Et_2O/hexanes, linear gradient) to provide **S53** as a colorless gum that crystallized into a white solid upon standing (2.07 g, 92%). ^{1}H NMR ($CDCl_3$, 400 MHz) δ 7.34 – 7.18 (m, 10H), 6.77 (d, J = 1.9 Hz, 1H), 6.75 (s, 1H), 6.69 (dd, J = 8.4, 2.1 Hz, 1H), 6.59 (s, 1H), 6.41 (d, J = 8.4 Hz, 1H), 4.44 (s, 2H), 4.42 (s, 2H),

4.07 (s, 2H), 3.37 – 3.27 (m, 4H), 2.79 – 2.68 (m, 4H), 2.03 – 1.93 (m, 4H), 1.63 (bs, 1H), 1.45 (s, 6H); ^{13}C NMR ($CDCl_3$, 101 MHz) δ 144.7 (C), 143.9 (C), 143.6 (C), 139.5 (C), 139.4 (C), 133.9 (CH), 130.5 (C), 129.4 (CH), 128.7 (CH), 128.6 (CH), 127.4 (CH), 126.96 (CH), 126.91 (CH), 126.85 (CH), 126.82 (CH), 126.0 (C), 122.5 (C), 121.3 (C), 111.3 (CH), 109.1 (CH), 74.2 (C), 56.0 (CH_2), 55.7 (CH_2), 50.4 (CH_2), 50.1 (CH_2), 38.0 (CH_2), 31.9 (CH_3), 28.4 (CH_2), 27.6 (CH_2), 22.7 (CH_2); HRMS (ESI) calcd for $C_{36}H_{40}N_2ONa$ $[M+Na]^+$ 539.3033, found 539.3024.

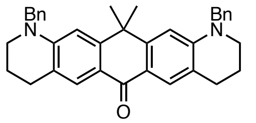

1,11-Dibenzyl-13,13-dimethyl-1,2,3,4,8,9,10,11-octahydrobenzo[1,2-*g*:5,4-*g*']diquinolin-6(13*H*)-one (45): 2-(1-Benzyl-6-((1-benzyl-1,2,3,4-tetrahydroquinolin-6-yl)methyl)-1,2,3,4-tetrahydroquinolin-7-yl)propan-2-ol (**S53**; 1.85 g, 3.58 mmol) was dissolved in CH_2Cl_2 (50 mL) and cooled to 0 °C under nitrogen. BBr_3 (1.0 M in CH_2Cl_2, 14.3 mL, 14.3 mmol, 4 eq) was added dropwise; the reaction was then allowed to warm to room temperature overnight while stirring (18 h). It was carefully neutralized through the sequential addition of H_2O (40 mL) and saturated $NaHCO_3$. The mixture was diluted with CH_2Cl_2 (50 mL), filtered through a hydrophobic phase separator column, and concentrated to give a blue residue. The crude material was taken up in acetone (35 mL) and cooled to −15 °C. $KMnO_4$ (1.13 g, 7.16 mmol, 2 eq) was added in 4 equal portions 15 min apart (45 min total). After stirring for another 30 min at −15 °C, the brown mixture was diluted with CH_2Cl_2, filtered through Celite, and concentrated *in vacuo*. Flash chromatography on silica gel (10–50% EtOAc/hexanes, linear gradient) provided 1.093 g (60%, 2 steps) of **45** as a photosensitive yellow solid. ^{1}H NMR ($CDCl_3$, 400 MHz) δ 7.96 (s, 2H), 7.34 – 7.29 (m, 4H), 7.27 – 7.21 (m, 6H), 6.48 (s, 2H), 4.57 (s, 4H), 3.52 – 3.43 (m, 4H), 2.89 (t, J = 6.2 Hz, 4H), 2.10 – 1.97 (m, 4H), 1.27 (s, 6H); ^{13}C NMR ($CDCl_3$, 101 MHz) δ 181.1 (C), 150.9 (C), 148.9 (C), 138.1 (C), 128.9 (CH), 127.8 (CH), 127.2 (CH), 126.6 (CH), 121.3 (C), 119.9 (C), 107.4 (CH), 55.5 (CH_2), 50.7 (CH_2), 37.5 (C), 33.3 (CH_3), 27.8 (CH_2), 22.3 (CH_2); HRMS (ESI) calcd for $C_{36}H_{37}N_2O$ $[M+H]^+$ 513.2900, found 513.2894.

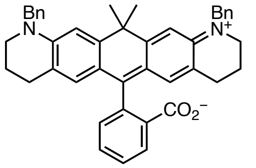

2-(1,11-Dibenzyl-13,13-dimethyl-2,3,4,8,9,10,11,13-octahydrobenzo[1,2-*g*:5,4-*g*′]diquinolin-6-ylium-6(1*H*)-yl)-benzoate (S55): *Step 1:* A solution of 1-(2-bromophenyl)-4-methyl-2,6,7-trioxabicyclo[2.2.2]octane[4] (**S54**; 556 mg, 1.95 mmol, 5 eq) in THF (30 mL) was cooled to −78 °C under nitrogen. *tert*-Butyllithium (1.7 M in pentane, 2.29 mL, 3.90 mmol, 10 eq) was added dropwise, and the reaction was stirred for 30 min at -78 °C. A solution of 1,11-dibenzyl-13,13-dimethyl-1,2,3,4,8,9,10,11-octahydrobenzo[1,2-*g*:5,4-*g*']diquinolin-6(13*H*)-one (**45**; 200 mg, 0.390 mmol) in THF (10 mL) was then added dropwise. The reaction was allowed to slowly warm to room temperature while stirring overnight (18 h). It was subsequently quenched with 1 N HCl (~20 mL), stirred for 10 min, diluted with water, and extracted with 15% *i*-PrOH/$CHCl_3$ (2×). The combined organic extracts were dried over anhydrous $MgSO_4$, filtered, and evaporated. Silica gel chromatography (0–15% MeOH/CH_2Cl_2, linear gradient, with constant 1% v/v AcOH

additive) afforded the 2,2-bis(hydroxymethyl)propyl ester intermediate as a dark blue solid (280 mg, 92%, acetate salt).

Step 2: The ester intermediate from Step 1 (280 mg, 0.359 mmol) was taken up in 2,2,2-trifluoroethanol (18 mL), and 25% w/w NaOH (6 mL) was added. The reaction was stirred at room temperature for 7 days. It was then diluted with water and extracted with CH_2Cl_2 (3×). The combined organic extracts were dried over anhydrous $MgSO_4$, filtered, and concentrated *in vacuo*. Purification by flash chromatography on silica gel (5–75% EtOAc/toluene, linear gradient) afforded 175 mg (79%) of **S55** as a pale blue solid. ^{1}H NMR ($CDCl_3$, 400 MHz) δ 7.99 (d, J = 7.4 Hz, 1H), 7.58 (td, J = 7.5, 1.4 Hz, 1H), 7.53 (td, J = 7.2, 1.2 Hz, 1H), 7.35 – 7.29 (m, 4H), 7.28 – 7.20 (m, 6H), 7.07 (d, J = 7.3 Hz, 1H), 6.53 (s, 2H), 6.23 (s, 2H), 4.49 (AB quartet, ν_A = 1809.4 Hz, ν_B = 1781.5 Hz, J_{AB} = 16.8 Hz, 4H), 3.42 – 3.30 (m, 4H), 2.65 – 2.47 (m, 4H), 1.98 – 1.86 (m, 4H), 1.41 (s, 3H), 1.31 (s, 3H); ^{13}C NMR ($CDCl_3$, 101 MHz) δ 171.2 (C), 155.8 (C), 145.9 (C), 144.9 (C), 138.8 (C), 134.5 (CH), 128.75 (CH), 128.70 (CH), 128.0 (CH), 127.3 (C), 127.0 (CH), 126.7 (CH), 124.8 (CH), 124.1 (CH), 121.6 (C), 118.6 (C), 108.2 (CH), 88.7 (C), 55.6 (CH_2), 50.3 (CH_2), 37.7 (C), 34.9 (CH_3), 33.0 (CH_3), 27.7 (CH_2), 22.3 (CH_2); Analytical HPLC: t_R = 12.0 min, >99% purity (30–95% MeCN/H_2O, linear gradient, with constant 0.1% v/v TFA additive; 20 min run; 1 mL/min flow; ESI; positive ion mode; detection at 625 nm); HRMS (ESI) calcd for $C_{43}H_{41}N_2O_2$ [M+H]$^+$ 617.3163, found 617.3156.

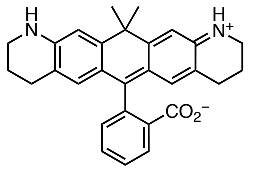

Carbo-Q-rhodamine (CRh$_Q$, 94): A round-bottom flask was charged with Pd/C (10%, 34.5 mg, 32.4 μmol, 0.2 eq) under nitrogen, and a solution of carborhodamine **S55** (100 mg, 0.162 mmol) in 1:1 THF/MeOH (15 mL) was added. The sealed flask was evacuated/backfilled with H_2 from a balloon (4×) and then stirred under the H_2 balloon at room temperature for 24 h. The reaction mixture was filtered through Celite with MeOH, and the filtrate was concentrated *in vacuo*. The blue residue was resuspended in 3:1 CH_2Cl_2/MeOH (8 mL), and *p*-chloranil (79.7 mg, 0.324 mmol, 2 eq) was added. After stirring the mixture at room temperature for 2 h, it was deposited onto Celite and concentrated to dryness. Silica gel chromatography (0–10% MeOH (2 M NH_3)/CH_2Cl_2, linear gradient; dry load with Celite) yielded 49.5 mg (70%) of **94** as a blue-purple solid. ^{1}H NMR (DMSO-d_6, 400 MHz) δ 7.91 (d, J = 7.6 Hz, 1H), 7.71 (td, J = 7.4, 1.1 Hz, 1H), 7.62 (td, J = 7.4, 1.0 Hz, 1H), 7.07 (d, J = 7.6 Hz, 1H), 6.68 (s, 2H), 6.01 (s, 2H), 5.92 (s, 2H), 3.20 – 3.06 (m, 4H), 2.45 (dt, J = 15.9, 5.9 Hz, 2H), 2.35 (dt, J = 15.8, 6.1 Hz, 2H), 1.68 (p, J = 6.1 Hz, 4H), 1.64 (s, 3H), 1.54 (s, 3H); ^{13}C NMR (DMSO-d_6, 101 MHz) δ 170.0 (C), 155.0 (C), 146.0 (C), 144.1 (C), 135.0 (CH), 129.1 (CH), 127.4 (CH), 126.3 (C), 124.4 (CH), 123.9 (CH), 119.1 (C), 117.9 (C), 109.7 (CH), 88.5 (C), 40.6 (CH_2), 36.8 (C), 35.0 (CH_3), 32.7 (CH_3), 26.3 (CH_2), 21.3 (CH_2); Analytical HPLC: t_R = 11.8 min, 98.3% purity (10–95% MeCN/H_2O, linear gradient, with constant 0.1% v/v TFA additive; 20 min run; 1 mL/min flow; ESI; positive ion mode; detection at 600 nm); HRMS (ESI) calcd for $C_{29}H_{29}N_2O_2$ [M+H]$^+$ 437.2224, found 437.2219.

XANTHENE SYNTHESIS VIA LITHIATION OF TETRAFLUOROBENZOIC ACID (SCHEME S6)

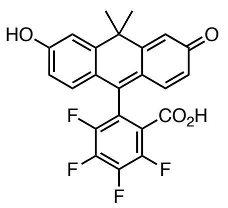

4,5,6,7-Tetrafluoro-carbofluorescein (51): A solution of 2,3,4,5-tetrafluorobenzoic acid (**6**; 281 mg, 1.45 mmol, 2 eq) in THF (5 mL) was cooled to -78 °C under nitrogen. *N*-Butyllithium (2.5 M in hexanes, 1.16 mL, 2.90 mmol, 4 eq) was added, and the reaction was stirred at -78 °C for 3 h. A solution of 3,6-bis((*tert*-butyldimethylsilyl)oxy)-10,10-dimethylanthracen-9(10*H*)-one[6] (**50**; 350 mg, 0.725 mmol) in THF (4 mL) was added; the reaction was warmed to room temperature and stirred for 1 h. It was subsequently diluted with saturated NH_4Cl and water and extracted with EtOAc (2×). The combined organic extracts were washed with brine, dried over anhydrous $MgSO_4$, filtered, and evaporated. The resulting yellow-orange residue was taken up in THF (10 mL) before adding TBAF (1.0 M in THF, 2.90 mL, 2.90 mmol, 4 eq). After stirring the reaction at room temperature for 30 min, it was acidified with 1 M HCl, diluted with water, and extracted with EtOAc (2×). The organic extracts were washed with brine, dried over anhydrous $MgSO_4$, filtered, and concentrated *in vacuo*. Flash chromatography (0–75% EtOAc/hexanes, linear gradient) afforded **51** (258 mg, 83%) as a pale yellow solid. ^{1}H NMR (DMSO-d_6, 400 MHz) δ 9.81 (s, 2H), 7.10 (d, J = 2.4 Hz, 2H), 6.84 (d, J = 8.6 Hz, 2H), 6.66 (dd, J = 8.6, 2.4 Hz, 2H), 1.69 (s, 3H), 1.60 (s, 3H); ^{19}F NMR (DMSO-d_6, 376 MHz) δ -139.94 – -140.16 (m, 1F), -143.10 – -143.30 (m, 1F), -143.78 (td, J = 21.4, 20.8, 8.4 Hz, 1F), -152.01 – -152.23 (m, 1F); Analytical HPLC: t_R = 12.8 min, >99% purity (10–95% MeCN/H_2O, linear gradient, with constant 0.1% v/v TFA additive; 20 min run; 1 mL/min flow; ESI; positive ion mode; detection at 254 nm); HRMS (ESI) calcd for $C_{23}H_{15}F_4O_4$ $[M+H]^+$ 431.0901, found 431.0899.

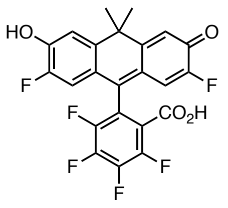

2′,4,5,6,7,7′-Hexafluorocarbofluorescein (53): A solution of 2,3,4,5-tetrafluorobenzoic acid (**6**; 281 mg, 1.45 mmol, 1.5 eq) in THF (5 mL) was cooled to -78 °C under nitrogen. *N*-Butyllithium (2.5 M in hexanes, 1.16 mL, 2.89 mmol, 3 eq) was added, and the reaction was stirred at -78 °C for 3 h. A solution of 3,6-bis((*tert*-butyldimethylsilyl)oxy)-2,7-difluoro-10,10-dimethylanthracen-9(10*H*)-one[7] (**52**; 500 mg, 0.964 mmol) in THF (5 mL) was added; the reaction was warmed to room temperature and stirred for 1 h. It was subsequently diluted with saturated NH_4Cl and water and extracted with EtOAc (2×). The combined organic extracts were washed with brine, dried over anhydrous $MgSO_4$, filtered, and evaporated. The resulting yellow-orange residue was taken up in THF (10 mL) before adding TBAF (1.0 M in THF, 3.86 mL, 3.86 mmol, 4 eq). After stirring the reaction at room temperature for 30 min, it was acidified with 1 M HCl, diluted with water, and extracted with EtOAc (2×). The organic extracts were washed with brine, dried over

anhydrous $MgSO_4$, filtered, and concentrated *in vacuo*. Flash chromatography (0–75% EtOAc/hexanes, linear gradient, with constant 0.1% v/v AcOH additive) afforded **53** (345 mg, 77%) as a pale yellow solid. ^1H NMR (DMSO-d_6, 400 MHz) δ 10.35 (s, 2H), 7.28 (d, $^4J_{HF}$ = 8.7 Hz, 2H), 6.87 (d, $^3J_{HF}$ = 12.0 Hz, 2H), 1.66 (s, 3H), 1.58 (s, 3H); ^{19}F NMR (DMSO-d_6, 376 MHz) δ -136.55 – -136.80 (m, 2F), -139.04 – -139.27 (m, 1F), -143.52 – -143.86 (m, 2F), -152.25 (t, J = 19.8 Hz, 1F); Analytical HPLC: t_R = 13.4 min, 98.9% purity (10–95% MeCN/H_2O, linear gradient, with constant 0.1% v/v TFA additive; 20 min run; 1 mL/min flow; ESI; positive ion mode; detection at 280 nm); HRMS (ESI) calcd for $C_{23}H_{13}F_6O_4$ $[M+H]^+$ 467.0713, found 467.0705.

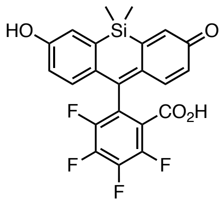

4,5,6,7-Tetrafluoro-Si-fluorescein (55): A solution of 2,3,4,5-tetrafluorobenzoic acid (**6**; 292 mg, 1.50 mmol, 1.5 eq) in THF (5 mL) was cooled to -78 °C under nitrogen. *N*-Butyllithium (2.5 M in hexanes, 1.20 mL, 3.01 mmol, 3 eq) was added, and the reaction was stirred at -78 °C for 3 h. A solution of 3,7-bis((*tert*-butyldimethylsilyl)oxy)-5,5-dimethyldibenzo[*b*,*e*]silin-10(5*H*)-one[8] (**54**; 500 mg, 1.00 mmol) in THF (5 mL) was added; the reaction was warmed to room temperature and stirred for 1 h. It was subsequently diluted with saturated NH_4Cl and water and extracted with EtOAc (2×). The combined organic extracts were washed with brine, dried over anhydrous $MgSO_4$, filtered, and evaporated. The resulting red-orange residue was taken up in THF (10 mL) before adding TBAF (1.0 M in THF, 4.00 mL, 4.00 mmol, 4 eq). After stirring the reaction at room temperature for 30 min, it was acidified with 1 M HCl, diluted with water, and extracted with EtOAc (2×). The organic extracts were washed with brine, dried over anhydrous $MgSO_4$, filtered, and concentrated *in vacuo*. Flash chromatography (10–100% EtOAc/hexanes, linear gradient) afforded **55** (422 mg, 94%) as an orange foam. The characterization data for **55** matched the previously reported spectra.[1]

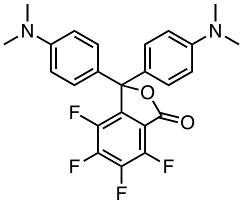

4,5,6,7-Tetrafluoro-Malachite Green lactone (FMGL, 49): A solution of 2,3,4,5-tetrafluorobenzoic acid (**6**; 868 mg, 4.47 mmol, 6 eq) in THF (15 mL) was cooled to -78 °C under nitrogen. *N*-Butyllithium (2.5 M in hexanes, 3.58 mL, 8.94 mmol, 12 eq) was added, and the reaction was stirred at -78 °C for 3 h. A solution of Michler's ketone (**48**; 200 mg, 0.745 mmol) in THF (10 mL) was added; the reaction was warmed to room temperature and stirred for 18 h. It was subsequently diluted with saturated NH_4Cl and water and extracted with EtOAc (2×). The combined organic extracts were washed with saturated $NaHCO_3$ and brine, dried over anhydrous $MgSO_4$, filtered, and concentrated *in vacuo*. Purification by silica gel chromatography (0–50% EtOAc/hexanes, linear gradient) yielded 266 mg (80%) of

49 as a blue-green foam. ^{1}H NMR ($CDCl_3$, 400 MHz) δ 7.15 (d, *J* = 9.0 Hz, 4H), 6.64 (d, *J* = 9.0 Hz, 4H), 2.96 (s, 12H); ^{19}F NMR ($CDCl_3$, 376 MHz) δ -137.82 – -137.98 (m, 1F), -138.99 (td, *J* = 20.0, 8.7 Hz, 1F), -143.58 (ddd, *J* = 21.1, 18.2, 8.7 Hz, 1F), -152.13 (ddd, *J* = 20.8, 18.3, 4.3 Hz, 1F); Analytical HPLC: t_R = 12.5 min, >99% purity (10–95% MeCN/H_2O, linear gradient, with constant 0.1% v/v TFA additive; 20 min run; 1 mL/min flow; ESI; positive ion mode; detection at 280 nm); HRMS (ESI) calcd for $C_{24}H_{21}F_4N_2O_2$ $[M+H]^+$ 445.1534, found 445.1531.

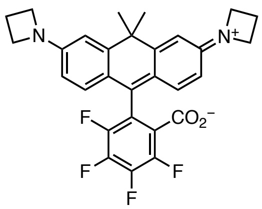

JF_{632} (44): A solution of 2,3,4,5-tetrafluorobenzoic acid (**6**; 525 mg, 2.71 mmol, 6 eq) in THF (9 mL) was cooled to -78 °C under nitrogen. *N*-Butyllithium (2.5 M in hexanes, 2.17 mL, 5.41 mmol, 12 eq) was added, and the reaction was stirred at -78 °C for 3 h. A solution of 3,6-di(azetidin-1-yl)-10,10-dimethylanthracen-9(10*H*)-one (**43**; 150 mg, 0.451 mmol) in THF (40 mL) was added; the reaction was warmed to room temperature and stirred for 18 h. It was subsequently diluted with saturated NH_4Cl and water and extracted with EtOAc (2×). The combined organic extracts were washed with saturated $NaHCO_3$ and brine, dried over anhydrous $MgSO_4$, filtered, and concentrated *in vacuo*. Purification by silica gel chromatography (0–10% MeOH (2 M NH_3)/CH_2Cl_2, linear gradient) yielded 121 mg (53%) of **44** as a deep blue solid. ^{1}H NMR ($CDCl_3$, 400 MHz) δ 6.66 (d, *J* = 8.6 Hz, 2H), 6.54 (d, *J* = 2.4 Hz, 2H), 6.27 (dd, *J* = 8.6, 2.3 Hz, 2H), 3.93 (t, *J* = 7.4 Hz, 8H), 2.39 (p, *J* = 7.2 Hz, 4H), 1.75 (s, 3H), 1.70 (s, 3H); ^{19}F NMR ($CDCl_3$, 376 MHz) δ -139.76 (td, *J* = 20.1, 8.6 Hz, 1F), -141.87 (td, *J* = 20.1, 3.9 Hz, 1F), -143.57 (ddd, *J* = 20.4, 18.0, 8.4 Hz, 1F), -152.12 (ddd, *J* = 20.4, 18.6, 3.8 Hz, 1F); Analytical HPLC: t_R = 12.0 min, >99% purity (10–95% MeCN/H_2O, linear gradient, with constant 0.1% v/v TFA additive; 20 min run; 1 mL/min flow; ESI; positive ion mode; detection at 625 nm); HRMS (ESI) calcd for $C_{29}H_{25}F_4N_2O_2$ $[M+H]^+$ 509.1847, found 509.1839.

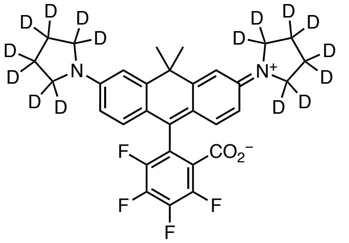

JFX_{637} (40): *Via lithiation of tetrafluorobenzoic acid:* A solution of 2,3,4,5-tetrafluorobenzoic acid (**6**; 557 mg, 2.87 mmol, 6 eq) in THF (10 mL) was cooled to -78 °C under nitrogen. *N*-Butyllithium (2.5 M in hexanes, 2.29 mL, 5.74 mmol, 12 eq) was added, and the reaction was stirred at -78 °C for 3 h. A solution of 10,10-dimethyl-3,6-bis(pyrrolidin-1-yl-d_8)anthracen-9(10*H*)-one (**42**; 180 mg, 0.478 mmol) in THF (15 mL) was added; the reaction was warmed to room temperature and stirred for 18 h. It was subsequently diluted with saturated NH_4Cl and water and extracted with EtOAc (2×). The combined organic extracts were washed with saturated $NaHCO_3$ and brine, dried over

anhydrous $MgSO_4$, filtered, and concentrated *in vacuo*. Purification by silica gel chromatography (0–10% MeOH (2 M NH_3)/CH_2Cl_2, linear gradient) yielded 150 mg (57%) of **40** as a dark blue solid.

Via dibromide route: A solution of 1,1'-(propane-2,2-diylbis(4-bromo-3,1-phenylene))bis(pyrrolidine-2,2,3,3,4,4,5,5-*d*8) (**39**; 600 mg, 1.18 mmol) in THF (30 mL) was cooled to -78 °C under nitrogen. *tert*-Butyllithium (1.7 M in pentane, 3.05 mL, 5.19 mmol, 4.4 eq) was added, and the reaction was stirred at -78 °C for 30 min. It was then warmed to -10 °C before adding a solution of $MgBr_2·OEt_2$ (670 mg, 2.60 mmol, 2.2 eq) in THF (20 mL). After an additional 30 min at -10 °C, a solution of tetrafluorophthalic anhydride (**4**; 571 mg, 2.60 mmol, 2.2 eq) in THF (20 mL) was added dropwise over 30 min via addition funnel. The reaction was then allowed to warm to room temperature overnight (18 h). It was subsequently diluted with saturated NH_4Cl and water and extracted with EtOAc (2×). The combined organic extracts were washed with saturated $NaHCO_3$ and brine, dried over anhydrous $MgSO_4$, filtered, and concentrated *in vacuo*. Silica gel chromatography (0–10% MeOH (2 M NH_3)/CH_2Cl_2, linear gradient) afforded 257 mg (39%) of **40** as a deep blue solid.

^{1}H NMR ($CDCl_3$, 400 MHz) δ 6.694 (d, *J* = 2.5 Hz, 2H), 6.691 (d, *J* = 8.7 Hz, 2H), 6.41 (dd, *J* = 8.7, 2.5 Hz, 2H), 1.81 (s, 3H), 1.75 (s, 3H); ^{19}F NMR ($CDCl_3$, 376 MHz) δ -140.08 (td, *J* = 20.1, 8.3 Hz, 1F), -141.94 (td, *J* = 20.1, 3.8 Hz, 1F), -143.94 – -144.24 (m, 1F), -152.52 (ddd, *J* = 21.8, 18.5, 3.6 Hz, 1F); Analytical HPLC: t_R = 13.0 min, >99% purity (10–95% MeCN/H_2O, linear gradient, with constant 0.1% v/v TFA additive; 20 min run; 1 mL/min flow; ESI; positive ion mode; detection at 650 nm); HRMS (ESI) calcd for $C_{31}H_{13}D_{16}F_4N_2O_2$ $[M+H]^+$ 553.3164, found 553.3154.

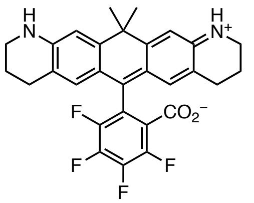

4,5,6,7-Tetrafluoro-carbo-Q-rhodamine (FCRh$_Q$, 47): *Step 1:* A solution of 2,3,4,5-tetrafluorobenzoic acid (**6**; 2.27 g, 11.70 mmol, 6 eq) in THF (40 mL) was cooled to -78 °C under nitrogen. *N*-Butyllithium (2.5 M in hexanes, 9.36 mL, 23.41 mmol, 12 eq) was added, and the reaction was stirred at -78 °C for 3 h. A solution of 1,11-dibenzyl-13,13-dimethyl-1,2,3,4,8,9,10,11-octahydrobenzo[1,2-*g*:5,4-*g*']diquinolin-6(13*H*)-one (**45**; 1.00 g, 1.95 mmol) in THF (40 mL) was added via cannula; the reaction was immediately warmed to room temperature and stirred for 48 h. It was subsequently diluted with saturated NH_4Cl and water and extracted with EtOAc (2×). The combined organic extracts were washed with saturated $NaHCO_3$ and brine, dried over anhydrous $MgSO_4$, filtered, and concentrated *in vacuo*. The residue was purified by silica gel chromatography (0–20% MeOH (2 M NH_3)/CH_2Cl_2, linear gradient) to yield 145 mg (11%) of 2-(1,11-dibenzyl-13,13-dimethyl-2,3,4,8,9,10,11,13-octahydrobenzo[1,2-*g*:5,4-*g*′]diquinolin-6-ylium-6(1*H*)-yl)-3,4,5,6-tetrafluorobenzoate (**46**) as a blue solid.

Step 2: A round-bottom flask was charged with Pd/C (10%, 44.8 mg, 42.1 μmol, 0.2 eq) under nitrogen, and a solution of the intermediate from Step 1 (**46**; 145 mg, 0.211 mmol) in MeOH (15 mL) was added. The sealed flask was evacuated/backfilled with H_2 from a balloon (4×) and then stirred under the H_2 balloon at room temperature for 18 h. The reaction mixture was filtered through Celite with MeOH and EtOAc, and the filtrate was concentrated *in vacuo*. The blue residue was resuspended in 3:1 CH_2Cl_2/MeOH (8 mL), and *p*-chloranil (104 mg, 0.421 mmol, 2 eq)

was added. After stirring the mixture at room temperature for 4 h, it was deposited onto Celite and concentrated to dryness. Silica gel chromatography (0–20% MeOH (2 M NH_3)/CH_2Cl_2, linear gradient; dry load with Celite) yielded 54.5 mg (51%) of **47** as a dark blue solid. ^{1}H NMR (CD_3OD, 400 MHz) δ 6.96 (s, 2H), 6.82 (s, 2H), 3.53 – 3.46 (m, 4H), 2.67 (t, *J* = 6.3 Hz, 4H), 1.91 (p, *J* = 6.2 Hz, 4H), 1.69 (s, 3H), 1.57 (s, 3H); ^{19}F NMR (CD_3OD, 376 MHz) δ -75.44 (s, 3F), -137.26 (ddd, *J* = 19.9, 11.9, 6.9 Hz, 1F), -138.11 (ddd, *J* = 22.2, 12.0, 4.4 Hz, 1F), -151.72 – -151.90 (m, 1F), -153.58 – -153.75 (m, 1F); Analytical HPLC: t_R = 12.1 min, >99% purity (10–95% MeCN/H_2O, linear gradient, with constant 0.1% v/v TFA additive; 20 min run; 1 mL/min flow; ESI; positive ion mode; detection at 625 nm); HRMS (ESI) calcd for $C_{29}H_{25}F_4N_2O_2$ $[M+H]^+$ 509.1847, found 509.1847.

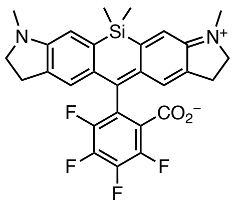

SiRF$_{712}$ (108): *Via tetrafluorobenzoic acid route:* A solution of 2,3,4,5-tetrafluorobenzoic acid (**6**; 835 mg, 4.30 mmol, 6 eq) in THF (14 mL) was cooled to -78 °C under nitrogen. *N*-Butyllithium (2.5 M in hexanes, 3.44 mL, 8.61 mmol, 12 eq) was added, and the reaction was stirred at -78 °C for 3 h. A solution of 1,9,11,11-tetramethyl-2,3,7,8,9,11-hexahydrosilino[3,2-*f*:5,6-*f'*]diindol-5(1*H*)-one[9] (**S26**; 250 mg, 0.717 mmol) in THF (100 mL) was added via cannula; the reaction was immediately warmed to room temperature and stirred for 48 h. It was subsequently diluted with saturated NH_4Cl and water and extracted with EtOAc (2×). The combined organic extracts were washed with saturated $NaHCO_3$ and brine, dried over anhydrous $MgSO_4$, filtered, and concentrated *in vacuo*. The residue was purified twice by silica gel chromatography (10–100% EtOAc/hexanes, linear gradient; then, 5–100% EtOAc/hexanes, linear gradient, with constant 20% v/v CH_2Cl_2 additive) to yield 45.9 mg (12%) of **108** as a pale blue solid.

Via dibromide route: A solution of bis(5-bromo-1-methylindolin-6-yl)dimethylsilane (**S9**; 500 mg, 1.04 mmol) in THF (25 mL) was cooled to -78 °C under nitrogen. *tert*-Butyllithium (1.7 M in pentane, 2.69 mL, 4.58 mmol, 4.4 eq) was added, and the reaction was stirred at -78 °C for 30 min. It was then warmed to -10 °C before adding a solution of $MgBr_2·OEt_2$ (591 mg, 2.29 mmol, 2.2 eq) in THF (10 mL). After an additional 30 min at -10 °C, a solution of tetrafluorophthalic anhydride (**4**; 504 mg, 2.29 mmol, 2.2 eq) in THF (10 mL) was added dropwise over 30 min via addition funnel. The reaction was then allowed to warm to room temperature overnight (18 h). It was subsequently diluted with saturated NH_4Cl and water and extracted with EtOAc (2×). The combined organic extracts were washed with saturated $NaHCO_3$ and brine, dried over anhydrous $MgSO_4$, filtered, and concentrated *in vacuo*. The crude material was redissolved in MeOH (10 mL); 1 M HCl (0.5 mL) was added, and the solution was aged 15 min. After adding 1 N NaOH (0.5 mL), the mixture was deposited onto Celite and concentrated to dryness. Silica gel chromatography (20–100% EtOAc/hexanes, linear gradient; dry load with Celite) afforded 232 mg (42%) of **108** as a light green solid.

^{1}H NMR ($CDCl_3$, 400 MHz) δ 6.63 (s, 2H), 6.60 (s, 2H), 3.33 (t, *J* = 8.2 Hz, 4H), 2.94 – 2.84 (m, 4H), 2.82 (s, 6H), 0.52 (s, 3H), 0.51 (s, 3H); ^{19}F NMR ($CDCl_3$, 376 MHz) δ -139.28 (td, *J* = 20.1, 8.5 Hz, 1F), -140.87 (td, *J* = 20.2, 3.8 Hz, 1F), -143.48 (ddd, *J* = 21.0, 18.3, 8.6 Hz, 1F), -152.48 (ddd, *J* = 21.6, 18.4, 3.7 Hz, 1F); Analytical HPLC: t_R

= 12.3 min, >99% purity (10–95% MeCN/H_2O, linear gradient, with constant 0.1% v/v TFA additive; 20 min run; 1 mL/min flow; ESI; positive ion mode; detection at 700 nm); HRMS (ESI) calcd for $C_{28}H_{25}F_4N_2O_2Si$ $[M+H]^+$ 525.1616, found 525.1614.

SYNTHESIS OF CARBORHODAMINES VIA $AlCl_3$ CONDENSATION (SCHEME S7)

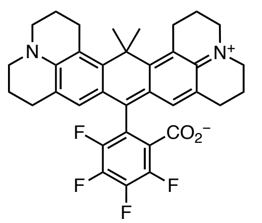

JF_{660} (57): A solution of tetrafluorophthalic anhydride (**4**; 85.4 mg, 0.388 mmol, 1.5 eq) and $AlCl_3$ (103 mg, 0.776 mmol, 3 eq) in 1,2-dichloroethane (5 mL) was added to a stirred solution of 8,8'-(propane-2,2-diyl)bis(2,3,6,7-tetrahydro-1*H*,5*H*-pyrido[3,2,1-*ij*]quinoline) (**56**; 100 mg, 0.259 mmol) in 1,2-dichloroethane (2 mL). After stirring the reaction at room temperature for 1 h, it was treated with Et_3N (108 µL, 0.776 mmol, 3 eq). A second portion of tetrafluorophthalic anhydride (57.5 mg, 0.388 mmol, 1.5 eq) and $AlCl_3$ (103 mg, 0.776 mmol, 3 eq) in 1,2-dichloroethane (5 mL) was added, and the reaction was stirred at room temperature an additional 1 h. It was then poured into ice water (50 mL), diluted with 1 M HCl (10 mL), and extracted with CH_2Cl_2 (2×). The organic extracts were washed with saturated $NaHCO_3$, dried over anhydrous $MgSO_4$, filtered, and concentrated *in vacuo*. Flash chromatography on silica gel (0–20% MeOH (2 M NH_3)/CH_2Cl_2, linear gradient) afforded 103 mg (68%) of **57** as a deep blue solid. ^{1}H NMR (CD_3OD, 400 MHz) δ 6.67 (s, 2H), 3.56 (t, *J* = 6.2 Hz, 4H), 3.51 (t, *J* = 5.9 Hz, 4H), 3.10 – 3.01 (m, 4H), 2.69 (dt, *J* = 15.9, 6.2 Hz, 2H), 2.59 (dt, *J* = 15.4, 6.3 Hz, 2H), 2.07 – 1.99 (m, 4H), 2.01 (s, 3H), 1.98 (s, 3H), 1.92 (p, *J* = 6.2 Hz, 4H); ^{19}F NMR (CD_3OD, 376 MHz) δ -139.88 (ddd, *J* = 21.8, 12.4, 3.2 Hz, 1F), -141.77 (ddd, *J* = 22.4, 12.5, 3.1 Hz, 1F), -155.38 (ddd, *J* = 22.4, 19.3, 3.1 Hz, 1F), -158.93 (ddd, *J* = 22.2, 19.2, 3.2 Hz, 1F); Analytical HPLC: t_R = 13.4 min, >99% purity (10–95% MeCN/H_2O, linear gradient, with constant 0.1% v/v TFA additive; 20 min run; 1 mL/min flow; ESI; positive ion mode; detection at 650 nm); HRMS (ESI) calcd for $C_{35}H_{33}F_4N_2O_2$ $[M+H]^+$ 589.2473, found 589.2476.

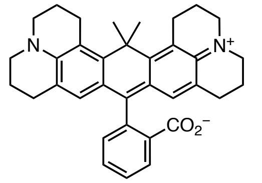

Carborhodamine 101 (CRh_{101}, 97): A solution of phthalic anhydride (**S15**; 57.5 mg, 0.388 mmol, 1.5 eq) and $AlCl_3$ (103 mg, 0.776 mmol, 3 eq) in 1,2-dichloroethane (5 mL) was added to a stirred solution of 8,8'-(propane-2,2-diyl)bis(2,3,6,7-tetrahydro-1*H*,5*H*-pyrido[3,2,1-*ij*]quinoline) (**56**; 100 mg, 0.259 mmol) in 1,2-dichloroethane (2 mL). After stirring the reaction at room temperature for 1 h, it was treated with Et_3N (108 µL, 0.776 mmol, 3 eq). A second portion of phthalic anhydride (57.5 mg, 0.388 mmol, 1.5 eq) and $AlCl_3$ (103 mg, 0.776 mmol, 3 eq) in 1,2-dichloroethane (5 mL) was added, and the reaction was stirred at room temperature an additional 1 h. It was then poured into ice water (50 mL), diluted with 1 M HCl (10 mL), and extracted with CH_2Cl_2 (2×). The organic extracts were washed with saturated $NaHCO_3$, dried over anhydrous $MgSO_4$, filtered, and concentrated *in vacuo*. Flash chromatography on silica gel (0–20% MeOH (2 M NH_3)/CH_2Cl_2, linear gradient) afforded 116 mg (87%) of **97** as a

deep blue solid. ^{1}H NMR ($CDCl_3$, 400 MHz) δ 7.97 – 7.92 (m, 1H), 7.52 (td, *J* = 7.4, 1.4 Hz, 1H), 7.47 (td, *J* = 7.4, 1.2 Hz, 1H), 7.05 – 7.01 (m, 1H), 6.11 (s, 2H), 3.20 (t, *J* = 6.7 Hz, 4H), 3.16 (t, *J* = 5.8 Hz, 4H), 2.98 – 2.85 (m, 4H), 2.56 – 2.37 (m, 4H), 2.07 (s, 3H), 2.02 (s, 3H), 1.95 (p, *J* = 6.4 Hz, 4H), 1.88 – 1.79 (m, 4H); ^{13}C NMR ($CDCl_3$, 101 MHz) δ 171.4 (C), 157.0 (C), 146.4 (C), 145.0 (C), 134.5 (CH), 128.4 (CH), 127.1 (C), 126.1 (CH), 124.7 (CH), 123.8 (CH), 121.3 (C), 119.6 (C), 116.4 (C), 50.8 (CH_2), 50.1 (CH_2), 37.0 (C), 34.6 (CH_3), 33.3 (CH_3), 28.4 (CH_2), 28.2 (CH_2), 22.3 (CH_2), 21.8 (CH_2); Analytical HPLC: t_R = 13.1 min, >99% purity (10–95% MeCN/H_2O, linear gradient, with constant 0.1% v/v TFA additive; 20 min run; 1 mL/min flow; ESI; positive ion mode; detection at 650 nm); HRMS (ESI) calcd for $C_{35}H_{37}N_2O_2$ $[M+H]^+$ 517.2850, found 517.2856.

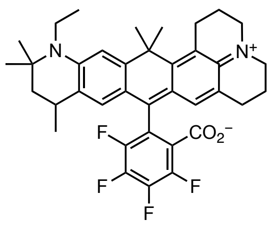

JF$_{657}$ (59): A solution of tetrafluorophthalic anhydride (**4**; 79.2 mg, 0.360 mmol, 1.5 eq) and $AlCl_3$ (96.0 mg, 0.720 mmol, 3 eq) in 1,2-dichloroethane (5 mL) was added to a stirred solution of 8-(2-(1-ethyl-2,2,4-trimethyl-1,2,3,4-tetrahydroquinolin-7-yl)propan-2-yl)-2,3,6,7-tetrahydro-1*H*,5*H*-pyrido[3,2,1-*ij*]quinoline (**58**; 100 mg, 0.240 mmol) in 1,2-dichloroethane (4 mL). After stirring the reaction at room temperature for 1 h, it was treated with Et_3N (100 µL, 0.720 mmol, 3 eq). A second portion of phthalic anhydride (79.2 mg, 0.360 mmol, 1.5 eq) and $AlCl_3$ (96.0 mg, 0.720 mmol, 3 eq) in 1,2-dichloroethane (5 mL) was added, and the reaction was stirred at room temperature an additional 2 h. It was then poured into ice water (50 mL), diluted with 1 M HCl (10 mL), and extracted with CH_2Cl_2 (2×). The organic extracts were washed with saturated $NaHCO_3$, dried over anhydrous $MgSO_4$, filtered, and concentrated *in vacuo*. Flash chromatography on silica gel (0–20% MeOH (2 M NH_3)/CH_2Cl_2, linear gradient) afforded 119 mg (80%) of **59** as a deep blue solid (mixture of two diastereomers). ^{1}H NMR (CD_3OD, 400 MHz) δ 6.91 (s, 1H), 6.88 – 6.83 (m, 2H), 3.82 (dq, *J* = 14.3, 7.1 Hz, 1H), 3.70 – 3.62 (m, 1H), 3.60 (t, *J* = 6.0 Hz, 2H), 3.55 (t, *J* = 5.9 Hz, 2H), 3.23 – 3.15 (m, 2H), 2.89 – 2.67 (m, 2H), 2.62 (dt, *J* = 15.5, 6.2 Hz, 1H), 2.10 – 2.00 (m, 2H), 1.98 – 1.90 (m, 2H), 1.90 – 1.82 (m, 1H), 1.89 (s, 1.5H), 1.87 (s, 1.5H), 1.86 (s, 1.5H), 1.85 (s, 1.5H), 1.55 (t, *J* = 13.3 Hz, 0.5H), 1.54 (t, *J* = 13.4 Hz, 0.5H), 1.48 (s, 1.5H), 1.47 (s, 1.5H), 1.36 (s, 1.5H), 1.35 (t, *J* = 7.1 Hz, 3H), 1.34 (s, 1.5H), 1.18 (d, *J* = 6.5 Hz, 1.5H), 1.14 (d, *J* = 6.5 Hz, 1.5H); ^{19}F NMR (CD_3OD, 376 MHz) δ -139.60 (ddd, *J* = 21.9, 12.4, 3.3 Hz, 0.5F), -139.68 (ddd, *J* = 21.6, 12.7, 3.2 Hz, 0.5F), -141.14 (ddd, *J* = 22.4, 12.3, 3.4 Hz, 0.5F), -141.31 (ddd, *J* = 22.5, 12.4, 3.2 Hz, 0.5F), -154.88 (ddd, *J* = 22.4, 19.2, 3.2 Hz, 0.5F), -155.04 (ddd, *J* = 22.5, 19.2, 3.0 Hz, 0.5F), -158.72 (ddd, *J* = 22.5, 18.8, 3.3 Hz, 0.5F), -159.07 (ddd, *J* = 22.0, 19.2, 3.2 Hz, 0.5F); Analytical HPLC: t_R (two isomers) = 14.1 min, 14.4 min; >99% total purity (10–95% MeCN/H_2O, linear gradient, with constant 0.1% v/v TFA additive; 20 min run; 1 mL/min flow; ESI; positive ion mode; detection at 650 nm); HRMS (ESI) calcd for $C_{37}H_{39}F_4N_2O_2$ $[M+H]^+$ 619.2942, found 619.2948.

A portion of this material (60 mg) was further purified by reverse phase HPLC (30–60% MeCN/H_2O, linear gradient, with constant 0.1% v/v TFA additive) to separate the isomeric mixture. The pooled fractions of each isomer

were partially concentrated to remove MeCN, diluted with saturated $NaHCO_3$, and extracted with CH_2Cl_2 (3×). The organic extracts were dried over anhydrous $MgSO_4$, filtered, and evaporated to provide the two individual diastereomers as blue solids: 15.7 mg of the faster-eluting isomer ("diastereomer 1") and 26.3 mg of the slower-eluting isomer ("diastereomer 2").

Diastereomer 1: ^1H NMR (CD_3OD, 400 MHz) δ 6.91 (s, 1H), 6.86 (s, 1H), 6.85 (s, 1H), 3.82 (dq, *J* = 14.2, 7.1 Hz, 1H), 3.66 (dq, *J* = 14.1, 7.4 Hz, 1H), 3.60 (t, *J* = 6.1 Hz, 2H), 3.55 (t, *J* = 5.8 Hz, 2H), 3.23 – 3.15 (m, 2H), 2.82 – 2.67 (m, 2H), 2.62 (dt, *J* = 15.4, 6.2 Hz, 1H), 2.10 – 2.00 (m, 2H), 1.98 – 1.90 (m, 2H), 1.90 – 1.82 (m, 1H), 1.89 (s, 3H), 1.85 (s, 3H), 1.55 (t, *J* = 13.3 Hz, 1H), 1.47 (s, 3H), 1.36 (s, 3H), 1.35 (t, *J* = 7.1 Hz, 3H), 1.18 (d, *J* = 6.6 Hz, 3H); ^{19}F NMR (CD_3OD, 376 MHz) δ -139.61 (ddd, *J* = 21.9, 12.4, 3.1 Hz, 1F), -141.17 (ddd, *J* = 22.6, 12.3, 3.3 Hz, 1F), -155.05 (ddd, *J* = 22.3, 19.0, 3.2 Hz, 1F), -158.78 (ddd, *J* = 22.4, 19.3, 3.2 Hz, 1F); Analytical HPLC: t_R = 14.1 min, >99% purity (10–95% MeCN/H_2O, linear gradient, with constant 0.1% v/v TFA additive; 20 min run; 1 mL/min flow; ESI; positive ion mode; detection at 650 nm); HRMS (ESI) calcd for $C_{37}H_{39}F_4N_2O_2$ $[M+H]^+$ 619.2942, found 619.2945.

Diastereomer 2: ^1H NMR (CD_3OD, 400 MHz) δ 6.91 (s, 1H), 6.88 – 6.83 (m, 2H), 3.82 (dq, *J* = 14.2, 7.0 Hz, 1H), 3.64 (dq, *J* = 14.2, 7.4 Hz, 1H), 3.60 (t, *J* = 5.9 Hz, 2H), 3.55 (t, *J* = 5.9 Hz, 2H), 3.23 – 3.15 (m, 2H), 2.89 – 2.78 (m, 1H), 2.73 (dt, *J* = 15.8, 6.1 Hz, 1H), 2.62 (dt, *J* = 15.4, 6.2 Hz, 1H), 2.10 – 2.00 (m, 2H), 1.98 – 1.90 (m, 2H), 1.90 – 1.83 (m, 1H), 1.87 (s, 3H), 1.86 (s, 3H), 1.54 (t, *J* = 13.4 Hz, 1H), 1.48 (s, 3H), 1.35 (t, *J* = 7.1 Hz, 3H), 1.34 (s, 3H), 1.14 (d, *J* = 6.5 Hz, 3H); ^{19}F NMR (CD_3OD, 376 MHz) δ -139.69 (ddd, *J* = 21.8, 12.6, 3.3 Hz, 1F), -141.32 (ddd, *J* = 22.5, 12.5, 3.1 Hz, 1F), -154.88 (ddd, *J* = 22.5, 19.3, 3.2 Hz, 1F), -159.09 (ddd, *J* = 21.7, 19.2, 3.1 Hz, 1F); Analytical HPLC: t_R = 14.4 min, >99% purity (10–95% MeCN/H_2O, linear gradient, with constant 0.1% v/v TFA additive; 20 min run; 1 mL/min flow; ESI; positive ion mode; detection at 650 nm); HRMS (ESI) calcd for $C_{37}H_{39}F_4N_2O_2$ $[M+H]^+$ 619.2942, found 619.2948.

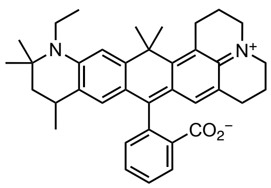

ATTO 647N free dye (S57): A solution of phthalic anhydride (**S15**; 53.3 mg, 0.360 mmol, 1.5 eq) and $AlCl_3$ (96.0 mg, 0.720 mmol, 3 eq) in 1,2-dichloroethane (5 mL) was added to a stirred solution of 8-(2-(1-ethyl-2,2,4-trimethyl-1,2,3,4-tetrahydroquinolin-7-yl)propan-2-yl)-2,3,6,7-tetrahydro-1*H*,5*H*-pyrido[3,2,1-*ij*]quinoline (**58**; 100 mg, 0.240 mmol) in 1,2-dichloroethane (4 mL). After stirring the reaction at room temperature for 1 h, it was treated with Et_3N (100 μL, 0.720 mmol, 3 eq). A second portion of phthalic anhydride (53.3 mg, 0.360 mmol, 1.5 eq) and $AlCl_3$ (96.0 mg, 0.720 mmol, 3 eq) in 1,2-dichloroethane (5 mL) was added, and the reaction was stirred at room temperature an additional 2 h. It was then poured into ice water (50 mL), diluted with 1 M HCl (10 mL), and extracted with CH_2Cl_2 (2×). The organic extracts were washed with saturated $NaHCO_3$, dried over anhydrous $MgSO_4$, filtered, and concentrated *in vacuo*. Flash chromatography on silica gel (0–20% MeOH (2 M NH_3)/CH_2Cl_2, linear gradient) afforded 110 mg (84%) of **S57** as a blue solid (mixture of two diastereomers). ^1H NMR (CD_3OD, 400 MHz) δ 8.10 –

8.01 (m, 1H), 7.61 – 7.52 (m, 2H), 7.13 – 7.07 (m, 1H), 6.881 (s, 0.5H), 6.876 (s, 0.5H), 6.85 – 6.78 (m, 2H), 3.78 (dq, *J* = 14.5, 7.1 Hz, 1H), 3.65 – 3.56 (m, 1H), 3.56 – 3.51 (m, 2H), 3.51 – 3.45 (m, 2H), 3.23 – 3.14 (m, 2H), 2.80 – 2.63 (m, 1H), 2.62 – 2.45 (m, 2H), 2.04 (p, *J* = 6.1 Hz, 2H), 1.93 – 1.85 (m, 2H), 1.910 (s, 1.5H), 1.901 (s, 1.5H), 1.892 (s, 1.5H), 1.885 (s, 1.5H), 1.85 – 1.78 (m, 1H), 1.52 – 1.45 (m, 1H), 1.45 (s, 1.5H), 1.44 (s, 1.5H), 1.36 – 1.30 (m, 3H), 1.31 (s, 1.5H), 1.30 (s, 1.5H), 1.01 (d, *J* = 6.6 Hz, 1.5H), 0.98 (d, *J* = 6.5 Hz, 1.5H); Analytical HPLC: t_R (two isomers) = 14.0 min, 14.3 min; >99% total purity (10–95% MeCN/H_2O, linear gradient, with constant 0.1% v/v TFA additive; 20 min run; 1 mL/min flow; ESI; positive ion mode; detection at 650 nm); HRMS (ESI) calcd for $C_{37}H_{43}N_2O_2$ $[M+H]^+$ 547.3319, found 547.3325.

A portion of this material (80 mg) was further purified by reverse phase HPLC (30–60% MeCN/H_2O, linear gradient, with constant 0.1% v/v TFA additive) to separate the isomeric mixture. The pooled fractions of each isomer were partially concentrated to remove MeCN, diluted with saturated $NaHCO_3$, and extracted with CH_2Cl_2 (2×). The organic extracts were dried over anhydrous $MgSO_4$, filtered, and evaporated to provide the two individual diastereomers as blue solids: 30.3 mg of the faster-eluting isomer (“diastereomer 1”) and 25.4 mg of the slower-eluting isomer (“diastereomer 2”).

Diastereomer 1: ^{1}H NMR (CD_3OD, 400 MHz) δ 8.10 – 8.05 (m, 1H), 7.57 (td, *J* = 7.4, 1.6 Hz, 1H), 7.54 (td, *J* = 7.4, 1.6 Hz, 1H), 7.11 – 7.07 (m, 1H), 6.87 (s, 1H), 6.82 (d, *J* = 1.6 Hz, 1H), 6.81 – 6.78 (m, 1H), 3.78 (dq, *J* = 14.1, 7.0 Hz, 1H), 3.59 (dq, *J* = 14.2, 7.1 Hz, 1H), 3.53 (t, *J* = 6.3 Hz, 2H), 3.47 (t, *J* = 5.8 Hz, 2H), 3.22 – 3.14 (m, 2H), 2.74 – 2.63 (m, 1H), 2.62 – 2.45 (m, 2H), 2.03 (p, *J* = 6.3 Hz, 2H), 1.92 – 1.85 (m, 2H), 1.91 (s, 3H), 1.89 (s, 3H), 1.80 (dd, *J* = 13.2, 4.3 Hz, 1H), 1.49 (d, *J* = 13.2 Hz, 1H), 1.44 (s, 3H), 1.33 (t, *J* = 7.0 Hz, 3H), 1.31 (s, 3H), 1.01 (d, *J* = 6.5 Hz, 3H); ^{13}C NMR (CD_3OD, 101 MHz) δ 173.3 (C), 165.2 (C), 158.7 (C), 153.5 (C), 152.8 (C), 151.0 (C), 140.5 (C), 139.8 (C), 135.6 (CH), 131.5 (CH), 130.6 (CH), 130.3 (CH), 130.1 (CH), 129.7 (CH), 129.3 (C), 123.7 (C), 123.4 (C), 122.2 (C), 120.5 (C), 111.8 (CH), 58.4 (C), 52.8 (CH_2), 52.2 (CH_2), 46.6 (CH_2), 41.5 (C), 40.7 (CH_2), 31.4 (CH_3), 31.0 (CH_3), 29.5 (CH_3), 28.7 (CH_2), 27.6 (CH), 26.2 (CH_3), 22.0 (CH_2), 21.9 (CH_2), 19.1 (CH_3), 14.7 (CH_3); Analytical HPLC: t_R = 14.1 min, >99% purity (10–95% MeCN/H_2O, linear gradient, with constant 0.1% v/v TFA additive; 20 min run; 1 mL/min flow; ESI; positive ion mode; detection at 650 nm); HRMS (ESI) calcd for $C_{37}H_{43}N_2O_2$ $[M+H]^+$ 547.3319, found 547.3328.

Diastereomer 2: ^{1}H NMR (CD_3OD, 400 MHz) δ 8.06 – 8.01 (m, 1H), 7.57 (td, *J* = 7.4, 1.6 Hz, 1H), 7.54 (td, *J* = 7.4, 1.7 Hz, 1H), 7.13 – 7.09 (m, 1H), 6.88 (s, 1H), 6.84 (d, *J* = 1.6 Hz, 1H), 6.83 – 6.80 (m, 1H), 3.78 (dq, *J* = 14.2, 7.0 Hz, 1H), 3.60 (dq, *J* = 14.2, 7.1 Hz, 1H), 3.54 (t, *J* = 6.2 Hz, 2H), 3.48 (t, *J* = 5.8 Hz, 2H), 3.23 – 3.15 (m, 2H), 2.80 – 2.68 (m, 1H), 2.62 – 2.45 (m, 2H), 2.04 (p, *J* = 6.2 Hz, 2H), 1.93 – 1.85 (m, 2H), 1.90 (s, 3H), 1.89 (s, 3H), 1.82 (dd, *J* = 13.3, 4.4 Hz, 1H), 1.46 (t, *J* = 13.2 Hz, 1H), 1.45 (s, 3H), 1.34 (t, *J* = 7.0 Hz, 3H), 1.30 (s, 3H), 0.98 (d, *J* = 6.5 Hz, 3H); ^{13}C NMR (CD_3OD, 101 MHz) δ 173.8 (C), 166.4 (C), 158.9 (C), 153.6 (C), 153.0 (C), 151.2 (C), 140.9 (C), 139.1 (C), 135.8 (CH), 132.1 (CH), 130.5 (CH), 130.4 (CH), 130.1 (CH), 129.7 (CH), 129.4 (C), 123.8 (C), 123.5 (C), 122.3 (C), 120.6 (C), 111.9 (CH), 58.4 (C), 52.9 (CH_2), 52.2 (CH_2), 46.6 (CH_2), 41.6 (C), 40.8 (CH_2), 31.3 (CH_3), 31.0 (CH_3), 29.5 (CH_3), 28.67 (CH_2), 28.65 (CH_2), 27.5 (CH), 26.1 (CH_3), 22.0 (CH_2), 21.9 (CH_2), 19.4 (CH_3), 14.7 (CH_3); Analytical HPLC: t_R = 14.4 min, 98.7% purity (10–95% MeCN/H_2O, linear gradient, with constant 0.1%

v/v TFA additive; 20 min run; 1 mL/min flow; ESI; positive ion mode; detection at 650 nm); HRMS (ESI) calcd for $C_{37}H_{43}N_2O_2$ $[M+H]^+$ 547.3319, found 547.3319.

Rhodamine 110 and N-Aryl Rhodamine Synthesis via Cross-Coupling (Scheme 11)

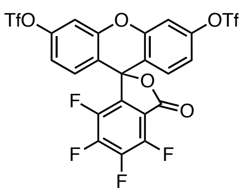

4,5,6,7-Tetrafluoro-fluorescein ditriflate (61): 4,5,6,7-Tetrafluoro-fluorescein[10] (**60**; 1.00 g, 2.47 mmol) was taken up in CH_2Cl_2 (25 mL) and cooled to 0 °C. Pyridine (1.60 mL, 19.79 mmol, 8 eq) and trifluoromethanesulfonic anhydride (1.66 mL, 9.89 mmol, 4 eq) were added, and the reaction was allowed to warm to room temperature overnight (18 h). It was subsequently diluted with water and extracted with CH_2Cl_2 (2×). The combined organic extracts were washed with saturated $CuSO_4$ and brine, dried over anhydrous $MgSO_4$, filtered, and concentrated *in vacuo*. Flash chromatography on silica gel (0–40% EtOAc/hexanes, linear gradient) afforded 1.10 g (67%) of **61** as a white foam. ^{1}H NMR ($CDCl_3$, 400 MHz) δ 7.35 – 7.32 (m, 2H), 7.16 – 7.10 (m, 4H); ^{19}F NMR ($CDCl_3$, 376 MHz) δ -73.07 (s, 6F), -136.01 (td, *J* = 19.8, 10.3 Hz, 1F), -139.59 (ddd, *J* = 20.7, 18.0, 10.3 Hz, 1F), -141.78 (td, *J* = 19.9, 5.1 Hz, 1F), -147.64 (ddd, *J* = 20.2, 18.0, 5.1 Hz, 1F); Analytical HPLC: t_R = 16.2 min, >99% purity (30–95% MeCN/H_2O, linear gradient, with constant 0.1% v/v TFA additive; 20 min run; 1 mL/min flow; ESI; positive ion mode; detection at 280 nm); HRMS (ESI) calcd for $C_{22}H_7F_{10}O_9S_2$ $[M+H]^+$ 668.9366, found 668.9361.

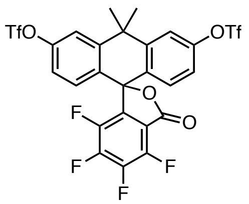

4,5,6,7-Tetrafluoro-carbofluorescein ditriflate (62): To a solution of 4,5,6,7-tetrafluoro-carbofluorescein (**51**; 445 mg, 1.03 mmol) in CH_2Cl_2 (10 mL) were added pyridine (669 µL, 8.27 mmol, 8 eq) and trifluoromethanesulfonic anhydride (696 µL, 4.14 mmol, 4 eq). The reaction was stirred at room temperature for 1 h. It was subsequently diluted with water and extracted with CH_2Cl_2 (2×). The combined organic extracts were washed with saturated $CuSO_4$ and brine, dried over anhydrous $MgSO_4$, filtered, and concentrated *in vacuo*. Flash chromatography on silica gel (0–30% EtOAc/hexanes, linear gradient) afforded 684 mg (95%) of **62** as a white solid. ^{1}H NMR ($CDCl_3$, 400 MHz) δ 7.56 (d, *J* = 2.6 Hz, 2H), 7.19 (dd, *J* = 8.8, 2.5 Hz, 2H), 7.02 (d, *J* = 8.8 Hz, 2H), 1.84 (s, 3H), 1.79 (s, 3H); ^{19}F NMR ($CDCl_3$, 376 MHz) δ -73.10 (s, 6F), -136.52 (td, *J* = 19.9, 10.1 Hz, 1F), -140.09 (ddd, *J* = 20.4, 18.1, 10.1 Hz, 1F), -141.51 (td, *J* = 19.9, 4.8 Hz, 1F), -148.41 (ddd, *J* = 20.5, 18.2, 4.8 Hz, 1F); Analytical HPLC: t_R = 16.4 min, 97.7% purity (30–95% MeCN/H_2O, linear gradient, with constant 0.1% v/v TFA additive; 20 min run; 1 mL/min flow; ESI; positive ion mode; detection at 280 nm); HRMS (EI) calcd for $C_{25}H_{12}F_{10}O_8S_2$ $[M^{\bullet}]^+$ 693.9808, found 693.9820.

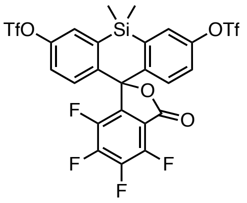

4,5,6,7-Tetrafluoro-Si-fluorescein ditriflate (63): To a solution of 4,5,6,7-tetrafluoro-Si-fluorescein (**55**; 170 mg, 0.381 mmol) in CH_2Cl_2 (5 mL) were added pyridine (246 µL, 3.05 mmol, 8 eq) and trifluoromethanesulfonic anhydride (256 µL, 1.52 mmol, 4 eq). The reaction was stirred at room temperature for 1 h. It was subsequently diluted with water and extracted with CH_2Cl_2 (2×). The combined organic extracts were washed with saturated $CuSO_4$ and brine, dried over anhydrous $MgSO_4$, filtered, and concentrated *in vacuo*. Flash chromatography on silica gel (0–25% EtOAc/hexanes, linear gradient) afforded 261 mg (96%) of **63** as a white foam. ^{1}H NMR ($CDCl_3$, 400 MHz) δ 7.58 (d, J = 2.7 Hz, 2H), 7.27 (dd, J = 8.9, 2.7 Hz, 2H), 7.15 (dd, J = 8.9, 1.4 Hz, 2H), 0.69 (s, 3H), 0.67 (s, 3H); ^{19}F NMR ($CDCl_3$, 376 MHz) δ -73.17 (s, 6F), -135.84 (td, J = 19.8, 10.1 Hz, 1F), -137.58 – -137.80 (m, 1F), -140.68 (ddd, J = 21.0, 18.3, 10.1 Hz, 1F), -148.14 (ddd, J = 20.5, 18.1, 4.9 Hz, 1F); Analytical HPLC: t_R = 16.9 min, 97.8% purity (30–95% MeCN/H_2O, linear gradient, with constant 0.1% v/v TFA additive; 20 min run; 1 mL/min flow; ESI; positive ion mode; detection at 280 nm); HRMS (ESI) calcd for $C_{24}H_{13}F_{10}O_8S_2Si$ $[M+H]^+$ 710.9656, found 710.9660.

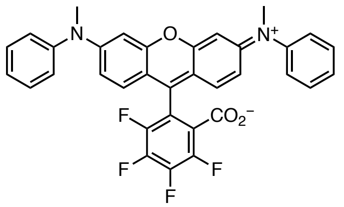

2-(3,6-Bis(methyl(phenyl)amino)xanthylium-9-yl)-3,4,5,6-tetrafluorobenzoate (64): A vial was charged with 4,5,6,7-tetrafluoro-fluorescein ditriflate (**61**; 90 mg, 0.135 mmol), Pd_2dba_3 (12.3 mg, 13.5 µmol, 0.1 eq), XPhos (19.3 mg, 40.4 µmol, 0.3 eq), and Cs_2CO_3 (123 mg, 0.377 mmol, 2.8 eq). The vial was sealed and evacuated/backfilled with nitrogen (3×). Dioxane (1 mL) was added, and the reaction was flushed again with nitrogen (3×). Following the addition of *N*-methylaniline (32.1 µL, 0.296 mmol, 2.2 eq), the reaction was stirred at 80 °C for 4 h. It was then cooled to room temperature, diluted with CH_2Cl_2, deposited onto Celite, and concentrated to dryness. Purification by silica gel chromatography (10–100% EtOAc/hexanes, linear gradient; dry load with Celite) afforded **64** (70.7 mg, 90%) as a pale purple solid. ^{1}H NMR ($CDCl_3$, 400 MHz) δ 7.41 – 7.35 (m, 4H), 7.22 – 7.16 (m, 6H), 6.67 (d, J = 8.8 Hz, 2H), 6.60 (d, J = 2.4 Hz, 2H), 6.52 (dd, J = 8.8, 2.5 Hz, 2H), 3.33 (s, 6H); ^{19}F NMR ($CDCl_3$, 376 MHz) δ -139.02 (td, J = 20.0, 8.7 Hz, 1F), -141.95 (td, J = 20.0, 4.0 Hz, 1F), -143.05 (ddd, J = 20.4, 18.0, 8.6 Hz, 1F), -150.82 – -151.01 (m, 1F); Analytical HPLC: t_R = 11.0 min, >99% purity (30–95% MeCN/H_2O, linear gradient, with constant 0.1% v/v TFA additive; 20 min run; 1 mL/min flow; ESI; positive ion mode; detection at 550 nm); HRMS (ESI) calcd for $C_{34}H_{23}F_4N_2O_3$ $[M+H]^+$ 583.1639, found 583.1631.

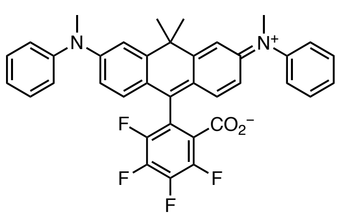

JQ_{645} (65): A vial was charged with 4,5,6,7-tetrafluoro-carbofluorescein ditriflate (**62**; 250 mg, 0.360 mmol), Pd_2dba_3 (33.0 mg, 36.0 μmol, 0.1 eq), XPhos (51.5 mg, 0.108 mmol, 0.3 eq), and Cs_2CO_3 (328 mg, 1.01 mmol, 2.8 eq). The vial was sealed and evacuated/backfilled with nitrogen (3×). Dioxane (2 mL) was added, and the reaction was flushed again with nitrogen (3×). Following the addition of *N*-methylaniline (85.8 μL, 0.792 mmol, 2.2 eq), the reaction was stirred at 80 °C for 8 h. It was then cooled to room temperature, filtered through Celite with CH_2Cl_2, and concentrated to dryness. Purification by silica gel chromatography (0–25% EtOAc/hexanes, linear gradient) afforded **65** (167 mg, 76%) as an off-white solid. ^{1}H NMR ($CDCl_3$, 400 MHz) δ 7.37 – 7.31 (m, 4H), 7.17 – 7.06 (m, 8H), 6.74 (dd, J = 8.8, 2.4 Hz, 2H), 6.69 (d, J = 8.8 Hz, 2H), 3.36 (s, 6H), 1.65 (s, 3H), 1.63 (s, 3H); ^{19}F NMR ($CDCl_3$, 376 MHz) δ -139.31 (td, J = 20.0, 8.6 Hz, 1F), -141.79 (td, J = 20.1, 3.9 Hz, 1F), -143.16 (ddd, J = 21.0, 18.6, 8.7 Hz, 1F), -151.67 (ddd, J = 21.2, 18.2, 3.7 Hz, 1F); Analytical HPLC: t_R = 16.4 min, >99% purity (30–95% MeCN/H_2O, linear gradient, with constant 0.1% v/v TFA additive; 20 min run; 1 mL/min flow; ESI; positive ion mode; detection at 650 nm); HRMS (ESI) calcd for $C_{37}H_{29}F_4N_2O_2$ [M+H]$^+$ 609.2160, found 609.2149.

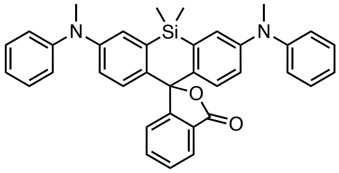

5,5-Dimethyl-3,7-bis(methyl(phenyl)amino)-3'*H*,5*H*-spiro[dibenzo[*b*,*e*]siline-10,1'-isobenzofuran]-3'-one (92): A vial was charged with Si-fluorescein ditriflate[11] (120 mg, 0.188 mmol), Pd_2dba_3 (17.2 mg, 18.8 μmol, 0.1 eq), XPhos (26.9 mg, 56.4 μmol, 0.3 eq), and Cs_2CO_3 (171 mg, 0.526 mmol, 2.8 eq). The vial was sealed and evacuated/backfilled with nitrogen (3×). Dioxane (1.5 mL) was added, and the reaction was flushed again with nitrogen (3×). Following the addition of *N*-methylaniline (44.8 μL, 0.413 mmol, 2.2 eq), the reaction was stirred at 100 °C for 3 h. It was then cooled to room temperature, filtered through Celite with CH_2Cl_2, and concentrated to dryness. Purification by silica gel chromatography (0–40% EtOAc/hexanes, linear gradient) afforded **92** (71.1 mg, 68%) as a white solid. ^{1}H NMR ($CDCl_3$, 400 MHz) δ 7.97 (dt, J = 7.7, 1.0 Hz, 1H), 7.67 (td, J = 7.5, 1.2 Hz, 1H), 7.56 (td, J = 7.5, 0.9 Hz, 1H), 7.37 (dt, J = 7.7, 1.0 Hz, 1H), 7.32 – 7.26 (m, 4H), 7.24 (d, J = 2.6 Hz, 2H), 7.11 – 7.05 (m, 4H), 7.05 – 7.00 (m, 2H), 6.81 (d, J = 8.7 Hz, 2H), 6.76 (dd, J = 8.8, 2.6 Hz, 2H), 3.32 (s, 6H), 0.56 (s, 3H), 0.53 (s, 3H); ^{13}C NMR ($CDCl_3$, 101 MHz) δ 170.5 (C), 153.7 (C), 148.4 (C), 148.2 (C), 137.4 (C), 135.6 (C), 133.8 (CH), 129.5 (CH), 129.1 (CH), 128.1 (CH), 127.1 (C), 126.0 (CH), 124.8 (CH), 122.92 (CH), 122.89 (CH), 122.5 (CH), 119.3 (CH), 91.4 (C), 40.2 (CH_3), 0.4 (CH_3), -1.7 (CH_3); Analytical HPLC: t_R = 15.3 min, >99% purity (50–95% MeCN/H_2O, linear gradient, with constant 0.1% v/v TFA additive; 20 min run; 1 mL/min flow; ESI; positive ion mode; detection at 254 nm); HRMS (ESI) calcd for $C_{36}H_{33}N_2O_2Si$ [M+H]$^+$ 553.2306, found 553.2304.

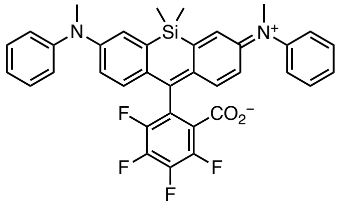

2-(5,5-Dimethyl-3,7-bis(methyl(phenyl)amino)dibenzo[*b*,*e*]silin-10-ylium-10(5*H*)-yl)-3,4,5,6-tetrafluoro-benzoate (66): A vial was charged with 4,5,6,7-tetrafluoro-Si-fluorescein ditriflate (**63**; 175 mg, 0.246 mmol), Pd_2dba_3 (22.6 mg, 24.6 µmol, 0.1 eq), XPhos (35.2 mg, 73.9 µmol, 0.3 eq), and Cs_2CO_3 (225 mg, 0.690 mmol, 2.8 eq). The vial was sealed and evacuated/backfilled with nitrogen (3×). Dioxane (2 mL) was added, and the reaction was flushed again with nitrogen (3×). Following the addition of *N*-methylaniline (58.7 µL, 0.542 mmol, 2.2 eq), the reaction was stirred at 80 °C for 3 h. It was then cooled to room temperature, filtered through Celite with CH_2Cl_2, and concentrated to dryness. Purification by silica gel chromatography (0–25% EtOAc/hexanes, linear gradient) afforded **66** (144 mg, 94%) as an off-white solid. ^{1}H NMR ($CDCl_3$, 400 MHz) δ 7.36 – 7.30 (m, 4H), 7.20 – 7.06 (m, 8H), 6.82 – 6.75 (m, 4H), 3.35 (s, 6H), 0.50 (s, 3H), 0.47 (s, 3H); ^{19}F NMR ($CDCl_3$, 376 MHz) δ -138.60 (td, *J* = 19.8, 3.0 Hz, 1F), -138.76 (td, *J* = 19.4, 7.8 Hz, 1F), -143.54 – -143.71 (m, 1F), -151.30 – -151.47 (m, 1F); Analytical HPLC: t_R = 16.3 min, >99% purity (50–95% MeCN/H_2O, linear gradient, with constant 0.1% v/v TFA additive; 20 min run; 1 mL/min flow; ESI; positive ion mode; detection at 675 nm); HRMS (ESI) calcd for $C_{36}H_{29}F_4N_2O_2Si$ [M+H]$^+$ 625.1929, found 625.1922.

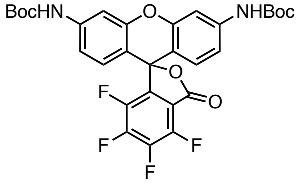

4,5,6,7-Tetrafluoro-rhodamine 110 bis(*tert*-butyl carbamate) (67): A vial was charged with 4,5,6,7-tetrafluoro-fluorescein ditriflate (**61**; 200 mg, 0.299 mmol), *tert*-butyl carbamate (84.1 mg, 0.718 mmol, 2.4 eq), Pd_2dba_3 (27.4 mg, 29.9 µmol, 0.1 eq), XPhos (42.8 mg, 89.8 µmol, 0.3 eq), and Cs_2CO_3 (273 mg, 0.838 mmol, 2.8 eq). The vial was sealed and evacuated/backfilled with nitrogen (3×). Dioxane (2 mL) was added; after flushing the reaction again with nitrogen (3×), it was stirred at 80 °C for 4 h. It was then cooled to room temperature, filtered through Celite with CH_2Cl_2, and evaporated. The residue was purified by silica gel chromatography (0–30% EtOAc/hexanes, linear gradient) to afford **67** (172 mg, 95%) as an off-white solid. ^{1}H NMR ($CDCl_3$, 400 MHz) δ 7.49 (d, *J* = 2.2 Hz, 2H), 7.01 (dd, *J* = 8.7, 2.3 Hz, 2H), 6.82 (d, *J* = 8.7 Hz, 2H), 6.61 (s, 2H), 1.53 (s, 18H); ^{19}F NMR ($CDCl_3$, 376 MHz) δ -138.19 (td, *J* = 19.8, 9.4 Hz, 1F), -141.93 (ddd, *J* = 20.5, 17.6, 9.2 Hz, 1F), -142.12 (td, *J* = 19.8, 4.0 Hz, 1F), -149.98 (ddd, *J* = 20.5, 17.7, 3.9 Hz, 1F); Analytical HPLC: t_R = 15.6 min, >99% purity (30–95% MeCN/H_2O, linear gradient, with constant 0.1% v/v TFA additive; 20 min run; 1 mL/min flow; ESI; positive ion mode; detection at 254 nm); HRMS (ESI) calcd for $C_{30}H_{27}F_4N_2O_7$ [M+H]$^+$ 603.1749, found 603.1750.

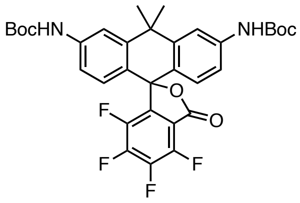

4,5,6,7-Tetrafluoro-carborhodamine 110 bis(*tert*-butyl carbamate) (68): A vial was charged with 4,5,6,7-tetrafluoro-carbofluorescein ditriflate (**62**; 350 mg, 0.504 mmol), *tert*-butyl carbamate (142 mg, 1.21 mmol, 2.4 eq), Pd_2dba_3 (46.1 mg, 50.4 μmol, 0.1 eq), XPhos (72.1 mg, 0.151 mmol, 0.3 eq), and Cs_2CO_3 (460 mg, 1.41 mmol, 2.8 eq). The vial was sealed and evacuated/backfilled with nitrogen (3×). Dioxane (3.5 mL) was added; after flushing the reaction again with nitrogen (3×), it was stirred at 80 °C for 4 h. It was then cooled to room temperature, filtered through Celite with CH_2Cl_2, and evaporated. The residue was purified by silica gel chromatography (0–30% EtOAc/hexanes, linear gradient) to afford **68** (305 mg, 96%) as an off-white solid. ^1H NMR ($CDCl_3$, 400 MHz) δ 7.79 (d, *J* = 2.2 Hz, 2H), 7.11 (dd, *J* = 8.6, 2.2 Hz, 2H), 6.77 (d, *J* = 8.6 Hz, 2H), 6.59 (s, 2H), 1.81 (s, 3H), 1.74 (s, 3H), 1.53 (s, 18H); ^{19}F NMR ($CDCl_3$, 376 MHz) δ -138.65 (td, *J* = 20.0, 9.1 Hz), -141.98 (td, *J* = 20.0, 4.0 Hz), -142.47 (td, *J* = 19.5, 8.8 Hz), -150.82 – -151.01 (m); Analytical HPLC: t_R = 15.7 min, 98.2% purity (30–95% $MeCN/H_2O$, linear gradient, with constant 0.1% v/v TFA additive; 20 min run; 1 mL/min flow; ESI; positive ion mode; detection at 254 nm); HRMS (ESI) calcd for $C_{33}H_{33}F_4N_2O_6$ $[M+H]^+$ 629.2269, found 629.2280.

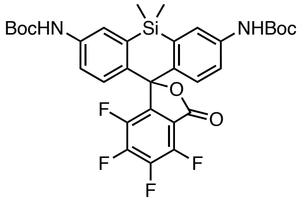

4,5,6,7-Tetrafluoro-Si-rhodamine 110 bis(*tert*-butyl carbamate) (69): A vial was charged with 4,5,6,7-tetrafluoro-Si-fluorescein ditriflate (**63**; 245 mg, 0.345 mmol), *tert*-butyl carbamate (96.9 mg, 0.828 mmol, 2.4 eq), Pd_2dba_3 (31.6 mg, 34.5 μmol, 0.1 eq), XPhos (49.3 mg, 0.103 mmol, 0.3 eq), and Cs_2CO_3 (315 mg, 0.965 mmol, 2.8 eq). The vial was sealed and evacuated/backfilled with nitrogen (3×). Dioxane (3 mL) was added; after flushing the reaction again with nitrogen (3×), it was stirred at 80 °C for 5 h. It was then cooled to room temperature, filtered through Celite with CH_2Cl_2, and evaporated. The residue was purified by silica gel chromatography (0–30% EtOAc/hexanes, linear gradient) to afford **69** (212 mg, 95%) as an off-white solid. ^1H NMR ($CDCl_3$, 400 MHz) δ 7.70 (d, *J* = 2.5 Hz, 2H), 7.30 (dd, *J* = 8.7, 2.5 Hz, 2H), 6.90 (d, *J* = 8.7 Hz, 2H), 6.56 (s, 2H), 1.52 (s, 18H), 0.60 (s, 3H), 0.56 (s, 3H); ^{19}F NMR ($CDCl_3$, 376 MHz) δ -138.04 (td, *J* = 20.0, 8.8 Hz, 1F), -139.27 – -139.47 (m, 1F), -142.79 (td, *J* = 19.4, 8.8 Hz, 1F), -150.67 – -150.88 (m, 1F); Analytical HPLC: t_R = 16.1 min, 98.4% purity (30–95% $MeCN/H_2O$, linear gradient, with constant 0.1% v/v TFA additive; 20 min run; 1 mL/min flow; ESI; positive ion mode; detection at 254 nm); HRMS (ESI) calcd for $C_{32}H_{33}F_4N_2O_6Si$ $[M+H]^+$ 645.2039, found 645.2049.

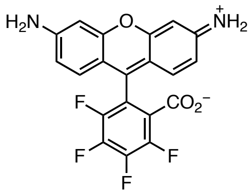

4,5,6,7-Tetrafluoro-rhodamine 110 (FRh$_{110}$, 70): 4,5,6,7-Tetrafluoro-rhodamine 110 bis(*tert*-butyl carbamate) (**67**; 100 mg, 0.166 mmol) was taken up in CH_2Cl_2 (5 mL), and trifluoroacetic acid (1 mL) was added. The reaction was stirred at room temperature for 3 h. It was then diluted with toluene (5 mL) and concentrated to dryness. Purification by reverse phase HPLC (10–40% MeCN/H_2O, linear gradient, with constant 0.1% v/v TFA additive) yielded 82.6 mg (96%, TFA salt) of **70** as a red solid. ^{1}H NMR (CD_3OD, 400 MHz) δ 7.29 (dd, J = 9.2, 0.9 Hz, 2H), 6.90 (dd, J = 9.2, 2.1 Hz, 2H), 6.83 (d, J = 2.1 Hz, 2H); ^{19}F NMR (CD_3OD, 376 MHz) δ -75.77 (s, 3F), -135.40 (ddd, J = 20.4, 11.9, 8.1 Hz, 1F), -137.30 (ddd, J = 21.7, 11.9, 5.1 Hz, 1F), -150.52 (ddd, J = 21.8, 18.9, 7.9 Hz, 1F), -151.93 (ddd, J = 21.1, 19.0, 5.3 Hz, 1F); Analytical HPLC: t_R = 9.5 min, >99% purity (10–95% MeCN/H_2O, linear gradient, with constant 0.1% v/v TFA additive; 20 min run; 1 mL/min flow; ESI; positive ion mode; detection at 525 nm); HRMS (ESI) calcd for $C_{20}H_{11}F_4N_2O_3$ $[M+H]^+$ 403.0700, found 403.0703.

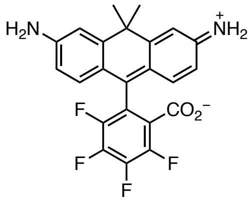

4,5,6,7-Tetrafluoro-carborhodamine 110 (FCRh$_{110}$, 71): 4,5,6,7-Tetrafluoro-carborhodamine 110 bis(*tert*-butyl carbamate) (**68**; 90 mg, 0.143 mmol) was taken up in CH_2Cl_2 (5 mL), and trifluoroacetic acid (1 mL) was added. The reaction was stirred at room temperature for 4 h. It was then diluted with toluene (5 mL) and concentrated to dryness. The residue was taken up in saturated $NaHCO_3$ and extracted with EtOAc (2×). The combined organic extracts were washed with brine, dried over anhydrous $MgSO_4$, filtered, deposited onto Celite, and concentrated to dryness. Flash chromatography (10–100% EtOAc/hexanes, linear gradient; dry load with Celite) afforded 52.2 mg (85%) of **71** as a dark purple solid. ^{1}H NMR (CD_3OD, 400 MHz) δ 7.09 (d, J = 2.2 Hz, 2H), 7.06 (dd, J = 8.9, 0.8 Hz, 2H), 6.62 (dd, J = 8.9, 2.2 Hz, 2H), 1.72 (s, 3H), 1.66 (s, 3H); ^{19}F NMR (CD_3OD, 376 MHz) δ -140.30 (ddd, J = 21.2, 13.8, 3.5 Hz, 1F), -141.26 (ddd, J = 21.9, 13.9, 4.6 Hz, 1F), -154.43 (ddd, J = 22.4, 18.8, 3.5 Hz, 1F), -155.33 – -155.56 (m, 1F); Analytical HPLC: t_R = 9.9 min, >99% purity (10–95% MeCN/H_2O, linear gradient, with constant 0.1% v/v TFA additive; 20 min run; 1 mL/min flow; ESI; positive ion mode; detection at 575 nm); HRMS (ESI) calcd for $C_{23}H_{17}F_4N_2O_2$ $[M+H]^+$ 429.1221, found 429.1223.

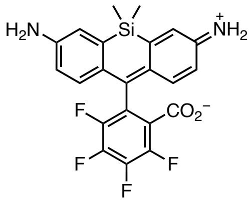

4,5,6,7-Tetrafluoro-Si-rhodamine 110 (FSiRh$_{110}$, 72): 4,5,6,7-Tetrafluoro-Si-rhodamine 110 bis(*tert*-butyl carbamate) (**69**; 50 mg, 77.6 µmol) was taken up in CH_2Cl_2 (4 mL), and trifluoroacetic acid (0.8 mL) was added. The reaction was stirred at room temperature for 4 h. It was then diluted with toluene (4 mL) and concentrated to dryness. The residue was taken up in saturated $NaHCO_3$ and extracted with EtOAc (2×). The combined organic extracts were washed with brine, dried over anhydrous $MgSO_4$, filtered, deposited onto Celite, and concentrated to dryness. Flash chromatography (10–100% EtOAc/hexanes, linear gradient; dry load with Celite) afforded 32.2 mg (93%) of **72** as a light blue solid. ^{1}H NMR ($CDCl_3$, 400 MHz) δ 6.94 (d, *J* = 2.6 Hz, 2H), 6.72 (dd, *J* = 8.5, 1.2 Hz, 2H), 6.58 (dd, *J* = 8.5, 2.7 Hz, 2H), 3.82 (s, 4H), 0.54 (s, 3H), 0.52 (s, 3H); ^{19}F NMR ($CDCl_3$, 376 MHz) δ -138.90 (td, *J* = 19.8, 8.4 Hz, 1F), -139.14 (td, *J* = 19.9, 3.4 Hz, 1F), -143.59 (ddd, *J* = 20.5, 18.3, 8.3 Hz, 1F), -151.59 (ddd, *J* = 20.2, 18.3, 3.8 Hz, 1F); Analytical HPLC: t_R = 9.9 min, 98.6% purity (10–95% MeCN/H_2O, linear gradient, with constant 0.1% v/v TFA additive; 20 min run; 1 mL/min flow; ESI; positive ion mode; detection at 625 nm); HRMS (ESI) calcd for $C_{22}H_{17}F_4N_2O_2Si$ [M+H]$^+$ 445.0990, found 445.0998.

MAC SUBSTITUTION OF 4,5,6,7-TETRAFLUOROXANTHENES

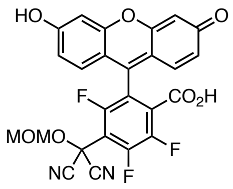

4,5,7-Trifluoro-6-(MOM-MAC)-fluorescein (60$_{MAC}$): 4,5,6,7-Tetrafluoro-fluorescein[10] (**60**; 100 mg, 0.247 mmol) and 2-(methoxymethoxy)malononitrile (**3**; 31.2 mg, 0.247 mmol, 1 eq) were combined in DMF (5 mL), and DIEA (129 µL, 0.742 mmol, 3 eq) was added. After stirring the reaction at room temperature for 18 h, it was evaporated to dryness and purified by reverse phase HPLC (30–60% MeCN/H_2O, linear gradient, with constant 0.1% v/v TFA additive). The pooled product fractions were partially concentrated to remove MeCN and extracted with CH_2Cl_2 (3×). The organic extracts were dried over anhydrous $MgSO_4$, filtered, and evaporated to provide **60$_{MAC}$** (69 mg, 55%) as a yellow-orange solid. ^{1}H NMR (DMSO-d_6, 400 MHz) δ 10.26 (s, 2H), 7.05 (d, J = 8.7 Hz, 2H), 6.70 (d, J = 2.3 Hz, 2H), 6.60 (dd, J = 8.7, 2.4 Hz, 2H), 5.11 (s, 2H), 3.34 (s, 3H); ^{19}F NMR (DMSO-d_6, 376 MHz) δ -118.57 (d, J = 21.4 Hz, 1F), -129.27 (d, J = 21.1 Hz, 1F), -140.99 (t, J = 21.3 Hz, 1F); Analytical HPLC: t_R = 12.9 min, >99% purity (10–95% MeCN/H_2O, linear gradient, with constant 0.1% v/v TFA additive; 20 min run; 1 mL/min flow; ESI; positive ion mode; detection at 254 nm); HRMS (ESI) calcd for $C_{25}H_{14}F_3N_2O_7$ $[M+H]^+$ 511.0748, found 511.0746.

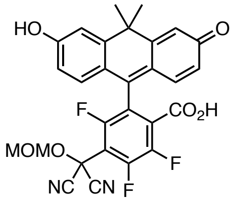

4,5,7-Trifluoro-6-(MOM-MAC)-carbofluorescein (51$_{MAC}$): 4,5,6,7-Tetrafluoro-carbofluorescein (**51**; 125 mg, 0.290 mmol) and 2-(methoxymethoxy)malononitrile (**3**; 36.6 mg, 0.290 mmol, 1 eq) were combined in DMF (4 mL), and DIEA (152 µL, 0.871 mmol, 3 eq) was added. After stirring the reaction at room temperature for 2 h, it was diluted with 10% citric acid and extracted with EtOAc (2×). The combined organic extracts were washed with water and brine, dried over anhydrous $MgSO_4$, filtered, and concentrated *in vacuo*. Flash chromatography on silica gel (0–75% EtOAc/toluene, linear gradient) afforded 92 mg (59%) of **51$_{MAC}$** as an orange solid. ^{1}H NMR (DMSO-d_6, 400 MHz) δ 9.83 (s, 2H), 7.10 (d, J = 2.5 Hz, 2H), 6.87 (d, J = 8.6 Hz, 2H), 6.66 (dd, J = 8.7, 2.4 Hz, 2H), 5.10 (s, 2H), 3.32 (s, 3H), 1.68 (s, 3H), 1.60 (s, 3H); ^{19}F NMR (DMSO-d_6, 376 MHz) δ -117.77 (d, J = 21.4 Hz, 1F), -129.80 (d, J = 21.4 Hz, 1F), -141.28 (t, J = 21.5 Hz, 1F); Analytical HPLC: t_R = 13.1 min, 99.0% purity (10–95% MeCN/H_2O, linear gradient, with constant 0.1% v/v TFA additive; 20 min run; 1 mL/min flow; ESI; positive ion mode; detection at 254 nm); HRMS (ESI) calcd for $C_{28}H_{20}F_3N_2O_6$ $[M+H]^+$ 537.1268, found 537.1268.

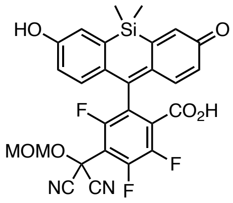

4,5,7-Trifluoro-6-(MOM-MAC)-Si-fluorescein (55$_{MAC}$): 4,5,6,7-Tetrafluoro-Si-fluorescein (**55**; 45 mg, 0.101 mmol) and 2-(methoxymethoxy)malononitrile (**3**; 12.7 mg, 0.101 mmol, 1 eq) were combined in DMF (2 mL), and DIEA (52.7 µL, 0.302 mmol, 3 eq) was added. After stirring the reaction at room temperature for 3 h, it was diluted with 10% citric acid and extracted with EtOAc (2×). The combined organic extracts were washed with water and brine, dried over anhydrous $MgSO_4$, filtered, and concentrated *in vacuo*. Flash chromatography on silica gel (0–60% EtOAc/toluene, linear gradient) afforded 31.6 mg (57%) of **55$_{MAC}$** as an off-white solid. ^{1}H NMR ($CDCl_3$, 400 MHz) δ 7.14 (d, J = 2.6 Hz, 2H), 6.82 (d, J = 8.8 Hz, 2H), 6.78 (dd, J = 8.7, 2.6 Hz, 2H), 5.17 (s, 2H), 5.01 (s, 2H), 3.52 (s, 3H), 0.57 (s, 3H), 0.54 (s, 3H); ^{19}F NMR ($CDCl_3$, 376 MHz) δ -113.71 (d, J = 22.6 Hz, 1F), -126.48 (d, J = 19.9 Hz, 1F), -138.58 (dd, J = 22.7, 20.1 Hz, 1F); Analytical HPLC: t_R = 13.5 min, >99% purity (10–95% MeCN/H_2O, linear gradient, with constant 0.1% v/v TFA additive; 20 min run; 1 mL/min flow; ESI; positive ion mode; detection at 254 nm); HRMS (ESI) calcd for $C_{27}H_{20}F_3N_2O_6Si$ [M+H]$^+$ 553.1037, found 553.1039.

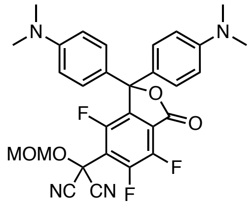

6-(MOM-MAC)-FMGL (49$_{MAC}$): 4,5,6,7-Tetrafluoro-MGL (**49**; 300 mg, 0.675 mmol) and 2-(methoxymethoxy)-malononitrile (**3**; 85.1 mg, 0.675 mmol, 1 eq) were combined in DMF (10 mL), and DIEA (235 µL, 1.35 mmol, 2 eq) was added. After stirring the reaction at room temperature for 2 h, it was concentrated *in vacuo* and purified by flash chromatography on silica gel (0–50% EtOAc/hexanes, linear gradient) to provide **49$_{MAC}$** (225 mg, 61%) as a yellow foam. ^{1}H NMR ($CDCl_3$, 400 MHz) δ 7.16 – 7.09 (m, 4H), 6.65 (d, J = 9.0 Hz, 4H), 5.18 (s, 2H), 3.53 (s, 3H), 2.97 (s, 12H); ^{19}F NMR ($CDCl_3$, 376 MHz) δ -112.40 (d, J = 22.4 Hz, 1F), -127.57 (d, J = 20.4 Hz, 1F), -139.50 (dd, J = 22.5, 20.3 Hz, 1F); Analytical HPLC: t_R = 13.3 min, >99% purity (10–95% MeCN/H_2O, linear gradient, with constant 0.1% v/v TFA additive; 20 min run; 1 mL/min flow; ESI; positive ion mode; detection at 280 nm); HRMS (ESI) calcd for $C_{29}H_{26}F_3N_4O_4$ [M+H]$^+$ 551.1901, found 551.1910.

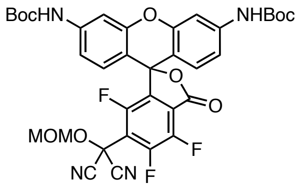

4,5,7-Trifluoro-6-(MOM-MAC)-rhodamine 110 bis(*tert*-butyl carbamate) ($\mathbf{67_{MAC}}$): 4,5,6,7-Tetrafluoro-rhodamine 110 bis(*tert*-butyl carbamate) (**67**; 300 mg, 0.498 mmol) and 2-(methoxymethoxy)malononitrile (**3**; 62.8 mg, 0.498 mmol, 1 eq) were combined in DMF (5 mL), and DIEA (173 µL, 0.996 mmol, 2 eq) was added. After stirring the reaction at room temperature for 2 h, it was concentrated *in vacuo* and purified by silica gel chromatography (0–50% EtOAc/hexanes, linear gradient) to yield 183 mg (52%) of $\mathbf{67_{MAC}}$ as an off-white solid. ^{1}H NMR ($CDCl_3$, 400 MHz) δ 7.48 (d, J = 2.2 Hz, 2H), 7.06 (dd, J = 8.7, 2.2 Hz, 2H), 6.82 (d, J = 8.6 Hz, 2H), 6.63 (s, 2H), 5.13 (s, 2H), 3.51 (s, 3H), 1.53 (s, 18H); ^{19}F NMR ($CDCl_3$, 376 MHz) δ -116.95 (d, J = 22.9 Hz, 1F), -125.48 (d, J = 19.9 Hz, 1F), -138.65 (dd, J = 23.0, 20.2 Hz, 1F); Analytical HPLC: t_R = 15.2 min, >99% purity (30–95% MeCN/H_2O, linear gradient, with constant 0.1% v/v TFA additive; 20 min run; 1 mL/min flow; ESI; positive ion mode; detection at 254 nm); HRMS (ESI) calcd for $C_{35}H_{32}F_3N_4O_9$ $[M+H]^+$ 709.2116, found 709.2130.

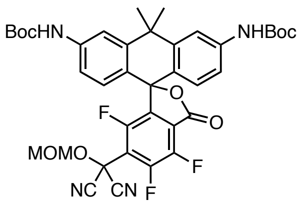

4,5,7-Trifluoro-6-(MOM-MAC)-carborhodamine 110 bis(*tert*-butyl carbamate) ($\mathbf{68_{MAC}}$): 4,5,6,7-Tetrafluoro-carborhodamine 110 bis(*tert*-butyl carbamate) (**68**; 180 mg, 0.286 mmol) and 2-(methoxymethoxy)malononitrile (**3**; 36.1 mg, 0.286 mmol, 1 eq) were combined in DMF (3 mL), and DIEA (99.7 µL, 0.573 mmol, 2 eq) was added. After stirring the reaction at room temperature for 2 h, it was concentrated *in vacuo* and purified by silica gel chromatography (0–50% EtOAc/hexanes, linear gradient) to yield 107 mg (51%) of $\mathbf{68_{MAC}}$ as an off-white solid. ^{1}H NMR ($CDCl_3$, 400 MHz) δ 7.78 (d, J = 2.2 Hz, 2H), 7.16 (dd, J = 8.6, 2.2 Hz, 2H), 6.75 (d, J = 8.6 Hz, 2H), 6.61 (s, 2H), 5.12 (s, 2H), 3.50 (s, 3H), 1.81 (s, 3H), 1.75 (s, 3H), 1.53 (s, 18H); ^{19}F NMR ($CDCl_3$, 376 MHz) δ -116.84 (d, J = 22.7 Hz, 1F), -126.32 (d, J = 19.7 Hz, 1F), -139.19 (dd, J = 22.9, 20.1 Hz, 1F); Analytical HPLC: t_R = 15.2 min, >99% purity (30–95% MeCN/H_2O, linear gradient, with constant 0.1% v/v TFA additive; 20 min run; 1 mL/min flow; ESI; positive ion mode; detection at 254 nm); HRMS (ESI) calcd for $C_{38}H_{38}F_3N_4O_8$ $[M+H]^+$ 735.2636, found 735.2641.

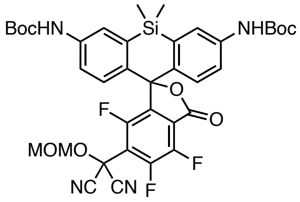

4,5,7-Trifluoro-6-(MOM-MAC)-Si-rhodamine 110 bis(*tert*-butyl carbamate) ($\mathbf{69_{MAC}}$): 4,5,6,7-Tetrafluoro-Si-rhodamine 110 bis(*tert*-butyl carbamate) (**69**; 150 mg, 0.233 mmol) and 2-(methoxymethoxy)malononitrile (**3**; 29.3

mg, 0.233 mmol, 1 eq) were combined in DMF (3 mL), and DIEA (81.1 µL, 0.465 mmol, 2 eq) was added. After stirring the reaction at room temperature for 2 h, it was concentrated *in vacuo* and purified by silica gel chromatography (0–50% EtOAc/hexanes, linear gradient) to yield 89 mg (51%) of **69_{MAC}** as an off-white solid. ^{1}H NMR ($CDCl_3$, 400 MHz) δ 7.68 (d, *J* = 2.5 Hz, 2H), 7.36 (dd, *J* = 8.8, 2.5 Hz, 2H), 6.88 (d, *J* = 8.7 Hz, 2H), 6.58 (s, 2H), 5.16 (s, 2H), 3.52 (s, 3H), 1.52 (s, 18H), 0.60 (s, 3H), 0.56 (s, 3H); ^{19}F NMR ($CDCl_3$, 376 MHz) δ -114.17 (d, *J* = 22.5 Hz, 1F), -126.44 (d, *J* = 20.0 Hz, 1F), -138.55 (t, *J* = 21.4 Hz, 1F); Analytical HPLC: t_R = 15.7 min, >99% purity (30–95% $MeCN/H_2O$, linear gradient, with constant 0.1% v/v TFA additive; 20 min run; 1 mL/min flow; ESI; positive ion mode; detection at 254 nm); HRMS (ESI) calcd for $C_{37}H_{38}F_3N_4O_8Si$ $[M+H]^+$ 751.2406, found 751.2413.

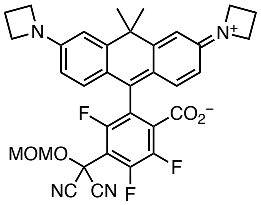

6-(MOM-MAC)-JF_{632} (44_{MAC}): JF_{632} (**44**; 45.0 mg, 88.5 µmol) and 2-(methoxymethoxy)malononitrile (**3**; 11.2 mg, 88.5 µmol, 1 eq) were combined in DMF (2 mL), and DIEA (30.8 µL, 0.177 mmol, 2 eq) was added. After stirring the reaction at room temperature for 3 h, it was concentrated *in vacuo* and purified by reverse phase HPLC (30–50% $MeCN/H_2O$, linear gradient, with constant 0.1% v/v TFA additive). The pooled HPLC product fractions were partially concentrated to remove MeCN, diluted with saturated $NaHCO_3$, and extracted with CH_2Cl_2 (2×). The organic extracts were dried over anhydrous $MgSO_4$, filtered, and evaporated to yield 23.5 mg (43%) of **44_{MAC}** as a blue solid. ^{1}H NMR ($CDCl_3$, 400 MHz) δ 6.63 (d, *J* = 8.6 Hz, 2H), 6.55 (d, *J* = 2.3 Hz, 2H), 6.28 (dd, *J* = 8.6, 2.4 Hz, 2H), 5.11 (s, 2H), 3.95 (t, *J* = 7.3 Hz, 8H), 3.50 (s, 3H), 2.40 (p, *J* = 7.2 Hz, 4H), 1.75 (s, 3H), 1.70 (s, 3H); ^{19}F NMR ($CDCl_3$, 376 MHz) δ -116.34 (d, *J* = 22.7 Hz, 1F), -127.69 (d, *J* = 20.0 Hz, 1F), -140.39 (dd, *J* = 22.8, 20.1 Hz, 1F); Analytical HPLC: t_R = 12.5 min, >99% purity (10–95% $MeCN/H_2O$, linear gradient, with constant 0.1% v/v TFA additive; 20 min run; 1 mL/min flow; ESI; positive ion mode; detection at 625 nm); HRMS (ESI) calcd for $C_{34}H_{30}F_3N_4O_4$ $[M+H]^+$ 615.2214, found 615.2207.

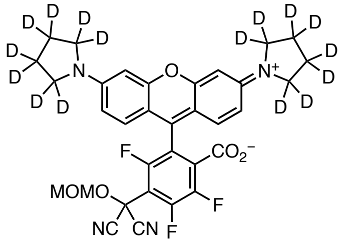

6-(MOM-MAC)-JFX_{576} (8_{MAC}): JFX_{576} (**8**; 250 mg, 0.475 mmol) and 2-(methoxymethoxy)malononitrile (**3**; 59.9 mg, 0.475 mmol, 1 eq) were combined in DMF (9 mL), and DIEA (165 µL, 0.950 mmol, 2 eq) was added. After stirring the reaction at room temperature for 2 h, it was concentrated *in vacuo* and purified by silica gel chromatography (0–15% $MeOH/CH_2Cl_2$, linear gradient, with constant 1% v/v AcOH additive) to yield **8_{MAC}** as a dark purple solid (188 mg, 57%, acetate salt). ^{1}H NMR (CD_3OD, 400 MHz) δ 7.32 (dd, *J* = 9.4, 0.8 Hz, 2H), 6.94 (dd, *J* = 9.4, 2.3 Hz,

2H), 6.75 (d, *J* = 2.3 Hz, 2H), 5.20 (s, 2H), 3.53 (s, 3H); ^{19}F NMR (CD_3OD, 376 MHz) δ -112.06 (d, *J* = 14.9 Hz, 1F), -128.39 (d, *J* = 21.8 Hz, 1F), -141.73 (dd, *J* = 21.7, 14.9 Hz, 1F); Analytical HPLC: t_R = 12.7 min, >99% purity (10–95% MeCN/H_2O, linear gradient, with constant 0.1% v/v TFA additive; 20 min run; 1 mL/min flow; ESI; positive ion mode; detection at 575 nm); HRMS (ESI) calcd for $C_{33}H_{12}D_{16}F_3N_4O_5$ $[M+H]^+$ 633.3011, found 633.3006.

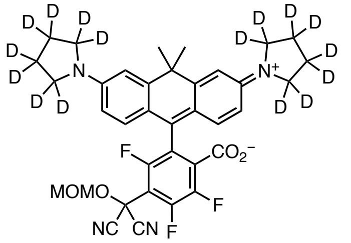

6-(MOM-MAC)-JFX$_{637}$ (40$_{MAC}$): JFX$_{637}$ (**40**; 40 mg, 72.4 μmol) and 2-(methoxymethoxy)malononitrile (**3**; 9.1 mg, 72.4 μmol, 1 eq) were combined in DMF (2 mL), and DIEA (25.2 μL, 0.145 mmol, 2 eq) was added. After stirring the reaction at room temperature for 1 h, it was concentrated *in vacuo* and purified by reverse phase HPLC (30–50% MeCN/H_2O, linear gradient, with constant 0.1% v/v TFA additive). The pooled HPLC product fractions were partially concentrated to remove MeCN, diluted with saturated $NaHCO_3$, and extracted with CH_2Cl_2 (2×). The organic extracts were dried over anhydrous $MgSO_4$, filtered, and evaporated to yield 19.2 mg (40%) of **40$_{MAC}$** as a blue solid. ^{1}H NMR (CD_3OD, 400 MHz) δ 7.20 (dd, *J* = 9.3, 0.9 Hz, 2H), 7.08 (d, *J* = 2.4 Hz, 2H), 6.72 (dd, *J* = 9.3, 2.4 Hz, 2H), 5.19 (s, 2H), 3.53 (s, 3H), 1.80 (s, 3H), 1.74 (s, 3H); ^{19}F NMR (CD_3OD, 376 MHz) δ -112.49 (d, *J* = 14.6 Hz, 1F), -129.79 (d, *J* = 22.3 Hz, 1F), -142.26 (dd, *J* = 21.8, 14.9 Hz, 1F); Analytical HPLC: t_R = 13.0 min, >99% purity (10–95% MeCN/H_2O, linear gradient, with constant 0.1% v/v TFA additive; 20 min run; 1 mL/min flow; ESI; positive ion mode; detection at 650 nm); HRMS (ESI) calcd for $C_{36}H_{18}D_{16}F_3N_4O_4$ $[M+H]^+$ 659.3531, found 659.3522.

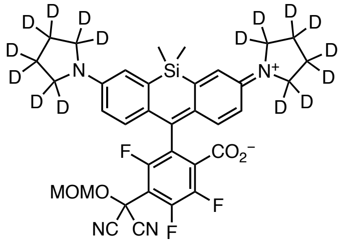

6-(MOM-MAC)-JFX$_{673}$ (25$_{MAC}$): JFX$_{673}$ (**25**; 300 mg, 0.527 mmol) and 2-(methoxymethoxy)malononitrile (**3**; 66.5 mg, 0.527 mmol, 1 eq) were combined in DMF (7 mL), and DIEA (184 μL, 1.05 mmol, 2 eq) was added. After stirring the reaction at room temperature for 2 h, it was concentrated *in vacuo* and purified by silica gel chromatography (10–100% EtOAc/hexanes, linear gradient) to yield 166 mg (47%) of **25$_{MAC}$** as a blue-green solid. ^{1}H NMR ($CDCl_3$, 400 MHz) δ 6.77 (d, *J* = 2.7 Hz, 2H), 6.73 (dd, *J* = 8.8, 1.1 Hz, 2H), 6.46 (dd, *J* = 8.8, 2.8 Hz, 2H), 5.16 (s, 2H), 3.53 (s, 3H), 0.57 (s, 3H), 0.55 (s, 3H); ^{19}F NMR ($CDCl_3$, 376 MHz) δ -113.90 (d, *J* = 22.5 Hz, 1F), -128.13 (d, *J* = 20.2 Hz, 1F), -140.21 (dd, *J* = 22.7, 20.2 Hz, 1F); Analytical HPLC: t_R = 13.2 min, 97.5% purity (10–95% MeCN/H_2O, linear gradient, with constant 0.1% v/v TFA additive; 20 min run; 1 mL/min flow; ESI; positive ion mode; detection at 675 nm); HRMS (ESI) calcd for $C_{35}H_{18}D_{16}F_3N_4O_4Si$ $[M+H]^+$ 675.3300, found 675.3302.

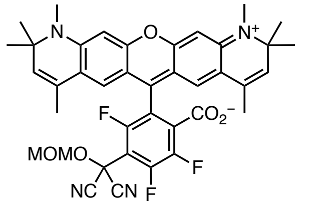

4-(Dicyano(methoxymethoxy)methyl)-2,3,5-trifluoro-6-(1,2,2,4,8,10,10,11-octamethyl-1,2,10,11-tetrahydro-pyrano[3,2-*g*:5,6-*g'*]diquinolin-13-ium-6-yl)benzoate (11$_{\mathbf{MAC}}$): Rhodamine **11** (100 mg, 0.169 mmol) and 2-(methoxymethoxy)malononitrile (**3**; 21.4 mg, 0.169 mmol, 1 eq) were combined in DMF (4 mL), and DIEA (59.0 µL, 0.339 mmol, 2 eq) was added. After stirring the reaction at room temperature for 4 h, it was evaporated to dryness. Flash chromatography on silica gel (0–10% MeOH/CH_2Cl_2, linear gradient, with constant 1% v/v AcOH additive) afforded **11$_{\mathbf{MAC}}$** as a dark blue solid (93.8 mg, 73%, acetate salt). ^{1}H NMR (CD_3OD, 400 MHz) δ 6.94 (d, J = 1.1 Hz, 2H), 6.81 (s, 2H), 5.68 (q, J = 1.4 Hz, 2H), 5.19 (s, 2H), 3.51 (s, 3H), 3.20 (s, 6H), 1.94 (d, J = 1.4 Hz, 6H), 1.52 (s, 6H), 1.51 (s, 6H); ^{19}F NMR (CD_3OD, 376 MHz) δ -111.06 (d, J = 14.9 Hz, 1F), -127.94 (d, J = 21.9 Hz, 1F), -140.94 (dd, J = 21.8, 14.9 Hz, 1F); Analytical HPLC: t_R = 14.2 min, >99% purity (10–95% MeCN/H_2O, linear gradient, with constant 0.1% v/v TFA additive; 20 min run; 1 mL/min flow; ESI; positive ion mode; detection at 600 nm); HRMS (ESI) calcd for $C_{39}H_{36}F_3N_4O_5$ [M+H]$^+$ 697.2632, found 697.2622.

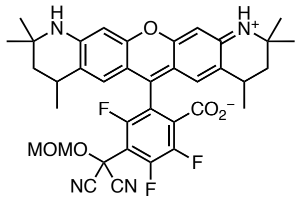

6-(MOM-MAC)-JF$_{563}$ (18$_{\mathbf{MAC}}$): JF$_{563}$ (**18**; 50 mg, 88.2 µmol) and 2-(methoxymethoxy)malononitrile (**3**; 11.1 mg, 88.2 µmol, 1 eq) were combined in DMSO (5 mL), and DIEA (30.7 µL, 0.176 mmol, 2 eq) was added. The resulting dark red solution was stirred at room temperature for 4 h. A second portion of 2-(methoxymethoxy)malononitrile (**3**; 2.8 mg, 22.1 µmol, 0.25 eq) was added, and stirring was continued for an additional 2 h at room temperature. It was subsequently diluted with water and extracted with CH_2Cl_2 (2×). The combined organic extracts were dried over anhydrous $MgSO_4$, filtered, and concentrated *in vacuo*. Purification by silica gel chromatography (0–20% MeOH/CH_2Cl_2, linear gradient, with constant 1% v/v AcOH additive) provided 30.5 mg (47%, acetate salt) of **18$_{\mathbf{MAC}}$** as a dark red-purple solid (mixture of diastereomers). ^{1}H NMR (CD_3OD, 400 MHz) δ 7.16 – 7.09 (m, 2H), 6.64 – 6.59 (m, 2H), 5.21 – 5.18 (m, 2H), 3.55 – 3.50 (m, 3H), 3.00 – 2.85 (m, 2H), 1.91 – 1.84 (m, 2H), 1.49 – 1.23 (m, 20H); ^{19}F NMR (CD_3OD, 376 MHz) δ -111.22 – -111.37 (m, 1F), -128.02 – -128.46 (m, 1F), -140.92 – -141.30 (m, 1F); Analytical HPLC: t_R (three isomers) = 13.2 min, 13.4 min, 13.6 min; 95.1% total purity (10–95% MeCN/H_2O, linear gradient, with constant 0.1% v/v TFA additive; 20 min run; 1 mL/min flow; ESI; positive ion mode; detection at 550 nm); HRMS (ESI) calcd for $C_{37}H_{36}F_3N_4O_5$ [M+H]$^+$ 673.2632, found 673.2623.

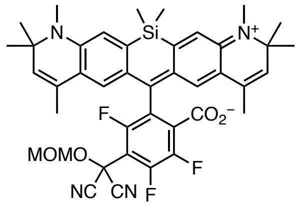

2-(1,2,2,4,8,10,10,11,13,13-decamethyl-2,10,11,13-Tetrahydrosilino[3,2-*g*:5,6-*g'*]diquinolin-6-ylium-6(1*H*)-yl)-4-(dicyano(methoxymethoxy)methyl)-3,5,6-trifluorobenzoate (38$_{MAC}$): Si-rhodamine **38** (425 mg, 0.672 mmol) and 2-(methoxymethoxy)malononitrile (**3**; 84.7 mg, 0.672 mmol, 1 eq) were combined in DMF (13 mL); DIEA (234 µL, 1.34 mmol, 2 eq) was added, and the reaction was stirred at room temperature for 1 h. LC/MS analysis indicated that the reaction was incomplete, so additional 2-(methoxymethoxy)malononitrile (**3**; 25.4 mg, 0.201 mmol, 1 eq) was added. After stirring the reaction for another 1 h at room temperature, it was concentrated *in vacuo* and purified by silica gel chromatography (10–100% EtOAc/hexanes, linear gradient) to yield 152 mg (31%) of **38$_{MAC}$** as a green solid. ^{1}H NMR ($CDCl_3$, 400 MHz) δ 6.69 (s, 2H), 6.46 (s, 2H), 5.27 (q, *J* = 1.4 Hz, 2H), 5.16 (s, 2H), 3.52 (s, 3H), 2.87 (s, 6H), 1.75 (d, *J* = 1.5 Hz, 6H), 1.33 (s, 6H), 1.30 (s, 6H), 0.56 (s, 3H), 0.55 (s, 3H); ^{19}F NMR ($CDCl_3$, 376 MHz) δ -112.08 (d, *J* = 22.4 Hz, 1F), -128.21 (d, *J* = 19.7 Hz, 1F), -139.61 (dd, *J* = 22.5, 20.3 Hz, 1F); Analytical HPLC: t_R = 11.5 min, 98.0% purity (30–95% MeCN/H_2O, linear gradient, with constant 0.1% v/v TFA additive; 20 min run; 1 mL/min flow; ESI; positive ion mode; detection at 750 nm); HRMS (ESI) calcd for $C_{41}H_{42}F_3N_4O_4Si$ $[M+H]^+$ 739.2922, found 739.2917.

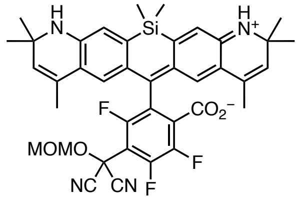

4-(Dicyano(methoxymethoxy)methyl)-2,3,5-trifluoro-6-(2,2,4,8,10,10,13,13-octamethyl-2,10,11,13-tetrahydro-silino[3,2-*g*:5,6-*g'*]diquinolin-6-ylium-6(1*H*)-yl)benzoate (36$_{MAC}$): Si-rhodamine **36** (150 mg, 0.248 mmol) and 2-(methoxymethoxy)malononitrile (**3**; 31.3 mg, 0.248 mmol, 1 eq) were combined in DMF (5 mL); DIEA (86.4 µL, 0.496 mmol, 2 eq) was added, and the reaction was stirred at room temperature for 1 h. A second portion of 2-(methoxymethoxy)malononitrile (**3**; 15.6 mg, 0.124 mmol, 0.5 eq) was added, and stirring was continued for an additional 1 h at room temperature. The reaction was then concentrated *in vacuo* and purified by silica gel chromatography (25–100% EtOAc/toluene, linear gradient) to yield 30 mg (17%) of **36$_{MAC}$** as a green solid. ^{1}H NMR (CD_3OD, 400 MHz) δ 6.94 (s, 2H), 6.83 (s, 2H), 5.51 (q, *J* = 1.5 Hz, 2H), 5.19 (s, 2H), 3.53 (s, 3H), 1.77 (d, *J* = 1.4 Hz, 6H), 1.40 (s, 6H), 1.39 (s, 6H), 0.52 (s, 3H), 0.48 (s, 3H); ^{19}F NMR (CD_3OD, 376 MHz) δ -112.09 (dd, *J* = 14.6, 2.3 Hz, 1F), -130.38 (dd, *J* = 21.9, 2.5 Hz, 1F), -141.65 (dd, *J* = 21.9, 14.7 Hz, 1F); Analytical HPLC: t_R = 13.9 min, >99% purity (10–95% MeCN/H_2O, linear gradient, with constant 0.1% v/v TFA additive; 20 min run; 1 mL/min flow; ESI; positive ion mode; detection at 725 nm); HRMS (ESI) calcd for $C_{39}H_{38}F_3N_4O_4Si$ $[M+H]^+$ 711.2609, found 711.2614.

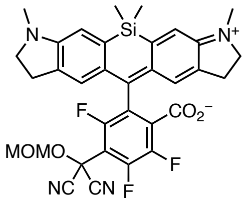

6-(MOM-MAC)-SiRF$_{712}$ (108$_{MAC}$): SiRF$_{712}$ (**108**; 180 mg, 0.343 mmol) and 2-(methoxymethoxy)malononitrile (**3**; 43.3 mg, 0.343 mmol, 1 eq) were combined in DMF (5 mL), and DIEA (120 µL, 0.686 mmol, 2 eq) was added. After stirring the reaction at room temperature for 1 h, it was concentrated *in vacuo* and purified by silica gel chromatography (10–100% acetone/CH_2Cl_2, linear gradient) followed by reverse phase HPLC (30–50% MeCN/H_2O, linear gradient, with constant 0.1% v/v TFA additive). The pooled HPLC product fractions were partially concentrated to remove MeCN, diluted with saturated $NaHCO_3$, and extracted with CH_2Cl_2 (2×). The organic extracts were dried over anhydrous $MgSO_4$, filtered, and evaporated to yield 99 mg (46%) of **108$_{MAC}$** as a green solid. ^{1}H NMR (CD_3OD, 400 MHz) δ 7.09 (s, 2H), 6.81 (s, 2H), 5.18 (s, 2H), 3.83 (t, *J* = 8.1 Hz, 4H), 3.51 (s, 3H), 3.21 (s, 6H), 3.06 (dt, *J* = 16.3, 8.0 Hz, 2H), 2.95 (dt, *J* = 16.8, 8.0 Hz, 2H), 0.54 (s, 3H), 0.53 (s, 3H); ^{19}F NMR (CD_3OD, 376 MHz) δ -112.97 (d, *J* = 14.8 Hz, 1F), -130.78 (d, *J* = 21.8 Hz, 1F), -142.67 (dd, *J* = 21.7, 14.9 Hz, 1F); Analytical HPLC: t_R = 12.4 min, >99% purity (10–95% MeCN/H_2O, linear gradient, with constant 0.1% v/v TFA additive; 20 min run; 1 mL/min flow; ESI; positive ion mode; detection at 725 nm); HRMS (ESI) calcd for $C_{33}H_{30}F_3N_4O_4Si$ $[M+H]^+$ 631.1983, found 631.1989.

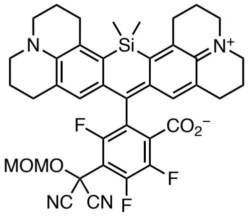

6-(MOM-MAC)-JF$_{698}$ (31$_{MAC}$): JF$_{698}$ (**31**; 150 mg, 0.248 mmol) and 2-(methoxymethoxy)malononitrile (**3**; 31.3 mg, 0.248 mmol, 1 eq) were combined in DMF (4 mL), and DIEA (86.4 µL, 0.496 mmol, 2 eq) was added. After stirring the reaction at room temperature for 2 h, it was concentrated *in vacuo* and purified by silica gel chromatography (0–15% MeOH/CH_2Cl_2, linear gradient, with constant 1% v/v AcOH additive) followed by reverse phase HPLC (40–50% MeCN/H_2O, linear gradient, with constant 0.1% v/v TFA additive). The pooled HPLC product fractions were partially concentrated to remove MeCN, diluted with saturated $NaHCO_3$, and extracted with CH_2Cl_2 (2×). The organic extracts were dried over anhydrous $MgSO_4$, filtered, and evaporated to yield 46 mg (26%) of **31$_{MAC}$** as a dark green solid. ^{1}H NMR (CD_3OD, 400 MHz) δ 6.70 (s, 2H), 5.15 (s, 2H), 3.56 (t, *J* = 6.0 Hz, 4H), 3.53 (t, *J* = 5.9 Hz, 4H), 3.50 (s, 3H), 3.00 – 2.92 (m, 4H), 2.72 – 2.46 (m, 4H), 2.10 – 2.01 (m, 4H), 1.96 – 1.88 (m, 4H), 0.68 (s, 3H), 0.68 (s, 3H); ^{19}F NMR (CD_3OD, 376 MHz) δ -112.34 (dd, *J* = 14.3, 2.3 Hz, 1F), -130.94 (dd, *J* = 22.0, 2.3 Hz, 1F), -142.74 (dd, *J* = 22.0, 14.6 Hz, 1F); Analytical HPLC: t_R = 13.2 min, >99% purity (10–95% MeCN/H_2O, linear gradient, with constant 0.1% v/v TFA additive; 20 min run; 1 mL/min flow; ESI; positive ion mode; detection at 700 nm); HRMS (ESI) calcd for $C_{39}H_{38}F_3N_4O_4Si$ $[M+H]^+$ 711.2609, found 711.2615.

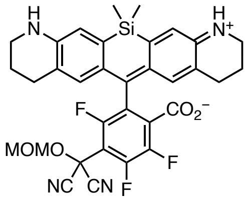

6-(MOM-MAC)-FSiRh$_Q$ (29$_{MAC}$): FSiRh$_Q$ (**29**; 170 mg, 0.324 mmol) and 2-(methoxymethoxy)malononitrile (**3**; 40.9 mg, 0.324 mmol, 1 eq) were combined in DMF (5 mL), and DIEA (113 µL, 0.648 mmol, 2 eq) was added. After stirring the reaction at room temperature for 1 h, it was concentrated *in vacuo* and purified by silica gel chromatography (0–20% MeOH/CH_2Cl_2, linear gradient, with constant 1% v/v AcOH additive) followed by reverse phase HPLC (30–50% MeCN/H_2O, linear gradient, with constant 0.1% v/v TFA additive). The pooled HPLC product fractions were partially concentrated to remove MeCN, diluted with saturated $NaHCO_3$, and extracted with 15% *i*-PrOH/$CHCl_3$ (2×) followed by 20% MeOH/CH_2Cl_2 (3×). The combined organic extracts were dried over anhydrous $MgSO_4$, filtered, and evaporated to yield 86 mg (42%) of **29$_{MAC}$** as a dark blue solid. ^{1}H NMR (CD_3OD, 400 MHz) δ 6.95 (s, 2H), 6.84 (s, 2H), 5.17 (s, 2H), 3.51 (s, 3H), 3.50 – 3.45 (m, 4H), 2.68 (dt, *J* = 16.2, 6.0 Hz, 2H), 2.56 (dt, *J* = 16.1, 6.3 Hz, 2H), 1.89 (p, *J* = 6.2 Hz, 4H), 0.47 (s, 3H), 0.44 (s, 3H); ^{19}F NMR (CD_3OD, 376 MHz) δ -112.41 (dd, *J* = 15.0, 2.0 Hz, 1F), -130.57 (dd, *J* = 21.8, 2.0 Hz, 1F), -142.51 (dd, *J* = 21.9, 14.8 Hz, 1F); Analytical HPLC: t_R = 12.2 min, >99% purity (10–95% MeCN/H_2O, linear gradient, with constant 0.1% v/v TFA additive; 20 min run; 1 mL/min flow; ESI; positive ion mode; detection at 675 nm); HRMS (ESI) calcd for $C_{33}H_{30}F_3N_4O_4Si$ $[M+H]^+$ 631.1983, found 631.1988.

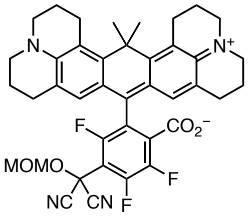

6-(MOM-MAC)-JF$_{660}$ (57$_{MAC}$): JF$_{660}$ (**57**; 275 mg, 0.467 mmol) and 2-(methoxymethoxy)malononitrile (**3**; 58.9 mg, 0.467 mmol, 1 eq) were combined in DMF (8 mL), and DIEA (163 µL, 0.934 mmol, 2 eq) was added. After stirring the reaction at room temperature for 2 h, it was evaporated to dryness. Flash chromatography on silica gel (0–20% MeOH/CH_2Cl_2, linear gradient, with constant 0.1% v/v AcOH additive) afforded **57$_{MAC}$** as a dark blue solid (133 mg, 41%). ^{1}H NMR (CD_3OD, 400 MHz) δ 6.63 (s, 2H), 5.20 (s, 2H), 3.58 (t, *J* = 6.1 Hz, 4H), 3.55 – 3.51 (m, 4H), 3.51 (s, 3H), 3.11 – 3.05 (m, 4H), 2.68 – 2.58 (m, 4H), 2.08 – 2.00 (m, 4H), 2.05 (s, 3H), 1.97 (s, 3H), 1.93 (p, *J* = 6.1 Hz, 4H); ^{19}F NMR (CD_3OD, 376 MHz) δ -111.21 (d, *J* = 14.7 Hz, 1F), -129.21 (d, *J* = 20.6 Hz, 1F), -139.69 (dd, *J* = 20.5, 14.5 Hz, 1F); Analytical HPLC: t_R = 13.1 min, >99% purity (10–95% MeCN/H_2O, linear gradient, with constant 0.1% v/v TFA additive; 20 min run; 1 mL/min flow; ESI; positive ion mode; detection at 675 nm); HRMS (ESI) calcd for $C_{40}H_{38}F_3N_4O_4$ $[M+H]^+$ 695.2840, found 695.2842.

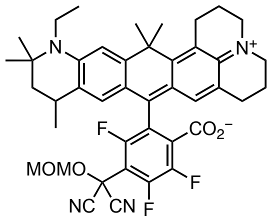

6-(MOM-MAC)-JF$_{657}$ (59$_{MAC}$): JF$_{657}$ (**59**; 150 mg, 0.242 mmol) and 2-(methoxymethoxy)malononitrile (**3**; 30.6 mg, 0.242 mmol, 1 eq) were combined in DMF (5 mL), and DIEA (84.5 µL, 0.485 mmol, 2 eq) was added. After stirring the reaction at room temperature for 2 h, it was concentrated *in vacuo* and purified by flash chromatography on silica gel (0–20% MeOH/CH_2Cl_2, linear gradient, with constant 0.1% v/v AcOH additive) to provide 83.8 mg (48%) of **59$_{MAC}$** as a dark blue solid (mixture of two diastereomers). ^{1}H NMR (CD_3OD, 400 MHz) δ 6.92 (s, 1H), 6.89 – 6.80 (m, 2H), 5.22 – 5.14 (m, 2H), 3.82 (dq, *J* = 14.2, 7.0 Hz, 1H), 3.66 (dq, *J* = 14.5, 7.3 Hz, 1H), 3.60 (t, *J* = 5.8 Hz, 2H), 3.56 (t, *J* = 6.0 Hz, 2H), 3.53 (s, 1.5H), 3.52 (s, 1.5H), 3.24 – 3.16 (m, 2H), 2.87 – 2.69 (m, 2H), 2.61 (dt, *J* = 15.4, 6.2 Hz, 1H), 2.11 – 2.00 (m, 2H), 1.97 – 1.92 (m, 2H), 1.91 – 1.83 (m, 1H), 1.90 (s, 1.5H), 1.88 (s, 1.5H), 1.86 (s, 1.5H), 1.85 (s, 1.5H), 1.60 – 1.49 (m, 1H), 1.48 (s, 1.5H), 1.47 (s, 1.5H), 1.36 (s, 1.5H), 1.35 (t, *J* = 7.1 Hz, 3H), 1.33 (s, 1.5H), 1.19 (d, *J* = 6.6 Hz, 1.5H), 1.15 (d, *J* = 6.5 Hz, 1.5H); ^{19}F NMR (CD_3OD, 376 MHz) δ -111.78 (d, *J* = 14.7 Hz, 1F), -129.97 (dd, *J* = 21.8, 2.0 Hz, 0.5F), -130.09 (dd, *J* = 21.8, 2.0 Hz, 0.5F), -141.98 (dd, *J* = 21.9, 14.5 Hz, 0.5F), -142.00 (dd, *J* = 22.1, 14.6 Hz, 0.5F); Analytical HPLC: t_R (two isomers) = 13.8 min, 14.1 min; >99% total purity (10–95% MeCN/H_2O, linear gradient, with constant 0.1% v/v TFA additive; 20 min run; 1 mL/min flow; ESI; positive ion mode; detection at 650 nm); HRMS (ESI) calcd for $C_{42}H_{44}F_3N_4O_4$ $[M+H]^+$ 725.3309, found 725.3310.

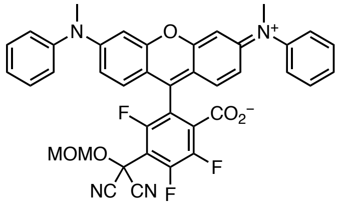

2-(3,6-Bis(methyl(phenyl)amino)xanthylium-9-yl)-4-(dicyano(methoxymethoxy)methyl)-3,5,6-trifluoro-benzoate (64$_{MAC}$): Rhodamine **64** (165 mg, 0.283 mmol) and 2-(methoxymethoxy)malononitrile (**3**; 35.7 mg, 0.283 mmol, 1 eq) were combined in DMF (6 mL), and DIEA (98.7 µL, 0.566 mmol, 2 eq) was added. After stirring the reaction at room temperature for 2 h, it was evaporated to dryness. Flash chromatography on silica gel (25–100% EtOAc/hexanes, linear gradient) afforded **64$_{MAC}$** (115 mg, 59%) as a dark purple solid. ^{1}H NMR ($CDCl_3$, 400 MHz) δ 7.45 – 7.37 (m, 4H), 7.26 – 7.19 (m, 6H), 6.78 (d, *J* = 8.9 Hz, 2H), 6.61 (d, *J* = 2.4 Hz, 2H), 6.58 (dd, *J* = 8.9, 2.5 Hz, 2H), 5.14 (s, 2H), 3.53 (s, 3H), 3.38 (s, 6H); ^{19}F NMR ($CDCl_3$, 376 MHz) δ -116.29 (d, *J* = 21.6 Hz, 1F), -126.47 (d, *J* = 20.8 Hz, 1F), -139.41 (t, *J* = 21.2 Hz, 1F); Analytical HPLC: t_R = 11.4 min, >99% purity (30–95% MeCN/H_2O, linear gradient, with constant 0.1% v/v TFA additive; 20 min run; 1 mL/min flow; ESI; positive ion mode; detection at 550 nm); HRMS (ESI) calcd for $C_{39}H_{28}F_3N_4O_5$ $[M+H]^+$ 689.2006, found 689.1999.

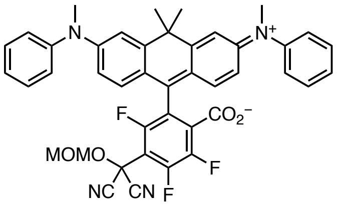

6-(MOM-MAC)-JQ$_{645}$ (65$_{MAC}$): Carborhodamine **65** (150 mg, 0.246 mmol) and 2-(methoxymethoxy)malononitrile (**3**; 31.1 mg, 0.246 mmol, 1 eq) were combined in DMF (6 mL), and DIEA (85.8 µL, 0.493 mmol, 2 eq) was added. After stirring the reaction at room temperature for 2 h, it was evaporated to dryness. Flash chromatography on silica gel (0–75% EtOAc/hexanes, linear gradient) afforded **65$_{MAC}$** as a yellow-green solid (93.3 mg, 53%). ^{1}H NMR ($CDCl_3$, 400 MHz) δ 7.39 – 7.32 (m, 4H), 7.20 – 7.15 (m, 4H), 7.14 – 7.09 (m, 2H), 7.09 (d, *J* = 2.5 Hz, 2H), 6.74 (dd, *J* = 8.8, 2.5 Hz, 2H), 6.64 (d, *J* = 8.8 Hz, 2H), 5.14 (s, 2H), 3.51 (s, 3H), 3.37 (s, 6H), 1.65 (s, 3H), 1.63 (s, 3H); ^{19}F NMR ($CDCl_3$, 376 MHz) δ -116.51 (d, *J* = 22.7 Hz, 1F), -127.06 (d, *J* = 20.5 Hz, 1F), -140.00 (dd, *J* = 22.9, 20.3 Hz, 1F); Analytical HPLC: t_R = 14.1 min, >99% purity (30–95% MeCN/H_2O, linear gradient, with constant 0.1% v/v TFA additive; 20 min run; 1 mL/min flow; ESI; positive ion mode; detection at 650 nm); HRMS (ESI) calcd for $C_{42}H_{34}F_3N_4O_4$ $[M+H]^+$ 715.2527, found 715.2518.

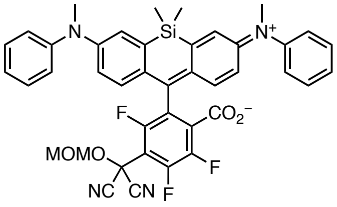

4-(Dicyano(methoxymethoxy)methyl)-2-(5,5-dimethyl-3,7-bis(methyl(phenyl)amino)dibenzo[*b*,*e*]silin-10-ylium-10(5*H*)-yl)-3,5,6-trifluorobenzoate (66$_{MAC}$): Si-rhodamine **66** (125 mg, 0.200 mmol) and 2-(methoxymethoxy)malononitrile (**3**; 25.2 mg, 0.200 mmol, 1 eq) were combined in DMF (5 mL), and DIEA (69.7 µL, 0.400 mmol, 2 eq) was added. After stirring the reaction at room temperature for 2 h, it was evaporated to dryness. Flash chromatography on silica gel (0–40% EtOAc/hexanes, linear gradient) afforded **66$_{MAC}$** as an off-white solid (79.9 mg, 55%). ^{1}H NMR ($CDCl_3$, 400 MHz) δ 7.38 – 7.31 (m, 4H), 7.19 – 7.09 (m, 8H), 6.79 (dd, *J* = 8.9, 2.7 Hz, 2H), 6.71 (dd, *J* = 8.9, 0.7 Hz, 2H), 5.18 (s, 2H), 3.54 (s, 3H), 3.36 (s, 6H), 0.50 (s, 3H), 0.47 (s, 3H); ^{19}F NMR ($CDCl_3$, 376 MHz) δ -113.25 (d, *J* = 22.4 Hz, 1F), -127.11 (d, *J* = 20.2 Hz, 1F), -139.39 (dd, *J* = 22.7, 20.2 Hz, 1F); Analytical HPLC: t_R = 15.0 min, >99% purity (50–95% MeCN/H_2O, linear gradient, with constant 0.1% v/v TFA additive; 20 min run; 1 mL/min flow; ESI; positive ion mode; detection at 675 nm); HRMS (ESI) calcd for $C_{41}H_{34}F_3N_4O_4Si$ $[M+H]^+$ 731.2296, found 731.2290.

CONVERSION OF MAC XANTHENES TO HALOTAG LIGANDS (SCHEME S8)

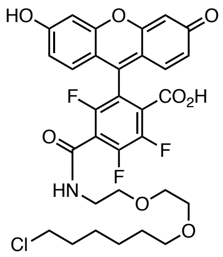

4,5,7-Trifluoro-fluorescein–HaloTag ligand ($\mathbf{60_{HTL}}$): 4,5,7-Trifluoro-6-(MOM-MAC)-fluorescein ($\mathbf{60_{MAC}}$; 30 mg, 58.8 µmol) was taken up in CH_2Cl_2 (4 mL); triethylsilane (400 µL) was added, followed by trifluoroacetic acid (800 µL). The reaction was stirred at room temperature for 24 h. Toluene (5 mL) was added, and the reaction mixture was concentrated to dryness. The residue was combined with a premixed solution of HaloTag(O2)amine (HTL-NH_2, **111**; TFA salt; 39.7 mg, 0.118 mmol, 2 eq) and DIEA (102 µL, 0.588 mmol, 10 eq) in DMF (4 mL), and the reaction was stirred at room temperature for 18 h. The solvent was removed by rotary evaporation, and the crude material was purified by reverse phase HPLC (20–70% MeCN/H_2O, linear gradient, with constant 0.1% v/v TFA additive) to yield 27.1 mg (72%) of $\mathbf{60_{HTL}}$ as an orange solid. ^1H NMR (CD_3OD, 400 MHz) δ 9.05 (t, J = 5.1 Hz, 1H), 6.89 (d, J = 8.7 Hz, 2H), 6.70 (d, J = 2.4 Hz, 2H), 6.63 (dd, J = 8.7, 2.4 Hz, 2H), 3.61 – 3.47 (m, 10H), 3.40 (t, J = 6.5 Hz, 2H), 1.78 – 1.69 (m, 2H), 1.55 – 1.46 (m, 2H), 1.46 – 1.38 (m, 2H), 1.37 – 1.28 (m, 2H); ^{19}F NMR (CD_3OD, 376 MHz) δ -122.09 – -122.44 (m, 1F), -133.85 (d, J = 20.3 Hz, 1F), -142.82 – -143.23 (m, 1F); Analytical HPLC: t_R = 13.7 min, >99% purity (10–95% MeCN/H_2O, linear gradient, with constant 0.1% v/v TFA additive; 20 min run; 1 mL/min flow; ESI; positive ion mode; detection at 254 nm); HRMS (ESI) calcd for $C_{31}H_{30}ClF_3NO_8$ $[M+H]^+$ 636.1607, found 636.1611.

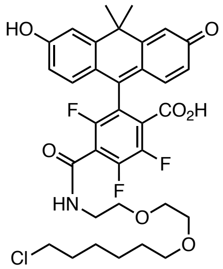

4,5,7-Trifluoro-carbofluorescein–HaloTag ligand ($\mathbf{51_{HTL}}$): 4,5,7-Trifluoro-6-(MOM-MAC)-carbofluorescein ($\mathbf{51_{MAC}}$; 50 mg, 93.2 µmol) was taken up in CH_2Cl_2 (3 mL); triethylsilane (300 µL) was added, followed by trifluoroacetic acid (600 µL). The reaction was stirred at room temperature for 18 h. Toluene (4 mL) was added, and the reaction mixture was concentrated to dryness. The residue was combined with a premixed solution of HaloTag(O2)amine (HTL-NH_2, **111**; TFA salt; 63.0 mg, 0.186 mmol, 2 eq) and DIEA (162 µL, 0.932 mmol, 10 eq) in DMF (3 mL), and the reaction was stirred at room temperature for 2 h. It was then diluted with 10% citric acid and extracted with EtOAc (2×). The combined organic extracts were dried over anhydrous $MgSO_4$, filtered, and concentrated *in vacuo*. Purification by reverse phase HPLC (30–70% MeCN/H_2O, linear gradient, with constant 0.1%

v/v TFA additive) yielded 14.2 mg (23%) of **51$_{HTL}$** as a light pink solid. ^{1}H NMR (CD_3OD, 400 MHz) δ 9.07 (t, *J* = 5.6 Hz, 1H), 7.09 (d, *J* = 2.5 Hz, 2H), 6.77 (d, *J* = 8.6 Hz, 2H), 6.67 (dd, *J* = 8.6, 2.5 Hz, 2H), 3.60 – 3.46 (m, 10H), 3.40 (t, *J* = 6.5 Hz, 2H), 1.78 – 1.68 (m, 2H), 1.74 (s, 3H), 1.69 (s, 3H), 1.56 – 1.46 (m, 2H), 1.46 – 1.38 (m, 2H), 1.38 – 1.28 (m, 2H); ^{19}F NMR (CD_3OD, 376 MHz) δ -122.22 (d, *J* = 22.5 Hz, 1F), -134.87 (d, *J* = 20.4 Hz, 1F), -143.66 (t, *J* = 21.6 Hz, 1F); Analytical HPLC: t_R = 13.9 min, >99% purity (10–95% MeCN/H_2O, linear gradient, with constant 0.1% v/v TFA additive; 20 min run; 1 mL/min flow; ESI; positive ion mode; detection at 254 nm); HRMS (ESI) calcd for $C_{34}H_{36}ClF_3NO_7$ $[M+H]^+$ 662.2127, found 662.2129.

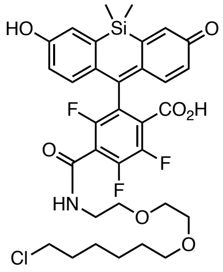

4,5,7-Trifluoro-Si-fluorescein–HaloTag ligand (55$_{HTL}$): 4,5,7-Trifluoro-6-(MOM-MAC)-Si-fluorescein (**55$_{MAC}$**; 30 mg, 54.3 μmol) was taken up in CH_2Cl_2 (3 mL); triethylsilane (300 μL) was added, followed by trifluoroacetic acid (600 μL). The reaction was stirred at room temperature for 24 h. Toluene (4 mL) was added, and the reaction mixture was concentrated to dryness. The residue was combined with a premixed solution of HaloTag(O2)amine (HTL-NH_2, **111**; TFA salt; 36.7 mg, 0.109 mmol, 2 eq) and DIEA (94.6 μL, 0.543 mmol, 10 eq) in DMF (3 mL), and the reaction was stirred at room temperature for 2 h. The solvent was removed by rotary evaporation, and the crude material was purified by reverse phase HPLC (20–70% MeCN/H_2O, linear gradient, with constant 0.1% v/v TFA additive) to yield 20.4 mg (55%) of **55$_{HTL}$** as a white solid. ^{1}H NMR (CD_3OD, 400 MHz) δ 9.15 (t, *J* = 5.3 Hz, 1H), 7.14 (d, *J* = 2.7 Hz, 2H), 6.87 (dd, *J* = 8.7, 1.0 Hz, 2H), 6.74 (dd, *J* = 8.7, 2.8 Hz, 2H), 3.65 – 3.52 (m, 8H), 3.50 (t, *J* = 6.7 Hz, 2H), 3.42 (t, *J* = 6.6 Hz, 2H), 1.74 – 1.65 (m, 2H), 1.55 – 1.46 (m, 2H), 1.44 – 1.26 (m, 4H), 0.56 (s, 3H), 0.52 (s, 3H); ^{19}F NMR (CD_3OD, 376 MHz) δ -118.77 (d, *J* = 22.2 Hz, 1F), -134.57 – -134.69 (m, 1F), -143.21 (t, *J* = 21.6 Hz, 1F); Analytical HPLC: t_R = 14.2 min, >99% purity (10–95% MeCN/H_2O, linear gradient, with constant 0.1% v/v TFA additive; 20 min run; 1 mL/min flow; ESI; positive ion mode; detection at 254 nm); HRMS (ESI) calcd for $C_{33}H_{36}ClF_3NO_7Si$ $[M+H]^+$ 678.1896, found 678.1907.

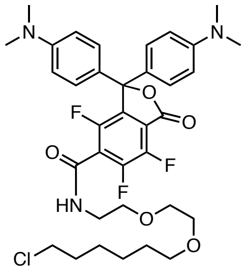

FMGL–HaloTag ligand (49$_{HTL}$): 4,5,7-Trifluoro-6-(MOM-MAC)-MGL (**49$_{MAC}$**; 75 mg, 0.136 mmol) was taken up in CH_2Cl_2 (5 mL); triethylsilane (500 μL) was added, followed by trifluoroacetic acid (1 mL). The reaction was stirred

at room temperature for 18 h. Toluene (5 mL) was added, and the reaction mixture was concentrated to dryness. The residue was combined with a premixed solution of HaloTag(O2)amine (HTL-NH_2, **111**; TFA salt; 92.0 mg, 0.272 mmol, 2 eq) and DIEA (237 µL, 1.36 mmol, 10 eq) in CH_2Cl_2 (4 mL), and the reaction was stirred at room temperature for 6 h. The solvent was removed by rotary evaporation, and the crude material was purified by reverse phase HPLC (20–80% MeCN/H_2O, linear gradient, with constant 0.1% v/v TFA additive) to yield 49.5 mg (46%, TFA salt) of **49$_{HTL}$** as a blue solid. ^{1}H NMR (CD_3OD, 400 MHz) δ 9.09 (t, *J* = 5.4 Hz, 1H), 7.21 (d, *J* = 9.0 Hz, 4H), 6.92 (d, *J* = 8.9 Hz, 4H), 3.66 – 3.54 (m, 8H), 3.52 (t, *J* = 6.7 Hz, 2H), 3.45 (t, *J* = 6.5 Hz, 2H), 3.03 (s, 12H), 1.77 – 1.68 (m, 2H), 1.58 – 1.50 (m, 2H), 1.47 – 1.29 (m, 4H); ^{19}F NMR (CD_3OD, 376 MHz) δ -75.79 (s, 3F), -117.70 (d, *J* = 22.0 Hz, 1F), -134.45 (d, *J* = 21.0 Hz, 1F), -142.89 (t, *J* = 21.5 Hz, 1F); Analytical HPLC: t_R = 13.5 min, >99% purity (10–95% MeCN/H_2O, linear gradient, with constant 0.1% v/v TFA additive; 20 min run; 1 mL/min flow; ESI; positive ion mode; detection at 280 nm); HRMS (ESI) calcd for $C_{35}H_{42}ClF_3N_3O_5$ $[M+H]^+$ 676.2760, found 676.2767.

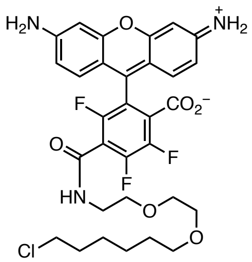

FRh$_{110}$–HaloTag ligand (70$_{HTL}$): 4,5,7-Trifluoro-6-(MOM-MAC)-rhodamine 110 bis(*tert*-butyl carbamate) (**67$_{MAC}$**; 90 mg, 0.127 mmol) was taken up in CH_2Cl_2 (5 mL); triethylsilane (500 µL) was added, followed by trifluoroacetic acid (1 mL). The reaction was stirred at room temperature for 8 h. Toluene (5 mL) was added, and the reaction mixture was concentrated to dryness. The residue was combined with a premixed solution of HaloTag(O2)amine (HTL-NH_2, **111**; TFA salt; 85.8 mg, 0.254 mmol, 2 eq) and DIEA (221 µL, 1.27 mmol, 10 eq) in DMF (3 mL), and the reaction was stirred at room temperature for 18 h. The solvent was removed by rotary evaporation, and the crude material was purified by reverse phase HPLC (30–40% MeCN/H_2O, linear gradient, with constant 0.1% v/v TFA additive) to afford **70$_{HTL}$** as a red-orange solid (20.5 mg, 22%, TFA salt). ^{1}H NMR (CD_3OD, 400 MHz) δ 9.12 (t, *J* = 5.5 Hz, 1H), 7.29 (dd, *J* = 9.2, 0.9 Hz, 2H), 6.88 (dd, *J* = 9.2, 2.1 Hz, 2H), 6.80 (d, *J* = 2.1 Hz, 2H), 3.68 – 3.54 (m, 8H), 3.51 (t, *J* = 6.6 Hz, 2H), 3.42 (t, *J* = 6.5 Hz, 2H), 1.76 – 1.66 (m, 2H), 1.54 – 1.45 (m, 2H), 1.44 – 1.27 (m, 4H); ^{19}F NMR (CD_3OD, 376 MHz) δ -75.34 (s, 3F), -116.65 (d, *J* = 15.3 Hz, 1F), -132.55 (d, *J* = 21.7 Hz, 1F), -140.18 – -140.53 (m, 1F); Analytical HPLC: t_R = 11.0 min, >99% purity (10–95% MeCN/H_2O, linear gradient, with constant 0.1% v/v TFA additive; 20 min run; 1 mL/min flow; ESI; positive ion mode; detection at 525 nm); HRMS (ESI) calcd for $C_{31}H_{32}ClF_3N_3O_6$ $[M+H]^+$ 634.1926, found 634.1926.

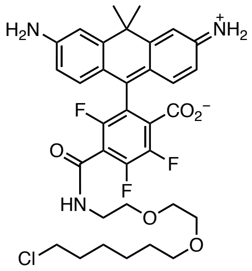

FCRh$_{110}$–HaloTag ligand (71$_{HTL}$): 4,5,7-Trifluoro-6-(MOM-MAC)-carborhodamine 110 bis(*tert*-butyl carbamate) (**68$_{MAC}$**; 90 mg, 0.122 mmol) was taken up in CH_2Cl_2 (5 mL); triethylsilane (500 µL) was added, followed by trifluoroacetic acid (1 mL). The reaction was stirred at room temperature for 6 h. Toluene (5 mL) was added, and the reaction mixture was concentrated to dryness. The residue was combined with a premixed solution of HaloTag(O2)amine (HTL-NH_2, **111**; TFA salt; 82.7 mg, 0.245 mmol, 2 eq) and DIEA (213 µL, 1.22 mmol, 10 eq) in DMF (3 mL), and the reaction was stirred at room temperature for 18 h. The solvent was removed by rotary evaporation, and the crude material was purified by reverse phase HPLC (30–50% MeCN/H_2O, linear gradient, with constant 0.1% v/v TFA additive) to provide **71$_{HTL}$** as a purple solid (30.5 mg, 32%, TFA salt). ^{1}H NMR (CD_3OD, 400 MHz) δ 9.14 (t, *J* = 5.4 Hz, 1H), 7.153 (dd, *J* = 9.1, 0.9 Hz, 2H), 7.148 (d, *J* = 2.2 Hz, 2H), 6.67 (dd, *J* = 9.0, 2.2 Hz, 2H), 3.68 – 3.55 (m, 8H), 3.52 (t, *J* = 6.6 Hz, 2H), 3.44 (t, *J* = 6.5 Hz, 2H), 1.75 (s, 3H), 1.74 – 1.67 (m, 2H), 1.64 (s, 3H), 1.56 – 1.47 (m, 2H), 1.45 – 1.28 (m, 4H); ^{19}F NMR (CD_3OD, 376 MHz) δ -75.34 (s, 3F), -117.19 (d, *J* = 15.3 Hz, 1F), -133.70 (d, *J* = 22.2 Hz, 1F), -140.45 (dd, *J* = 22.1, 15.6 Hz, 1F); Analytical HPLC: t_R = 11.3 min, >99% purity (10–95% MeCN/H_2O, linear gradient, with constant 0.1% v/v TFA additive; 20 min run; 1 mL/min flow; ESI; positive ion mode; detection at 575 nm); HRMS (ESI) calcd for $C_{34}H_{38}ClF_3N_3O_5$ $[M+H]^+$ 660.2447, found 660.2459.

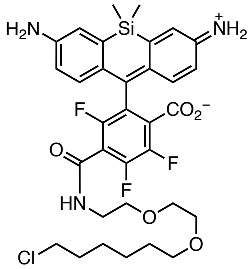

FSiRh$_{110}$–HaloTag ligand (72$_{HTL}$): 4,5,7-Trifluoro-6-(MOM-MAC)-Si-rhodamine 110 bis(*tert*-butyl carbamate) (**69$_{MAC}$**; 75 mg, 0.100 mmol) was taken up in CH_2Cl_2 (4 mL); triethylsilane (400 µL) was added, followed by trifluoroacetic acid (800 µL). The reaction was stirred at room temperature for 6 h. Toluene (4 mL) was added, and the reaction mixture was concentrated to dryness. The residue was combined with a premixed solution of HaloTag(O2)amine (HTL-NH_2, **111**; TFA salt; 67.5 mg, 0.200 mmol, 2 eq) and DIEA (174 µL, 1.00 mmol, 10 eq) in DMF (3 mL), and the reaction was stirred at room temperature for 18 h. The solvent was removed by rotary evaporation, and the crude material was purified by reverse phase HPLC (30–50% MeCN/H_2O, linear gradient, with constant 0.1% v/v TFA additive) to provide **72$_{HTL}$** as a bright blue solid (30.7 mg, 39%, TFA salt). ^{1}H NMR (CD_3OD, 400 MHz) δ 9.17 (t, *J* = 5.0 Hz, 1H), 7.21 (d, *J* = 2.5 Hz, 2H), 6.99 (dd, *J* = 8.9, 1.3 Hz, 2H), 6.76 (dd, *J* = 8.9, 2.5 Hz, 2H), 3.67 – 3.54 (m, 8H), 3.51 (t, *J* = 6.6 Hz, 2H), 3.44 (t, *J* = 6.5 Hz, 2H), 1.75 – 1.66 (m, 2H), 1.56 – 1.47 (m, 2H),

1.45 – 1.28 (m, 4H), 0.57 (s, 3H), 0.51 (s, 3H); ^{19}F NMR (CD_3OD, 376 MHz) δ -75.40 (s, 3F), -118.23 (d, J = 19.4 Hz, 1F), -134.29 (d, J = 21.2 Hz, 1F), -141.77 – -142.06 (m, 1F); Analytical HPLC: t_R = 11.7 min, >99% purity (10–95% MeCN/H_2O, linear gradient, with constant 0.1% v/v TFA additive; 20 min run; 1 mL/min flow; ESI; positive ion mode; detection at 625 nm); HRMS (ESI) calcd for $C_{33}H_{38}ClF_3N_3O_5Si$ $[M+H]^+$ 676.2216, found 676.2222.

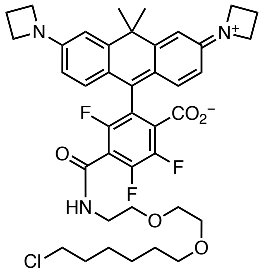

JF$_{632}$–HaloTag ligand (44$_{HTL}$): 6-(MOM-MAC)-JF$_{632}$ (**44$_{MAC}$**; 20.0 mg, 32.5 μmol) was taken up in CH_2Cl_2 (2 mL); triethylsilane (200 μL) was added, followed by trifluoroacetic acid (400 μL). The reaction was stirred at room temperature for 6 h. Toluene (3 mL) was added, and the reaction mixture was concentrated to dryness. The residue was combined with a premixed solution of HaloTag(O2)amine (HTL-NH_2, **111**; TFA salt; 22.0 mg, 65.1 μmol, 2 eq) and DIEA (56.7 μL, 0.325 mmol, 10 eq) in CH_2Cl_2 (2 mL), and the reaction was stirred at room temperature for 18 h. The solvent was removed by rotary evaporation, and the crude material was purified by reverse phase HPLC (20–80% MeCN/H_2O, linear gradient, with constant 0.1% v/v TFA additive) to yield 20.2 mg (73%, TFA salt) of **44$_{HTL}$** as a deep blue solid. ^1H NMR (CD_3OD, 400 MHz) δ 9.09 (t, J = 5.4 Hz, 1H), 7.15 (dd, J = 9.1, 0.5 Hz, 2H), 6.81 (d, J = 2.2 Hz, 2H), 6.45 (dd, J = 9.1, 2.2 Hz, 2H), 4.37 (t, J = 7.7 Hz, 8H), 3.67 – 3.55 (m, 8H), 3.52 (t, J = 6.7 Hz, 2H), 3.44 (t, J = 6.5 Hz, 2H), 2.56 (p, J = 7.8 Hz, 4H), 1.77 (s, 3H), 1.75 – 1.68 (m, 2H), 1.66 (s, 3H), 1.56 – 1.48 (m, 2H), 1.44 – 1.30 (m, 4H); ^{19}F NMR (CD_3OD, 376 MHz) δ -75.31 (s, 3F), -117.35 (d, J = 15.2 Hz, 1F), -133.92 (d, J = 22.5 Hz, 1F), -140.91 (dd, J = 22.1, 15.3 Hz, 1F); Analytical HPLC: t_R = 12.9 min, >99% purity (10–95% MeCN/H_2O, linear gradient, with constant 0.1% v/v TFA additive; 20 min run; 1 mL/min flow; ESI; positive ion mode; detection at 625 nm); HRMS (ESI) calcd for $C_{40}H_{46}ClF_3N_3O_5$ $[M+H]^+$ 740.3073, found 740.3068.

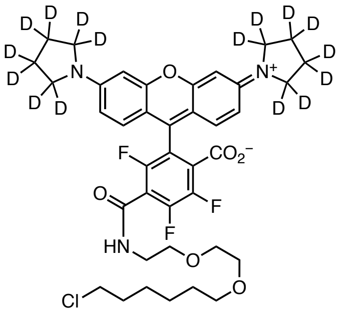

JFX$_{576}$–HaloTag ligand (8$_{HTL}$): 6-(MOM-MAC)-JFX$_{576}$ (**8$_{MAC}$**; 60 mg, 86.6 μmol) was taken up in CH_2Cl_2 (5 mL); triethylsilane (500 μL) was added, followed by trifluoroacetic acid (1 mL). The reaction was stirred at room temperature for 6 h. Toluene (5 mL) was added, and the reaction mixture was concentrated to dryness. The residue was combined with a premixed solution of HaloTag(O2)amine (HTL-NH_2, **111**; TFA salt; 58.5 mg, 0.173 mmol, 2

eq) and DIEA (151 µL, 0.866 mmol, 10 eq) in CH_2Cl_2 (4 mL), and the reaction was stirred at room temperature for 18 h. The solvent was removed by rotary evaporation, and the crude material was purified by reverse phase HPLC (30–60% MeCN/H_2O, linear gradient, with constant 0.1% v/v TFA additive) to afford **8**$_{\mathbf{HTL}}$ as a dark red-purple solid (49.2 mg, 65%, TFA salt). ^{1}H NMR (CD_3OD, 400 MHz) δ 9.13 (t, *J* = 5.5 Hz, 1H), 7.33 (d, *J* = 9.4 Hz, 2H), 6.97 (dd, *J* = 9.4, 2.3 Hz, 2H), 6.82 (d, *J* = 2.3 Hz, 2H), 3.67 – 3.55 (m, 8H), 3.51 (t, *J* = 6.6 Hz, 2H), 3.42 (t, *J* = 6.5 Hz, 2H), 1.74 – 1.66 (m, 2H), 1.50 (p, *J* = 6.8 Hz, 2H), 1.43 – 1.26 (m, 4H); ^{19}F NMR (CD_3OD, 376 MHz) δ -75.36 (s, 3F), -116.57 (d, *J* = 15.1 Hz, 1F), -132.50 (d, *J* = 22.3 Hz, 1F), -140.03 – -140.23 (m, 1F); Analytical HPLC: t_R = 13.1 min, >99% purity (10–95% MeCN/H_2O, linear gradient, with constant 0.1% v/v TFA additive; 20 min run; 1 mL/min flow; ESI; positive ion mode; detection at 575 nm); HRMS (ESI) calcd for $C_{39}H_{28}D_{16}ClF_3N_3O_6$ $[M+H]^+$ 758.3870, found 758.3861.

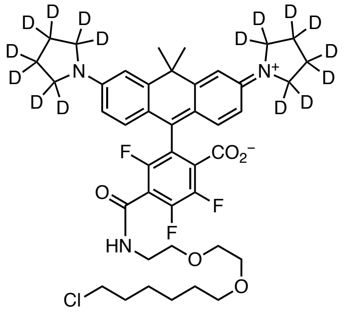

JFX$_{637}$–HaloTag ligand (40$_{\mathbf{HTL}}$): 6-(MOM-MAC)-JFX$_{637}$ (**40**$_{\mathbf{MAC}}$; 28.0 mg, 42.5 µmol) was taken up in CH_2Cl_2 (3 mL); triethylsilane (300 µL) was added, followed by trifluoroacetic acid (600 µL). The reaction was stirred at room temperature for 6 h. Toluene (4 mL) was added, and the reaction mixture was concentrated to dryness. The residue was combined with a premixed solution of HaloTag(O2)amine (HTL-NH_2, **111**; TFA salt; 28.7 mg, 85.0 µmol, 2 eq) and DIEA (74.0 µL, 0.425 mmol, 10 eq) in CH_2Cl_2 (3 mL), and the reaction was stirred at room temperature for 18 h. The solvent was removed by rotary evaporation, and the crude material was purified by reverse phase HPLC (30–70% MeCN/H_2O, linear gradient, with constant 0.1% v/v TFA additive) to yield 18.9 mg (49%, TFA salt) of **40**$_{\mathbf{HTL}}$ as a deep blue solid. ^{1}H NMR (CD_3OD, 400 MHz) δ 9.15 (t, *J* = 5.4 Hz, 1H), 7.21 (dd, *J* = 9.3, 1.0 Hz, 2H), 7.10 (d, *J* = 2.4 Hz, 2H), 6.74 (dd, *J* = 9.3, 2.4 Hz, 2H), 3.68 – 3.55 (m, 8H), 3.51 (t, *J* = 6.6 Hz, 2H), 3.43 (t, *J* = 6.5 Hz, 2H), 1.83 (s, 3H), 1.75 – 1.66 (m, 2H), 1.71 (s, 3H), 1.55 – 1.47 (m, 2H), 1.44 – 1.26 (m, 4H); ^{19}F NMR (CD_3OD, 376 MHz) δ -75.29 (s, 3F), -117.12 (d, *J* = 15.0 Hz, 1F), -133.84 (d, *J* = 22.1 Hz, 1F), -140.64 – -140.81 (m, 1F); Analytical HPLC: t_R = 13.4 min, >99% purity (10–95% MeCN/H_2O, linear gradient, with constant 0.1% v/v TFA additive; 20 min run; 1 mL/min flow; ESI; positive ion mode; detection at 650 nm); HRMS (ESI) calcd for $C_{42}H_{34}D_{16}ClF_3N_3O_5$ $[M+H]^+$ 784.4390, found 784.4385.

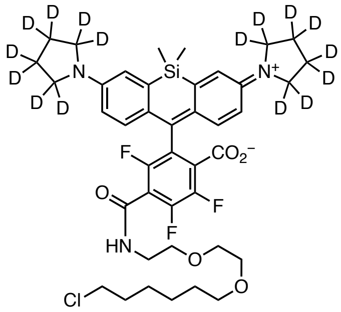

JFX$_{673}$–HaloTag ligand (25$_{HTL}$): 6-(MOM-MAC)-JFX$_{673}$ (**25$_{MAC}$**; 50 mg, 74.1 μmol) was taken up in CH_2Cl_2 (3 mL); triethylsilane (300 μL) was added, followed by trifluoroacetic acid (600 μL). The reaction was stirred at room temperature for 6 h. Toluene (4 mL) was added, and the reaction mixture was concentrated to dryness. The residue was combined with a premixed solution of HaloTag(O2)amine (HTL-NH_2, **111**; TFA salt; 50.0 mg, 0.148 mmol, 2 eq) and DIEA (129 μL, 0.741 mmol, 10 eq) in CH_2Cl_2 (3 mL), and the reaction was stirred at room temperature for 18 h. The solvent was removed by rotary evaporation, and the crude material was purified by reverse phase HPLC (20–70% MeCN/H_2O, linear gradient, with constant 0.1% v/v TFA additive) to afford **25$_{HTL}$** as a blue solid (40.4 mg, 60%, TFA salt). ^{1}H NMR (CD_3OD, 400 MHz) δ 9.12 (t, J = 5.4 Hz, 1H), 7.16 (dd, J = 9.4, 1.0 Hz, 2H), 7.14 (d, J = 2.7 Hz, 2H), 6.64 (dd, J = 9.4, 2.7 Hz, 2H), 3.67 – 3.54 (m, 8H), 3.50 (t, J = 6.6 Hz, 2H), 3.43 (t, J = 6.5 Hz, 2H), 1.75 – 1.66 (m, 2H), 1.55 – 1.47 (m, 2H), 1.44 – 1.27 (m, 4H), 0.58 (s, 3H), 0.54 (s, 3H); ^{19}F NMR (CD_3OD, 376 MHz) δ -75.24 (s, 3F), -118.19 (d, J = 16.4 Hz, 1F), -135.10 (d, J = 22.6 Hz, 1F), -142.57 – -142.90 (m, 1F); Analytical HPLC: t_R = 13.5 min, >99% purity (10–95% MeCN/H_2O, linear gradient, with constant 0.1% v/v TFA additive; 20 min run; 1 mL/min flow; ESI; positive ion mode; detection at 675 nm); HRMS (ESI) calcd for $C_{41}H_{34}D_{16}ClF_3N_3O_5Si$ $[M+H]^+$ 800.4159, found 800.4167.

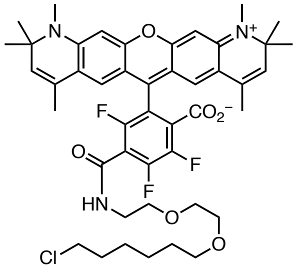

4-((2-(2-((6-Chlorohexyl)oxy)ethoxy)ethyl)carbamoyl)-2,3,5-trifluoro-6-(1,2,2,4,8,10,10,11-octamethyl-1,2,10,11-tetrahydropyrano[3,2-*g*:5,6-*g'*]diquinolin-13-ium-6-yl)benzoate (11$_{HTL}$): Rhodamine **11$_{MAC}$** (acetate salt; 60 mg, 79.3 μmol) was taken up in CH_2Cl_2 (5 mL), and trifluoroacetic acid (1 mL) was added. The reaction was stirred at room temperature for 18 h. Toluene (5 mL) was added, and the reaction mixture was concentrated to dryness. The residue was combined with a premixed solution of HaloTag(O2)amine (HTL-NH_2, **111**; TFA salt; 53.6 mg, 0.159 mmol, 2 eq) and DIEA (138 μL, 0.793 mmol, 10 eq) in DMF (4 mL), and the reaction was stirred at room temperature for 2 h. The solvent was removed by rotary evaporation, and the crude material was purified by reverse phase HPLC (40–60% MeCN/H_2O, linear gradient, with constant 0.1% v/v TFA additive) to afford **11$_{HTL}$** as a purple solid (37.3 mg, 50%, TFA salt). ^{1}H NMR (CD_3OD, 400 MHz) δ 9.12 (t, J = 5.4 Hz, 1H), 6.89 (s, 2H), 6.84 (s, 2H), 5.74 – 5.70

(m, 2H), 3.67 – 3.55 (m, 8H), 3.50 (t, *J* = 6.7 Hz, 2H), 3.42 (t, *J* = 6.5 Hz, 2H), 3.21 (s, 6H), 1.92 (d, *J* = 1.4 Hz, 6H), 1.73 – 1.66 (m, 2H), 1.525 (s, 6H), 1.523 (s, 6H), 1.50 – 1.45 (m, 2H), 1.41 – 1.28 (m, 4H); ^{19}F NMR (CD_3OD, 376 MHz) δ -75.36 (s, 3F), -115.98 (d, *J* = 15.4 Hz, 1F), -131.78 (d, *J* = 22.2 Hz, 1F), -139.85 – -140.17 (m, 1F); Analytical HPLC: t_R = 14.1 min, >99% purity (10–95% MeCN/H_2O, linear gradient, with constant 0.1% v/v TFA additive; 20 min run; 1 mL/min flow; ESI; positive ion mode; detection at 600 nm); HRMS (ESI) calcd for $C_{45}H_{52}ClF_3N_3O_6$ $[M+H]^+$ 822.3491, found 822.3490.

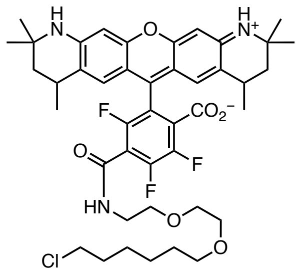

JF$_{563}$–HaloTag ligand (18$_{HTL}$): 6-(MOM-MAC)-JF$_{563}$ (**18$_{MAC}$**, acetate salt; 25 mg, 34.1 μmol) was taken up in CH_2Cl_2 (2 mL); anisole (200 μL) was added, followed by trifluoroacetic acid (400 μL). The reaction was stirred at room temperature for 24 h. Toluene (3 mL) was added, and the reaction mixture was concentrated to dryness. The residue was combined with a premixed solution of HaloTag(O2)amine (HTL-NH_2, **111**; TFA salt; 23.0 mg, 68.2 μmol, 2 eq) and DIEA (59.4 μL, 0.341 mmol, 10 eq) in DMF (2 mL), and the reaction was stirred at room temperature for 3 h. The solvent was removed by rotary evaporation, and the crude material was purified by reverse phase HPLC (35–65% MeCN/H_2O, linear gradient, with constant 0.1% v/v TFA additive) to yield 17.3 mg (56%, TFA salt) of **18$_{HTL}$** as a purple solid (mixture of diastereomers). ^{1}H NMR (CD_3OD, 400 MHz) δ 9.19 – 9.08 (m, 1H), 7.15 – 7.05 (m, 2H), 6.67 – 6.61 (m, 2H), 3.70 – 3.52 (m, 8H), 3.53 – 3.47 (m, 2H), 3.45 – 3.40 (m, 2H), 2.98 – 2.87 (m, 2H), 1.95 – 1.86 (m, 2H), 1.75 – 1.66 (m, 2H), 1.54 – 1.19 (m, 26H); ^{19}F NMR (CD_3OD, 376 MHz) δ -75.35 (s, 3F), -116.07 – -116.23 (m, 1F), -132.05 – -132.22 (m, 1F), -139.69 – -140.86 (m, 1F); Analytical HPLC: t_R (three isomers) = 13.7 min, 13.8 min, 14.0 min; >99% total purity (10–95% MeCN/H_2O, linear gradient, with constant 0.1% v/v TFA additive; 20 min run; 1 mL/min flow; ESI; positive ion mode; detection at 550 nm); HRMS (ESI) calcd for $C_{43}H_{52}ClF_3N_3O_6$ $[M+H]^+$ 798.3491, found 798.3488.

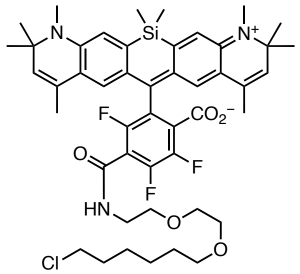

4-((2-(2-((6-Chlorohexyl)oxy)ethoxy)ethyl)carbamoyl)-2-(1,2,2,4,8,10,10,11,13,13-decamethyl-2,10,11,13-tetra-hydrosilino[3,2-*g*:5,6-*g'*]diquinolin-6-ylium-6(1*H*)-yl)-3,5,6-trifluorobenzoate (38$_{HTL}$): Si-rhodamine **38$_{MAC}$** (50 mg, 67.7 μmol) was taken up in CH_2Cl_2 (4 mL), and trifluoroacetic acid (800 μL) was added. The reaction was stirred

at room temperature for 18 h. Toluene (5 mL) was added, and the reaction mixture was concentrated to dryness. The residue was combined with a premixed solution of HaloTag(O2)amine (HTL-NH_2, **111**; TFA salt; 45.7 mg, 0.135 mmol, 2 eq) and DIEA (118 µL, 0.677 mmol, 10 eq) in CH_2Cl_2 (4 mL), and the reaction was stirred at room temperature for 1 h. The solvent was removed by rotary evaporation, and the crude material was purified by flash chromatography on silica gel (10–100% EtOAc/hexanes, linear gradient) to afford **38$_{HTL}$** as a yellow-green solid (36.3 mg, 62%). ^{1}H NMR ($CDCl_3$, 400 MHz) δ 6.622 (s, 2H), 6.621 (s, 1H), 6.59 (s, 2H), 5.28 (q, *J* = 1.3 Hz, 2H), 3.64 – 3.56 (m, 6H), 3.53 – 3.47 (m, 4H), 3.33 (t, *J* = 6.6 Hz, 2H), 2.86 (s, 6H), 1.79 (d, *J* = 1.4 Hz, 6H), 1.77 – 1.69 (m, 2H), 1.50 – 1.35 (m, 4H), 1.31 (s, 12H), 1.30 – 1.25 (m, 2H), 0.51 (s, 3H), 0.51 (s, 3H); ^{19}F NMR ($CDCl_3$, 376 MHz) δ -119.92 (d, *J* = 22.8 Hz, 1F), -134.05 (d, *J* = 21.5 Hz, 1F), -142.39 (t, *J* = 22.2 Hz, 1F); Analytical HPLC: t_R = 11.9 min, 98.2% purity (30–95% MeCN/H_2O, linear gradient, with constant 0.1% v/v TFA additive; 20 min run; 1 mL/min flow; ESI; positive ion mode; detection at 750 nm); HRMS (ESI) calcd for $C_{47}H_{58}ClF_3N_3O_5Si$ $[M+H]^+$ 864.3781, found 864.3777.

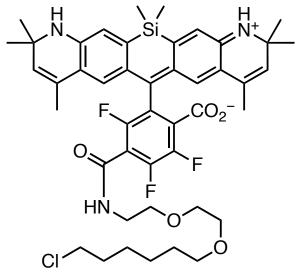

4-((2-(2-((6-Chlorohexyl)oxy)ethoxy)ethyl)carbamoyl)-2,3,5-trifluoro-6-(2,2,4,8,10,10,13,13-octamethyl-2,10,11,13-tetrahydrosilino[3,2-*g*:5,6-*g'*]diquinolin-6-ylium-6(1*H*)-yl)benzoate (36$_{HTL}$): Si-rhodamine **36$_{MAC}$** (20 mg, 28.1 µmol) was taken up in CH_2Cl_2 (2 mL); anisole (200 µL) was added, followed by trifluoroacetic acid (400 µL). The reaction was stirred at room temperature for 18 h. Toluene (2 mL) was added, and the reaction mixture was concentrated to dryness. The residue was combined with a premixed solution of HaloTag(O2)amine (HTL-NH_2, **111**; TFA salt; 19.0 mg, 56.3 µmol, 2 eq) and DIEA (49.0 µL, 0.281 mmol, 10 eq) in DMF (2 mL), and the reaction was stirred at room temperature for 2 h. The solvent was removed by rotary evaporation, and the crude material was purified by reverse phase HPLC (30–60% MeCN/H_2O, linear gradient, with constant 0.1% v/v TFA additive) to afford **36$_{HTL}$** as a green solid (12.9 mg, 48%, TFA salt). ^{1}H NMR (CD_3OD, 400 MHz) δ 9.16 (t, *J* = 5.5 Hz, 1H), 6.96 (s, 2H), 6.82 (s, 2H), 5.55 (q, *J* = 1.2 Hz, 2H), 3.68 – 3.54 (m, 8H), 3.51 (t, *J* = 6.6 Hz, 2H), 3.43 (t, *J* = 6.5 Hz, 2H), 1.78 (d, *J* = 1.4 Hz, 6H), 1.75 – 1.67 (m, 2H), 1.51 (p, *J* = 6.8 Hz, 2H), 1.45 – 1.28 (m, 4H), 1.414 (s, 6H), 1.405 (s, 6H), 0.52 (s, 3H), 0.48 (s, 3H); ^{19}F NMR (CD_3OD, 376 MHz) δ -75.33 (s, 3F), -116.54 (d, *J* = 15.6 Hz, 1F), -133.68 (d, *J* = 21.7 Hz, 1F), -141.14 (dd, *J* = 22.1, 15.5 Hz, 1F); Analytical HPLC: t_R = 14.0 min, 99.0% purity (10–95% MeCN/H_2O, linear gradient, with constant 0.1% v/v TFA additive; 20 min run; 1 mL/min flow; ESI; positive ion mode; detection at 725 nm); HRMS (ESI) calcd for $C_{45}H_{54}ClF_3N_3O_5Si$ $[M+H]^+$ 836.3468, found 836.3472.

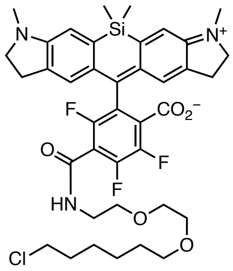

SiRF$_{712}$–HaloTag ligand (108$_{HTL}$): 6-(MOM-MAC)-SiRF$_{712}$ (**108$_{MAC}$**; 40 mg, 63.4 µmol) was taken up in CH_2Cl_2 (3 mL); triethylsilane (300 µL) was added, followed by trifluoroacetic acid (600 µL). The reaction was stirred at room temperature for 6 h. Toluene (4 mL) was added, and the reaction mixture was concentrated to dryness. The residue was combined with a premixed solution of HaloTag(O2)amine (HTL-NH_2, **111**; TFA salt; 42.8 mg, 0.127 mmol, 2 eq) and DIEA (110 µL, 0.634 mmol, 10 eq) in DMF (3 mL), and the reaction was stirred at room temperature for 18 h. The solvent was removed by rotary evaporation, and the crude material was purified by reverse phase HPLC (30–50% MeCN/H_2O, linear gradient, with constant 0.1% v/v TFA additive) to afford **108$_{HTL}$** as a blue-green solid (29.9 mg, 54%, TFA salt). ^{1}H NMR (CD_3OD, 400 MHz) δ 7.10 (s, 2H), 6.81 (s, 2H), 3.83 (t, J = 8.0 Hz, 4H), 3.68 – 3.55 (m, 8H), 3.51 (t, J = 6.7 Hz, 2H), 3.44 (t, J = 6.5 Hz, 2H), 3.22 (s, 6H), 3.08 – 2.93 (m, 4H), 1.76 – 1.67 (m, 2H), 1.56 – 1.48 (m, 2H), 1.45 – 1.28 (m, 4H), 0.57 (s, 3H), 0.51 (s, 3H); ^{19}F NMR (CD_3OD, 376 MHz) δ -75.29 (s, 3F), -118.27 (d, J = 15.5 Hz, 1F), -135.07 (d, J = 22.5 Hz, 1F), -141.70 (dd, J = 22.3, 15.9 Hz, 1F); Analytical HPLC: t_R = 12.8 min, >99% purity (10–95% MeCN/H_2O, linear gradient, with constant 0.1% v/v TFA additive; 20 min run; 1 mL/min flow; ESI; positive ion mode; detection at 725 nm); HRMS (ESI) calcd for $C_{39}H_{46}ClF_3N_3O_5Si$ $[M+H]^+$ 756.2842, found 756.2845.

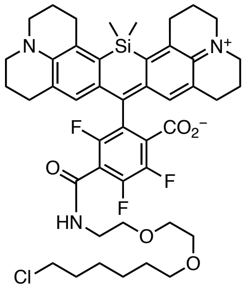

JF$_{698}$–HaloTag ligand (31$_{HTL}$): 6-(MOM-MAC)-JF$_{698}$ (**31$_{MAC}$**; 35 mg, 49.2 µmol) was taken up in CH_2Cl_2 (3 mL); triethylsilane (300 µL) was added, followed by trifluoroacetic acid (600 µL). The reaction was stirred at room temperature for 6 h. Toluene (4 mL) was added, and the reaction mixture was concentrated to dryness. The residue was combined with a premixed solution of HaloTag(O2)amine (HTL-NH_2, **111**; TFA salt; 33.3 mg, 98.5 µmol, 2 eq) and DIEA (85.8 µL, 0.492 mmol, 10 eq) in DMF (3 mL), and the reaction was stirred at room temperature for 18 h. The solvent was removed by rotary evaporation, and the crude material was purified by reverse phase HPLC (30–60% MeCN/H_2O, linear gradient, with constant 0.1% v/v TFA additive) to afford **31$_{HTL}$** as a blue-green solid (27.9 mg, 60%, TFA salt). ^{1}H NMR (CD_3OD, 400 MHz) δ 9.17 (t, J = 5.3 Hz, 1H), 6.66 (s, 2H), 3.68 – 3.54 (m, 16H), 3.52 (t, J = 6.7 Hz, 2H), 3.45 (t, J = 6.5 Hz, 2H), 3.01 – 2.94 (m, 4H), 2.63 – 2.56 (m, 4H), 2.13 – 2.02 (m, 4H), 1.98 – 1.88 (m, 4H), 1.77 – 1.68 (m, 2H), 1.57 – 1.49 (m, 2H), 1.46 – 1.30 (m, 4H), 0.71 (s, 3H), 0.67 (s, 3H); ^{19}F NMR (CD_3OD,

376 MHz) δ -75.37 (s, 3F), -117.65 (dd, J = 15.0, 2.0 Hz, 1F), -134.78 (dd, J = 21.9, 2.0 Hz, 1F), -141.18 (dd, J = 22.3, 15.1 Hz, 1F); Analytical HPLC: t_R = 13.6 min, >99% purity (10–95% MeCN/H_2O, linear gradient, with constant 0.1% v/v TFA additive; 20 min run; 1 mL/min flow; ESI; positive ion mode; detection at 700 nm); HRMS (ESI) calcd for $C_{45}H_{54}ClF_3N_3O_5Si$ $[M+H]^+$ 836.3468, found 836.3476.

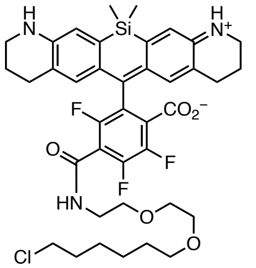

FSiRh$_Q$–HaloTag ligand (29$_{HTL}$): 6-(MOM-MAC)-FSiRh$_Q$ (**29$_{MAC}$**; 40 mg, 63.4 μmol) was taken up in CH_2Cl_2 (3 mL); triethylsilane (300 μL) was added, followed by trifluoroacetic acid (600 μL). The reaction was stirred at room temperature for 8 h. Toluene (4 mL) was added, and the reaction mixture was concentrated to dryness. The residue was combined with a premixed solution of HaloTag(O2)amine (HTL-NH_2, **111**; TFA salt; 42.8 mg, 0.127 mmol, 2 eq) and DIEA (110 μL, 0.634 mmol, 10 eq) in DMF (3 mL), and the reaction was stirred at room temperature for 18 h. The solvent was removed by rotary evaporation, and the crude material was purified by reverse phase HPLC (30–50% MeCN/H_2O, linear gradient, with constant 0.1% v/v TFA additive) to afford **29$_{HTL}$** as a deep blue solid (23.2 mg, 42%, TFA salt). ^1H NMR (CD_3OD, 400 MHz) δ 9.16 (t, J = 5.3 Hz, 1H), 6.98 (s, 2H), 6.82 (s, 2H), 3.68 – 3.56 (m, 8H), 3.54 – 3.48 (m, 6H), 3.44 (t, J = 6.5 Hz, 2H), 2.66 – 2.59 (m, 4H), 1.95 – 1.87 (m, 4H), 1.76 – 1.68 (m, 2H), 1.56 – 1.48 (m, 2H), 1.44 – 1.29 (m, 4H), 0.49 (s, 3H), 0.43 (s, 3H); ^{19}F NMR (CD_3OD, 376 MHz) δ -75.32 (s, 3F), -117.66 (d, J = 15.2 Hz, 1F), -134.57 (d, J = 22.5 Hz, 1F), -141.14 (dd, J = 22.3, 15.2 Hz, 1F); Analytical HPLC: t_R = 12.6 min, >99% purity (10–95% MeCN/H_2O, linear gradient, with constant 0.1% v/v TFA additive; 20 min run; 1 mL/min flow; ESI; positive ion mode; detection at 675 nm); HRMS (ESI) calcd for $C_{39}H_{46}ClF_3N_3O_5Si$ $[M+H]^+$ 756.2842, found 756.2849.

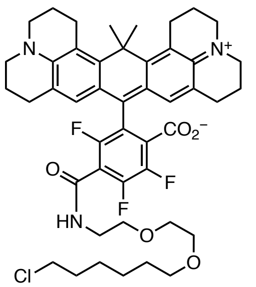

JF$_{660}$–HaloTag ligand (57$_{HTL}$): 6-(MOM-MAC)-JF$_{660}$ (**57$_{MAC}$**; 60 mg, 86.4 μmol) was taken up in CH_2Cl_2 (3 mL); triethylsilane (300 μL) was added, followed by trifluoroacetic acid (600 μL). The reaction was stirred at room temperature for 8 h. Toluene (4 mL) was added, and the reaction mixture was concentrated to dryness. The residue was combined with a premixed solution of HaloTag(O2)amine (HTL-NH_2, **111**; TFA salt; 58.3 mg, 0.173 mmol, 2 eq) and DIEA (150 μL, 0.864 mmol, 10 eq) in CH_2Cl_2 (3 mL), and the reaction was stirred at room temperature for

18 h. The solvent was removed by rotary evaporation, and the crude material was purified by reverse phase HPLC (20–60% MeCN/H_2O, linear gradient, with constant 0.1% v/v TFA additive) to afford **57$_{HTL}$** as a blue solid (33.0 mg, 41%, TFA salt). ^1H NMR (CD_3OD, 400 MHz) δ 9.15 (t, J = 5.2 Hz, 1H), 6.64 (s, 2H), 3.68 – 3.50 (m, 18H), 3.44 (t, J = 6.5 Hz, 2H), 3.13 – 3.02 (m, 4H), 2.67 – 2.60 (m, 4H), 2.10 – 1.99 (m, 4H), 2.04 (s, 3H), 1.98 – 1.90 (m, 4H), 1.96 (s, 3H), 1.76 – 1.68 (m, 2H), 1.57 – 1.49 (m, 2H), 1.45 – 1.30 (m, 4H); ^{19}F NMR (CD_3OD, 376 MHz) δ -75.36 (s, 3F), -117.34 (d, J = 15.1 Hz, 1F), -134.31 (d, J = 22.5 Hz, 1F), -140.92 (dd, J = 22.2, 15.3 Hz, 1F); Analytical HPLC: t_R = 13.5 min, >99% purity (10–95% MeCN/H_2O, linear gradient, with constant 0.1% v/v TFA additive; 20 min run; 1 mL/min flow; ESI; positive ion mode; detection at 650 nm); HRMS (ESI) calcd for $C_{46}H_{54}ClF_3N_3O_5$ $[M+H]^+$ 820.3699, found 820.3688.

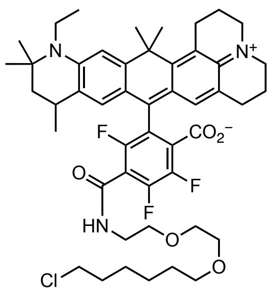

JF$_{657}$–HaloTag ligand (59$_{HTL}$): 6-(MOM-MAC)-JF$_{657}$ (**59$_{MAC}$**; 55 mg, 75.9 μmol) was taken up in CH_2Cl_2 (3 mL); triethylsilane (300 μL) was added, followed by trifluoroacetic acid (600 μL). The reaction was stirred at room temperature for 8 h. Toluene (4 mL) was added, and the reaction mixture was concentrated to dryness. The residue was combined with a premixed solution of HaloTag(O2)amine (HTL-NH_2, **111**; TFA salt; 51.3 mg, 0.152 mmol, 2 eq) and DIEA (132 μL, 0.759 mmol, 10 eq) in CH_2Cl_2 (3 mL), and the reaction was stirred at room temperature for 18 h. The solvent was removed by rotary evaporation, and the crude material was purified by reverse phase HPLC (30–60% MeCN/H_2O, linear gradient, with constant 0.1% v/v TFA additive) to provide 33.0 mg (45%, TFA salt) of **59$_{HTL}$** as a blue solid (mixture of two diastereomers). ^1H NMR (CD_3OD, 400 MHz) δ 9.18 (t, J = 5.1 Hz, 0.5H), 9.12 (t, J = 5.4 Hz, 0.5H), 6.95 (s, 1H), 6.86 – 6.79 (m, 2H), 3.84 (dq, J = 14.4, 6.8 Hz, 1H), 3.71 – 3.56 (m, 13H), 3.51 (t, J = 6.6 Hz, 2H), 3.45 (t, J = 6.5 Hz, 1H), 3.44 (t, J = 6.6 Hz, 1H), 3.25 – 3.17 (m, 2H), 2.86 – 2.75 (m, 1H), 2.72 – 2.63 (m, 2H), 2.14 – 2.02 (m, 2H), 1.96 (s, 2H), 1.93 – 1.86 (m, 1H), 1.92 (s, 1.5H), 1.91 (s, 1.5H), 1.84 (s, 1.5H), 1.83 (s, 1.5H), 1.72 (p, J = 6.9 Hz, 2H), 1.59 – 1.47 (m, 3H), 1.49 (s, 3H), 1.46 – 1.28 (m, 4H), 1.362 (s, 1.5H), 1.361 (t, J = 6.9 Hz, 3H), 1.35 (s, 1.5H), 1.174 (d, J = 6.6 Hz, 1.5H), 1.165 (d, J = 6.4 Hz, 1.5H); ^{19}F NMR (CD_3OD, 376 MHz) δ -75.38 (s, 3F), -116.63 (d, J = 15.1 Hz, 0.5F), -116.78 (d, J = 15.4 Hz, 0.5F), -133.56 (d, J = 22.2 Hz, 0.5F), -133.70 (d, J = 22.5 Hz, 0.5F), -140.50 (dd, J = 22.0, 15.3 Hz, 0.5F), -140.72 (dd, J = 22.0, 14.9 Hz, 0.5F); Analytical HPLC: t_R (two isomers) = 14.2 min, 14.4 min; >99% total purity (10–95% MeCN/H_2O, linear gradient, with constant 0.1% v/v TFA additive; 20 min run; 1 mL/min flow; ESI; positive ion mode; detection at 625 nm); HRMS (ESI) calcd for $C_{48}H_{60}ClF_3N_3O_5$ $[M+H]^+$ 850.4168, found 850.4160.

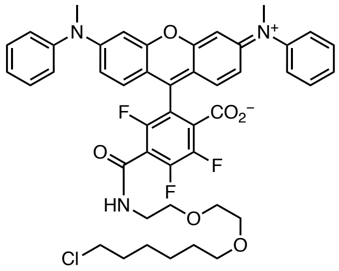

2-(3,6-Bis(methyl(phenyl)amino)xanthylium-9-yl)-4-((2-(2-((6-chlorohexyl)oxy)ethoxy)ethyl)carbamoyl)-3,5,6-trifluorobenzoate ($\mathbf{64_{HTL}}$): 2-(3,6-Bis(methyl(phenyl)amino)xanthylium-9-yl)-4-(dicyano(methoxymethoxy)-methyl)-3,5,6-trifluorobenzoate ($\mathbf{64_{MAC}}$; 100 mg, 0.145 mmol) was taken up in CH_2Cl_2 (10 mL); triethylsilane (1 mL) was added, followed by trifluoroacetic acid (2 mL). The reaction was stirred at room temperature for 6 h. Toluene (10 mL) was added, and the reaction mixture was concentrated to dryness. The residue was combined with a premixed solution of HaloTag(O2)amine (HTL-NH_2, **111**; TFA salt; 98.1 mg, 0.290 mmol, 2 eq) and DIEA (253 µL, 1.45 mmol, 10 eq) in CH_2Cl_2 (7 mL), and the reaction was stirred at room temperature for 1 h. The solvent was removed by rotary evaporation, and the crude material was purified by silica gel chromatography (25–100% EtOAc/toluene, linear gradient) to yield 78.7 mg (67%) of $\mathbf{64_{HTL}}$ as a dark purple solid. ^{1}H NMR ($CDCl_3$, 400 MHz) δ 7.41 – 7.35 (m, 4H), 7.22 – 7.16 (m, 6H), 6.77 (s, 1H), 6.72 (d, J = 8.9 Hz, 2H), 6.59 (d, J = 2.4 Hz, 2H), 6.54 (dd, J = 8.8, 2.5 Hz, 2H), 3.65 – 3.57 (m, 6H), 3.54 – 3.48 (m, 4H), 3.37 (t, J = 6.7 Hz, 2H), 3.34 (s, 6H), 1.77 – 1.69 (m, 2H), 1.53 – 1.45 (m, 2H), 1.44 – 1.36 (m, 2H), 1.34 – 1.26 (m, 2H); ^{19}F NMR ($CDCl_3$, 376 MHz) δ -121.01 (d, J = 22.5 Hz, 1F), -132.34 (d, J = 21.6 Hz, 1F), -142.02 (t, J = 22.0 Hz, 1F); Analytical HPLC: t_R = 11.5 min, >99% purity (30–95% MeCN/H_2O, linear gradient, with constant 0.1% v/v TFA additive; 20 min run; 1 mL/min flow; ESI; positive ion mode; detection at 550 nm); HRMS (ESI) calcd for $C_{45}H_{44}ClF_3N_3O_6$ $[M+H]^+$ 814.2865, found 814.2858.

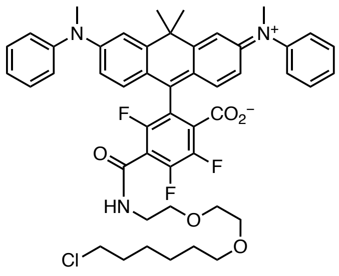

$\mathbf{JQ_{645}}$–HaloTag ligand ($\mathbf{65_{HTL}}$): 4-(Dicyano(methoxymethoxy)methyl)-2-(10,10-dimethyl-3,6-bis(methyl(phenyl)-amino)anthracen-9-ylium-9(10*H*)-yl)-3,5,6-trifluorobenzoate ($\mathbf{65_{MAC}}$; 50 mg, 70.0 µmol) was taken up in CH_2Cl_2 (4 mL); triethylsilane (400 µL) was added, followed by trifluoroacetic acid (800 µL). The reaction was stirred at room temperature for 6 h. Toluene (4 mL) was added, and the reaction mixture was concentrated to dryness. The residue was combined with a premixed solution of HaloTag(O2)amine (HTL-NH_2, **111**; TFA salt; 47.3 mg, 0.140 mmol, 2 eq) and DIEA (122 µL, 0.700 mmol, 10 eq) in CH_2Cl_2 (4 mL), and the reaction was stirred at room temperature for 18 h. The solvent was removed by rotary evaporation, and the crude material was purified by silica gel chromatography (5–75% EtOAc/hexanes, linear gradient) to yield 43.0 mg (73%) of $\mathbf{65_{HTL}}$ as a pale blue-green solid. ^{1}H NMR ($CDCl_3$, 400 MHz) δ 7.37 – 7.30 (m, 4H), 7.18 – 7.12 (m, 4H), 7.11 – 7.05 (m, 4H), 6.75 (dd, J = 8.7, 2.4 Hz, 2H), 6.72 (bs,

1H), 6.71 (d, J = 8.7 Hz, 2H), 3.65 – 3.57 (m, 6H), 3.54 – 3.47 (m, 4H), 3.37 (t, J = 6.6 Hz, 2H), 3.36 (s, 6H), 1.78 – 1.69 (m, 2H), 1.63 (s, 6H), 1.54 – 1.46 (m, 2H), 1.45 – 1.37 (m, 2H), 1.34 – 1.27 (m, 2H); ^{19}F NMR ($CDCl_3$, 376 MHz) δ -120.93 (d, J = 22.7 Hz, 1F), -133.20 (d, J = 21.8 Hz, 1F), -142.37 (t, J = 22.2 Hz, 1F); Analytical HPLC: t_R = 15.0 min, >99% purity (30–95% MeCN/H_2O, linear gradient, with constant 0.1% v/v TFA additive; 20 min run; 1 mL/min flow; ESI; positive ion mode; detection at 650 nm); HRMS (ESI) calcd for $C_{48}H_{50}ClF_3N_3O_5$ $[M+H]^+$ 840.3386, found 840.3377.

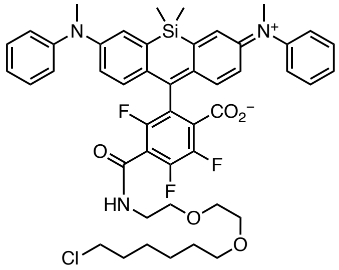

4-((2-(2-((6-Chlorohexyl)oxy)ethoxy)ethyl)carbamoyl)-2-(5,5-dimethyl-3,7-bis(methyl(phenyl)amino)dibenzo-[*b*,*e*]silin-10-ylium-10(5*H*)-yl)-3,5,6-trifluorobenzoate (66$_{\text{HTL}}$): 4-(Dicyano(methoxymethoxy)methyl)-2-(5,5-dimethyl-3,7-bis(methyl(phenyl)amino)dibenzo[*b*,*e*]silin-10-ylium-10(5*H*)-yl)-3,5,6-trifluorobenzoate (**66**$_{\text{MAC}}$; 40 mg, 54.7 μmol) was taken up in CH_2Cl_2 (3 mL); triethylsilane (300 μL) was added, followed by trifluoroacetic acid (600 μL). The reaction was stirred at room temperature for 6 h. Toluene (3 mL) was added, and the reaction mixture was concentrated to dryness. The residue was combined with a premixed solution of HaloTag(O2)amine (HTL-NH_2, **111**; TFA salt; 37.0 mg, 0.109 mmol, 2 eq) and DIEA (95.3 μL, 0.547 mmol, 10 eq) in CH_2Cl_2 (3 mL), and the reaction was stirred at room temperature for 18 h. The solvent was removed by rotary evaporation, and the crude material was purified by silica gel chromatography (5–75% EtOAc/hexanes, linear gradient) to yield 24.4 mg (52%) of **66**$_{\text{HTL}}$ as an off-white solid. ^{1}H NMR ($CDCl_3$, 400 MHz) δ 7.36 – 7.29 (m, 4H), 7.19 – 7.11 (m, 6H), 7.11 – 7.05 (m, 2H), 6.86 (s, 1H), 6.84 – 6.77 (m, 4H), 3.67 – 3.58 (m, 6H), 3.55 – 3.51 (m, 2H), 3.49 (t, J = 6.7 Hz, 2H), 3.37 (t, J = 6.7 Hz, 2H), 3.35 (s, 6H), 1.76 – 1.68 (m, 2H), 1.52 – 1.44 (m, 2H), 1.43 – 1.35 (m, 2H), 1.32 – 1.25 (m, 2H), 0.49 (s, 3H), 0.45 (s, 3H); ^{19}F NMR ($CDCl_3$, 376 MHz) δ -118.19 (d, J = 22.7 Hz, 1F), -133.12 (d, J = 21.4 Hz, 1F), -141.91 (t, J = 22.1 Hz, 1F); Analytical HPLC: t_R = 15.9 min, 98.8% purity (50–95% MeCN/H_2O, linear gradient, with constant 0.1% v/v TFA additive; 20 min run; 1 mL/min flow; ESI; positive ion mode; detection at 675 nm); HRMS (ESI) calcd for $C_{47}H_{50}ClF_3N_3O_5Si$ $[M+H]^+$ 856.3155, found 856.3150.

SYNTHESIS OF OTHER HALOTAG AND SNAP-TAG LIGANDS

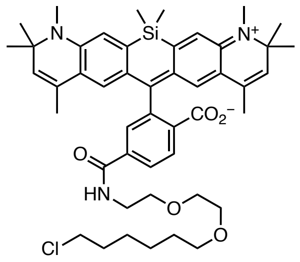

4-((2-(2-((6-Chlorohexyl)oxy)ethoxy)ethyl)carbamoyl)-2-(1,2,2,4,8,10,10,11,13,13-decamethyl-2,10,11,13-tetrahydrosilino[3,2-*g*:5,6-*g*']diquinolin-6-ylium-6(1*H*)-yl)benzoate ($\mathbf{104_{HTL}}$): 4-Carboxy-2-(1,2,2,4,8,10,10,11,13,13-decamethyl-2,10,11,13-tetrahydrosilino[3,2-*g*:5,6-*g*']diquinolin-6-ylium-6(1*H*)-yl)benzoate (**110**; 40 mg, 66.1 μmol) was combined with DSC (40.7 mg, 0.159 mmol, 2.4 eq) in DMF (3 mL). After adding Et_3N (55.3 μL, 0.397 mmol, 6 eq) and DMAP (0.8 mg, 6.6 μmol, 0.1 eq), the reaction was stirred at room temperature for 30 min. A solution of HaloTag(O2)amine (HTL–NH_2, **111**; TFA salt; 53.6 mg, 0.159 mmol, 2.4 eq) in DMF (200 μL) was then added. The reaction was stirred an additional 3 h at room temperature. Purification of the crude reaction mixture by reverse phase HPLC (30–95% MeCN/H_2O, linear gradient, with constant 0.1% v/v TFA additive) afforded 39.4 mg (64%, TFA salt) of $\mathbf{104_{HTL}}$ as a green solid. ^{1}H NMR (CD_3OD, 400 MHz) δ 8.74 (t, *J* = 5.5 Hz, 1H), 8.27 (d, *J* = 8.1 Hz, 1H), 8.12 (dd, *J* = 8.2, 1.7 Hz, 1H), 7.72 (d, *J* = 1.6 Hz, 1H), 7.10 (s, 2H), 6.61 (s, 2H), 5.46 (q, *J* = 1.2 Hz, 2H), 3.65 (t, *J* = 5.5 Hz, 2H), 3.62 – 3.53 (m, 6H), 3.50 (t, *J* = 6.6 Hz, 2H), 3.41 (t, *J* = 6.5 Hz, 2H), 3.20 (s, 6H), 1.70 (dq, *J* = 8.0, 6.7 Hz, 2H), 1.55 (d, *J* = 1.4 Hz, 6H), 1.52 – 1.47 (m, 2H), 1.45 (s, 6H), 1.43 (s, 6H), 1.41 – 1.28 (m, 4H), 0.63 (s, 3H), 0.58 (s, 3H); Analytical HPLC: t_R = 11.5 min, >99% purity (30–95% MeCN/H_2O, linear gradient, with constant 0.1% v/v TFA additive; 20 min run; 1 mL/min flow; ESI; positive ion mode; detection at 725 nm); HRMS (ESI) calcd for $C_{47}H_{61}ClN_3O_5Si$ $[M+H]^+$ 810.4064, found 810.4046.

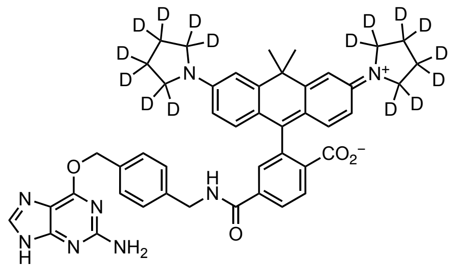

$\mathbf{JFX_{612}}$–SNAP-tag ligand ($\mathbf{88_{STL}}$): JFX_{612}-NHS[5] (25 mg, 40.2 μmol) and 6-((4-(aminomethyl)benzyl)oxy)-9*H*-purin-2-amine (BG–NH_2; 16.3 mg, 60.3 μmol, 1.5 eq) were combined in DMF (1.5 mL), and DIEA (21.0 μL, 0.121 mmol, 3 eq) was added. After stirring the reaction at room temperature for 4 h, it was directly purified by reverse phase HPLC (10–50% MeCN/H_2O, linear gradient, with constant 0.1% v/v TFA additive) to yield 33.6 mg (94%, TFA salt) of $\mathbf{88_{STL}}$ as a purple solid. ^{1}H NMR (CD_3OD, 400 MHz) δ 9.29 (t, *J* = 6.1 Hz, 1H), 8.35 (d, *J* = 8.2 Hz, 1H), 8.23 (s, 1H), 8.16 (dd, *J* = 8.3, 1.8 Hz, 1H), 7.78 (d, *J* = 1.7 Hz, 1H), 7.51 (d, *J* = 8.2 Hz, 2H), 7.40 (d, *J* = 8.2 Hz, 2H), 7.09 (d, *J* = 2.4 Hz, 2H), 6.98 (d, *J* = 9.2 Hz, 2H), 6.65 (dd, *J* = 9.3, 2.4 Hz, 2H), 5.60 (s, 2H), 4.60 (d, *J* = 6.0 Hz, 2H), 1.86 (s,

3H), 1.75 (s, 3H); Analytical HPLC: t_R = 10.3 min, >99% purity (10–95% MeCN/H_2O, linear gradient, with constant 0.1% v/v TFA additive; 20 min run; 1 mL/min flow; ESI; positive ion mode; detection at 600 nm); HRMS (ESI) calcd for $C_{45}H_{29}D_{16}N_8O_4$ $[M+H]^+$ 777.4563, found 777.4567.

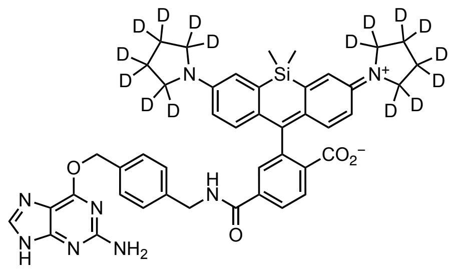

JFX$_{650}$–SNAP-tag ligand (89$_{STL}$): JFX$_{650}$-NHS[5] (75 mg, 0.118 mmol) and 6-((4-(aminomethyl)benzyl)oxy)-9*H*-purin-2-amine (BG–NH_2; 47.7 mg, 0.176 mmol, 1.5 eq) were combined in DMF (5 mL), and DIEA (61.4 µL, 0.353 mmol, 3 eq) was added. After stirring the reaction at room temperature for 18 h, it was diluted with saturated $NaHCO_3$ and extracted with 10% MeOH/CH_2Cl_2 (3×). The combined organic extracts were dried over anhydrous $MgSO_4$, filtered, and evaporated. Flash chromatography on silica gel (0–10% MeOH/EtOAc, linear gradient) provided **89$_{STL}$** as a blue solid (73 mg, 78%). ^{1}H NMR (CD_3OD, 400 MHz) δ 8.02 (dd, *J* = 8.0, 1.4 Hz, 1H), 7.98 (dd, *J* = 8.0, 0.5 Hz, 1H), 7.81 (s, 1H), 7.69 – 7.67 (m, 1H), 7.42 (d, *J* = 8.2 Hz, 2H), 7.29 (d, *J* = 8.1 Hz, 2H), 6.84 (d, *J* = 2.8 Hz, 2H), 6.67 (d, *J* = 8.9 Hz, 2H), 6.40 (dd, *J* = 8.9, 2.8 Hz, 2H), 5.47 (s, 2H), 4.50 (s, 2H), 0.60 (s, 3H), 0.53 (s, 3H); Analytical HPLC: t_R = 10.4 min, >99% purity (10–95% MeCN/H_2O, linear gradient, with constant 0.1% v/v TFA additive; 20 min run; 1 mL/min flow; ESI; positive ion mode; detection at 650 nm); HRMS (ESI) calcd for $C_{44}H_{29}D_{16}N_8O_4Si$ $[M+H]^+$ 793.4332, found 793.4341.

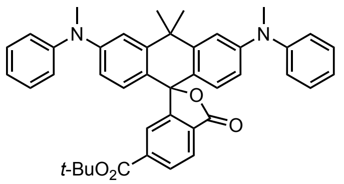

***tert*-Butyl 10,10-dimethyl-3,6-bis(methyl(phenyl)amino)-3'-oxo-3'*H*,10*H*-spiro[anthracene-9,1'-isobenzofuran] -6'-carboxylate (S59):** A vial was charged with 6-*tert*-butoxycarbonylcarbofluorescein ditriflate[12] (**S58**; 200 mg, 0.277 mmol), Pd_2dba_3 (25.3 mg, 27.7 µmol, 0.1 eq), XPhos (39.6 mg, 83.0 µmol, 0.3 eq), and Cs_2CO_3 (252 mg, 0.775 mmol, 2.8 eq). The vial was sealed and evacuated/backfilled with nitrogen (3×). Dioxane (2 mL) was added, and the reaction was flushed again with nitrogen (3×). Following the addition of *N*-methylaniline (66.0 µL, 0.609 mmol, 2.2 eq), the reaction was stirred at 100 °C for 4 h. It was then cooled to room temperature, filtered through Celite with CH_2Cl_2, and concentrated to dryness. Purification by silica gel chromatography (0–30% EtOAc/hexanes, linear gradient) afforded **S59** (150 mg, 85%) as an off-white solid. ^{1}H NMR ($CDCl_3$, 400 MHz) δ 8.15 (dd, *J* = 8.0, 1.3 Hz, 1H), 8.04 – 7.99 (m, 1H), 7.71 – 7.68 (m, 1H), 7.35 – 7.28 (m, 4H), 7.16 (d, *J* = 2.5 Hz, 2H), 7.14 – 7.09 (m, 4H), 7.08 – 7.01 (m, 2H), 6.70 (dd, *J* = 8.7, 2.4 Hz, 2H), 6.57 (d, *J* = 8.7 Hz, 2H), 3.35 (s, 6H), 1.73 (s, 3H), 1.67 (s, 3H), 1.55 (s, 9H); ^{13}C NMR ($CDCl_3$, 101 MHz) δ 170.0 (C), 164.6 (C), 155.3 (C), 149.6 (C), 148.4 (C), 146.6 (C), 137.9

(C), 130.2 (CH), 130.1 (C), 129.5 (CH), 128.9 (CH), 125.1 (CH), 125.0 (CH), 123.0 (CH), 122.7 (CH), 122.3 (C), 117.3 (CH), 115.5 (CH), 87.7 (C), 82.5 (C), 40.3 (CH_3), 38.5 (C), 35.0 (CH_3), 33.0 (CH_3), 28.2 (CH_3); Analytical HPLC: t_R = 12.7 min, >99% purity (50–95% $MeCN/H_2O$, linear gradient, with constant 0.1% v/v TFA additive; 20 min run; 1 mL/min flow; ESI; positive ion mode; detection at 254 nm); HRMS (ESI) calcd for $C_{42}H_{41}N_2O_4$ $[M+H]^+$ 637.3061, found 637.3061.

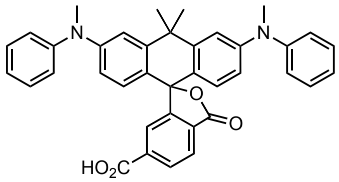

10,10-Dimethyl-3,6-bis(methyl(phenyl)amino)-3'-oxo-3'*H*,10*H*-spiro[anthracene-9,1'-isobenzofuran]-6'-carboxylic acid (S60): Ester **S59** (120 mg, 0.188 mmol) was taken up in CH_2Cl_2 (5 mL), and trifluoroacetic acid (1 mL) was added. The reaction was stirred at room temperature for 6 h. Toluene (5 mL) was added; the reaction mixture was concentrated to dryness and then azeotroped with MeOH three times to provide **S60** as a dark blue solid (128 mg, 98%, TFA salt). Analytical HPLC and NMR indicated that the material was >95% pure and did not require further purification prior to amide coupling. ^1H NMR (CD_3OD, 400 MHz) δ 8.34 – 8.28 (m, 2H), 7.87 – 7.82 (m, 1H), 7.56 – 7.48 (m, 4H), 7.43 – 7.36 (m, 2H), 7.34 – 7.29 (m, 4H), 7.15 (d, J = 2.5 Hz, 2H), 6.94 (d, J = 9.3 Hz, 2H), 6.71 (dd, J = 9.3, 2.4 Hz, 2H), 3.57 (s, 6H), 1.67 (s, 3H), 1.59 (s, 3H); ^{13}C NMR (CD_3OD, 101 MHz) δ 168.0 (C), 167.8 (C), 156.9 (C), 146.8 (C), 136.7 (CH), 136.0 (C), 135.2 (C), 131.7 (C), 131.6 (CH), 131.5 (CH), 131.1 (CH), 128.7 (CH), 127.2 (CH), 123.0 (C), 116.0 (CH), 113.9 (CH), 42.5 (C), 41.6 (CH_3), 35.3 (CH_3), 32.1 (CH_3); Analytical HPLC: t_R = 11.6 min, >99% purity (30–95% $MeCN/H_2O$, linear gradient, with constant 0.1% v/v TFA additive; 20 min run; 1 mL/min flow; ESI; positive ion mode; detection at 254 nm); HRMS (ESI) calcd for $C_{38}H_{33}N_2O_4$ $[M+H]^+$ 581.2435, found 581.2428.

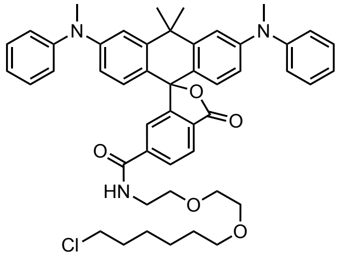

***N*-(2-(2-((6-Chlorohexyl)oxy)ethoxy)ethyl)-10,10-dimethyl-3,6-bis(methyl(phenyl)amino)-3'-oxo-3'*H*,10*H*-spiro[anthracene-9,1'-isobenzofuran]-6'-carboxamide (91_{HTL}):** Acid **S60** (50 mg, 72.0 μmol) was combined with DSC (44.2 mg, 0.173 mmol, 2.4 eq) in DMF (4 mL). After adding Et_3N (60.2 μL, 0.432 mmol, 6 eq) and DMAP (0.9 mg, 7.2 μmol, 0.1 eq), the reaction was stirred at room temperature for 30 min. A solution of HaloTag(O2)amine (HTL–NH_2, **111**; TFA salt; 72.9 mg, 0.216 mmol, 3 eq) in DMF (500 μL) was added; the reaction was then stirred for an additional 18 h at room temperature. It was subsequently diluted with saturated $NaHCO_3$ and extracted with EtOAc (2×). The combined organic extracts were washed with water and brine, dried over anhydrous $MgSO_4$, filtered, and

evaporated. Purification of the crude product by silica gel chromatography (5–75% EtOAc/toluene, linear gradient) provided **91**$_{\mathbf{HTL}}$ as a pale blue solid (44 mg, 78%). ^{1}H NMR ($CDCl_3$, 400 MHz) δ 8.03 (d, *J* = 7.9 Hz, 1H), 7.92 (dd, *J* = 8.0, 1.4 Hz, 1H), 7.52 (s, 1H), 7.35 – 7.28 (m, 4H), 7.16 (d, *J* = 2.4 Hz, 2H), 7.14 – 7.09 (m, 4H), 7.07 – 7.01 (m, 2H), 6.82 (t, *J* = 5.1 Hz, 1H), 6.70 (dd, *J* = 8.7, 2.4 Hz, 2H), 6.57 (d, *J* = 8.7 Hz, 2H), 3.67 – 3.58 (m, 6H), 3.57 – 3.53 (m, 2H), 3.50 (t, *J* = 6.6 Hz, 2H), 3.41 (t, *J* = 6.7 Hz, 2H), 3.35 (s, 6H), 1.78 – 1.68 (m, 2H), 1.73 (s, 3H), 1.66 (s, 3H), 1.57 – 1.50 (m, 2H), 1.46 – 1.37 (m, 2H), 1.36 – 1.28 (m, 2H); Analytical HPLC: t_R = 14.9 min, >99% purity (30–95% MeCN/H_2O, linear gradient, with constant 0.1% v/v TFA additive; 20 min run; 1 mL/min flow; ESI; positive ion mode; detection at 254 nm); HRMS (ESI) calcd for $C_{48}H_{53}ClN_3O_5$ [M+H]$^+$ 786.3668, found 786.3658.

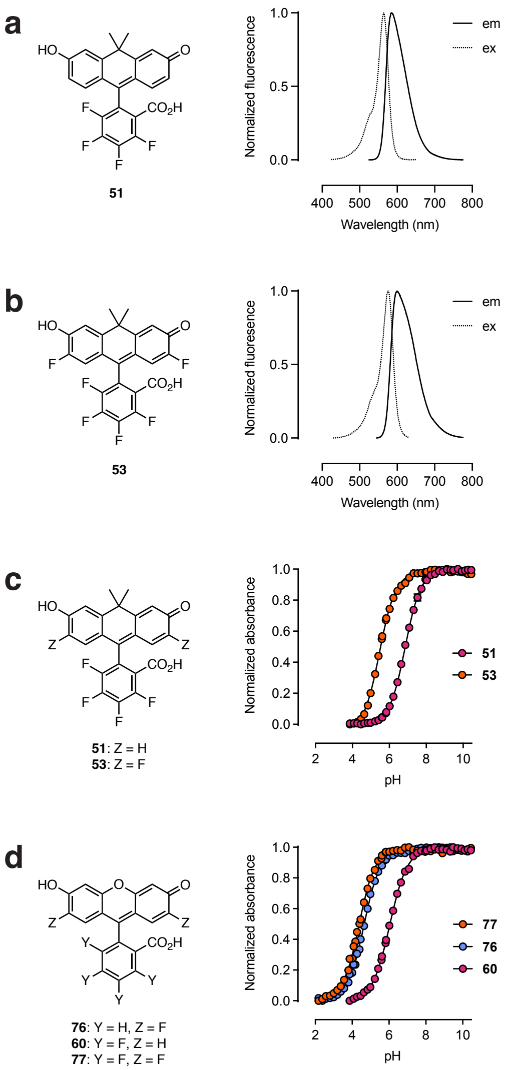

Figure S1. Spectral properties of fluorinated fluorescein dyes. (**a**) Normalized fluorescence excitation and emission spectra of carbofluorescein **51**. (**b**) Normalized fluorescence excitation and emission spectra of carbofluorescein **53**. (**c**) pH titrations (*i.e.*, absorbance *vs.* pH) for fluorinated carbofluoresceins **51** and **53**. (**d**) pH titrations (*i.e.*, absorbance *vs.* pH) for fluorinated fluoresceins **60**, **76**, and **77**.

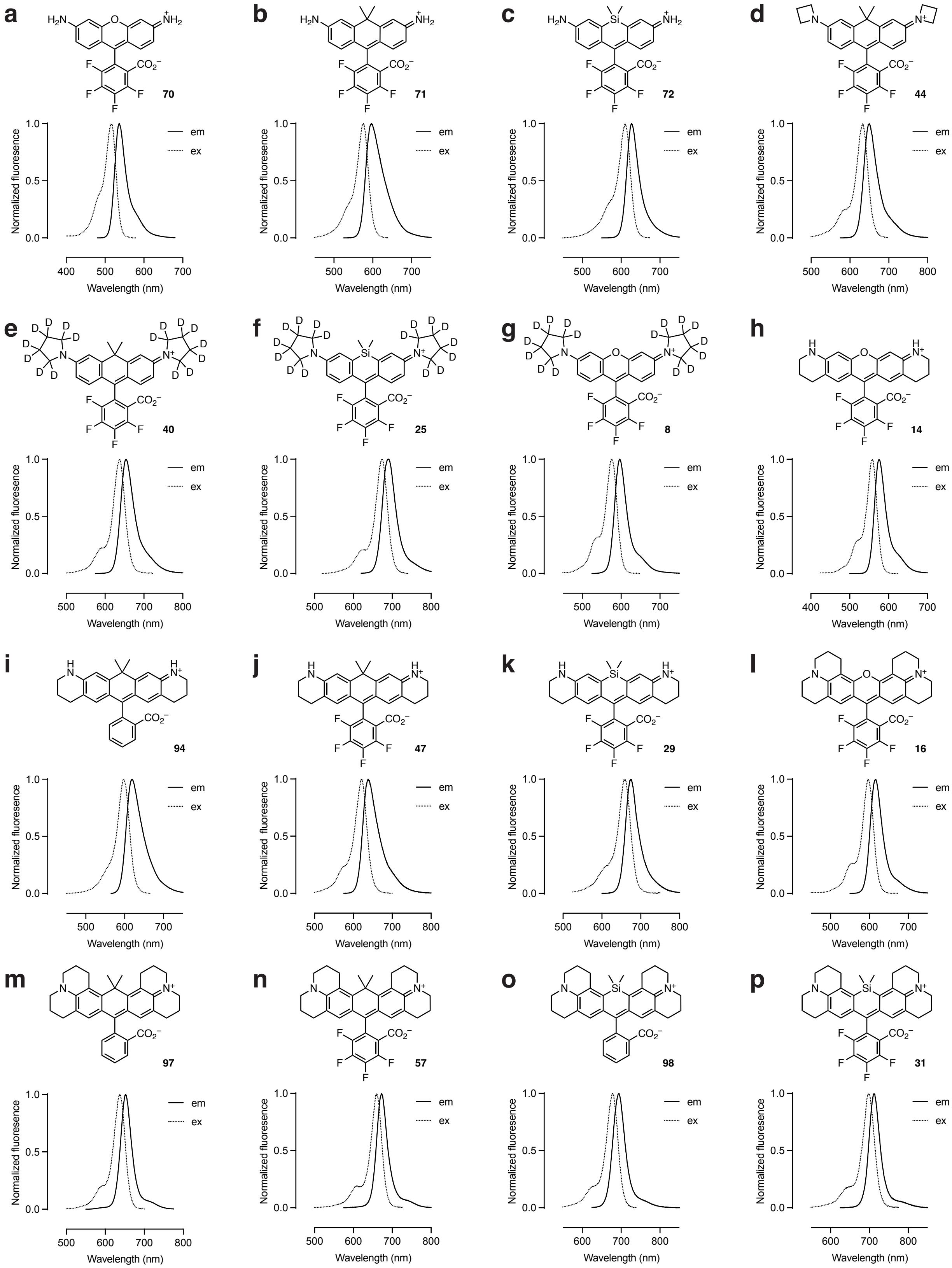

Figure S2. Normalized fluorescence excitation and emission spectra of rhodamines. (a) **70**. (b) **71**. (c) **72**. (d) **44**. (e) **40**. (f) **25**. (g) **8**. (h) **14**. (i) **94**. (j) **47**. (k) **29**. (l) **16**. (m) **97**. (n) **57**. (o) **98**. (p) **31**.

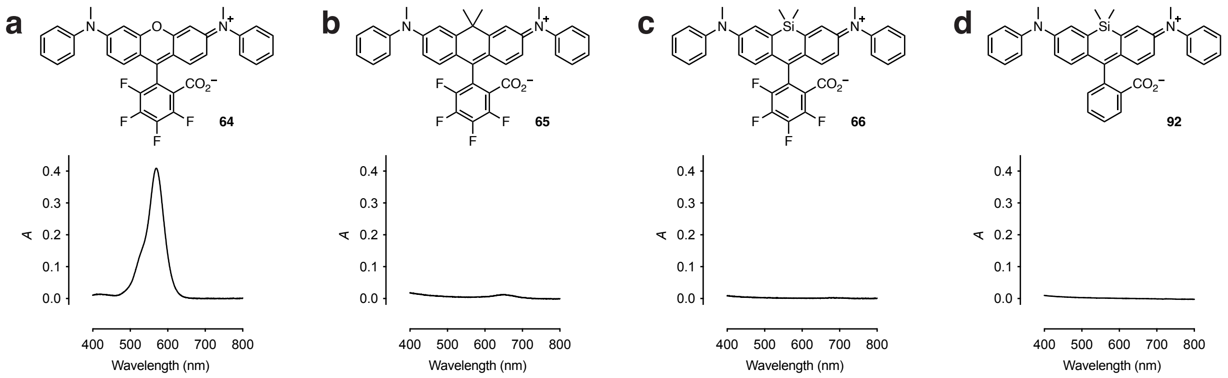

Figure S3. Absolute absorption spectra of *N*-arylrhodamines. (**a**) **64**. (**b**) **65**. (**c**) **66**. (**d**) **92**.

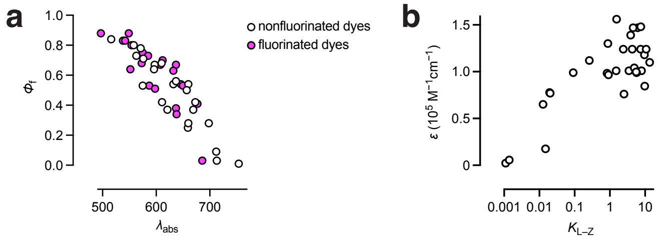

Figure S4. Trends in spectral and chemical properties of rhodamine dyes. (**a**) Plot of Φ_f *vs.* λ_{abs} for nonfluorinated rhodamines (white points) and fluorinated rhodamines (magenta points). (**b**) Plot of ε *vs.* K_{L-Z} for rhodamine dyes.

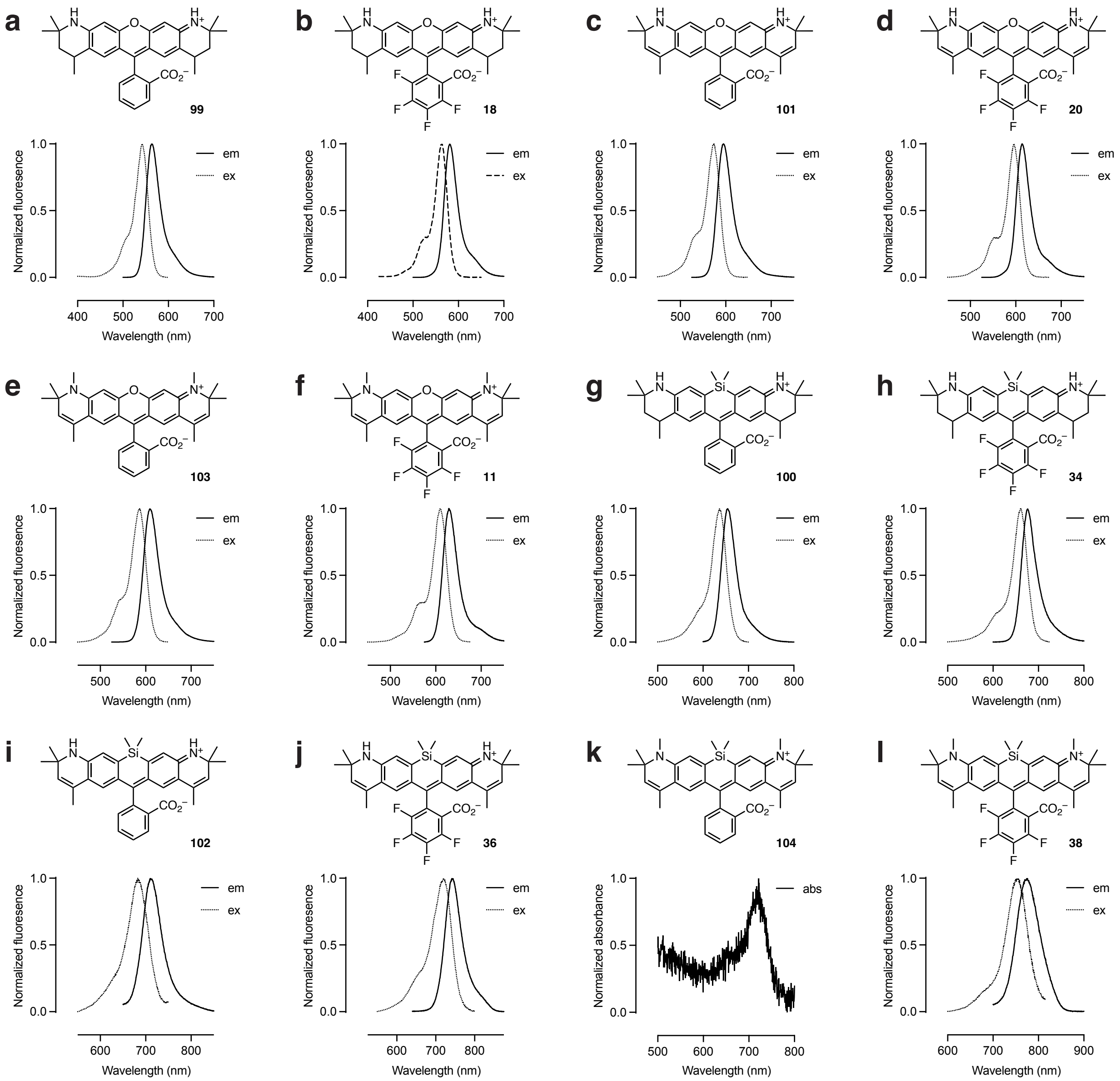

Figure S5. Spectral properties of rhodamines. (**a**–**j**,**l**) Normalized fluorescence excitation and emission spectra of rhodamines: (**a**) **99**. (**b**) **18**. (**c**) **101**. (**d**) **20**. (**e**) **103**. (**f**) **11**. (**g**) **100**. (**h**) **34**. (**i**) **102**. (**j**) **36**. (**l**) **38**. (**k**) Normalized absorption spectrum of **104**.

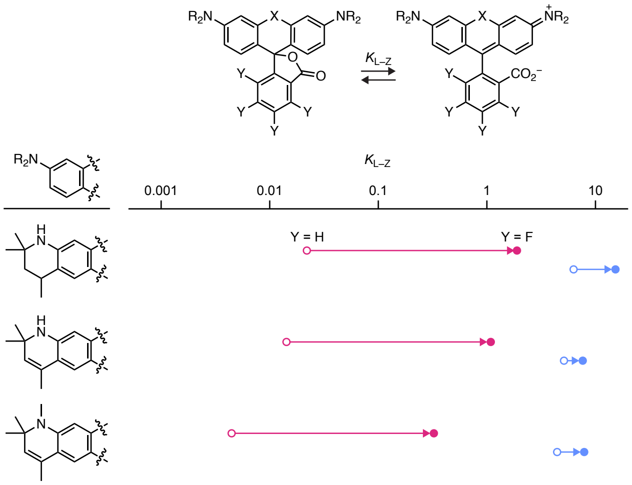

Figure S6. Plot of K_{L-Z} *vs.* auxochrome structure for rhodamines 11, 18, 20, 34, 36, 38, and 99–104. Arrow colors indicate Si-rhodamines (X = $Si(CH_3)_2$; magenta) or rhodamines (X = O; blue); white points indicate Y = H and colored points indicate Y = F.

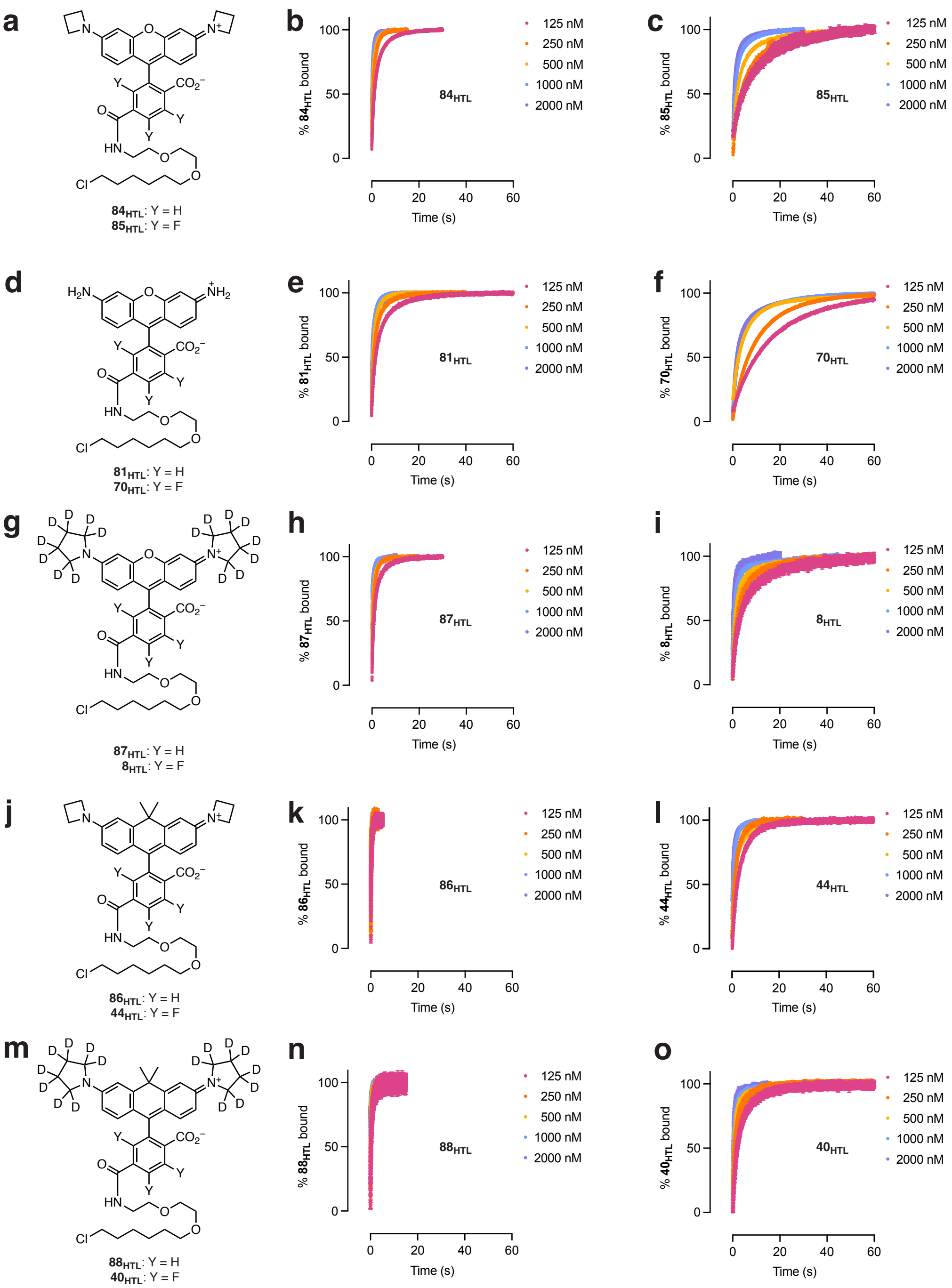

Figure S7. Labeling rates of nonfluorinated and fluorinated HaloTag ligands. (**a**) Chemical structures of **84_{HTL}** and **85_{HTL}**. (**b**) % of **84_{HTL}** bound *vs.* time using equimolar ligand and HaloTag protein. (**c**) % of **85_{HTL}** bound *vs.* time using equimolar ligand and HaloTag protein. (**d**) Chemical structures of **81_{HTL}** and **70_{HTL}**. (**e**) % of **81_{HTL}** bound *vs.* time using equimolar ligand and HaloTag protein. (**f**) % of **70_{HTL}** bound *vs.* time using equimolar ligand and HaloTag protein. (**g**) Chemical structures of **87_{HTL}** and **8_{HTL}**. (**h**) % of **87_{HTL}** bound *vs.* time using equimolar ligand and HaloTag protein. (**i**) % of **8_{HTL}** bound *vs.* time using equimolar ligand and HaloTag protein. (**j**) Chemical structures of **86_{HTL}** and **44_{HTL}**. (**k**) % of **86_{HTL}** bound *vs.* time using equimolar ligand and HaloTag protein. (**l**) % of **44_{HTL}** bound *vs.* time using equimolar ligand and HaloTag protein. (**m**) Chemical structures of **88_{HTL}** and **40_{HTL}**. (**n**) % of **88_{HTL}** bound *vs.* time using equimolar ligand and HaloTag protein. (**o**) % of **40_{HTL}** bound *vs.* time using equimolar ligand and HaloTag protein. % bound for all ligands was measured in a stopped-flow instrument using fluorescence polarization.

compound	base dye	labeling rate	
$\mathbf{84_{HTL}}$	JF_{549}	$6.49 \pm 0.94 \times 10^6\ M^{-1}s^{-1}$	4.0× slower
$\mathbf{85_{HTL}}$	JF_{571}	$1.61 \pm 0.75 \times 10^6\ M^{-1}s^{-1}$	
$\mathbf{81_{HTL}}$	Rh_{110}	$3.03 \pm 0.70 \times 10^6\ M^{-1}s^{-1}$	6.1× slower
$\mathbf{70_{HTL}}$	FRh_{110}	$5.0 \pm 1.6 \times 10^5\ M^{-1}s^{-1}$	
$\mathbf{87_{HTL}}$	JFX_{554}	$9.1 \pm 2.3 \times 10^6\ M^{-1}s^{-1}$	4.2× slower
$\mathbf{8_{HTL}}$	JFX_{576}	$2.17 \pm 0.2 \times 10^6\ M^{-1}s^{-1}$	
$\mathbf{86_{HTL}}$	JF_{608}	$4.0 \pm 2.0 \times 10^7\ M^{-1}s^{-1}$	13.6× slower
$\mathbf{44_{HTL}}$	JF_{632}	$2.95 \pm 1.28 \times 10^6\ M^{-1}s^{-1}$	
$\mathbf{88_{HTL}}$	JFX_{612}	$2.70 \pm 0.50 \times 10^7\ M^{-1}s^{-1}$	6.6× slower
$\mathbf{40_{HTL}}$	JFX_{637}	$4.1 \pm 0.4 \times 10^6\ M^{-1}s^{-1}$	

Table S3. Labeling rates of HaloTag ligands: 84_{HTL}, 85_{HTL}, 81_{HTL}, 70_{HTL}, 87_{HTL}, 8_{HTL}, 86_{HTL}, 44_{HTL}, 88_{HTL}, and 40_{HTL}.

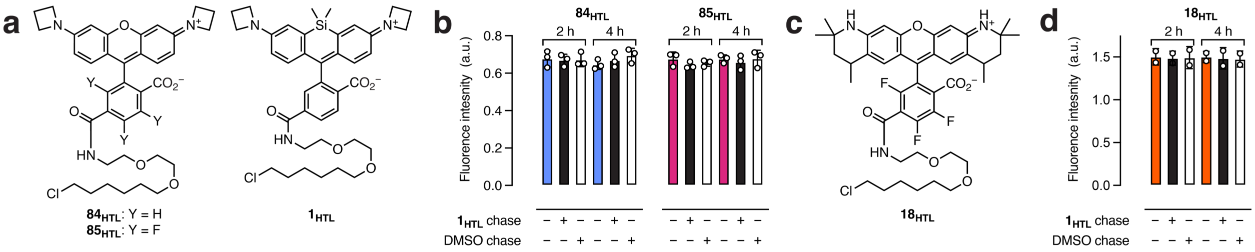

Figure S8. Stability of HaloTag conjugates of 84_{HTL}, 85_{HTL}, and 18_{HTL}. (**a**) Chemical structures of $\mathbf{84_{HTL}}$, $\mathbf{85_{HTL}}$, and chase ligand JF_{646}-HaloTag ligand ($\mathbf{1_{HTL}}$). (**b**) In-gel fluorescence of HaloTag protein labeled with nonfluorinated dye $\mathbf{84_{HTL}}$ or fluorinated dye $\mathbf{85_{HTL}}$ followed by incubation with $\mathbf{1_{HTL}}$ or DMSO; $n = 3$; error bars represent SD. (**c**) Chemical structure of $\mathbf{18_{HTL}}$. (**d**) In-gel fluorescence of HaloTag protein labeled with fluorinated dye $\mathbf{18_{HTL}}$ followed by incubation with $\mathbf{1_{HTL}}$ or DMSO; $n = 2$; error bars represent SD.

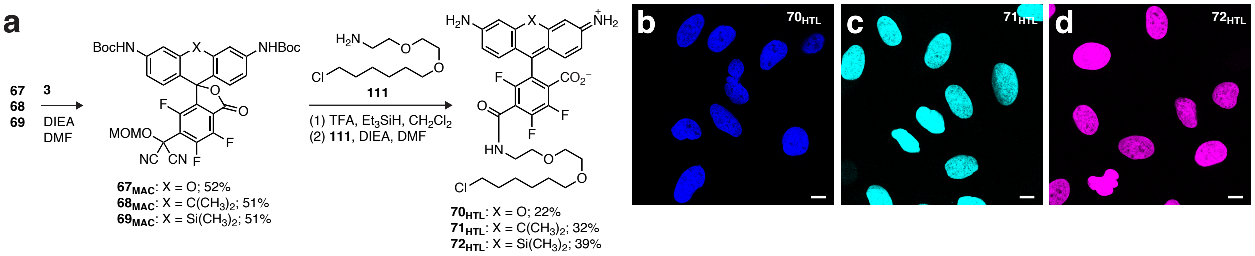

Figure S9. Synthesis of fluorinated rhodamine 110 HaloTag ligands and cellular imaging. (**a**) Synthesis of HaloTag ligands $\mathbf{70_{HTL}}$–$\mathbf{72_{HTL}}$ from **67**–**69**. (**b–d**) Fluorescence images of live U2OS cells expressing histone H2B–HaloTag fusion proteins and labeled with ligands $\mathbf{70_{HTL}}$ (**b**), $\mathbf{71_{HTL}}$ (**c**), and $\mathbf{72_{HTL}}$ (**d**); scale bars for all images: 10 μm.

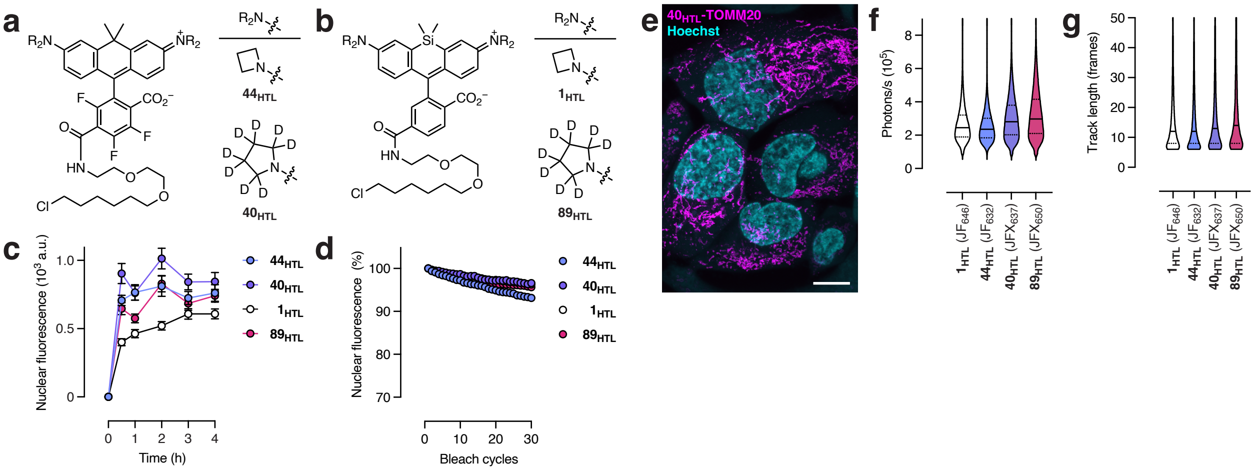

Figure S10. Performance of fluorinated carborhodamines. (**a**) Chemical structures of JF_{632}-HaloTag ligand (**44_{HTL}**) and JFX_{637}-HaloTag ligand (**40_{HTL}**). (**b**) Chemical structures of JF_{646}-HaloTag ligand (**1_{HTL}**) and JFX_{650}-HaloTag ligand (**89_{HTL}**). (**c**) Nuclear fluorescence *vs.* time upon addition of ligands **40_{HTL}**, **44_{HTL}**, **1_{HTL}**, and **89_{HTL}** (200 nM) to live cells expressing HaloTag–histone H2B; error bars indicate SEM; n = 100 nuclei from three fields of view. (**d**) Plot of fluorescence from fixed cells expressing HaloTag–histone H2B labeled with **40_{HTL}**, **44_{HTL}**, **1_{HTL}**, and **89_{HTL}** over 30 bleach cycles; error bars indicate SEM; n = 3 independent cellular samples. (**e**) Airyscan image of U2OS cells expressing TOMM20–HaloTag fusion proteins labeled with ligand **40_{HTL}** and counterstained with Hoechst 33342; scale bar: 10 µm. (**f**) Violin plot of intensity (photons/s) of ligands **40_{HTL}**, **44_{HTL}**, **1_{HTL}**, and **89_{HTL}** in SPT experiments. (**g**) Violin plot of track length (frames) of ligands **40_{HTL}**, **44_{HTL}**, **1_{HTL}**, and **89_{HTL}** in SPT experiments; only tracks >5 frames are plotted; imaging was performed at 50 Hz (20 ms/frame).

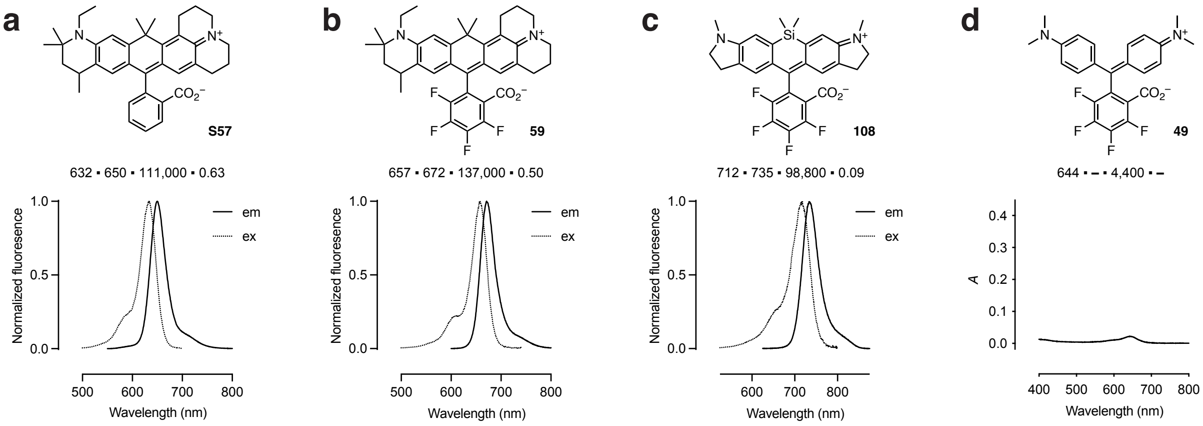

Figure S11. Spectral properties of rhodamines and fluorinated Malachite Green. (**a**–**c**) Normalized fluorescence excitation and emission spectra of rhodamines. (**a**) **S57**. (**b**) **59**. (**c**) **108**. (**d**) Absolute absorption spectrum of **49**. Numbers below each structure indicate: λ_{abs} (nm) ▪ λ_{em} (nm) ▪ ε ($M^{-1}cm^{-1}$) ▪ Φ_f.

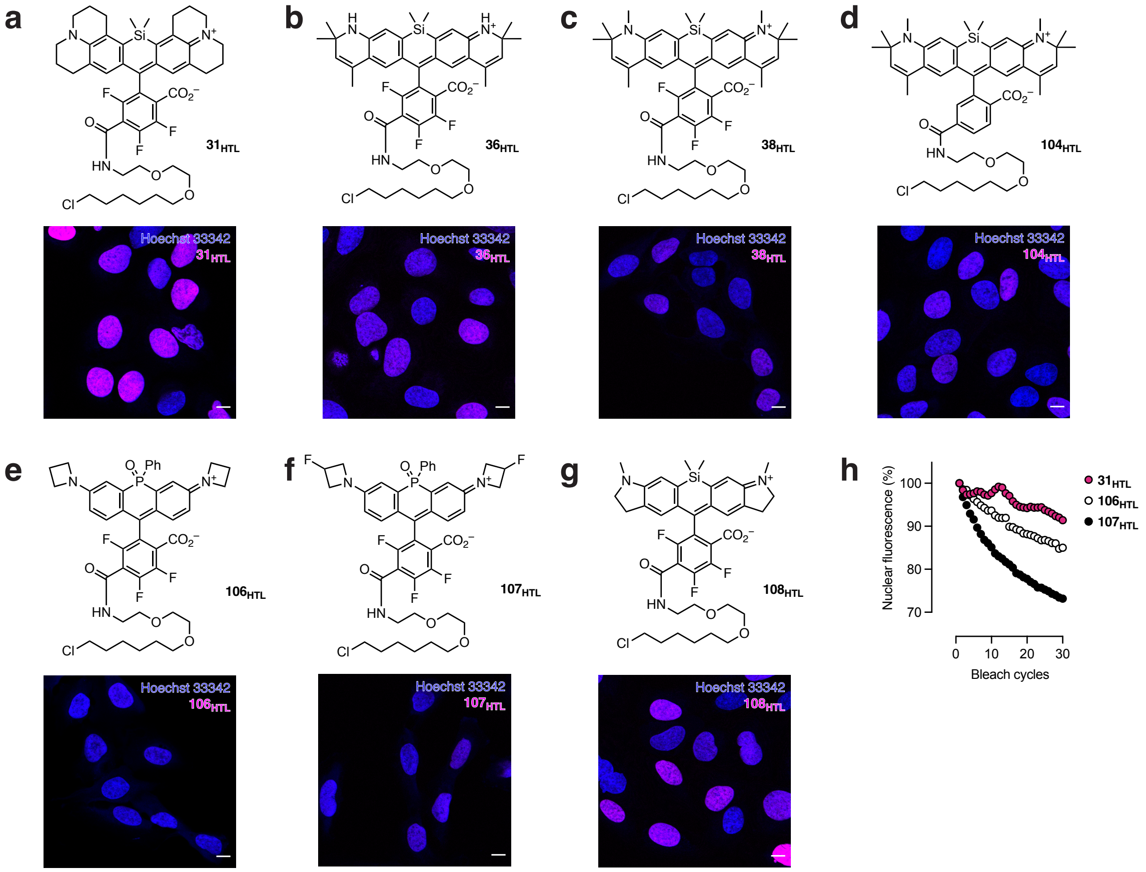

Figure S12. Performance of NIR-excited HaloTag ligands. (**a–h**) Chemical structures and fluorescence images of live U2OS cells expressing histone H2B–HaloTag fusion proteins and labeled with ligands **31**$_{\mathbf{HTL}}$ (**a**), **36**$_{\mathbf{HTL}}$ (**b**), **38**$_{\mathbf{HTL}}$ (**c**), **104**$_{\mathbf{HTL}}$ (**d**), **106**$_{\mathbf{HTL}}$ (**e**), **107**$_{\mathbf{HTL}}$ (**f**), and **108**$_{\mathbf{HTL}}$ (**g**) and counterstained with Hoescht 33342; scale bars for all images: 10 µm. (**h**) Plot of fluorescence from fixed cells expressing HaloTag–histone H2B labeled with **31**$_{\mathbf{HTL}}$, **106**$_{\mathbf{HTL}}$, and **107**$_{\mathbf{HTL}}$ over 30 bleach cycles; error bars indicate SEM; n = 3 fields of view.

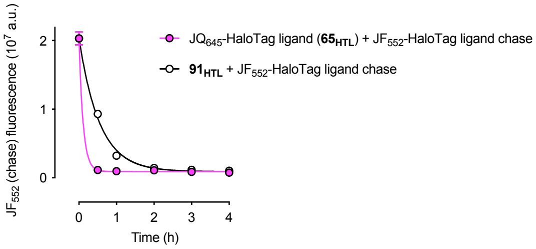

Figure S13. Cellular labeling of *N*-arylrhodamine-based HaloTag ligands 65$_{\mathbf{HTL}}$ **and 91**$_{\mathbf{HTL}}$**.** Plot of nuclear fluorescence from JF_{552}-HaloTag ligand (200 nM; chase) *vs.* incubation time with 200 nM of JQ_{645}-HaloTag ligand (**65**$_{\mathbf{HTL}}$) or **91**$_{\mathbf{HTL}}$; error bars indicate SEM; n = 100 nuclei from 3 fields of view.

OPTICAL SPECTROSCOPY AND MICROSCOPY METHODS

Compound sources. Compound **81**$_{\mathbf{HTL}}$ was purchased from Promega. Compounds **1**$_{\mathbf{HTL}}$, **84**$_{\mathbf{HTL}}$, **84**$_{\mathbf{HST}}$, **85**$_{\mathbf{HTL}}$, **86**$_{\mathbf{HTL}}$, **89**$_{\mathbf{HTL}}$, **105**$_{\mathbf{HTL}}$, **106**$_{\mathbf{HTL}}$, and **107**$_{\mathbf{HTL}}$ were available from previous work. Data for dyes **1**, **2**, **22**, **74**, **75**, **78–91**, **93**, and **95** were taken from published work from our laboratory; all spectral data was measured under identical conditions.

General UV–vis and fluorescence spectroscopy (Tables 1–3, Figure S1a,b, Figure S2, Figure S3, Figure S5, Figure S11). All dyes for spectroscopy were prepared as stock solutions in DMSO and diluted such that the final DMSO concentration did not exceed 1% v/v. Spectroscopy was performed using 1-cm path length, 3.5-mL quartz cuvettes or 1-cm path length, 1.4-mL semi-micro quartz cuvettes from Starna Cells. All measurements were taken at ambient temperature (22 ± 2 °C). Absorption spectra were recorded on a Cary Model 100 spectrometer (Agilent), and fluorescence spectra were recorded on a Cary Eclipse fluorometer (Varian). The spectra, maximum absorption wavelength (λ_{abs}), extinction coefficient at λ_{abs} (ε), and maximum emission wavelength (λ_{em}) were measured in 10 mM HEPES, pH 7.3 buffer (rhodamines and Malachite Green derivative **49**) or 0.1 M NaOH (fluoresceins); the reported values for ε are averages (n = 3). Normalized spectra are shown for clarity.

Quantum yield determination (Tables 1–3, Figure S11). All reported absolute fluorescence quantum yield values (Φ_f) were measured in our laboratory using a Quantaurus-QY spectrometer (model C11374, Hamamatsu). This instrument uses an integrating sphere to determine photons absorbed and emitted by a sample. Measurements were carried out using dilute samples (A < 0.1), and self-absorption corrections were performed using the instrument software.[13]

HaloTag protein purification. The bacterial expression vector pRSET-A (Invitrogen) was used to recombinantly express HaloTag (HT7; Promega).[14] The soluble 6×His-Tagged HaloTag protein was affinity purified by immobilized metal affinity chromatography (IMAC) on a 5-mL Fast Flow HiTrap Sepharose 6 column (Cytiva) with a 0–200 mM imidazole elution gradient using an ÄKTA Avant Protein Purification System (Cytiva). A_{280} peak fractions were pooled, concentrated by a spin concentrator, and dialyzed 3× into tris-buffered saline (TBS; 137 mM NaCl, 20 mM Tris·HCl, pH 7.4). The amino acid sequence of HaloTag (HT7) expressed from pRSET-A is:

```
MRGSHHHHHH GMASMTGGQQ MGRDLYDDDD KDRWGSMAEI GTGFPFDPHY VEVLGERMHY VDVGPRDGTP
VLFLHGNPTS SYVWRNIIPH VAPTHRCIAP DLIGMGKSDK PDLGYFFDDH VRFMDAFIEA LGLEEVVLVI
HDWGSALGFH WAKRNPERVK GIAFMEFIRP IPTWDEWPEF ARETFQAFRT TDVGRKLIID QNVFIEGTLP
MGVVRPLTEV EMDHYREPFL NPVDREPLWR FPNELPIAGE PANIVALVEE YMDWLHQSPV PKLLFWGTPG
VLIPPAEAAR LAKSLPNCKA VDIGPGLNLL QEDNPDLIGS EIARWLSTLE ISG
```

Absorption increase of ligands on binding HaloTag protein (Figure 5b). HaloTag protein was used as a 100 μM solution in 1× TBS. Absorption measurements were performed in 1.4-mL semi-micro quartz cuvettes. A 5 μM solution

of HaloTag ligand **65**$_{HTL}$ or **91**$_{HTL}$ was prepared in 10 mM HEPES, pH 7.3 containing 0.1 mg·mL^{-1} CHAPS. An aliquot of HaloTag protein (2 equiv, 10 µM final [HaloTag]) was added. To examine chromogenicty (**Figure 5b, left panel)**, the resulting mixture was incubated until a consistent absorption signal/spectrum was observed (<60 min for **65**$_{HTL}$); an equivalent volume of 1× TBS blank was added to separate "−HT" samples in place of enzyme to record the ligand-only absorption. Spectra are averages (n = 2). To compare rates of HaloTag lableing (**Figure 5c, right panel**) absorption at λ_{abs} (645 nm for **65**$_{HTL}$, 621 nm for **91**$_{HTL}$) was recorded at regular intervals (0.1 min, 0–5 min; 1 min, 5–120 min; 5 min, 2–24 h) immediately following addition of HaloTag protein.

Determination of $K_{L–Z}$ (Tables 2–3). We calculated $K_{L–Z}$ using the following equation:[12, 15] $K_{L–Z} = (\varepsilon_{dw}/\varepsilon_{max})/(1 - \varepsilon_{dw}/\varepsilon_{max})$. ε_{dw} is the extinction coefficient of the dyes in a 1:1 (v/v) dioxane/water solvent mixture; this dioxane/water mixture was chosen to give the maximum spread of $K_{L–Z}$ values across all classes of rhodamines.[12] ε_{max} refers to the maximal extinction coefficients measured in different solvent mixtures empirically determined depending on dye type: 0.1% v/v trifluoroacetic acid in ethanol for **18**, **20**, **29**, **34**, **36**, **70**–**72**, **81**–**83**, **93**–**95**, **99**–**102**, and **104**; 0.1% v/v trifluoroacetic acid in 2,2,2-trifluoroethanol for all other rhodamine variants. We note that accurate determination of low $K_{L–Z}$ values is complicated by the relatively poor sensitivity of absorbance measurements. We did not attempt to estimate $K_{L–Z}$ values when we observed no measurable absorbance of the dye in the dioxane/water solution.

pK_a determination (Table 1, Figure S1c,d). The pK_a values for compounds **51**, **53**, **60**, **76**, and **77** were determined by measuring the change in absorbance as a function of pH. For pH 2.0–4.0, the McIlvaine citrate/phosphate buffer system was used. For other pH ranges dye absorbance was measured in buffers containing 150 mM NaCl and 10 mM buffer using the following systems: citrate (pH 4.0– 6.0), phosphate (pH 5.8– 8.0); tris (pH 7.8–9.0); carbonate (pH 9.2–10.0). Buffer solutions containing 5 µM of each fluorophore were prepared (36 samples in duplicate from pH 4.0–10.0 for **51**, **53**, and **60**; 48 samples in duplicate from pH 2.0–10.0 for **76** and **77**). Absorbance values were recorded on a Cary Model 100 spectrometer and plotted using GraphPad Prism software. The points were fitted to a sigmoidal dose response curve with a variable slope to determine the Hill coefficient (η_H).

General cell culture methods. U2OS cells (ATCC) and U2OS cells stably expressing an integrated HaloTag–histone H2B fusion protein (U2OS.H2B.HaloTag) were cultured in Dulbecco's modified Eagle medium (DMEM, phenol red-free; Life Technologies) supplemented with 10% (v/v) fetal bovine serum (FBS, Life Technologies), 1 mM GlutaMAX (Life Technologies) and maintained at 37 °C in a humidified 5% (v/v) CO_2 environment. These cell lines undergo regular mycoplasma testing by the Janelia Cell Culture Facility. For mitochondrial imaging using the HaloTag system, U2OS cells were transiently transfected using nucleofection (Lonza) with a plasmid constitutively expressing a HaloTag–TOMM20 fusion protein, which is located on the outer mitochondrial membrane. For 4-color imaging, U2OS.H2B.HaloTag stable cells were transiently transfected by nucleofection with plasmids constitutively expressing a SNAP-tag–TOMM20 fusion protein, and a GFP–SKL (SKL: serine–lysine–leucine) fusion protein, a prototypical peroxisomal matrix targeting signal. All labeled cells were imaged 18–24 h post-transfection or post-plating.

Labeling and confocal microscopy using ligands 70_{HTL}–72_{HTL} (Figure S9b–d). The U2OS.H2B.HaloTag stable cells were incubated with 100 nM HaloTag ligand conjugated dyes (**70_{HTL}**–**72_{HTL}**) for 2 h at 37 °C, washed 3× with dye-free media then imaged live. Confocal imaging was performed on a Leica SP8 using an HC PL APO 86×/1.20 water objective. Single plane images were processed using FIJI.[16]

Loading and photostability experiments using ligands 40_{HTL}, 44_{HTL}, 1_{HTL}, and 89_{HTL} (Figure S10c,d). For loading curve experiments (**Figure S10c**), U2OS.H2B.HaloTag stable cells were incubated with 200 nM HaloTag ligand conjugated dyes (**40_{HTL}**, **44_{HTL}**, **1_{HTL}**, or **89_{HTL}**) for 0–4 h at 37 °C, washed 3× with dye-free media then fixed with 4% paraformaldehyde in 0.1 M phosphate buffer, pH 7.4 for 15 min at 37 °C. Confocal imaging was performed on a Leica Stellaris with an HC PL APO 40×/1.25 glycerol objective using the tunable white light laser (WLL) to excite dyes at their λ_{abs} in constant power mode. Fluorescence was quantified as the integrated density of nuclear signals in single-plane images analyzed in FIJI;[16] n = 100 nuclear signals per compound.

For photostability experiments (**Figure S10d**), U2OS.H2B.HaloTag stable cells were incubated with 200 nM HaloTag ligands (**40_{HTL}**, **44_{HTL}**, **1_{HTL}**, or **89_{HTL}**) for 2 h at 37 °C, washed 3× with dye-free media then fixed with 4% paraformaldehyde in 0.1 M phosphate buffer, pH 7.4 for 15 min at 37 °C. Confocal imaging was performed on a Leica SP8 with an HC PL APO 86×/1.20 water objective using adaptive focus control (AFC). To measure photobleaching, we used the tunable white light laser (WLL) to excite dyes at their λ_{abs} in constant power mode over 30 cycles; n = 3 where n indicates the number of separate cellular experiments where the intensity of the entire field of view was measured at the indicated time points. Single plane images were processed using FIJI.[16]

Labeling and multicolor confocal microscopy using 31_{HTL} (Figure 4d). To achieve a 4-color image, live U2OS.H2B.HaloTag stable cells that were transiently transfected with plasmids constitutively expressing a SNAP-tag–TOMM20 fusion protein and an GFP–SKL fusion protein were incubated with 200 nM JF_{698}-HaloTag ligand (**31_{HTL}**) and JFX_{650}-SNAP-tag ligand (**89_{HTL}**) for 1 h at 37 °C, washed 3× with dye-free media, and then fixed with 4% paraformaldehyde in 0.1 M phosphate buffer, pH 7.4 for 15 min at 37 °C. Fixed cells were then washed 3× in 1× phosphate buffered saline, pH 7.4 (PBS) and incubated with JF_{549}-Hoechst (**84_{HST}**; 3µM) for 15 min at ambient temperature (22±2 °C) as a nuclear counterstain. Confocal imaging was performed on a Leica Stellaris with an HC PL APO CS2 86×/1.20 water objective using the tunable white light laser (WLL) to excite each dye or fluorescent protein at their λ_{abs}. Confocal image stacks were processed using FIJI and displayed as maximum intensity image projections.

Loading experiments using ligands 65_{HTL} and 91_{HTL} (Figure S13). For loading curve experiments, U2OS.H2B.HaloTag stable cells were incubated with 200 nM **65_{HTL} and 91_{HTL}** for 0–4 h at 37 °C, washed 3× with dye-free media then chased with 200 nM JF_{552}-HaloTag ligand[17] for 30 min at 37 °C, washed 3× with dye-free media and fixed with 4% paraformaldehyde in 0.1 M phosphate buffer, pH 7.4 for 15 min at 37 °C. Confocal imaging was performed on a Zeiss LSM 980 using a Plan-Apochromat 20×/0.8 M27 objective, and excited by a 561nm laser.

Fluorescence was quantified as the integrated density of nuclear signals from confocal image stack projections analyzed in FIJI;[16] n = 100 nuclear signals per compound.

Labeling and Airyscan microscopy using ligand $\mathbf{40_{HTL}}$ (Figure S10e). U2OS cells transiently transfected with a plasmid constitutively expressing a HaloTag–TOMM20 fusion protein were incubated with 200 nM JFX_{637}-HaloTag ligand (**$\mathbf{40_{HTL}}$**) for 1 h at 37°C, washed 3× with dye-free media then fixed with 4% paraformaldehyde in 0.1 M phosphate buffer, pH 7.4 for 15 min at 37 °C. Fixed cells were then washed 3× in 1× PBS and incubated with Hoechst 33342 (5 µg/mL; nuclear co-stain) for 15 min at ambient temperature (22±2 °C) as a nuclear counterstain. High-resolution Airyscan imaging was performed on a Zeiss LSM 980 with Airyscan 2 confocal microscope using a Plan APO 63×/1.4 oil DIC M27 objective. Confocal image stacks were bulk processed in ZEN Blue (Zeiss) with automatic Airyscan settings; the final images were processed in FIJI.[16]

Labeling and confocal microscopy using ligands $\mathbf{59_{HTL}}$ and $\mathbf{105_{HTL}}$ (Figure 3b,c). U2OS.H2B.HaloTag stable cells were incubated with 200 nM of either JF_{657}-HaloTag ligand (**$\mathbf{59_{HTL}}$**) or ATTO 647N-HaloTag ligand (**$\mathbf{105_{HTL}}$**) for 1 h at 37 °C, washed 3× with dye-free media, stained with Hoechst 33342 (5 µg/mL; nuclear co-stain) and MitoTracker Green FM (100 nM; mitochondrial stain) for 15 min at 37 °C then imaged live. Standard confocal imaging was performed on a Zeiss LSM 980 using a Plan APO 63×/1.4 oil DIC M27 objective. Confocal image stacks were processed using FIJI[16] and displayed as maximum intensity image projections.

Loading and photostability experiments using ligands $\mathbf{31_{HTL}}$, $\mathbf{36_{HTL}}$, $\mathbf{38_{HTL}}$, $\mathbf{104_{HTL}}$, $\mathbf{106_{HTL}}$, $\mathbf{107_{HTL}}$, and $\mathbf{108_{HTL}}$ (Figure 4c, Figure S12h). For loading curve experiments (**Figure 4c**), U2OS.H2B.HaloTag stable cells were incubated with 200 nM HaloTag ligand conjugated dyes (**$\mathbf{31_{HTL}}$**, **$\mathbf{36_{HTL}}$**, **$\mathbf{38_{HTL}}$**, **$\mathbf{104_{HTL}}$**, **$\mathbf{106_{HTL}}$**, **$\mathbf{107_{HTL}}$**, and **$\mathbf{108_{HTL}}$**) for 0–4 h at 37 °C, washed 3× with dye-free media then fixed with 4% paraformaldehyde in 0.1 M phosphate buffer, pH 7.4 for 15 min at 37 °C. Confocal imaging was performed on a Leica Stellaris with an HC PL APO 40×/1.25 glycerol objective using the tunable white light laser (WLL) to excite dyes at their λ_{abs} in constant power mode. Fluorescence was quantified as the integrated density of nuclear signals in single-plane images analyzed in FIJI;[16] n = 100 nuclear signals per compound.

For photostability experiments (**Figure S12h**), U2OS.H2B.HaloTag stable cells were incubated with 200 nM HaloTag ligands (**$\mathbf{31_{HTL}}$**, **$\mathbf{106_{HTL}}$**, and **$\mathbf{107_{HTL}}$**) for 3 h at 37°C, washed 3× with dye-free media then fixed with 4% paraformaldehyde in 0.1 M phosphate buffer, pH 7.4 for 15 min at 37 °C. Fixed cells were then washed 3× in 1× PBS and incubated with Hoechst 33342 (5 µg/mL; nuclear costain) for 15 min at ambient temperature (22±2 °C) as a nuclear counterstain. Confocal imaging was performed on a Leica Stellaris with an HC PL APO 86×/1.20 water objective using adaptive focus control (AFC). Based on Leica recommendations we used the Hoechst 33342 "blue" channel to engage the AFC for imaging using the far-red dyes with emission collection windows >750 nm. To measure photobleaching, we used the tunable white light laser (WLL) to excite dyes at their λ_{abs} in constant power mode over

30 cycles; $n = 3$ where n indicates the number of separate cellular experiments where the intensity of the entire field of view was measured at the indicated time points. Single plane images were processed using FIJI.[16]

Measurement of HaloTag ligand labeling rates (Figure S7, Table S3). To measure labeling rates we used a stopped-flow protocol where equimolar amounts of purified HaloTag protein and HaloTag ligand were mixed; the labeling was monitored by fluorescence polarization.[18] HaloTag protein and HaloTag ligands were serially diluted in 50 mM HEPES buffer, pH 7.2 containing 50 mM NaCl, and 0.5 mg/mL bovine serum albumin (BSA) to yield final concentrations of 2 µM, 1 µM, 500 nM, 250 nM, and 125 nM. Sample concentrations were evaluated using a Cary Model 100 spectrometer (Agilent) using ε_{280} (64,430 $M^{-1}\cdot cm^{-1}$) for the HaloTag protein or the ε values for the different fluorophores. Stopped-flow experiments were performed at 37 °C using an Applied Photophysics Stopped Flow Spectrometer, model SX20, in fluorescence polarization mode. Appropriate long-pass filters were used for each dye type. Five replicates of each concentration pair were collected ($n = 5$). The plots of fluorescence polarization *vs.* time were fit using DynaFit (Biokin) to calculate k_1, k_{-1}, and k_2; timepoints <3 ms were omitted from the analysis. The capture rate (k_{app}) was calculated using the following equation: $k_{app} = k_1(k_2 / k_{-1} \times k_2)$. Reported values are averages of the k_{app} values measured for the different concentrations.

Conjugate stability experiments (Figure S8). Purified HaloTag protein was incubated with a 10-fold molar excess of "pulse" HaloTag ligands in 1× PBS in LoBind microcentrifuge tubes (Eppendorf) and incubated for 2–4 h at 4 °C while protected from light. For displacement experiments, unbound pulse dye was removed by passing the reaction mixture through two successive Zeba Spin Desalting Column (ThermoFisher) using 1× PBS. These samples were then incubated with a 10-fold molar excess of JF_{646}-HaloTag ligand ($\mathbf{1_{HTL}}$) or a DMSO control for 1 h at 4 °C. Reactions were stopped by adding 4× NuPAGE LDS buffer (ThermoFisher) with NuPAGE Sample Reducing Agent (ThermoFisher) and heated for 10 min at 90 °C. Samples were run on NuPAGE 4–12% Bis-Tris 1.0–1.5 mm Mini Protein Gels (ThermoFisher) in NuPAGE MES SDS Running Buffer (ThermoFisher) for 60 min at 200 V. Gels were washed in Milli-Q water for 3 min then imaged on a black tray using a ChemiDoc MP Imaging system (Biorad) and 602/50 or 700/50 emission filter. Exposure times during imaging were set to avoid signal saturation. After fluorescence imaging, the gels were stained with Simply Blue Safe Stain (ThermoFisher) and reimaged using the white tray for calorimetric detection. ImageJ was used for signal quantification with background subtraction.

Single-particle tracking (SPT) experiments (Figure 3e,f, Figure S10f,g). SPT experiments were carried out as previously described.[19] JM8.N4 mouse embryonic stem cells (ESCs) stably expressing histone H2B–HaloTag fusion proteins were generated by co-expressing the PiggyBac EF1a-H2B-HaloTag-IRES-Neo vector with the super piggyBac transposase as described previously,[20] followed by G418 selection (500 µg/mL) for 2 weeks and verified by FACS sorting/confocal imaging staining with JF_{549}-HaloTag ligand ($\mathbf{84_{HTL}}$; 100 nM, 30 min). Cells were seeded on 25 mm #1.5 coverglass pre-cleaned with KOH and ethanol and coated with iMatrix-511 (TaKaRa #T304) or rhLaminin-521 (Gibco™ #A29248) according to the manufacturers' instruction. All live-cell imaging experiments were conducted using an ESC imaging medium composed of FluoroBrite DMEM (ThermoFisher) plus 15% v/v ESC-

qualified FBS, 1× GlutaMax, 1× NEAA, 0.1 mM 2-mercaptoethanol, and LIF. For comparison of $\mathbf{59}_{\mathbf{HTL}}$ and $\mathbf{89}_{\mathbf{HTL}}$ (**Figure 3e,f**) the histone H2B–HaloTag ESCs were stained with 5 nM $\mathbf{59}_{\mathbf{HTL}}$ for 1 h or 0.1 nM $\mathbf{89}_{\mathbf{HTL}}$ for 15 min at 37 °C with 5% (v/v) CO_2 to achieve sparse labeling with approximately equal localizations per region of interest (ROI), and then washed with dye-free media 3× for 30 min each wash For comparison of $\mathbf{40}_{\mathbf{HTL}}$, $\mathbf{44}_{\mathbf{HTL}}$, $\mathbf{1}_{\mathbf{HTL}}$, or $\mathbf{89}_{\mathbf{HTL}}$ (**Figure S10**) the histone H2B–HaloTag ESCs were stained with 1 nM of HaloTag ligands for 15 min at 37 °C with 5% (v/v) CO_2 to achieve sparse labeling, and then washed with dye-free media 3× for 30 min each wash.

To quantify the brightness and photostability of the HaloTag ligands, labeled cells were mounted onto a high speed motorized Nikon Eclipse Ti-E inverted microscope equipped with the following: 100× Apo TIRF 1.49 NA objective with a correction collar; four excitation laser lines (405/488/561/642 nm) and matching TIRF quad cube (405/488/561/640 nm reflection bands); automatic TIRF illuminator with motorized X axis and manual Y axis for beam positioning and focus: perfect focus 3 system; Triple DU-897 iXon Ultra EMCCD cameras on a Cairn Tri-cam emission splitter with filters 525/50 (GFP) 600/50 (RFP) and 705/72 (Cy5); humidified incubation chamber maintained at 37 °C with 5% v/v CO_2 (Tokai Hit). SPT was performed at 100 Hz for the comparison of $\mathbf{59}_{\mathbf{HTL}}$ and $\mathbf{89}_{\mathbf{HTL}}$ (**Figure 3e,f**) and 50 Hz for the comparison of $\mathbf{40}_{\mathbf{HTL}}$, $\mathbf{44}_{\mathbf{HTL}}$, $\mathbf{1}_{\mathbf{HTL}}$, or $\mathbf{89}_{\mathbf{HTL}}$ (**Figure S10**); the imaging session was performed for ~5000 frames using 100% of maximal excitation power (~120mW at the fiber end before the TIRF unit). The excitation laser was controlled by an acousto-optic tunable filter (AOTF) and reflected into the objective by a multi-band dichroic (405/488/561/647 BrightLine quad-band bandpass filter; Semrock). For the comparison of HaloTag ligands $\mathbf{59}_{\mathbf{HTL}}$ and $\mathbf{89}_{\mathbf{HTL}}$ (**Figure 3e,f**) and the comparison of compounds $\mathbf{40}_{\mathbf{HTL}}$, $\mathbf{44}_{\mathbf{HTL}}$, $\mathbf{1}_{\mathbf{HTL}}$, and $\mathbf{90}_{\mathbf{HTL}}$ (**Figure S10**), the fluorophores were excited with a 647 nm laser. To minimize drift during imaging, the incubation chamber was fully thermally equilibrated, and all imaging was conducted in an isolated ultra-clean room with minimal mechanical vibrations. The microscope, laser lines, and camera integration were controlled using the Nikon NIS-Elements software. Cells with obvious movement during acquisition were removed from further analysis. SMT analysis was based on multiple target tracing[21] (MTT) with tracking parameters similar to previous work.[19] For SPT brightness (photon counts/molecule/s), intensity values from sparsely-labeled molecules were analyzed. For the comparison of HaloTag ligands $\mathbf{59}_{\mathbf{HTL}}$ and $\mathbf{89}_{\mathbf{HTL}}$ (**Figure 3e,f**): n = 147,298 single-molecule events using $\mathbf{59}_{\mathbf{HTL}}$; n = 100,392 single-molecule events using $\mathbf{89}_{\mathbf{HTL}}$. For the comparison of dyes $\mathbf{40}_{\mathbf{HTL}}$, $\mathbf{44}_{\mathbf{HTL}}$, $\mathbf{1}_{\mathbf{HTL}}$, and $\mathbf{90}_{\mathbf{HTL}}$ (**Figure S10**): n = 655,357 single-molecule events using $\mathbf{40}_{\mathbf{HTL}}$; n = 1,048,575 single-molecule events using $\mathbf{44}_{\mathbf{HTL}}$; n = 424,136 single-molecule events using $\mathbf{1}_{\mathbf{HTL}}$; n = 564,263 single-molecule events using $\mathbf{89}_{\mathbf{HTL}}$. For the track length analysis, single-molecule trajectories of each cell with at least >5 constitutive frames were combined for each condition. For the comparison of dyes $\mathbf{40}_{\mathbf{HTL}}$, $\mathbf{44}_{\mathbf{HTL}}$, $\mathbf{1}_{\mathbf{HTL}}$, and $\mathbf{90}_{\mathbf{HTL}}$ (**Figure S10**): n = 53 cells using $\mathbf{40}_{\mathbf{HTL}}$; n = 62 cells using $\mathbf{44}_{\mathbf{HTL}}$; n = 54 cells using $\mathbf{1}_{\mathbf{HTL}}$; n = 62 cells using $\mathbf{89}_{\mathbf{HTL}}$.

cAMP FLIM–FRET sensor ScAMPI (Figure 5). The Epac-based cAMP sensor "Epac-S^{H189}" was obtained from Addgene (Plasmid #170348). This sensor consists of a mTurquoise fluorescent protein and a tdDark Venus protein linked via the Epac1 protein with the following sequence:

MVSKGEELFT GVVPILVELD GDVNGHKFSV SGEGEGDATY GKLTLKFICT TGKLPVPWPT LVTTLSWGVQ
CFARYPDHMK QHDFFKSAMP EGYVQERTIF FKDDGNYKTR AEVKFEGDTL VNRIELKGID FKEDGNILGH
KLEYNYFSDN VYITADKQKN GIKANFKIRH NIEDGGVQLA DHYQQNTPIG DGPVLLPDNH YLSTQSKLSK
DPNEKRDHMV LLEFVTAARY QPVGTHEMEE ELAEAVALLS QRGPDALLTV ALRKPPGQRT DEELDLIFEE
LLHIKAVAHL SNSVKRELAA VLLFEPHSKA GTVLFSQGDK GTSWYIIWKG SVNVVTHGKG LVTTLHEGDD
FGELALVNDA PRAATIILRE DNCHFLRVDK QDFNRIIKDV EAKTMRLEEH GKVVLVLERA SQGAGPSRPP
TPGRNRYTVM SGTPEKILEL LLEAMGPDSS AHDPTETFLS DFLLTHRVFM PSAQLCAALL HHFHVEPAGG
SEQERSTYVC NKRQQILRLV SQWVALYGSM LHTDPVATSF LQKLSDLVGR DTRLSNLLRE QWPERRRCHR
LENGCGNASP QMKARNLPVW LPNQDEPLPG SSCAIQVGDK VPYDICRPDH SVLTLQLPVT ASVREVMAAL
AQEDGWTKGQ VLVKVNSAGD AIGLQPDARG VATSLGLNER LFVVNPQEAH ELIPHPDQLG PTVGSAEGLD
LVSAKDLAGQ LTDHDWSLFN SIHQVELIHY VLGPQHLRDV TTANLERFMR RFNELQYWVA TELCLCPVPG
PRAQLLRKFI KLAAHLKEQK NLNSFFAVMF GLSNSAISRL AHTWERLPHK VRKLYSALER LLDPSWNHRV
YRLALAKLSP PVIPFMPLLL KDMAAIHEGN HTLVENLINF EKMRMMARAA RMLHHCRSHN PVPLSPLRSR
VSHLHEDSQV ARISTCSEQS LSTRSPASTW AYVQQLKVID NQRELSRLSR ELEPASELMD GGVQLADHYQ
QNTPIGDGPV LLPDNHYLSY QSALSKDPNE KRDHMVLLEF VTAAGITLGM DELYKGGSGG MVSKGEELFT
GVVPILVELD GDVNGHKFSV SGEGEGDATY GKLTLKLICT TGKLPVPWPT LVTTLGYGLQ CFARYPDHMK
QHDFFKSAMP EGYVQERTIF FKDDGNYKTR AEVKFEGDTL VNRIELKGID FKEDGNILGH KLEYNWNSHN
VYITADKQKN GIKANFKIRH NIEPNFVFLI GAAGILFVSS ELMDGGVQLA DHYQQNTPIG DGPVLLPDNH
YLSYQSALSK DPNEKRDHMV LLEFVTAAGI TLGMDELYKG GSGGMVSKGE ELFTGVVPIL VELDGDVNGH
KFSVSGEGEG DATYGKLTLK LICTTGKLPV PWPTLVTTLG YGLQCFARYP DHMKQHDFFK SAMPEGYVQE
RTIFFKDDGN YKTRAEVKFE GDTLVNRIEL KGIDFKEDGN ILGHKLEYNW NSHNVYITAD KQKNGIKANF
KIRHNIEPNS LEVNAARL*

To construct the SNAP-tag-Epac1-HaloTag sensor, the sequence encoding the tdDark Venus domain in Epac-S^{H189} was first replaced with the HaloTag sequence using Nhe1 and Asc1 fragment ligation (NEB), creating a construct with mTurquoise and HaloTag flanking the Epac1 domain (*i.e*, mTurquoise-Epac1-HaloTag) with the following sequence:

MVSKGEELFT GVVPILVELD GDVNGHKFSV SGEGEGDATY GKLTLKFICT TGKLPVPWPT LVTTLSWGVQ
CFARYPDHMK QHDFFKSAMP EGYVQERTIF FKDDGNYKTR AEVKFEGDTL VNRIELKGID FKEDGNILGH
KLEYNYFSDN VYITADKQKN GIKANFKIRH NIEDGGVQLA DHYQQNTPIG DGPVLLPDNH YLSTQSKLSK
DPNEKRDHMV LLEFVTAARY QPVGTHEMEE ELAEAVALLS QRGPDALLTV ALRKPPGQRT DEELDLIFEE
LLHIKAVAHL SNSVKRELAA VLLFEPHSKA GTVLFSQGDK GTSWYIIWKG SVNVVTHGKG LVTTLHEGDD
FGELALVNDA PRAATIILRE DNCHFLRVDK QDFNRIIKDV EAKTMRLEEH GKVVLVLERA SQGAGPSRPP
TPGRNRYTVM SGTPEKILEL LLEAMGPDSS AHDPTETFLS DFLLTHRVFM PSAQLCAALL HHFHVEPAGG
SEQERSTYVC NKRQQILRLV SQWVALYGSM LHTDPVATSF LQKLSDLVGR DTRLSNLLRE QWPERRRCHR
LENGCGNASP QMKARNLPVW LPNQDEPLPG SSCAIQVGDK VPYDICRPDH SVLTLQLPVT ASVREVMAAL
AQEDGWTKGQ VLVKVNSAGD AIGLQPDARG VATSLGLNER LFVVNPQEAH ELIPHPDQLG PTVGSAEGLD
LVSAKDLAGQ LTDHDWSLFN SIHQVELIHY VLGPQHLRDV TTANLERFMR RFNELQYWVA TELCLCPVPG
PRAQLLRKFI KLAAHLKEQK NLNSFFAVMF GLSNSAISRL AHTWERLPHK VRKLYSALER LLDPSWNHRV
YRLALAKLSP PVIPFMPLLL KDMAAIHEGN HTLVENLINF EKMRMMARAA RMLHHCRSHN PVPLSPLRSR
VSHLHEDSQV ARISTCSEQS LSTRSPASTW AYVQQLKVID NQRELSRLSR ELEPASELEI GTGFPFDPHY
VEVLGERMHY VDVGPRDGTP VLFLHGNPTS SYVWRNIIPH VAPTHRCIAP DLIGMGKSDK PDLGYFFDDH
VRFMDAFIEA LGLEEVVLVI HDWGSALGFH WAKRNPERVK GIAFMEFIRP IPTWDEWPEF ARETFQAFRT
TDVGRKLIID QNVFIEGTLP MGVVRPLTEV EMDHYREPFL NPVDREPLWR FPNELPIAGE PANIVALVEE
YMDWLHQSPV PKLLFWGTPG VLIPPAEAAR LAKSLPNCKA VDIGPGLNLL QEDNPDLIGS EIARWLSTLE
ISG*

A gene block encoding the SNAP-tag self-labeling protein (IDT) was then used to replace the portion encoding the mTurquoise domain in the mTurquoise-Epac1-HaloTag using HindIII and AgeI fragment ligation. The sequence of this final plasmid encoding ScAMPI (*i.e*, SNAP-tag-Epac1-HaloTag) was confirmed through sequence analysis (Genewiz) with the following sequence:

```
MDKDCEMKRT TLDSPLGKLE LSGCEQGLHR IIFLGKGTSA ADAVEVPAPA AVLGGPEPLM QATAWLNAYF
HQPEAIEEFP VPALHHPVFQ QESFTRQVLW KLLKVVKFGE VISYSHLAAL AGNPAATAAV KTALSGNPVP
ILIPCHRVVQ GDLDVGGYEG GLAVKEWLLA HEGHRLGKPG LGRYQPVGTH EMEEELAEAV ALLSQRGPDA
LLTVALRKPP GQRTDEELDL IFEELLHIKA VAHLSNSVKR ELAAVLLFEP HSKAGTVLFS QGDKGTSWYI
IWKGSVNVVT HGKGLVTTLH EGDDFGELAL VNDAPRAATI ILREDNCHFL RVDKQDFNRI IKDVEAKTMR
LEEHGKVVLV LERASQGAGP SRPPTPGRNR YTVMSGTPEK ILELLLEAMG PDSSAHDPTE TFLSDFLLTH
RVFMPSAQLC AALLHHFHVE PAGGSEQERS TYVCNKRQQI LRLVSQWVAL YGSMLHTDPV ATSFLQKLSD
LVGRDTRLSN LLREQWPERR RCHRLENGCG NASPQMKARN LPVWLPNQDE PLPGSSCAIQ VGDKVPYDIC
RPDHSVLTLQ LPVTASVREV MAALAQEDGW TKGQVLVKVN SAGDAIGLQP DARGVATSLG LNERLFVVNP
QEAHELIPHP DQLGPTVGSA EGLDLVSAKD LAGQLTDHDW SLFNSIHQVE LIHYVLGPQH LRDVTTANLE
RFMRRFNELQ YWVATELCLC PVPGPRAQLL RKFIKLAAHL KEQKNLNSFF AVMFGLSNSA ISRLAHTWER
LPHKVRKLYS ALERLLDPSW NHRVYRLALA KLSPPVIPFM PLLLKDMAAI HEGNHTLVEN LINFEKMRMM
ARAARMLHHC RSHNPVPLSP LRSRVSHLHE DSQVARISTC SEQSLSTRSP ASTWAYVQQL KVIDNQRELS
RLSRELEPAS ELEIGTGFPF DPHYVEVLGE RMHYVDVGPR DGTPVLFLHG NPTSSYVWRN IIPHVAPTHR
CIAPDLIGMG KSDKPDLGYF FDDHVRFMDA FIEALGLEEV VLVIHDWGSA LGFHWAKRNP ERVKGIAFME
FIRPIPTWDE WPEFARETFQ AFRTTDVGRK LIIDQNVFIE GTLPMGVVRP LTEVEMDHYR EPFLNPVDRE
PLWRFPNELP IAGEPANIVA LVEEYMDWLH QSPVPKLLFW GTPGVLIPPA EAARLAKSLP NCKAVDIGPG
LNLLQEDNPD LIGSEIARWL STLEISG*
```

To evaluate this semisynthetic system in cells, U2OS cells (ATCC) were maintained in DMEM (Corning) containing 10% v/v FBS (ATCC), 1% v/v L-glutamine (Gibco) and 1% v/v Pen/Strep (Gibco). Cells were transfected with plasmid DNA using nucleofection (Lonza) and labeled 48 h post-transfection with 100 nM of JFX_{612}-SNAP-tag (**88_{STL}**) and 200 nM of the JQ_{645}-HaloTag ligand (**65_{HTL}**) for 1 h. Cells were washed with 1× PBS (3×) and recovered in fresh medium for 30 min before imaging. Fluorescence lifetime imaging was performed with a Leica SP8 Falcon using LASX software for image acquisition and analysis. Excitation was achieved with a pulsed white light laser at 40 and 20 MHz using a 40× oil immersion objective at 1.3 NA. Fluorescence emission was collected by the HyD detector. The SNAP-tag donor dye was excited at 610 nm and emission was collected at 620–720 nm. FLIM images were acquired before and 10 min after stimulation with 25 µM forskolin. FLIM data were fitted with two-component exponential fitting function yielding quenched lifetime (τ_1) and unquenched lifetime (τ_2). Mean amplitude weighted lifetime image (τ_{mean}) was calculated by fixing the two lifetimes and performing an image fit for their respective amplitudes (a_1 and a_2). FRET efficiency was calculated using the mean lifetime obtained in absence ($\tau_{mean,-forskolin}$) and ($\tau_{mean,+forskolin}$) presence of forskolin as $1-(\tau_{mean,-forskolin}/\tau_{mean,+forskolin})$.

REFERENCES

(1) Grimm, J. B.; Brown, T. A.; Tkachuk, A. N.; Lavis, L. D. General synthetic method for Si-fluoresceins and Si-rhodamines. *ACS Cent. Sci.* **2017**, *3*, 975-985.

(2) Wang, D. X.; Wang, L. L.; Xue, L.; Zhou, D. B.; Feng, S. Y.; Zhao, X. Tetrahedral silicon-based luminescent molecules: Synthesis and comparison of thermal and photophysical properties by various effect factors. *J. Organomet. Chem.* **2013**, *735*, 58-64.

(3) Dwight, S. J.; Levin, S. Scalable regioselective synthesis of rhodamine dyes. *Org. Lett.* **2016**, *18*, 5316-5319.

(4) Grimm, J. B.; Klein, T.; Kopek, B. G.; Shtengel, G.; Hess, H. F.; Sauer, M.; Lavis, L. D. Synthesis of a far-red photoactivatable silicon-containing rhodamine for super-resolution microscopy. *Angew. Chem. Int. Ed.* **2016**, *55*, 1723-1727.

(5) Grimm, J. B.; Xie, L.; Casler, J. C.; Patel, R.; Tkachuk, A. N.; Falco, N.; Choi, H.; Lippincott-Schwartz, J.; Brown, T. A.; Glick, B. S.; Liu, Z.; Lavis, L. D. A general method to improve fluorophores using deuterated auxochromes. *JACS Au* **2021**, *1*, 690-696.

(6) Grimm, J. B.; Sung, A. J.; Legant, W. R.; Hulamm, P.; Matlosz, S. M.; Betzig, E.; Lavis, L. D. Carbofluoresceins and carborhodamines as scaffolds for high-contrast fluorogenic probes. *ACS Chem. Biol.* **2013**, *8*, 1303-1310.

(7) Grimm, J. B.; Gruber, T. D.; Ortiz, G.; Brown, T. A.; Lavis, L. D. Virginia Orange: A versatile, red-shifted fluorescein scaffold for single- and dual-input fluorogenic probes. *Bioconjugate Chem.* **2016**, *27*, 474-480.

(8) Egawa, T.; Koide, Y.; Hanaoka, K.; Komatsu, T.; Terai, T.; Nagano, T. Development of a fluorescein analogue, TokyoMagenta, as a novel scaffold for fluorescence probes in red region. *Chem. Commun.* **2011**, *47*, 4162-4164.

(9) Lukinavičius, G.; Reymond, L.; Umezawa, K.; Sallin, O.; D'Este, E.; Gottfert, F.; Ta, H.; Hell, S. W.; Urano, Y.; Johnsson, K. Fluorogenic probes for multicolor imaging in living cells. *J. Am. Chem. Soc.* **2016**, *138*, 9365-9368.

(10) Sun, W.-C.; Gee, K. R.; Klaubert, D. H.; Haugland, R. P. Synthesis of fluorinated fluoresceins. *J. Org. Chem.* **1997**, *62*, 6469-6475.

(11) Grimm, J. B.; English, B. P.; Chen, J.; Slaughter, J. P.; Zhang, Z.; Revyakin, A.; Patel, R.; Macklin, J. J.; Normanno, D.; Singer, R. H.; Lionnet, T.; Lavis, L. D. A general method to improve fluorophores for live-cell and single-molecule microscopy. *Nat. Methods* **2015**, *12*, 244-250.

(12) Grimm, J. B.; Muthusamy, A. K.; Liang, Y.; Brown, T. A.; Lemon, W. C.; Patel, R.; Lu, R.; Macklin, J. J.; Keller, P. J.; Ji, N.; Lavis, L. D. A general method to fine-tune fluorophores for live-cell and in vivo imaging. *Nat. Methods* **2017**, *14*, 987-994.

(13) Suzuki, K.; Kobayashi, A.; Kaneko, S.; Takehira, K.; Yoshihara, T.; Ishida, H.; Shiina, Y.; Oishi, S.; Tobita, S. Reevaluation of absolute luminescence quantum yields of standard solutions using a spectrometer with an integrating sphere and a back-thinned CCD detector. *Phys. Chem. Chem. Phys.* **2009**, *11*, 9850-9860.

(14) Encell, L. P.; Friedman Ohana, R.; Zimmerman, K.; Otto, P.; Vidugiris, G.; Wood, M. G.; Los, G. V.; McDougall, M. G.; Zimprich, C.; Karassina, N.; Learish, R. D.; Hurst, R.; Hartnett, J.; Wheeler, S.; Stecha, P.; English, J.; Zhao, K.; Mendez, J.; Benink, H. A.; Murphy, N.; Daniels, D. L.; Slater, M. R.; Urh, M.; Darzins, A.; Klaubert,

D. H.; Bulleit, R. F.; Wood, K. V. Development of a dehalogenase-based protein fusion tag capable of rapid, selective and covalent attachment to customizable ligands. *Curr. Chem. Genomics* **2012**, *6*, 55-71.

(15) Grimm, J. B.; Tkachuk, A. N.; Xie, L.; Choi, H.; Mohar, B.; Falco, N.; Schaefer, K.; Patel, R.; Zheng, Q.; Liu, Z.; Lippincott-Schwartz, J.; Brown, T. A.; Lavis, L. D. A general method to optimize and functionalize red-shifted rhodamine dyes. *Nat. Methods* **2020**, *17*, 815-821.

(16) Schindelin, J.; Arganda-Carreras, I.; Frise, E.; Kaynig, V.; Longair, M.; Pietzsch, T.; Preibisch, S.; Rueden, C.; Saalfeld, S.; Schmid, B.; Tinevez, J. Y.; White, D. J.; Hartenstein, V.; Eliceiri, K.; Tomancak, P.; Cardona, A. Fiji: An open-source platform for biological-image analysis. *Nat. Methods* **2012**, *9*, 676-682.

(17) Zheng, Q.; Ayala, A. X.; Chung, I.; Weigel, A. V.; Ranjan, A.; Falco, N.; Grimm, J. B.; Tkachuk, A. N.; Wu, C.; Lippincott-Schwartz, J.; Singer, R. H.; Lavis, L. D. Rational design of fluorogenic and spontaneously blinking labels for super-resolution imaging. *ACS Cent. Sci.* **2019**, *5*, 1602-1613.

(18) Wilhelm, J.; Kuhn, S.; Tarnawski, M.; Gotthard, G.; Tunnermann, J.; Tanzer, T.; Karpenko, J.; Mertes, N.; Xue, L.; Uhrig, U.; Reinstein, J.; Hiblot, J.; Johnsson, K. Kinetic and structural characterization of the self-labeling protein tags HaloTag7, SNAP-tag, and CLIP-tag. *Biochemistry* **2021**, *60*, 2560-2575.

(19) Xie, L.; Torigoe, S. E.; Xiao, J.; Mai, D. H.; Li, L.; Davis, F. P.; Dong, P.; Marie-Nelly, H.; Grimm, J.; Lavis, L.; Darzacq, X.; Cattoglio, C.; Liu, Z.; Tjian, R. A dynamic interplay of enhancer elements regulates Klf4 expression in naïve pluripotency. *Genes Dev.* **2017**, *31*, 1795-1808.

(20) Chen, J.; Zhang, Z.; Li, L.; Chen, B.-C.; Revyakin, A.; Hajj, B.; Legant, W.; Dahan, M.; Lionnet, T.; Betzig, E.; Tjian, R.; Liu, Z. Single-molecule dynamics of enhanceosome assembly in embryonic stem cells. *Cell* **2014**, *156*, 1274-1285.

(21) Sergé, A.; Bertaux, N.; Rigneault, H.; Marguet, D. Dynamic multiple-target tracing to probe spatiotemporal cartography of cell membranes. *Nat. Methods* **2008**, *5*, 687-694.

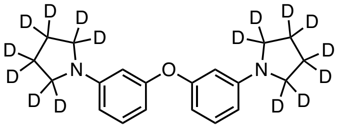

9

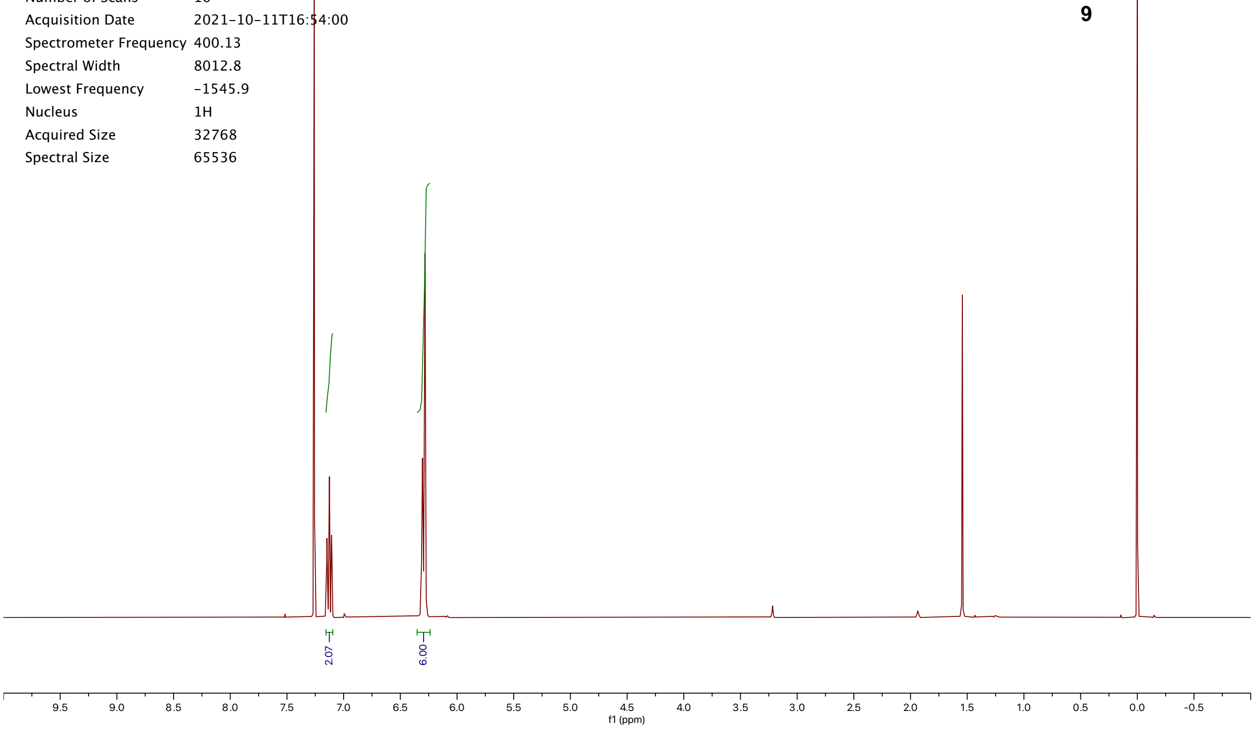
Origin Bruker BioSpin GmbH
Solvent CDCl3
Temperature 295.5
Pulse Sequence zg30
Experiment 1D
Number of Scans 16
Acquisition Date 2021-10-11T16:54:00
Spectrometer Frequency 400.13
Spectral Width 8012.8
Lowest Frequency -1545.9
Nucleus 1H
Acquired Size 32768
Spectral Size 65536
2.07
6.00
9.5 9.0 8.5 8.0 7.5 7.0 6.5 6.0 5.5 5.0 4.5 4.0 3.5 3.0 2.5 2.0 1.5 1.0 0.5 0.0 -0.5
f1 (ppm)

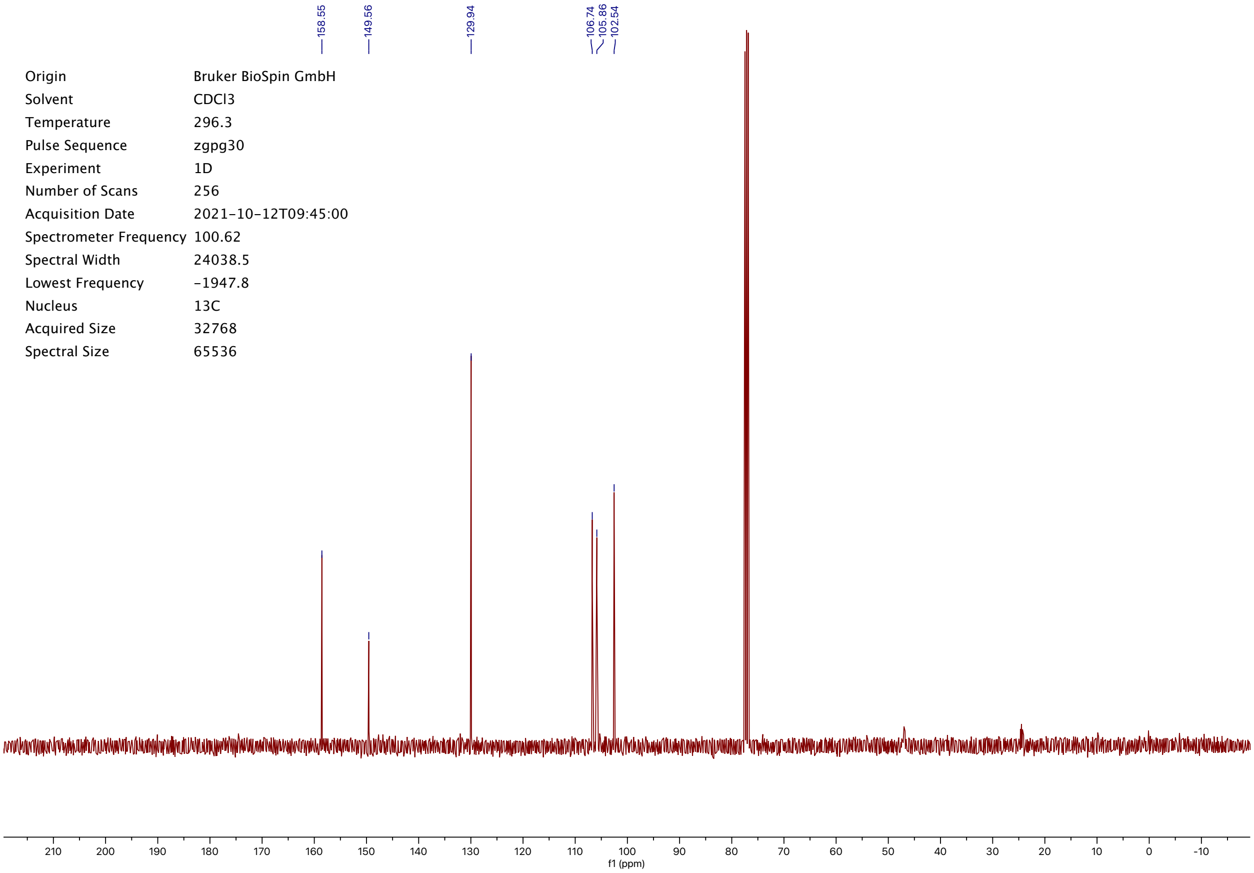
158.55
149.56
129.94
106.74
105.86
102.54
Origin Bruker BioSpin GmbH
Solvent CDCl3
Temperature 296.3
Pulse Sequence zgpg30
Experiment 1D
Number of Scans 256
Acquisition Date 2021-10-12T09:45:00
Spectrometer Frequency 100.62
Spectral Width 24038.5
Lowest Frequency -1947.8
Nucleus 13C
Acquired Size 32768
Spectral Size 65536
210 200 190 180 170 160 150 140 130 120 110 100 90 80 70 60 50 40 30 20 10 0 -10
f1 (ppm)

Origin	Bruker BioSpin GmbH
Solvent	CDCl3
Temperature	295.4
Pulse Sequence	zg30
Experiment	1D
Number of Scans	16
Acquisition Date	2020-01-30T16:04:00
Spectrometer Frequency	400.13
Spectral Width	8012.8
Lowest Frequency	-1547.3
Nucleus	1H
Acquired Size	32768
Spectral Size	65536

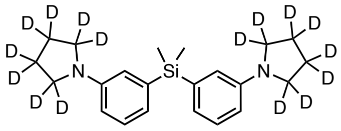

26

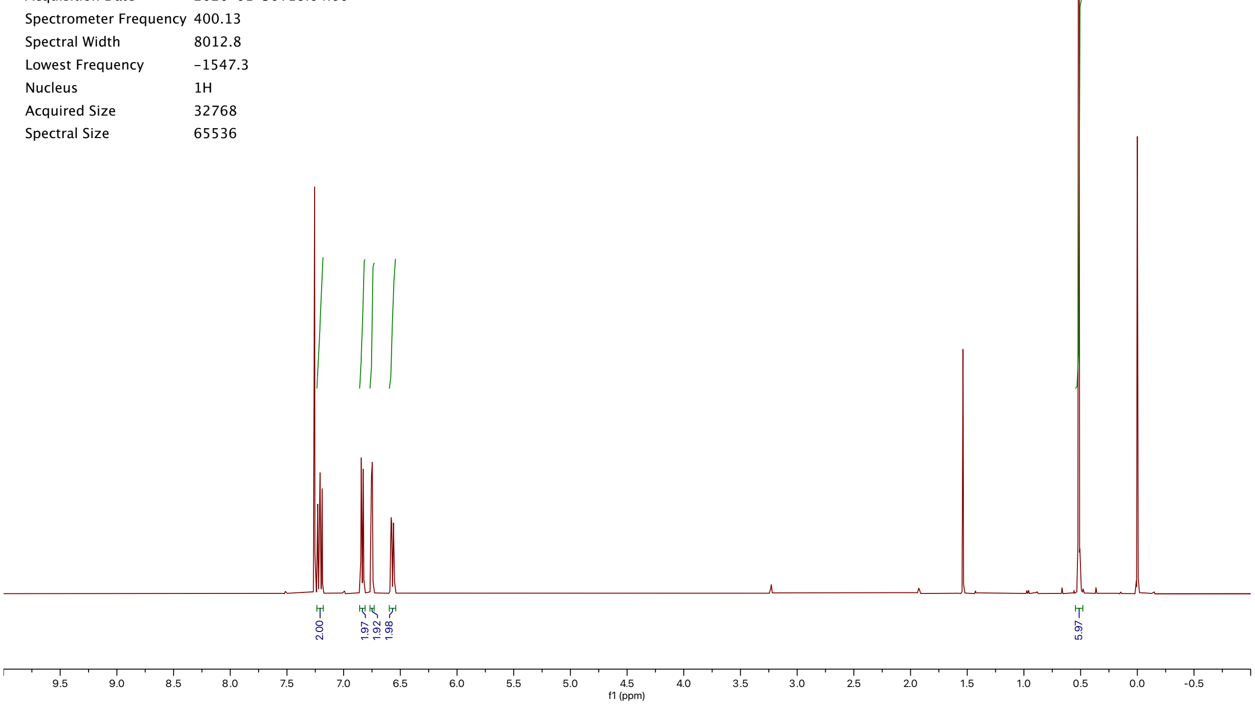

Origin	Bruker BioSpin GmbH
Solvent	CDCl3
Temperature	296.1
Pulse Sequence	zgpg30
Experiment	1D
Number of Scans	256
Acquisition Date	2020-02-11T09:50:00
Spectrometer Frequency	100.62
Spectral Width	24038.5
Lowest Frequency	-1948.8
Nucleus	13C
Acquired Size	32768
Spectral Size	65536

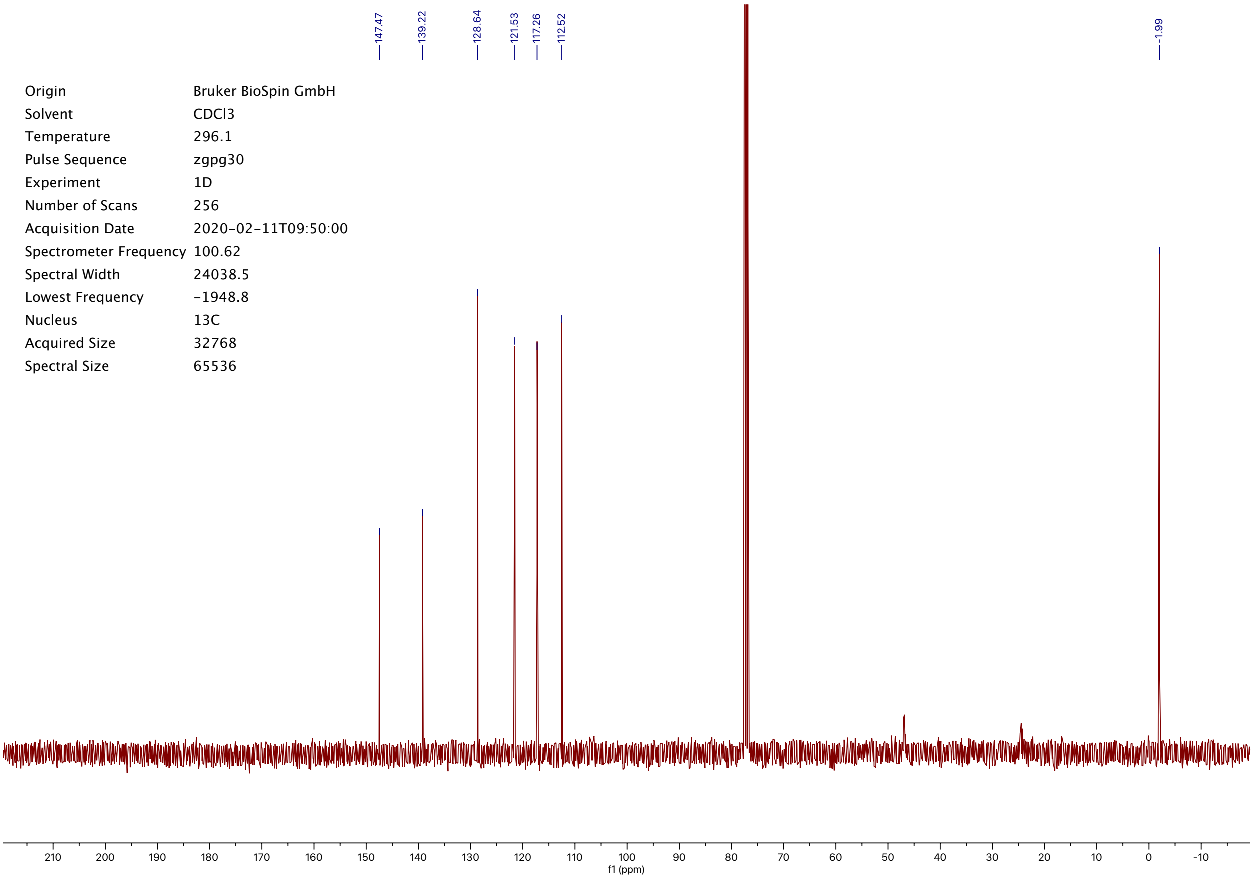

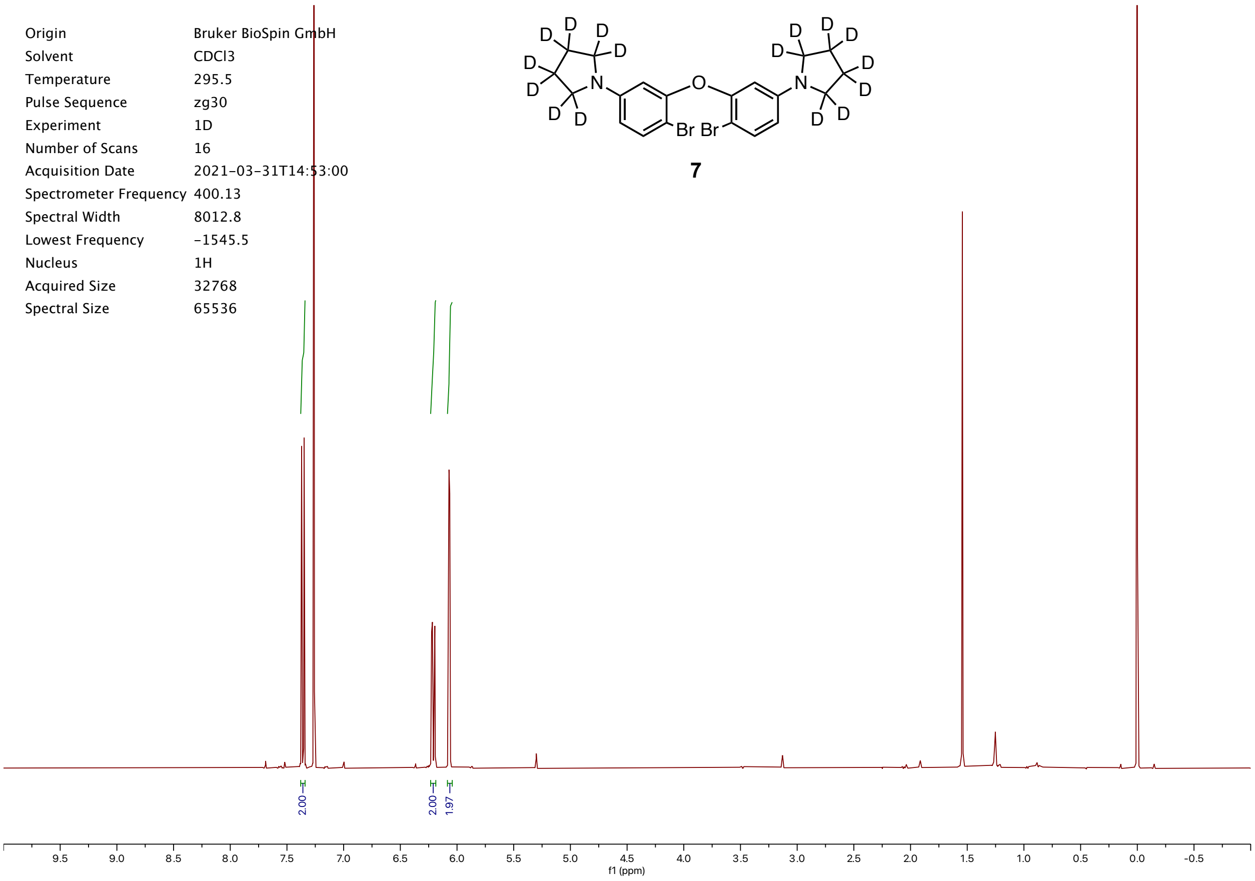
D
N
O
Br Br
7
Origin Bruker BioSpin GmbH
Solvent CDCl3
Temperature 295.5
Pulse Sequence zg30
Experiment 1D
Number of Scans 16
Acquisition Date 2021-03-31T14:53:00
Spectrometer Frequency 400.13
Spectral Width 8012.8
Lowest Frequency -1545.5
Nucleus 1H
Acquired Size 32768
Spectral Size 65536
2.00
2.00
1.97
9.5 9.0 8.5 8.0 7.5 7.0 6.5 6.0 5.5 5.0 4.5 4.0 3.5 3.0 2.5 2.0 1.5 1.0 0.5 0.0 -0.5
f1 (ppm)

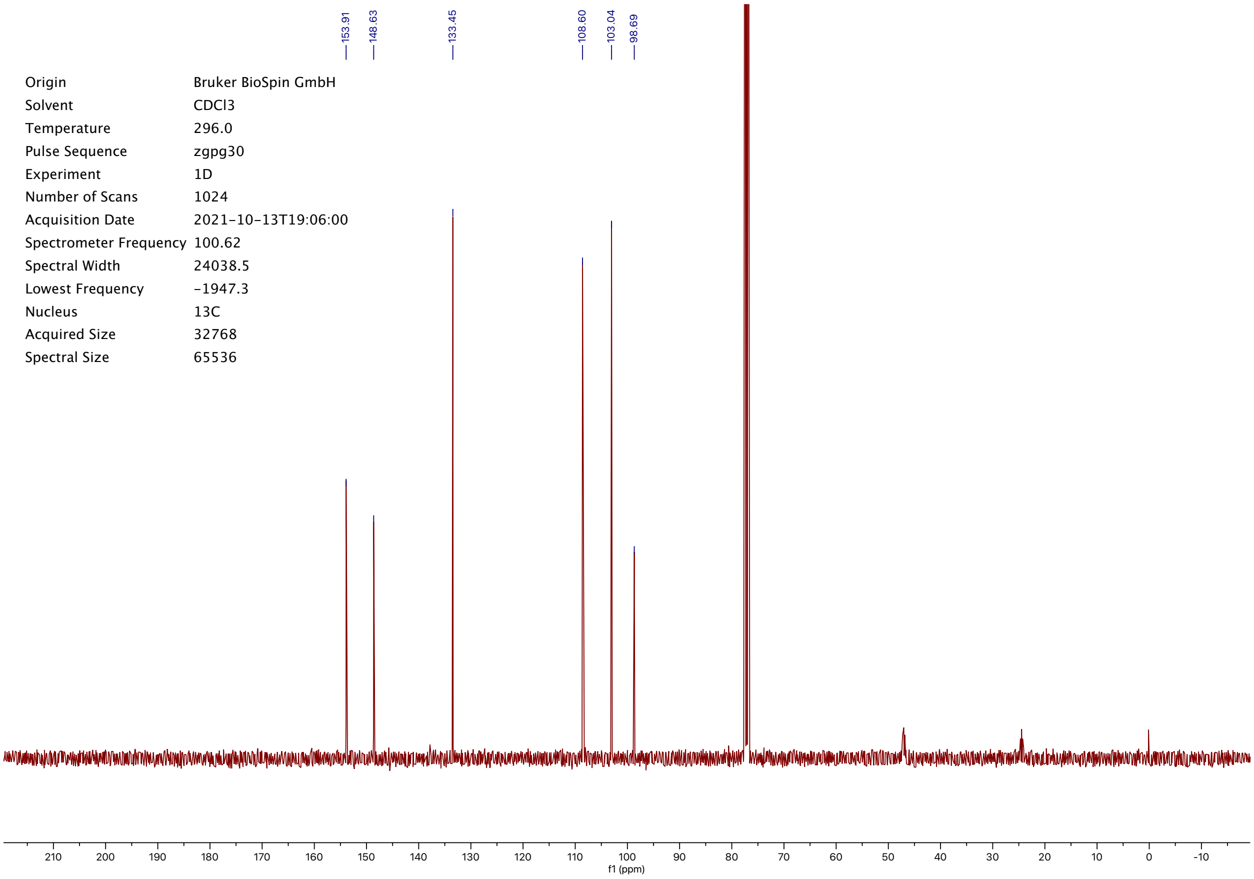
153.91
148.63
133.45
108.60
103.04
98.69
Origin Bruker BioSpin GmbH
Solvent CDCl3
Temperature 296.0
Pulse Sequence zgpg30
Experiment 1D
Number of Scans 1024
Acquisition Date 2021-10-13T19:06:00
Spectrometer Frequency 100.62
Spectral Width 24038.5
Lowest Frequency -1947.3
Nucleus 13C
Acquired Size 32768
Spectral Size 65536
210 200 190 180 170 160 150 140 130 120 110 100 90 80 70 60 50 40 30 20 10 0 -10
f1 (ppm)

Origin	Bruker BioSpin GmbH
Solvent	CDCl3
Temperature	300.0
Pulse Sequence	zg30
Experiment	1D
Number of Scans	16
Acquisition Date	2019-09-05T14:38:00
Spectrometer Frequency	400.13
Spectral Width	8012.8
Lowest Frequency	-1547.1
Nucleus	1H
Acquired Size	32768
Spectral Size	65536

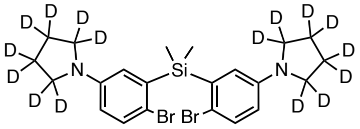

24

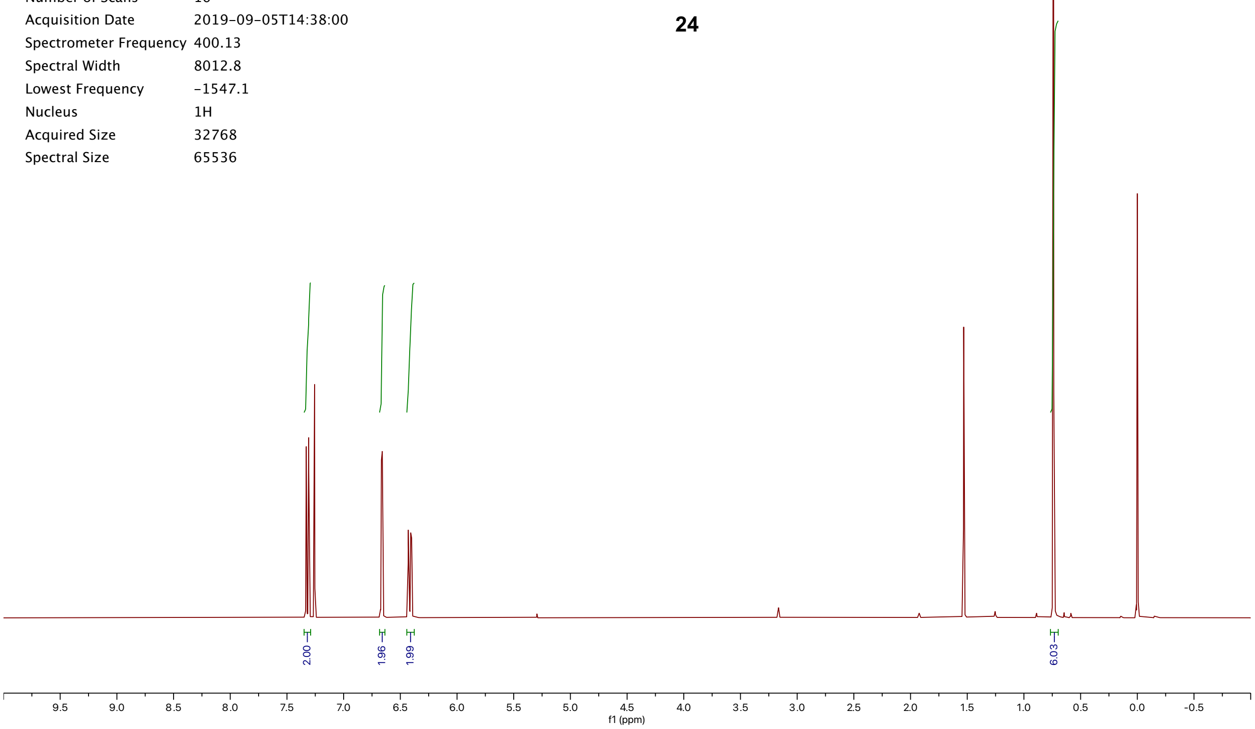

Origin	Bruker BioSpin GmbH
Solvent	CDCl3
Temperature	296.1
Pulse Sequence	zgpg30
Experiment	1D
Number of Scans	256
Acquisition Date	2020-02-11T15:38:00
Spectrometer Frequency	100.62
Spectral Width	24038.5
Lowest Frequency	-1947.0
Nucleus	13C
Acquired Size	32768
Spectral Size	65536

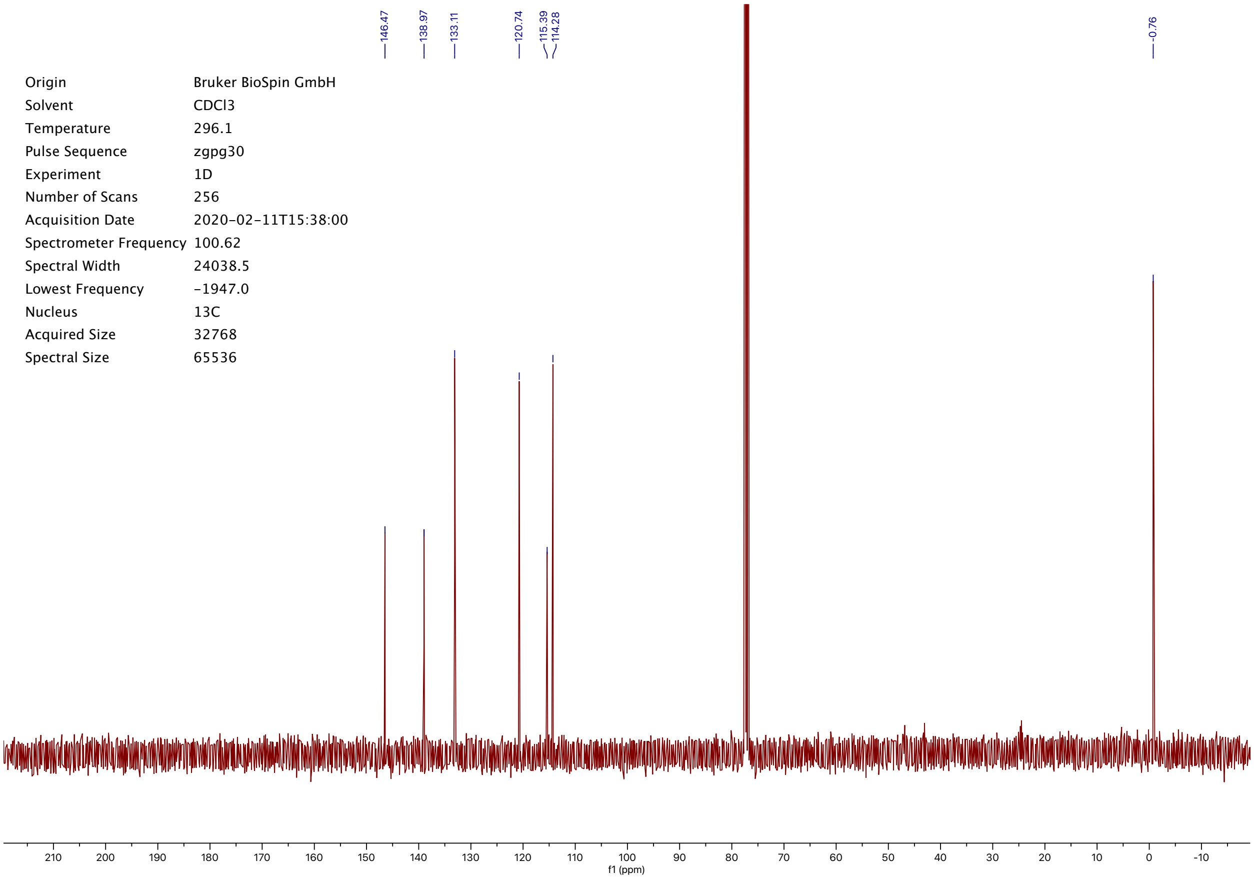

19

Origin	Bruker BioSpin GmbH
Solvent	CDCl3
Temperature	295.4
Pulse Sequence	zg30
Experiment	1D
Number of Scans	16
Acquisition Date	2020-09-25T09:08:00
Spectrometer Frequency	400.13
Spectral Width	8012.8
Lowest Frequency	-1546.4
Nucleus	1H
Acquired Size	32768
Spectral Size	65536

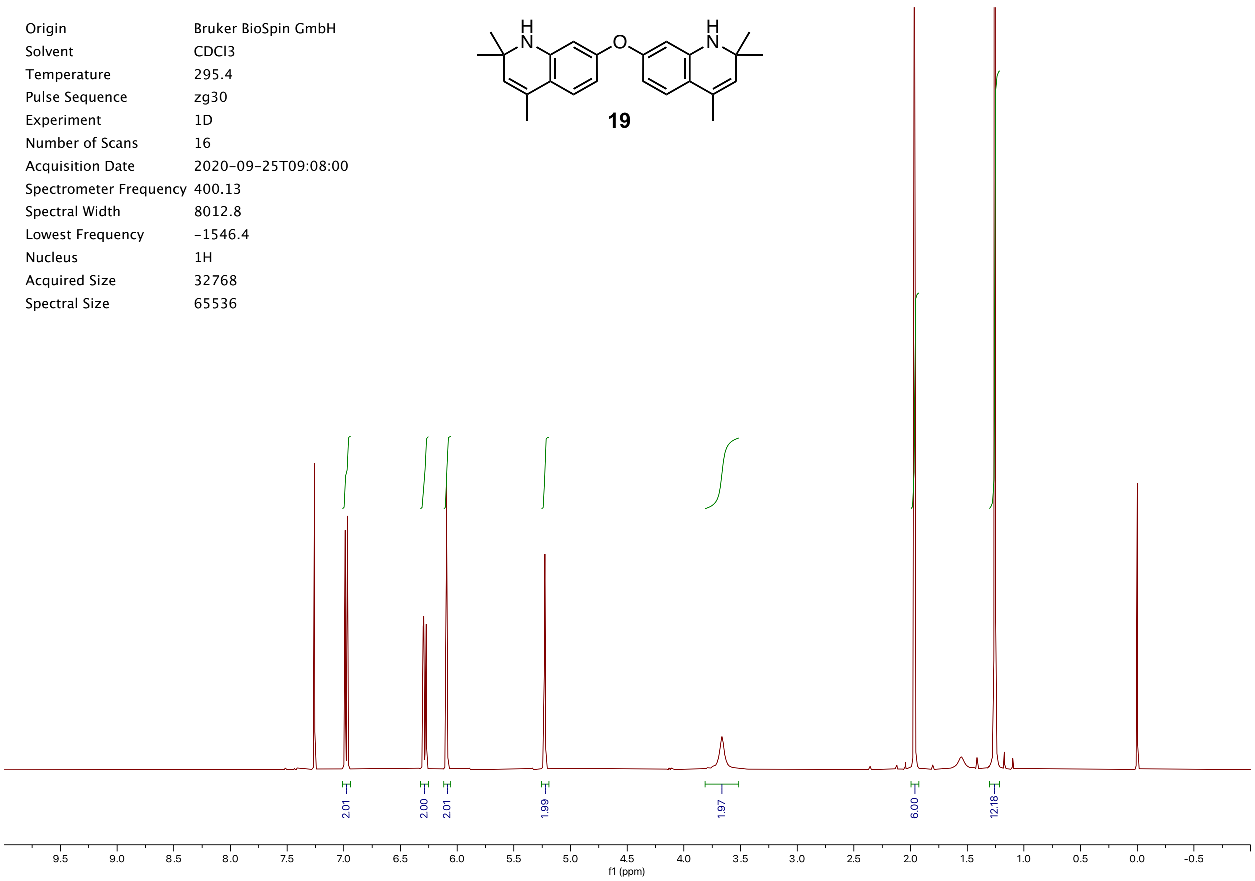

Origin	Bruker BioSpin GmbH
Solvent	CDCl3
Temperature	300.0
Pulse Sequence	zgpg30
Experiment	1D
Number of Scans	1024
Acquisition Date	2020-11-23T17:05:00
Spectrometer Frequency	100.62
Spectral Width	24038.5
Lowest Frequency	-1945.9
Nucleus	13C
Acquired Size	32768
Spectral Size	65536

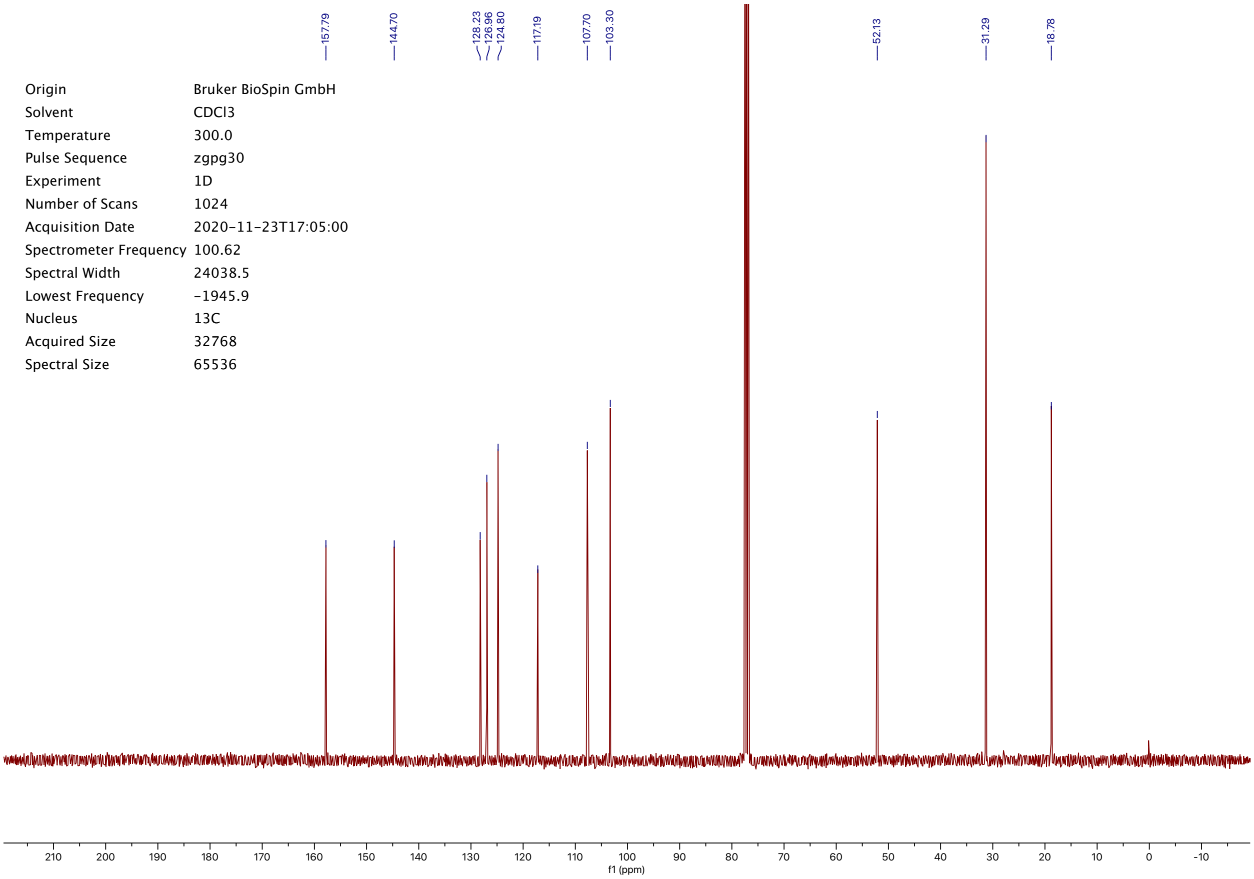

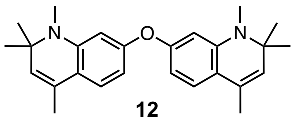

12

Origin	Bruker BioSpin GmbH
Solvent	CDCl3
Temperature	295.4
Pulse Sequence	zg30
Experiment	1D
Number of Scans	16
Acquisition Date	2020-10-02T12:06:00
Spectrometer Frequency	400.13
Spectral Width	8012.8
Lowest Frequency	-1545.8
Nucleus	1H
Acquired Size	32768
Spectral Size	65536

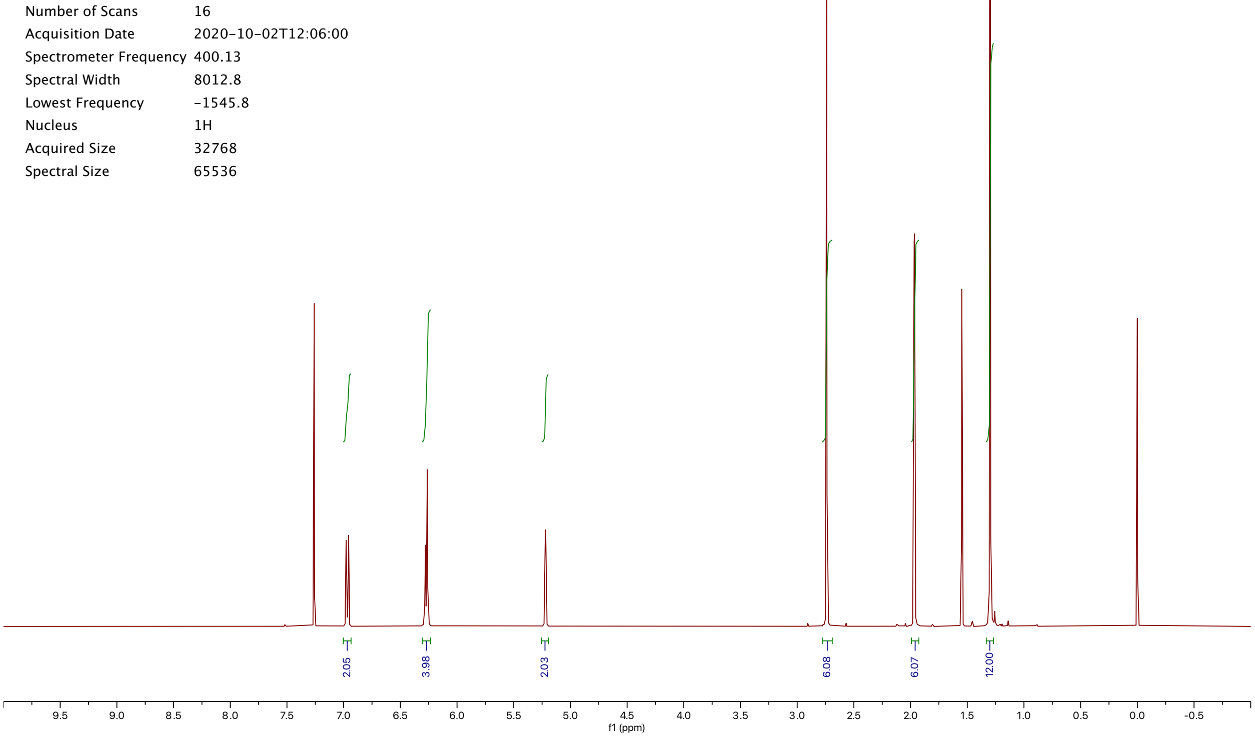

Origin	Bruker BioSpin GmbH
Solvent	CDCl3
Temperature	300.0
Pulse Sequence	zgpg30
Experiment	1D
Number of Scans	1024
Acquisition Date	2020-11-23T20:05:00
Spectrometer Frequency	100.62
Spectral Width	24038.5
Lowest Frequency	-1946.6
Nucleus	13C
Acquired Size	32768
Spectral Size	65536

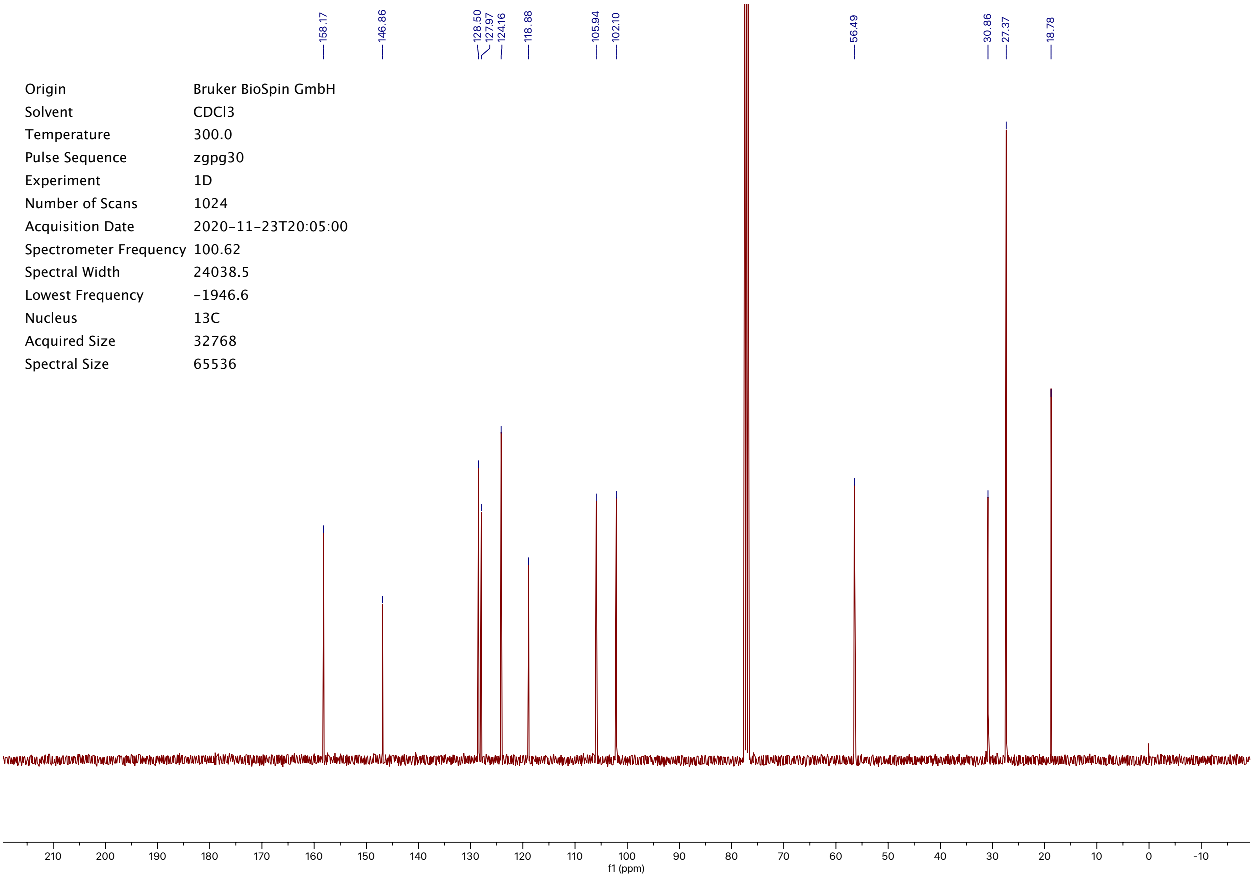

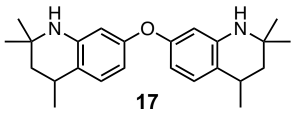

17

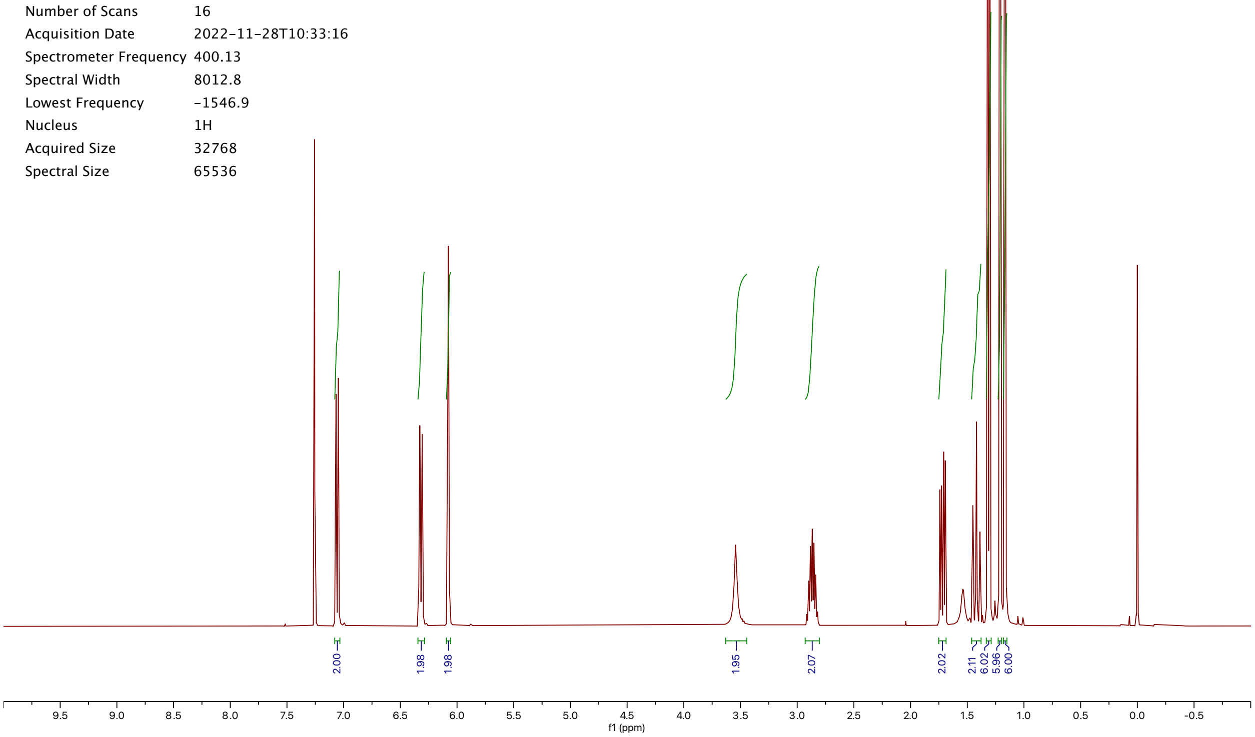

Origin Bruker BioSpin GmbH
Solvent CDCl3
Temperature 300.0
Pulse Sequence zg30
Experiment 1D
Number of Scans 16
Acquisition Date 2022-11-28T10:33:16
Spectrometer Frequency 400.13
Spectral Width 8012.8
Lowest Frequency -1546.9
Nucleus 1H
Acquired Size 32768
Spectral Size 65536
2.00
1.98
1.98
1.95
2.07
2.02
2.11
6.02
5.96
6.00
9.5 9.0 8.5 8.0 7.5 7.0 6.5 6.0 5.5 5.0 4.5 4.0 3.5 3.0 2.5 2.0 1.5 1.0 0.5 0.0 -0.5
f1 (ppm)

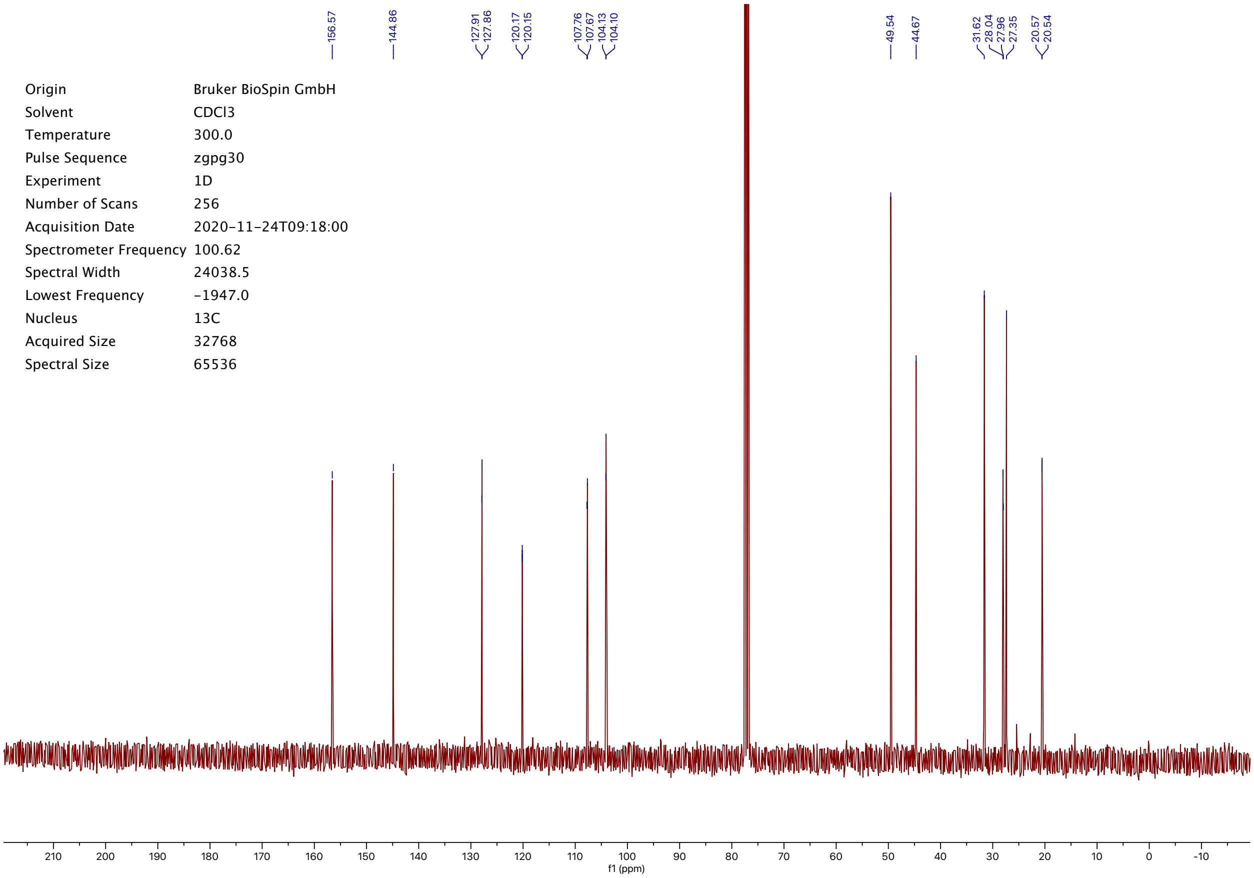

156.57
144.86
127.91
127.86
120.17
120.15
107.76
107.67
104.13
104.10
49.54
44.67
31.62
28.04
27.96
27.35
20.57
20.54
Origin Bruker BioSpin GmbH
Solvent CDCl3
Temperature 300.0
Pulse Sequence zgpg30
Experiment 1D
Number of Scans 256
Acquisition Date 2020-11-24T09:18:00
Spectrometer Frequency 100.62
Spectral Width 24038.5
Lowest Frequency -1947.0
Nucleus 13C
Acquired Size 32768
Spectral Size 65536
210 200 190 180 170 160 150 140 130 120 110 100 90 80 70 60 50 40 30 20 10 0 -10
f1 (ppm)

Origin	Bruker BioSpin GmbH
Solvent	CDCl3
Temperature	296.2
Pulse Sequence	zg30
Experiment	1D
Number of Scans	16
Acquisition Date	2012-03-22T20:03:00
Spectrometer Frequency	400.13
Spectral Width	8223.7
Lowest Frequency	-1655.0
Nucleus	1H
Acquired Size	32768
Spectral Size	65536

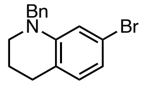

S4

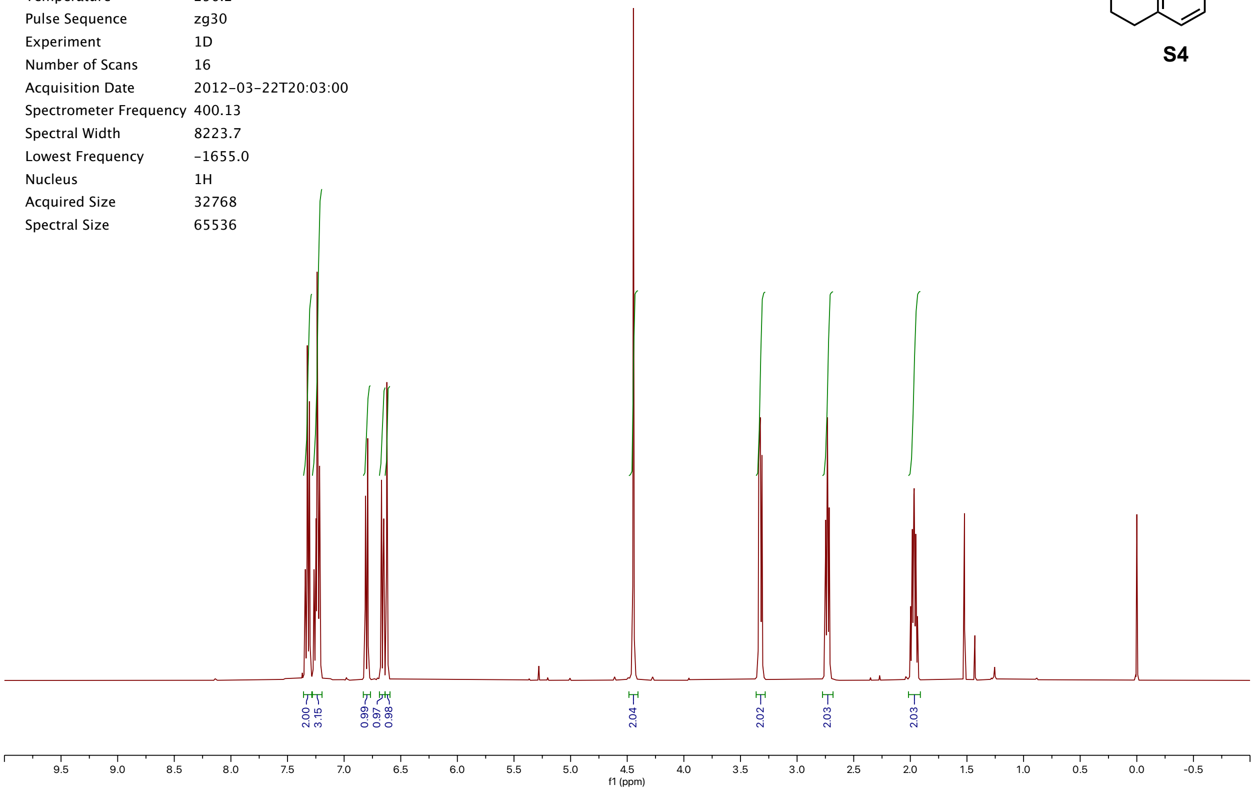

Origin	Bruker BioSpin GmbH
Solvent	CDCl3
Temperature	297.3
Pulse Sequence	zgpg30
Experiment	1D
Number of Scans	2048
Acquisition Date	2012-03-22T22:02:00
Spectrometer Frequency	100.62
Spectral Width	24038.5
Lowest Frequency	-1945.9
Nucleus	13C
Acquired Size	32768
Spectral Size	65536

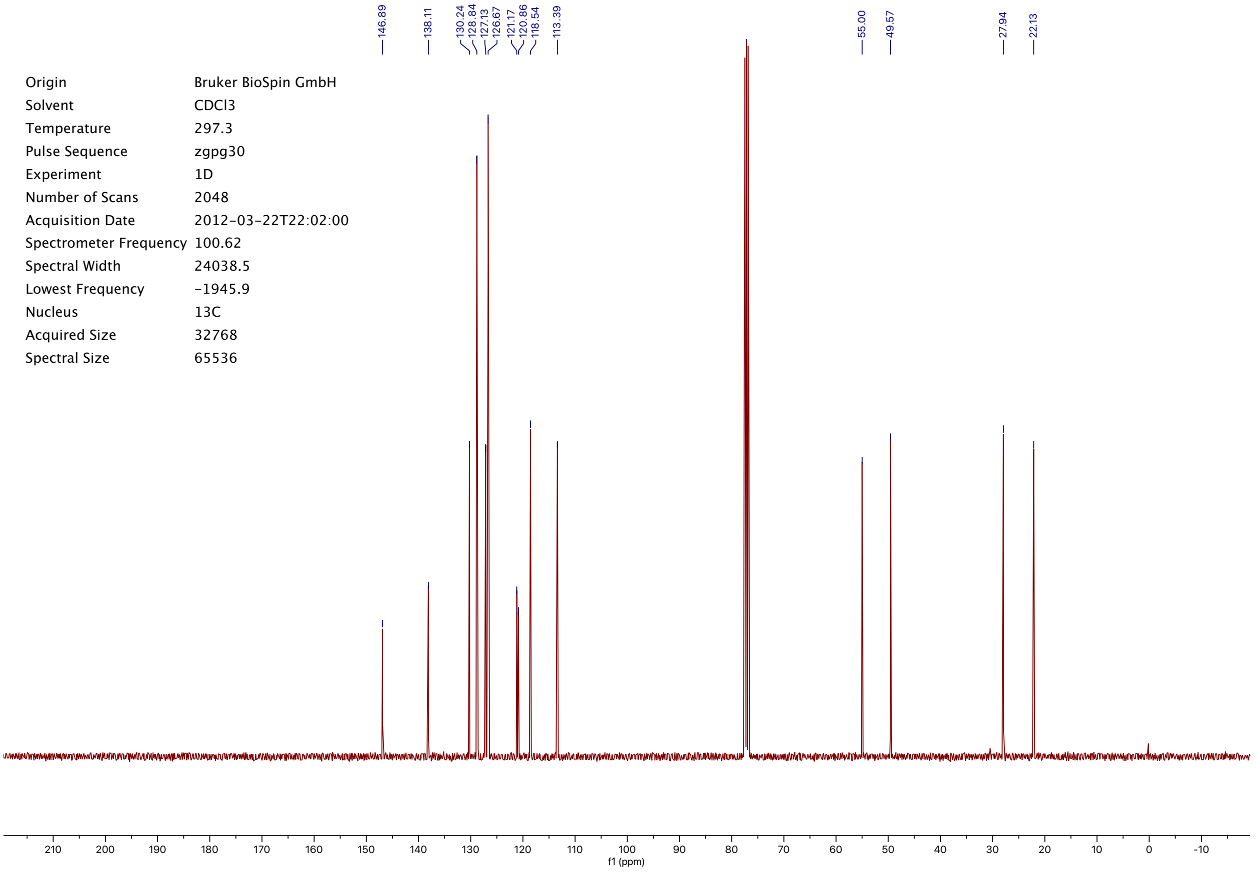

Origin	Bruker BioSpin GmbH
Solvent	CDCl3
Temperature	295.7
Pulse Sequence	zg30
Experiment	1D
Number of Scans	16
Acquisition Date	2021-10-12T01:40:00
Spectrometer Frequency	400.13
Spectral Width	8012.8
Lowest Frequency	-1556.1
Nucleus	1H
Acquired Size	32768
Spectral Size	65536

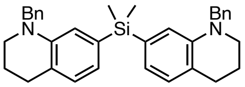

27

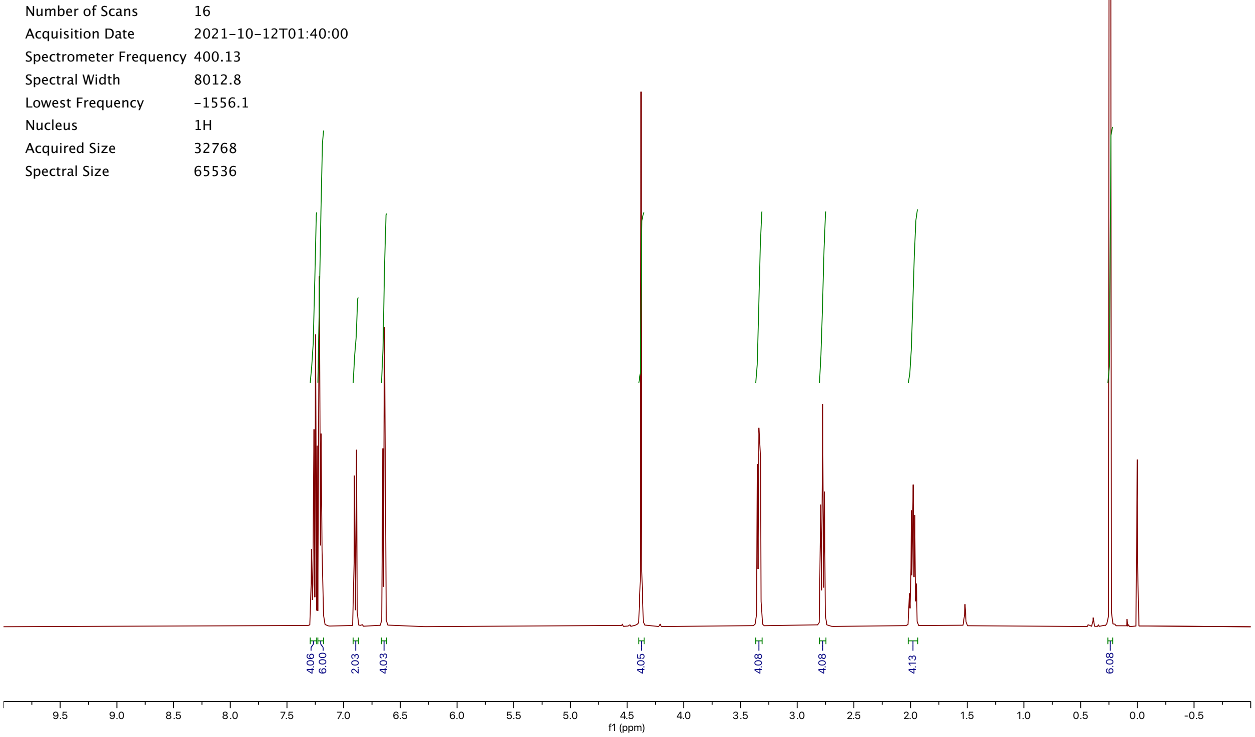

Origin	Bruker BioSpin GmbH
Solvent	CDCl3
Temperature	296.3
Pulse Sequence	zgpg30
Experiment	1D
Number of Scans	1024
Acquisition Date	2021-10-12T02:41:00
Spectrometer Frequency	100.62
Spectral Width	24038.5
Lowest Frequency	-1948.1
Nucleus	13C
Acquired Size	32768
Spectral Size	65536

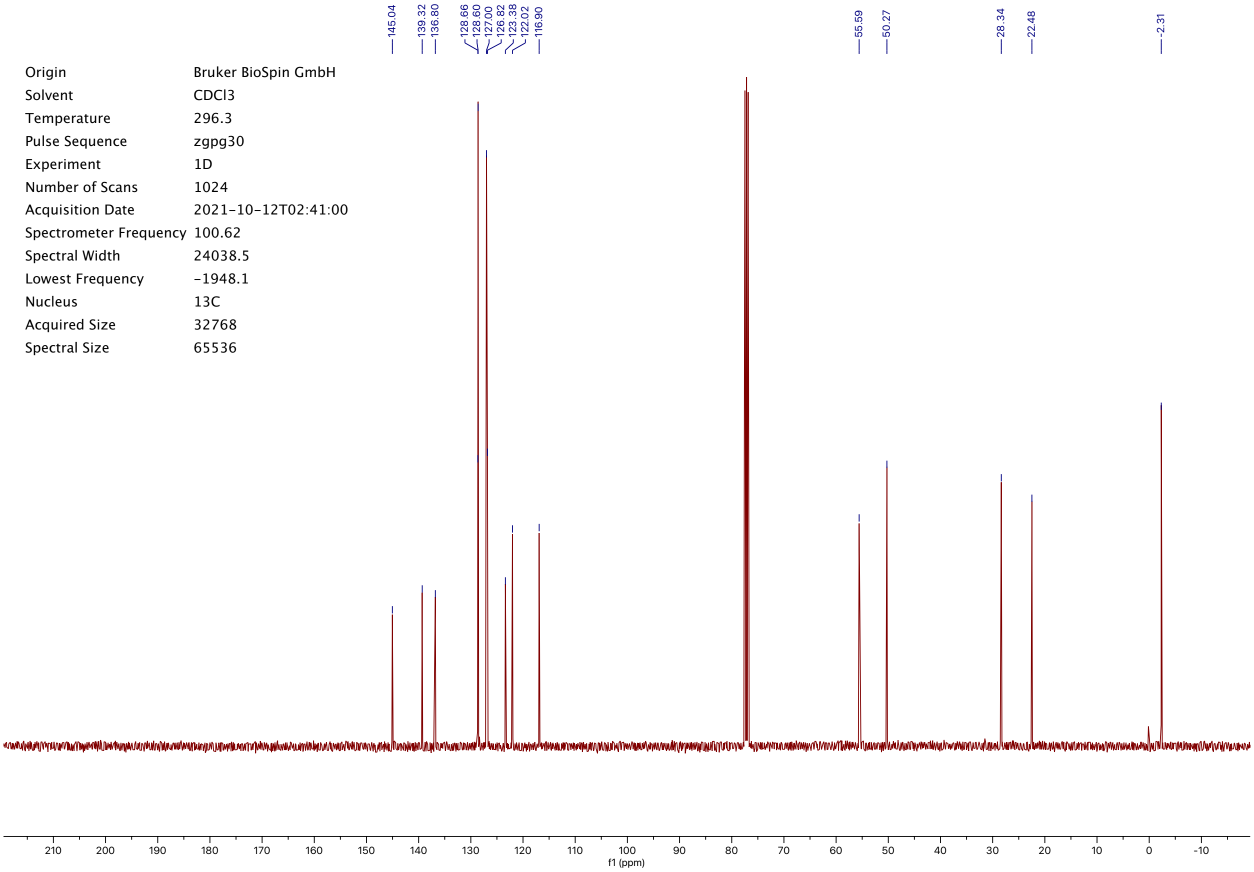

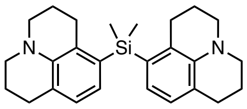

30

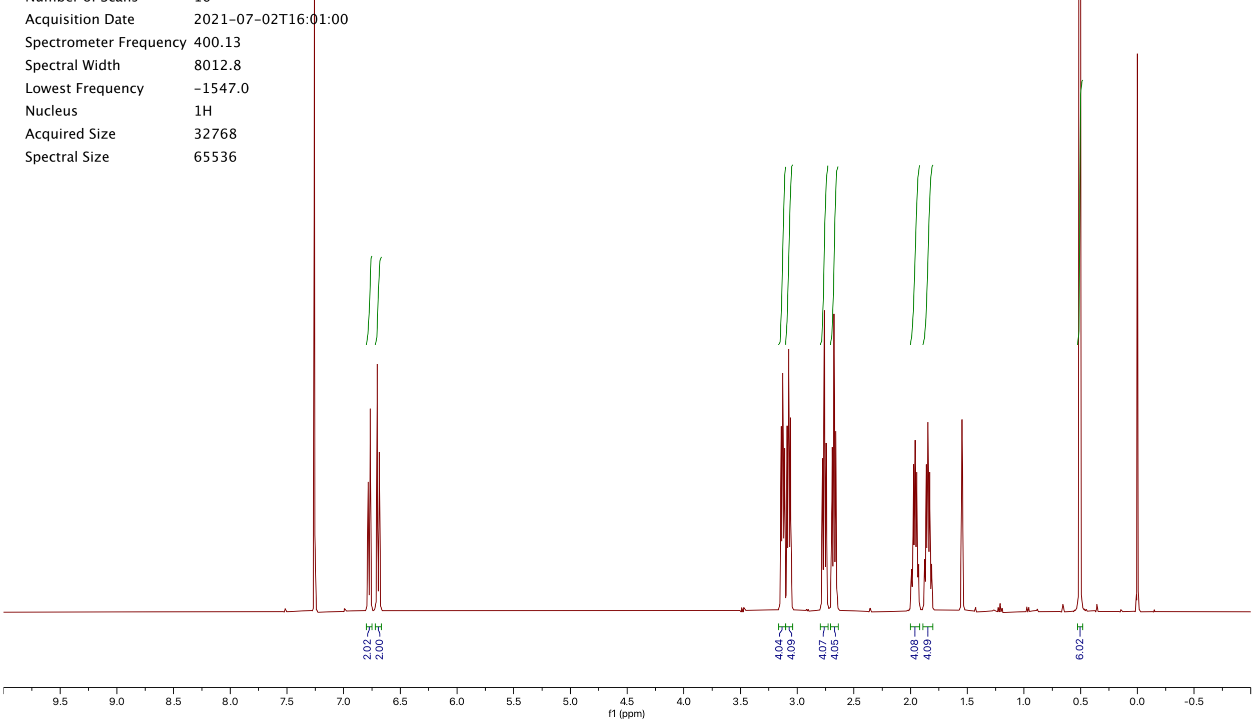

Origin Bruker BioSpin GmbH
Solvent CDCl3
Temperature 295.3
Pulse Sequence zg30
Experiment 1D
Number of Scans 16
Acquisition Date 2021-07-02T16:01:00
Spectrometer Frequency 400.13
Spectral Width 8012.8
Lowest Frequency -1547.0
Nucleus 1H
Acquired Size 32768
Spectral Size 65536
2.02
2.00
4.04
4.09
4.07
4.05
4.08
4.09
6.02
9.5 9.0 8.5 8.0 7.5 7.0 6.5 6.0 5.5 5.0 4.5 4.0 3.5 3.0 2.5 2.0 1.5 1.0 0.5 0.0 -0.5
f1 (ppm)

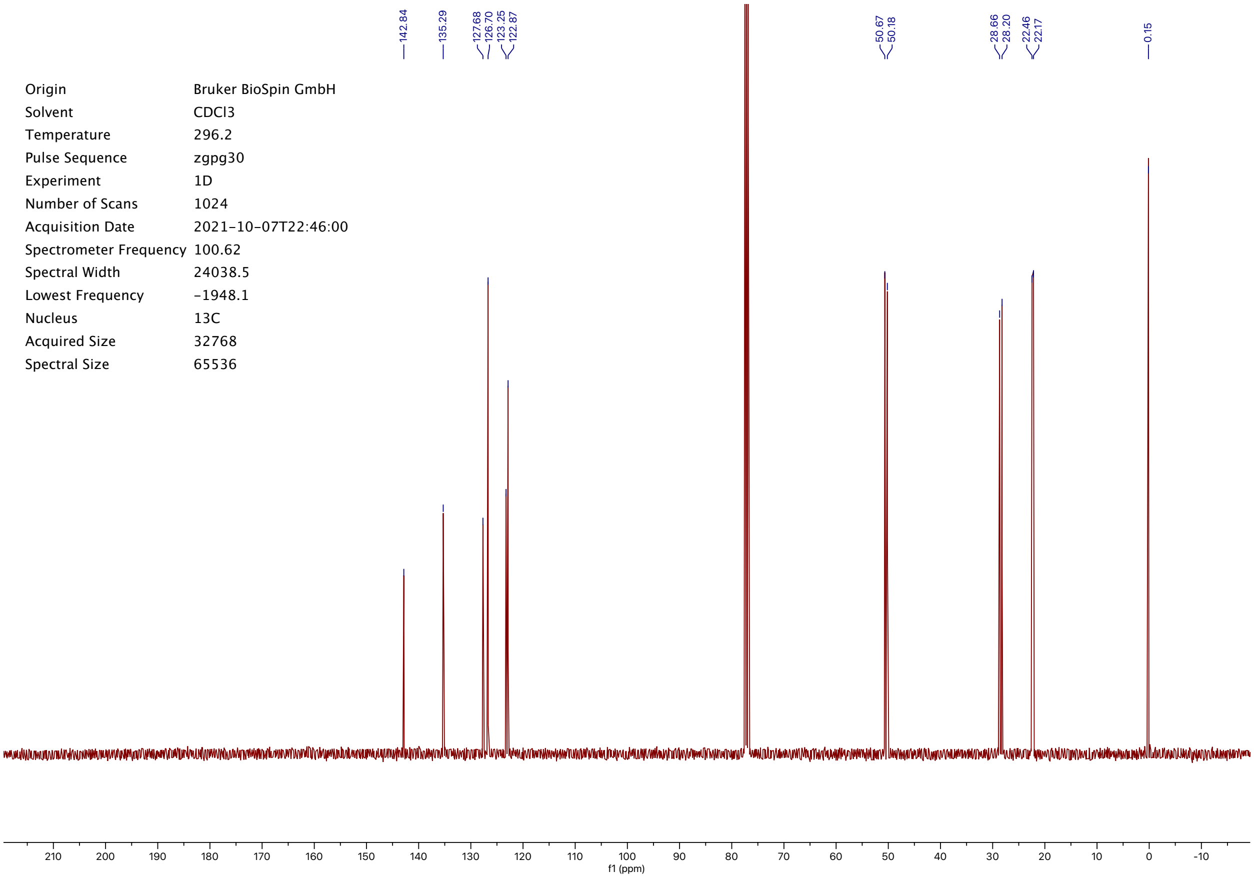

142.84
135.29
127.68
126.70
123.25
122.87
50.67
50.18
28.66
28.20
22.46
22.17
0.15
Origin Bruker BioSpin GmbH
Solvent CDCl3
Temperature 296.2
Pulse Sequence zgpg30
Experiment 1D
Number of Scans 1024
Acquisition Date 2021-10-07T22:46:00
Spectrometer Frequency 100.62
Spectral Width 24038.5
Lowest Frequency -1948.1
Nucleus 13C
Acquired Size 32768
Spectral Size 65536
210 200 190 180 170 160 150 140 130 120 110 100 90 80 70 60 50 40 30 20 10 0 -10
f1 (ppm)

Origin	Bruker BioSpin GmbH
Solvent	DMSO
Temperature	350.2
Pulse Sequence	zg30
Experiment	1D
Number of Scans	16
Acquisition Date	2021-10-28T16:43:00
Spectrometer Frequency	400.13
Spectral Width	8012.8
Lowest Frequency	-1538.4
Nucleus	1H
Acquired Size	32768
Spectral Size	65536

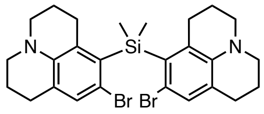

S6

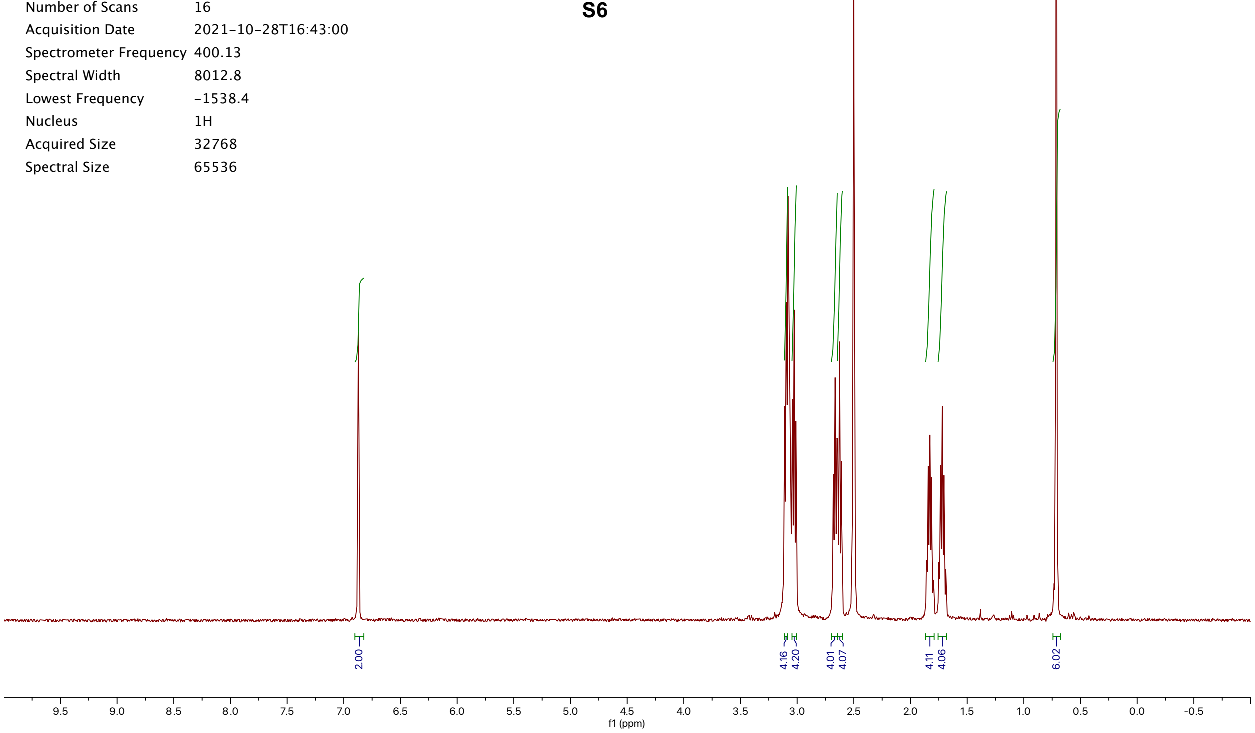

Origin	Bruker BioSpin GmbH
Solvent	DMSO
Temperature	350.0
Pulse Sequence	zgpg30
Experiment	1D
Number of Scans	2048
Acquisition Date	2021-10-29T13:50:00
Spectrometer Frequency	100.62
Spectral Width	24038.5
Lowest Frequency	-2047.0
Nucleus	13C
Acquired Size	32768
Spectral Size	65536

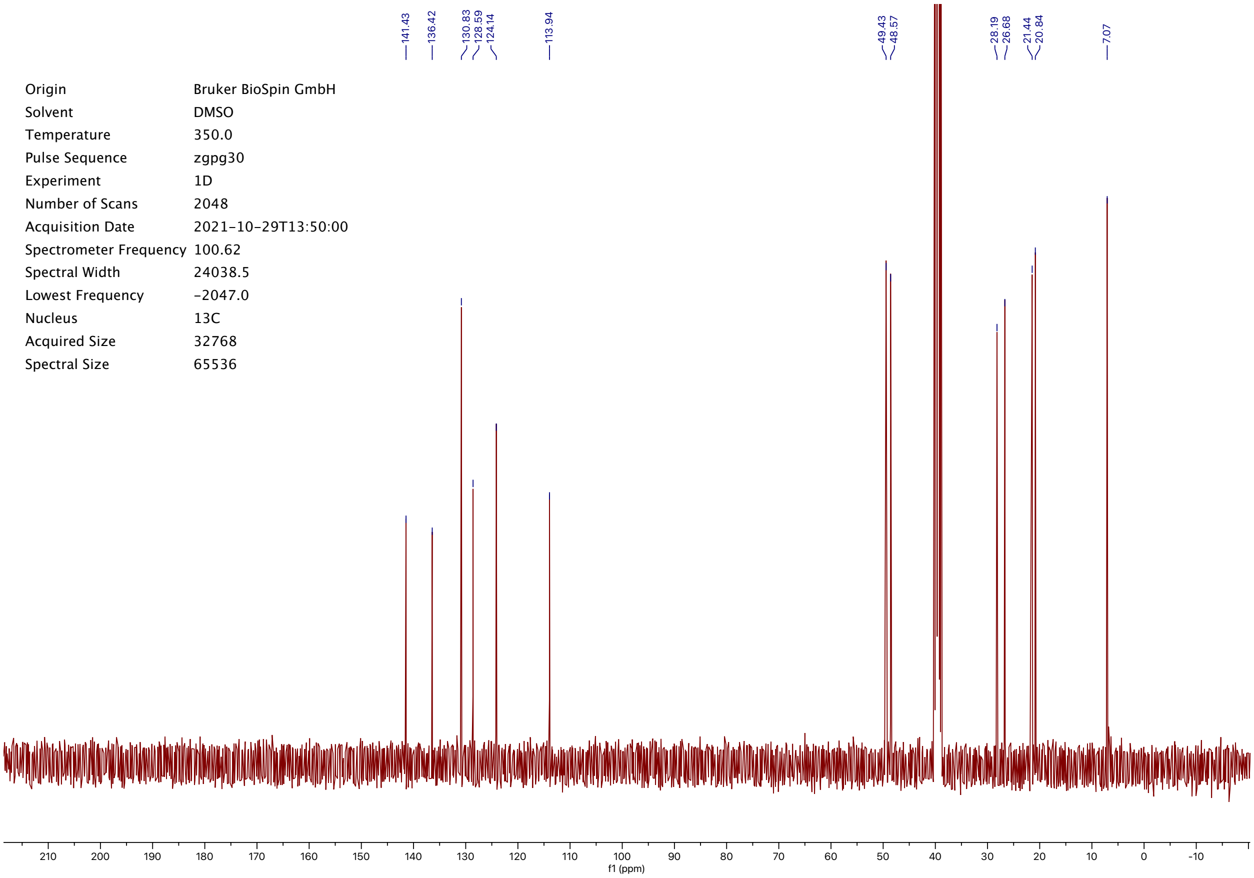

Origin	Bruker BioSpin GmbH
Solvent	CDCl3
Temperature	295.3
Pulse Sequence	zg30
Experiment	1D
Number of Scans	16
Acquisition Date	2021-09-09T11:44:00
Spectrometer Frequency	400.13
Spectral Width	8012.8
Lowest Frequency	-1548.7
Nucleus	1H
Acquired Size	32768
Spectral Size	65536

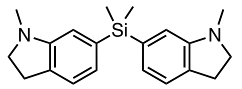

S8

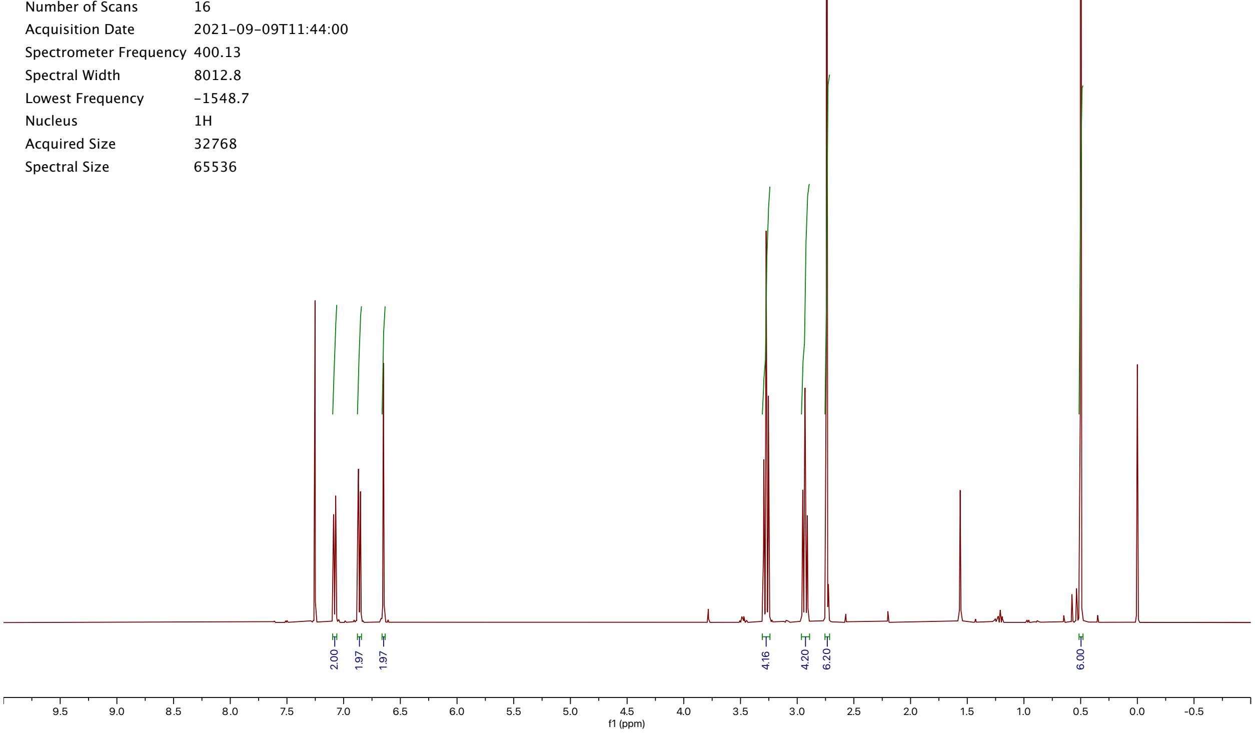

Origin	Bruker BioSpin GmbH
Solvent	CDCl3
Temperature	296.2
Pulse Sequence	zgpg30
Experiment	1D
Number of Scans	1024
Acquisition Date	2021-10-08T02:27:00
Spectrometer Frequency	100.62
Spectral Width	24038.5
Lowest Frequency	-1948.4
Nucleus	13C
Acquired Size	32768
Spectral Size	65536

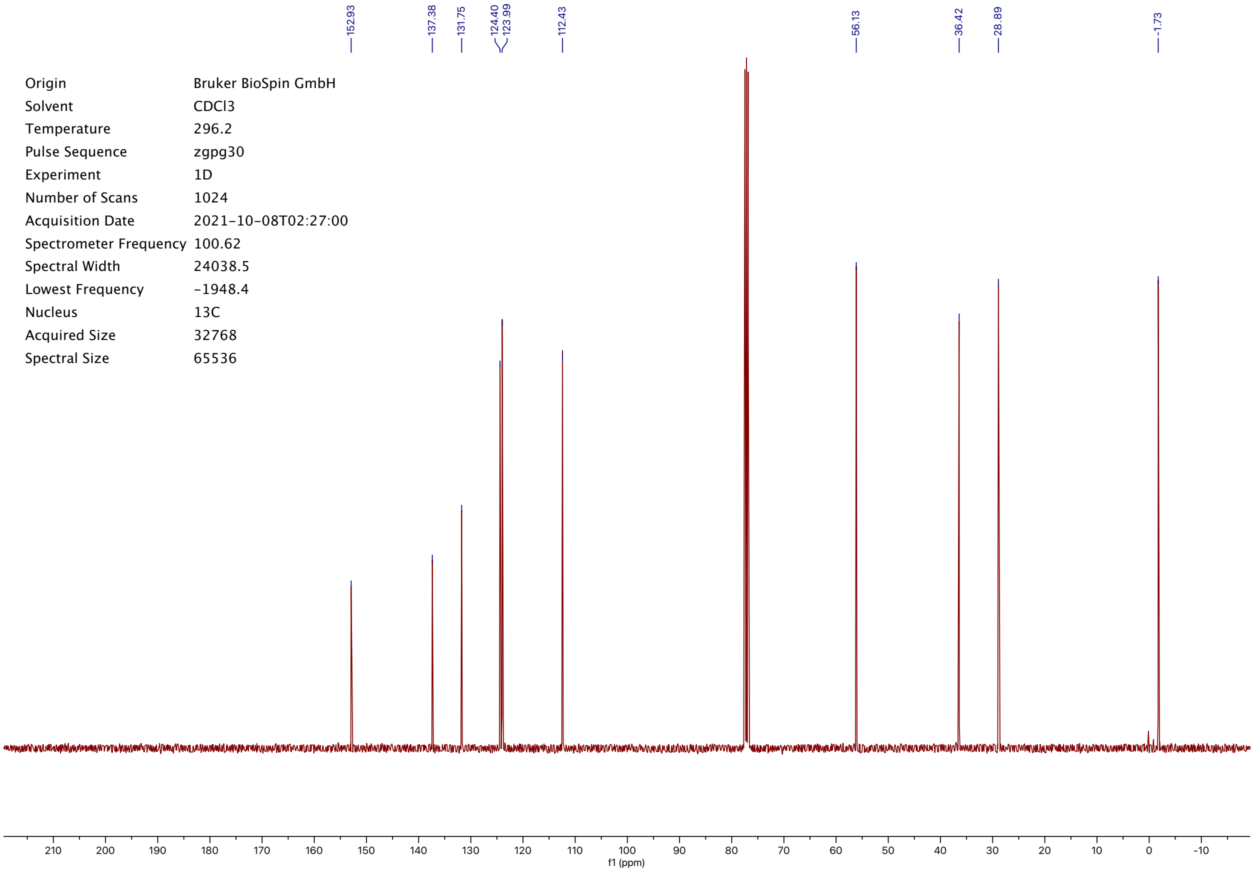

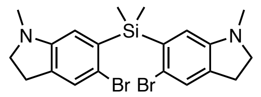
N
Si
N
Br Br

S9

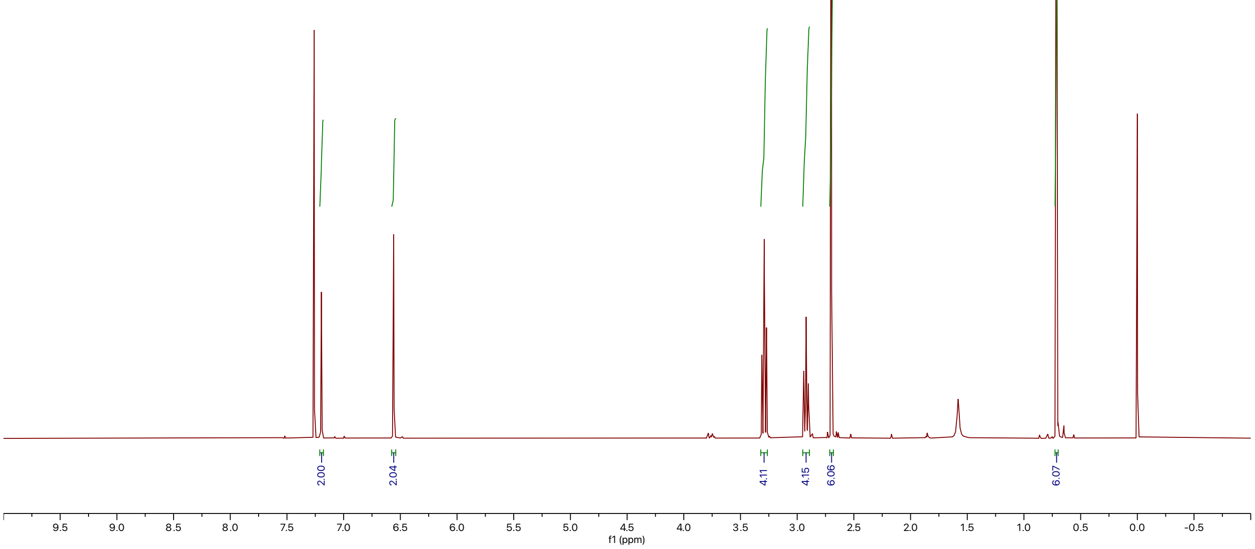
Origin Bruker BioSpin GmbH
Solvent CDCl3
Temperature 295.3
Pulse Sequence zg30
Experiment 1D
Number of Scans 16
Acquisition Date 2021-09-14T11:02:00
Spectrometer Frequency 400.13
Spectral Width 8012.8
Lowest Frequency -1545.7
Nucleus 1H
Acquired Size 32768
Spectral Size 65536
2.00
2.04
4.11
4.15
6.06
6.07
f1 (ppm)

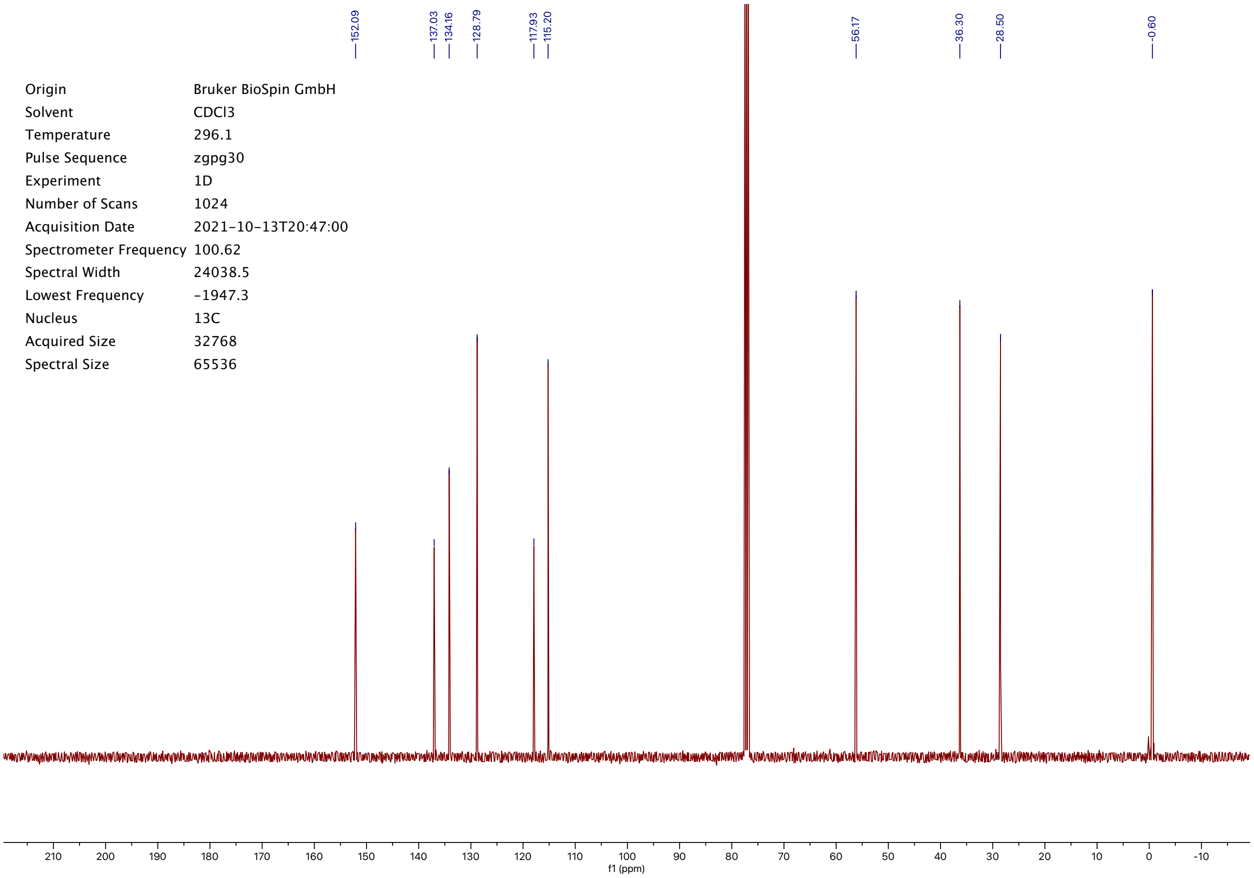
152.09
137.03
134.16
128.79
117.93
115.20
56.17
36.30
28.50
-0.60
Origin Bruker BioSpin GmbH
Solvent CDCl3
Temperature 296.1
Pulse Sequence zgpg30
Experiment 1D
Number of Scans 1024
Acquisition Date 2021-10-13T20:47:00
Spectrometer Frequency 100.62
Spectral Width 24038.5
Lowest Frequency -1947.3
Nucleus 13C
Acquired Size 32768
Spectral Size 65536
f1 (ppm)

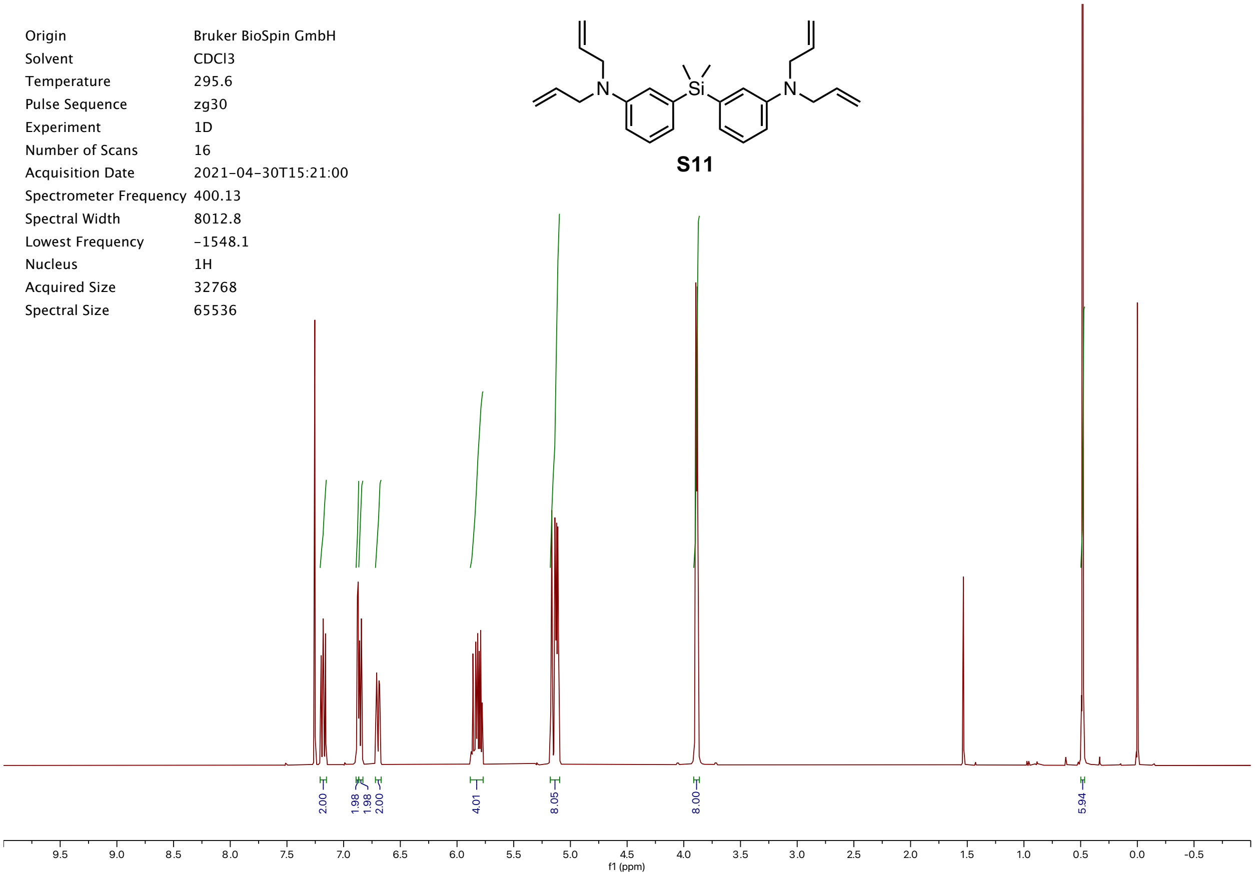

S11
Origin Bruker BioSpin GmbH
Solvent CDCl3
Temperature 295.6
Pulse Sequence zg30
Experiment 1D
Number of Scans 16
Acquisition Date 2021-04-30T15:21:00
Spectrometer Frequency 400.13
Spectral Width 8012.8
Lowest Frequency -1548.1
Nucleus 1H
Acquired Size 32768
Spectral Size 65536
2.00
1.98
1.98
2.00
4.01
8.05
8.00
5.94
f1 (ppm)

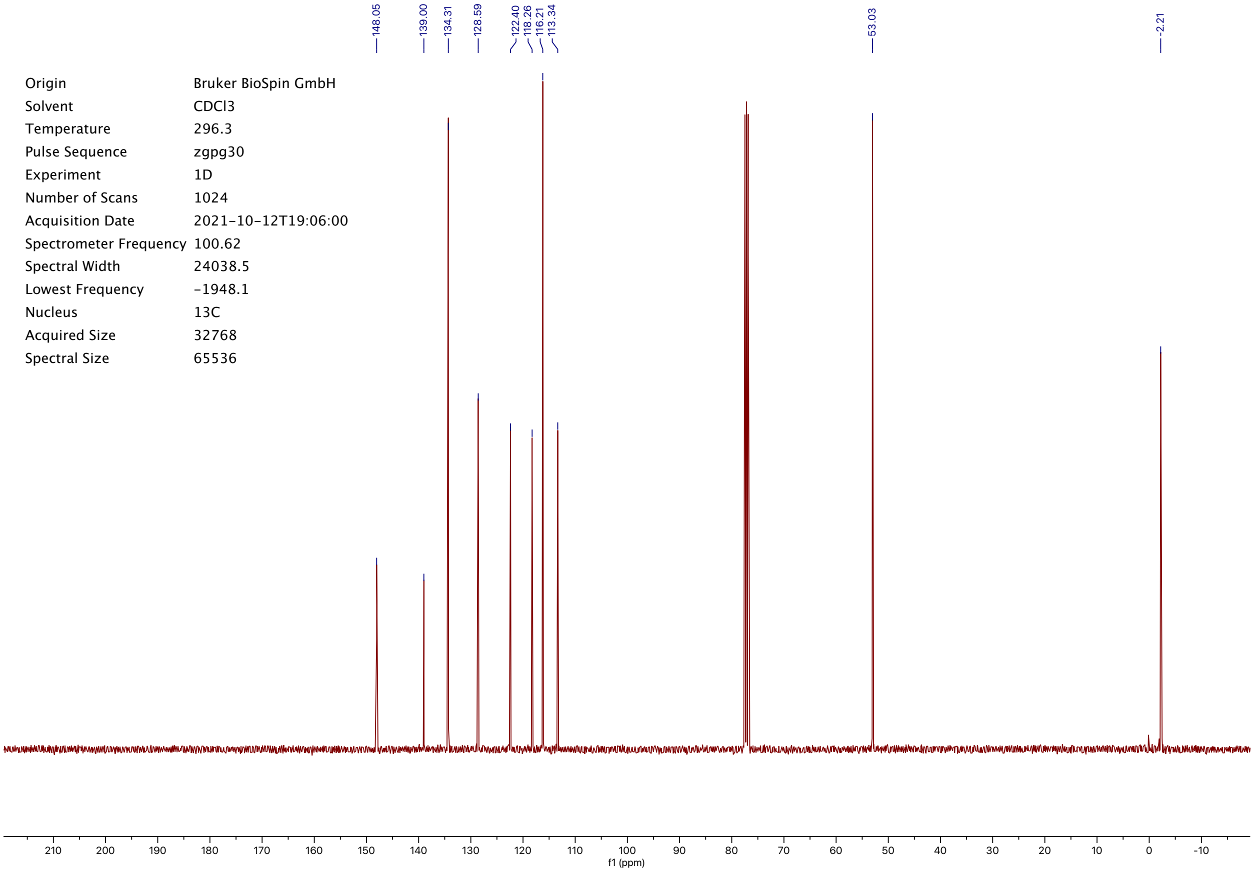

148.05
139.00
134.31
128.59
122.40
118.26
116.21
113.34
53.03
-2.21
Origin Bruker BioSpin GmbH
Solvent CDCl3
Temperature 296.3
Pulse Sequence zgpg30
Experiment 1D
Number of Scans 1024
Acquisition Date 2021-10-12T19:06:00
Spectrometer Frequency 100.62
Spectral Width 24038.5
Lowest Frequency -1948.1
Nucleus 13C
Acquired Size 32768
Spectral Size 65536
f1 (ppm)

Origin	Bruker BioSpin GmbH
Solvent	CDCl3
Temperature	295.6
Pulse Sequence	zg30
Experiment	1D
Number of Scans	16
Acquisition Date	2021-10-07T18:05:00
Spectrometer Frequency	400.13
Spectral Width	8012.8
Lowest Frequency	-1554.4
Nucleus	1H
Acquired Size	32768
Spectral Size	65536

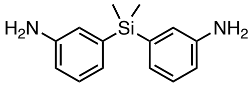

S12

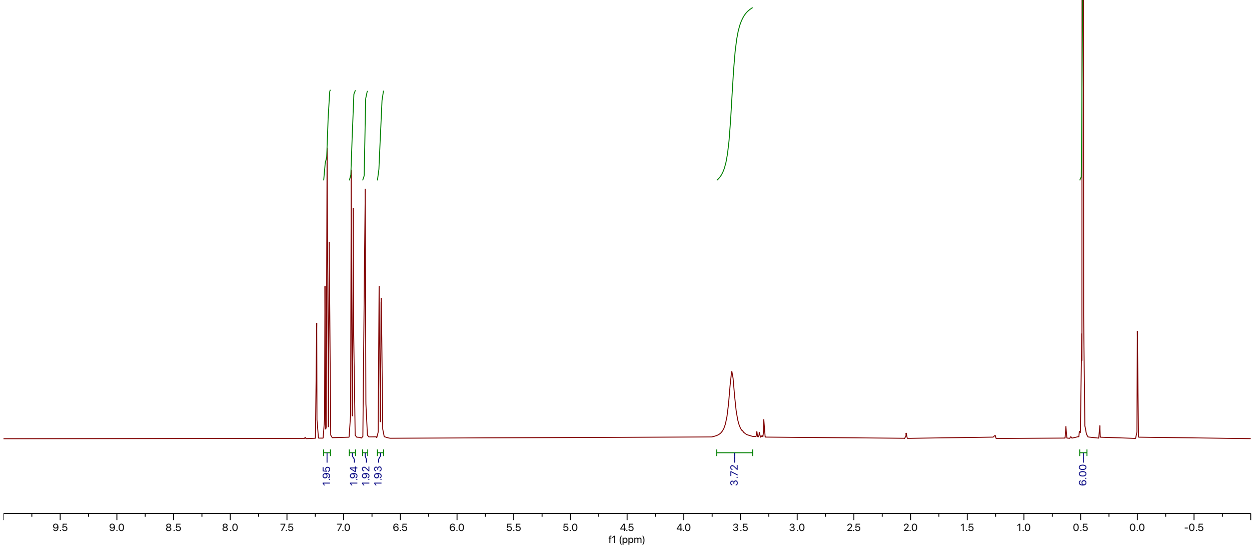

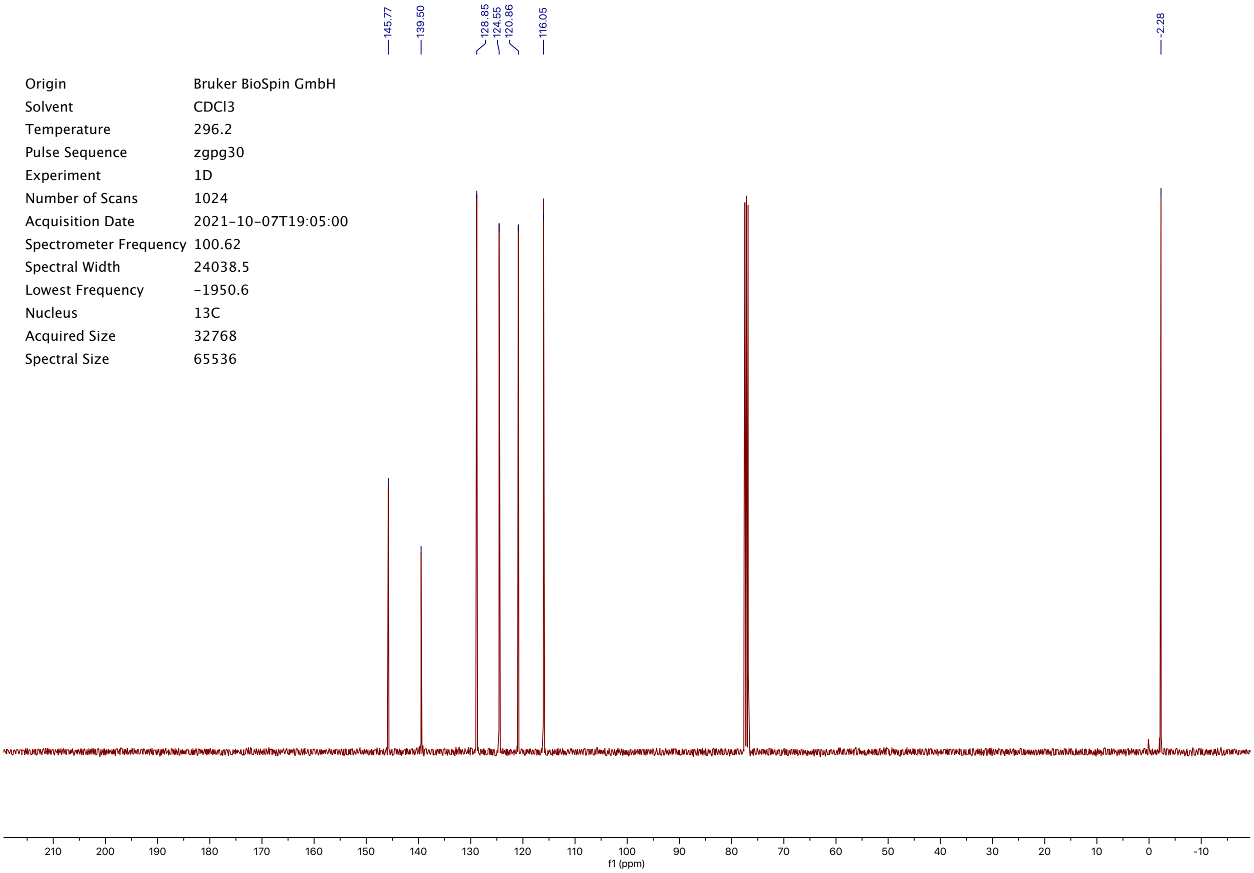

Origin	Bruker BioSpin GmbH
Solvent	CDCl3
Temperature	296.2
Pulse Sequence	zgpg30
Experiment	1D
Number of Scans	1024
Acquisition Date	2021-10-07T19:05:00
Spectrometer Frequency	100.62
Spectral Width	24038.5
Lowest Frequency	-1950.6
Nucleus	13C
Acquired Size	32768
Spectral Size	65536

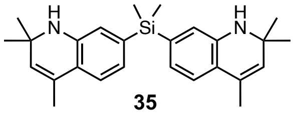

35

Origin	Bruker BioSpin GmbH
Solvent	CDCl3
Temperature	295.6
Pulse Sequence	zg30
Experiment	1D
Number of Scans	16
Acquisition Date	2021-10-11T22:00:00
Spectrometer Frequency	400.13
Spectral Width	8012.8
Lowest Frequency	-1551.9
Nucleus	1H
Acquired Size	32768
Spectral Size	65536

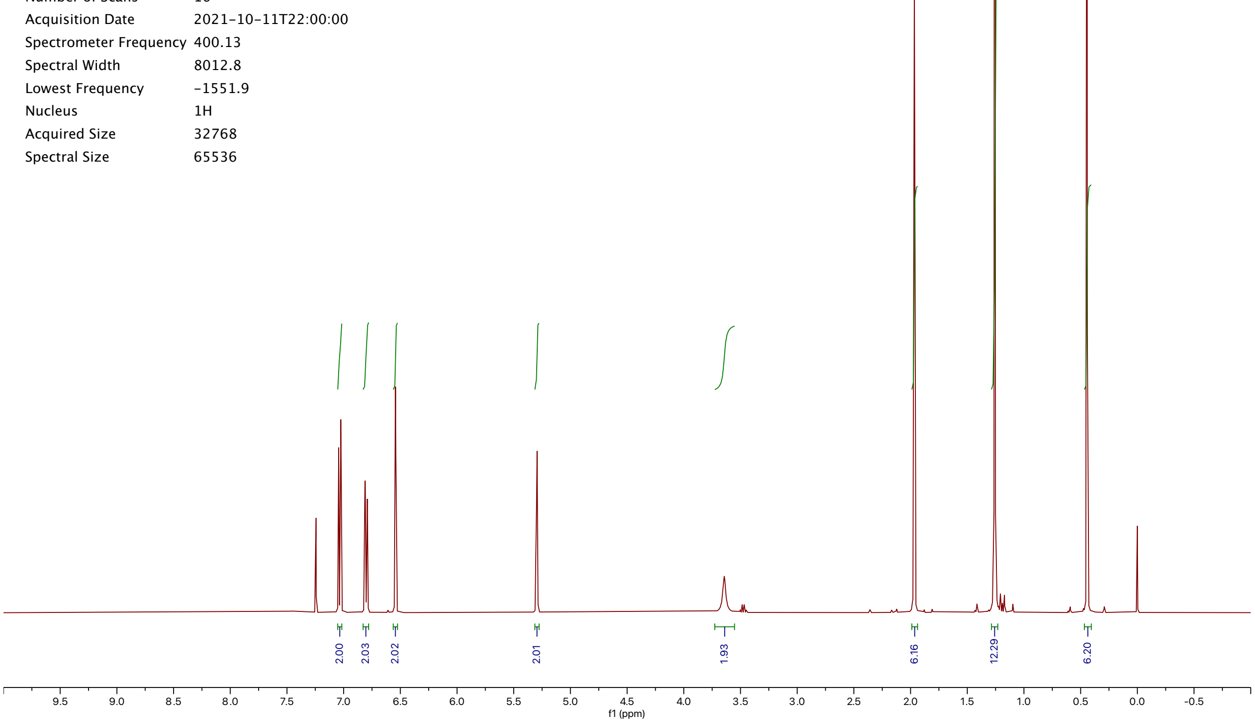

Origin	Bruker BioSpin GmbH
Solvent	CDCl3
Temperature	296.1
Pulse Sequence	zgpg30
Experiment	1D
Number of Scans	1024
Acquisition Date	2021-10-11T23:00:00
Spectrometer Frequency	100.62
Spectral Width	24038.5
Lowest Frequency	-1948.1
Nucleus	13C
Acquired Size	32768
Spectral Size	65536

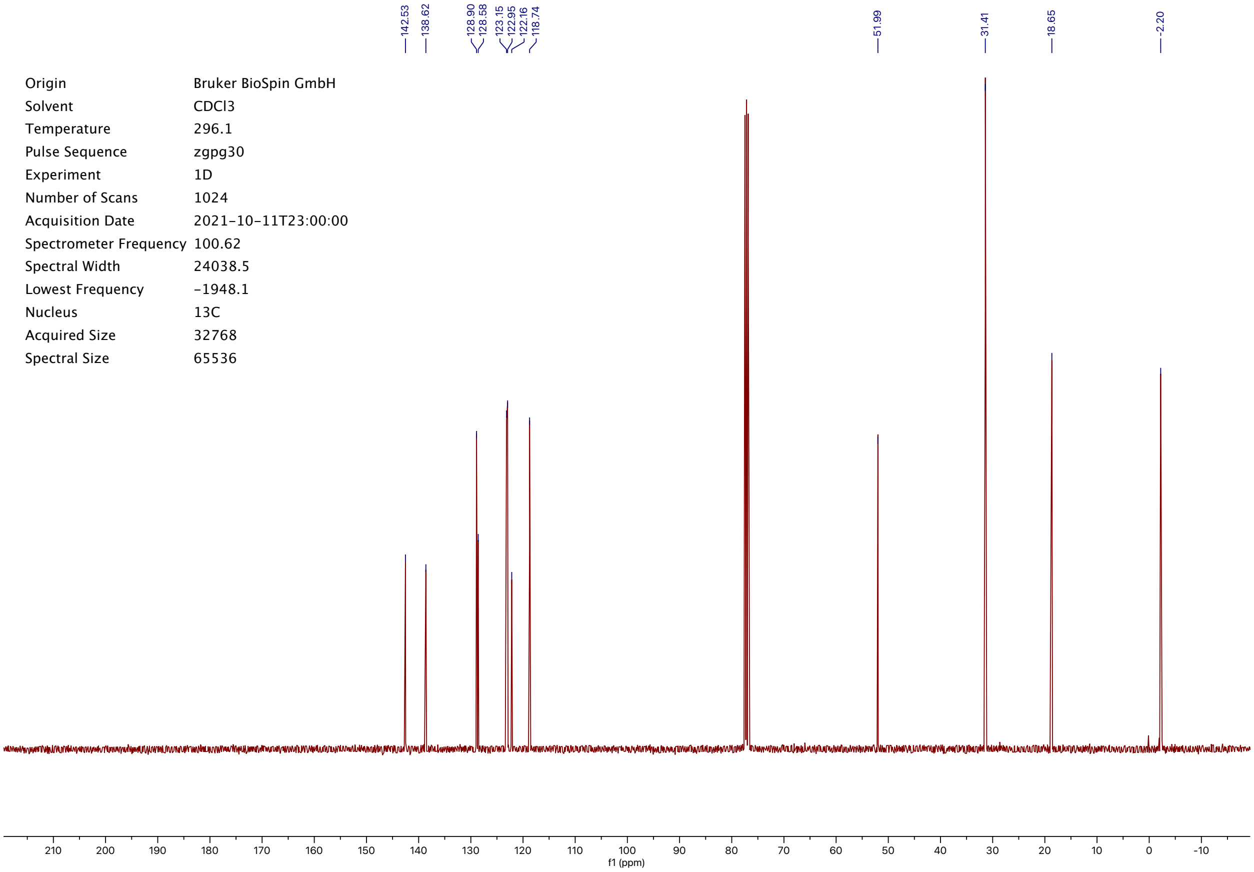

Origin	Bruker BioSpin GmbH
Solvent	CDCl3
Temperature	295.6
Pulse Sequence	zg30
Experiment	1D
Number of Scans	16
Acquisition Date	2021-10-13T21:29:00
Spectrometer Frequency	400.13
Spectral Width	8012.8
Lowest Frequency	-1551.9
Nucleus	1H
Acquired Size	32768
Spectral Size	65536

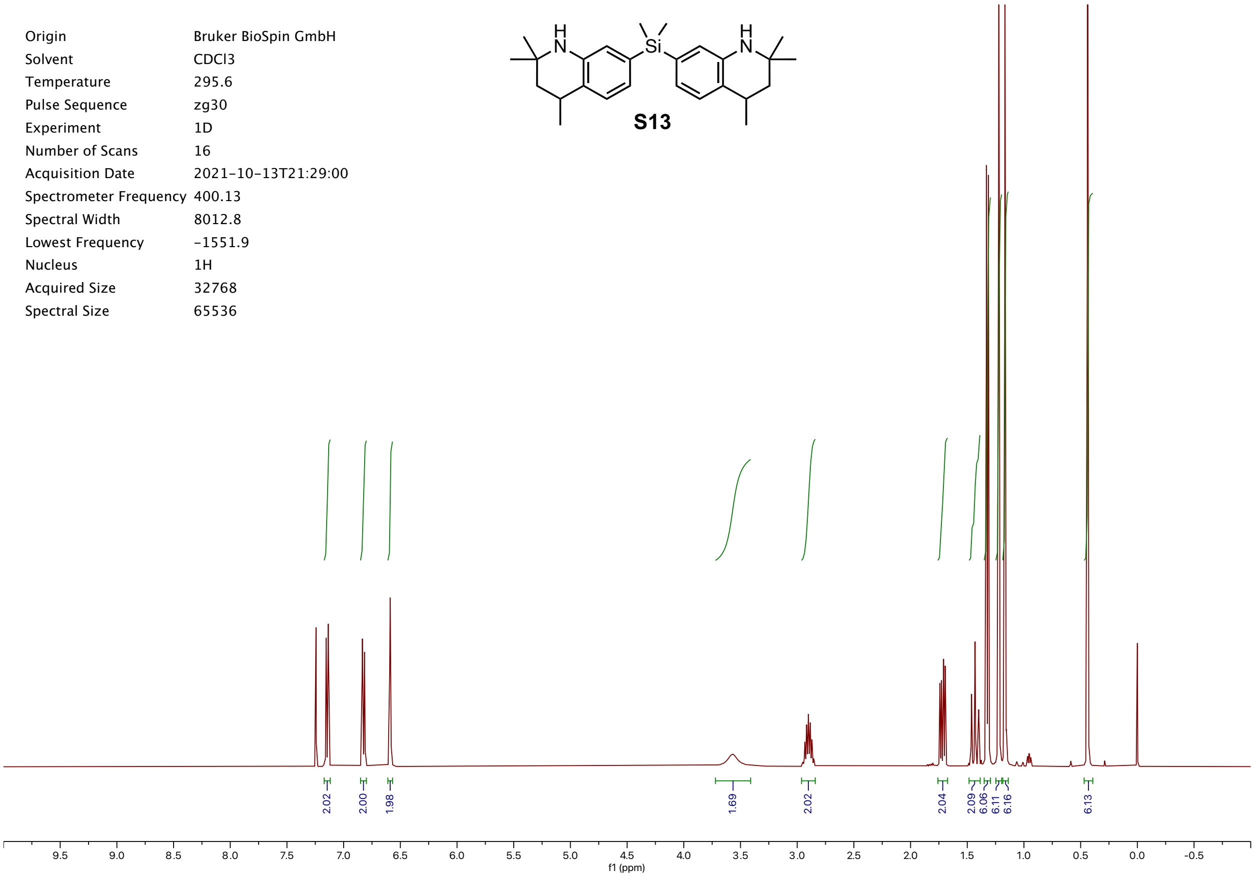

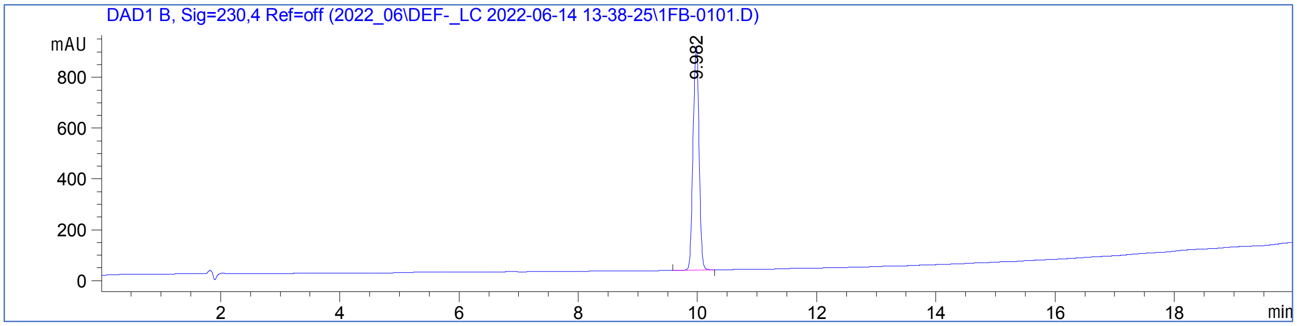

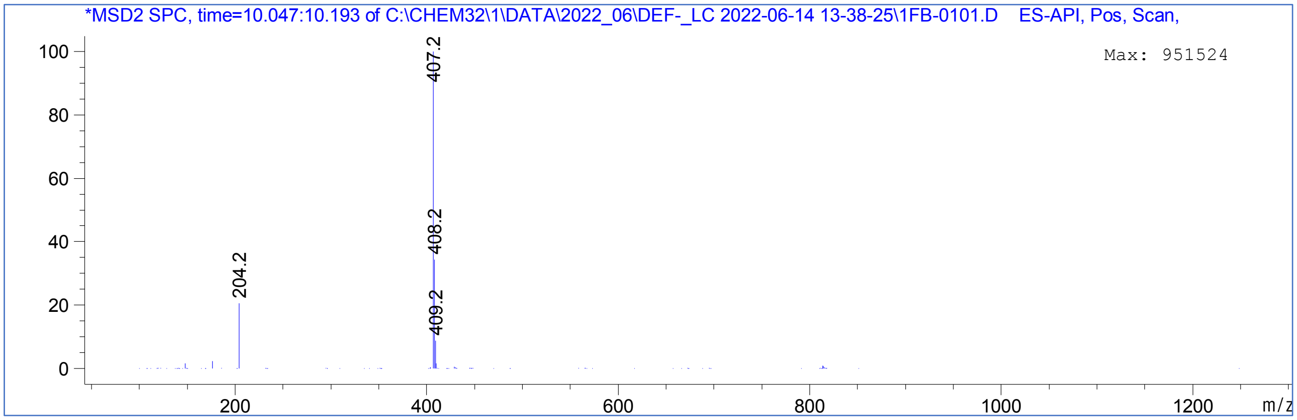

Origin	Bruker BioSpin GmbH
Solvent	CDCl3
Temperature	299.0
Pulse Sequence	zg30
Experiment	1D
Number of Scans	16
Acquisition Date	2022-11-15T12:03:00
Spectrometer Frequency	400.13
Spectral Width	8012.8
Lowest Frequency	-1551.0
Nucleus	1H
Acquired Size	32768
Spectral Size	65536

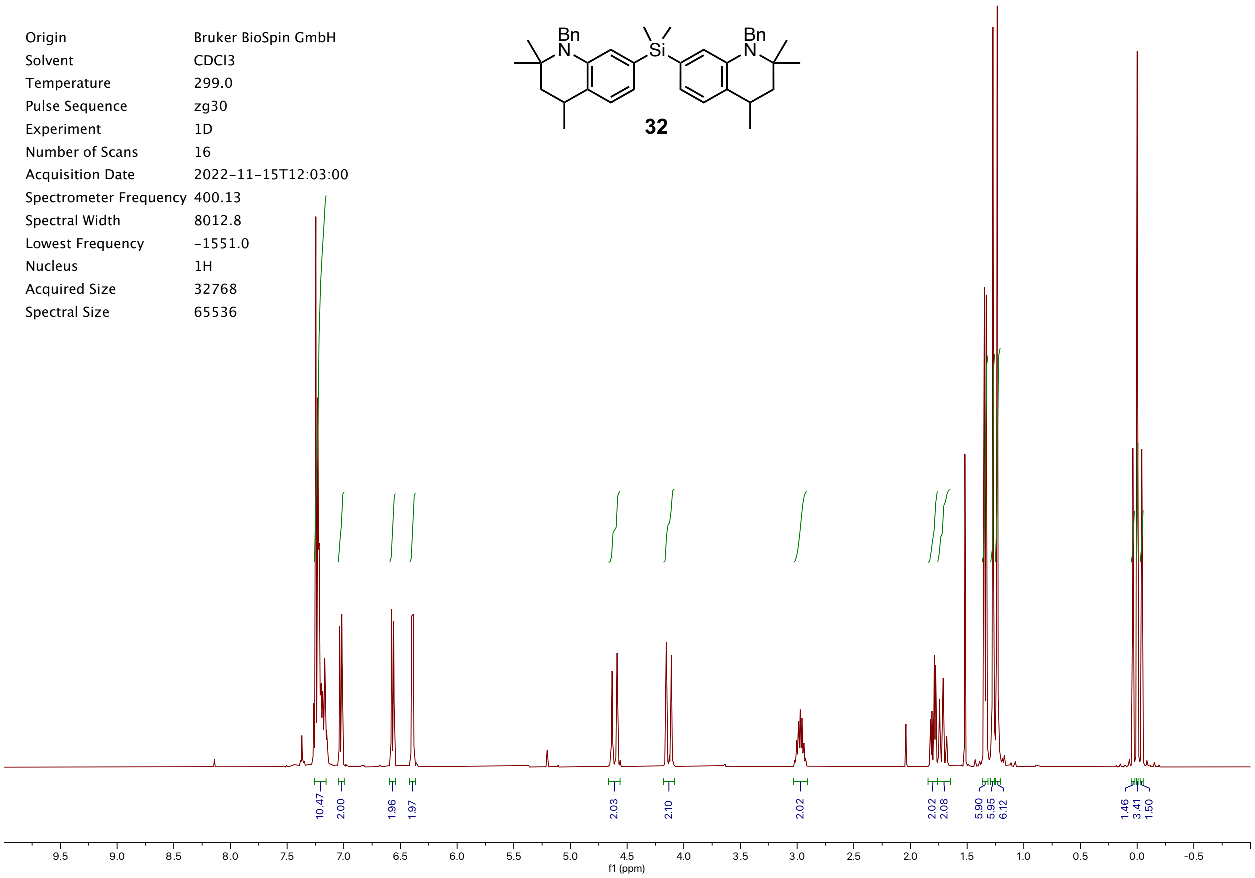

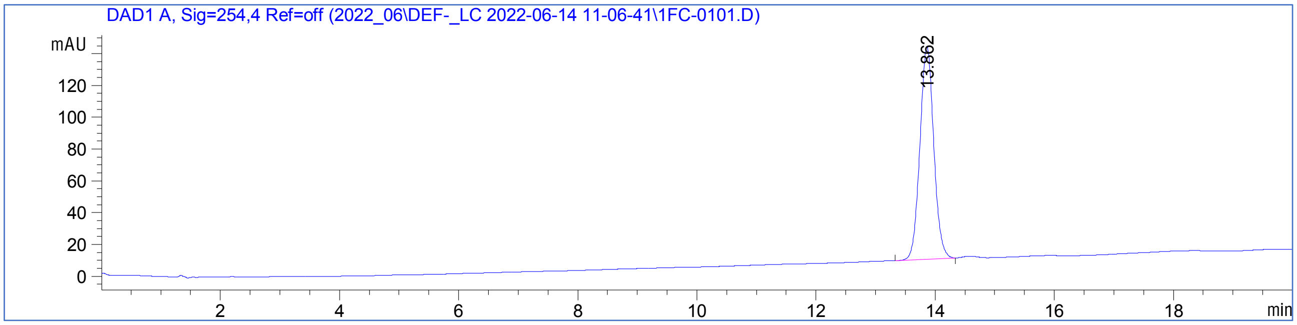

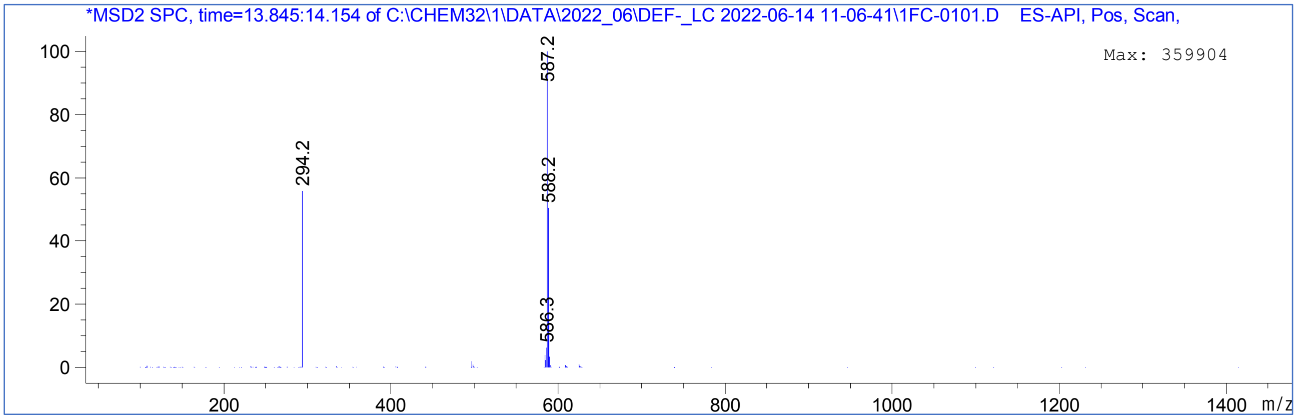

Origin	Bruker BioSpin GmbH
Solvent	CDCl3
Temperature	302.2
Pulse Sequence	zg30
Experiment	1D
Number of Scans	16
Acquisition Date	2020-11-11T11:48:00
Spectrometer Frequency	400.13
Spectral Width	8012.8
Lowest Frequency	-1556.7
Nucleus	1H
Acquired Size	32768
Spectral Size	65536

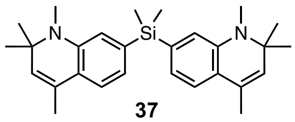

37

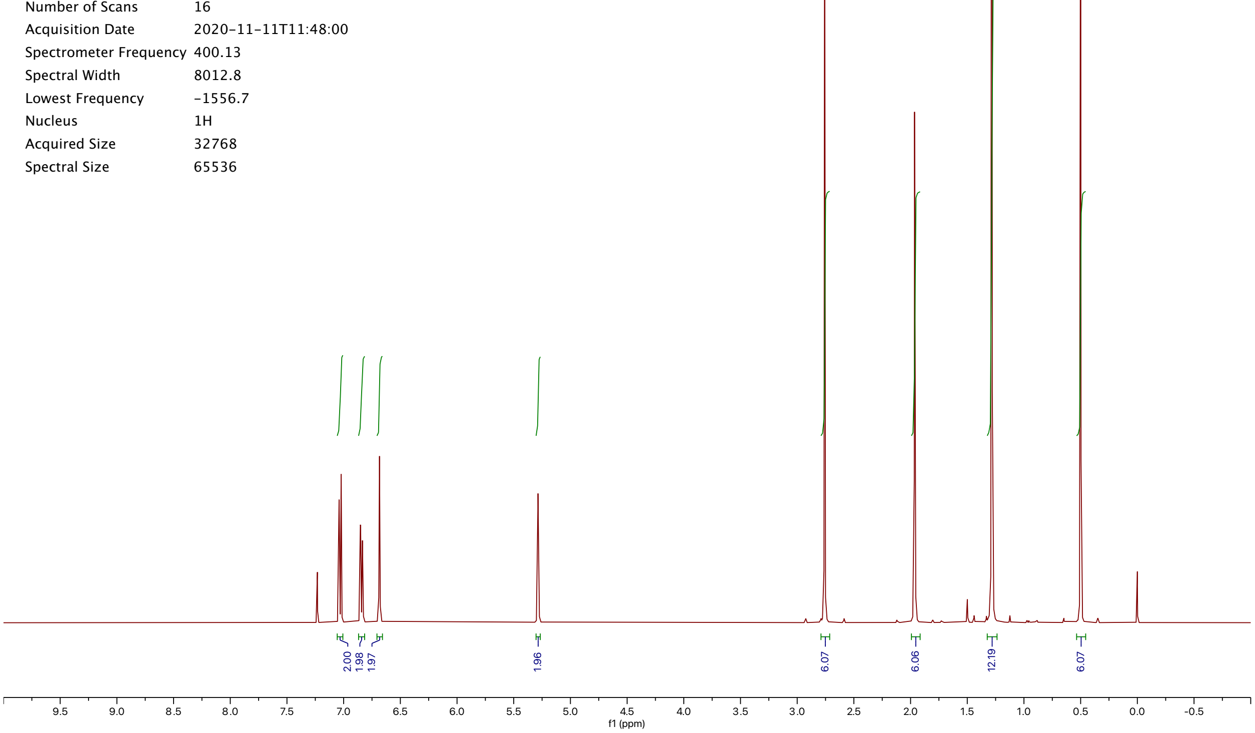

Origin	Bruker BioSpin GmbH
Solvent	CDCl3
Temperature	300.0
Pulse Sequence	zgpg30
Experiment	1D
Number of Scans	1024
Acquisition Date	2020-11-11T12:49:00
Spectrometer Frequency	100.62
Spectral Width	24038.5
Lowest Frequency	-1947.4
Nucleus	13C
Acquired Size	32768
Spectral Size	65536

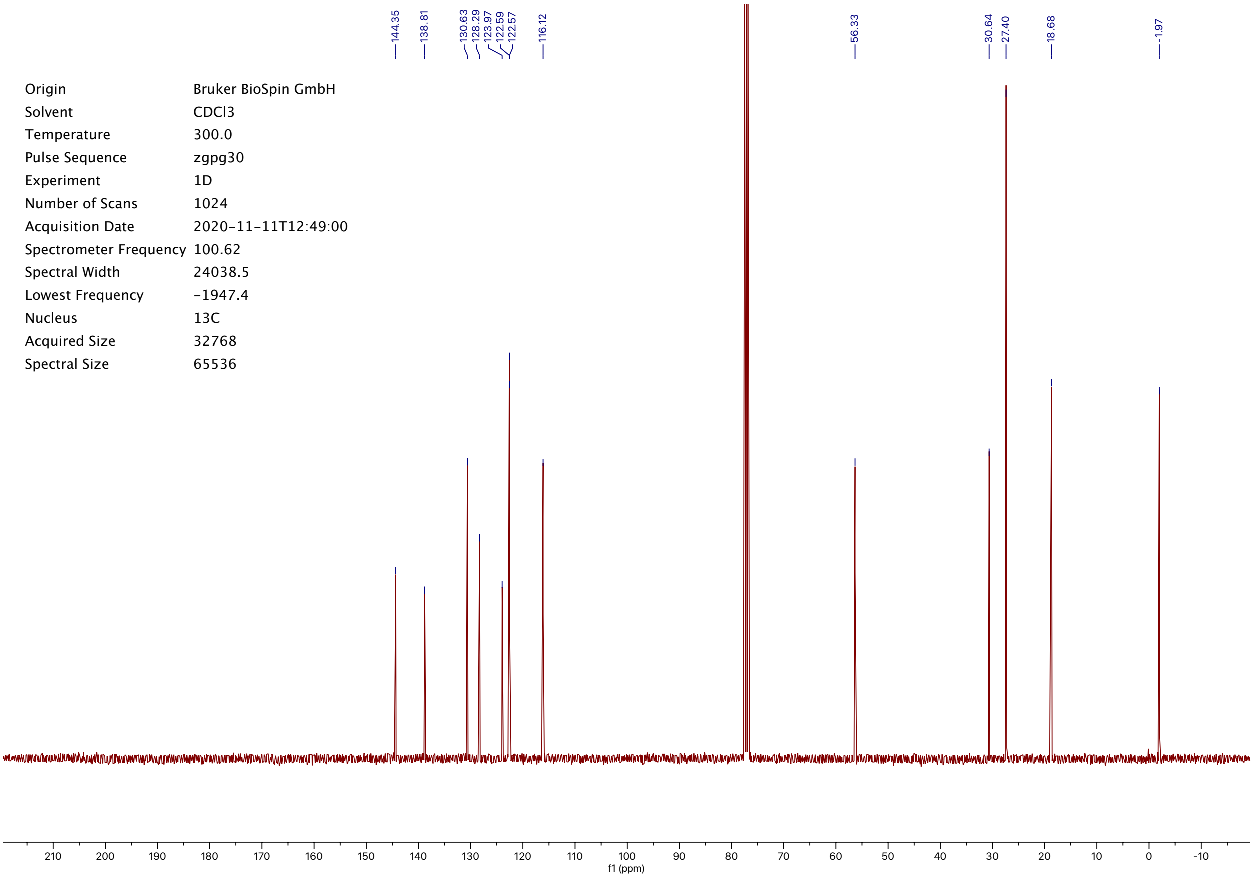

Origin	Bruker BioSpin GmbH
Solvent	DMSO
Temperature	295.4
Pulse Sequence	zg30
Experiment	1D
Number of Scans	16
Acquisition Date	2020-02-10T16:05:00
Spectrometer Frequency	400.13
Spectral Width	8012.8
Lowest Frequency	-1539.2
Nucleus	1H
Acquired Size	32768
Spectral Size	65536

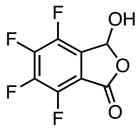

5

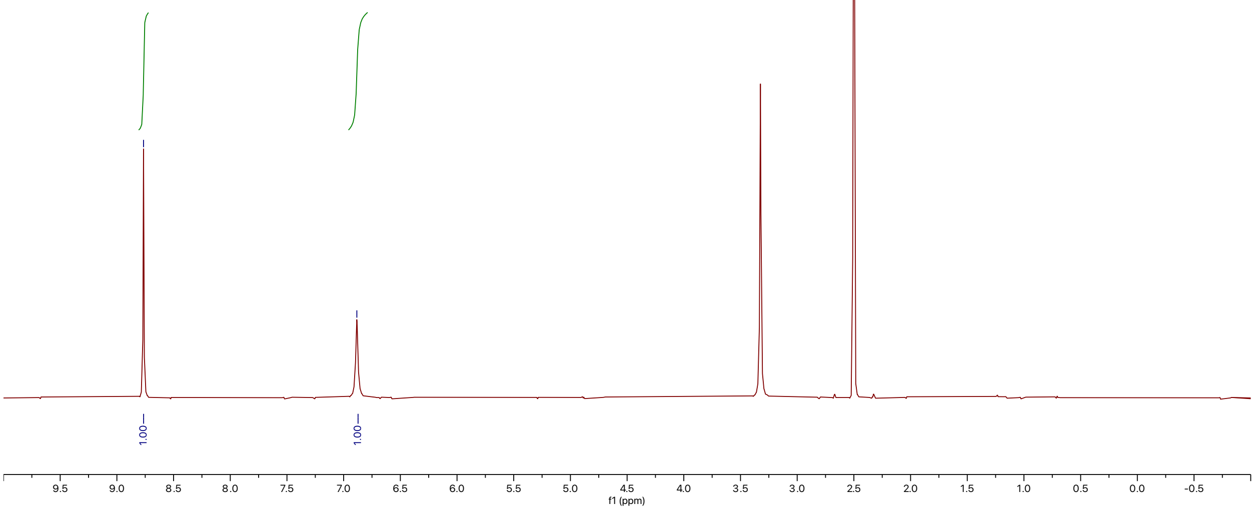

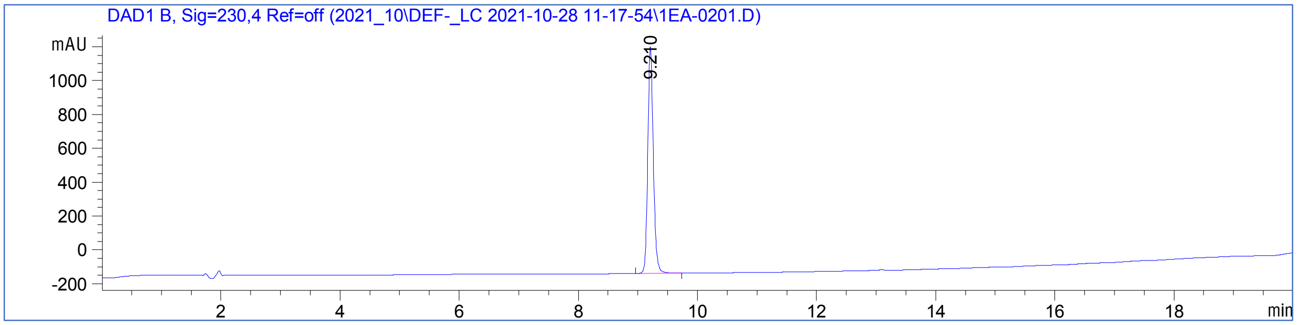

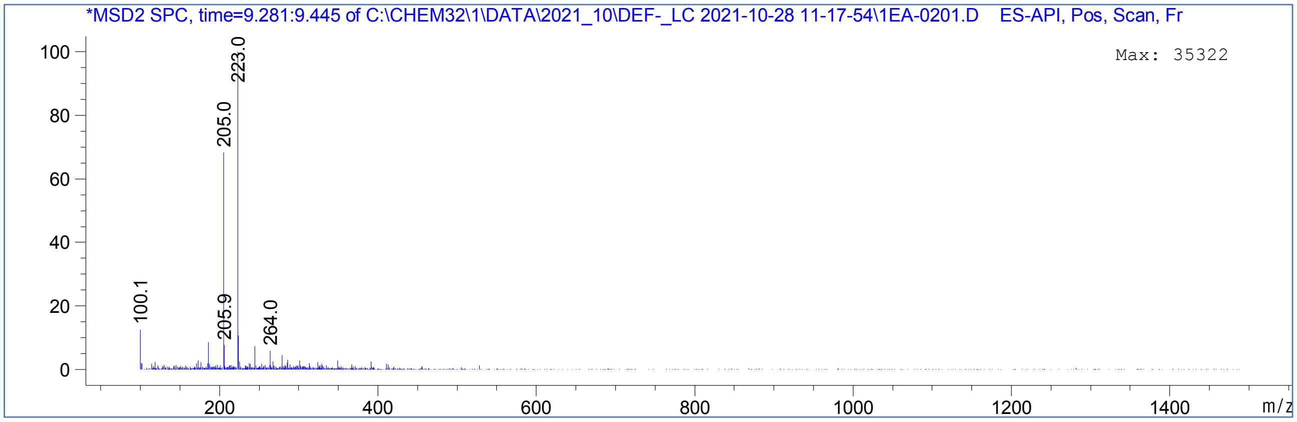

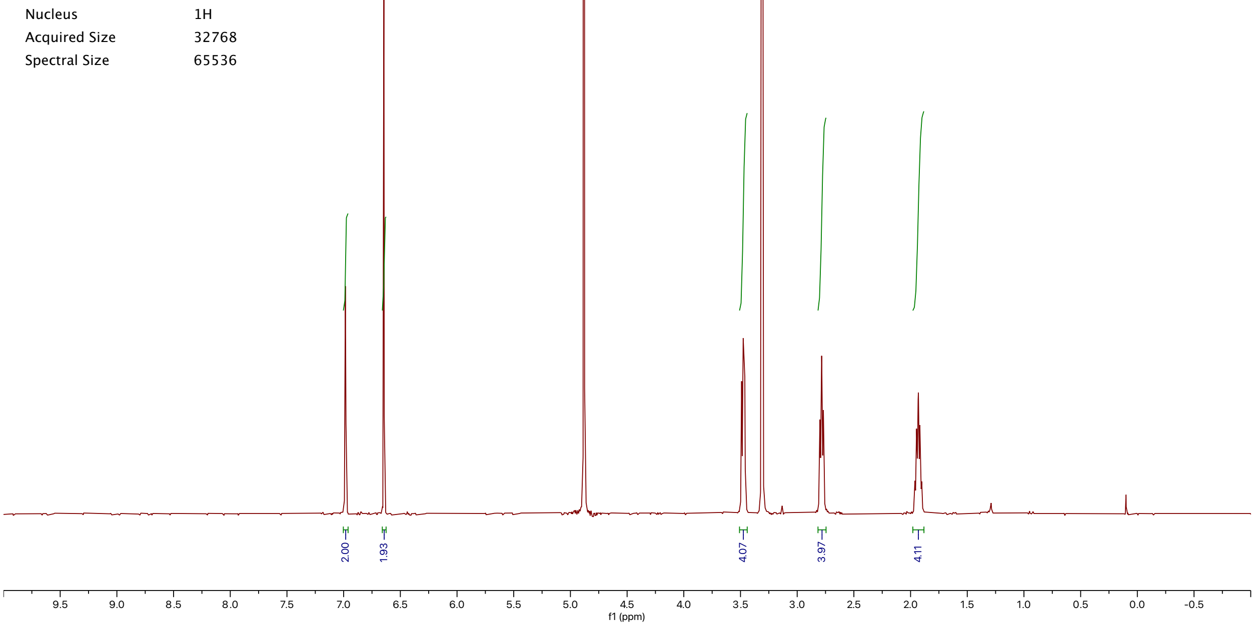

Origin Bruker BioSpin GmbH
Solvent MeOD
Temperature 295.3
Pulse Sequence zg30
Experiment 1D
Number of Scans 16
Acquisition Date 2021-09-15T14:57:00
Spectrometer Frequency 400.13
Spectral Width 8012.8
Lowest Frequency -1543.3
Nucleus 1H
Acquired Size 32768
Spectral Size 65536
2.00
1.93
4.07
3.97
4.11
f1 (ppm)

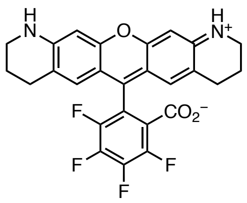

14

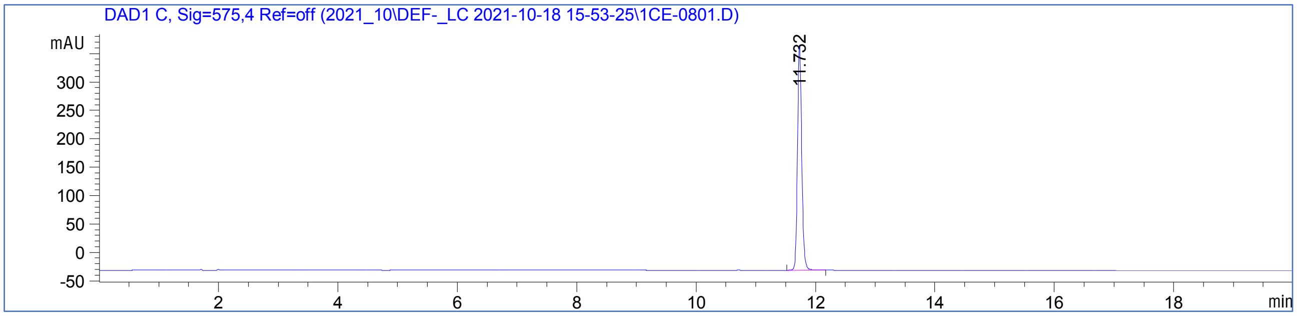

DAD1 C, Sig=575,4 Ref=off (2021_10\DEF-_LC 2021-10-18 15-53-25\1CE-0801.D)
mAU
11.732
min

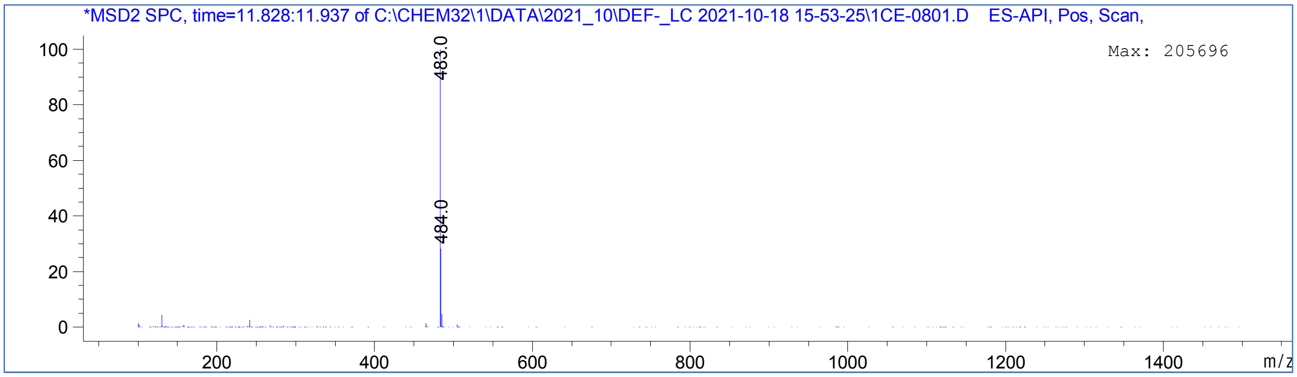

*MSD2 SPC, time=11.828:11.937 of C:\CHEM32\1\DATA\2021_10\DEF-_LC 2021-10-18 15-53-25\1CE-0801.D ES-API, Pos, Scan,
Max: 205696
483.0
484.0
m/z

Origin	Bruker BioSpin GmbH
Solvent	DMSO
Temperature	298.3
Pulse Sequence	zg30
Experiment	1D
Number of Scans	16
Acquisition Date	2022-11-10T15:03:00
Spectrometer Frequency	400.13
Spectral Width	8012.8
Lowest Frequency	-1538.8
Nucleus	1H
Acquired Size	32768
Spectral Size	65536

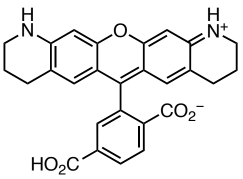

S17

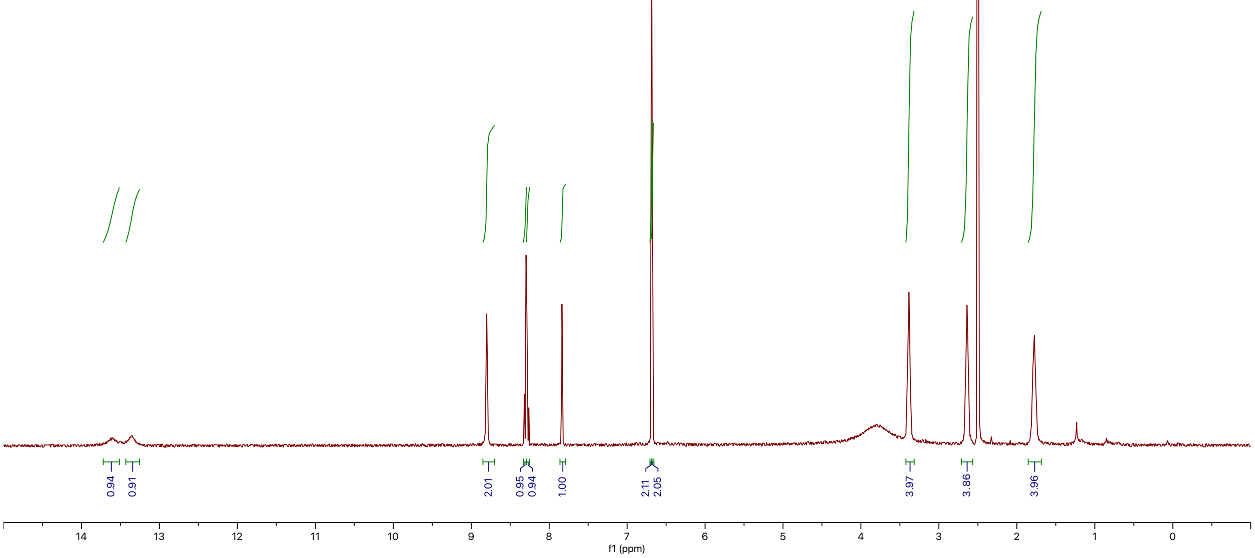

Origin	Bruker BioSpin GmbH
Solvent	DMSO
Temperature	299.1
Pulse Sequence	zgpg30
Experiment	1D
Number of Scans	4096
Acquisition Date	2022-11-11T00:20:00
Spectrometer Frequency	100.62
Spectral Width	24038.5
Lowest Frequency	-2006.6
Nucleus	13C
Acquired Size	32768
Spectral Size	65536

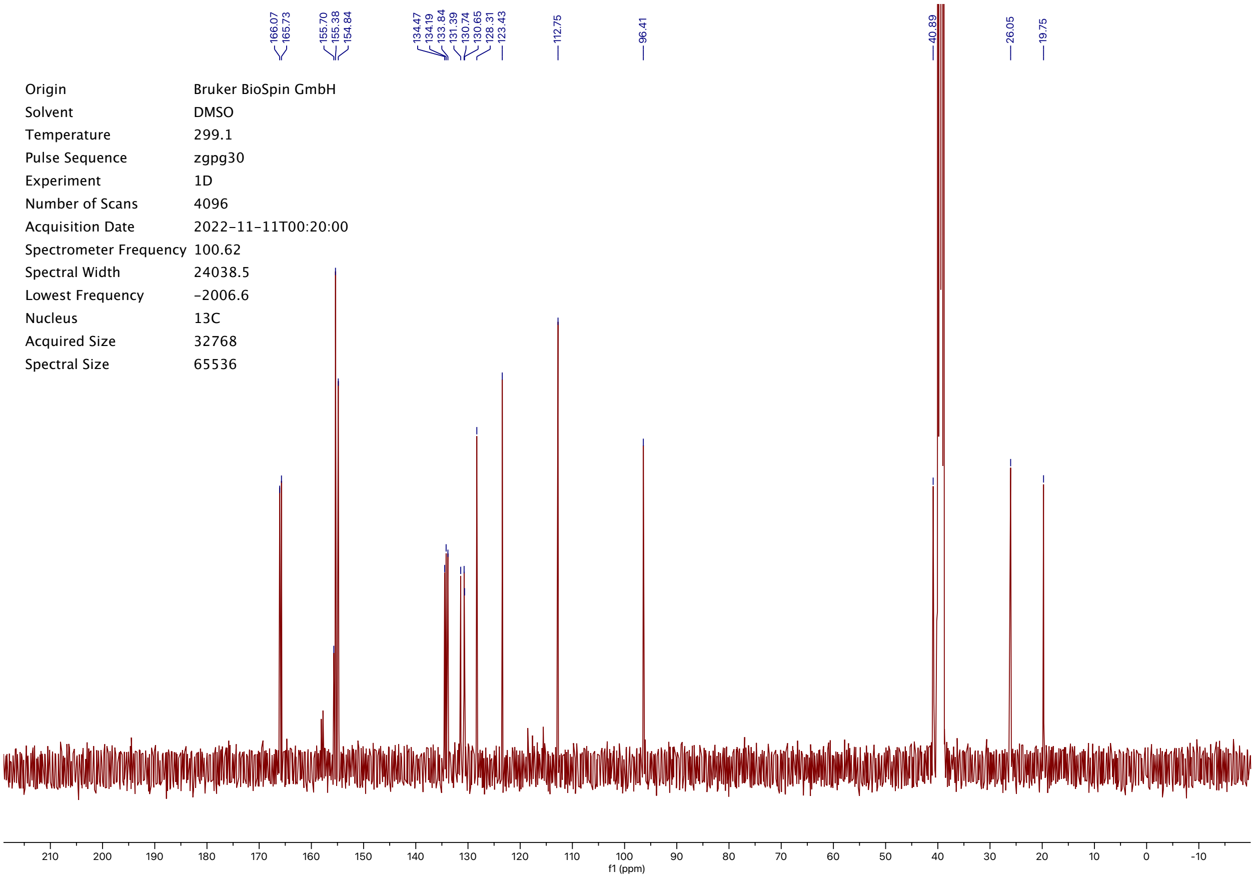

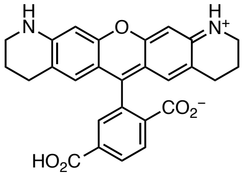

S17

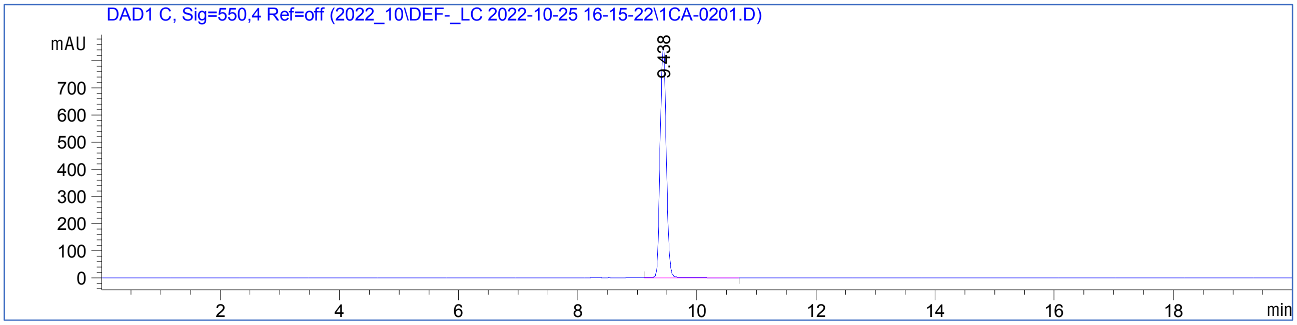

DAD1 C, Sig=550,4 Ref=off (2022_10\DEF-_LC 2022-10-25 16-15-22\1CA-0201.D)
mAU
700
600
500
400
300
200
100
0
9.438
2
4
6
8
10
12
14
16
18
min

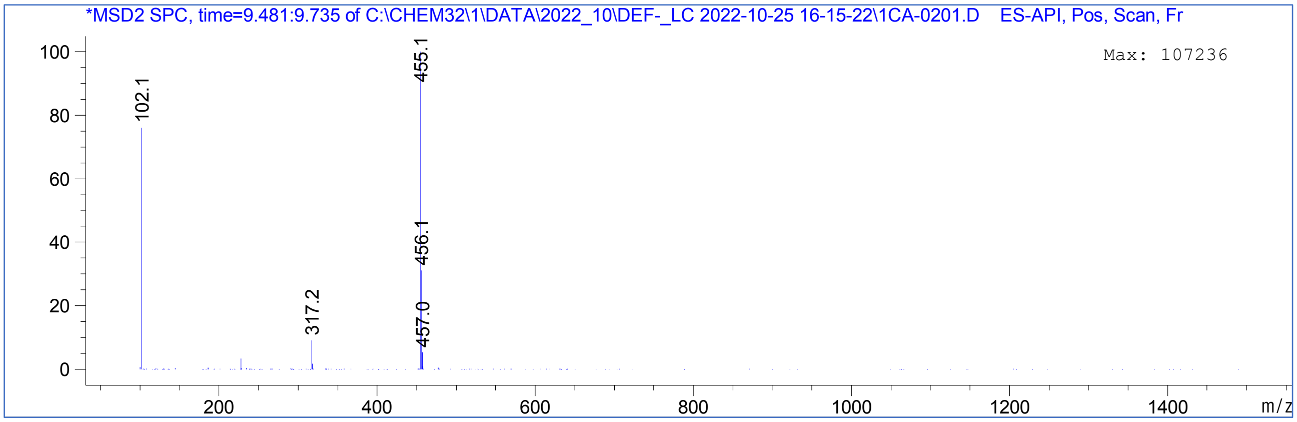

*MSD2 SPC, time=9.481:9.735 of C:\CHEM32\1\DATA\2022_10\DEF-_LC 2022-10-25 16-15-22\1CA-0201.D ES-API, Pos, Scan, Fr
Max: 107236
100
80
60
40
20
0
102.1
317.2
455.1
456.1
457.0
200
400
600
800
1000
1200
1400
m/z

Origin	Bruker BioSpin GmbH
Solvent	MeOD
Temperature	295.3
Pulse Sequence	zg30
Experiment	1D
Number of Scans	16
Acquisition Date	2021-06-29T15:41:00
Spectrometer Frequency	400.13
Spectral Width	8012.8
Lowest Frequency	-1543.3
Nucleus	1H
Acquired Size	32768
Spectral Size	65536

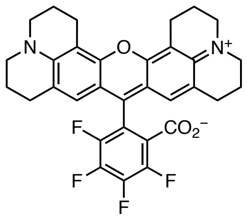

16

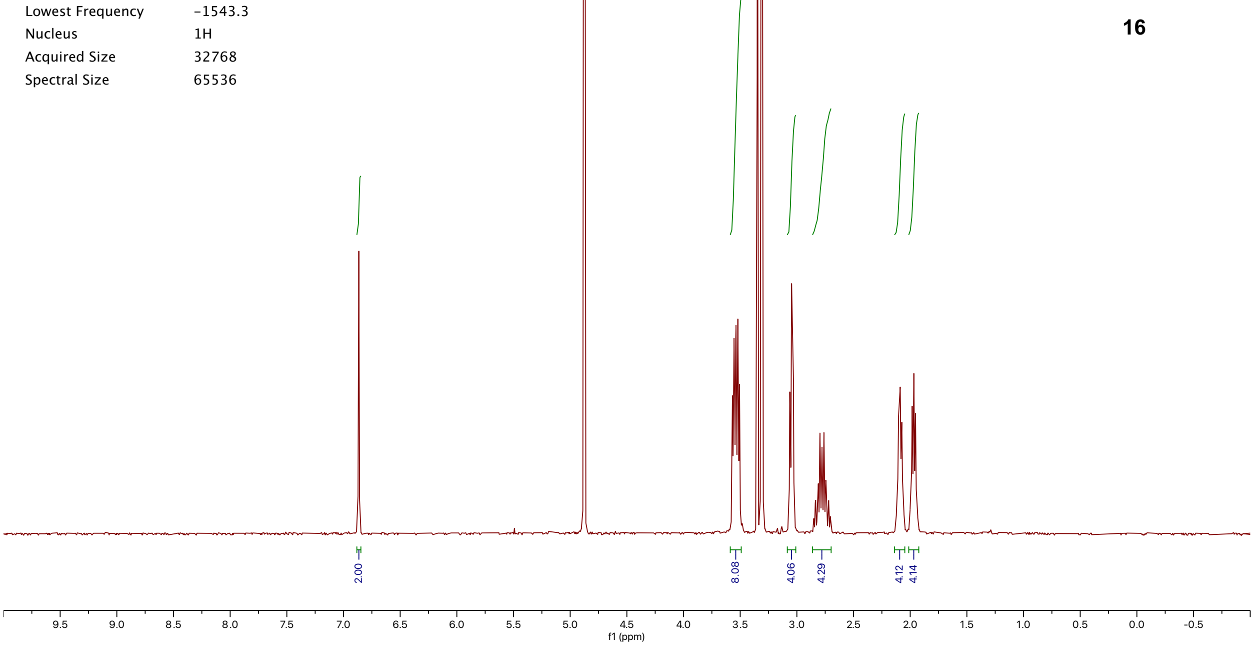

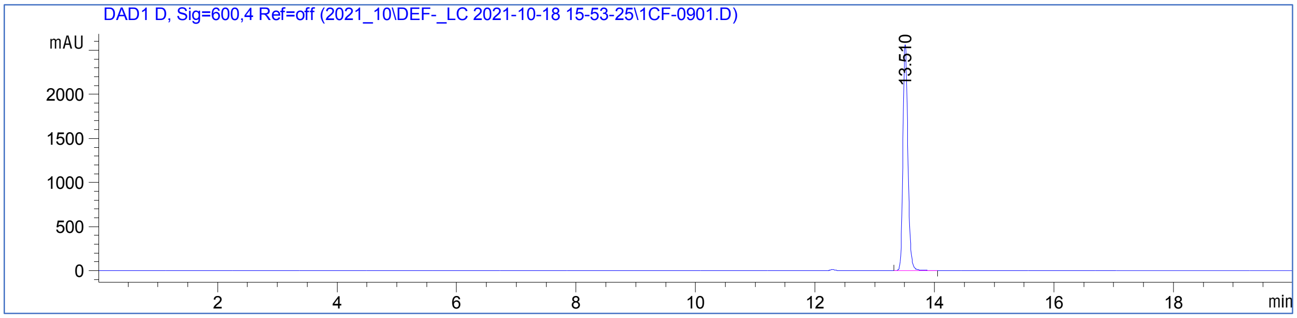

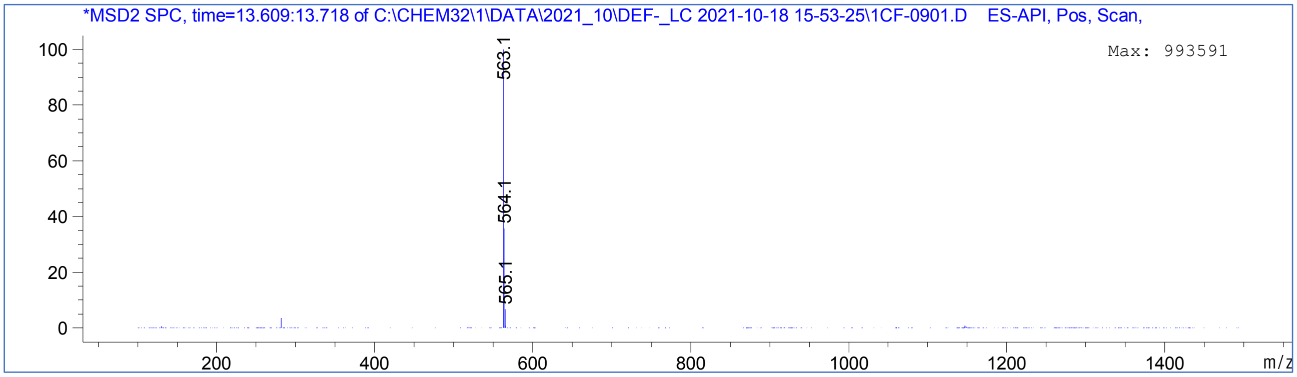

Origin	Bruker BioSpin GmbH
Solvent	MeOD
Temperature	295.5
Pulse Sequence	zg30
Experiment	1D
Number of Scans	16
Acquisition Date	2020-10-12T14:16:00
Spectrometer Frequency	400.13
Spectral Width	8012.8
Lowest Frequency	-1543.2
Nucleus	1H
Acquired Size	32768
Spectral Size	65536

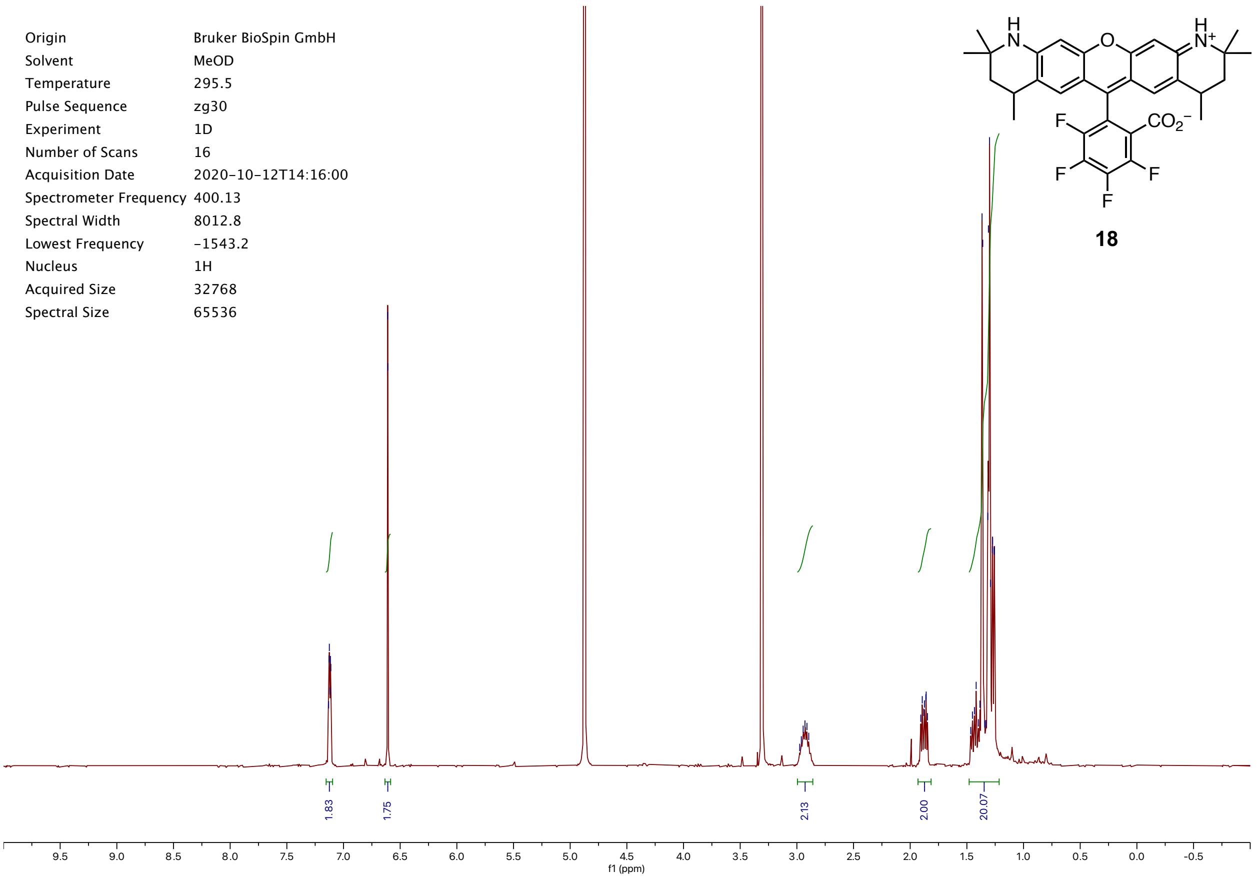

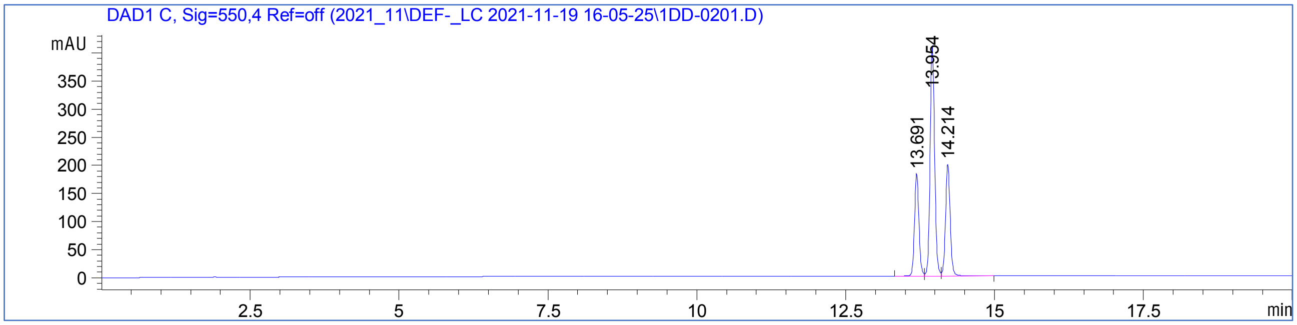

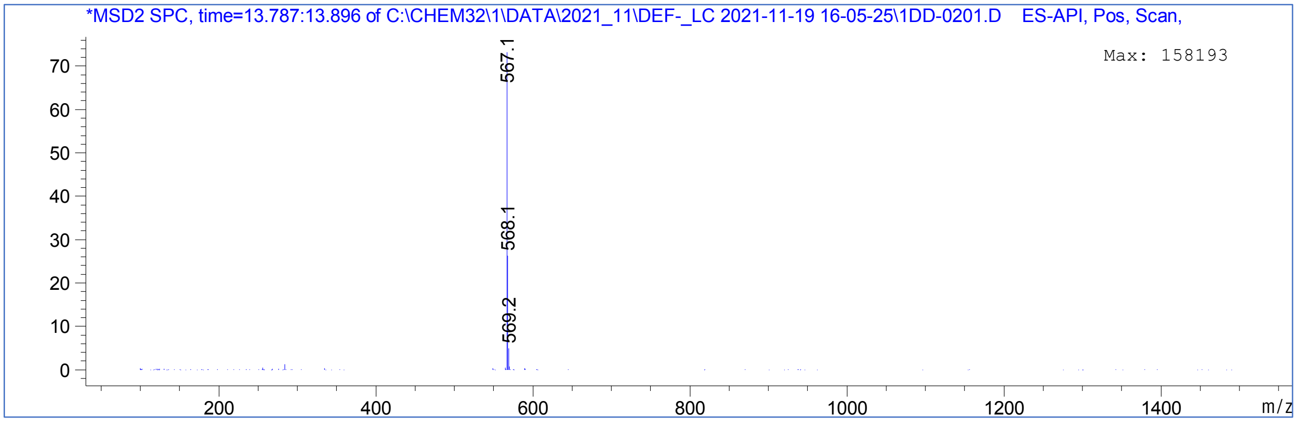

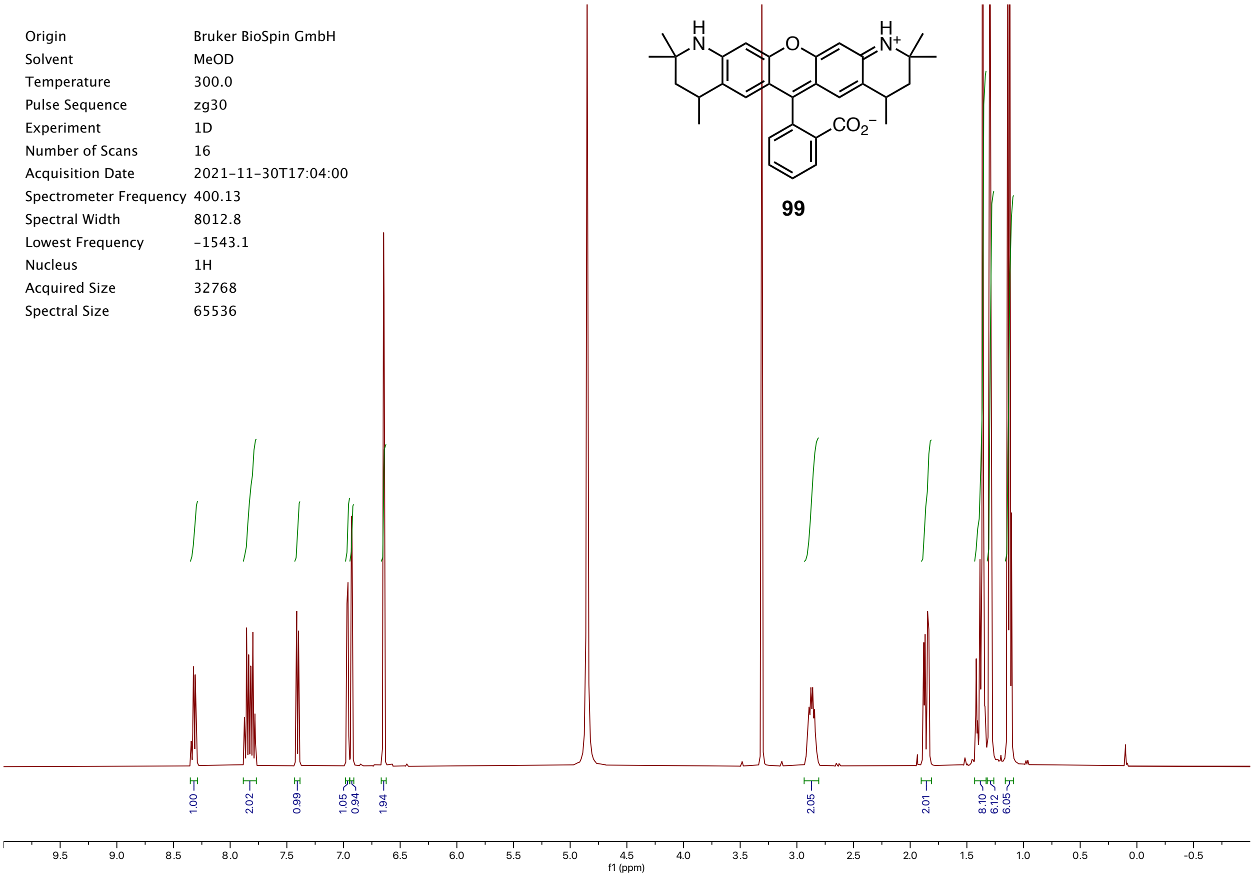
Origin Bruker BioSpin GmbH
Solvent MeOD
Temperature 300.0
Pulse Sequence zg30
Experiment 1D
Number of Scans 16
Acquisition Date 2021-11-30T17:04:00
Spectrometer Frequency 400.13
Spectral Width 8012.8
Lowest Frequency -1543.1
Nucleus 1H
Acquired Size 32768
Spectral Size 65536
99
1.00
2.02
0.99
1.05
0.94
1.94
2.05
2.01
8.10
6.12
6.05
f1 (ppm)

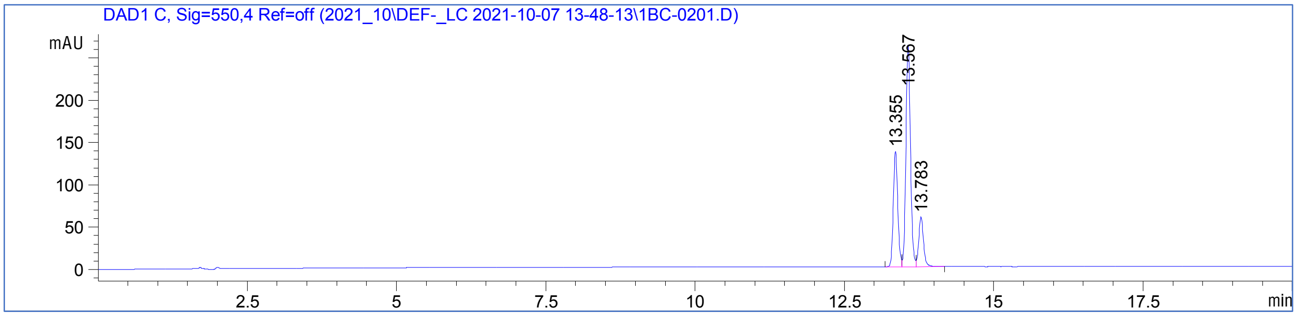
DAD1 C, Sig=550,4 Ref=off (2021_10\DEF-_LC 2021-10-07 13-48-13\1BC-0201.D)
mAU
13.355
13.567
13.783
min

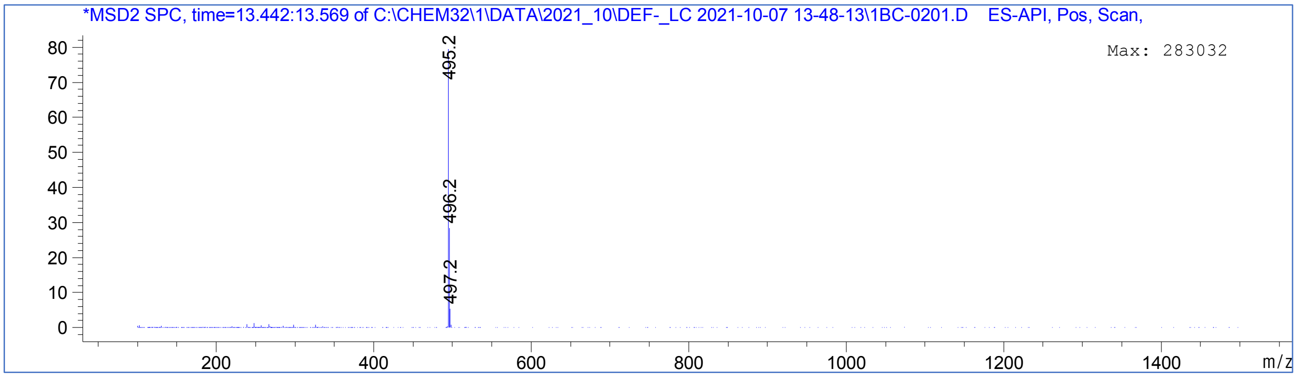
*MSD2 SPC, time=13.442:13.569 of C:\CHEM32\1\DATA\2021_10\DEF-_LC 2021-10-07 13-48-13\1BC-0201.D ES-API, Pos, Scan,
Max: 283032
495.2
496.2
497.2
m/z

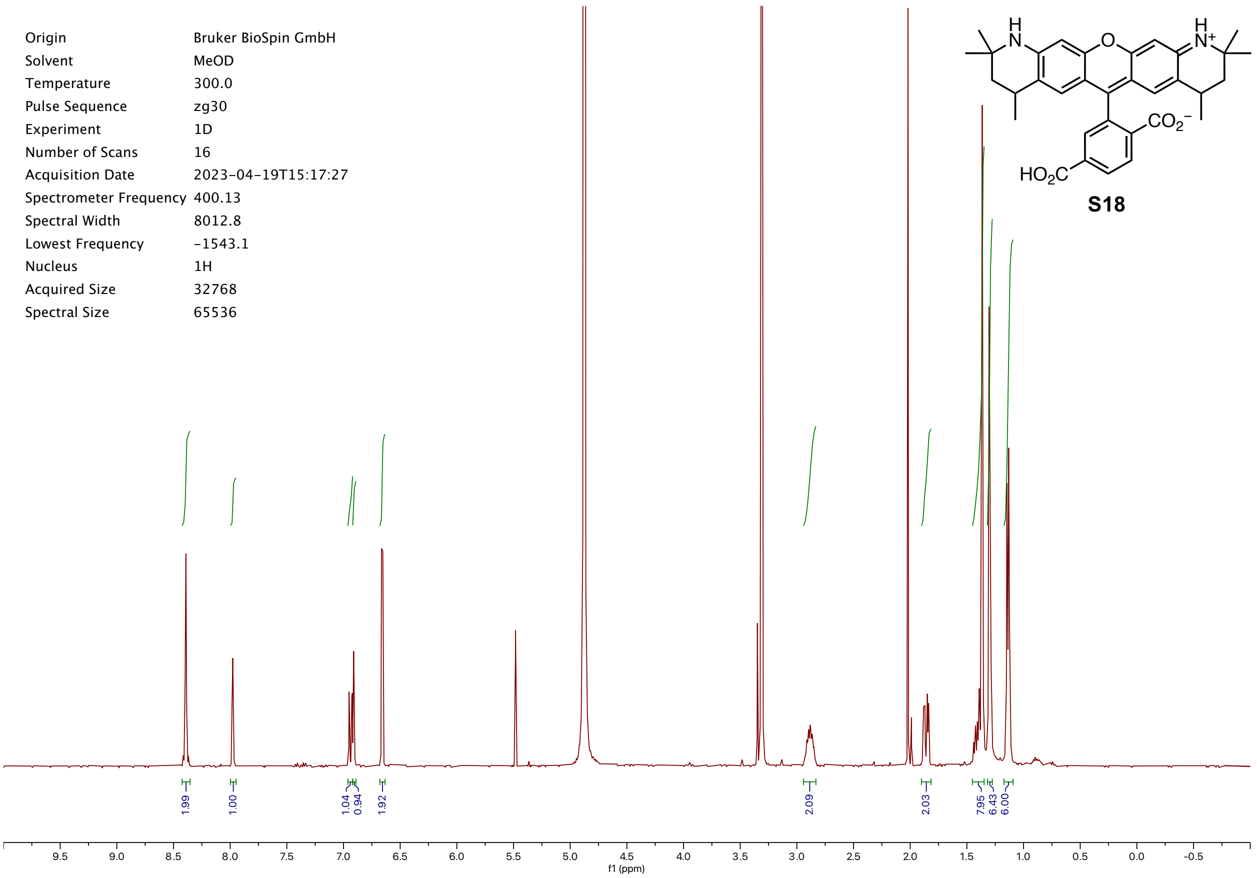
Origin Bruker BioSpin GmbH
Solvent MeOD
Temperature 300.0
Pulse Sequence zg30
Experiment 1D
Number of Scans 16
Acquisition Date 2023-04-19T15:17:27
Spectrometer Frequency 400.13
Spectral Width 8012.8
Lowest Frequency -1543.1
Nucleus 1H
Acquired Size 32768
Spectral Size 65536
S18
1.99
1.00
1.04
0.94
1.92
2.09
2.03
7.95
6.43
6.00
f1 (ppm)

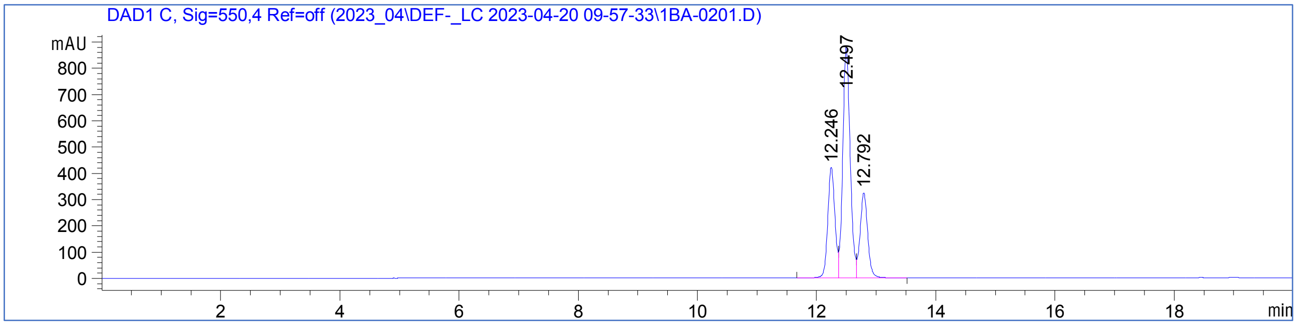
DAD1 C, Sig=550,4 Ref=off (2023_04\DEF-_LC 2023-04-20 09-57-33\1BA-0201.D)
mAU
12.246
12.497
12.792
min

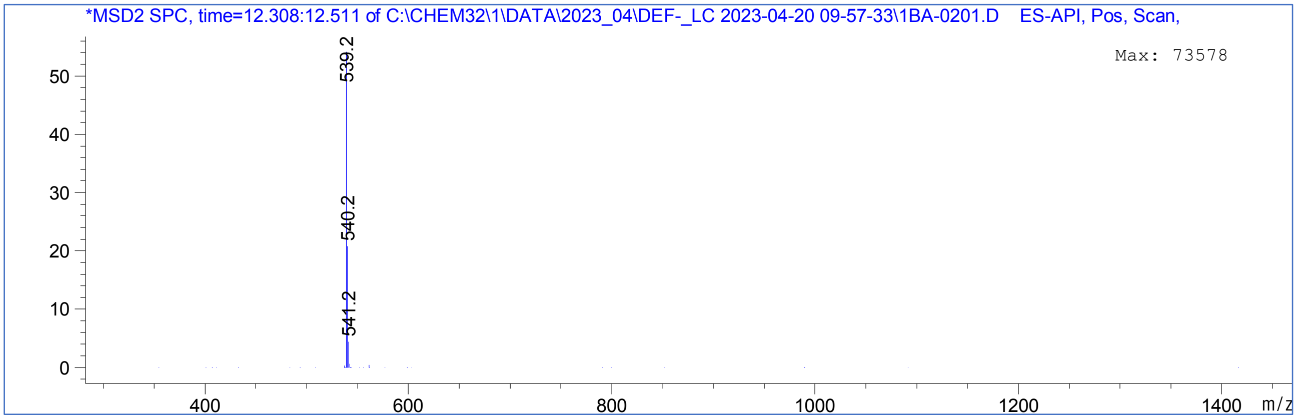
*MSD2 SPC, time=12.308:12.511 of C:\CHEM32\1\DATA\2023_04\DEF-_LC 2023-04-20 09-57-33\1BA-0201.D ES-API, Pos, Scan,
Max: 73578
539.2
540.2
541.2
m/z

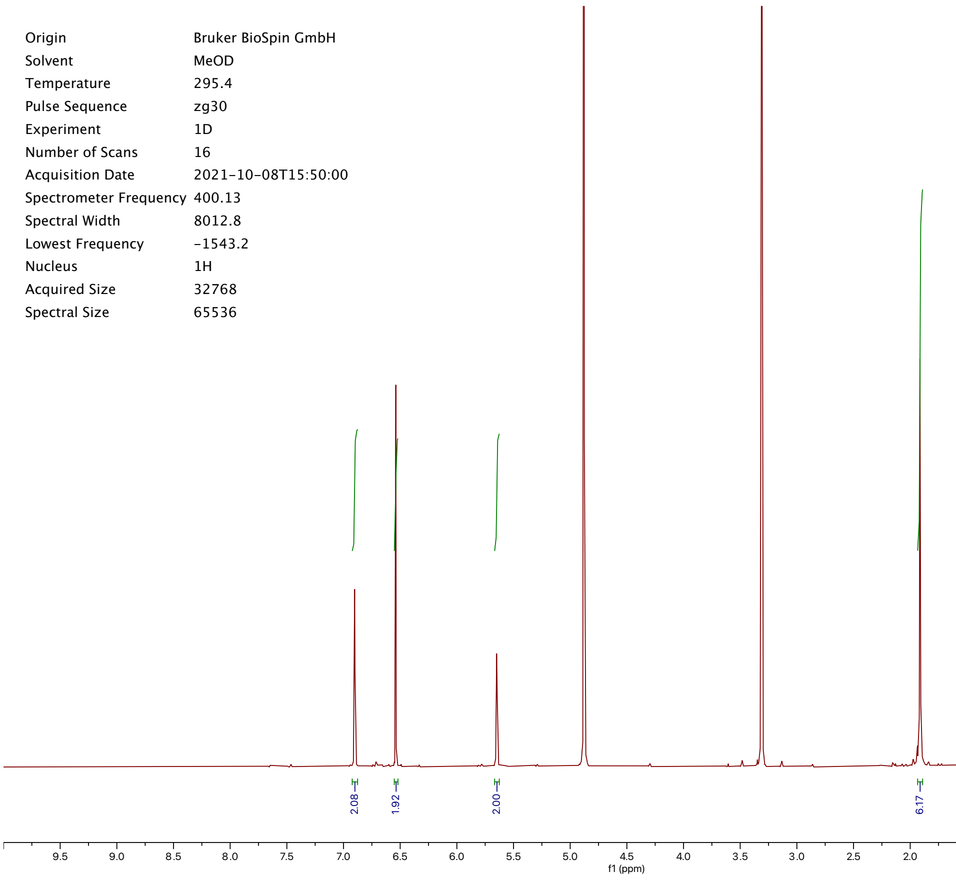

Origin Bruker BioSpin GmbH
Solvent MeOD
Temperature 295.4
Pulse Sequence zg30
Experiment 1D
Number of Scans 16
Acquisition Date 2021-10-08T15:50:00
Spectrometer Frequency 400.13
Spectral Width 8012.8
Lowest Frequency -1543.2
Nucleus 1H
Acquired Size 32768
Spectral Size 65536
2.08
1.92
2.00
6.17
6.05
5.91
f1 (ppm)

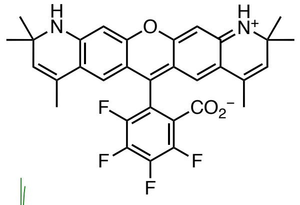

H
N
O
H
N+
F
CO2−
F
F
F

20

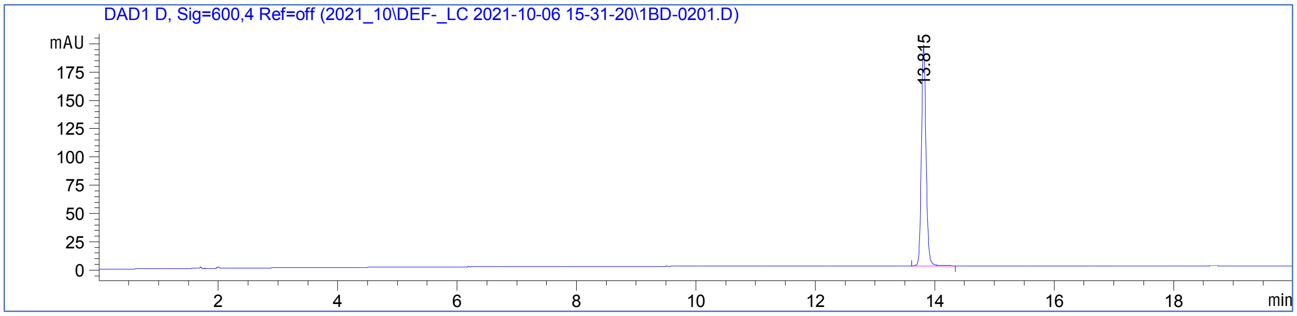

DAD1 D, Sig=600,4 Ref=off (2021_10\DEF-_LC 2021-10-06 15-31-20\1BD-0201.D)
mAU
13.815
min

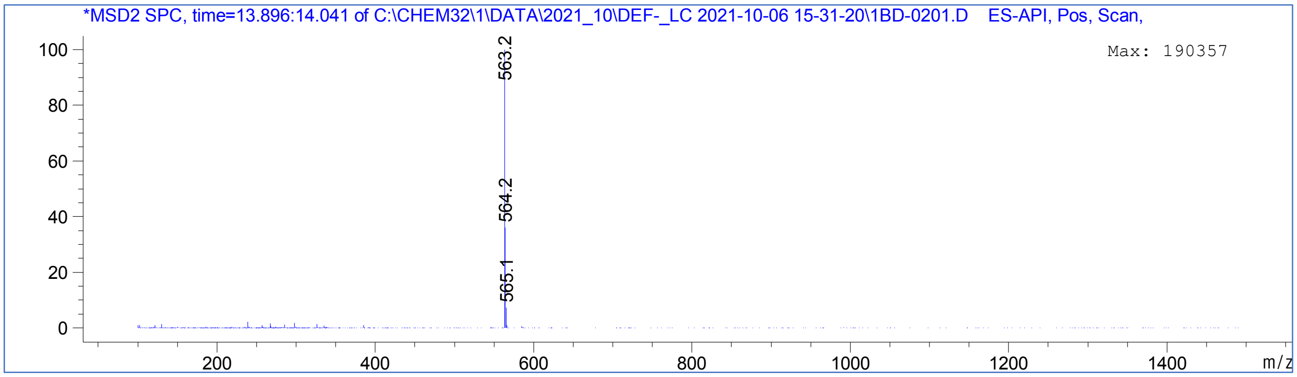

*MSD2 SPC, time=13.896:14.041 of C:\CHEM32\1\DATA\2021_10\DEF-_LC 2021-10-06 15-31-20\1BD-0201.D ES-API, Pos, Scan,
Max: 190357
563.2
564.2
565.1
m/z

Origin	Bruker BioSpin GmbH
Solvent	MeOD
Temperature	295.4
Pulse Sequence	zg30
Experiment	1D
Number of Scans	16
Acquisition Date	2021-10-11T14:48:00
Spectrometer Frequency	400.13
Spectral Width	8012.8
Lowest Frequency	-1543.2
Nucleus	1H
Acquired Size	32768
Spectral Size	65536

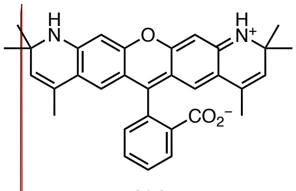

101

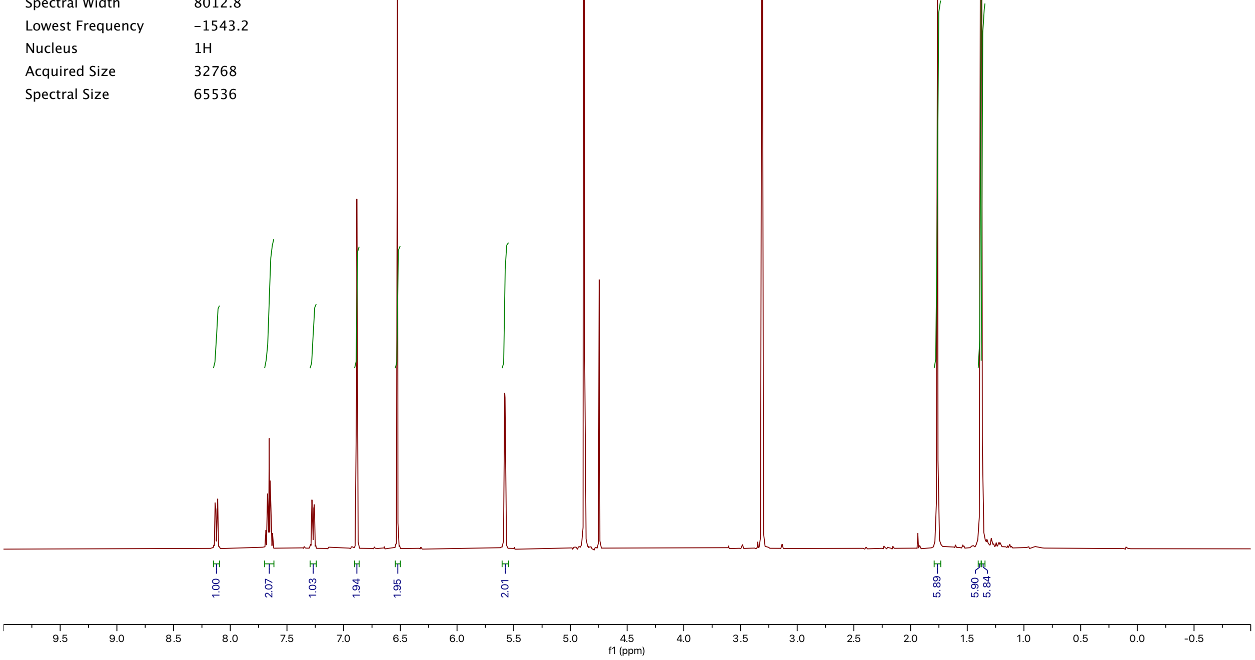

Origin	Bruker BioSpin GmbH
Solvent	MeOD
Temperature	300.0
Pulse Sequence	zgpg30
Experiment	1D
Number of Scans	8192
Acquisition Date	2022-02-05T01:14:00
Spectrometer Frequency	100.62
Spectral Width	24038.5
Lowest Frequency	-1817.2
Nucleus	13C
Acquired Size	32768
Spectral Size	65536

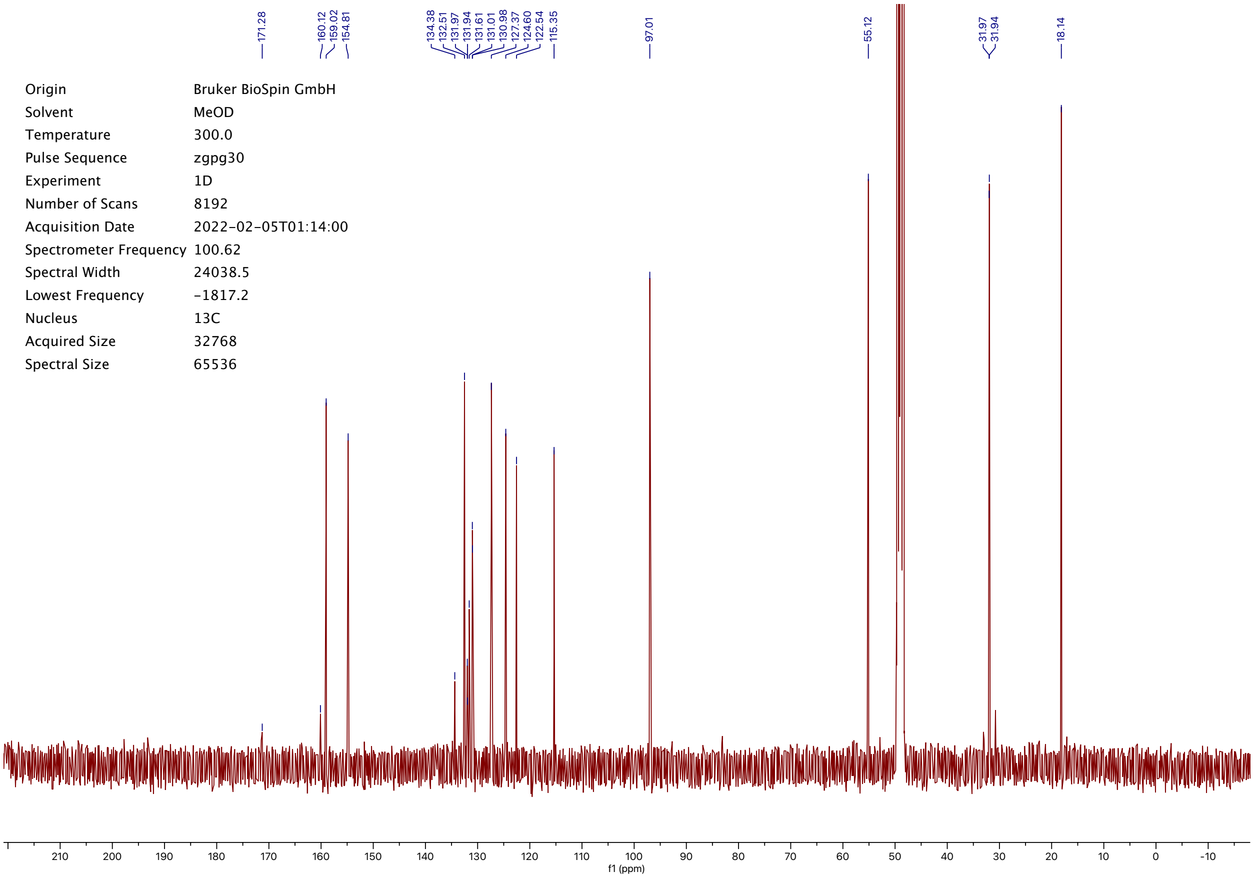

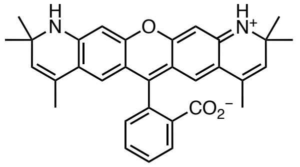

101

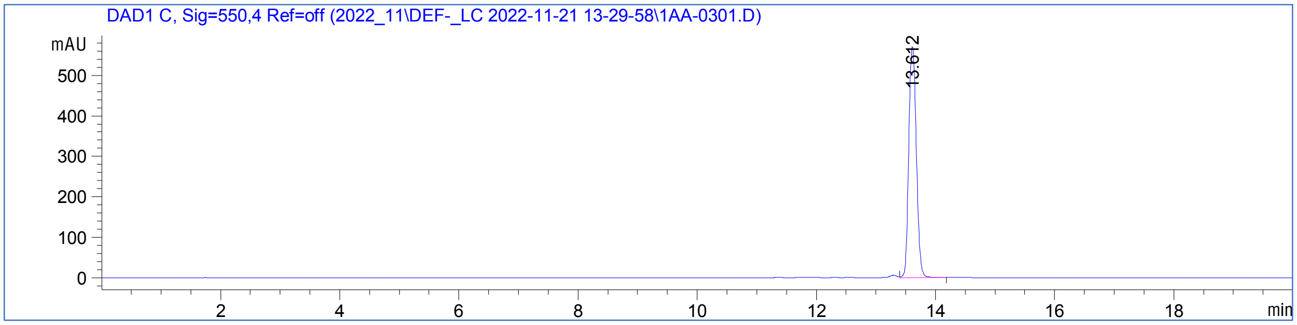

DAD1 C, Sig=550,4 Ref=off (2022_11\DEF-_LC 2022-11-21 13-29-58\1AA-0301.D)
mAU
500
400
300
200
100
0
13.612
2
4
6
8
10
12
14
16
18
min

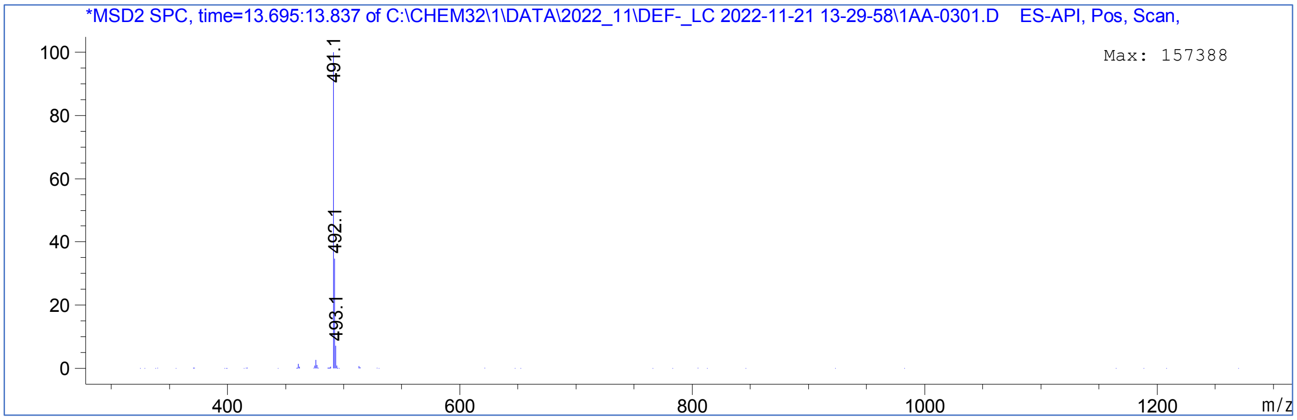

*MSD2 SPC, time=13.695:13.837 of C:\CHEM32\1\DATA\2022_11\DEF-_LC 2022-11-21 13-29-58\1AA-0301.D ES-API, Pos, Scan,
Max: 157388
100
80
60
40
20
0
491.1
492.1
493.1
400
600
800
1000
1200
m/z

Origin	Bruker BioSpin GmbH
Solvent	MeOD
Temperature	300.0
Pulse Sequence	zg30
Experiment	1D
Number of Scans	16
Acquisition Date	2022-01-10T14:21:00
Spectrometer Frequency	400.13
Spectral Width	8012.8
Lowest Frequency	-1543.2
Nucleus	1H
Acquired Size	32768
Spectral Size	65536

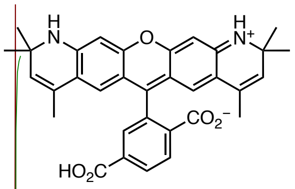

S19

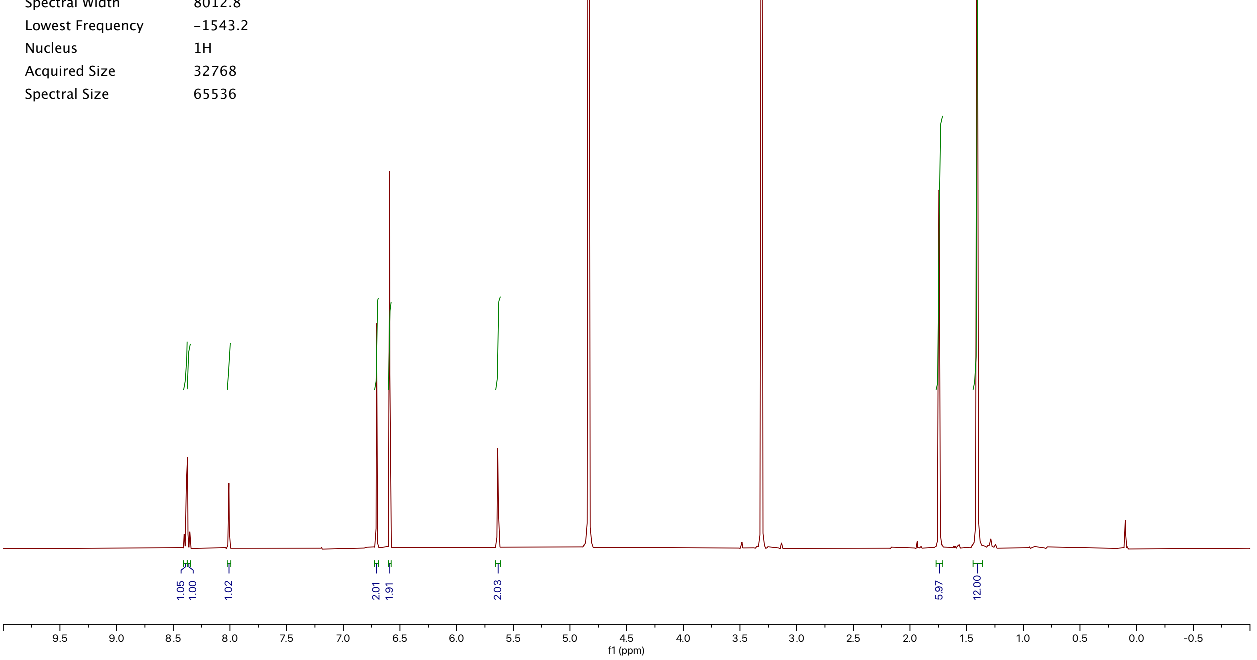

Origin	Bruker BioSpin GmbH
Solvent	MeOD
Temperature	299.8
Pulse Sequence	zgpg30
Experiment	1D
Number of Scans	4096
Acquisition Date	2022-11-16T05:36:00
Spectrometer Frequency	100.62
Spectral Width	24038.5
Lowest Frequency	-1817.2
Nucleus	13C
Acquired Size	32768
Spectral Size	65536

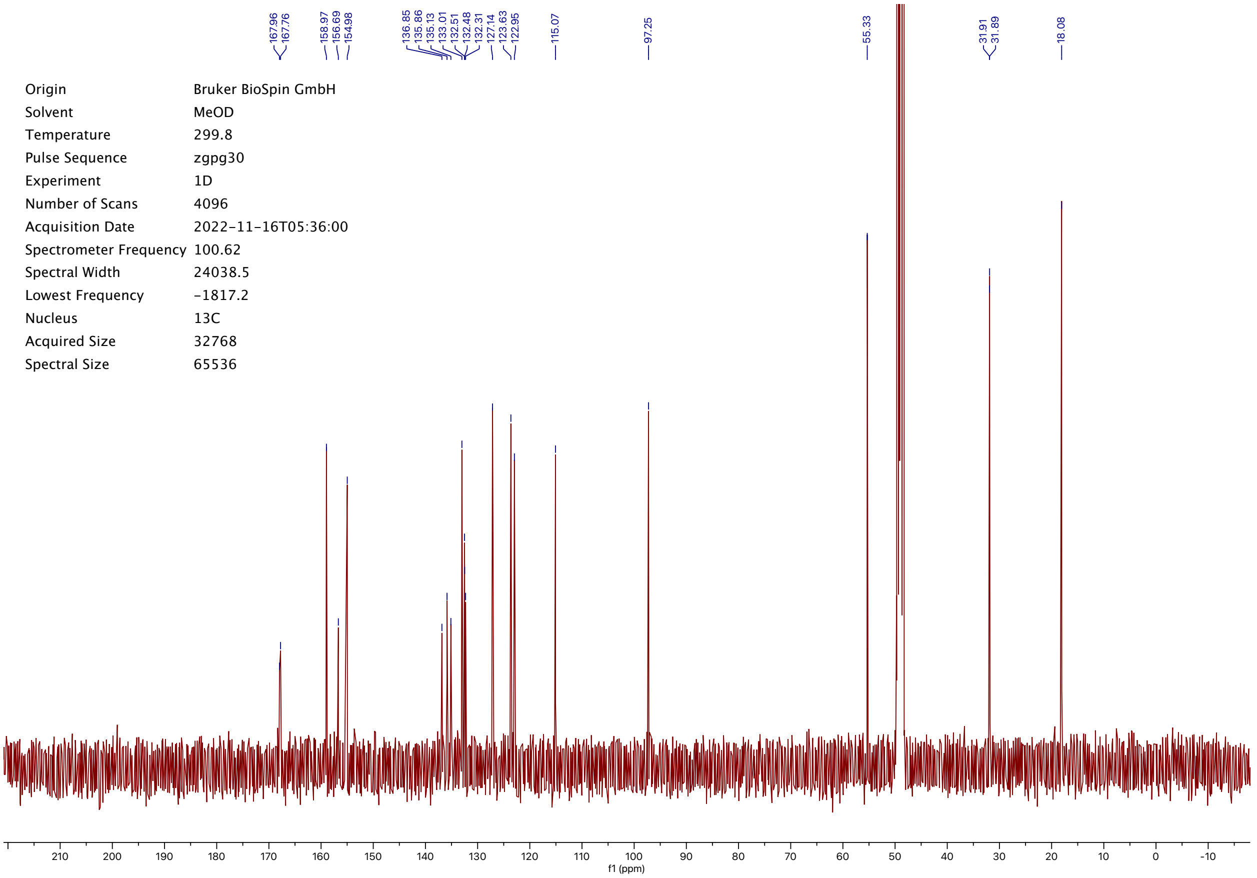

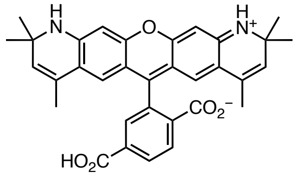
H
N
O
H
N+
CO2−
HO2C

S19

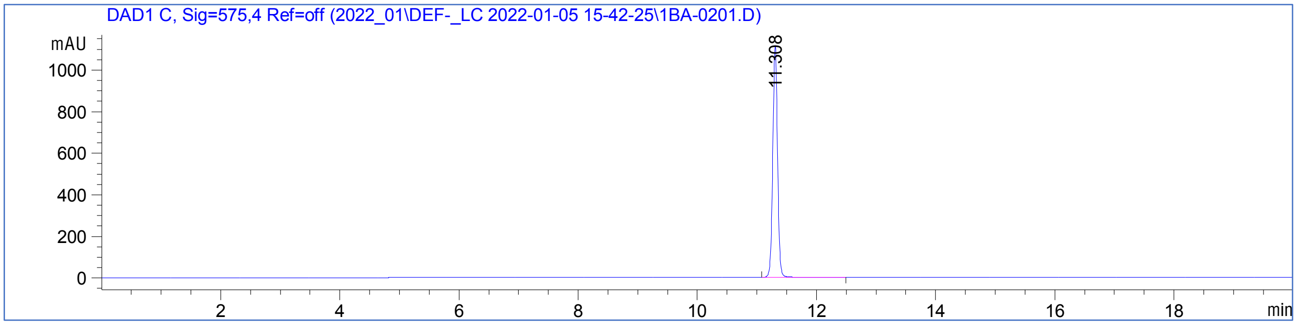
DAD1 C, Sig=575,4 Ref=off (2022_01\DEF-_LC 2022-01-05 15-42-25\1BA-0201.D)
mAU
1000
800
600
400
200
0
11.308
2
4
6
8
10
12
14
16
18
min

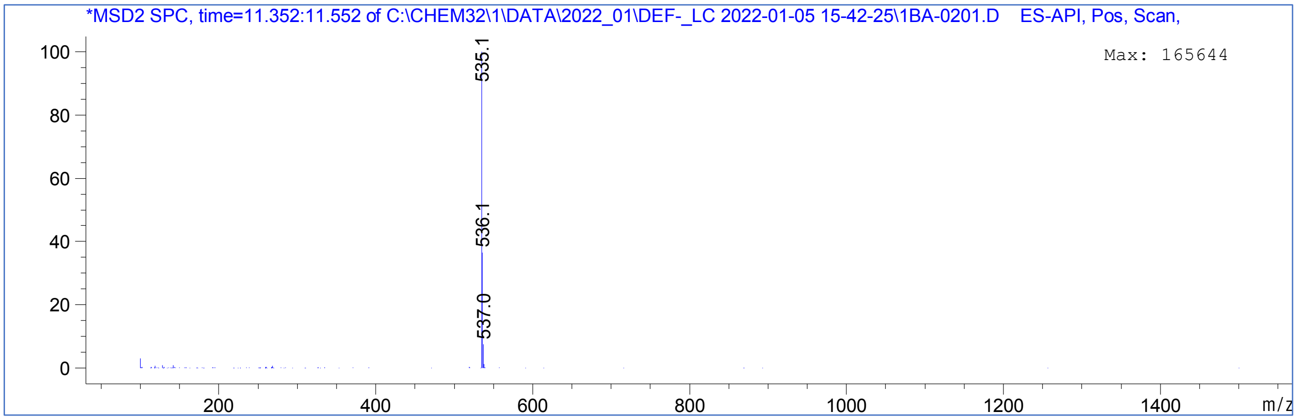
*MSD2 SPC, time=11.352:11.552 of C:\CHEM32\1\DATA\2022_01\DEF-_LC 2022-01-05 15-42-25\1BA-0201.D ES-API, Pos, Scan,
Max: 165644
100
80
60
40
20
0
535.1
536.1
537.0
200
400
600
800
1000
1200
1400
m/z

Origin	Bruker BioSpin GmbH
Solvent	MeOD
Temperature	295.5
Pulse Sequence	zg30
Experiment	1D
Number of Scans	16
Acquisition Date	2020-10-06T10:00:00
Spectrometer Frequency	400.13
Spectral Width	8012.8
Lowest Frequency	-1543.2
Nucleus	1H
Acquired Size	32768
Spectral Size	65536

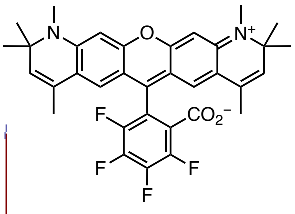

11

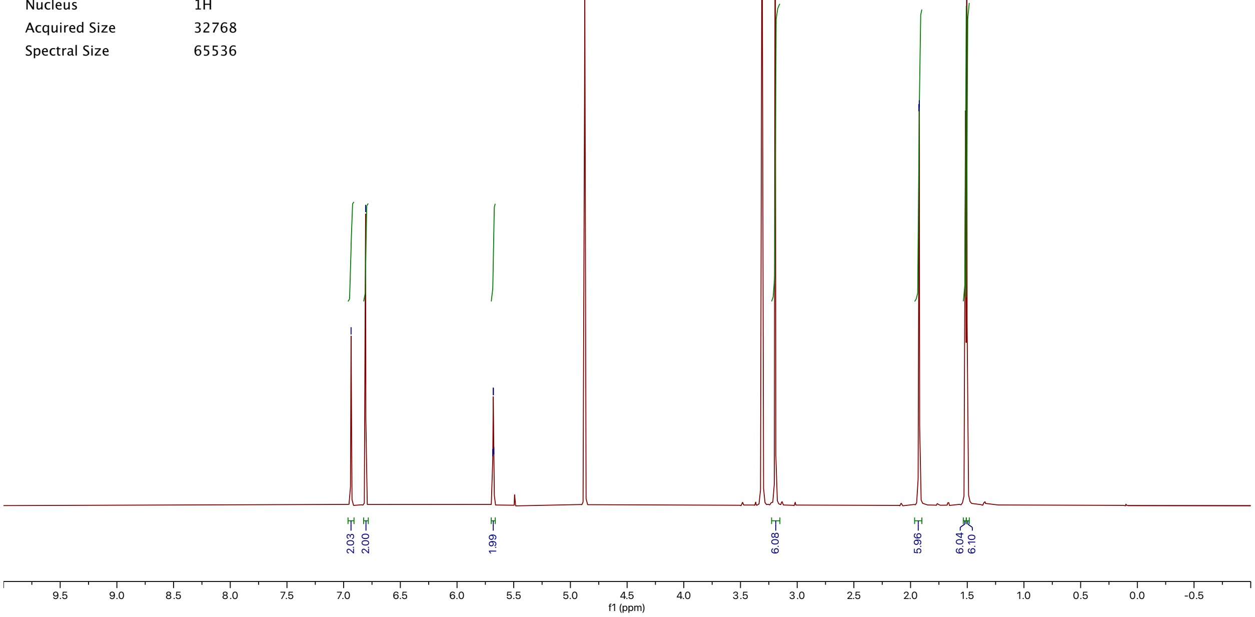

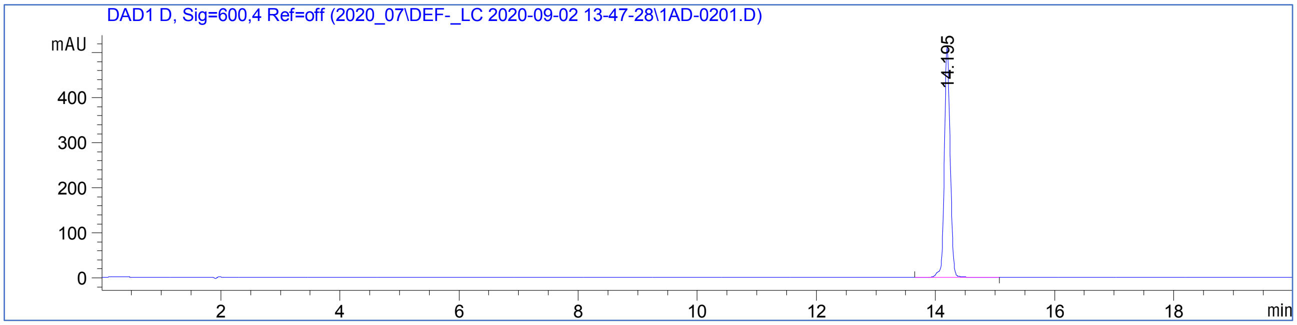

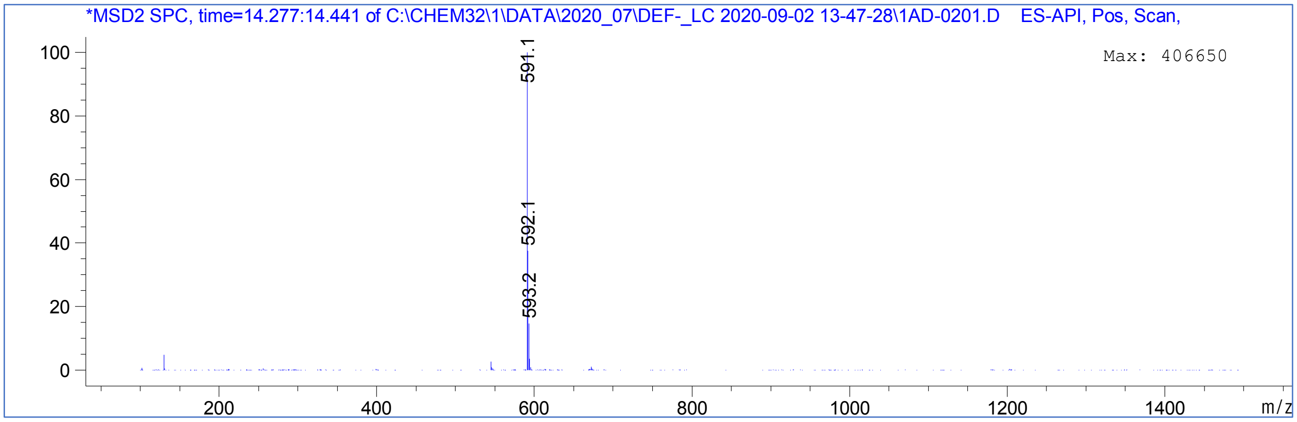

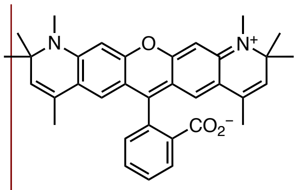

103

Origin	Bruker BioSpin GmbH
Solvent	MeOD
Temperature	300.0
Pulse Sequence	zg30
Experiment	1D
Number of Scans	16
Acquisition Date	2019-05-31T11:05:00
Spectrometer Frequency	400.13
Spectral Width	8012.8
Lowest Frequency	-1543.2
Nucleus	1H
Acquired Size	32768
Spectral Size	65536

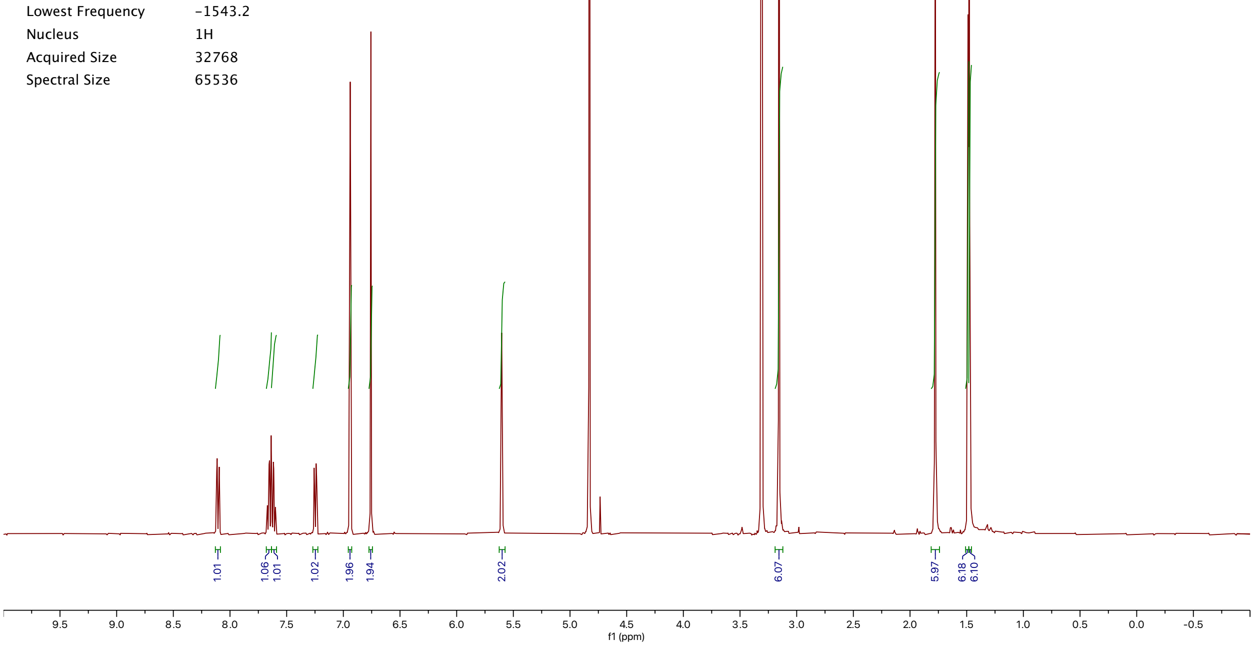

Origin	Bruker BioSpin GmbH
Solvent	MeOD
Temperature	300.0
Pulse Sequence	zgpg30
Experiment	1D
Number of Scans	2048
Acquisition Date	2019-06-05T06:09:00
Spectrometer Frequency	100.62
Spectral Width	24038.5
Lowest Frequency	-1819.3
Nucleus	13C
Acquired Size	32768
Spectral Size	65536

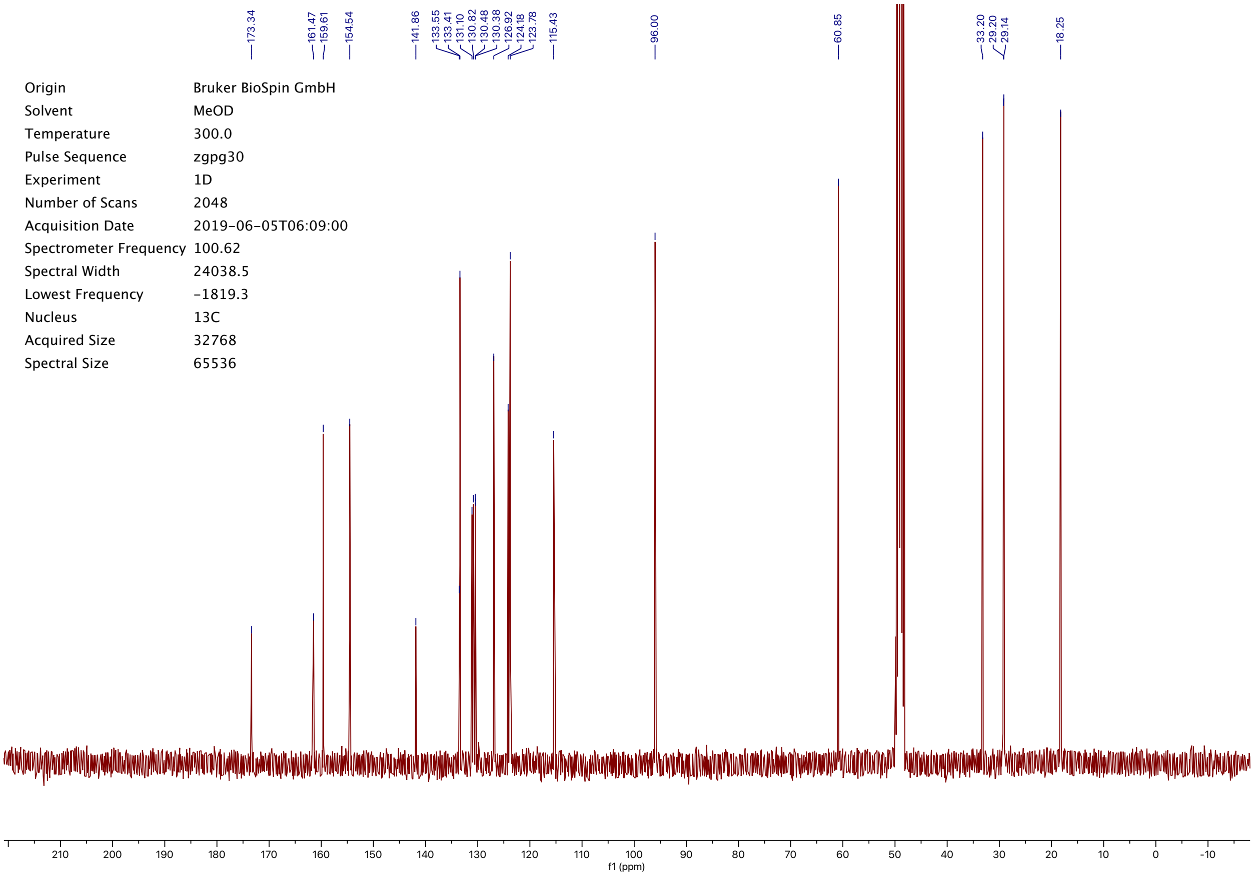

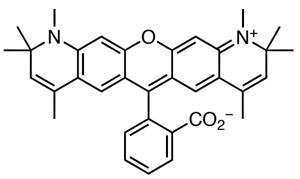

N
O
N+
CO2−

103

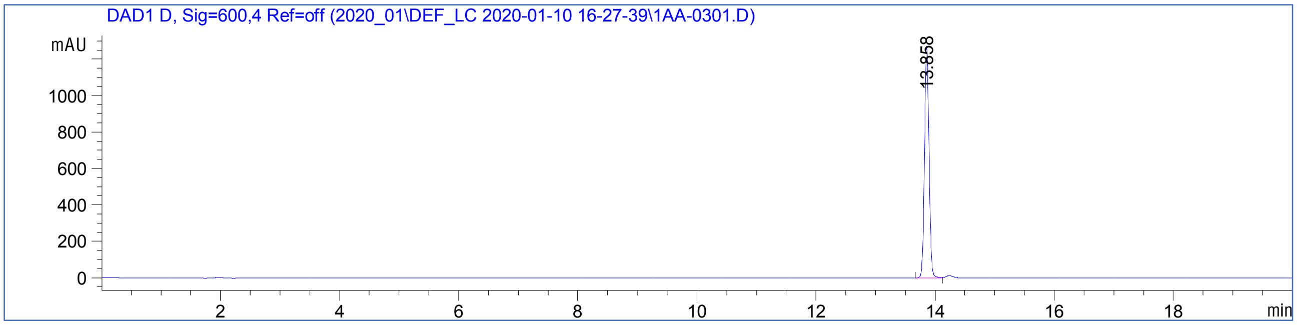

DAD1 D, Sig=600,4 Ref=off (2020_01\DEF_LC 2020-01-10 16-27-39\1AA-0301.D)
mAU
1000
800
600
400
200
0
13.858
2
4
6
8
10
12
14
16
18
min

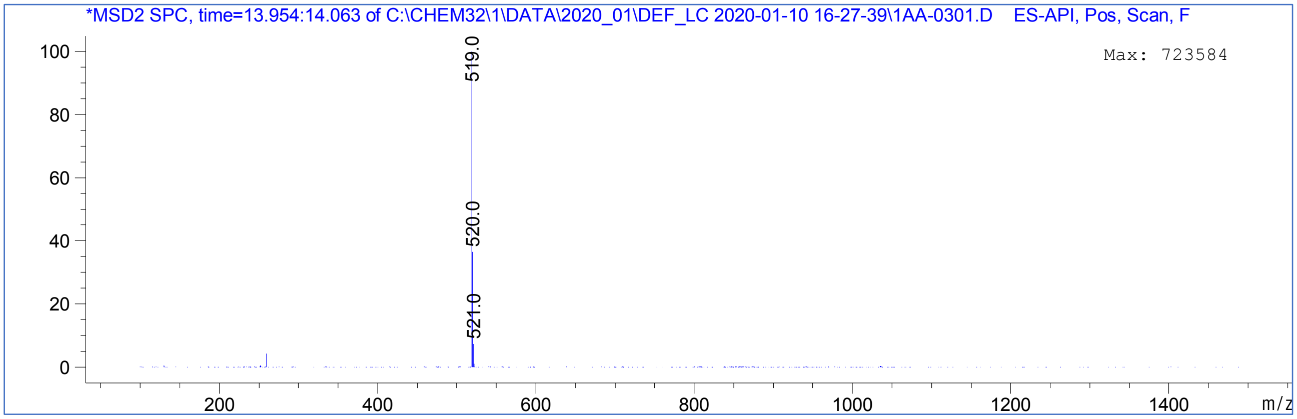

*MSD2 SPC, time=13.954:14.063 of C:\CHEM32\1\DATA\2020_01\DEF_LC 2020-01-10 16-27-39\1AA-0301.D ES-API, Pos, Scan, F
Max: 723584
100
80
60
40
20
0
519.0
520.0
521.0
200
400
600
800
1000
1200
1400
m/z

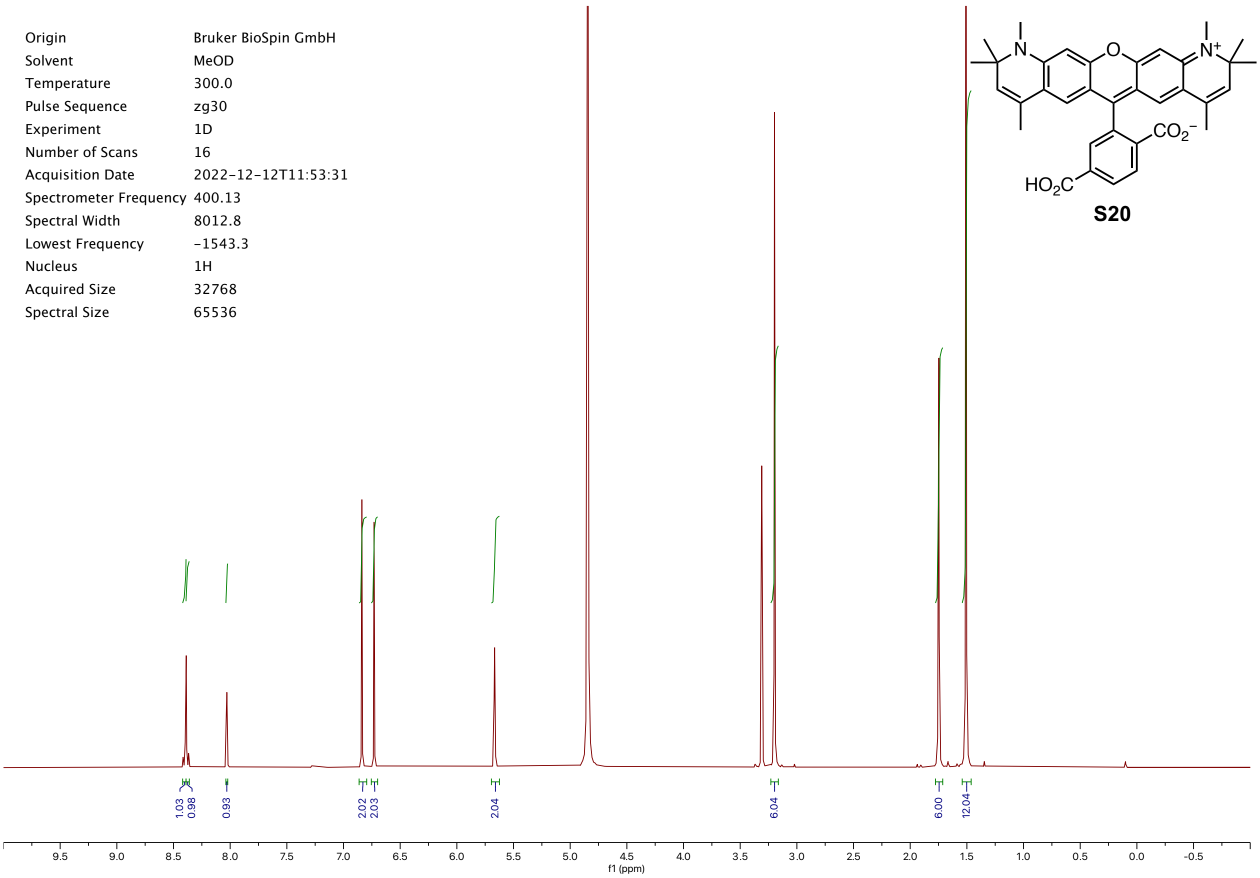
Origin Bruker BioSpin GmbH
Solvent MeOD
Temperature 300.0
Pulse Sequence zg30
Experiment 1D
Number of Scans 16
Acquisition Date 2022-12-12T11:53:31
Spectrometer Frequency 400.13
Spectral Width 8012.8
Lowest Frequency -1543.3
Nucleus 1H
Acquired Size 32768
Spectral Size 65536
1.03
0.98
0.93
2.02
2.03
2.04
6.04
6.00
12.04
f1 (ppm)

S20

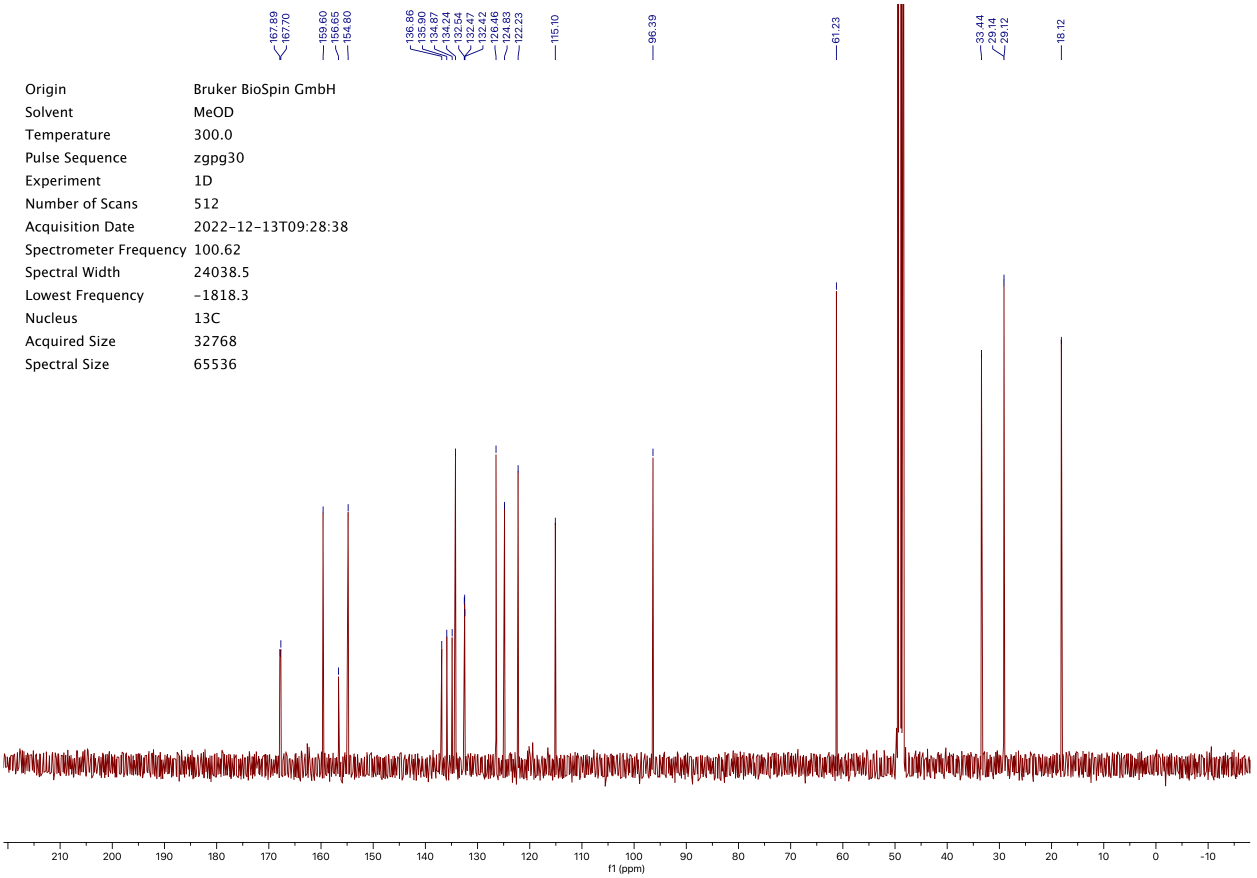
167.89
167.70
159.60
156.65
154.80
136.86
135.90
134.87
134.24
132.54
132.47
132.42
126.46
124.83
122.23
115.10
96.39
61.23
33.44
29.14
29.12
18.12
Origin Bruker BioSpin GmbH
Solvent MeOD
Temperature 300.0
Pulse Sequence zgpg30
Experiment 1D
Number of Scans 512
Acquisition Date 2022-12-13T09:28:38
Spectrometer Frequency 100.62
Spectral Width 24038.5
Lowest Frequency -1818.3
Nucleus 13C
Acquired Size 32768
Spectral Size 65536
f1 (ppm)

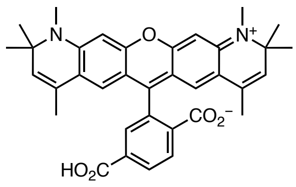

S20

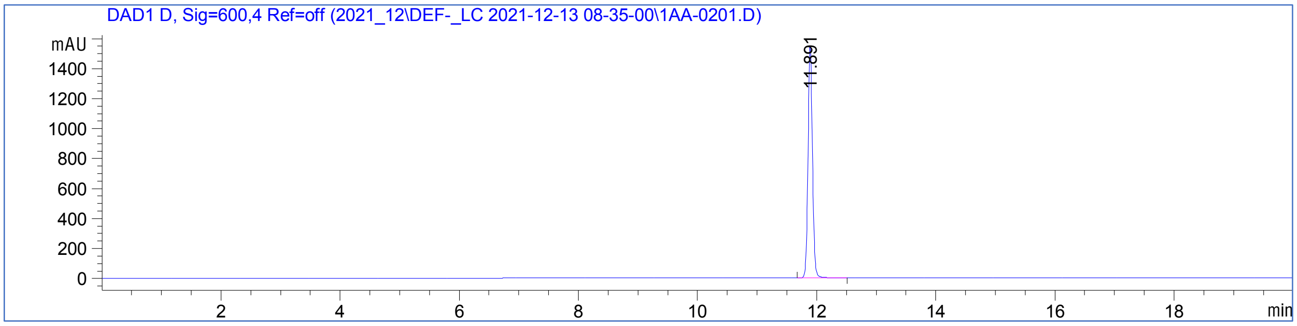
DAD1 D, Sig=600,4 Ref=off (2021_12\DEF-_LC 2021-12-13 08-35-00\1AA-0201.D)
mAU
1400
1200
1000
800
600
400
200
0
11.891
2
4
6
8
10
12
14
16
18
min

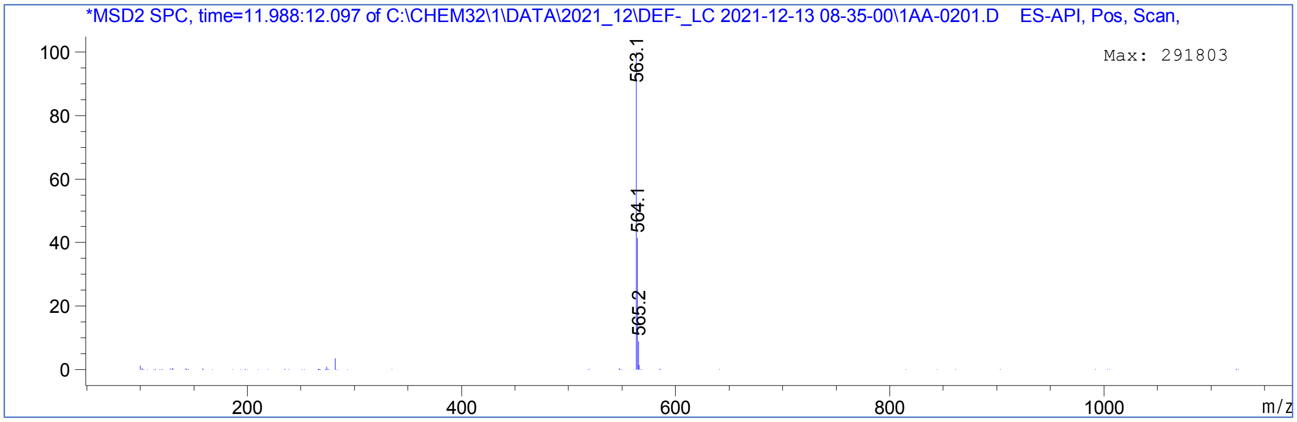
*MSD2 SPC, time=11.988:12.097 of C:\CHEM32\1\DATA\2021_12\DEF-_LC 2021-12-13 08-35-00\1AA-0201.D ES-API, Pos, Scan,
Max: 291803
100
80
60
40
20
0
563.1
564.1
565.2
200
400
600
800
1000
m/z

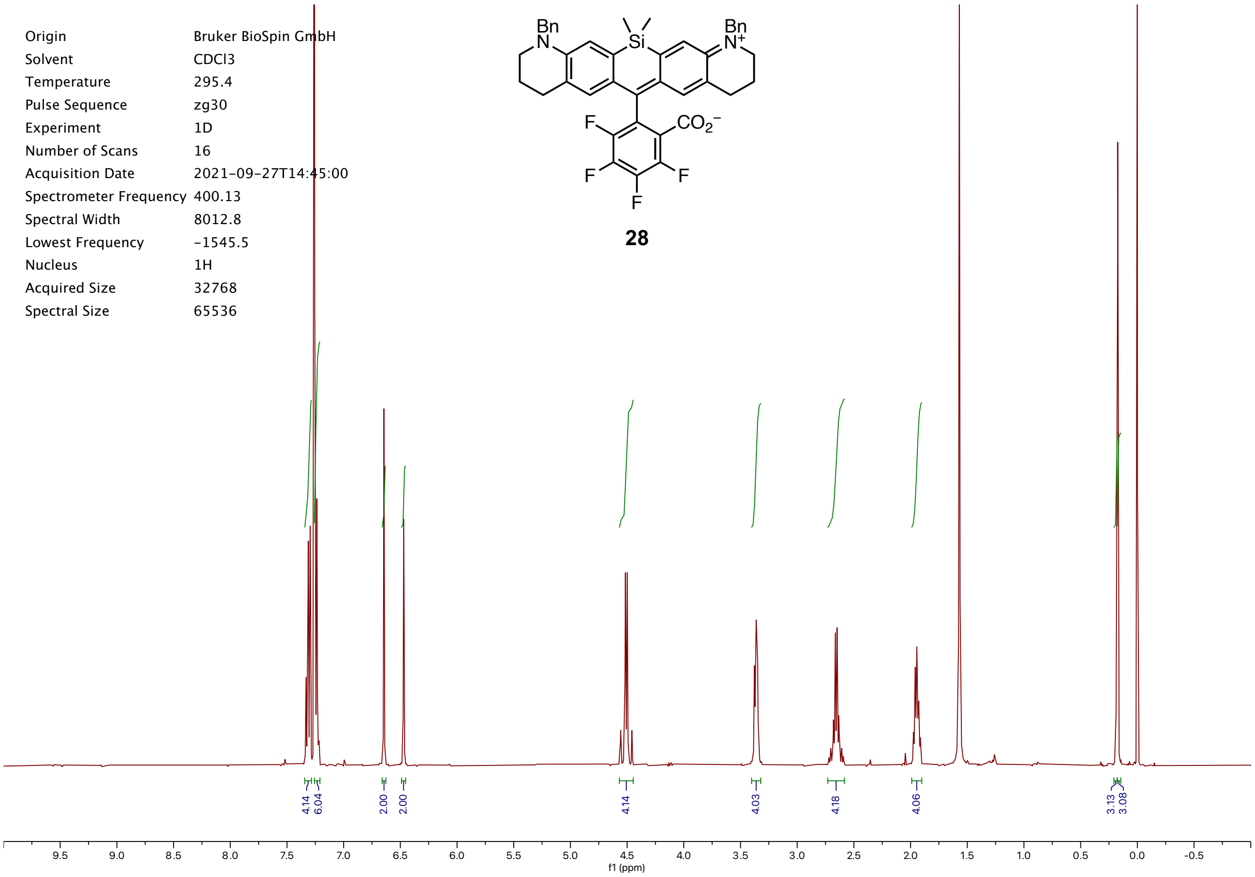
Origin Bruker BioSpin GmbH
Solvent CDCl3
Temperature 295.4
Pulse Sequence zg30
Experiment 1D
Number of Scans 16
Acquisition Date 2021-09-27T14:45:00
Spectrometer Frequency 400.13
Spectral Width 8012.8
Lowest Frequency -1545.5
Nucleus 1H
Acquired Size 32768
Spectral Size 65536
4.14
6.04
2.00
2.00
4.14
4.03
4.18
4.06
3.13
3.08
9.5 9.0 8.5 8.0 7.5 7.0 6.5 6.0 5.5 5.0 4.5 4.0 3.5 3.0 2.5 2.0 1.5 1.0 0.5 0.0 -0.5
f1 (ppm)
Bn
Si
Bn
N
N+
F
CO2−
F
F
F

28

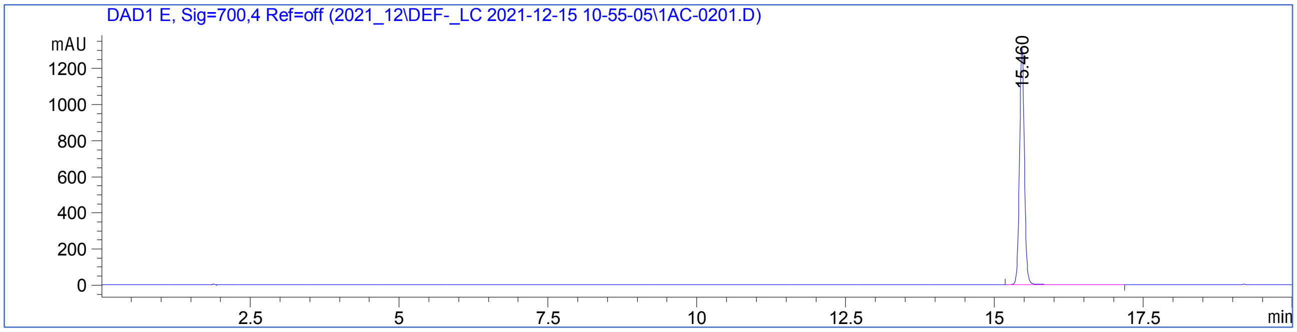
DAD1 E, Sig=700,4 Ref=off (2021_12\DEF-_LC 2021-12-15 10-55-05\1AC-0201.D)
mAU
1200
1000
800
600
400
200
0
15.460
2.5
5
7.5
10
12.5
15
17.5
min

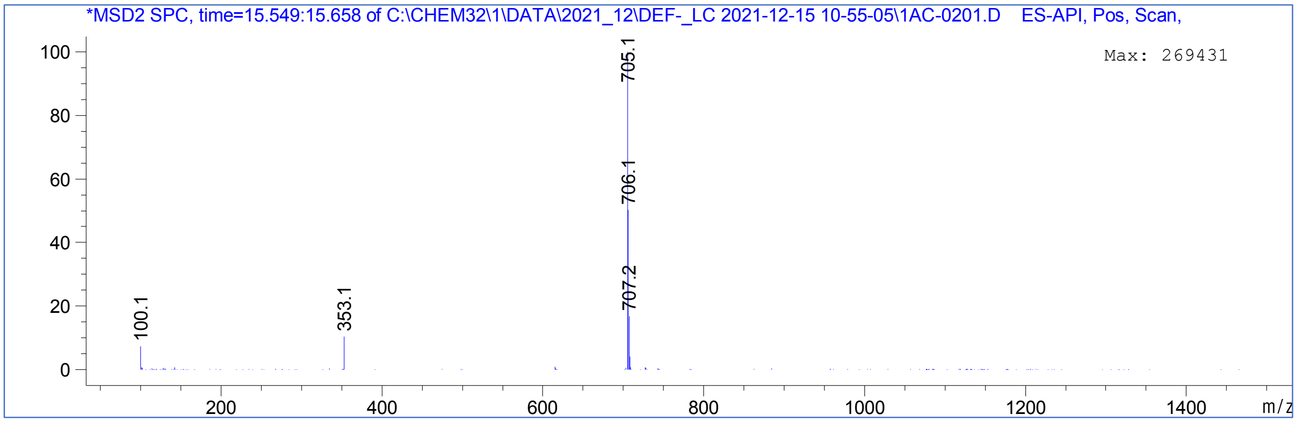
*MSD2 SPC, time=15.549:15.658 of C:\CHEM32\1\DATA\2021_12\DEF-_LC 2021-12-15 10-55-05\1AC-0201.D ES-API, Pos, Scan,
Max: 269431
100
80
60
40
20
0
705.1
706.1
707.2
100.1
353.1
200
400
600
800
1000
1200
1400
m/z

Origin	Bruker BioSpin GmbH
Solvent	MeOD
Temperature	295.3
Pulse Sequence	zg30
Experiment	1D
Number of Scans	16
Acquisition Date	2021-09-22T15:30:00
Spectrometer Frequency	400.13
Spectral Width	8012.8
Lowest Frequency	-1543.2
Nucleus	1H
Acquired Size	32768
Spectral Size	65536

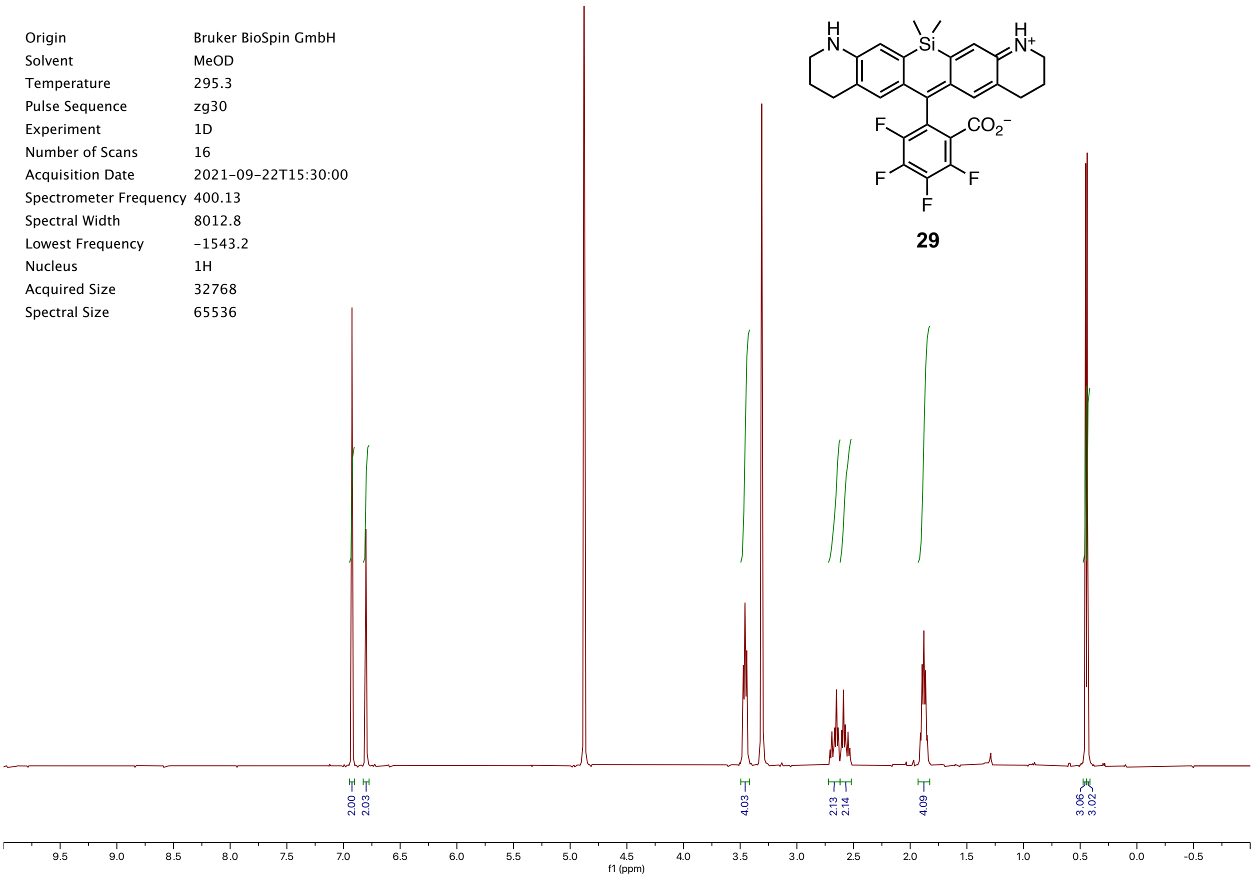

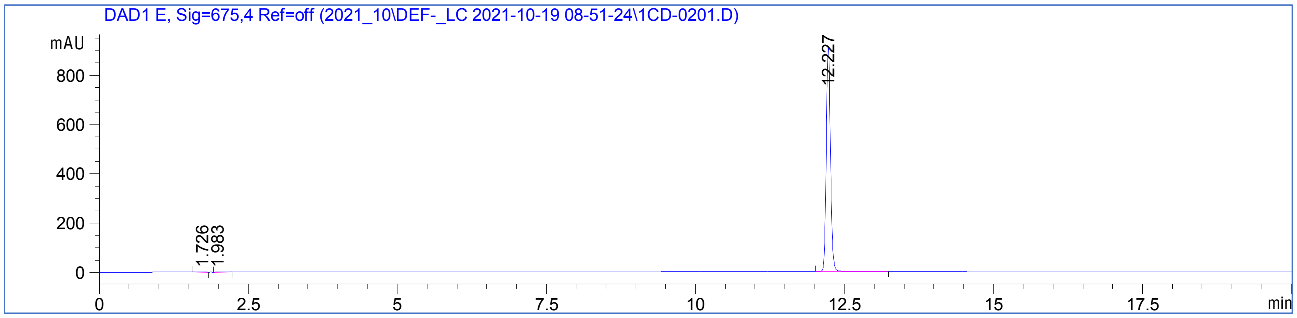

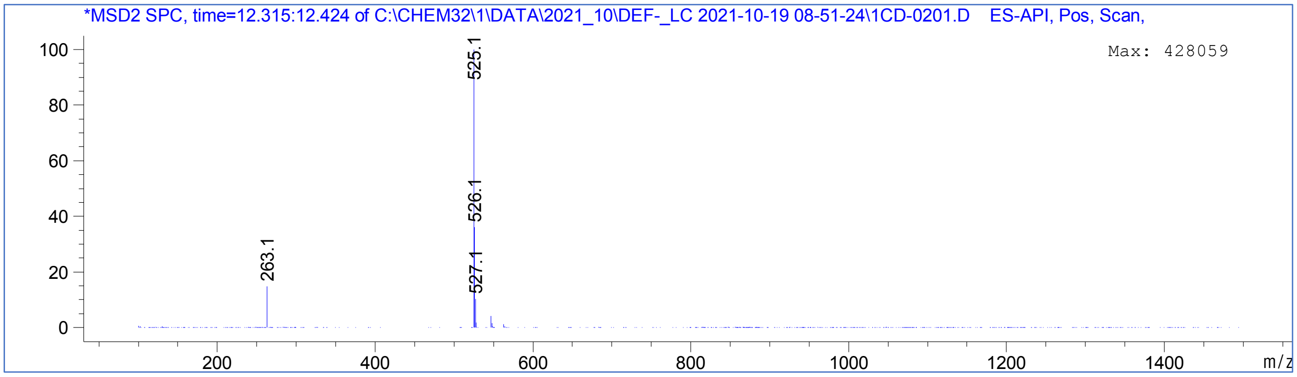

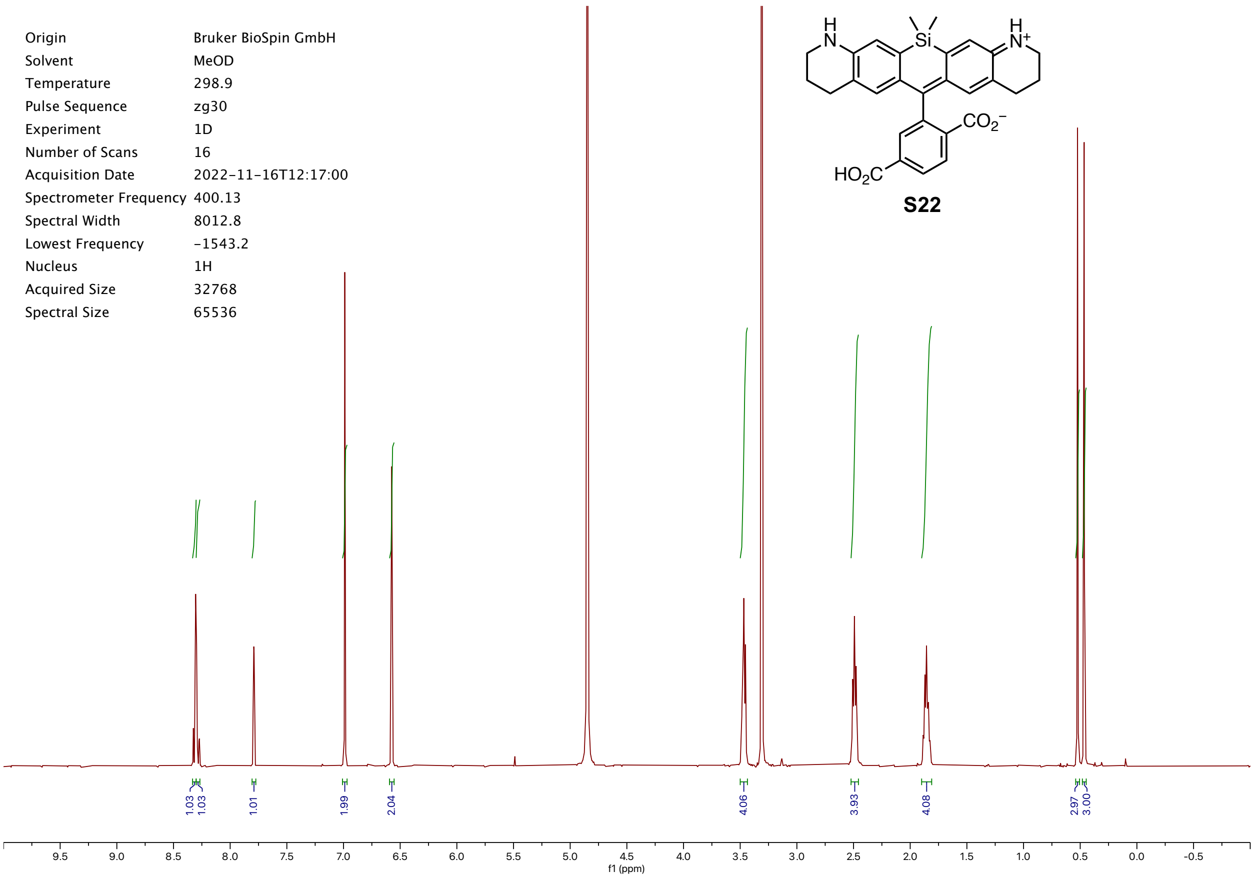
Origin Bruker BioSpin GmbH
Solvent MeOD
Temperature 298.9
Pulse Sequence zg30
Experiment 1D
Number of Scans 16
Acquisition Date 2022-11-16T12:17:00
Spectrometer Frequency 400.13
Spectral Width 8012.8
Lowest Frequency -1543.2
Nucleus 1H
Acquired Size 32768
Spectral Size 65536
S22
1.03
1.03
1.01
1.99
2.04
4.06
3.93
4.08
2.97
3.00
f1 (ppm)

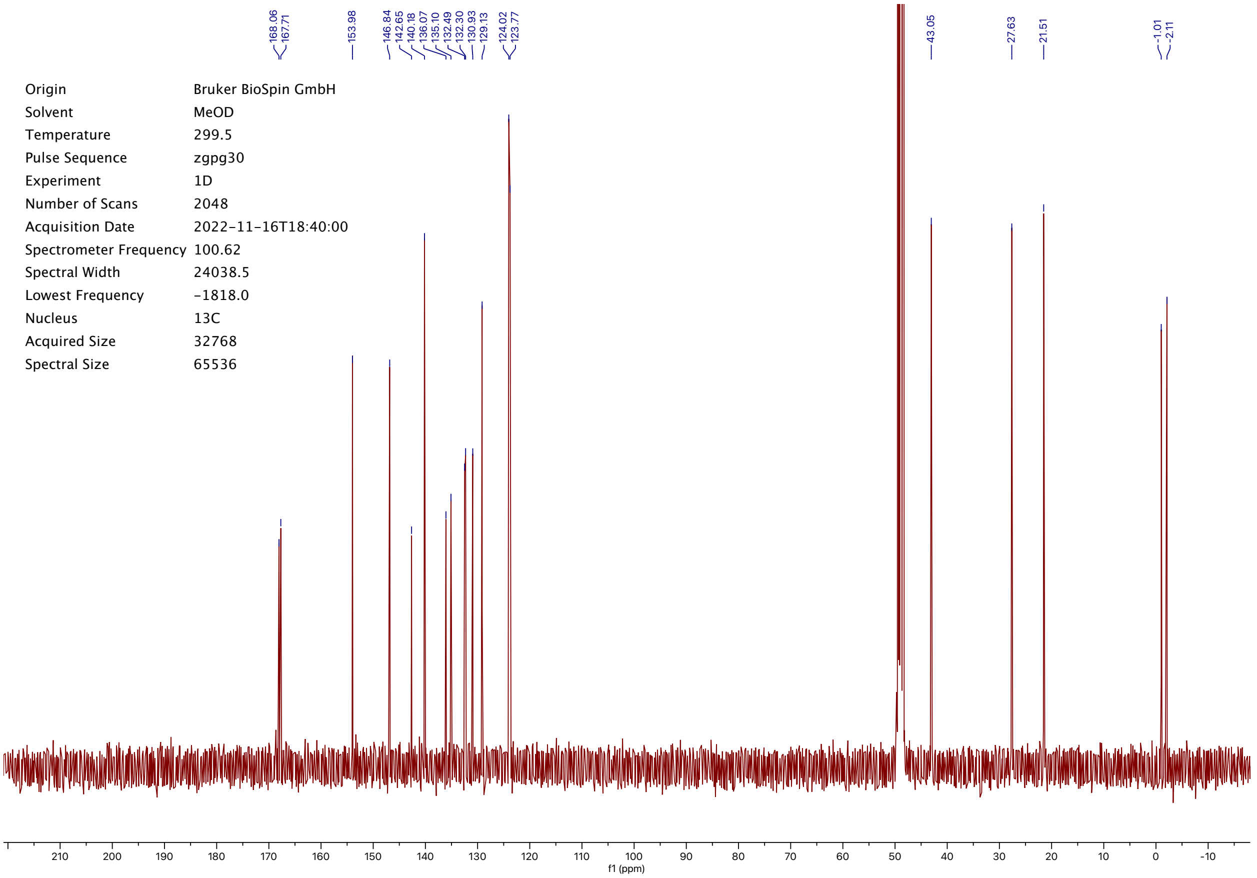
168.06
167.71
153.98
146.84
142.65
140.18
136.07
135.10
132.49
132.30
130.93
129.13
124.02
123.77
43.05
27.63
21.51
-1.01
-2.11
Origin Bruker BioSpin GmbH
Solvent MeOD
Temperature 299.5
Pulse Sequence zgpg30
Experiment 1D
Number of Scans 2048
Acquisition Date 2022-11-16T18:40:00
Spectrometer Frequency 100.62
Spectral Width 24038.5
Lowest Frequency -1818.0
Nucleus 13C
Acquired Size 32768
Spectral Size 65536
f1 (ppm)

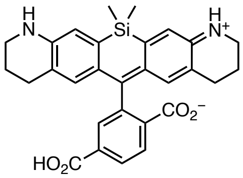

S22

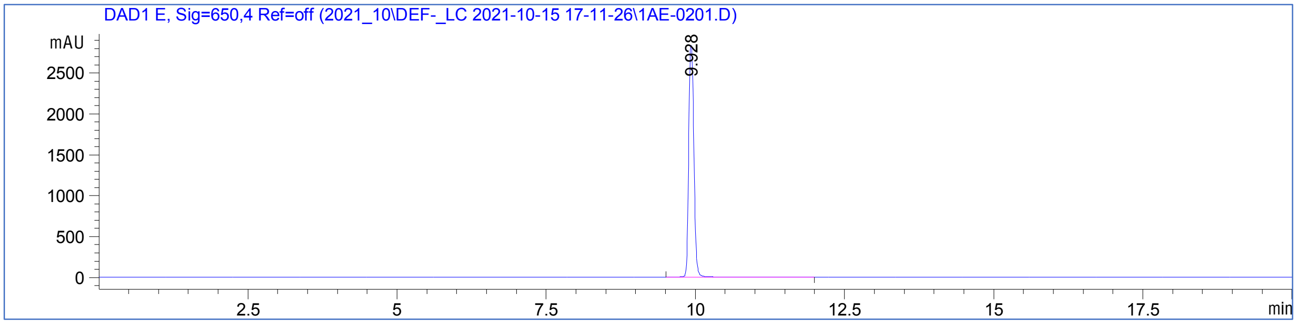
DAD1 E, Sig=650,4 Ref=off (2021_10\DEF-_LC 2021-10-15 17-11-26\1AE-0201.D)
mAU
2500
2000
1500
1000
500
0
9.928
2.5
5
7.5
10
12.5
15
17.5
min

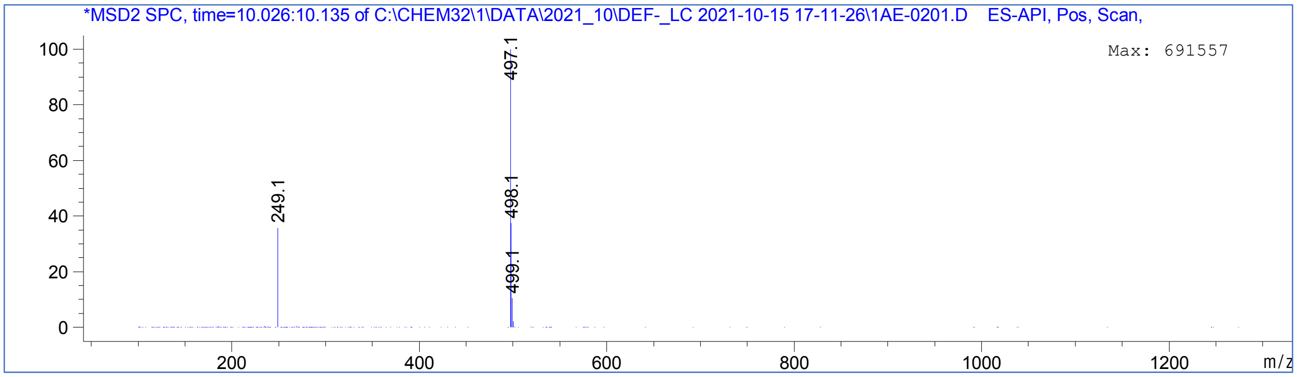
*MSD2 SPC, time=10.026:10.135 of C:\CHEM32\1\DATA\2021_10\DEF-_LC 2021-10-15 17-11-26\1AE-0201.D ES-API, Pos, Scan,
Max: 691557
100
80
60
40
20
0
497.1
249.1
498.1
499.1
200
400
600
800
1000
1200
m/z

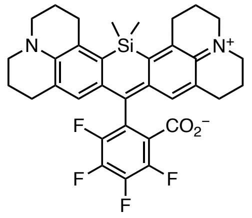

F
CO_2^-
F
F
F
Si
N
N+

31

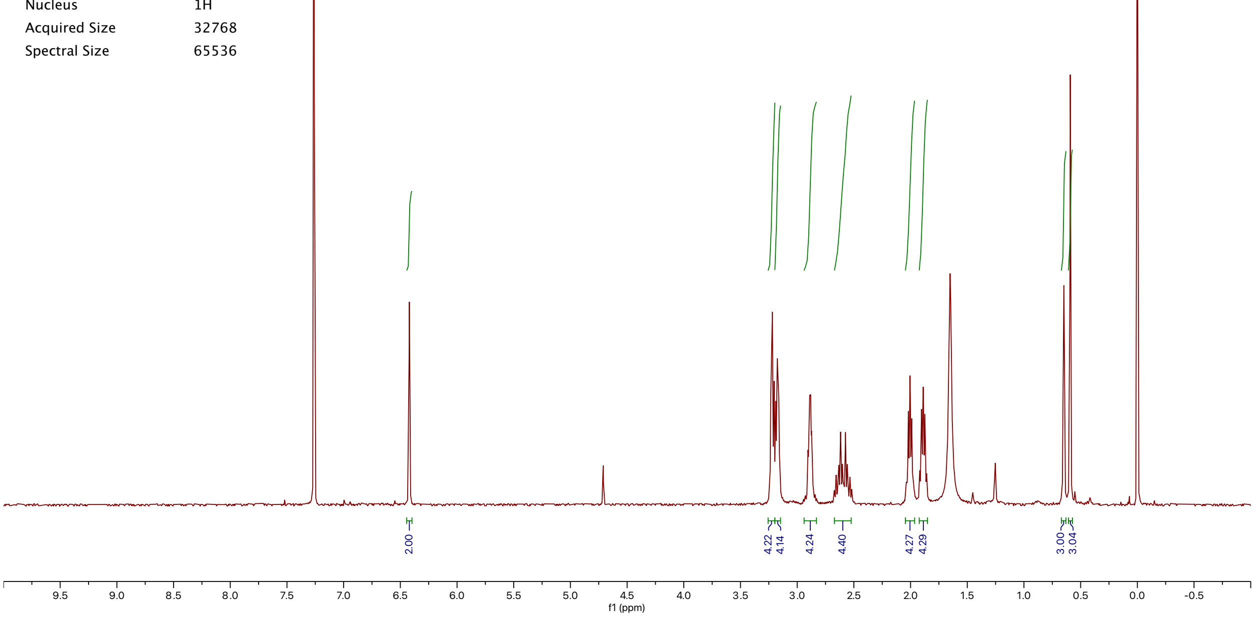

Origin Bruker BioSpin GmbH
Solvent CDCl3
Temperature 295.3
Pulse Sequence zg30
Experiment 1D
Number of Scans 16
Acquisition Date 2021-09-29T17:32:00
Spectrometer Frequency 400.13
Spectral Width 8012.8
Lowest Frequency -1544.9
Nucleus 1H
Acquired Size 32768
Spectral Size 65536
2.00
4.22
4.14
4.24
4.40
4.27
4.29
3.00
3.04
9.5 9.0 8.5 8.0 7.5 7.0 6.5 6.0 5.5 5.0 4.5 4.0 3.5 3.0 2.5 2.0 1.5 1.0 0.5 0.0 -0.5
f1 (ppm)

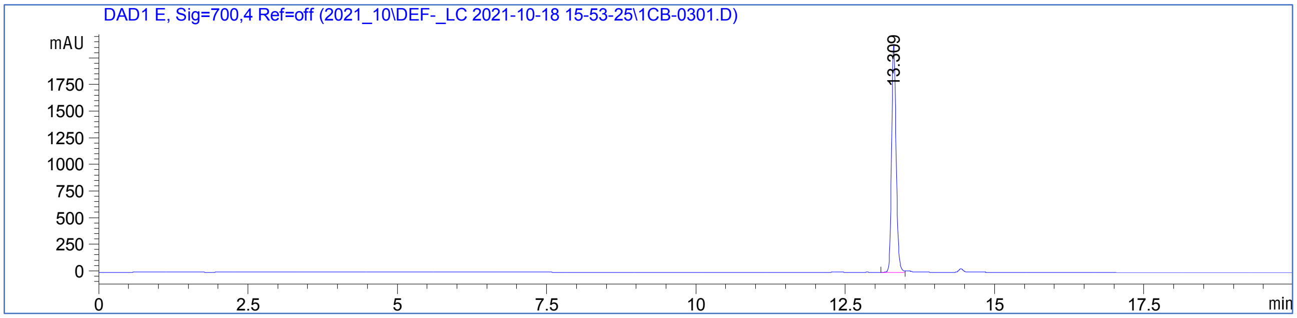

DAD1 E, Sig=700,4 Ref=off (2021_10\DEF-_LC 2021-10-18 15-53-25\1CB-0301.D)
mAU
13.309
1750
1500
1250
1000
750
500
250
0
0 2.5 5 7.5 10 12.5 15 17.5 min

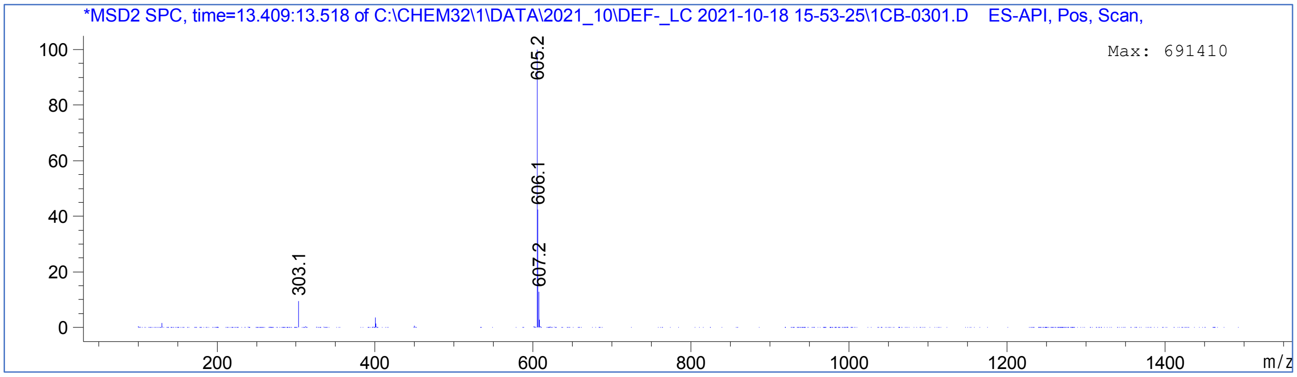

*MSD2 SPC, time=13.409:13.518 of C:\CHEM32\1\DATA\2021_10\DEF-_LC 2021-10-18 15-53-25\1CB-0301.D ES-API, Pos, Scan,
Max: 691410
605.2
606.1
607.2
303.1
100
80
60
40
20
0
200 400 600 800 1000 1200 1400 m/z

Origin	Bruker BioSpin GmbH
Solvent	MeOD
Temperature	300.0
Pulse Sequence	zg30
Experiment	1D
Number of Scans	16
Acquisition Date	2021-11-08T10:17:00
Spectrometer Frequency	400.13
Spectral Width	8012.8
Lowest Frequency	-1543.1
Nucleus	1H
Acquired Size	32768
Spectral Size	65536

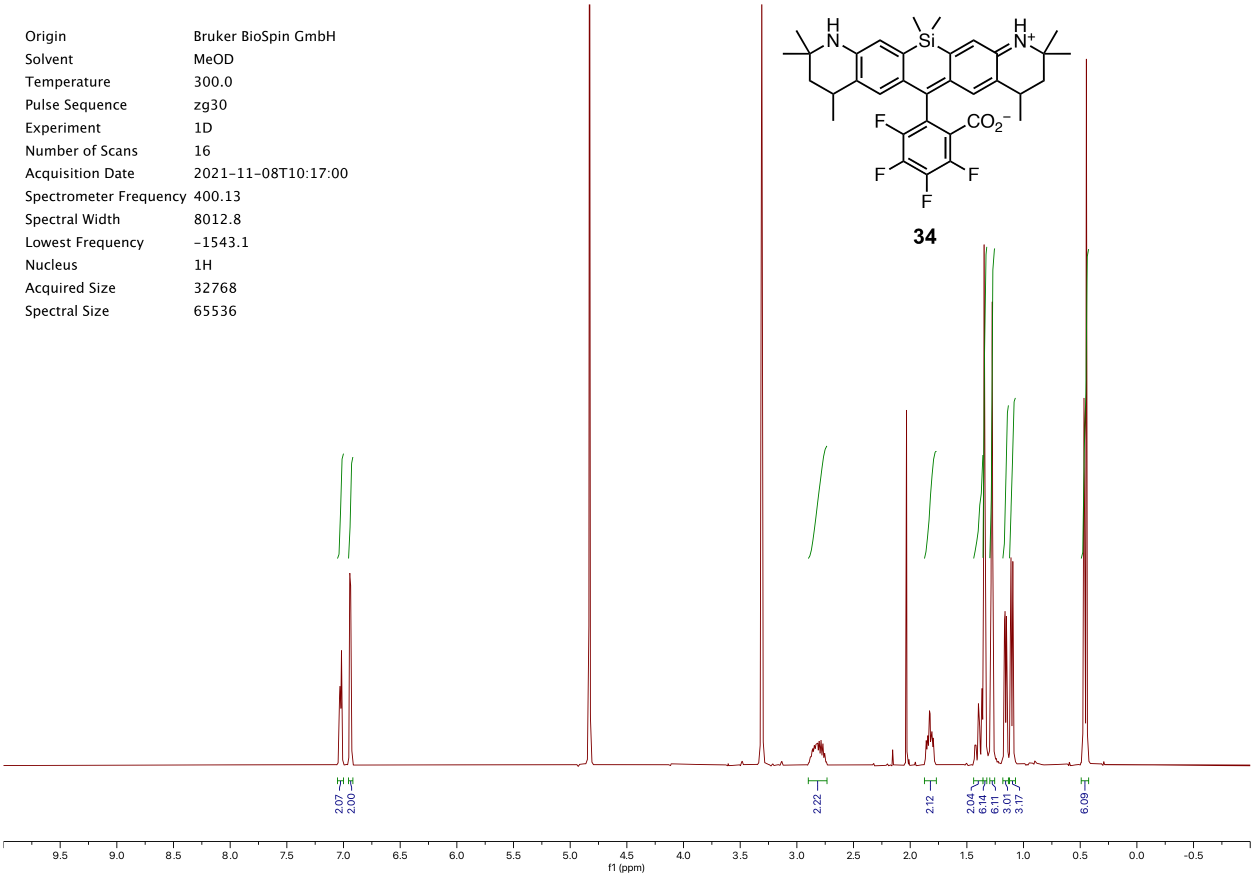

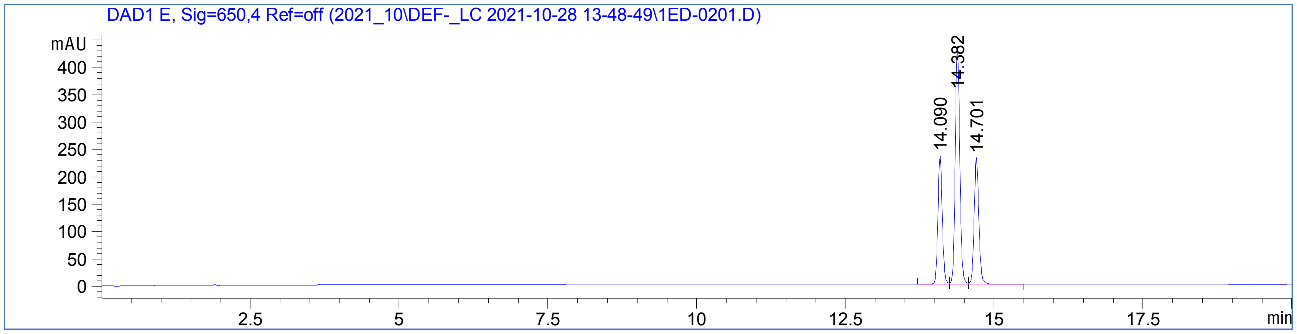

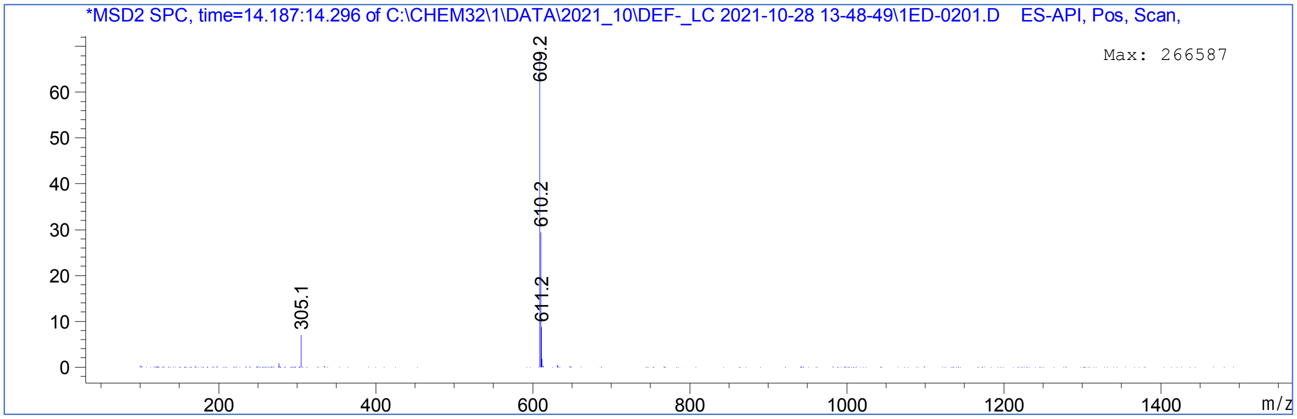

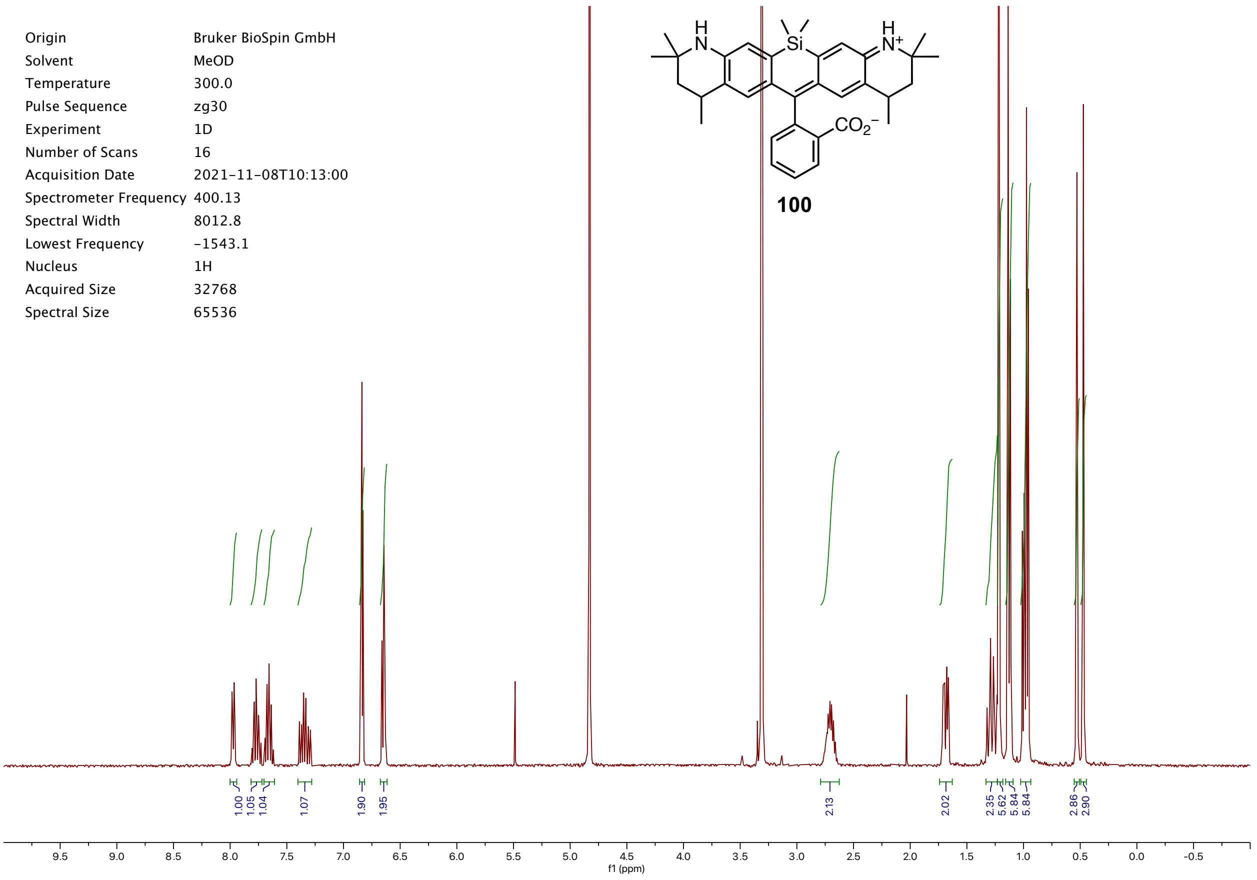
Origin Bruker BioSpin GmbH
Solvent MeOD
Temperature 300.0
Pulse Sequence zg30
Experiment 1D
Number of Scans 16
Acquisition Date 2021-11-08T10:13:00
Spectrometer Frequency 400.13
Spectral Width 8012.8
Lowest Frequency -1543.1
Nucleus 1H
Acquired Size 32768
Spectral Size 65536
100
1.00 1.05 1.04 1.07 1.90 1.95 2.13 2.02 2.35 5.62 5.84 5.84 2.86 2.90
f1 (ppm)

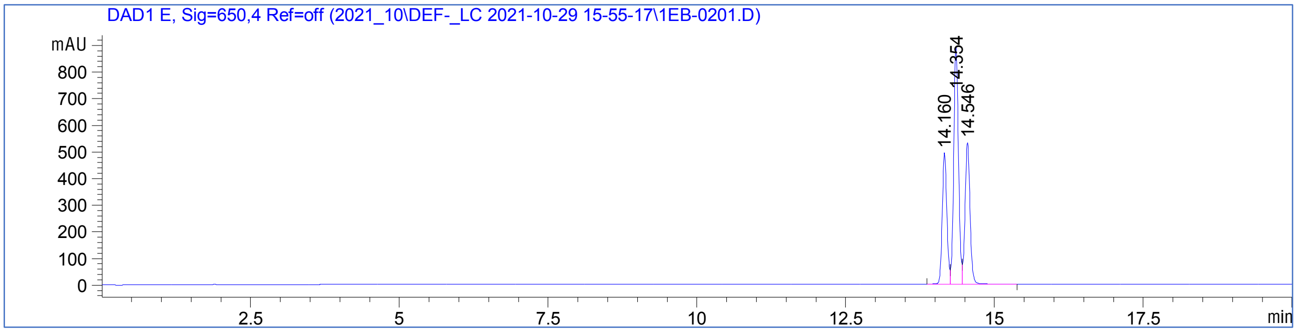
DAD1 E, Sig=650,4 Ref=off (2021_10\DEF-_LC 2021-10-29 15-55-17\1EB-0201.D)
mAU
14.160
14.354
14.546
min

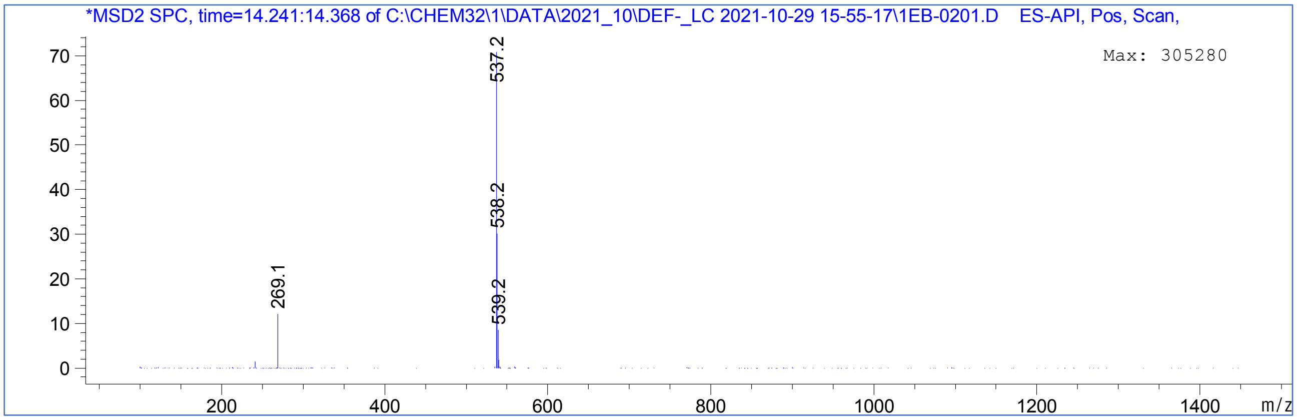
*MSD2 SPC, time=14.241:14.368 of C:\CHEM32\1\DATA\2021_10\DEF-_LC 2021-10-29 15-55-17\1EB-0201.D ES-API, Pos, Scan,
Max: 305280
537.2
538.2
539.2
269.1
m/z

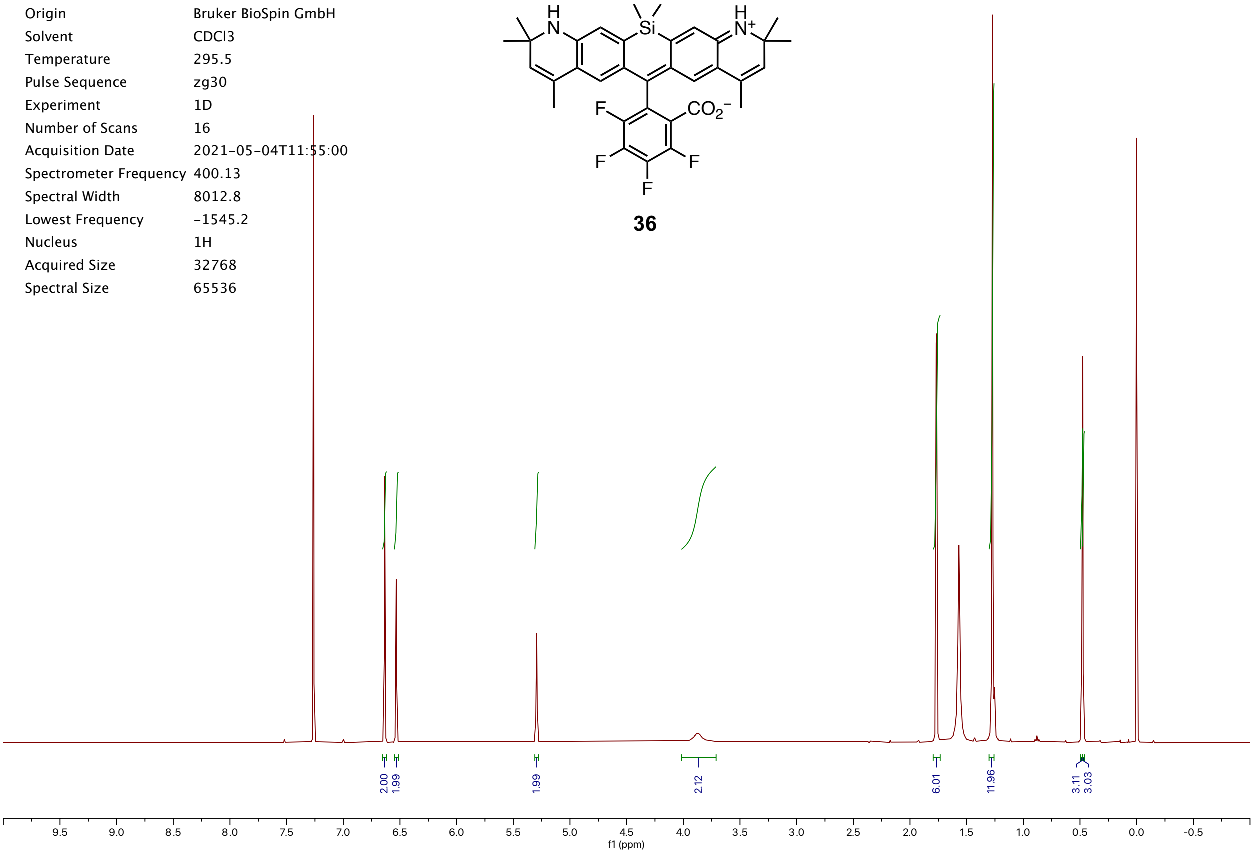
Origin Bruker BioSpin GmbH
Solvent CDCl3
Temperature 295.5
Pulse Sequence zg30
Experiment 1D
Number of Scans 16
Acquisition Date 2021-05-04T11:55:00
Spectrometer Frequency 400.13
Spectral Width 8012.8
Lowest Frequency -1545.2
Nucleus 1H
Acquired Size 32768
Spectral Size 65536
36
2.00
1.99
1.99
2.12
6.01
11.96
3.11
3.03
f1 (ppm)

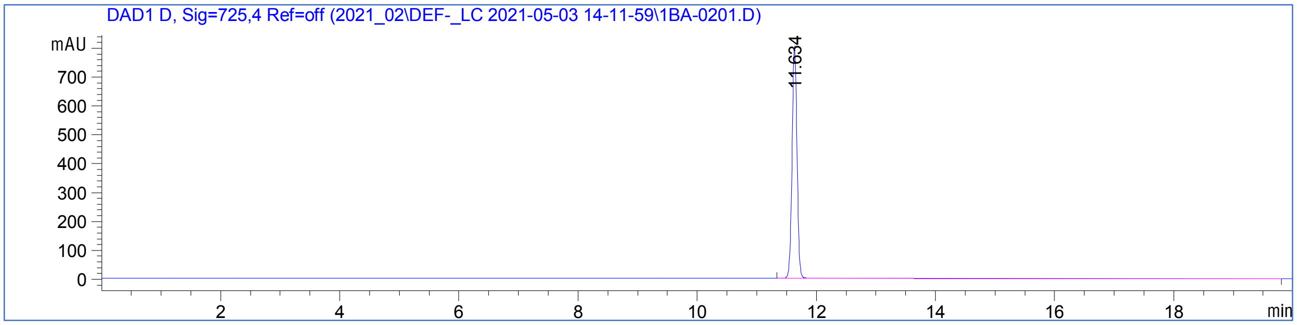
DAD1 D, Sig=725,4 Ref=off (2021_02\DEF-_LC 2021-05-03 14-11-59\1BA-0201.D)
mAU
11.634
min

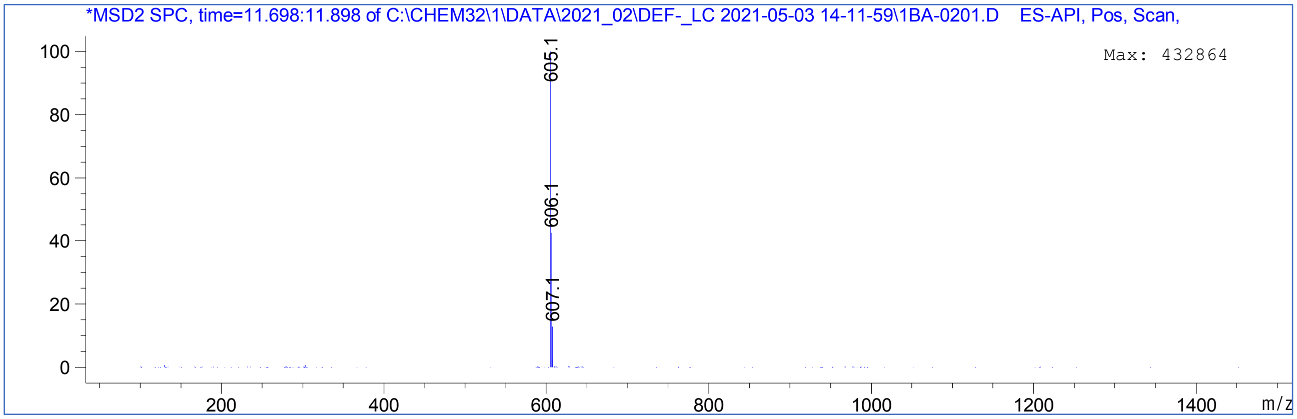
*MSD2 SPC, time=11.698:11.898 of C:\CHEM32\1\DATA\2021_02\DEF-_LC 2021-05-03 14-11-59\1BA-0201.D ES-API, Pos, Scan,
Max: 432864
605.1
606.1
607.1
m/z

Origin	Bruker BioSpin GmbH
Solvent	CDCl3
Temperature	295.4
Pulse Sequence	zg30
Experiment	1D
Number of Scans	16
Acquisition Date	2021-10-25T14:53:00
Spectrometer Frequency	400.13
Spectral Width	8012.8
Lowest Frequency	-1544.5
Nucleus	1H
Acquired Size	32768
Spectral Size	65536

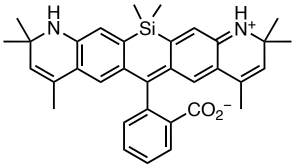

102

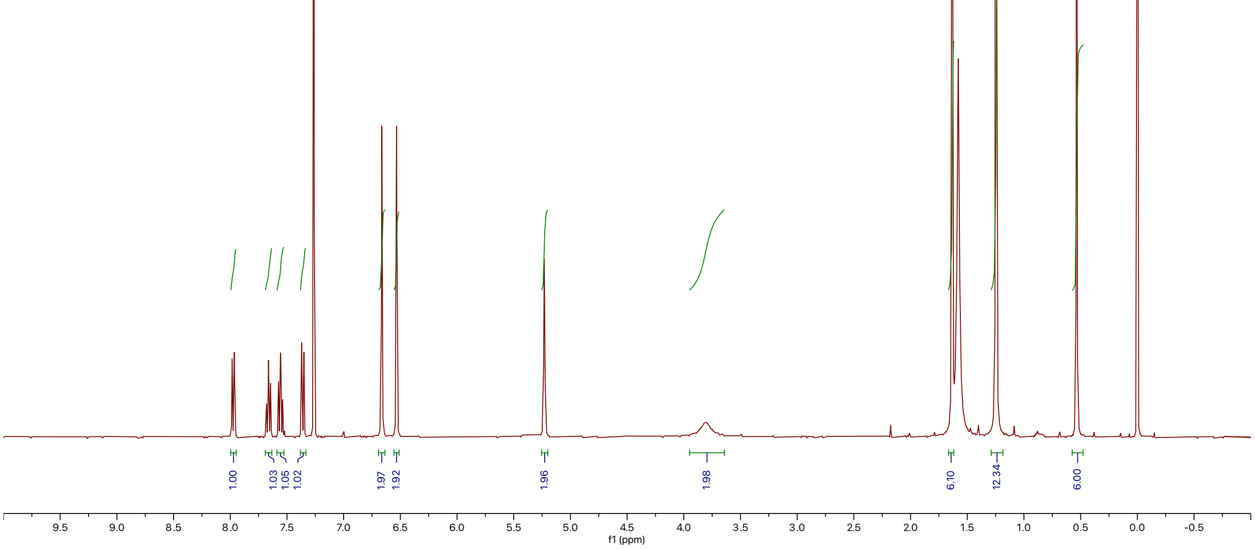

Origin	Bruker BioSpin GmbH
Solvent	CDCl3
Temperature	296.2
Pulse Sequence	zgpg30
Experiment	1D
Number of Scans	4096
Acquisition Date	2021-10-25T22:02:00
Spectrometer Frequency	100.62
Spectral Width	24038.5
Lowest Frequency	-1946.2
Nucleus	13C
Acquired Size	32768
Spectral Size	65536

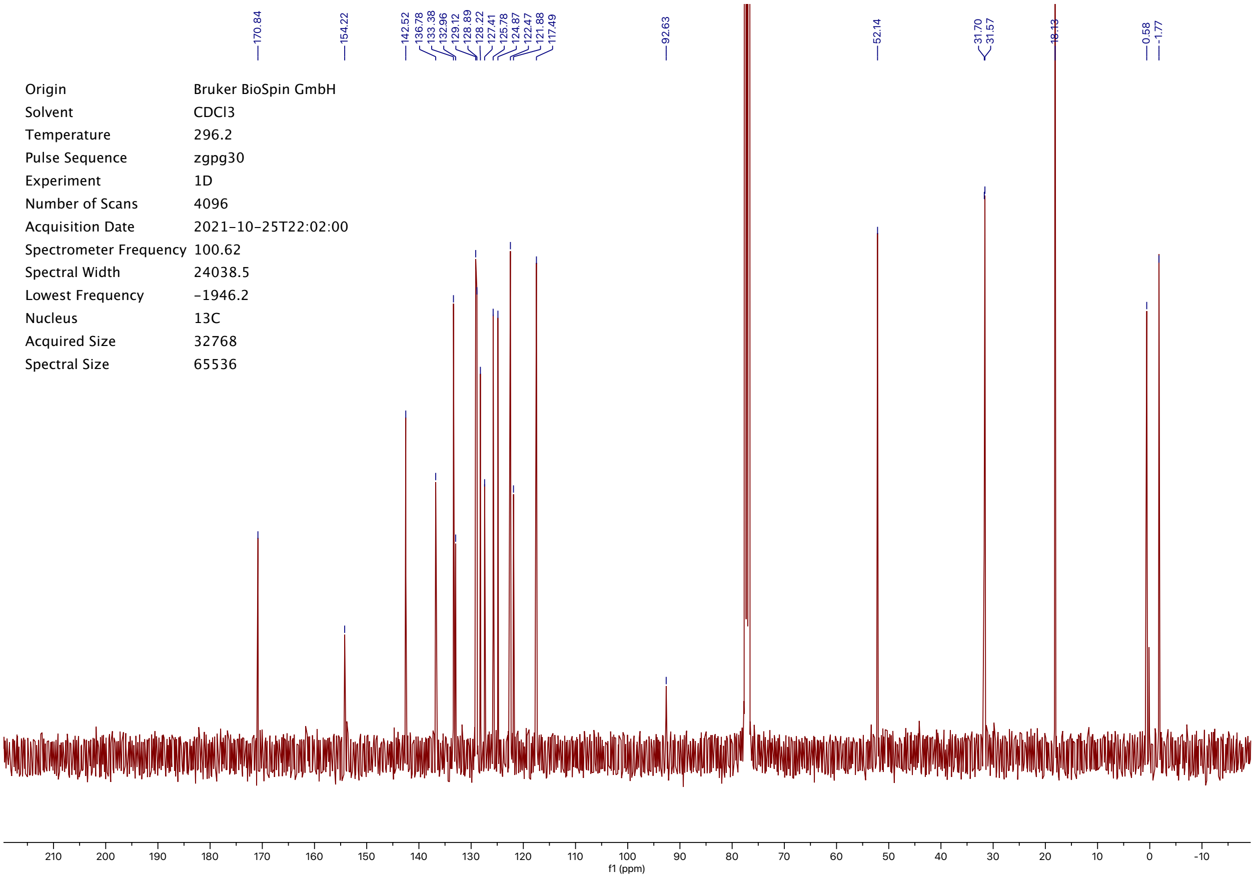

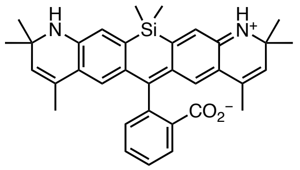
H
N
Si
H
N+
CO2−

102

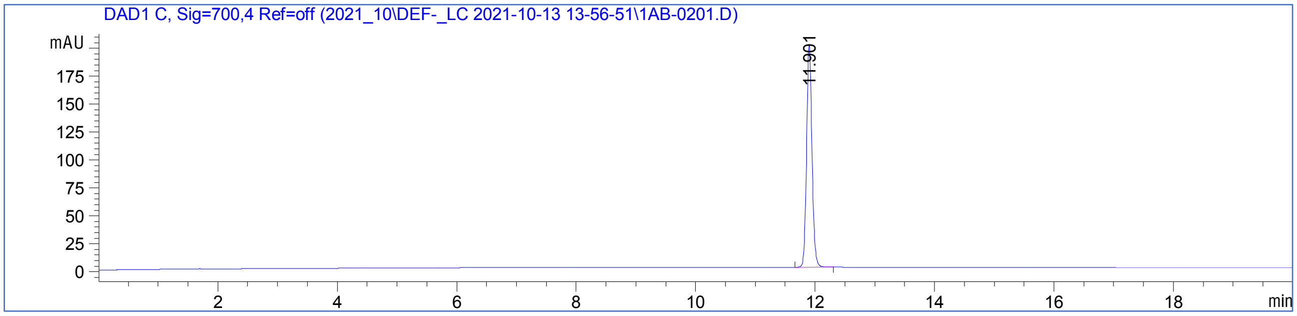
DAD1 C, Sig=700,4 Ref=off (2021_10\DEF-_LC 2021-10-13 13-56-51\1AB-0201.D)
mAU
175
150
125
100
75
50
25
0
11.901
2
4
6
8
10
12
14
16
18
min

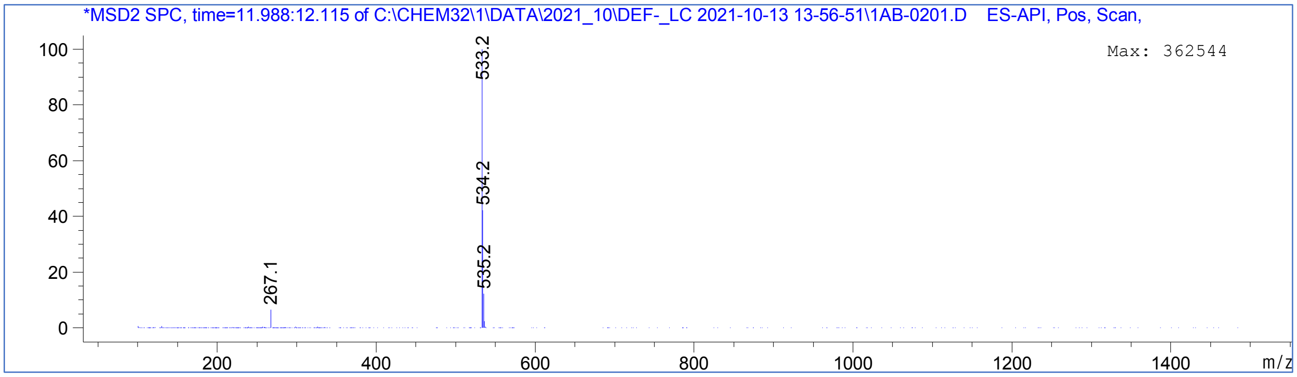
*MSD2 SPC, time=11.988:12.115 of C:\CHEM32\1\DATA\2021_10\DEF-_LC 2021-10-13 13-56-51\1AB-0201.D ES-API, Pos, Scan,
Max: 362544
100
80
60
40
20
0
533.2
534.2
535.2
267.1
200
400
600
800
1000
1200
1400
m/z

Origin	Bruker BioSpin GmbH
Solvent	CDCl3
Temperature	295.5
Pulse Sequence	zg30
Experiment	1D
Number of Scans	16
Acquisition Date	2020-12-17T12:34:00
Spectrometer Frequency	400.13
Spectral Width	8012.8
Lowest Frequency	-1545.3
Nucleus	1H
Acquired Size	32768
Spectral Size	65536

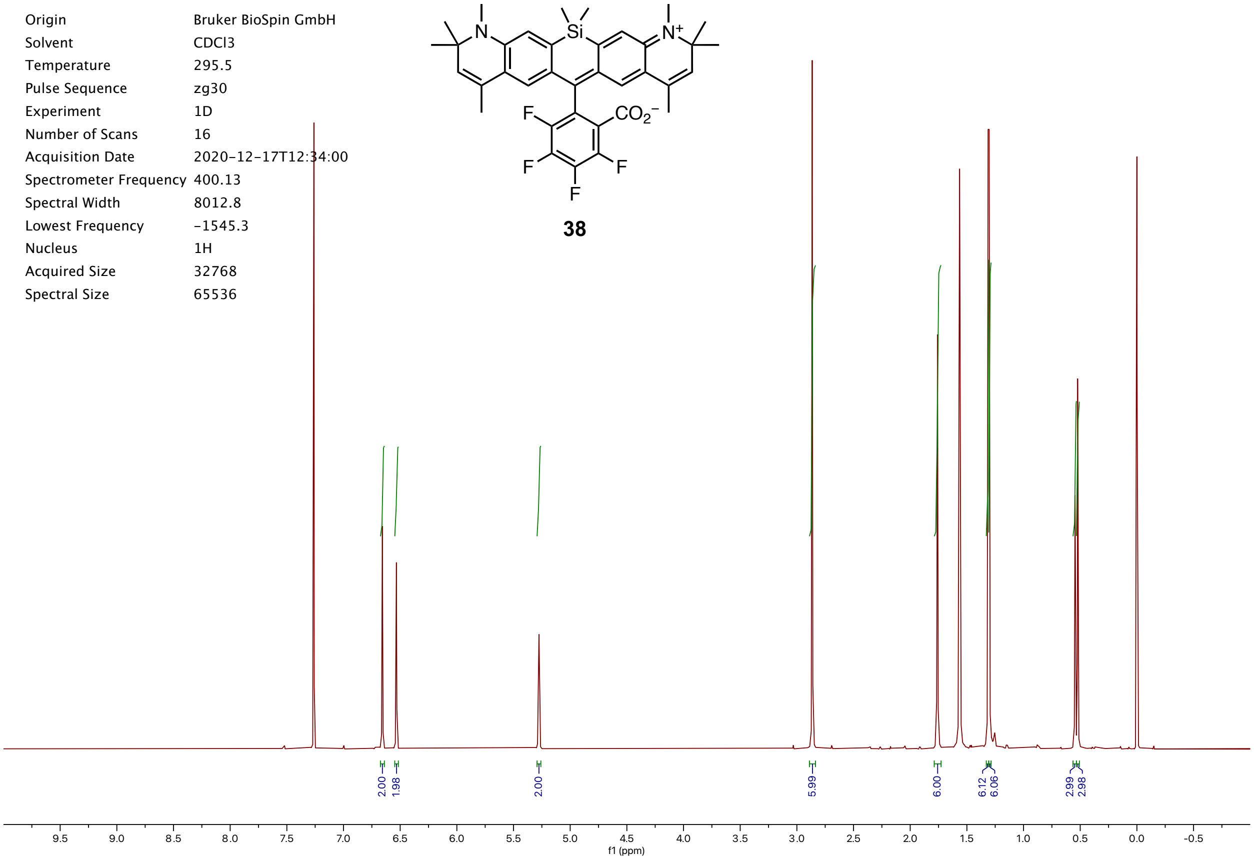

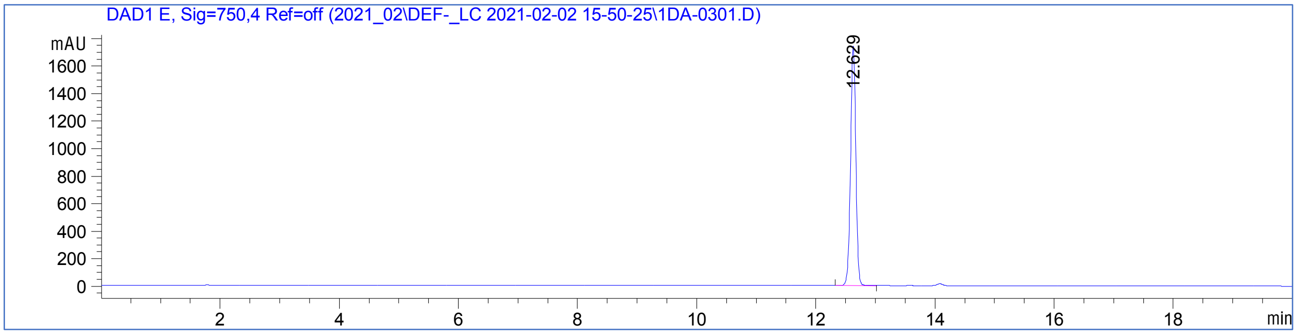

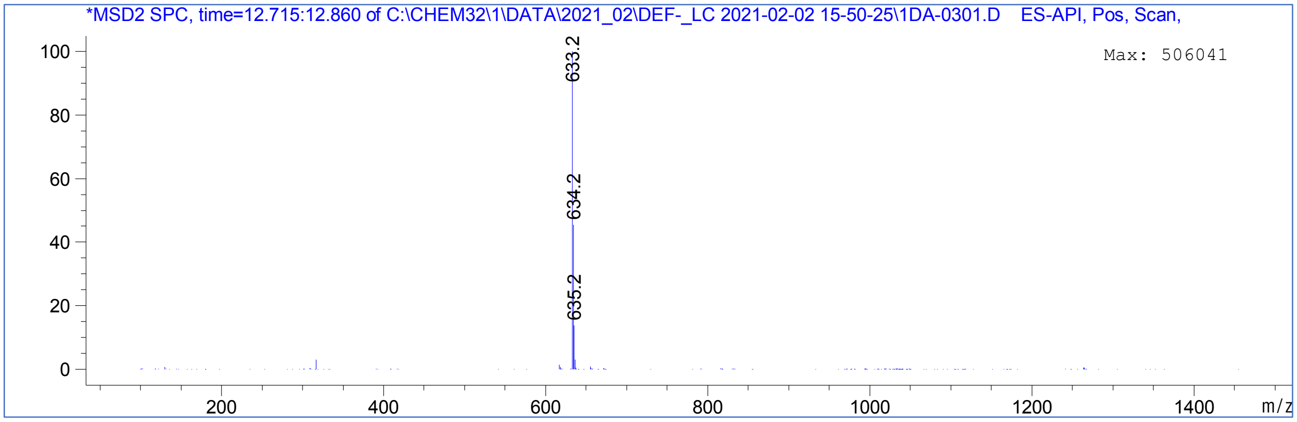

Origin	Bruker BioSpin GmbH
Solvent	CDCl3
Temperature	300.0
Pulse Sequence	zg30
Experiment	1D
Number of Scans	16
Acquisition Date	2021-01-11T10:28:00
Spectrometer Frequency	400.13
Spectral Width	8012.8
Lowest Frequency	-1546.1
Nucleus	1H
Acquired Size	32768
Spectral Size	65536

104

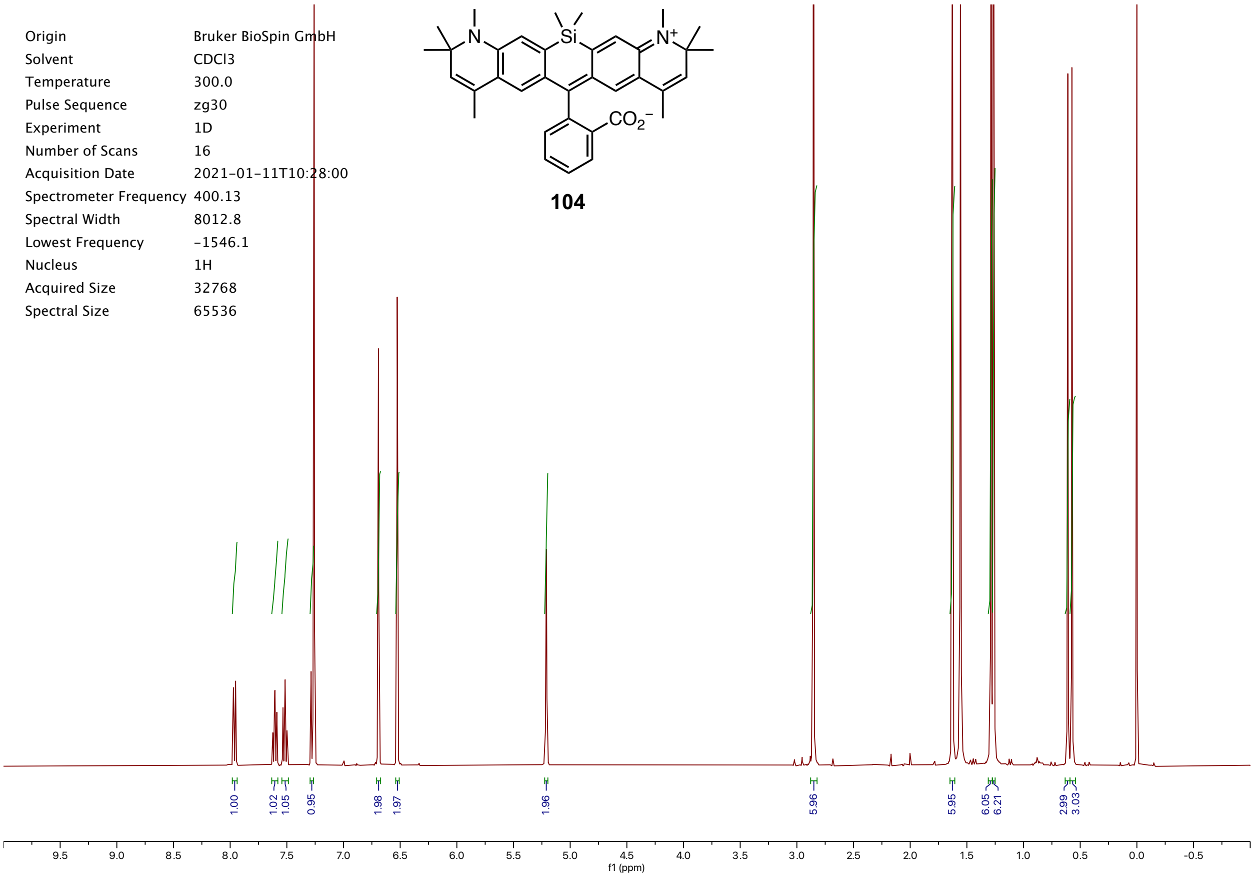

Origin	Bruker BioSpin GmbH
Solvent	CDCl3
Temperature	300.0
Pulse Sequence	zgpg30
Experiment	1D
Number of Scans	2048
Acquisition Date	2021-01-11T18:58:00
Spectrometer Frequency	100.62
Spectral Width	24038.5
Lowest Frequency	-1946.2
Nucleus	13C
Acquired Size	32768
Spectral Size	65536

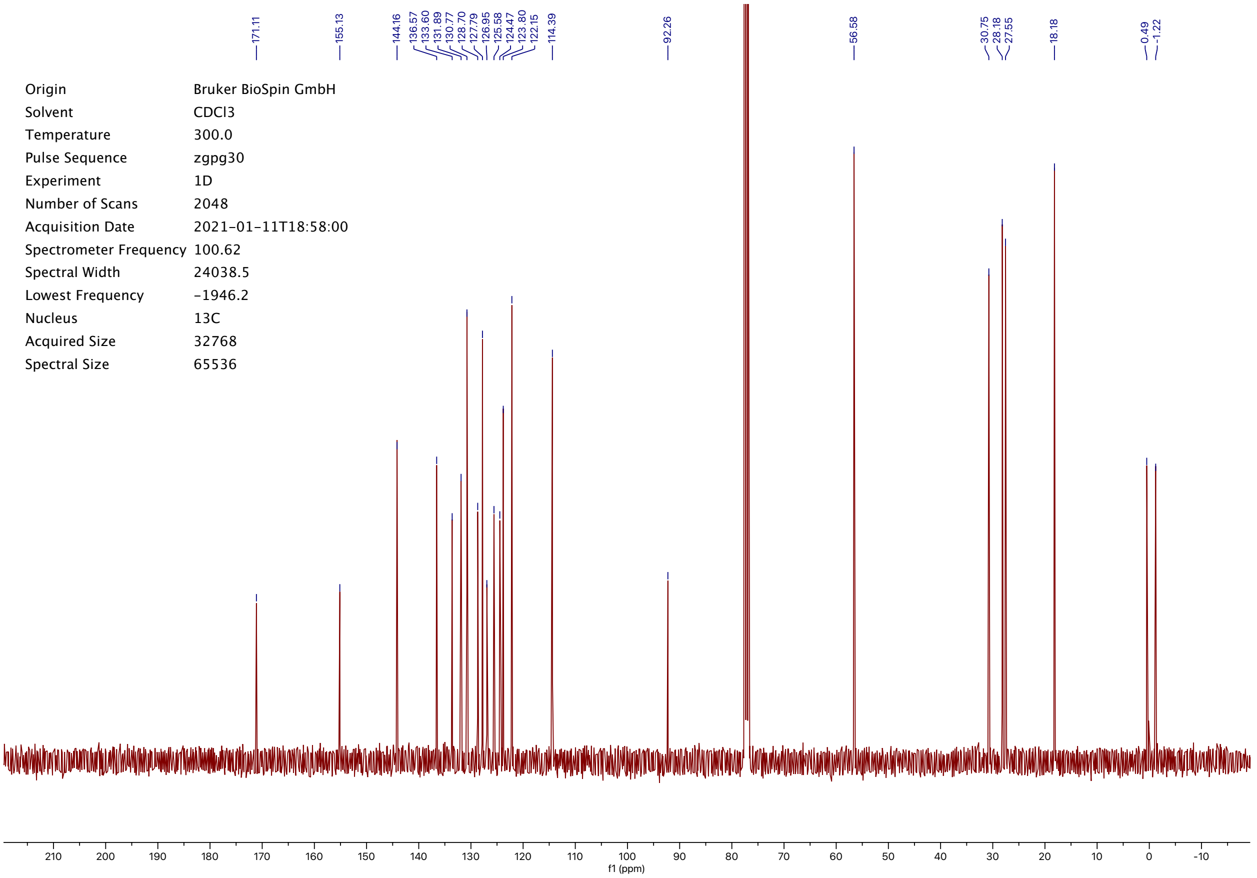

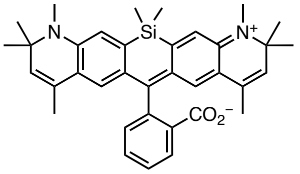
N
Si
N+
CO2−

104

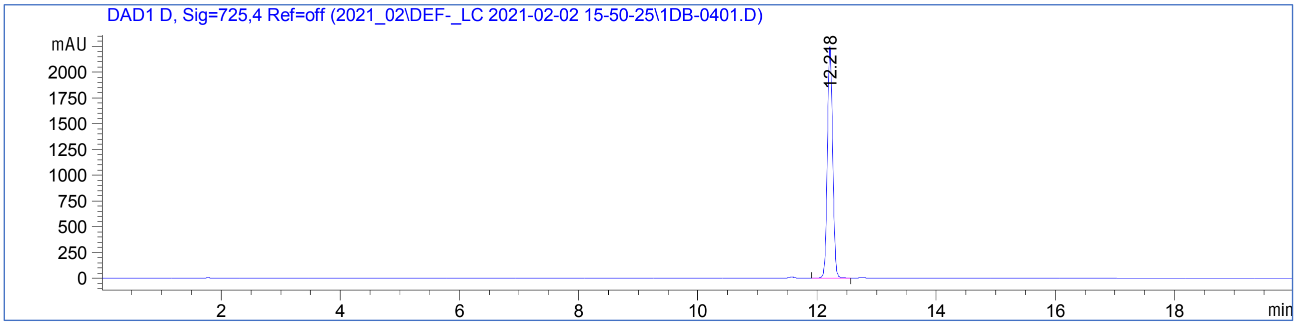
DAD1 D, Sig=725,4 Ref=off (2021_02\DEF-_LC 2021-02-02 15-50-25\1DB-0401.D)
mAU
2000
1750
1500
1250
1000
750
500
250
0
12.218
2
4
6
8
10
12
14
16
18
min

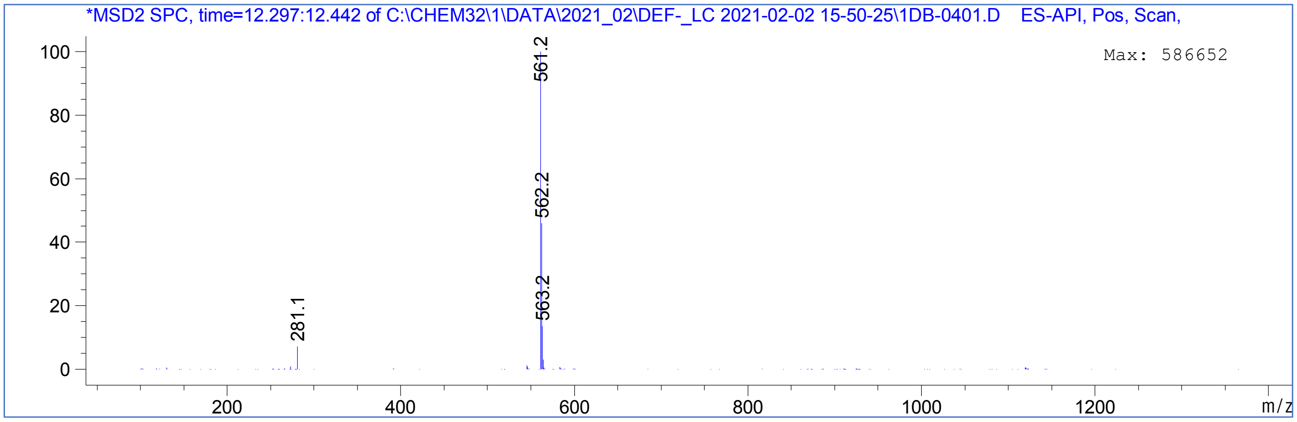
*MSD2 SPC, time=12.297:12.442 of C:\CHEM32\1\DATA\2021_02\DEF-_LC 2021-02-02 15-50-25\1DB-0401.D ES-API, Pos, Scan,
Max: 586652
100
80
60
40
20
0
561.2
562.2
563.2
281.1
200
400
600
800
1000
1200
m/z

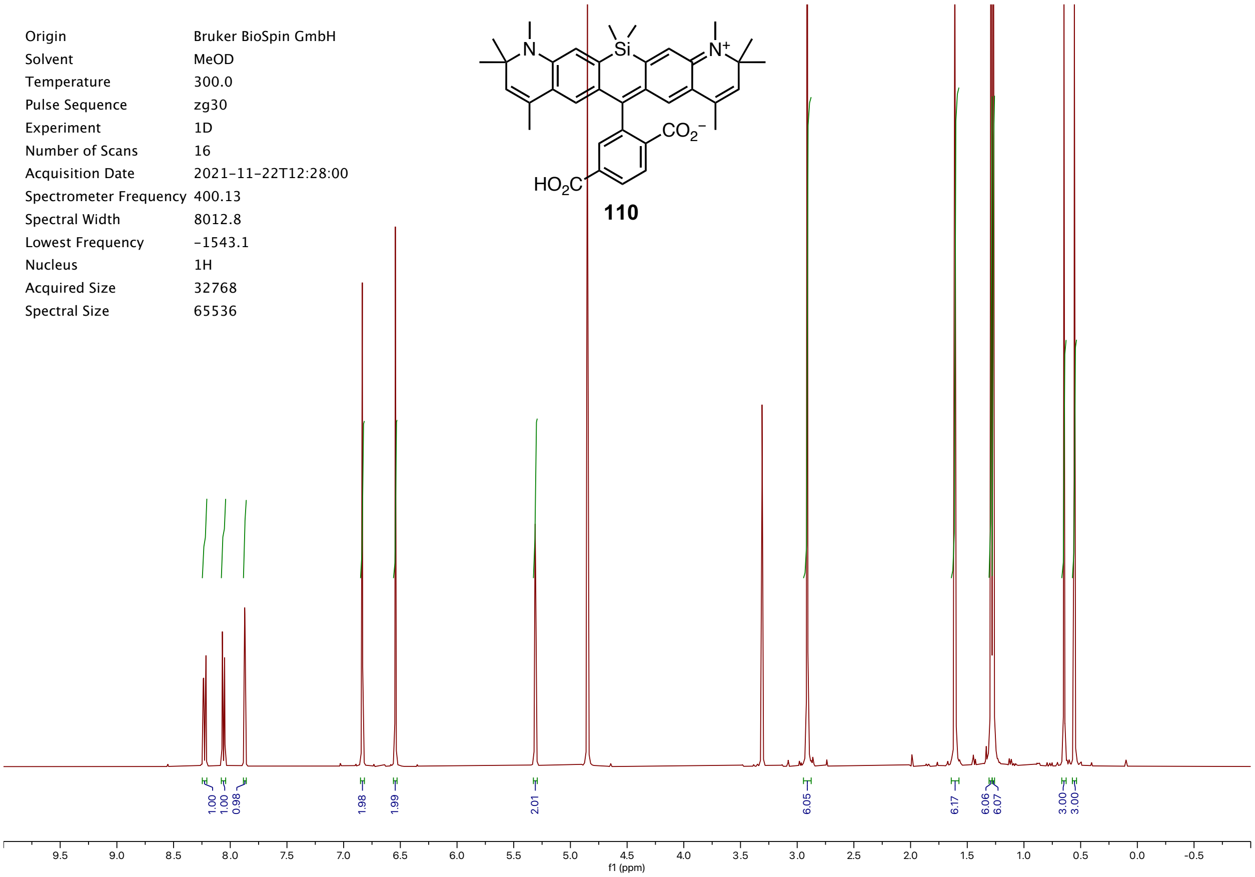
Origin Bruker BioSpin GmbH
Solvent MeOD
Temperature 300.0
Pulse Sequence zg30
Experiment 1D
Number of Scans 16
Acquisition Date 2021-11-22T12:28:00
Spectrometer Frequency 400.13
Spectral Width 8012.8
Lowest Frequency -1543.1
Nucleus 1H
Acquired Size 32768
Spectral Size 65536
N
Si
N+
CO2−
HO2C
110
1.00
1.00
0.98
1.98
1.99
2.01
6.05
6.17
6.06
6.07
3.00
3.00
f1 (ppm)

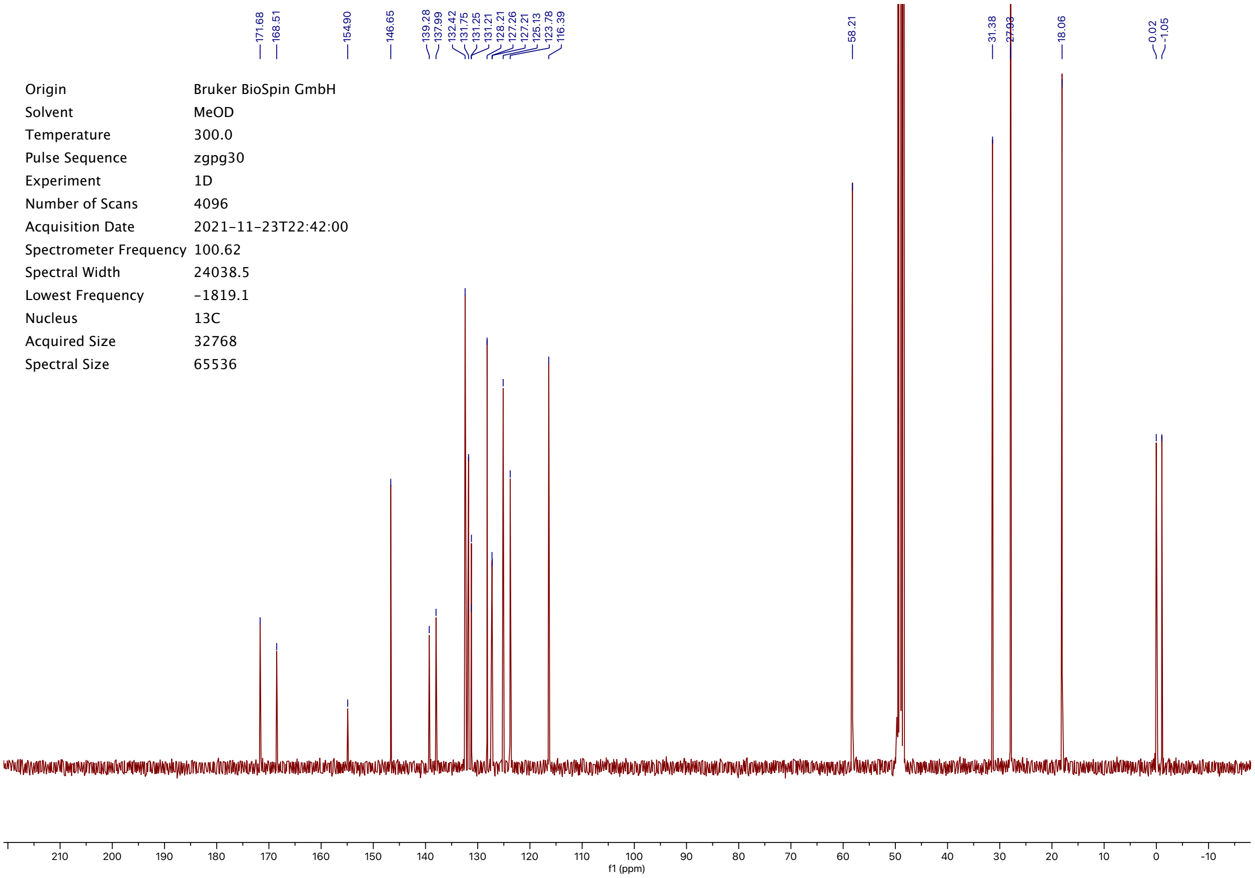
171.68
168.51
154.90
146.65
139.28
137.99
132.42
131.75
131.25
131.21
128.21
127.26
127.21
125.13
123.78
116.39
58.21
31.38
27.93
18.06
0.02
-1.05
Origin Bruker BioSpin GmbH
Solvent MeOD
Temperature 300.0
Pulse Sequence zgpg30
Experiment 1D
Number of Scans 4096
Acquisition Date 2021-11-23T22:42:00
Spectrometer Frequency 100.62
Spectral Width 24038.5
Lowest Frequency -1819.1
Nucleus 13C
Acquired Size 32768
Spectral Size 65536
f1 (ppm)

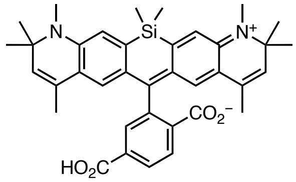
N
Si
N+
CO2−
HO2C

110

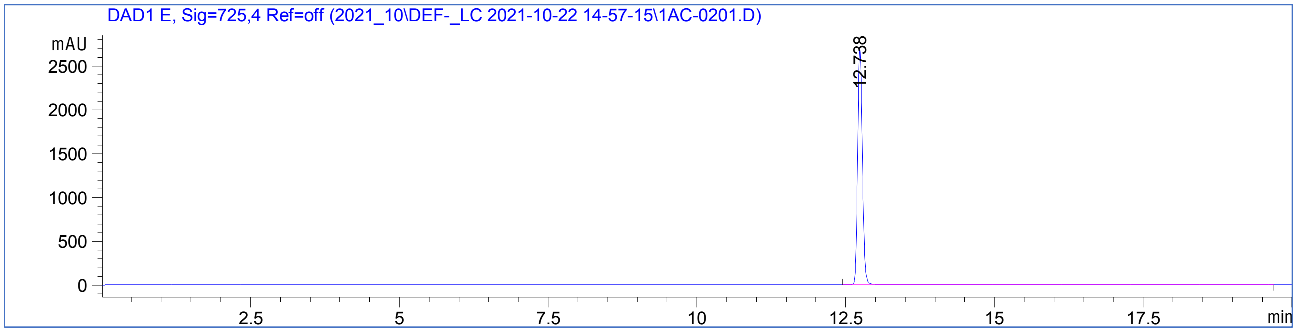
DAD1 E, Sig=725,4 Ref=off (2021_10\DEF-_LC 2021-10-22 14-57-15\1AC-0201.D)
mAU
2500
2000
1500
1000
500
0
12.738
2.5
5
7.5
10
12.5
15
17.5
min

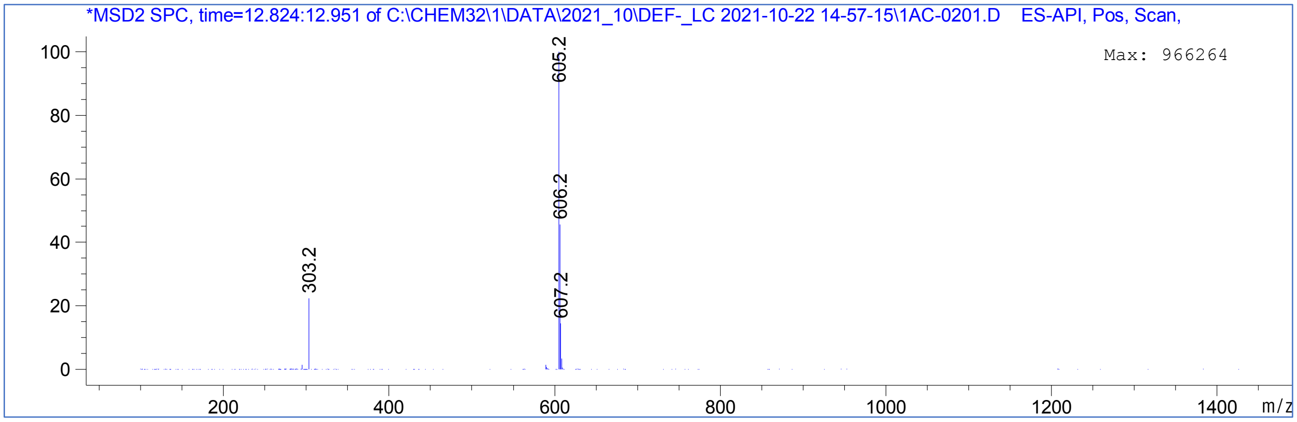
*MSD2 SPC, time=12.824:12.951 of C:\CHEM32\1\DATA\2021_10\DEF-_LC 2021-10-22 14-57-15\1AC-0201.D ES-API, Pos, Scan,
Max: 966264
100
80
60
40
20
0
605.2
606.2
607.2
303.2
200
400
600
800
1000
1200
1400
m/z

Origin	Bruker BioSpin GmbH
Solvent	MeOD
Temperature	295.6
Pulse Sequence	zg30
Experiment	1D
Number of Scans	16
Acquisition Date	2021-04-05T14:28:00
Spectrometer Frequency	400.13
Spectral Width	8012.8
Lowest Frequency	-1543.3
Nucleus	1H
Acquired Size	32768
Spectral Size	65536

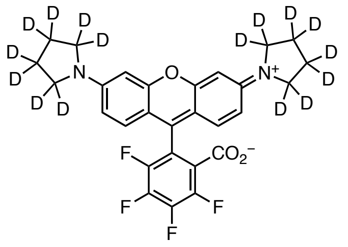

8

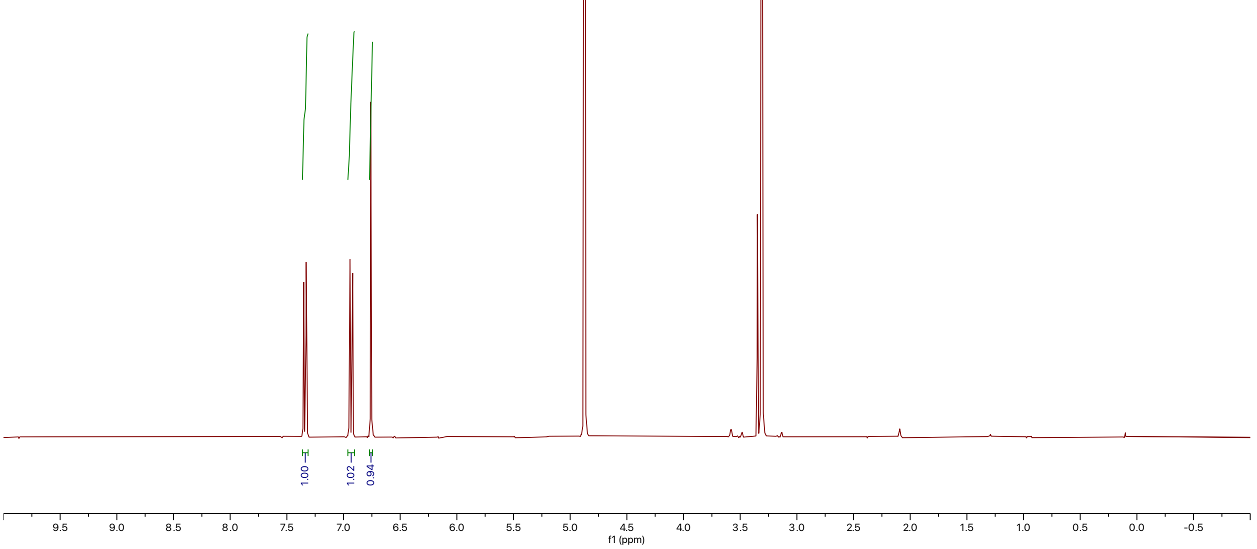

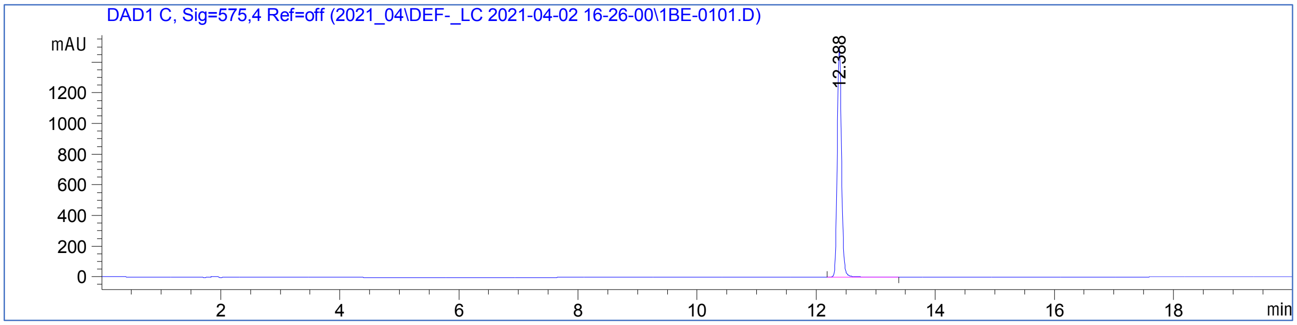

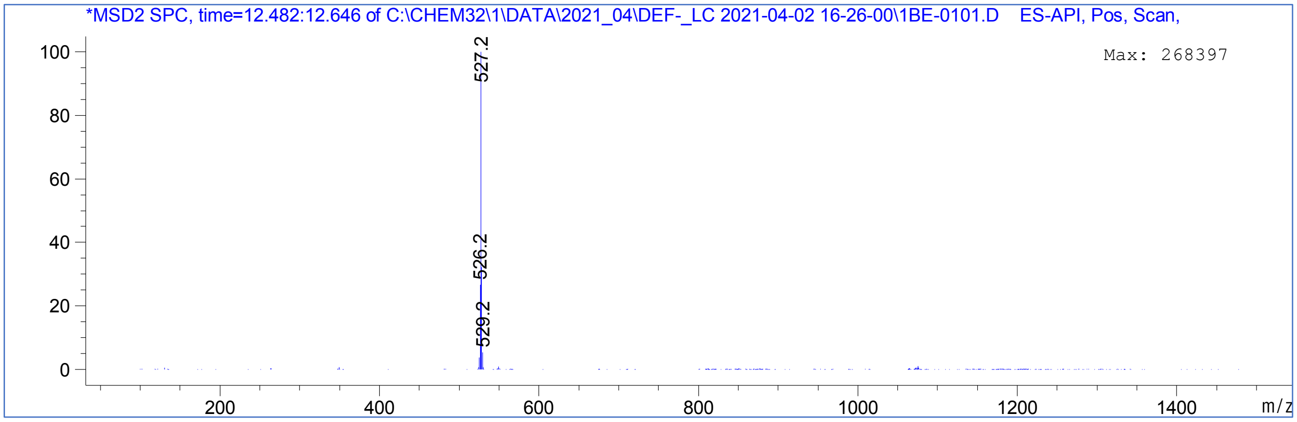

Origin	Bruker BioSpin GmbH
Solvent	CDCl3
Temperature	300.0
Pulse Sequence	zg30
Experiment	1D
Number of Scans	16
Acquisition Date	2019-11-18T13:21:00
Spectrometer Frequency	400.13
Spectral Width	8012.8
Lowest Frequency	-1546.3
Nucleus	1H
Acquired Size	32768
Spectral Size	65536

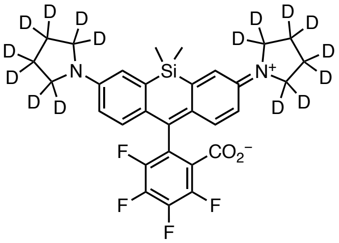

25

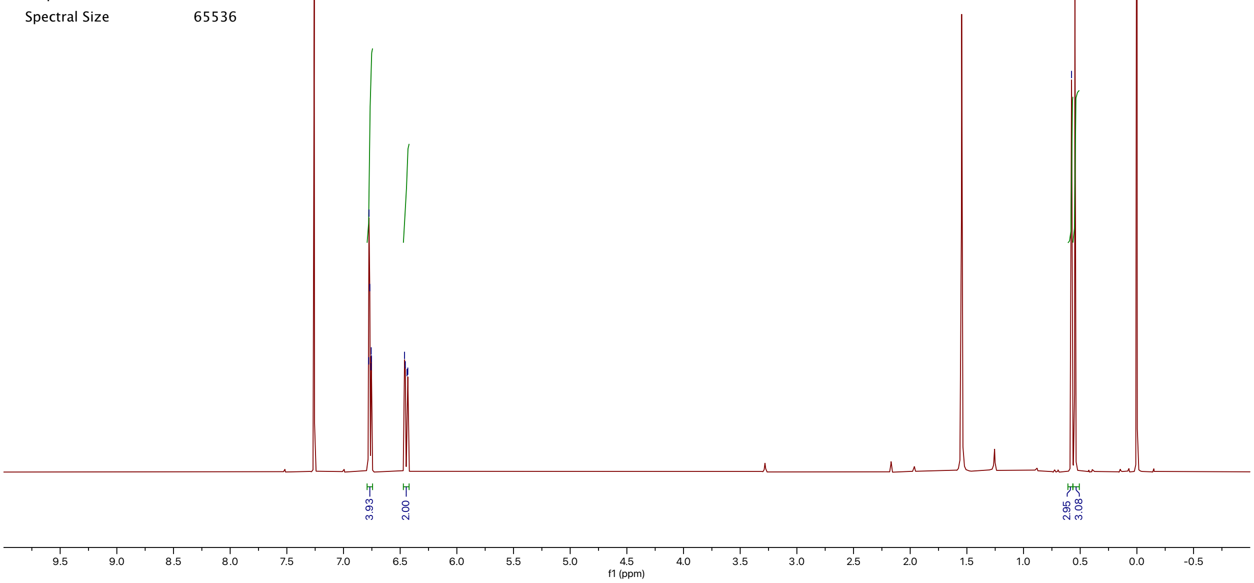

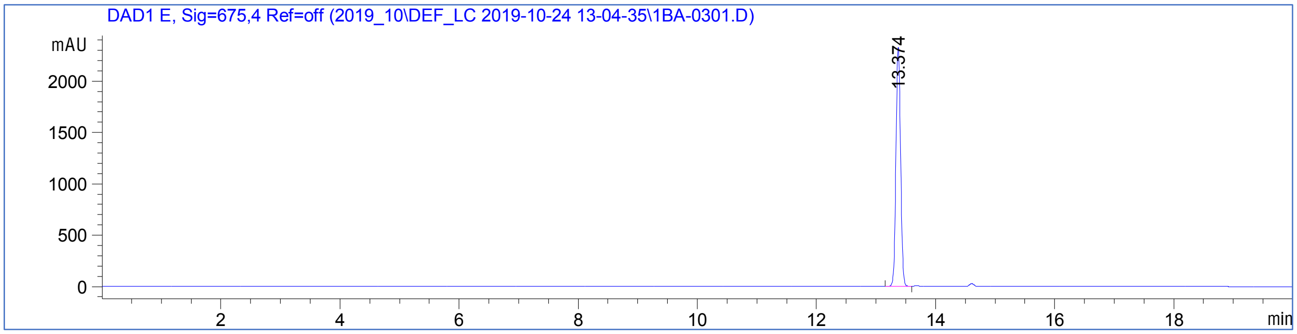

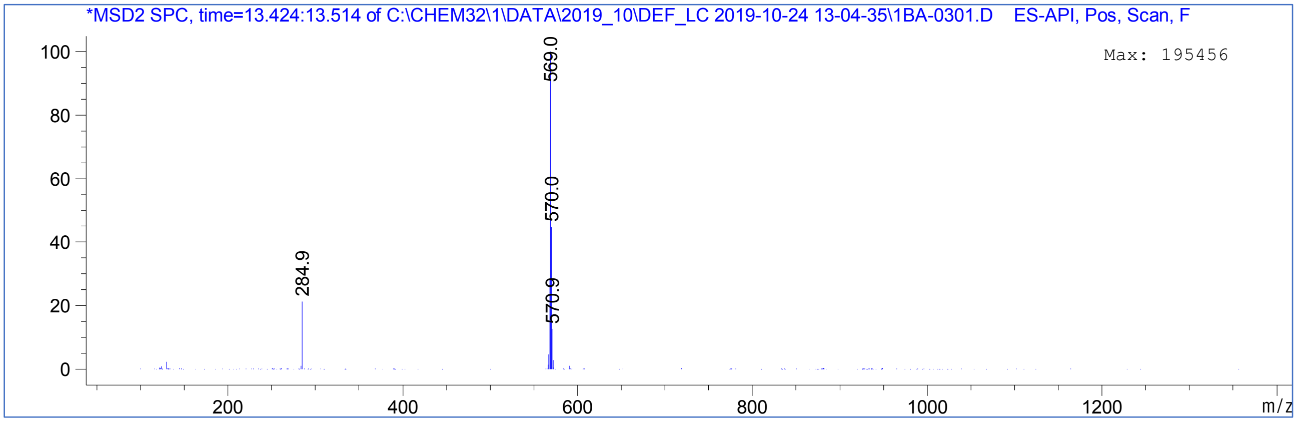

Origin	Bruker BioSpin GmbH
Solvent	CDCl3
Temperature	295.4
Pulse Sequence	zg30
Experiment	1D
Number of Scans	16
Acquisition Date	2021-10-08T16:53:00
Spectrometer Frequency	400.13
Spectral Width	8012.8
Lowest Frequency	-1545.3
Nucleus	1H
Acquired Size	32768
Spectral Size	65536

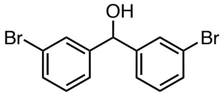

S29

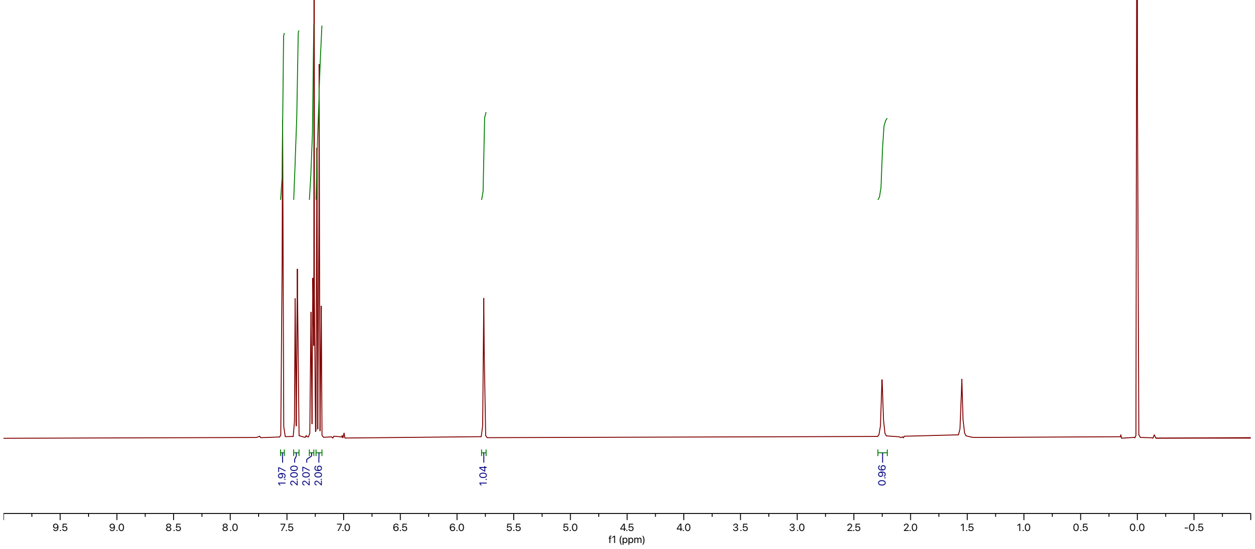

Origin	Bruker BioSpin GmbH
Solvent	CDCl3
Temperature	296.1
Pulse Sequence	zgpg30
Experiment	1D
Number of Scans	1024
Acquisition Date	2021-10-08T20:56:00
Spectrometer Frequency	100.62
Spectral Width	24038.5
Lowest Frequency	-1947.7
Nucleus	13C
Acquired Size	32768
Spectral Size	65536

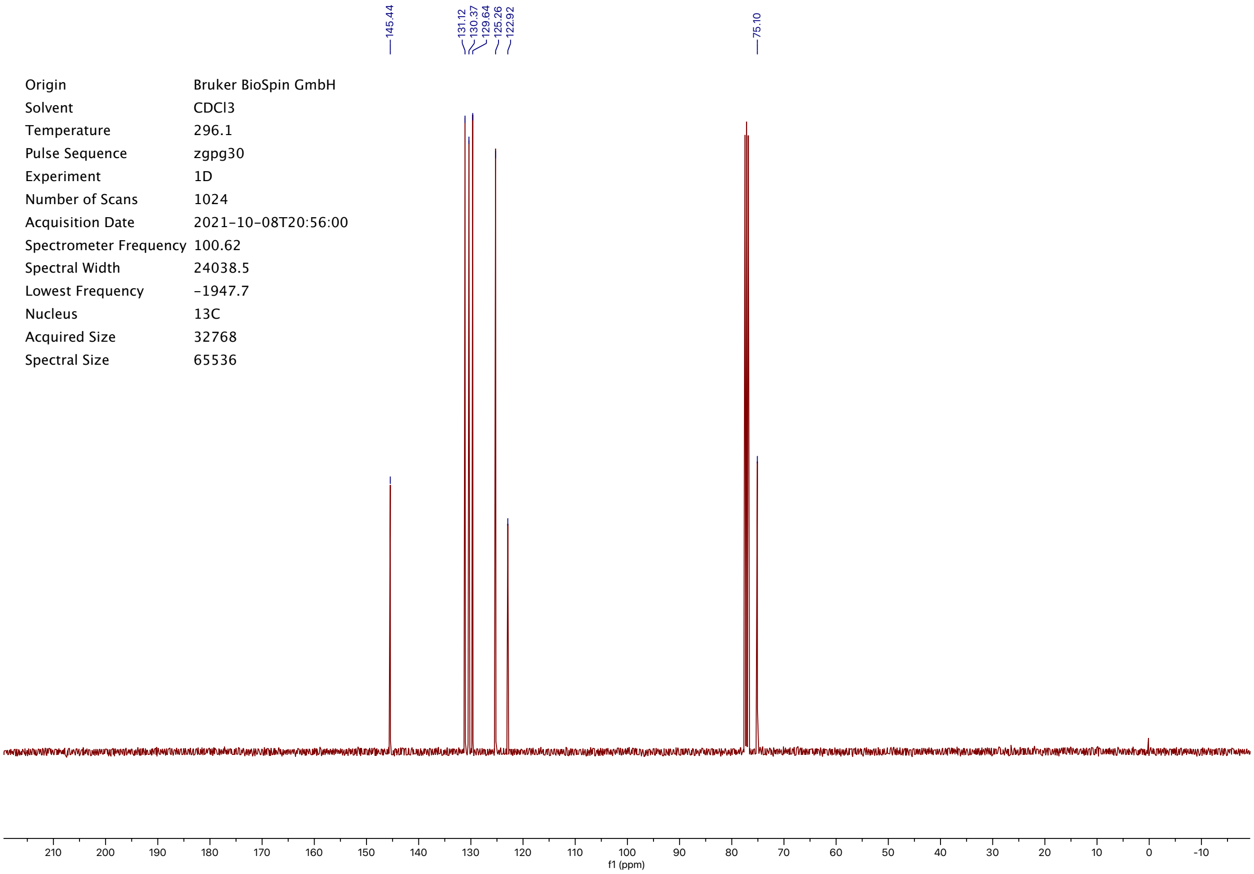

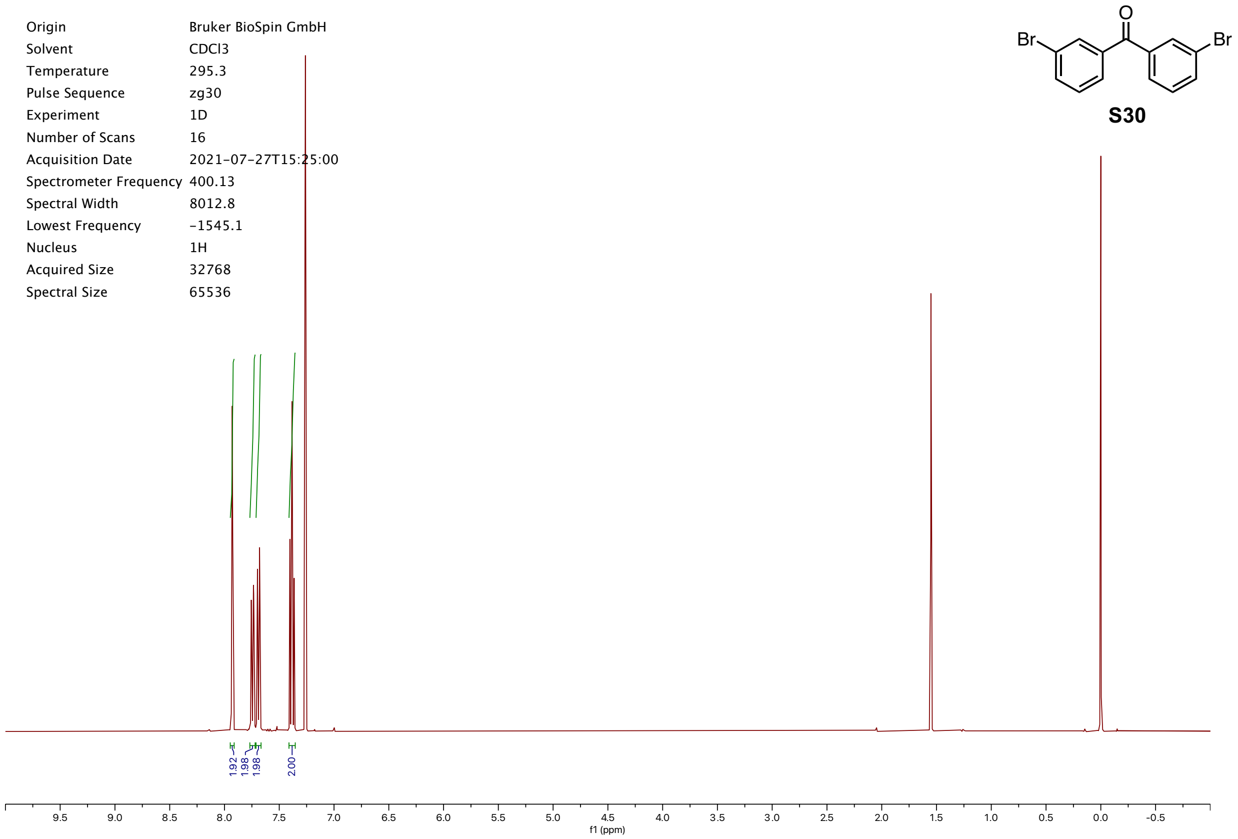

S30

Origin Bruker BioSpin GmbH
Solvent CDCl3
Temperature 295.3
Pulse Sequence zg30
Experiment 1D
Number of Scans 16
Acquisition Date 2021-07-27T15:25:00
Spectrometer Frequency 400.13
Spectral Width 8012.8
Lowest Frequency -1545.1
Nucleus 1H
Acquired Size 32768
Spectral Size 65536
1.92
1.98
1.98
2.00
f1 (ppm)

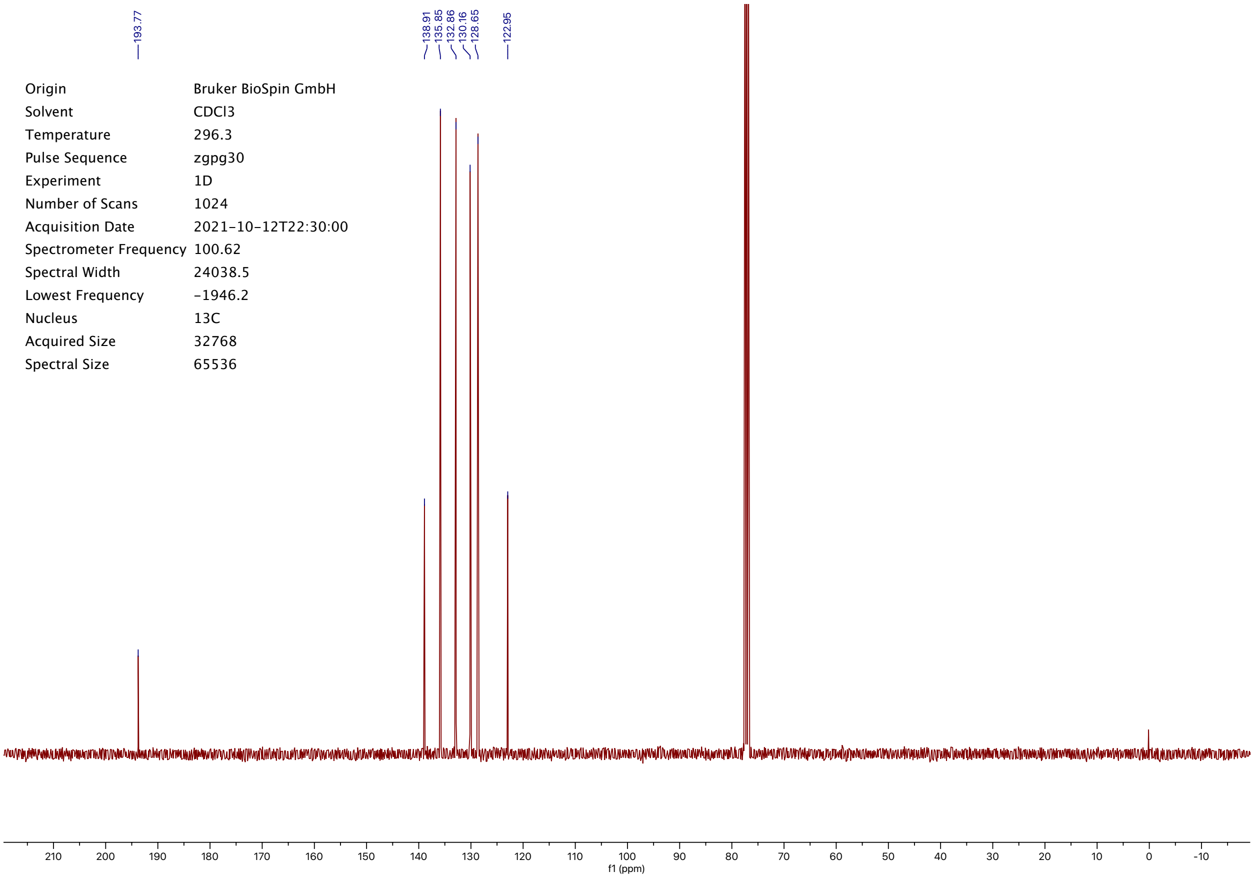
193.77
138.91
135.85
132.86
130.16
128.65
122.95
Origin Bruker BioSpin GmbH
Solvent CDCl3
Temperature 296.3
Pulse Sequence zgpg30
Experiment 1D
Number of Scans 1024
Acquisition Date 2021-10-12T22:30:00
Spectrometer Frequency 100.62
Spectral Width 24038.5
Lowest Frequency -1946.2
Nucleus 13C
Acquired Size 32768
Spectral Size 65536
f1 (ppm)

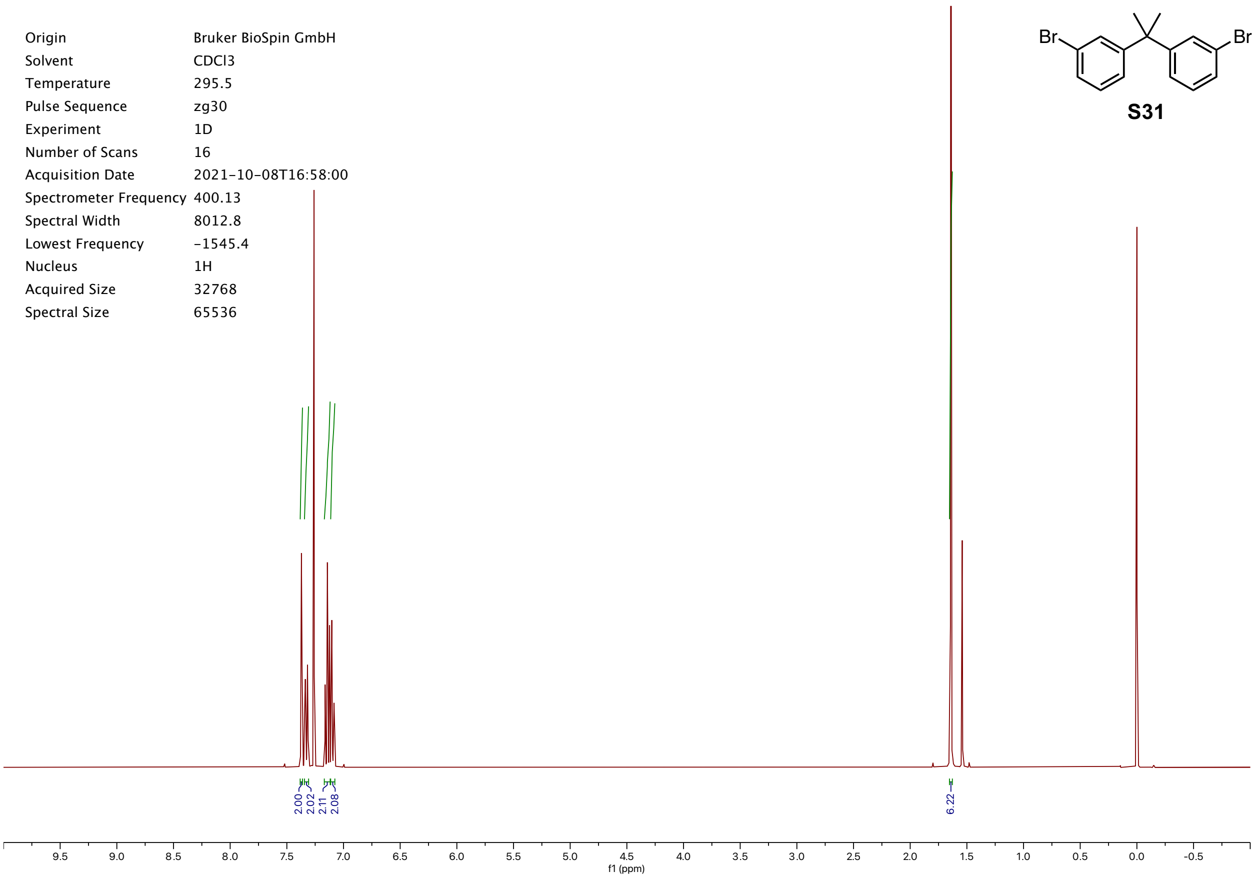
Br
Br

S31

Origin Bruker BioSpin GmbH
Solvent CDCl3
Temperature 295.5
Pulse Sequence zg30
Experiment 1D
Number of Scans 16
Acquisition Date 2021-10-08T16:58:00
Spectrometer Frequency 400.13
Spectral Width 8012.8
Lowest Frequency -1545.4
Nucleus 1H
Acquired Size 32768
Spectral Size 65536
2.00
2.02
2.11
2.08
6.22
9.5 9.0 8.5 8.0 7.5 7.0 6.5 6.0 5.5 5.0 4.5 4.0 3.5 3.0 2.5 2.0 1.5 1.0 0.5 0.0 -0.5
f1 (ppm)

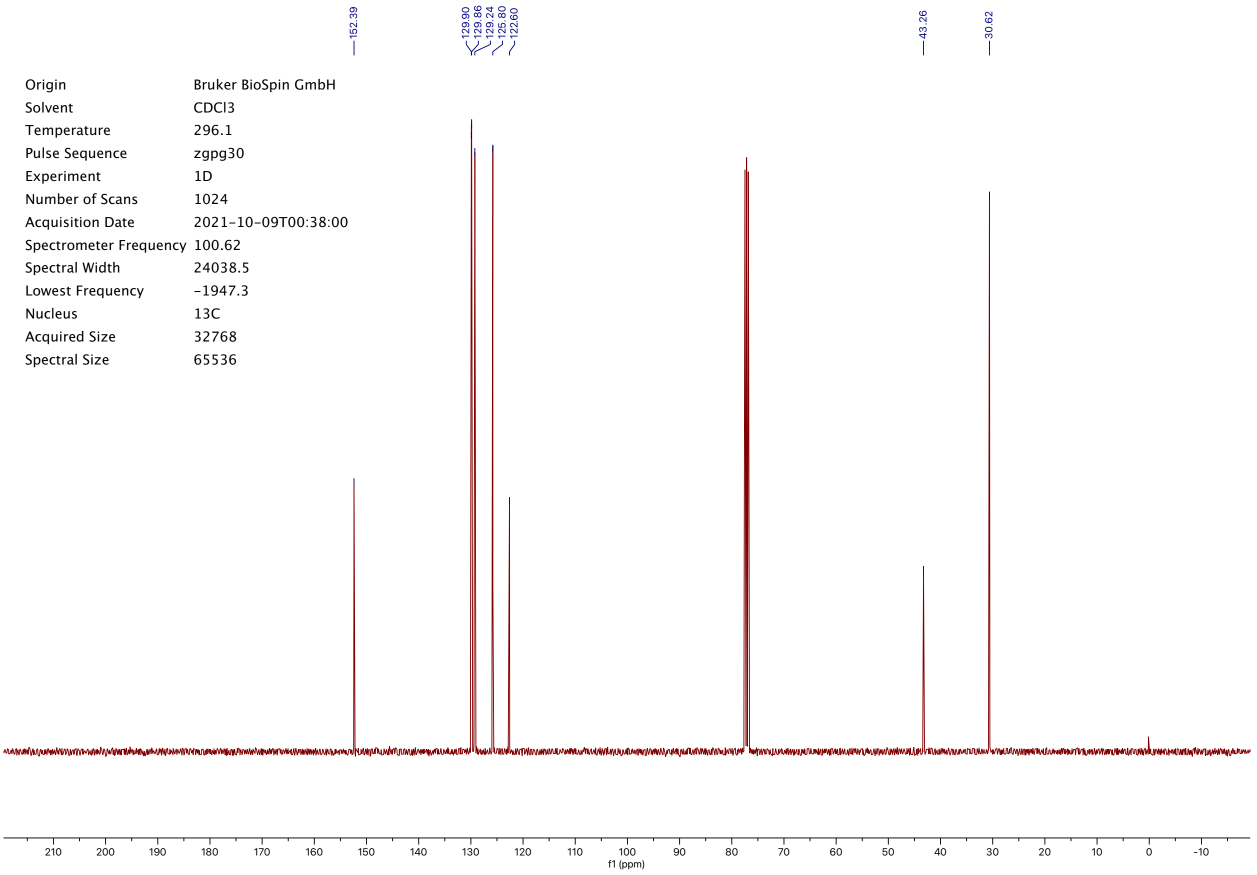
152.39
129.90
129.86
129.24
125.80
122.60
43.26
30.62
Origin Bruker BioSpin GmbH
Solvent CDCl3
Temperature 296.1
Pulse Sequence zgpg30
Experiment 1D
Number of Scans 1024
Acquisition Date 2021-10-09T00:38:00
Spectrometer Frequency 100.62
Spectral Width 24038.5
Lowest Frequency -1947.3
Nucleus 13C
Acquired Size 32768
Spectral Size 65536
210 200 190 180 170 160 150 140 130 120 110 100 90 80 70 60 50 40 30 20 10 0 -10
f1 (ppm)

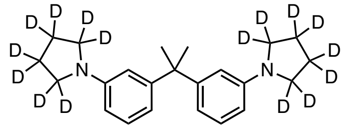

41

Origin	Bruker BioSpin GmbH
Solvent	CDCl3
Temperature	295.4
Pulse Sequence	zg30
Experiment	1D
Number of Scans	16
Acquisition Date	2021-10-08T17:04:00
Spectrometer Frequency	400.13
Spectral Width	8012.8
Lowest Frequency	-1546.2
Nucleus	1H
Acquired Size	32768
Spectral Size	65536

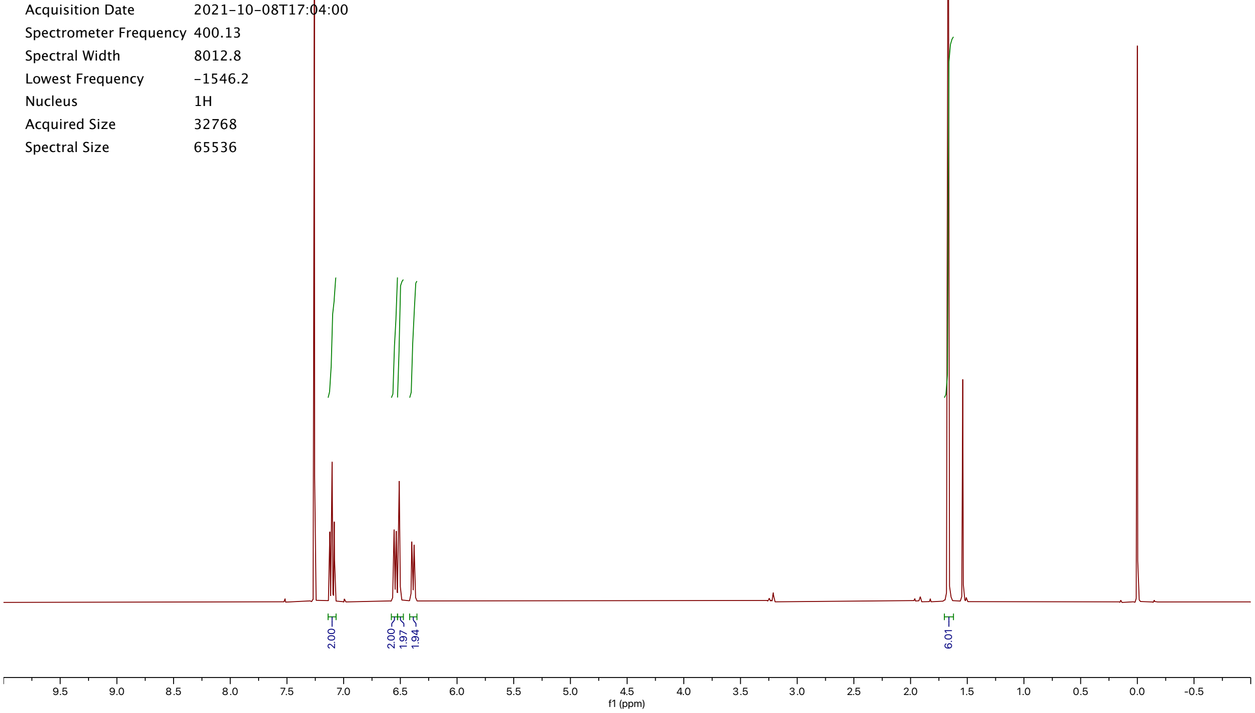

Origin	Bruker BioSpin GmbH
Solvent	CDCl3
Temperature	296.2
Pulse Sequence	zgpg30
Experiment	1D
Number of Scans	256
Acquisition Date	2021-10-11T15:08:00
Spectrometer Frequency	100.62
Spectral Width	24038.5
Lowest Frequency	-1948.4
Nucleus	13C
Acquired Size	32768
Spectral Size	65536

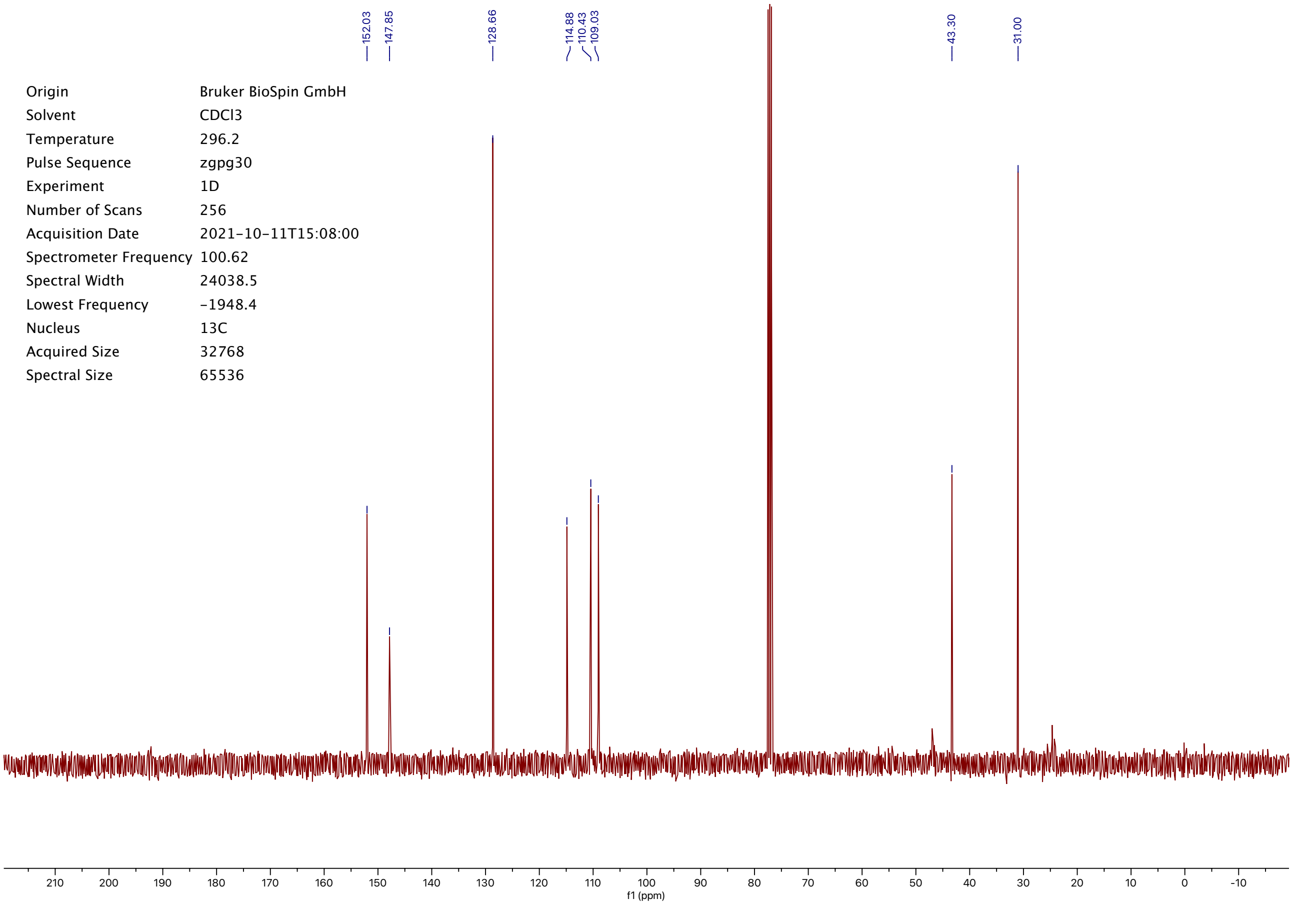

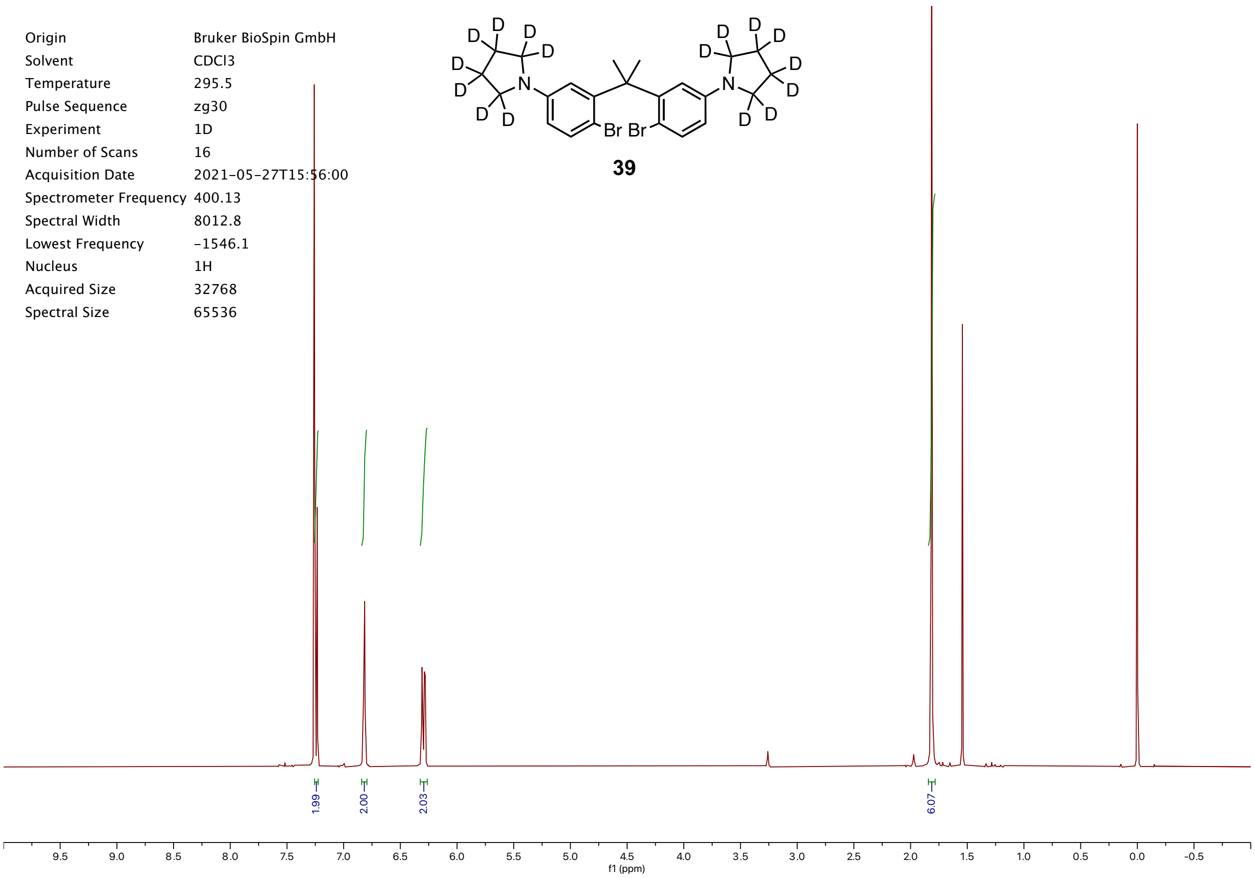
39
Origin Bruker BioSpin GmbH
Solvent CDCl3
Temperature 295.5
Pulse Sequence zg30
Experiment 1D
Number of Scans 16
Acquisition Date 2021-05-27T15:56:00
Spectrometer Frequency 400.13
Spectral Width 8012.8
Lowest Frequency -1546.1
Nucleus 1H
Acquired Size 32768
Spectral Size 65536
1.99
2.00
2.03
6.07
f1 (ppm)

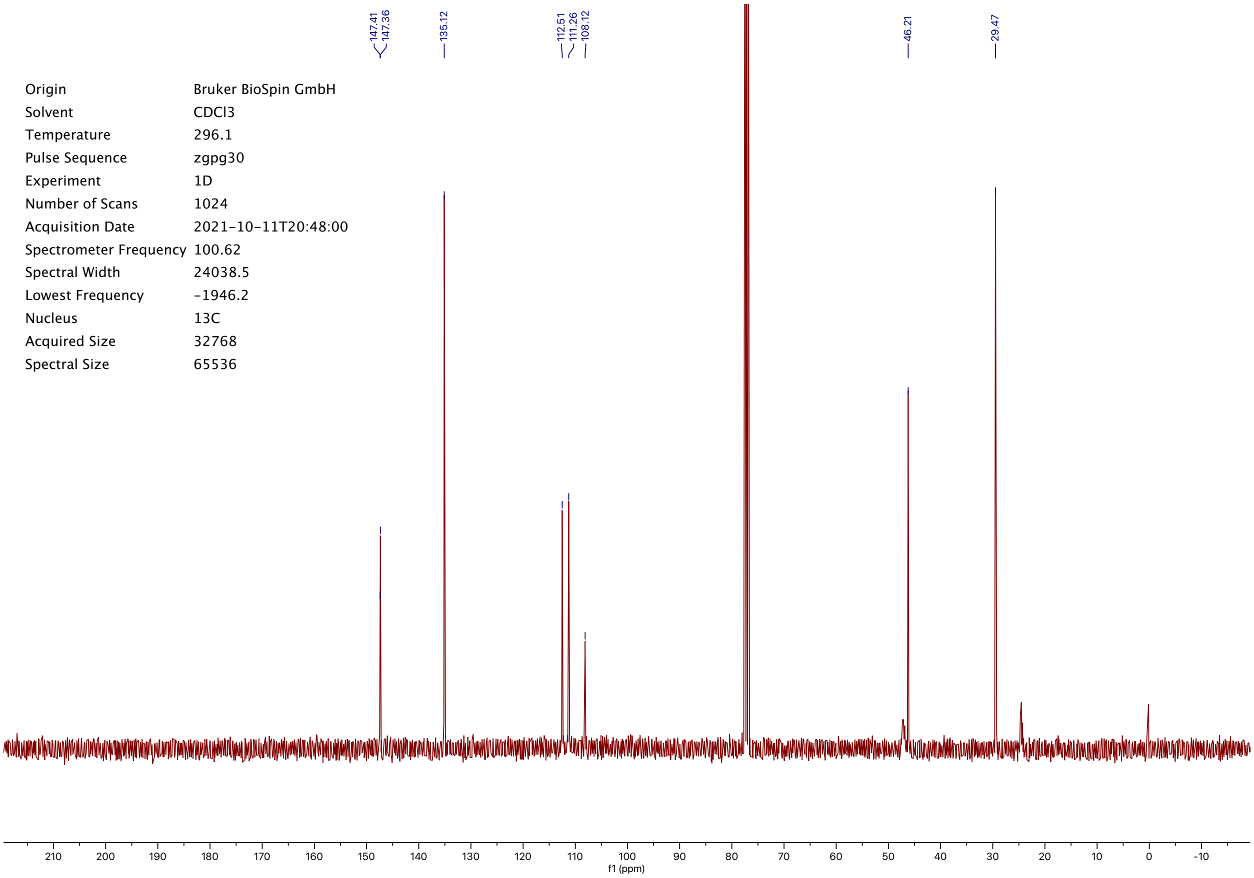
147.41
147.36
135.12
112.51
111.26
108.12
46.21
29.47
Origin Bruker BioSpin GmbH
Solvent CDCl3
Temperature 296.1
Pulse Sequence zgpg30
Experiment 1D
Number of Scans 1024
Acquisition Date 2021-10-11T20:48:00
Spectrometer Frequency 100.62
Spectral Width 24038.5
Lowest Frequency -1946.2
Nucleus 13C
Acquired Size 32768
Spectral Size 65536
f1 (ppm)

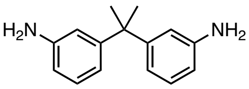

S33

Origin	Bruker BioSpin GmbH
Solvent	CDCl3
Temperature	295.3
Pulse Sequence	zg30
Experiment	1D
Number of Scans	16
Acquisition Date	2021-08-06T15:29:00
Spectrometer Frequency	400.13
Spectral Width	8012.8
Lowest Frequency	-1546.8
Nucleus	1H
Acquired Size	32768
Spectral Size	65536

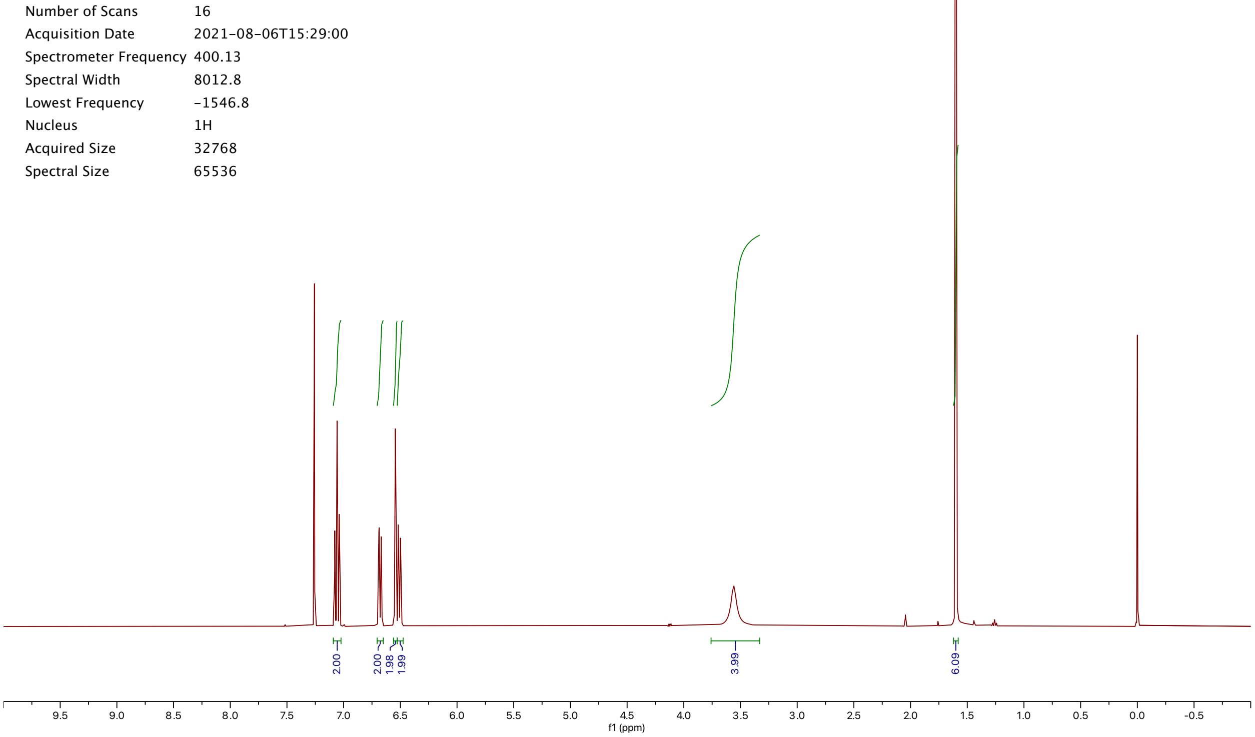

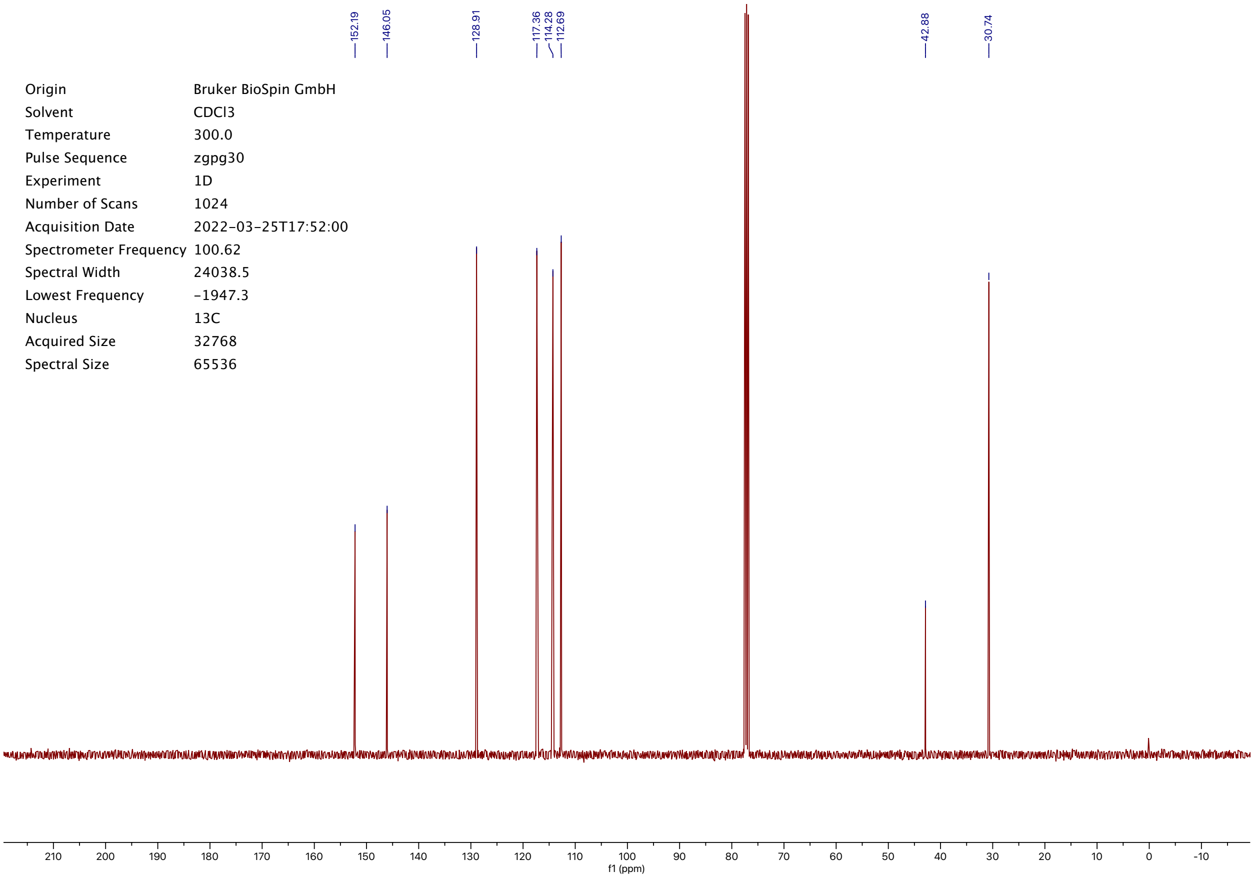

Origin	Bruker BioSpin GmbH
Solvent	CDCl3
Temperature	300.0
Pulse Sequence	zgpg30
Experiment	1D
Number of Scans	1024
Acquisition Date	2022-03-25T17:52:00
Spectrometer Frequency	100.62
Spectral Width	24038.5
Lowest Frequency	-1947.3
Nucleus	13C
Acquired Size	32768
Spectral Size	65536

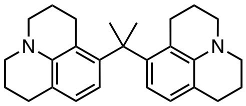

56

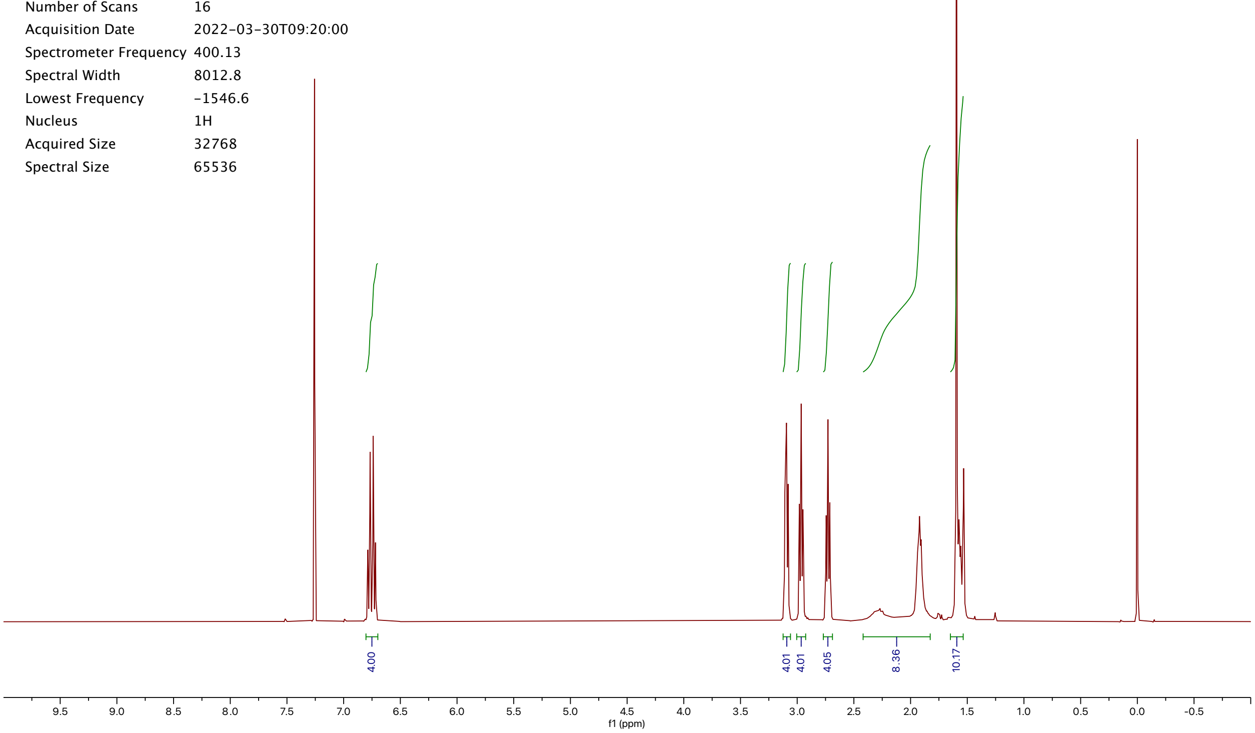

Origin Bruker BioSpin GmbH
Solvent CDCl3
Temperature 300.0
Pulse Sequence zg30
Experiment 1D
Number of Scans 16
Acquisition Date 2022-03-30T09:20:00
Spectrometer Frequency 400.13
Spectral Width 8012.8
Lowest Frequency -1546.6
Nucleus 1H
Acquired Size 32768
Spectral Size 65536
4.00
4.01
4.01
4.05
8.36
10.17
9.5 9.0 8.5 8.0 7.5 7.0 6.5 6.0 5.5 5.0 4.5 4.0 3.5 3.0 2.5 2.0 1.5 1.0 0.5 0.0 -0.5
f1 (ppm)

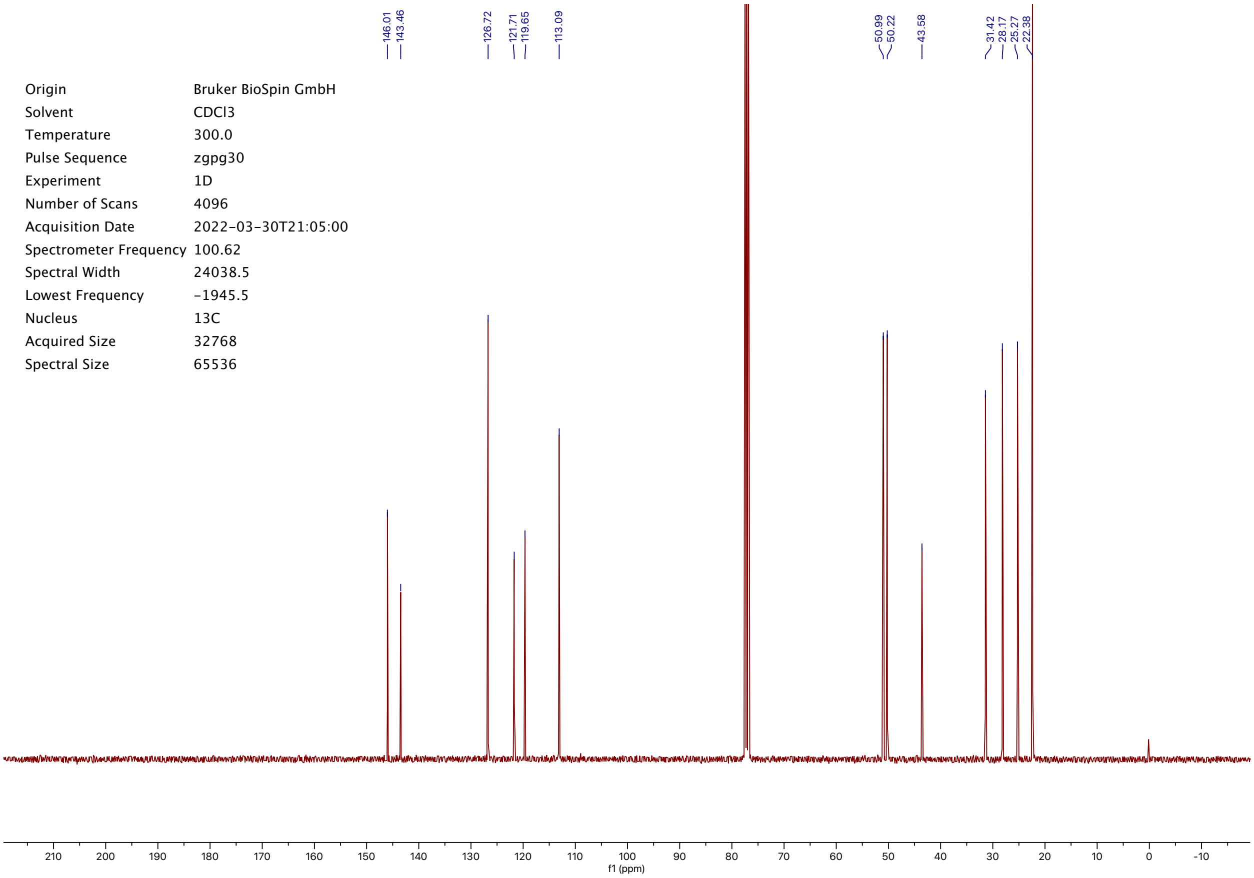

146.01
143.46
126.72
121.71
119.65
113.09
50.99
50.22
43.58
31.42
28.17
25.27
22.38
Origin Bruker BioSpin GmbH
Solvent CDCl3
Temperature 300.0
Pulse Sequence zgpg30
Experiment 1D
Number of Scans 4096
Acquisition Date 2022-03-30T21:05:00
Spectrometer Frequency 100.62
Spectral Width 24038.5
Lowest Frequency -1945.5
Nucleus 13C
Acquired Size 32768
Spectral Size 65536
210 200 190 180 170 160 150 140 130 120 110 100 90 80 70 60 50 40 30 20 10 0 -10
f1 (ppm)

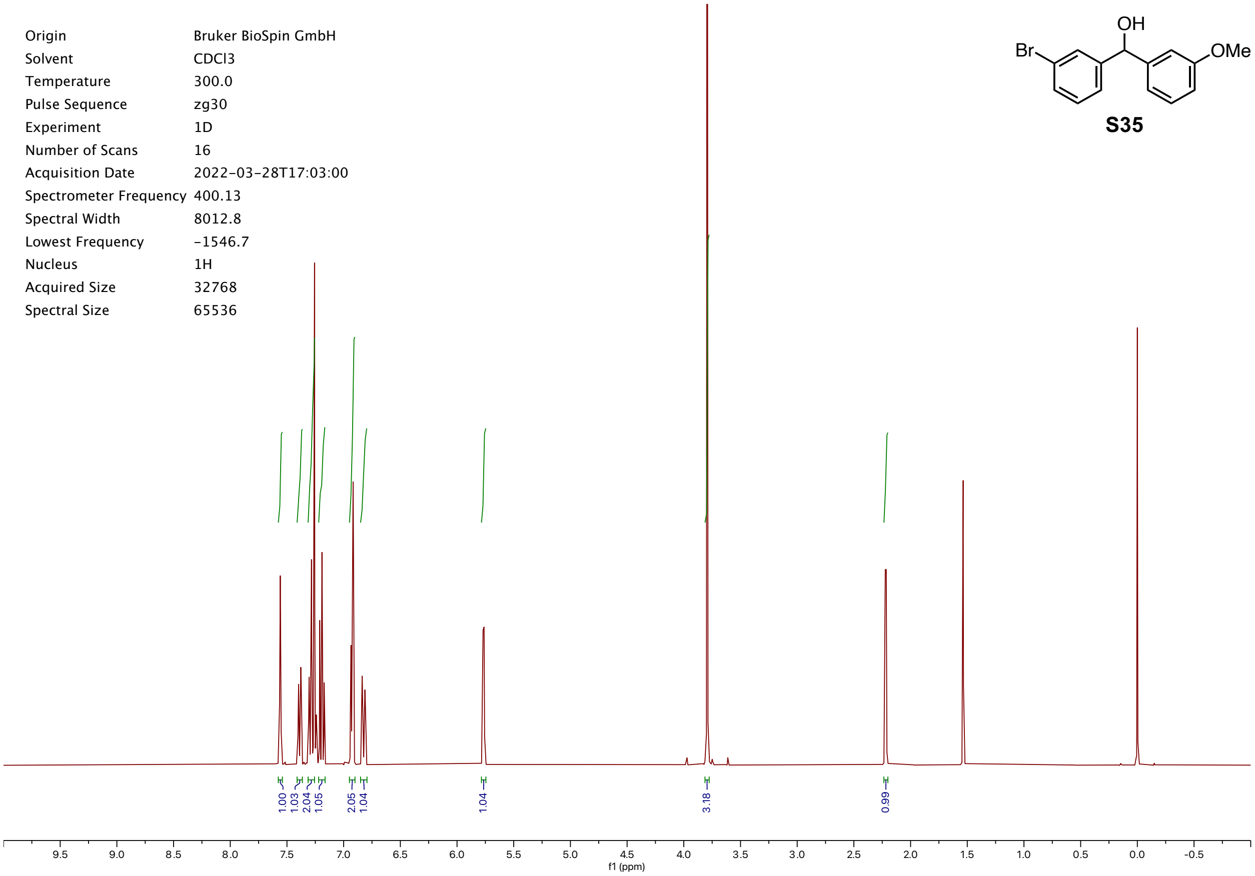

S35

Origin	Bruker BioSpin GmbH
Solvent	CDCl3
Temperature	300.0
Pulse Sequence	zg30
Experiment	1D
Number of Scans	16
Acquisition Date	2022-03-28T17:03:00
Spectrometer Frequency	400.13
Spectral Width	8012.8
Lowest Frequency	-1546.7
Nucleus	1H
Acquired Size	32768
Spectral Size	65536

Origin	Bruker BioSpin GmbH
Solvent	CDCl3
Temperature	300.0
Pulse Sequence	zgpg30
Experiment	1D
Number of Scans	2048
Acquisition Date	2022-03-28T20:06:00
Spectrometer Frequency	100.62
Spectral Width	24038.5
Lowest Frequency	-1945.9
Nucleus	13C
Acquired Size	32768
Spectral Size	65536

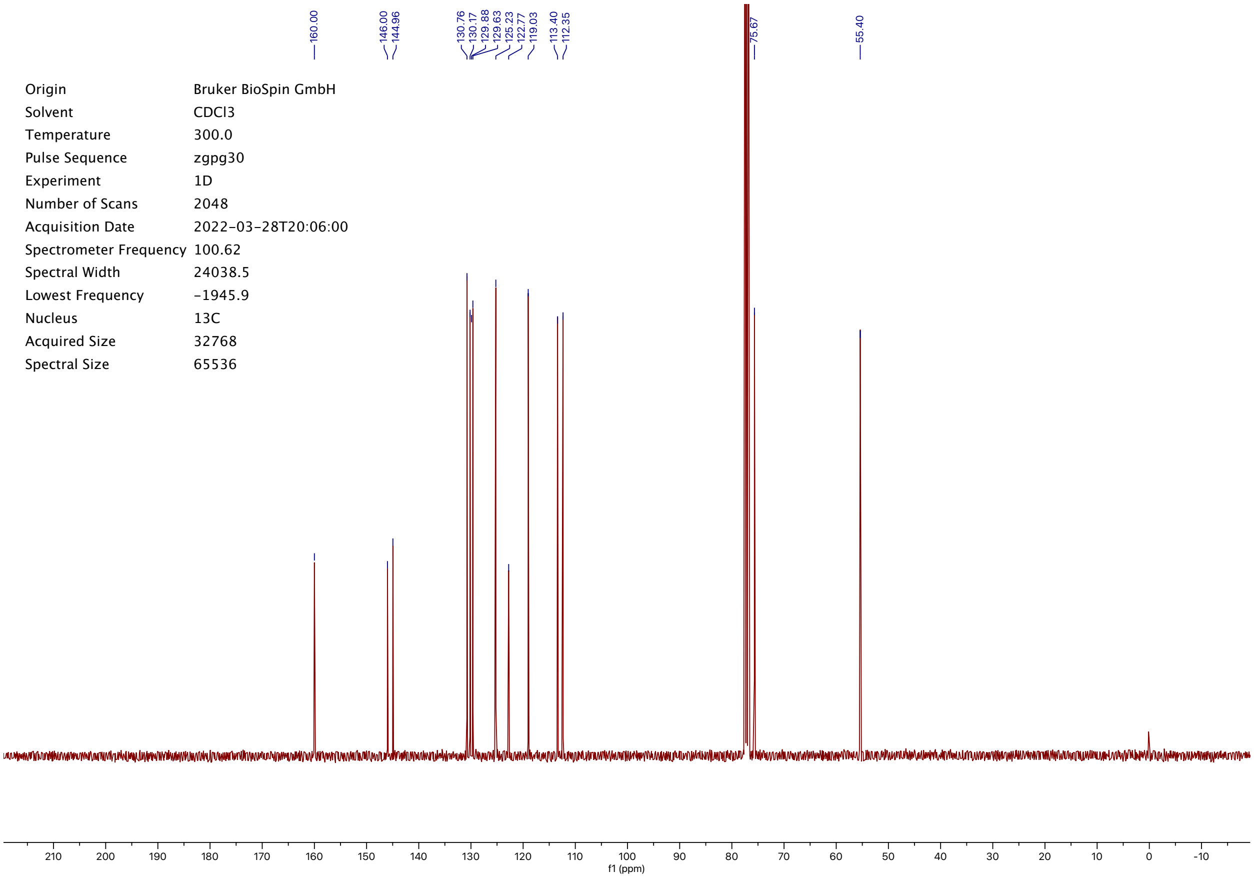

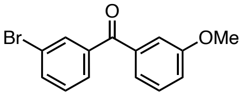

S36

Origin	Bruker BioSpin GmbH
Solvent	CDCl3
Temperature	300.0
Pulse Sequence	zg30
Experiment	1D
Number of Scans	16
Acquisition Date	2022-03-28T16:52:00
Spectrometer Frequency	400.13
Spectral Width	8012.8
Lowest Frequency	-1545.7
Nucleus	1H
Acquired Size	32768
Spectral Size	65536

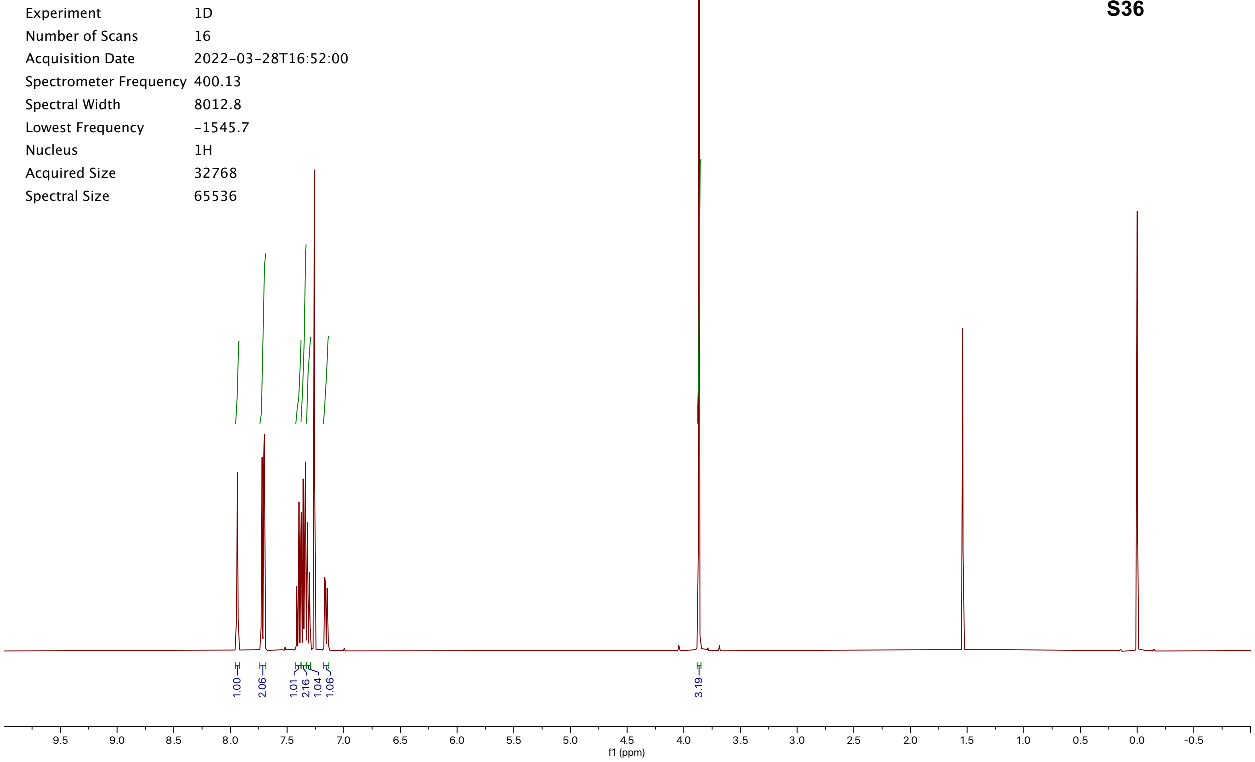

Origin	Bruker BioSpin GmbH
Solvent	CDCl3
Temperature	300.0
Pulse Sequence	zgpg30
Experiment	1D
Number of Scans	2048
Acquisition Date	2022-03-28T23:47:00
Spectrometer Frequency	100.62
Spectral Width	24038.5
Lowest Frequency	-1945.5
Nucleus	13C
Acquired Size	32768
Spectral Size	65536

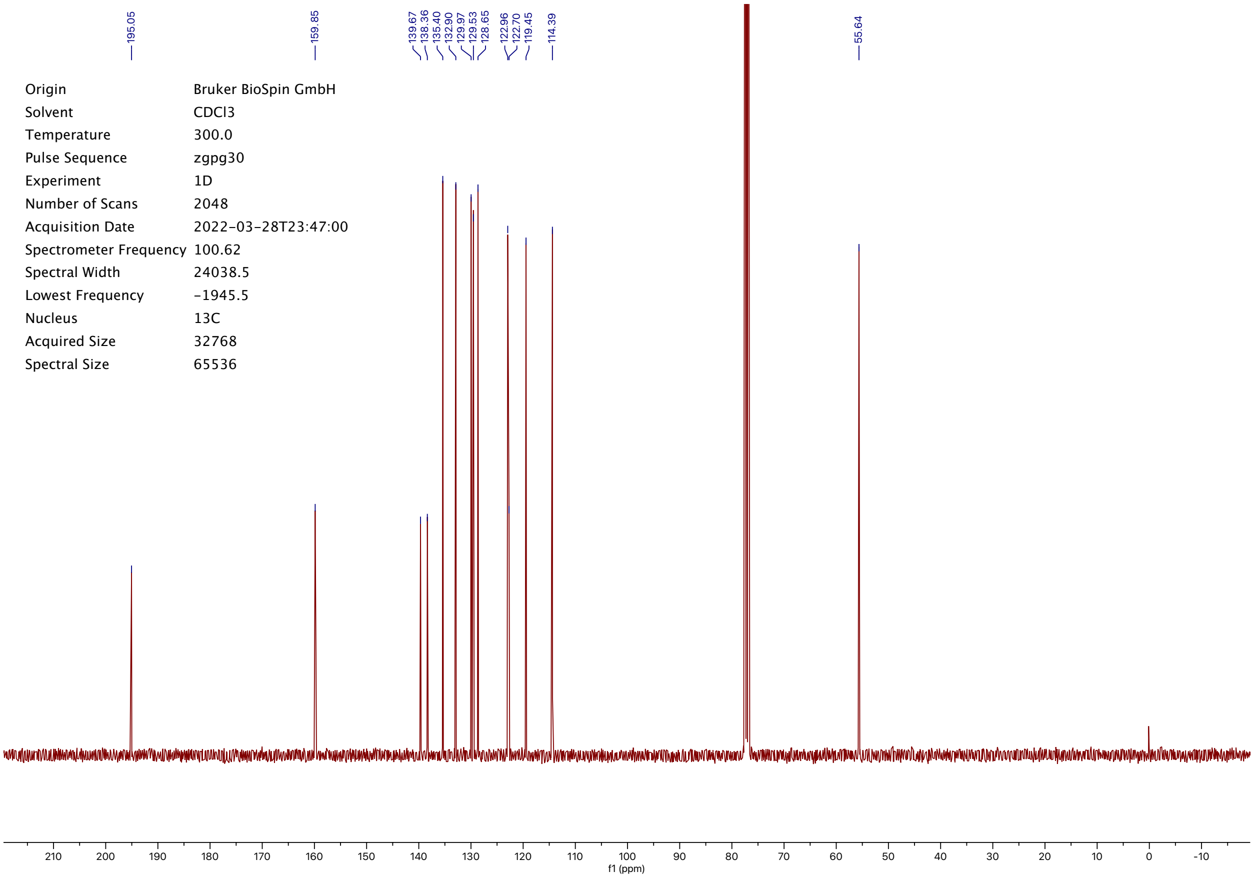

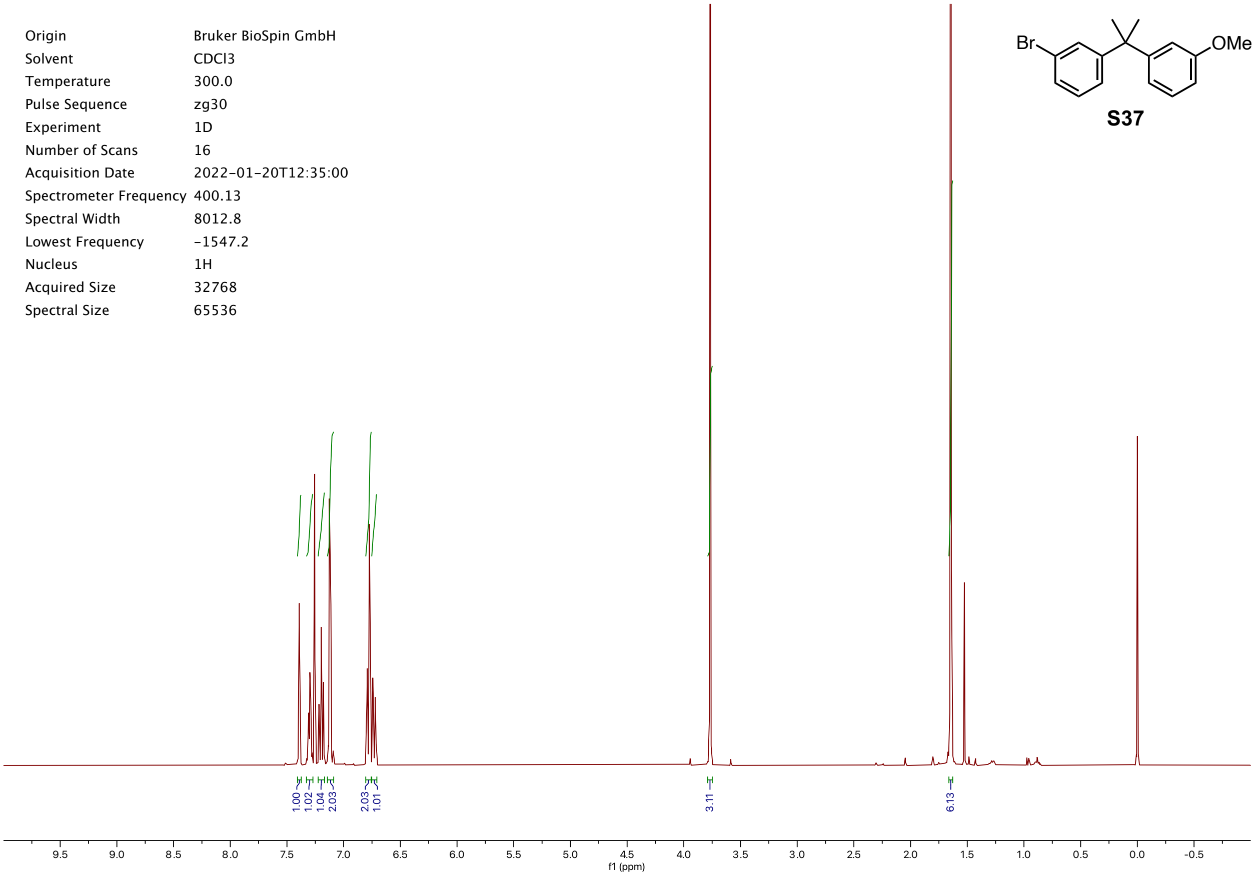
Br
OMe
S37
Origin Bruker BioSpin GmbH
Solvent CDCl3
Temperature 300.0
Pulse Sequence zg30
Experiment 1D
Number of Scans 16
Acquisition Date 2022-01-20T12:35:00
Spectrometer Frequency 400.13
Spectral Width 8012.8
Lowest Frequency -1547.2
Nucleus 1H
Acquired Size 32768
Spectral Size 65536
1.00
1.02
1.04
2.03
2.03
1.01
3.11
6.13
9.5
9.0
8.5
8.0
7.5
7.0
6.5
6.0
5.5
5.0
4.5
4.0
3.5
3.0
2.5
2.0
1.5
1.0
0.5
0.0
-0.5
f1 (ppm)

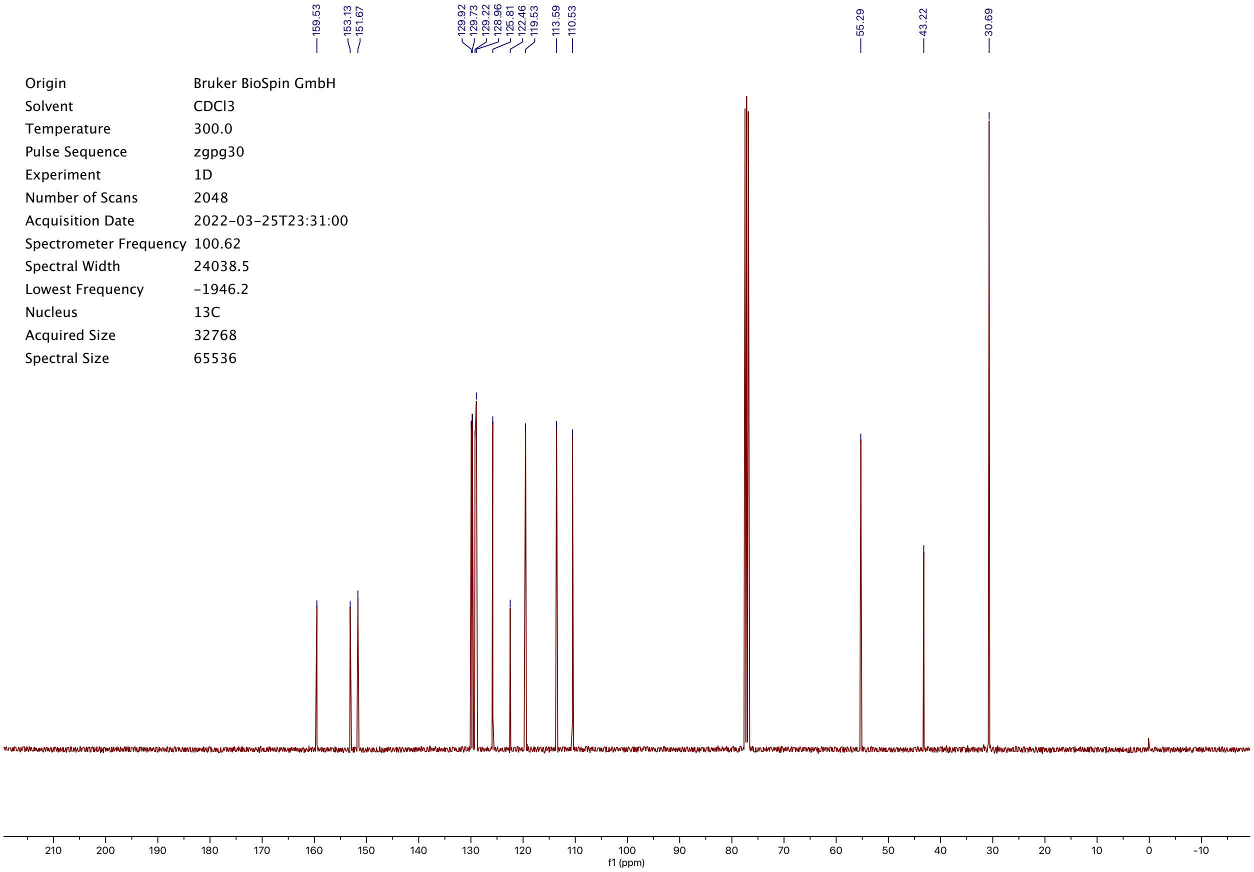
159.53
153.13
151.67
129.92
129.73
129.22
128.96
125.81
122.46
119.53
113.59
110.53
55.29
43.22
30.69
Origin Bruker BioSpin GmbH
Solvent CDCl3
Temperature 300.0
Pulse Sequence zgpg30
Experiment 1D
Number of Scans 2048
Acquisition Date 2022-03-25T23:31:00
Spectrometer Frequency 100.62
Spectral Width 24038.5
Lowest Frequency -1946.2
Nucleus 13C
Acquired Size 32768
Spectral Size 65536
210
200
190
180
170
160
150
140
130
120
110
100
90
80
70
60
50
40
30
20
10
0
-10
f1 (ppm)

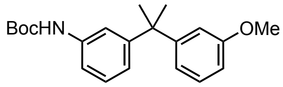

S38

Origin	Bruker BioSpin GmbH
Solvent	CDCl3
Temperature	300.0
Pulse Sequence	zg30
Experiment	1D
Number of Scans	16
Acquisition Date	2022-03-29T00:29:00
Spectrometer Frequency	400.13
Spectral Width	8012.8
Lowest Frequency	-1548.9
Nucleus	1H
Acquired Size	32768
Spectral Size	65536

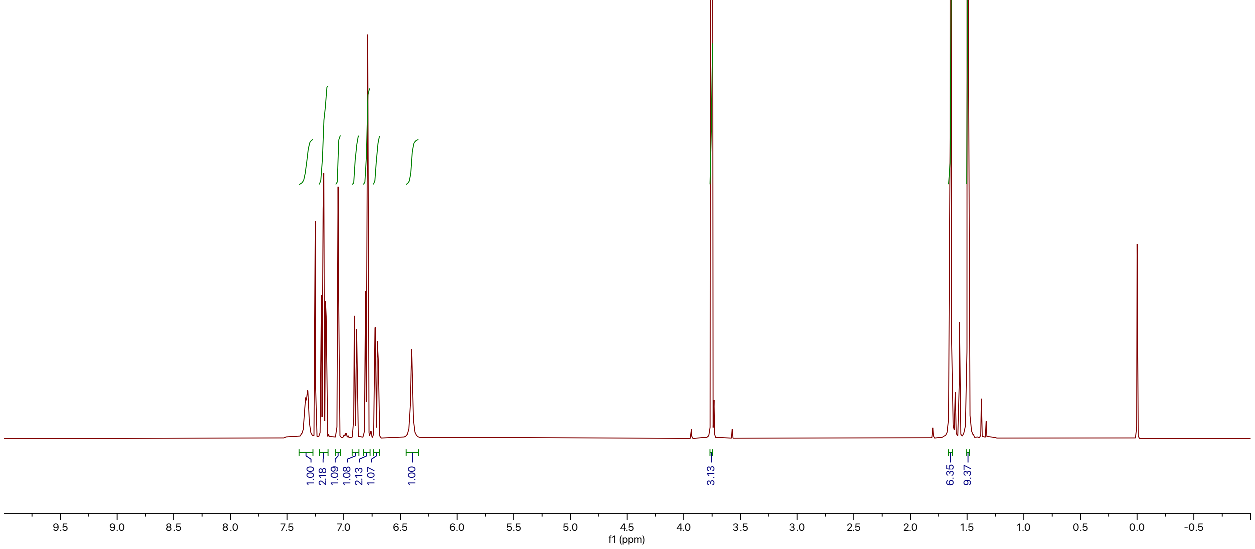

Origin	Bruker BioSpin GmbH
Solvent	CDCl3
Temperature	300.0
Pulse Sequence	zgpg30
Experiment	1D
Number of Scans	2048
Acquisition Date	2022-03-29T03:28:00
Spectrometer Frequency	100.62
Spectral Width	24038.5
Lowest Frequency	-1945.5
Nucleus	13C
Acquired Size	32768
Spectral Size	65536

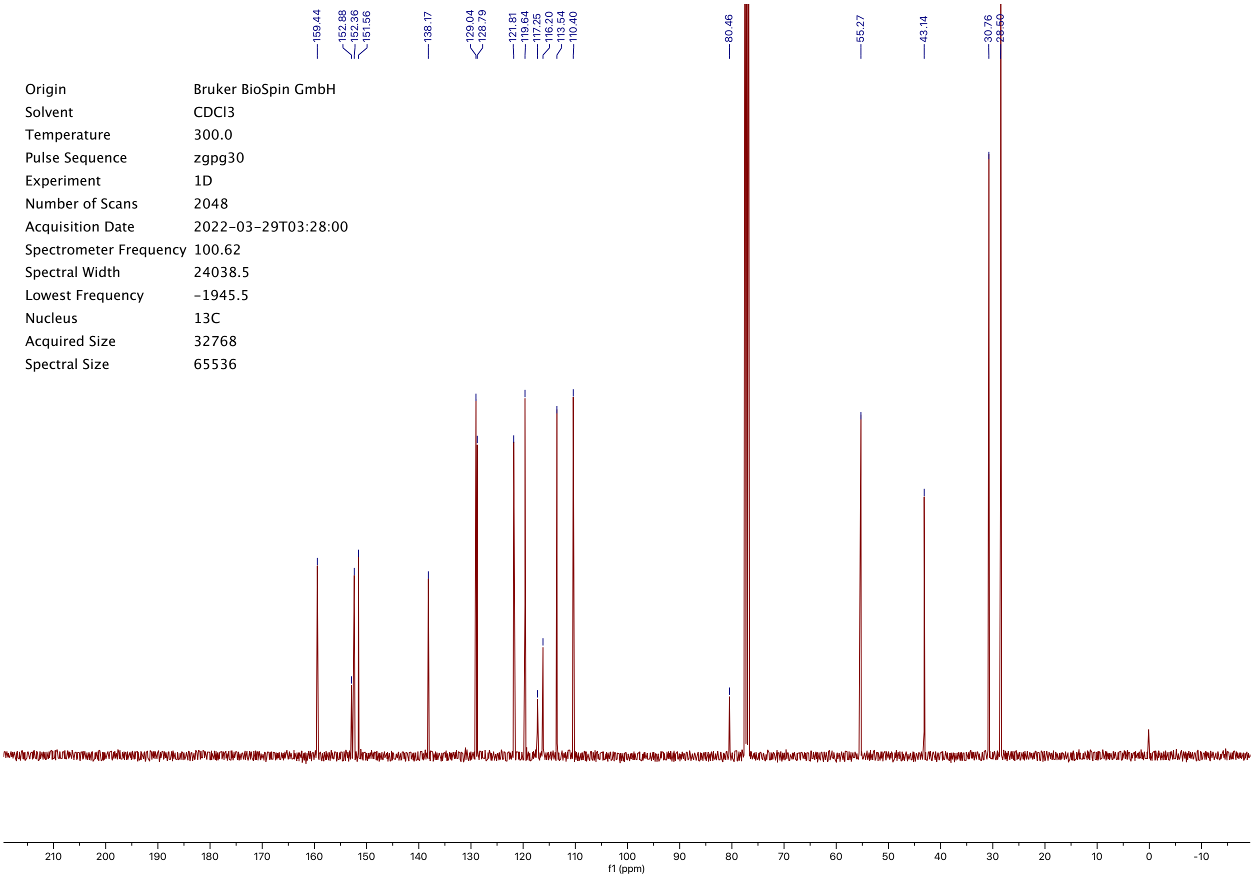

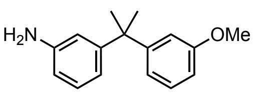

S39

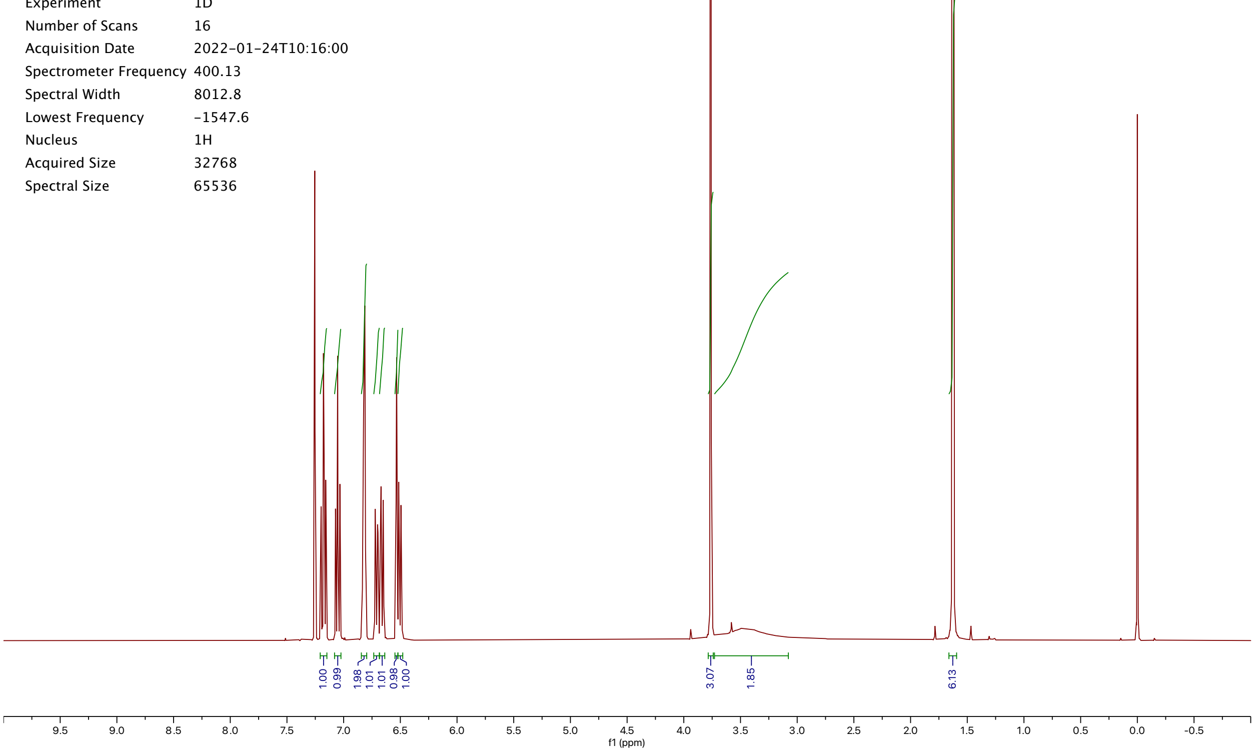

Origin Bruker BioSpin GmbH
Solvent CDCl3
Temperature 300.0
Pulse Sequence zg30
Experiment 1D
Number of Scans 16
Acquisition Date 2022-01-24T10:16:00
Spectrometer Frequency 400.13
Spectral Width 8012.8
Lowest Frequency -1547.6
Nucleus 1H
Acquired Size 32768
Spectral Size 65536
1.00
0.99
1.98
1.01
1.01
0.98
1.00
3.07
1.85
6.13
f1 (ppm)

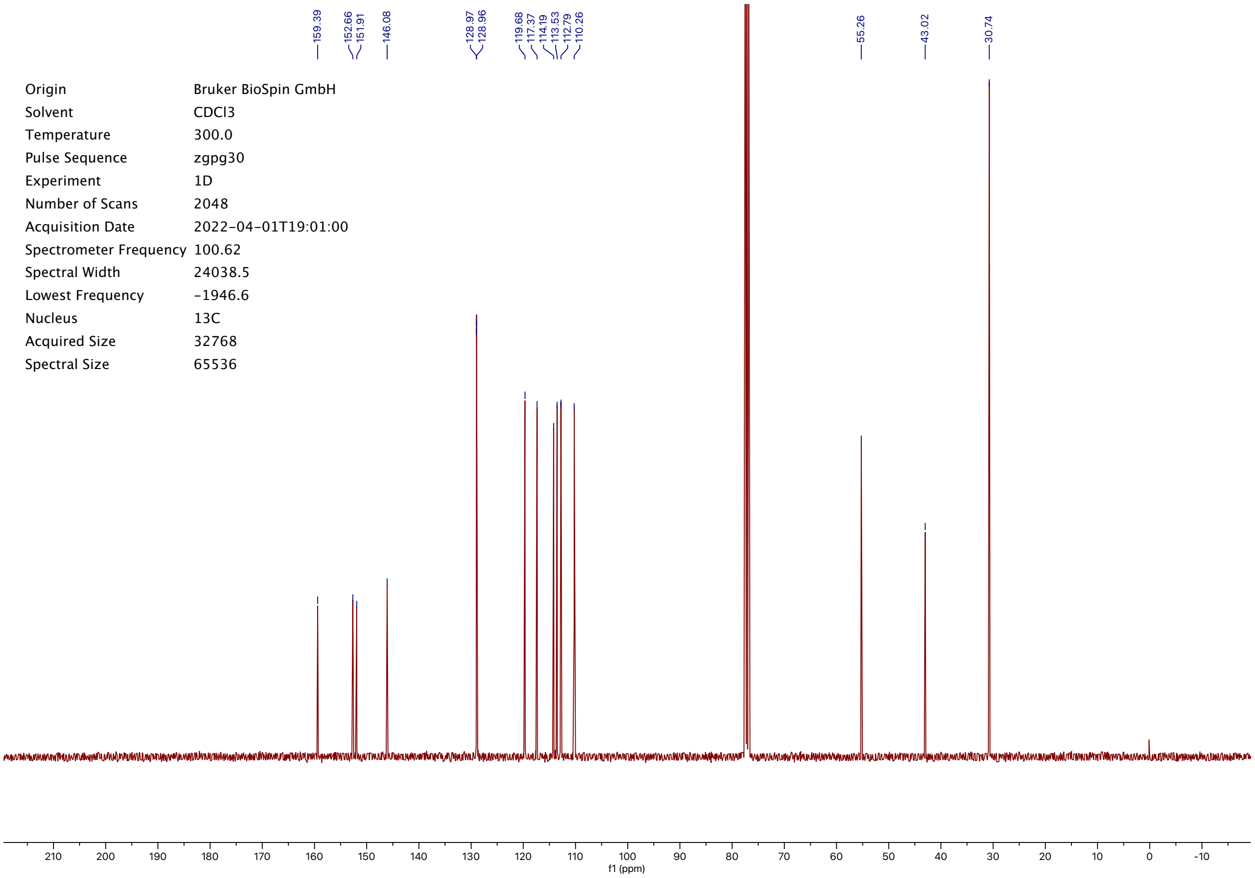

159.39
152.66
151.91
146.08
128.97
128.96
119.68
117.37
114.19
113.53
112.79
110.26
55.26
43.02
30.74
Origin Bruker BioSpin GmbH
Solvent CDCl3
Temperature 300.0
Pulse Sequence zgpg30
Experiment 1D
Number of Scans 2048
Acquisition Date 2022-04-01T19:01:00
Spectrometer Frequency 100.62
Spectral Width 24038.5
Lowest Frequency -1946.6
Nucleus 13C
Acquired Size 32768
Spectral Size 65536
f1 (ppm)

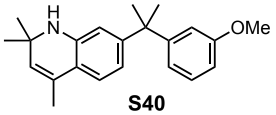

S40

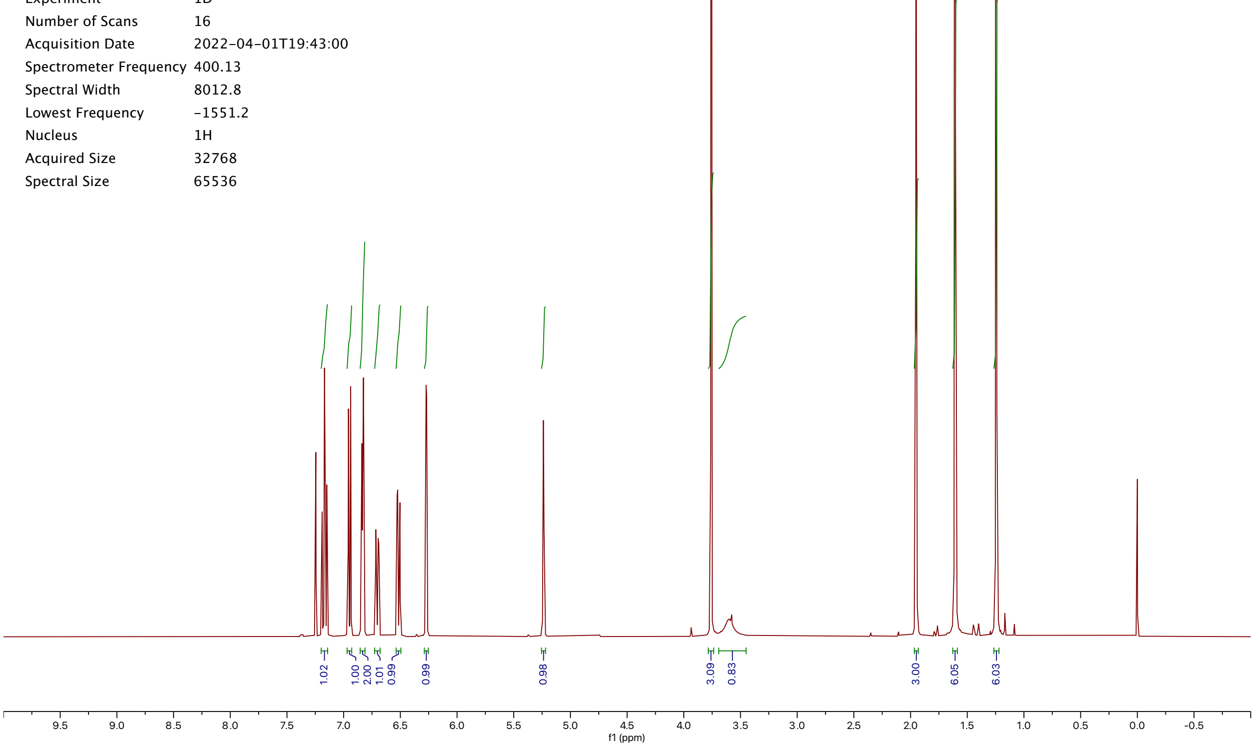

Origin Bruker BioSpin GmbH
Solvent CDCl3
Temperature 300.0
Pulse Sequence zg30
Experiment 1D
Number of Scans 16
Acquisition Date 2022-04-01T19:43:00
Spectrometer Frequency 400.13
Spectral Width 8012.8
Lowest Frequency -1551.2
Nucleus 1H
Acquired Size 32768
Spectral Size 65536
1.02
1.00
2.00
1.01
0.99
0.99
0.98
3.09
0.83
3.00
6.05
6.03
9.5 9.0 8.5 8.0 7.5 7.0 6.5 6.0 5.5 5.0 4.5 4.0 3.5 3.0 2.5 2.0 1.5 1.0 0.5 0.0 -0.5
f1 (ppm)

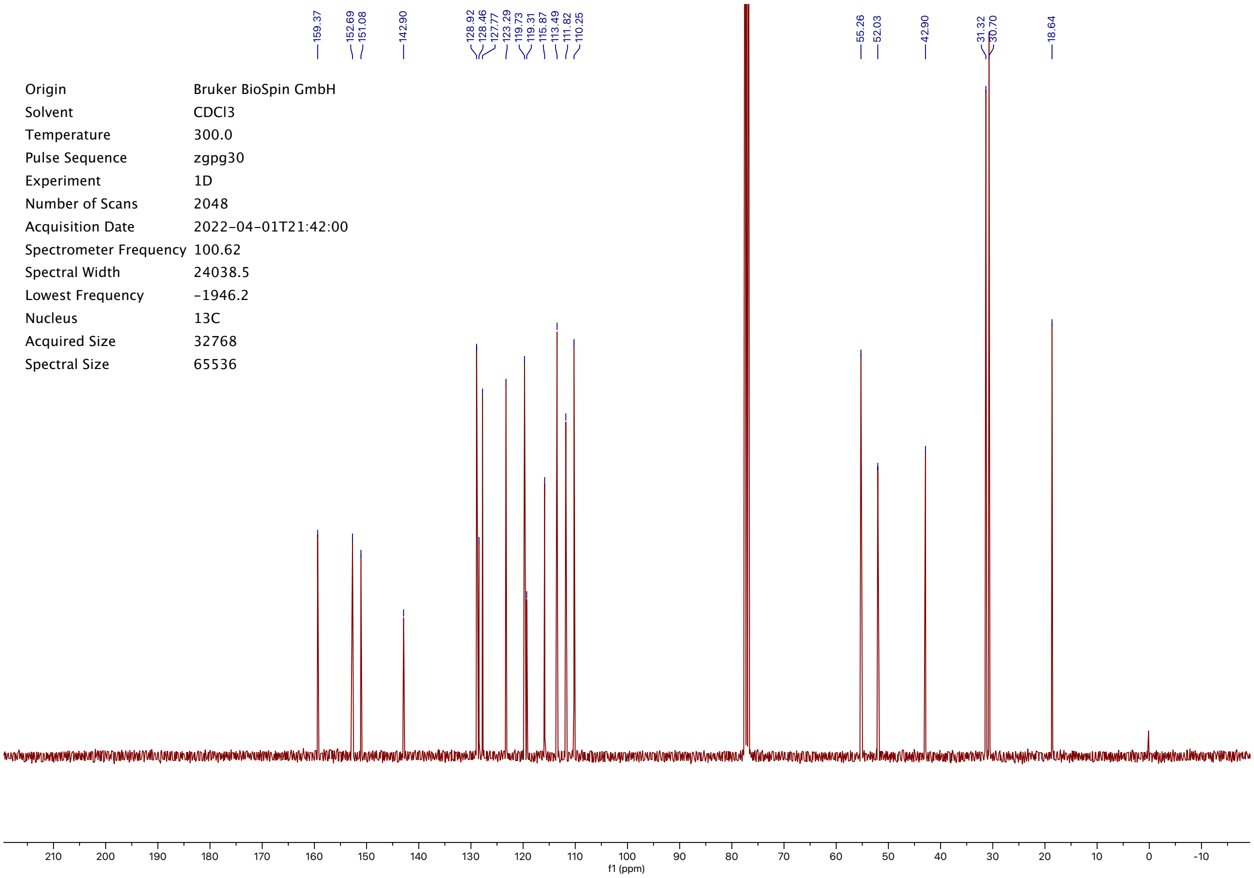

159.37
152.69
151.08
142.90
128.92
128.46
127.77
123.29
119.73
119.31
115.87
113.49
111.82
110.25
55.26
52.03
42.90
31.32
30.70
18.64
Origin Bruker BioSpin GmbH
Solvent CDCl3
Temperature 300.0
Pulse Sequence zgpg30
Experiment 1D
Number of Scans 2048
Acquisition Date 2022-04-01T21:42:00
Spectrometer Frequency 100.62
Spectral Width 24038.5
Lowest Frequency -1946.2
Nucleus 13C
Acquired Size 32768
Spectral Size 65536
210 200 190 180 170 160 150 140 130 120 110 100 90 80 70 60 50 40 30 20 10 0 -10
f1 (ppm)

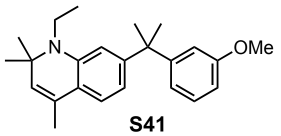
N
OMe

S41

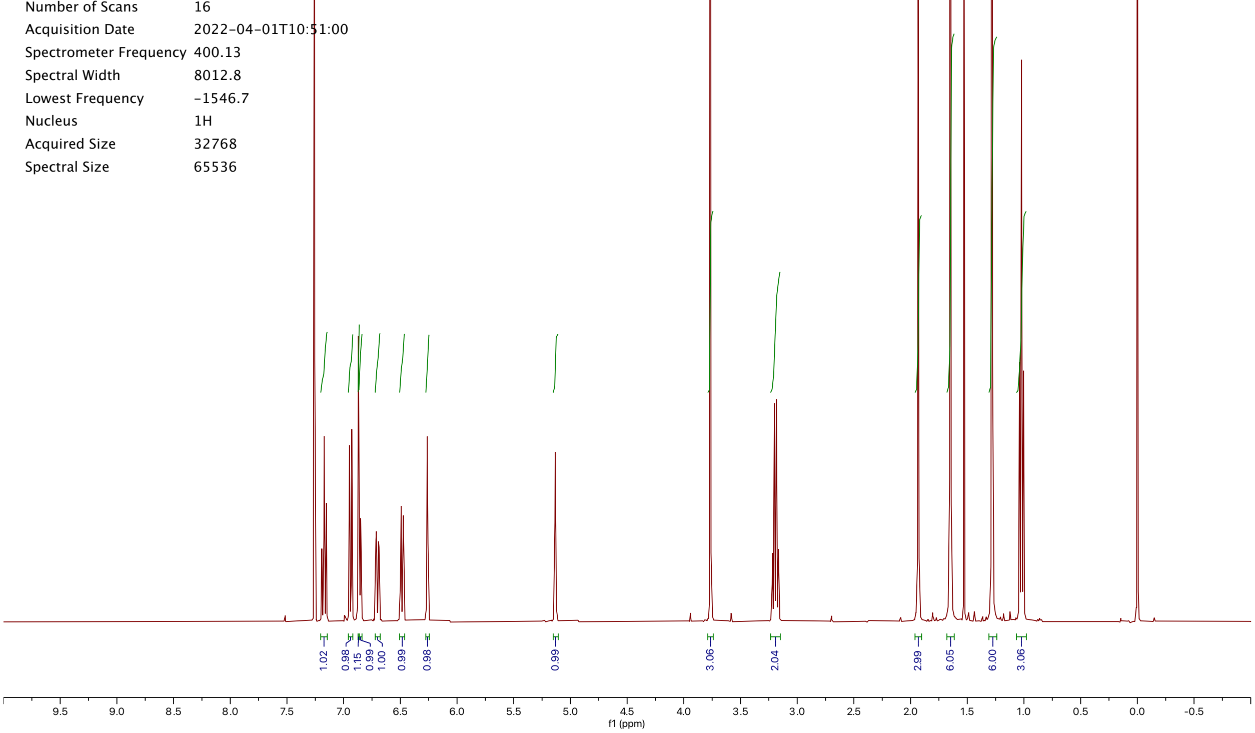
Origin Bruker BioSpin GmbH
Solvent CDCl3
Temperature 300.0
Pulse Sequence zg30
Experiment 1D
Number of Scans 16
Acquisition Date 2022-04-01T10:51:00
Spectrometer Frequency 400.13
Spectral Width 8012.8
Lowest Frequency -1546.7
Nucleus 1H
Acquired Size 32768
Spectral Size 65536
1.02
0.98
1.15
0.99
1.00
0.99
0.98
0.99
3.06
2.04
2.99
6.05
6.00
3.06
f1 (ppm)

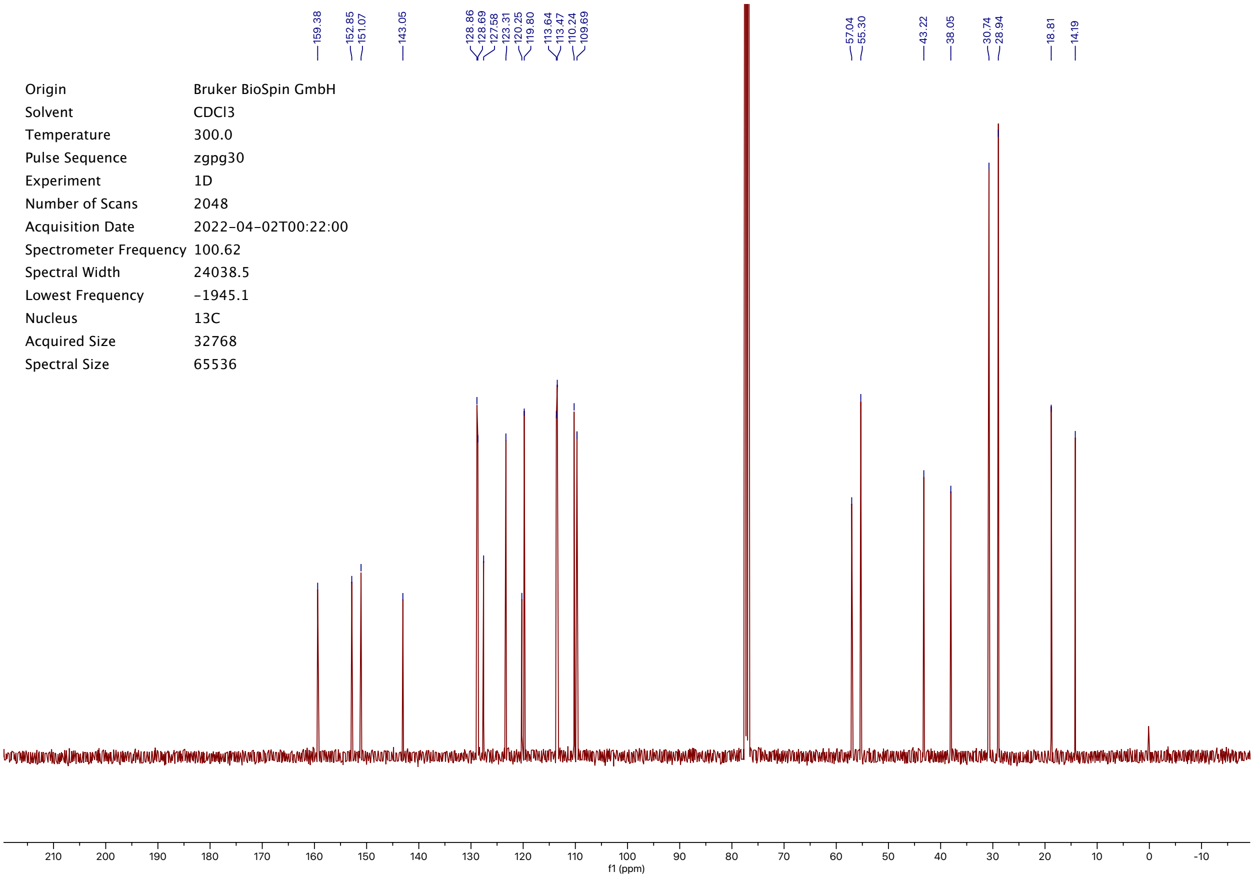
159.38
152.85
151.07
143.05
128.86
128.69
127.58
123.31
120.25
119.80
113.64
113.47
110.24
109.69
57.04
55.30
43.22
38.05
30.74
28.94
18.81
14.19
Origin Bruker BioSpin GmbH
Solvent CDCl3
Temperature 300.0
Pulse Sequence zgpg30
Experiment 1D
Number of Scans 2048
Acquisition Date 2022-04-02T00:22:00
Spectrometer Frequency 100.62
Spectral Width 24038.5
Lowest Frequency -1945.1
Nucleus 13C
Acquired Size 32768
Spectral Size 65536
f1 (ppm)

Origin	Bruker BioSpin GmbH
Solvent	CDCl3
Temperature	300.0
Pulse Sequence	zg30
Experiment	1D
Number of Scans	16
Acquisition Date	2022-05-12T16:42:00
Spectrometer Frequency	400.13
Spectral Width	8012.8
Lowest Frequency	-1547.4
Nucleus	1H
Acquired Size	32768
Spectral Size	65536

S42

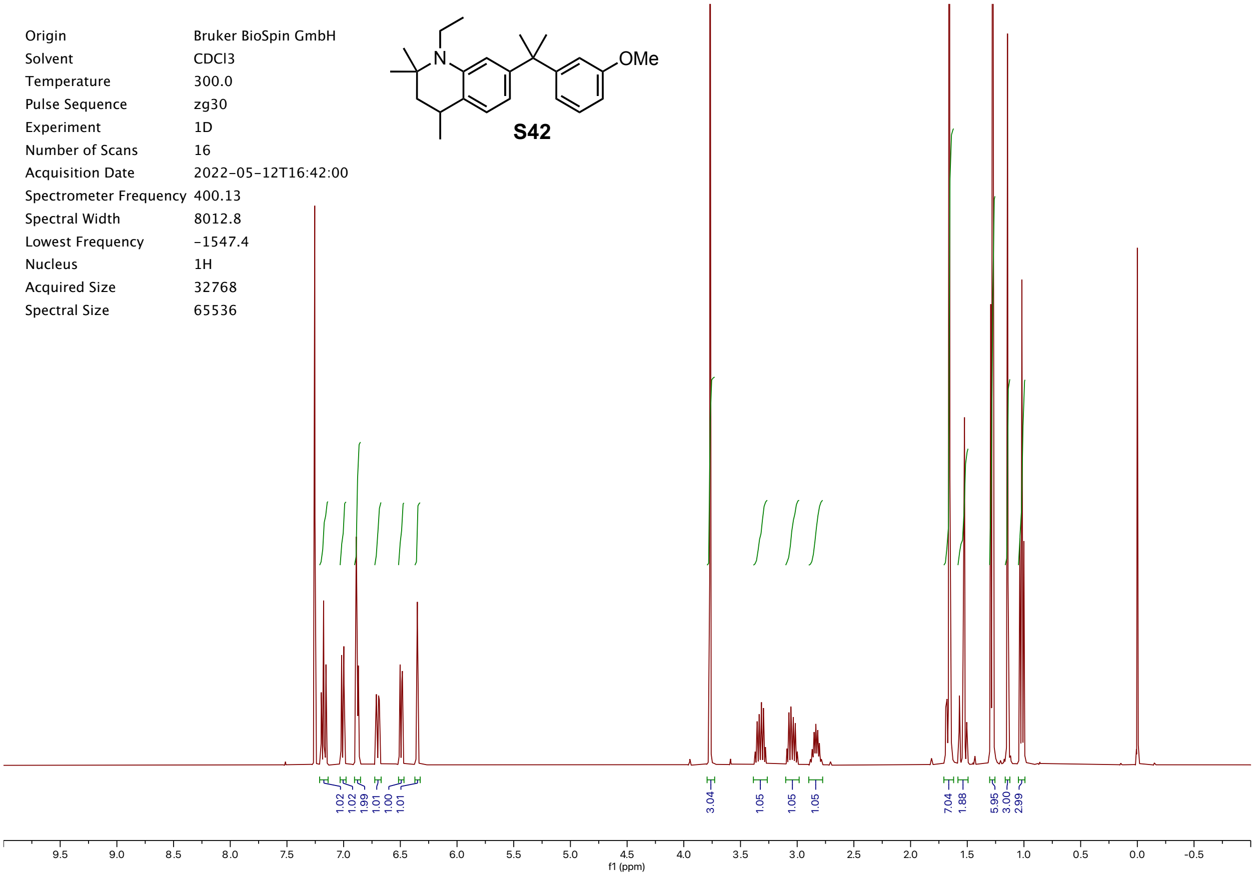

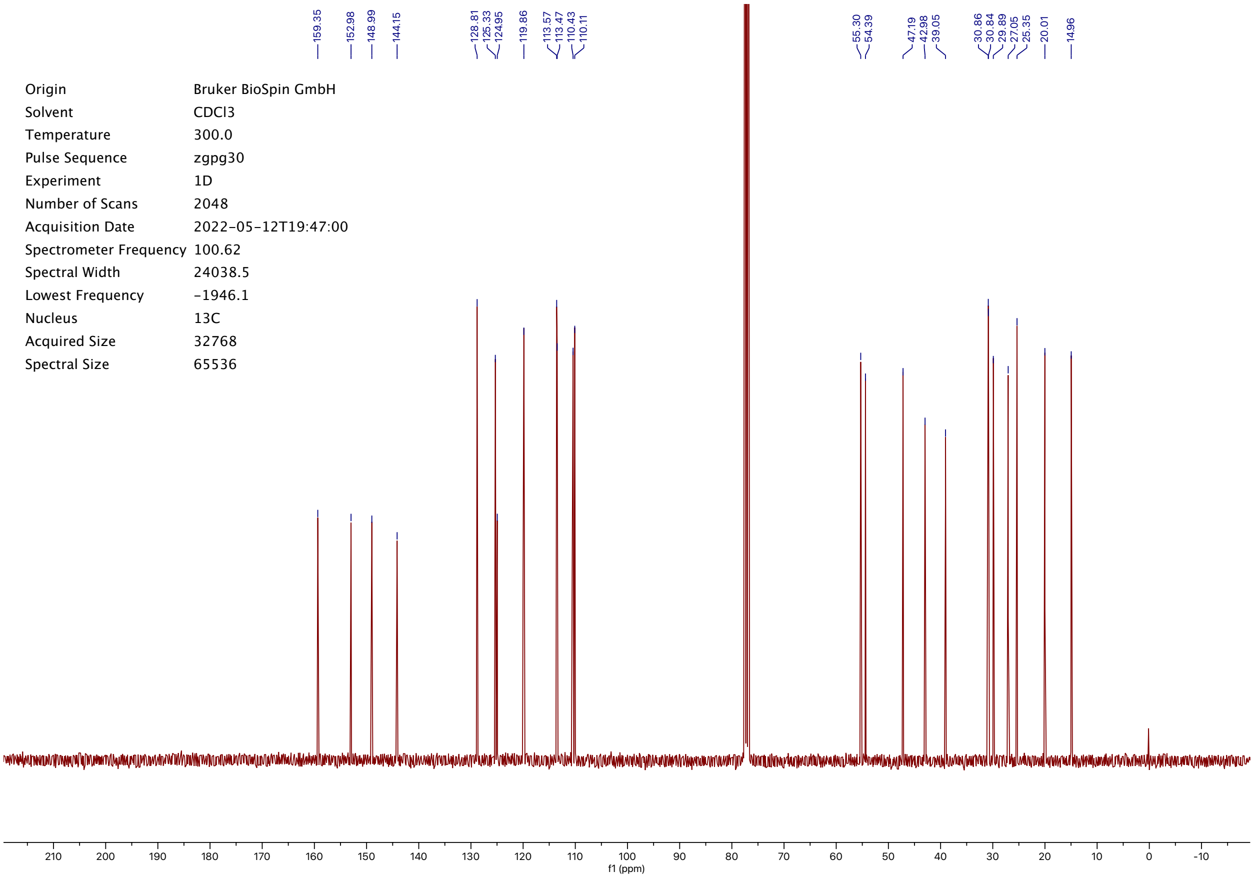

Origin	Bruker BioSpin GmbH
Solvent	CDCl3
Temperature	300.0
Pulse Sequence	zgpg30
Experiment	1D
Number of Scans	2048
Acquisition Date	2022-05-12T19:47:00
Spectrometer Frequency	100.62
Spectral Width	24038.5
Lowest Frequency	-1946.1
Nucleus	13C
Acquired Size	32768
Spectral Size	65536

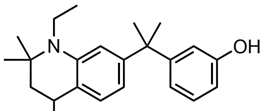
OH
N

S43

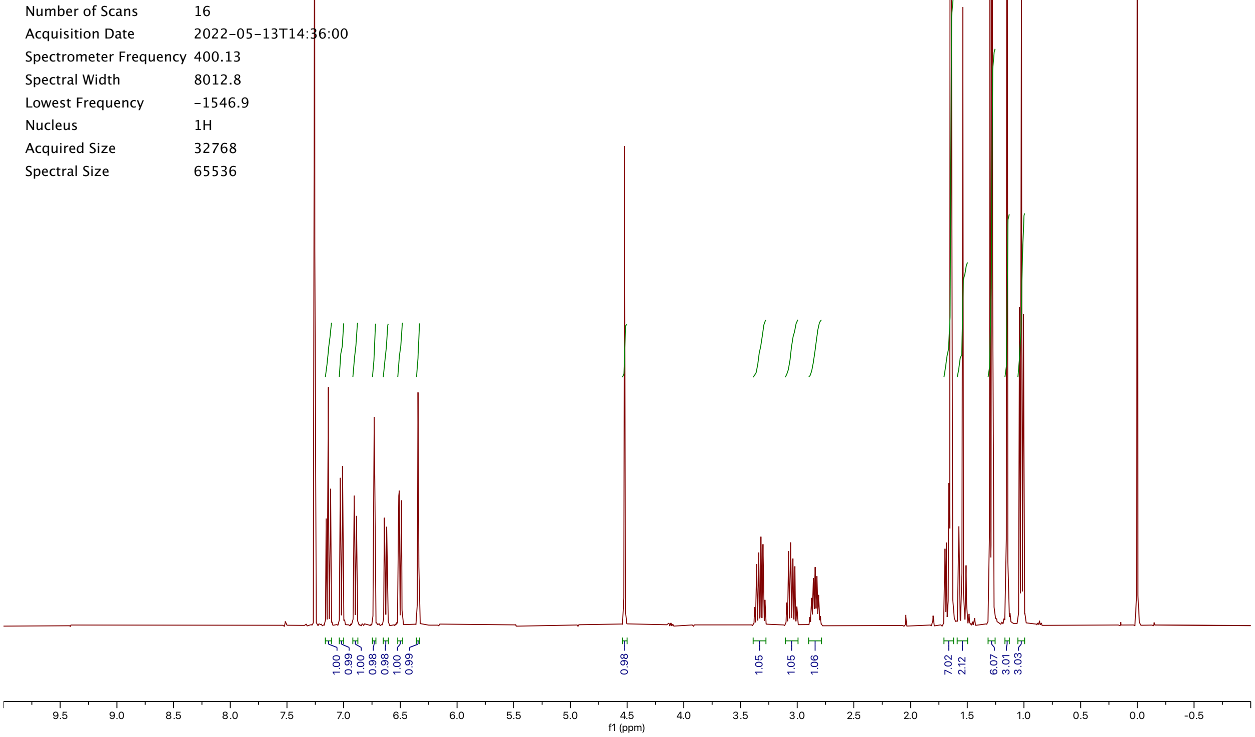
Origin Bruker BioSpin GmbH
Solvent CDCl3
Temperature 300.0
Pulse Sequence zg30
Experiment 1D
Number of Scans 16
Acquisition Date 2022-05-13T14:36:00
Spectrometer Frequency 400.13
Spectral Width 8012.8
Lowest Frequency -1546.9
Nucleus 1H
Acquired Size 32768
Spectral Size 65536
1.00 0.99 1.00 0.98 0.98 1.00 0.99
0.98
1.05 1.05 1.06
7.02 2.12 6.07 3.01 3.03
f1 (ppm)

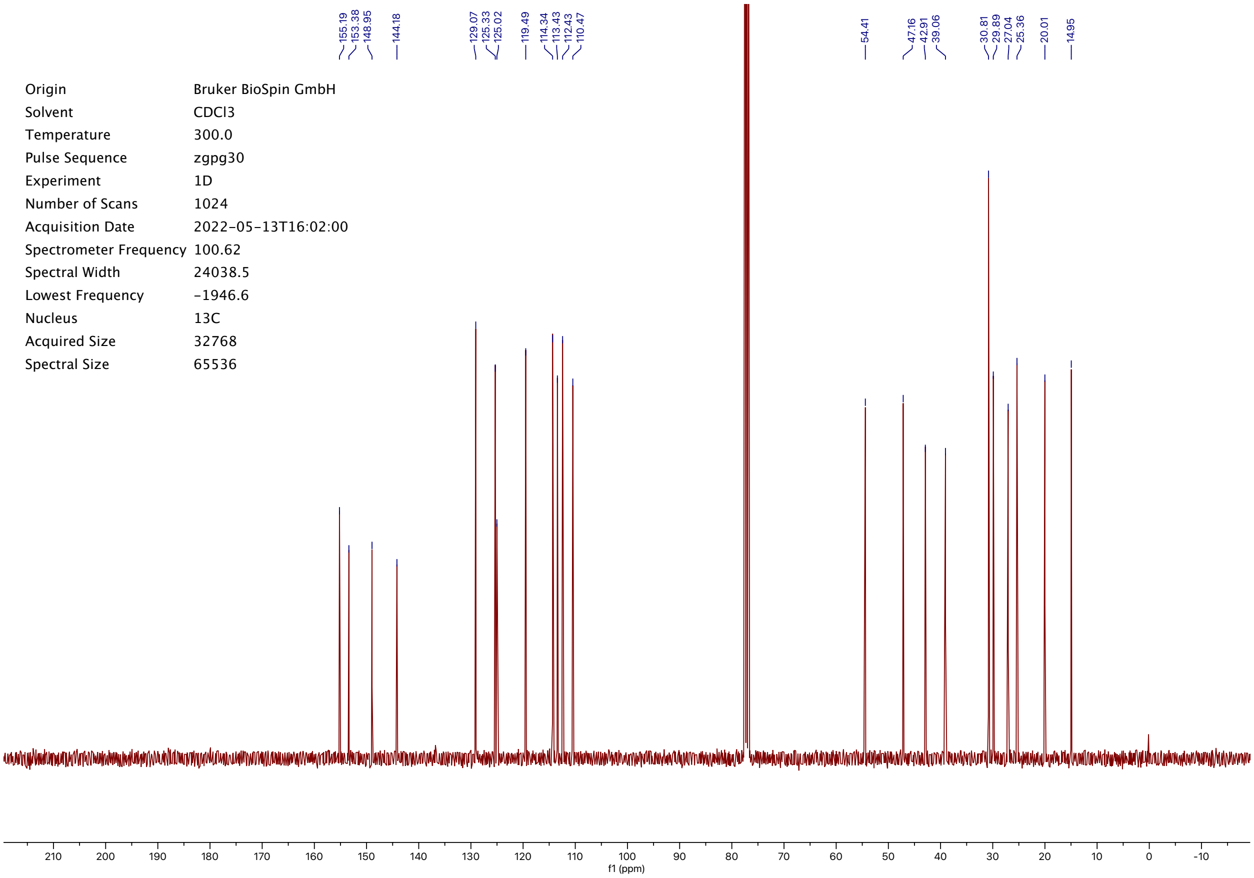
155.19 153.38 148.95 144.18
129.07 125.33 125.02 119.49 114.34 113.43 112.43 110.47
54.41 47.16 42.91 39.06 30.81 29.89 27.04 25.36 20.01 14.95
Origin Bruker BioSpin GmbH
Solvent CDCl3
Temperature 300.0
Pulse Sequence zgpg30
Experiment 1D
Number of Scans 1024
Acquisition Date 2022-05-13T16:02:00
Spectrometer Frequency 100.62
Spectral Width 24038.5
Lowest Frequency -1946.6
Nucleus 13C
Acquired Size 32768
Spectral Size 65536
f1 (ppm)

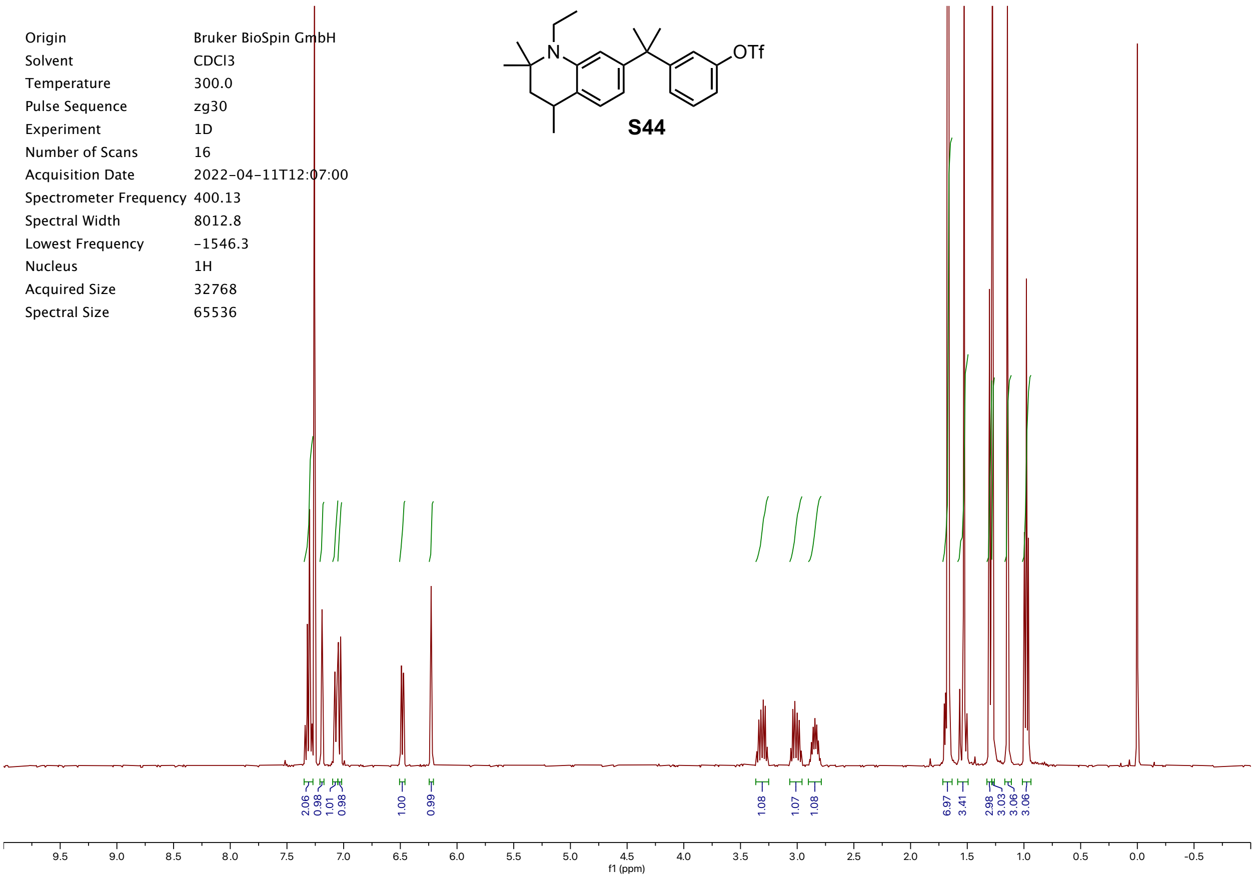
Origin Bruker BioSpin GmbH
Solvent CDCl3
Temperature 300.0
Pulse Sequence zg30
Experiment 1D
Number of Scans 16
Acquisition Date 2022-04-11T12:07:00
Spectrometer Frequency 400.13
Spectral Width 8012.8
Lowest Frequency -1546.3
Nucleus 1H
Acquired Size 32768
Spectral Size 65536
N
OTf
S44
2.06 0.98 1.01 0.98 1.00 0.99 1.08 1.07 1.08 6.97 3.41 2.98 3.03 3.06 3.06
f1 (ppm)

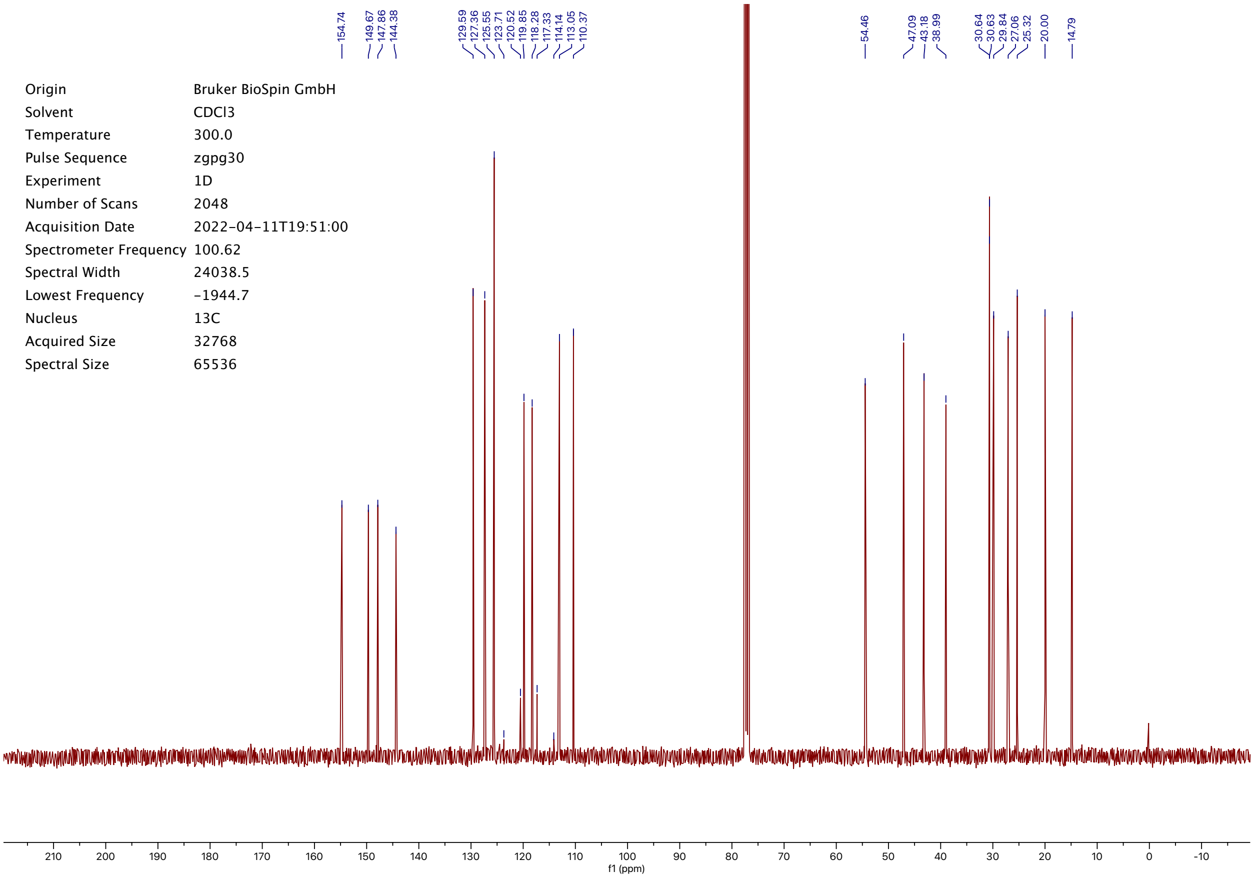
154.74
149.67 147.86 144.38
129.59 127.36 125.55 123.71 120.52 119.85 118.28 117.33 114.14 113.05 110.37
54.46
47.09 43.18 38.99
30.64 30.63 29.84 27.06 25.32 20.00
14.79
Origin Bruker BioSpin GmbH
Solvent CDCl3
Temperature 300.0
Pulse Sequence zgpg30
Experiment 1D
Number of Scans 2048
Acquisition Date 2022-04-11T19:51:00
Spectrometer Frequency 100.62
Spectral Width 24038.5
Lowest Frequency -1944.7
Nucleus 13C
Acquired Size 32768
Spectral Size 65536
f1 (ppm)

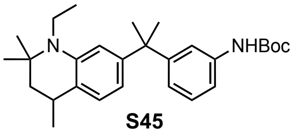

S45

Origin	Bruker BioSpin GmbH
Solvent	CDCl3
Temperature	300.0
Pulse Sequence	zg30
Experiment	1D
Number of Scans	16
Acquisition Date	2022-05-13T08:50:00
Spectrometer Frequency	400.13
Spectral Width	8012.8
Lowest Frequency	-1549.1
Nucleus	1H
Acquired Size	32768
Spectral Size	65536

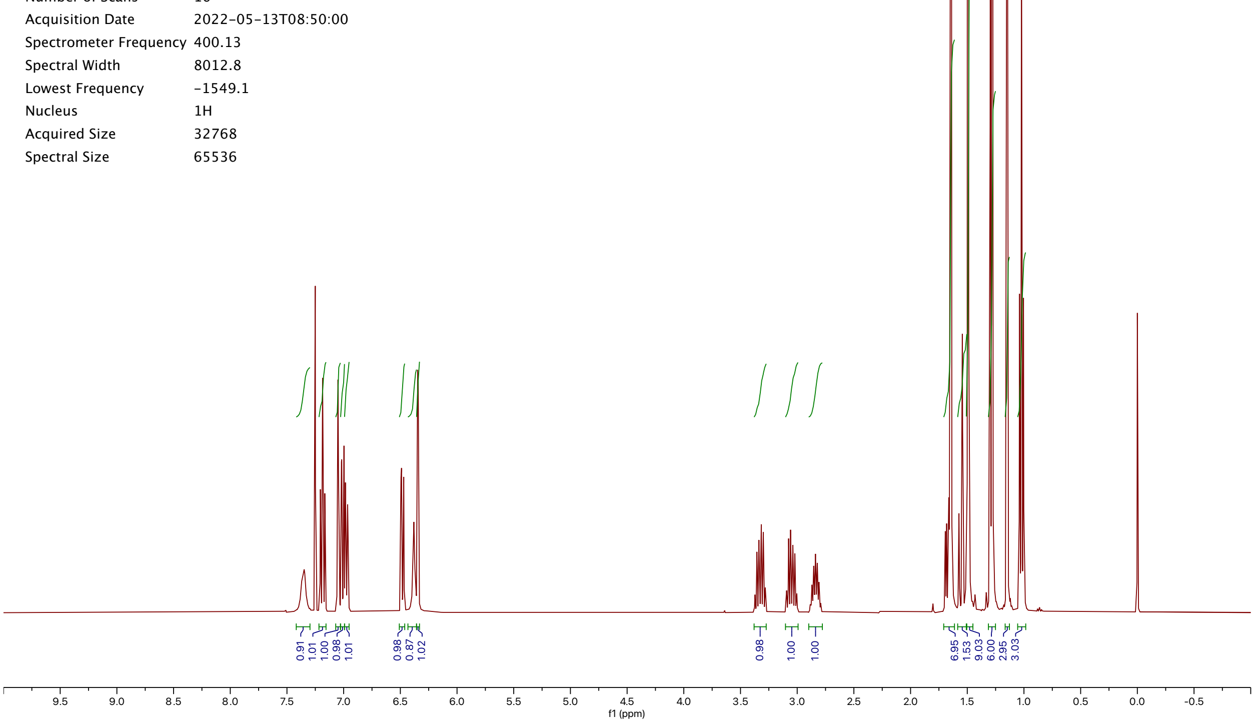

Origin	Bruker BioSpin GmbH
Solvent	CDCl3
Temperature	300.0
Pulse Sequence	zgpg30
Experiment	1D
Number of Scans	4096
Acquisition Date	2022-05-13T21:22:00
Spectrometer Frequency	100.62
Spectral Width	24038.5
Lowest Frequency	-1945.1
Nucleus	13C
Acquired Size	32768
Spectral Size	65536

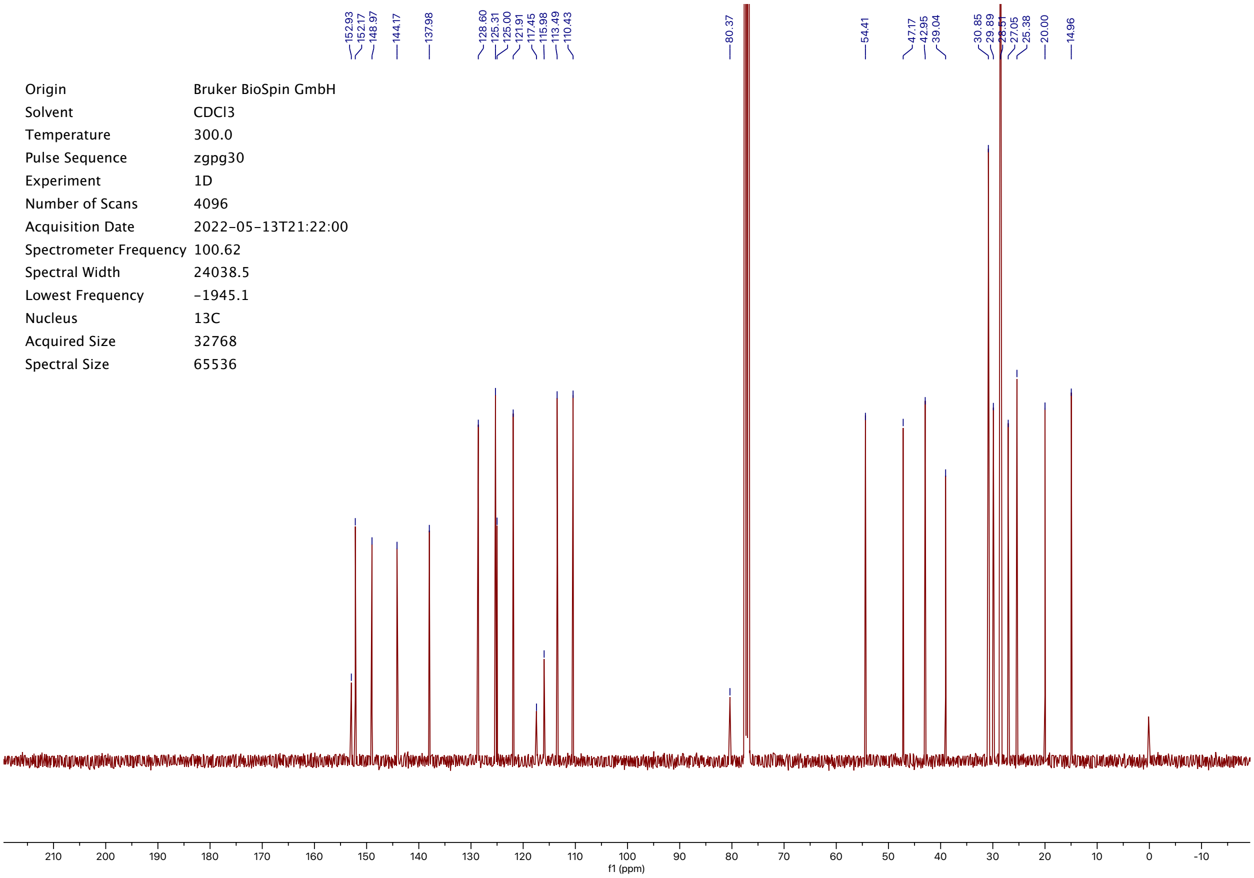

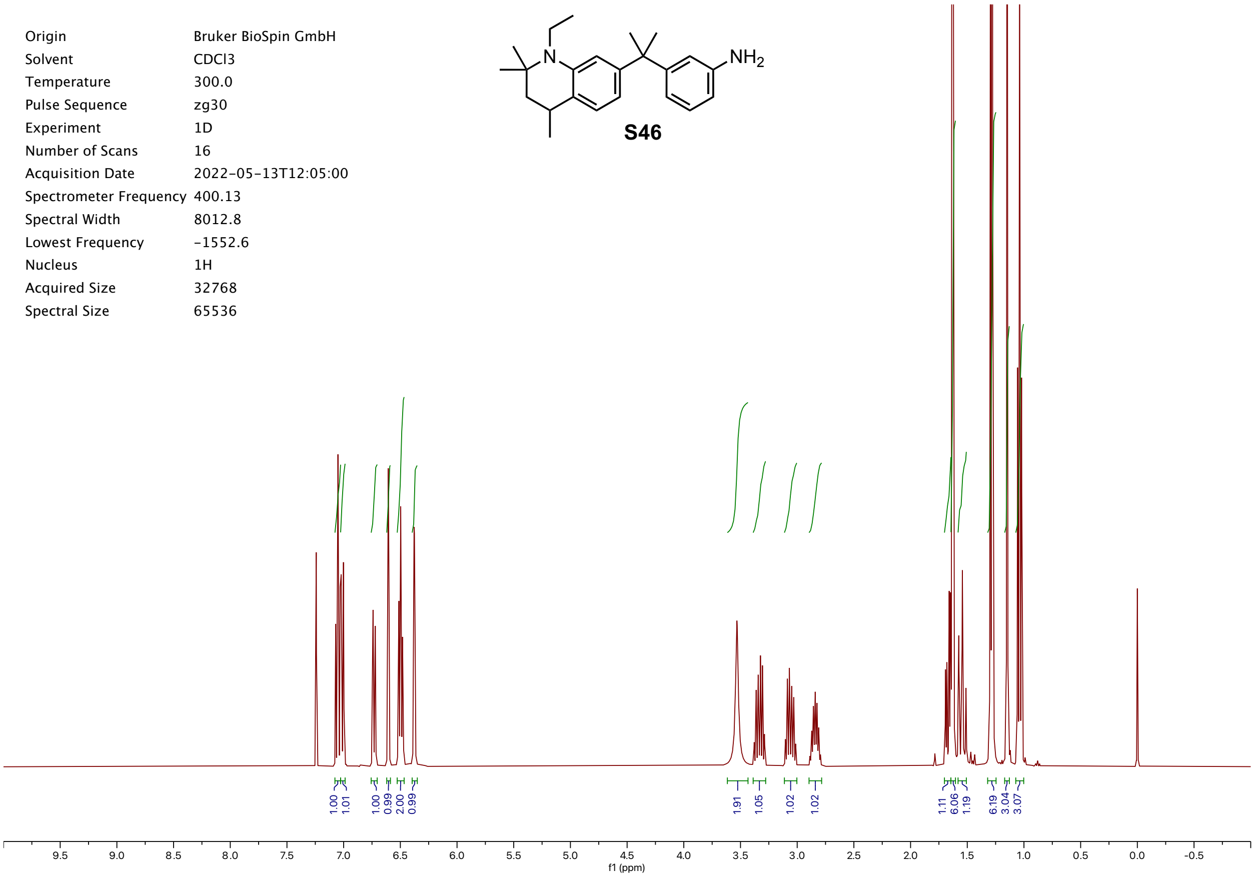
Origin Bruker BioSpin GmbH
Solvent CDCl3
Temperature 300.0
Pulse Sequence zg30
Experiment 1D
Number of Scans 16
Acquisition Date 2022-05-13T12:05:00
Spectrometer Frequency 400.13
Spectral Width 8012.8
Lowest Frequency -1552.6
Nucleus 1H
Acquired Size 32768
Spectral Size 65536
N
NH2
S46
1.00 1.01 1.00 0.99 2.00 0.99 1.91 1.05 1.02 1.02 1.11 6.06 1.19 6.19 3.04 3.07
f1 (ppm)

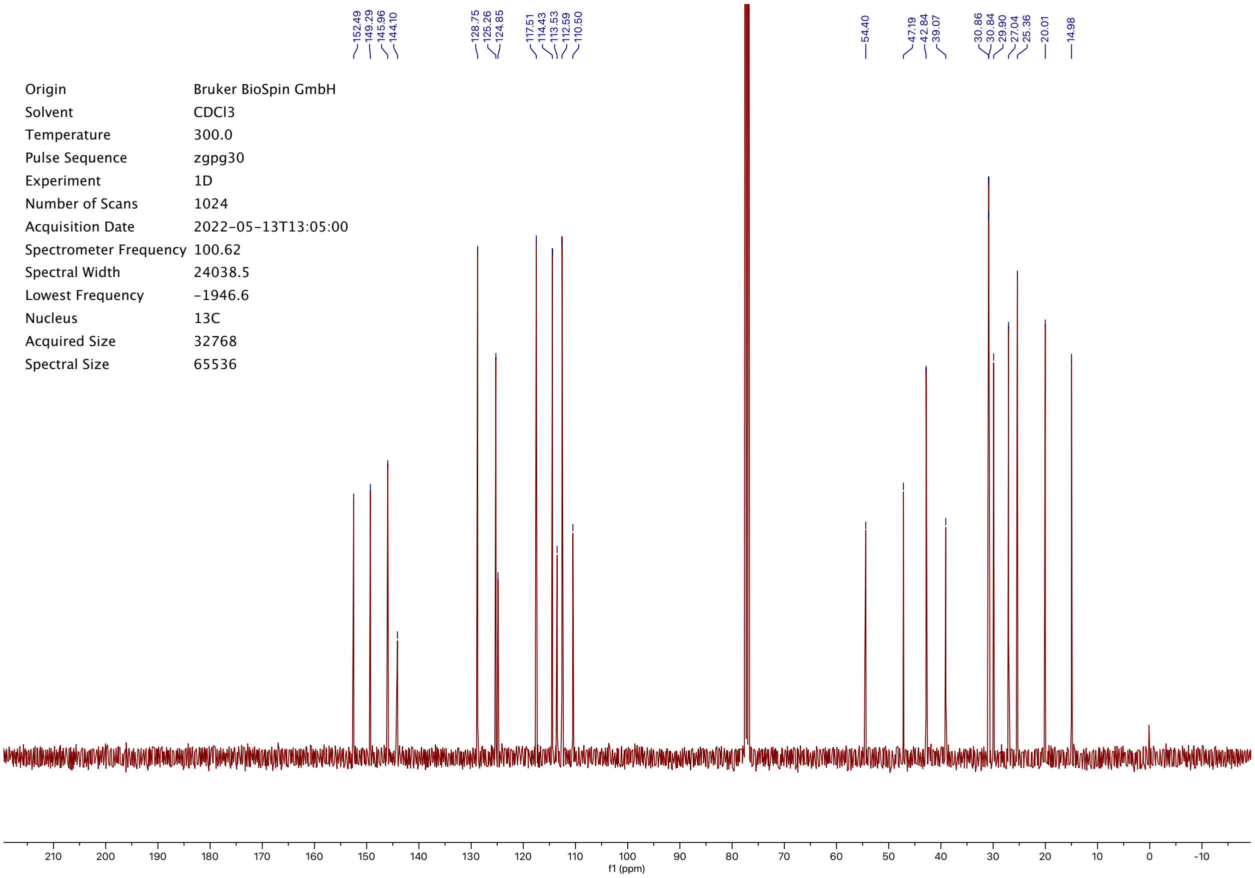
152.49 149.29 145.96 144.10 128.75 125.26 124.85 117.51 114.43 113.53 112.59 110.50 54.40 47.19 42.84 39.07 30.86 30.84 29.90 27.04 25.36 20.01 14.98
Origin Bruker BioSpin GmbH
Solvent CDCl3
Temperature 300.0
Pulse Sequence zgpg30
Experiment 1D
Number of Scans 1024
Acquisition Date 2022-05-13T13:05:00
Spectrometer Frequency 100.62
Spectral Width 24038.5
Lowest Frequency -1946.6
Nucleus 13C
Acquired Size 32768
Spectral Size 65536
f1 (ppm)

Origin	Bruker BioSpin GmbH
Solvent	CDCl3
Temperature	300.0
Pulse Sequence	zg30
Experiment	1D
Number of Scans	16
Acquisition Date	2022-05-12T10:55:00
Spectrometer Frequency	400.13
Spectral Width	8012.8
Lowest Frequency	-1547.0
Nucleus	1H
Acquired Size	32768
Spectral Size	65536

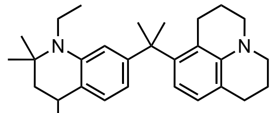

58

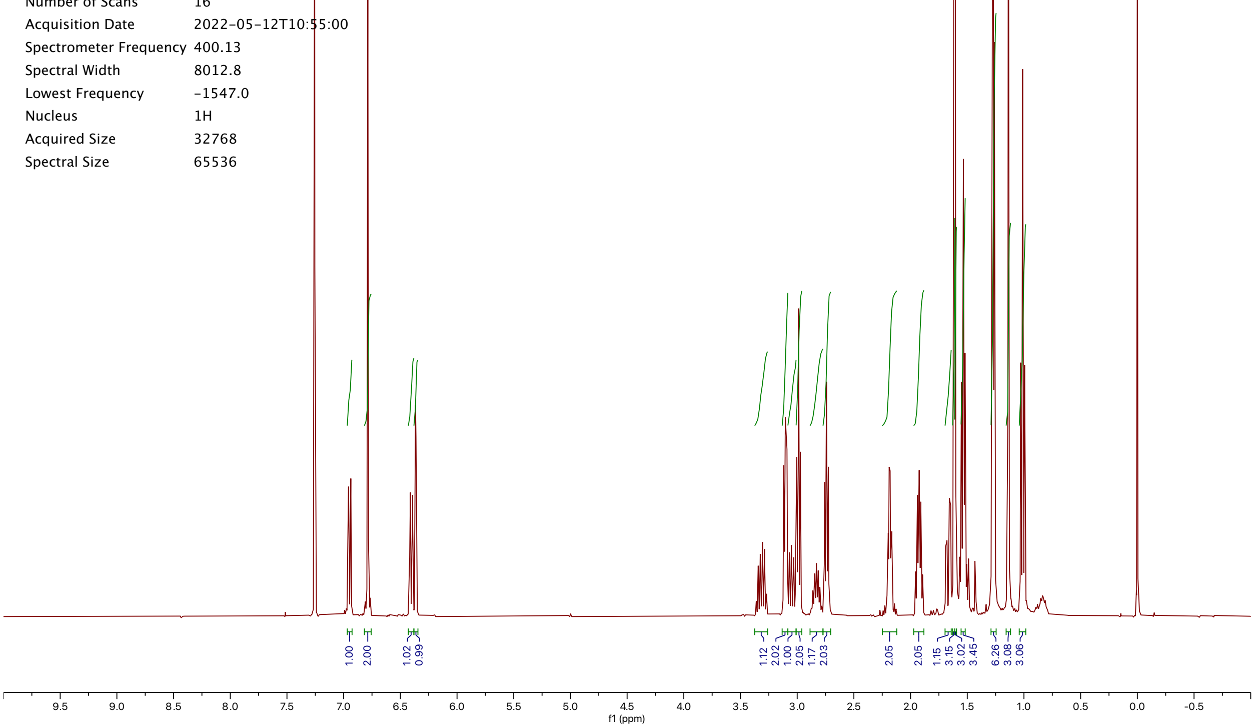

Origin	Bruker BioSpin GmbH
Solvent	CDCl3
Temperature	300.0
Pulse Sequence	zgpg30
Experiment	1D
Number of Scans	1024
Acquisition Date	2022-05-12T14:54:00
Spectrometer Frequency	100.62
Spectral Width	24038.5
Lowest Frequency	-1945.8
Nucleus	13C
Acquired Size	32768
Spectral Size	65536

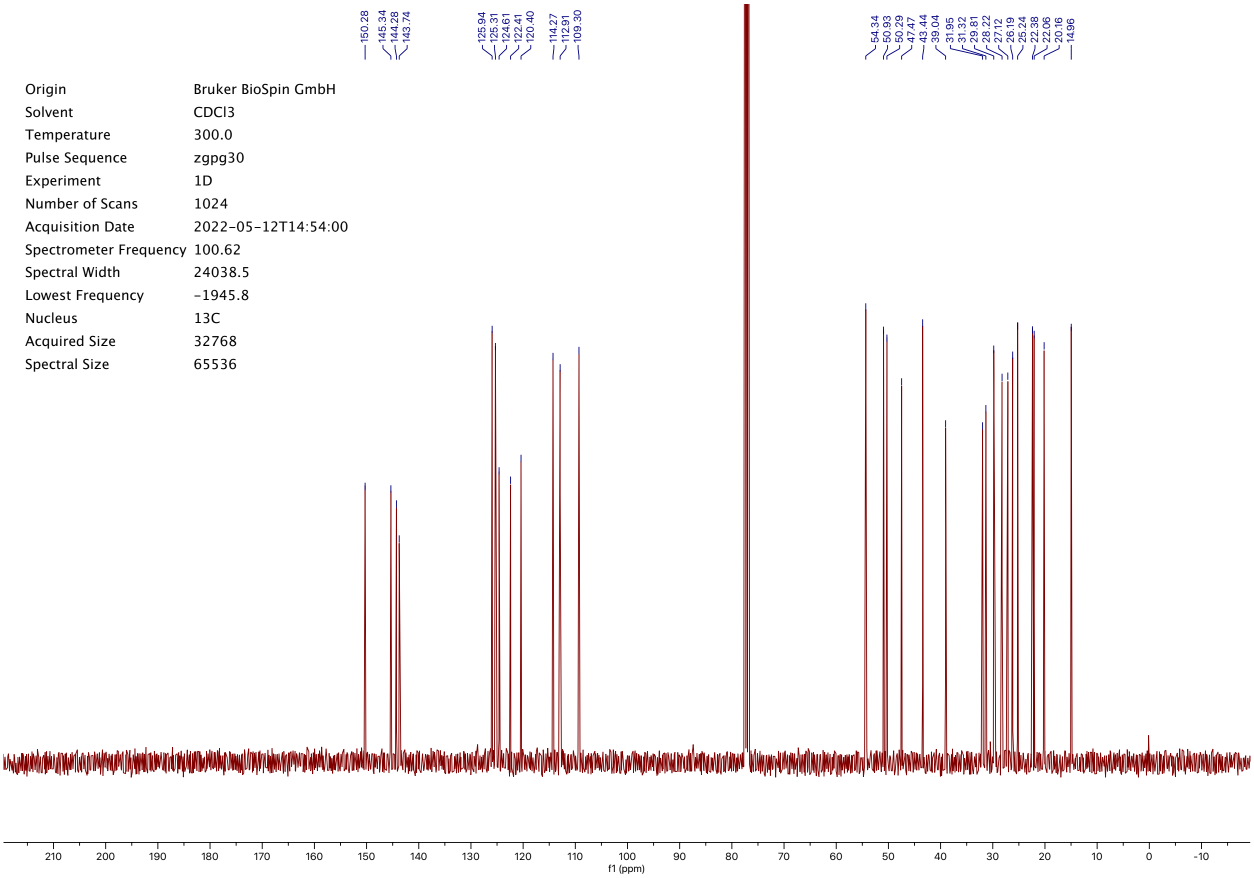

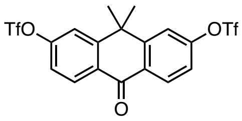
TfO
OTf
O

S48

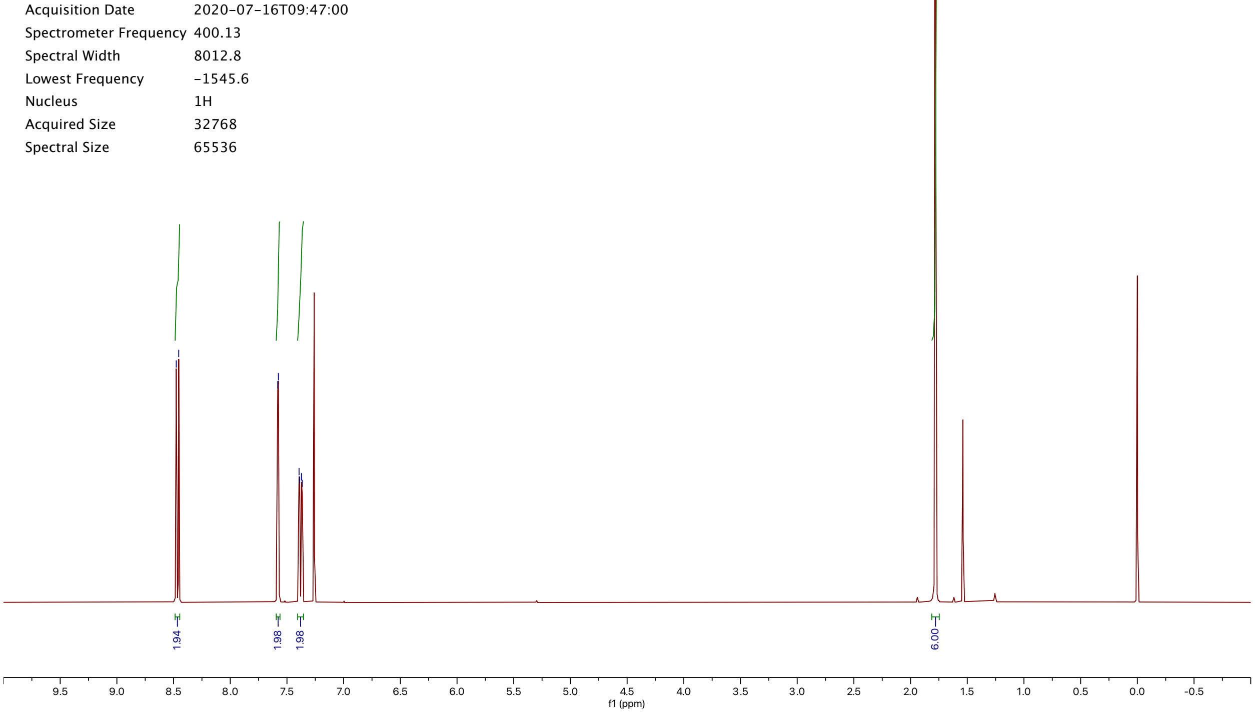
Origin Bruker BioSpin GmbH
Solvent CDCl3
Temperature 300.0
Pulse Sequence zg30
Experiment 1D
Number of Scans 16
Acquisition Date 2020-07-16T09:47:00
Spectrometer Frequency 400.13
Spectral Width 8012.8
Lowest Frequency -1545.6
Nucleus 1H
Acquired Size 32768
Spectral Size 65536
1.94
1.98
1.98
6.00
f1 (ppm)

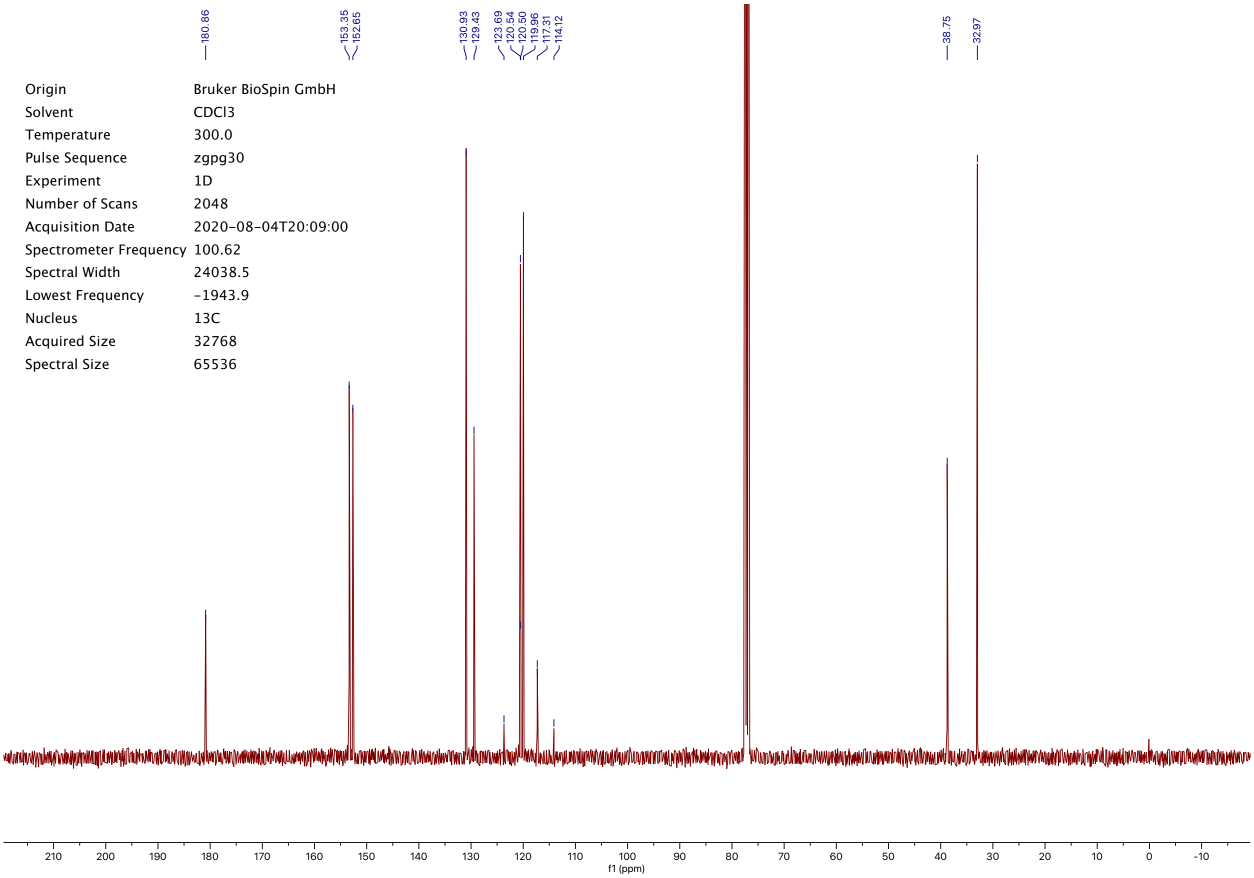
180.86
153.35
152.65
130.93
129.43
123.69
120.54
120.50
119.96
117.31
114.12
38.75
32.97
Origin Bruker BioSpin GmbH
Solvent CDCl3
Temperature 300.0
Pulse Sequence zgpg30
Experiment 1D
Number of Scans 2048
Acquisition Date 2020-08-04T20:09:00
Spectrometer Frequency 100.62
Spectral Width 24038.5
Lowest Frequency -1943.9
Nucleus 13C
Acquired Size 32768
Spectral Size 65536
f1 (ppm)

Origin	Bruker BioSpin GmbH
Solvent	CDCl3
Temperature	295.4
Pulse Sequence	zg30
Experiment	1D
Number of Scans	16
Acquisition Date	2020-12-04T17:33:00
Spectrometer Frequency	400.13
Spectral Width	8012.8
Lowest Frequency	-1545.4
Nucleus	1H
Acquired Size	32768
Spectral Size	65536

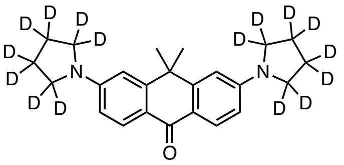

42

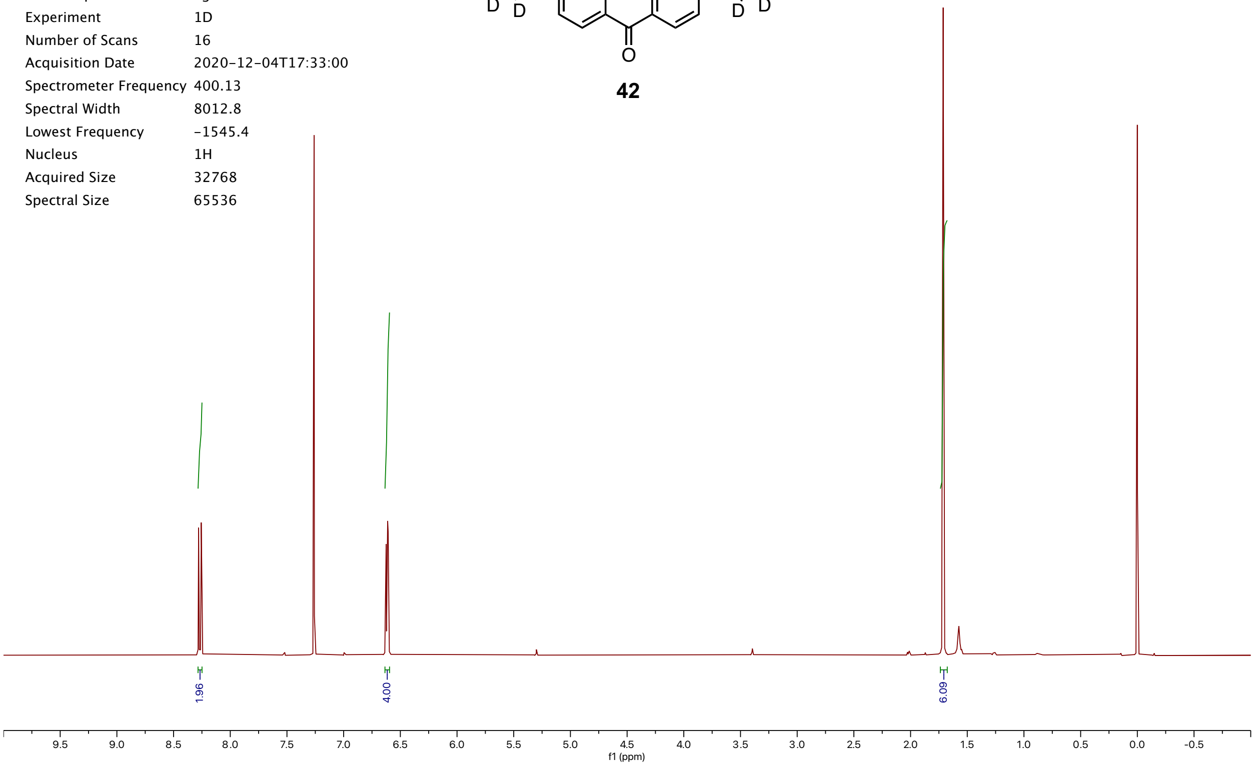

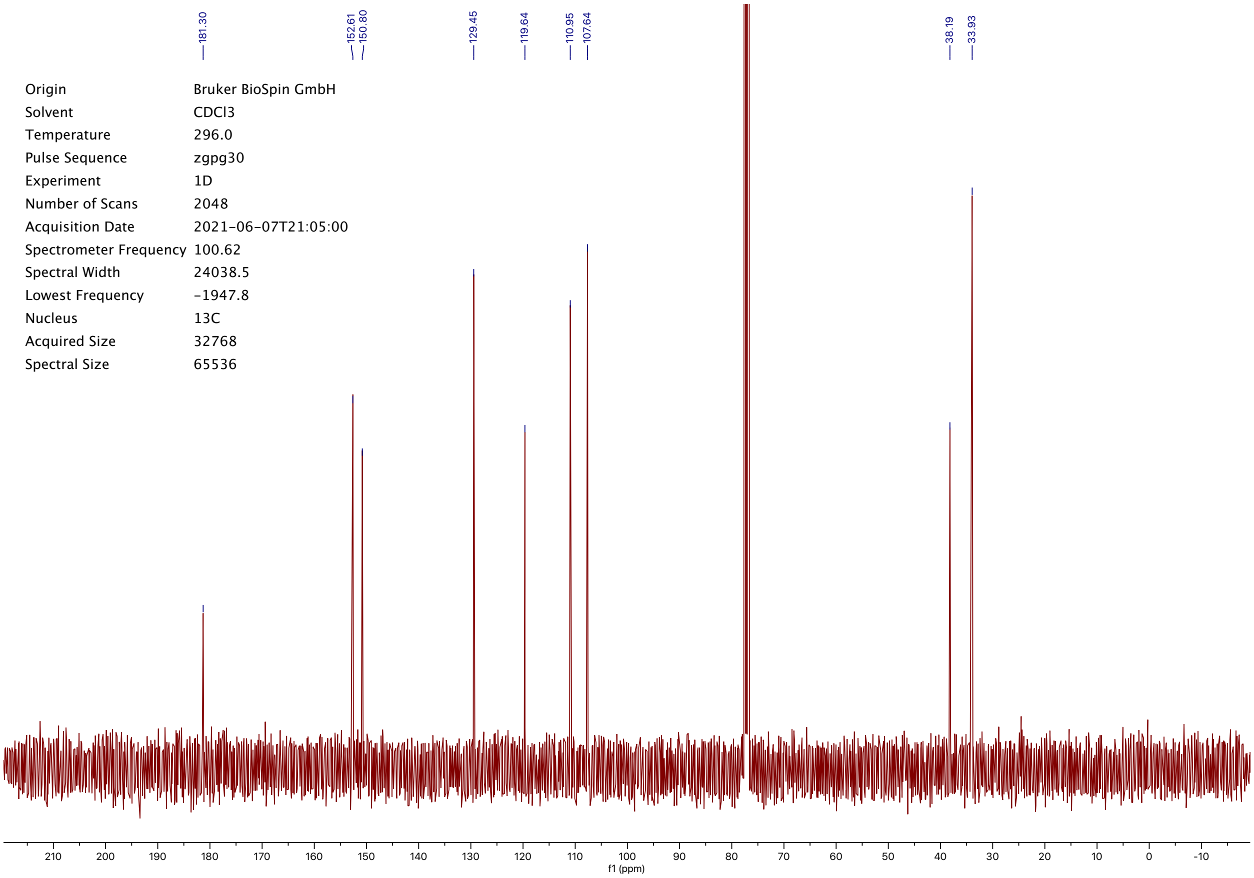

Origin	Bruker BioSpin GmbH
Solvent	CDCl3
Temperature	296.0
Pulse Sequence	zgpg30
Experiment	1D
Number of Scans	2048
Acquisition Date	2021-06-07T21:05:00
Spectrometer Frequency	100.62
Spectral Width	24038.5
Lowest Frequency	-1947.8
Nucleus	13C
Acquired Size	32768
Spectral Size	65536

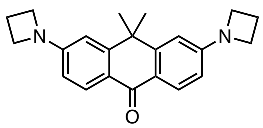

43

Origin	Bruker BioSpin GmbH
Solvent	CDCl3
Temperature	295.3
Pulse Sequence	zg30
Experiment	1D
Number of Scans	16
Acquisition Date	2020-11-10T12:28:00
Spectrometer Frequency	400.13
Spectral Width	8012.8
Lowest Frequency	-1545.7
Nucleus	1H
Acquired Size	32768
Spectral Size	65536

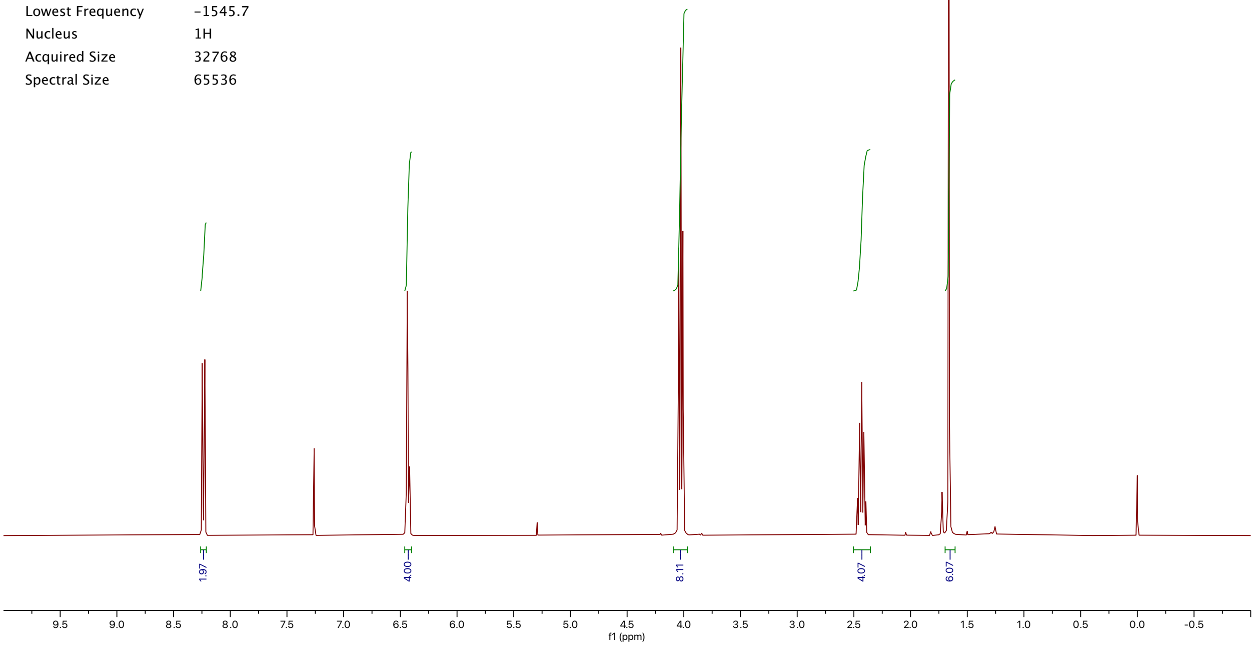

Origin	Bruker BioSpin GmbH
Solvent	CDCl3
Temperature	295.9
Pulse Sequence	zgpg30
Experiment	1D
Number of Scans	512
Acquisition Date	2020-11-10T12:59:00
Spectrometer Frequency	100.62
Spectral Width	24038.5
Lowest Frequency	-1948.3
Nucleus	13C
Acquired Size	32768
Spectral Size	65536

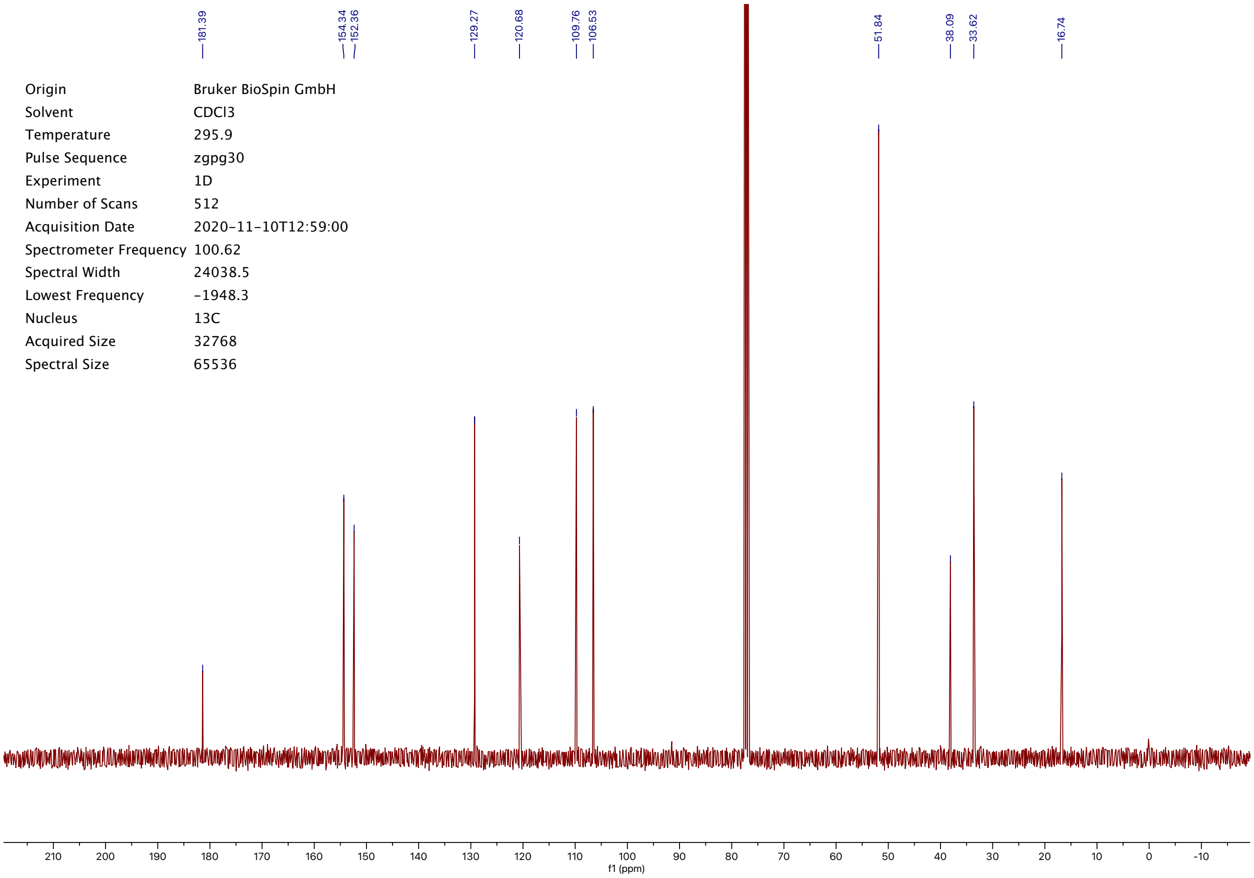

Origin	Bruker BioSpin GmbH
Solvent	CDCl3
Temperature	296.0
Pulse Sequence	zg30
Experiment	1D
Number of Scans	16
Acquisition Date	2012-03-16T09:11:00
Spectrometer Frequency	400.13
Spectral Width	8223.7
Lowest Frequency	-1647.6
Nucleus	1H
Acquired Size	32768
Spectral Size	65536

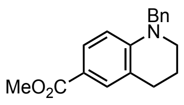

S50

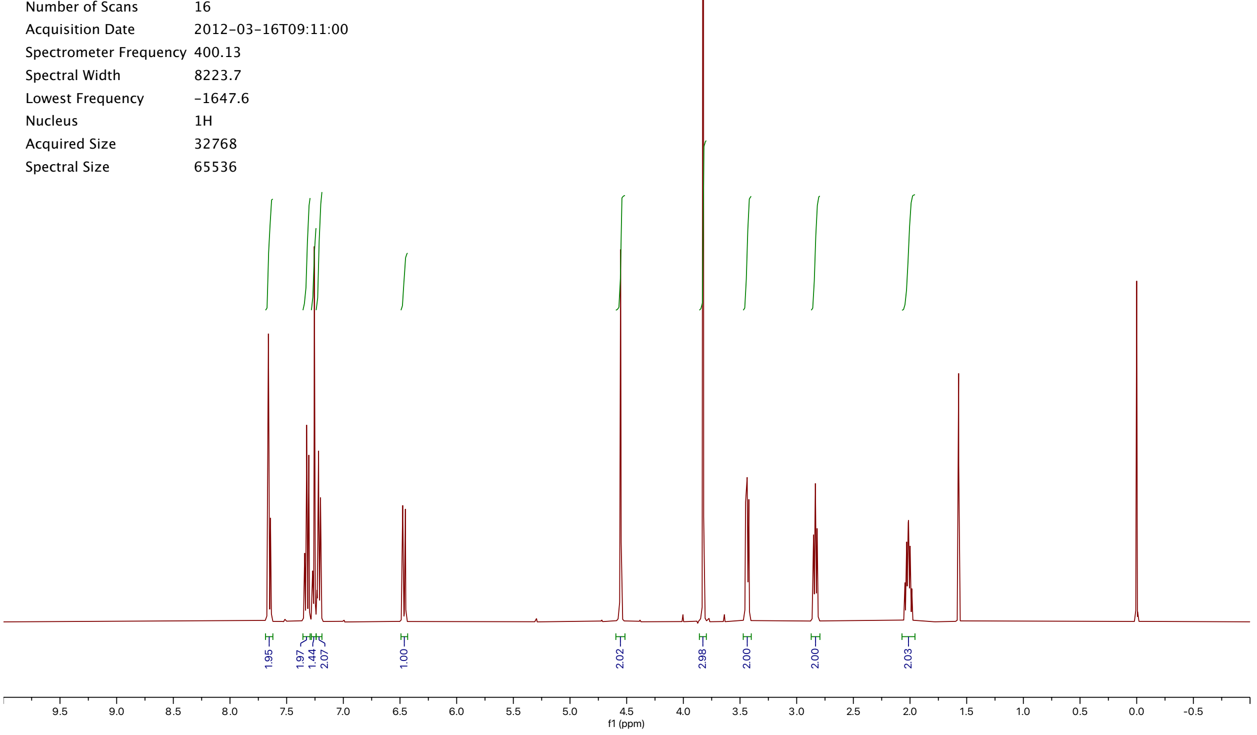

Origin	Bruker BioSpin GmbH
Solvent	CDCl3
Temperature	297.4
Pulse Sequence	zgpg30
Experiment	1D
Number of Scans	2048
Acquisition Date	2012-03-23T00:39:00
Spectrometer Frequency	100.62
Spectral Width	24038.5
Lowest Frequency	-1947.8
Nucleus	13C
Acquired Size	32768
Spectral Size	65536

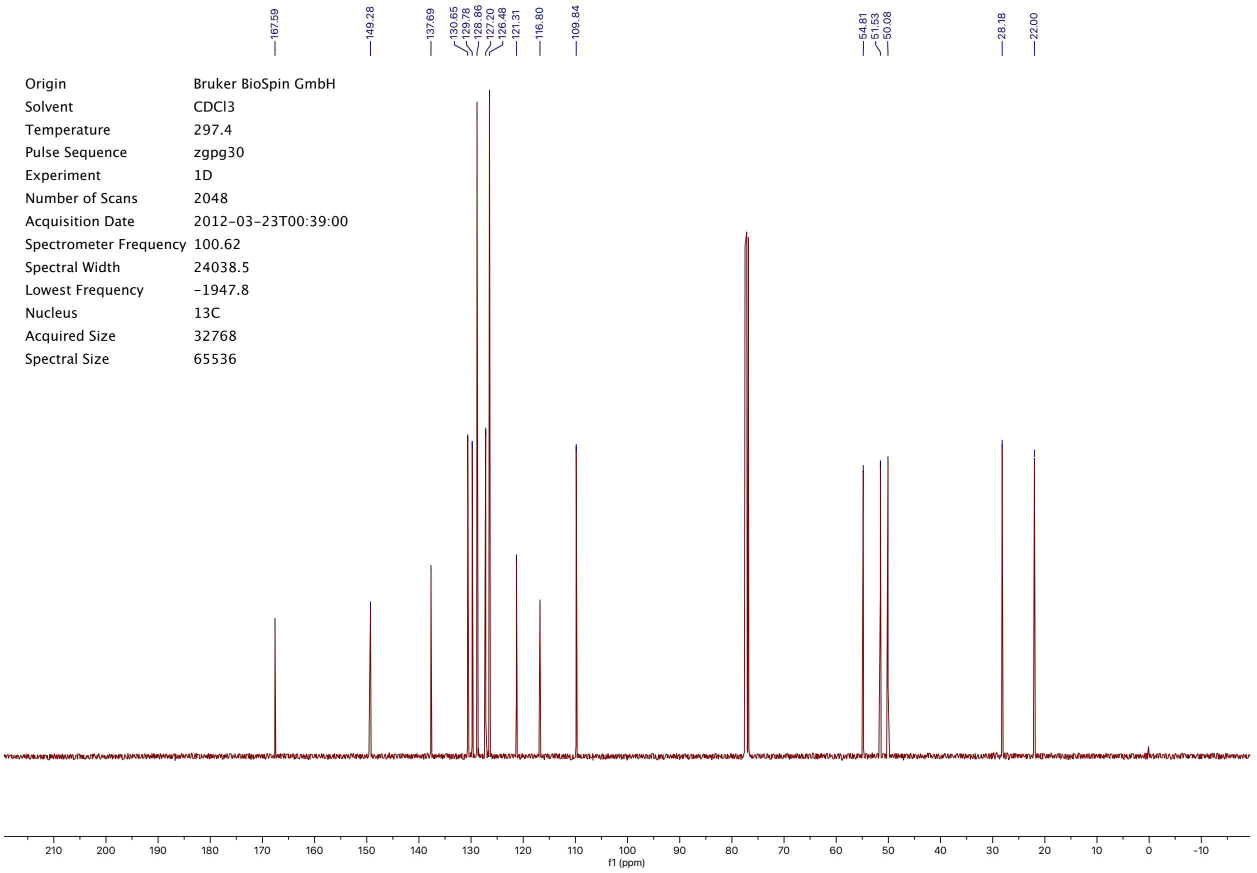

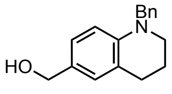

S51

Origin	Bruker BioSpin GmbH
Solvent	Acetone
Temperature	296.2
Pulse Sequence	zg30
Experiment	1D
Number of Scans	16
Acquisition Date	2012-03-22T17:27:00
Spectrometer Frequency	400.13
Spectral Width	8223.7
Lowest Frequency	-1645.7
Nucleus	1H
Acquired Size	32768
Spectral Size	65536

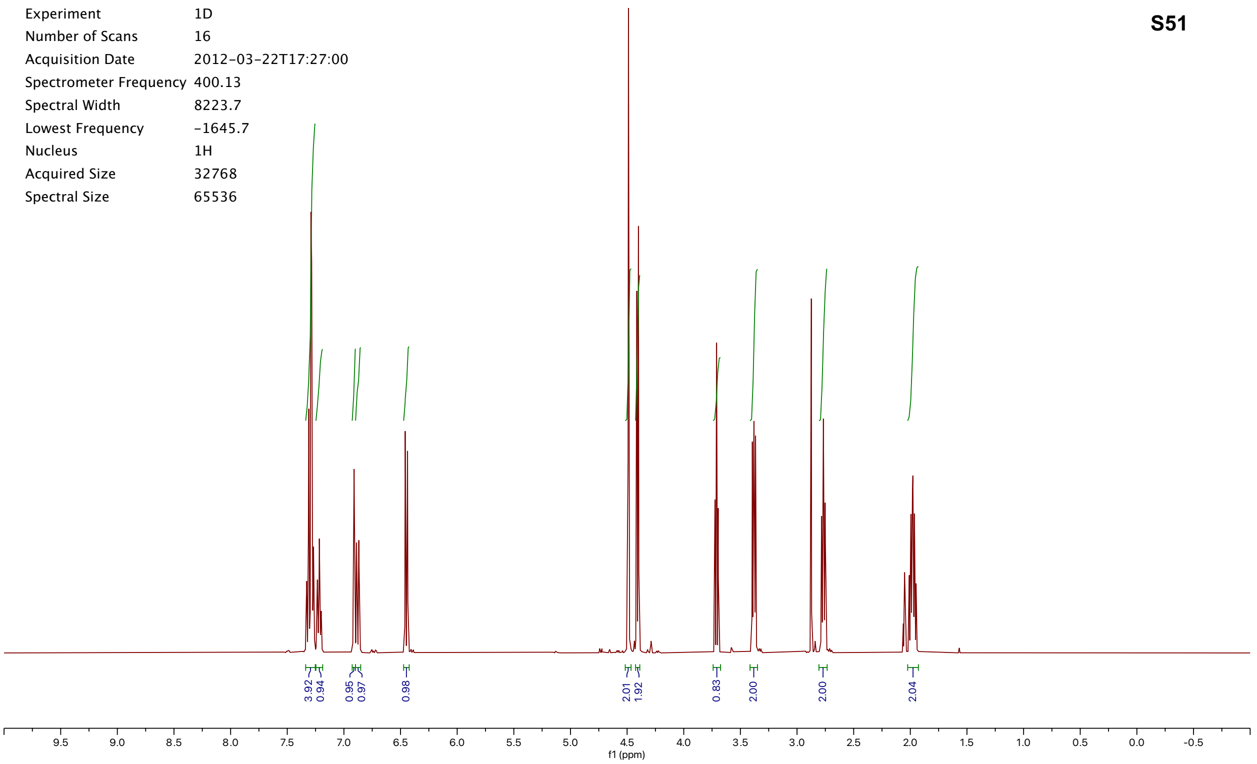

Origin	Bruker BioSpin GmbH
Solvent	Acetone
Temperature	297.3
Pulse Sequence	zgpg30
Experiment	1D
Number of Scans	2048
Acquisition Date	2012-03-22T19:26:00
Spectrometer Frequency	100.62
Spectral Width	24038.5
Lowest Frequency	-1870.9
Nucleus	13C
Acquired Size	32768
Spectral Size	65536

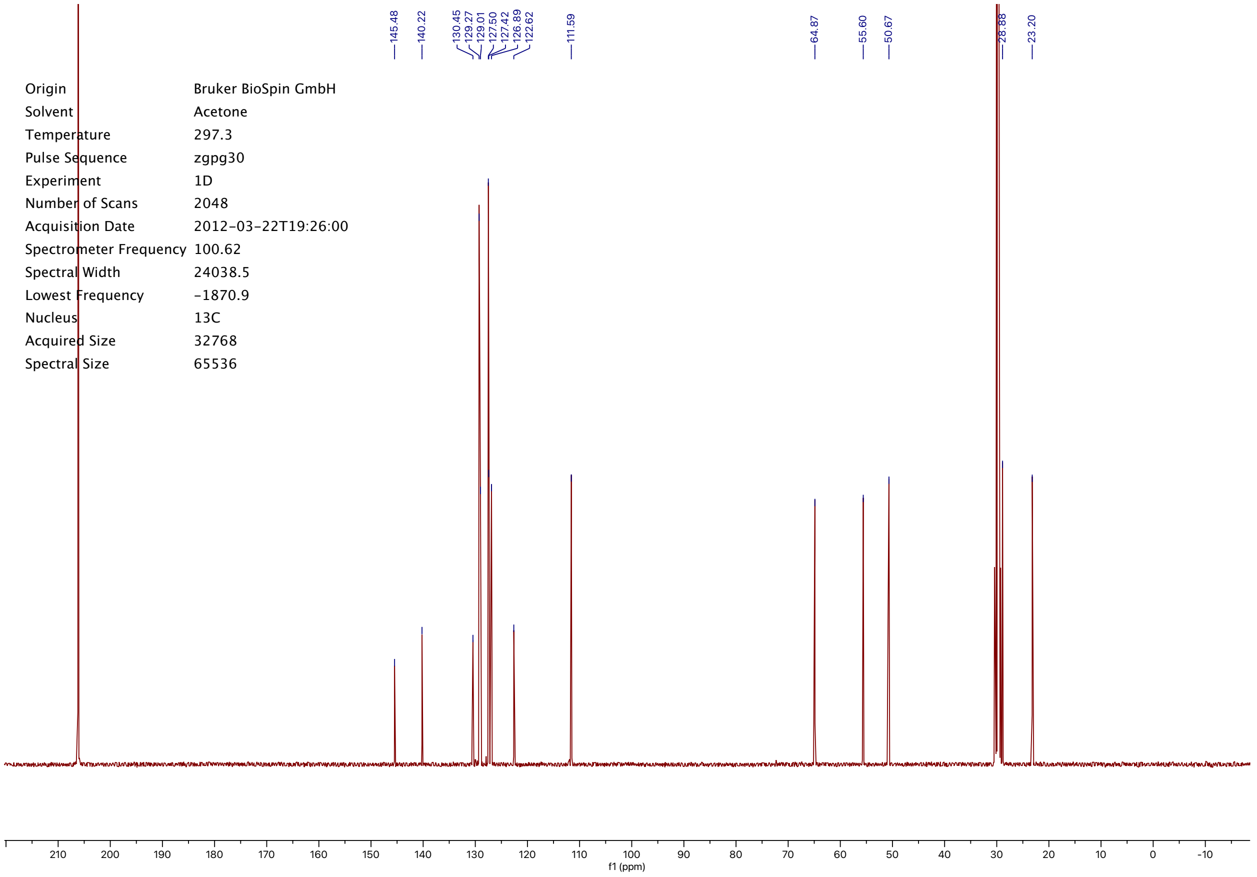

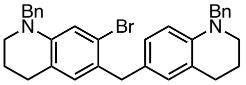

S52

Origin	Bruker BioSpin GmbH
Solvent	CDCl3
Temperature	300.0
Pulse Sequence	zg30
Experiment	1D
Number of Scans	16
Acquisition Date	2012-10-02T13:31:00
Spectrometer Frequency	400.13
Spectral Width	8223.7
Lowest Frequency	-1648.4
Nucleus	1H
Acquired Size	32768
Spectral Size	65536

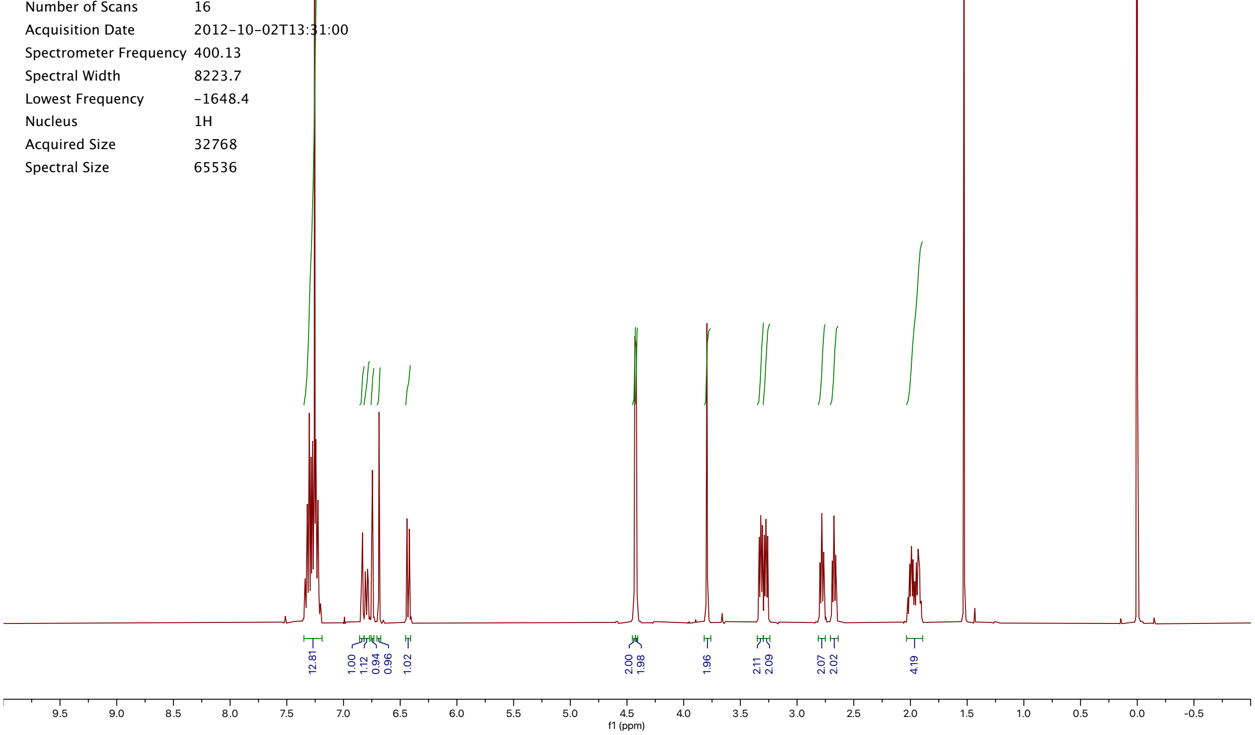

Origin	Bruker BioSpin GmbH
Solvent	CDCl3
Temperature	300.0
Pulse Sequence	zgpg30
Experiment	1D
Number of Scans	2048
Acquisition Date	2012-10-01T18:54:00
Spectrometer Frequency	100.62
Spectral Width	24038.5
Lowest Frequency	-1945.1
Nucleus	13C
Acquired Size	32768
Spectral Size	65536

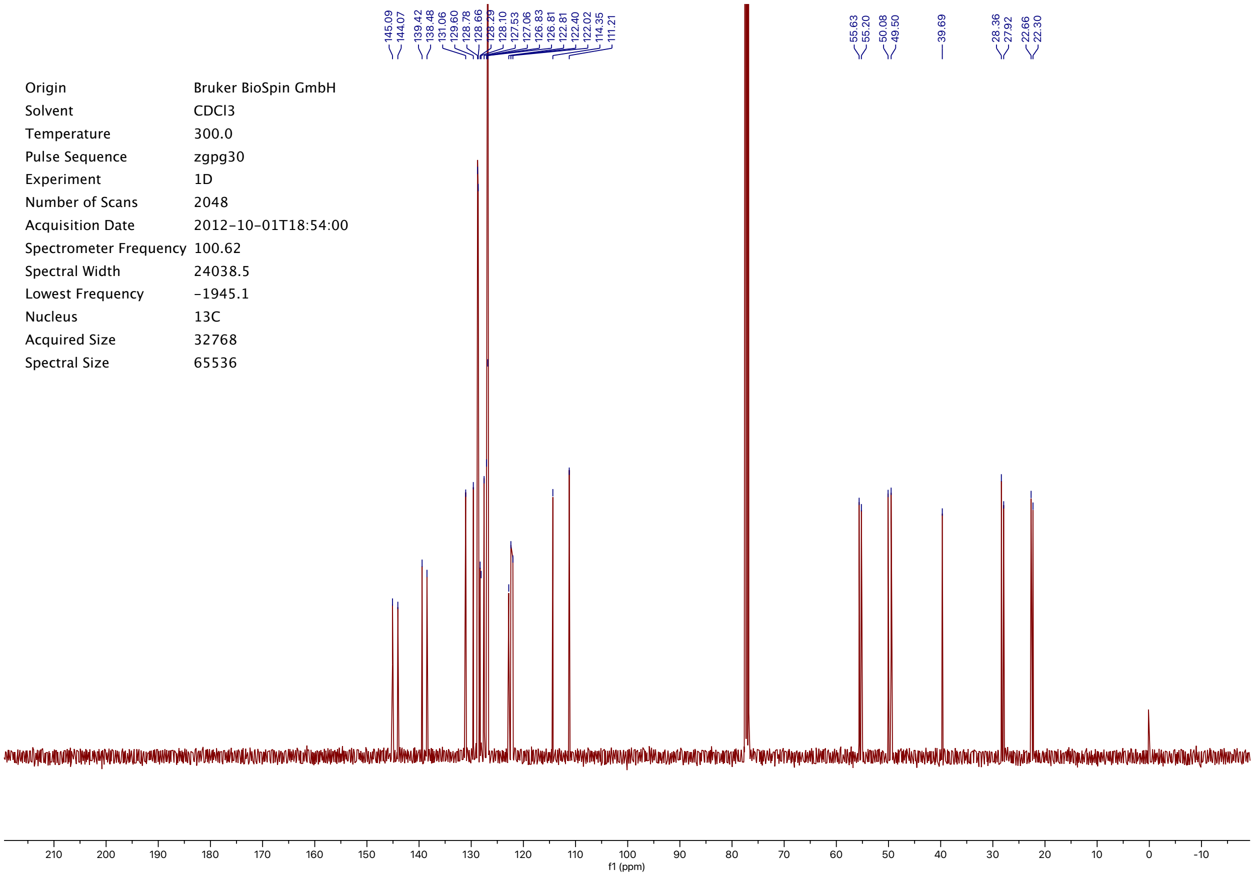

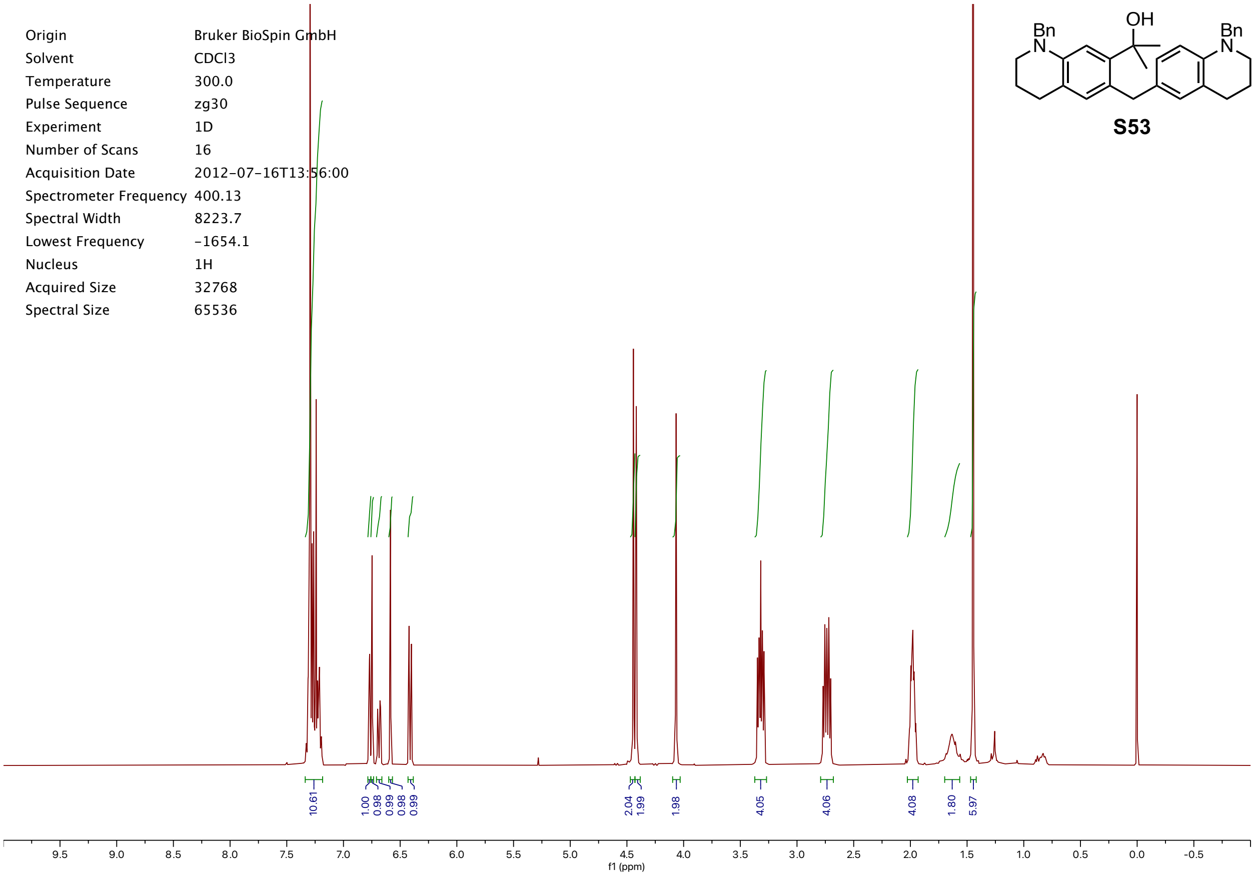
Bn
N
OH
Bn
N
S53
Origin Bruker BioSpin GmbH
Solvent CDCl3
Temperature 300.0
Pulse Sequence zg30
Experiment 1D
Number of Scans 16
Acquisition Date 2012-07-16T13:56:00
Spectrometer Frequency 400.13
Spectral Width 8223.7
Lowest Frequency -1654.1
Nucleus 1H
Acquired Size 32768
Spectral Size 65536
10.61
1.00
0.98
0.99
0.98
0.99
2.04
1.99
1.98
4.05
4.06
4.08
1.80
5.97
9.5
9.0
8.5
8.0
7.5
7.0
6.5
6.0
5.5
5.0
4.5
4.0
3.5
3.0
2.5
2.0
1.5
1.0
0.5
0.0
-0.5
f1 (ppm)

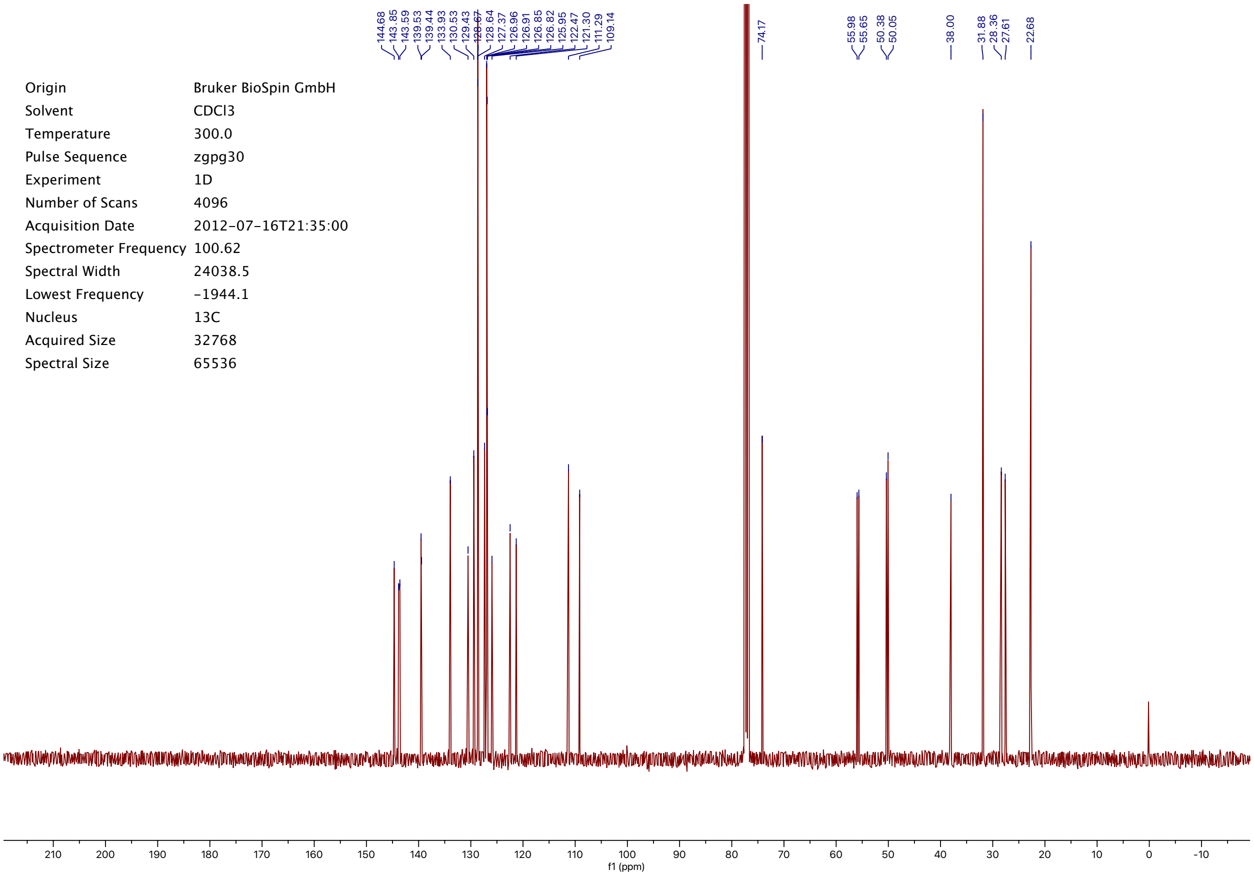
144.68
143.85
143.59
139.53
139.44
133.93
130.53
129.43
128.67
128.64
127.37
126.96
126.91
126.85
126.82
125.95
122.47
121.30
111.29
109.14
74.17
55.98
55.65
50.38
50.05
38.00
31.88
28.36
27.61
22.68
Origin Bruker BioSpin GmbH
Solvent CDCl3
Temperature 300.0
Pulse Sequence zgpg30
Experiment 1D
Number of Scans 4096
Acquisition Date 2012-07-16T21:35:00
Spectrometer Frequency 100.62
Spectral Width 24038.5
Lowest Frequency -1944.1
Nucleus 13C
Acquired Size 32768
Spectral Size 65536
210
200
190
180
170
160
150
140
130
120
110
100
90
80
70
60
50
40
30
20
10
0
-10
f1 (ppm)

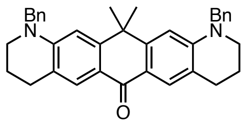

45

Origin	Bruker BioSpin GmbH
Solvent	CDCl3
Temperature	300.0
Pulse Sequence	zg30
Experiment	1D
Number of Scans	16
Acquisition Date	2012-10-02T13:38:00
Spectrometer Frequency	400.13
Spectral Width	8223.7
Lowest Frequency	-1647.6
Nucleus	1H
Acquired Size	32768
Spectral Size	65536

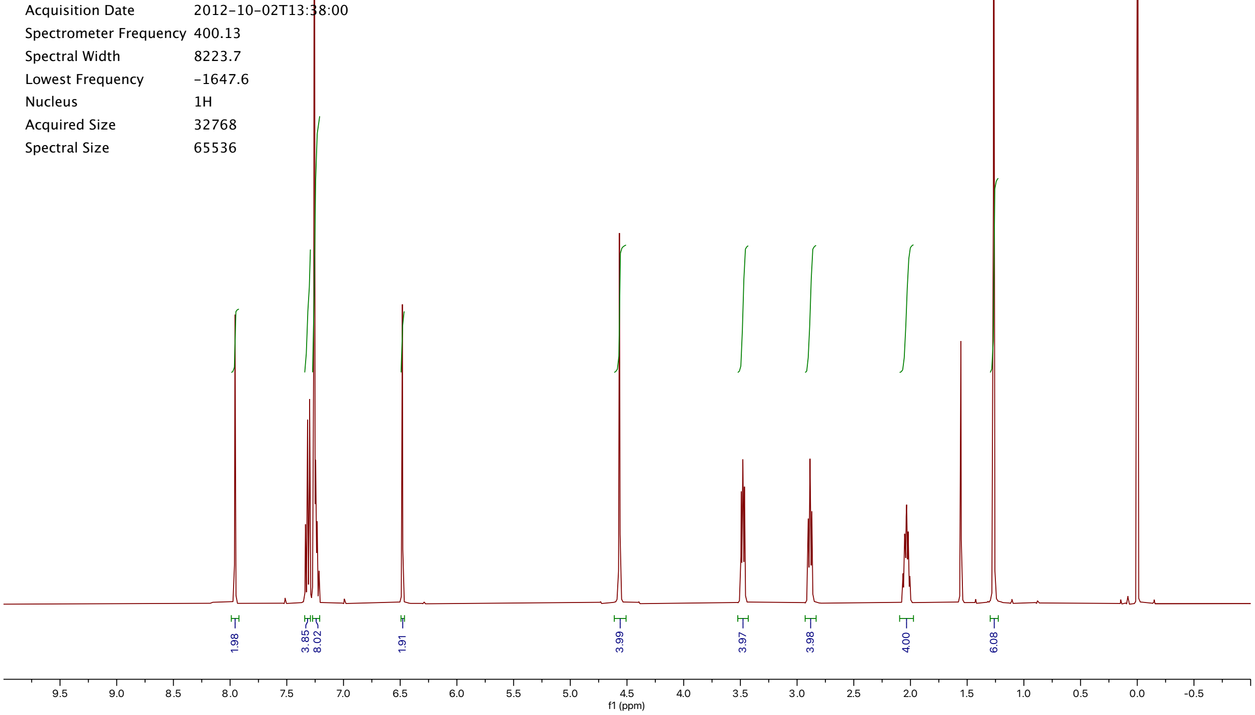

Origin	Bruker BioSpin GmbH
Solvent	CDCl3
Temperature	300.0
Pulse Sequence	zgpg30
Experiment	1D
Number of Scans	2048
Acquisition Date	2012-10-02T19:19:00
Spectrometer Frequency	100.62
Spectral Width	24038.5
Lowest Frequency	-1944.6
Nucleus	13C
Acquired Size	32768
Spectral Size	65536

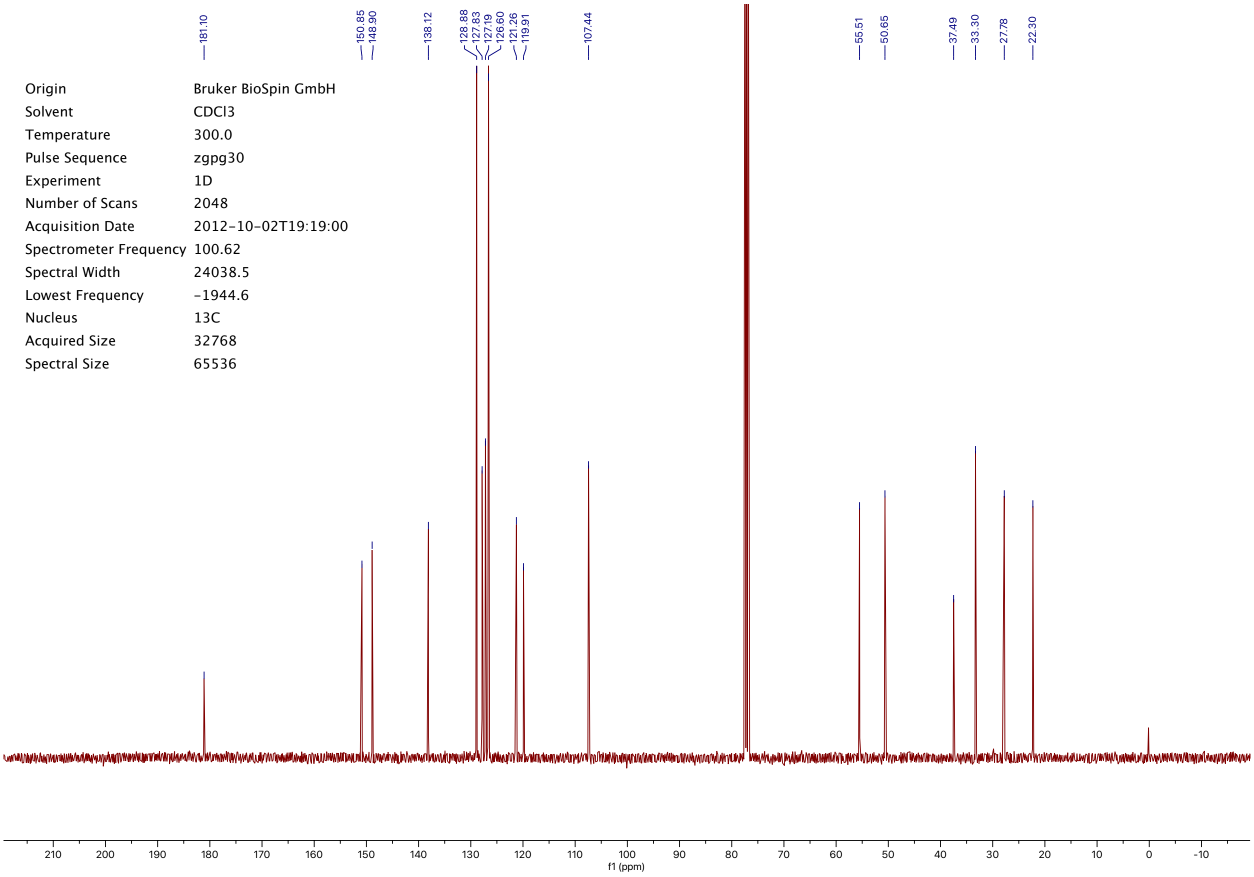

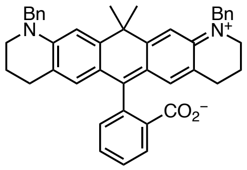
Bn
N
Bn
N+
CO2−

S55

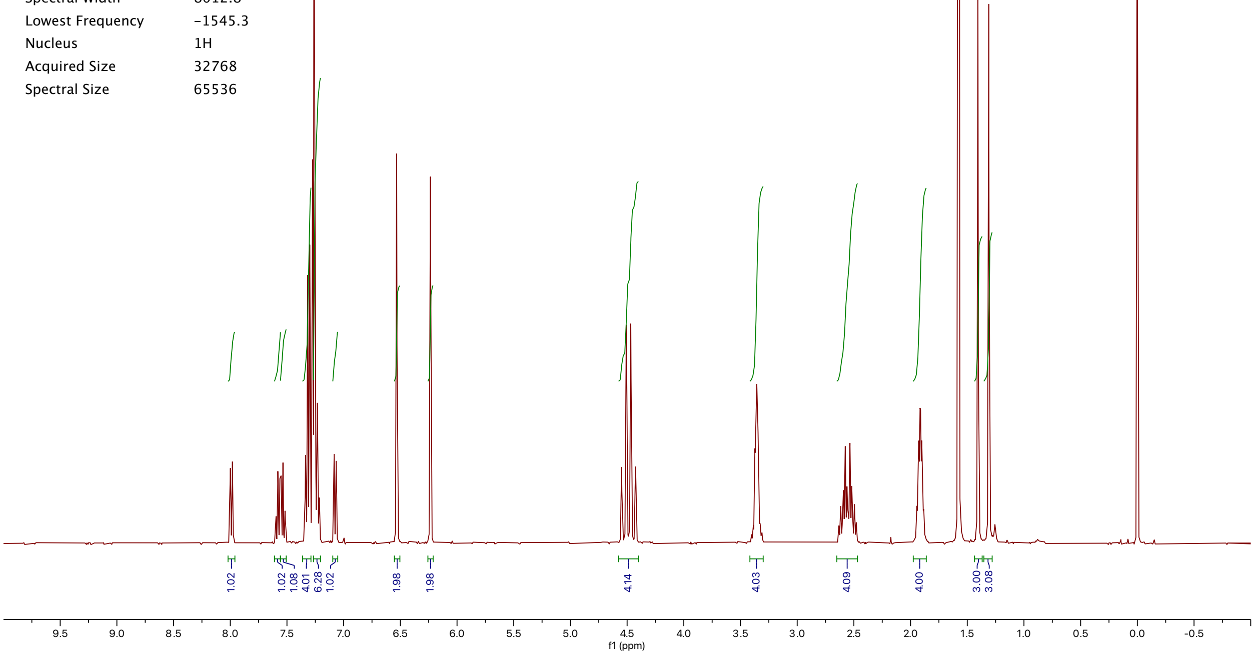
Origin Bruker BioSpin GmbH
Solvent CDCl3
Temperature 295.6
Pulse Sequence zg30
Experiment 1D
Number of Scans 16
Acquisition Date 2022-09-07T09:09:00
Spectrometer Frequency 400.13
Spectral Width 8012.8
Lowest Frequency -1545.3
Nucleus 1H
Acquired Size 32768
Spectral Size 65536
1.02
1.02
1.08
4.01
6.28
1.02
1.98
1.98
4.14
4.03
4.09
4.00
3.00
3.08
9.5 9.0 8.5 8.0 7.5 7.0 6.5 6.0 5.5 5.0 4.5 4.0 3.5 3.0 2.5 2.0 1.5 1.0 0.5 0.0 -0.5
f1 (ppm)

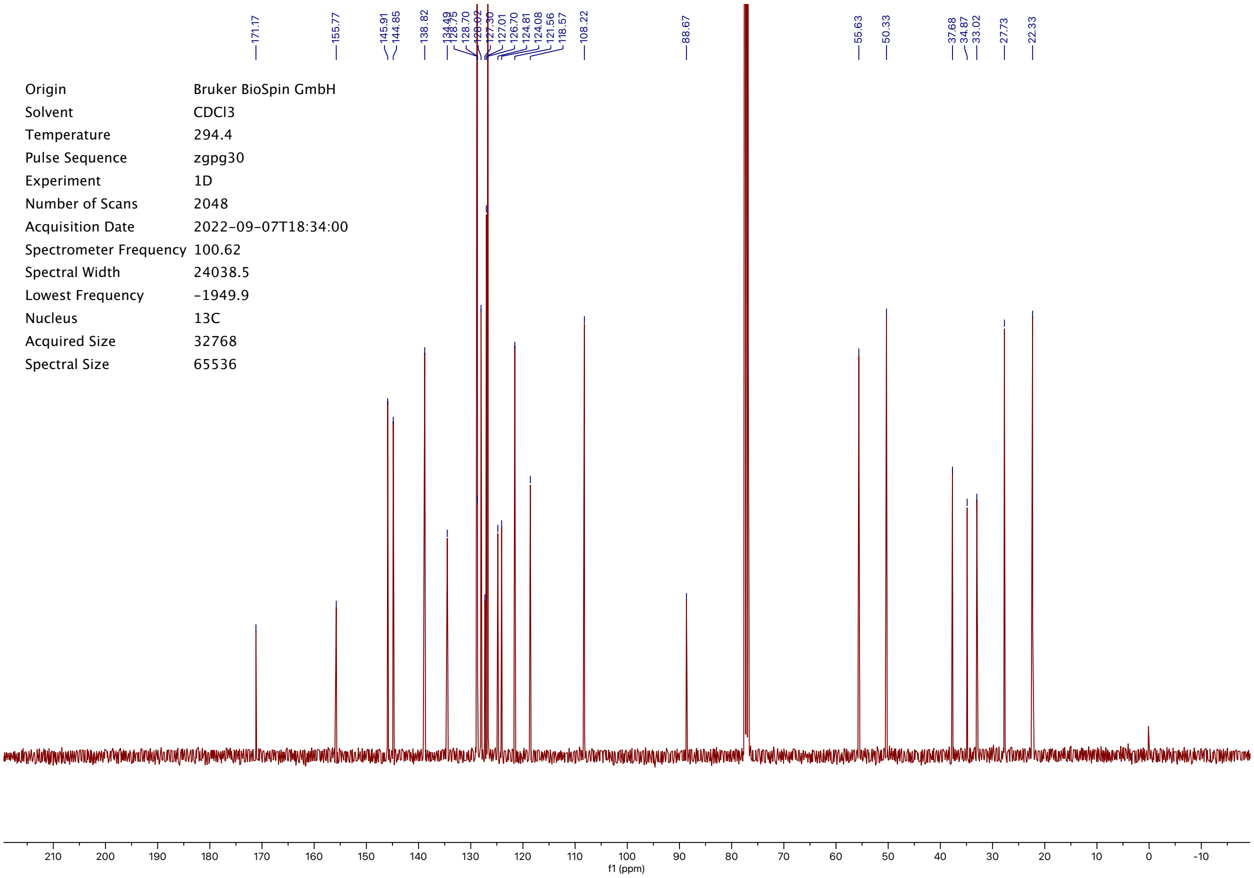
171.17
155.77
145.91
144.85
138.82
134.49
128.75
128.70
128.02
127.30
127.01
126.70
124.81
124.08
121.56
118.57
108.22
88.67
55.63
50.33
37.68
34.87
33.02
27.73
22.33
Origin Bruker BioSpin GmbH
Solvent CDCl3
Temperature 294.4
Pulse Sequence zgpg30
Experiment 1D
Number of Scans 2048
Acquisition Date 2022-09-07T18:34:00
Spectrometer Frequency 100.62
Spectral Width 24038.5
Lowest Frequency -1949.9
Nucleus 13C
Acquired Size 32768
Spectral Size 65536
210 200 190 180 170 160 150 140 130 120 110 100 90 80 70 60 50 40 30 20 10 0 -10
f1 (ppm)

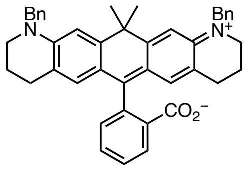

Bn
N
Bn
N+
CO2−

S55

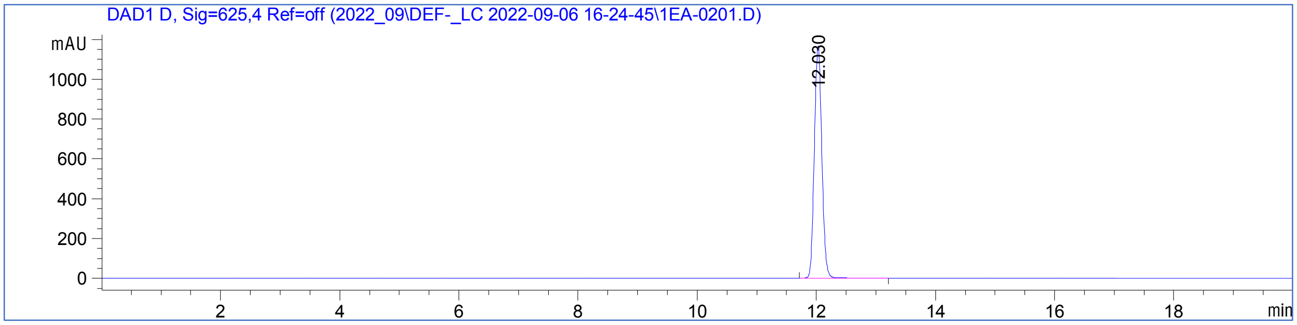

DAD1 D, Sig=625,4 Ref=off (2022_09\DEF-_LC 2022-09-06 16-24-45\1EA-0201.D)
mAU
12.030
min

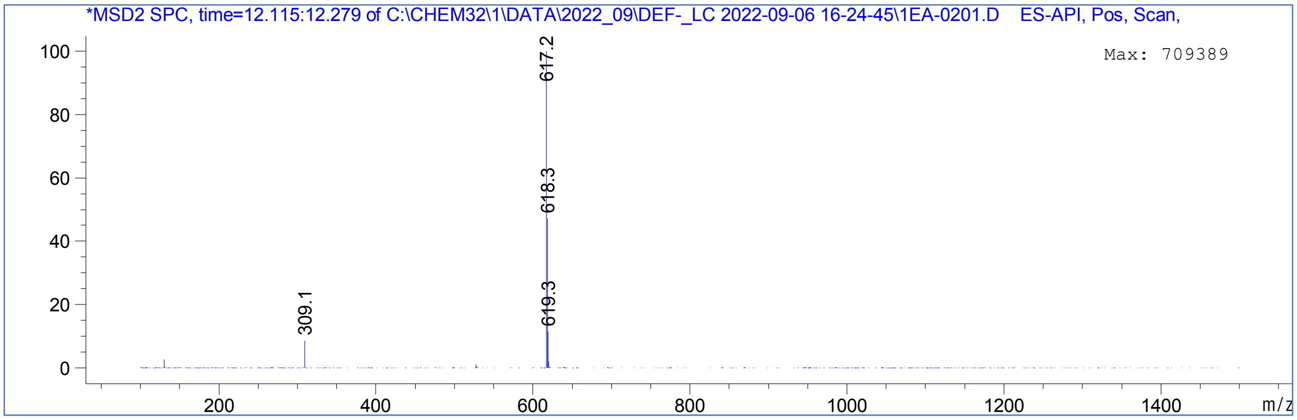

*MSD2 SPC, time=12.115:12.279 of C:\CHEM32\1\DATA\2022_09\DEF-_LC 2022-09-06 16-24-45\1EA-0201.D ES-API, Pos, Scan,
Max: 709389
617.2
618.3
619.3
309.1
m/z

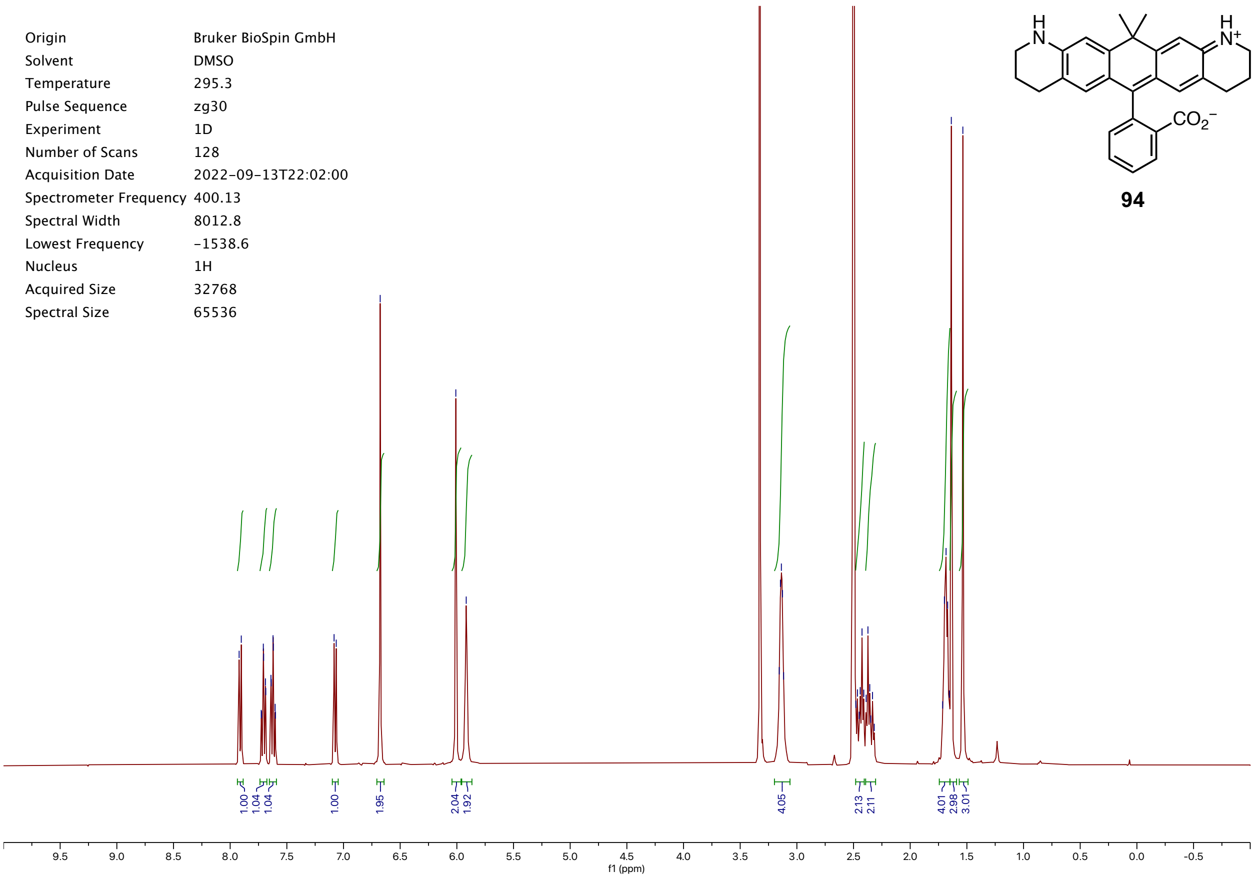
Origin Bruker BioSpin GmbH
Solvent DMSO
Temperature 295.3
Pulse Sequence zg30
Experiment 1D
Number of Scans 128
Acquisition Date 2022-09-13T22:02:00
Spectrometer Frequency 400.13
Spectral Width 8012.8
Lowest Frequency -1538.6
Nucleus 1H
Acquired Size 32768
Spectral Size 65536
1.00
1.04
1.04
1.00
1.95
2.04
1.92
4.05
2.13
2.11
4.01
2.98
3.01
f1 (ppm)

94

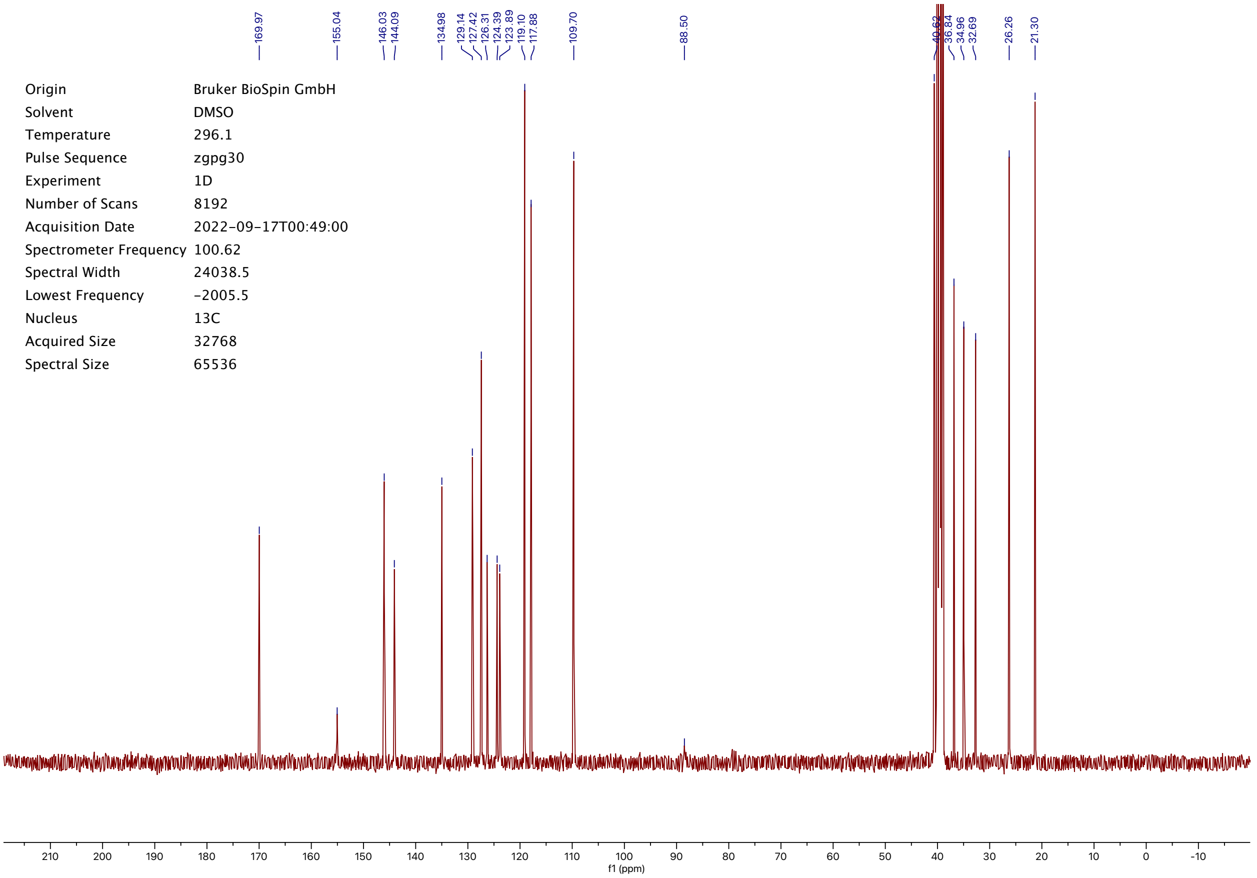
169.97
155.04
146.03
144.09
134.98
129.14
127.42
126.31
124.39
123.89
119.10
117.88
109.70
88.50
40.62
36.84
34.96
32.69
26.26
21.30
Origin Bruker BioSpin GmbH
Solvent DMSO
Temperature 296.1
Pulse Sequence zgpg30
Experiment 1D
Number of Scans 8192
Acquisition Date 2022-09-17T00:49:00
Spectrometer Frequency 100.62
Spectral Width 24038.5
Lowest Frequency -2005.5
Nucleus 13C
Acquired Size 32768
Spectral Size 65536
f1 (ppm)

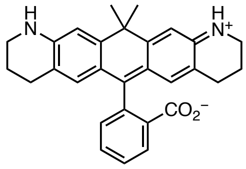

H
N
H
N+
CO2−

94

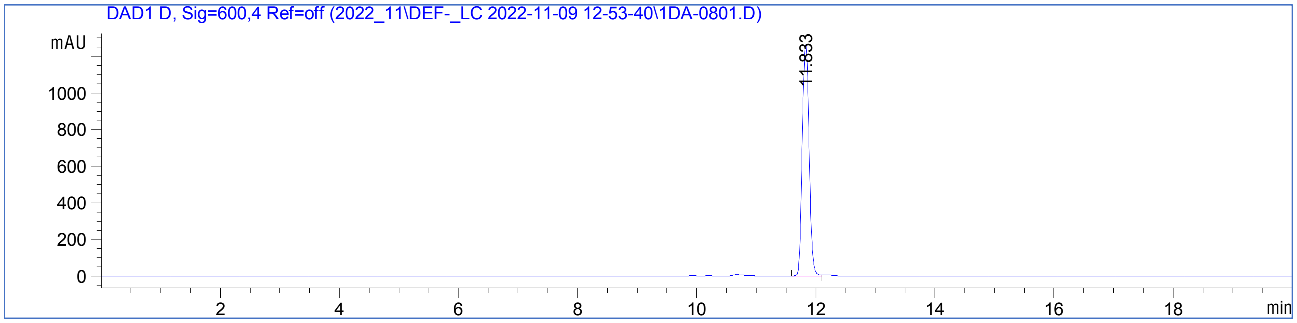

DAD1 D, Sig=600,4 Ref=off (2022_11\DEF-_LC 2022-11-09 12-53-40\1DA-0801.D)
mAU
1000
800
600
400
200
0
11.833
2
4
6
8
10
12
14
16
18
min

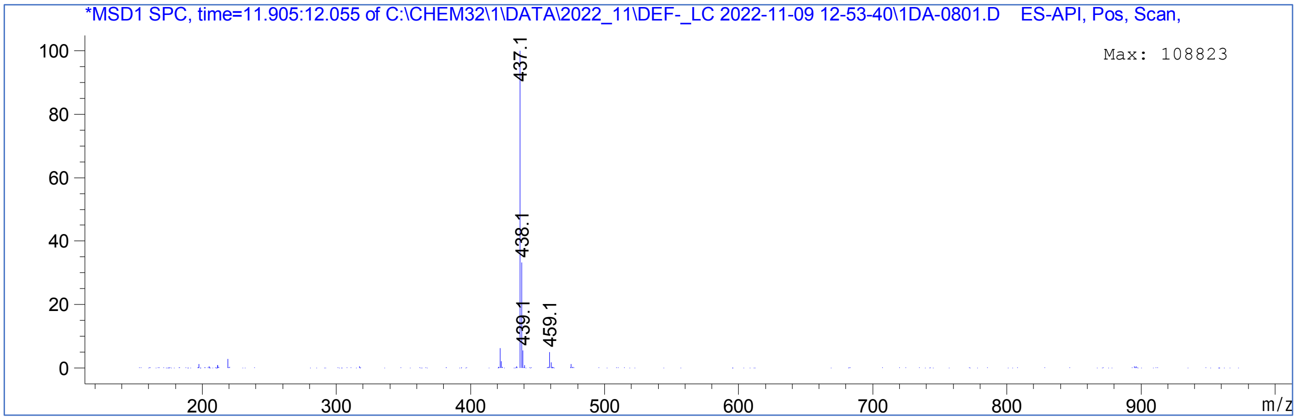

*MSD1 SPC, time=11.905:12.055 of C:\CHEM32\1\DATA\2022_11\DEF-_LC 2022-11-09 12-53-40\1DA-0801.D ES-API, Pos, Scan,
Max: 108823
100
80
60
40
20
0
437.1
438.1
439.1
459.1
200
300
400
500
600
700
800
900
m/z

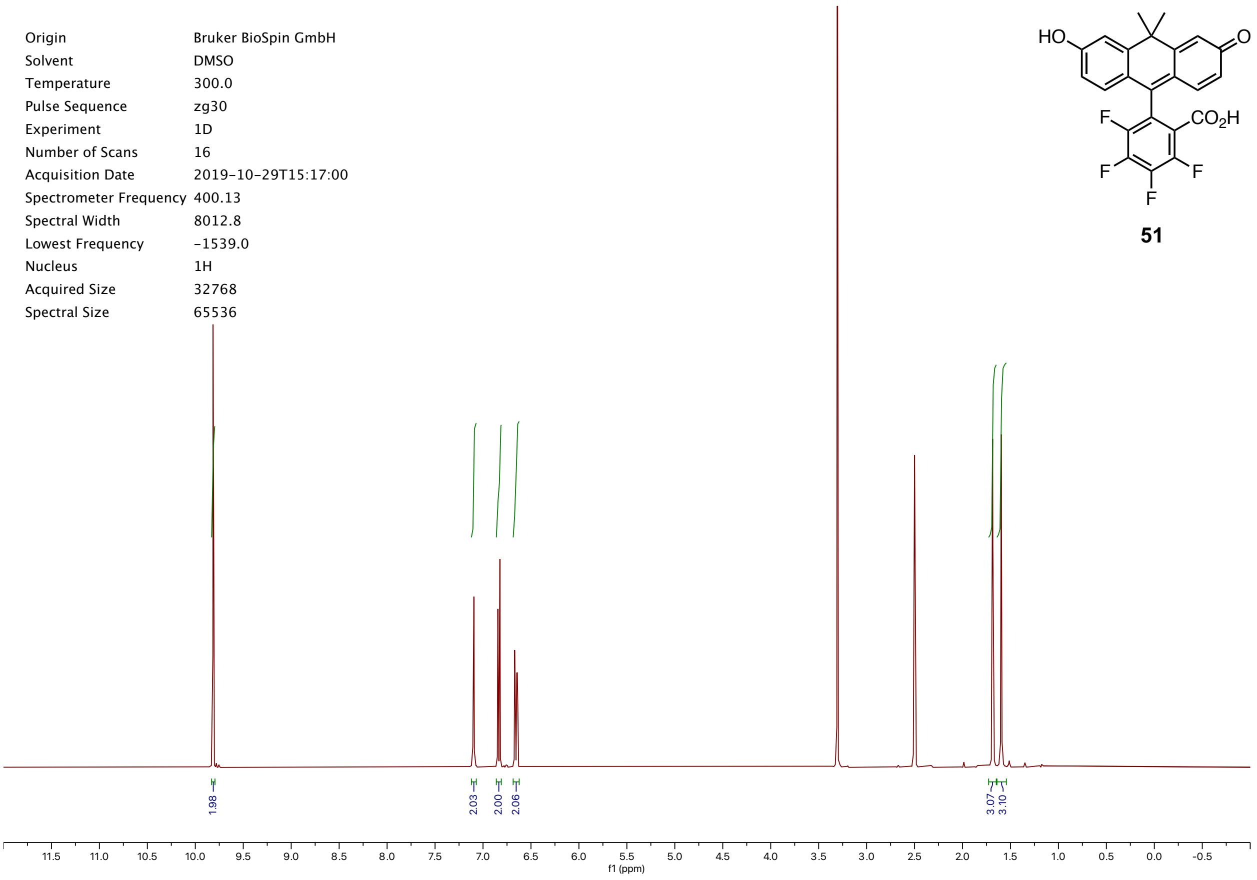
Origin Bruker BioSpin GmbH
Solvent DMSO
Temperature 300.0
Pulse Sequence zg30
Experiment 1D
Number of Scans 16
Acquisition Date 2019-10-29T15:17:00
Spectrometer Frequency 400.13
Spectral Width 8012.8
Lowest Frequency -1539.0
Nucleus 1H
Acquired Size 32768
Spectral Size 65536
HO
O
F
CO_2H
F
F
F
51
1.98
2.03
2.00
2.06
3.07
3.10
f1 (ppm)

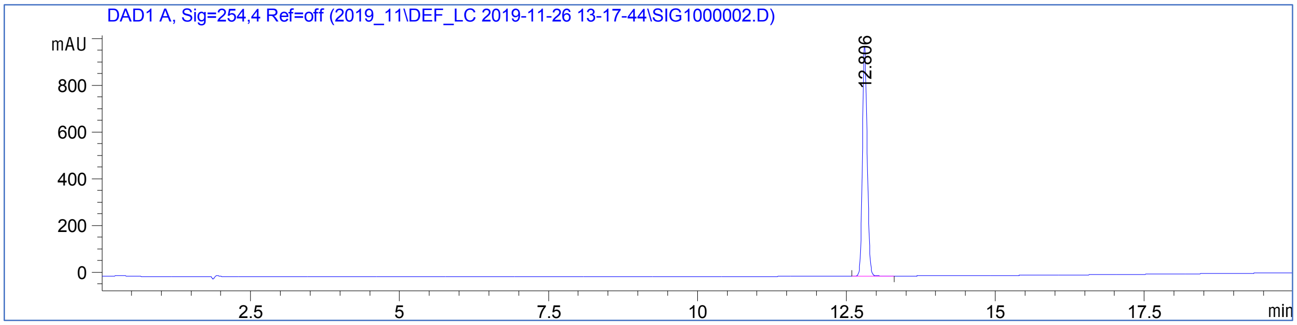
DAD1 A, Sig=254,4 Ref=off (2019_11\DEF_LC 2019-11-26 13-17-44\SIG1000002.D)
mAU
12.806
min

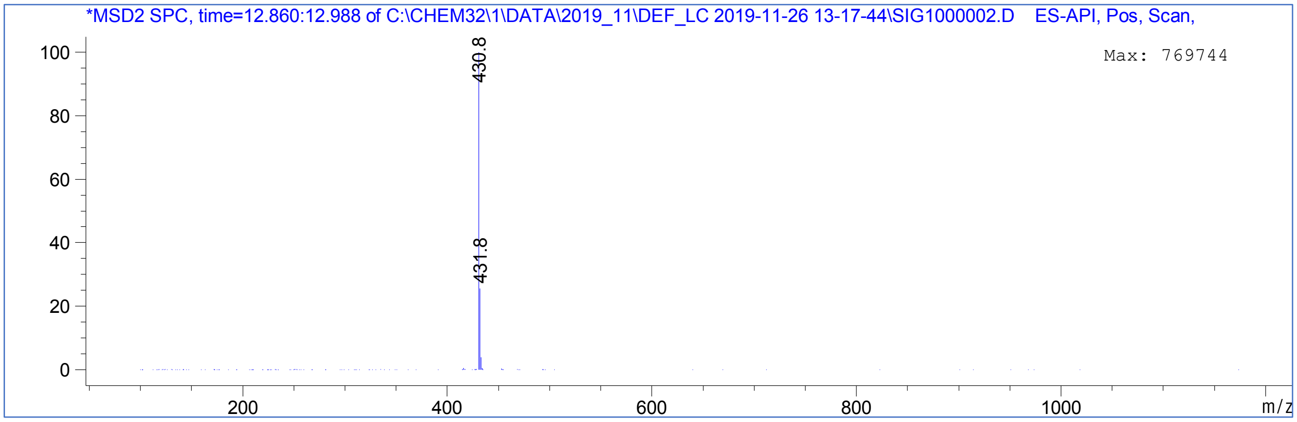
*MSD2 SPC, time=12.860:12.988 of C:\CHEM32\1\DATA\2019_11\DEF_LC 2019-11-26 13-17-44\SIG1000002.D ES-API, Pos, Scan,
Max: 769744
430.8
431.8
m/z

Origin	Bruker BioSpin GmbH
Solvent	DMSO
Temperature	300.0
Pulse Sequence	zg30
Experiment	1D
Number of Scans	16
Acquisition Date	2022-01-28T16:39:00
Spectrometer Frequency	400.13
Spectral Width	8012.8
Lowest Frequency	-1538.7
Nucleus	1H
Acquired Size	32768
Spectral Size	65536

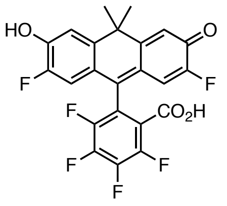

53

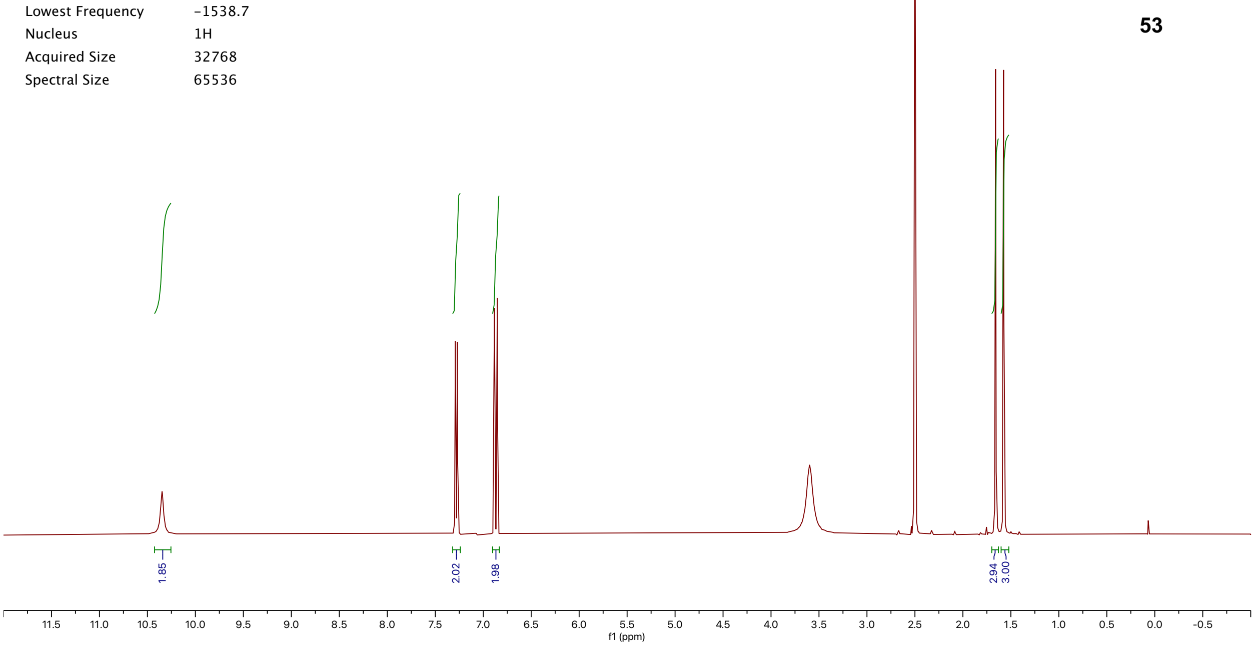

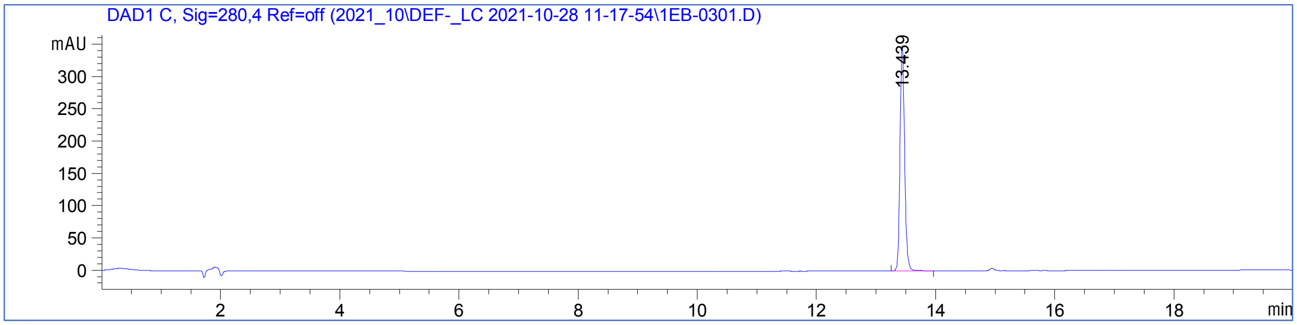

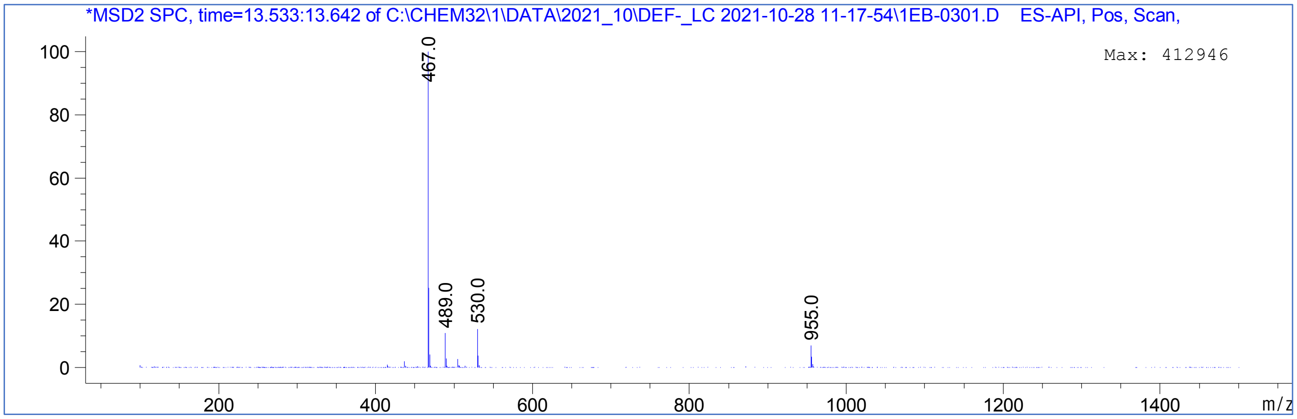

Origin	Bruker BioSpin GmbH
Solvent	CDCl3
Temperature	300.0
Pulse Sequence	zg30
Experiment	1D
Number of Scans	16
Acquisition Date	2019-07-24T10:38:00
Spectrometer Frequency	400.13
Spectral Width	8012.8
Lowest Frequency	-1546.6
Nucleus	1H
Acquired Size	32768
Spectral Size	65536

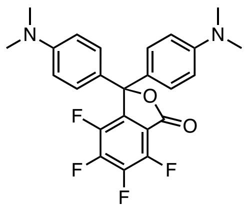

49

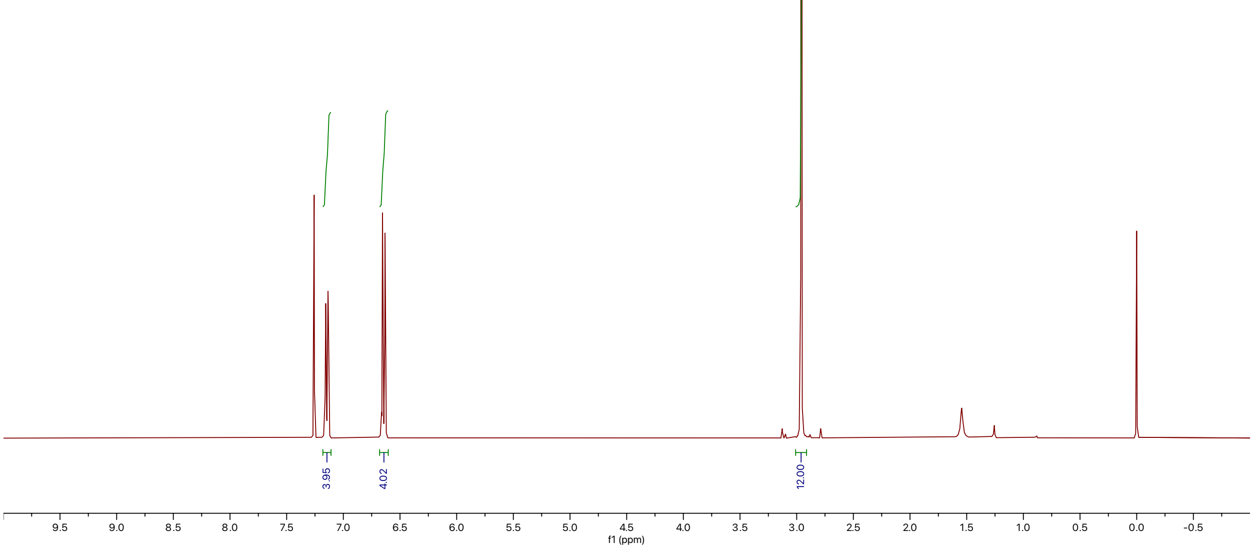

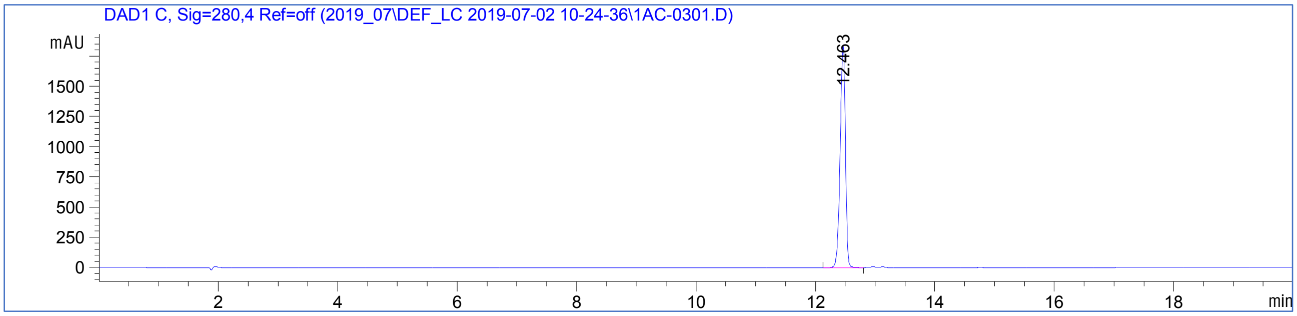

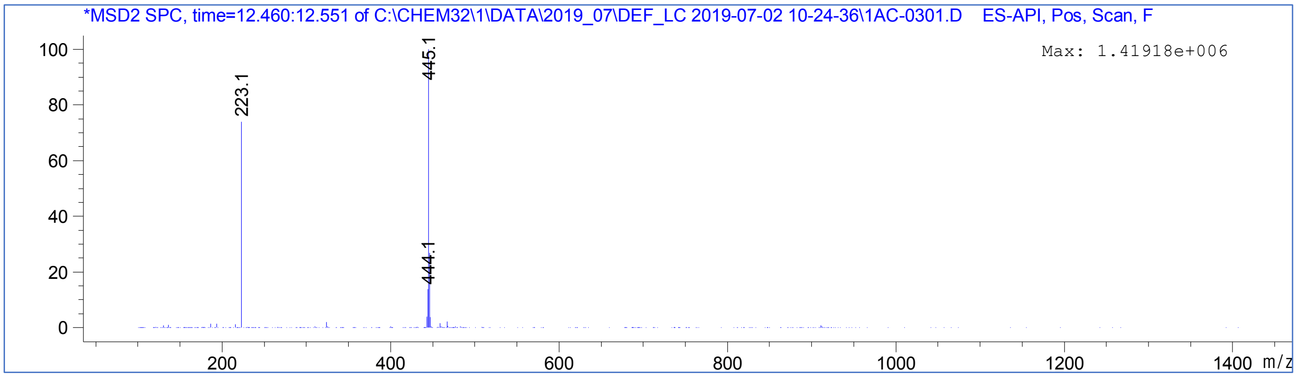

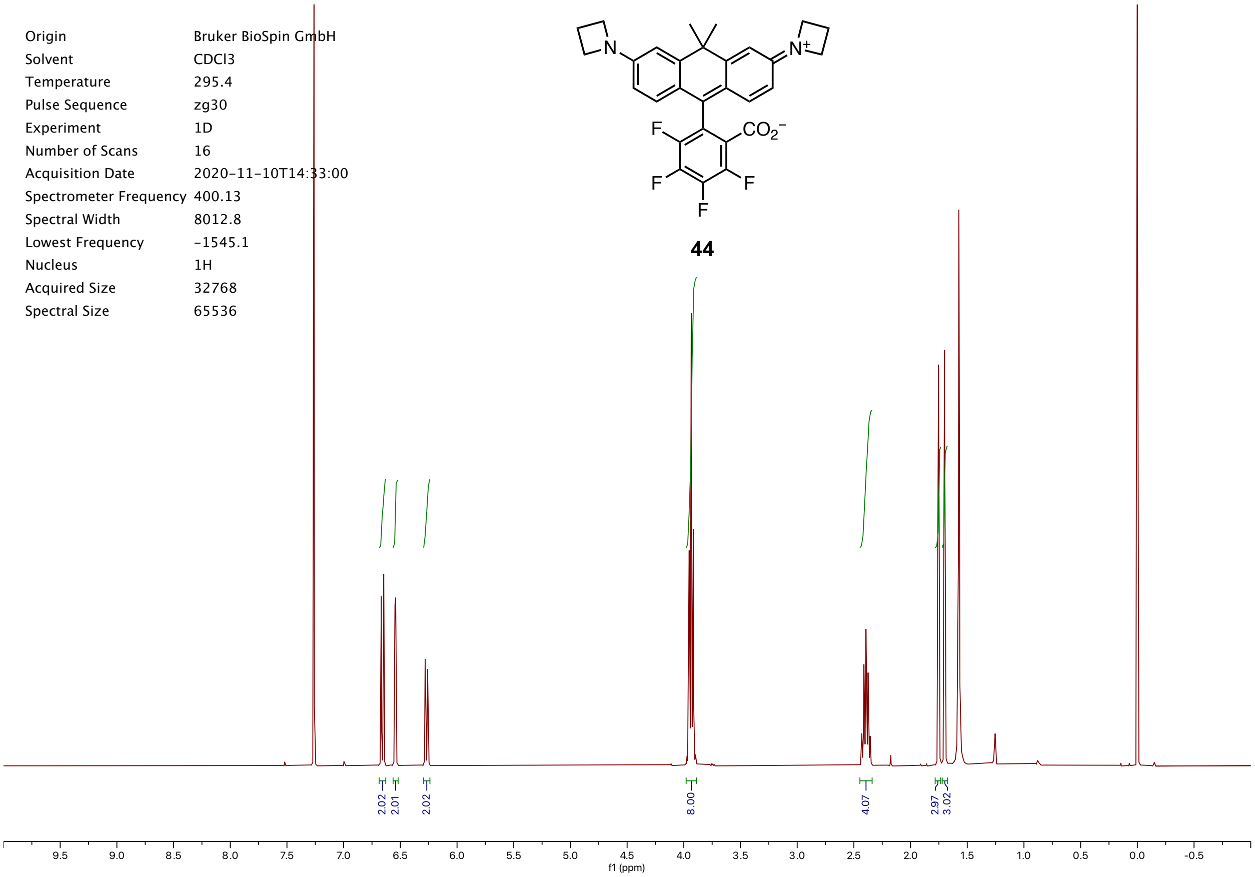
Origin Bruker BioSpin GmbH
Solvent CDCl3
Temperature 295.4
Pulse Sequence zg30
Experiment 1D
Number of Scans 16
Acquisition Date 2020-11-10T14:33:00
Spectrometer Frequency 400.13
Spectral Width 8012.8
Lowest Frequency -1545.1
Nucleus 1H
Acquired Size 32768
Spectral Size 65536
44
2.02
2.01
2.02
8.00
4.07
2.97
3.02
f1 (ppm)

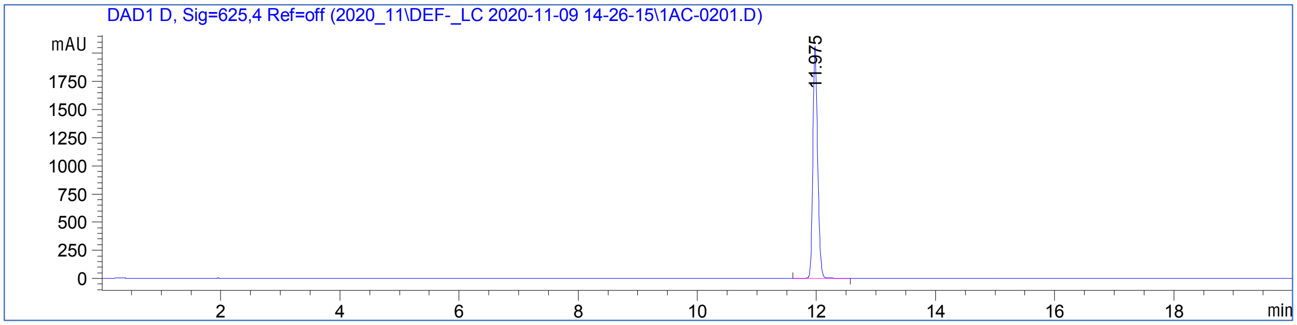
DAD1 D, Sig=625,4 Ref=off (2020_11\DEF-_LC 2020-11-09 14-26-15\1AC-0201.D)
mAU
11.975
min

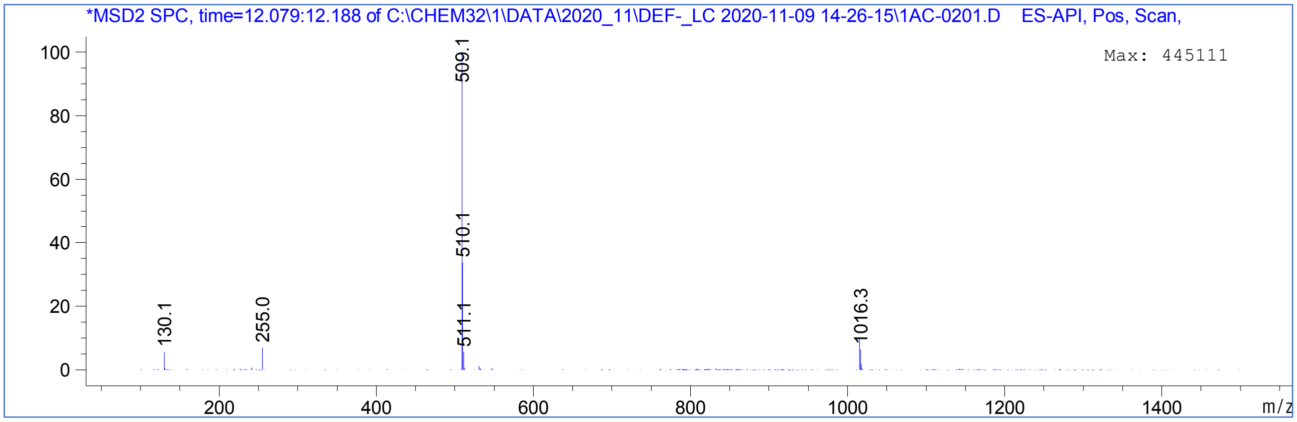
*MSD2 SPC, time=12.079:12.188 of C:\CHEM32\1\DATA\2020_11\DEF-_LC 2020-11-09 14-26-15\1AC-0201.D ES-API, Pos, Scan,
Max: 445111
509.1
510.1
511.1
130.1
255.0
1016.3
m/z

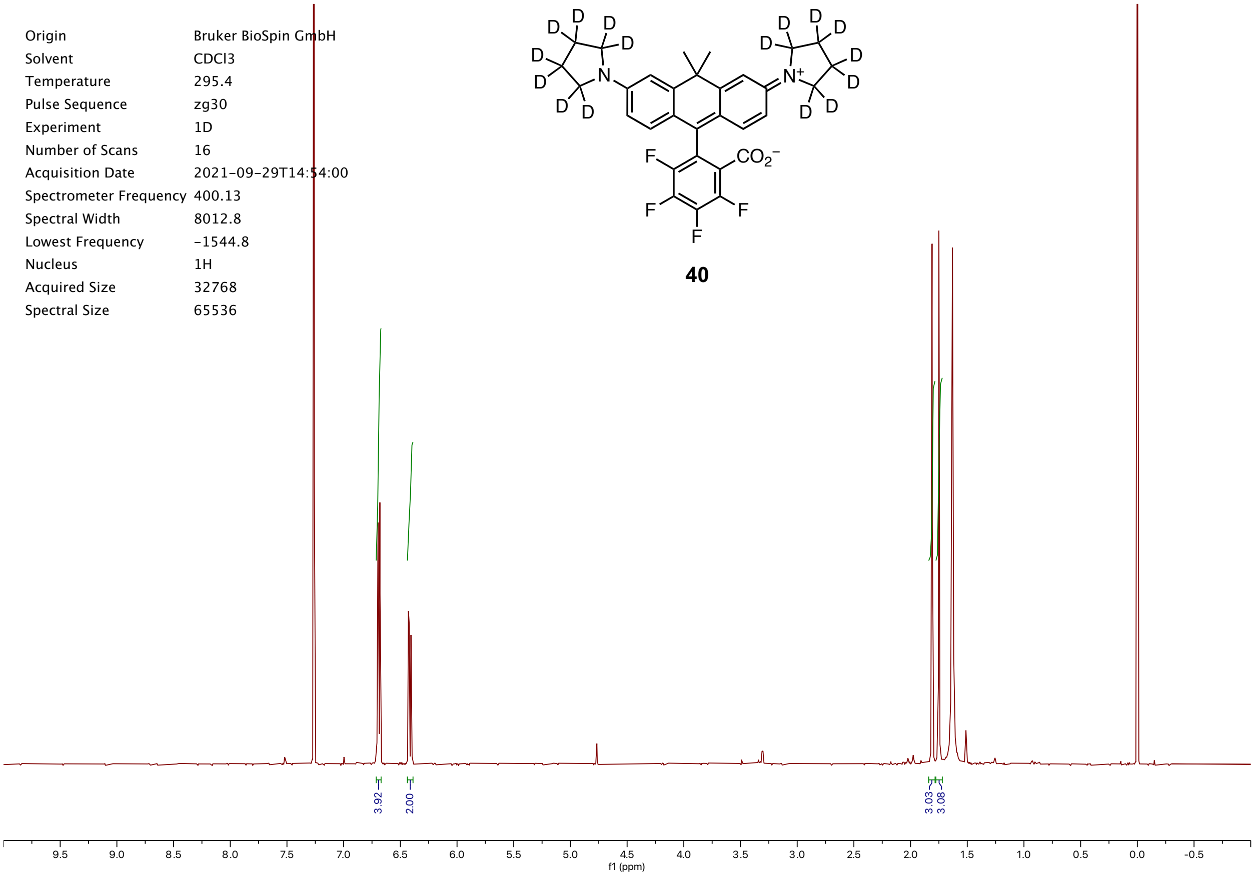
Origin Bruker BioSpin GmbH
Solvent CDCl3
Temperature 295.4
Pulse Sequence zg30
Experiment 1D
Number of Scans 16
Acquisition Date 2021-09-29T14:54:00
Spectrometer Frequency 400.13
Spectral Width 8012.8
Lowest Frequency -1544.8
Nucleus 1H
Acquired Size 32768
Spectral Size 65536
40
3.92
2.00
3.03
3.08
f1 (ppm)

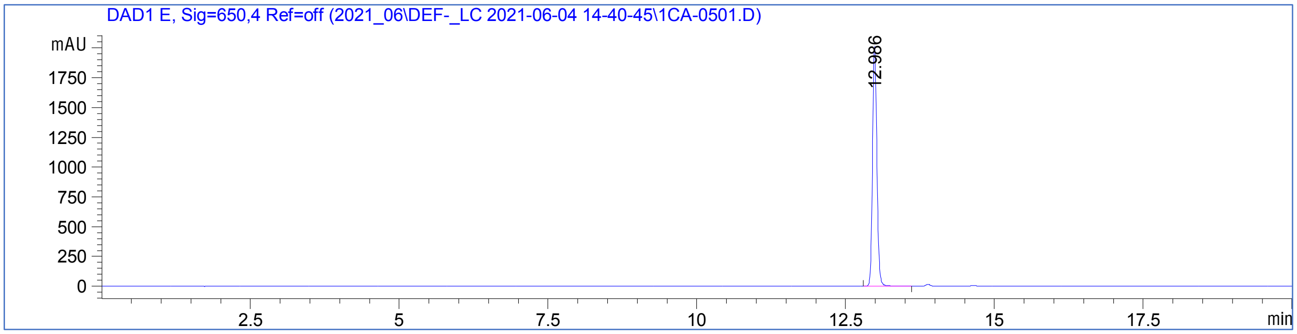
DAD1 E, Sig=650,4 Ref=off (2021_06\DEF-_LC 2021-06-04 14-40-45\1CA-0501.D)
mAU
12.986
min

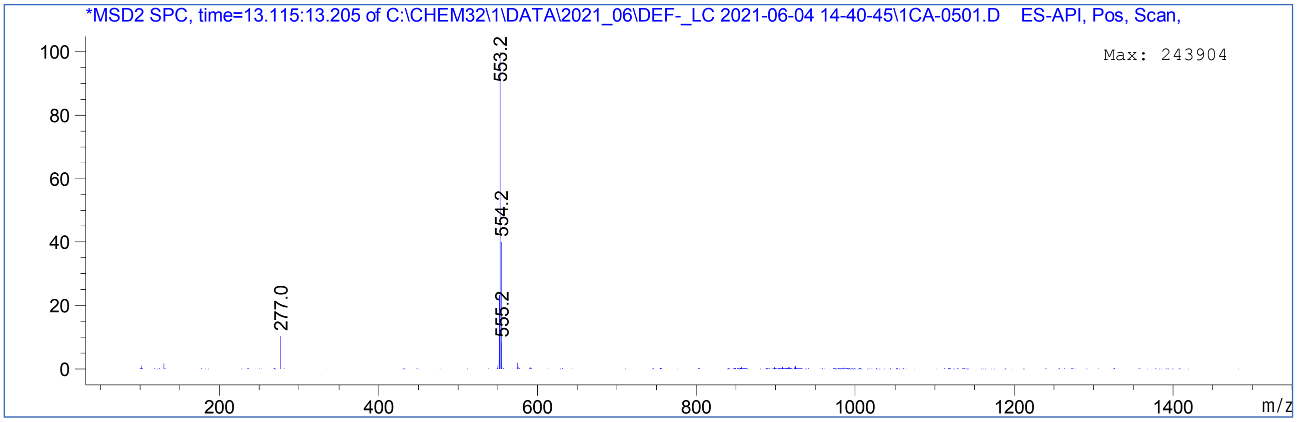
*MSD2 SPC, time=13.115:13.205 of C:\CHEM32\1\DATA\2021_06\DEF-_LC 2021-06-04 14-40-45\1CA-0501.D ES-API, Pos, Scan,
Max: 243904
553.2
554.2
555.2
277.0
m/z

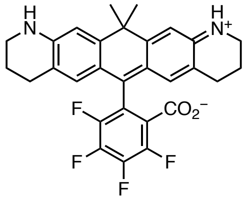
H
N
H
N+
F
CO2−
F
F
F

47

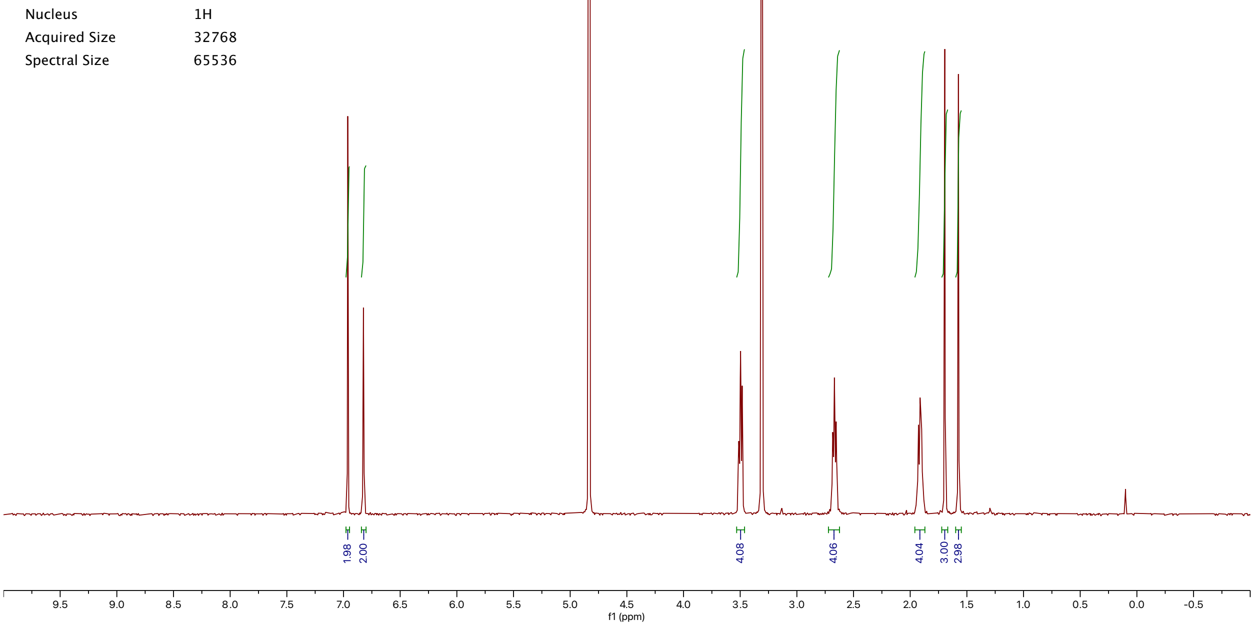
Origin Bruker BioSpin GmbH
Solvent MeOD
Temperature 300.0
Pulse Sequence zg30
Experiment 1D
Number of Scans 16
Acquisition Date 2022-05-09T12:02:00
Spectrometer Frequency 400.13
Spectral Width 8012.8
Lowest Frequency -1543.1
Nucleus 1H
Acquired Size 32768
Spectral Size 65536
1.98
2.00
4.08
4.06
4.04
3.00
2.98
9.5 9.0 8.5 8.0 7.5 7.0 6.5 6.0 5.5 5.0 4.5 4.0 3.5 3.0 2.5 2.0 1.5 1.0 0.5 0.0 -0.5
f1 (ppm)

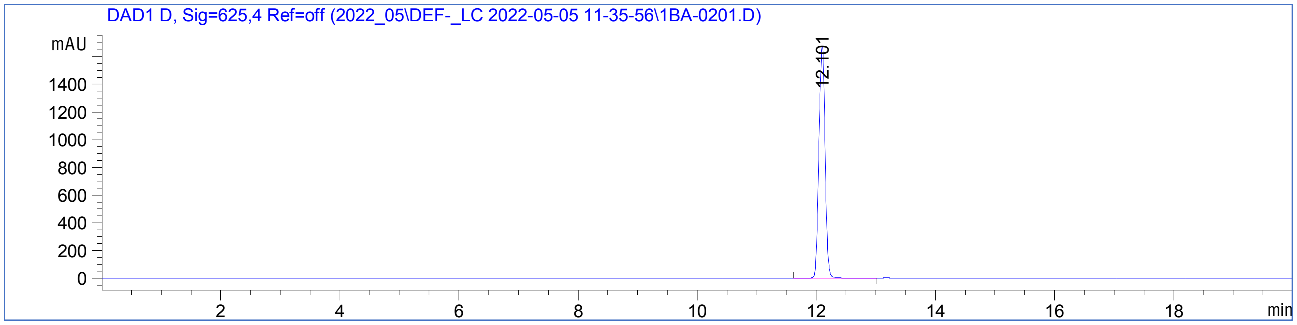
DAD1 D, Sig=625,4 Ref=off (2022_05\DEF-_LC 2022-05-05 11-35-56\1BA-0201.D)
mAU
1400
1200
1000
800
600
400
200
0
12.101
2 4 6 8 10 12 14 16 18 min

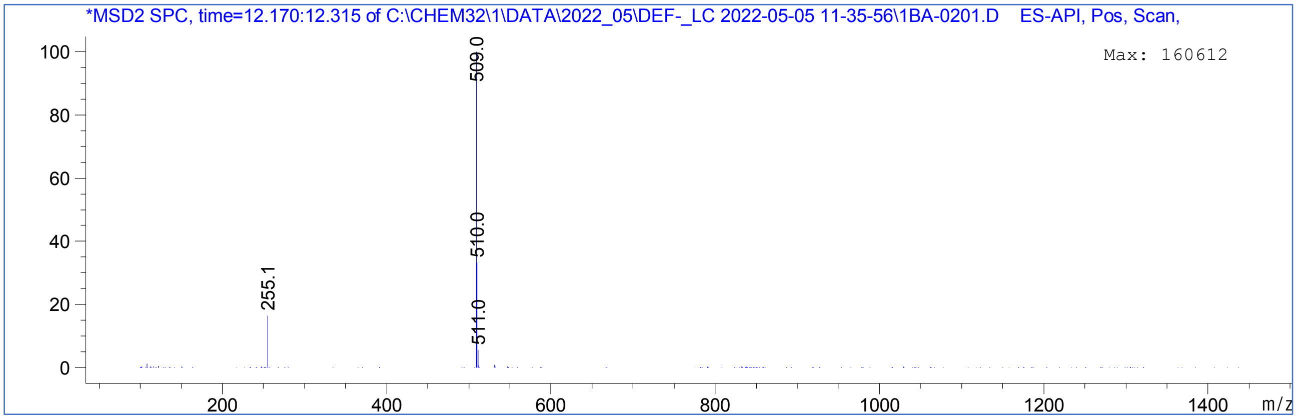
*MSD2 SPC, time=12.170:12.315 of C:\CHEM32\1\DATA\2022_05\DEF-_LC 2022-05-05 11-35-56\1BA-0201.D ES-API, Pos, Scan,
Max: 160612
100
80
60
40
20
0
509.0
510.0
511.0
255.1
200 400 600 800 1000 1200 1400 m/z

Origin	Bruker BioSpin GmbH
Solvent	CDCl3
Temperature	295.5
Pulse Sequence	zg30
Experiment	1D
Number of Scans	16
Acquisition Date	2021-09-29T17:23:00
Spectrometer Frequency	400.13
Spectral Width	8012.8
Lowest Frequency	-1545.0
Nucleus	1H
Acquired Size	32768
Spectral Size	65536

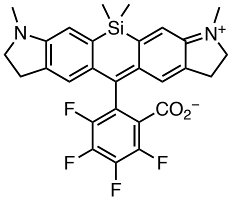

108

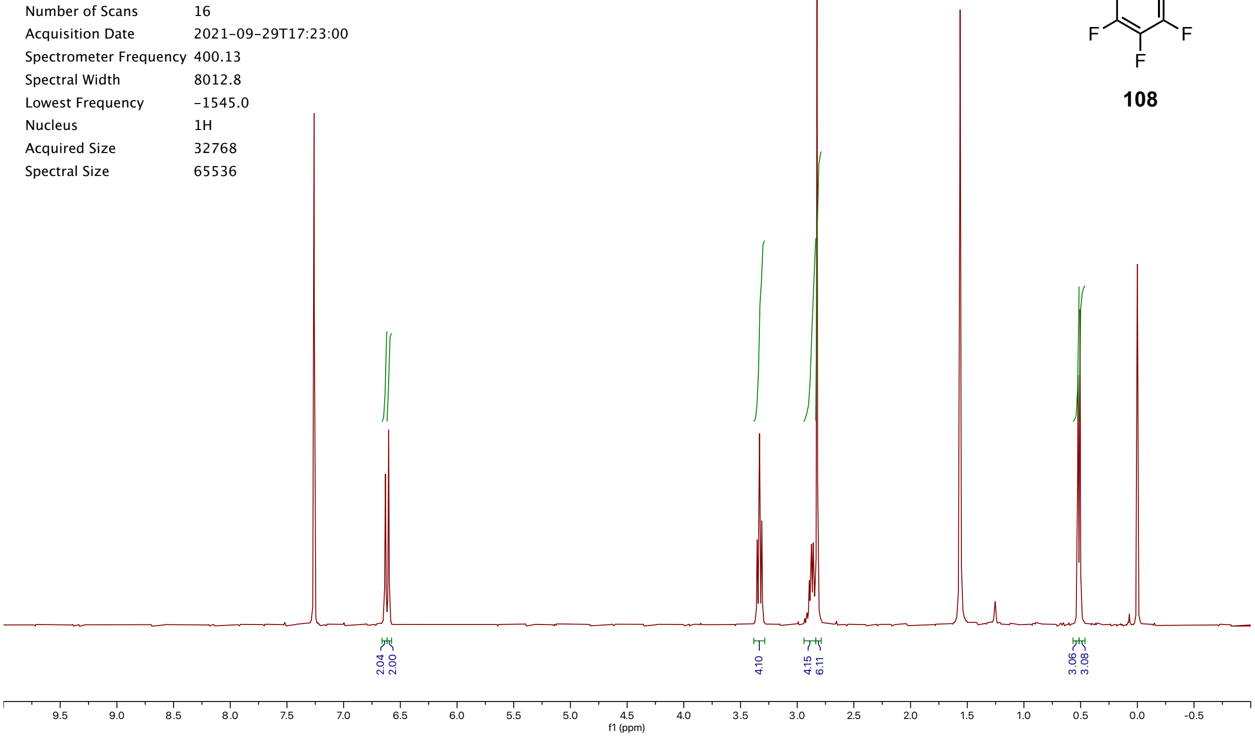

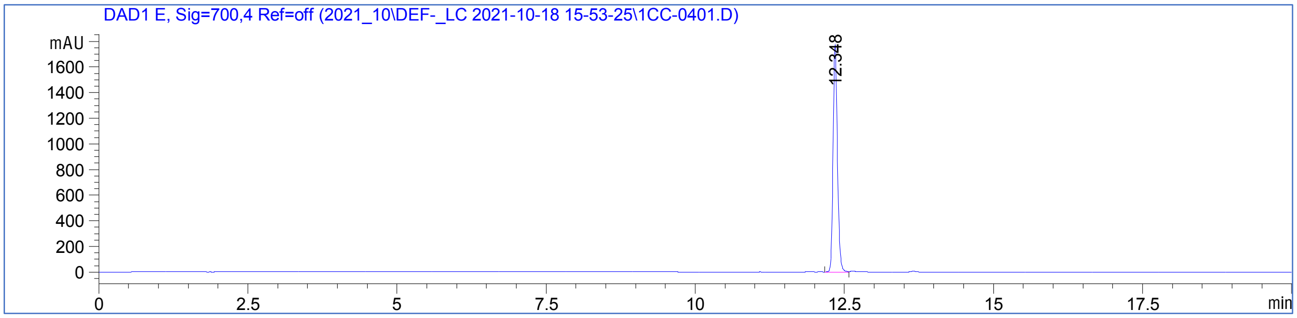

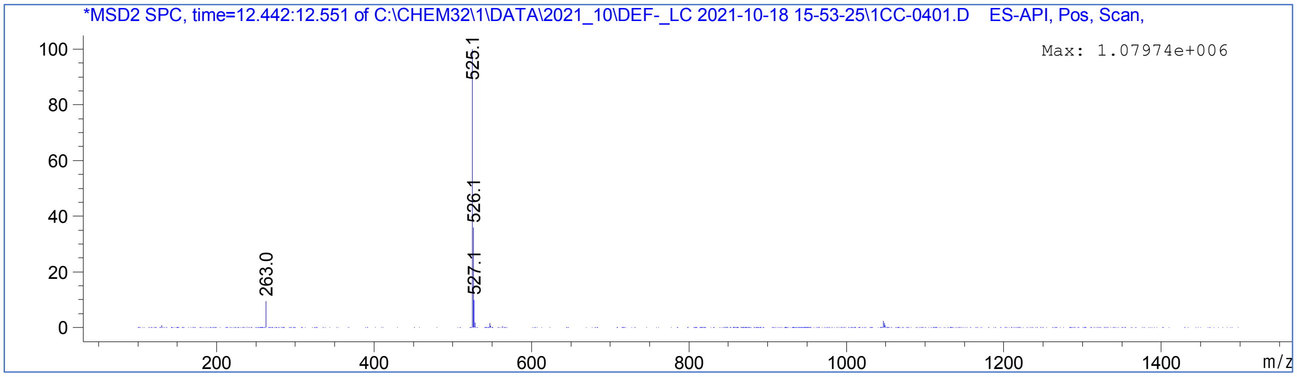

Origin	Bruker BioSpin GmbH
Solvent	MeOD
Temperature	300.0
Pulse Sequence	zg30
Experiment	1D
Number of Scans	16
Acquisition Date	2022-01-24T16:50:00
Spectrometer Frequency	400.13
Spectral Width	8012.8
Lowest Frequency	-1543.1
Nucleus	1H
Acquired Size	32768
Spectral Size	65536

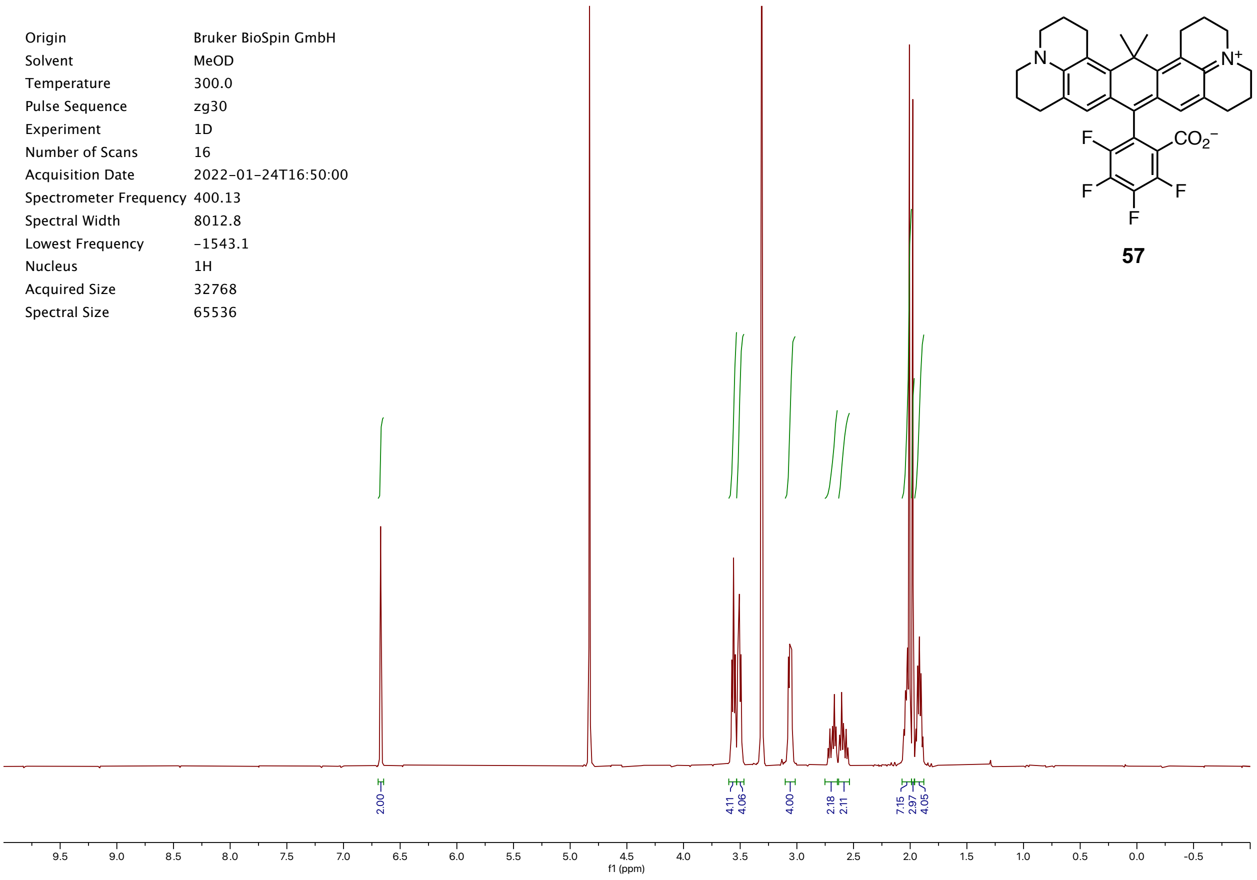

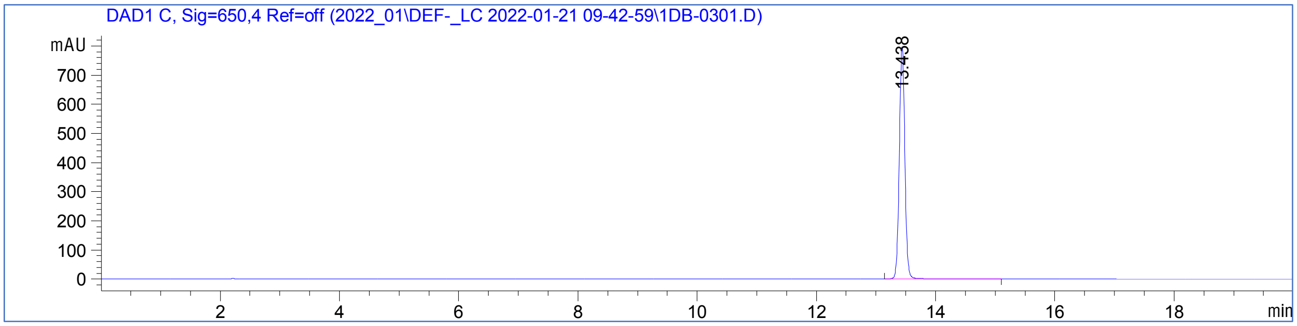

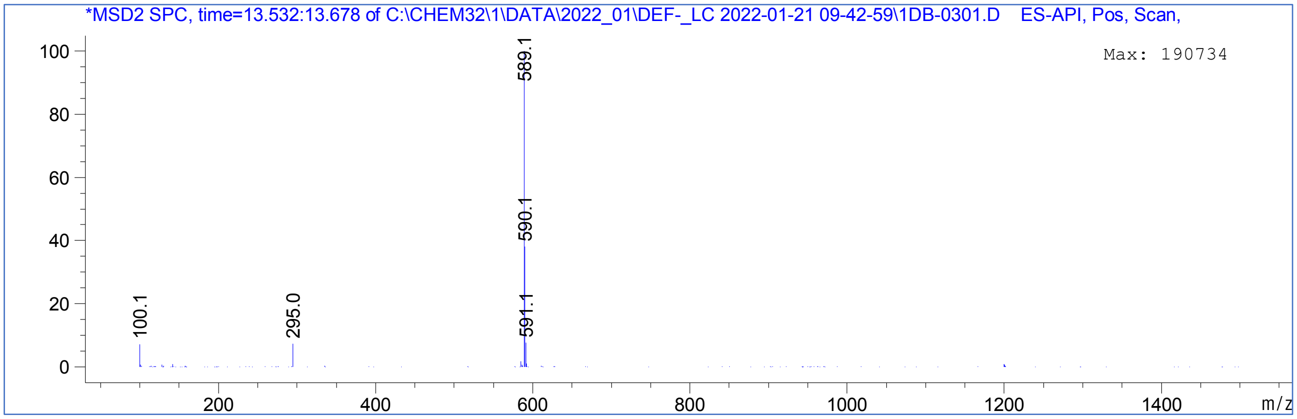

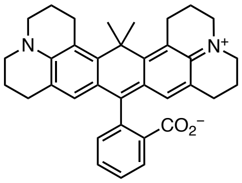
N
N+
CO2−

97

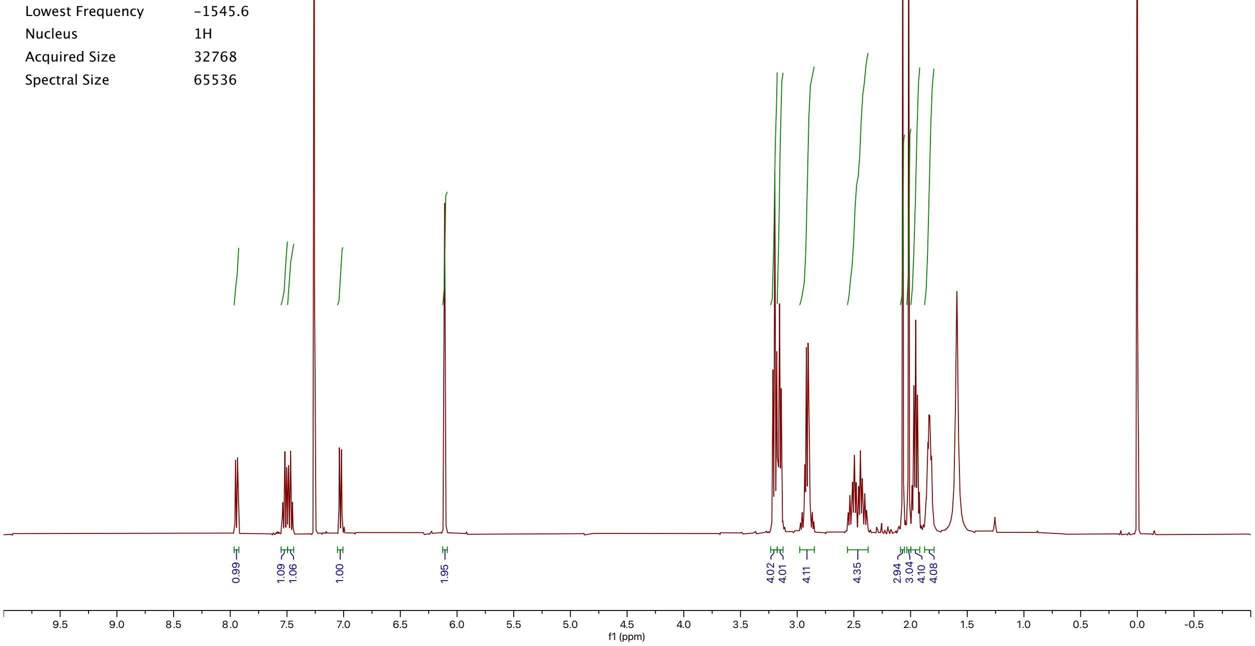
Origin Bruker BioSpin GmbH
Solvent CDCl3
Temperature 300.0
Pulse Sequence zg30
Experiment 1D
Number of Scans 16
Acquisition Date 2022-01-24T10:02:00
Spectrometer Frequency 400.13
Spectral Width 8012.8
Lowest Frequency -1545.6
Nucleus 1H
Acquired Size 32768
Spectral Size 65536
0.99
1.09
1.06
1.00
1.95
4.02
4.01
4.11
4.35
2.94
3.04
4.10
4.08
9.5 9.0 8.5 8.0 7.5 7.0 6.5 6.0 5.5 5.0 4.5 4.0 3.5 3.0 2.5 2.0 1.5 1.0 0.5 0.0 -0.5
f1 (ppm)

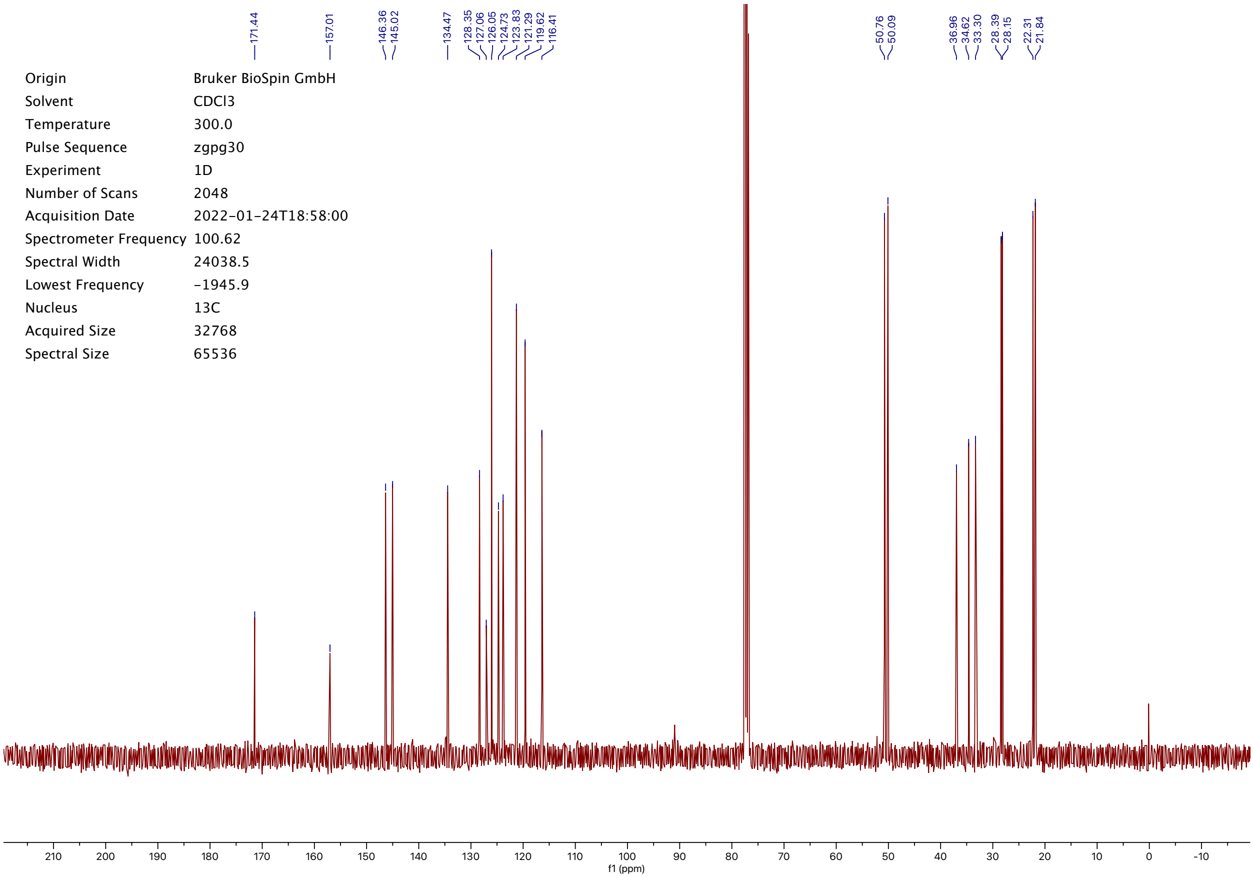
171.44
157.01
146.36
145.02
134.47
128.35
127.06
126.05
124.73
123.83
121.29
119.62
116.41
50.76
50.09
36.96
34.62
33.30
28.39
28.15
22.31
21.84
Origin Bruker BioSpin GmbH
Solvent CDCl3
Temperature 300.0
Pulse Sequence zgpg30
Experiment 1D
Number of Scans 2048
Acquisition Date 2022-01-24T18:58:00
Spectrometer Frequency 100.62
Spectral Width 24038.5
Lowest Frequency -1945.9
Nucleus 13C
Acquired Size 32768
Spectral Size 65536
210 200 190 180 170 160 150 140 130 120 110 100 90 80 70 60 50 40 30 20 10 0 -10
f1 (ppm)

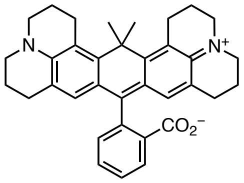

N
N+
CO2−

97

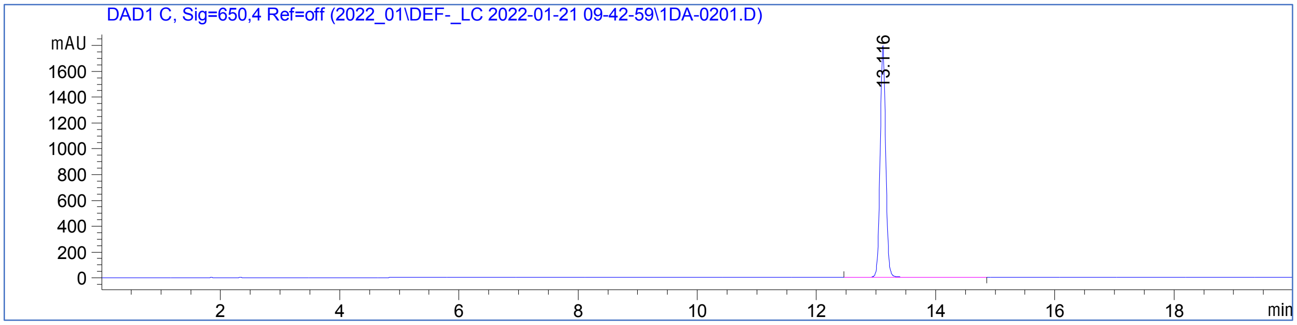

DAD1 C, Sig=650,4 Ref=off (2022_01\DEF-_LC 2022-01-21 09-42-59\1DA-0201.D)
mAU
1600
1400
1200
1000
800
600
400
200
0
13.116
2
4
6
8
10
12
14
16
18
min

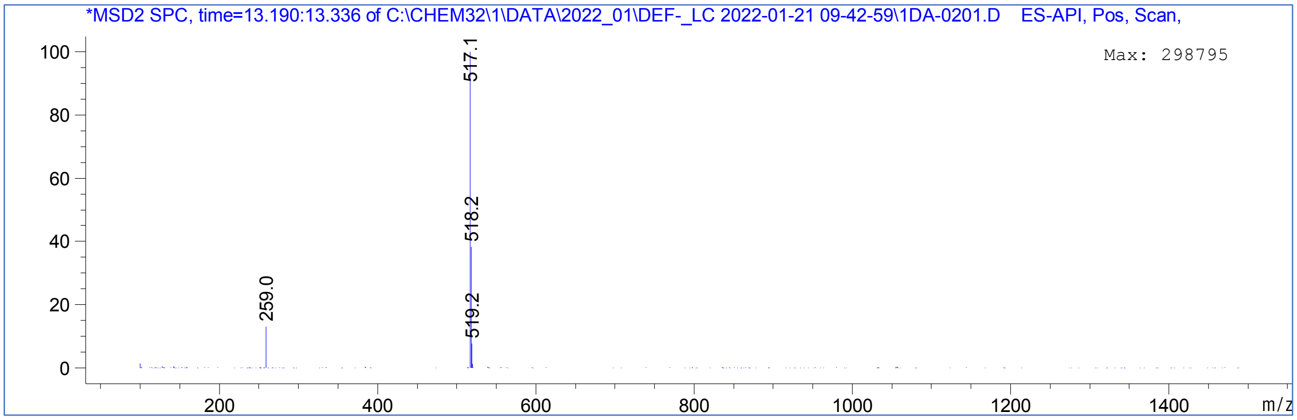

*MSD2 SPC, time=13.190:13.336 of C:\CHEM32\1\DATA\2022_01\DEF-_LC 2022-01-21 09-42-59\1DA-0201.D ES-API, Pos, Scan,
Max: 298795
100
80
60
40
20
0
517.1
518.2
519.2
259.0
200
400
600
800
1000
1200
1400
m/z

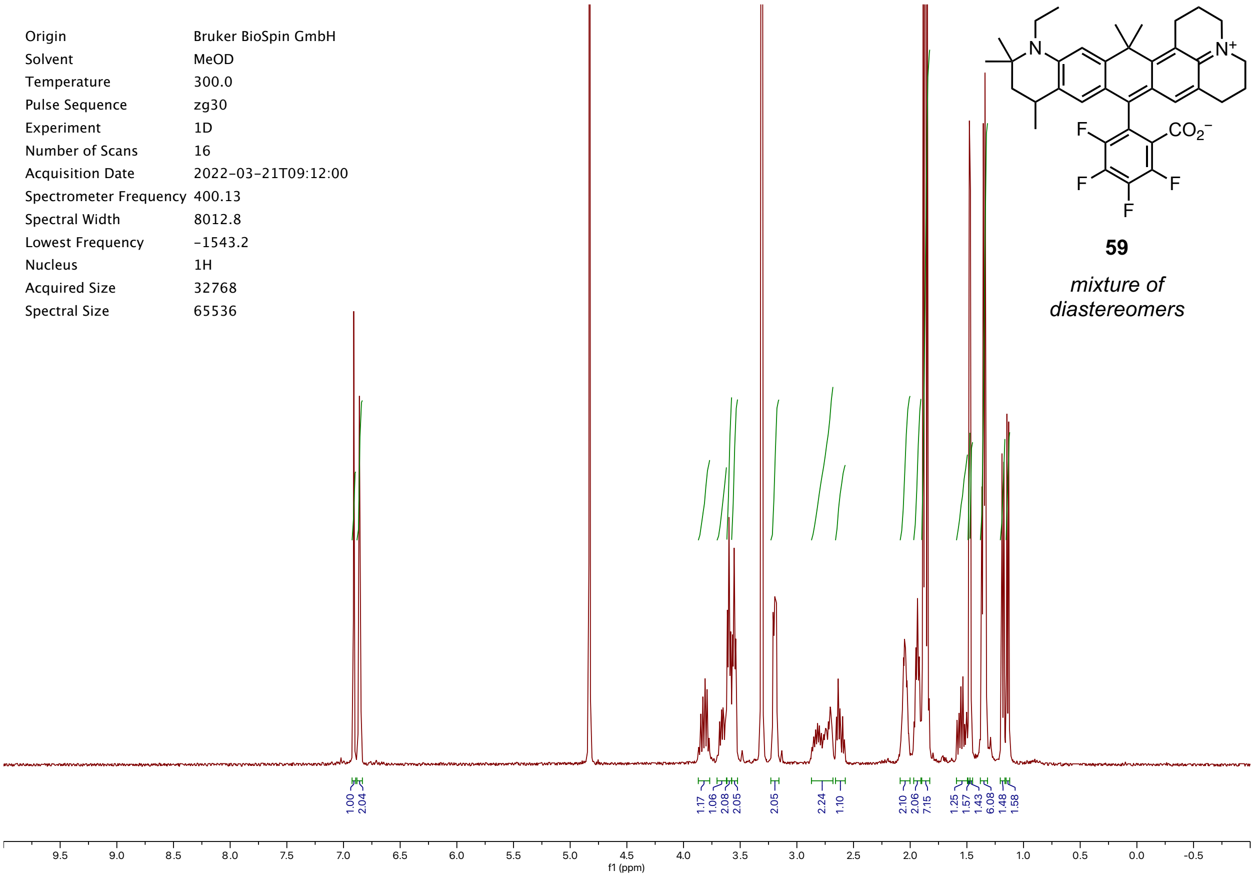

59

mixture of diastereomers

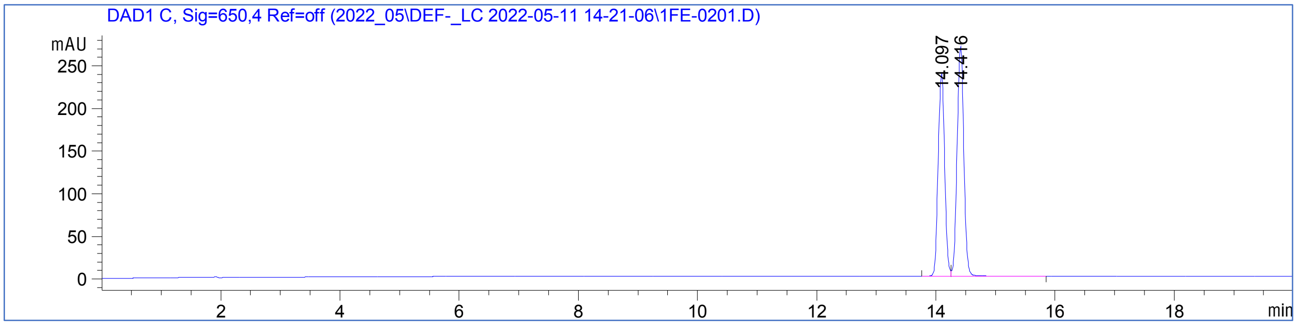

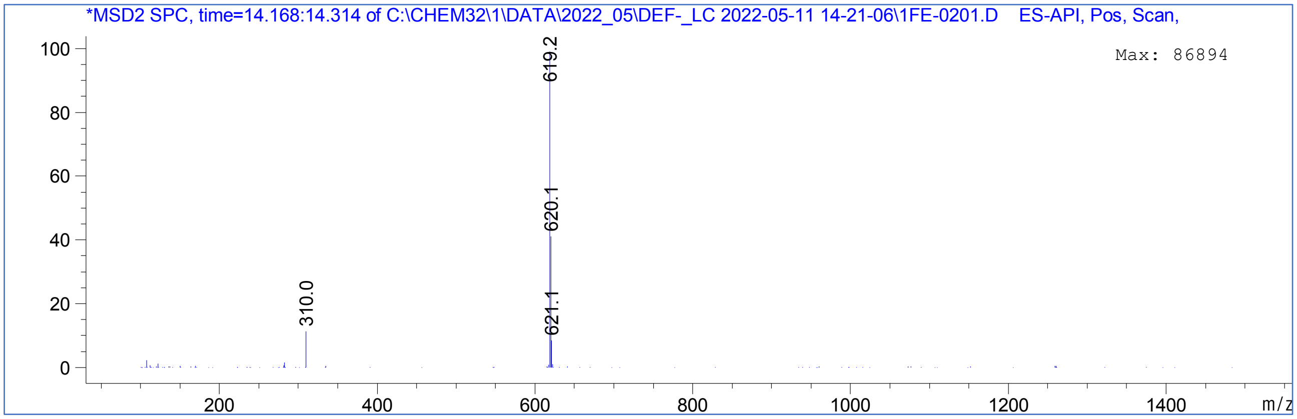

Origin	Bruker BioSpin GmbH
Solvent	MeOD
Temperature	300.0
Pulse Sequence	zg30
Experiment	1D
Number of Scans	16
Acquisition Date	2022-03-31T10:02:00
Spectrometer Frequency	400.13
Spectral Width	8012.8
Lowest Frequency	-1543.2
Nucleus	1H
Acquired Size	32768
Spectral Size	65536

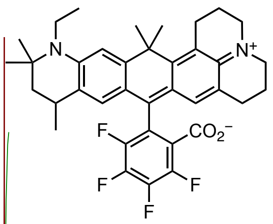

59

diastereomer 1

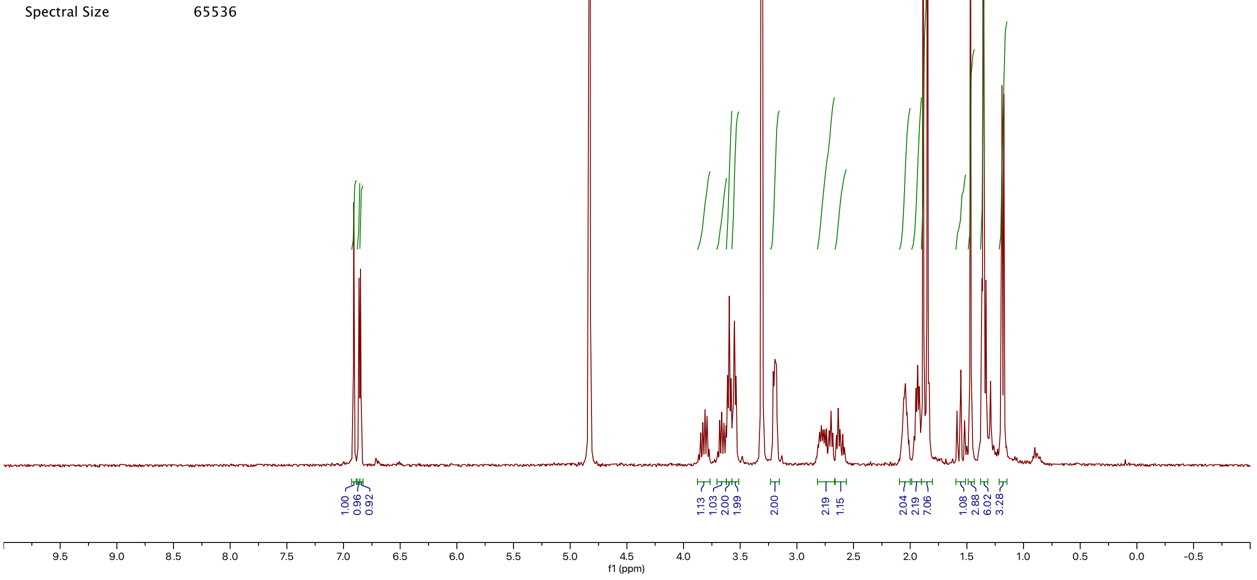

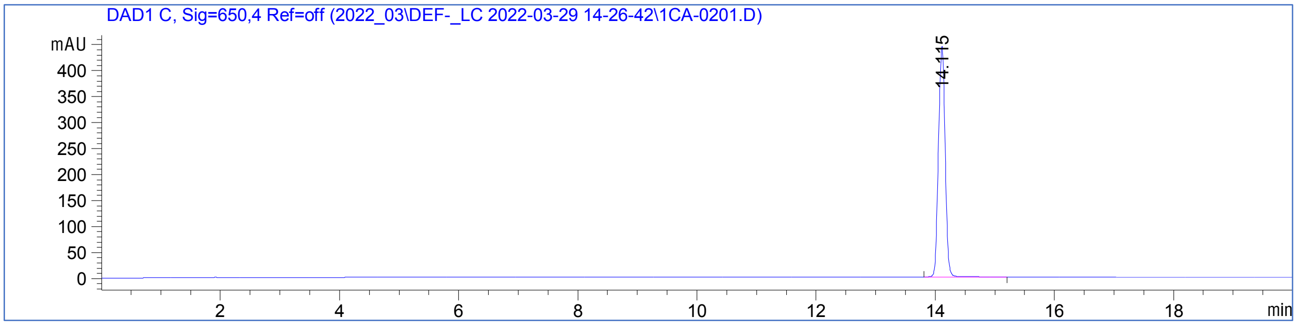

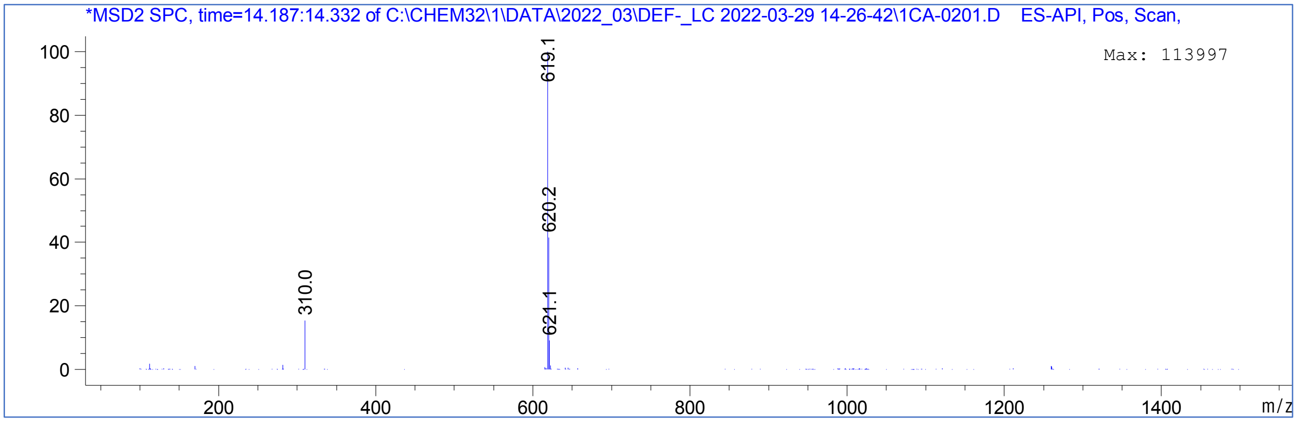

Origin	Bruker BioSpin GmbH
Solvent	MeOD
Temperature	300.0
Pulse Sequence	zg30
Experiment	1D
Number of Scans	16
Acquisition Date	2022-03-31T10:11:00
Spectrometer Frequency	400.13
Spectral Width	8012.8
Lowest Frequency	-1543.1
Nucleus	1H
Acquired Size	32768
Spectral Size	65536

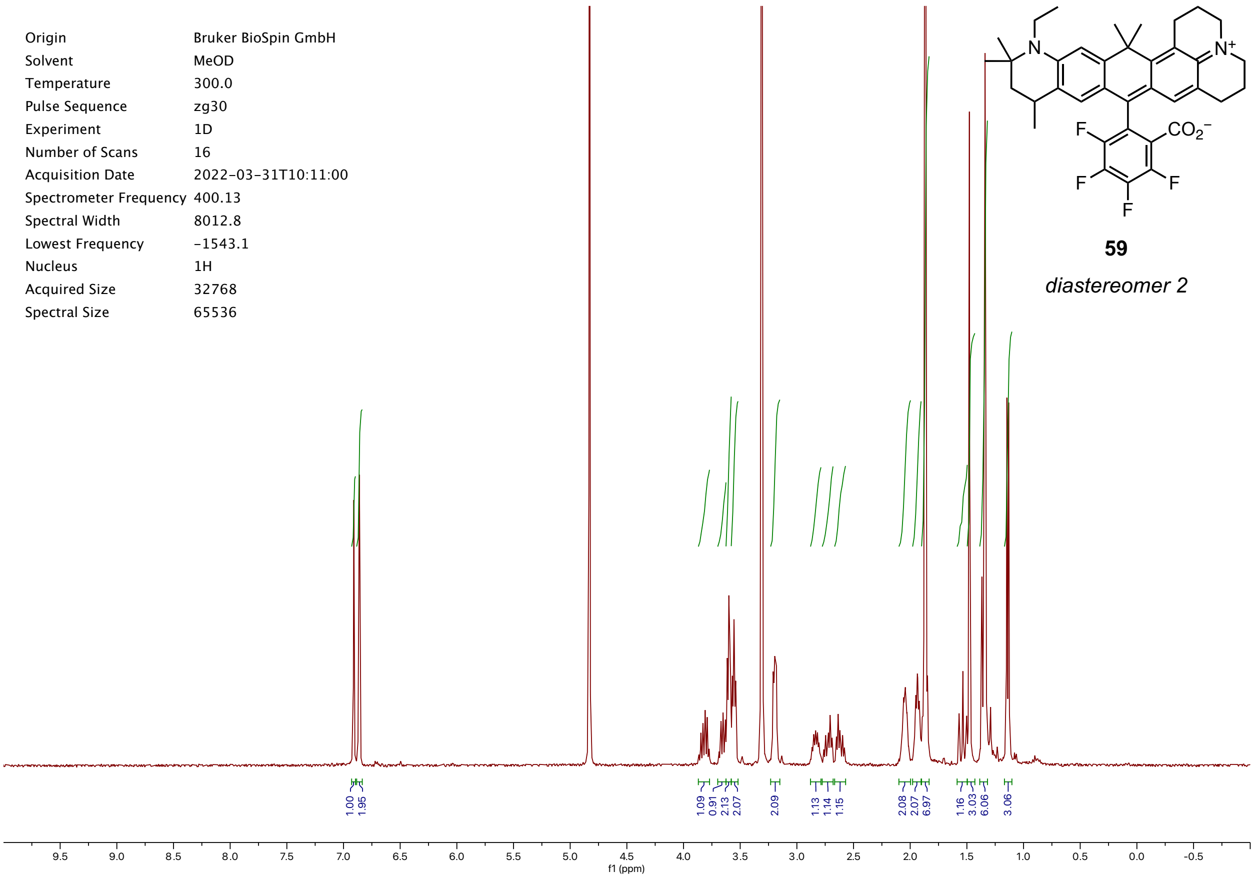

59

diastereomer 2

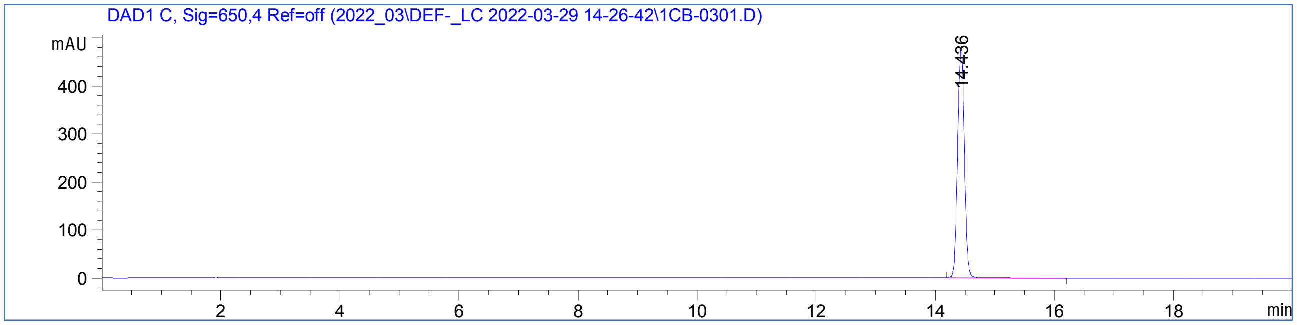

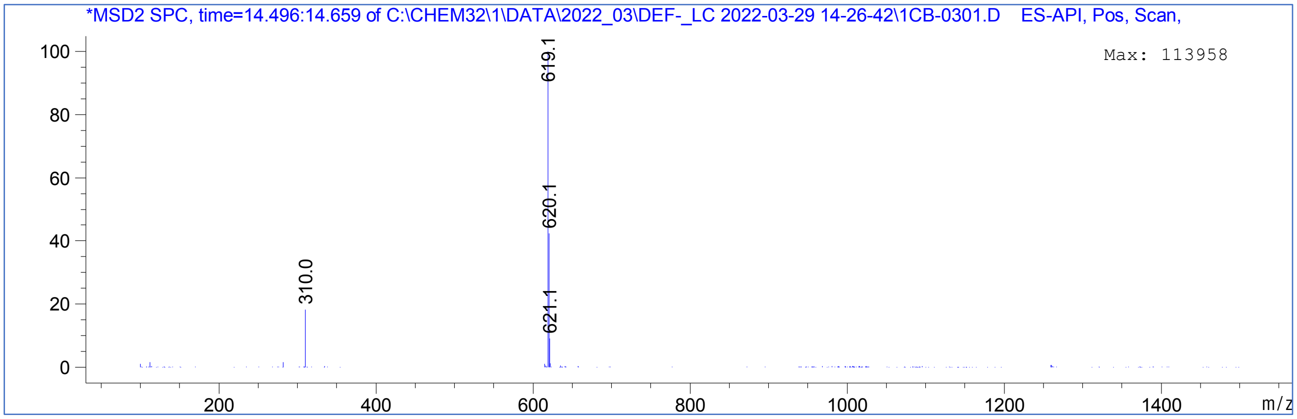

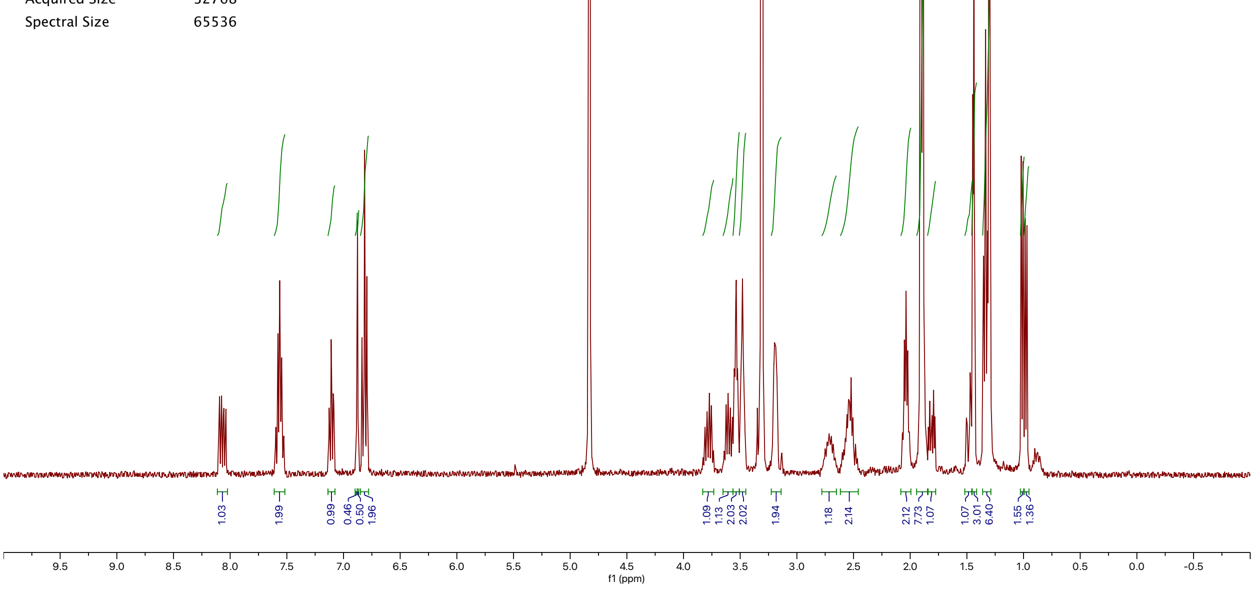

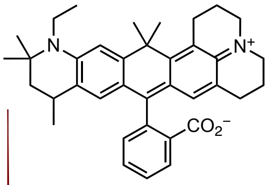

S57

mixture of diastereomers

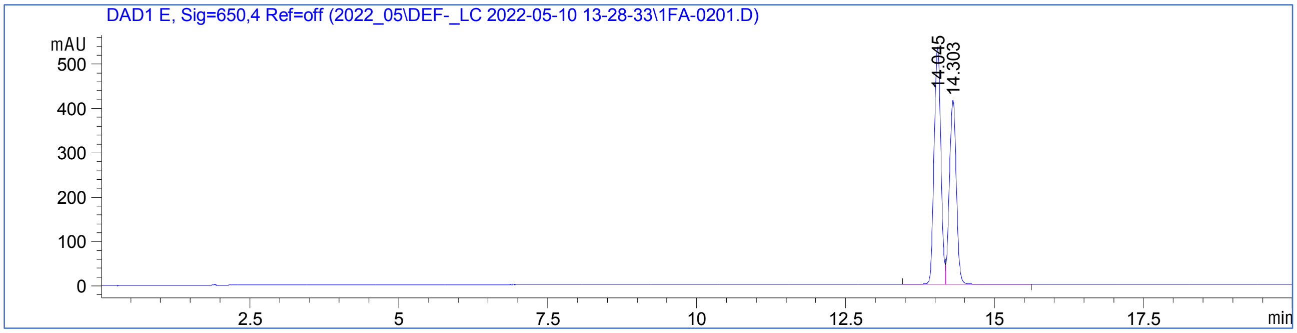

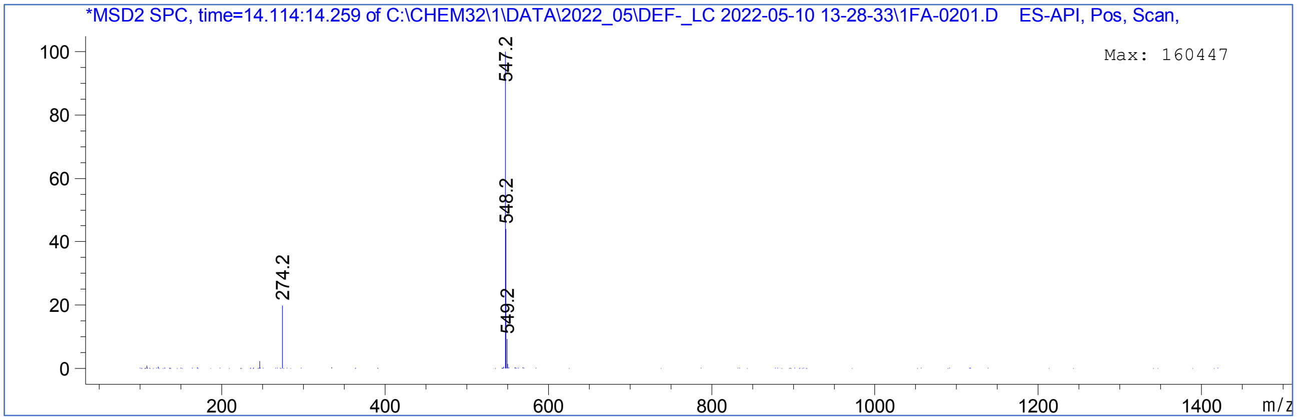

Origin	Bruker BioSpin GmbH
Solvent	MeOD
Temperature	300.0
Pulse Sequence	zg30
Experiment	1D
Number of Scans	16
Acquisition Date	2022-05-09T11:53:00
Spectrometer Frequency	400.13
Spectral Width	8012.8
Lowest Frequency	-1543.1
Nucleus	1H
Acquired Size	32768
Spectral Size	65536

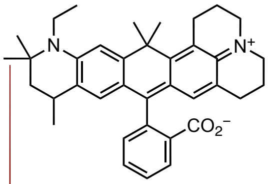

S57

diastereomer 1

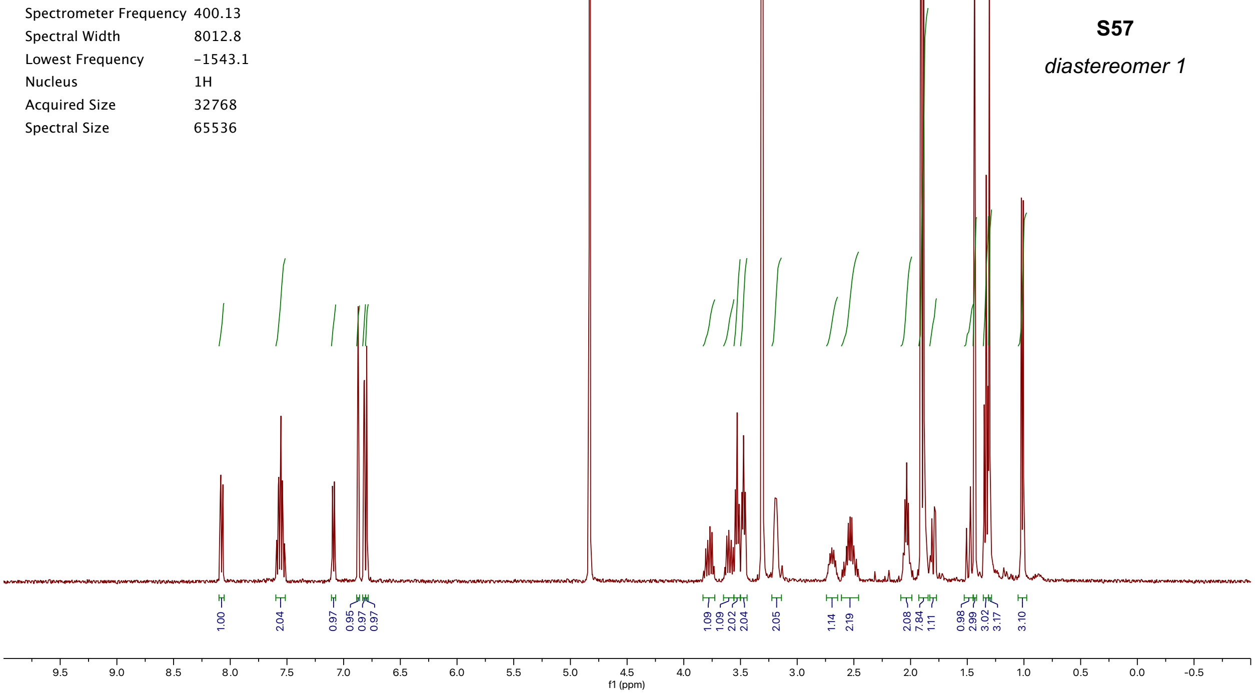

Origin	Bruker BioSpin GmbH
Solvent	MeOD
Temperature	300.0
Pulse Sequence	zgpg30
Experiment	1D
Number of Scans	4096
Acquisition Date	2022-05-11T22:56:00
Spectrometer Frequency	100.62
Spectral Width	24038.5
Lowest Frequency	-1818.3
Nucleus	13C
Acquired Size	32768
Spectral Size	65536

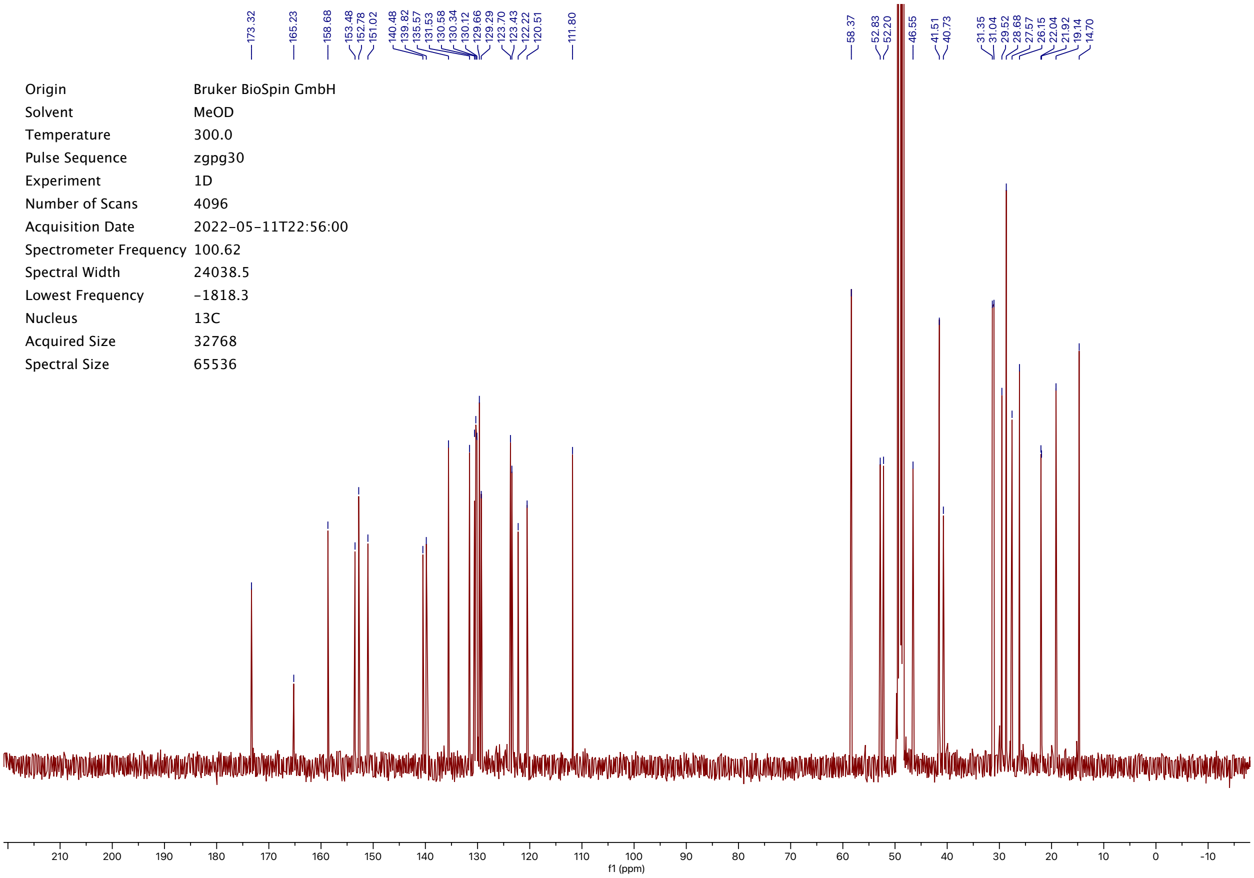

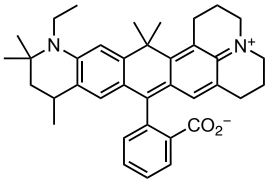

S57

diastereomer 1

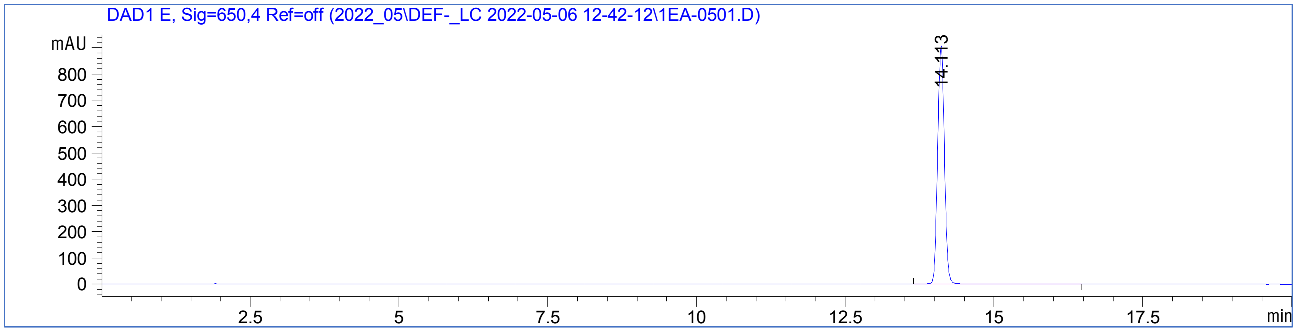

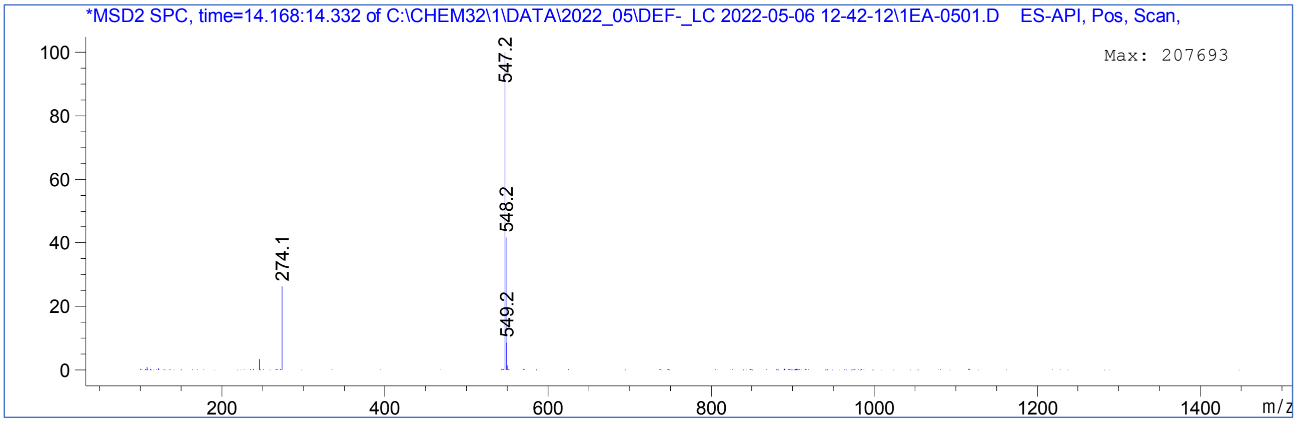

Origin	Bruker BioSpin GmbH
Solvent	MeOD
Temperature	300.0
Pulse Sequence	zg30
Experiment	1D
Number of Scans	16
Acquisition Date	2022-05-09T11:58:00
Spectrometer Frequency	400.13
Spectral Width	8012.8
Lowest Frequency	-1543.1
Nucleus	1H
Acquired Size	32768
Spectral Size	65536

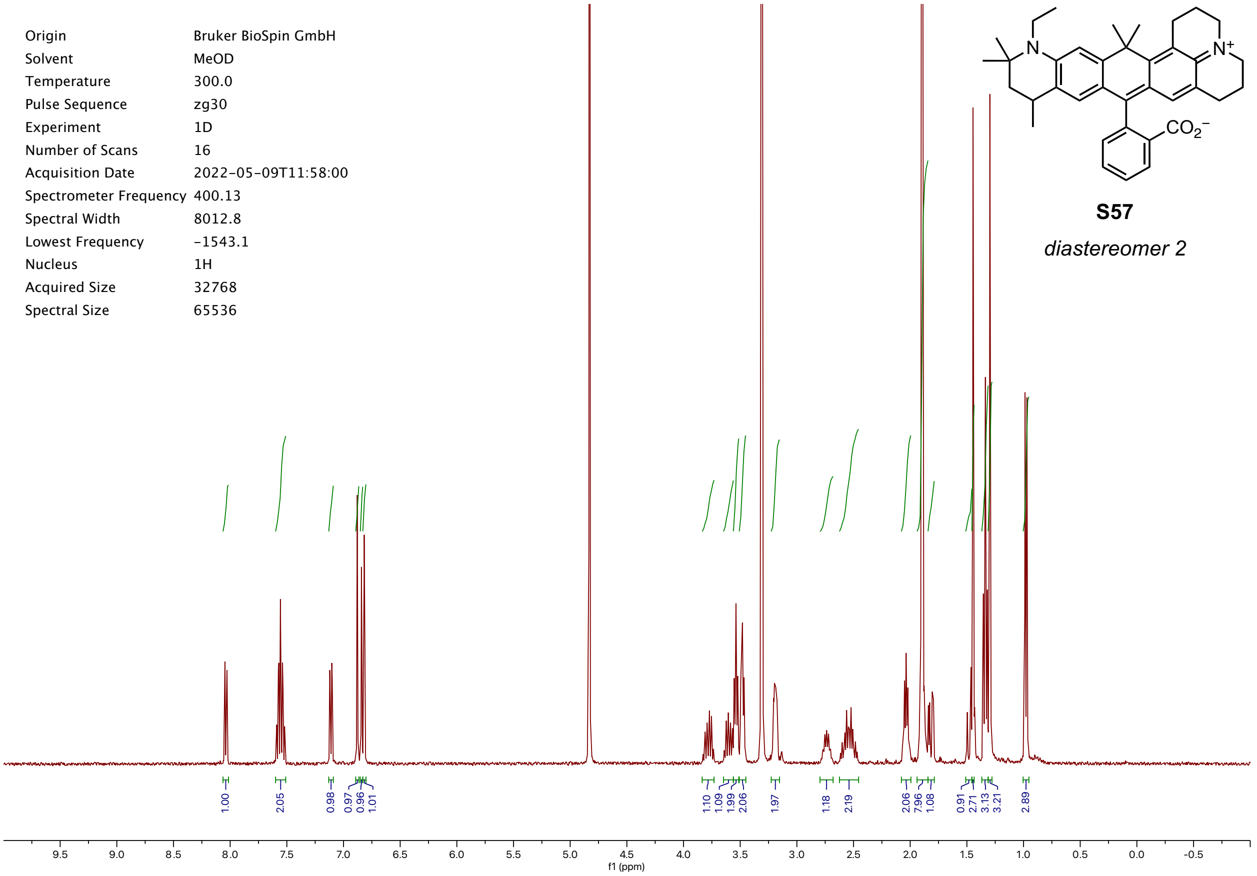

Origin	Bruker BioSpin GmbH
Solvent	MeOD
Temperature	300.0
Pulse Sequence	zgpg30
Experiment	1D
Number of Scans	2048
Acquisition Date	2022-05-12T13:12:00
Spectrometer Frequency	100.62
Spectral Width	24038.5
Lowest Frequency	-1818.0
Nucleus	13C
Acquired Size	32768
Spectral Size	65536

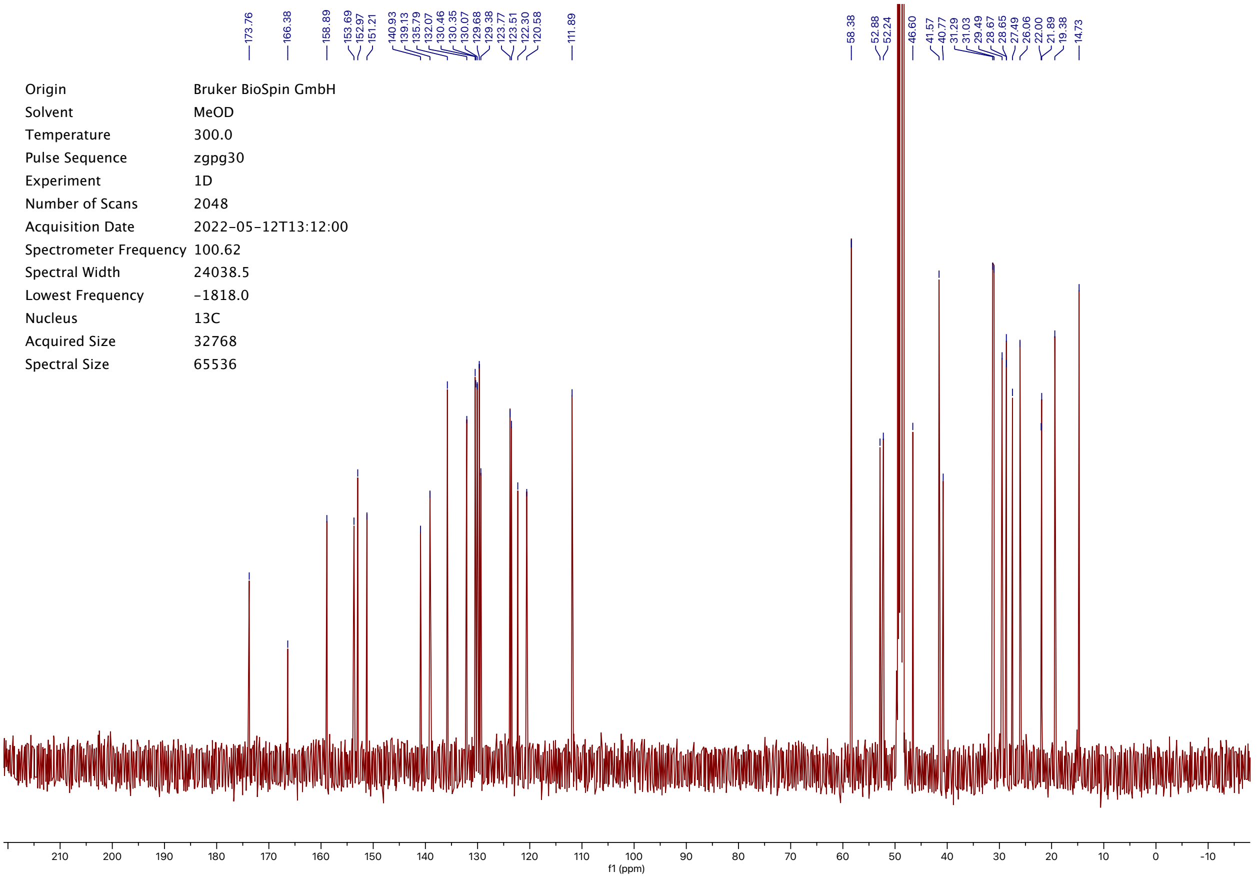

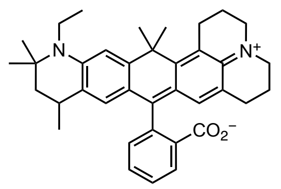

S57

diastereomer 2

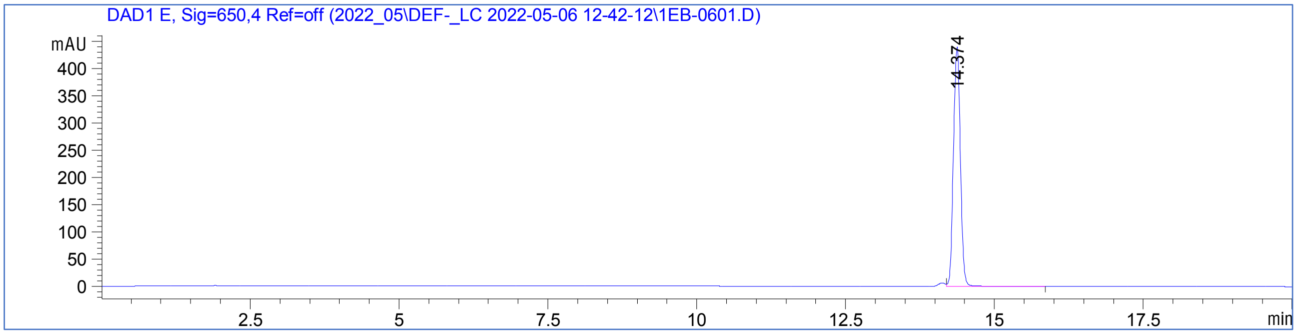

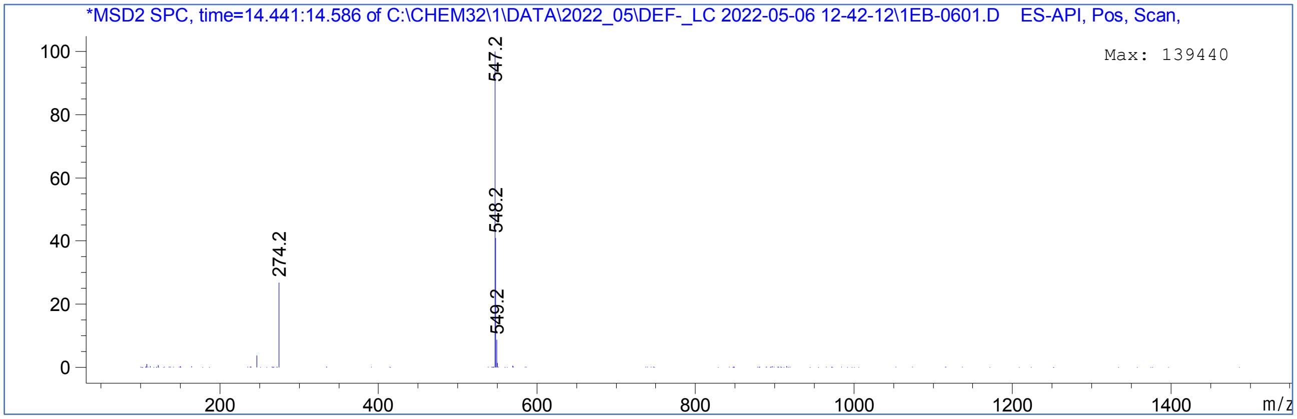

Origin	Bruker BioSpin GmbH
Solvent	CDCl3
Temperature	300.0
Pulse Sequence	zg30
Experiment	1D
Number of Scans	16
Acquisition Date	2019-11-01T16:14:00
Spectrometer Frequency	400.13
Spectral Width	8012.8
Lowest Frequency	-1545.8
Nucleus	1H
Acquired Size	32768
Spectral Size	65536

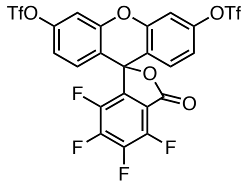

61

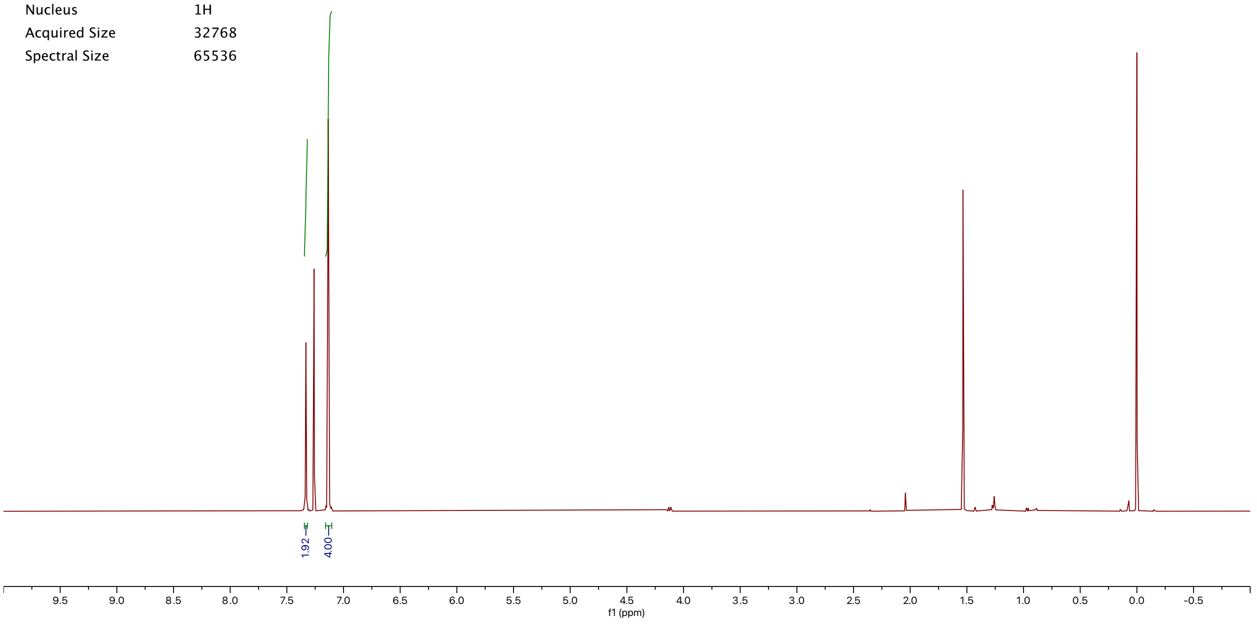

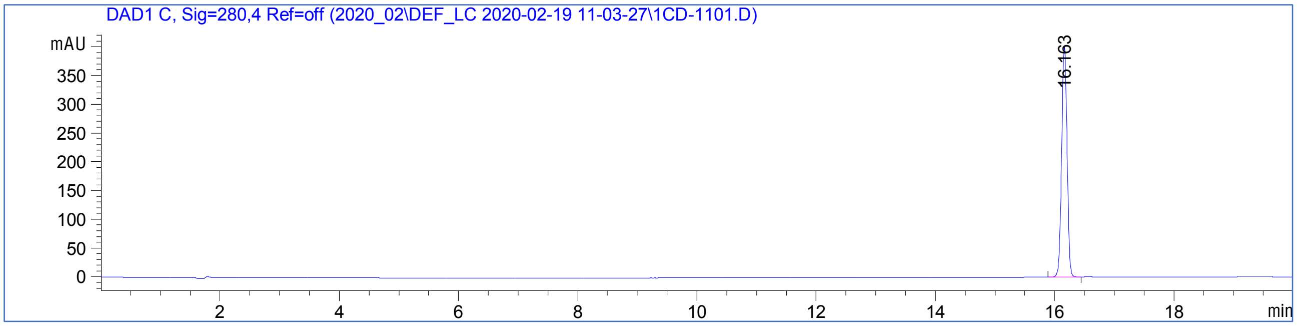

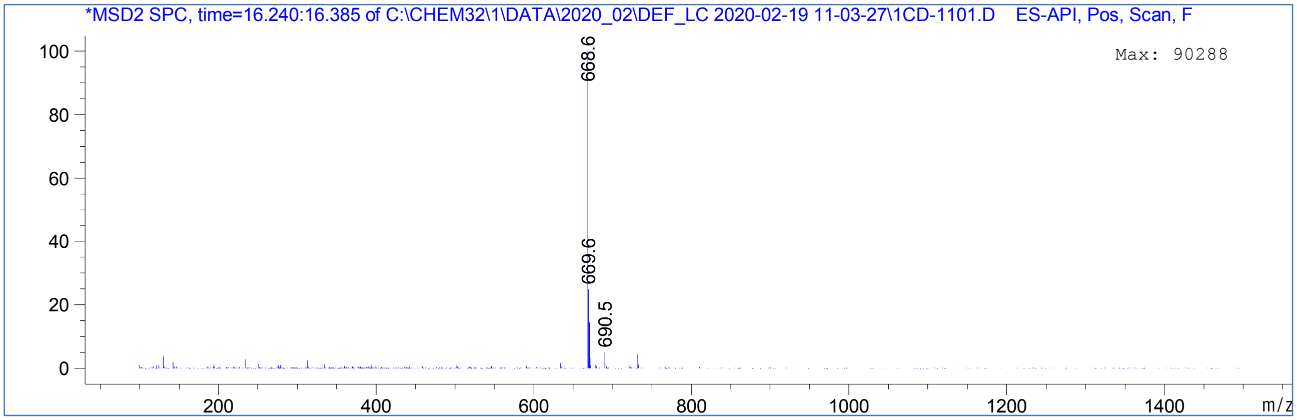

Origin	Bruker BioSpin GmbH
Solvent	CDCl3
Temperature	295.4
Pulse Sequence	zg30
Experiment	1D
Number of Scans	16
Acquisition Date	2019-12-20T14:25:00
Spectrometer Frequency	400.13
Spectral Width	8012.8
Lowest Frequency	-1545.1
Nucleus	1H
Acquired Size	32768
Spectral Size	65536

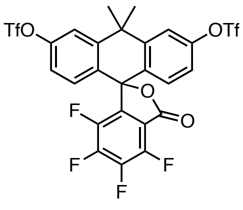

62

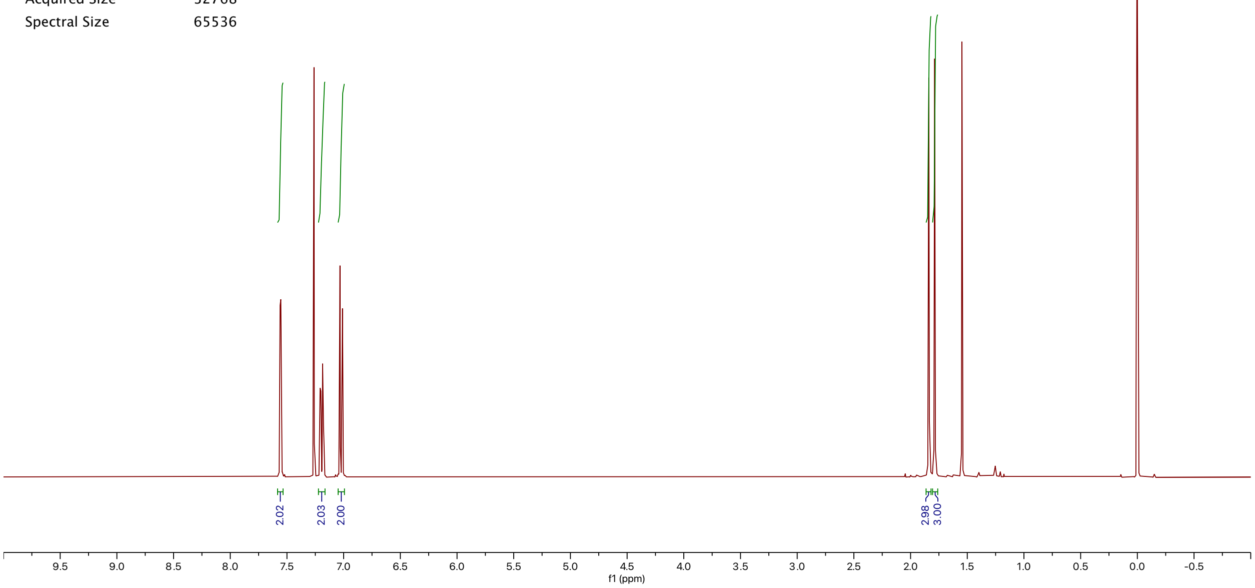

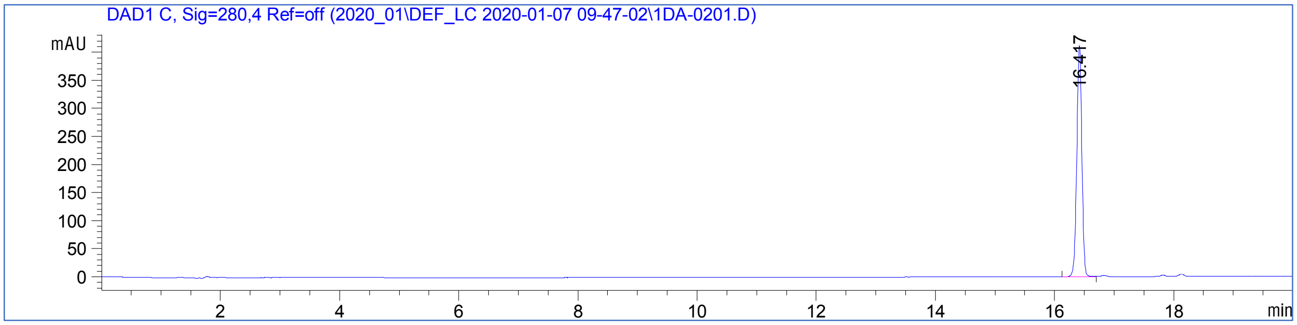

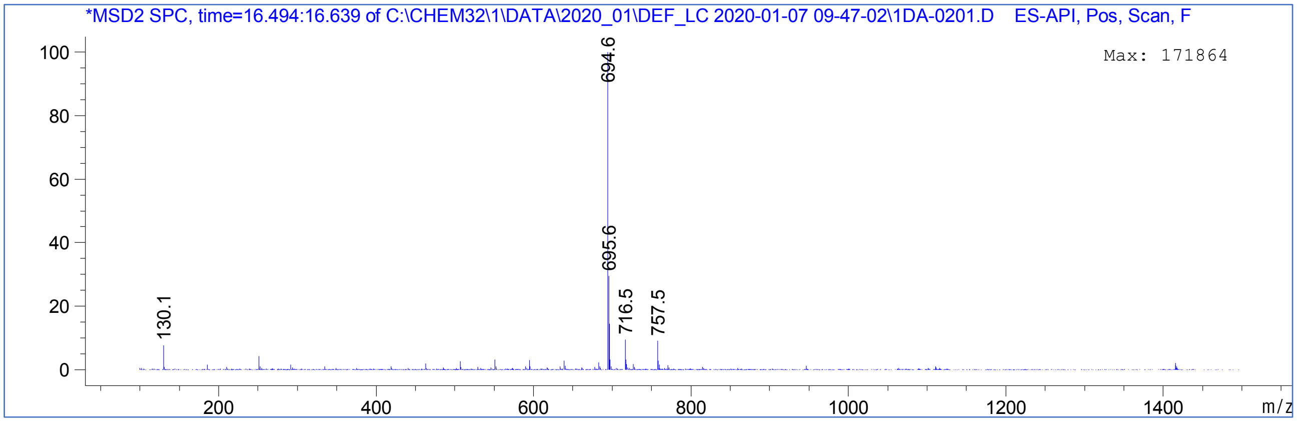

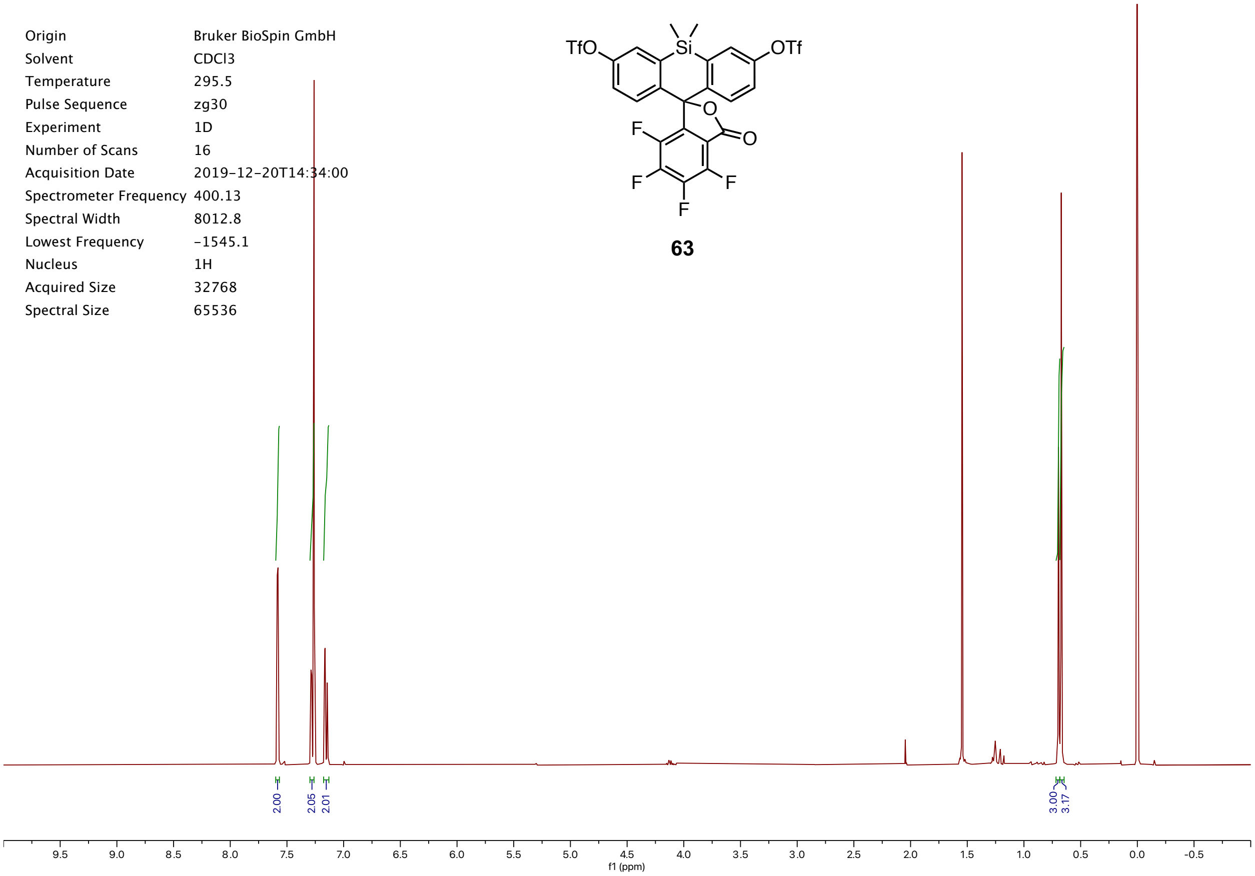
TfO
Si
OTf
O
F
O
F
F
F

63

Origin Bruker BioSpin GmbH
Solvent CDCl3
Temperature 295.5
Pulse Sequence zg30
Experiment 1D
Number of Scans 16
Acquisition Date 2019-12-20T14:34:00
Spectrometer Frequency 400.13
Spectral Width 8012.8
Lowest Frequency -1545.1
Nucleus 1H
Acquired Size 32768
Spectral Size 65536
2.00
2.05
2.01
3.00
3.17
9.5 9.0 8.5 8.0 7.5 7.0 6.5 6.0 5.5 5.0 4.5 4.0 3.5 3.0 2.5 2.0 1.5 1.0 0.5 0.0 -0.5
f1 (ppm)

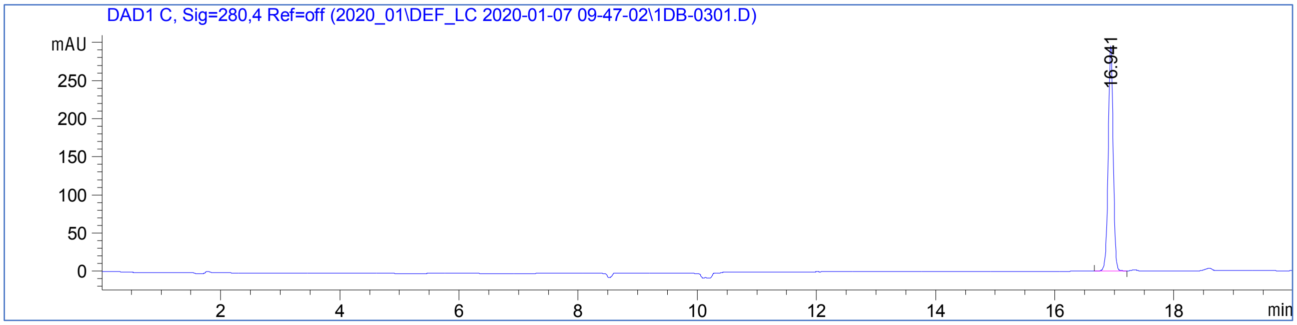
DAD1 C, Sig=280,4 Ref=off (2020_01\DEF_LC 2020-01-07 09-47-02\1DB-0301.D)
mAU
250
200
150
100
50
0
16.941
2 4 6 8 10 12 14 16 18
min

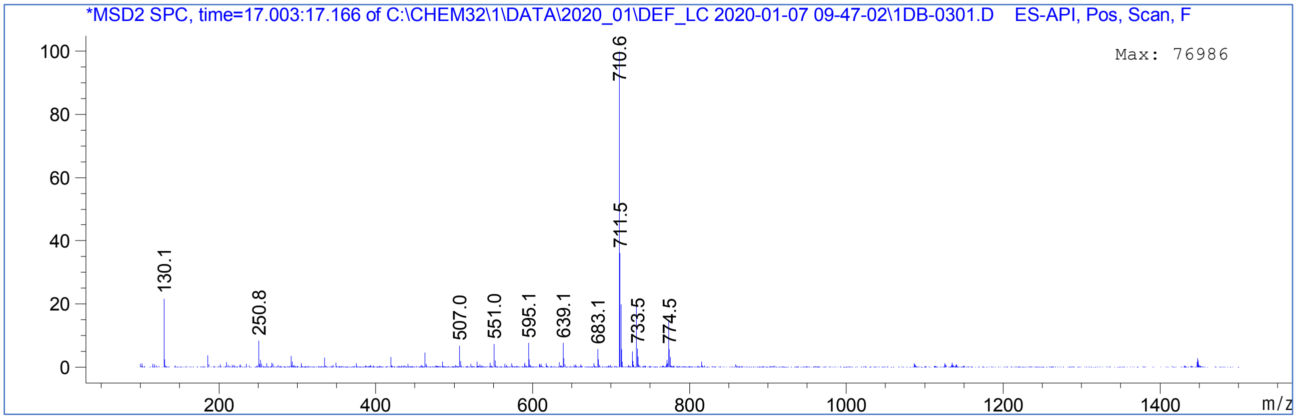
*MSD2 SPC, time=17.003:17.166 of C:\CHEM32\1\DATA\2020_01\DEF_LC 2020-01-07 09-47-02\1DB-0301.D ES-API, Pos, Scan, F
Max: 76986
100
80
60
40
20
0
710.6
711.5
130.1
250.8
507.0
551.0
595.1
639.1
683.1
733.5
774.5
200 400 600 800 1000 1200 1400
m/z

Origin	Bruker BioSpin GmbH
Solvent	CDCl3
Temperature	295.4
Pulse Sequence	zg30
Experiment	1D
Number of Scans	16
Acquisition Date	2020-10-19T12:34:00
Spectrometer Frequency	400.13
Spectral Width	8012.8
Lowest Frequency	-1545.2
Nucleus	1H
Acquired Size	32768
Spectral Size	65536

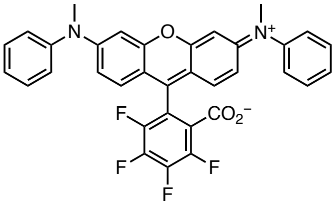

64

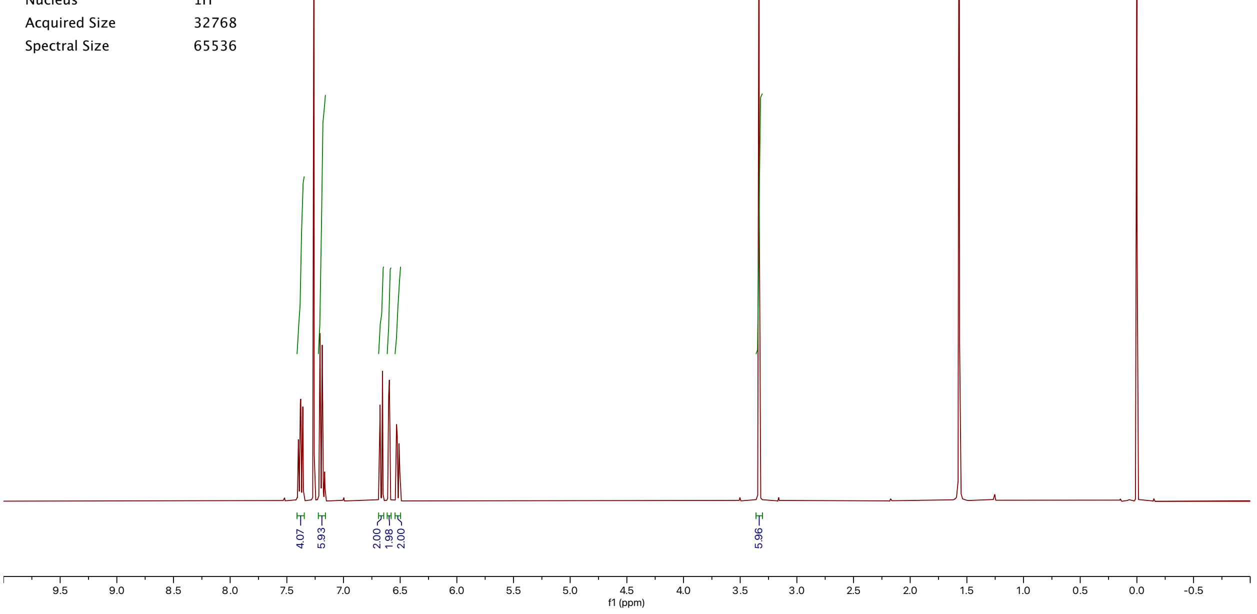

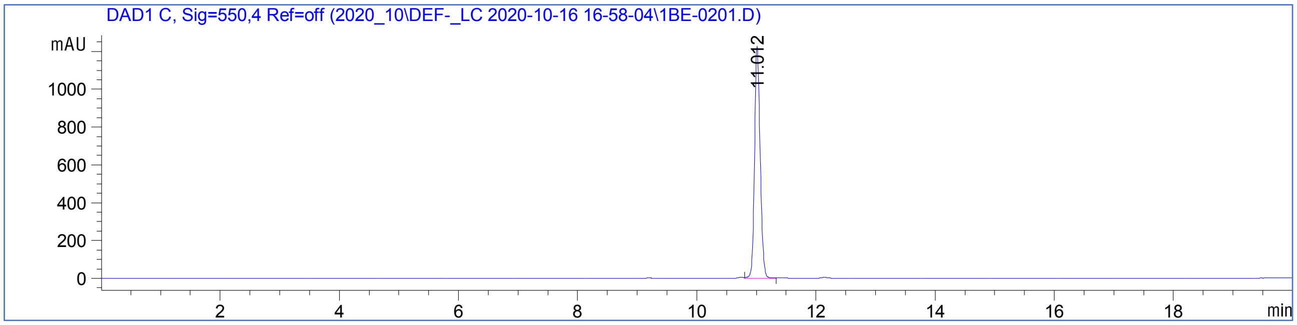

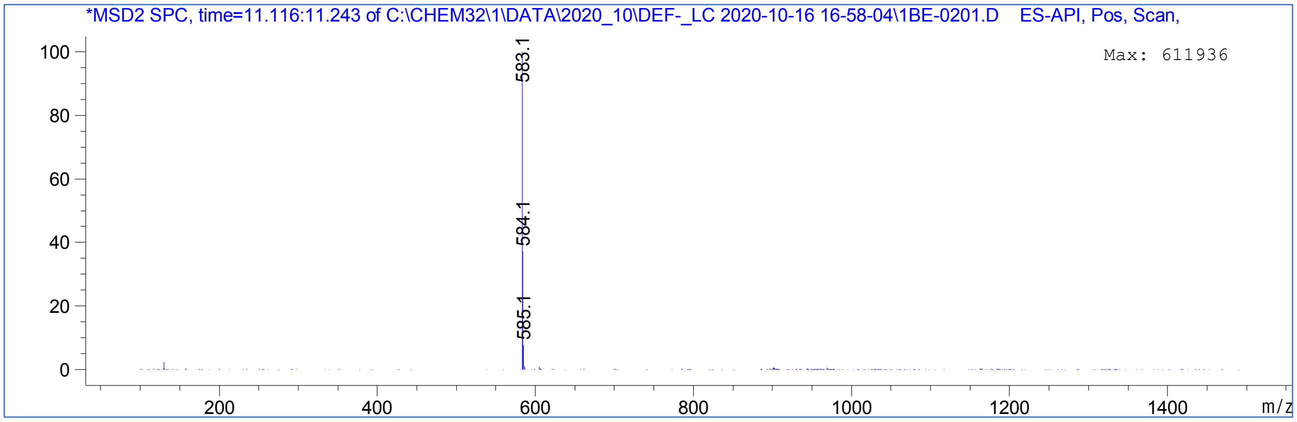

Origin	Bruker BioSpin GmbH
Solvent	CDCl3
Temperature	295.5
Pulse Sequence	zg30
Experiment	1D
Number of Scans	16
Acquisition Date	2020-10-19T12:43:00
Spectrometer Frequency	400.13
Spectral Width	8012.8
Lowest Frequency	-1545.7
Nucleus	1H
Acquired Size	32768
Spectral Size	65536

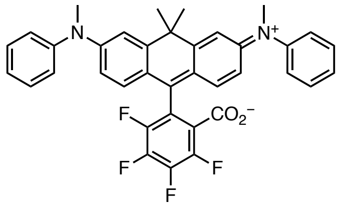

65

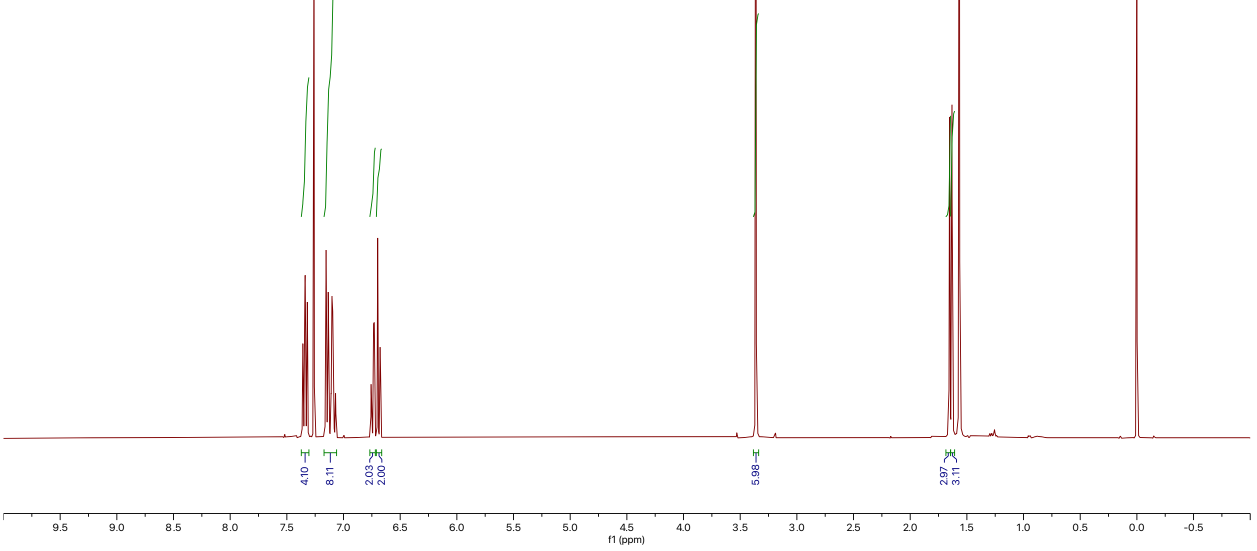

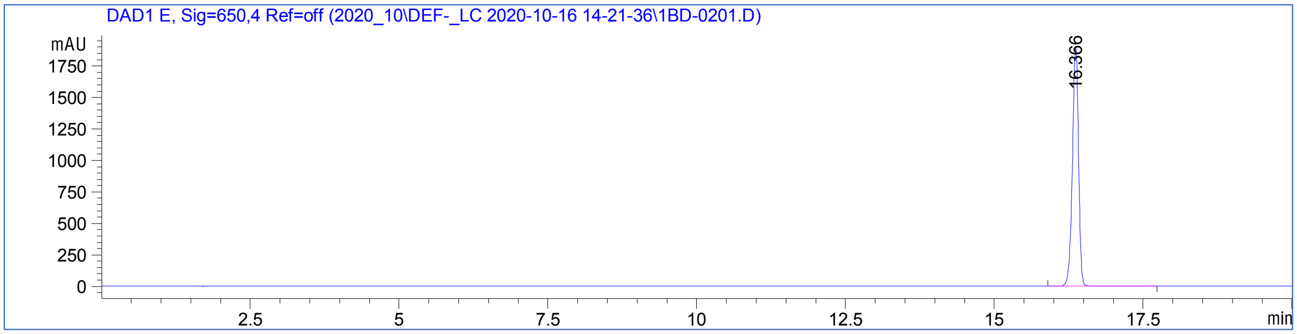

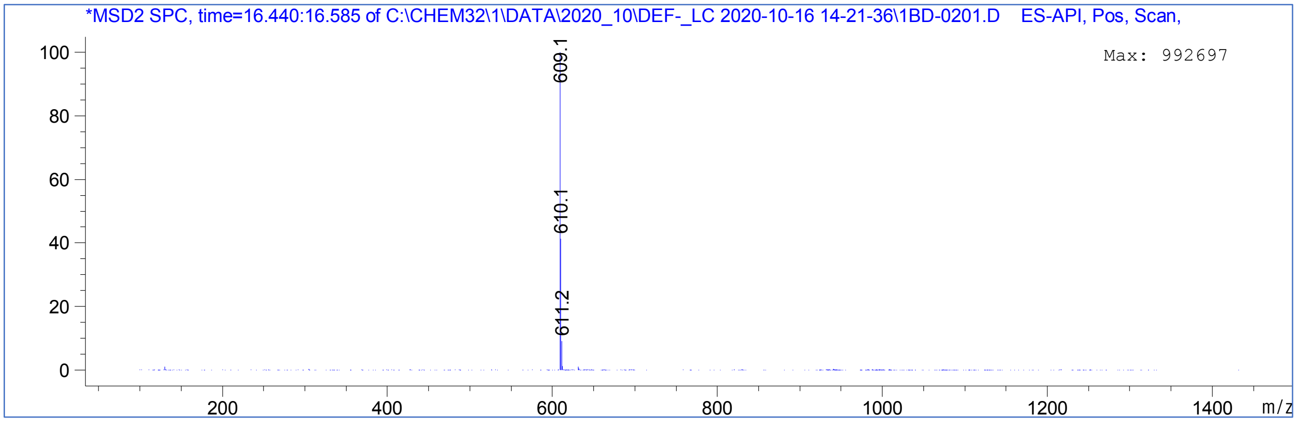

Origin	Bruker BioSpin GmbH
Solvent	CDCl3
Temperature	298.6
Pulse Sequence	zg30
Experiment	1D
Number of Scans	16
Acquisition Date	2022-11-07T09:36:00
Spectrometer Frequency	400.13
Spectral Width	8012.8
Lowest Frequency	-1546.3
Nucleus	1H
Acquired Size	32768
Spectral Size	65536

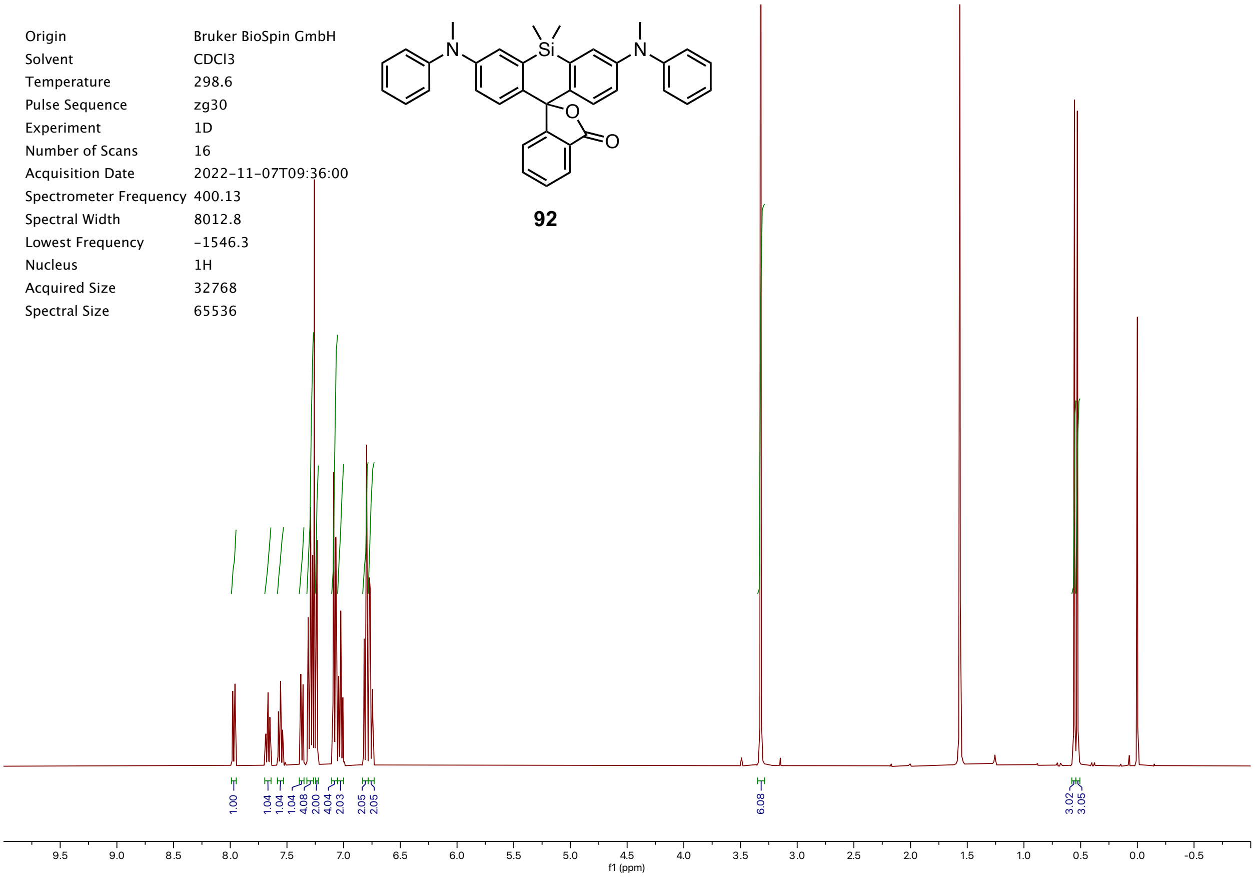

92

Origin	Bruker BioSpin GmbH
Solvent	CDCl3
Temperature	299.3
Pulse Sequence	zgpg30
Experiment	1D
Number of Scans	2048
Acquisition Date	2022-11-07T18:48:00
Spectrometer Frequency	100.62
Spectral Width	24038.5
Lowest Frequency	-1948.4
Nucleus	13C
Acquired Size	32768
Spectral Size	65536

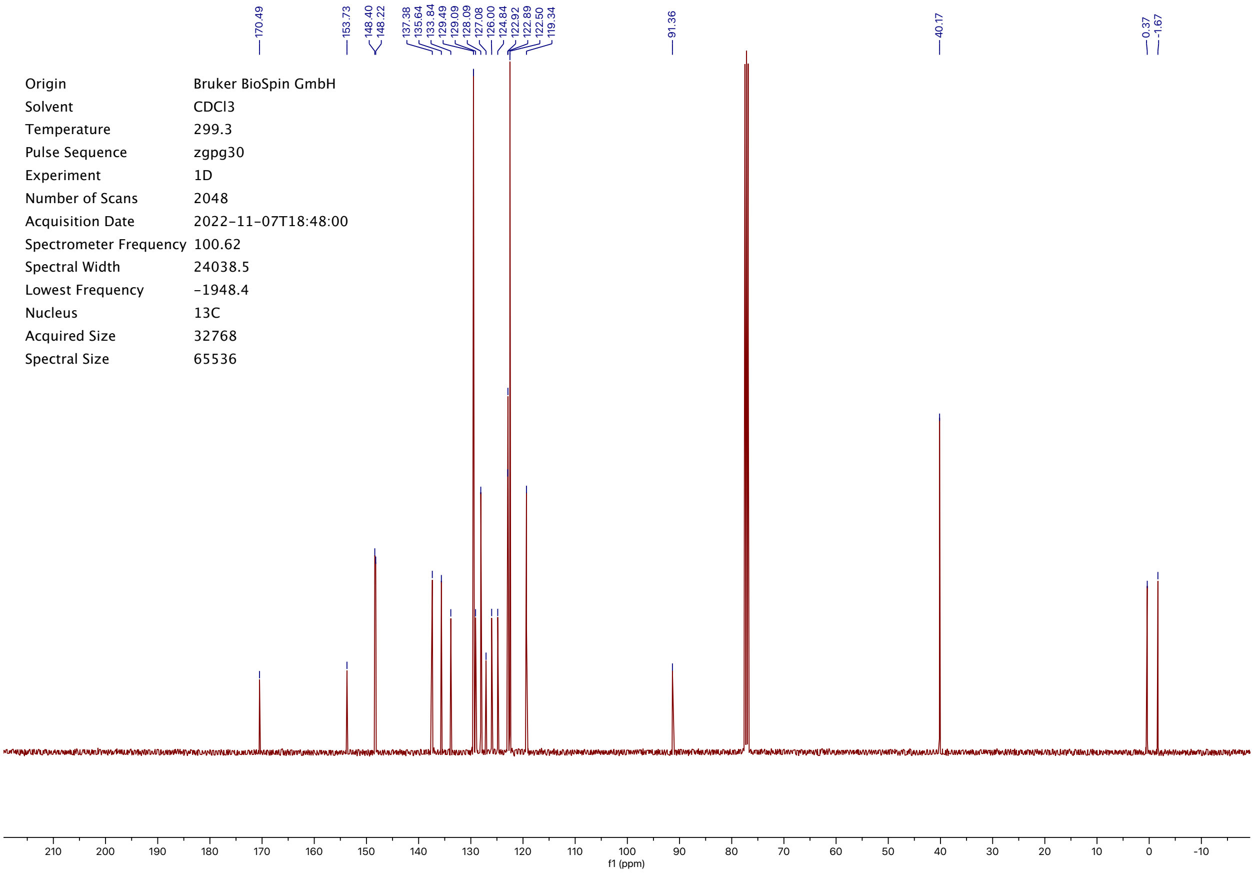

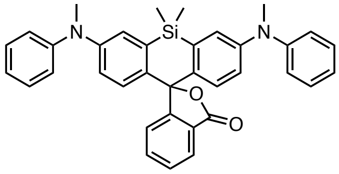

92

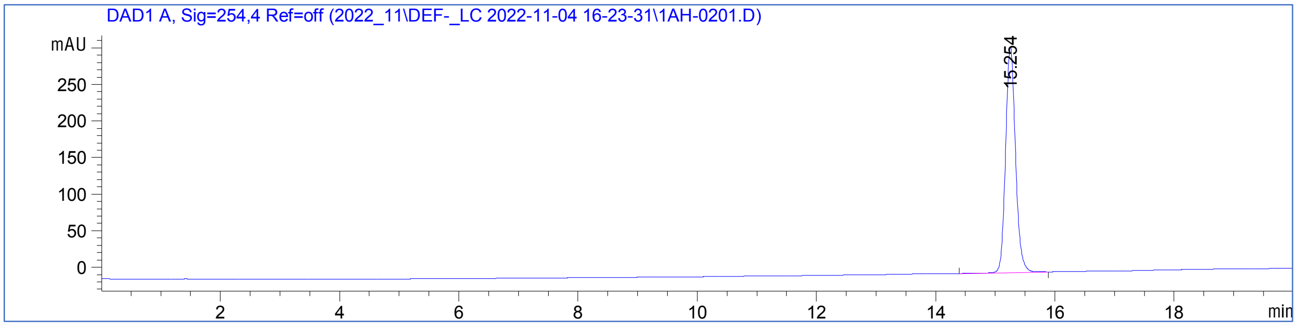

DAD1 A, Sig=254,4 Ref=off (2022_11\DEF-_LC 2022-11-04 16-23-31\1AH-0201.D)
mAU
15.254
min

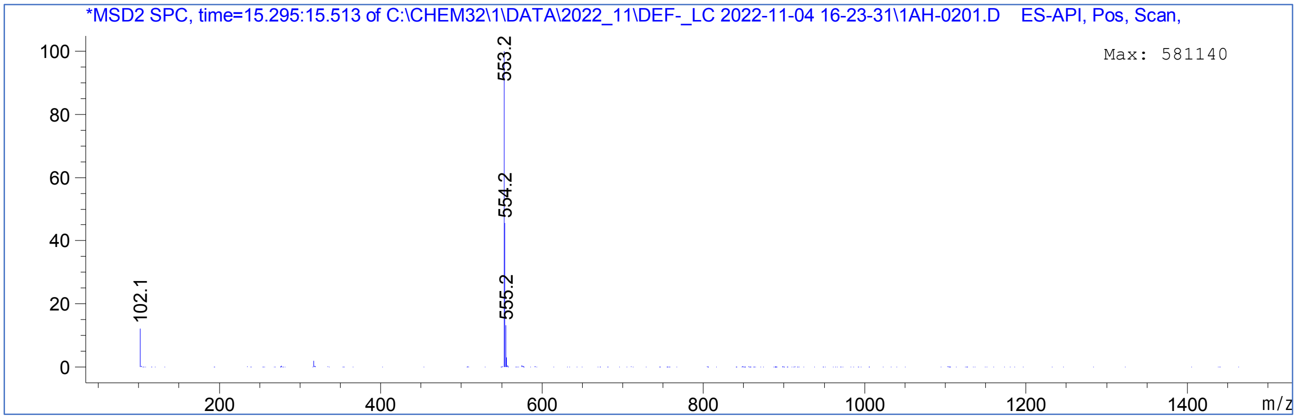

*MSD2 SPC, time=15.295:15.513 of C:\CHEM32\1\DATA\2022_11\DEF-_LC 2022-11-04 16-23-31\1AH-0201.D ES-API, Pos, Scan,
Max: 581140
553.2
554.2
555.2
102.1
m/z

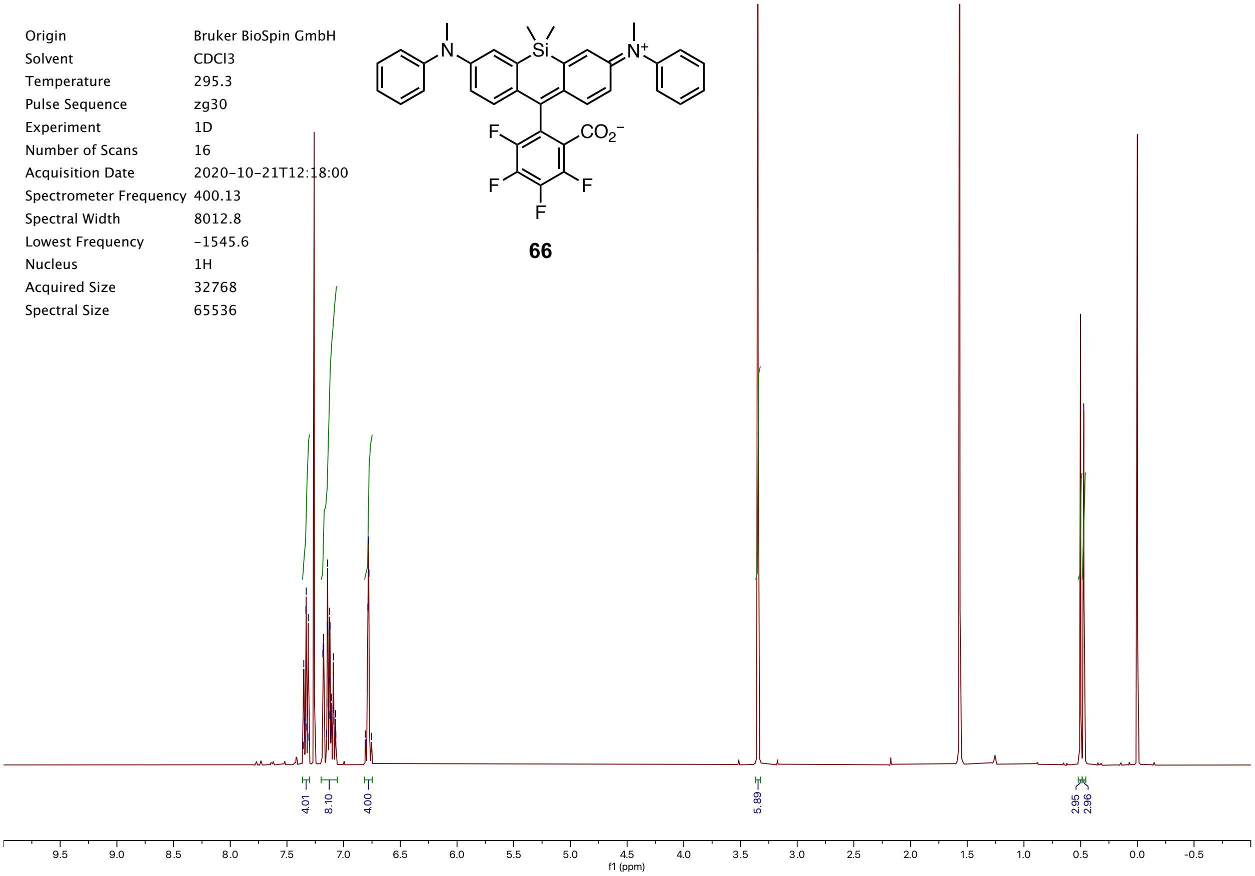
Origin Bruker BioSpin GmbH
Solvent CDCl3
Temperature 295.3
Pulse Sequence zg30
Experiment 1D
Number of Scans 16
Acquisition Date 2020-10-21T12:18:00
Spectrometer Frequency 400.13
Spectral Width 8012.8
Lowest Frequency -1545.6
Nucleus 1H
Acquired Size 32768
Spectral Size 65536
4.01
8.10
4.00
5.89
2.95
2.96
f1 (ppm)
Si
N
N+
F
F
F
F
CO2−

66

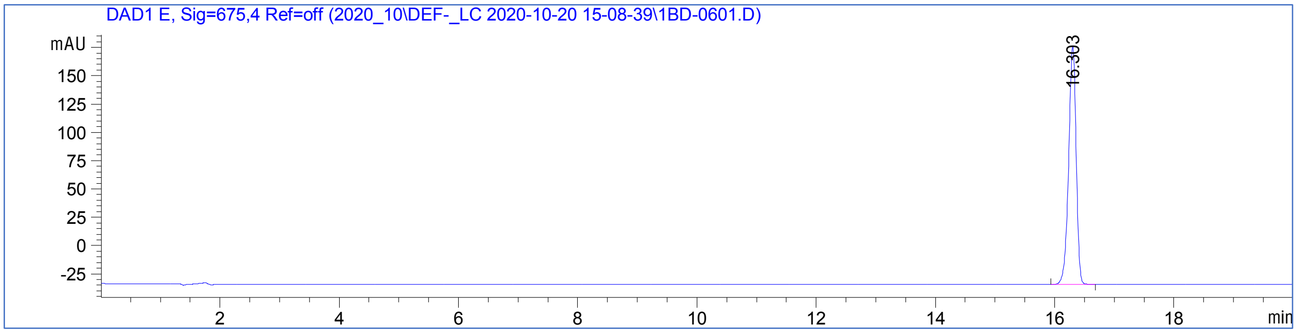
DAD1 E, Sig=675,4 Ref=off (2020_10\DEF-_LC 2020-10-20 15-08-39\1BD-0601.D)
mAU
16.303
min

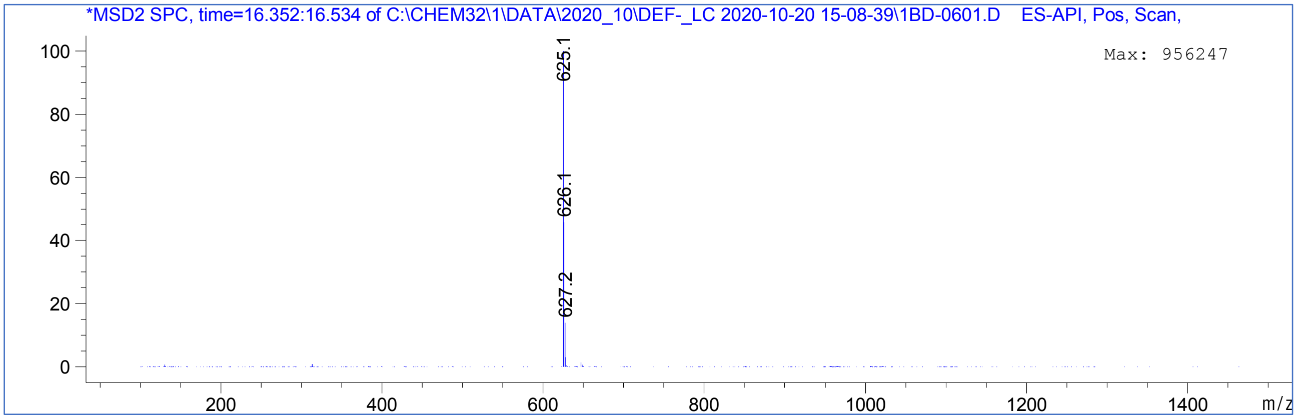
*MSD2 SPC, time=16.352:16.534 of C:\CHEM32\1\DATA\2020_10\DEF-_LC 2020-10-20 15-08-39\1BD-0601.D ES-API, Pos, Scan,
Max: 956247
625.1
626.1
627.2
m/z

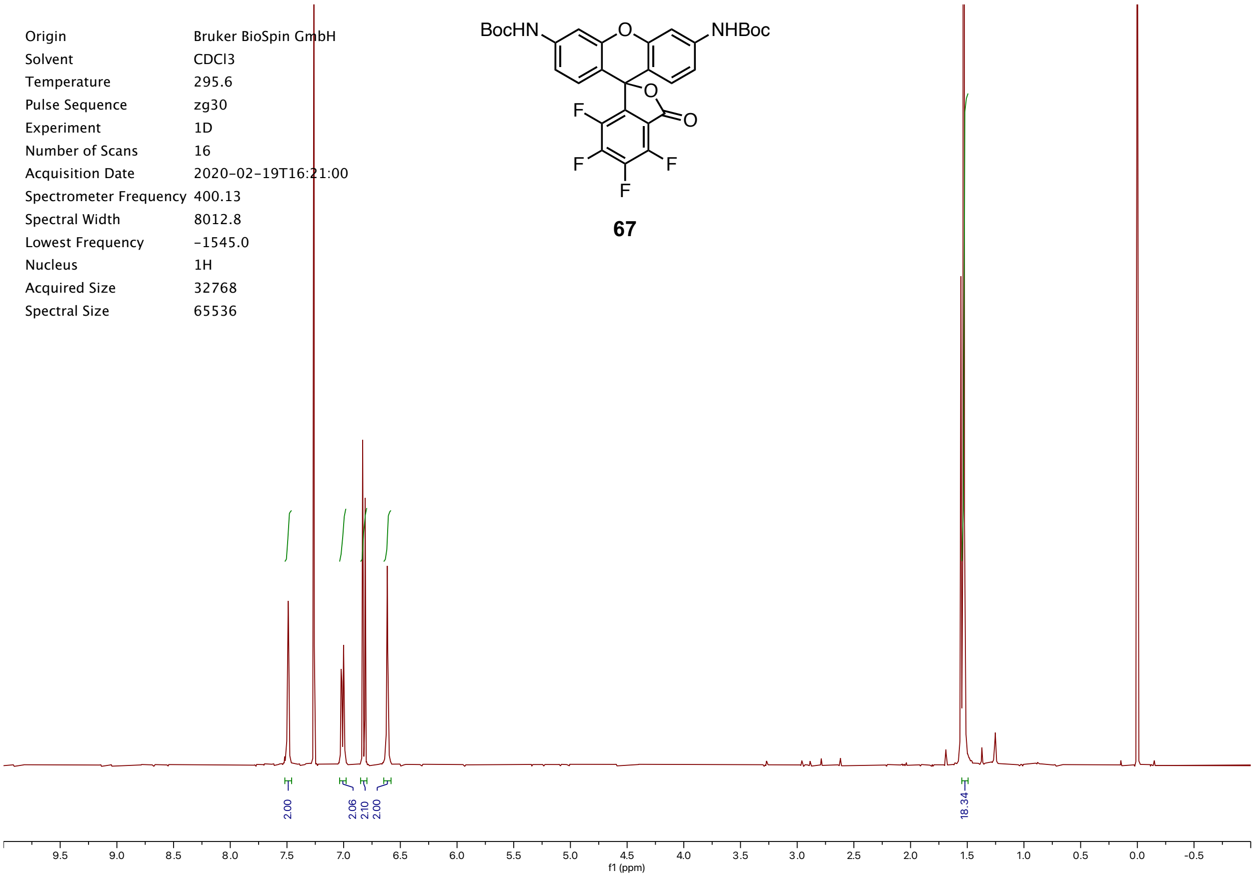
Origin Bruker BioSpin GmbH
Solvent CDCl3
Temperature 295.6
Pulse Sequence zg30
Experiment 1D
Number of Scans 16
Acquisition Date 2020-02-19T16:21:00
Spectrometer Frequency 400.13
Spectral Width 8012.8
Lowest Frequency -1545.0
Nucleus 1H
Acquired Size 32768
Spectral Size 65536
2.00
2.06
2.10
2.00
18.34
f1 (ppm)
BocHN
NHBoc
O
O
O
F
F
F
F

67

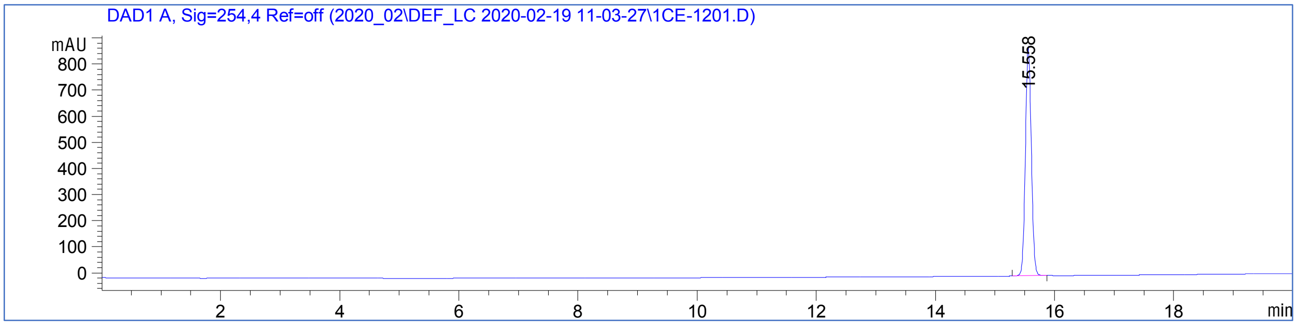
DAD1 A, Sig=254,4 Ref=off (2020_02\DEF_LC 2020-02-19 11-03-27\1CE-1201.D)
mAU
15.558
min

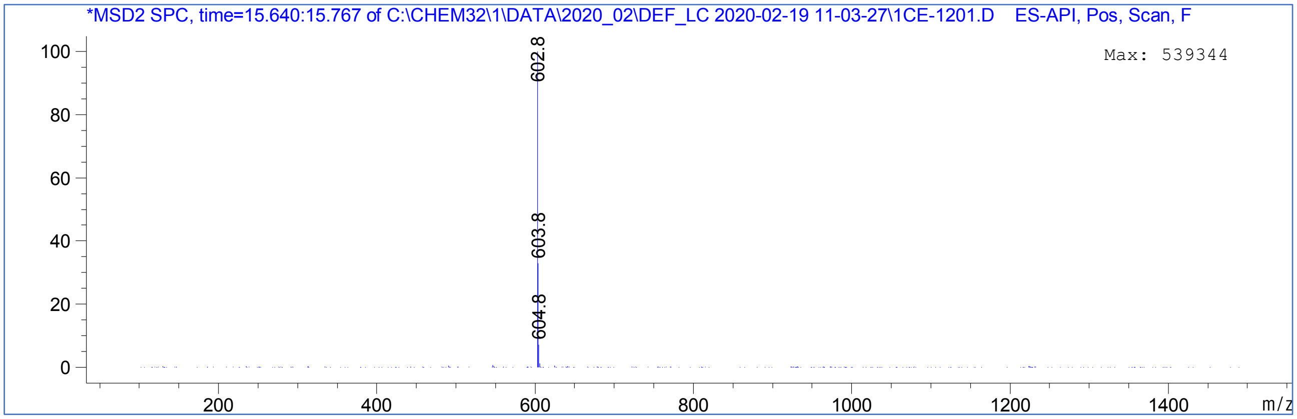
*MSD2 SPC, time=15.640:15.767 of C:\CHEM32\1\DATA\2020_02\DEF_LC 2020-02-19 11-03-27\1CE-1201.D ES-API, Pos, Scan, F
Max: 539344
602.8
603.8
604.8
m/z

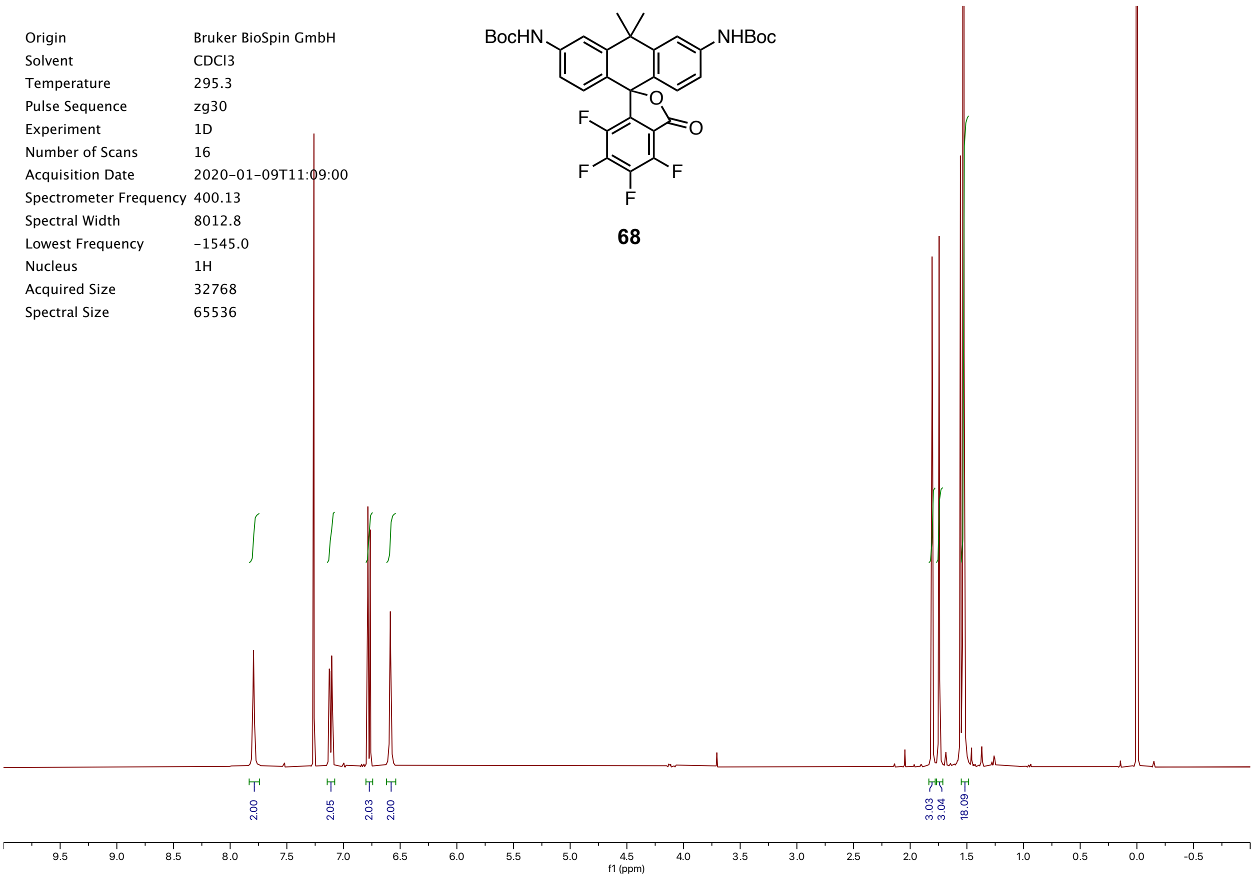
Origin Bruker BioSpin GmbH
Solvent CDCl3
Temperature 295.3
Pulse Sequence zg30
Experiment 1D
Number of Scans 16
Acquisition Date 2020-01-09T11:09:00
Spectrometer Frequency 400.13
Spectral Width 8012.8
Lowest Frequency -1545.0
Nucleus 1H
Acquired Size 32768
Spectral Size 65536
BocHN
NHBoc
O
O
F
F
F
F
68
2.00
2.05
2.03
2.00
3.03
3.04
18.09
f1 (ppm)

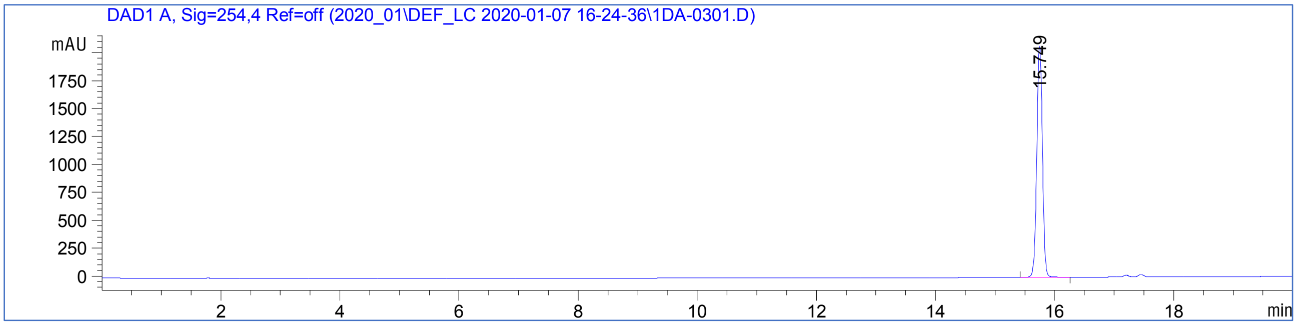
DAD1 A, Sig=254,4 Ref=off (2020_01\DEF_LC 2020-01-07 16-24-36\1DA-0301.D)
mAU
15.749
min

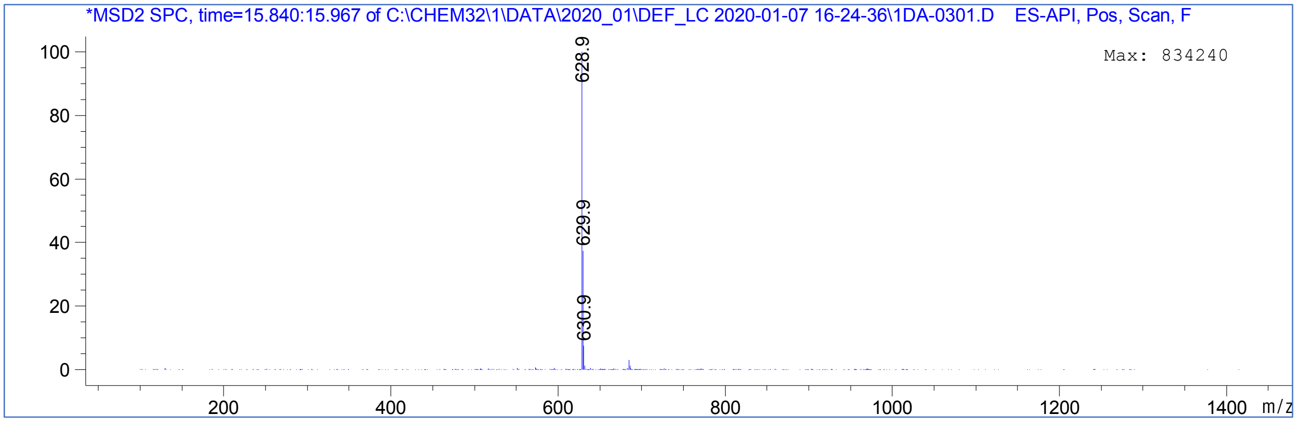
*MSD2 SPC, time=15.840:15.967 of C:\CHEM32\1\DATA\2020_01\DEF_LC 2020-01-07 16-24-36\1DA-0301.D ES-API, Pos, Scan, F
Max: 834240
628.9
629.9
630.9
m/z

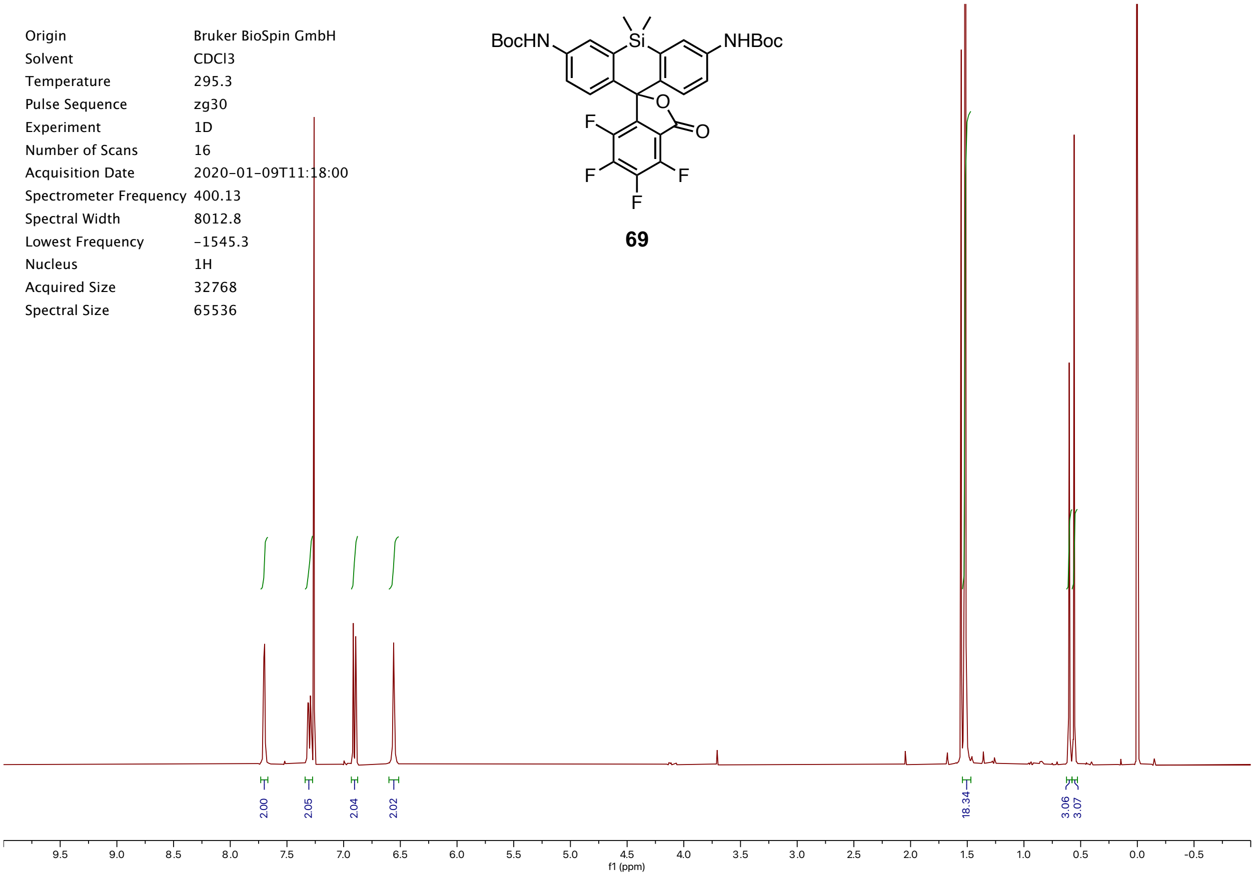
Origin Bruker BioSpin GmbH
Solvent CDCl3
Temperature 295.3
Pulse Sequence zg30
Experiment 1D
Number of Scans 16
Acquisition Date 2020-01-09T11:18:00
Spectrometer Frequency 400.13
Spectral Width 8012.8
Lowest Frequency -1545.3
Nucleus 1H
Acquired Size 32768
Spectral Size 65536
BocHN
NHBoc
Si
O
O
F
F
F
F
69
2.00
2.05
2.04
2.02
18.34
3.06
3.07
f1 (ppm)

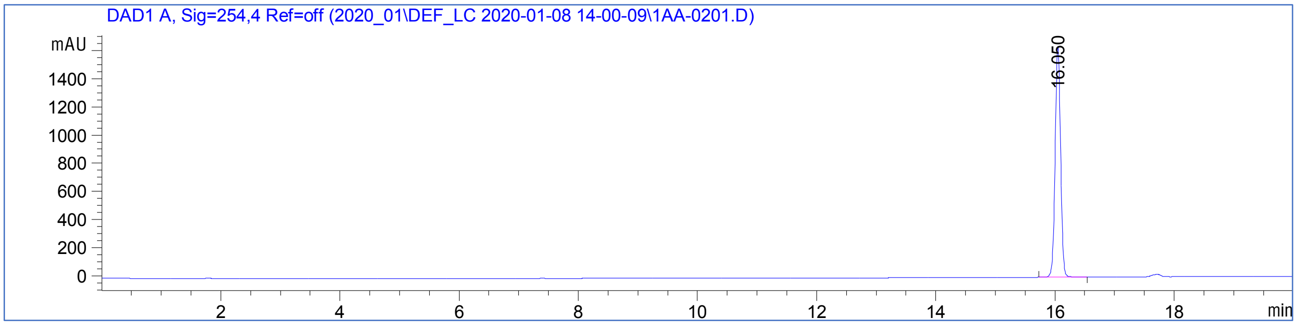
DAD1 A, Sig=254,4 Ref=off (2020_01\DEF_LC 2020-01-08 14-00-09\1AA-0201.D)
mAU
16.050
min

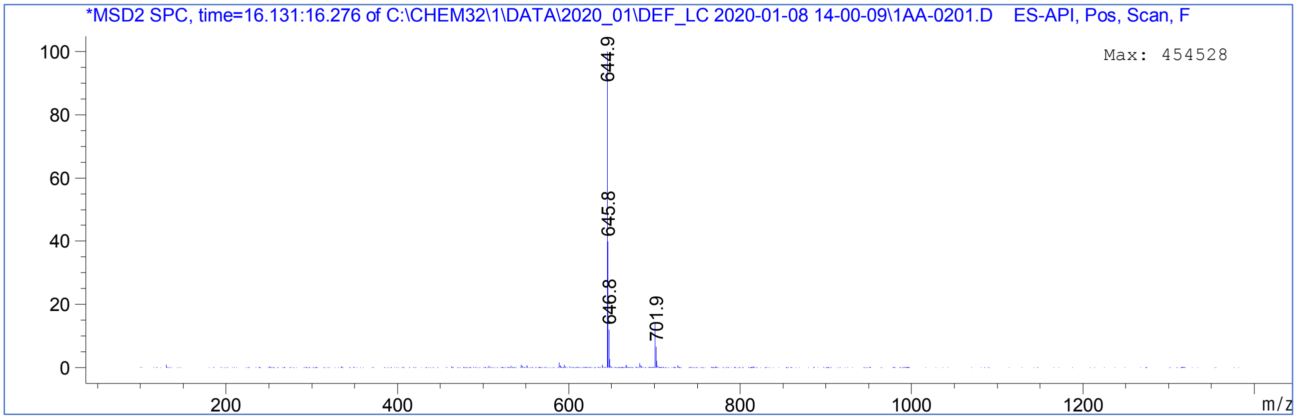
*MSD2 SPC, time=16.131:16.276 of C:\CHEM32\1\DATA\2020_01\DEF_LC 2020-01-08 14-00-09\1AA-0201.D ES-API, Pos, Scan, F
Max: 454528
644.9
645.8
646.8
701.9
m/z

Origin	Bruker BioSpin GmbH
Solvent	MeOD
Temperature	300.0
Pulse Sequence	zg30
Experiment	1D
Number of Scans	16
Acquisition Date	2019-11-18T14:14:00
Spectrometer Frequency	400.13
Spectral Width	8012.8
Lowest Frequency	-1543.2
Nucleus	1H
Acquired Size	32768
Spectral Size	65536

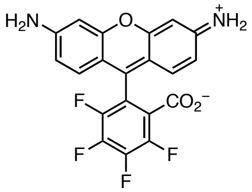

70

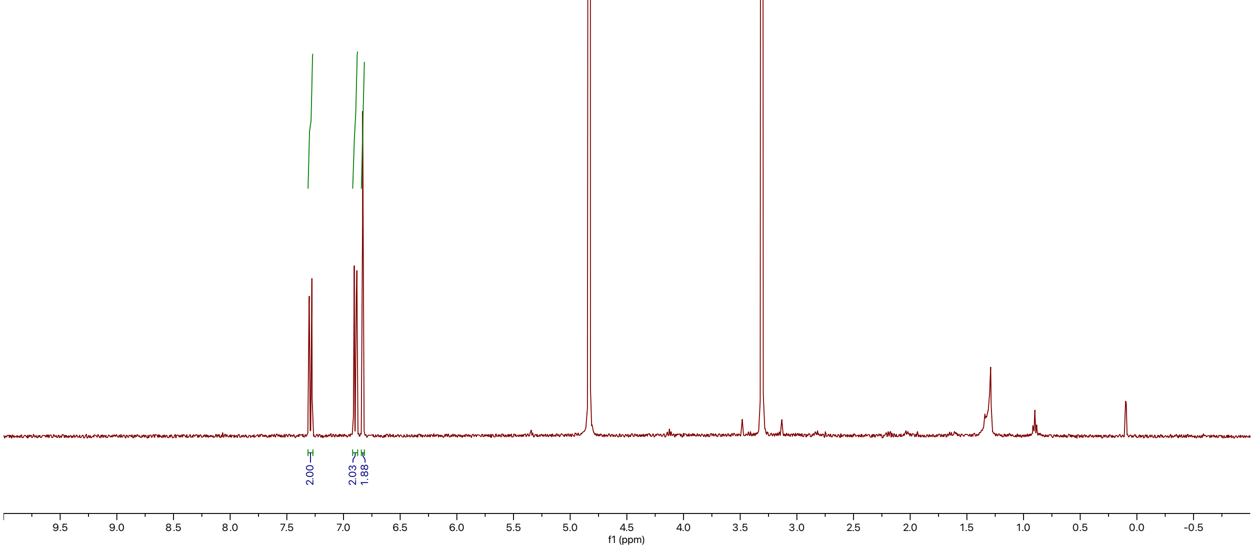

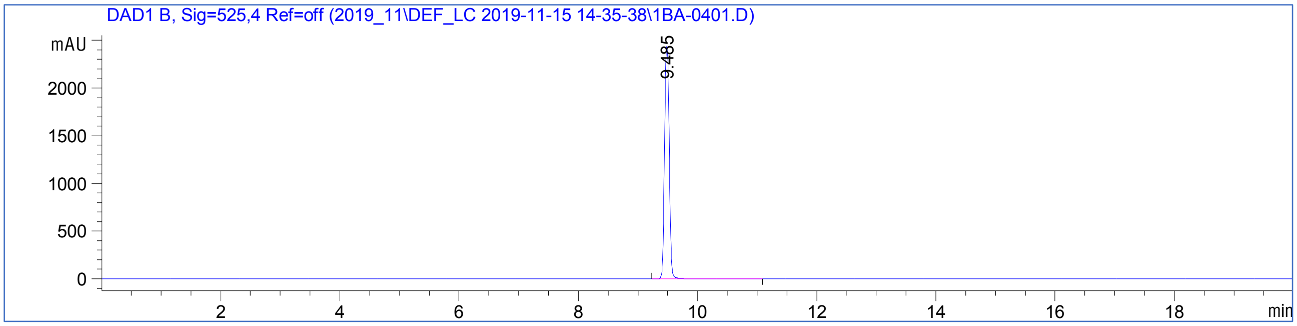

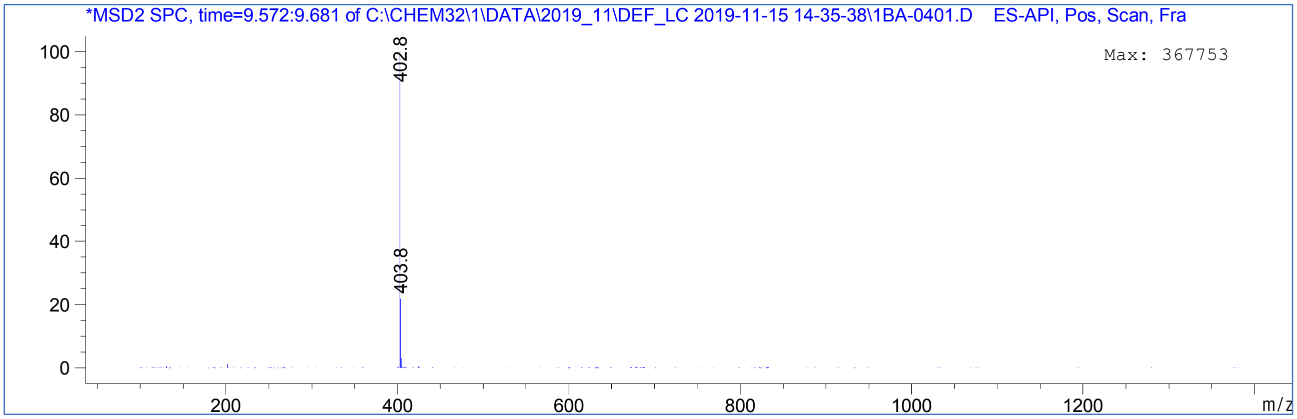

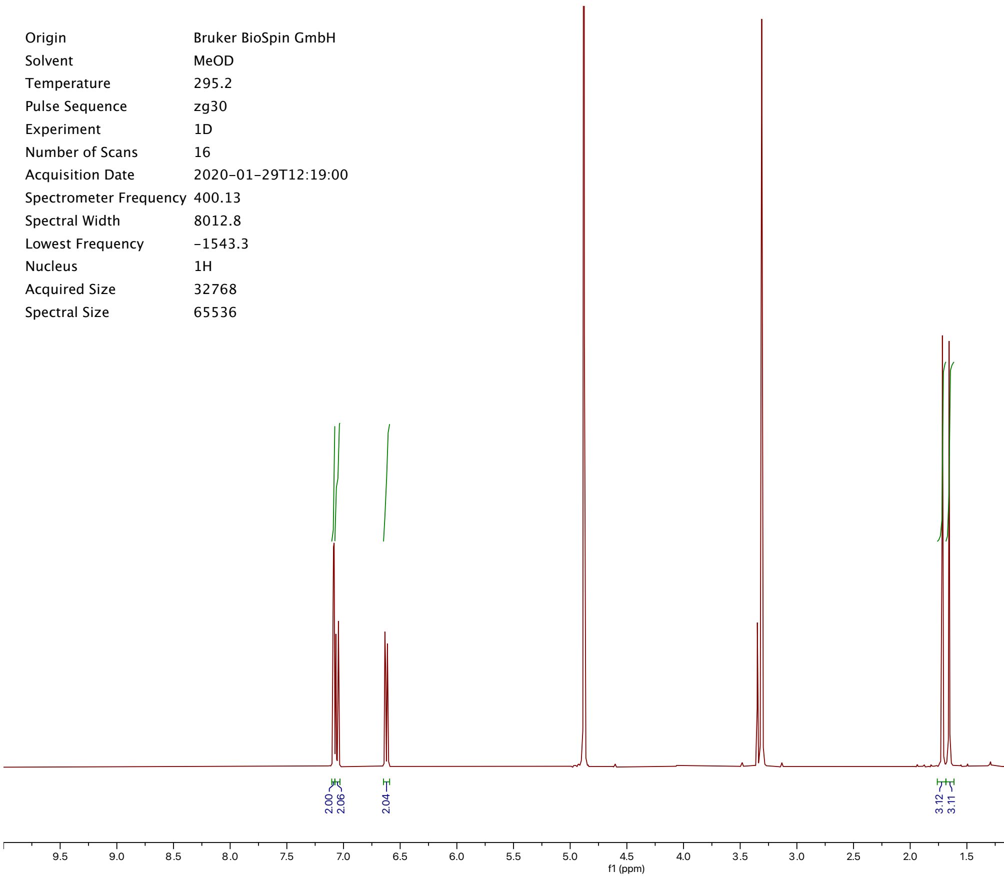
Origin Bruker BioSpin GmbH
Solvent MeOD
Temperature 295.2
Pulse Sequence zg30
Experiment 1D
Number of Scans 16
Acquisition Date 2020-01-29T12:19:00
Spectrometer Frequency 400.13
Spectral Width 8012.8
Lowest Frequency -1543.3
Nucleus 1H
Acquired Size 32768
Spectral Size 65536
2.00
2.06
2.04
3.12
3.11
f1 (ppm)

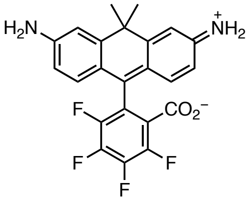

71

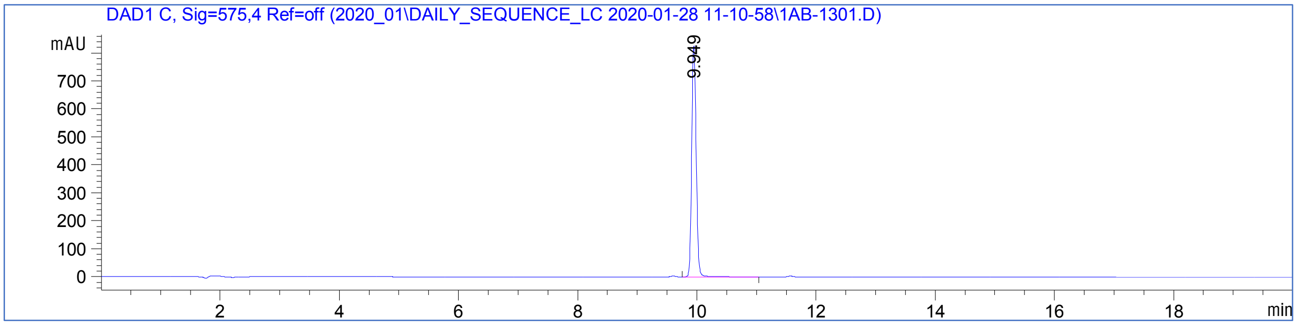
DAD1 C, Sig=575,4 Ref=off (2020_01\DAILY_SEQUENCE_LC 2020-01-28 11-10-58\1AB-1301.D)
mAU
9.949
min

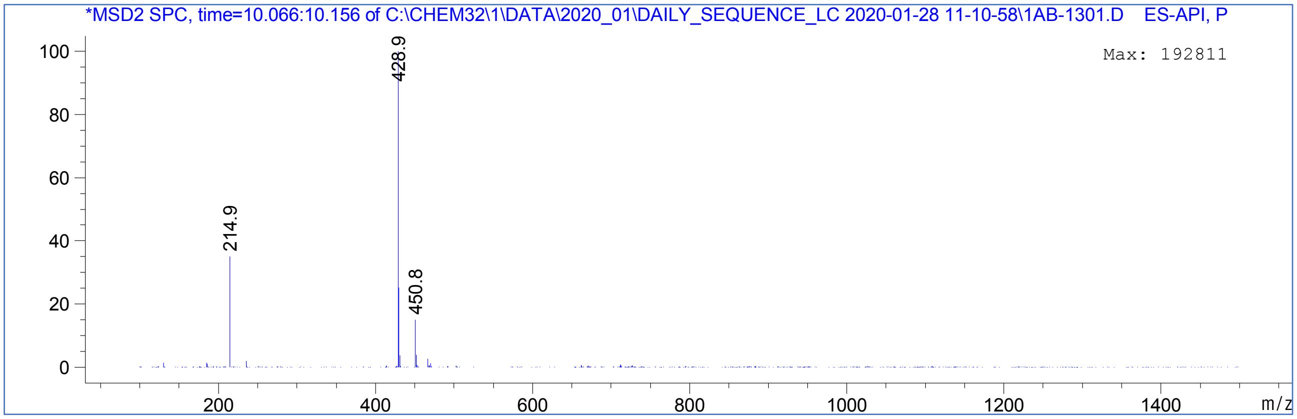
*MSD2 SPC, time=10.066:10.156 of C:\CHEM32\1\DATA\2020_01\DAILY_SEQUENCE_LC 2020-01-28 11-10-58\1AB-1301.D ES-API, P
Max: 192811
428.9
214.9
450.8
m/z

Origin	Bruker BioSpin GmbH
Solvent	CDCl3
Temperature	295.5
Pulse Sequence	zg30
Experiment	1D
Number of Scans	16
Acquisition Date	2020-02-19T16:30:00
Spectrometer Frequency	400.13
Spectral Width	8012.8
Lowest Frequency	-1544.8
Nucleus	1H
Acquired Size	32768
Spectral Size	65536

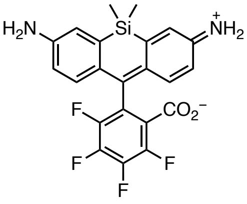

72

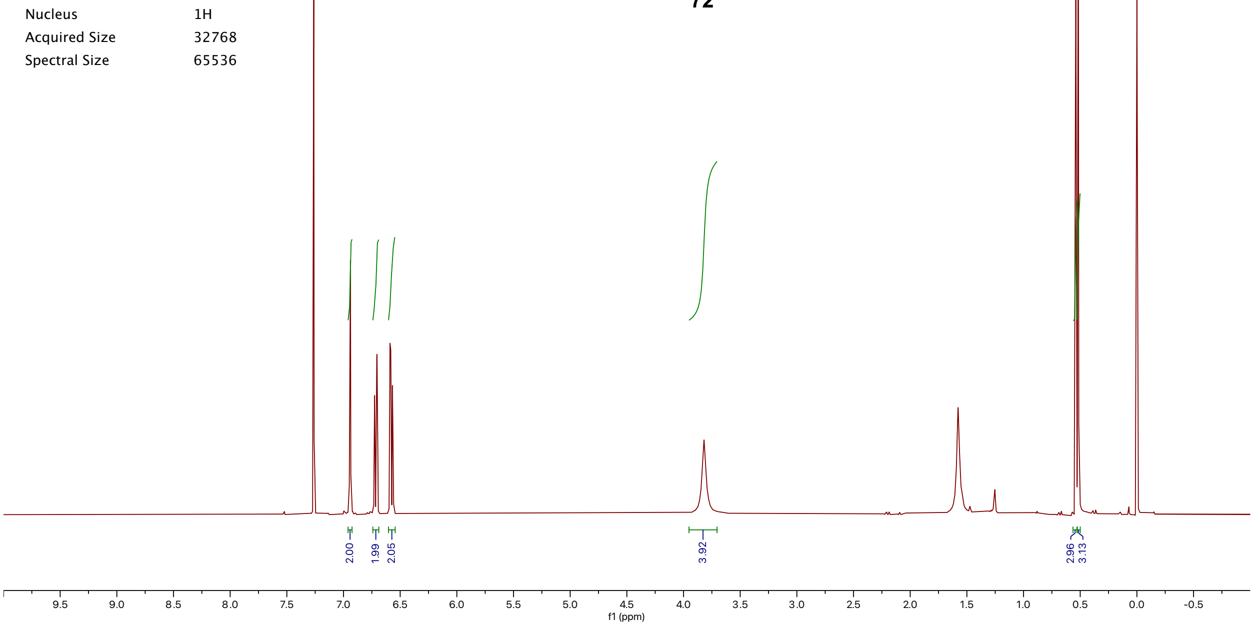

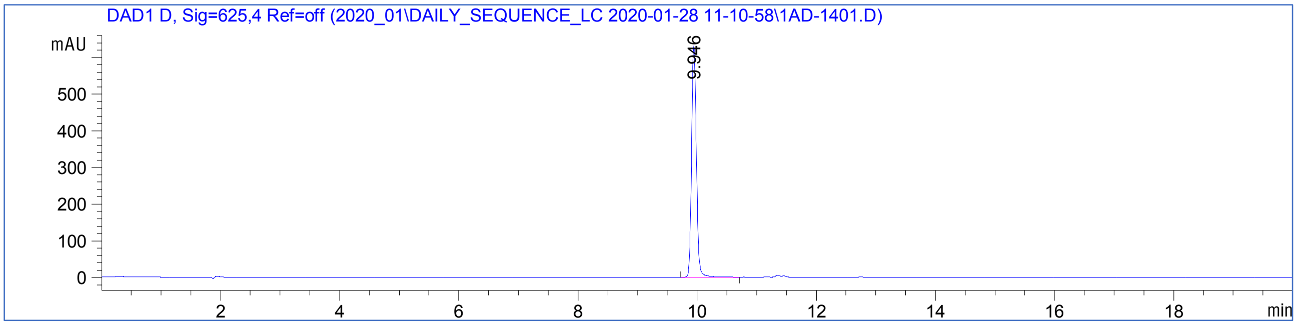

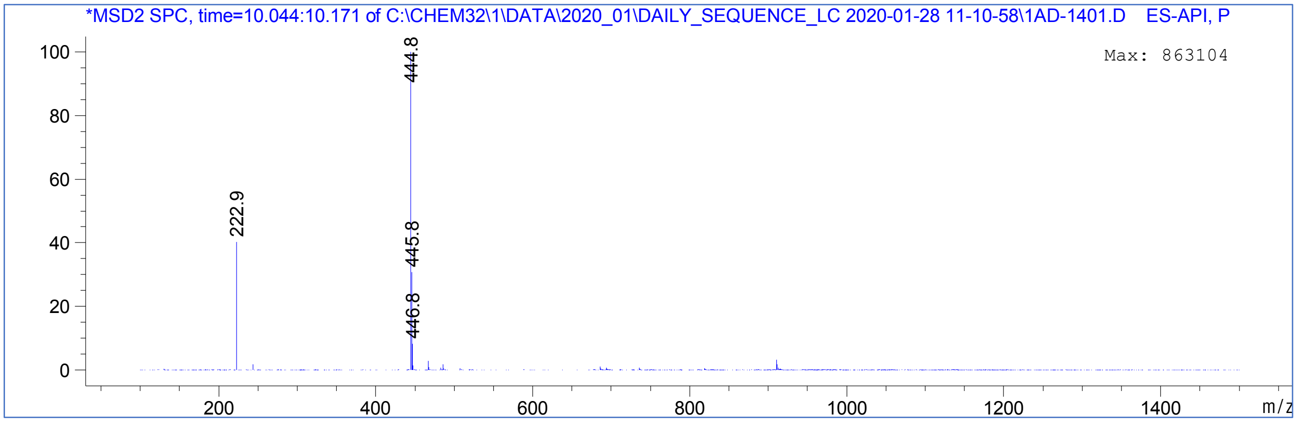

Origin	Bruker BioSpin GmbH
Solvent	DMSO
Temperature	300.0
Pulse Sequence	zg30
Experiment	1D
Number of Scans	16
Acquisition Date	2019-06-11T16:19:00
Spectrometer Frequency	400.13
Spectral Width	8012.8
Lowest Frequency	-1539.0
Nucleus	1H
Acquired Size	32768
Spectral Size	65536

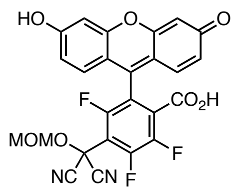

60_{MAC}

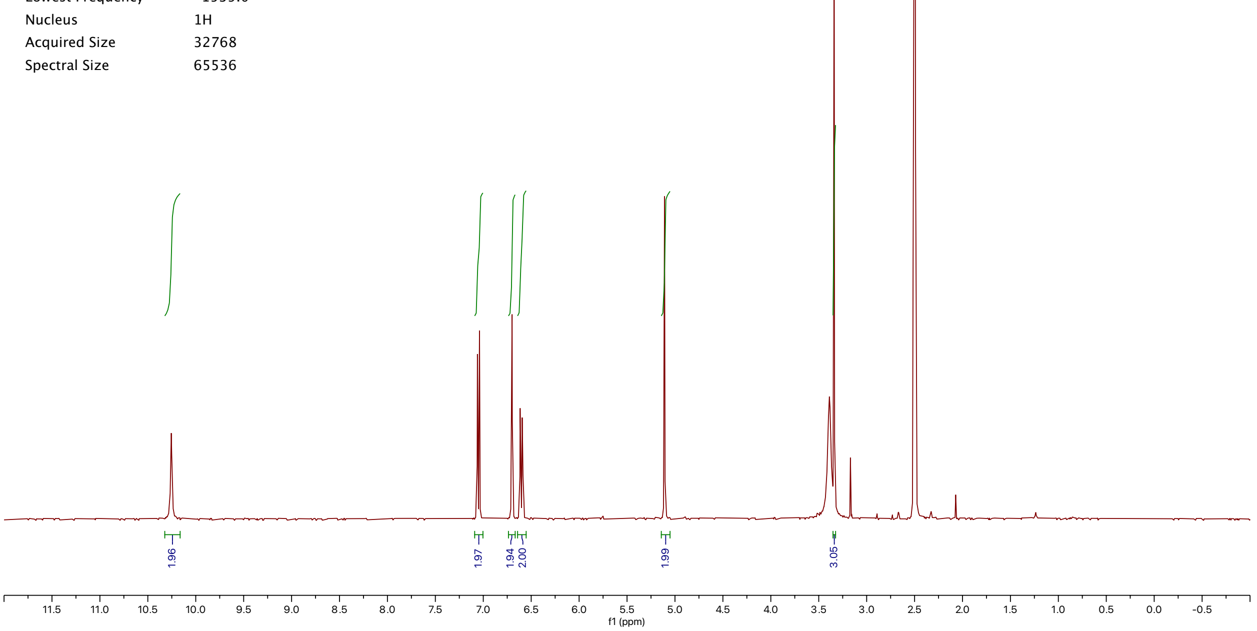

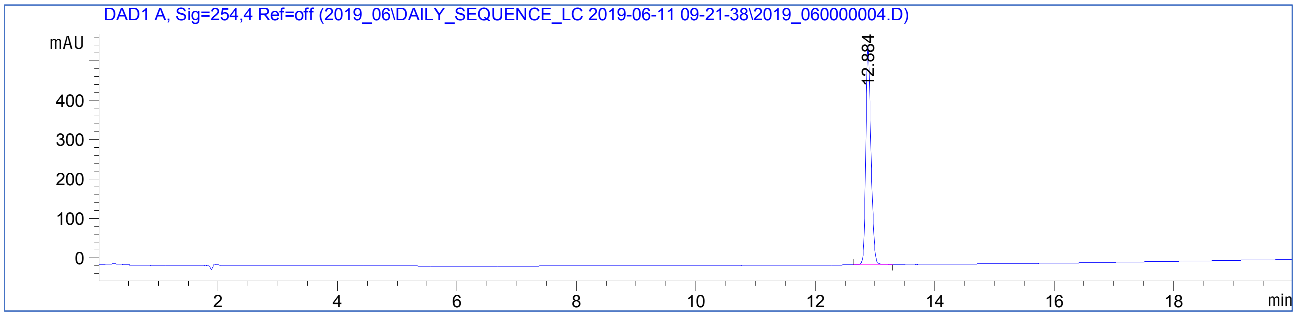

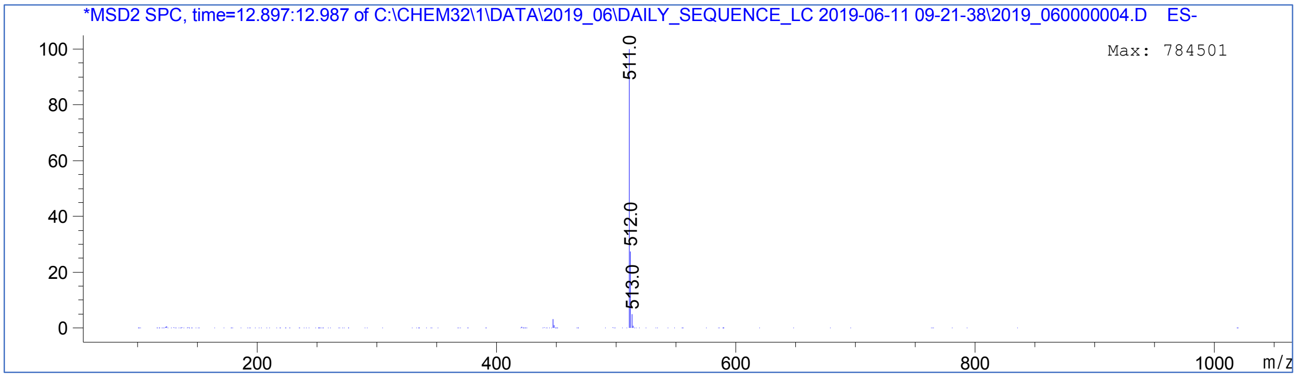

Origin	Bruker BioSpin GmbH
Solvent	DMSO
Temperature	300.0
Pulse Sequence	zg30
Experiment	1D
Number of Scans	16
Acquisition Date	2019-11-25T09:28:00
Spectrometer Frequency	400.13
Spectral Width	8012.8
Lowest Frequency	-1539.0
Nucleus	1H
Acquired Size	32768
Spectral Size	65536

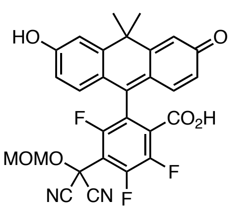

51_{MAC}

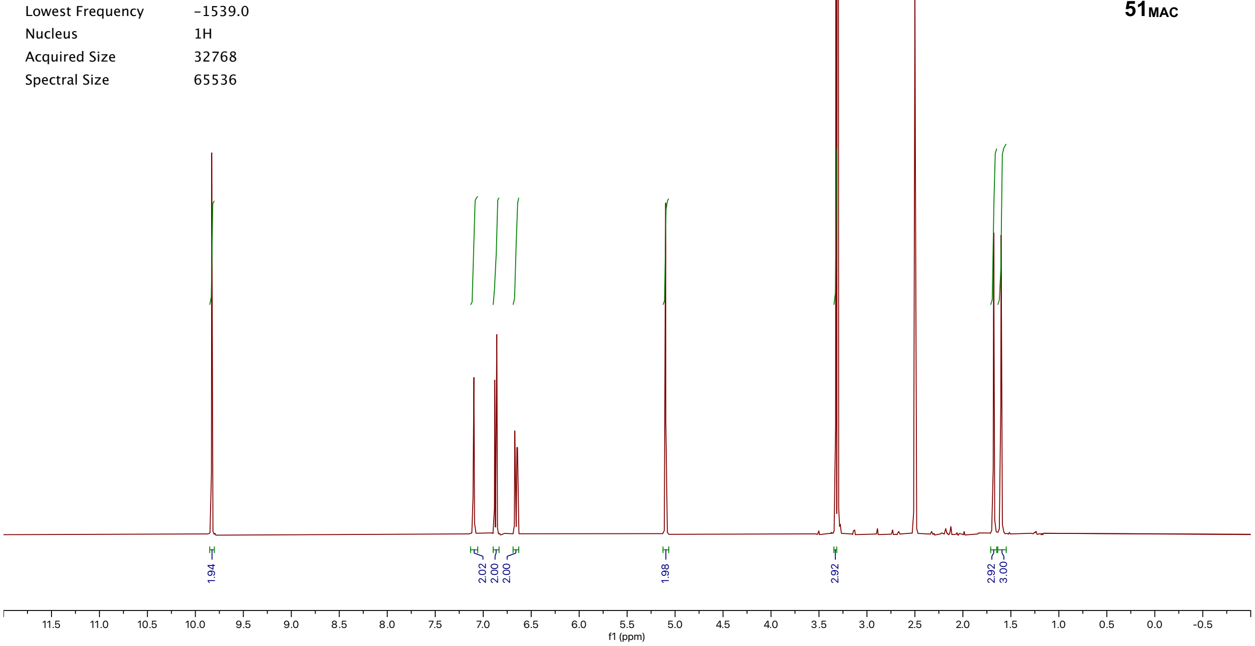

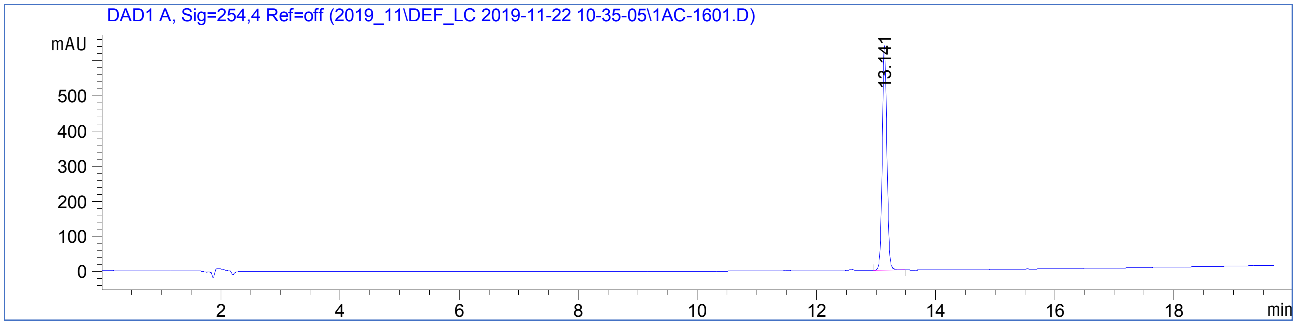

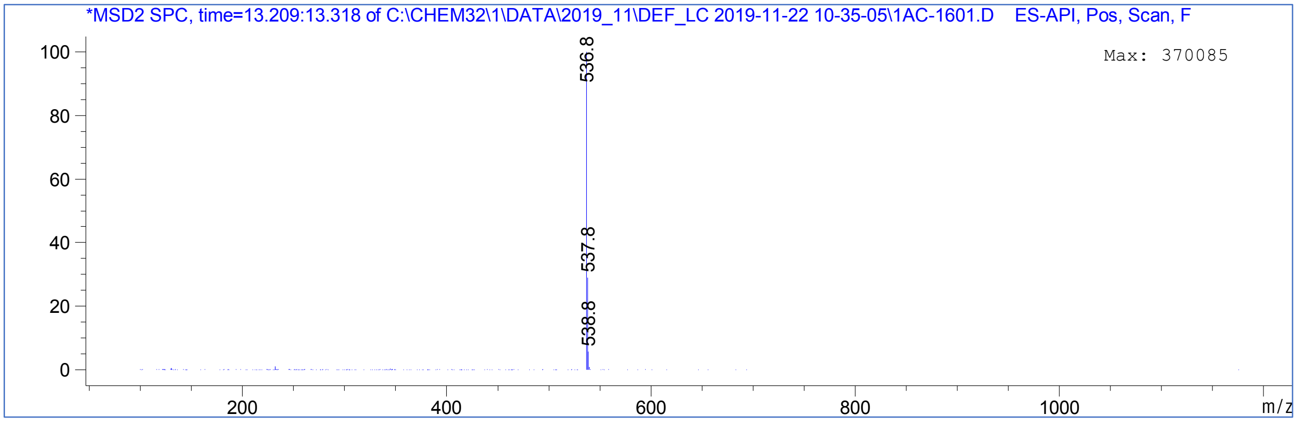

Origin	Bruker BioSpin GmbH
Solvent	CDCl3
Temperature	300.0
Pulse Sequence	zg30
Experiment	1D
Number of Scans	16
Acquisition Date	2019-06-04T11:06:00
Spectrometer Frequency	400.13
Spectral Width	8012.8
Lowest Frequency	-1546.1
Nucleus	1H
Acquired Size	32768
Spectral Size	65536

55MAC

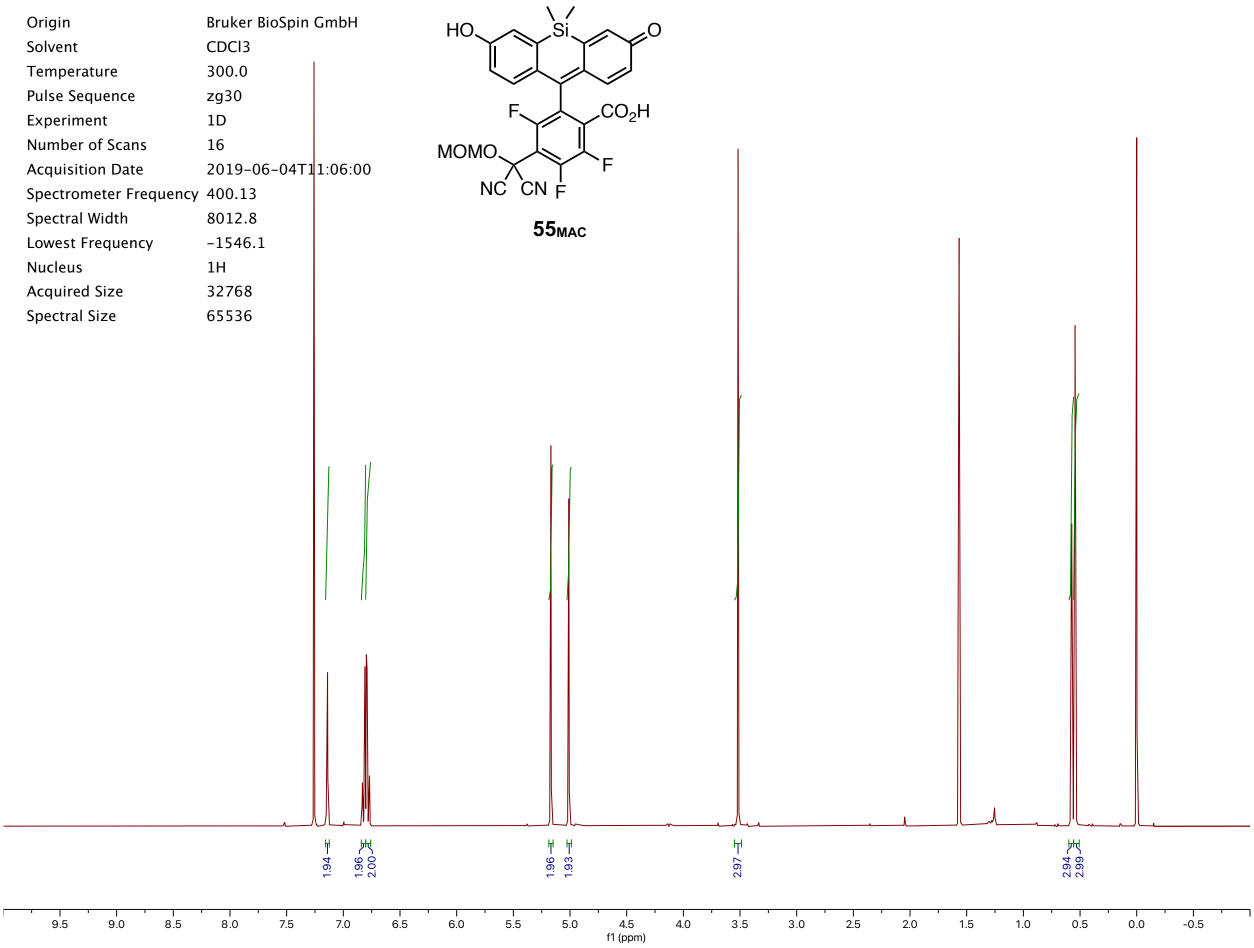

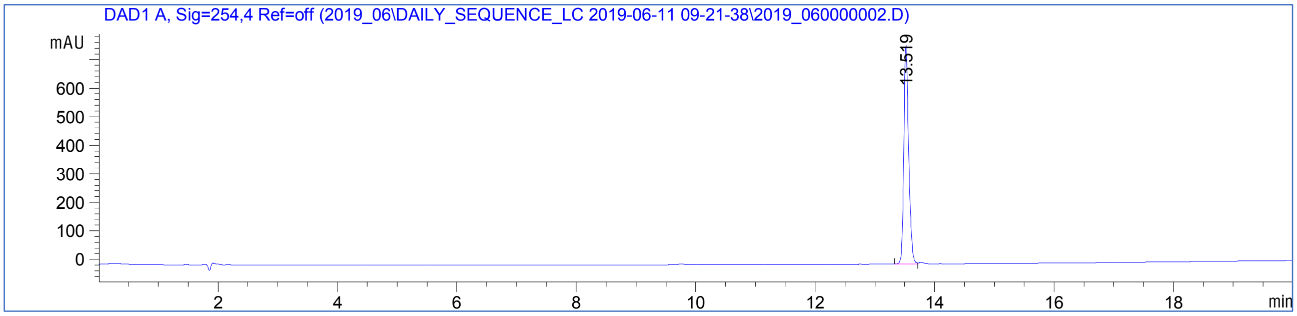

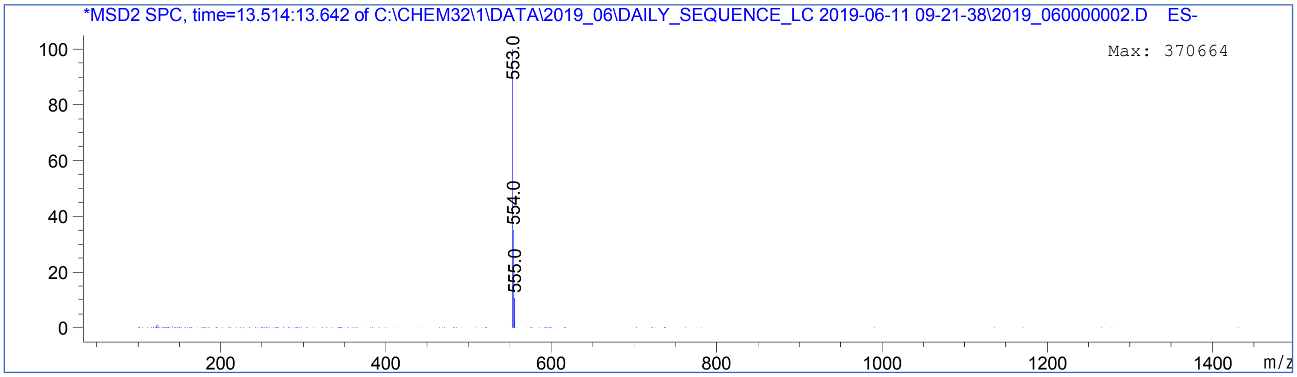

Origin	Bruker BioSpin GmbH
Solvent	CDCl3
Temperature	300.0
Pulse Sequence	zg30
Experiment	1D
Number of Scans	16
Acquisition Date	2019-07-24T10:43:00
Spectrometer Frequency	400.13
Spectral Width	8012.8
Lowest Frequency	-1546.3
Nucleus	1H
Acquired Size	32768
Spectral Size	65536

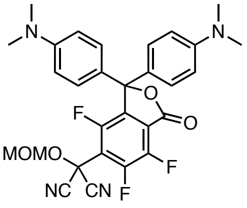

49_{MAC}

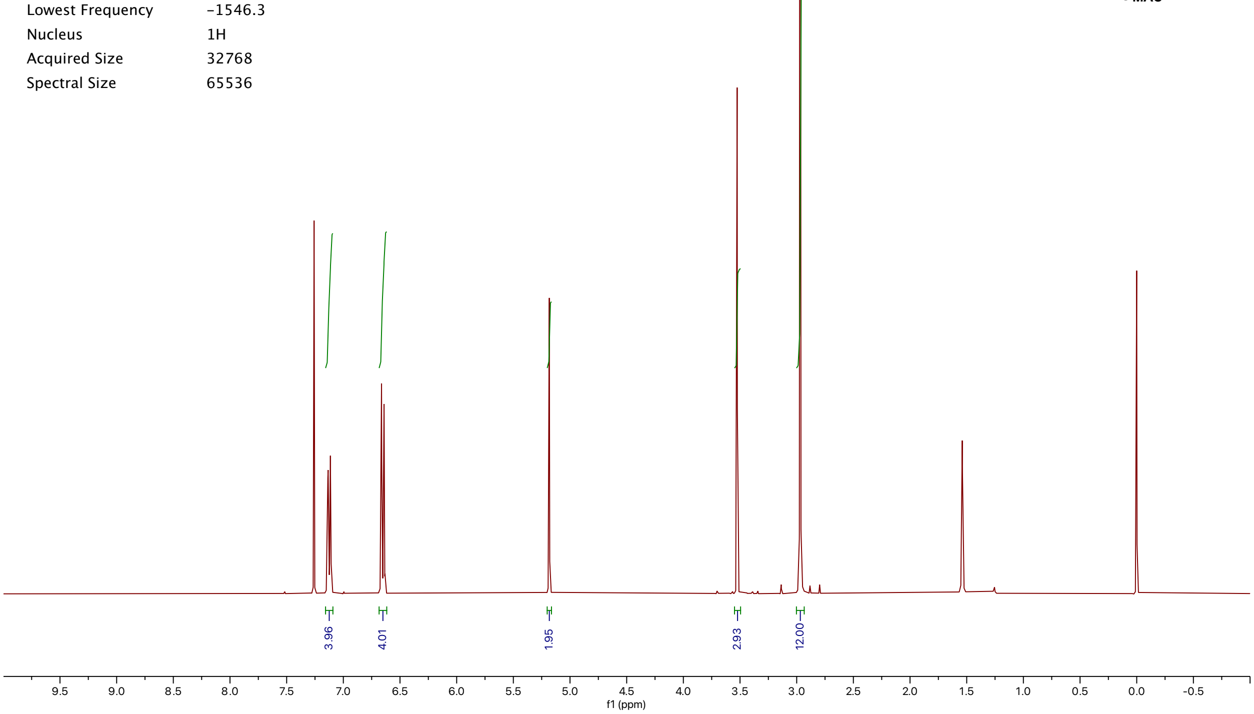

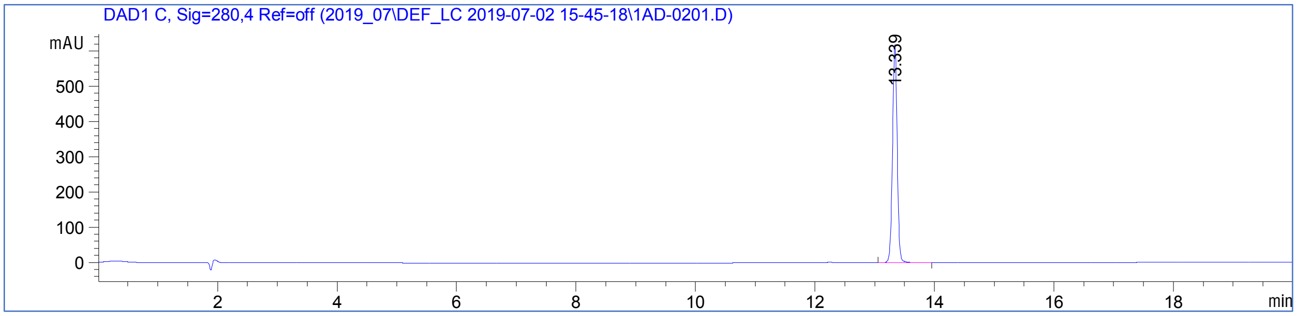

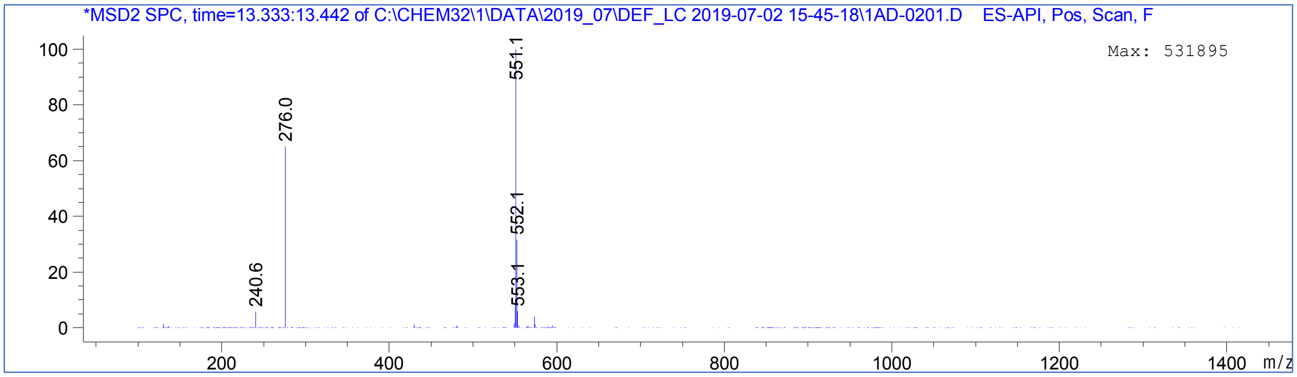

Origin	Bruker BioSpin GmbH
Solvent	CDCl3
Temperature	295.3
Pulse Sequence	zg30
Experiment	1D
Number of Scans	16
Acquisition Date	2020-01-14T11:48:00
Spectrometer Frequency	400.13
Spectral Width	8012.8
Lowest Frequency	-1545.1
Nucleus	1H
Acquired Size	32768
Spectral Size	65536

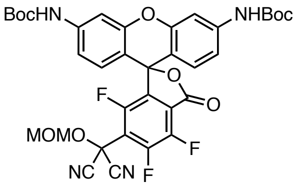

67$_{\mathbf{MAC}}$

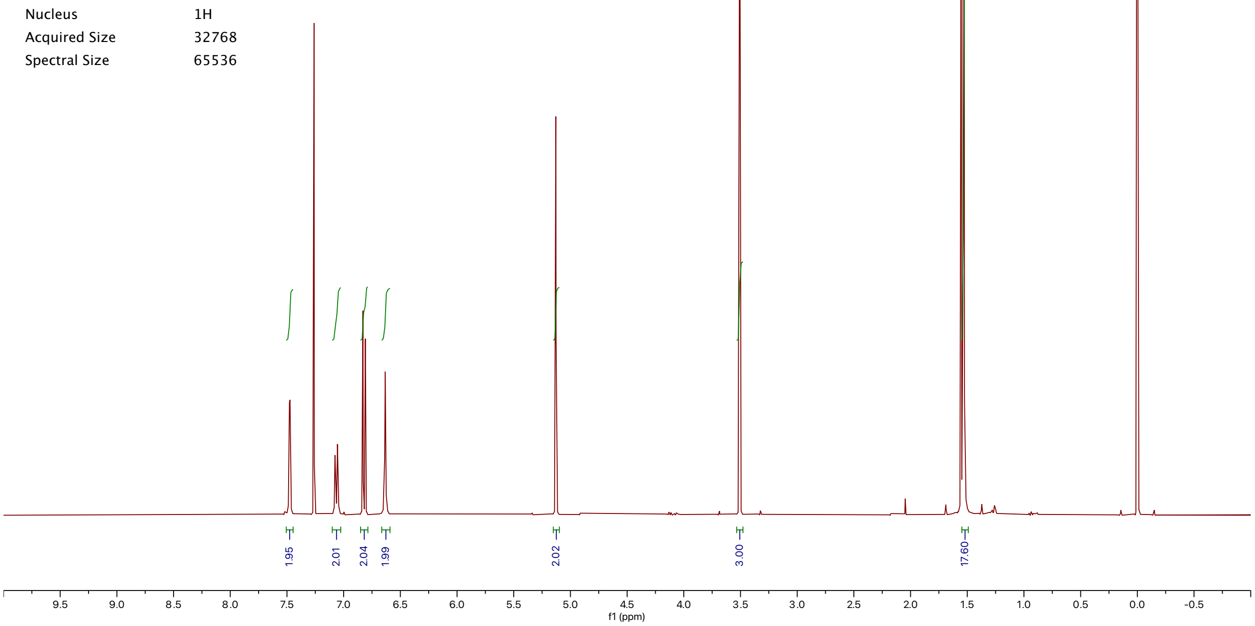

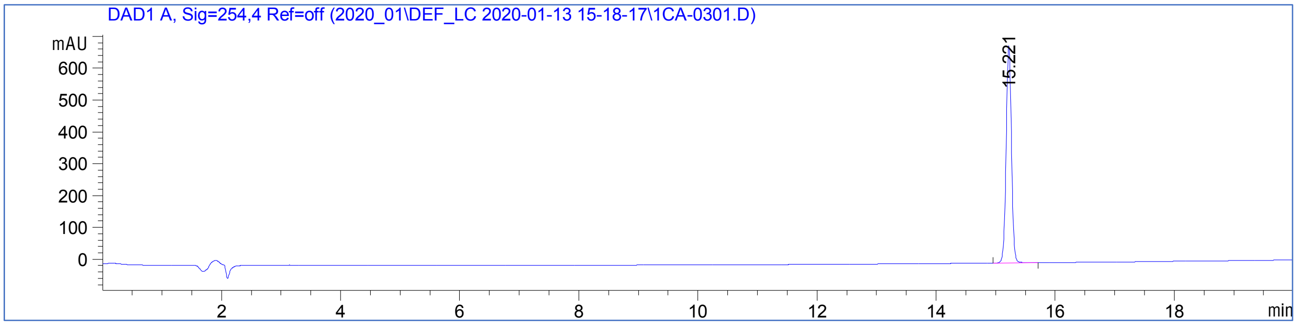

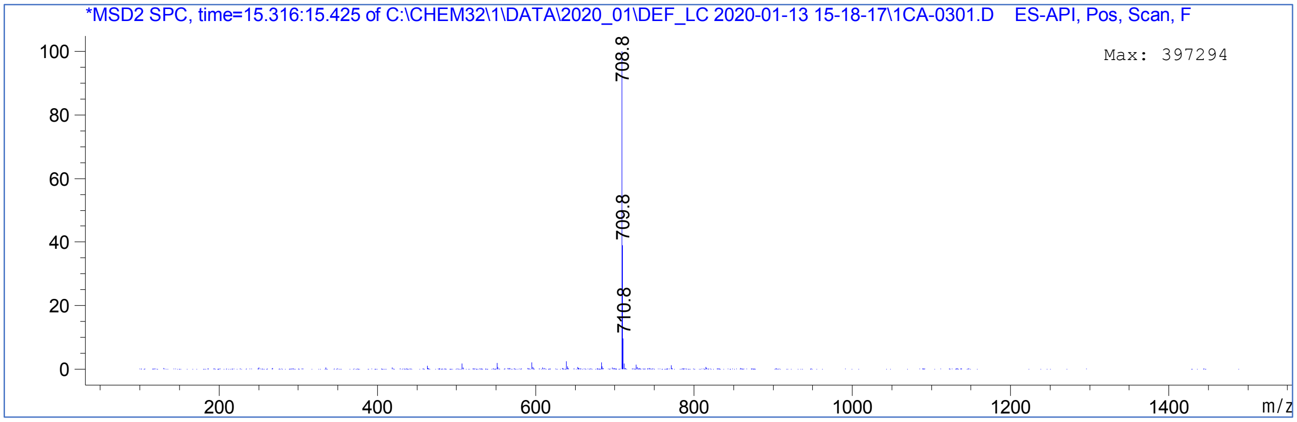

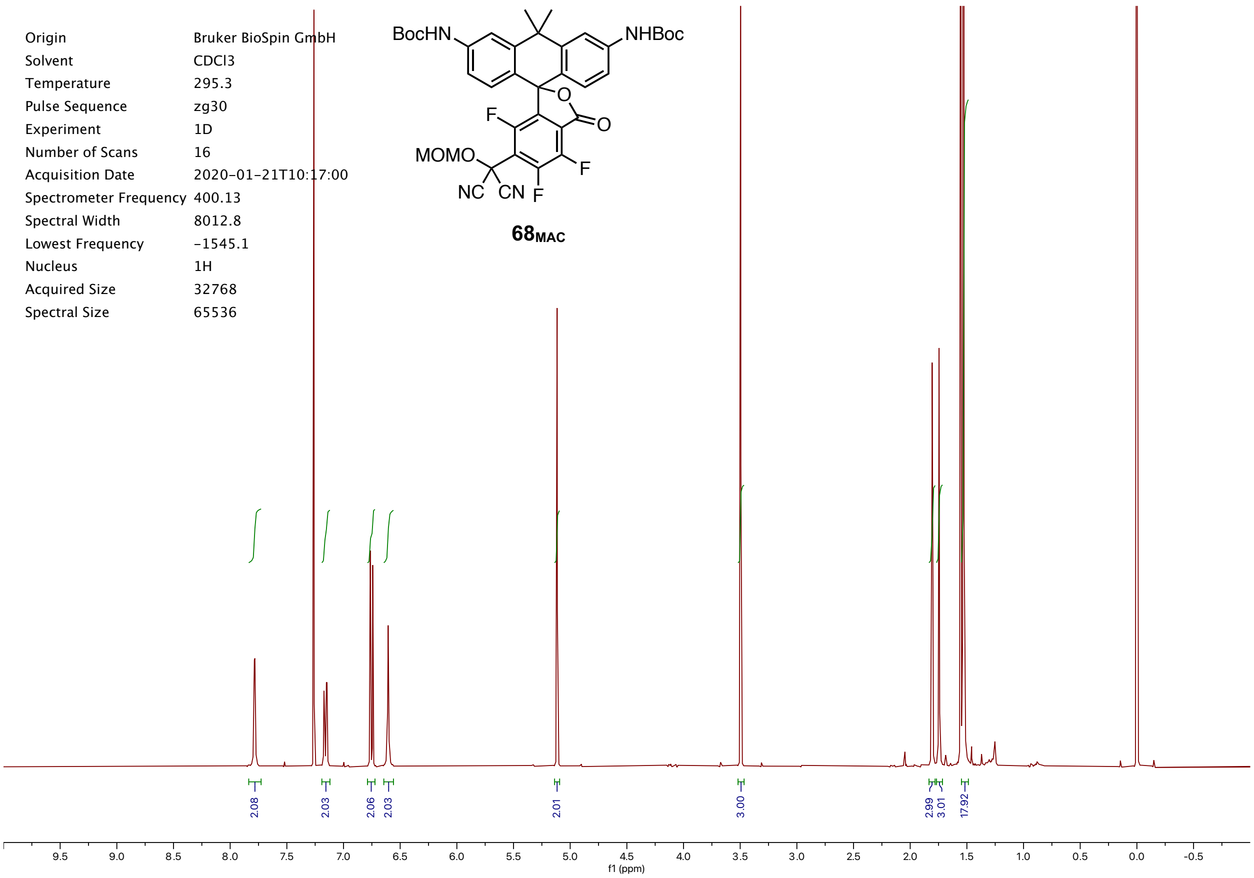
Origin Bruker BioSpin GmbH
Solvent CDCl3
Temperature 295.3
Pulse Sequence zg30
Experiment 1D
Number of Scans 16
Acquisition Date 2020-01-21T10:17:00
Spectrometer Frequency 400.13
Spectral Width 8012.8
Lowest Frequency -1545.1
Nucleus 1H
Acquired Size 32768
Spectral Size 65536
BocHN
NHBoc
O
O
F
F
F
MOMO
NC
CN
68MAC
2.08
2.03
2.06
2.03
2.01
3.00
2.99
3.01
17.92
f1 (ppm)

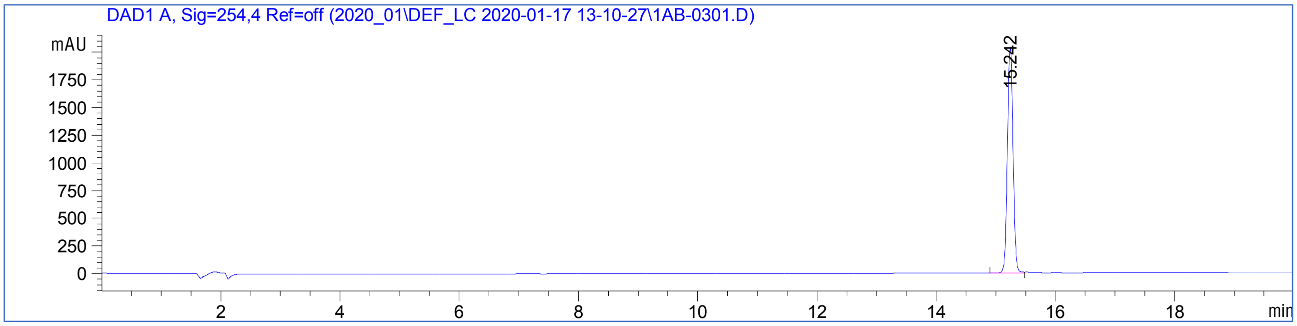
DAD1 A, Sig=254,4 Ref=off (2020_01\DEF_LC 2020-01-17 13-10-27\1AB-0301.D)
mAU
15.242
min

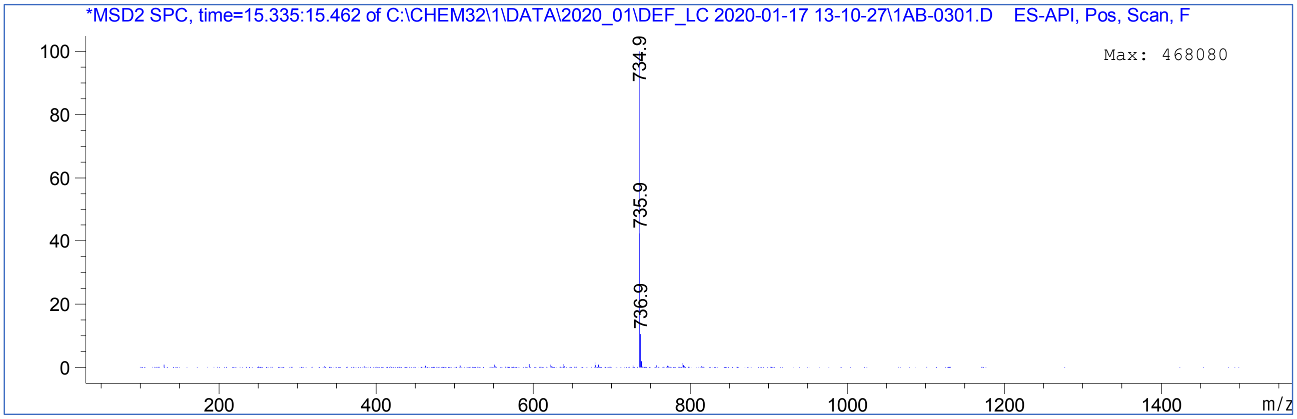
*MSD2 SPC, time=15.335:15.462 of C:\CHEM32\1\DATA\2020_01\DEF_LC 2020-01-17 13-10-27\1AB-0301.D ES-API, Pos, Scan, F
Max: 468080
734.9
735.9
736.9
m/z

Origin	Bruker BioSpin GmbH
Solvent	CDCl3
Temperature	295.2
Pulse Sequence	zg30
Experiment	1D
Number of Scans	16
Acquisition Date	2020-01-21T10:26:00
Spectrometer Frequency	400.13
Spectral Width	8012.8
Lowest Frequency	-1545.1
Nucleus	1H
Acquired Size	32768
Spectral Size	65536

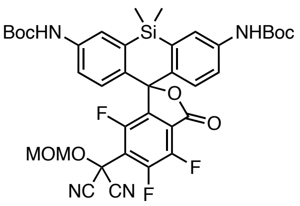

69_{MAC}

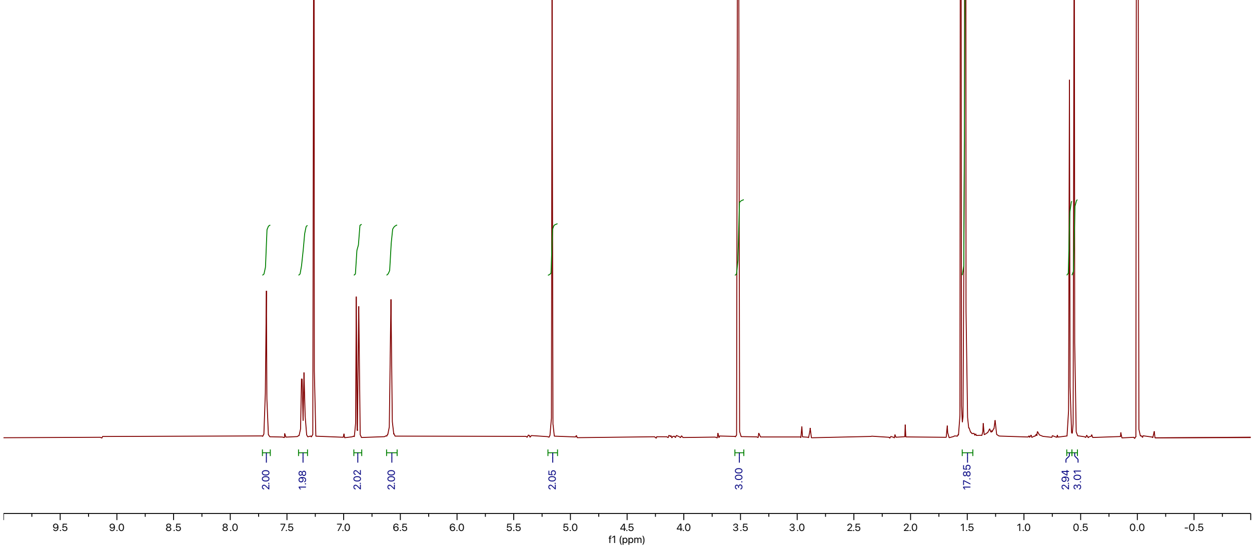

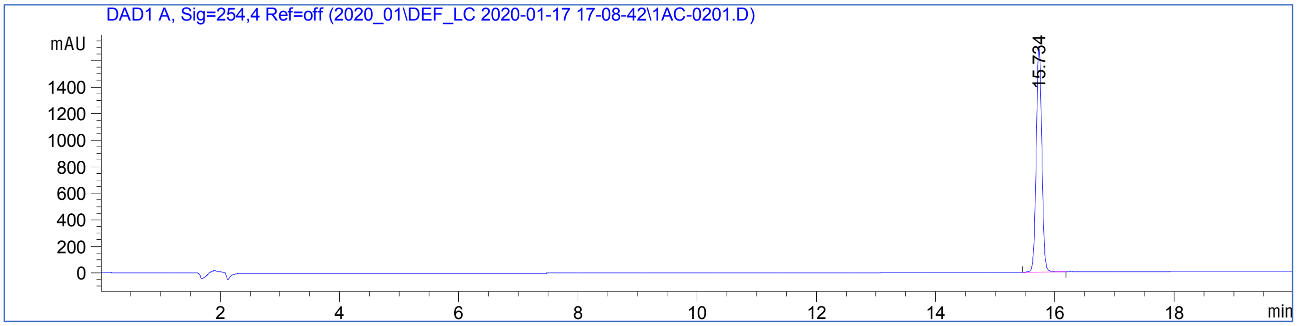

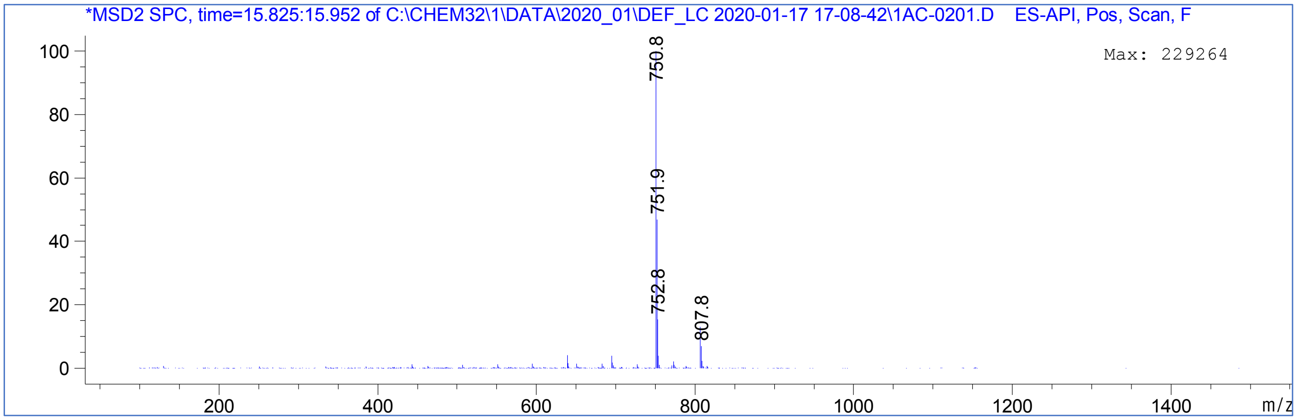

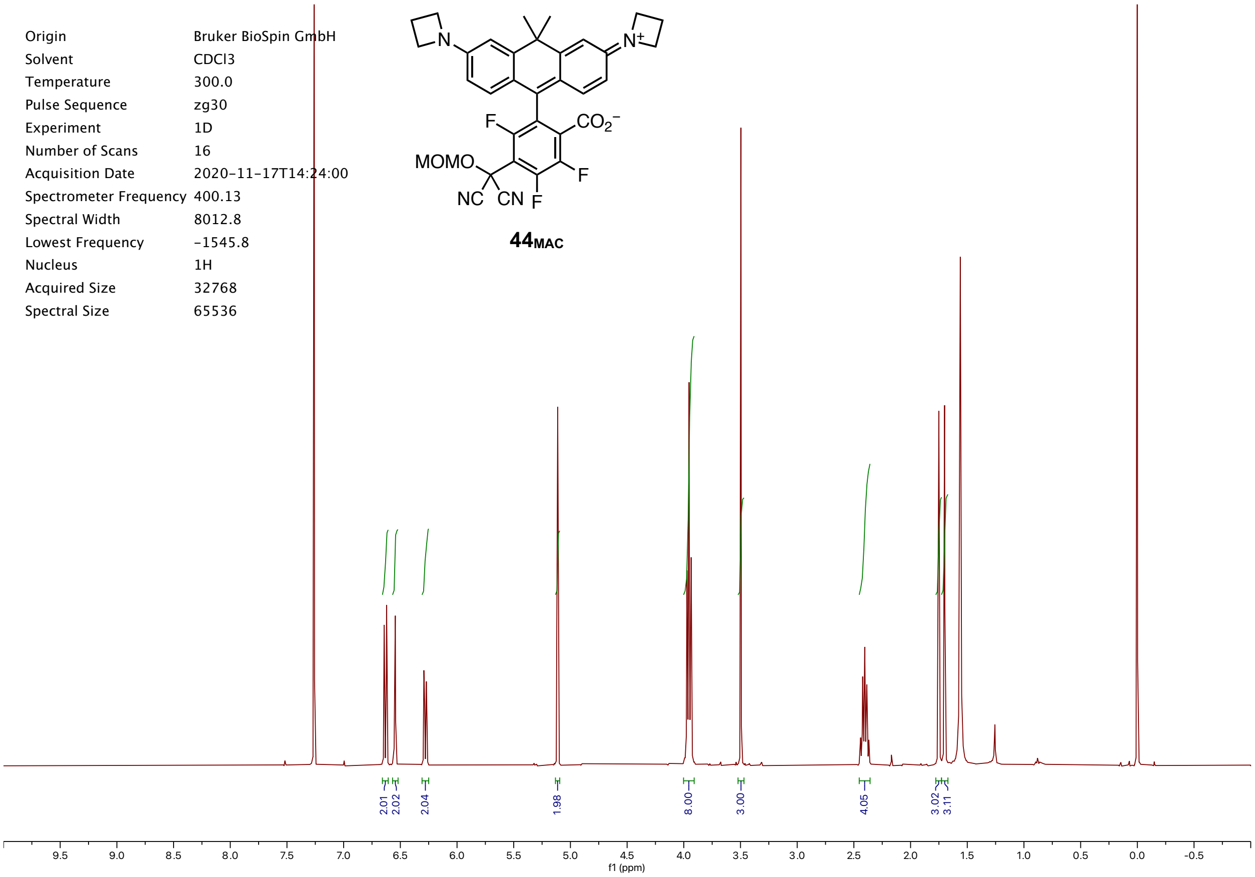
Origin Bruker BioSpin GmbH
Solvent CDCl3
Temperature 300.0
Pulse Sequence zg30
Experiment 1D
Number of Scans 16
Acquisition Date 2020-11-17T14:24:00
Spectrometer Frequency 400.13
Spectral Width 8012.8
Lowest Frequency -1545.8
Nucleus 1H
Acquired Size 32768
Spectral Size 65536
MOMO
NC CN
F
CO2−
N+
N
44MAC
2.01
2.02
2.04
1.98
8.00
3.00
4.05
3.02
3.11
f1 (ppm)

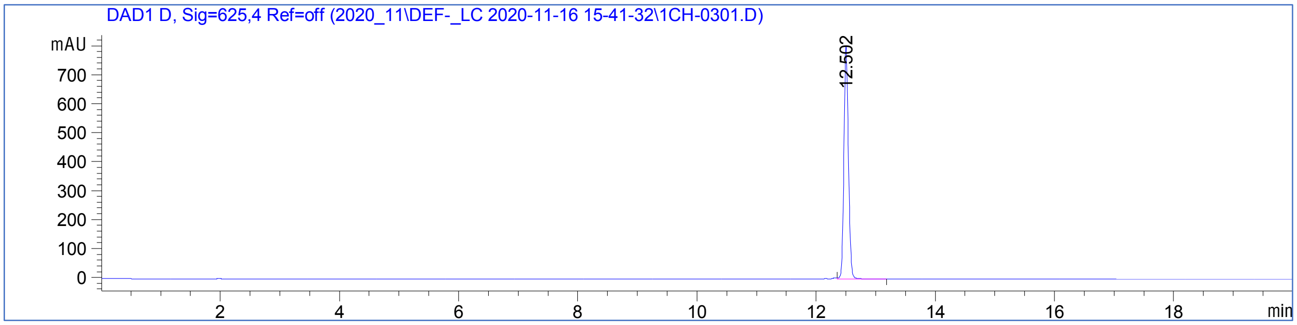
DAD1 D, Sig=625,4 Ref=off (2020_11\DEF-_LC 2020-11-16 15-41-32\1CH-0301.D)
mAU
12.502
min

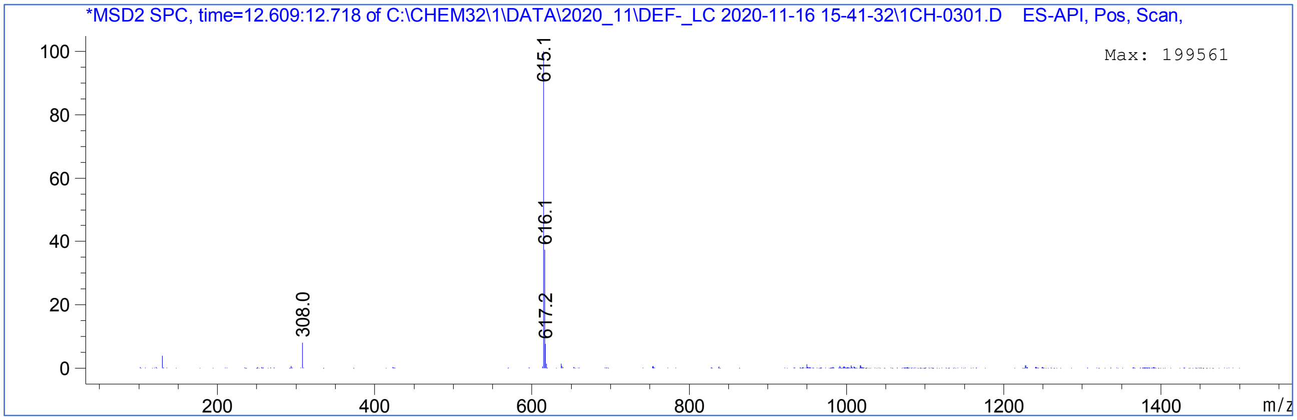
*MSD2 SPC, time=12.609:12.718 of C:\CHEM32\1\DATA\2020_11\DEF-_LC 2020-11-16 15-41-32\1CH-0301.D ES-API, Pos, Scan,
Max: 199561
615.1
616.1
617.2
308.0
m/z

Origin	Bruker BioSpin GmbH
Solvent	MeOD
Temperature	300.0
Pulse Sequence	zg30
Experiment	1D
Number of Scans	16
Acquisition Date	2022-01-26T16:48:00
Spectrometer Frequency	400.13
Spectral Width	8012.8
Lowest Frequency	-1543.1
Nucleus	1H
Acquired Size	32768
Spectral Size	65536

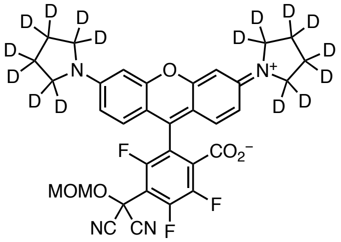

8_{MAC}

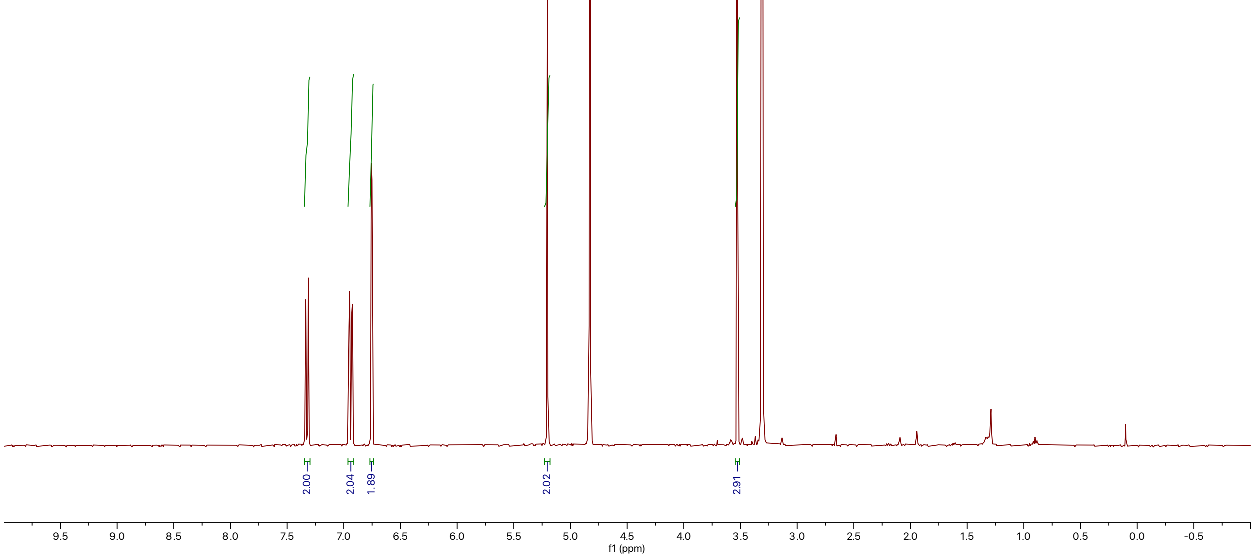

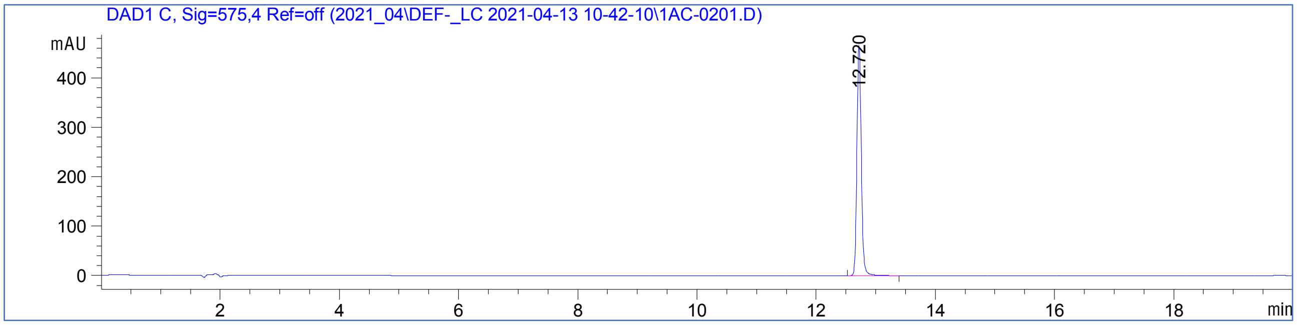

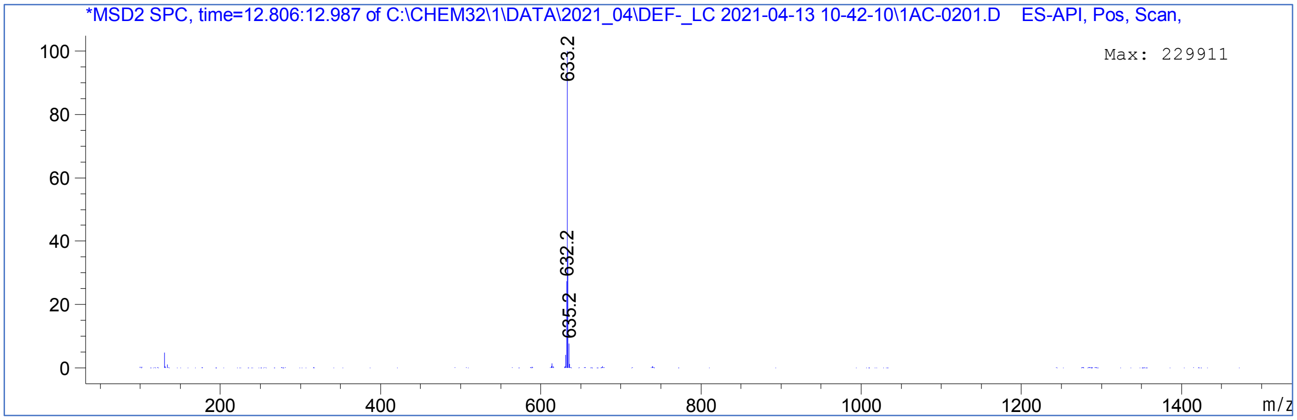

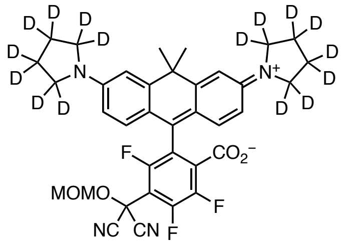

D
N
N+
F
CO2−
MOMO
F
NC
CN
F

40MAC

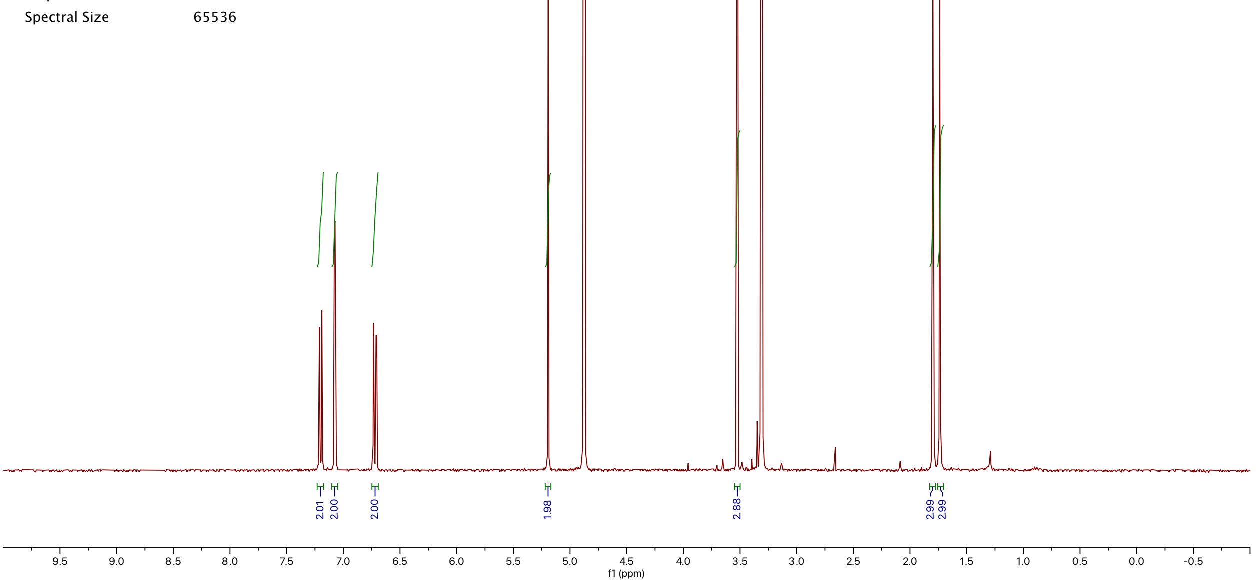

Origin Bruker BioSpin GmbH
Solvent MeOD
Temperature 295.5
Pulse Sequence zg30
Experiment 1D
Number of Scans 16
Acquisition Date 2021-08-30T11:50:00
Spectrometer Frequency 400.13
Spectral Width 8012.8
Lowest Frequency -1543.3
Nucleus 1H
Acquired Size 32768
Spectral Size 65536
2.01
2.00
2.00
1.98
2.88
2.99
2.99
9.5
9.0
8.5
8.0
7.5
7.0
6.5
6.0
5.5
5.0
4.5
4.0
3.5
3.0
2.5
2.0
1.5
1.0
0.5
0.0
-0.5
f1 (ppm)

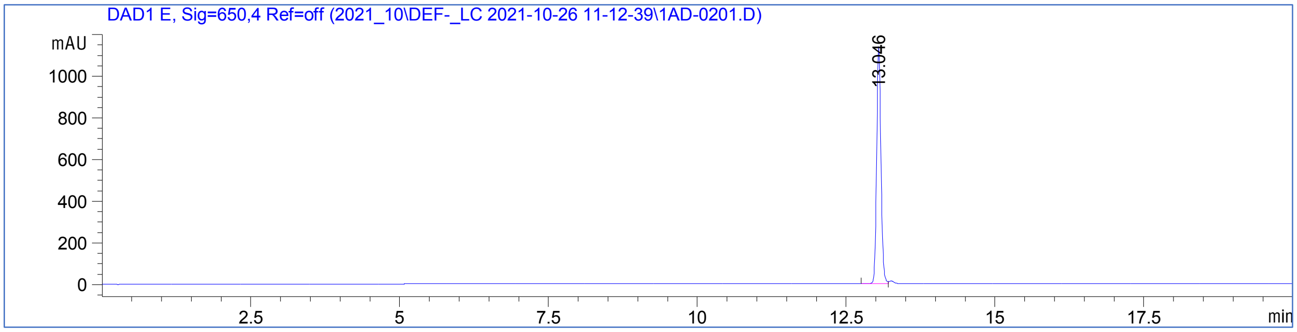

DAD1 E, Sig=650,4 Ref=off (2021_10\DEF-_LC 2021-10-26 11-12-39\1AD-0201.D)
mAU
1000
800
600
400
200
0
13.046
2.5
5
7.5
10
12.5
15
17.5
min

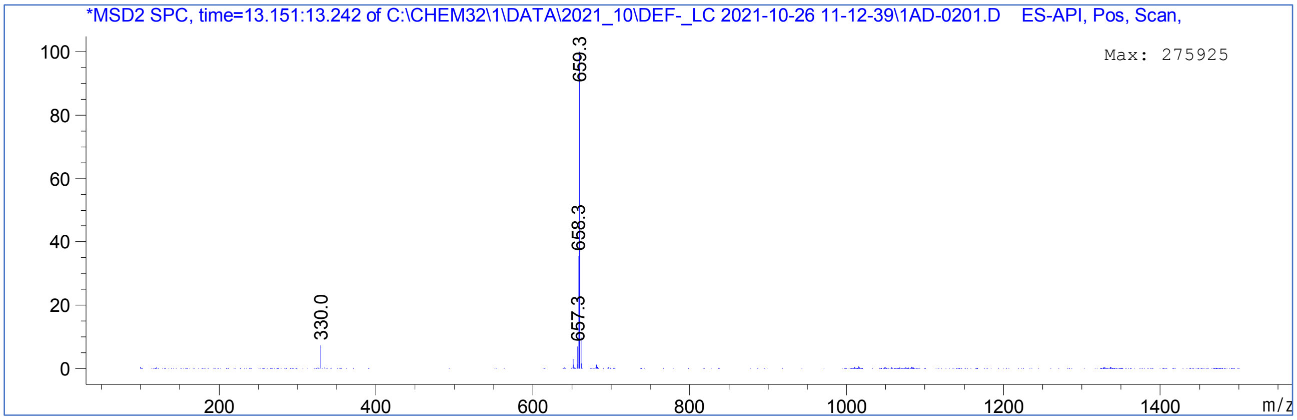

*MSD2 SPC, time=13.151:13.242 of C:\CHEM32\1\DATA\2021_10\DEF-_LC 2021-10-26 11-12-39\1AD-0201.D ES-API, Pos, Scan,
Max: 275925
100
80
60
40
20
0
659.3
658.3
657.3
330.0
200
400
600
800
1000
1200
1400
m/z

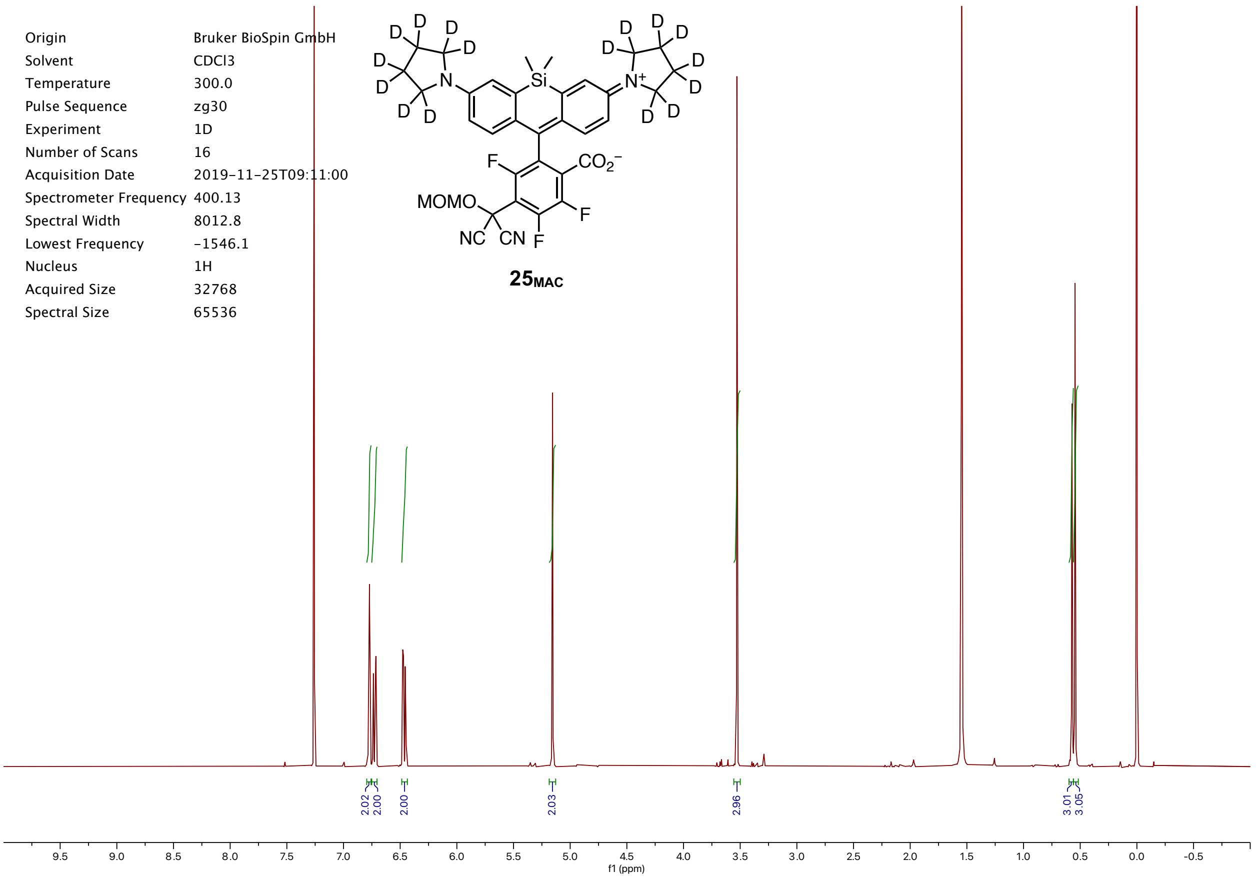
Origin Bruker BioSpin GmbH
Solvent CDCl3
Temperature 300.0
Pulse Sequence zg30
Experiment 1D
Number of Scans 16
Acquisition Date 2019-11-25T09:11:00
Spectrometer Frequency 400.13
Spectral Width 8012.8
Lowest Frequency -1546.1
Nucleus 1H
Acquired Size 32768
Spectral Size 65536
MOMO
NC CN
25MAC
2.02 2.00 2.00 2.03 2.96 3.01 3.05
f1 (ppm)

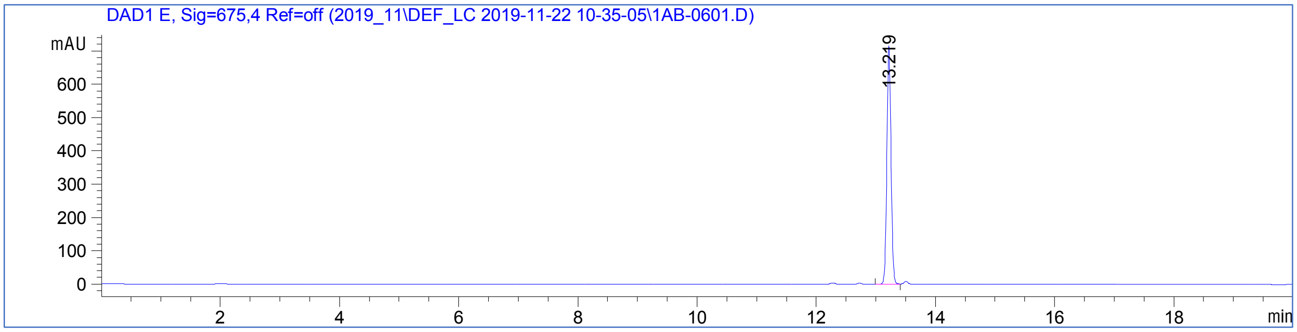
DAD1 E, Sig=675,4 Ref=off (2019_11\DEF_LC 2019-11-22 10-35-05\1AB-0601.D)
mAU
13.219
min

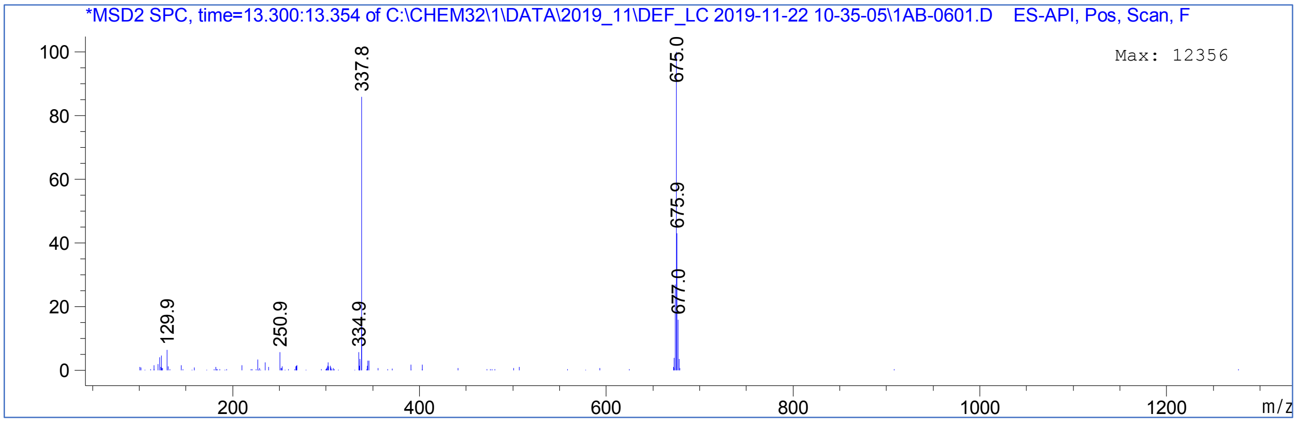
*MSD2 SPC, time=13.300:13.354 of C:\CHEM32\1\DATA\2019_11\DEF_LC 2019-11-22 10-35-05\1AB-0601.D ES-API, Pos, Scan, F
Max: 12356
129.9
250.9
334.9
337.8
675.0
675.9
677.0
m/z

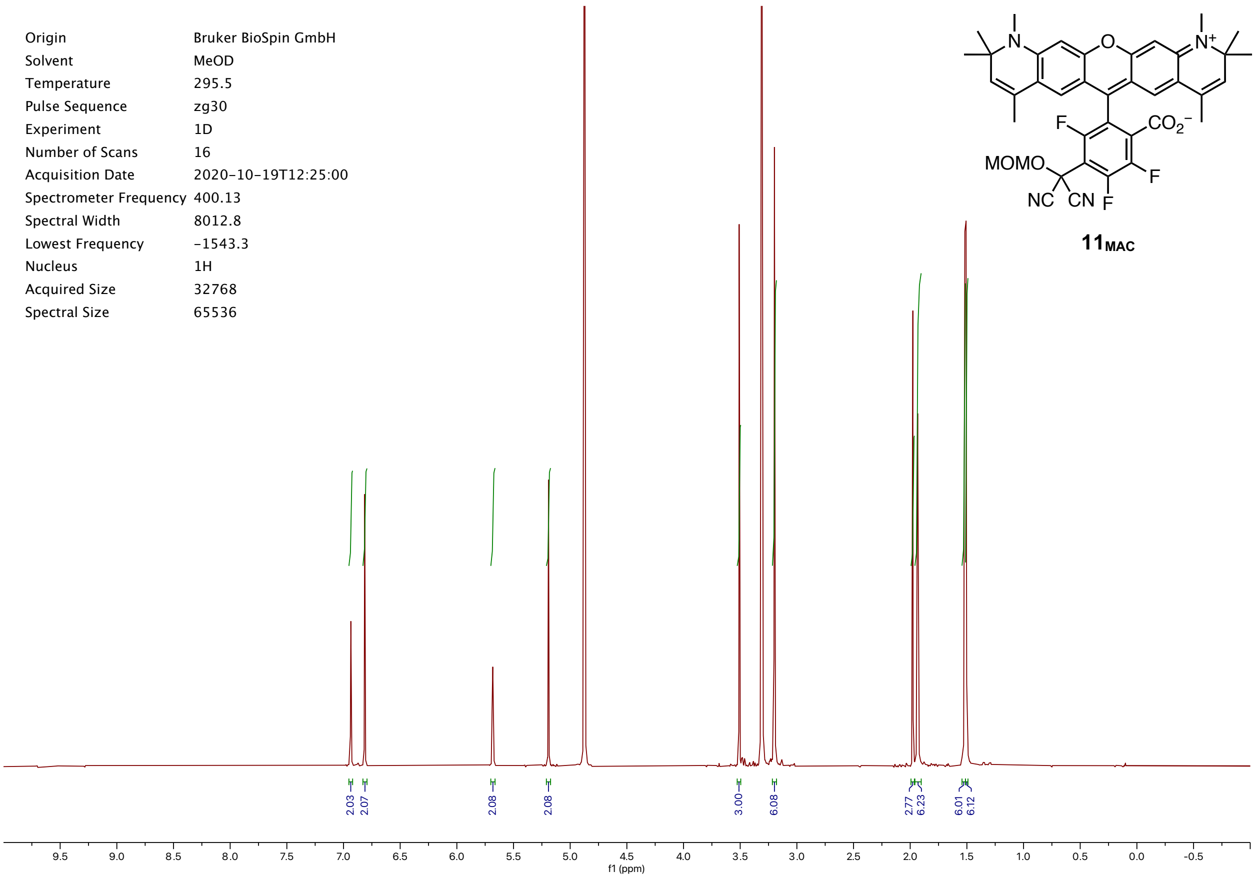
Origin Bruker BioSpin GmbH
Solvent MeOD
Temperature 295.5
Pulse Sequence zg30
Experiment 1D
Number of Scans 16
Acquisition Date 2020-10-19T12:25:00
Spectrometer Frequency 400.13
Spectral Width 8012.8
Lowest Frequency -1543.3
Nucleus 1H
Acquired Size 32768
Spectral Size 65536
MOMO
NC CN
11MAC
2.03
2.07
2.08
2.08
3.00
6.08
2.77
6.23
6.01
6.12
f1 (ppm)

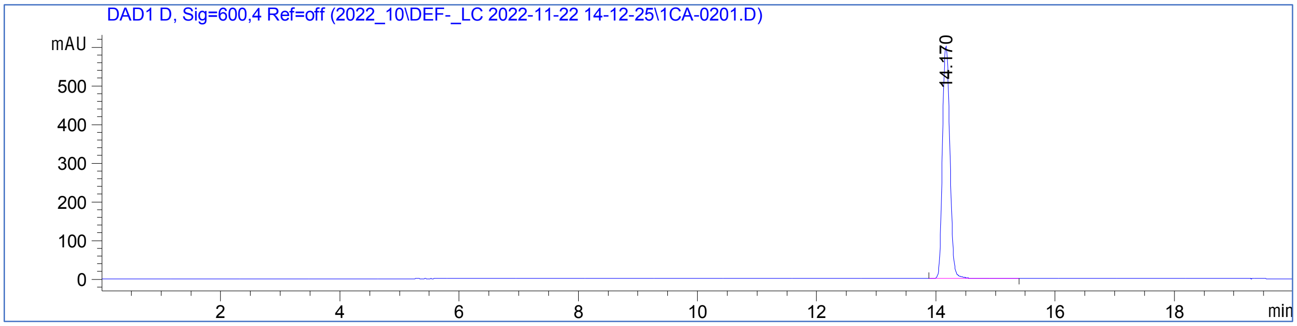
DAD1 D, Sig=600,4 Ref=off (2022_10\DEF-_LC 2022-11-22 14-12-25\1CA-0201.D)
mAU
14.170
min

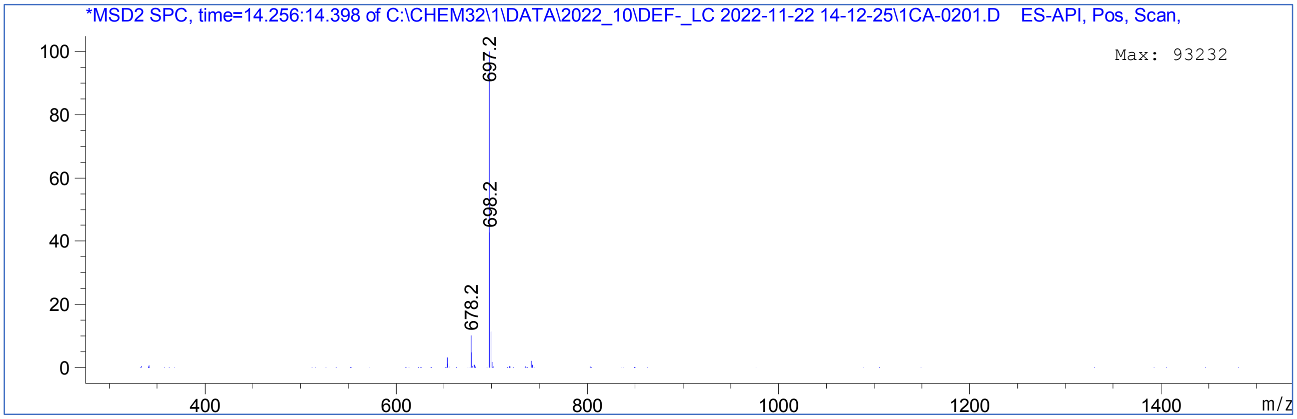
*MSD2 SPC, time=14.256:14.398 of C:\CHEM32\1\DATA\2022_10\DEF-_LC 2022-11-22 14-12-25\1CA-0201.D ES-API, Pos, Scan,
Max: 93232
697.2
698.2
678.2
m/z

Origin	Bruker BioSpin GmbH
Solvent	MeOD
Temperature	295.3
Pulse Sequence	zg30
Experiment	1D
Number of Scans	16
Acquisition Date	2020-10-21T12:09:00
Spectrometer Frequency	400.13
Spectral Width	8012.8
Lowest Frequency	-1543.3
Nucleus	1H
Acquired Size	32768
Spectral Size	65536

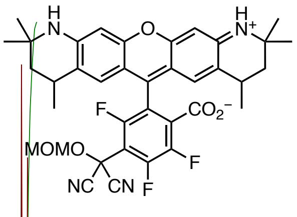

18MAC

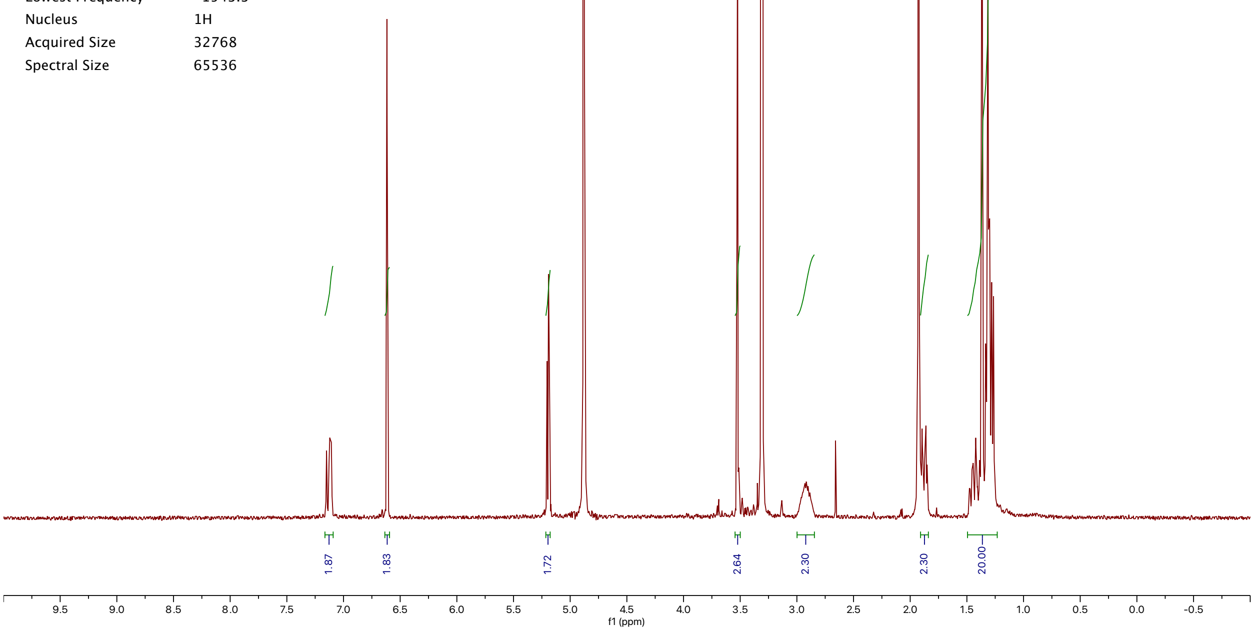

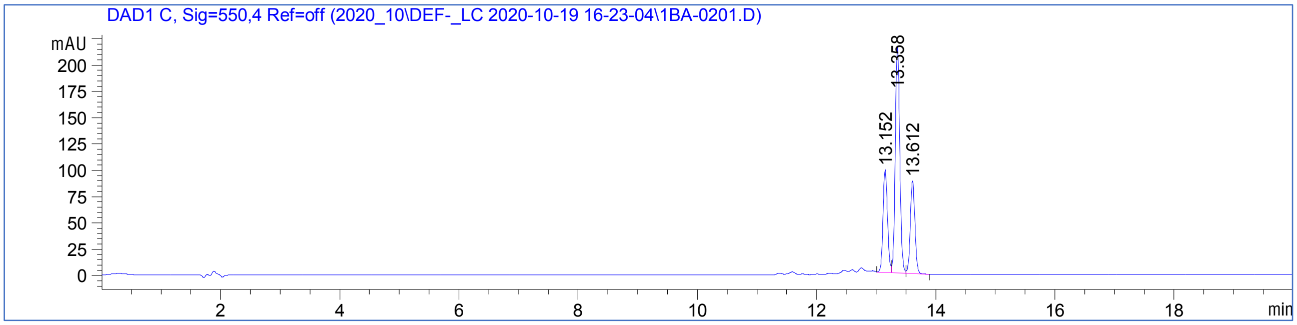

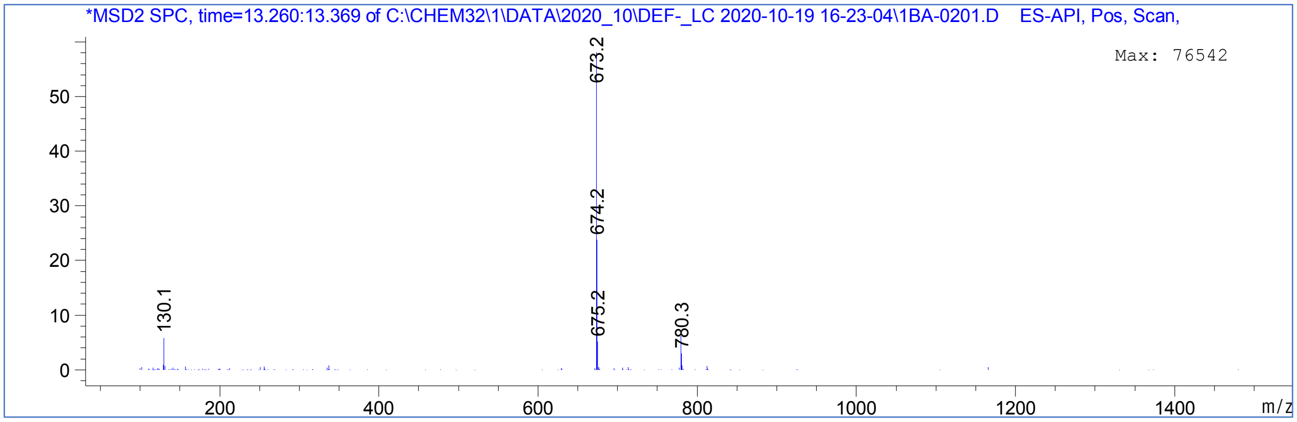

Origin	Bruker BioSpin GmbH
Solvent	CDCl3
Temperature	300.0
Pulse Sequence	zg30
Experiment	1D
Number of Scans	16
Acquisition Date	2021-01-15T15:17:00
Spectrometer Frequency	400.13
Spectral Width	8012.8
Lowest Frequency	-1546.1
Nucleus	1H
Acquired Size	32768
Spectral Size	65536

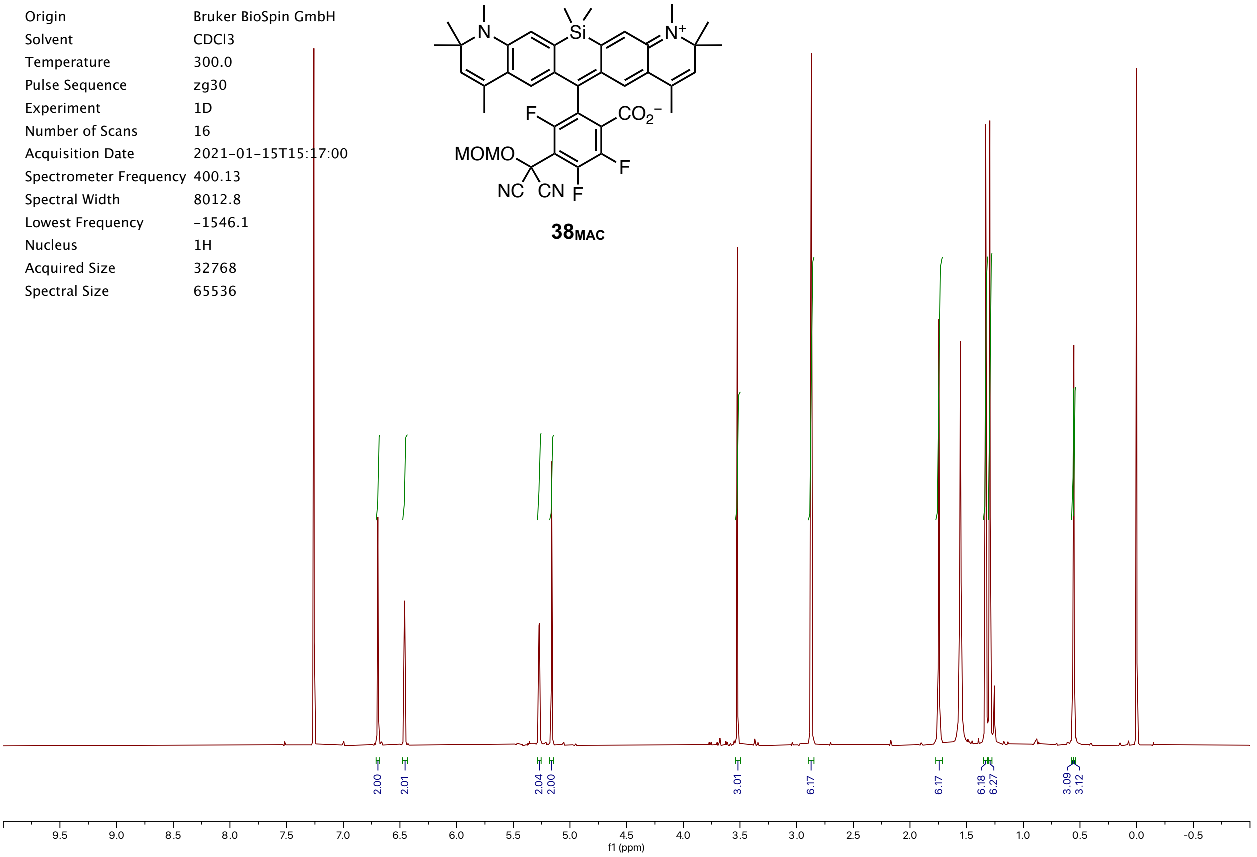

38MAC

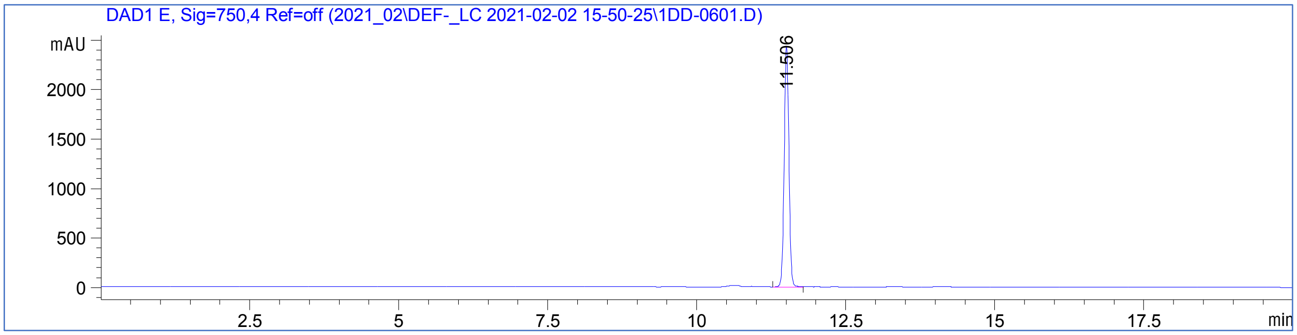

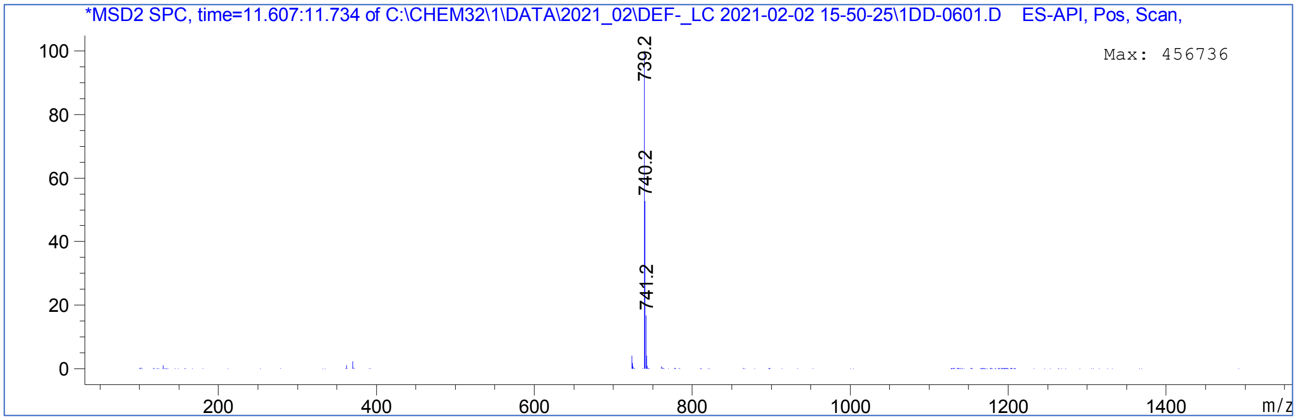

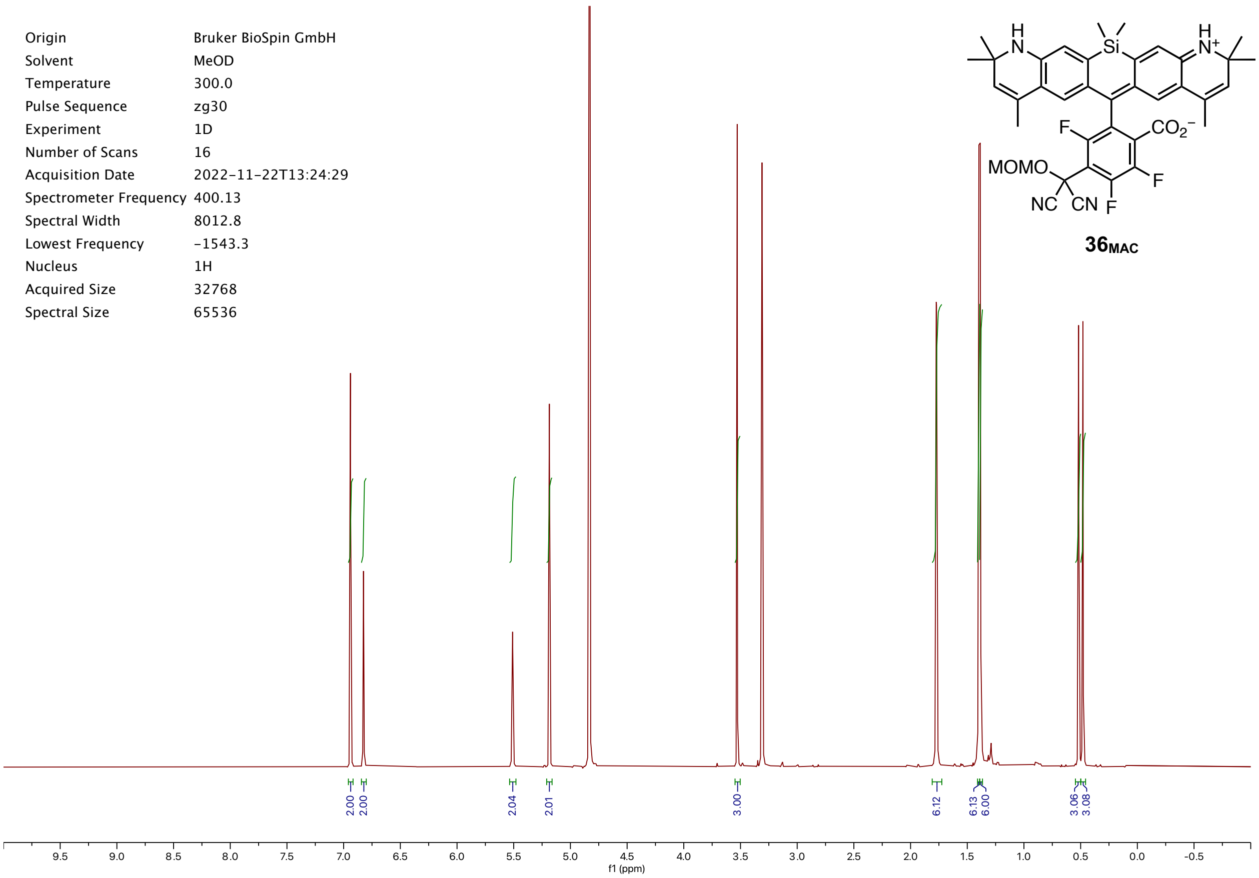

Origin Bruker BioSpin GmbH
Solvent MeOD
Temperature 300.0
Pulse Sequence zg30
Experiment 1D
Number of Scans 16
Acquisition Date 2022-11-22T13:24:29
Spectrometer Frequency 400.13
Spectral Width 8012.8
Lowest Frequency -1543.3
Nucleus 1H
Acquired Size 32768
Spectral Size 65536
2.00 2.00 2.04 2.01 3.00 6.12 6.13 6.00 3.06 3.08
9.5 9.0 8.5 8.0 7.5 7.0 6.5 6.0 5.5 5.0 4.5 4.0 3.5 3.0 2.5 2.0 1.5 1.0 0.5 0.0 -0.5
f1 (ppm)
H
N
Si
H
N+
F
CO2−
MOMO
F
NC
CN
F

36MAC

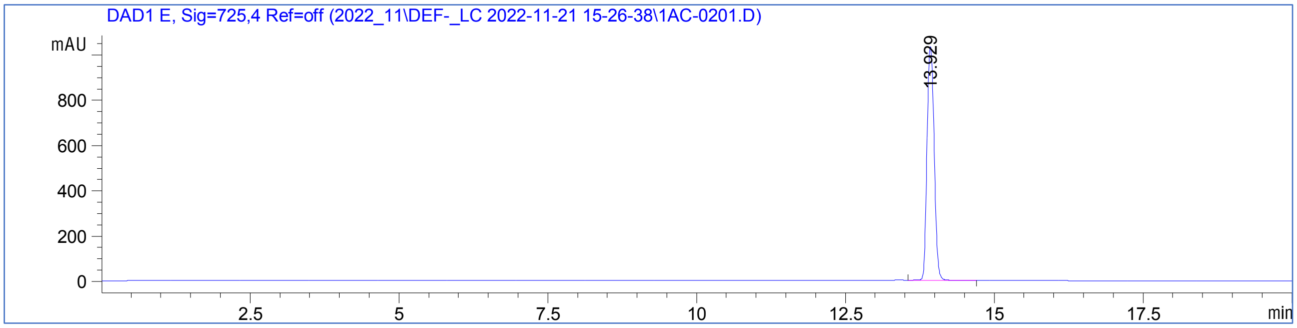

DAD1 E, Sig=725,4 Ref=off (2022_11\DEF-_LC 2022-11-21 15-26-38\1AC-0201.D)
mAU
13.929
800
600
400
200
0
2.5 5 7.5 10 12.5 15 17.5
min

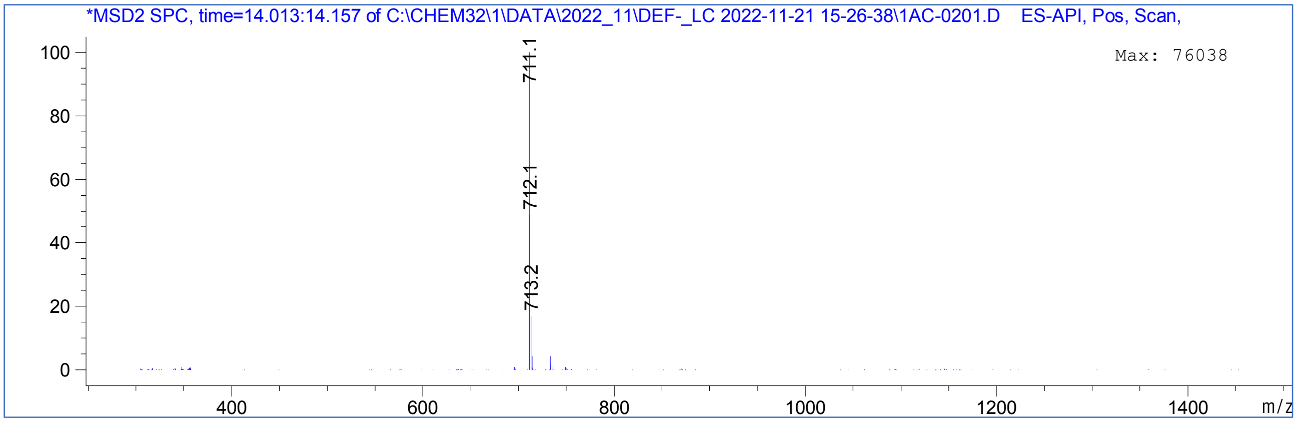

*MSD2 SPC, time=14.013:14.157 of C:\CHEM32\1\DATA\2022_11\DEF-_LC 2022-11-21 15-26-38\1AC-0201.D ES-API, Pos, Scan,
Max: 76038
711.1
712.1
713.2
100
80
60
40
20
0
400 600 800 1000 1200 1400
m/z

Origin	Bruker BioSpin GmbH
Solvent	MeOD
Temperature	300.0
Pulse Sequence	zg30
Experiment	1D
Number of Scans	16
Acquisition Date	2021-11-18T09:49:00
Spectrometer Frequency	400.13
Spectral Width	8012.8
Lowest Frequency	-1543.2
Nucleus	1H
Acquired Size	32768
Spectral Size	65536

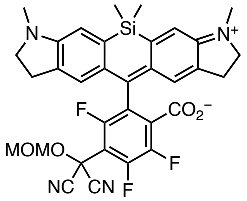

108MAC

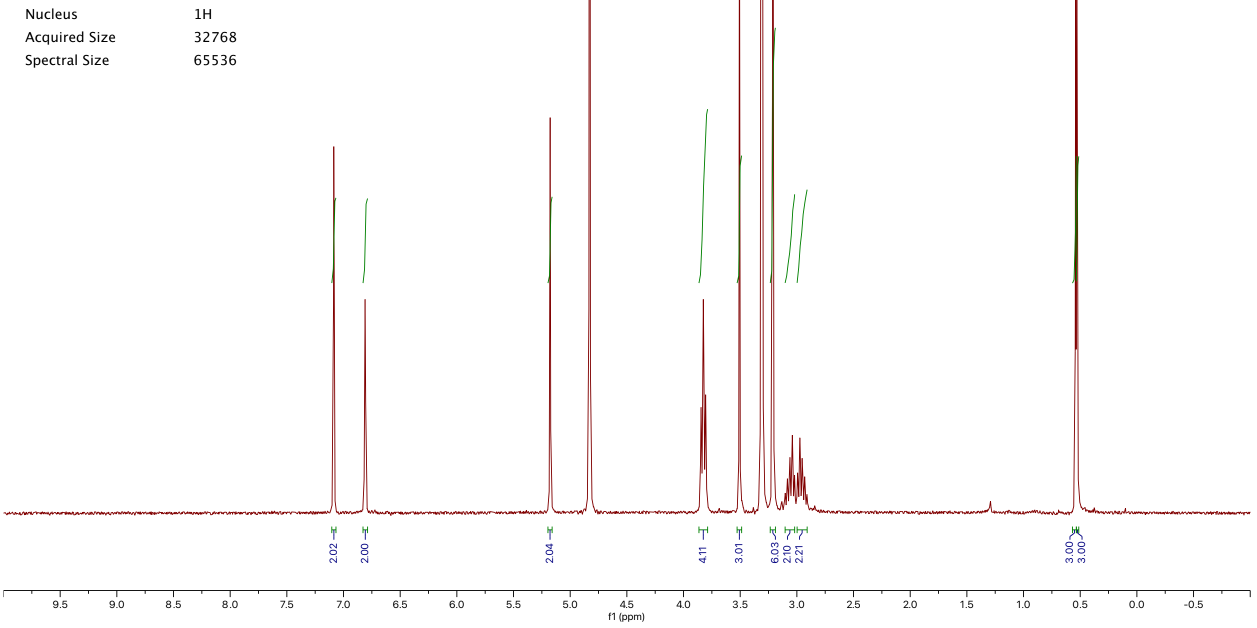

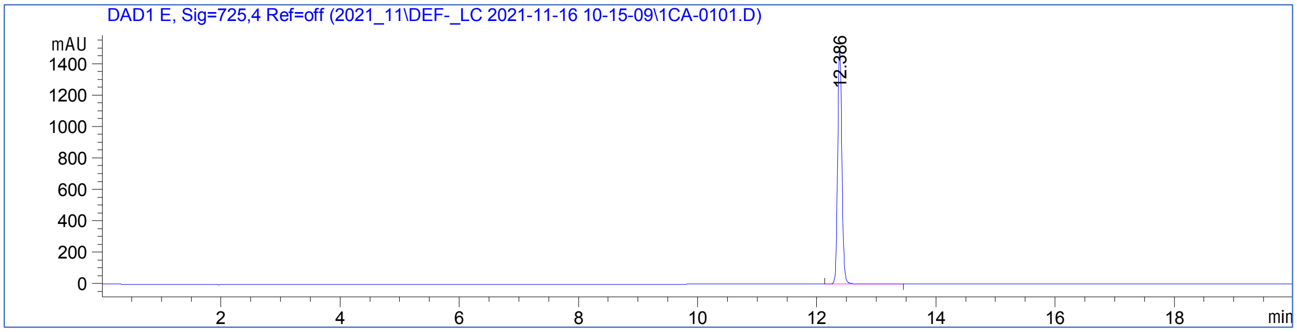

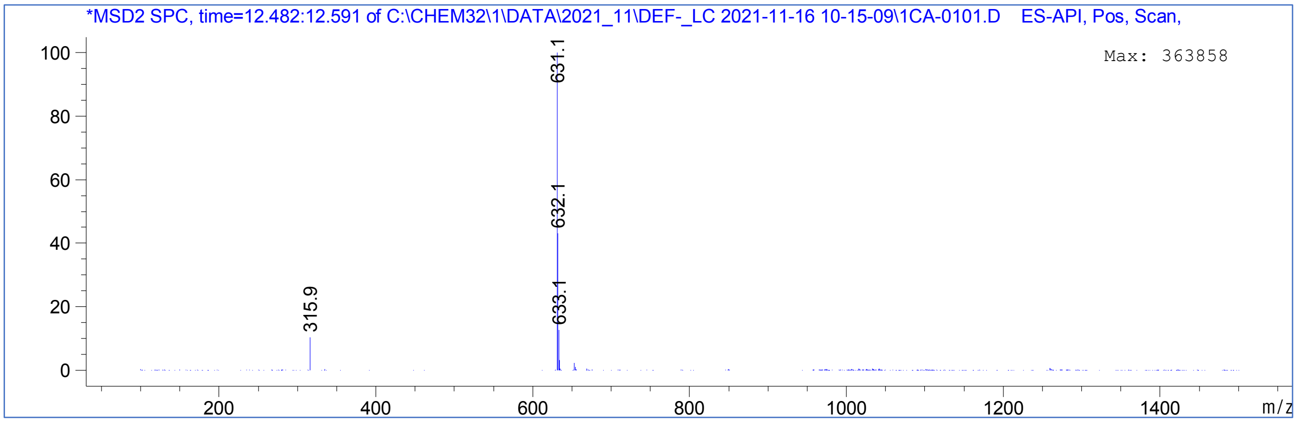

Origin	Bruker BioSpin GmbH
Solvent	MeOD
Temperature	300.0
Pulse Sequence	zg30
Experiment	1D
Number of Scans	16
Acquisition Date	2021-11-18T09:58:00
Spectrometer Frequency	400.13
Spectral Width	8012.8
Lowest Frequency	-1543.1
Nucleus	1H
Acquired Size	32768
Spectral Size	65536

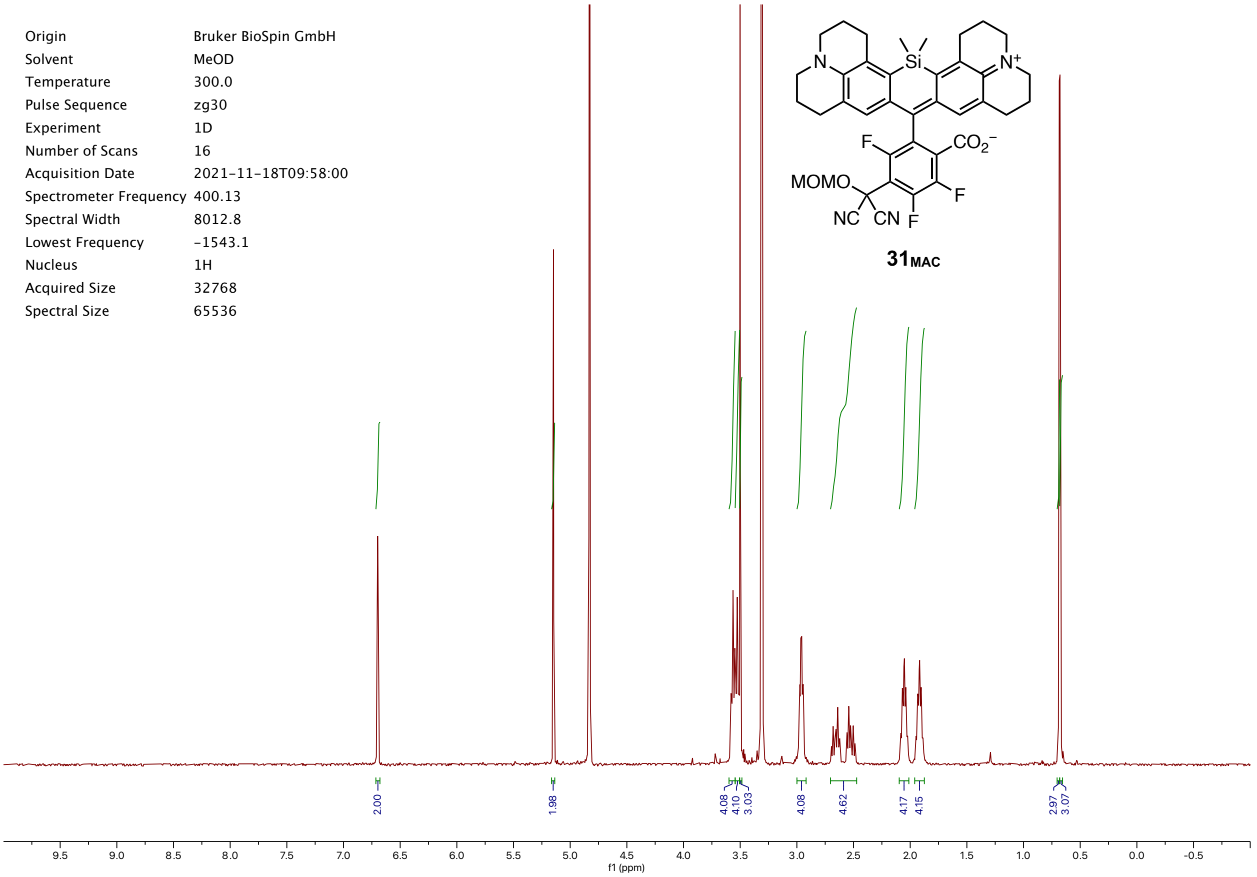

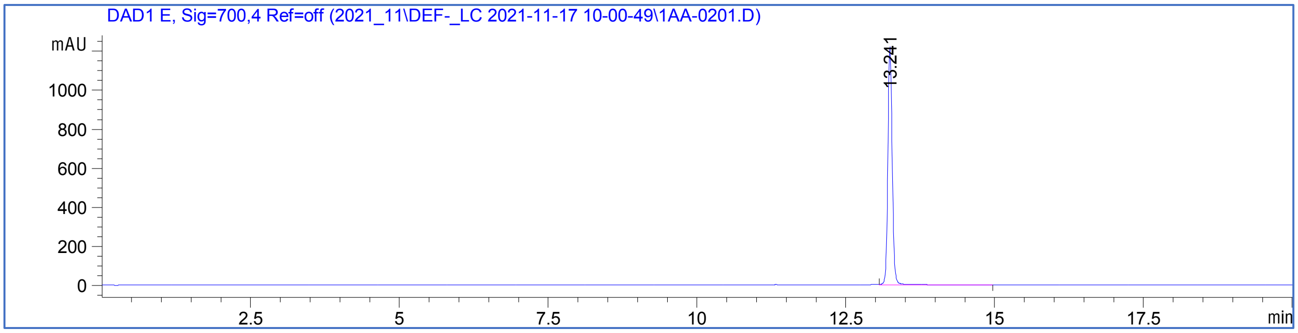

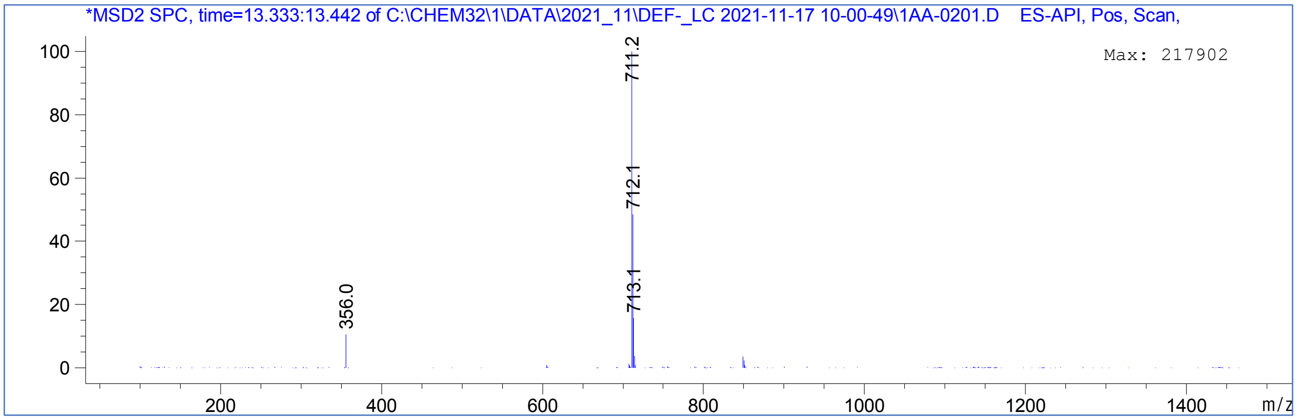

Origin	Bruker BioSpin GmbH
Solvent	MeOD
Temperature	300.0
Pulse Sequence	zg30
Experiment	1D
Number of Scans	16
Acquisition Date	2021-11-19T15:45:00
Spectrometer Frequency	400.13
Spectral Width	8012.8
Lowest Frequency	-1543.1
Nucleus	1H
Acquired Size	32768
Spectral Size	65536

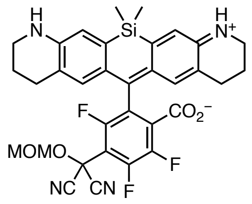

29MAC

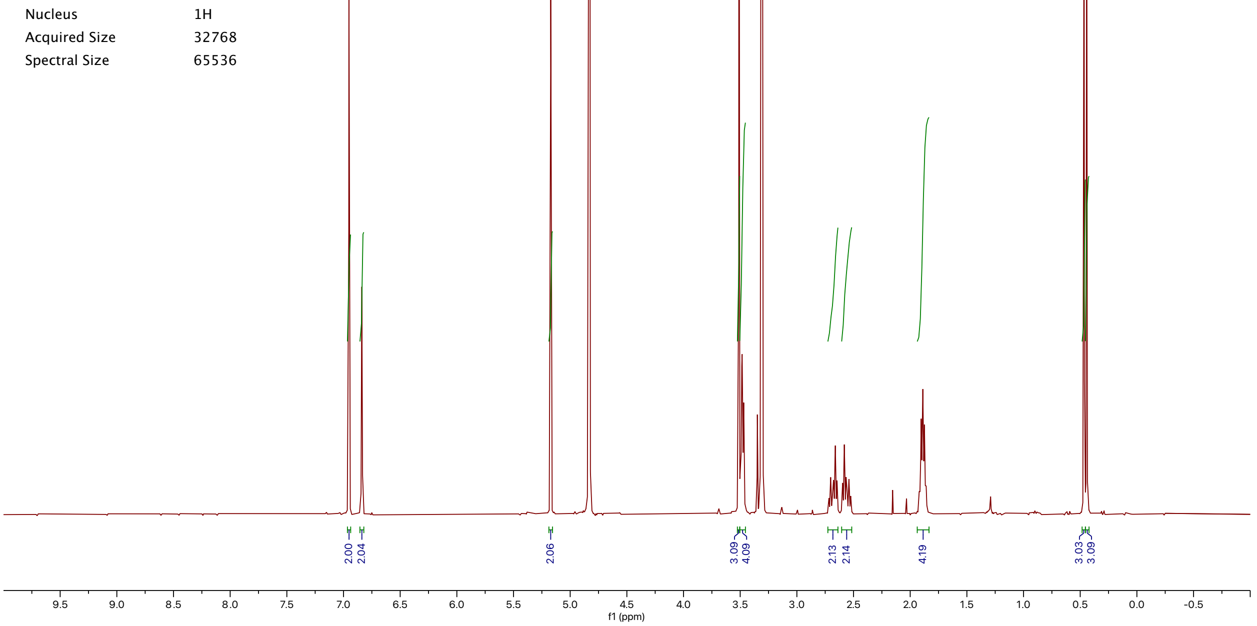

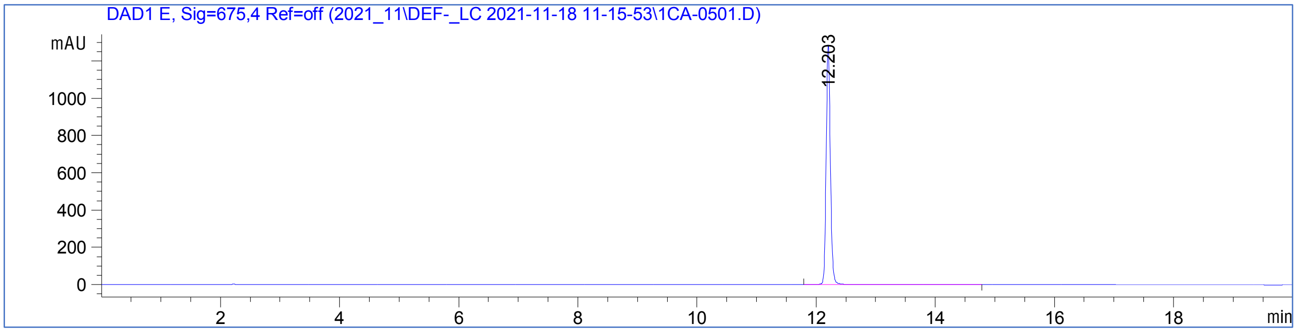

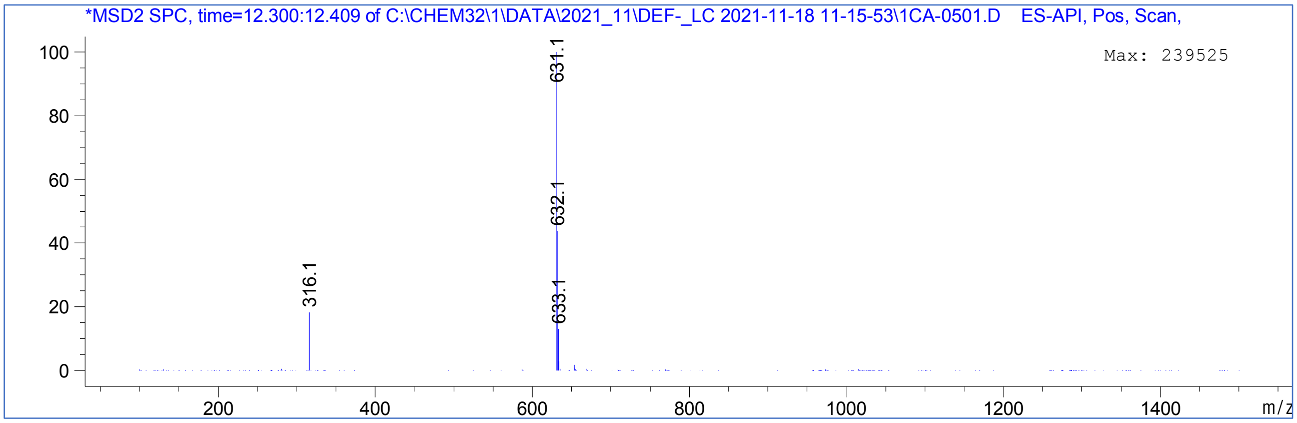

Origin	Bruker BioSpin GmbH
Solvent	MeOD
Temperature	300.0
Pulse Sequence	zg30
Experiment	1D
Number of Scans	16
Acquisition Date	2022-05-12T16:33:00
Spectrometer Frequency	400.13
Spectral Width	8012.8
Lowest Frequency	-1543.2
Nucleus	1H
Acquired Size	32768
Spectral Size	65536

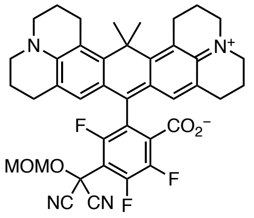

57MAC

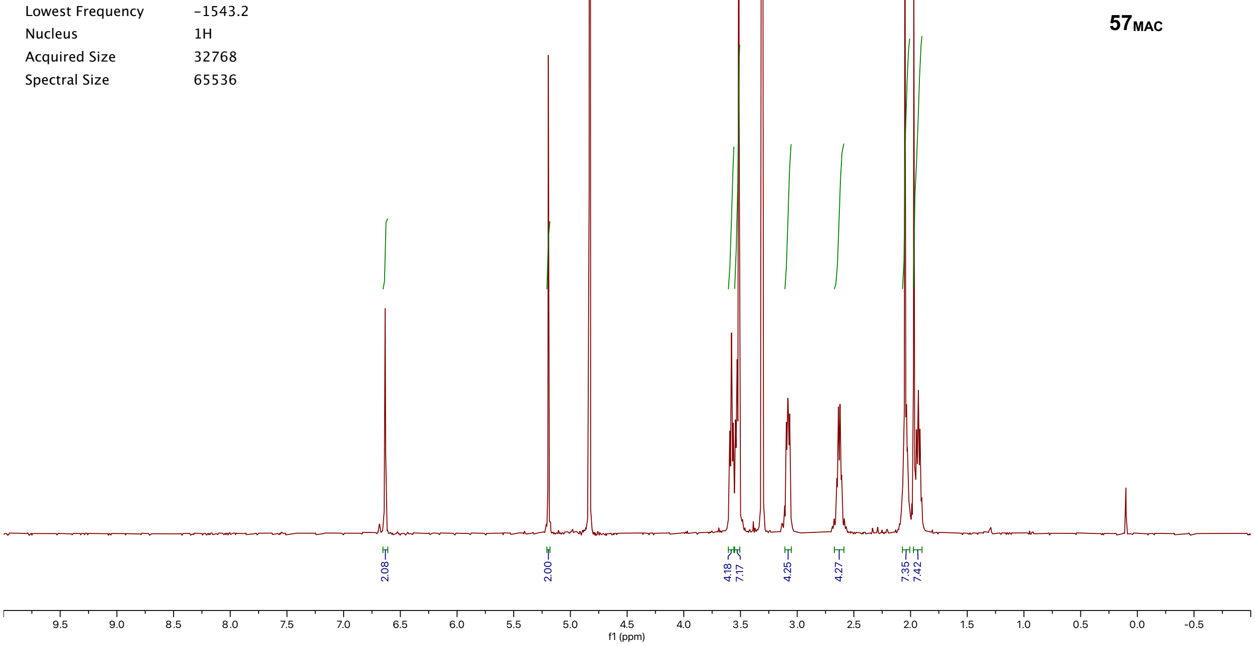

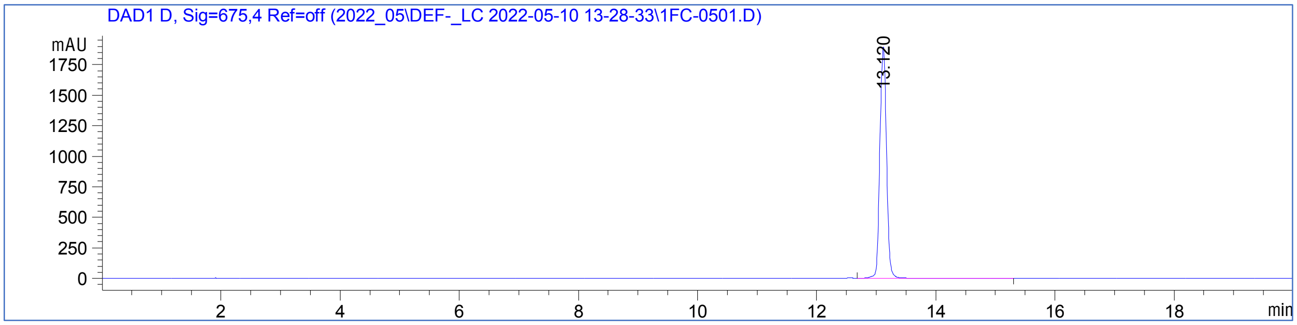

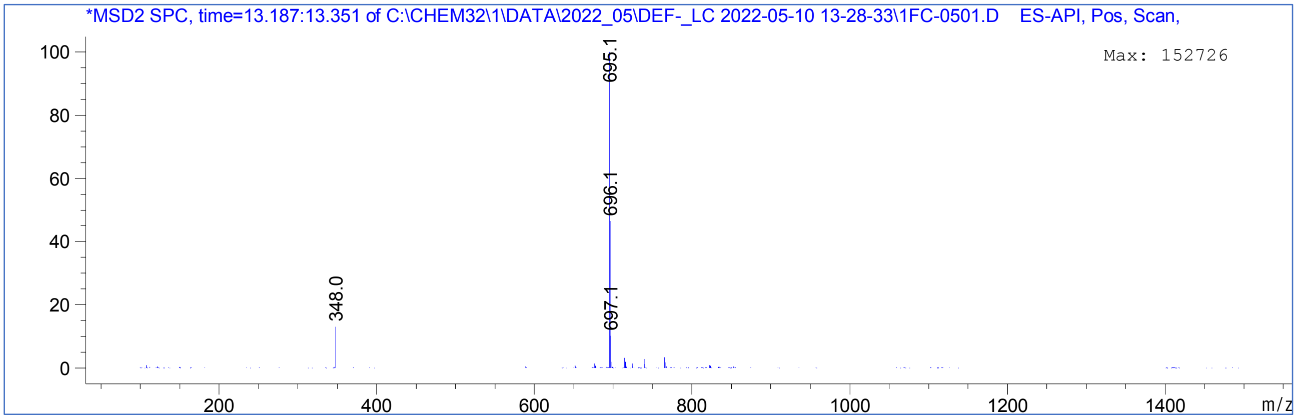

Origin	Bruker BioSpin GmbH
Solvent	MeOD
Temperature	300.0
Pulse Sequence	zg30
Experiment	1D
Number of Scans	128
Acquisition Date	2022-05-03T16:08:00
Spectrometer Frequency	400.13
Spectral Width	8012.8
Lowest Frequency	-1543.2
Nucleus	1H
Acquired Size	32768
Spectral Size	65536

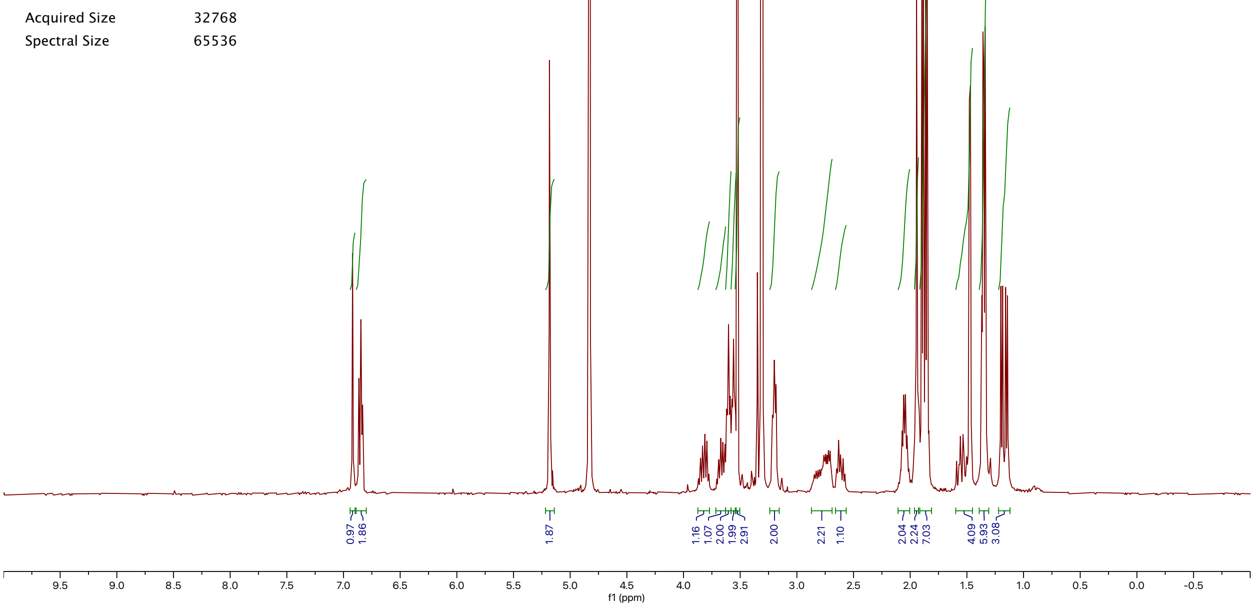

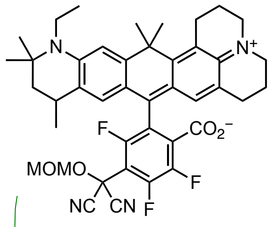

59MAC

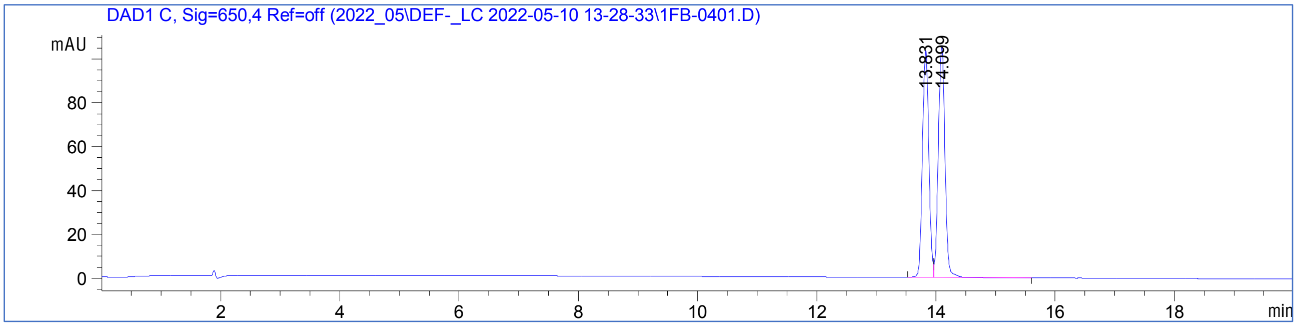

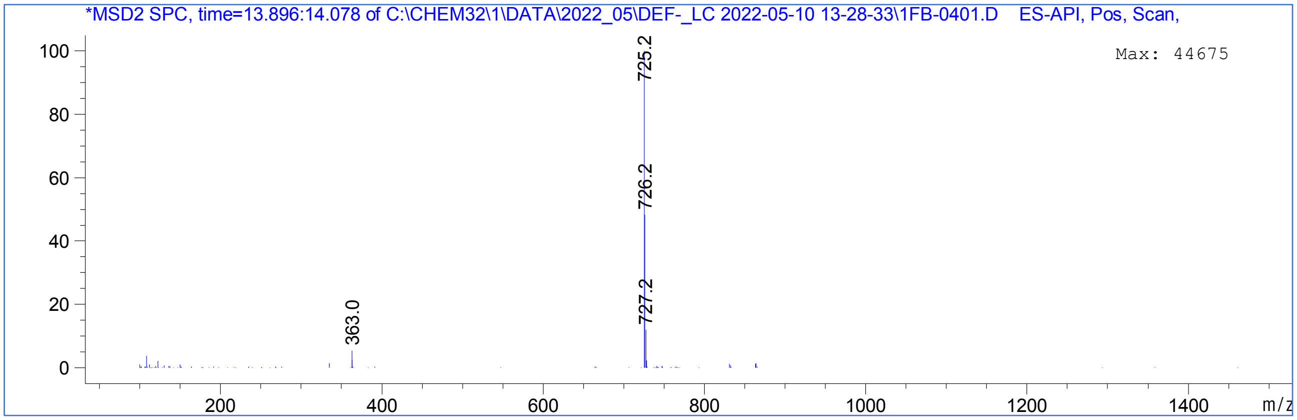

Origin	Bruker BioSpin GmbH
Solvent	CDCl3
Temperature	295.5
Pulse Sequence	zg30
Experiment	1D
Number of Scans	16
Acquisition Date	2020-10-26T14:06:00
Spectrometer Frequency	400.13
Spectral Width	8012.8
Lowest Frequency	-1545.2
Nucleus	1H
Acquired Size	32768
Spectral Size	65536

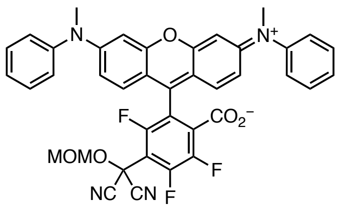

64_{MAC}

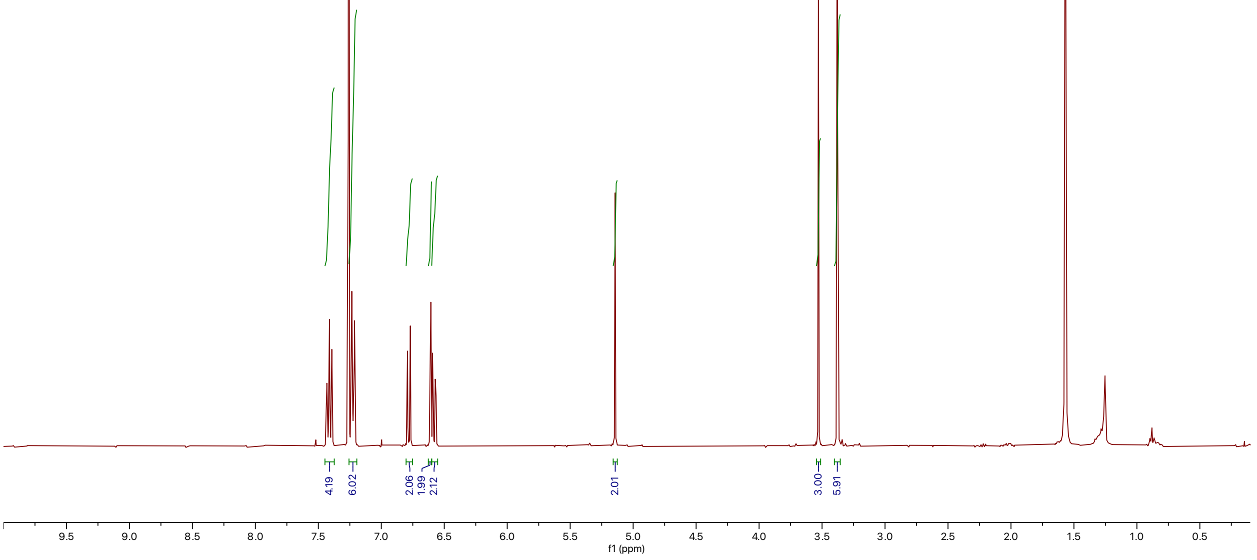

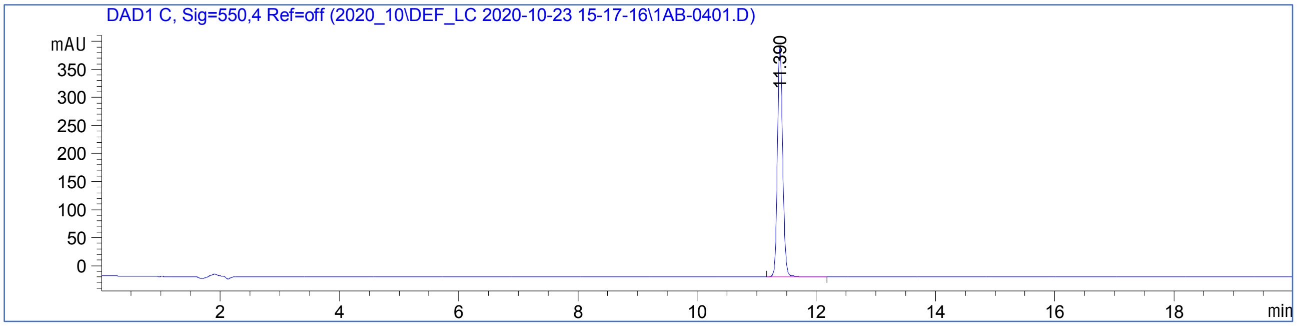

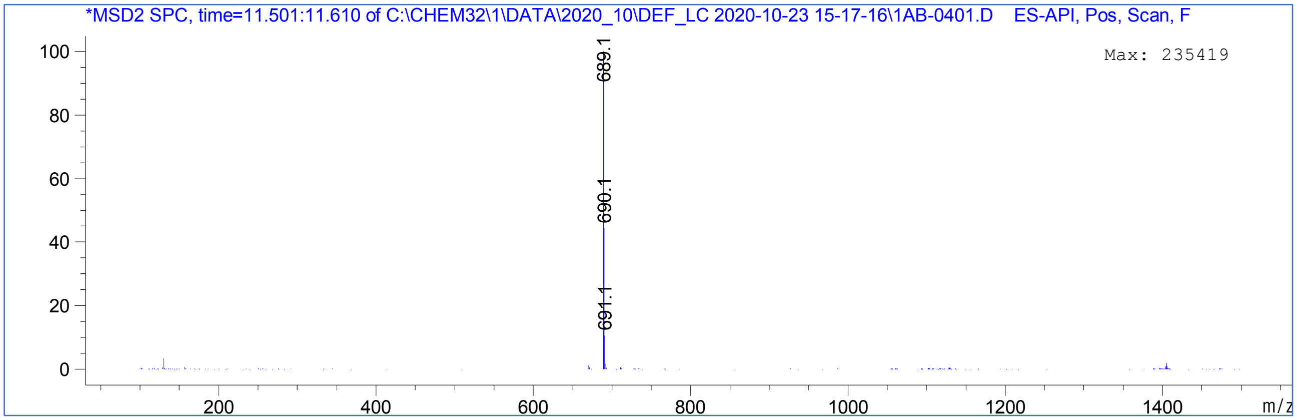

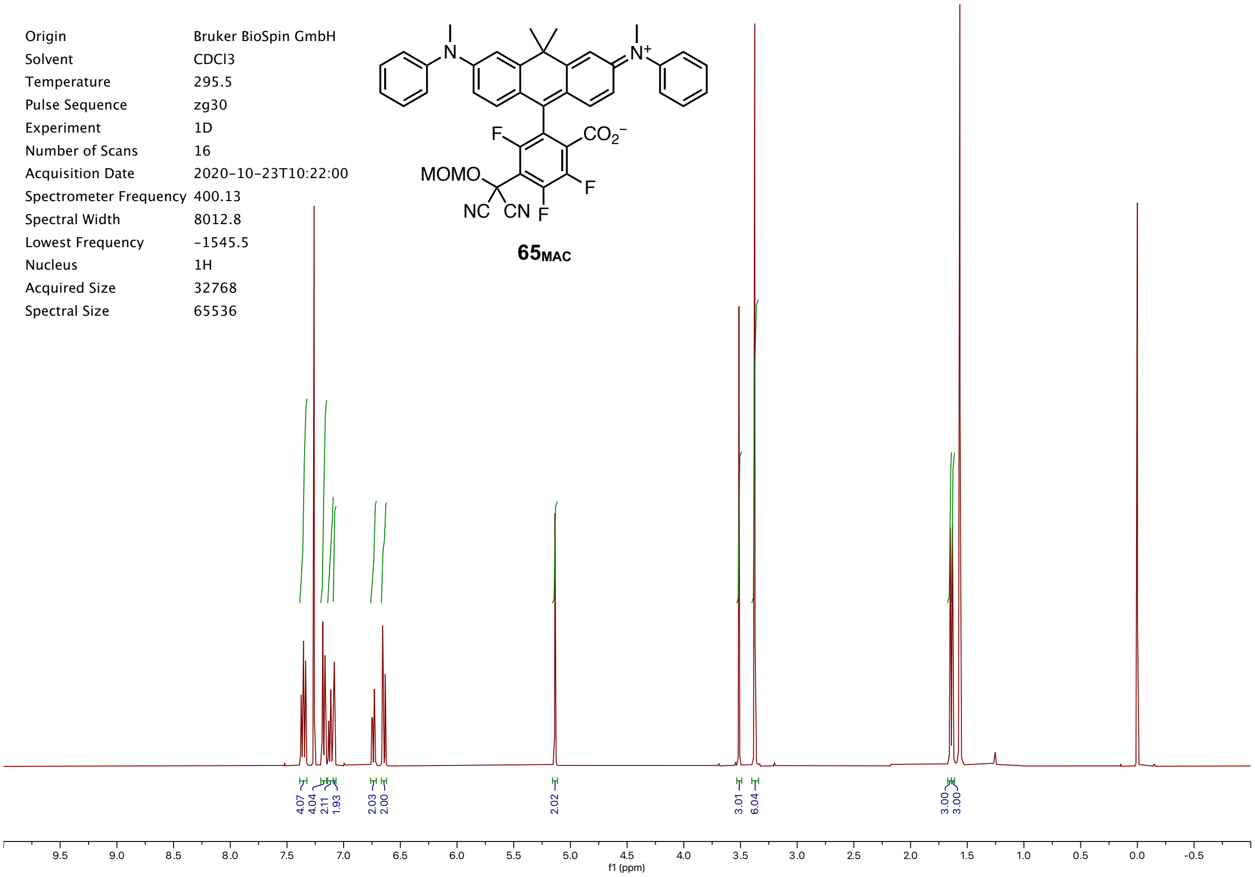
Origin Bruker BioSpin GmbH
Solvent CDCl3
Temperature 295.5
Pulse Sequence zg30
Experiment 1D
Number of Scans 16
Acquisition Date 2020-10-23T10:22:00
Spectrometer Frequency 400.13
Spectral Width 8012.8
Lowest Frequency -1545.5
Nucleus 1H
Acquired Size 32768
Spectral Size 65536
MOMO
NC
CN
F
F
F
CO_2^-
N
N^+
65MAC
4.07
4.04
2.11
1.93
2.03
2.00
2.02
3.01
6.04
3.00
3.00
f1 (ppm)

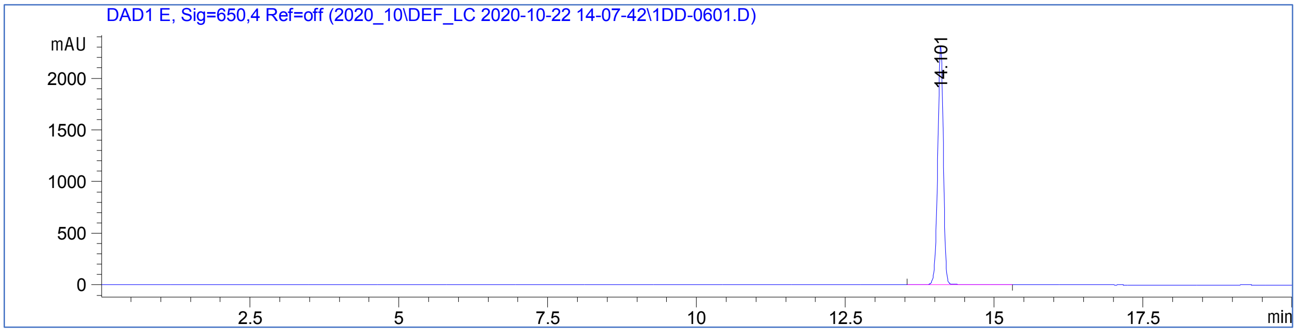
DAD1 E, Sig=650,4 Ref=off (2020_10\DEF_LC 2020-10-22 14-07-42\1DD-0601.D)
mAU
14.101
min

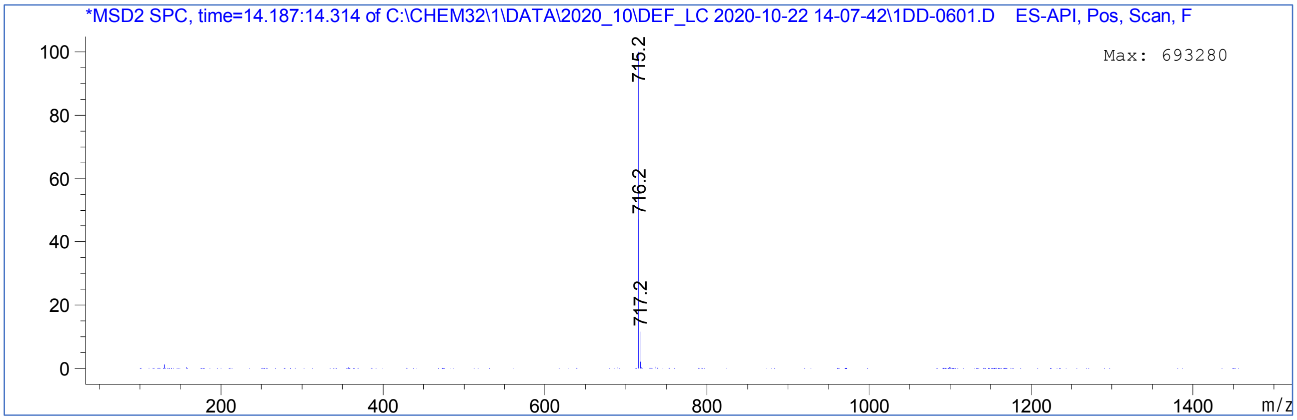
*MSD2 SPC, time=14.187:14.314 of C:\CHEM32\1\DATA\2020_10\DEF_LC 2020-10-22 14-07-42\1DD-0601.D ES-API, Pos, Scan, F
Max: 693280
715.2
716.2
717.2
m/z

Origin	Bruker BioSpin GmbH
Solvent	CDCl3
Temperature	295.4
Pulse Sequence	zg30
Experiment	1D
Number of Scans	16
Acquisition Date	2020-10-23T10:31:00
Spectrometer Frequency	400.13
Spectral Width	8012.8
Lowest Frequency	-1545.5
Nucleus	1H
Acquired Size	32768
Spectral Size	65536

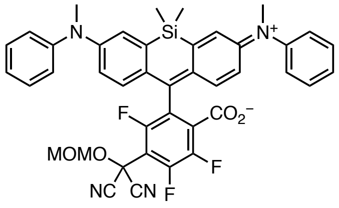

66MAC

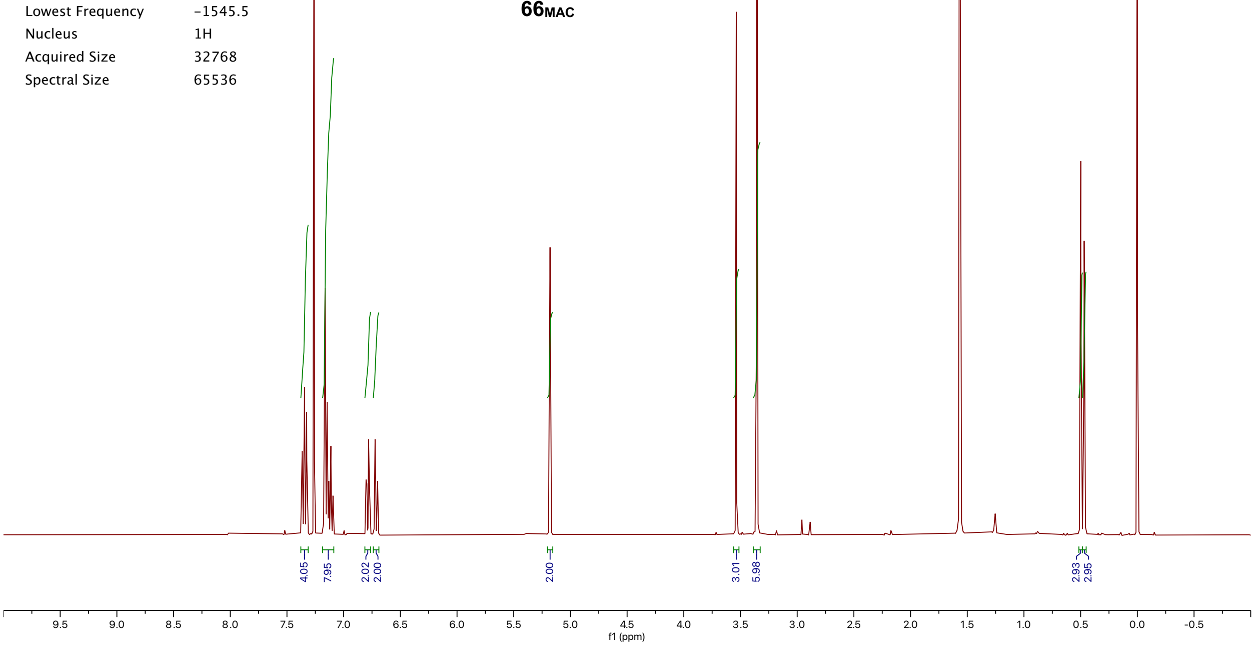

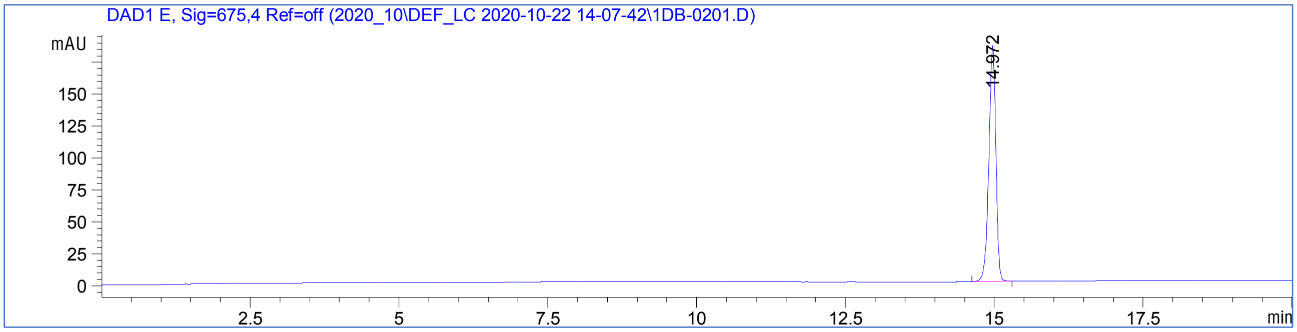

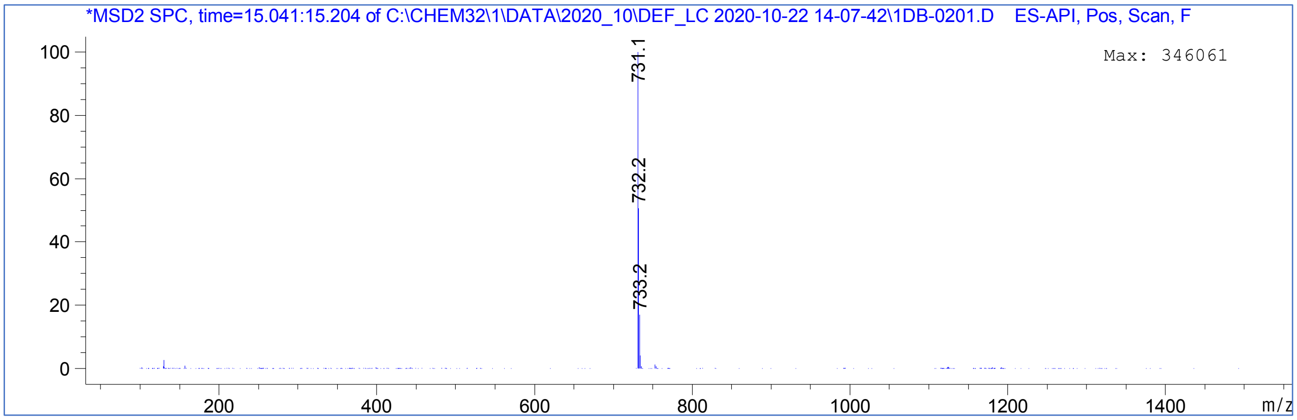

Origin	Bruker BioSpin GmbH
Solvent	MeOD
Temperature	300.0
Pulse Sequence	zg30
Experiment	1D
Number of Scans	16
Acquisition Date	2019-06-17T15:22:00
Spectrometer Frequency	400.13
Spectral Width	8012.8
Lowest Frequency	-1543.2
Nucleus	1H
Acquired Size	32768
Spectral Size	65536

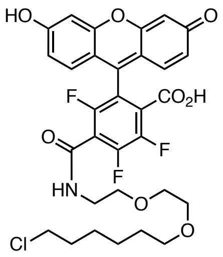

60$_{HTL}$

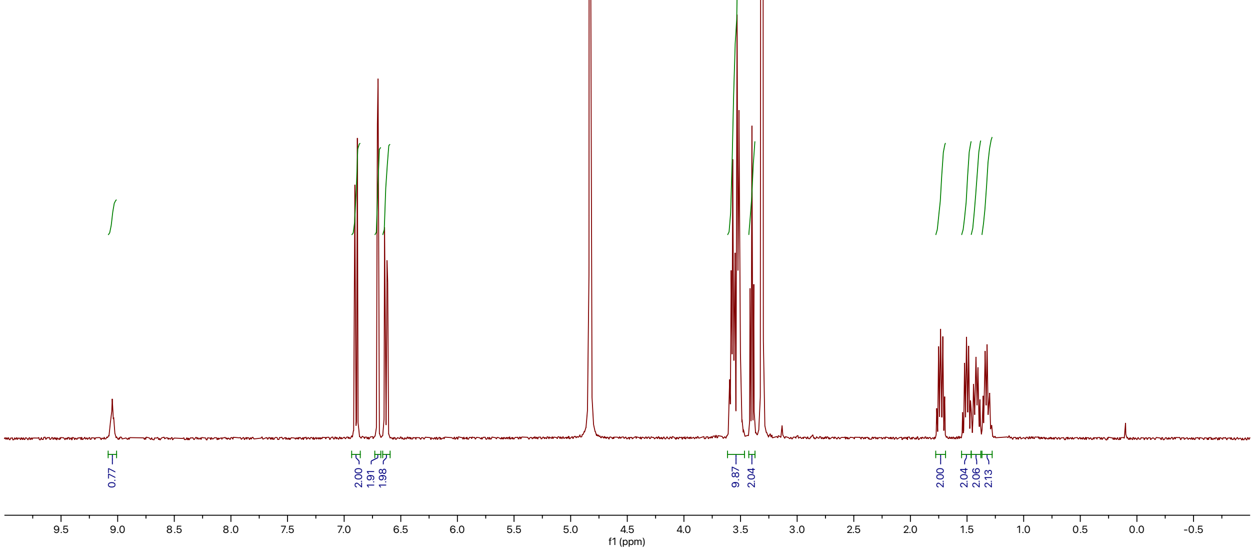

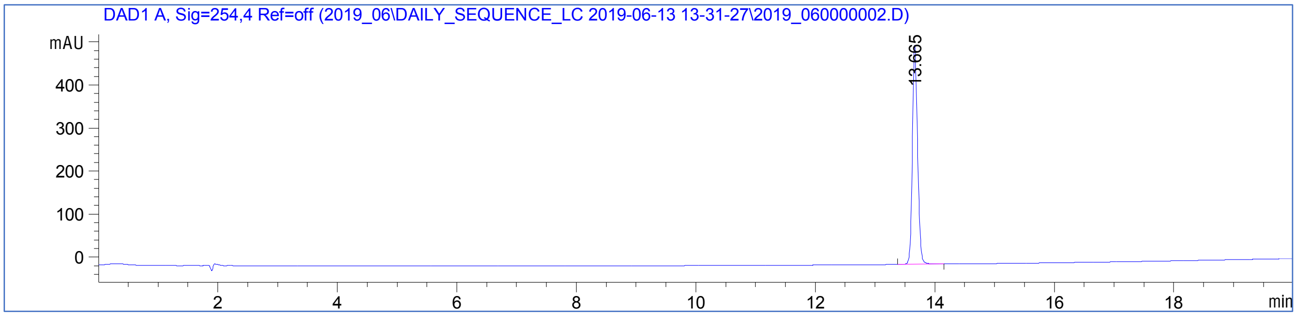

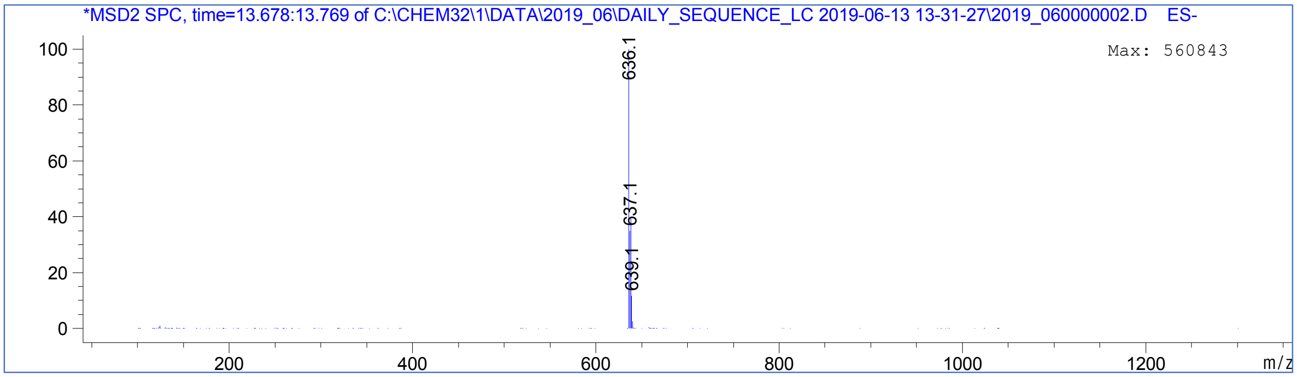

Origin	Bruker BioSpin GmbH
Solvent	MeOD
Temperature	295.5
Pulse Sequence	zg30
Experiment	1D
Number of Scans	16
Acquisition Date	2019-12-09T14:30:00
Spectrometer Frequency	400.13
Spectral Width	8012.8
Lowest Frequency	-1543.2
Nucleus	1H
Acquired Size	32768
Spectral Size	65536

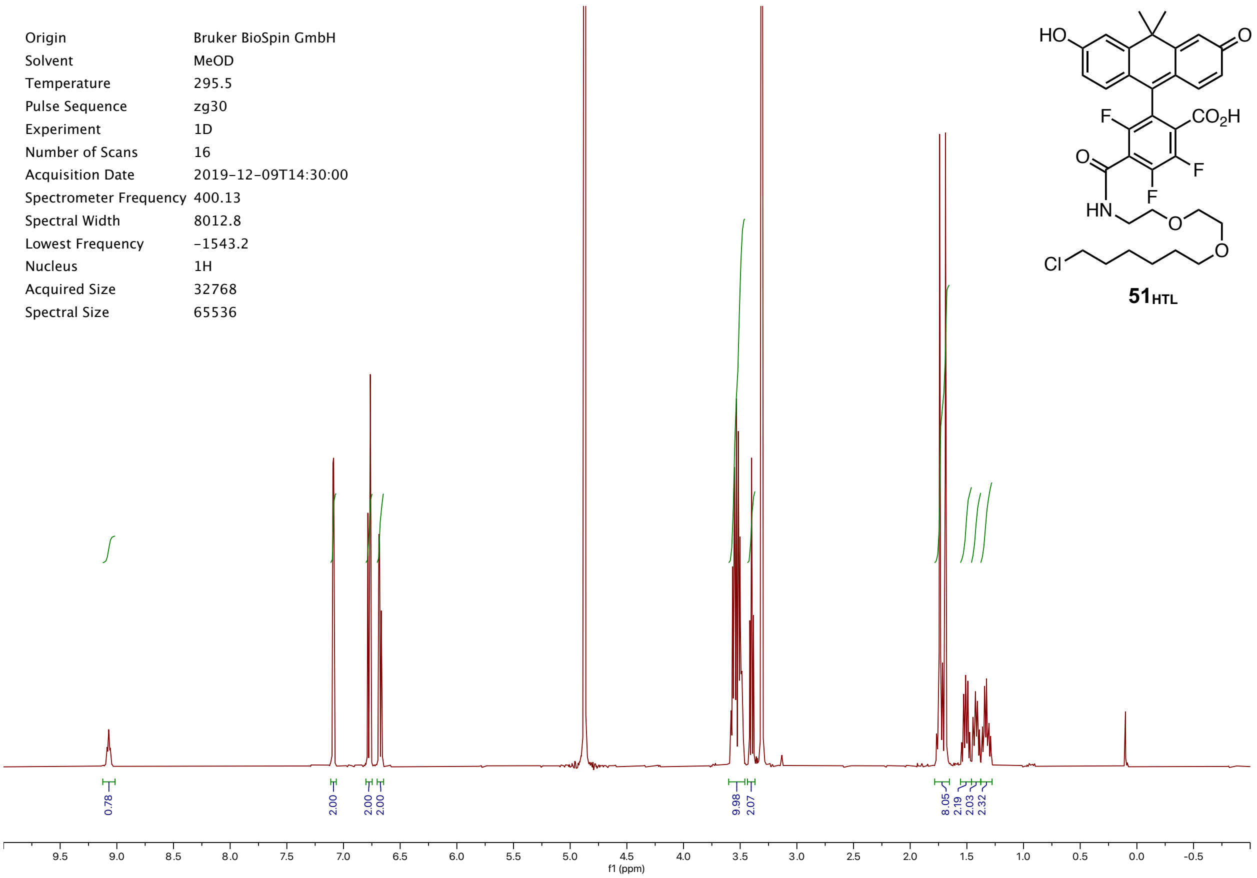

51HTL

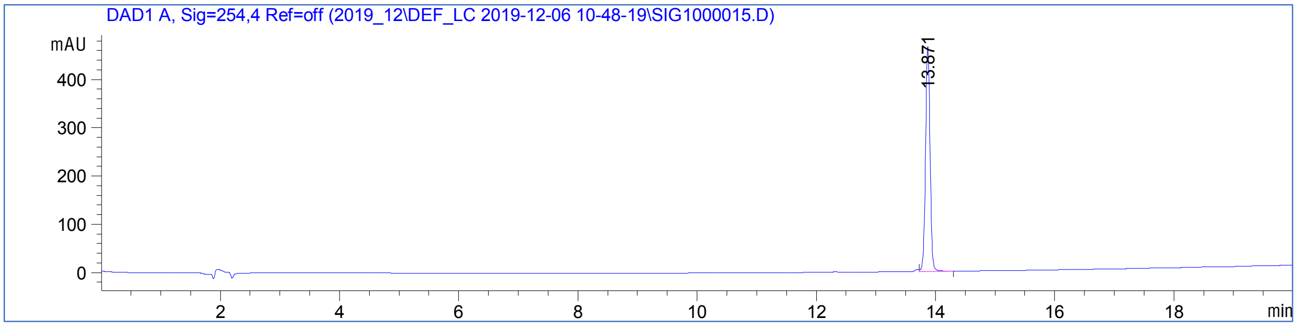

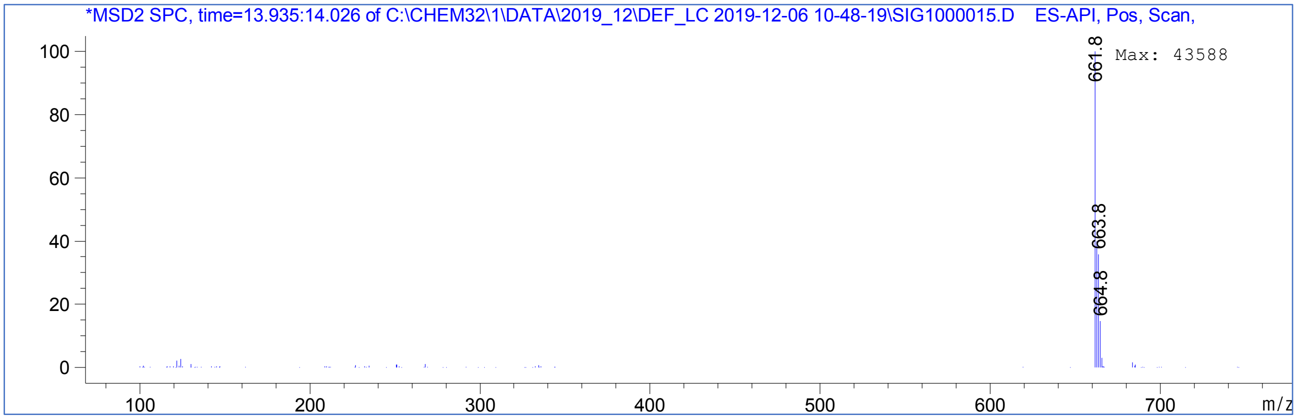

Origin	Bruker BioSpin GmbH
Solvent	MeOD
Temperature	300.0
Pulse Sequence	zg30
Experiment	1D
Number of Scans	16
Acquisition Date	2019-06-24T13:46:00
Spectrometer Frequency	400.13
Spectral Width	8012.8
Lowest Frequency	-1543.2
Nucleus	1H
Acquired Size	32768
Spectral Size	65536

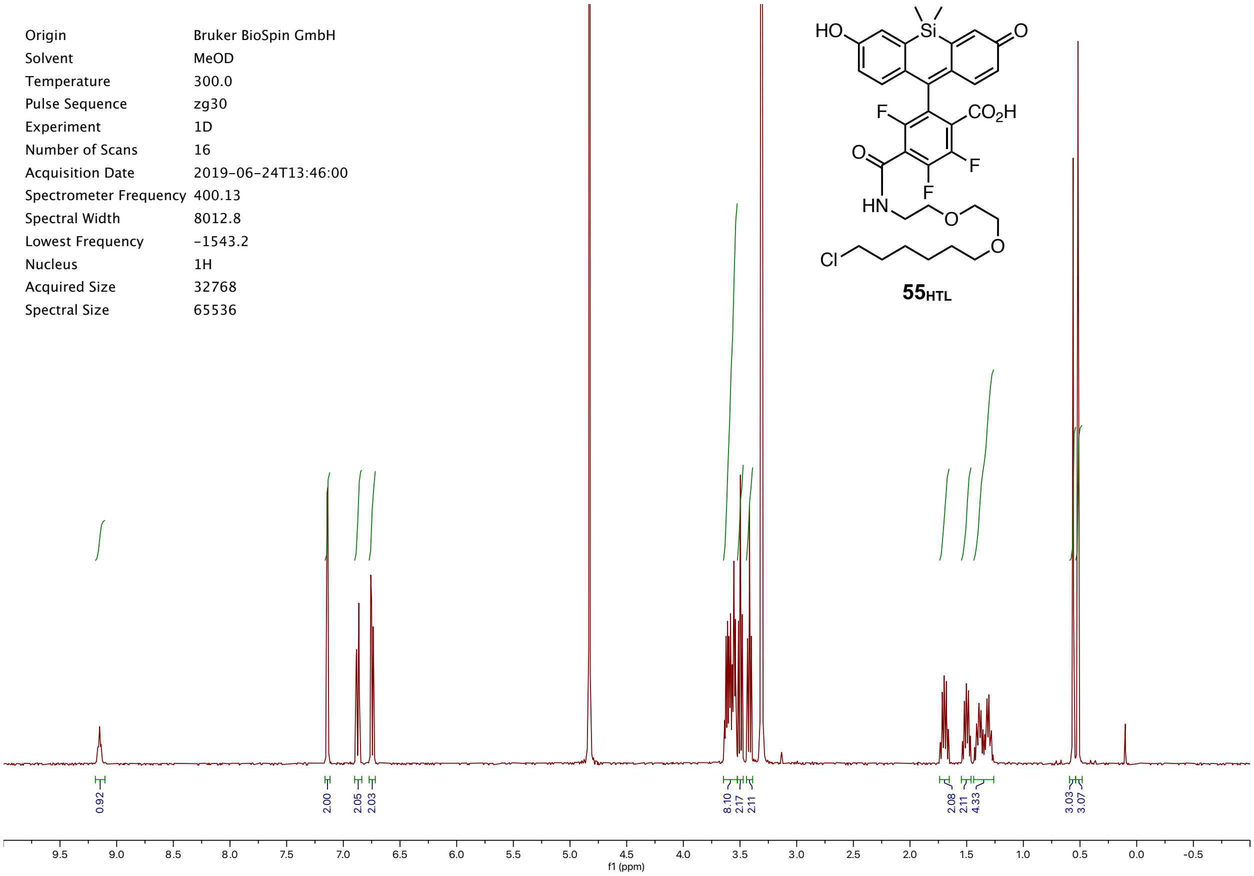

$\mathbf{55_{HTL}}$

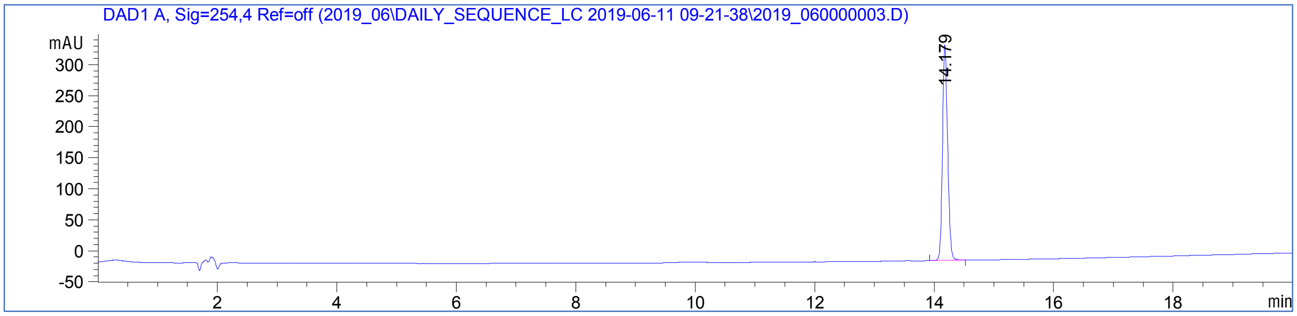

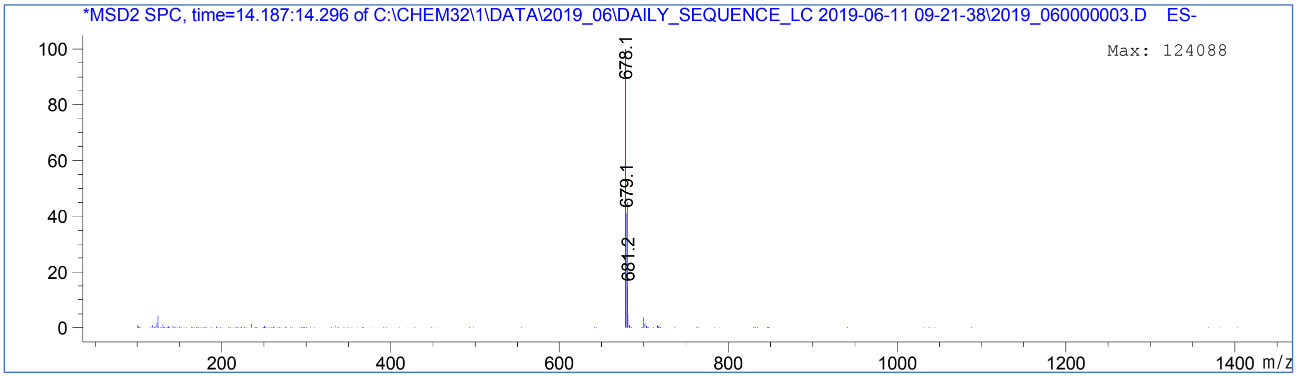

Origin	Bruker BioSpin GmbH
Solvent	MeOD
Temperature	300.0
Pulse Sequence	zg30
Experiment	1D
Number of Scans	16
Acquisition Date	2019-07-12T14:46:00
Spectrometer Frequency	400.13
Spectral Width	8012.8
Lowest Frequency	-1543.2
Nucleus	1H
Acquired Size	32768
Spectral Size	65536

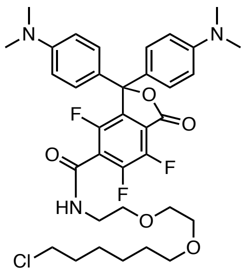

49HTL

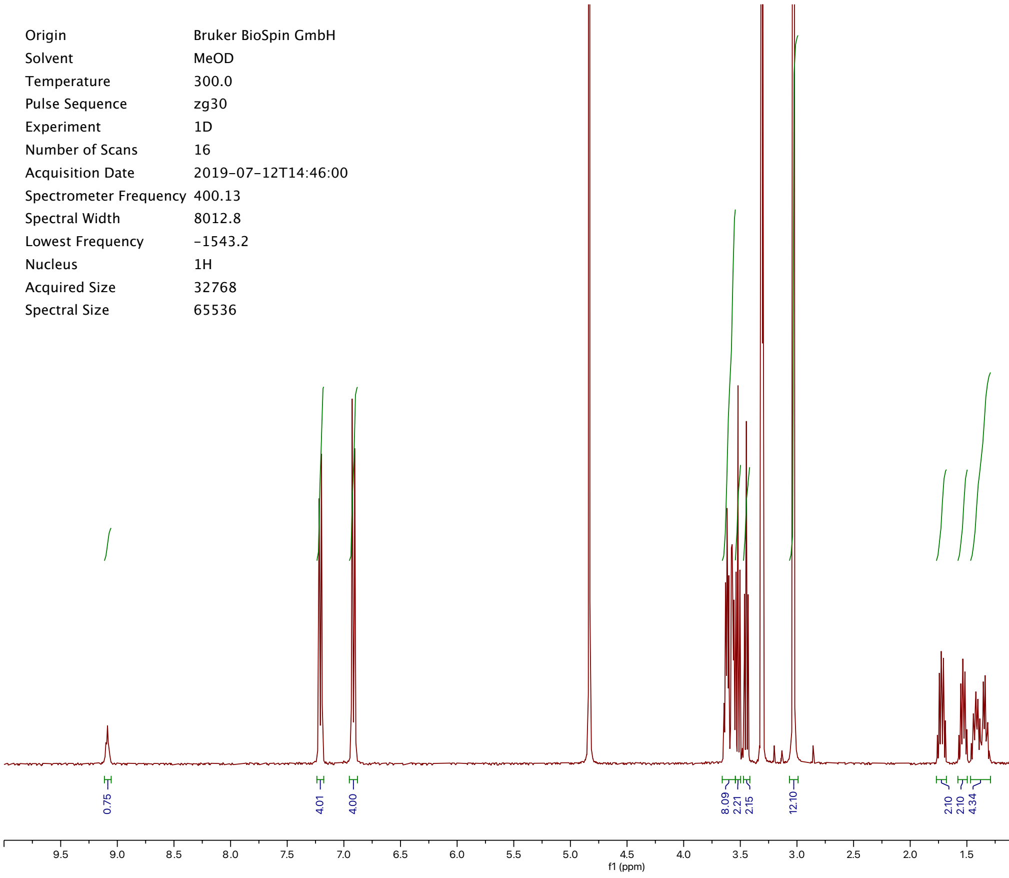

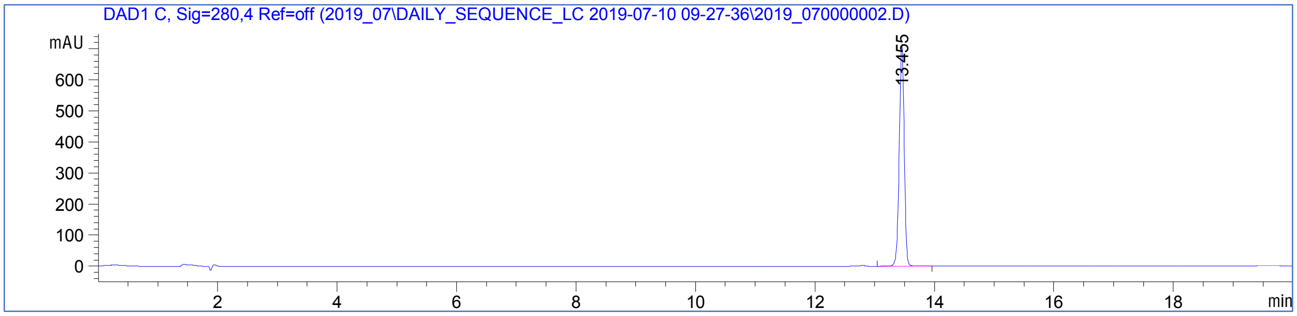

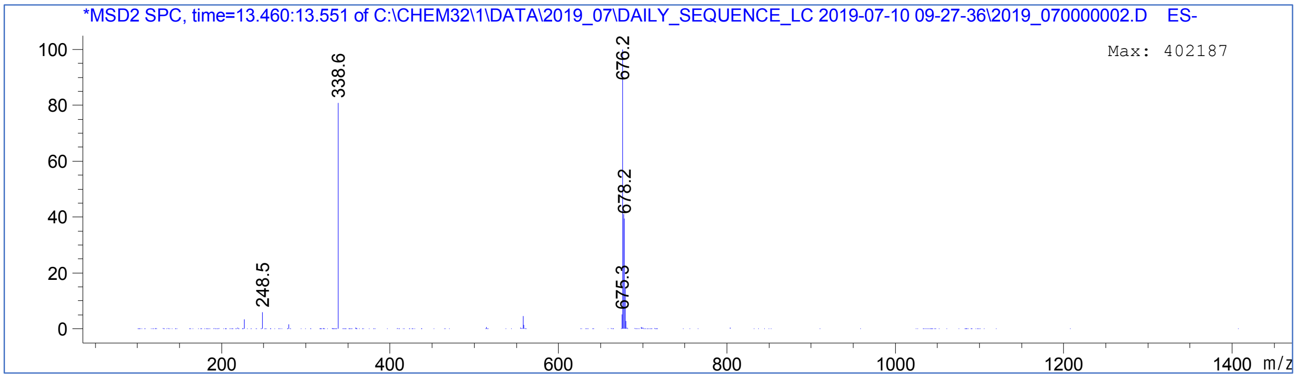

Origin	Bruker BioSpin GmbH
Solvent	MeOD
Temperature	295.2
Pulse Sequence	zg30
Experiment	1D
Number of Scans	16
Acquisition Date	2020-01-21T09:59:00
Spectrometer Frequency	400.13
Spectral Width	8012.8
Lowest Frequency	-1543.2
Nucleus	1H
Acquired Size	32768
Spectral Size	65536

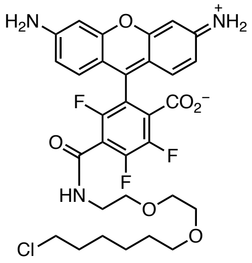

70HTL

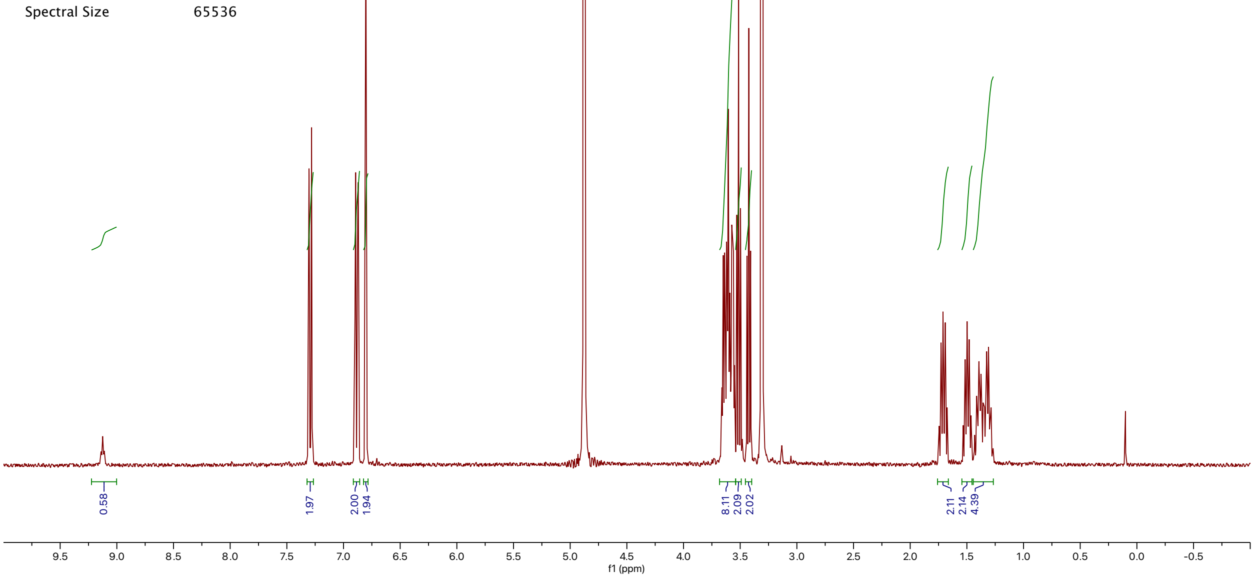

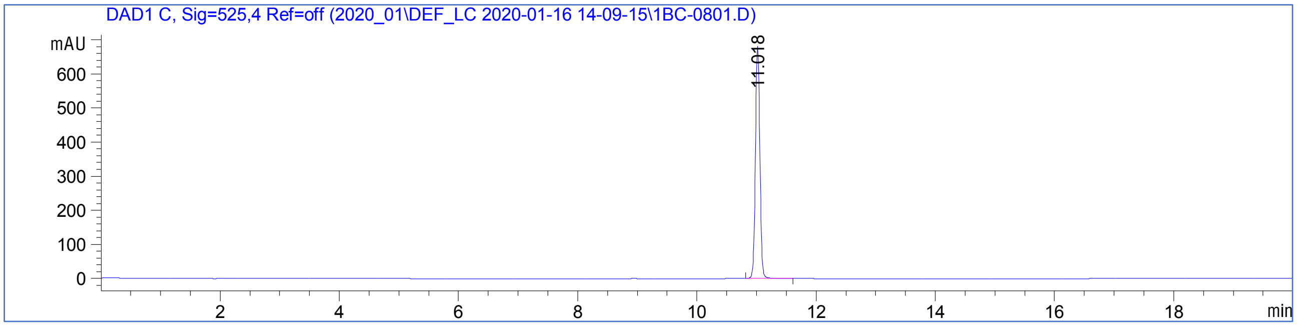

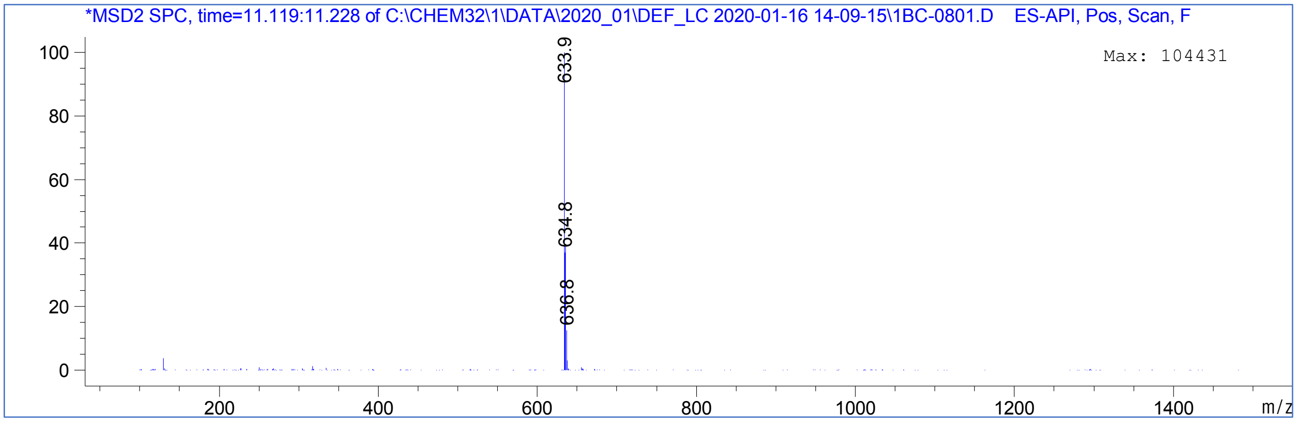

Origin	Bruker BioSpin GmbH
Solvent	MeOD
Temperature	295.2
Pulse Sequence	zg30
Experiment	1D
Number of Scans	16
Acquisition Date	2020-01-27T14:05:00
Spectrometer Frequency	400.13
Spectral Width	8012.8
Lowest Frequency	-1543.3
Nucleus	1H
Acquired Size	32768
Spectral Size	65536

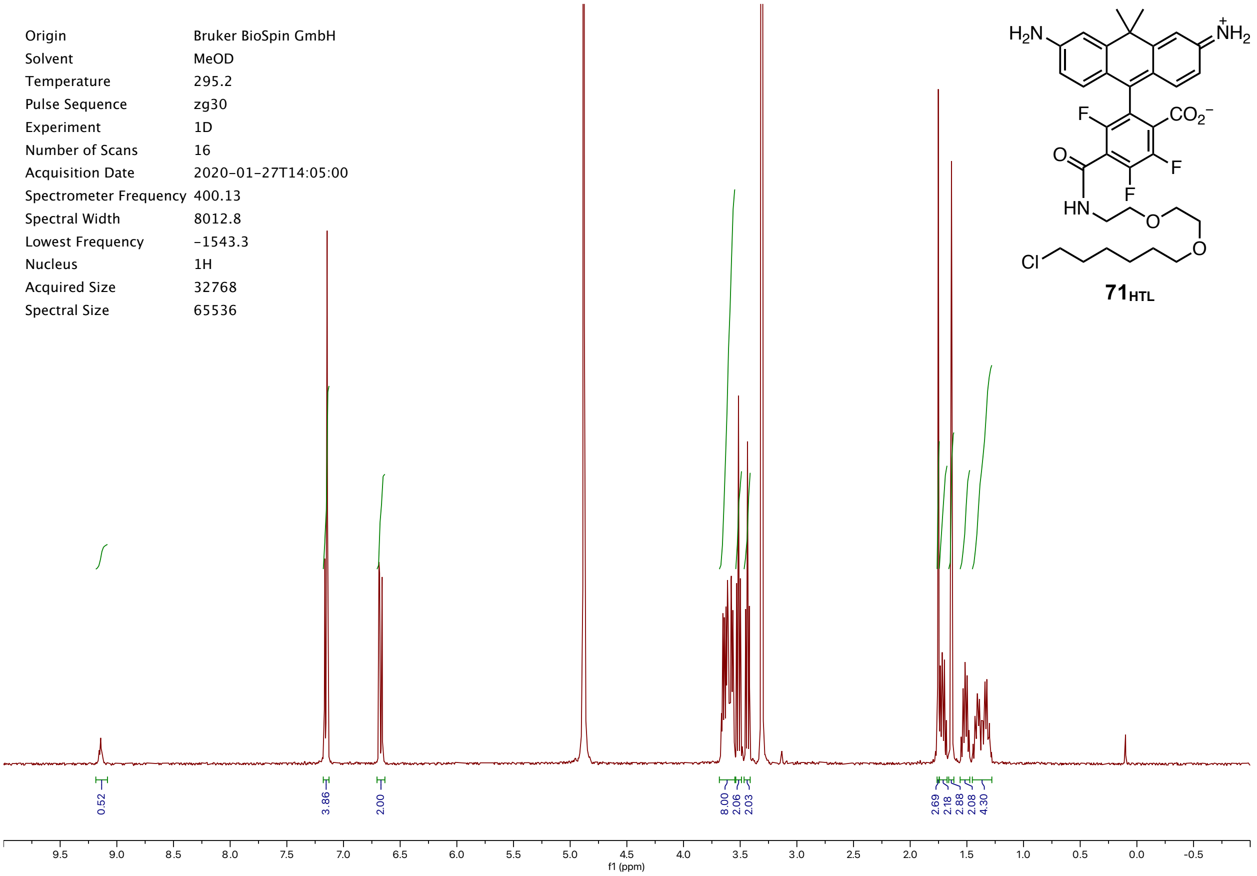

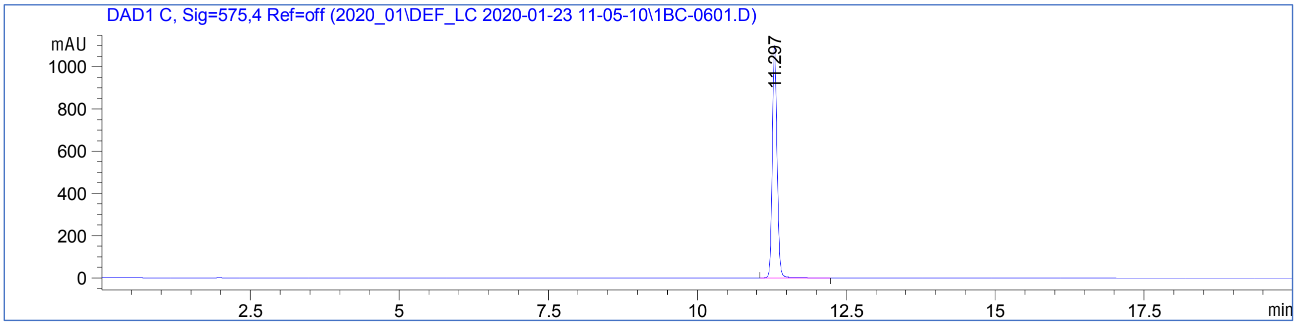

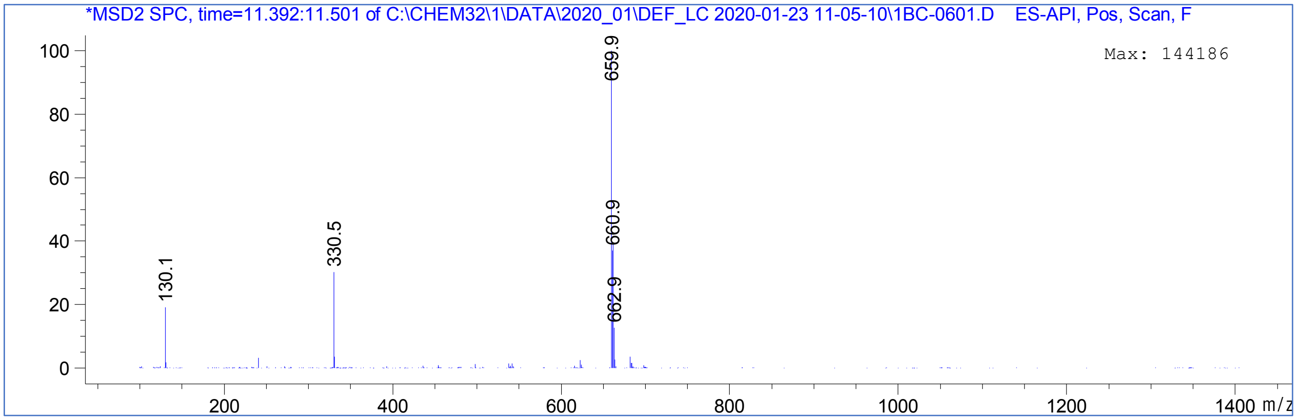

Origin	Bruker BioSpin GmbH
Solvent	MeOD
Temperature	295.4
Pulse Sequence	zg30
Experiment	1D
Number of Scans	16
Acquisition Date	2020-01-27T14:14:00
Spectrometer Frequency	400.13
Spectral Width	8012.8
Lowest Frequency	-1543.3
Nucleus	1H
Acquired Size	32768
Spectral Size	65536

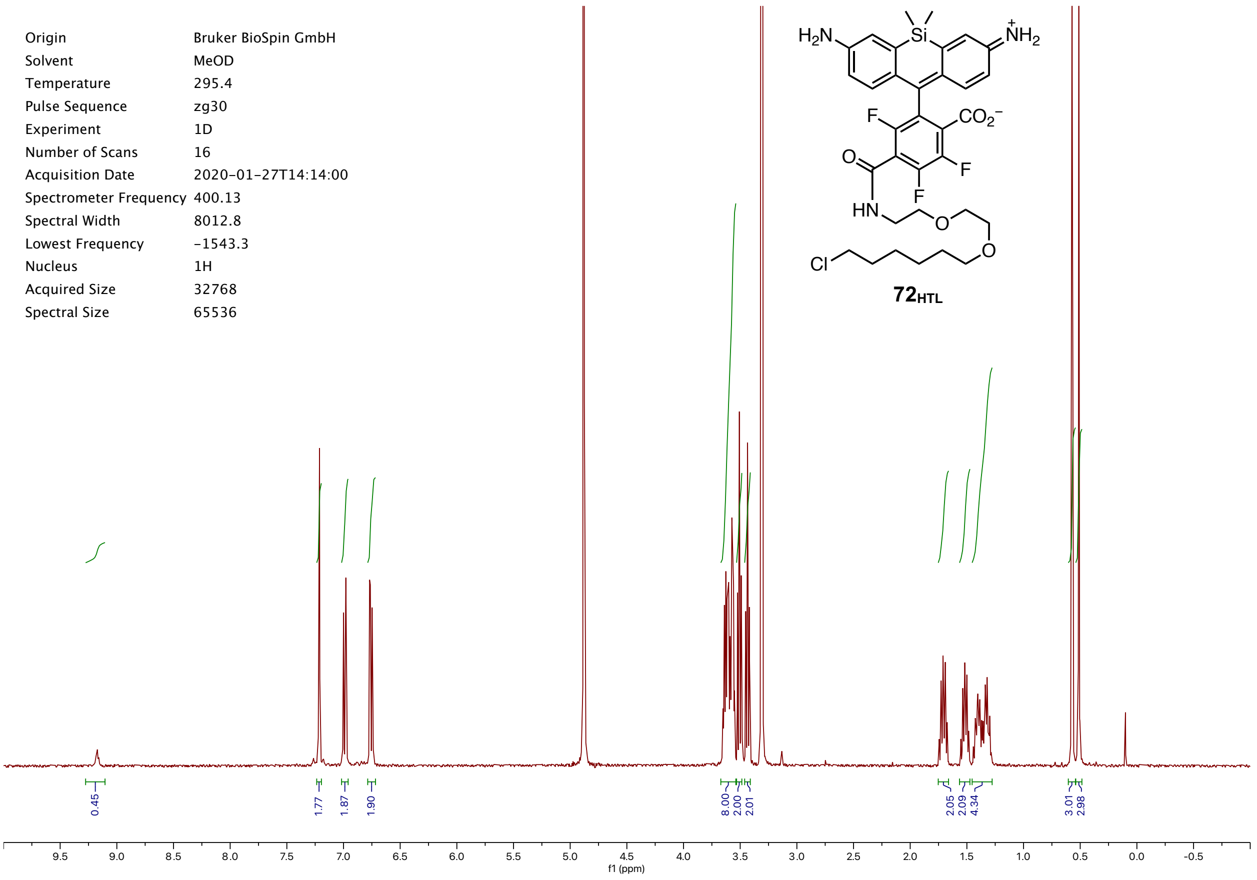

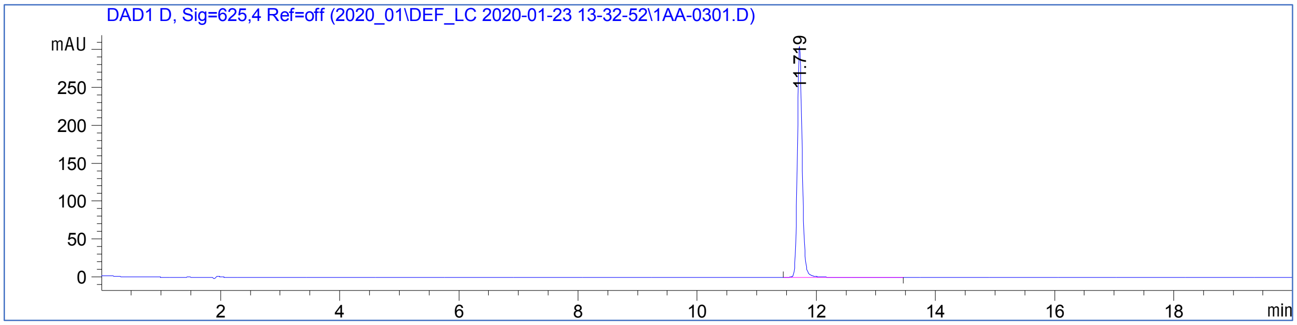

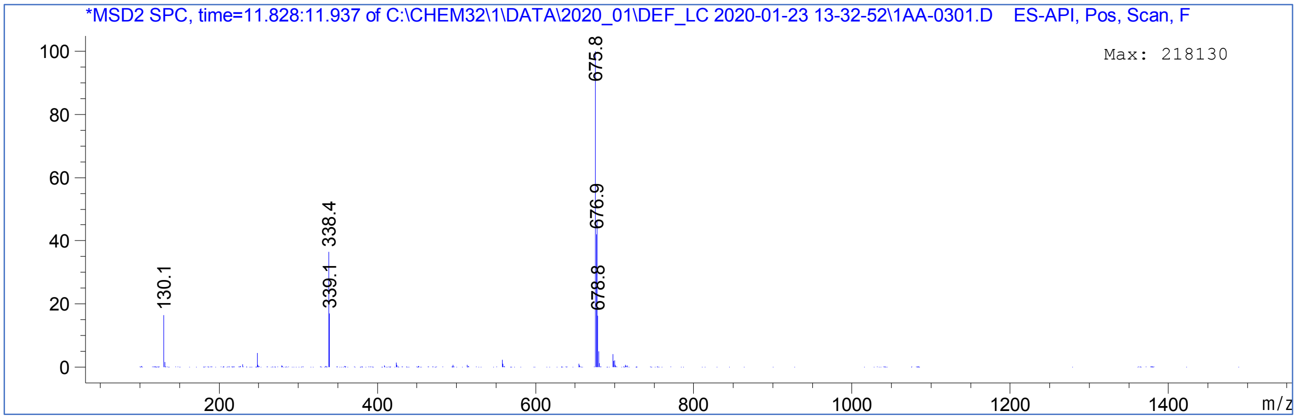

Origin	Bruker BioSpin GmbH
Solvent	MeOD
Temperature	300.0
Pulse Sequence	zg30
Experiment	1D
Number of Scans	16
Acquisition Date	2020-11-23T09:50:00
Spectrometer Frequency	400.13
Spectral Width	8012.8
Lowest Frequency	-1543.2
Nucleus	1H
Acquired Size	32768
Spectral Size	65536

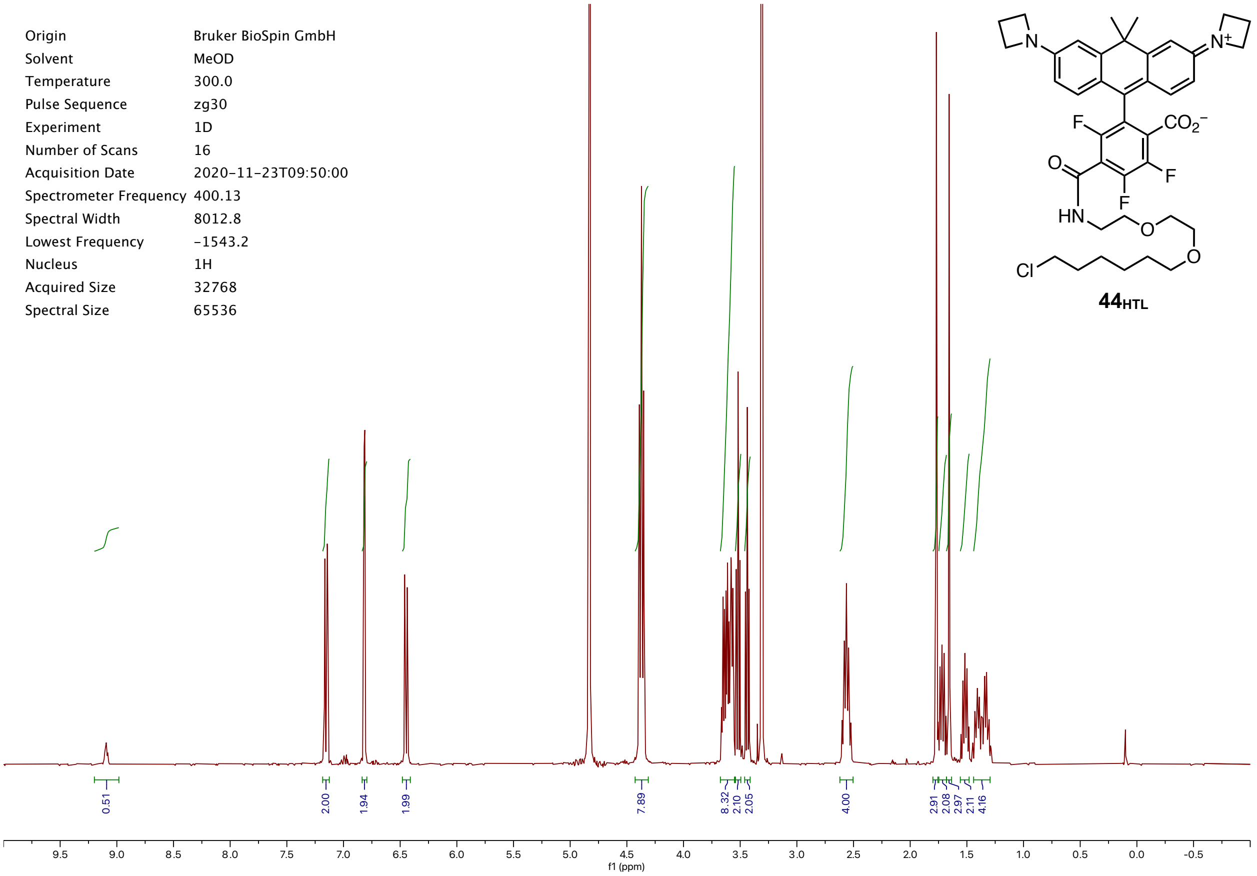

44$_{\mathbf{HTL}}$

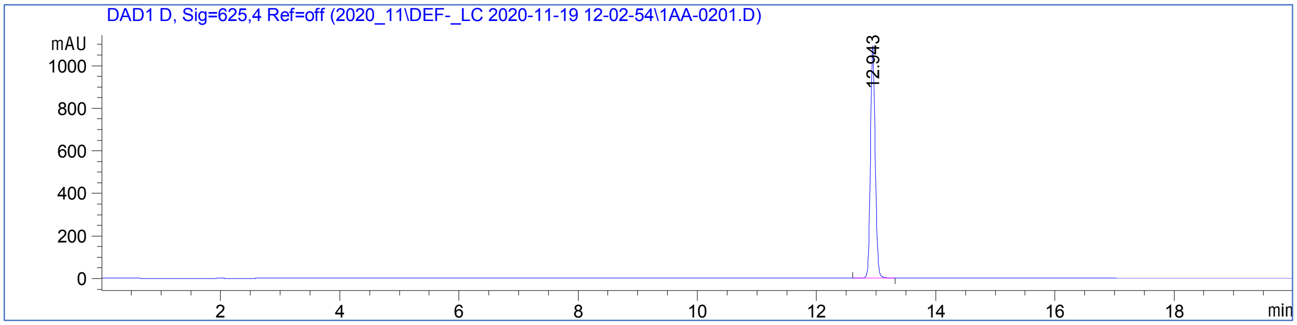

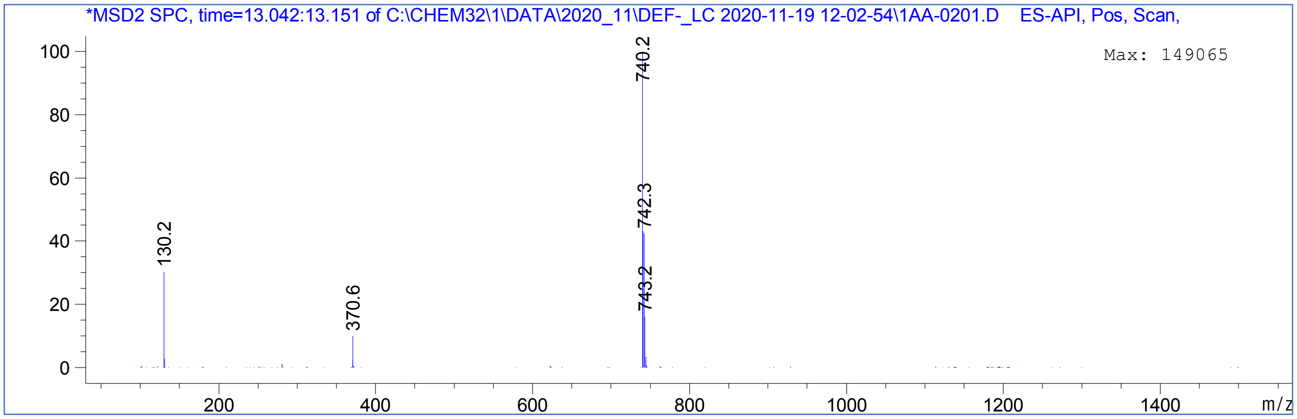

Origin	Bruker BioSpin GmbH
Solvent	MeOD
Temperature	295.6
Pulse Sequence	zg30
Experiment	1D
Number of Scans	16
Acquisition Date	2021-04-26T09:43:00
Spectrometer Frequency	400.13
Spectral Width	8012.8
Lowest Frequency	-1543.3
Nucleus	1H
Acquired Size	32768
Spectral Size	65536

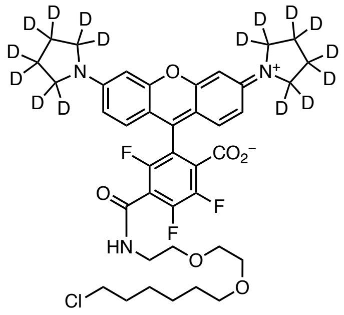

8HTL

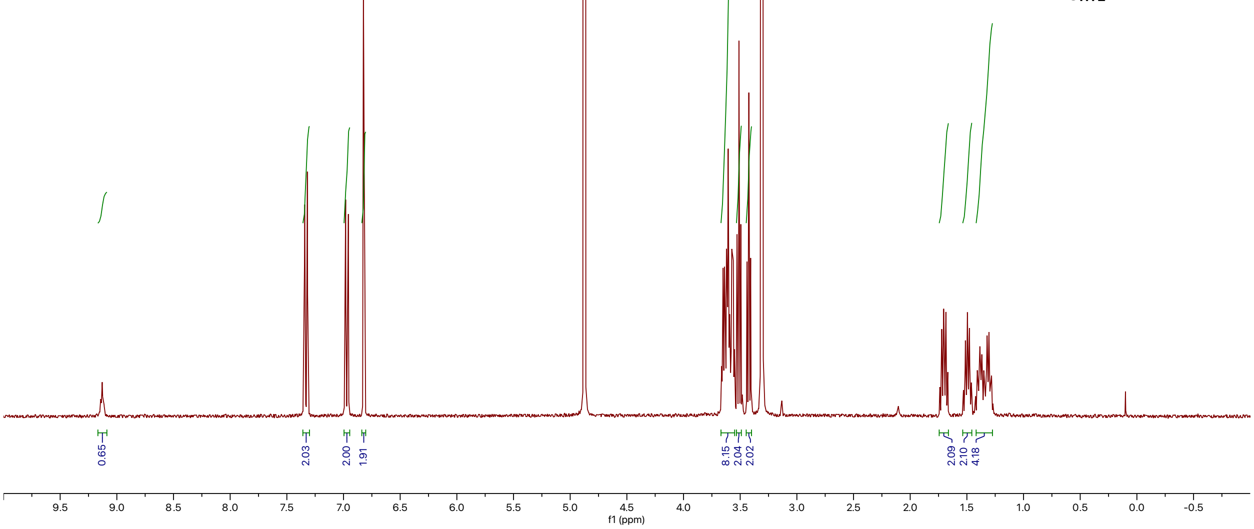

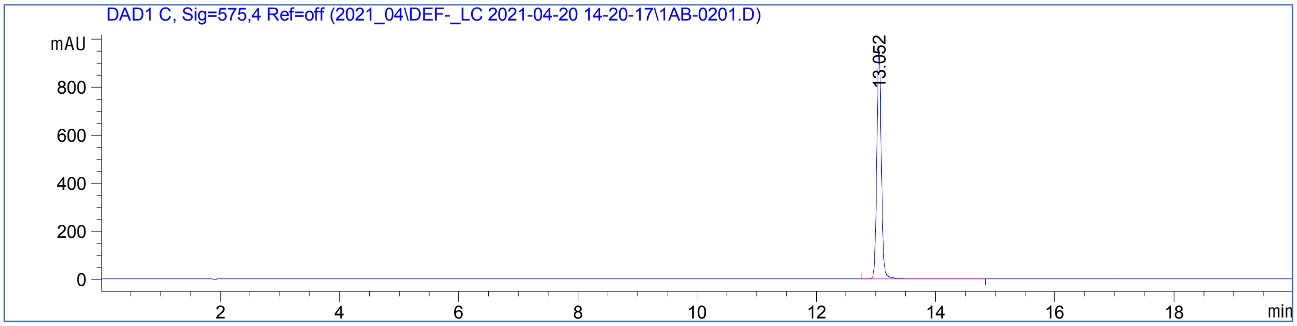

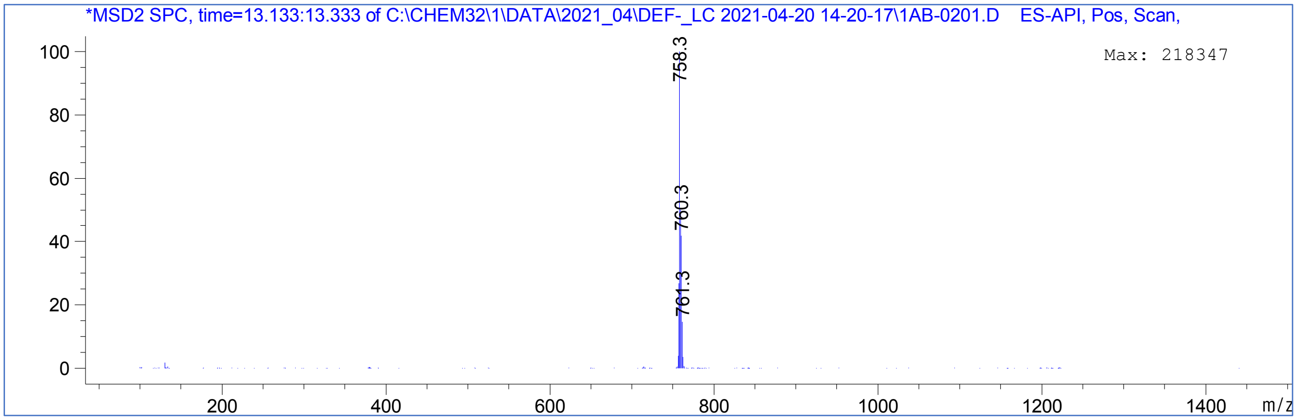

Origin	Bruker BioSpin GmbH
Solvent	MeOD
Temperature	295.5
Pulse Sequence	zg30
Experiment	1D
Number of Scans	16
Acquisition Date	2021-09-03T14:37:00
Spectrometer Frequency	400.13
Spectral Width	8012.8
Lowest Frequency	-1543.2
Nucleus	1H
Acquired Size	32768
Spectral Size	65536

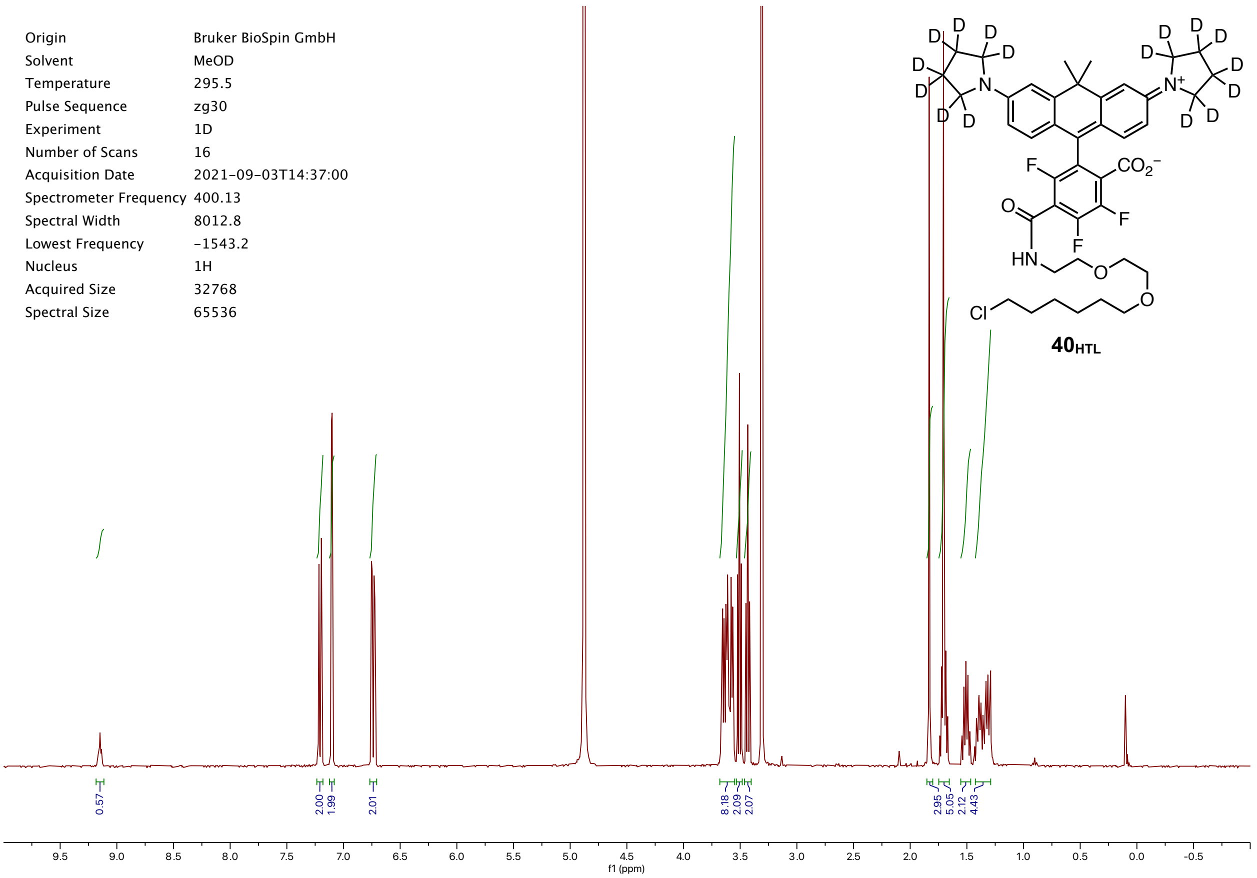

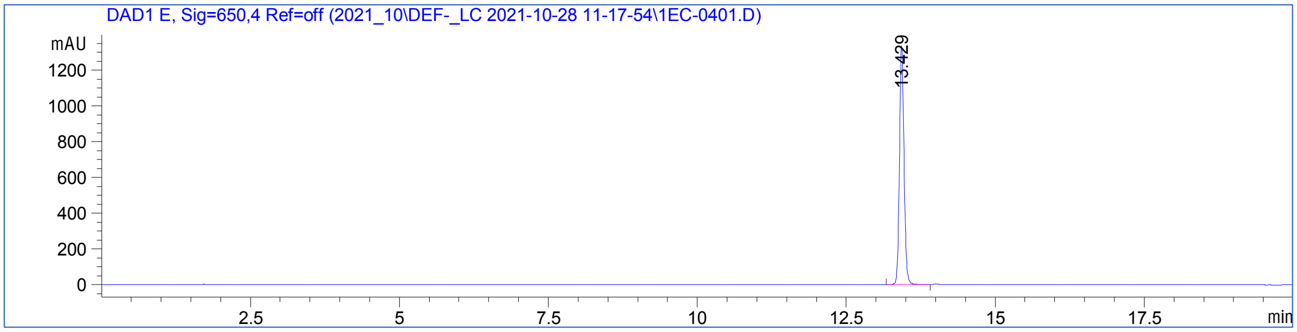

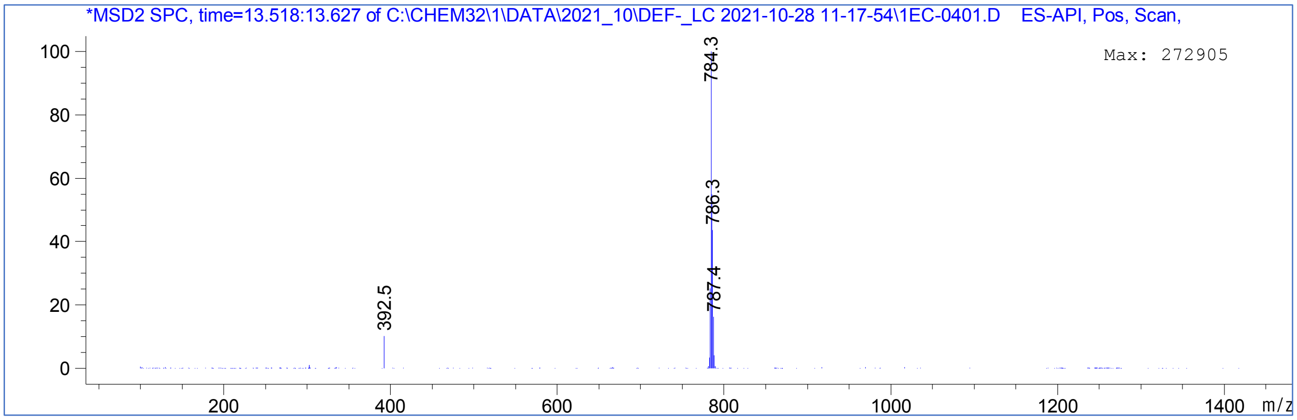

Origin	Bruker BioSpin GmbH
Solvent	MeOD
Temperature	295.3
Pulse Sequence	zg30
Experiment	1D
Number of Scans	16
Acquisition Date	2020-01-09T10:52:00
Spectrometer Frequency	400.13
Spectral Width	8012.8
Lowest Frequency	-1543.3
Nucleus	1H
Acquired Size	32768
Spectral Size	65536

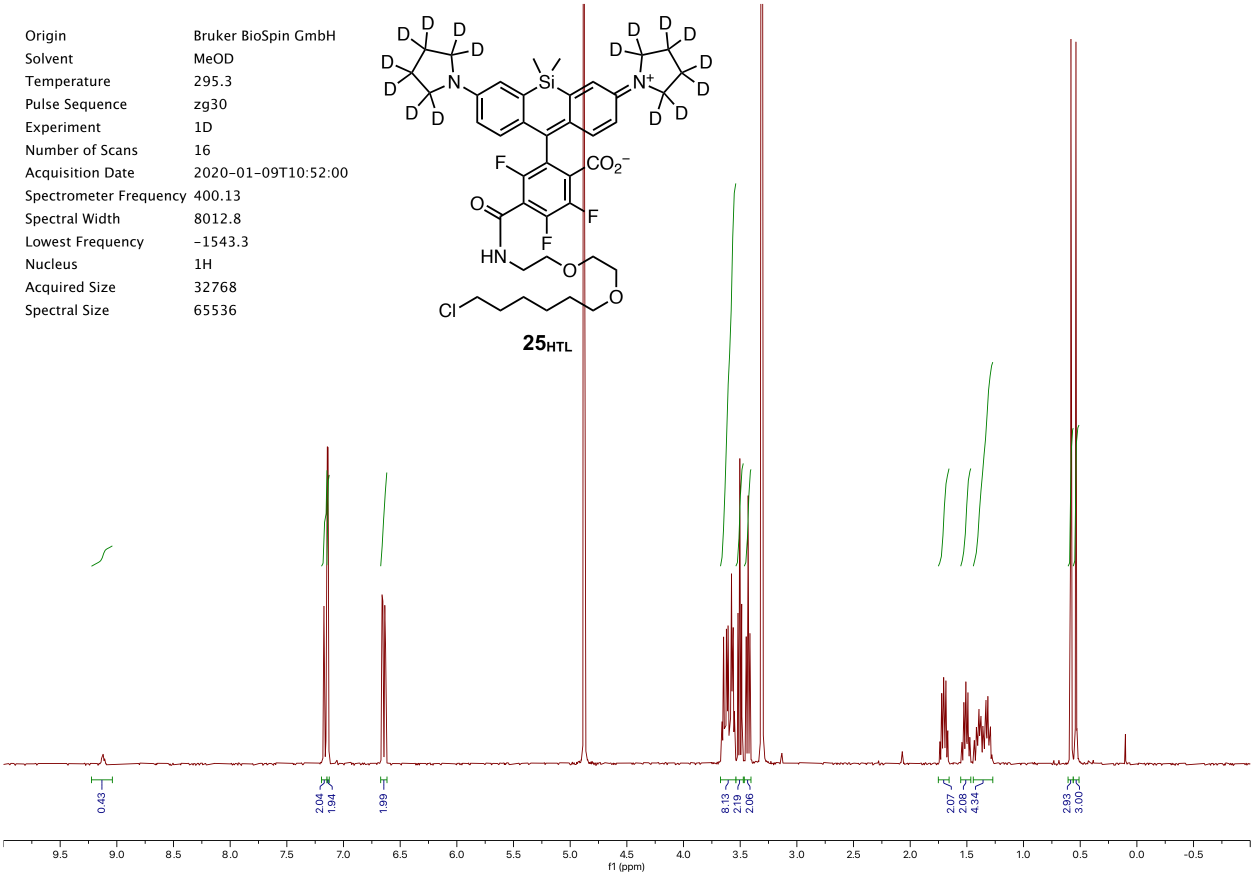

25HTL

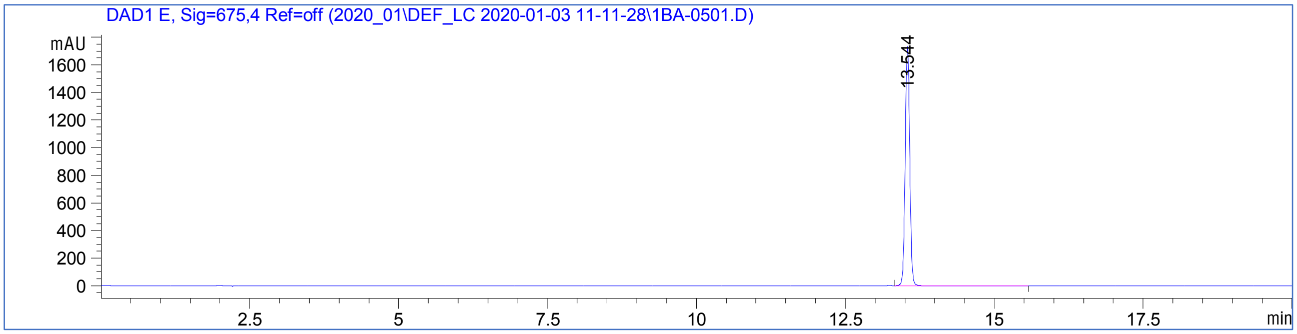

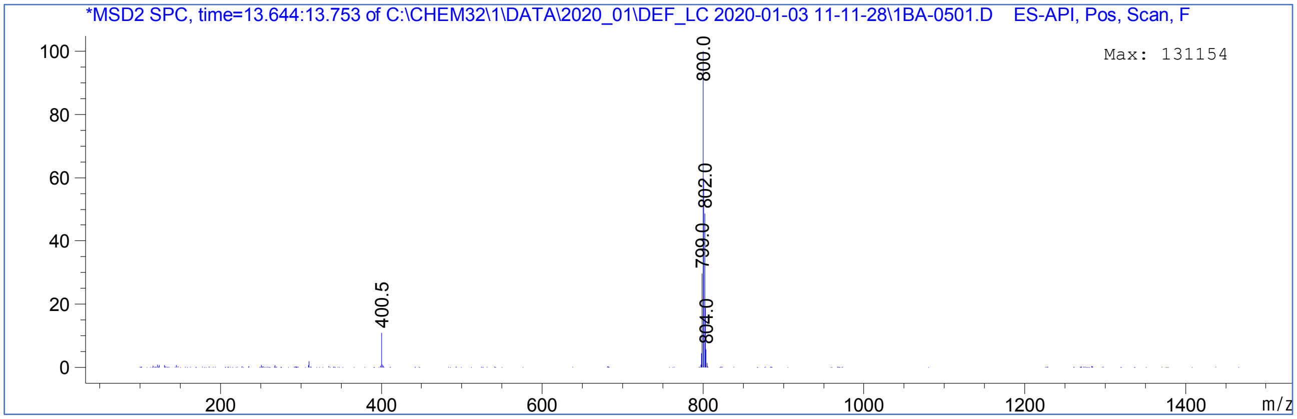

Origin	Bruker BioSpin GmbH
Solvent	MeOD
Temperature	300.0
Pulse Sequence	zg30
Experiment	1D
Number of Scans	128
Acquisition Date	2020-11-24T10:34:00
Spectrometer Frequency	400.13
Spectral Width	8012.8
Lowest Frequency	-1543.2
Nucleus	1H
Acquired Size	32768
Spectral Size	65536

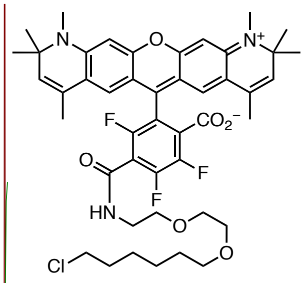

11$_{HTL}$

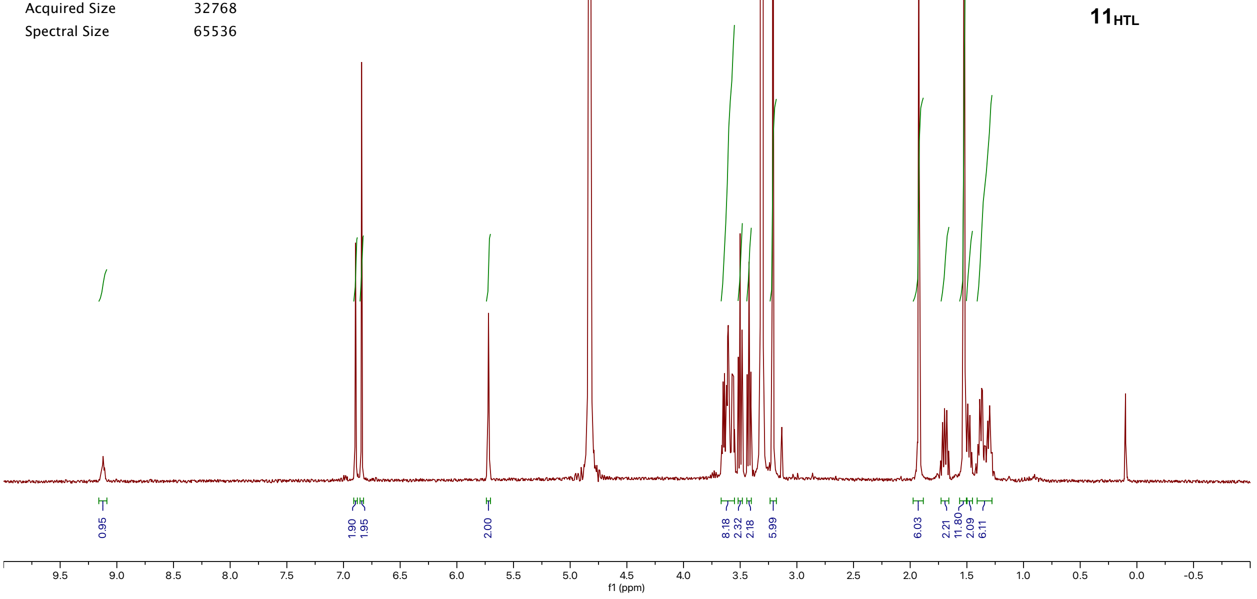

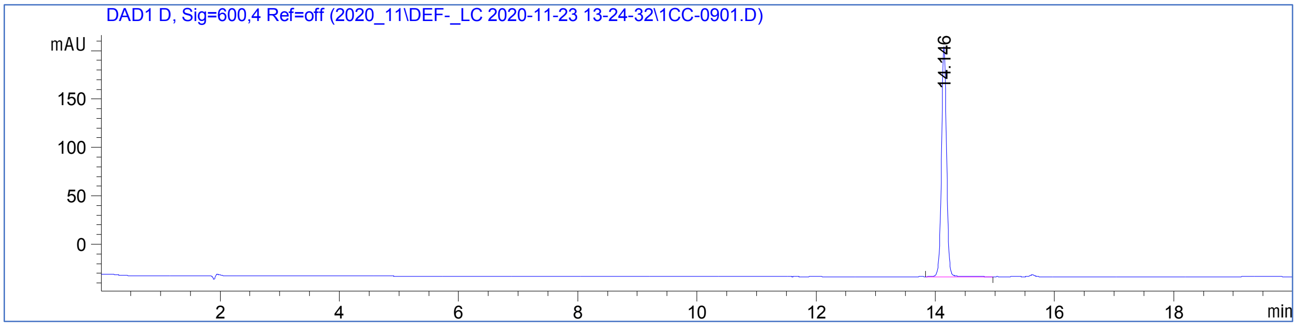

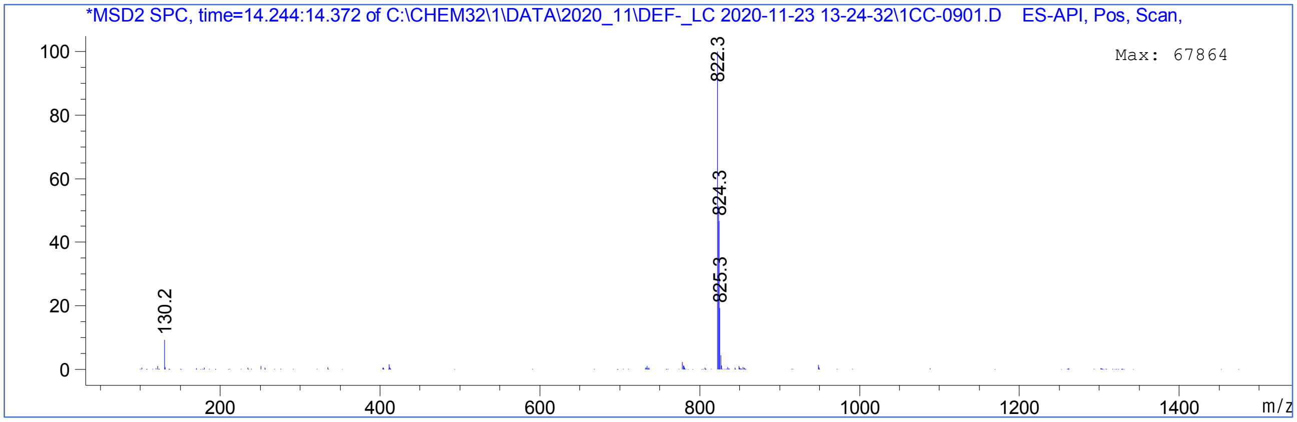

Origin	Bruker BioSpin GmbH
Solvent	MeOD
Temperature	300.0
Pulse Sequence	zg30
Experiment	1D
Number of Scans	128
Acquisition Date	2020-11-23T10:53:00
Spectrometer Frequency	400.13
Spectral Width	8012.8
Lowest Frequency	-1543.2
Nucleus	1H
Acquired Size	32768
Spectral Size	65536

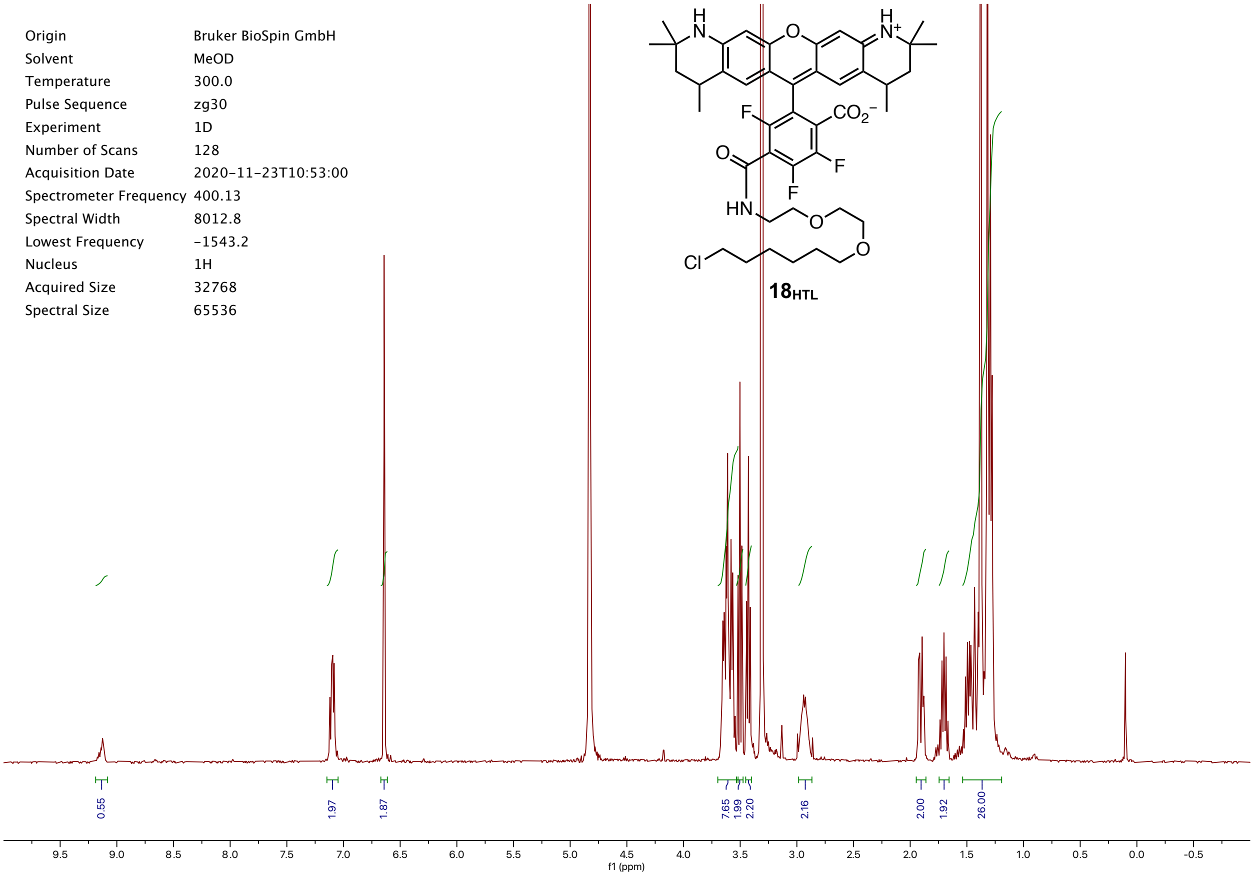

$\mathbf{18_{HTL}}$

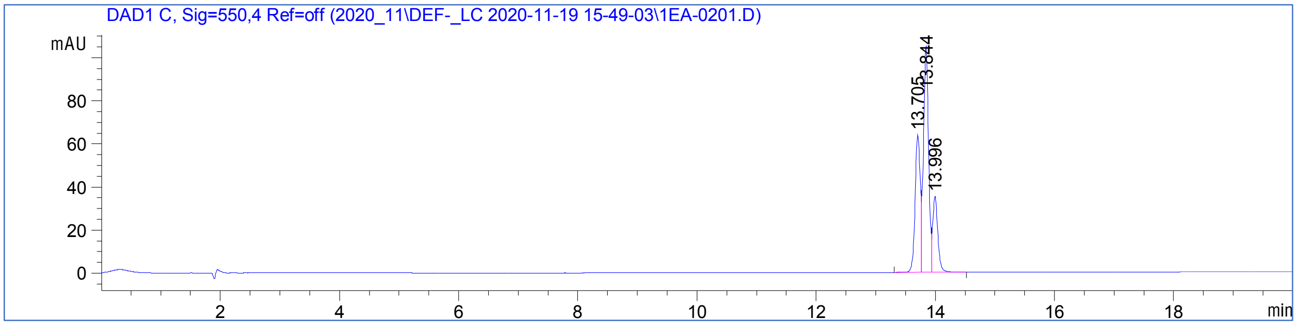

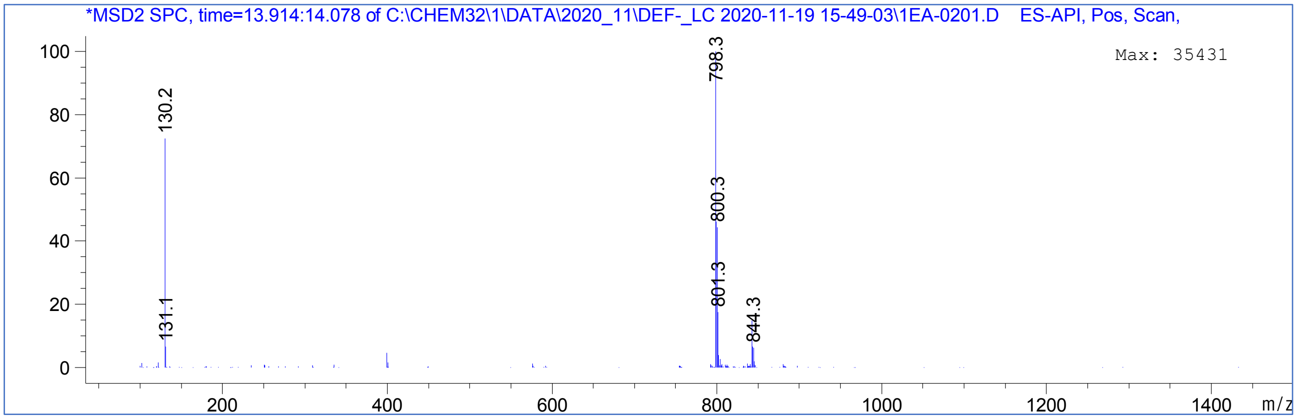

Origin	Bruker BioSpin GmbH
Solvent	CDCl3
Temperature	300.0
Pulse Sequence	zg30
Experiment	1D
Number of Scans	16
Acquisition Date	2021-01-21T14:21:00
Spectrometer Frequency	400.13
Spectral Width	8012.8
Lowest Frequency	-1545.6
Nucleus	1H
Acquired Size	32768
Spectral Size	65536

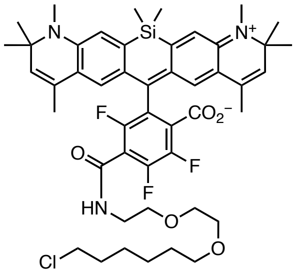

38$_{HTL}$

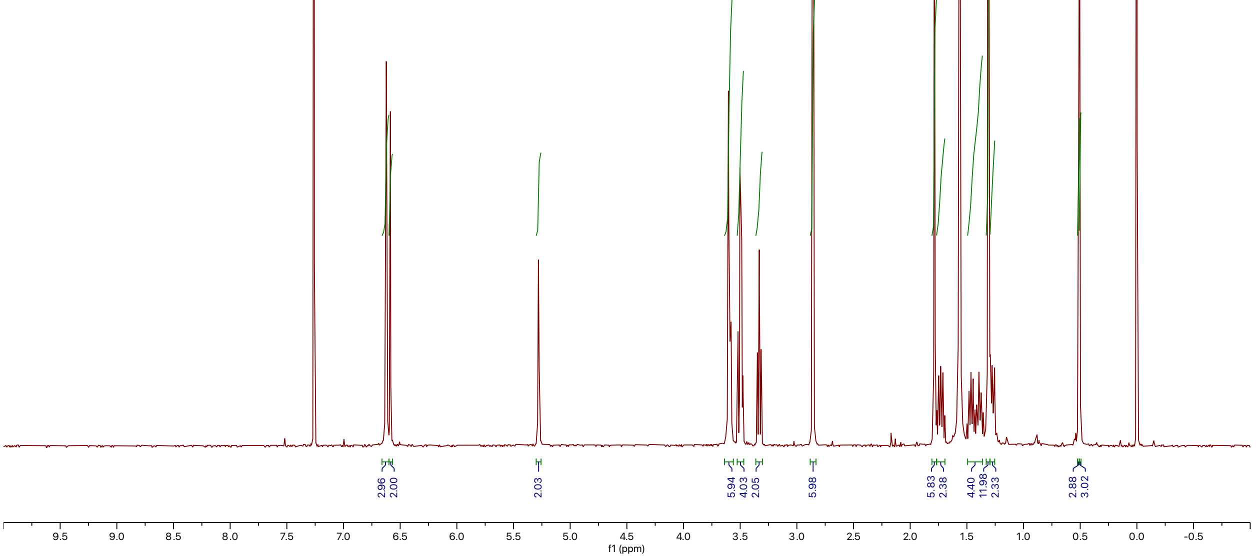

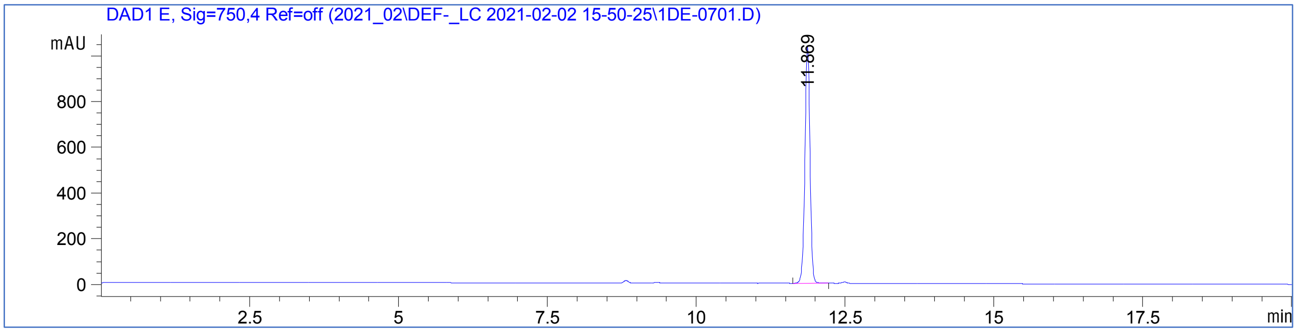

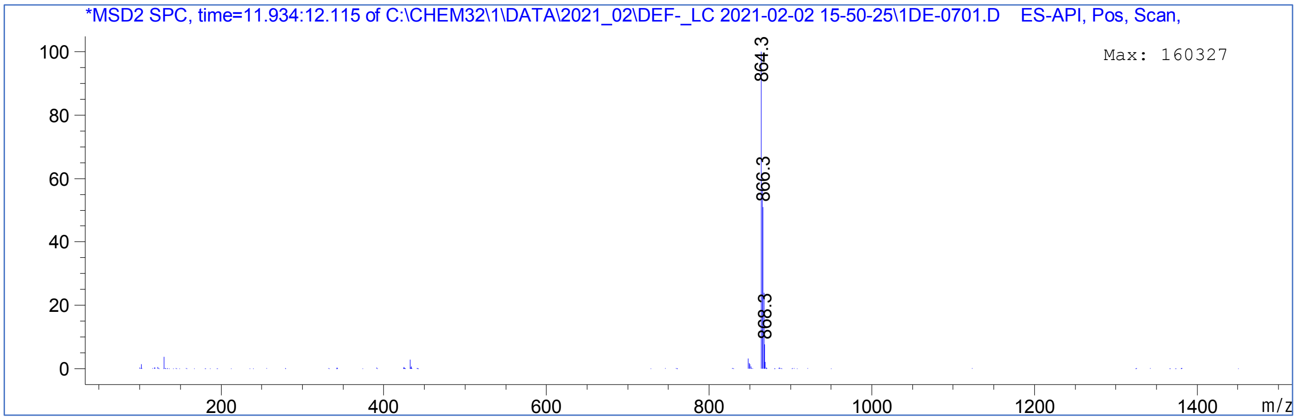

Origin	Bruker BioSpin GmbH
Solvent	MeOD
Temperature	300.0
Pulse Sequence	zg30
Experiment	1D
Number of Scans	16
Acquisition Date	2021-12-10T11:02:00
Spectrometer Frequency	400.13
Spectral Width	8012.8
Lowest Frequency	-1543.1
Nucleus	1H
Acquired Size	32768
Spectral Size	65536

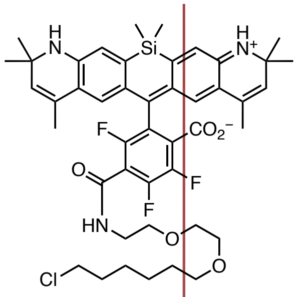

36HTL

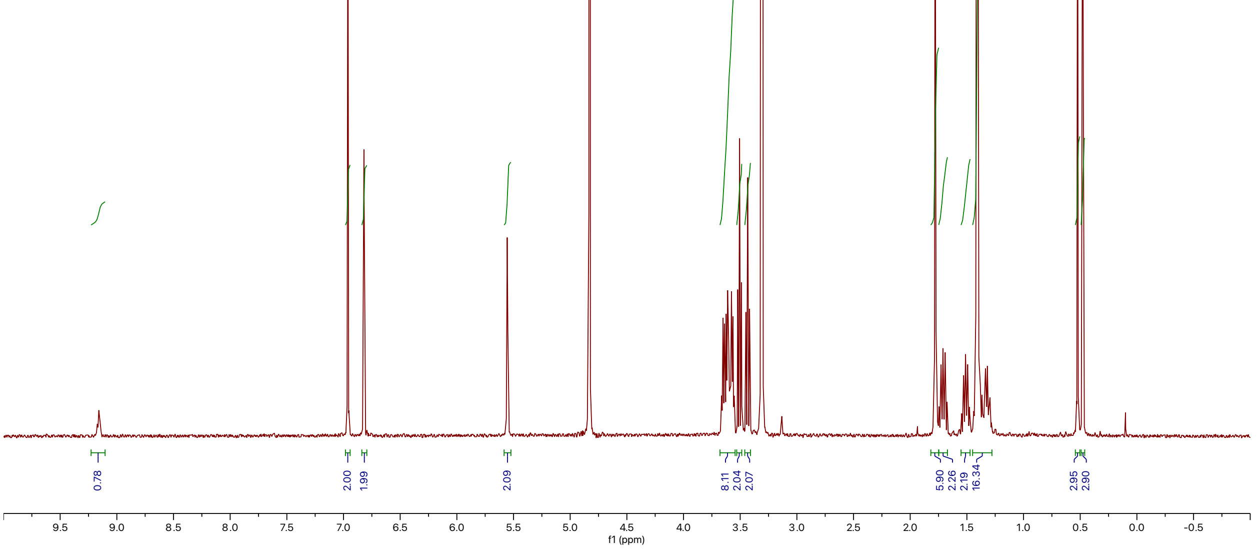

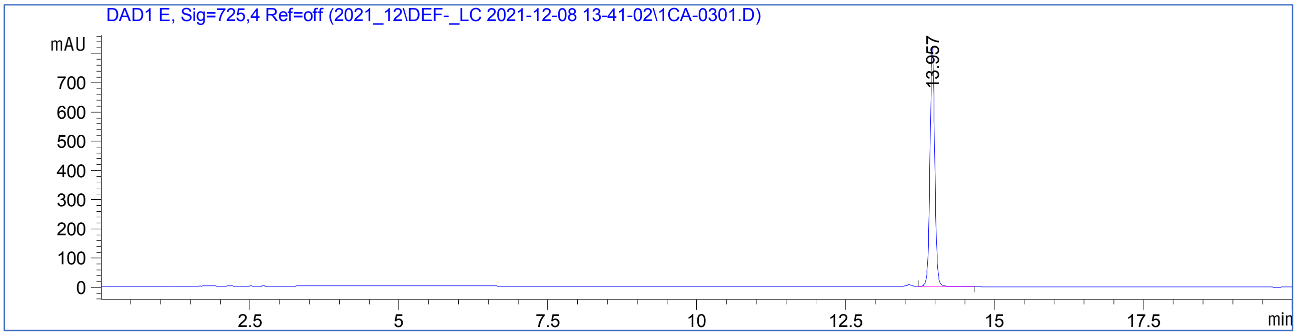

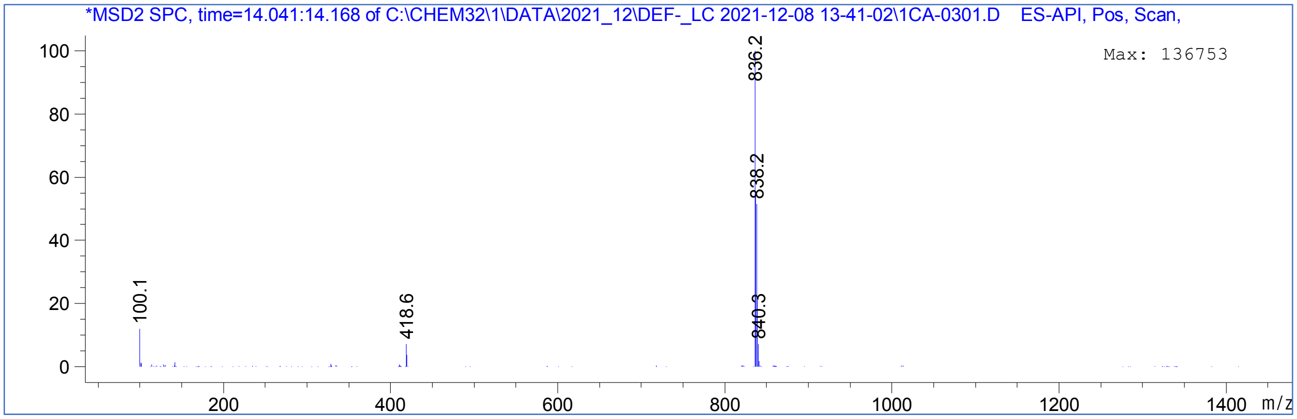

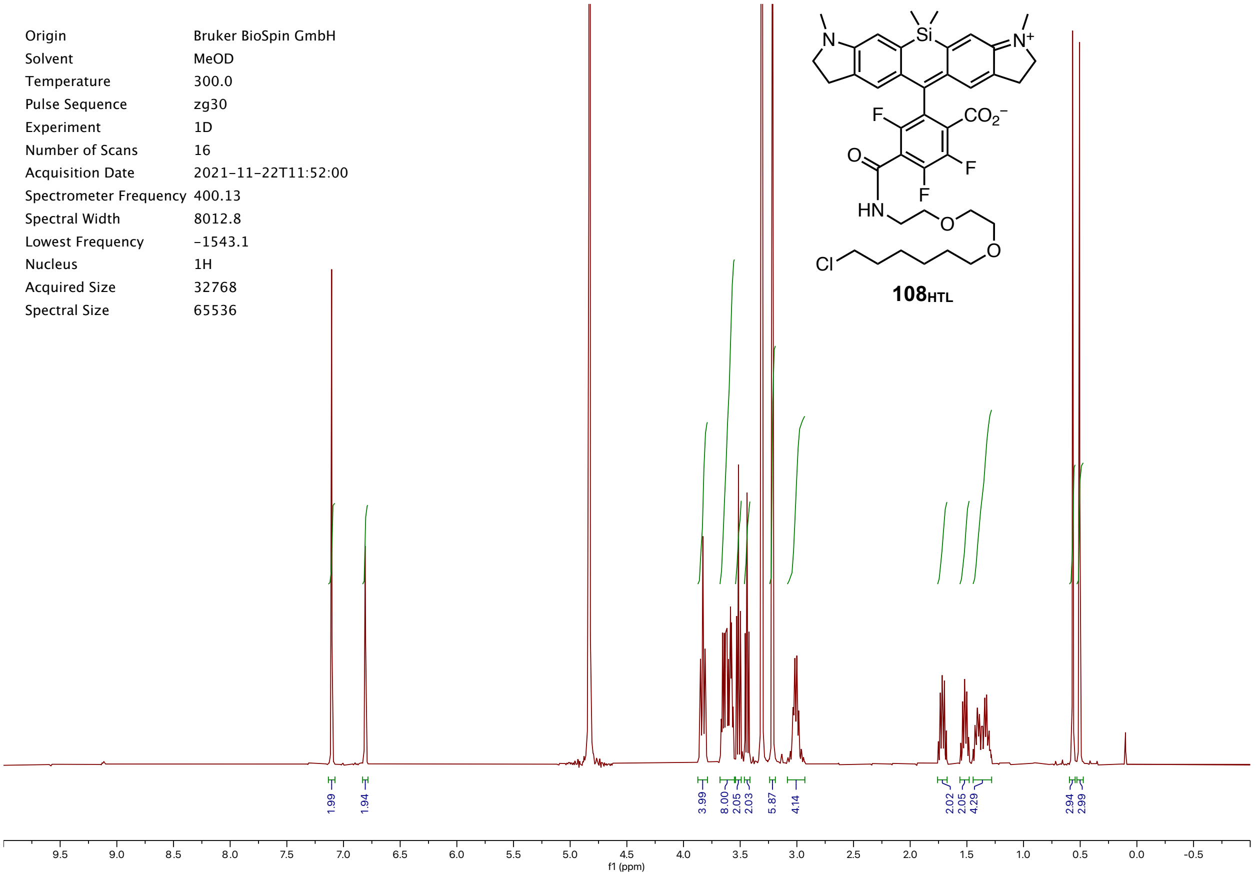

Origin Bruker BioSpin GmbH
Solvent MeOD
Temperature 300.0
Pulse Sequence zg30
Experiment 1D
Number of Scans 16
Acquisition Date 2021-11-22T11:52:00
Spectrometer Frequency 400.13
Spectral Width 8012.8
Lowest Frequency -1543.1
Nucleus 1H
Acquired Size 32768
Spectral Size 65536
108HTL
1.99
1.94
3.99
8.00
2.05
2.03
5.87
4.14
2.02
2.05
4.29
2.94
2.99
f1 (ppm)

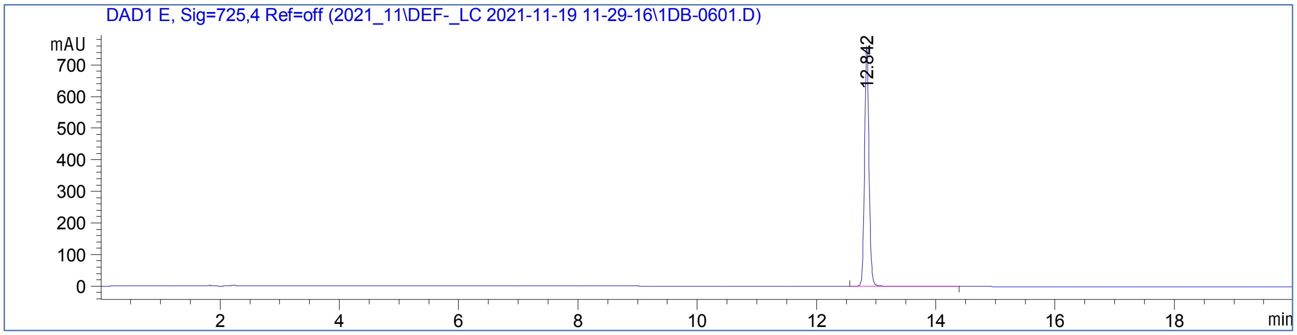

DAD1 E, Sig=725,4 Ref=off (2021_11\DEF-_LC 2021-11-19 11-29-16\1DB-0601.D)
mAU
12.842
min

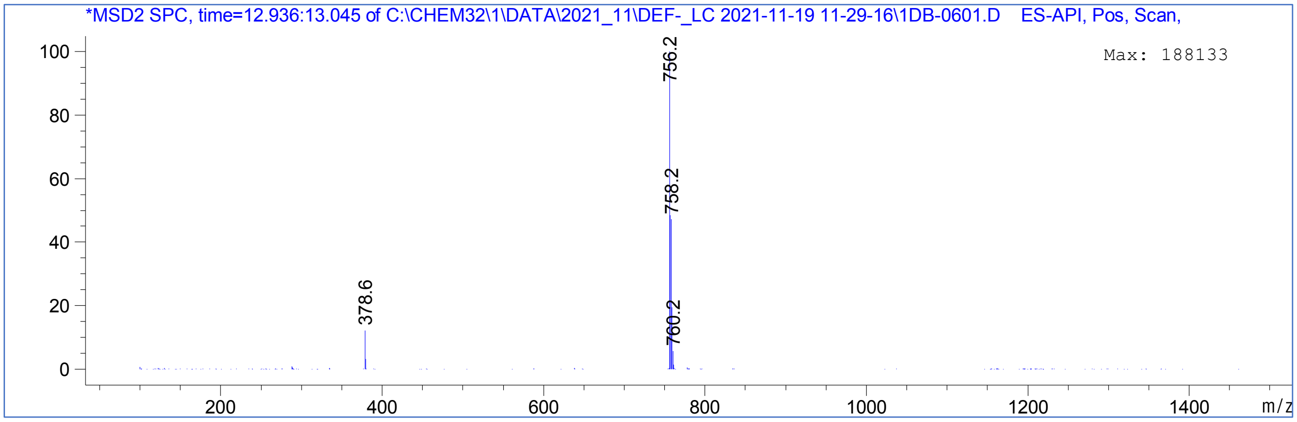

*MSD2 SPC, time=12.936:13.045 of C:\CHEM32\1\DATA\2021_11\DEF-_LC 2021-11-19 11-29-16\1DB-0601.D ES-API, Pos, Scan,
Max: 188133
756.2
758.2
760.2
378.6
m/z

Origin	Bruker BioSpin GmbH
Solvent	MeOD
Temperature	299.0
Pulse Sequence	zg30
Experiment	1D
Number of Scans	16
Acquisition Date	2022-11-15T11:58:00
Spectrometer Frequency	400.13
Spectral Width	8012.8
Lowest Frequency	-1543.2
Nucleus	1H
Acquired Size	32768
Spectral Size	65536

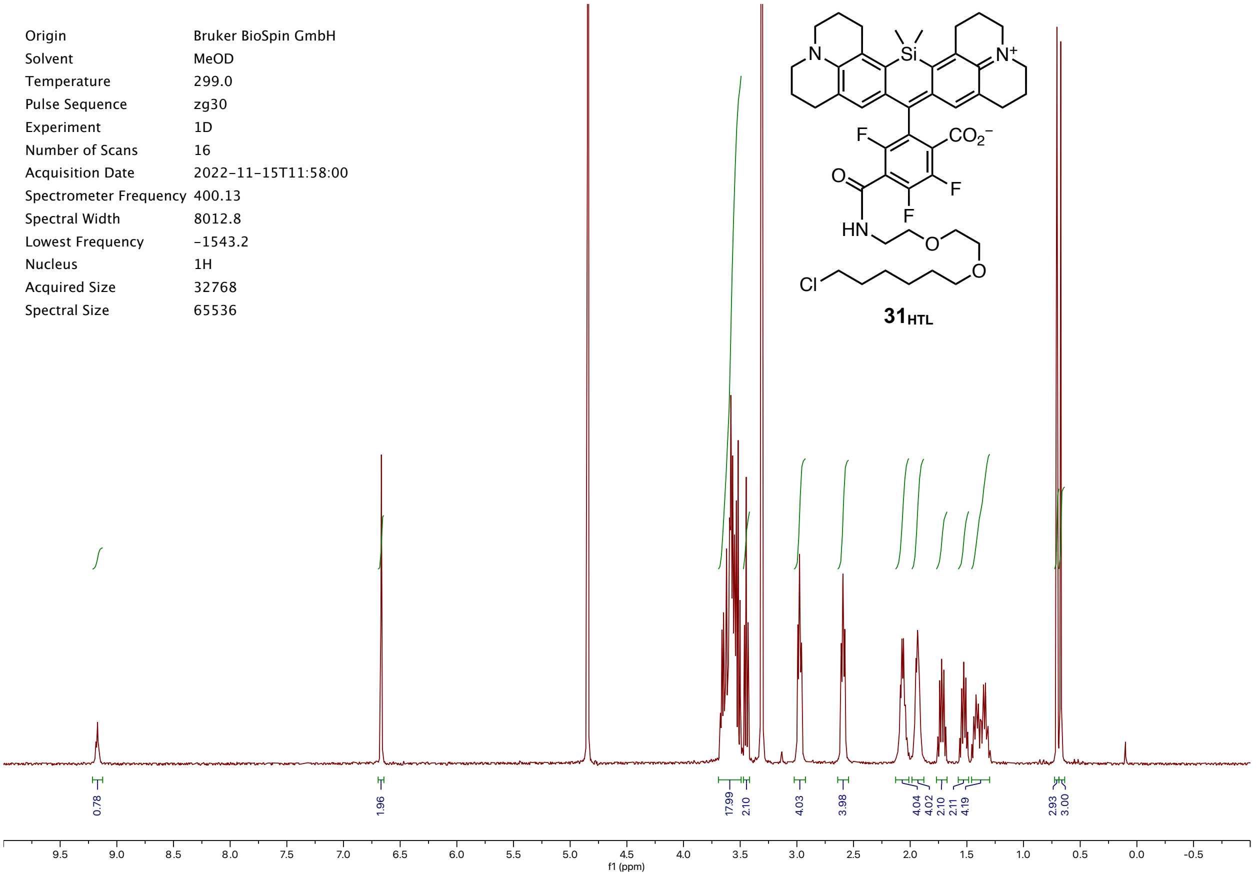

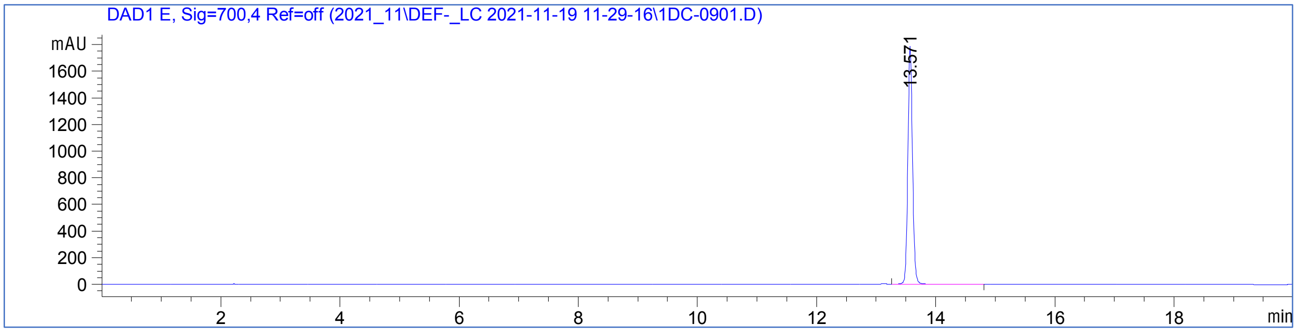

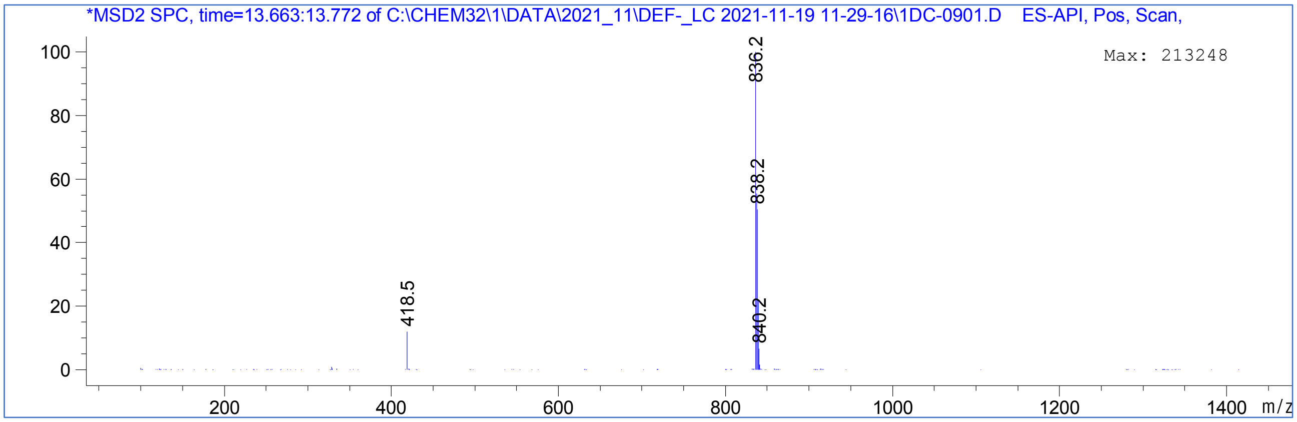

Origin	Bruker BioSpin GmbH
Solvent	MeOD
Temperature	300.0
Pulse Sequence	zg30
Experiment	1D
Number of Scans	16
Acquisition Date	2021-11-29T11:55:00
Spectrometer Frequency	400.13
Spectral Width	8012.8
Lowest Frequency	-1543.1
Nucleus	1H
Acquired Size	32768
Spectral Size	65536

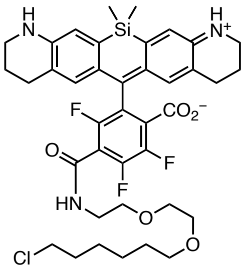

29HTL

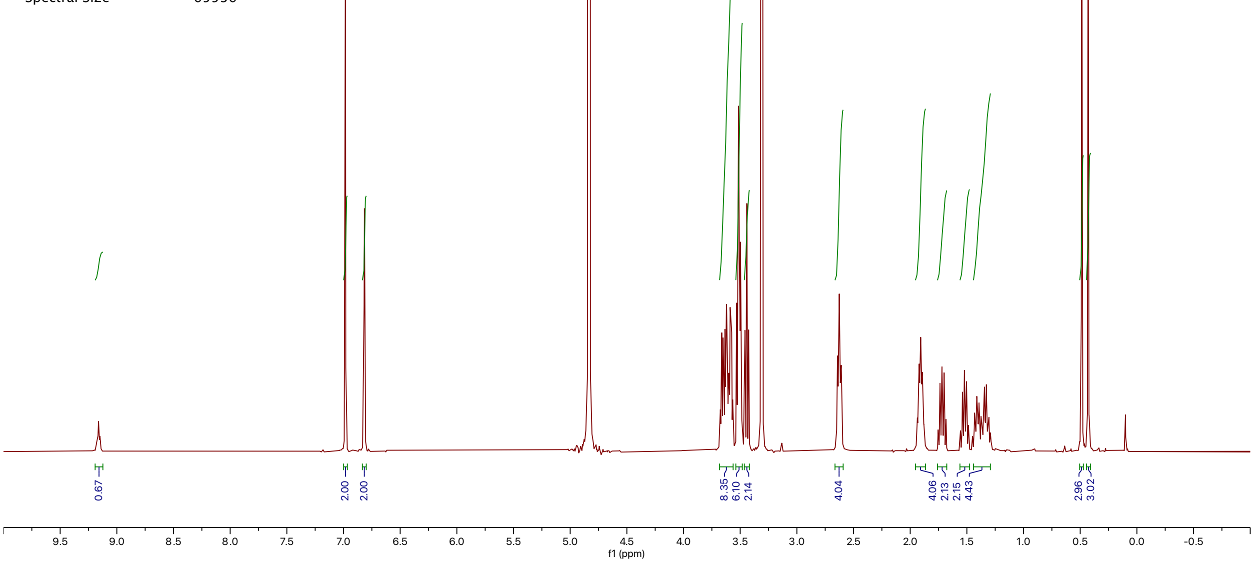

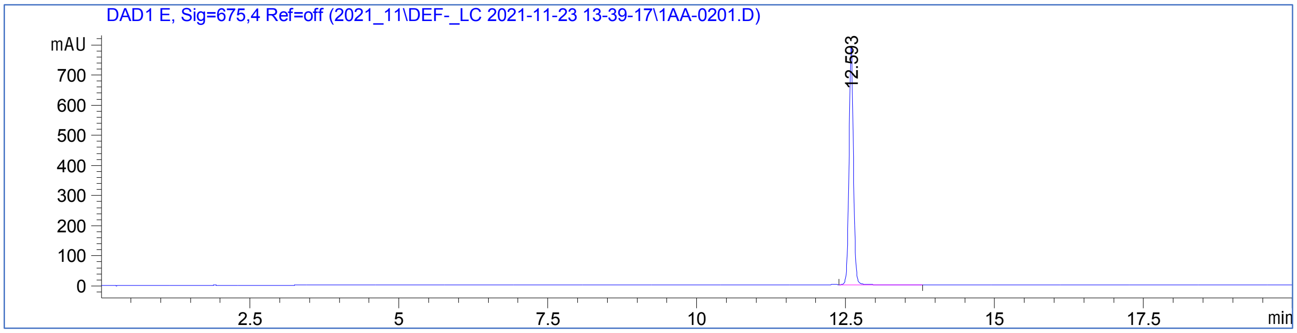

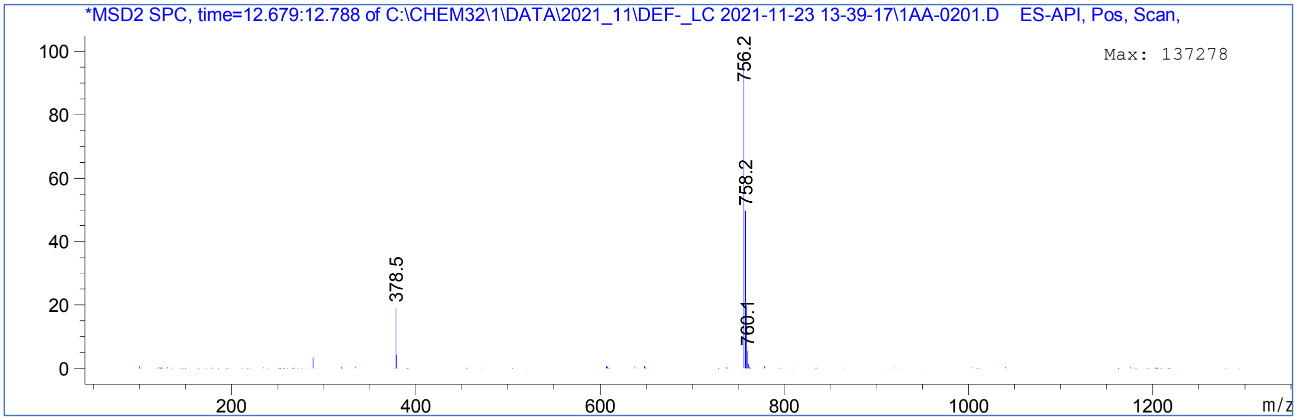

Origin	Bruker BioSpin GmbH
Solvent	MeOD
Temperature	300.0
Pulse Sequence	zg30
Experiment	1D
Number of Scans	16
Acquisition Date	2022-05-09T12:19:00
Spectrometer Frequency	400.13
Spectral Width	8012.8
Lowest Frequency	-1543.1
Nucleus	1H
Acquired Size	32768
Spectral Size	65536

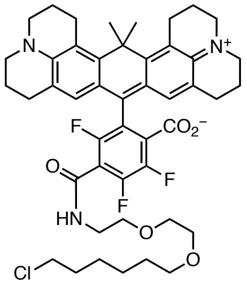

57HTL

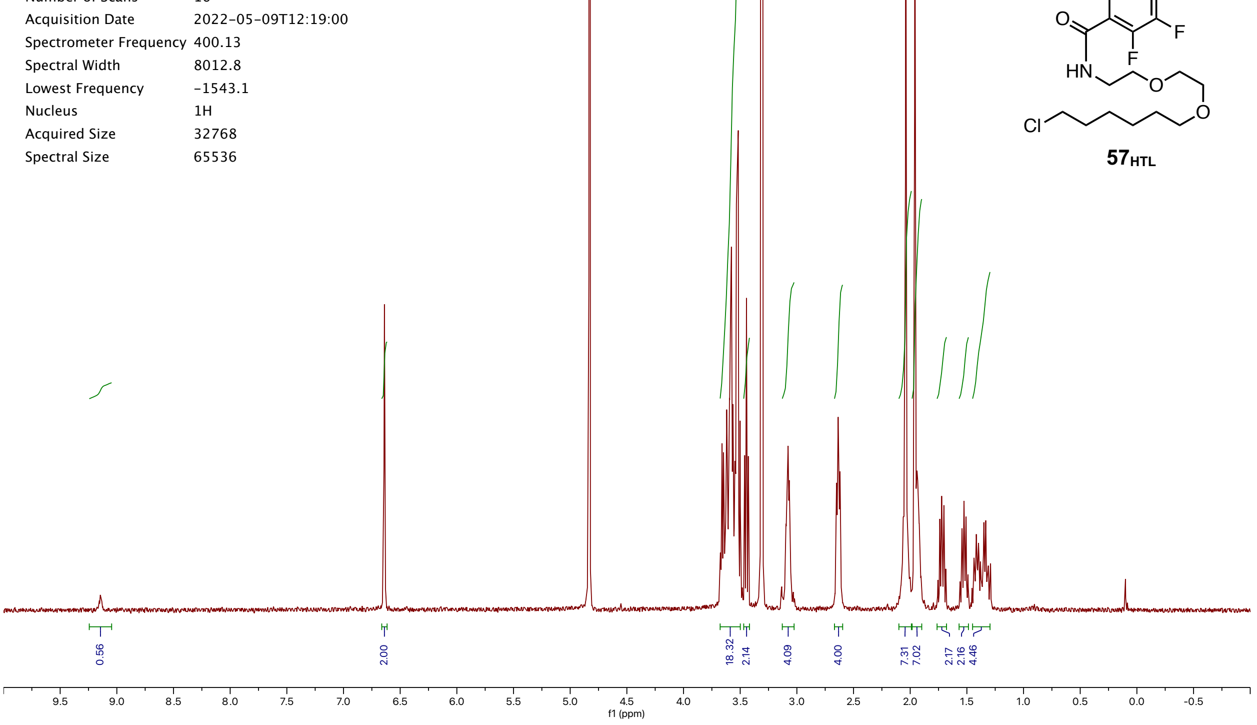

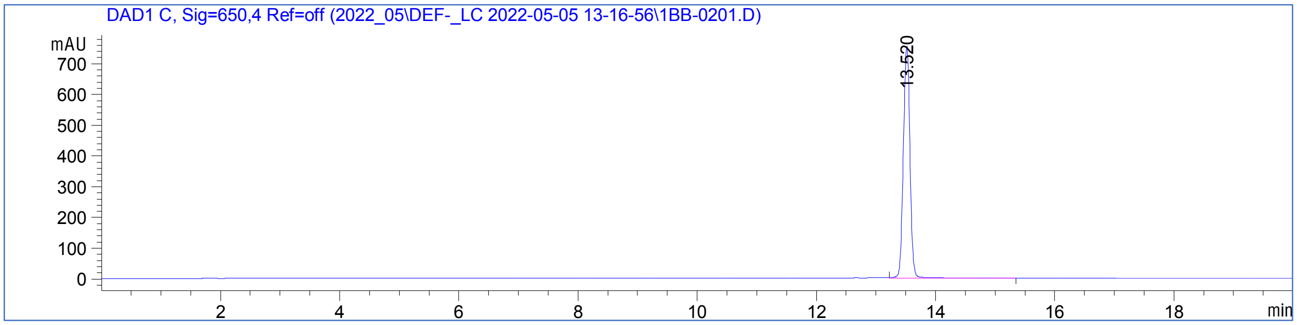

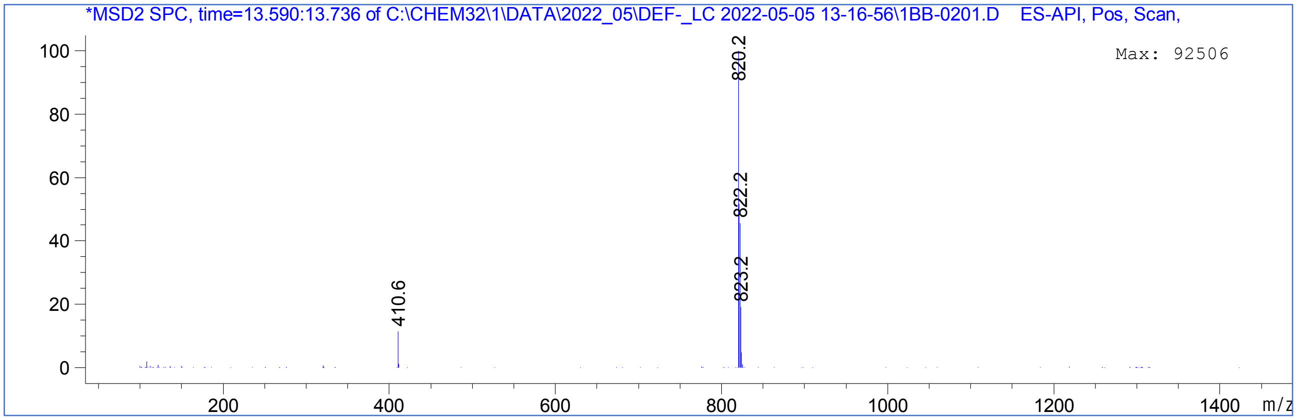

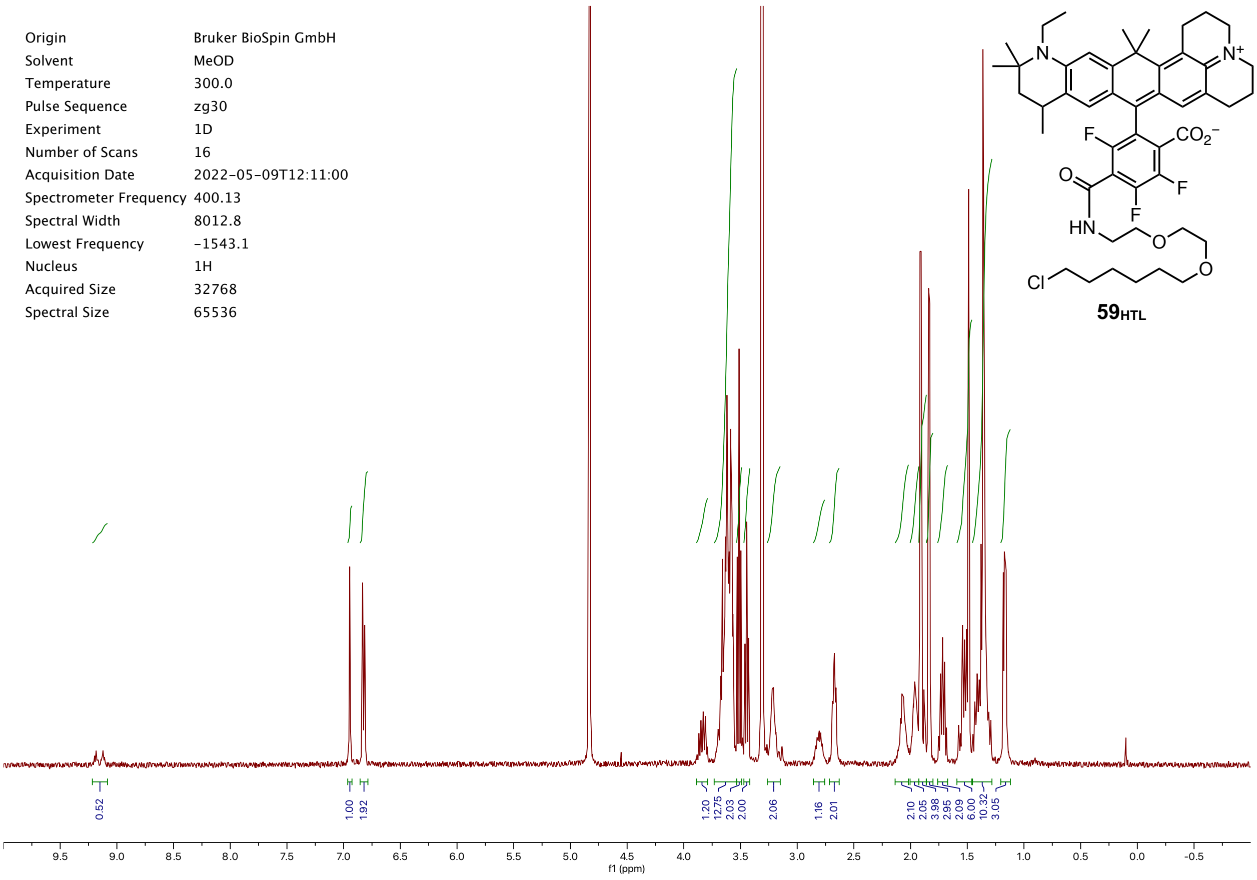
Origin Bruker BioSpin GmbH
Solvent MeOD
Temperature 300.0
Pulse Sequence zg30
Experiment 1D
Number of Scans 16
Acquisition Date 2022-05-09T12:11:00
Spectrometer Frequency 400.13
Spectral Width 8012.8
Lowest Frequency -1543.1
Nucleus 1H
Acquired Size 32768
Spectral Size 65536
0.52
1.00
1.92
1.20
12.75
2.03
2.00
2.06
1.16
2.01
2.10
2.05
3.98
2.95
2.09
6.00
10.32
3.05
f1 (ppm)

59HTL

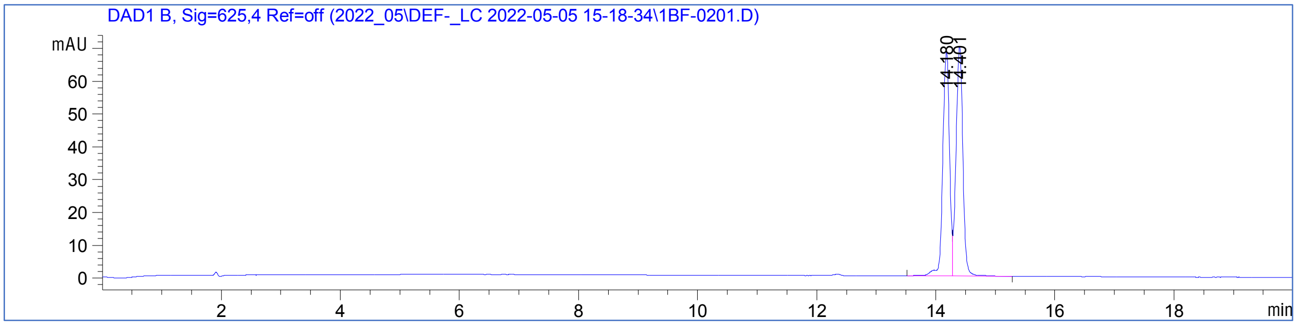
DAD1 B, Sig=625,4 Ref=off (2022_05\DEF-_LC 2022-05-05 15-18-34\1BF-0201.D)
mAU
14.180
14.401
min

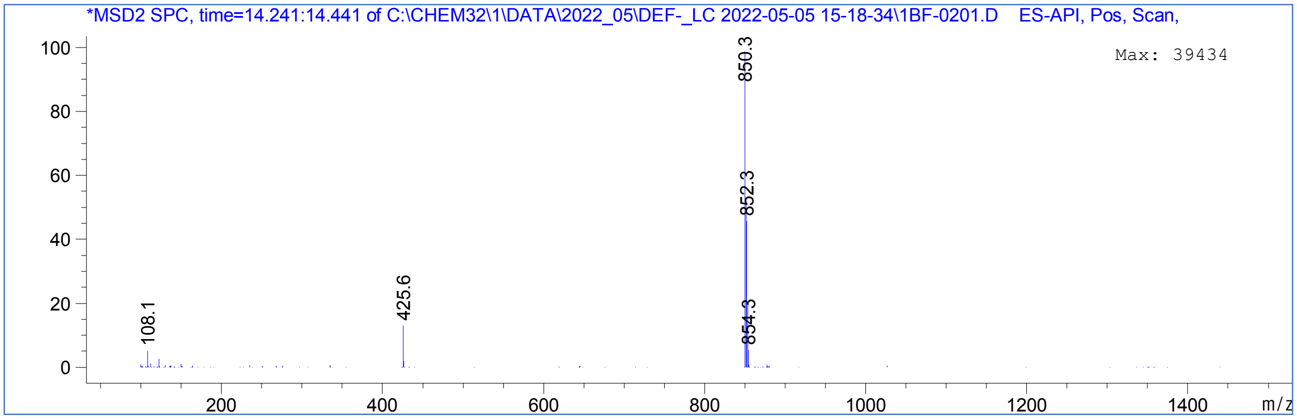
*MSD2 SPC, time=14.241:14.441 of C:\CHEM32\1\DATA\2022_05\DEF-_LC 2022-05-05 15-18-34\1BF-0201.D ES-API, Pos, Scan,
Max: 39434
850.3
852.3
854.3
425.6
108.1
m/z

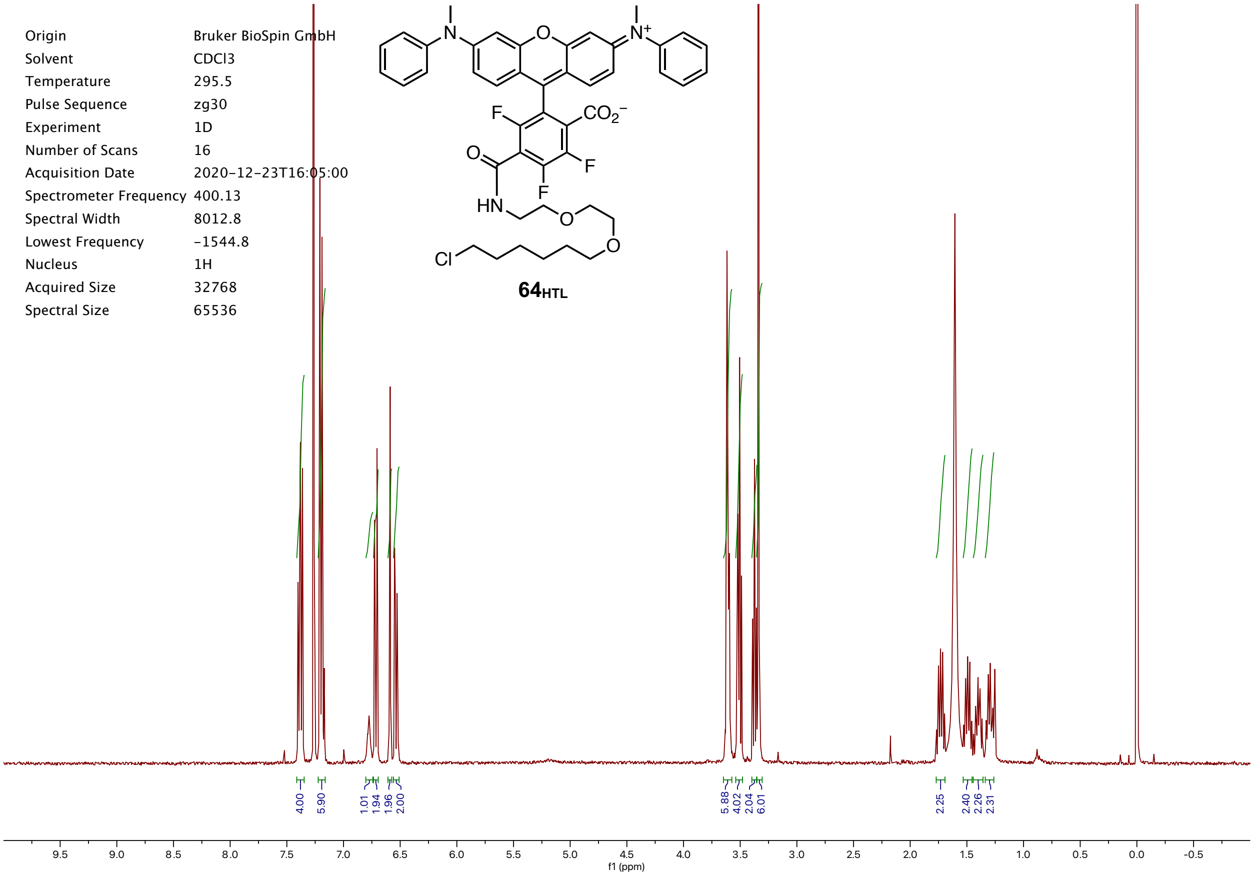

Origin Bruker BioSpin GmbH
Solvent CDCl3
Temperature 295.5
Pulse Sequence zg30
Experiment 1D
Number of Scans 16
Acquisition Date 2020-12-23T16:05:00
Spectrometer Frequency 400.13
Spectral Width 8012.8
Lowest Frequency -1544.8
Nucleus 1H
Acquired Size 32768
Spectral Size 65536
64HTL
4.00
5.90
1.01
1.94
1.96
2.00
5.88
4.02
2.04
6.01
2.25
2.40
2.26
2.31
f1 (ppm)

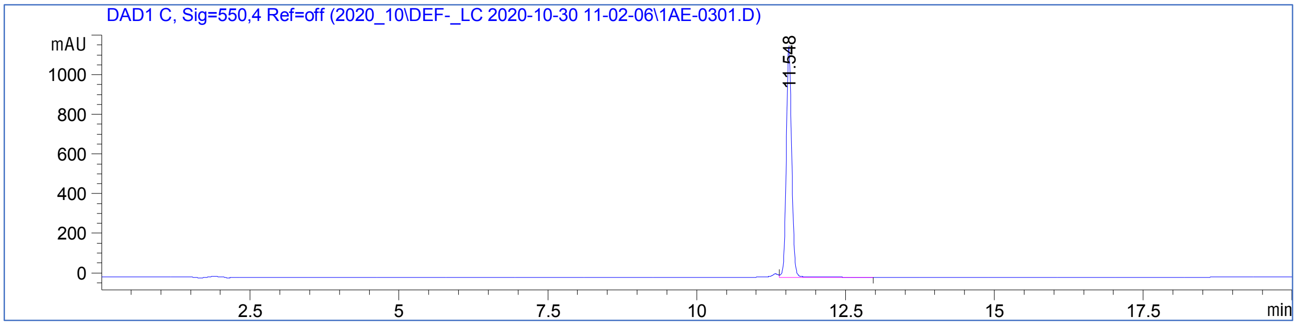

DAD1 C, Sig=550,4 Ref=off (2020_10\DEF-_LC 2020-10-30 11-02-06\1AE-0301.D)
mAU
11.548
min

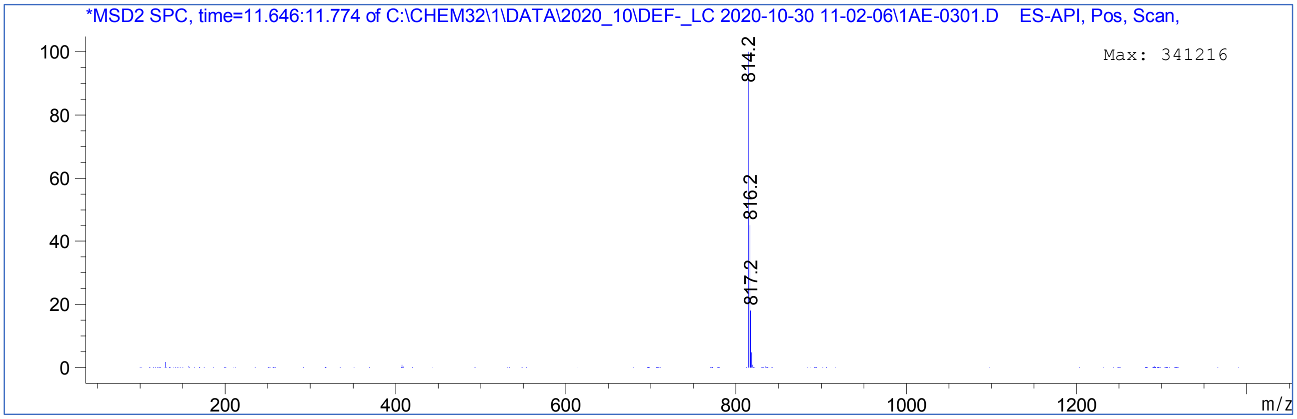

*MSD2 SPC, time=11.646:11.774 of C:\CHEM32\1\DATA\2020_10\DEF-_LC 2020-10-30 11-02-06\1AE-0301.D ES-API, Pos, Scan,
Max: 341216
814.2
816.2
817.2
m/z

Origin	Bruker BioSpin GmbH
Solvent	CDCl3
Temperature	295.3
Pulse Sequence	zg30
Experiment	1D
Number of Scans	16
Acquisition Date	2020-11-02T10:38:00
Spectrometer Frequency	400.13
Spectral Width	8012.8
Lowest Frequency	-1545.1
Nucleus	1H
Acquired Size	32768
Spectral Size	65536

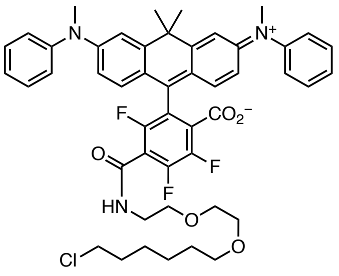

65_{HTL}

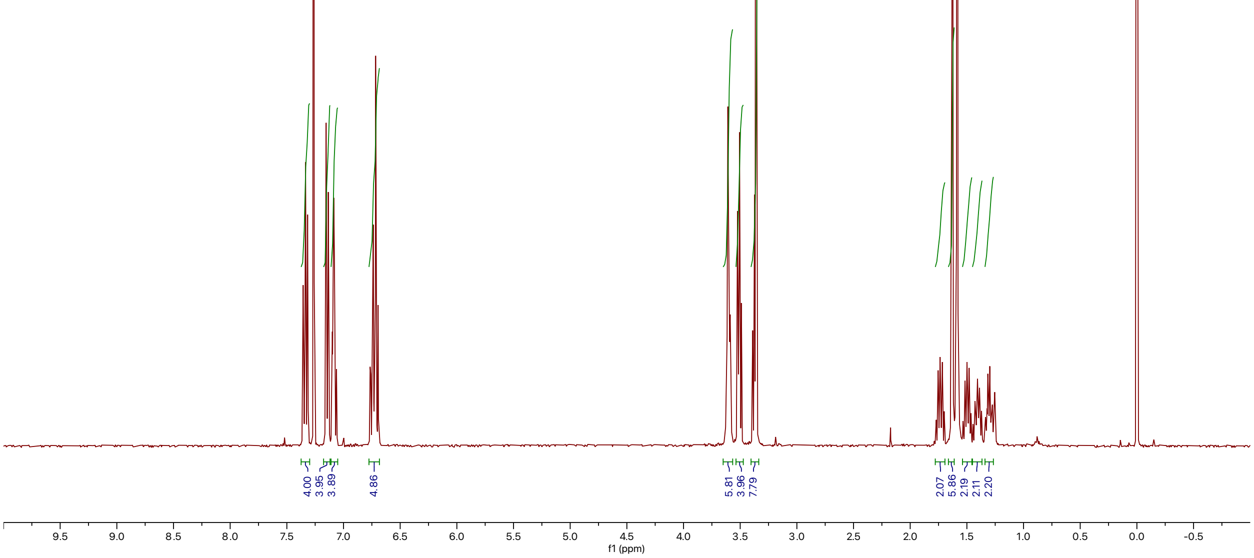

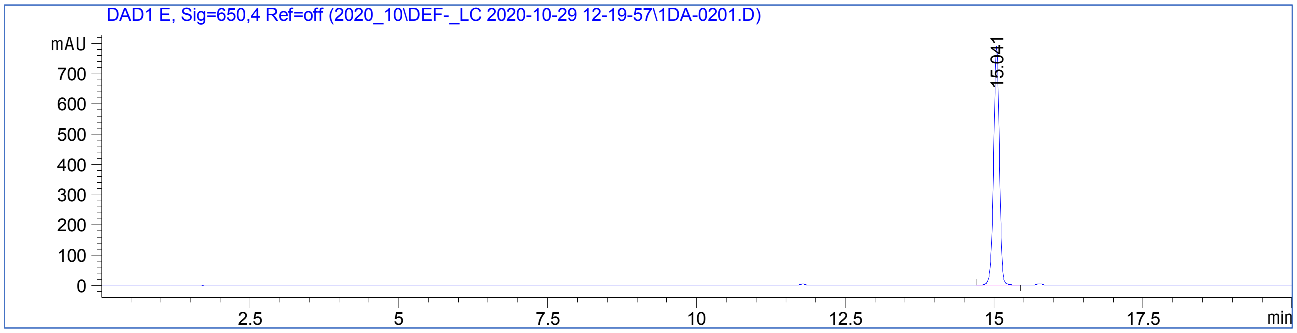

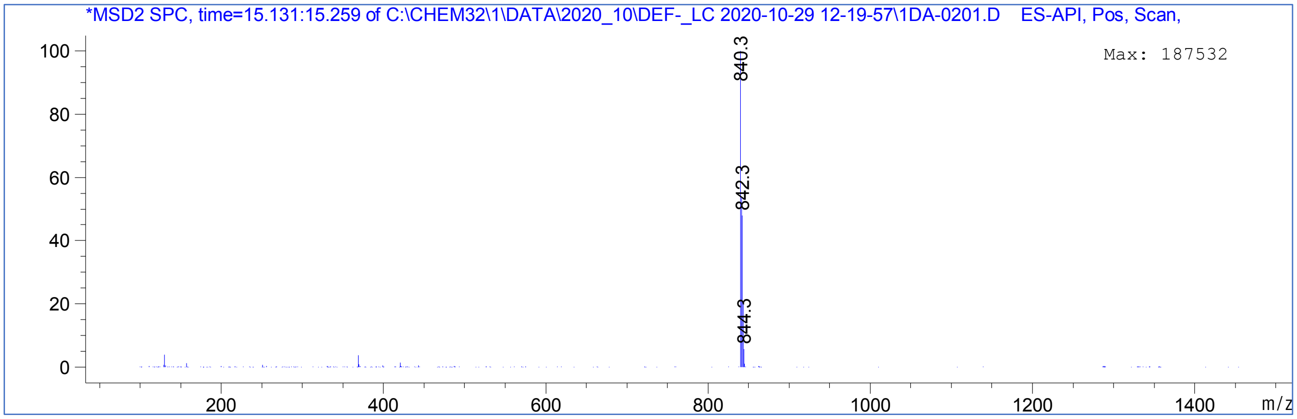

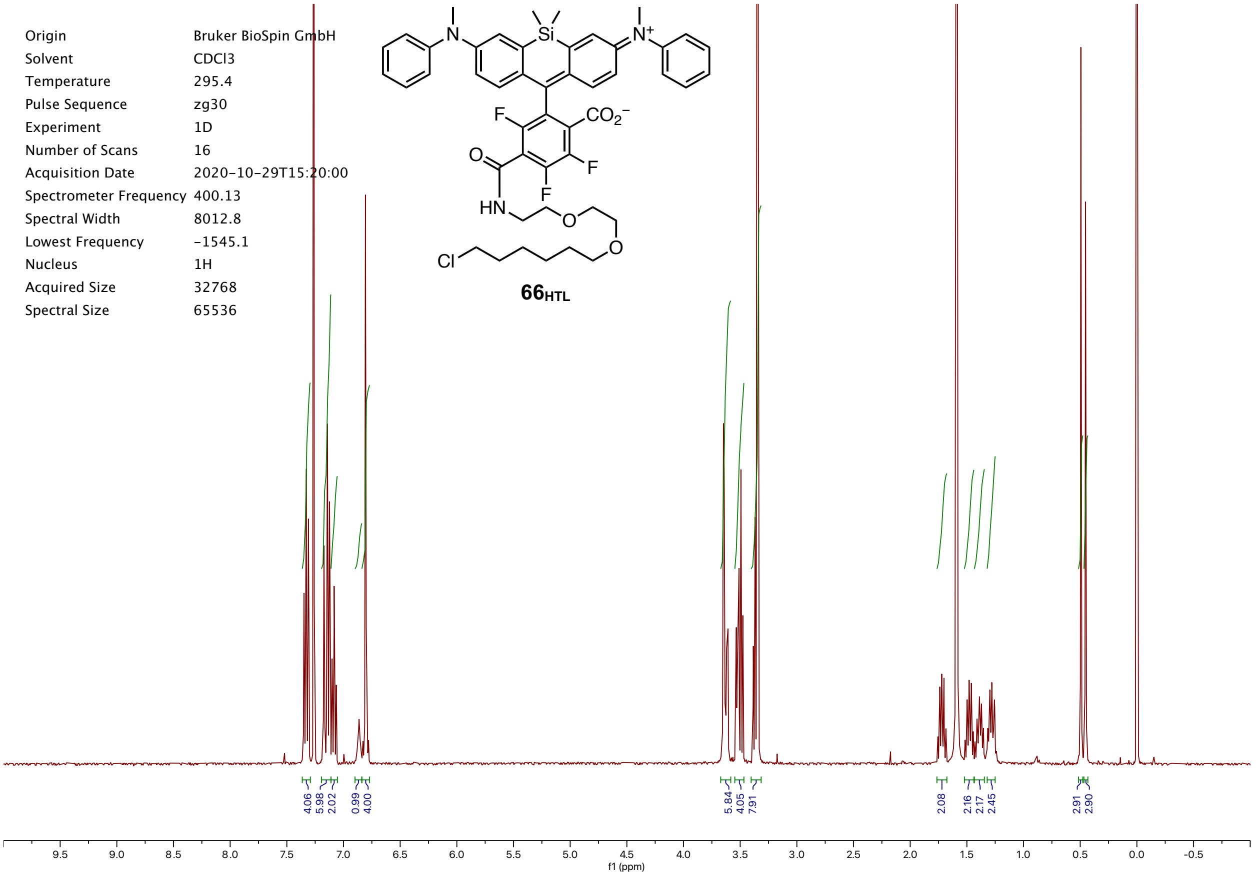

Origin Bruker BioSpin GmbH
Solvent CDCl3
Temperature 295.4
Pulse Sequence zg30
Experiment 1D
Number of Scans 16
Acquisition Date 2020-10-29T15:20:00
Spectrometer Frequency 400.13
Spectral Width 8012.8
Lowest Frequency -1545.1
Nucleus 1H
Acquired Size 32768
Spectral Size 65536
66HTL
f1 (ppm)

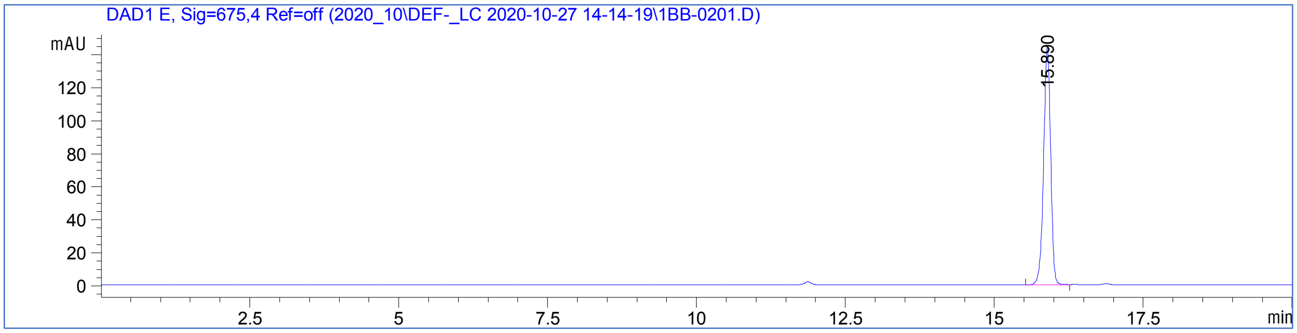

DAD1 E, Sig=675,4 Ref=off (2020_10\DEF-_LC 2020-10-27 14-14-19\1BB-0201.D)
mAU
15.890
min

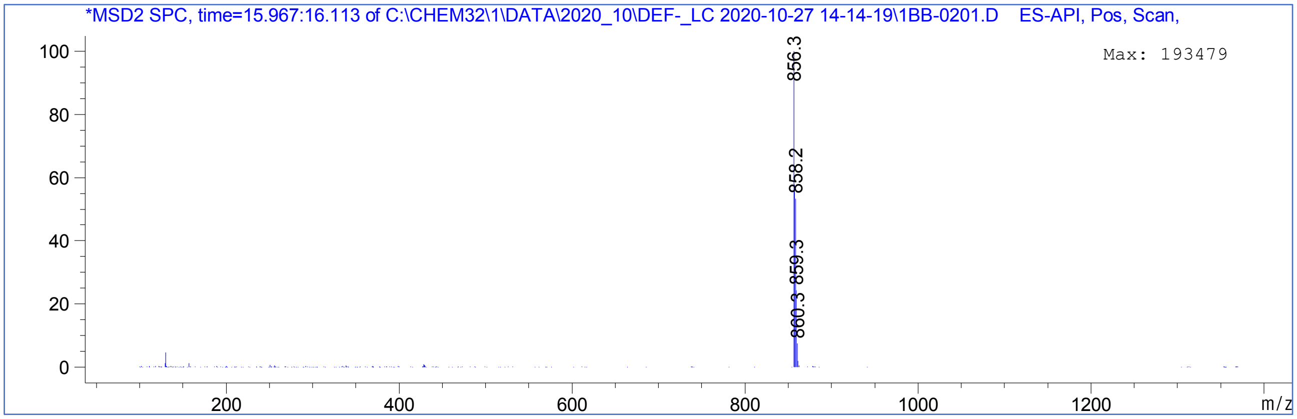

*MSD2 SPC, time=15.967:16.113 of C:\CHEM32\1\DATA\2020_10\DEF-_LC 2020-10-27 14-14-19\1BB-0201.D ES-API, Pos, Scan,
Max: 193479
856.3
858.2
859.3
860.3
m/z

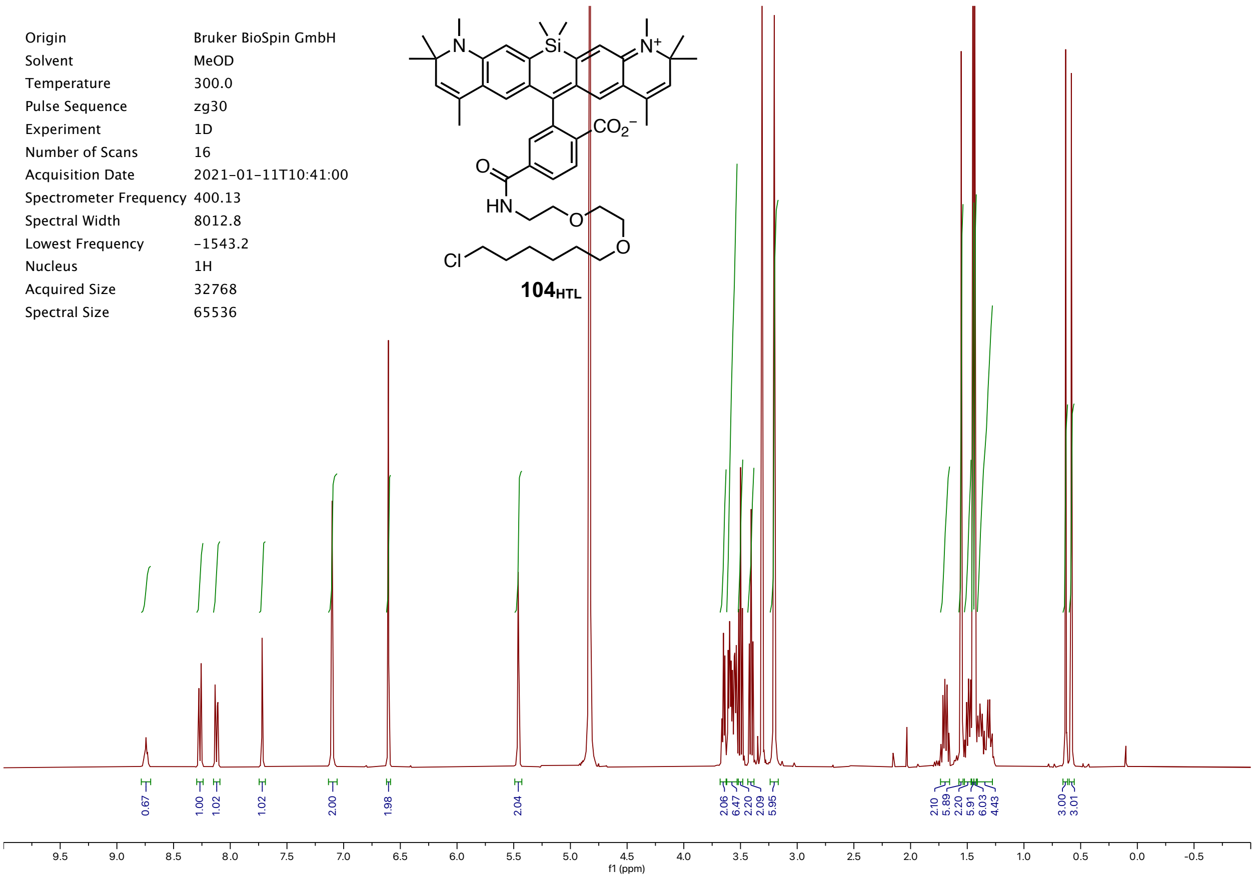
Origin Bruker BioSpin GmbH
Solvent MeOD
Temperature 300.0
Pulse Sequence zg30
Experiment 1D
Number of Scans 16
Acquisition Date 2021-01-11T10:41:00
Spectrometer Frequency 400.13
Spectral Width 8012.8
Lowest Frequency -1543.2
Nucleus 1H
Acquired Size 32768
Spectral Size 65536
104HTL
f1 (ppm)

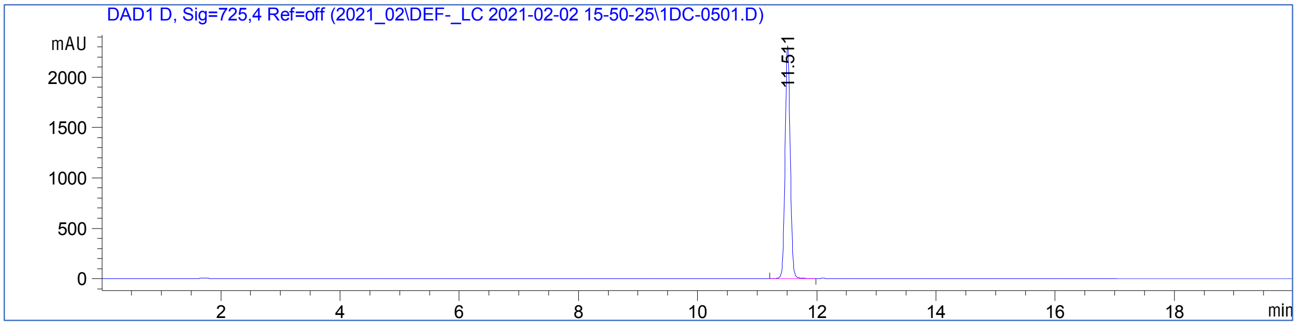
DAD1 D, Sig=725,4 Ref=off (2021_02\DEF-_LC 2021-02-02 15-50-25\1DC-0501.D)
mAU
11.511
min

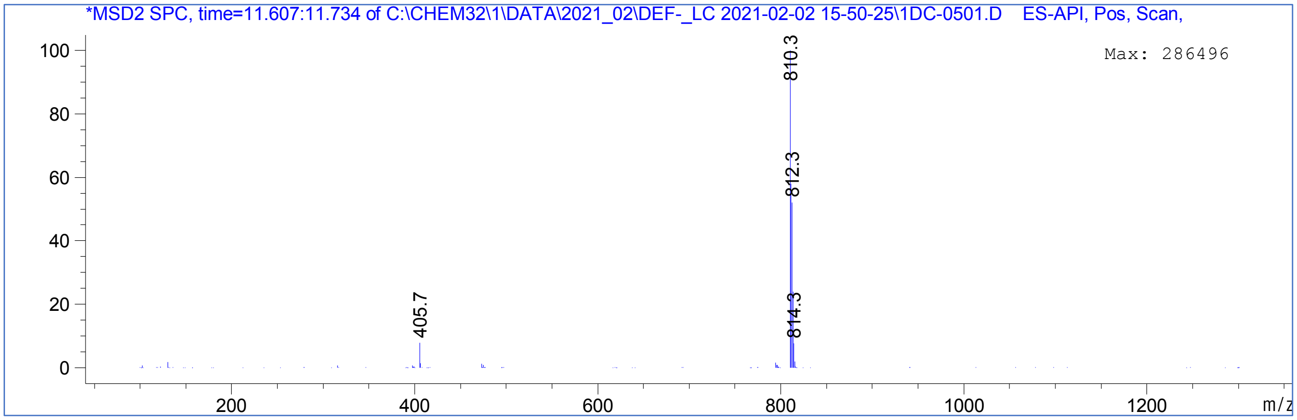
*MSD2 SPC, time=11.607:11.734 of C:\CHEM32\1\DATA\2021_02\DEF-_LC 2021-02-02 15-50-25\1DC-0501.D ES-API, Pos, Scan,
Max: 286496
810.3
812.3
814.3
405.7
m/z

Origin	Bruker BioSpin GmbH
Solvent	MeOD
Temperature	300.0
Pulse Sequence	zg30
Experiment	1D
Number of Scans	16
Acquisition Date	2020-06-08T10:52:00
Spectrometer Frequency	400.13
Spectral Width	8012.8
Lowest Frequency	-1543.2
Nucleus	1H
Acquired Size	32768
Spectral Size	65536

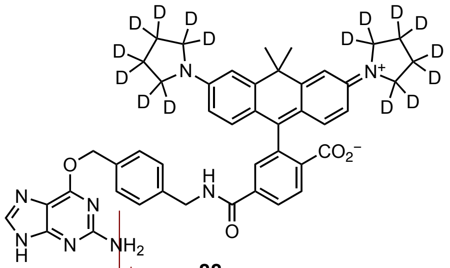

88$_{STL}$

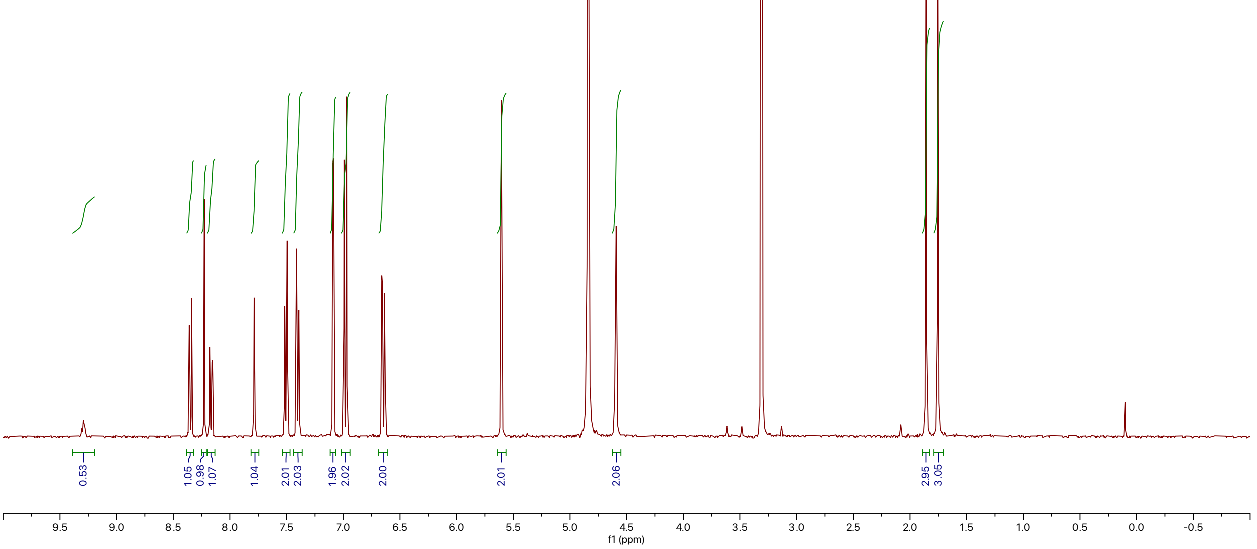

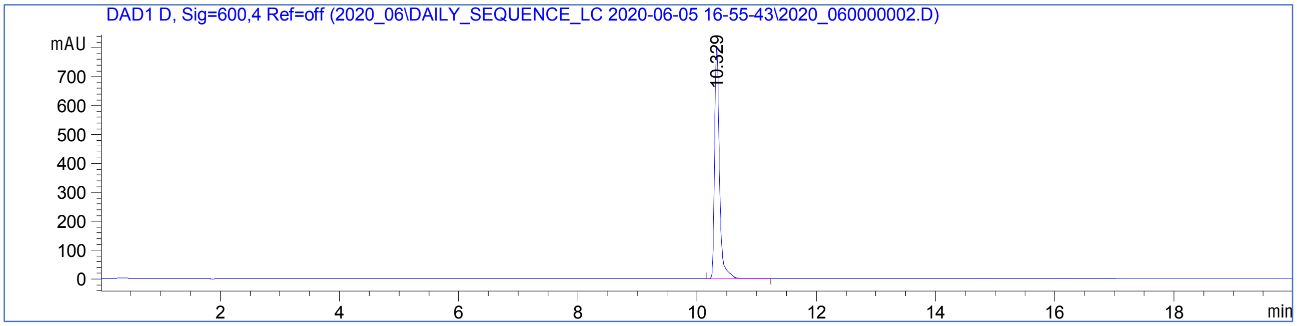

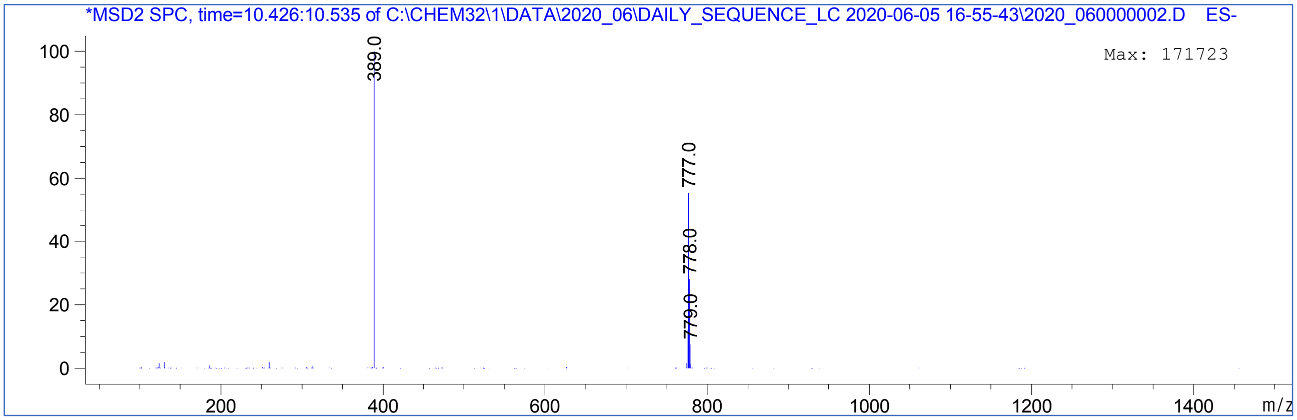

Origin	Bruker BioSpin GmbH
Solvent	MeOD
Temperature	295.5
Pulse Sequence	zg30
Experiment	1D
Number of Scans	16
Acquisition Date	2018-12-13T12:00:00
Spectrometer Frequency	400.13
Spectral Width	8012.8
Lowest Frequency	-1543.2
Nucleus	1H
Acquired Size	32768
Spectral Size	65536

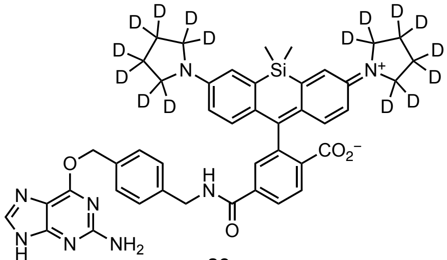

89STL

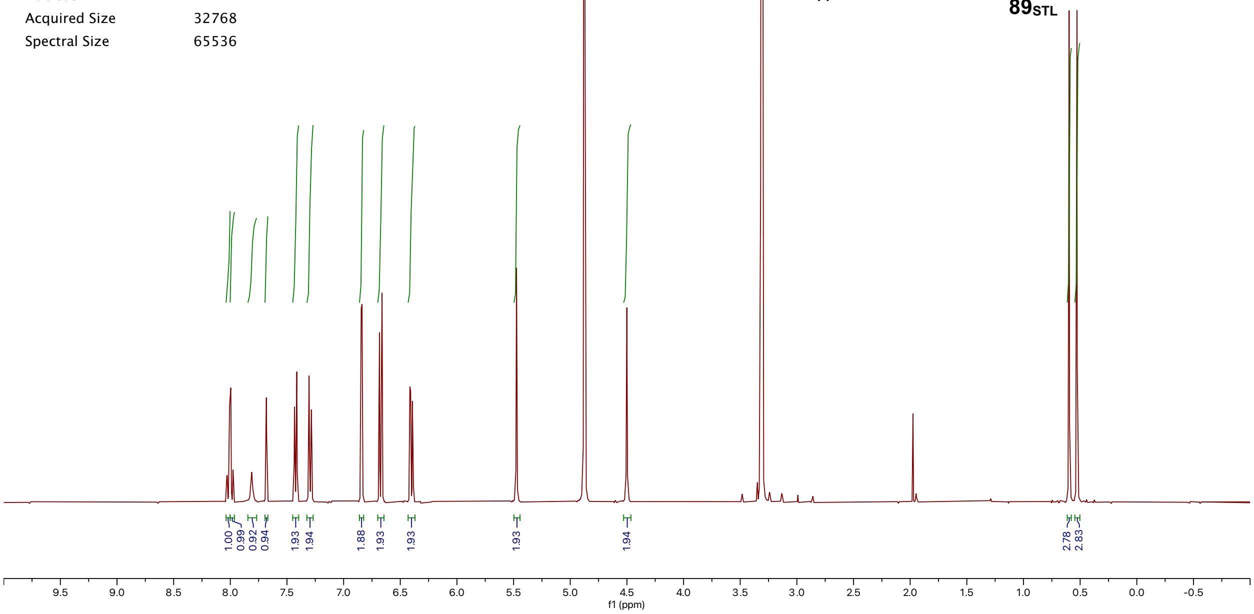

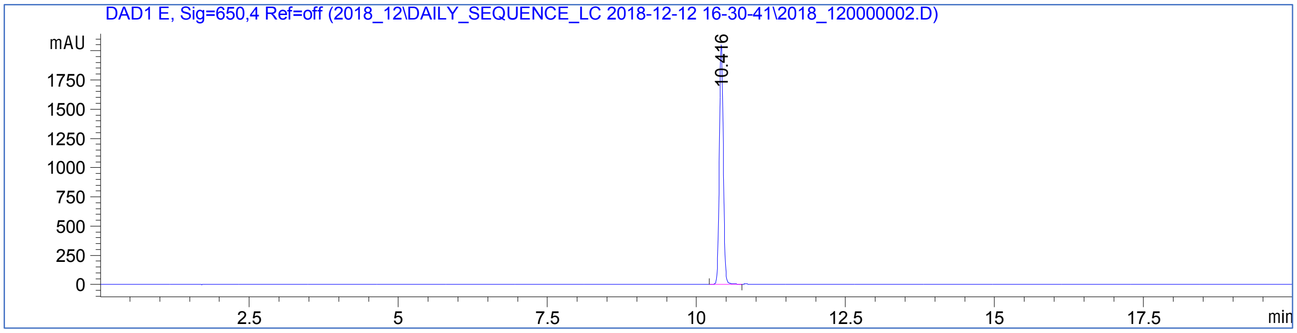

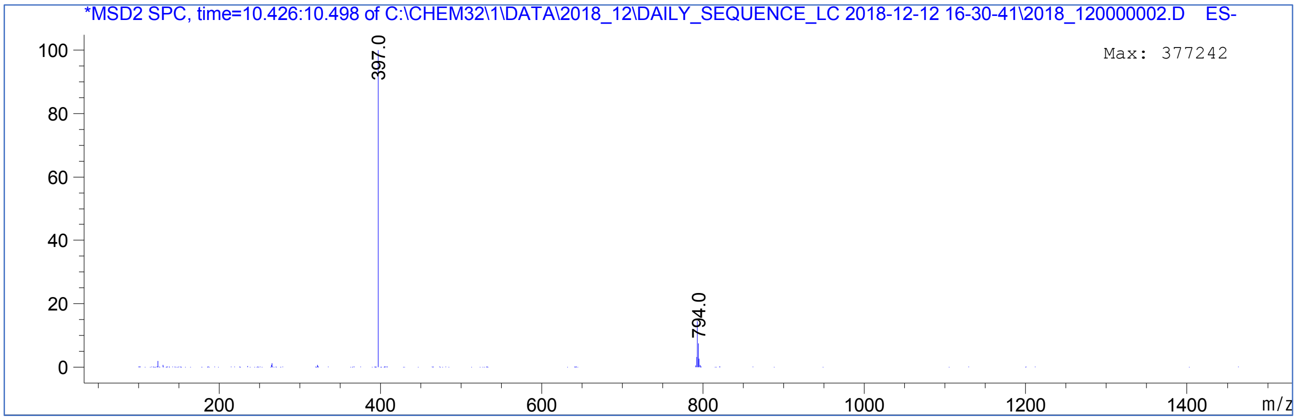

Origin	Bruker BioSpin GmbH
Solvent	CDCl3
Temperature	295.5
Pulse Sequence	zg30
Experiment	1D
Number of Scans	16
Acquisition Date	2023-06-28T11:18:29
Spectrometer Frequency	400.13
Spectral Width	8012.8
Lowest Frequency	-1545.3
Nucleus	1H
Acquired Size	32768
Spectral Size	65536

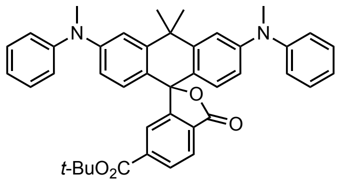

S59

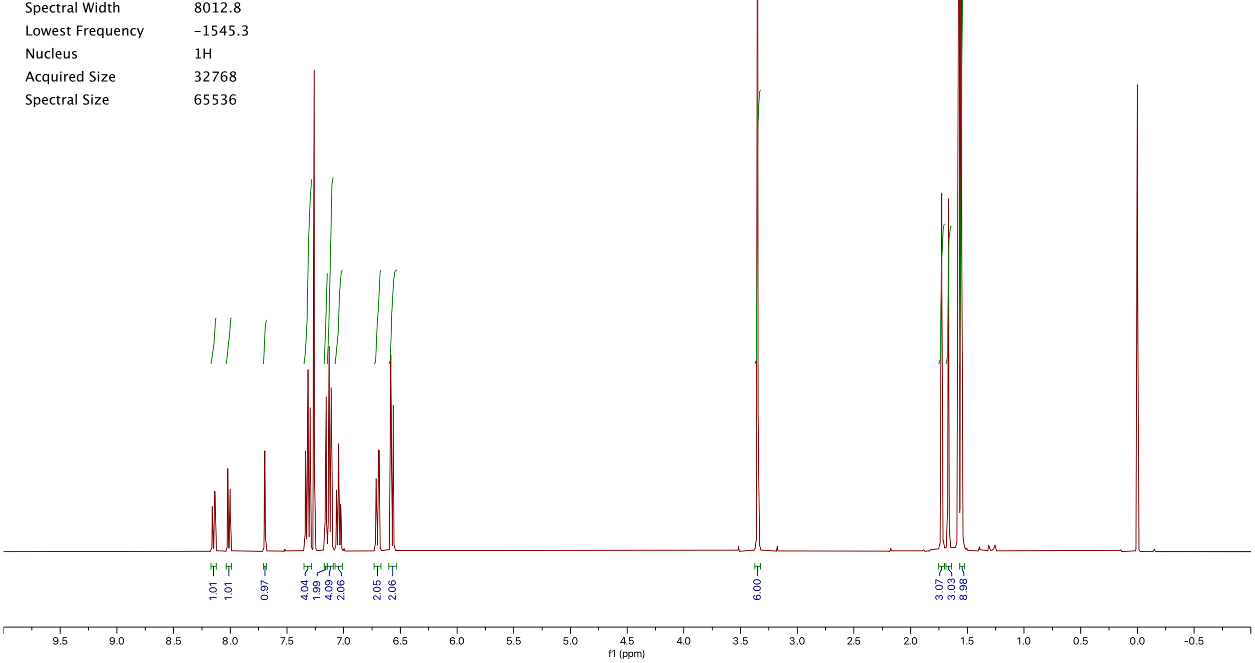

Origin	Bruker BioSpin GmbH
Solvent	CDCl3
Temperature	296.1
Pulse Sequence	zgpg30
Experiment	1D
Number of Scans	2048
Acquisition Date	2023-06-28T18:42:43
Spectrometer Frequency	100.62
Spectral Width	24038.5
Lowest Frequency	-1947.7
Nucleus	13C
Acquired Size	32768
Spectral Size	65536

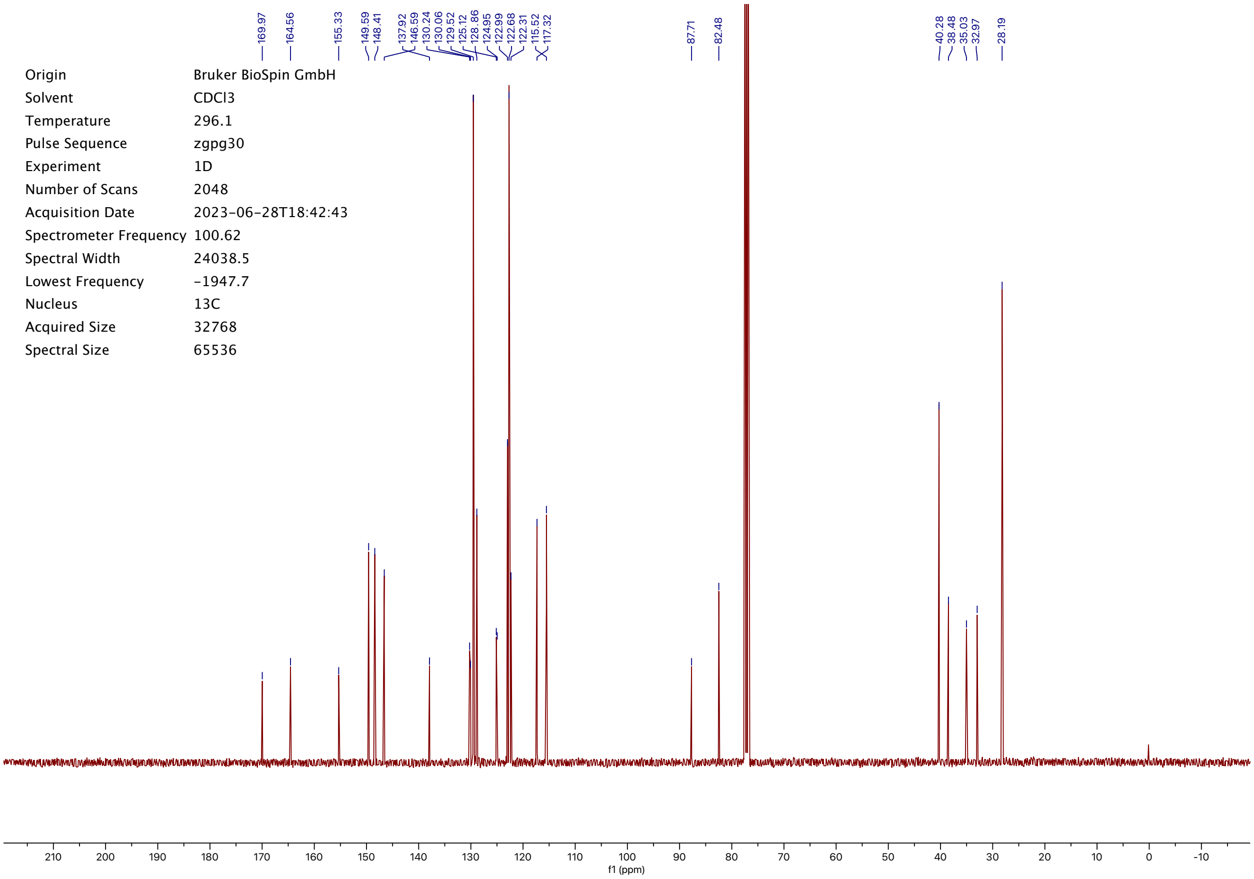

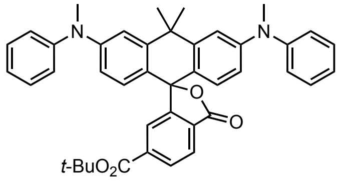

S59

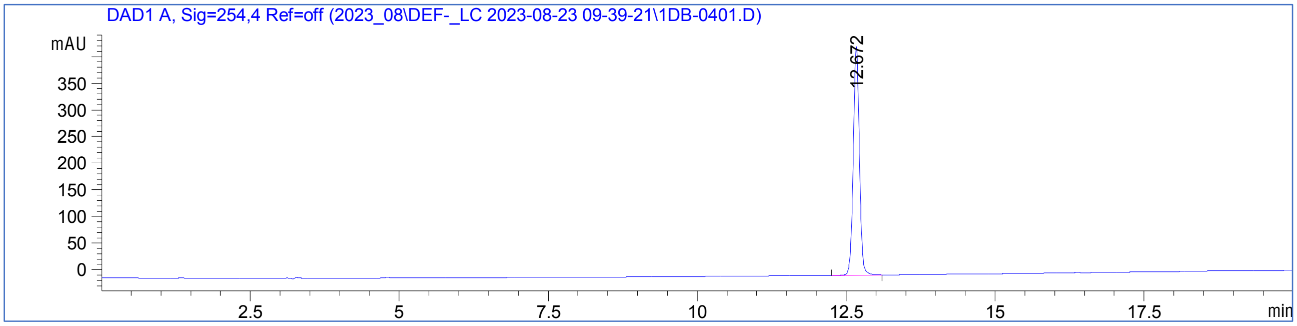
DAD1 A, Sig=254,4 Ref=off (2023_08\DEF-_LC 2023-08-23 09-39-21\1DB-0401.D)
mAU
350
300
250
200
150
100
50
0
12.672
2.5
5
7.5
10
12.5
15
17.5
min

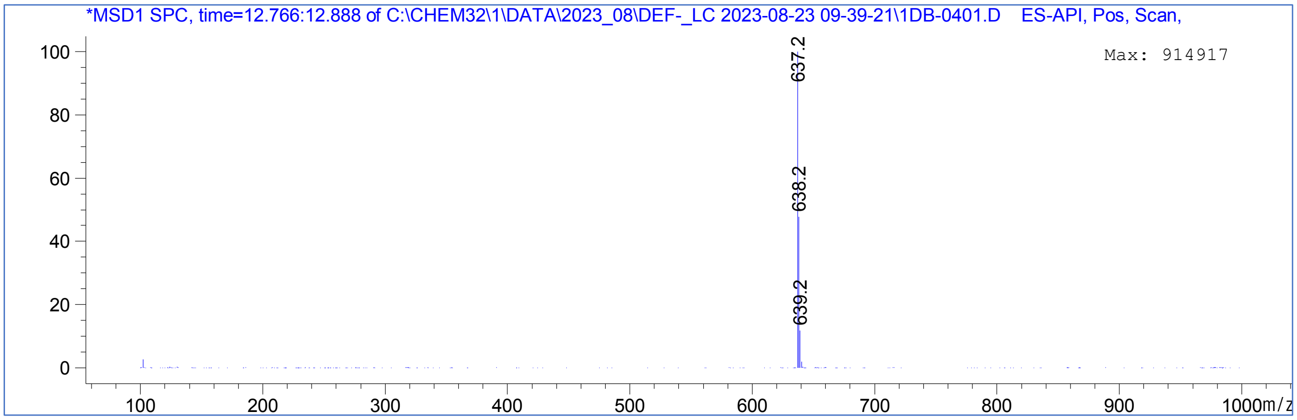
*MSD1 SPC, time=12.766:12.888 of C:\CHEM32\1\DATA\2023_08\DEF-_LC 2023-08-23 09-39-21\1DB-0401.D ES-API, Pos, Scan,
Max: 914917
100
80
60
40
20
0
637.2
638.2
639.2
100
200
300
400
500
600
700
800
900
1000
m/z

Origin	Bruker BioSpin GmbH
Solvent	MeOD
Temperature	295.2
Pulse Sequence	zg30
Experiment	1D
Number of Scans	16
Acquisition Date	2023-07-05T16:31:13
Spectrometer Frequency	400.13
Spectral Width	8012.8
Lowest Frequency	-1543.3
Nucleus	1H
Acquired Size	32768
Spectral Size	65536

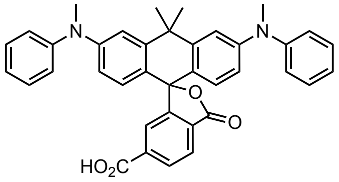

S60

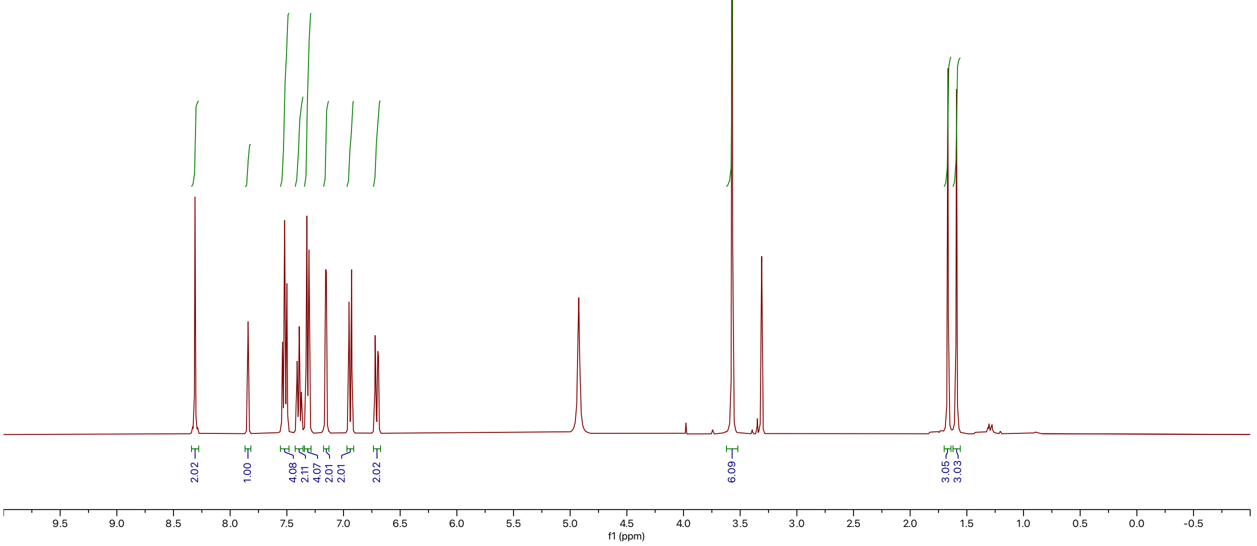

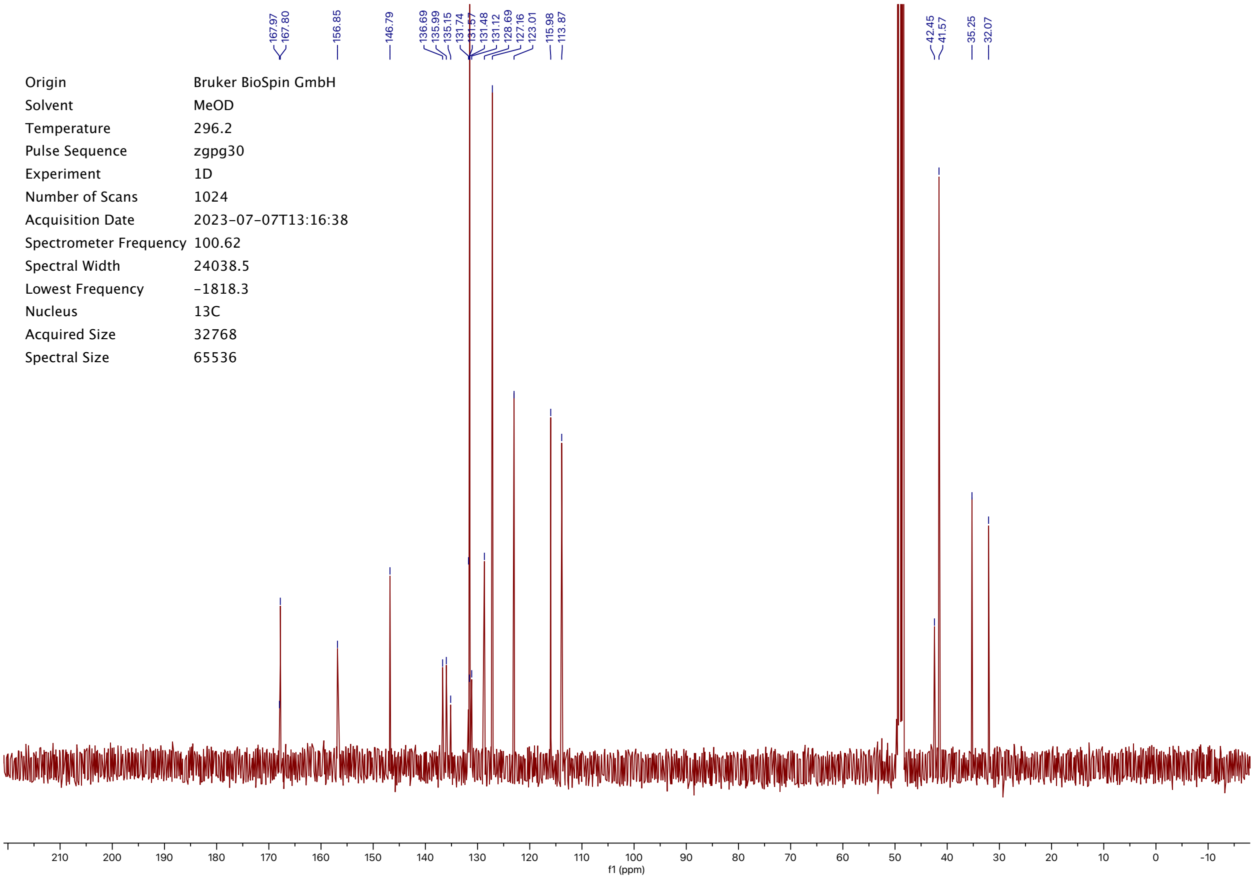

Origin	Bruker BioSpin GmbH
Solvent	MeOD
Temperature	296.2
Pulse Sequence	zgpg30
Experiment	1D
Number of Scans	1024
Acquisition Date	2023-07-07T13:16:38
Spectrometer Frequency	100.62
Spectral Width	24038.5
Lowest Frequency	-1818.3
Nucleus	13C
Acquired Size	32768
Spectral Size	65536

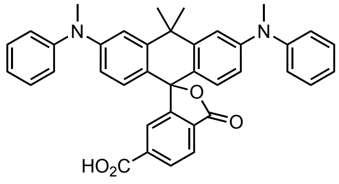

S60

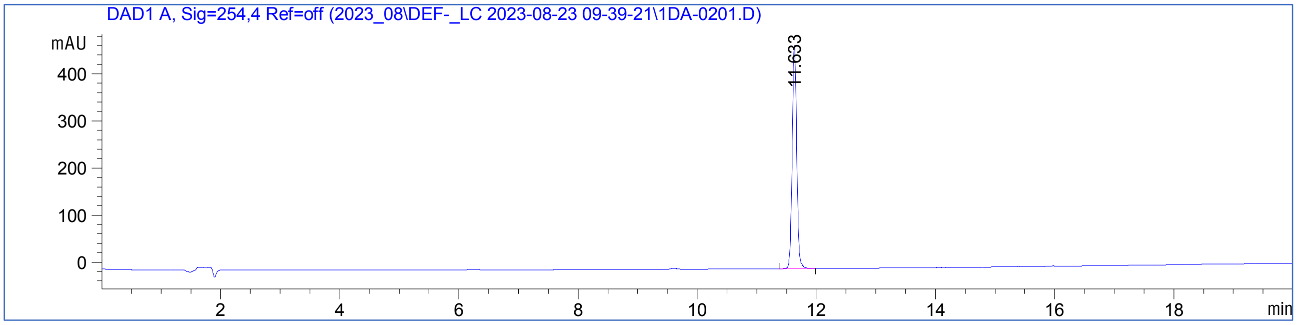

DAD1 A, Sig=254,4 Ref=off (2023_08\DEF-_LC 2023-08-23 09-39-21\1DA-0201.D)
mAU
400
300
200
100
0
11.633
2
4
6
8
10
12
14
16
18
min

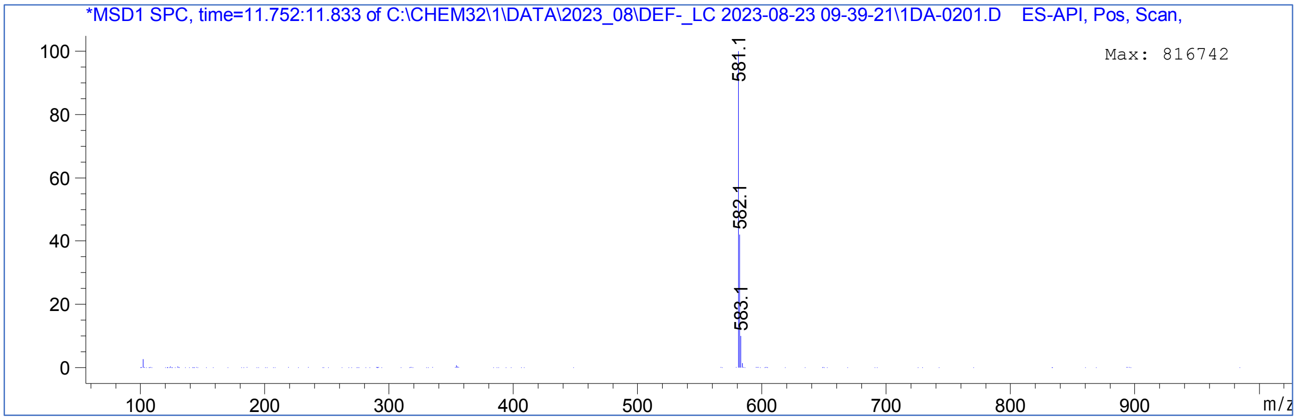

*MSD1 SPC, time=11.752:11.833 of C:\CHEM32\1\DATA\2023_08\DEF-_LC 2023-08-23 09-39-21\1DA-0201.D ES-API, Pos, Scan,
Max: 816742
100
80
60
40
20
0
581.1
582.1
583.1
100
200
300
400
500
600
700
800
900
m/z

Origin	Bruker BioSpin GmbH
Solvent	CDCl3
Temperature	295.5
Pulse Sequence	zg30
Experiment	1D
Number of Scans	16
Acquisition Date	2023-07-10T11:31:14
Spectrometer Frequency	400.13
Spectral Width	8012.8
Lowest Frequency	-1544.8
Nucleus	1H
Acquired Size	32768
Spectral Size	65536

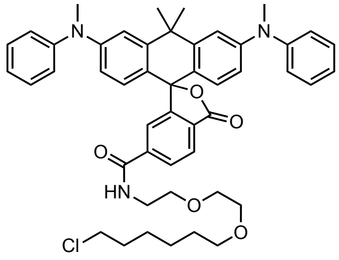

91$_{HTL}$

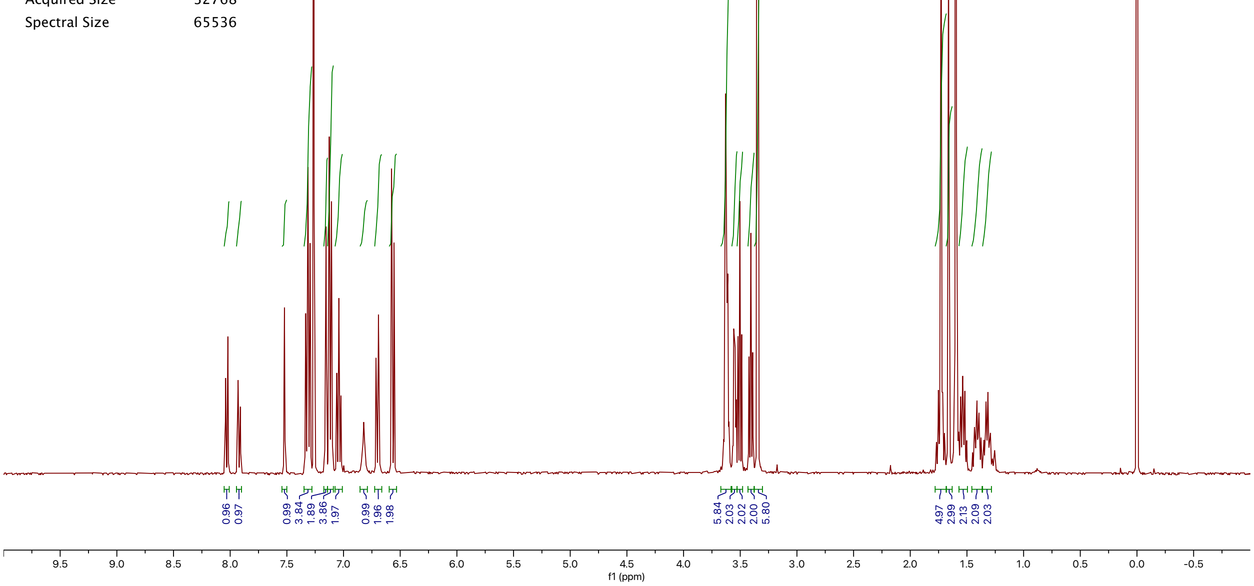

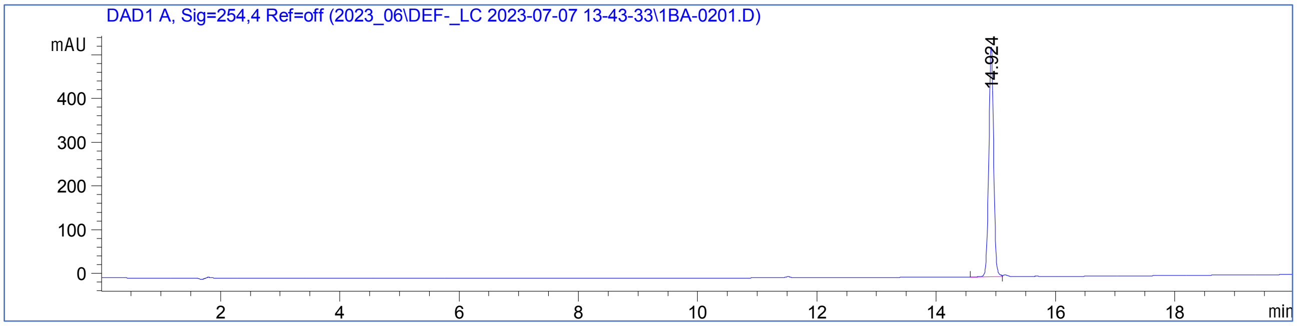

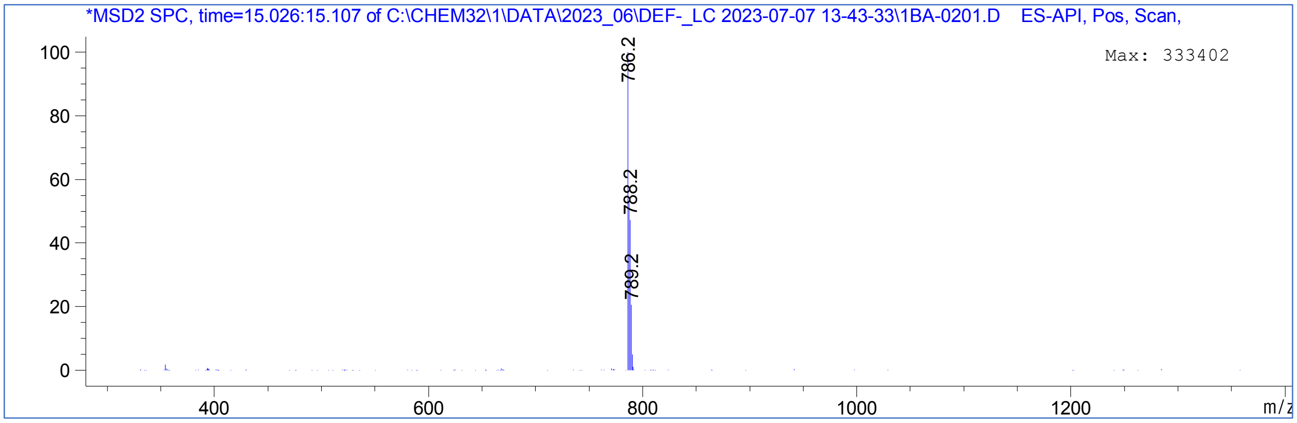

Supplement: Supplementary file 1 — ja3c05273_si_001.pdf [file ja3c05273_si_001.pdf]
